# Identifying potential drug targets for sepsis-related adult respiratory distress syndrome through comprehensive genetic analysis and druggability assessment

**DOI:** 10.7189/jogh.15.04117

**Published:** 2025-03-21

**Authors:** Jinsen Weng, Xiaojing Wang, Jingping Lin, Yong Ye, Junjie Wei, Rongguo Yu, Xiuling Shang

**Affiliations:** 1 Shengli Clinical Medical College of Fujian Medical University, Fuzhou, Fujian, China; 2 Department of Critical Care Medicine, Clinical Oncology School of Fujian Medical University, Fujian Cancer Hospital, Fuzhou, Fujian, China; 3 Department of Critical Care Medicine, Fuzhou Second General Hospital, Fuzhou, Fujian, China; 4 Department of Critical Care Medicine, Shengli Clinical Medical College of Fujian Medical University, Fuzhou University Affiliated Provincial Hospital, Fujian Provincial Hospital, Fujian Provincial Center for Critical Care Medicine, Fujian Provincial Key Laboratory of Critical Care Medicine, Fuzhou, Fujian, China

## Abstract

**Background:**

Sepsis-related adult respiratory distress syndrome (ARDS) is a life-threatening condition characterised by a high mortality rate. This underscores the pressing requirement to identify and develop potential therapeutic targets for the severe condition. This study investigated the genetic predisposition to sepsis-related ARDS in this study.

**Methods:**

We utilised summary-based Mendelian randomisation (SMR), two-sample MR (TSMR), mediating MR, and multivariate MR (MVMR) analysis to explore the genetic susceptibility of sepsis-related ARDS by integrating over 10 000 cis-expression quantitative trait loci (cis-eQTLs) and over 100 000 participants. Subsequently, we performed drug target analysis to identify potentially druggable cis-eQTL genes.

**Results:**

The SMR analysis identified 677 cis-eQTL genes associated with sepsis. Further TSMR validation filtered 72 cis-eQTL genes causally associated with sepsis. Sepsis was causally associated with ARDS (beta = 1.80, standard error (SE) = 0.36, *P* < 0.001). After conducting the mediating MR and MVMR analysis, 50 cis-eQTL genes were reported to be causally associated with sepsis-related ARDS. Subsequent drug target analysis confirmed the role of four targets (PSMA4, PDK2, RPS18, and NDUFV3) as druggable genes for sepsis-related ARDS.

**Conclusions:**

Through an extensive analysis, we identified potential drug targets for sepsis-related ARDS. Additional research is imperative to substantiate our discoveries and to pave the way for the development of novel pharmaceuticals aimed at these specific targets.

Sepsis, a critical condition arising from an exaggerated immune reaction to infection, manifests as a systemic inflammatory response [1]. This response, if not swiftly and effectively addressed, can escalate to multiple organ dysfunction syndrome, posing a significant threat to life [2]. The lungs frequently bear the brunt of sepsis, often being the initial organ compromised by this condition. Typically, this manifests as adult respiratory distress syndrome (ARDS), underscoring the need for vigilant monitoring and immediate therapeutic intervention [3–5]. Despite the progress made in supportive care approaches, the mortality rate associated with ARDS remains distressingly high [5–7]. This highlights an urgent need to identify and develop novel therapeutic targets for individuals who are at an elevated risk of developing this critical condition.

Recently, emerging studies concentrated on the biomarkers of both sepsis and ARDS [8–12]. Those studies, which encompassed a limited scope by focusing on a few thousand protein biomarkers or participants, failed to address the interplay between these two entities. This oversight urges a comprehensive understanding of sepsis-related ARDS and the development of targeted therapies. Hence, a drug-target Mendelian randomisation (MR) analysis is aptly suited for exploring the relationship in such scenarios. In the MR analysis, the genetic variants are utilised as instrumental variables to establish causality between drug targets and disease outcomes, offering a robust approach to identifying potential therapeutic interventions [13–17]. Based on the literature review, we found two articles focusing on the genetic predisposition to sepsis-related ARDS through MR analysis [18,19]. Nevertheless, they did not conduct a genome-wide MR analysis and they genotyped on a relatively small sample size. To advance this research, we investigated the genetic predisposition to sepsis-related ARDS at a genome-wide scale. In this study, we delved into the genetic susceptibility of sepsis-related ARDS by integrating over 10 000 cis-expression quantitative trait loci (cis-eQTLs) and over 100 000 participants.

## METHODS

**Figure 1:**
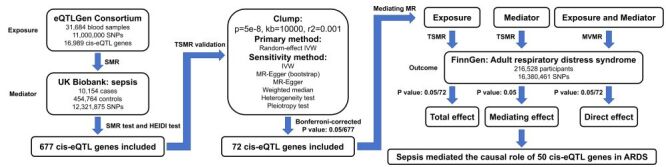
The study flowchart indicated the application of SMR, TSMR, mediating MR, and MVMR analysis to investigate the sepsis-mediated causal role of cis-eQTL genes in ARDS. ARDS – adult respiratory distress syndrome, cis-eQTL – cis-expression quantitative trait locus, HEIDI – heterogeneity in dependent instruments, IVW – inverse variance weighted, MVMR – multivariate Mendelian randomisation, SMR – summary-based Mendelian randomisation, SNP – single nucleotide polymorphism, TSMR – two-sample Mendelian randomisation.

This study utilised the genome-wide association study (GWAS) data from the IEU OpenGWAS database (https://gwas.mrcieu.ac.uk) and the eQTLGen Consortium (https://www.eqtlgen.org/). We applied the summary-based MR (SMR), two-sample MR (TSMR), mediating MR, and multivariate MR (MVMR) analysis to investigate the sepsis-mediated causal role of cis-eQTL genes in ARDS. Further drug target analysis was conducted to find potentially druggable cis-eQTL genes (**Figure 1**).

### Exposure, mediator, and outcome phenotypes

The 16 989 cis-eQTL genes served as the exposure traits, which derived from the eQTLGen Consortium [20]. The eQTLGen consortium was a collaborative endeavour on a grand scale, with the primary objective of meticulously charting the influence of genetic variations on gene expression. This consortium amassed and scrutinised both genetic and transcriptomic data from a vast array of individuals, numbering in the tens of thousands. The overarching aim was to pinpoint the genetic variants that modulate gene expression and to harness this knowledge to deepen our comprehension of the genetic underpinnings of complex diseases [20] (Table S1 in the **Online Supplementary Document**).

The sepsis data were included as the mediator trait in this study. We extracted the GWAS data of sepsis from the UK Biobank [21]. A total of 10 154 cases and 454 764 controls with over 12 million single nucleotide polymorphisms (SNPs) were included. The data could be accessed through https://gwas.mrcieu.ac.uk [22–24] (Table S1 in the **Online Supplementary Document**).

The ARDS data were incorporated to serve as the outcome trait. The GWAS data came from the FinnGen study [25]. The research encompassed 216 528 subjects and incorporated 16 380 461 SNPs, which was collected and curated by the IEU OpenGWAS database and could be accessed through https://gwas.mrcieu.ac.uk [22–24] (Table S1 in the **Online Supplementary Document**).

### SMR analysis

We performed the SMR analysis to investigate the causal role of 16 989 cis-eQTL genes in sepsis [26]. Genes with a *P*-value of <0.05 for the SMR test and >0.05 for the heterogeneity in dependent instruments (HEIDI) test were considered to have a causal association with the sepsis phenotype.

### TSMR validation

For those cis-eQTL genes that passed the SMR analysis, the TSMR analysis was used to reconfirm and validate their causal role. We also performed a TSMR analysis between those cis-eQTL genes and ARDS traits and between sepsis and ARDS traits. Single nucleotide polymorphisms (SNPs) were clumped with the following parameters: *P*-value = 0.00000005, kilobase (kb) = 10 000, coefficient of correlation (r2) = 0.001. The TSMR effect based on the inverse variance weighted (IVW) with multiplicative random effects was considered the primary outcome. A Bonferroni-corrected *P*-value was used to infer a causal association.

### Sensitivity analysis

For the TSMR validation, sensitivity analysis was performed. The TSMR effect based on another four methods was calculated: IVW, MR-Egger, MR-Egger (bootstrap), and weighted median. Heterogeneity and pleiotropy analysis were conducted. The MR-Egger (bootstrap) method was recommended if a significant pleiotropy existed. The IVW with multiplicative random effects method was recommended if a significant heterogeneity existed.

### MVMR analysis

To investigate the direct effect of those cis-eQTL genes that passed the SMR analysis and TSMR validation on ARDS, an MVMR analysis was conducted. Similarly, a Bonferroni-corrected *P*-value was used to infer a causal association.

### Mediating MR analysis

The mediating MR analysis was performed to explore the relationship among those cis-eQTL genes that passed the SMR analysis and TSMR validation, sepsis, and ARDS traits. The total effect was defined as the TSMR effect of those cis-eQTL genes on ARDS. The direct effect was defined as the MVMR effect of those cis-eQTL genes on ARDS. The mediating effect was defined as the product of the TSMR effect of those cis-eQTL genes on sepsis and the TSMR effect of sepsis on ARDS. In the clinical setting, the ARDS disease often arises as a consequence of sepsis. Therefore, our study concentrated on those cis-eQTL genes that were implicated in the pathogenesis of ARDS via a sepsis-mediated mechanism. In other words, a cis-eQTL gene that demonstrated a significant mediating effect on ARDS, while exhibiting no substantial total or direct effects, held greater significance in the context of this study. Thus, we focused on the cis-eQTL genes that only yielded significant mediating effects.

### Drug target analysis

For those cis-eQTL genes with only significant mediating effects on ARDS, a comprehensive database search was applied to identify druggable targets. Generally, three databases were reviewed: DrugBank [27], ChEMBL [28], and DGIdb [29]. We presented targets with available drugs across all three databases. A drug target was deemed adequately validated if corresponding drugs were identified across all three databases.

### Statistical analysis

The principal analysis was conducted using *R* version 4.4.0 (R Core Team, Vienna, Austria), GraphPad Prism version 9 (San Diego, California, USA), and the Hiplot Pro platform (https://hiplot.com.cn/). The TwoSampleMR [23], Rmediation [30,31], and Cmplot (https://github.com/YinLiLin/CMplot) were the main packages. The data were presented as beta effect size with corresponding confidence interval (CI).

## RESULTS

The general design was presented in **Figure 1**.

### The SMR analysis identified 677 cis-eQTL genes associated with sepsis

**Figure 2:**
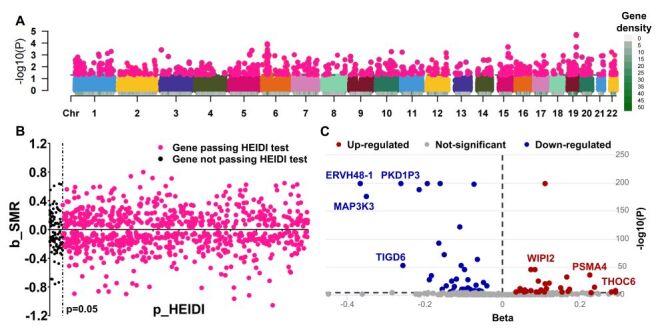
The SMR and TSMR validation of cis-eQTL genes from the eQTLGen Consortium. **Panel A.** The scatter plot of all cis-eQTL genes. The pink points indicated genes with a *P*-value <0.05. **Panel B.** The scatter plot of cis-eQTL genes that passed the SMR test in **Panel A**. The pink points indicated genes with a HEIDI-test *P*-value >0.05. **Panel C.** The TSMR validation of cis-eQTL genes that passed both the SMR and HEIDI tests in **Panels A–B**. Chr – chromosome, HEIDI – heterogeneity in dependent instruments, SMR – summary-based Mendelian randomisation.

After the SMR test, a total of 900 cis-eQTL genes were considered causally associated with sepsis, with an SMR-test *P* < 0.05 (**Figure 2**, Panel A). The 900 genes were forwarded to the HEIDI test, 796 of which yielded a HEIDI-test *P*-value >0.05 (**Figure 2**, Panel B). We further excluded 119 cis-eQTL genes without a gene symbol name (Table S2 in the **Online Supplementary Document**). Finally, 677 genes were included for further TSMR validation (**Figure 1**).

### Subsequent TSMR validation filtered 72 cis-eQTL genes causally associated with sepsis

Those 677 cis-eQTL genes were forwarded to further TSMR validation and a Bonferroni-corrected *P*-value (0.05 / 677) was used to deduce a causal association (**Figure 1**). We identified a total of 72 genes that exhibited a consistent causal association with sepsis (**Figure 2**, Panel C). The TSMR results, heterogeneity test, and pleiotropy test are presented in Table S3 in the **Online Supplementary Document**.

### The mediating MR analysis narrowed down to 50 cis-eQTL genes with sepsis-mediated effect on ARDS

**Figure 3:**
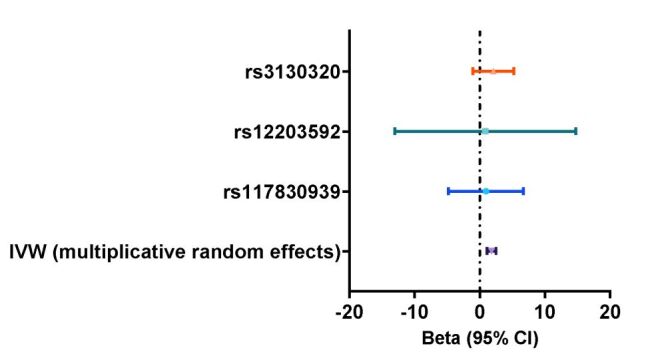
The MR analysis indicated a positive causal effect of sepsis on ARDS. ARDS – adult respiratory distress syndrome, CI – confidence interval, IVW – inverse variance weighted, MR – Mendelian randomisation.

The causal effect of sepsis on ARDS was calculated. The random-effect IVW method indicated a positive causal effect of sepsis on ARDS (beta = 1.80, standard error (SE) = 0.36, *P* < 0.001) (**Figure 3**; Table S4 in the **Online Supplementary Document**).

The total MR effect of the 72 cis-eQTL genes mentioned above on ARDS was investigated. Based on a Bonferroni-corrected *P*-value (0.05 / 72), a total of 8 cis-eQTL genes were thought significantly associated with the ARDS phenotype (Figure S1 and Table S5 in the **Online Supplementary Document**).

Furthermore, the direct effect of the 72 cis-eQTL genes on ARDS was calculated, which was derived from the MVMR analysis (the effect of both cis-eQTL genes and sepsis on ARDS). Based on a Bonferroni-corrected *P*-value (0.05 / 72), a total of 2 cis-eQTL genes were thought significantly associated with the ARDS phenotype (Table S6 in the **Online Supplementary Document**).

Focusing on the cis-eQTL genes that only yielded significant mediating effects, 50 cis-eQTL genes were filtered as a result of their significant association with sepsis (Figure S2 in the **Online Supplementary Document**). The mediating effects of these 50 genes on ARDS are described in **Table 1**.

**Table 1:** The mediation effect of sepsis in the relationship between 50 cis-eQTL genes and ARDS

Gene	Ensembl ID	Mediation estimate	Mediation SE	Mediation 95% LCI	Mediation 95% UCI
THOC6	ENSG00000131652	0.528537929	0.146587279	0.270349911	0.842467754
SCRT2	ENSG00000215397	0.528369276	0.159124611	0.250335962	0.871043373
LINC00863	ENSG00000224914	0.428609697	0.102347927	0.242730276	0.643212118
PSMA4	ENSG00000041357	0.408480231	0.087526865	0.243171387	0.586442599
RNF8	ENSG00000112130	0.313543069	0.080215658	0.170336067	0.483764696
JAKMIP3	ENSG00000188385	0.307312411	0.09005709	0.149626611	0.500961309
ALDH4A1	ENSG00000159423	0.301374235	0.0650878	0.178857672	0.434097964
WNK2	ENSG00000165238	0.280059593	0.085178087	0.13132544	0.463575069
LINC00908	ENSG00000266256	0.273575672	0.066563915	0.153316475	0.413666391
EVI5L	ENSG00000142459	0.217545779	0.048960468	0.126874325	0.318720419
LSM2	ENSG00000204392	0.206760981	0.052036423	0.113529442	0.316914886
TSPAN31	ENSG00000135452	0.200308695	0.045613421	0.116192414	0.294875944
GLIPR2	ENSG00000122694	0.196960348	0.063245409	0.08679654	0.333484278
LOC100129534	ENSG00000269896	0.192085843	0.054037444	0.097075309	0.307950314
OSGIN2	ENSG00000164823	0.180425923	0.048057425	0.095274538	0.282930021
AGAP1	ENSG00000157985	0.172155512	0.054303271	0.077508216	0.289315451
TATDN3	ENSG00000203705	0.166987385	0.037033471	0.098021989	0.243179188
WIPI2	ENSG00000157954	0.152771276	0.032239801	0.091470689	0.217930376
COPS6	ENSG00000168090	0.133873367	0.036258185	0.069798456	0.211348938
TPK1	ENSG00000196511	0.133637607	0.028207504	0.08000875	0.19065172
WASHC2A	ENSG00000099290	0.111529997	0.032153266	0.055146036	0.1805964
ENGASE	ENSG00000167280	0.105496715	0.027700751	0.056292667	0.164481726
ZC3H12C	ENSG00000149289	0.102334481	0.031776109	0.046911909	0.170854345
CCDC88B	ENSG00000168071	0.081069052	0.022234108	0.041848686	0.128636906
SLC12A7	ENSG00000113504	0.063459851	0.019910433	0.028749466	0.106409254
C2CD2L	ENSG00000172375	−0.096409301	0.028390227	−0.157474144	−0.046719791
BEGAIN	ENSG00000183092	−0.105020811	0.028333391	−0.165538596	−0.054920787
TM2D1	ENSG00000162604	−0.108036915	0.029122406	−0.170234451	−0.056534997
SLC66A3	ENSG00000162976	−0.120165064	0.027687981	−0.177745918	−0.069312574
COPB1	ENSG00000129083	−0.123983838	0.037988977	−0.205857229	−0.057678281
GINS2	ENSG00000131153	−0.128118848	0.035620656	−0.204420064	−0.065399074
EMB	ENSG00000170571	−0.138703792	0.037213028	−0.218140794	−0.072844923
RMC1	ENSG00000141452	−0.148978829	0.03831752	−0.230351253	−0.0806463
PCNX4	ENSG00000126773	−0.159968845	0.035096835	−0.23192693	−0.094331707
NDUFV3	ENSG00000160194	−0.177345736	0.045689535	−0.274395854	−0.095893887
KCNK13	ENSG00000152315	−0.179201351	0.052756082	−0.292673162	−0.086863824
RNFT1	ENSG00000189050	−0.184077345	0.05009639	−0.291173922	−0.095611102
PLXNB2	ENSG00000196576	−0.185289721	0.058012307	−0.31042083	−0.08414605
TUBGCP2	ENSG00000130640	−0.186409733	0.047527998	−0.287217056	−0.101498301
ITIH2	ENSG00000151655	−0.188967611	0.039539804	−0.268594601	−0.113494395
LINC01857	ENSG00000224137	−0.191983976	0.058853657	−0.318827339	−0.089264328
RPS18	ENSG00000231500	−0.197442023	0.040076805	−0.277006452	−0.119824302
PDK2	ENSG00000005882	−0.293512406	0.059998715	−0.413049774	−0.177713565
ZNF790-AS1	ENSG00000267254	−0.327953418	0.070443946	−0.471313316	−0.195048112
LOC105379362	ENSG00000247134	−0.339512189	0.074451414	−0.492132876	−0.200247515
GAMT	ENSG00000130005	−0.385787966	0.077663542	−0.539307778	−0.234752819
TIGD6	ENSG00000164296	−0.462131371	0.096682522	−0.656822392	−0.277572014
PKD1P3	ENSG00000183458	−0.471124831	0.093480737	−0.654422114	−0.287925984
MAP3K3	ENSG00000198909	−0.630369271	0.127025479	−0.881596474	−0.383460432
ERVH48-1	ENSG00000233056	−0.660367865	0.131184623	−0.917770773	−0.403505101

ARDS – adult respiratory distress syndrome, cis-eQTL – cis-expression quantitative trait locus, LCI – lower confidence interval, SE – standard error, UCI – upper confidence interval

### The drug target analysis confirmed the role of the PSMA4 target as a druggable gene for sepsis-related ARDS

Three mainstream drug target databases were gone through: DrugBan, ChEMBL, and DGIdb. Among the 50 cis-eQTL genes, 4 gene targets were reported simultaneously in the three databases: PSMA4, PDK2, RPS18, and NDUFV3 (**Table 2**). However, upon integrating the causal roles of cis-eQTL genes in ARDS with the analysis of their corresponding drug mechanisms, we just discovered the PSMA4 gene as a promising candidate for a druggable target in ARDS.

**Table 2:** The druggable targets across the mainstream database

Target	Causality*	Drug bank			ChEMBL			DGIdb		
		**Accession ID**	**Chemical formula**	**Drug mechanism**	**Accession ID**	**Chemical formula**	**Drug mechanism**	**Accession ID**	**Chemical formula**	**Drug mechanism**
PSMA4	Positive	DB08515	C15H21NO4	Unknown	CHEMBL371405	C15H20ClNO4	Inhibitor	IXAZOMIB	C14H19BCl2N2O4	Inhibitor
					CHEMBL3545432	C20H23BCl2N2O9	Inhibitor	BORTEZOMIB	C19H25BN4O4	Inhibitor
					CHEMBL451887	C40H57N5O7	Inhibitor	CARFILZOMIB	C40H57N5O7	Inhibitor
					CHEMBL2141296	C14H19BCl2N2O4	Inhibitor	OPROZOMIB	C25H32N4O7S	Inhibitor
					CHEMBL325041	C19H25BN4O4	Inhibitor	MARIZOMIB	C15H20ClNO4	Inhibitor
					CHEMBL5315122	C25H35BN4O8	Inhibitor	COTININE	C10H12N2O	Inhibitor
					CHEMBL2103884	C25H32N4O7S	Inhibitor	IXAZOMIB CITRATE	C20H23BCl2N2O9	Inhibitor
PDK2	Negative	DB08608	C18H20F3N3O3	Unknown	CHEMBL306823	C2HCl2NaO2	Inhibitor	AZD7545	C19H18ClF3N2O5S	Inhibitor
		DB08609	C19H21F3N2O4S	Unknown				DEVIMISTAT	C22H28O2S2	Inhibitor
		DB08610	C20H25ClN2O2	Unknown				VER-246608	C28H23ClF2N4O4	Inhibitor
								SODIUM DICHLOROACETATE	C2HCl2NaO2	Inhibitor
RPS18	Negative	DB11638	C15H24O5	Unknown	CHEMBL256997	C15H9FN2O3	Modulator	CYCLOHEXIMIDE	C15H23NO4	Inhibitor
					CHEMBL4297744	C19H38N4O10	Modulator	EXALUREN	C19H38N4O10	Modulator
					CHEMBL123292	C15H23NO4	Inhibitor	ATALUREN	C15H9FN2O3	Modulator
					CHEMBL4297789	Unknown	Inhibitor	MT-3724	Unknown	Inhibitor
					CHEMBL2109124	Unknown	Inhibitor	DORLIMOMAB ARITOX	Unknown	Inhibitor
NDUFV3	Negative	DB00157	C21H29N7O14P2	Unknown	CHEMBL1703	C4H12ClN5	Inhibitor	NV-128	Unknown	Inhibitor
		DB09270	C59H90O4	Cofactor	CHEMBL3545135	Unknown	Inhibitor	ME-344	C22H20O4	Inhibitor
					CHEMBL5314386	C22H20O4	Inhibitor	METFORMIN HYDROCHLORIDE	C4H12ClN5	Inhibitor

ARDS – adult respiratory distress syndrome

*The sepsis-mediated effect of gene targets on ARDS.

## DISCUSSION

In this study, we found 50 cis-eQTL genes with causal impacts on sepsis-mediated ARDS. The number was narrowed down to 4 cis-eQTL genes after drug target analysis across the DrugBan, ChEMBL, and DGIdb databases (PSMA4, PDK2, RPS18, and NDUFV3). In our recent investigation, the PSMA4 gene emerged as a promising candidate for a drug target in ARDS. Several inhibitors have been developed on the target (IXAZOMIB, BORTEZOMIB, CARFILZOMIB, OPROZOMIB, MARIZOMIB, COTININE, and IXAZOMIB CITRATE). Conversely, to date, no agonists have been formulated to specifically target the PDK2, RPS18, and NDUFV3 genes. The databases only provided antagonists on these targets. From this point of view, we should be cautious about utilising those drugs among patients with sepsis or sepsis-mediated ARDS.

Sepsis often triggers the malfunction of crucial organs, with a notable increase in vulnerability particularly in the lungs. ARDS, a severe pathological respiratory condition, is commonly viewed as a prevalent clinical complication during sepsis. Despite considerable research endeavors to elucidate the pathogenesis of sepsis-related ARDS and to discover new therapeutic targets and approaches, the rates of morbidity and mortality associated with this condition have persistently remained high [4–7,32–36]. This underscores the need for ongoing research to uncover effective treatment strategies and preventative measures for sepsis-mediated ARDS. Thus, we conducted such a study to explore the potential drug target for sepsis-related ARDS. As the ARDS disease usually follows sepsis in the clinical scenario, we paid attention to the genetic susceptibility of sepsis-mediated ARDS. That was to say, genes that yielded a significant total effect or direct effect on ARDS were not within the scope of our study. Based on such criteria, we identified 50 cis-eQTL genes with causal impacts on sepsis-mediated ARDS. Additional research is imperative to substantiate our discoveries and to pave the way for the development of novel pharmaceuticals aimed at these specific genes.

In our study, we identified the significant role of PSMA4 in the development of sepsis-related ARDS. Furthermore, we have discovered existing inhibitors that target PSMA4, which could potentially be leveraged for therapeutic purposes. PSMA4, as a vital component of the proteasome complex that consists of the 19S regulatory particle and the 20S core particle, is essential for the process of protein degradation [37]. This function is critical for maintaining cellular homeostasis by ensuring that damaged or unneeded proteins are effectively broken down and recycled. Indeed, the PSMA4 gene has been reported to impact a series of inflammatory disorders, including nonalcoholic steatohepatitis [38] and ankylosing spondylitis arthritis [39]. Moreover, it was recognised that the proteasome inhibitor (BORTEZOMIB) (**Table 2**), which targeted components like PSMA4, had the potential to modulate excessive inflammation and regulate the expression of cytokines that were triggered by a variety of stimuli [40–42]. This regulatory effect could be beneficial in managing inflammatory responses that are often dysregulated in conditions such as sepsis and ARDS, where an overactive immune response can lead to tissue damage and organ dysfunction. Additionally, we also provided several other proteasome inhibitors targeting PSMA4 (**Table 2**). These novel insights could serve as a valuable guide for future research endeavors focusing on sepsis and ARDS. We must also be cautious about the potential challenges in the clinical application of these drugs. For example, the common side effects of IXAZOMIB include thrombocytopenia and diarrhea, which should be avoided in certain patient populations.

We assumed that another three cis-eQTL genes (PDK2, RPS18, and NDUFV3), which were negatively associated with sepsis-induced ARDS, could be targeted with potential drugs in ARDS treatment. However, only inhibitors were found across the three databases. The application of these inhibitors in patients suffering from sepsis and ARDS should be approached with caution, as there might be a potential risk of worsening the conditions. The literature review yielded conflicting results on the role of PDK2, RPS18, and NDUFV3 genes in inflammatory diseases [43–50]. We anticipate conducting further research to explore the role of these targets in sepsis-related ARDS and to develop agonists that can modulate their activity.

Our results provided potential drug targets for sepsis-related ARDS through a comprehensive analysis, including SMR, TSMR, mediating MR, and MVMR methods with a Bonferroni-corrected *P*-value. Additionally, the filtered cis-eQTL genes were examined across the three mainstream databases, and we finally discovered PSMA4 as a promising druggable target for sepsis-related ARDS treatment. We have successfully navigated around the constraints typically encountered in conventional new drug development processes and our results would provide significant understanding to guide future pharmaceutical development initiatives targeting genes found in our study.

Several limitations should be admitted. We only extracted the summary statistics of GWAS data, which prevented us from validating the primary sources of data and developing a multigene predictor set. Our study exclusively included participants of European ancestry, which restricted the generalisability of our findings to other ethnic groups. The lack of cellular and animal studies impeded the validation of our research findings. Individual samples in the real-world clinical setting should also be collected to test our hypothesis. Despite these challenges, we conducted a series of sensitivity analysis including the heterogeneity and pleiotropy tests to ensure the robustness of the research results.

## CONCLUSIONS

We discovered potential drug targets for sepsis-related ARDS through a comprehensive analysis, among which four druggable targets (PSMA4, PDK2, RPS18, and NDUFV3) should be dialectically treated. Preclinical studies and randomised control trials would be needed to assess their effectiveness and safety. Also, the development of agonists for underexplored targets (like PDK2, RPS18, and NDUFV3) will be impactful.

## Additional material


Online Supplementary Document
