# Supplementary material for: Identifying potential drug targets for sepsis-related adult respiratory distress syndrome through comprehensive genetic analysis and druggability assessment
Source: J Glob Health. 2025 Mar 21;15:04117. doi: 10.7189/jogh.15.04117 (PMC11927037; doi:10.7189/jogh.15.04117)
Supplement: Online Supplementary Document [file jogh-15-04117-s001.pdf]

Supplement to: Weng J, Wang X, Lin J, Ye Y, Wei J, Yu R, Shang X. Identifying potential drug targets for sepsis-related adult respiratory distress syndrome through comprehensive genetic analysis and druggability assessment. J Glob Health. 2025;15:04117.

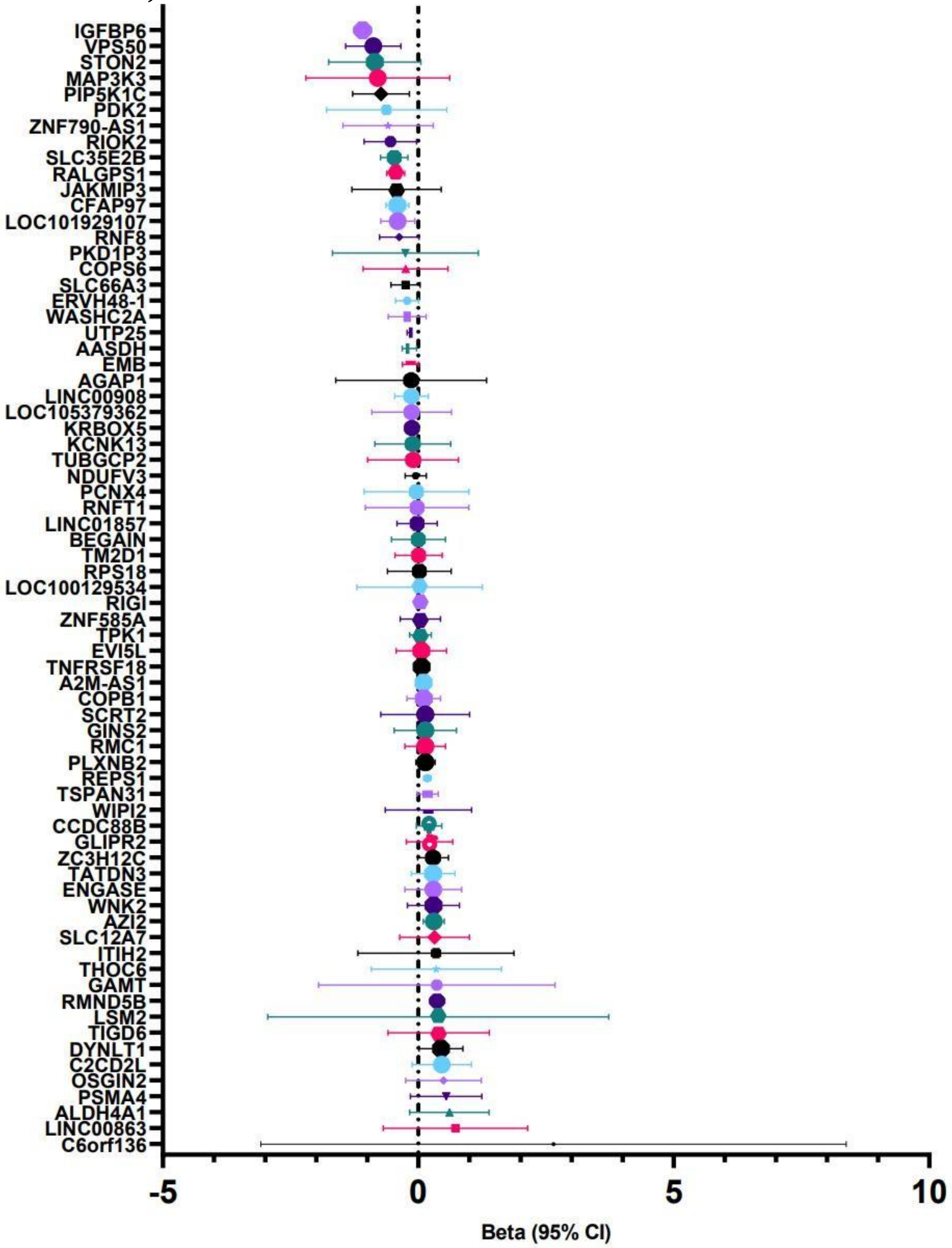

Figure S1. The MR effect of 72 cis-eQTL genes on ARDS.

The effect size was calculated by the IVW (multiplicative random effects) method.

IVW: inverse variance weighted; CI: confidence interval; MR: Mendelian randomization; cis-eQTL: cis-expression quantitative trait locus.

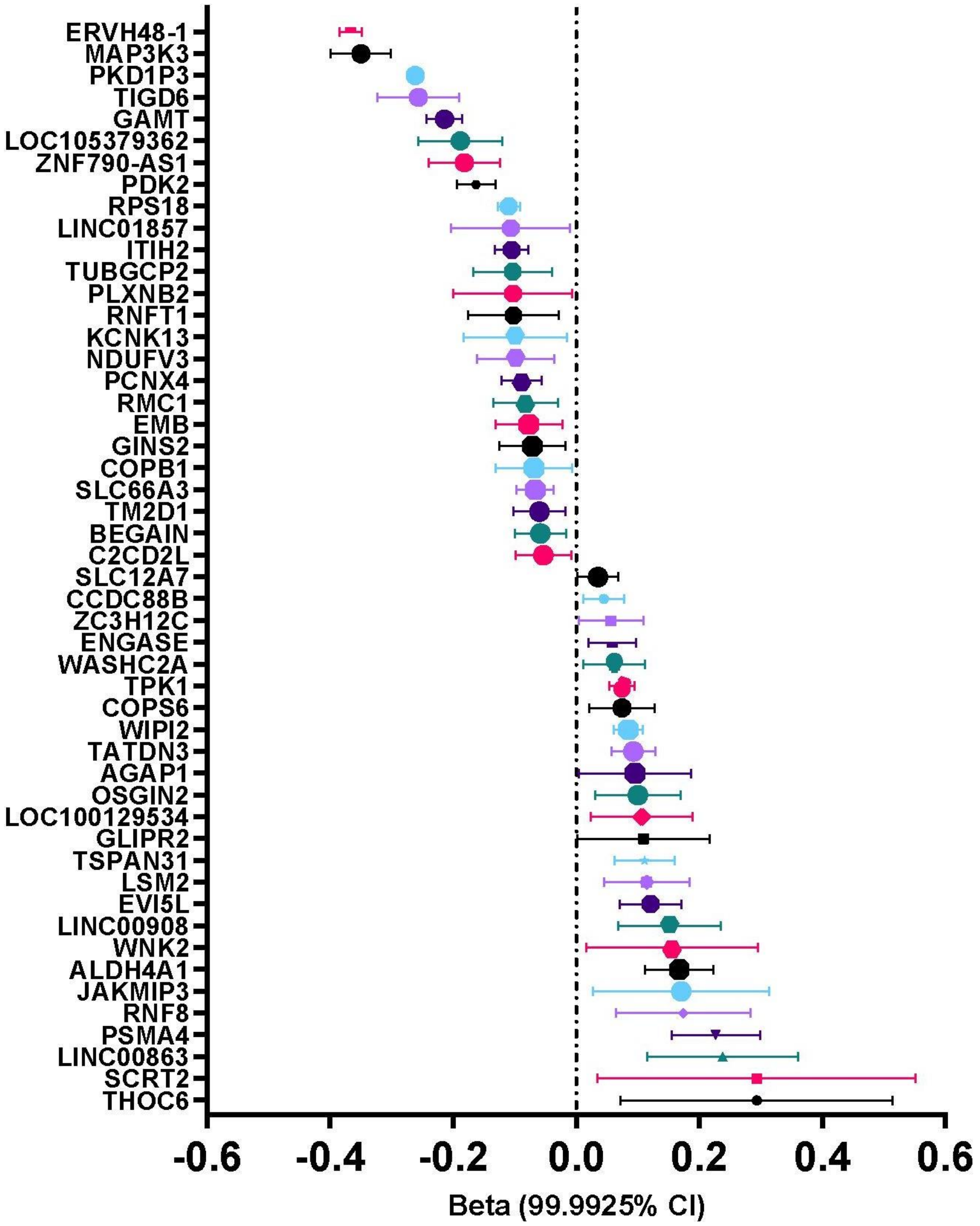

Figure S2. The MR analysis indicated significant associations between the 50 cis-eQTL genes and sepsis.

The effect size was calculated by the IVW (multiplicative random effects) method. IVW: inverse variance weighted; CI: confidence interval; MR: Mendelian randomization; cis-eQTL: cis-expression quantitative trait locus.

Table S1. Information about the GWAS data included in this study.

Trait	Population	Sex	Sample size	Consortium	SNPs	Year	publication DOI
16,989 cis-eQTL genes	European	Males and females	31,684	eQTLGen	11,000,000	2021	10.1038/s41588-021-00913-z
Sepsis	European	Males and females	462,918	UK Biobank	12,321,875	2020	10.7554/eLife.34408
ARDS	European	Males and females	216,528	FinnGen	16,380,461	2021	10.1038/s41586-022-05473-8

GWAS: genome-wide association study; SNP: single nucleotide polymorphism; ARDS: adult respiratory distress syndrome; cis-eQTL: cis-expression quantitative trait locus.

Table S2. The SMR results of the causal role of cis-eQTL genes in sepsis.

ENSEMBL	SYMBOL	Chr	bp	alt	ref	Freq_alt	b_GWAS	se_GWAS	p_GWAS	b_eQTL	se_eQTL	p_eQTL	b_SMR	se_SMR	p_SMR	p_HEIDI
ENSG00000000419	DPM1	20	49563248	A	G	0.347913	0.0220033	0.0145682	0.08300036	-0.0741559	0.00824705	2.43E-19	-0.296717	0.199206	0.1363561	0.6925524
ENSG00000000457	SCYL3	1	169842606	G	T	0.473161	0.00487624	0.0142346	0.84	-0.192913	0.00787703	1.86E-132	-0.0252769	0.0737948	0.7319528	0.8611465
ENSG00000000460	FIRRM	1	169727233	G	C	0.0964215	-0.0110723	0.024313	0.6999999	0.453899	0.0150365	3.56E-200	-0.0243938	0.0535709	0.648854	0.9964094
ENSG00000000938	FGR	1	27950181	T	C	0.0894632	0.0344206	0.0260436	0.2200002	0.194742	0.0174307	5.57E-29	0.176749	0.134666	0.1893511	0.8809153
ENSG00000000971	CFH	1	196668821	G	C	0.39662	-0.024753	0.0143454	0.1100001	-0.102882	0.00808786	4.54E-37	0.240595	0.140712	0.08729507	0.5024917
ENSG00000001036	FUCA2	6	143824720	C	T	0.238569	-0.013337	0.0171068	0.4100001	-0.175958	0.00956877	1.62E-75	0.0757964	0.0973081	0.4360199	0.9455917
ENSG00000001084	GCLC	6	53421953	C	T	0.411531	0.0118035	0.0144923	0.4100001	-0.142703	0.00798096	1.68E-71	-0.0827139	0.101661	0.4158608	0.9361187
ENSG00000001167	NFYA	6	41054199	A	G	0.132207	-0.016125	0.0231199	0.56	0.381592	0.0129413	4.31E-191	-0.0422572	0.0606049	0.4856425	0.540882
ENSG00000001460	STPG1	1	24713456	T	C	0.49006	-0.00263128	0.0142553	0.9599999	0.0950473	0.00884161	5.93E-27	-0.0276839	0.150003	0.8535778	0.2952897
ENSG00000001461	NIPAL3	1	24770875	G	A	0.237575	-0.0278172	0.0166658	0.07599937	0.251792	0.00916678	4.25E-166	-0.110477	0.0663108	0.09570432	0.6806516
ENSG00000001561	ENPP4	6	46106083	A	T	0.465209	0.00210833	0.0142141	0.7899998	-0.448988	0.00757903	0	-0.00469573	0.0316582	0.8820853	0.4989201
ENSG00000001629	ANKIB1	7	91953123	T	C	0.0735586	-0.0202368	0.027955	0.32	0.464863	0.0167768	5.49E-169	-0.0435329	0.0601566	0.4692749	0.3016381
ENSG00000001630	CYP51A1	7	91756865	T	A	0.388668	0.00209052	0.0145294	0.95	-0.466317	0.0161573	3.69E-183	-0.00448304	0.0311582	0.885595	0.5782108
ENSG00000001631	KRIT1	7	91851881	T	G	0.12326	0.000686084	0.0204832	0.8800001	0.184966	0.0122601	1.98E-51	0.00370924	0.110741	0.9732799	0.6245488
ENSG00000002016	RAD52	12	1060799	G	C	0.187873	-0.00967225	0.0180447	0.5700002	-0.227891	0.010932	1.65E-96	0.0424425	0.0792076	0.5920698	0.9480296
ENSG00000002330	BAD	11	64044739	T	C	0.139165	-0.0122373	0.0189229	0.6100002	0.0907893	0.0111022	2.90E-16	-0.134788	0.209077	0.5191346	0.06852454
ENSG00000002549	LAP3	4	17594205	G	A	0.346918	0.0208007	0.014648	0.16	0.786803	0.00744609	0	0.026437	0.0186188	0.1556343	0.9819796
ENSG00000002587	HS3ST1	4	11413081	A	G	0.4334	0.00327985	0.0142976	0.84	0.153322	0.00800811	1.05E-81	0.0213919	0.093259	0.8185719	0.9823071
ENSG00000002726	AOC1	7	150540153	G	A	0.373757	-0.000126326	0.0147444	0.9699999	0.66937	0.00800538	0	-0.000188724	0.0220273	0.993164	0.1923357
ENSG00000002745	WNT16	7	120973289	A	C	0.0586481	-0.0104074	0.0286003	0.7199992	-0.129318	0.0174467	1.24E-13	0.0804791	0.221429	0.7162661	0.9804862
ENSG00000002822	MAD1L1	7	2064153	A	G	0.346918	0.00739688	0.0149198	0.83	0.263946	0.00825058	1.45E-224	0.0280242	0.0565328	0.6200944	0.5806314
ENSG00000002834	LASP1	17	37052067	T	A	0.215706	-0.0149988	0.0172758	0.56	0.105483	0.0100277	7.05E-26	-0.142192	0.164335	0.3868988	0.4373646
ENSG00000002919	SNX11	17	46190577	A	G	0.230616	0.0327191	0.0176168	0.0990011	-0.529423	0.00894807	0	-0.0618014	0.0332919	0.06340332	0.136737
ENSG00000002933	TMEM176A	7	150499849	G	A	0.373757	-0.000126326	0.0147444	0.9699999	1.06103	0.00569462	0	-0.00011906	0.0138963	0.993164	0.1877466
ENSG00000003056	M6PR	12	9097755	A	G	0.33996	0.0273067	0.0149617	0.04900044	0.374867	0.00805955	0	0.0728438	0.0399428	0.06819694	0.0684313
ENSG00000003137	CYP26B1	2	72365767	G	A	0.150099	0.0181811	0.0190623	0.2099999	0.917831	0.0100948	0	0.0198088	0.02077	0.3402253	0.49195
ENSG00000003147	ICA1	7	8227565	T	C	0.397614	-0.00124564	0.0144955	0.81	-0.102427	0.00808259	8.39E-37	0.0121612	0.141523	0.9315213	0.8551909
ENSG00000003249	DBNDD1	16	90078904	T	C	0.0457256	-0.0129539	0.0294342	0.89	0.852871	0.0161445	0	-0.0151886	0.0345131	0.659878	0.4231925
ENSG00000003393	ALS2	2	202605594	A	G	0.202783	-0.0216608	0.0192419	0.2300001	-0.795639	0.00920125	0	0.0272244	0.0241862	0.2603287	0.9684489
ENSG00000003400	CASP10	2	202070866	T	C	0.480119	0.00817969	0.0142502	0.6100002	-0.118177	0.00796202	7.77E-50	-0.0692156	0.120674	0.5662544	0.3304516
ENSG00000003402	CFLAR	2	202004930	C	T	0.510934	0.000656224	0.0142046	0.8200001	0.285737	0.00867575	6.89E-238	0.0022966	0.0497121	0.9631525	0.5112965
ENSG00000003436	TFPI	2	188379722	G	A	0.0447316	-0.0395926	0.0381275	0.4	0.141212	0.0222017	2.01E-10	-0.280377	0.273577	0.3054305	0.9170106
ENSG00000003509	NDUFAF7	2	37469660	T	C	0.413519	-0.0273888	0.0145923	0.01700004	0.17105	0.00898683	9.02E-81	-0.160122	0.085724	0.06177949	0.4948187
ENSG00000004059	ARF5	7	127230079	C	T	0.375746	-0.0224444	0.0149397	0.0659994	-0.0702231	0.00822747	1.40E-17	0.319616	0.216017	0.1389834	0.03315873
ENSG00000004139	SARM1	17	26709721	G	T	0.500994	-0.0191441	0.0141993	0.1299999	-0.218767	0.00784953	6.15E-171	0.087509	0.0649819	0.1780873	0.0153283
ENSG00000004142	POLDIP2	17	26679102	A	G	0.0795229	0.0286259	0.0264548	0.3100002	0.388687	0.0143576	2.12E-161	0.0736477	0.0681163	0.279606	0.265603
ENSG00000004399	PLXND1	3	129299839	A	G	0.223658	0.0254163	0.0171915	0.1499999	-0.135909	0.00961505	2.31E-45	-0.18701	0.127183	0.1414531	0.7274484
ENSG00000004455	AK2	1	33510091	T	C	0.27833	-0.0193025	0.0161328	0.2399999	-0.126607	0.00887695	3.75E-46	0.15246	0.127872	0.2331486	0.264042
ENSG00000004468	CD38	4	15815483	G	A	0.210736	-0.0031708	0.0172015	0.8	0.0855691	0.0100111	1.26E-17	-0.0370554	0.201071	0.853786	0.6302257
ENSG00000004478	FKBP4	12	2909347	C	T	0.0367793	0.0160084	0.0368274	0.5199996	-0.165045	0.0187995	1.65E-18	-0.0969944	0.223409	0.6641756	0.53464
ENSG00000004487	KDM1A	1	23378061	T	C	0.158052	0.0142041	0.0199124	0.4100001	-0.201576	0.0210911	1.21E-21	-0.0704653	0.0990584	0.4768669	0.999101
ENSG00000004534	RBM6	3	50057459	A	C	0.478131	-0.0293824	0.0142116	0.016	0.754537	0.00740638	0	-0.038941	0.0188387	0.0387277	0.6512358
ENSG00000004660	CAMKK1	17	3780897	C	T	0.220676	0.0315574	0.0183667	0.1199999	0.197769	0.0129613	1.45E-52	0.159567	0.0934565	0.0877487	0.685256
ENSG00000004700	RECQL	12	21638224	G	T	0.228628	0.0189636	0.0178531	0.33	-0.306949	0.00993695	1.64E-209	-0.0617809	0.0581974	0.2884288	0.6922176
ENSG00000004766	VPS50	7	92924995	A	G	0.0506958	0.0612302	0.030002	0.04900044	-0.213474	0.0172243	2.82E-35	-0.286828	0.142434	0.04403568	0.6785084
ENSG00000004777	ARHGAP33	19	36272579	G	A	0.366799	-0.00396662	0.0145781	0.7400005	-0.437367	0.00777843	0	0.00906931	0.0333319	0.7855519	0.01484773
ENSG00000004779	NDUFAB1	16	23600000	C	T	0.0884692	0.0296789	0.0249889	0.2	-0.085254	0.0142704	2.31E-09	-0.348123	0.298847	0.2440648	0.152228
ENSG00000004799	PDK4	7	95219307	C	T	0.45825	0.0284239	0.0145054	0.07599937	-0.169701	0.00800635	1.04E-99	-0.167494	0.0858407	0.05103106	0.6270156

ENSG00000004809	SLC22A16	6	110771867	C	G	0.111332	-0.0038854	0.0232598	0.7899998	1.01488	0.01116	0	-0.00382843	0.0229188	0.867336	0.5990849
ENSG00000004838	ZMYND10	3	50381412	A	G	0.0109344	-0.339225	0.203536	0.04700023	0.317636	0.0435642	3.07E-13	-1.06797	0.657312	0.1042155	0.5153596
ENSG00000004864	SLC25A13	7	95850495	G	C	0.302187	0.0057284	0.0152223	0.6899999	0.324264	0.00827761	0	0.0176659	0.0469464	0.7066947	0.7775336
ENSG00000004866	ST7	7	116731724	C	T	0.23161	-0.0255149	0.0173943	0.14	-0.0776675	0.00954053	3.93E-16	0.328514	0.227565	0.1488497	0.6127638
ENSG00000004939	SLC4A1	17	42335631	C	T	0.28827	-0.00572691	0.0156003	0.6700003	-0.178167	0.0103529	2.26E-66	0.0321436	0.0875801	0.7136048	0.8020089
ENSG00000004975	DVL2	17	7133262	G	A	0.378728	-0.0108071	0.0146137	0.6499995	0.117543	0.00824075	3.69E-46	-0.0919418	0.124494	0.4601946	0.8742508
ENSG00000005001	PRSS22	16	2905449	T	C	0.459245	-0.00500885	0.0142887	0.6999999	-0.171833	0.00855717	1.09E-89	0.0291495	0.0831672	0.7259689	0.7098704
ENSG00000005020	SKAP2	7	26870769	C	T	0.50994	-0.0113182	0.0142256	0.4899999	0.809578	0.00658082	0	-0.0139804	0.017572	0.4262615	0.8526197
ENSG00000005059	MCUB	4	110545617	G	C	0.34493	-0.0156108	0.0149895	0.25	-0.446104	0.0152396	2.30E-188	0.0349936	0.0336221	0.2979728	0.3104772
ENSG00000005075	POLR2J	7	102116459	A	G	0.23161	0.0141448	0.0165679	0.4299995	-0.497386	0.00945453	0	-0.0284383	0.0333143	0.3933073	0.007110461
ENSG00000005100	DHX33	17	5358306	T	C	0.163022	-0.00125758	0.0204613	0.95	0.139439	0.0115437	1.36E-33	-0.00901886	0.146742	0.9509924	0.6927388
ENSG00000005102	MEOX1	17	41728539	A	G	0.418489	0.0220289	0.0145999	0.2099999	0.329023	0.00787281	0	0.0669525	0.0444024	0.1315912	0.8445013
ENSG00000005156	LIG3	17	33319798	A	G	0.477137	-0.0260983	0.0142481	0.08	-0.520419	0.00740533	0	0.0501487	0.0273875	0.0670883	0.9132161
ENSG00000005175	RPAP3	12	48078457	C	G	0.0785288	-0.0104667	0.0269345	0.5500004	-0.796144	0.0141378	0	0.0131467	0.033832	0.6975802	0.1810982
ENSG00000005187	ACSM3	16	20715234	A	G	0.126243	-0.0107279	0.0208752	0.6100002	-0.223423	0.0114526	9.32E-85	0.0480162	0.0934661	0.607442	0.985641
ENSG00000005189	REXO5	16	20839369	C	T	0.0437376	-0.0136903	0.0380674	0.6499995	-0.234181	0.0236959	4.94E-23	0.0584602	0.162663	0.7192994	0.993453
ENSG00000005194	CIAPIN1	16	57471760	G	A	0.133201	0.0166899	0.0215391	0.28	-0.139807	0.0182344	1.76E-14	-0.119378	0.154848	0.4407436	0.4291918
ENSG00000005206	SPPL2B	19	2341858	G	T	0.122266	0.0194979	0.0221002	0.33	0.120156	0.0163379	1.92E-13	0.162272	0.185249	0.3810466	0.409441
ENSG00000005238	ATOSB	9	35110054	T	C	0.0168986	0.0101602	0.0626449	0.9400001	-0.342816	0.0394722	3.79E-18	-0.0296375	0.182768	0.8711807	0.7448839
ENSG00000005243	COPZ2	17	46109462	C	T	0.0785288	-0.00176453	0.023816	0.91	0.365651	0.0135897	1.84E-159	-0.00482572	0.0651333	0.9409389	0.08819074
ENSG00000005249	PRKAR2B	7	106743675	G	A	0.513917	0.00202439	0.0142536	0.98	-0.10717	0.00792725	1.20E-41	-0.0188895	0.133007	0.8870652	0.6464198
ENSG00000005379	TSPOAP1	17	56392372	G	A	0.213718	0.0193679	0.0181475	0.2599998	0.140844	0.0106985	1.40E-39	0.137513	0.129271	0.2874378	0.1629144
ENSG00000005381	MPO	17	56352756	C	T	0.172962	-0.00379341	0.0193098	0.8800001	0.409196	0.0111353	1.27E-295	-0.00927039	0.0471903	0.8442602	0.1311927
ENSG00000005436	GCFC2	2	75908620	G	A	0.240557	-0.011355	0.0163389	0.4600002	0.259856	0.0174806	5.53E-50	-0.0436973	0.0629454	0.4875511	0.05899034
ENSG00000005469	CROT	7	87002054	G	A	0.0218688	-0.0112192	0.0388247	0.6600001	-0.510144	0.0236121	1.60E-103	0.0219922	0.0761122	0.7726236	0.7318377
ENSG00000005471	ABCB4	7	87070382	G	A	0.191849	-0.00615984	0.0184386	0.9199999	-0.232152	0.0105812	1.08E-106	0.0265337	0.079434	0.7383536	0.3699042
ENSG00000005483	KMT2E	7	104704717	C	A	0.136183	-0.0334678	0.0219875	0.08100093	0.0767974	0.0128686	2.40E-09	-0.435793	0.295471	0.1402368	0.8931032
ENSG00000005486	RHBDD2	7	75495082	A	G	0.171968	-0.00687911	0.0192434	0.8499999	0.295034	0.0151592	2.29E-84	-0.0233163	0.0652353	0.7207784	0.7428373
ENSG00000005513	SOX8	16	1034393	C	G	0.383698	0.00671411	0.0146887	0.6200004	-0.135503	0.00821679	4.26E-61	-0.0495495	0.108443	0.6477299	0.6965556
ENSG00000005700	IBTK	6	82918585	C	T	0.486083	0.0340953	0.0141987	0.0129999	0.0821529	0.00796441	6.03E-25	0.415023	0.177454	0.01934787	0.7900427
ENSG00000005801	ZNF195	11	3380469	G	A	0.207753	-0.00358938	0.0170362	0.6700003	-0.370526	0.00900516	0	0.00968725	0.045979	0.8331303	0.2652562
ENSG00000005810	MYCBP2	13	77759988	A	G	0.0427435	0.0549949	0.0349035	0.0990011	-0.206346	0.023972	7.45E-18	-0.266518	0.171961	0.1211713	0.2017112
ENSG00000005812	FBXL3	13	77584035	T	C	0.00994036	0.119413	0.0683334	0.09200046	0.396344	0.0615958	1.24E-10	0.301286	0.178654	0.09171483	0.5027021
ENSG00000005844	ITGAL	16	30509242	G	C	0.441352	0.0160896	0.0142851	0.16	0.206377	0.0079162	7.94E-150	0.0779622	0.0692831	0.2604743	0.8245822
ENSG00000005882	PDK2	17	48180808	G	A	0.279324	0.035881	0.0155405	0.0179999	-0.173592	0.00979681	2.98E-70	-0.206698	0.0902801	0.02204942	0.942234
ENSG00000005884	ITGA3	17	48150588	A	G	0.0775348	0.037436	0.0251707	0.17	-0.158986	0.0127276	8.32E-36	-0.235468	0.159439	0.1397149	0.7077005
ENSG00000005955	NA	17	34923507	A	G	0.451292	0.0305014	0.0142338	0.032	-0.609276	0.0107207	0	-0.0500617	0.0233784	0.03224473	0.6833429
ENSG00000006007	GDE1	16	19523241	A	G	0.0745527	0.00991366	0.0275017	0.5500004	-0.859566	0.0148205	0	-0.0115333	0.0319955	0.7184976	0.8404904
ENSG00000006015	REX1BD	19	18701320	T	C	0.526839	0.0174962	0.0142242	0.2599998	0.509675	0.00898265	0	0.0343282	0.0279149	0.2187939	0.3532034
ENSG00000006025	OSBPL7	17	45891969	G	A	0.44831	-0.0156475	0.0142429	0.4400003	-0.115012	0.00794962	1.94E-47	0.136051	0.124195	0.2733135	0.1082604
ENSG00000006042	TMEM98	17	31263526	A	G	0.198807	0.000316745	0.0181469	0.9199999	0.222452	0.0102566	2.63E-104	0.00142388	0.0815767	0.986074	0.6501716
ENSG00000006047	YBX2	17	7194752	A	G	0.172962	0.0223763	0.0190704	0.29	0.182086	0.0109915	1.23E-61	0.122889	0.104995	0.2418319	0.5166667
ENSG00000006062	MAP3K14	17	43367451	A	G	0.475149	0.00278365	0.0142806	0.84	-0.0708162	0.00796171	5.86E-19	-0.0393081	0.201706	0.8454881	0.5748009
ENSG00000006075	NA	17	34416558	A	G	0.254473	-0.00422507	0.0169511	0.6100002	0.262637	0.0236104	9.61E-29	-0.0160871	0.064558	0.8032154	0.9267459
ENSG00000006114	NA	17	35922222	C	T	0.143141	0.0138109	0.0216599	0.5099998	0.173147	0.0136257	5.38E-37	0.079764	0.125253	0.5242394	0.9179457
ENSG00000006125	AP2B1	17	33979250	A	G	0.238569	-0.00328105	0.0169624	0.7700005	-0.227622	0.00964091	3.04E-123	0.0144145	0.0745226	0.8466267	0.3500243
ENSG00000006194	ZNF263	16	3332572	A	G	0.26839	-0.0207239	0.0164919	0.1800002	0.131299	0.00917892	2.05E-46	-0.157838	0.126089	0.2106464	0.1884572
ENSG00000006282	SPATA20	17	48626816	C	A	0.349901	0.0282047	0.0146935	0.03400008	-1.00849	0.00674709	0	-0.0279672	0.014571	0.05493657	0.2582465
ENSG00000006432	MAP3K9	14	71232747	G	A	0.406561	0.0115342	0.0144016	0.35	0.236275	0.00798316	1.65E-192	0.0488168	0.0609749	0.4233609	0.9567317
ENSG00000006451	RALA	7	39705402	T	C	0.356859	0.0132724	0.0144733	0.3900004	0.187669	0.0140288	8.20E-41	0.0707223	0.0773023	0.3602549	0.6747

ENSG00000006453	BAIAP2L1	7	97975671	A	T	0.181909	-0.000334809	0.0183072	0.9299999	-0.207596	0.0102892	1.58E-90	0.00161279	0.0881865	0.9854088	0.2795999
ENSG00000006459	KDM7A	7	139830690	G	A	0.536779	-0.0189355	0.014227	0.0990011	0.151694	0.00990144	5.59E-53	-0.124827	0.0941408	0.1848526	0.4595033
ENSG00000006530	AGK	7	141303016	T	C	0.457256	0.00315495	0.0143778	0.6800001	0.138743	0.00801723	4.27E-67	0.0227395	0.103637	0.8263273	0.5576902
ENSG00000006534	ALDH3B1	11	67786396	G	A	0.215706	0.0138557	0.016674	0.4100001	-0.211399	0.0094167	1.30E-111	-0.0655428	0.0789284	0.4063083	0.1931083
ENSG00000006555	TTC22	1	55256162	T	C	0.294235	0.00706691	0.0161877	0.95	0.0699751	0.0112972	5.87E-10	0.100992	0.231909	0.6632139	0.7327194
ENSG00000006576	PHTF2	7	77507470	T	C	0.0298211	0.0263704	0.0424221	0.6499995	-0.273531	0.0296131	2.54E-20	-0.0964075	0.155442	0.5351153	0.7630346
ENSG00000006607	FARP2	2	242364957	C	T	0.26839	0.00767343	0.0168115	0.7800007	0.316309	0.00938042	2.93E-249	0.0242593	0.0531539	0.6481041	0.8455609
ENSG00000006625	GGCT	7	30563666	C	T	0.205765	0.00881673	0.0172735	0.7199992	0.381743	0.00939142	0	0.023096	0.0452527	0.6097858	0.1392985
ENSG00000006638	TBXA2R	19	3600671	G	A	0.39662	0.00899419	0.0144419	0.3900004	0.10246	0.00928903	2.73E-28	0.0877825	0.141176	0.5340773	0.3977686
ENSG00000006652	IFRD1	7	112092047	T	C	0.253479	0.024987	0.0168567	0.1100001	-0.36237	0.0090186	0	-0.0689543	0.0465495	0.1385237	0.5174119
ENSG00000006695	COX10	17	14042403	T	C	0.0367793	-0.0540174	0.0356379	0.089	0.429324	0.0225926	1.62E-80	-0.12582	0.0832731	0.1308062	0.3983699
ENSG00000006715	VPS41	7	38867278	G	A	0.163022	-0.0120582	0.0195734	0.58	0.240451	0.0107666	1.77E-110	-0.0501482	0.0814337	0.5380159	0.2859661
ENSG00000006740	ARHGAP44	17	12793908	T	C	0.195825	0.0312702	0.0184812	0.0519996	0.20285	0.0111468	5.35E-74	0.154154	0.0915005	0.09203988	0.1672574
ENSG00000006744	ELAC2	17	12908606	C	T	0.393638	0.0121696	0.0146513	0.3900004	-0.356458	0.00797326	0	-0.0341403	0.0411095	0.4062718	0.6722105
ENSG00000006747	SCIN	7	12651715	T	G	0.45825	-0.00689165	0.0142188	0.6300007	-0.0889874	0.00815097	9.52E-28	0.0774452	0.159942	0.6282377	0.6107635
ENSG00000006831	ADIPOR2	12	1847792	G	A	0.348907	-0.00863346	0.0152755	0.4600002	0.190023	0.00841354	6.03E-113	-0.0454337	0.0804127	0.5720693	0.3345744
ENSG00000006837	CDKL3	5	133624021	G	C	0.111332	0.0637757	0.0272227	0.03099988	-0.0977949	0.0142098	5.89E-12	-0.652137	0.294051	0.02657067	0.1258048
ENSG00000007038	PRSS21	16	2871734	T	G	0.249503	-0.00881217	0.0160426	0.6700003	0.126581	0.0087318	1.27E-47	-0.0696166	0.126828	0.5830712	0.5210718
ENSG00000007047	MARK4	19	45695543	G	C	0.328032	-0.0162565	0.0154247	0.3900004	0.389357	0.0087167	0	-0.0417522	0.0396269	0.2920509	0.9200622
ENSG00000007080	CCDC124	19	18049312	A	G	0.346918	-0.0181999	0.0148342	0.1900002	-0.13229	0.00947159	2.48E-44	0.137576	0.112566	0.2216389	0.765641
ENSG00000007129	CEACAM21	19	42074541	T	A	0.22664	0.0297024	0.0174925	0.08300036	-0.737518	0.00965812	0	-0.0402735	0.0237239	0.08958553	0.8446021
ENSG00000007168	PAFAH1B1	17	2542706	G	A	0.429423	-0.00906877	0.0146591	0.5300002	0.148718	0.00894404	4.40E-62	-0.0609797	0.0986381	0.5364325	0.3982381
ENSG00000007171	NOS2	17	26105658	C	A	0.242545	0.00874388	0.0162913	0.4400003	0.156786	0.00963772	1.67E-59	0.0557694	0.103964	0.5916621	0.588415
ENSG00000007202	BLTP2	17	26956965	T	C	0.0129225	-0.0578135	0.0815102	0.28	-0.405106	0.0729921	2.86E-08	0.142712	0.202843	0.4817086	0.09898105
ENSG00000007237	GAS7	17	9957897	A	G	0.299205	-0.0110677	0.0156917	0.3800004	0.228215	0.00848181	1.85E-159	-0.0484968	0.0687821	0.4807606	0.5170408
ENSG00000007255	TRAPPC6A	19	45673840	C	G	0.233598	0.0271933	0.0171497	0.2099999	-0.240997	0.00907297	1.86E-155	-0.112837	0.0712881	0.1134613	0.1791507
ENSG00000007264	MATK	19	3790049	C	G	0.437376	-0.0289849	0.0142699	0.04499974	0.0927073	0.00805856	1.26E-30	-0.31265	0.156305	0.04547294	0.576677
ENSG00000007312	CD79B	17	62007907	A	C	0.395626	0.0290773	0.0145439	0.03799969	-0.107378	0.0082081	4.17E-39	-0.270793	0.137018	0.04811722	0.3064679
ENSG00000007314	SCN4A	17	62033096	C	T	0.166004	0.0349618	0.0203018	0.1199999	0.0813177	0.0129125	3.02E-10	0.429941	0.258827	0.09669009	0.8095278
ENSG00000007341	ST7L	1	113114793	T	C	0.481113	0.012812	0.0141883	0.5	0.474066	0.00748603	0	0.0270258	0.029932	0.3665759	0.6139984
ENSG00000007372	PAX6	11	31822924	T	A	0.466203	-0.0156281	0.0141789	0.2300001	0.0822242	0.00803425	1.39E-24	-0.190067	0.173439	0.2731352	0.3109157
ENSG00000007376	RPUSD1	16	836685	G	C	0.196819	0.00935813	0.0175855	0.6200004	-0.111769	0.0106838	1.30E-25	-0.0837273	0.157541	0.5950978	0.9548283
ENSG00000007392	LUC7L	16	259215	C	A	0.468191	0.00273988	0.0142541	0.9699999	0.147306	0.0100874	2.69E-48	0.0185999	0.0967735	0.8475853	0.49613
ENSG00000007402	CACNA2D2	3	50470954	A	G	0.10835	0.0077261	0.0223678	0.83	-0.142467	0.0128005	8.98E-29	-0.0542307	0.157079	0.7299098	0.1457533
ENSG00000007516	BAIAP3	16	1391520	G	C	0.279324	0.0041361	0.0154307	0.5500004	0.310138	0.0104586	3.03E-193	0.0133363	0.0497563	0.7886742	0.8997806
ENSG00000007520	TSR3	16	1400576	A	G	0.0258449	0.0198934	0.0498149	0.7800007	-0.424924	0.0323836	2.48E-39	-0.0468164	0.117287	0.6897742	0.1115243
ENSG00000007541	PIGQ	16	625565	A	G	0.308151	-0.00437878	0.0153047	0.7800007	0.2905	0.00974005	1.84E-195	-0.0150733	0.0526864	0.7748063	0.8371952
ENSG00000007545	CRAMP1	16	1695117	A	G	0.0735586	0.00953503	0.0273433	0.9199999	0.406183	0.0156007	1.93E-149	0.0234747	0.0673237	0.7273265	0.4888511
ENSG00000007944	MYLIP	6	16138917	C	T	0.0874751	-0.0223708	0.0260718	0.3599996	0.310919	0.0135513	1.70E-116	-0.0719505	0.0839125	0.3911982	0.3089411
ENSG00000007968	E2F2	1	23845317	T	C	0.0705765	0.0307966	0.0261185	0.2200002	0.113694	0.0151847	7.03E-14	0.270872	0.232557	0.2441177	0.2391086
ENSG00000008018	PSMB1	6	170853317	C	G	0.432406	-0.0146619	0.0144946	0.3100002	0.334229	0.00803308	0	-0.0438678	0.0433801	0.3118999	0.9012041
ENSG00000008083	JARID2	6	15384389	A	T	0.122266	-0.0229201	0.0202061	0.3900004	-0.512864	0.0114804	0	0.0446904	0.0394113	0.2568154	0.3572819
ENSG00000008128	CDK11A	1	1644967	T	G	0.0228628	-0.121048	0.0610349	0.032	0.916164	0.0887154	5.32E-25	-0.132125	0.0678375	0.05145478	0.4028275
ENSG00000008130	NADK	1	1697283	A	G	0.0775348	-0.0381589	0.031079	0.1499999	0.175216	0.0232363	4.68E-14	-0.217783	0.179712	0.2255721	0.1479699
ENSG00000008226	DLEC1	3	38123106	A	G	0.368787	0.0129003	0.0148843	0.4199997	-0.107622	0.00831265	2.45E-38	-0.119866	0.138611	0.3871652	0.2904884
ENSG00000008256	CYTH3	7	6256841	G	A	0.0258449	-0.0133477	0.0425865	0.6499995	-0.365017	0.0229918	9.29E-57	0.0365673	0.116693	0.7540037	0.8754023
ENSG00000008277	ADAM22	7	87697831	T	C	0.134195	-0.0256901	0.0204847	0.14	-0.0852073	0.0111706	2.39E-14	0.301501	0.243638	0.2159025	0.8952039
ENSG00000008282	SYPL1	7	105741985	T	C	0.254473	0.0198622	0.0163425	0.16	0.100604	0.00923448	1.23E-27	0.197429	0.163451	0.2270942	0.04540298
ENSG00000008283	CYB561	17	61516702	C	G	0.465209	-0.0060868	0.0143057	0.7499995	-0.119171	0.00795914	1.11E-50	0.0510764	0.120092	0.6706114	0.2695161
ENSG00000008294	SPAG9	17	49118880	T	C	0.0954274	0.000236341	0.0250992	0.8200001	0.238317	0.0144045	1.75E-61	0.000991708	0.105319	0.992487	0.4032987

ENSG00000008300	CELSR3	3	48687125	A	G	0.119284	-0.0196554	0.0225975	0.2	-0.258129	0.0132211	6.86E-85	0.0761457	0.0876303	0.3848787	0.5217793
ENSG00000008311	AASS	7	121750017	A	G	0.0775348	0.0240144	0.0228765	0.16	-0.457361	0.0136596	8.61E-246	-0.0525065	0.0500431	0.2940744	0.4260554
ENSG00000008323	PLEKHG6	12	6428637	A	G	0.367793	0.0226867	0.0145382	0.0519996	0.0722407	0.00831902	3.83E-18	0.314043	0.20447	0.1245661	0.3488291
ENSG00000008324	SS18L2	3	42629969	A	T	0.0904573	-0.0433719	0.0243783	0.09699961	0.109316	0.0141855	1.30E-14	-0.396756	0.228873	0.08300323	0.3609564
ENSG00000008382	MPND	19	4351803	G	A	0.480119	-0.0156821	0.0142337	0.25	-0.269111	0.0131879	1.48E-92	0.0582736	0.0529685	0.2712641	0.9865993
ENSG00000008394	MGST1	12	16631134	A	T	0.0586481	-0.0388667	0.0290234	0.17	-0.38738	0.0156365	1.71E-135	0.100332	0.0750317	0.1811579	0.9092692
ENSG00000008405	CRY1	12	107436374	A	G	0.426441	-0.0163069	0.0143339	0.17	-0.124341	0.00798809	1.24E-54	0.131147	0.115586	0.2565348	0.08268327
ENSG00000008438	PGLYRP1	19	46524367	T	C	0.272366	-0.013227	0.0152337	0.4299995	-0.41807	0.00818924	0	0.0316383	0.0364435	0.385314	0.5466997
ENSG00000008441	NFIX	19	13158016	A	G	0.0149105	0.0442293	0.0611747	0.4899999	0.317734	0.0435443	2.95E-13	0.139202	0.193477	0.4718473	0.5731887
ENSG00000008513	ST3GAL1	8	134525637	A	C	0.198807	-0.0104969	0.0184096	0.4400003	-0.292417	0.0100696	2.08E-185	0.035897	0.0629688	0.5686259	0.3558977
ENSG00000008516	MMP25	16	3103704	T	C	0.309145	0.0214028	0.0149906	0.2	0.405043	0.00901507	0	0.0528409	0.0370286	0.1535719	0.4259283
ENSG00000008517	IL32	16	3123603	A	T	0.354871	0.00579286	0.0148648	0.6200004	-0.471484	0.00999121	0	-0.0122864	0.0315288	0.6967659	0.5516692
ENSG00000008710	PKD1	16	2162305	C	T	0.182903	0.0025361	0.0184536	0.9400001	-0.136142	0.0146201	1.25E-20	-0.0186283	0.135561	0.8907018	0.9545156
ENSG00000008735	MAPK8IP2	22	51045761	C	T	0.459245	0.0246739	0.0142823	0.0519996	-0.0656916	0.00946647	3.94E-12	-0.375602	0.22405	0.09365662	0.6179634
ENSG00000008838	MED24	17	38196409	A	G	0.383698	-0.0067637	0.0145825	0.9	-0.15803	0.00812645	3.13E-84	0.0428001	0.0923029	0.642869	0.8200718
ENSG00000008853	RHOBTB2	8	22861321	A	G	0.0636183	-0.0109259	0.0292456	0.89	-0.200051	0.0160924	1.77E-35	0.0546156	0.146257	0.7088339	0.8272601
ENSG00000008869	HEATR5B	2	37253505	C	A	0.364811	0.0184402	0.0147312	0.1199999	0.172877	0.00903045	1.09E-81	0.106667	0.085394	0.2116245	0.8609506
ENSG00000008952	SEC62	3	169700292	C	A	0.203777	0.0349061	0.0180905	0.05499966	-0.151678	0.0115907	3.95E-39	-0.230133	0.120559	0.05627657	0.4059508
ENSG00000008988	RPS20	8	56983461	G	A	0.00994036	0.0361977	0.0583374	0.4600002	-0.291502	0.0391341	9.42E-14	-0.124177	0.20082	0.5363466	0.6228126
ENSG00000009335	UBE3C	7	156996836	C	A	0.227634	0.00451095	0.0167358	0.6300007	-0.360841	0.00928818	0	-0.0125012	0.0463811	0.78752	0.02949649
ENSG00000009413	REV3L	6	111712576	A	C	0.0347913	-0.0120477	0.0427992	0.91	-0.189182	0.0212674	5.82E-19	0.063683	0.226346	0.7784399	0.3896329
ENSG00000009724	MASP2	1	11096935	G	A	0.17992	0.0194925	0.0184187	0.4199997	0.100572	0.0112547	4.04E-19	0.193816	0.184419	0.2932793	0.9681927
ENSG00000009780	FAM76A	1	28071061	C	A	0.0685885	0.00544779	0.0260387	0.8499999	0.118768	0.0213736	2.75E-08	0.045869	0.219394	0.8343929	NA
ENSG00000009790	TRAF3IP3	1	209942522	G	A	0.465209	-0.0414609	0.0142828	0.005	-0.297404	0.00782968	0	0.139409	0.0481649	0.003798655	0.3417499
ENSG00000009830	POMT2	14	77764263	T	G	0.12326	0.0225009	0.0229776	0.3599996	0.293901	0.0138098	1.67E-100	0.0765595	0.0782642	0.3279662	0.08897035
ENSG00000009844	VTA1	6	142505226	A	T	0.0815109	0.0222738	0.0271295	0.4700002	0.420137	0.0167157	2.10E-139	0.0530156	0.0646075	0.4118855	0.5819557
ENSG00000009954	BAZ1B	7	72895668	T	C	0.468191	-0.00690643	0.0141982	0.4400003	-0.0885969	0.0118189	6.57E-14	0.0779534	0.160593	0.6273862	0.7929662
ENSG00000010030	ETV7	6	36339291	C	T	0.474155	0.0035989	0.0142663	0.9299999	-0.501349	0.00753428	0	-0.00717843	0.028456	0.800837	0.2833247
ENSG00000010072	SPRTN	1	231481809	C	T	0.349901	0.0267041	0.0146607	0.03400008	0.171436	0.00841001	2.28E-92	0.155767	0.0858576	0.06963974	0.4918664
ENSG00000010165	METTL13	1	171766975	A	G	0.244533	-0.010177	0.0159913	0.58	0.482777	0.00858931	0	-0.0210801	0.0331257	0.5245368	0.8846765
ENSG00000010219	DYRK4	12	4697347	G	A	0.431412	0.00648375	0.0143212	0.6800001	0.201558	0.00802581	3.52E-139	0.0321681	0.0710639	0.6507905	0.4732907
ENSG00000010256	UQCRC1	3	48642422	A	G	0.0427435	0.00389335	0.0366011	0.95	0.57278	0.0379878	2.26E-51	0.00679729	0.0639024	0.9152889	0.9106461
ENSG00000010270	STARD3NL	7	38244048	C	A	0.222664	-0.00455231	0.0164501	0.91	-0.214369	0.00929234	9.40E-118	0.0212359	0.0767429	0.7819993	0.3144689
ENSG00000010278	CD9	12	6328153	A	G	0.397614	0.00719006	0.0143673	0.5400003	0.424451	0.00803704	0	0.0169397	0.0338506	0.6167768	0.5227329
ENSG00000010292	NCAPD2	12	6621821	C	T	0.180915	-0.0250323	0.0181447	0.2300001	-0.546867	0.00973796	0	0.045774	0.0331893	0.1678402	0.7335516
ENSG00000010295	IFFO1	12	6656390	G	A	0.285288	-0.0187006	0.016003	0.3599996	-0.319744	0.00890288	1.80E-282	0.0584861	0.0500758	0.2428268	0.55127
ENSG00000010310	GIPR	19	46179242	A	C	0.233598	0.00885552	0.0169277	0.7099994	-0.0707909	0.00939221	4.80E-14	-0.125094	0.239698	0.6017523	0.7607789
ENSG00000010322	NISCH	3	52508110	G	A	0.427435	0.0301634	0.014619	0.09499921	-0.0773754	0.008036	6.05E-22	-0.389832	0.193225	0.0436434	0.4137167
ENSG00000010327	STAB1	3	52543932	A	G	0.0606362	0.0118287	0.0312055	0.58	-0.750308	0.0178534	0	-0.0157651	0.041592	0.7046561	0.5087897
ENSG00000010361	FUZ	19	50315379	T	C	0.0626243	0.00186518	0.031532	0.91	-0.250993	0.018184	2.44E-43	-0.00743119	0.12563	0.9528315	0.6244689
ENSG00000010379	SLC6A13	12	350914	T	C	0.00397614	-0.0764901	0.0880965	0.25	0.583423	0.0853581	8.20E-12	-0.131106	0.152213	0.3890553	0.2807158
ENSG00000010438	PRSS3	9	33774872	C	A	0.0198807	-0.126675	0.0514593	0.01199997	-0.413746	0.0606098	8.71E-12	0.306166	0.132214	0.02057518	0.5184596
ENSG00000010539	ZNF200	16	3279273	G	C	0.485089	-0.0192536	0.0142228	0.16	0.258088	0.00784115	1.36E-237	-0.0746008	0.0551548	0.1761929	0.3227607
ENSG00000010610	CD4	12	6912999	C	G	0.318091	-0.0225089	0.0152844	0.1299999	0.236626	0.00860315	1.55E-166	-0.0951242	0.0646855	0.1414088	0.4094116
ENSG00000010626	LRRC23	12	7003070	G	C	0.297217	-0.0193557	0.0158492	0.1900002	0.474841	0.00847503	0	-0.0407625	0.0333859	0.2221044	0.6759265
ENSG00000010704	HFE	6	26093040	G	A	0.369781	-0.0289832	0.0151067	0.01899984	-0.0488651	0.00830728	4.05E-09	0.593127	0.32518	0.06815267	0.08854015
ENSG00000010803	SCMH1	1	41600349	G	T	0.313121	-0.0123178	0.0150224	0.4700002	0.157578	0.00844966	1.29E-77	-0.0781697	0.0954254	0.412689	0.3445949
ENSG00000010810	FYN	6	112088095	C	T	0.526839	0.00234553	0.0142006	0.8700001	-0.096533	0.00795008	6.30E-34	-0.0242977	0.14712	0.8688213	0.8600251
ENSG00000010818	HIVEP2	6	143169471	T	G	0.33499	0.0153863	0.0150519	0.28	-0.07414	0.00829473	3.96E-19	-0.20753	0.204343	0.3098216	0.5586985
ENSG00000011007	ELOA	1	24079097	A	G	0.368787	-0.00333102	0.0144793	1	-0.458623	0.00775664	0	0.00726309	0.0315715	0.8180512	0.4838857

ENSG00000011009	LYPLA2	1	24119744	C	T	0.0904573	0.0425846	0.023883	0.06299992	-0.175161	0.0150301	2.19E-31	-0.243117	0.137935	0.077978	0.4027411
ENSG00000011021	CLCN6	1	11884704	A	C	0.0994036	-0.00590809	0.0229593	0.8800001	0.597607	0.0122197	0	-0.00988625	0.0384193	0.7969277	0.7654533
ENSG00000011105	TSPAN9	12	3291125	A	G	0.0675944	-0.0159213	0.0349786	0.6499995	-0.173088	0.0169771	2.08E-24	0.0919839	0.202287	0.6493109	0.9111847
ENSG00000011114	BTBD7	14	93751667	A	G	0.49006	-0.0111676	0.0141905	0.4500005	-0.180686	0.00788644	3.61E-116	0.0618066	0.078583	0.4315668	0.2114028
ENSG00000011132	APBA3	19	3756257	T	A	0.143141	0.0392572	0.0223481	0.08400014	0.226053	0.0133187	1.31E-64	0.173664	0.0993904	0.080587	0.1633002
ENSG00000011143	MKS1	17	56289884	C	T	0.326044	0.0136513	0.0152202	0.4600002	-0.0563914	0.00835057	1.45E-11	-0.242081	0.272273	0.3739429	0.2836146
ENSG00000011198	ABHD5	3	43753734	G	A	0.0477137	0.00673587	0.0304123	0.7600007	-0.370458	0.0177302	6.05E-97	-0.0181825	0.0820983	0.8247245	0.9314918
ENSG00000011260	UTP18	17	49356593	T	C	0.464215	0.0202064	0.0141804	0.1299999	0.179165	0.0117686	2.45E-52	0.112781	0.0794932	0.1559714	0.1195185
ENSG00000011275	RNF216	7	5740524	T	C	0.148111	0.00573273	0.0213014	0.89	0.208168	0.0115804	3.00E-72	0.0275389	0.102339	0.787857	0.2276457
ENSG00000011295	TTC19	17	15925511	A	G	0.470179	-0.00735302	0.0142331	0.6999999	-0.398418	0.00766019	0	0.0184555	0.0357258	0.6054434	0.2745588
ENSG00000011376	LARS2	3	45510455	G	A	0.332008	0.00548378	0.0154131	0.8700001	-0.310982	0.00846336	1.42E-295	-0.0176338	0.049565	0.7220126	0.8198758
ENSG00000011405	PIK3C2A	11	17164403	A	G	0.497018	-0.00556642	0.0141826	0.8	0.487577	0.0089052	0	-0.0114165	0.0290886	0.6947093	0.4552171
ENSG00000011422	PLAUR	19	44162473	G	A	0.149105	-0.0343661	0.0195864	0.04	0.136305	0.0118193	9.06E-31	-0.252127	0.145349	0.08280667	0.9078709
ENSG00000011454	RABGAP1	9	125785128	G	A	0.338966	-0.0183584	0.0152216	0.2700001	0.0871209	0.0102698	2.19E-17	-0.210723	0.176475	0.2324513	0.6776562
ENSG00000011478	QPCTL	19	46201494	A	G	0.285288	0.0124066	0.0151366	0.2	-0.100765	0.00872002	6.92E-31	-0.123124	0.150594	0.4135923	0.06756613
ENSG00000011485	PPP5C	19	46873244	A	C	0.302187	-0.0073024	0.0153833	0.59	-0.446251	0.0085142	0	0.0163639	0.0344737	0.6350171	0.7838884
ENSG00000011523	CEP68	2	65298819	C	T	0.38668	-0.0274401	0.0146624	0.04700023	-0.298558	0.00943638	1.07E-219	0.0919089	0.0491966	0.0617349	0.1780045
ENSG00000011590	ZBTB32	19	36201684	G	A	0.130219	-0.014234	0.0234536	0.3800004	0.257853	0.0120499	1.37E-101	-0.055202	0.0909938	0.5440789	0.2333184
ENSG00000011600	TYROBP	19	36397257	A	G	0.0337972	-0.00202967	0.0350831	0.9	-0.393665	0.0211561	2.79E-77	0.00515583	0.0891197	0.9538658	0.5123996
ENSG00000011638	LDAF1	16	21180817	A	G	0.177932	0.000895485	0.0201708	0.9599999	-0.139593	0.0110823	2.22E-36	-0.00641498	0.144498	0.9645897	0.9982923
ENSG00000012048	BRCA1	17	41259301	T	C	0.358847	-0.0233744	0.0150807	0.089	0.266028	0.0092019	8.91E-184	-0.0878645	0.0567698	0.1216869	0.3612936
ENSG00000012061	ERCC1	19	45946338	C	T	0.129225	0.00784897	0.0211073	0.6800001	-0.386927	0.0117674	4.09E-237	-0.0202854	0.0545547	0.7100145	0.9962278
ENSG00000012124	CD22	19	35824211	C	A	0.132207	-0.0189523	0.0208371	0.3700002	0.221104	0.0135495	7.33E-60	-0.0857167	0.0943875	0.3638062	0.2729023
ENSG00000012223	LTF	3	46501930	A	G	0.0238569	0.0419755	0.0603212	0.5400003	-0.344774	0.0487521	1.53E-12	-0.121748	0.175803	0.4886096	0.7892722
ENSG00000012232	EXTL3	8	28535551	A	G	0.16501	0.0115735	0.0193016	0.4199997	0.0619639	0.0108842	1.25E-08	0.186778	0.31322	0.5509648	0.7441735
ENSG00000012660	ELOVL5	6	53173071	T	C	0.136183	-0.00710094	0.0218387	0.7499995	0.226326	0.0122647	4.90E-76	-0.0313748	0.0965072	0.7451027	0.5168868
ENSG00000012779	ALOX5	10	45905611	C	T	0.395626	0.00990345	0.0146912	0.5500004	-0.215043	0.00801504	1.45E-158	-0.0460534	0.0683391	0.5003781	0.4627082
ENSG00000012822	CALCOCO1	12	54113216	G	A	0.125249	0.0336311	0.0216246	0.2099999	-0.157096	0.0117003	4.22E-41	-0.21408	0.138573	0.1223717	0.8170729
ENSG00000012963	UBR7	14	93684481	T	C	0.329026	-0.0131854	0.0150526	0.4700002	-0.364468	0.00831428	0	0.0361771	0.0413084	0.3811496	0.9057466
ENSG00000012983	MAP4K5	14	50956531	A	C	0.365805	-0.0133156	0.0149798	0.4199997	-0.0912864	0.00853304	1.04E-26	0.145866	0.164662	0.3756978	0.604314
ENSG00000013016	EHD3	2	31474596	G	A	0.300199	0.00860763	0.0158174	0.4500005	0.185661	0.00896047	2.29E-95	0.0463621	0.0852245	0.5864416	0.7280851
ENSG00000013288	MAN2B2	4	6600995	T	C	0.168986	0.0134283	0.0187771	0.5099998	-0.610244	0.0113977	0	-0.0220048	0.0307726	0.4745605	0.0838634
ENSG00000013306	SLC25A39	17	42399615	T	C	0.390656	-0.0271401	0.0146652	0.04700023	0.116312	0.0080916	7.49E-47	-0.233339	0.127126	0.06643212	0.08609677
ENSG00000013364	MVP	16	29845535	T	C	0.136183	0.0214488	0.0200362	0.2200002	0.295115	0.0125835	1.24E-121	0.0726794	0.0679635	0.2848945	0.5394977
ENSG00000013374	NUB1	7	151057160	A	G	0.404573	-0.0227074	0.014386	0.1499999	-0.168236	0.00803996	3.17E-97	0.134973	0.0857536	0.1154954	0.1333729
ENSG00000013375	PGM3	6	83887262	C	G	0.27833	-0.00554288	0.0167113	0.89	-0.163439	0.00953241	6.78E-66	0.0339141	0.102267	0.7401743	0.1161582
ENSG00000013392	RWDD2A	6	83904740	G	A	0.281312	0.000788207	0.0165859	0.81	-0.225888	0.00927917	6.78E-131	-0.00348936	0.0734253	0.9620967	0.4271019
ENSG00000013503	POLR3B	12	106827706	T	C	0.0228628	-0.0614875	0.050061	0.1499999	0.673835	0.0241856	7.93E-171	-0.09125	0.0743648	0.2198001	0.09315106
ENSG00000013523	ANGEL1	14	77273088	C	T	0.360835	0.0215984	0.0147576	0.1199999	-0.300071	0.00889615	2.07E-249	-0.0719777	0.0492267	0.143695	0.416023
ENSG00000013561	RNF14	5	141353874	T	C	0.445328	0.0504609	0.0142805	9.70E-05	0.464057	0.00778704	0	0.108739	0.0308272	0.00041973	0.2755085
ENSG00000013573	DDX11	12	31242252	A	G	0.431412	0.0181552	0.0142817	0.17	-0.891517	0.00680218	0	-0.0203644	0.0160203	0.2036712	0.9445248
ENSG00000013583	HEBP1	12	13140502	C	T	0.493042	-0.00963468	0.0142561	0.5700002	-0.360035	0.00841283	0	0.0267604	0.0396014	0.499203	0.4300954
ENSG00000013725	CD6	11	60763482	T	C	0.409543	0.0228219	0.0145117	0.1100001	-0.247298	0.00817089	3.24E-201	-0.0922851	0.0587602	0.1162904	0.7625979
ENSG00000013810	TACC3	4	1735062	G	A	0.441352	-0.0105068	0.0142091	0.56	-0.628911	0.0107151	0	0.0167063	0.022595	0.4596745	0.3180344
ENSG00000014123	UFL1	6	96986311	A	G	0.336978	-0.00172076	0.0154365	0.6800001	0.181265	0.00944544	4.42E-82	-0.00949304	0.0851611	0.9112424	0.7507306
ENSG00000014138	POLA2	11	65051146	G	C	0.139165	0.0132028	0.01978	0.2700001	-0.414363	0.0119926	1.34E-261	-0.0318629	0.0477448	0.5045436	0.2681497
ENSG00000014164	ZC3H3	8	144571724	A	G	0.497018	0.0125999	0.0142306	0.33	0.369607	0.00888349	0	0.03409	0.0385107	0.3760442	0.4546209
ENSG00000014216	CAPN1	11	64963757	C	T	0.305169	0.0114757	0.0158339	0.4799997	0.443557	0.00826634	0	0.025872	0.0357008	0.4686428	0.3454809
ENSG00000014257	ACP3	3	132061676	A	G	0.114314	0.026334	0.0207459	0.1100001	-0.648477	0.0112312	0	-0.040609	0.0319995	0.2044223	0.9456276
ENSG00000014641	MDH1	2	63825037	G	A	0.295229	0.00384746	0.0155926	0.64	0.175877	0.0131177	5.46E-41	0.0218759	0.0886714	0.8051347	0.1676353

ENSG00000014824	SLC30A9	4	42041020	G	A	0.0357853	0.0450987	0.0468941	0.25	-0.725503	0.0296484	3.07E-132	-0.062162	0.0646866	0.3365663	0.09338412
ENSG00000014914	MTMR11	1	149904667	C	T	0.0904573	-0.00204964	0.0262156	1	0.272432	0.0159388	1.69E-65	-0.00752349	0.096229	0.9376824	0.6197091
ENSG00000014919	COX15	10	101481729	A	T	0.408549	-0.0179956	0.0144032	0.32	-0.168908	0.00807587	3.89E-97	0.106541	0.0854243	0.2123266	0.80784
ENSG00000015133	CCDC88C	14	91810927	A	C	0.459245	0.0453269	0.0144896	0.001499996	-0.0968915	0.00881361	4.11E-28	-0.467811	0.155481	0.002622873	0.4811472
ENSG00000015153	YAF2	12	42591528	G	C	0.470179	-0.0352112	0.0141882	0.01899984	-0.101152	0.00796732	6.24E-37	0.348103	0.142922	0.01486601	0.6869325
ENSG00000015171	ZMYND11	10	240491	T	C	0.126243	-0.0146425	0.0202234	0.4100001	0.0842842	0.0118864	1.33E-12	-0.173728	0.241191	0.4713451	0.5185709
ENSG00000015475	BID	22	18237221	A	G	0.510934	0.00305001	0.0141776	0.91	0.115453	0.00794574	7.80E-48	0.0264177	0.122813	0.8296853	0.9178683
ENSG00000015532	XYLT2	17	48431976	T	A	0.223658	0.0117084	0.016987	0.5500004	-0.133173	0.00992795	5.01E-41	-0.0879184	0.127724	0.4912334	0.3229696
ENSG00000015676	NUDCD3	7	44476224	G	T	0.16501	0.0183308	0.0180813	0.2	0.417872	0.0105673	0	0.043867	0.0432841	0.3108377	0.5912594
ENSG00000016391	CHDH	3	53865402	T	C	0.413519	-0.0079642	0.0146276	0.4	-0.0488887	0.00884032	3.20E-08	0.162905	0.300648	0.5879259	0.985988
ENSG00000016864	GLT8D1	3	52734276	C	T	0.430417	-0.00356512	0.0144189	0.59	-0.118882	0.00873798	3.73E-42	0.0299887	0.121308	0.8047438	0.6169116
ENSG00000017260	ATP2C1	3	130652497	C	T	0.195825	-0.000962462	0.0176834	0.8600001	0.157973	0.0103075	5.13E-53	-0.00609258	0.11194	0.9565949	0.6140998
ENSG00000017797	RALBP1	18	9506560	T	C	0.197813	0.0183003	0.017308	0.1800002	0.642366	0.00940282	0	0.0284889	0.0269474	0.2904179	0.1576052
ENSG00000018189	RUFY3	4	71621476	C	T	0.0308151	-0.0248207	0.041882	0.35	0.351999	0.0223625	7.97E-56	-0.0705136	0.119068	0.553707	0.07947016
ENSG00000018280	SLC11A1	2	219254184	A	C	0.340954	0.000452876	0.0147134	0.7300002	0.442065	0.00792122	0	0.00102446	0.0332833	0.9754451	0.9340112
ENSG00000018510	AGPS	2	178332968	T	C	0.200795	-0.00552167	0.0178476	0.9299999	0.294934	0.0101059	3.07E-187	-0.0187217	0.0605172	0.7570466	0.557469
ENSG00000018699	TTC27	2	32949608	A	G	0.120278	-0.0338756	0.0221775	0.1199999	-0.495562	0.0128586	0	0.0683579	0.0447873	0.1269407	0.5020474
ENSG00000019102	VSIG2	11	124619751	C	A	0.276342	0.00409392	0.0163828	0.6899999	0.101845	0.00906111	2.60E-29	0.0401975	0.1609	0.8027191	0.05755043
ENSG00000019169	MARCO	2	119725989	G	A	0.238569	0.00170146	0.0169135	0.9299999	-0.855237	0.00769829	0	-0.00198946	0.0197764	0.9198698	0.4130595
ENSG00000019186	CYP24A1	20	52780250	G	A	0.206759	0.0264303	0.0175176	0.06900014	-0.152048	0.00977384	1.44E-54	-0.173829	0.115752	0.1331645	0.6732643
ENSG00000019485	PRDM11	11	45181649	A	T	0.417495	0.0235487	0.0144315	0.05	0.0905811	0.00815385	1.13E-28	0.259974	0.161031	0.1064334	0.2109608
ENSG00000019991	HGF	7	81364038	C	T	0.235586	-0.0248244	0.0165942	0.1299999	-0.068431	0.00945885	4.67E-13	0.362766	0.247625	0.1429273	0.2414697
ENSG00000019995	ZRANB1	10	126653725	T	C	0.0964215	0.0300311	0.0238009	0.2	0.691039	0.0136616	0	0.0434579	0.0344529	0.2071753	0.4339748
ENSG00000020181	ADGRA2	8	37672061	C	T	0.45825	-0.0262055	0.0144983	0.06299992	0.265016	0.00866604	2.19E-205	-0.0988826	0.0548027	0.0711782	0.2214971
ENSG00000020256	ZFP64	20	50744524	A	G	0.209742	-0.0198037	0.0172606	0.29	-0.141529	0.00979657	2.63E-47	0.139927	0.122342	0.2527338	0.06738649
ENSG00000020426	MNAT1	14	61319065	T	C	0.055666	-0.00638847	0.0302569	0.8600001	0.203244	0.0203888	2.10E-23	-0.0314326	0.148904	0.8328142	0.1119984
ENSG00000020577	SAMD4A	14	55146924	A	G	0.0447316	-0.0267068	0.0353013	0.32	-0.395251	0.0353794	5.60E-29	0.0675692	0.0895182	0.450363	0.7508017
ENSG00000020633	RUNX3	1	25258807	T	C	0.372763	0.0079105	0.0147413	0.6200004	-0.11168	0.0081889	2.38E-42	-0.0708317	0.132098	0.5918157	0.7676004
ENSG00000020922	MRE11	11	94189984	T	C	0.33499	0.00886563	0.0155087	0.56	0.670596	0.0083596	0	0.0132205	0.0231273	0.5675654	0.001059914
ENSG00000021300	PLEKHB1	11	73365543	G	T	0.145129	-0.00987763	0.0211031	0.5300002	0.32474	0.0114495	5.84E-177	-0.0304171	0.0649935	0.6397834	0.86568
ENSG00000021355	SERPINB1	6	2837403	G	A	0.28827	0.0169669	0.0154779	0.2099999	0.418556	0.00896898	0	0.0405368	0.0369895	0.2731228	0.6189929
ENSG00000021488	SLC7A9	19	33341043	G	A	0.184891	0.0137124	0.0169857	0.28	-0.299582	0.00975933	6.24E-207	-0.0457718	0.0567176	0.4196597	0.7094907
ENSG00000021574	SPAST	2	32335693	G	C	0.17495	0.0251403	0.0195543	0.2300001	-0.0851451	0.0115536	1.71E-13	-0.295264	0.233127	0.2053208	0.3061082
ENSG00000021762	OSBPL5	11	3148157	C	T	0.418489	-0.0295708	0.0144824	0.07100027	-0.426986	0.00891829	0	0.0692547	0.0339486	0.04135136	0.1130548
ENSG00000021776	AQR	15	35204886	A	G	0.142147	0.0395023	0.0190676	0.01499996	-0.127675	0.0109936	3.51E-31	-0.309398	0.151703	0.04139997	0.6902365
ENSG00000022277	RTF2	20	55068795	G	A	0.449304	0.0172125	0.0142798	0.16	0.207434	0.00975838	2.84E-100	0.0829782	0.0689508	0.2288058	0.7359203
ENSG00000022556	NLRP2	19	55488504	C	T	0.483101	-0.0170398	0.0141809	0.3800004	0.552371	0.00738437	0	-0.0308485	0.0256761	0.2295778	0.4168036
ENSG00000022567	SLC45A4	8	142267834	A	G	0.350895	-0.00542756	0.0148322	0.8800001	-0.584494	0.00870123	0	0.00928591	0.0253765	0.7144207	0.4047734
ENSG00000022840	RNF10	12	120993340	G	A	0.411531	0.00514374	0.0145511	0.7700005	-0.280547	0.00797164	2.55E-271	-0.0183347	0.0518696	0.7237308	0.3226162
ENSG00000022976	ZNF839	14	102796379	G	A	0.2833	0.00812282	0.015999	0.59	0.0990469	0.00985698	9.34E-24	0.0820099	0.161736	0.6121119	0.5618932
ENSG00000023041	ZDHHC6	10	114198365	G	T	0.328032	0.0401152	0.0147279	0.00329997	-0.161755	0.00845948	1.68E-81	-0.247999	0.0919696	0.007006501	0.01414128
ENSG00000023171	GRAMD1B	11	123447413	T	A	0.197813	-0.0208988	0.0170617	0.2200002	0.232414	0.0110686	6.90E-98	-0.0899204	0.0735355	0.2213989	0.2454376
ENSG00000023191	RNH1	11	500906	T	C	0.0974155	-0.0184065	0.0208188	0.3800004	-0.328975	0.0130645	6.50E-140	0.0559511	0.0633228	0.3769207	0.6890969
ENSG00000023228	NDUFS1	2	207005238	G	A	0.475149	0.0180416	0.0144017	0.2300001	-0.0703235	0.00973094	4.95E-13	-0.256551	0.207846	0.2170788	0.6436498
ENSG00000023318	ERP44	9	102801391	G	A	0.446322	0.00796303	0.0142828	0.4500005	-0.0976487	0.00847042	9.51E-31	-0.0815477	0.146438	0.5776128	0.1541981
ENSG00000023330	ALAS1	3	52240222	A	T	0.473161	3.67E-05	0.0142251	0.8600001	-0.0965741	0.00921808	1.11E-25	-0.000380417	0.147297	0.9979393	0.2667886
ENSG00000023445	BIRC3	11	102199174	G	A	0.0646123	-0.00939925	0.0264285	0.8	-0.408723	0.0169462	1.59E-128	0.0229966	0.0646682	0.722133	0.6880301
ENSG00000023516	AKAP11	13	42871842	T	C	0.358847	-0.000851483	0.0148945	0.8200001	0.154736	0.00831617	2.84E-77	-0.00550282	0.0962582	0.9544119	0.9974706
ENSG00000023572	GLRX2	1	193070421	G	T	0.0626243	-0.0252432	0.0306125	0.3700002	-0.188529	0.0185272	2.54E-24	0.133896	0.162908	0.4111281	0.1856104
ENSG00000023608	SNAPC1	14	62246110	G	A	0.284294	-0.00456289	0.015758	0.8	0.263278	0.00856839	2.50E-207	-0.017331	0.0598556	0.7721625	0.5277919

ENSG00000023697	DERA	12	16127163	T	C	0.0208748	-0.0255177	0.0411599	0.5400003	-0.285111	0.0239855	1.39E-32	0.089501	0.144561	0.535835	0.5575263
ENSG00000023839	ABCC2	10	101577219	G	T	0.429423	0.0212944	0.0143798	0.1100001	0.144948	0.00799732	2.04E-73	0.14691	0.0995371	0.1399608	0.3343162
ENSG00000023892	DEF6	6	35277571	C	T	0.186879	0.00883603	0.0181254	0.64	-0.660831	0.00985682	0	-0.0133711	0.0274289	0.6259172	0.5142547
ENSG00000023902	PLEKHO1	1	150126816	C	A	0.0874751	-0.0927008	0.0273625	0.001099993	0.330627	0.017827	8.72E-77	-0.280379	0.0841289	0.000859984	0.9644392
ENSG00000023909	GCLM	1	94362863	T	C	0.164016	-0.0008227	0.0191252	0.8600001	0.154856	0.0106974	1.72E-47	-0.00531269	0.123504	0.9656885	0.5959053
ENSG00000024048	UBR2	6	42596521	G	A	0.0745527	0.00521259	0.0328264	0.8800001	-0.446253	0.0273075	4.98E-60	-0.0116808	0.0735635	0.8738382	0.1611414
ENSG00000024862	CCDC28A	6	139104556	C	T	0.363817	0.0190395	0.0154614	0.1900002	0.224418	0.00840994	7.05E-157	0.0848393	0.0689687	0.218655	0.459701
ENSG00000025039	RRAGD	6	90098172	C	T	0.23161	-0.0244677	0.0167098	0.17	0.537486	0.00906537	0	-0.0455225	0.0310983	0.1432415	0.7605488
ENSG00000025156	HSF2	6	122737477	G	A	0.441352	0.024352	0.0142643	0.1100001	-0.385842	0.00773731	0	-0.063114	0.036991	0.08797093	0.7556064
ENSG00000025293	PHF20	20	34449099	T	C	0.101392	0.037852	0.0235699	0.1199999	-0.107367	0.0127769	4.34E-17	-0.352549	0.2235	0.1147038	0.4396598
ENSG00000025434	NR1H3	11	47280123	T	C	0.297217	0.0122543	0.0154408	0.4199997	-0.370563	0.00828558	0	-0.0330694	0.0416751	0.4274829	0.5214906
ENSG00000025708	TYMP	22	50966333	T	C	0.32008	0.0144156	0.0151113	0.33	-0.641352	0.0102073	0	-0.0224769	0.0235643	0.3401587	0.3220203
ENSG00000025770	NCAPH2	22	50954273	T	C	0.15507	0.0364487	0.0189276	0.04200007	0.0863921	0.011972	5.35E-13	0.421898	0.226756	0.0628033	0.5065416
ENSG00000025772	TOMM34	20	43579949	A	G	0.213718	0.0412597	0.0167683	0.005600025	-0.787382	0.0087382	0	-0.0524011	0.0213042	0.01390685	0.5603746
ENSG00000025796	SEC63	6	108234176	C	T	0.15507	-0.00692023	0.0202705	0.6800001	0.191187	0.0113277	6.55E-64	-0.0361961	0.106046	0.7328594	0.652748
ENSG00000025800	KPNA6	1	32607904	C	T	0.05666	0.0112016	0.0290746	0.7499995	0.196993	0.0147472	1.06E-40	0.0568628	0.147653	0.7001553	0.7758452
ENSG00000026025	VIM	10	17274925	A	C	0.262425	0.00116737	0.0158704	0.9699999	-0.240431	0.0132789	2.85E-73	-0.00485532	0.0660087	0.9413638	0.4280653
ENSG00000026036	RTEL1-TNFRSF6B	20	62310345	G	A	0.400596	0.0148127	0.0142423	0.16	-0.0609233	0.0082655	1.70E-13	-0.243137	0.23609	0.3030814	0.08476683
ENSG00000026103	FAS	10	90762978	C	G	0.323062	-0.00111846	0.0148836	0.83	-0.398263	0.00809591	0	0.00280834	0.0373713	0.9400977	0.00177398
ENSG00000026297	RNASET2	6	167356835	T	A	0.494036	0.0190734	0.0142042	0.1299999	0.890936	0.00676029	0	0.0214083	0.0159438	0.1793593	0.5572243
ENSG00000026508	CD44	11	35207183	T	A	0.346918	0.0186468	0.0151211	0.25	-0.133577	0.00818085	6.24E-60	-0.139596	0.113524	0.2188236	0.06894936
ENSG00000026559	KCNG1	20	49629929	C	A	0.139165	0.0185463	0.0196883	0.2999998	-0.0838817	0.0106916	4.31E-15	-0.221101	0.236401	0.3496447	0.06972066
ENSG00000026652	AGPAT4	6	161623052	C	T	0.133201	0.0324727	0.0204015	0.0530005	0.173044	0.011564	1.26E-50	0.187656	0.118563	0.1134777	0.7570949
ENSG00000026751	SLAMF7	1	160716824	T	C	0.300199	-0.0115814	0.0151577	0.5	-0.183701	0.00862824	1.39E-100	0.063045	0.0825662	0.4451241	0.1522733
ENSG00000026950	BTN3A1	6	26408954	T	C	0.450298	-0.0229261	0.0143447	0.07199959	0.351049	0.00777621	0	-0.0653075	0.040888	0.1102151	0.849851
ENSG00000027001	MIPEP	13	24383943	C	T	0.253479	-0.00538976	0.016838	0.6300007	-0.130787	0.00920103	7.46E-46	0.0412102	0.128776	0.7489578	0.3941593
ENSG00000027075	PRKCH	14	61835985	G	A	0.280318	-0.0039995	0.0156195	0.7300002	0.0799524	0.0087132	4.47E-20	-0.0500235	0.195436	0.7979829	0.888635
ENSG00000027697	IFNGR1	6	137529603	T	C	0.230616	-0.00331764	0.01736	0.9	-0.170565	0.00979547	6.62E-68	0.0194509	0.101786	0.8484498	0.1823498
ENSG00000027847	B4GALT7	5	177032224	C	G	0.408549	-0.00877571	0.01435	0.83	-0.330607	0.00782016	0	0.0265443	0.0434096	0.5408794	0.7775115
ENSG00000027869	SH2D2A	1	156781344	G	A	0.055666	0.00201584	0.0333742	0.9199999	-0.13058	0.0181286	5.89E-13	-0.0154376	0.255593	0.9518378	0.9337277
ENSG00000028116	VRK2	2	58260920	T	C	0.264414	-0.0233834	0.0160393	0.2	-0.294136	0.00953103	4.00E-209	0.0794985	0.054591	0.1453214	0.016724
ENSG00000028137	TNFRSF1B	1	12248172	C	T	0.224652	-0.0106883	0.0164286	0.5099998	-0.17622	0.00953407	2.82E-76	0.0606531	0.0932854	0.5155705	0.8504654
ENSG00000028203	VEZT	12	95654044	G	A	0.054672	0.0344892	0.0312974	0.28	0.366956	0.0190415	9.34E-83	0.0939872	0.0854285	0.2712512	0.5717375
ENSG00000028310	BRD9	5	871672	A	G	0.0387674	0.0750334	0.0327533	0.01199997	0.335056	0.0194253	1.15E-66	0.223943	0.0986132	0.02315163	0.3735547
ENSG00000028528	SNX1	15	64412305	A	G	0.255467	0.00215531	0.0159878	0.9199999	0.265139	0.00890423	7.83E-195	0.00812899	0.0603004	0.8927635	0.8605626
ENSG00000028839	TBPL1	6	134290972	G	A	0.397614	0.00576942	0.0150025	0.83	0.222652	0.0121187	2.18E-75	0.0259123	0.0673958	0.7006229	0.8671629
ENSG00000029364	SLC39A9	14	69896918	G	T	0.0725646	0.0434218	0.0287895	0.09599973	0.564117	0.0155456	2.52E-288	0.0769731	0.0510787	0.1318234	0.9249941
ENSG00000029534	ANK1	8	41632509	C	T	0.462227	-0.00189467	0.0142603	0.95	-0.168	0.00793397	1.63E-99	0.0112778	0.0848843	0.8943036	0.7118431
ENSG00000029639	TFB1M	6	155607135	A	G	0.426441	-0.0035989	0.0144571	0.59	0.218996	0.00799102	2.37E-165	-0.0164336	0.066018	0.8034178	0.05223774
ENSG00000029725	RABEP1	17	5237343	C	T	0.457256	0.0122518	0.014429	0.33	0.614076	0.0072277	0	0.0199516	0.0234983	0.395844	0.3523479
ENSG00000030066	NUP160	11	47834873	T	C	0.213718	0.00689547	0.0177181	0.6999999	-0.154333	0.00974457	1.71E-56	-0.0446793	0.114839	0.6972324	0.9764416
ENSG00000030110	BAK1	6	33544174	T	C	0.27336	0.0192053	0.0155294	0.1499999	0.668346	0.0121344	0	0.0287355	0.0232414	0.2163121	0.7418232
ENSG00000030419	IKZF2	2	213940790	G	C	0.0805169	-0.00796098	0.0269333	0.5700002	-0.130492	0.0137482	2.28E-21	0.0610073	0.206498	0.7676592	0.6787206
ENSG00000030582	GRN	17	42426542	A	G	0.0854871	-0.00442005	0.0245533	0.8800001	0.343539	0.0150199	8.79E-116	-0.0128662	0.0714739	0.8571424	0.3451581
ENSG00000031081	ARHGAP31	3	119076390	G	A	0.210736	0.0371391	0.0169927	0.01899984	-0.200667	0.0101723	1.27E-86	-0.185078	0.0851991	0.02983308	0.3103145
ENSG00000031691	CENPQ	6	49445958	G	A	0.4334	0.00357922	0.0144765	0.89	0.397768	0.00778976	0	0.00899826	0.0363948	0.8047221	0.2157439
ENSG00000031698	SARS1	1	109768665	A	G	0.0775348	-0.0204007	0.0275749	0.4700002	0.203471	0.0147671	3.43E-43	-0.100264	0.135718	0.4600501	0.2131859
ENSG00000031823	RANBP3	19	5947151	T	C	0.303181	-0.0190369	0.0161965	0.1900002	0.0852727	0.00888146	7.90E-22	-0.223247	0.191356	0.243347	0.2105709
ENSG00000032219	ARID4A	14	58802854	G	A	0.0606362	0.0346613	0.0306239	0.32	0.254729	0.0174728	3.84E-48	0.136071	0.120583	0.2591331	0.3371876
ENSG00000032389	EIPR1	2	3287174	G	A	0.0924453	-0.0104231	0.024615	0.5700002	0.284711	0.0127104	3.95E-111	-0.0366094	0.0864716	0.6720258	0.214807

ENSG00000032444	PNPLA6	19	7612770	A	G	0.468191	0.00473202	0.0142811	0.56	0.137618	0.00795717	5.15E-67	0.0343853	0.103793	0.7404272	0.1859942
ENSG00000032742	IFT88	13	21203044	C	T	0.469185	0.00945406	0.0142106	0.4700002	0.337987	0.00771714	0	0.0279717	0.0420497	0.5059191	0.5704252
ENSG00000033030	ZCCHC8	12	122971467	T	C	0.252485	0.0288167	0.0162382	0.0530005	-0.0877633	0.00927613	3.04E-21	-0.328346	0.188249	0.08112301	0.4735636
ENSG00000033050	ABCF2	7	150914619	C	T	0.316103	-0.017532	0.015129	0.17	0.270205	0.00833607	1.74E-230	-0.064884	0.0560265	0.2468256	0.1501068
ENSG00000033100	CHPF2	7	150932741	A	G	0.449304	0.0287708	0.0144431	0.02399993	-0.184385	0.00800427	2.04E-117	-0.156037	0.0786237	0.0471886	0.7322743
ENSG00000033122	LRRC7	1	70325854	G	T	0.176938	-0.0144721	0.0189089	0.4799997	0.583462	0.013039	0	-0.0248038	0.0324128	0.4441243	0.7448755
ENSG00000033170	FUT8	14	66044074	G	A	0.375746	-0.00107329	0.0151193	0.64	-0.321031	0.00817704	0	0.00334326	0.0470962	0.9434073	0.9939531
ENSG00000033178	UBA6	4	68524188	C	T	0.421471	0.0111949	0.0145343	0.5999997	0.102095	0.00869445	7.71E-32	0.109651	0.142666	0.4421377	0.3006931
ENSG00000033327	GAB2	11	78027868	T	C	0.175944	-0.00418512	0.0195918	0.99	0.181692	0.0110719	1.62E-60	-0.0230342	0.107839	0.8308607	0.575504
ENSG00000033627	ATP6V0A1	17	40642745	T	G	0.0765408	-0.00538701	0.0275504	0.9699999	0.208998	0.0154584	1.19E-41	-0.0257754	0.131835	0.8449918	0.2204283
ENSG00000033800	PIAS1	15	68414806	T	G	0.4334	-0.0116672	0.0142965	0.4799997	-0.251744	0.00786816	1.27E-224	0.0463455	0.0568083	0.4146017	0.6918926
ENSG00000033867	SLC4A7	3	27470062	C	T	0.254473	-0.00940619	0.0160473	0.8	0.193766	0.0090376	5.66E-102	-0.0485441	0.0828489	0.5579191	0.4254193
ENSG00000034053	APBA2	15	29270073	C	T	0.109344	0.0339794	0.0222554	0.17	0.276929	0.011854	1.05E-120	0.122701	0.0805365	0.1276229	0.7456379
ENSG00000034063	NA	19	4932628	C	G	0.432406	0.00408889	0.0142887	0.7199992	0.0842893	0.0133129	2.43E-10	0.0485102	0.169693	0.7749772	0.6096569
ENSG00000034152	MAP2K3	17	21203268	T	C	0.336978	0.000531467	0.0150311	1	-0.0647512	0.00851609	2.88E-14	-0.00820783	0.232139	0.9717947	0.9744768
ENSG00000034239	CLXN	8	49635609	C	A	0.394632	0.0124362	0.0144827	0.2999998	0.101644	0.00813076	7.35E-36	0.12235	0.14282	0.3916246	0.3579656
ENSG00000034510	TMSB10	2	85133272	T	C	0.12326	-0.0097397	0.0207448	0.6899999	-0.195053	0.0171027	3.96E-30	0.0499336	0.106445	0.6389957	0.7921078
ENSG00000034533	ASTE1	3	130739606	G	A	0.217694	0.00283255	0.0171082	0.95	-0.14668	0.00985208	3.93E-50	-0.0193111	0.116644	0.8685059	0.7408684
ENSG00000034677	RNF19A	8	101308867	G	C	0.190855	0.0143968	0.0188809	0.4	0.106992	0.0107195	1.85E-23	0.13456	0.176985	0.4470809	0.1863129
ENSG00000034713	GABARAPL2	16	75606014	G	A	0.0407555	0.0340587	0.0327001	0.2999998	-0.210836	0.0161895	9.05E-39	-0.161541	0.155593	0.299162	0.7157633
ENSG00000035115	SH3YL1	2	242064	A	G	0.333996	-0.00778427	0.0148853	0.5999997	-0.813834	0.00698941	0	0.00956494	0.0182905	0.6010126	0.9505438
ENSG00000035141	FAM136A	2	70526164	G	T	0.314115	0.0107468	0.0151529	0.3599996	-0.192897	0.00836194	9.62E-118	-0.0557127	0.0785916	0.4783933	0.7080333
ENSG00000035403	VCL	10	75818895	A	G	0.130219	-0.0176298	0.0210948	0.4100001	-0.483672	0.0115025	0	0.0364499	0.0436225	0.4033932	0.8033687
ENSG00000035499	DEPDC1B	5	59944378	T	C	0.450298	0.00152369	0.0143616	0.9	0.0448238	0.0079877	2.00E-08	0.0339929	0.320458	0.9155222	0.6198168
ENSG00000035664	DAPK2	15	64281733	A	G	0.151093	-0.0119094	0.0204602	0.4600002	-0.23169	0.0111693	1.41E-95	0.0514024	0.0883434	0.5606695	0.9523134
ENSG00000035681	NSMAF	8	59534233	A	G	0.363817	0.00975611	0.0152196	0.4299995	0.352141	0.00820324	0	0.0277051	0.043225	0.5215543	0.8242598
ENSG00000035687	ADSS2	1	244593616	C	T	0.157058	-0.00476749	0.0206237	0.7400005	-0.705009	0.0102986	0	0.00676231	0.0292533	0.8171872	0.8000681
ENSG00000035720	STAP1	4	68448750	T	C	0.0984095	-0.032398	0.0232501	0.07499978	0.190362	0.0149025	2.30E-37	-0.170191	0.122861	0.1659795	0.7138625
ENSG00000035862	TIMP2	17	76885264	T	C	0.0994036	0.0463232	0.0249714	0.05800027	-0.685872	0.0149519	0	-0.0675391	0.036438	0.06380569	0.1781158
ENSG00000035928	RFC1	4	39328535	G	A	0.435388	-0.00400375	0.0142954	0.5	0.167538	0.00794975	1.36E-98	-0.0238976	0.085334	0.7794408	0.1002071
ENSG00000036054	TBC1D23	3	100011969	A	T	0.440358	-0.00436372	0.0143937	0.8499999	0.0966784	0.0121675	1.93E-15	-0.0451365	0.148991	0.7619293	0.8414151
ENSG00000036257	CUL3	2	225392488	A	G	0.131213	0.000249545	0.0210631	0.9400001	0.165586	0.0121178	1.65E-42	0.00150705	0.127204	0.9905473	0.3604967
ENSG00000036448	MYOM2	8	2053315	G	T	0.375746	0.0109109	0.0149856	0.4500005	0.912031	0.00663057	0	0.0119633	0.0164312	0.4665633	0.5898774
ENSG00000036530	CYP46A1	14	100172139	T	A	0.374751	0.0126075	0.0148088	0.29	-0.11977	0.0123707	3.60E-22	-0.105264	0.12412	0.3963937	0.8571934
ENSG00000036549	ZZZ3	1	78088602	T	C	0.22167	0.0229865	0.01745	0.17	-0.165078	0.00994313	6.72E-62	-0.139246	0.10604	0.1891321	0.8460636
ENSG00000036565	SLC18A1	8	20021541	C	T	0.373757	-0.0060902	0.0146789	0.56	0.397845	0.00794536	0	-0.015308	0.0368972	0.6782293	0.8007344
ENSG00000037042	TUBG2	17	40815173	C	T	0.501988	0.0250728	0.014238	0.14	-0.120763	0.00867211	4.44E-44	-0.20762	0.11884	0.08062626	0.1107634
ENSG00000037280	FLT4	5	180052565	C	T	0.0934394	-0.00340485	0.0225428	0.9699999	0.316978	0.0142657	2.22E-109	-0.0107416	0.0711195	0.8799474	0.2483052
ENSG00000037474	NSUN2	5	6616378	T	C	0.324056	-0.0167679	0.0153791	0.2300001	0.689315	0.00745879	0	-0.0243255	0.0223122	0.2756125	0.1196731
ENSG00000037637	FBXO42	1	16626141	G	C	0.241551	0.0208683	0.0167051	0.28	-0.102044	0.00933904	8.60E-28	-0.204503	0.164771	0.2145569	0.8848668
ENSG00000037749	MFAP3	5	153509252	G	A	0.205765	0.0181659	0.0176227	0.2	-0.0924334	0.00990138	1.01E-20	-0.19653	0.191812	0.3055536	0.228847
ENSG00000037757	MRI1	19	13880221	G	A	0.26839	0.0139295	0.0165151	0.4100001	-0.403643	0.0100953	0	-0.0345094	0.0409242	0.3990872	0.1725256
ENSG00000037897	METTL1	12	58164415	G	A	0.319085	-0.0422178	0.0151109	0.009599973	0.117536	0.00975029	1.83E-33	-0.359191	0.131972	0.006494314	0.7926915
ENSG00000038002	AGA	4	178357790	C	T	0.341948	-0.015629	0.0147994	0.2399999	-0.801697	0.00708593	0	0.0194949	0.0184609	0.2909635	0.1833718
ENSG00000038210	PI4K2B	4	25221488	A	G	0.00497018	-0.178617	0.185102	0.32	-1.10656	0.0733084	1.76E-51	0.161416	0.167618	0.3355478	0.9147627
ENSG00000038219	BOD1L1	4	13599854	G	A	0.134195	-0.0115218	0.0199471	0.4400003	0.167052	0.0127749	4.48E-39	-0.0689714	0.119523	0.5639022	0.6822851
ENSG00000038274	MAT2B	5	162938231	C	T	0.307157	-0.00689002	0.0151092	0.91	0.136499	0.00858205	5.83E-57	-0.0504766	0.110736	0.648514	0.4533799
ENSG00000038358	EDC4	16	67912666	G	A	0.0347913	0.0150788	0.0352392	0.6800001	0.23312	0.0223672	1.96E-25	0.0646826	0.151291	0.6689873	0.7004494
ENSG00000038382	TRIO	5	14338023	T	G	0.0526839	-0.0364862	0.0325076	0.2099999	0.255853	0.0183771	4.63E-44	-0.142606	0.127468	0.2632425	0.2992255
ENSG00000038427	VCAN	5	82822703	G	C	0.524851	0.0124969	0.0142184	0.56	-0.0563314	0.00795326	1.41E-12	-0.221846	0.254342	0.3830805	0.7361787

ENSG00000038532	CLEC16A	16	11157195	T	C	0.135189	-0.00428468	0.0214238	0.7499995	0.183885	0.012829	1.35E-46	-0.0233008	0.116518	0.8414987	0.6248019
ENSG00000038945	MSR1	8	16195193	T	A	0.417495	-0.00475696	0.0144068	0.8700001	-0.379173	0.00778131	0	0.0125456	0.0379962	0.7412632	0.7314244
ENSG00000039068	CDH1	16	68820286	T	C	0.429423	-0.00106912	0.0143919	0.7700005	-0.229472	0.00794555	2.09E-183	0.00465904	0.0627176	0.9407828	0.5343056
ENSG00000039319	ZFYVE16	5	79739500	T	C	0.235586	0.0154451	0.016492	0.4799997	0.0511719	0.00934749	4.39E-08	0.301828	0.326968	0.3559497	0.9127965
ENSG00000039523	RIPOR1	16	67566506	T	C	0.0874751	-0.0105807	0.0256534	0.4799997	-0.339654	0.0140854	1.78E-128	0.0311514	0.075539	0.6800551	0.6332323
ENSG00000039560	RAI14	5	34744537	A	C	0.128231	-0.0375065	0.0220401	0.064	0.200673	0.0114976	3.24E-68	-0.186904	0.110352	0.09032089	0.1527687
ENSG00000039650	PNKP	19	50367813	T	C	0.406561	-0.0185934	0.0144857	0.2200002	0.462933	0.00879044	0	-0.0401644	0.0313004	0.1994261	0.4077552
ENSG00000040199	PHLPP2	16	71715171	G	A	0.16004	0.00478376	0.0189029	0.95	0.34105	0.0103307	5.22E-239	0.0140266	0.0554272	0.8002197	0.8974612
ENSG00000040275	SPDL1	5	169021210	A	T	0.0477137	-0.0732836	0.0301896	0.008099906	-0.641093	0.0179387	1.02E-279	0.11431	0.0471993	0.01544109	0.3827691
ENSG00000040341	STAU2	8	74496273	A	C	0.082505	0.0210115	0.025128	0.2700001	-0.116439	0.014606	1.56E-15	-0.180451	0.216988	0.4056254	0.08789894
ENSG00000040487	SLC66A1	1	19647307	G	A	0.305169	0.0308373	0.0155236	0.0259998	-0.146239	0.00871391	3.29E-63	-0.21087	0.106894	0.04852897	0.9727912
ENSG00000040531	CTNS	17	3552299	T	C	0.232604	-0.0122924	0.0172346	0.64	-0.363394	0.0105864	3.17E-258	0.0338267	0.047437	0.4757927	0.2626015
ENSG00000040608	RTN4R	22	20249853	A	G	0.166004	-0.000458859	0.019176	1	-0.0731917	0.0109033	1.91E-11	0.00626928	0.261999	0.9809095	0.3363748
ENSG00000040633	PHF23	17	7140694	G	A	0.137177	-0.0112238	0.0202755	0.3700002	-0.25174	0.0112831	2.88E-110	0.0445848	0.0805661	0.5799934	0.9506073
ENSG00000040933	INPP4A	2	99134406	T	C	0.0198807	0.0200539	0.0443044	0.7300002	-0.172979	0.0300373	8.47E-09	-0.115933	0.256916	0.6518113	0.9140012
ENSG00000041353	RAB27B	18	52473919	A	G	0.257455	0.0106855	0.0161899	0.59	0.140151	0.00898021	6.56E-55	0.076243	0.115621	0.5096256	0.4823001
ENSG00000041357	PSMA4	15	78837175	C	T	0.387674	0.0562334	0.0149735	0.00021	0.182602	0.00831792	8.14E-107	0.307957	0.0831922	0.000214117	0.6579103
ENSG00000041515	MYO16	13	109554427	C	T	0.249503	-0.00780968	0.0168876	0.32	0.498011	0.00964301	0	-0.0156818	0.0339115	0.6437715	0.4916787
ENSG00000041802	LSG1	3	194377361	T	C	0.164016	-0.0233095	0.0195111	0.2200002	0.847611	0.0105322	0	-0.0275002	0.0230215	0.2322639	0.9556027
ENSG00000041880	PARP3	3	51979622	G	T	0.166004	-0.0152413	0.0197656	0.33	0.0713196	0.0124672	1.06E-08	-0.213704	0.279648	0.4447534	0.8427508
ENSG00000041988	THAP3	1	6690286	G	A	0.332008	-0.0161507	0.0148856	0.2099999	-0.113372	0.015408	1.87E-13	0.142458	0.132719	0.2831	0.9424385
ENSG00000042088	TDP1	14	90466194	G	A	0.398608	-0.00558111	0.0144272	0.8	-0.128548	0.00835238	1.89E-53	0.0434165	0.112267	0.6989604	0.1437121
ENSG00000042286	AIFM2	10	71875334	T	C	0.406561	0.0258831	0.0147566	0.0530005	-0.212478	0.00813937	3.20E-150	-0.121815	0.0696065	0.0801087	0.0348977
ENSG00000042317	SPATA7	14	88893981	A	G	0.222664	-0.00852429	0.0172927	0.7800007	0.275182	0.0102105	5.58E-160	-0.030977	0.0628516	0.6221122	0.5812295
ENSG00000042429	MED17	11	93532627	T	C	0.251491	-0.024405	0.0161335	0.08500021	0.174229	0.00998905	3.96E-68	-0.140074	0.092947	0.1318019	0.5211928
ENSG00000042445	RETSAT	2	85575477	G	A	0.478131	-0.00521226	0.0141854	0.8	-0.60798	0.0107139	0	0.00857308	0.0233325	0.7132978	0.9968291
ENSG00000042493	CAPG	2	85633713	G	A	0.12326	0.00416086	0.019981	0.7099994	-0.769264	0.0167138	0	-0.00540888	0.0259744	0.8350426	0.9998379
ENSG00000042753	AP2S1	19	47347821	G	C	0.431412	0.0213385	0.0144372	0.08500021	-0.138621	0.0079953	2.44E-67	-0.153934	0.104526	0.1408372	0.2376746
ENSG00000042832	TG	8	134013175	T	C	0.360835	-0.031741	0.0148235	0.03400008	0.257952	0.00820079	3.64E-217	-0.12305	0.0575992	0.03265351	0.5352203
ENSG00000042980	ADAM28	8	24184042	A	C	0.385686	-0.0179925	0.0147245	0.28	-0.194626	0.00813153	1.33E-126	0.0924463	0.0757537	0.2223308	0.7322342
ENSG00000043093	DCUN1D1	3	182679801	A	G	0.237575	-0.0113813	0.017105	0.29	-0.11766	0.0136376	6.27E-18	0.0967305	0.145808	0.5070689	0.7759336
ENSG00000043143	JADE2	5	133889460	C	T	0.135189	-0.000810329	0.0204238	0.83	0.0885641	0.0130452	1.13E-11	-0.00914964	0.230614	0.9683522	0.6623867
ENSG00000043462	LCP2	5	169699236	C	G	0.16004	-0.0313526	0.0204343	0.1199999	0.220372	0.0110303	8.42E-89	-0.142271	0.0929993	0.1260643	0.5032191
ENSG00000043514	TRIT1	1	40327945	A	G	0.100398	0.0095017	0.023323	0.6300007	-0.592786	0.012592	0	-0.0160289	0.0393462	0.6837283	0.5342233
ENSG00000043591	ADRB1	10	115805236	C	T	0.241551	0.0177	0.0163447	0.2700001	0.249181	0.0100003	4.82E-137	0.0710327	0.0656556	0.2792976	0.6192449
ENSG00000044090	CUL7	6	43013519	C	T	0.0586481	0.00796802	0.0272993	0.5700002	-0.419556	0.0147846	3.79E-177	-0.0189916	0.0650706	0.770393	0.3500802
ENSG00000044115	CTNNA1	5	138108689	C	T	0.274354	0.00985684	0.0155448	0.5099998	-0.804681	0.00722673	0	-0.0122494	0.0193183	0.5260272	0.3510981
ENSG00000044459	CNTLN	9	17319450	T	G	0.340954	0.0150987	0.0156746	0.4899999	0.143999	0.00850625	2.77E-64	0.104853	0.109028	0.3361986	0.9801219
ENSG00000044574	HSPA5	9	128000370	G	A	0.0208748	-0.00359136	0.0494643	0.9	0.456062	0.0287707	1.37E-56	-0.00787471	0.108461	0.942121	0.09974294
ENSG00000046604	DSG2	18	29103488	T	C	0.410537	-0.0228945	0.0145179	0.07699987	-0.0530085	0.00914254	6.71E-09	0.431903	0.283829	0.1280836	0.103761
ENSG00000047056	WDR37	10	1136857	A	G	0.0208748	-0.00650197	0.0425344	0.7499995	0.436983	0.0248317	2.56E-69	-0.0148792	0.0973401	0.8785102	0.4825307
ENSG00000047188	YTHDC2	5	112890181	A	G	0.209742	-0.0328366	0.0176226	0.05999983	0.324491	0.00966762	5.50E-247	-0.101194	0.0543921	0.06282067	0.2660357
ENSG00000047315	POLR2B	4	57870611	G	A	0.500994	-0.0217435	0.0141929	0.2200002	-0.163525	0.0117673	6.64E-44	0.132967	0.0873193	0.1278155	0.04982149
ENSG00000047346	ATOSA	15	52937764	A	G	0.254473	0.0403138	0.0161574	0.01499996	0.127775	0.00997739	1.51E-37	0.315507	0.12883	0.01432457	0.8552527
ENSG00000047365	ARAP2	4	36156875	A	G	0.0457256	0.0185297	0.0354344	0.6600001	-0.595355	0.0223787	6.17E-156	-0.0311238	0.0595296	0.6010935	0.4529753
ENSG00000047410	TPR	1	186313889	C	G	0.151093	0.0150403	0.0188634	0.3599996	-0.265596	0.0106779	1.44E-136	-0.0566284	0.0710593	0.4254988	0.2115044
ENSG00000047578	KATNIP	16	27676572	T	C	0.15507	0.0138228	0.0198902	0.4100001	-0.140216	0.0127769	5.09E-28	-0.098582	0.142138	0.4879544	0.1070374
ENSG00000047579	DTNBP1	6	15593160	G	A	0.147117	0.00679624	0.0199149	0.7099994	0.541811	0.0114492	0	0.0125436	0.0367571	0.7329115	0.1147167
ENSG00000047617	ANO2	12	5848216	A	G	0.307157	-0.000389057	0.0157716	0.9199999	0.0786823	0.0104717	5.74E-14	-0.00494466	0.200448	0.9803197	0.7456814
ENSG00000047621	C12orf4	12	4622284	C	A	0.183897	-0.0190832	0.0180258	0.5300002	-0.200712	0.0109536	5.34E-75	0.0950775	0.089959	0.2905582	0.9668897

ENSG00000047662	FAM184B	4	17707032	A	G	0.37674	0.0204583	0.0144212	0.17	0.211985	0.0113298	4.07E-78	0.0965085	0.0682248	0.1571956	0.8920187
ENSG00000047849	MAP4	3	48011475	C	T	0.315109	-0.00127461	0.0152271	0.9199999	-0.185536	0.00862436	1.18E-102	0.00686988	0.0820715	0.9332901	0.3159052
ENSG00000048028	USP28	11	113707444	A	T	0.0994036	0.0196549	0.0212846	0.2	-0.122159	0.0134067	8.10E-20	-0.160896	0.17513	0.3582384	0.835553
ENSG00000048140	TSPAN17	5	176080223	C	A	0.406561	-0.00313472	0.0146123	0.6600001	0.185794	0.00805002	7.35E-118	-0.016872	0.0786511	0.830144	0.1262523
ENSG00000048162	NOP16	5	175813462	T	C	0.0636183	0.0228766	0.0289862	0.5300002	0.441875	0.0182426	1.30E-129	0.0517716	0.0656329	0.4302258	0.8405828
ENSG00000048342	CC2D2A	4	15537334	T	C	0.454274	-0.0104907	0.0143062	0.4700002	0.0998589	0.00877263	5.08E-30	-0.105055	0.143561	0.4643025	0.2369744
ENSG00000048392	RRM2B	8	103234038	T	C	0.148111	0.0170144	0.0198303	0.25	0.197155	0.0116349	2.09E-64	0.0862995	0.100711	0.3914988	0.3717141
ENSG00000048405	ZNF800	7	127029411	A	T	0.407555	-0.00773943	0.0145312	0.3599996	-0.160878	0.00816383	1.91E-86	0.0481075	0.0903574	0.5944397	0.1615392
ENSG00000048462	TNFRSF17	16	12060444	C	T	0.356859	0.0068641	0.0149763	0.6899999	-0.0788685	0.00827408	1.54E-21	-0.0870322	0.190109	0.6470948	0.7488959
ENSG00000048471	SNX29	16	12369370	C	G	0.21173	0.00269634	0.017717	0.7099994	0.751101	0.0105679	0	0.00358985	0.0235881	0.8790378	0.7496519
ENSG00000048544	MRPS10	6	42180071	G	C	0.362823	0.00526582	0.0146914	0.7600007	-0.173399	0.0122227	1.11E-45	-0.0303683	0.0847532	0.720108	0.3842142
ENSG00000048649	RSF1	11	77451552	A	T	0.0984095	-0.00964354	0.023116	0.5400003	-0.204521	0.0126915	2.01E-58	0.0471518	0.113063	0.6766487	0.6868894
ENSG00000048740	CELF2	10	11212962	C	G	0.417495	-0.0175093	0.014444	0.1900002	0.107129	0.0079905	5.50E-41	-0.163442	0.135379	0.2273187	0.4150821
ENSG00000048828	FAM120A	9	96271285	C	T	0.366799	0.0116785	0.0150079	0.5	-0.0892111	0.00850326	9.46E-26	-0.130909	0.168691	0.4377341	0.6938933
ENSG00000048991	R3HDM1	2	136385932	C	T	0.0745527	0.0117793	0.0344624	0.7899998	0.149637	0.0172036	3.38E-18	0.078719	0.230484	0.7326987	0.4494114
ENSG00000049089	COL9A2	1	40774823	T	C	0.0427435	0.0147163	0.0339656	0.81	-0.2667	0.0167372	3.65E-57	-0.0551792	0.127402	0.6649344	0.4137844
ENSG00000049167	ERCC8	5	60205279	G	A	0.429423	0.0111806	0.0145603	0.4	0.273279	0.00802618	4.29E-254	0.0409128	0.0532936	0.442673	0.590696
ENSG00000049192	ADAMTS6	5	64611155	G	A	0.399602	-0.0183855	0.0148304	0.1900002	0.261609	0.00929018	1.82E-174	-0.0702786	0.0567442	0.2155243	0.8931114
ENSG00000049239	H6PD	1	9313115	G	T	0.324056	-0.0106788	0.0150738	0.58	0.392922	0.00807308	0	-0.0271779	0.0383674	0.4787221	0.6121497
ENSG00000049245	VAMP3	1	7836410	A	T	0.293241	-0.00241978	0.0155791	0.81	0.271988	0.0086531	7.30E-217	-0.00889665	0.0572794	0.8765688	0.3095181
ENSG00000049246	PER3	1	7874808	C	T	0.0238569	-0.0174562	0.0531141	0.5999997	1.18578	0.0271132	0	-0.0147213	0.044794	0.7424237	0.5327386
ENSG00000049247	UTS2	1	7908357	G	A	0.026839	-0.0129942	0.0524508	0.6300007	1.34647	0.0263027	0	-0.0096506	0.0389549	0.8043373	0.2723041
ENSG00000049249	TNFRSF9	1	7990416	T	C	0.0238569	0.00188621	0.0545796	0.89	-0.410609	0.026934	1.78E-52	-0.00459368	0.132924	0.9724316	0.3582684
ENSG00000049449	RCN1	11	31980620	G	C	0.492048	0.00714145	0.0142154	0.5999997	0.155432	0.00919847	4.69E-64	0.0459458	0.0914977	0.6155604	0.4128723
ENSG00000049541	RFC2	7	73657301	G	A	0.0666004	0.0227879	0.0270573	0.4600002	-0.250227	0.0146323	1.46E-65	-0.0910691	0.108262	0.4002418	0.1416353
ENSG00000049656	CLPTM1L	5	1331536	G	A	0.215706	0.0100645	0.0169839	0.4899999	0.0776798	0.00994314	5.61E-15	0.129564	0.219268	0.554592	0.8645145
ENSG00000049759	NEDD4L	18	55890185	T	C	0.359841	0.012872	0.0146843	0.2599998	0.155407	0.00824999	3.74E-79	0.0828278	0.0945918	0.3812285	0.2902474
ENSG00000049860	HEXB	5	73977160	G	A	0.426441	-0.00828577	0.014351	0.5199996	-0.340752	0.0144935	3.18E-122	0.0243161	0.0421284	0.5638102	0.1877752
ENSG00000049883	PTCD2	5	71636123	G	A	0.259443	0.00559551	0.0161907	0.83	-0.0875509	0.00928666	4.20E-21	-0.0639115	0.185053	0.7298175	0.3373975
ENSG00000050130	JKAMP	14	59961644	C	T	0.0586481	-0.0164368	0.0321236	0.6100002	0.671952	0.016354	0	-0.0244613	0.0478101	0.6089072	0.5822453
ENSG00000050165	DKK3	11	12007984	A	C	0.256461	-0.014162	0.0167312	0.35	-0.450115	0.00945272	0	0.0314631	0.0371768	0.3973805	0.2111904
ENSG00000050327	ARHGEF5	7	144065053	A	G	0.423459	0.000169466	0.0145467	0.9	0.0829091	0.00814079	2.33E-24	0.002044	0.175454	0.990705	0.5294281
ENSG00000050344	NFE2L3	7	26209302	A	G	0.21173	-0.00473388	0.017505	0.81	-0.574902	0.0141579	0	0.00823425	0.0304494	0.786834	0.3062538
ENSG00000050405	LIMA1	12	50623450	C	G	0.338966	0.004428	0.0148436	0.4500005	0.353166	0.00832951	0	0.012538	0.0420311	0.7654719	0.8345046
ENSG00000050426	LETMD1	12	51447976	A	C	0.083499	0.0241142	0.0271907	0.4	-0.375162	0.0156653	9.56E-127	-0.0642767	0.0725268	0.3754843	0.808929
ENSG00000050438	SLC4A8	12	51844040	A	G	0.105368	-0.00697774	0.0218629	1	-0.238164	0.0126461	4.05E-79	0.029298	0.0918107	0.7496407	0.1252837
ENSG00000050555	LAMC3	9	133927164	G	A	0.497018	0.00448304	0.0142062	0.7099994	0.350686	0.00929011	0	0.0127836	0.0405112	0.7523378	0.3312078
ENSG00000050730	TNIP3	4	122100592	T	G	0.195825	0.0132028	0.0173884	0.5700002	0.125271	0.00986561	6.08E-37	0.105393	0.139054	0.4484905	0.43067
ENSG00000050748	MAPK9	5	179689621	T	C	0.410537	-0.0181445	0.0145691	0.1299999	0.266875	0.00803629	8.06E-242	-0.0679888	0.0546299	0.2133028	0.06523298
ENSG00000050767	COL23A1	5	177841087	A	G	0.322068	-0.00482049	0.0152035	0.7199992	0.235579	0.0124892	2.32E-79	-0.0204623	0.0645459	0.7512286	0.7098188
ENSG00000050820	BCAR1	16	75282439	T	C	0.104374	-0.0211579	0.0243919	0.29	0.266643	0.0127201	1.45E-97	-0.0793492	0.091556	0.386121	0.005558425
ENSG00000051009	FHIP1B	11	6244253	T	C	0.260437	0.0162674	0.0158265	0.3599996	0.473935	0.00847873	0	0.0343242	0.0333995	0.3040982	0.8643061
ENSG00000051108	HERPUD1	16	56971879	G	A	0.498012	-0.00926887	0.0142434	0.25	0.215958	0.00787433	1.36E-165	-0.0429197	0.065973	0.5153271	0.5101062
ENSG00000051128	HOMER3	19	19046040	C	T	0.348907	-0.00239703	0.0174495	0.7600007	-0.0567634	0.0104096	4.95E-08	0.0422284	0.307505	0.8907731	0.21952
ENSG00000051180	RAD51	15	41005663	A	G	0.028827	-0.0152421	0.0418884	0.6600001	0.181339	0.0265186	8.02E-12	-0.0840529	0.231321	0.716336	0.9785609
ENSG00000051382	PIK3CB	3	138463320	G	T	0.457256	0.00296951	0.0141867	0.6999999	-0.0469842	0.00800631	4.40E-09	-0.0632023	0.302138	0.834305	0.5018756
ENSG00000051523	CYBA	16	88713625	T	G	0.575547	-0.00590627	0.0146653	0.5	-0.361705	0.0141254	1.29E-144	0.016329	0.04055	0.6871778	0.04037055
ENSG00000051596	THOC3	5	175403279	A	G	0.251491	0.0159545	0.0179956	0.4700002	0.355716	0.0220727	1.98E-58	0.0448518	0.0506664	0.3760276	0.5916127
ENSG00000051620	HEBP2	6	138729489	T	G	0.0526839	-0.0361333	0.0308971	0.14	-0.922913	0.01485	0	0.0391514	0.0334837	0.2422968	0.6764723
ENSG00000051825	MPHOSPH9	12	123682714	A	G	0.2167	-0.00223807	0.0176389	0.8200001	0.29073	0.0160081	1.04E-73	-0.00769811	0.0606726	0.8990357	0.9726988

ENSG00000052126	PLEKHA5	12	19405991	T	C	0.182903	-0.00606671	0.0193202	0.5999997	-0.358992	0.0103345	2.17E-264	0.0168993	0.0538202	0.7535242	0.860415
ENSG00000052723	SIKE1	1	115317703	C	T	0.243539	-0.00966974	0.0168343	0.4899999	0.068002	0.00930463	2.70E-13	-0.142198	0.248319	0.5668877	0.9907998
ENSG00000052749	RRP12	10	99138621	A	G	0.33499	-0.0110183	0.0151581	0.58	0.845065	0.00703283	0	-0.0130384	0.0179375	0.4673003	0.9105968
ENSG00000052795	FNIP2	4	159759745	G	T	0.372763	0.0220578	0.0146116	0.16	-0.241778	0.00816206	7.74E-193	-0.0912315	0.0605123	0.1316435	0.6454748
ENSG00000052841	TTC17	11	43448482	T	C	0.0954274	0.00987418	0.0248738	0.6200004	-0.21689	0.0205404	4.61E-26	-0.0455261	0.114765	0.6915954	0.8790738
ENSG00000053108	FSTL4	5	132740201	G	A	0.342942	-0.0123833	0.0149071	0.4100001	0.0949506	0.00845433	2.87E-29	-0.130418	0.157427	0.4074241	0.3836667
ENSG00000053254	FOXN3	14	89838354	A	G	0.16501	-0.0209262	0.018222	0.17	-0.0857432	0.0105835	5.42E-16	0.244057	0.214643	0.255523	0.6703114
ENSG00000053371	AKR7A2	1	19634549	A	G	0.27833	0.0171306	0.016359	0.33	-0.248637	0.00896025	1.80E-169	-0.068898	0.0658415	0.2953663	0.942137
ENSG00000053372	MRTO4	1	19582327	A	G	0.225646	0.030808	0.0163242	0.0519996	-0.216275	0.00928731	5.98E-120	-0.142448	0.0757262	0.05995939	0.4173133
ENSG00000053501	USE1	19	17328396	T	A	0.178926	-0.0154765	0.018413	0.4	0.161092	0.0171517	5.88E-21	-0.0960724	0.114758	0.4024944	0.9362293
ENSG00000053524	MCF2L2	3	183021198	G	C	0.437376	-0.0022763	0.0142461	0.9299999	-0.125643	0.0088246	5.34E-46	0.0181172	0.113393	0.8730591	0.6023171
ENSG00000053770	AP5M1	14	57746212	T	C	0.0576541	-0.0240525	0.0302098	0.3700002	-0.143581	0.0199696	6.48E-13	0.167518	0.211688	0.4287427	0.8200505
ENSG00000053900	ANAPC4	4	25399477	T	C	0.449304	0.0328221	0.0142492	0.02800013	0.495164	0.0074607	0	0.0662854	0.0287941	0.02133243	0.1294572
ENSG00000053918	KCNQ1	11	2668126	C	T	0.459245	-0.00381988	0.01451	0.9	0.0772208	0.00807192	1.10E-21	-0.049467	0.187974	0.7924282	0.5772111
ENSG00000054116	TRAPPC3	1	36608635	C	T	0.0258449	0.0468853	0.0495664	0.4799997	-0.66915	0.037116	1.16E-72	-0.0700669	0.0741755	0.3448582	0.9404945
ENSG00000054118	THRAP3	1	36730487	T	C	0.0258449	-0.0628914	0.0475262	0.14	-0.390546	0.0312352	7.15E-36	0.161035	0.122371	0.1881908	0.2972172
ENSG00000054148	PHPT1	9	139744332	G	C	0.250497	-0.0259746	0.0161724	0.1199999	0.336621	0.00951958	6.82E-274	-0.0771628	0.0480929	0.1086143	0.6205656
ENSG00000054179	ENTPD2	9	139945523	C	G	0.364811	0.011483	0.0146666	0.4100001	-0.312851	0.0098548	3.59E-221	-0.0367043	0.0468947	0.4338048	0.03969721
ENSG00000054219	LY75	2	160694811	A	G	0.491054	0.00560609	0.0142187	0.7700005	-0.417491	0.00830906	0	-0.0134281	0.0340586	0.6933864	0.3978184
ENSG00000054267	ARID4B	1	235393241	T	C	0.115308	-0.00745501	0.0217021	0.6300007	0.122154	0.0127199	7.73E-22	-0.0610296	0.177775	0.7313758	0.8328681
ENSG00000054277	OPN3	1	241797041	T	C	0.338966	-0.015067	0.0150765	0.33	-0.400571	0.00947326	0	0.0376138	0.037648	0.3177507	0.1513639
ENSG00000054282	SDCCAG8	1	243541357	G	T	0.346918	0.000151671	0.0151737	0.6999999	0.547364	0.0115163	0	0.000277093	0.0277214	0.9920248	0.5614214
ENSG00000054356	PTPRN	2	220164357	C	T	0.173956	-0.01045	0.0184131	0.59	0.373853	0.0110261	5.42E-252	-0.0279522	0.0492592	0.5704076	0.08410512
ENSG00000054392	HHAT	1	210675617	T	G	0.146123	0.00568654	0.0197516	0.64	0.15053	0.0111449	1.43E-41	0.0377768	0.131243	0.7734712	0.1937312
ENSG00000054523	KIF1B	1	10356262	G	C	0.11332	0.0120685	0.0222173	0.6899999	0.955926	0.0106268	0	0.0126249	0.0232421	0.586997	0.9145188
ENSG00000054611	TBC1D22A	22	47364927	G	C	0.27833	0.00249989	0.0158688	0.59	0.173579	0.01012	6.07E-66	0.0144021	0.0914253	0.8748285	0.4729826
ENSG00000054654	SYNE2	14	64506424	A	G	0.405567	0.0185298	0.014299	0.1499999	0.206725	0.00802149	1.86E-146	0.089635	0.0692566	0.1955806	0.9896633
ENSG00000054690	PLEKHH1	14	68028173	T	C	0.429423	0.0161558	0.0142386	0.2300001	0.060047	0.00912569	4.70E-11	0.269052	0.240624	0.2635048	0.4457476
ENSG00000054793	ATP9A	20	50299113	T	C	0.454274	-0.00583938	0.0143678	0.6300007	-0.593881	0.00798569	0	0.00983258	0.0241934	0.6844375	0.002182632
ENSG00000054965	FAM168A	11	73210383	C	T	0.16998	0.010975	0.0188096	0.5500004	0.157166	0.0109829	1.90E-46	0.0698308	0.11978	0.5598972	0.9200933
ENSG00000054967	RELT	11	73097914	G	T	0.202783	-0.00422064	0.0173958	0.8700001	0.366882	0.00926906	0	-0.0115041	0.0474161	0.8083002	0.9906092
ENSG00000054983	GALC	14	88382086	T	C	0.254473	-0.00748619	0.0164802	0.7499995	-0.352513	0.00925421	0	0.0212366	0.0467539	0.6496696	0.617773
ENSG00000055044	NOP58	2	203149414	A	G	0.253479	0.0180712	0.0165062	0.09200046	0.274968	0.00908435	3.00E-201	0.0657211	0.0600688	0.2739123	0.8450037
ENSG00000055070	SZRD1	1	16701855	G	T	0.468191	0.00198645	0.0141852	0.98	0.107493	0.00806324	1.52E-40	0.0184799	0.131972	0.888637	0.5969917
ENSG00000055118	KCNH2	7	150658726	T	C	0.400596	-0.0200983	0.0148804	0.2	0.147718	0.00837872	1.44E-69	-0.136058	0.10103	0.1780738	0.977521
ENSG00000055130	CUL1	7	148446567	T	G	0.413519	0.00643294	0.0146879	0.5300002	-0.0929812	0.00822057	1.16E-29	-0.0691854	0.158085	0.6616418	0.759621
ENSG00000055147	FAM114A2	5	153394092	A	G	0.385686	-0.0147072	0.0145941	0.32	-0.107805	0.00820486	1.96E-39	0.136424	0.135772	0.3149938	0.03350619
ENSG00000055163	CYFIP2	5	156757847	C	G	0.375746	-0.0187411	0.0146493	0.1499999	0.12078	0.00819758	3.92E-49	-0.155167	0.121746	0.2024786	0.03251648
ENSG00000055208	TAB2	6	149636263	T	C	0.152087	-0.0390243	0.0191106	0.02999991	-0.164688	0.0104095	2.23E-56	0.236959	0.117004	0.04284435	0.007579644
ENSG00000055211	GINM1	6	149900157	T	G	0.357853	-0.0243398	0.0149185	0.08199927	-0.393619	0.00887498	0	0.061836	0.0379265	0.1030138	0.08994439
ENSG00000055332	EIF2AK2	2	37355280	A	G	0.210736	0.0262666	0.0169447	0.05800027	-0.208553	0.00951669	1.89E-106	-0.125947	0.0814521	0.1220387	0.4371208
ENSG00000055483	USP36	17	76810493	G	A	0.431412	0.0298982	0.0143726	0.04	-0.5972	0.00735037	0	-0.050064	0.0240745	0.03756769	0.08532888
ENSG00000055732	MCOLN3	1	85498973	A	G	0.274354	-0.0115517	0.0155862	0.3900004	-0.164894	0.00866525	9.74E-81	0.0700551	0.094594	0.4589434	0.940202
ENSG00000055813	CCDC85A	2	56512283	A	G	0.413519	0.0246875	0.0145012	0.09299937	-0.177122	0.00905604	3.50E-85	-0.139382	0.082181	0.08987977	0.1193411
ENSG00000055917	PUM2	2	20500223	T	C	0.0387674	0.00272914	0.0364231	0.9199999	-0.323156	0.0200471	1.85E-58	-0.00844528	0.112712	0.940272	0.249951
ENSG00000055950	MRPL43	10	102738243	C	G	0.183897	-0.0109562	0.0176103	0.5400003	-0.445281	0.00969298	0	0.0246051	0.0395523	0.5338825	0.5540852
ENSG00000055955	ITIH4	3	52856243	A	G	0.259443	0.00480385	0.0169667	0.9	0.619639	0.00941963	0	0.00775267	0.0273819	0.7770761	0.703603
ENSG00000056050	HPF1	4	170664860	T	C	0.215706	-0.0112419	0.0174759	0.5	-0.295574	0.0258156	2.37E-30	0.0380342	0.0592186	0.5206997	0.7847263
ENSG00000056097	ZFR	5	32399661	C	T	0.171968	-0.0140487	0.0194959	0.5099998	-0.0629863	0.0110884	1.34E-08	0.223044	0.312007	0.4746903	0.755623
ENSG00000056558	TRAF1	9	123678061	A	G	0.318091	0.00361248	0.0152608	0.9599999	-0.196991	0.00853408	6.89E-118	-0.0183383	0.0774738	0.8128866	0.437244

ENSG00000056586	RC3H2	9	125637198	G	T	0.136183	-0.0160691	0.021306	0.4700002	-0.17456	0.0120712	2.14E-47	0.0920551	0.122222	0.4513407	0.2033799
ENSG00000056661	NA	17	36898110	T	C	0.138171	-0.00435852	0.0202225	0.9299999	0.315666	0.0118296	7.14E-157	-0.0138074	0.064065	0.829361	0.9371577
ENSG00000056736	IL17RB	3	53890217	C	T	0.347913	0.00273036	0.0149267	0.6200004	-0.188718	0.00831644	5.35E-114	-0.0144679	0.0790977	0.8548671	0.3552807
ENSG00000056972	TRAF3IP2	6	111902569	G	T	0.0904573	-0.0260132	0.0228064	0.2	0.428201	0.0125218	2.74E-256	-0.06075	0.0532906	0.2542964	0.2974976
ENSG00000057019	DCBLD2	3	98567659	A	G	0.0626243	-0.0276185	0.0260275	0.3400001	0.559796	0.0168411	2.86E-242	-0.0493367	0.0465183	0.2888775	0.01371217
ENSG00000057252	SOAT1	1	179295370	A	G	0.340954	-0.00363921	0.0146483	0.7300002	-0.353684	0.00786547	0	0.0102894	0.0414169	0.8037976	0.3412932
ENSG00000057294	PKP2	12	32996726	T	C	0.337972	0.0091632	0.0154018	0.4799997	0.185031	0.0084213	5.36E-107	0.0495225	0.0832695	0.552027	0.4708165
ENSG00000057468	MSH4	1	76320745	A	C	0.00894632	-0.0683022	0.0672212	0.2700001	1.35577	0.0525074	5.21E-147	-0.0503788	0.0496199	0.309965	0.3084803
ENSG00000057593	F7	13	113767550	C	T	0.120278	-0.0331408	0.0223012	0.08600031	-0.0936977	0.0143598	6.80E-11	0.353699	0.244107	0.1473511	0.1121007
ENSG00000057608	GDI2	10	5845640	G	A	0.175944	0.0114391	0.0177063	0.4100001	-0.789463	0.013694	0	-0.0144897	0.0224297	0.5182753	0.4159245
ENSG00000057663	ATG5	6	106703008	C	T	0.0854871	0.00309817	0.0242151	0.83	-0.0821054	0.0137927	2.64E-09	-0.037734	0.294995	0.8982169	0.5756011
ENSG00000057704	TMCC3	12	95002619	G	A	0.276342	0.00457356	0.01575	1	-0.2944	0.00850011	7.57E-263	-0.0155352	0.0535006	0.7715297	0.5431478
ENSG00000057757	PITHD1	1	24109808	T	C	0.0904573	0.0267653	0.0243297	0.33	-0.103742	0.0171782	1.55E-09	-0.257998	0.23838	0.2791202	0.5175439
ENSG00000057935	MTA3	2	42852898	A	C	0.355865	0.0148669	0.0146548	0.2399999	-0.105418	0.00820823	9.42E-38	-0.141028	0.139449	0.3118619	0.2141841
ENSG00000058056	USP13	3	179438866	T	C	0.154076	-0.00509508	0.0198076	0.64	-0.325402	0.0139851	9.40E-120	0.0156578	0.0608749	0.7970145	0.01512097
ENSG00000058063	ATP11B	3	182575355	C	T	0.229622	-0.00859178	0.0173012	0.5700002	-0.240309	0.00946217	2.74E-142	0.0357531	0.0720095	0.6195379	0.7633887
ENSG00000058091	CDK14	7	90467821	G	T	0.235586	-0.00804115	0.0174398	0.6100002	0.0777101	0.00947015	2.29E-16	-0.103476	0.224775	0.645262	0.2523809
ENSG00000058272	PPP1R12A	12	80248291	A	G	0.136183	0.0234439	0.0227242	0.59	-0.100161	0.0124683	9.50E-16	-0.234063	0.228741	0.3061816	0.6049904
ENSG00000058335	RASGRF1	15	79317702	A	G	0.23161	0.00383329	0.016709	0.7800007	0.0606173	0.010345	4.64E-09	0.0632376	0.275859	0.8186831	0.9358005
ENSG00000058453	CROCC	1	17183121	T	C	0.310139	-0.0128637	0.0155617	0.2700001	0.291113	0.00893109	4.77E-233	-0.0441881	0.0534732	0.4085999	0.7318118
ENSG00000058600	POLR3E	16	22327577	G	A	0.287276	-0.00149205	0.0156764	0.9699999	0.368019	0.00945753	0	-0.00405427	0.0425968	0.9241736	0.784937
ENSG00000058668	ATP2B4	1	203654449	C	T	0.217694	-0.0132089	0.0179323	0.5	0.0863541	0.0103898	9.46E-17	-0.152962	0.208474	0.463118	0.4396554
ENSG00000058673	ZC3H11A	1	203794017	C	T	0.140159	-0.00868934	0.0200127	0.6999999	0.120637	0.0178832	1.52E-11	-0.0720291	0.166236	0.6648007	0.1773923
ENSG00000058729	RIOK2	5	96507767	T	C	0.483101	0.0302611	0.0142406	0.02800013	-0.177949	0.00789726	1.97E-112	-0.170055	0.0803814	0.03437876	0.6155456
ENSG00000058799	YIPF1	1	54336899	G	A	0.472167	-0.00443244	0.0142338	0.6600001	0.12638	0.00793234	3.79E-57	-0.0350724	0.112649	0.7555399	0.6752788
ENSG00000058804	NDC1	1	54267833	C	T	0.0387674	-0.0286901	0.0338252	0.5700002	-0.319468	0.0258194	3.65E-35	0.0898059	0.106128	0.3974405	0.8197311
ENSG00000058866	DGKG	3	185951741	G	T	0.398608	0.0236293	0.0143761	0.07499978	0.0530338	0.00825506	1.32E-10	0.445552	0.279805	0.1113033	0.08393224
ENSG00000059122	FLYWCH1	16	2981573	C	T	0.49503	0.0120871	0.0141797	0.32	0.521718	0.00743992	0	0.0231679	0.0271809	0.3940144	0.02684671
ENSG00000059377	TBXAS1	7	139598487	G	C	0.350895	-0.0284153	0.0149672	0.03099988	-0.284525	0.00813432	4.81E-268	0.0998694	0.0526817	0.05799781	0.2675757
ENSG00000059378	PARP12	7	139743532	A	G	0.411531	-0.0205241	0.014344	0.1299999	0.091499	0.00806599	7.96E-30	-0.224309	0.158009	0.1557239	0.2689049
ENSG00000059573	ALDH18A1	10	97391079	C	T	0.492048	0.00565783	0.0142299	0.7800007	-0.146116	0.00805196	1.37E-73	-0.0387216	0.0974113	0.6909943	0.9823762
ENSG00000059588	TARBP1	1	234570954	T	C	0.446322	-0.0293576	0.0143837	0.04900044	0.222855	0.00801828	5.21E-170	-0.131734	0.0647166	0.04179544	0.1277802
ENSG00000059691	GATB	4	152636915	C	T	0.378728	0.0126376	0.0147219	0.2999998	-0.350166	0.00900449	0	-0.0360903	0.0420528	0.390775	0.4930779
ENSG00000059728	MXD1	2	70147448	A	G	0.182903	0.00717375	0.0185185	0.5199996	0.103431	0.0104783	5.56E-23	0.0693576	0.179179	0.6986933	0.8102556
ENSG00000059758	CDK17	12	96733188	A	C	0.213718	0.00100866	0.0176443	0.84	-0.0822828	0.00945236	3.18E-18	-0.0122585	0.214439	0.9544137	0.9996136
ENSG00000059769	DNAJC25	9	114413070	T	C	0.276342	0.0197885	0.0169935	0.28	-0.143488	0.00914773	1.90E-55	-0.137911	0.118758	0.2455285	0.06458086
ENSG00000059804	SLC2A3	12	8080348	C	T	0.49006	-0.0109749	0.0142003	0.3800004	0.224165	0.00822488	1.47E-163	-0.0489591	0.063373	0.4397867	0.8897224
ENSG00000060069	CTDP1	18	77477155	G	C	0.43837	0.012883	0.0142575	0.3100002	-0.176661	0.00795956	3.86E-109	-0.0729251	0.0807724	0.3666073	0.6409569
ENSG00000060138	YBX3	12	10863797	T	C	0.357853	-0.00777001	0.0148672	0.4700002	-0.250286	0.00909092	7.39E-167	0.0310445	0.0594116	0.6012989	0.1459615
ENSG00000060140	STYK1	12	10799227	C	T	0.37674	-0.0218216	0.0145156	0.14	-0.113686	0.0080831	6.27E-45	0.191947	0.128409	0.1349651	0.3444671
ENSG00000060237	WNK1	12	941188	G	C	0.49503	0.0146295	0.0143499	0.4700002	-0.100418	0.00874261	1.55E-30	-0.145687	0.143464	0.3098713	0.7665565
ENSG00000060491	OGFR	20	61440769	C	T	0.44831	0.00350626	0.0142633	0.64	-0.148621	0.00910611	7.00E-60	-0.023592	0.0959819	0.8058401	0.7895575
ENSG00000060558	GNA15	19	3149979	C	T	0.333996	0.0264564	0.0154132	0.08100093	0.122767	0.00933691	1.73E-39	0.2155	0.126613	0.08874878	0.1562322
ENSG00000060566	CREB3L3	19	4163324	T	C	0.262425	-0.0071139	0.0161134	0.5099998	-0.0860576	0.00970866	7.72E-19	0.0826644	0.187472	0.6592542	0.9678908
ENSG00000060642	PIGV	1	27119426	A	G	0.0715706	-0.0426965	0.0261987	0.1299999	-0.523635	0.0146363	2.56E-280	0.0815387	0.0500843	0.1035185	0.2179651
ENSG00000060656	PTPRU	1	29608176	C	A	0.0159046	0.00734286	0.0562641	0.95	0.427327	0.0368792	4.79E-31	0.0171832	0.131674	0.8961718	0.6462737
ENSG00000060688	SNRNP40	1	31751039	G	A	0.377734	0.0167676	0.0146765	0.1900002	-0.171135	0.00900559	1.60E-80	-0.0979788	0.0859147	0.2541112	0.3256482
ENSG00000060709	RIMBP2	12	131040754	A	G	0.261431	0.00472796	0.0163514	0.58	0.212635	0.00918242	1.24E-118	0.022235	0.0769047	0.7724861	0.1113715
ENSG00000060749	QSER1	11	32964793	T	A	0.0417495	-0.0624603	0.0371329	0.1199999	0.342127	0.0218715	3.73E-55	-0.182565	0.109161	0.09443898	0.6319132
ENSG00000060762	MPC1	6	166787446	T	G	0.333002	0.0064219	0.015017	0.5	0.458913	0.00915068	0	0.0139937	0.0327242	0.6689231	0.5458619

ENSG00000060971	ACAA1	3	38171467	C	A	0.0506958	-0.0186453	0.0346532	0.3800004	-0.45176	0.0271233	2.75E-62	0.0412726	0.0767472	0.5907333	0.6591674
ENSG00000060982	BCAT1	12	25033344	C	T	0.213718	-0.0239217	0.0180467	0.1100001	0.967718	0.00837218	0	-0.0247197	0.0186499	0.1850196	0.6654732
ENSG00000061273	HDAC7	12	48201710	C	T	0.0477137	-0.0234581	0.0316015	0.29	-0.171433	0.018477	1.72E-20	0.136835	0.184926	0.4593332	0.816464
ENSG00000061337	LZTS1	8	20132575	G	A	0.186879	0.0279926	0.0186259	0.0990011	0.271005	0.0101867	6.12E-156	0.103292	0.0688385	0.1334866	0.08922679
ENSG00000061676	NCKAP1	2	183846595	C	T	0.272366	-0.013514	0.016449	0.2099999	0.228031	0.00884772	1.79E-146	-0.059264	0.0721717	0.4115594	0.328863
ENSG00000061794	MRPS35	12	27886467	A	G	0.172962	0.0184959	0.0189221	0.29	-0.235118	0.0118354	8.08E-88	-0.0786665	0.0805766	0.3289183	0.1110185
ENSG00000061936	SFSWAP	12	132239954	T	C	0.329026	-0.00766975	0.0152735	0.6100002	0.098859	0.00917008	4.25E-27	-0.0775827	0.154665	0.6159372	0.3789673
ENSG00000061938	TNK2	3	195614525	C	T	0.570577	0.0161211	0.0148234	0.17	0.105432	0.00864737	3.41E-34	0.152905	0.141155	0.2787005	0.7691713
ENSG00000061987	MON2	12	62925980	C	T	0.114314	0.0419639	0.0234369	0.09599973	0.17564	0.0123454	6.21E-46	0.23892	0.13449	0.07565179	0.9568671
ENSG00000062194	GPBP1	5	56515140	T	C	0.240557	-0.0461831	0.0162422	0.003400008	0.192125	0.00882709	4.94E-105	-0.24038	0.085258	0.004810684	0.03158345
ENSG00000062282	DGAT2	11	75496178	G	C	0.0775348	-0.00422085	0.025393	0.7700005	-0.256576	0.0131788	2.02E-84	0.0164507	0.0989722	0.8679878	0.03422791
ENSG00000062582	MRPS24	7	43907824	G	A	0.0864811	0.00444712	0.0277281	0.9400001	0.200117	0.0162106	5.20E-35	0.0222226	0.138571	0.8725899	0.9942794
ENSG00000062598	ELMO2	20	45028196	C	T	0.234592	-0.00296336	0.0168482	0.99	-0.120224	0.00979904	1.33E-34	0.0246486	0.140154	0.8603978	0.7569009
ENSG00000062650	WAPL	10	88238292	A	G	0.493042	-0.0143922	0.0142483	0.2	-0.125722	0.00795797	3.20E-56	0.114477	0.113564	0.3134347	0.9430026
ENSG00000062716	VMP1	17	57852084	C	T	0.150099	0.00927405	0.0194693	0.6700003	0.142015	0.0122727	5.74E-31	0.0653035	0.13721	0.6341177	0.2661509
ENSG00000062725	APPBP2	17	58562050	C	T	0.0765408	0.0711315	0.0243173	0.002100003	-0.0890273	0.0141259	2.93E-10	-0.798985	0.30113	0.00797126	0.3863541
ENSG00000062822	POLD1	19	50904367	T	C	0.234592	-0.012459	0.016934	0.3599996	0.0788342	0.0143937	4.33E-08	-0.158041	0.216735	0.4658861	0.8683235
ENSG00000063046	EIF4B	12	53417967	A	G	0.167992	-0.000773967	0.0200106	0.8700001	0.454099	0.0172808	3.46E-152	-0.0017044	0.0440667	0.9691473	0.2272334
ENSG00000063127	SLC6A16	19	49810688	T	C	0.296223	0.00415625	0.015743	0.7899998	-0.182458	0.00867235	2.88E-98	-0.0227793	0.0862898	0.7917911	0.5248655
ENSG00000063169	BICRA	19	48158993	T	C	0.139165	0.0191004	0.020986	0.2599998	0.221256	0.0117332	2.56E-79	0.0863272	0.0949599	0.3633019	0.5294486
ENSG00000063176	SPHK2	19	49128261	C	T	0.265408	0.000830237	0.0167374	0.6499995	0.171944	0.00870015	6.15E-87	0.00482852	0.0973422	0.9604383	0.3573044
ENSG00000063180	CA11	19	49145384	A	C	0.217694	0.00176527	0.0176645	0.7300002	0.216025	0.0152962	2.75E-45	0.00817161	0.0817727	0.9203993	0.1097419
ENSG00000063241	ISOC2	19	55969031	T	C	0.449304	0.00756343	0.0144198	0.7300002	0.189111	0.0100389	3.70E-79	0.0399947	0.07628	0.6000603	0.9652718
ENSG00000063244	U2AF2	19	56175796	G	C	0.0725646	-0.0334328	0.028867	0.2700001	0.157018	0.0152063	5.39E-25	-0.212924	0.184998	0.2497529	0.2603231
ENSG00000063245	EPN1	19	56203908	A	G	0.026839	-0.0412074	0.0438798	0.4	0.69987	0.0333233	6.24E-98	-0.0588787	0.0627597	0.3481622	0.6880587
ENSG00000063322	MED29	19	39886610	A	G	0.356859	0.0129558	0.0149923	0.3900004	-0.240486	0.00835439	3.25E-182	-0.0538734	0.0623698	0.3877118	0.6464452
ENSG00000063438	AHRR	5	371348	T	C	0.297217	-0.00441524	0.0158218	0.6100002	0.14667	0.00875013	4.63E-63	-0.0301033	0.107889	0.780228	0.8398483
ENSG00000063660	GPC1	2	241391290	C	T	0.466203	0.0205268	0.0145306	0.1499999	-0.0861302	0.00933842	2.88E-20	-0.238323	0.170673	0.1626012	0.5939011
ENSG00000063761	ADCK1	14	78333890	T	C	0.0477137	0.0470638	0.0300643	0.04600023	1.31824	0.0172335	0	0.0357019	0.0228111	0.117557	0.9661713
ENSG00000063854	HAGH	16	1861408	T	A	0.143141	0.0437949	0.0203403	0.05999983	0.376414	0.0115192	3.30E-234	0.116348	0.0541543	0.03167794	0.5632334
ENSG00000063978	RNF4	4	2545497	G	A	0.424453	-0.0114905	0.0144594	0.4799997	-0.119805	0.00852192	6.84E-45	0.0959104	0.120884	0.4275408	0.2535631
ENSG00000064012	CASP8	2	202125300	A	C	0.385686	0.00834255	0.0147938	0.64	-0.21219	0.00808379	7.38E-152	-0.0393165	0.0697357	0.5728957	0.6263007
ENSG00000064042	LIMCH1	4	41531842	G	A	0.395626	0.00202535	0.0146694	0.7800007	0.119294	0.00825437	2.43E-47	0.0169778	0.122974	0.890193	0.7156518
ENSG00000064102	INTS13	12	27074686	A	G	0.185885	-0.0184227	0.0181835	0.2300001	-0.101946	0.011475	6.44E-19	0.180711	0.17952	0.3141125	0.3772042
ENSG00000064115	TM7SF3	12	27146747	A	G	0.129225	-0.00536072	0.0227165	0.7300002	-0.292309	0.0136041	2.07E-102	0.0183392	0.0777186	0.8134565	0.999261
ENSG00000064199	SPA17	11	124555554	A	G	0.0198807	0.00353515	0.0459982	0.64	-0.577599	0.0278203	9.59E-96	-0.00612043	0.0796375	0.93874	0.4964114
ENSG00000064201	TSPAN32	11	2331328	A	T	0.430417	-0.0231676	0.0143994	0.1100001	-0.470057	0.00774491	0	0.0492868	0.030644	0.1077557	0.5971429
ENSG00000064225	ST3GAL6	3	98495562	A	T	0.342942	0.0305123	0.0147098	0.02199986	0.262373	0.00805267	7.35E-233	0.116294	0.056178	0.03844364	0.007887984
ENSG00000064270	ATP2C2	16	84449963	C	G	0.298211	0.00825119	0.0156429	0.7099994	0.26793	0.00935888	2.98E-180	0.0307961	0.0583942	0.5979281	0.987982
ENSG00000064300	NGFR	17	47582517	T	C	0.262425	-0.00739236	0.0161426	0.4899999	0.0707134	0.00975654	4.24E-13	-0.10454	0.228737	0.6476497	0.7024349
ENSG00000064309	CDON	11	125879460	A	G	0.144135	-0.00880061	0.0192891	0.8	0.116645	0.0112038	2.20E-25	-0.0754478	0.165525	0.6485264	0.7424603
ENSG00000064313	TAF2	8	120794059	C	T	0.196819	-0.00154834	0.0169643	0.9599999	-0.296509	0.00928377	7.83E-224	0.0052219	0.0572137	0.927278	0.8776203
ENSG00000064393	HIPK2	7	139361946	C	T	0.315109	0.00545082	0.0151984	0.9299999	-0.0717823	0.00829049	4.78E-18	-0.0759354	0.211911	0.7200911	0.9578439
ENSG00000064419	TNPO3	7	128645073	G	C	0.367793	0.00763045	0.0145899	0.6300007	0.399196	0.00784934	0	0.0191145	0.0365501	0.6009967	0.4501564
ENSG00000064489	NA	19	19279888	A	G	0.11332	0.0308026	0.0206078	0.1499999	-0.3221	0.025864	1.34E-35	-0.0956305	0.0644387	0.1377943	0.6000245
ENSG00000064490	RFXANK	19	19307843	T	G	0.028827	0.0352035	0.0402085	0.2700001	0.84787	0.0226132	1.13E-307	0.0415199	0.0474359	0.3814187	0.8728941
ENSG00000064545	TMEM161A	19	19239650	G	A	0.0298211	0.0193612	0.0387102	0.7400005	-0.327969	0.0227937	6.10E-47	-0.0590336	0.118101	0.6171767	0.987605
ENSG00000064547	LPAR2	19	19737108	C	G	0.241551	-0.0277782	0.0161397	0.07900053	-0.560702	0.00858213	0	0.0495418	0.0287948	0.0853392	0.5548668
ENSG00000064601	CTSA	20	44523525	C	G	0.138171	0.0007098	0.0194281	0.9299999	0.153281	0.0107567	4.50E-46	0.0046307	0.126749	0.9708561	0.9674978
ENSG00000064607	SUGP2	19	19123264	A	T	0.253479	-0.0119379	0.0158476	0.4100001	0.470076	0.00973785	0	-0.0253957	0.0337169	0.4513284	0.4232658

ENSG00000064651	SLC12A2	5	127472419	T	C	0.210736	0.054965	0.0165004	0.000549997	-0.18575	0.00943853	3.20E-86	-0.295908	0.0900947	0.001021949	0.7717512
ENSG00000064655	EYA2	20	45670377	A	G	0.377734	0.0165988	0.0146267	0.2399999	0.052244	0.00832335	3.46E-10	0.317717	0.284508	0.2641124	0.2527975
ENSG00000064666	CNN2	19	1032640	T	C	0.0874751	0.0184823	0.0256653	0.4799997	-0.681461	0.01811	21582699999999e-3	-0.0271216	0.0376691	0.4715274	0.9358422
ENSG00000064687	ABCA7	19	1052836	T	C	0.0874751	0.0184823	0.0256653	0.4799997	-0.533767	0.0184432	3.62E-184	-0.0346261	0.0480982	0.4715834	0.8293273
ENSG00000064703	DDX20	1	112304252	G	C	0.114314	-0.00967789	0.0222762	0.7199992	0.203861	0.0123431	2.81E-61	-0.0474731	0.109309	0.6640709	0.5788396
ENSG00000064726	BTBD1	15	83710640	G	A	0.324056	-0.00746242	0.0153106	0.6999999	0.121172	0.00855816	1.65E-45	-0.0615856	0.12643	0.6261778	0.8608978
ENSG00000064787	BCAS1	20	52620310	C	A	0.342942	0.0100984	0.0147651	0.5199996	0.212074	0.0119867	4.80E-70	0.0476174	0.0696745	0.4943373	0.4090855
ENSG00000064886	CHI3L2	1	111764727	A	C	0.0984095	-0.0224086	0.0227214	0.33	1.07466	0.011649	0	-0.0208518	0.021144	0.3240463	0.2027495
ENSG00000064933	PMS1	2	190695731	C	T	0.152087	-0.0226643	0.0203963	0.16	0.532675	0.0162375	4.95E-236	-0.0425481	0.0383123	0.2667579	0.3992106
ENSG00000064989	CALCRL	2	188260521	C	T	0.299205	-0.00344039	0.0155728	0.6600001	-0.212398	0.00845333	2.59E-139	0.0161978	0.0733217	0.825159	0.4968428
ENSG00000064995	TAF11	6	34850710	T	C	0.203777	0.0167487	0.0179911	0.58	0.0797864	0.00990618	8.00E-16	0.209919	0.226992	0.3550769	0.2540451
ENSG00000064999	ANKS1A	6	34958110	T	C	0.114314	-0.0187186	0.0232297	0.2999998	0.210828	0.0122421	1.83E-66	-0.0887862	0.110304	0.4208635	0.3633341
ENSG00000065000	AP3D1	19	2132726	C	T	0.196819	-0.0179419	0.0168528	0.2999998	-0.263758	0.010818	2.69E-131	0.068024	0.0639558	0.2875051	0.5373395
ENSG00000065029	ZNF76	6	35245224	G	C	0.167992	0.0164368	0.0186073	0.3599996	-0.280732	0.0106928	6.40E-152	-0.0585499	0.0663189	0.3773159	0.5347661
ENSG00000065054	NHERF2	16	2082192	C	T	0.0198807	-0.0220619	0.0483829	0.6300007	0.217025	0.0389885	2.60E-08	-0.101656	0.223683	0.6494955	0.5585896
ENSG00000065057	NTHL1	16	2093841	C	T	0.232604	0.00717981	0.0170178	0.8499999	0.109571	0.011519	1.87E-21	0.0655263	0.155465	0.6734005	0.9723251
ENSG00000065060	BLTP3A	6	34805386	G	A	0.335984	0.0387804	0.0150925	0.025	0.829128	0.00769514	0	0.0467725	0.018208	0.0102056	0.265981
ENSG00000065135	GNAI3	1	110114104	A	T	0.16998	-0.0114953	0.018526	0.6800001	-0.639824	0.00947388	0	0.0179663	0.028956	0.5349488	0.3272677
ENSG00000065150	IPO5	13	98641146	G	T	0.446322	-0.00695463	0.014331	0.58	-0.0565165	0.00798707	1.48E-12	0.123055	0.254167	0.6282805	0.3692469
ENSG00000065154	OAT	10	126096708	A	G	0.152087	-0.0214991	0.0213103	0.29	-0.31526	0.0112528	1.03E-172	0.0681949	0.0676399	0.3133555	0.2957987
ENSG00000065183	WDR3	1	118487696	A	G	0.0208748	-0.0201961	0.0402838	0.6100002	0.320022	0.025514	4.34E-36	-0.0631085	0.125979	0.6164095	0.7965494
ENSG00000065268	WDR18	19	988871	T	C	0.121272	-0.0081403	0.0218177	0.7499995	0.803024	0.0143349	0	-0.0101371	0.02717	0.7090762	0.3777928
ENSG00000065308	TRAM2	6	52401956	C	G	0.28827	-0.00938125	0.0155733	0.4100001	-0.231727	0.00860655	1.13E-159	0.040484	0.0672222	0.5470127	0.4955346
ENSG00000065320	NTN1	17	9036088	C	G	0.260437	0.0300593	0.0157558	0.06800017	-0.11096	0.00991189	4.33E-29	-0.270903	0.144043	0.06001164	0.226264
ENSG00000065361	ERBB3	12	56485465	C	T	0.414513	-0.0181673	0.014317	0.2	-0.167927	0.0081462	2.05E-94	0.108185	0.0854184	0.2053217	0.3704166
ENSG00000065413	ANKRD44	2	198003819	T	C	0.194831	-0.0214561	0.0183049	0.2300001	0.170274	0.0101126	1.29E-63	-0.126009	0.107763	0.2422744	0.4343079
ENSG00000065427	KARS1	16	75672081	T	G	0.0606362	-0.00932648	0.0349676	0.7400005	0.193467	0.0211911	6.88E-20	-0.0482072	0.180819	0.7897741	0.02925819
ENSG00000065457	ADAT1	16	75644038	A	G	0.277336	0.0194781	0.0162691	0.3400001	0.654308	0.0081064	0	0.029769	0.0248673	0.2312624	0.5088506
ENSG00000065485	PDIA5	3	122864991	G	A	0.411531	0.0149183	0.0144261	0.35	0.193943	0.0118878	7.80E-60	0.0769211	0.0745326	0.3020499	0.05209814
ENSG00000065491	TBC1D22B	6	37263147	G	A	0.150099	-0.00588269	0.0208876	0.5700002	-0.144264	0.0111998	5.76E-38	0.0407771	0.144822	0.7782746	0.245518
ENSG00000065526	SPEN	1	16220657	A	G	0.0984095	-0.00163667	0.0235	0.89	0.188421	0.0129057	2.82E-48	-0.00868625	0.124722	0.9444764	0.3809239
ENSG00000065534	MYLK	3	123466037	C	T	0.0725646	0.034054	0.0262335	0.17	-0.169163	0.0144544	1.23E-31	-0.201309	0.15603	0.1969817	0.6429898
ENSG00000065548	ZC3H15	2	187362486	T	G	0.16998	0.0177603	0.0207448	0.3400001	0.114804	0.0116316	5.62E-23	0.154701	0.181376	0.3936982	0.6035426
ENSG00000065559	MAP2K4	17	11985644	A	G	0.219682	-0.00757228	0.016996	0.7700005	0.151133	0.0143497	6.14E-26	-0.0501033	0.112557	0.6562222	0.4948387
ENSG00000065600	PACC1	1	212562758	A	G	0.367793	-0.0063448	0.0146308	0.4799997	-0.270914	0.00895672	5.71E-201	0.02342	0.0540109	0.6645674	0.9436081
ENSG00000065613	SLK	10	105757975	C	T	0.428429	-0.0132642	0.0144543	0.2700001	0.203759	0.00829097	2.28E-133	-0.0650975	0.0709877	0.3591292	0.04964187
ENSG00000065615	CYB5R4	6	84619754	T	C	0.027833	-0.00727173	0.0431964	0.89	0.235212	0.0332784	1.57E-12	-0.0309157	0.183701	0.8663525	0.4879019
ENSG00000065618	COL17A1	10	105818402	G	A	0.193837	-0.000652509	0.0183645	0.9599999	0.188781	0.0101556	3.96E-77	-0.00345644	0.0972797	0.9716564	0.006348861
ENSG00000065621	GSTO2	10	106044123	G	A	0.366799	-0.00628122	0.0149708	0.4500005	0.209255	0.00832319	1.76E-139	-0.0300171	0.0715533	0.6748461	0.347202
ENSG00000065665	SEC61A2	10	12191798	T	C	0.517893	-0.0033189	0.0141712	0.5999997	0.39336	0.00763174	0	-0.00843731	0.0360264	0.8148313	0.05720954
ENSG00000065675	PRKCQ	10	6545684	C	T	0.307157	-0.000946413	0.0151534	0.95	-0.158192	0.00876199	7.29E-73	0.00598267	0.0957915	0.9502004	0.8199827
ENSG00000065717	TLE2	19	3022634	G	A	0.479125	-0.00919485	0.0143174	0.3800004	0.13642	0.0104292	4.25E-39	-0.0674009	0.105077	0.5212353	0.5319619
ENSG00000065802	ASB1	2	239348137	T	A	0.441352	0.011179	0.0143223	0.3700002	-0.380626	0.00775204	0	-0.02937	0.037633	0.4351364	0.4171187
ENSG00000065809	FAM107B	10	14688726	T	A	0.168986	0.0134505	0.0200509	0.6700003	0.395004	0.0104961	09140500000001e-3	0.0340516	0.0507693	0.5024045	0.8180924
ENSG00000065833	ME1	6	84030452	C	T	0.459245	0.00135072	0.014246	0.6899999	-0.0835496	0.00796788	1.00E-25	-0.0161667	0.170516	0.9244657	0.6036406
ENSG00000065882	TBC1D1	4	38016752	T	A	0.124254	-0.00421605	0.0231437	0.8	0.27529	0.012193	7.16E-113	-0.0153149	0.0840729	0.8554553	0.03521593
ENSG00000065883	CDK13	7	40063184	C	T	0.348907	0.0106992	0.0148145	0.5099998	0.194951	0.00828408	1.87E-122	0.0548815	0.0760267	0.4703735	0.4163928
ENSG00000065911	MTHFD2	2	74435190	C	T	0.464215	0.00239756	0.0142181	0.8499999	0.17156	0.00794938	2.67E-103	0.013975	0.0828777	0.8660938	0.4373075
ENSG00000065970	FOXJ2	12	8196699	A	G	0.311133	-0.00650822	0.0154033	0.8200001	-0.273992	0.00833161	3.51E-237	0.0237534	0.0562228	0.6726701	0.7834585
ENSG00000065989	PDE4A	19	10553877	C	T	0.0854871	-0.0259847	0.0249053	0.2099999	-0.233278	0.0140429	5.72E-62	0.11139	0.106973	0.2977417	0.123375

ENSG00000066027	PPP2R5A	1	212497039	T	A	0.17992	-0.00149791	0.0183018	0.95	0.829425	0.00882159	0	-0.00180596	0.0220657	0.93477	0.2334687
ENSG00000066032	CTNNA2	2	80144131	C	T	0.449304	-0.00490253	0.0142984	0.5999997	-0.166852	0.00879723	3.23E-80	0.0293826	0.0857093	0.7317364	0.9581698
ENSG00000066056	TIE1	1	43777721	C	T	0.371769	0.00775524	0.0149933	0.5700002	0.0499438	0.00839082	2.65E-09	0.155279	0.301335	0.6063399	0.6104582
ENSG00000066084	DIP2B	12	51020609	A	G	0.342942	0.00951125	0.0148231	0.2599998	-0.222441	0.00919899	3.53E-129	-0.0427586	0.0666619	0.5212468	0.3008997
ENSG00000066117	SMARCD1	12	50486625	C	T	0.327038	-0.00388082	0.0149032	0.9199999	0.125481	0.00846677	1.08E-49	-0.0309276	0.118787	0.7945849	0.9371788
ENSG00000066135	KDM4A	1	44143507	T	G	0.246521	0.0139621	0.0163768	0.32	0.053819	0.00903247	2.55E-09	0.259427	0.307393	0.3986928	0.1093642
ENSG00000066136	NFYC	1	41197297	G	A	0.323062	0.00179948	0.0149416	0.7899998	-0.187905	0.00824154	4.61E-115	-0.00957652	0.0795177	0.9041405	0.8182249
ENSG00000066185	ZMYND12	1	42908969	C	T	0.163022	0.0150895	0.019432	0.5	-0.361201	0.0110643	9.28E-234	-0.0417759	0.0538135	0.4375667	0.5096709
ENSG00000066230	SLC9A3	5	498936	G	A	0.257455	0.0163707	0.01552	0.1499999	0.829886	0.0146349	0	0.0197264	0.0187046	0.2915943	0.5846173
ENSG00000066294	CD84	1	160530095	A	G	0.0914513	-0.0172813	0.0277545	0.2999998	0.529224	0.0134888	0	-0.032654	0.0524503	0.533567	0.377558
ENSG00000066322	ELOVL1	1	43831382	G	A	0.44831	-0.0213809	0.0142222	0.1299999	-0.0800604	0.00800972	1.60E-23	0.26706	0.179641	0.1371136	0.004185207
ENSG00000066336	SPI1	11	47388269	G	A	0.0298211	-0.00667043	0.0416359	0.8200001	-0.79572	0.0227688	1.40E-267	0.00838289	0.0523254	0.8727181	0.7596337
ENSG00000066379	POLR1H	6	30029681	A	G	0.0318091	-0.00606373	0.0357027	0.6999999	0.904646	0.0354124	6.08E-144	-0.00670288	0.0394668	0.8651393	0.9278676
ENSG00000066382	MPPED2	11	30507229	T	G	0.255467	-0.00465269	0.0161396	0.7600007	0.181793	0.00904895	9.03E-90	-0.0255933	0.088789	0.7731567	0.5092001
ENSG00000066427	ATXN3	14	92548930	A	G	0.0815109	0.0175247	0.0238414	0.6300007	0.329519	0.0134069	2.16E-133	0.0531827	0.0723846	0.4625071	0.1781259
ENSG00000066455	GOLGA5	14	93283442	C	T	0.316103	-0.0180355	0.015036	0.2700001	0.116748	0.00857002	2.93E-42	-0.154483	0.129289	0.2321398	0.688539
ENSG00000066468	FGFR2	10	123297910	A	G	0.518887	0.00454458	0.0142121	0.89	-0.405175	0.00761785	0	-0.0112163	0.0350771	0.7491483	0.997177
ENSG00000066557	LRRC40	1	70640895	C	T	0.17495	0.0148124	0.0195842	0.3800004	-0.0807951	0.0108636	1.03E-13	-0.183333	0.243644	0.4517725	0.5513081
ENSG00000066583	ISOC1	5	128440082	T	C	0.362823	0.0063269	0.0146531	0.6899999	-0.410308	0.00793554	0	-0.0154199	0.0357137	0.665913	0.9747233
ENSG00000066651	TRMT11	6	126334256	T	G	0.460239	-0.0280417	0.0143124	0.0329997	0.0755712	0.00794734	1.93E-21	-0.371063	0.193368	0.05499074	0.3184392
ENSG00000066654	THUMPD1	16	20749196	G	A	0.419483	0.00665668	0.0143984	0.6999999	0.189019	0.00805713	1.05E-121	0.0352171	0.0761893	0.6439156	0.9084747
ENSG00000066697	MSANTD3	9	103201474	T	C	0.166998	0.0151385	0.0183492	0.5300002	-0.311504	0.0199833	8.76E-55	-0.0485981	0.0589876	0.4100139	0.8177526
ENSG00000066735	KIF26A	14	104626145	A	C	0.457256	0.00286055	0.0145379	0.91	-0.33503	0.0117299	1.99E-179	-0.00853819	0.0433939	0.8440151	0.6660855
ENSG00000066739	ATG2B	14	96788901	T	C	0.224652	0.0110919	0.0171689	0.4799997	-0.148009	0.0110737	9.58E-41	-0.0749406	0.116134	0.5187375	0.7610875
ENSG00000066777	ARFGEF1	8	68170829	G	A	0.303181	0.00831798	0.0156853	0.6200004	-0.0733368	0.00873015	4.45E-17	-0.113422	0.214306	0.5966316	0.835821
ENSG00000066827	ZFAT	8	135607661	G	A	0.411531	-0.0049958	0.0145122	0.7499995	-0.125744	0.00807967	1.30E-54	0.03973	0.115439	0.7307229	0.4483064
ENSG00000066855	MTFR1	8	66620232	T	C	0.154076	-0.00224594	0.0199266	0.9699999	0.657497	0.0107522	0	-0.00341589	0.0303068	0.9102601	0.8908266
ENSG00000066923	STAG3	7	99797148	T	G	0.227634	0.0366002	0.0171669	0.02300011	-0.488035	0.00932571	0	-0.0749951	0.0352048	0.03315062	0.2575174
ENSG00000066926	FECH	18	55234759	C	A	0.173956	-0.0231249	0.0202444	0.32	0.595363	0.0107908	0	-0.0388417	0.0340107	0.2534368	0.5076933
ENSG00000066933	MYO9A	15	72262775	A	C	0.204771	0.00440957	0.0172436	0.7300002	-0.226075	0.0101935	5.55E-109	-0.0195049	0.0762788	0.7981785	0.4457233
ENSG00000067057	PFKP	10	3144808	G	A	0.382704	0.00624039	0.0146006	0.5400003	0.457736	0.00776186	0	0.0136332	0.0318982	0.6690915	0.5237089
ENSG00000067064	IDI1	10	1090479	C	G	0.333002	0.00971975	0.0149602	0.6999999	-0.13023	0.00832321	3.50E-55	-0.0746353	0.114974	0.5162428	0.5808313
ENSG00000067066	SP100	2	231344731	A	G	0.0864811	-0.0256317	0.0242571	0.28	0.198397	0.0138662	1.95E-46	-0.129194	0.122598	0.2919754	0.252272
ENSG00000067082	KLF6	10	3822830	C	T	0.542744	-0.00714191	0.0141917	0.58	0.0605348	0.00795234	2.69E-14	-0.11798	0.234951	0.6155624	0.8048193
ENSG00000067113	PLPP1	5	54775780	T	C	0.0506958	0.0926011	0.0351615	0.005099998	0.152464	0.02109	4.86E-13	0.607364	0.245449	0.01334204	0.0168557
ENSG00000067141	NEO1	15	73470799	T	C	0.350895	-0.0091694	0.0145515	0.4500005	-0.329542	0.00805375	0	0.0278247	0.044162	0.5286556	0.08660892
ENSG00000067167	TRAM1	8	71503149	G	C	0.483101	0.0190134	0.0141949	0.08999948	-0.207239	0.00787434	1.18E-152	-0.0917461	0.0685839	0.1809872	0.1267769
ENSG00000067182	TNFRSF1A	12	6444601	A	G	0.367793	0.0226867	0.0145382	0.0519996	0.253907	0.00813418	6.71E-214	0.0893505	0.0573295	0.1191047	0.681078
ENSG00000067208	EVI5	1	93116107	C	A	0.0934394	-0.00696539	0.0237332	0.64	0.288882	0.0131694	1.18E-106	-0.0241115	0.0821626	0.7691699	0.1776332
ENSG00000067221	STOML1	15	74281255	T	C	0.400596	-0.00756916	0.0145044	0.4700002	0.269127	0.00800338	6.86E-248	-0.0281249	0.0539008	0.6018167	0.2023143
ENSG00000067334	DNTTIP2	1	94339423	A	C	0.333996	0.00351782	0.0148203	0.7899998	-0.0887211	0.00835226	2.34E-26	-0.0396503	0.167085	0.8124196	0.6072468
ENSG00000067365	METTL22	16	8727810	A	G	0.269384	0.00099412	0.0158512	0.8	-0.237275	0.0100447	2.30E-123	-0.00418974	0.0668055	0.949993	0.2108722
ENSG00000067369	TP53BP1	15	43751166	C	G	0.0785288	0.0364958	0.0272884	0.2399999	-0.0913448	0.0142806	1.59E-10	-0.399539	0.305201	0.1905	0.1262462
ENSG00000067533	RRP15	1	218484977	C	T	0.404573	0.01581	0.0143605	0.33	0.266312	0.00790191	5.35E-249	0.0593664	0.0539523	0.2711798	0.4436083
ENSG00000067596	DHX8	17	41591532	A	G	0.478131	0.0352588	0.0142248	0.007100027	-0.14428	0.00793575	7.30E-74	-0.244378	0.0995037	0.01405065	0.6124647
ENSG00000067601	NA	7	66760211	C	G	0.417495	-0.0372133	0.0145004	0.004200007	-0.311487	0.0176193	6.11E-70	0.11947	0.0470401	0.0110934	0.632225
ENSG00000067606	PRKCZ	1	2049371	A	G	0.292247	-0.0131129	0.0159383	0.2999998	-0.148845	0.00947951	1.47E-55	0.0880974	0.107226	0.4113035	0.277897
ENSG00000067704	IARS2	1	220294412	T	C	0.0198807	-0.000541405	0.0693183	0.9400001	-0.324165	0.0488538	3.24E-11	0.00167015	0.213837	0.9937683	0.8874445
ENSG00000067715	SYT1	12	79551780	T	C	0.308151	0.00368971	0.0153652	0.83	-0.126549	0.0087853	4.84E-47	-0.0291565	0.121434	0.8102521	0.08453572
ENSG00000067798	NAV3	12	78415737	G	A	0.333002	0.0170345	0.0152744	0.2300001	-0.155944	0.00943707	2.44E-61	-0.109235	0.0981709	0.2658369	0.4339648

ENSG00000067836	ROGDI	16	4849960	A	G	0.269384	0.0267832	0.0159366	0.07399971	0.68475	0.00805023	0	0.0391138	0.0232781	0.09290256	0.550126
ENSG00000067900	ROCK1	18	18610756	A	G	0.0159046	0.0152697	0.0506287	0.84	0.428859	0.0462442	1.80E-20	0.0356054	0.118117	0.7630773	0.09791286
ENSG00000067955	CBFB	16	67098990	G	A	0.0308151	0.00775794	0.0417525	0.9699999	-0.122367	0.0201693	1.30E-09	-0.0633988	0.341366	0.8526638	0.459037
ENSG00000068024	HDAC4	2	240146606	T	C	0.428429	-0.0110286	0.0143151	0.4500005	-0.168582	0.00801878	4.01E-98	0.0654198	0.0849717	0.4413589	0.6743016
ENSG00000068028	RASSF1	3	50372815	T	C	0.0109344	-0.353643	0.200713	0.03699985	-0.523956	0.0429458	3.09E-34	0.674947	0.387046	0.08118651	0.04220552
ENSG00000068079	IFI35	17	41162607	T	C	0.0168986	0.0159557	0.0636251	0.84	-0.702972	0.0394052	3.48E-71	-0.0226975	0.0905176	0.8020058	0.3292972
ENSG00000068097	HEATR6	17	58138423	G	A	0.107356	0.0220069	0.0250039	0.5199996	1.03765	0.0115927	0	0.0212084	0.0240978	0.3788072	0.005530611
ENSG00000068120	COASY	17	40715890	A	C	0.0626243	0.0216358	0.0294679	0.6499995	-0.235658	0.019153	8.62E-35	-0.09181	0.125267	0.463612	0.1633708
ENSG00000068137	PLEKHH3	17	40824490	A	G	0.0497018	0.0247451	0.032106	0.5500004	0.140854	0.0169646	1.02E-16	0.175679	0.228918	0.4428248	0.2684563
ENSG00000068305	MEF2A	15	100137020	T	C	0.459245	-0.0371029	0.0142713	0.01899984	-0.153813	0.00795172	2.32E-83	0.24122	0.0936176	0.009976073	0.9963812
ENSG00000068383	INPP5A	10	134474151	C	T	0.375746	-0.0265591	0.0147187	0.06800017	-0.139079	0.00812703	1.18E-65	0.190964	0.106416	0.07273371	0.1877047
ENSG00000068489	PRR11	17	57257463	A	G	0.0457256	-0.0633893	0.0294796	0.02100003	0.435484	0.0237681	5.50E-75	-0.14556	0.0681584	0.0327104	0.07846295
ENSG00000068615	REEP1	2	86503161	A	G	0.127237	0.00226284	0.0220535	0.9199999	0.799618	0.0115531	0	0.0028299	0.0275801	0.9182751	0.5066037
ENSG00000068650	ATP11A	13	113443062	T	G	0.214712	0.000594333	0.0175471	0.98	-0.0931302	0.0116186	1.10E-15	-0.00638174	0.188416	0.9729805	0.952087
ENSG00000068654	POLR1A	2	86290308	T	C	0.159046	0.00623684	0.0194683	0.84	-0.207491	0.0108031	3.26E-82	-0.0300583	0.0938401	0.7487302	0.6200342
ENSG00000068697	LAPTM4A	2	20242100	A	T	0.328032	0.0214812	0.0147935	0.08999948	-0.0766298	0.00832607	3.46E-20	-0.280324	0.195439	0.1514786	0.5025367
ENSG00000068724	TTC7A	2	47223286	G	C	0.442346	-0.000724301	0.0143225	0.7400005	0.0771429	0.0087325	1.01E-18	-0.00938908	0.185665	0.9596682	0.3221992
ENSG00000068745	IP6K2	3	48751611	G	A	0.0646123	-0.0102719	0.030626	0.8499999	0.40653	0.0149888	5.39E-162	-0.0252673	0.0753409	0.7373442	0.1433514
ENSG00000068784	SRBD1	2	45727561	A	C	0.489066	0.0193446	0.0142557	0.2399999	-0.375254	0.00766621	0	-0.0515507	0.0380041	0.1749554	0.02882091
ENSG00000068793	NA	15	22949010	C	G	0.222664	-0.00412434	0.0172495	0.5700002	0.0855311	0.00973648	1.57E-18	-0.0482203	0.20175	0.8110974	0.199612
ENSG00000068796	KIF2A	5	61717532	C	T	0.477137	-0.00475616	0.0142119	0.6200004	-0.12697	0.00792747	9.82E-58	0.0374591	0.111956	0.7379364	0.810127
ENSG00000068831	RASGRP2	11	64503655	A	G	0.0467197	0.0184282	0.0313194	0.64	-0.0971126	0.0177771	4.69E-08	-0.189761	0.324372	0.5585396	0.1172869
ENSG00000068878	PSME4	2	54144590	G	T	0.082505	-0.0184009	0.0249842	0.4899999	-0.365906	0.0136779	1.18E-157	0.0502886	0.0683062	0.4615953	0.4537189
ENSG00000068903	SIRT2	19	39379849	G	T	0.389662	-0.00405818	0.0146321	0.6899999	0.0760785	0.00816031	1.13E-20	-0.053342	0.192414	0.7816073	0.3646504
ENSG00000068912	ERLEC1	2	54030068	A	G	0.0208748	-0.0504241	0.0605921	0.4299995	-0.382859	0.0423882	1.68E-19	0.131704	0.158933	0.4072858	0.8109366
ENSG00000068971	PPP2R5B	11	64693486	G	T	0.0467197	-0.0242774	0.0326088	0.4799997	-0.146045	0.021067	4.14E-12	0.166233	0.224564	0.4591496	0.2878761
ENSG00000069020	MAST4	5	66178799	G	A	0.271372	-0.0112547	0.0156194	0.6600001	-0.377599	0.0128962	1.87E-188	0.029806	0.0413776	0.4713151	0.1255903
ENSG00000069188	SDK2	17	71485375	A	G	0.138171	-0.0125174	0.0202696	0.7600007	-0.633003	0.0110388	0	0.0197746	0.0320232	0.5368986	0.2242987
ENSG00000069248	NUP133	1	229610574	T	C	0.202783	-0.00190862	0.0175195	0.8700001	-0.32296	0.00999251	3.64E-229	0.00590977	0.054247	0.9132485	0.596971
ENSG00000069275	NUCKS1	1	205700675	T	C	0.408549	-0.00953172	0.0143581	0.5099998	-0.320222	0.00789366	0	0.029766	0.044844	0.506839	0.9077278
ENSG00000069329	VPS35	16	46706742	G	A	0.162028	0.0146866	0.021005	0.5099998	0.144582	0.0108682	2.22E-40	0.10158	0.145482	0.4850323	0.0567939
ENSG00000069345	DNAJA2	16	46998499	G	A	0.055666	-0.0363329	0.0288066	0.2200002	-0.130838	0.0183046	8.82E-13	0.277695	0.223572	0.2142066	0.5235359
ENSG00000069399	BCL3	19	45257131	A	G	0.054672	0.00825846	0.0273355	0.6300007	0.0950631	0.0165252	8.79E-09	0.0868735	0.287947	0.7628815	0.6753774
ENSG00000069424	KCNAB2	1	6106389	C	G	0.451292	0.0010854	0.0142669	0.9400001	-0.232608	0.00790446	2.45E-190	-0.00466622	0.0613348	0.9393571	0.400018
ENSG00000069493	CLEC2D	12	9828841	A	T	0.445328	-0.00241567	0.0143366	0.7499995	-0.27238	0.00789245	5.39E-261	0.00886874	0.0526351	0.8661941	0.5007197
ENSG00000069667	RORA	15	61151000	C	T	0.199801	-0.000961021	0.017994	0.8800001	-0.129114	0.0107074	1.75E-33	0.00744319	0.139366	0.9574074	0.4084294
ENSG00000069696	DRD4	11	638999	T	C	0.0874751	0.0293419	0.0264655	0.2700001	-0.301483	0.0187585	4.02E-58	-0.0973251	0.0879929	0.2687021	0.1412991
ENSG00000069702	TGFBR3	1	92258897	T	C	0.242545	-0.0160402	0.0165501	0.2099999	0.107492	0.0090219	9.93E-33	-0.149222	0.154474	0.3340443	0.18468
ENSG00000069712	NA	1	92641411	T	G	0.0606362	0.0242428	0.0359013	0.4799997	0.199411	0.0255675	6.22E-15	0.121572	0.18071	0.5011091	0.5873661
ENSG00000069849	ATP1B3	3	141620161	A	G	0.122266	0.0154094	0.0197355	0.35	0.0963922	0.0113081	1.54E-17	0.159862	0.205599	0.4368396	0.6529719
ENSG00000069869	NEDD4	15	56202532	T	C	0.0248509	0.0311589	0.0419608	0.58	0.681098	0.0178888	0	0.045748	0.0616193	0.4578266	0.5624136
ENSG00000069943	PIGB	15	55629502	G	C	0.355865	-0.0021696	0.015019	0.95	0.192117	0.00896016	5.52E-102	-0.0112931	0.0781783	0.8851422	0.1821869
ENSG00000069956	MAPK6	15	52301382	T	C	0.158052	-0.0104694	0.0205741	0.56	-0.110311	0.0193196	1.13E-08	0.094908	0.187249	0.6122571	0.4058053
ENSG00000069966	GNB5	15	52448341	A	G	0.0377734	0.0097672	0.0369535	0.91	0.68186	0.02207	1.38E-209	0.0143244	0.0541972	0.7915481	0.8689897
ENSG00000069974	RAB27A	15	55553237	C	G	0.191849	0.037017	0.0185015	0.01700004	-0.136492	0.0101896	6.45E-41	-0.271202	0.137054	0.04783793	0.2202041
ENSG00000069998	HDHD5	22	17632289	C	G	0.2167	0.000847455	0.017813	0.95	-0.240892	0.00996403	3.96E-129	-0.00351798	0.073946	0.962055	0.04166712
ENSG00000070010	UFD1	22	19452101	T	A	0.481113	0.000802172	0.0142093	0.9699999	0.124776	0.00794075	1.22E-55	0.00642888	0.113879	0.9549804	0.1807935
ENSG00000070018	LRP6	12	12344452	A	G	0.27336	0.00440625	0.0154572	0.7700005	0.179263	0.00870938	3.91E-94	0.0245797	0.0862344	0.7756181	0.6354898
ENSG00000070019	GUCY2C	12	14807547	G	C	0.408549	-0.0129254	0.01469	0.3700002	0.210218	0.00815312	1.35E-146	-0.0614856	0.0699204	0.3792031	0.01119453
ENSG00000070047	PHRF1	11	594354	C	T	0.389662	0.00342938	0.0144268	0.7700005	0.105502	0.011538	6.03E-20	0.0325055	0.136791	0.8121691	0.9531766

ENSG00000070061	ELP1	9	111663096	C	A	0.194831	-0.00283532	0.0184028	0.98	-0.196704	0.0108197	7.41E-74	0.0144142	0.0935593	0.8775589	0.04121871
ENSG00000070081	NUCB2	11	17300610	G	T	0.429423	-0.00624785	0.0143571	0.8	0.413853	0.011508	3.26E-283	-0.0150968	0.0346939	0.6634586	0.7279822
ENSG00000070087	PFN2	3	149725633	T	C	0.378728	0.0293061	0.0147641	0.06100002	0.484872	0.00779969	0	0.0604409	0.030465	0.0472619	0.4146893
ENSG00000070182	SPTB	14	65279801	T	G	0.292247	-0.0141877	0.0155873	0.29	0.0979605	0.00949819	6.12E-25	-0.144831	0.159737	0.3645735	0.9027082
ENSG00000070190	DAPP1	4	100764650	A	T	0.39662	-0.00516113	0.0149389	0.59	-0.252692	0.00885472	4.02E-179	0.0204246	0.0591234	0.7297506	0.6600992
ENSG00000070214	SLC44A1	9	108104177	G	A	0.107356	-0.0165563	0.0226851	0.5700002	0.122092	0.0126437	4.62E-22	-0.135605	0.186333	0.4667631	0.3940204
ENSG00000070269	TMEM260	14	57036198	T	C	0.0427435	-0.100129	0.0327957	0.00179999	0.286659	0.0185723	9.55E-54	-0.349296	0.116623	0.002743723	0.8614908
ENSG00000070367	EXOC5	14	57703122	C	A	0.11829	-0.0201569	0.0203916	0.2700001	0.0783089	0.0115184	1.06E-11	-0.257402	0.263137	0.327973	0.4705177
ENSG00000070371	CLTCL1	22	19223112	T	C	0.37674	0.0275874	0.0147014	0.04600023	0.347456	0.00817928	0	0.0793983	0.0423528	0.0608358	0.9041835
ENSG00000070404	FSTL3	19	679888	A	G	0.358847	-0.00443722	0.0148971	0.6700003	0.155415	0.00952296	7.11E-60	-0.0285508	0.0958698	0.7658494	0.7832127
ENSG00000070413	DGCR2	22	19066881	C	T	0.310139	0.0112665	0.0153649	0.4700002	0.183578	0.00836826	1.14E-106	0.0613718	0.0837437	0.4636482	0.4818487
ENSG00000070423	RNF126	19	655367	A	G	0.0328032	-0.0019282	0.0327656	0.7499995	-0.447101	0.0273811	6.16E-60	0.00431268	0.0732851	0.9530732	0.6444399
ENSG00000070444	MNT	17	2295883	C	G	0.0497018	0.0112959	0.0339685	0.7099994	-0.187532	0.0175424	1.13E-26	-0.0602346	0.181222	0.7396026	0.2048565
ENSG00000070476	ZXDC	3	126175603	T	G	0.377734	-0.000905874	0.0148068	0.7499995	-0.203564	0.00811793	9.14E-139	0.00445008	0.0727382	0.9512163	0.5070763
ENSG00000070495	JMJD6	17	74715892	C	T	0.484095	-0.00594319	0.0142267	0.6300007	-0.37862	0.00778514	0	0.015697	0.0375765	0.6761419	0.8387504
ENSG00000070501	POLB	8	42212649	T	C	0.124254	-0.0178301	0.0219493	0.5400003	-0.249559	0.0118075	3.74E-99	0.0714466	0.0880175	0.4169455	0.8660214
ENSG00000070526	ST6GALNAC1	17	74630381	T	C	0.370775	0.00729732	0.0151356	0.6499995	0.380895	0.00837801	0	0.0191583	0.0397391	0.6297332	0.1170945
ENSG00000070540	WIPI1	17	66435371	G	T	0.10835	0.0261751	0.021444	0.1499999	-0.772764	0.0127677	0	-0.0338721	0.0277554	0.2223217	0.9138308
ENSG00000070610	GBA2	9	35743423	T	C	0.434394	0.0137971	0.01439	0.5099998	0.44484	0.00760156	0	0.0310159	0.0323531	0.3377255	0.09510846
ENSG00000070614	NDST1	5	149901577	C	T	0.0964215	0.00143958	0.0254245	0.8600001	-1.0271	0.0132817	0	-0.00140159	0.0247536	0.9548465	0.03254306
ENSG00000070669	ASNS	7	97491642	T	C	0.229622	-0.0251034	0.0172312	0.1	0.640389	0.00949323	0	-0.0392003	0.0269137	0.1452496	0.1784319
ENSG00000070718	AP3M2	8	42019827	A	G	0.398608	-0.0207819	0.0144341	0.16	-0.304514	0.00787614	0	0.0682462	0.0474334	0.1502127	0.1871264
ENSG00000070731	ST6GALNAC2	17	74571415	G	A	0.195825	0.0219738	0.0171535	0.2099999	-0.524777	0.00925911	0	-0.0418727	0.0326956	0.2003052	0.8186023
ENSG00000070756	PABPC1	8	101716540	C	T	0.146123	-0.0195602	0.0194918	0.4	0.285012	0.0251911	1.12E-29	-0.0686294	0.0686579	0.3175114	0.9884058
ENSG00000070759	TESK2	1	45883213	C	T	0.477137	0.00500969	0.0143432	0.5999997	-0.300833	0.00780182	0	-0.0166527	0.0476802	0.7268953	0.3364028
ENSG00000070761	CFAP20	16	58155425	T	C	0.0377734	0.0701336	0.0367925	0.04700023	-0.57947	0.0200941	7.19E-183	-0.121031	0.0636319	0.05716565	0.3490867
ENSG00000070770	CSNK2A2	16	58211817	A	G	0.141153	0.0127858	0.0193281	0.28	0.135963	0.0110257	6.14E-35	0.0940389	0.142362	0.5088921	0.6304586
ENSG00000070785	EIF2B3	1	45384366	G	A	0.2833	0.0218279	0.0151629	0.06800017	0.0684263	0.00942797	3.93E-13	0.318998	0.225911	0.1579348	0.08723428
ENSG00000070831	CDC42	1	22399278	T	C	0.214712	-0.00701499	0.016837	0.7400005	0.3435	0.00908022	0	-0.0204221	0.049019	0.6769593	0.3104193
ENSG00000070882	OSBPL3	7	24928705	C	G	0.377734	0.00642987	0.0147608	0.7600007	-0.283169	0.00822487	9.48E-260	-0.0227068	0.0521313	0.663149	0.9559988
ENSG00000070915	SLC12A3	16	56924440	T	C	0.286282	-0.0401561	0.0153144	0.01	-0.108513	0.0129754	6.11E-17	0.370056	0.147903	0.01234887	0.7513659
ENSG00000070950	RAD18	3	8911272	A	C	0.15507	-0.0203992	0.0187027	0.2700001	0.112206	0.0107005	1.00E-25	-0.181802	0.167581	0.2779855	0.8570018
ENSG00000071051	NCK2	2	106436042	C	T	0.45825	-0.00605697	0.0144078	0.5500004	0.158278	0.00804715	3.99E-86	-0.038268	0.0910494	0.6742669	0.4218496
ENSG00000071054	MAP4K4	2	102410880	G	A	0.352883	0.0310278	0.0149689	0.01700004	0.0568665	0.00936137	1.24E-09	0.545625	0.278131	0.04979133	0.737755
ENSG00000071073	MGAT4A	2	99291579	A	G	0.338966	-0.00959749	0.0150348	0.6100002	0.121371	0.00862045	5.08E-45	-0.0790758	0.124002	0.5236712	0.6419832
ENSG00000071082	RPL31	2	101629335	T	C	0.292247	-0.00473225	0.0154378	0.6499995	-0.380745	0.0123348	3.26E-209	0.0124289	0.0405483	0.7592077	0.4780379
ENSG00000071127	WDR1	4	10097268	T	C	0.290258	0.00474651	0.015416	0.7199992	0.191235	0.00850007	4.34E-112	0.0248204	0.0806206	0.7581841	0.3005303
ENSG00000071189	SNX13	7	17906295	C	T	0.119284	0.0117116	0.0225576	0.4799997	0.124227	0.0119099	1.80E-25	0.0942757	0.181808	0.6040784	0.3144127
ENSG00000071205	ARHGAP10	4	148823572	C	A	0.362823	-0.00159067	0.0145425	0.84	0.145508	0.00815159	2.88E-71	-0.0109319	0.0999451	0.9129021	0.6424054
ENSG00000071242	RPS6KA2	6	167071395	T	C	0.494036	0.0186366	0.0142071	0.1299999	0.311034	0.00777439	0	0.0599183	0.0457016	0.189832	0.372838
ENSG00000071243	ING3	7	120604036	G	A	0.212724	-0.00541363	0.0173768	0.9199999	-0.147441	0.00978116	2.40E-51	0.0367172	0.117881	0.755438	0.7948568
ENSG00000071246	VASH1	14	77238943	T	G	0.269384	-0.00107682	0.0165473	0.99	1.02853	0.00706455	0	-0.00104695	0.0160882	0.9481141	0.4816371
ENSG00000071282	LMCD1	3	8576599	T	C	0.506958	-0.00457782	0.0142126	0.6100002	-0.203809	0.00788532	2.66E-147	0.0224614	0.0697404	0.747399	0.6635308
ENSG00000071462	BUD23	7	73108423	C	T	0.0328032	0.0184433	0.0361302	0.5	1.02681	0.0245214	0	0.0179617	0.0351894	0.6097504	0.8646576
ENSG00000071537	SEL1L	14	81969049	G	A	0.474155	-0.00520373	0.0142638	0.7899998	0.0769654	0.00794571	3.44E-22	-0.0676113	0.185459	0.7154382	0.9855385
ENSG00000071564	TCF3	19	1630947	T	C	0.17495	-0.0234037	0.0189207	0.2	-0.124259	0.0126156	6.88E-23	0.188346	0.153464	0.2197112	0.4263318
ENSG00000071575	TRIB2	2	12869937	A	G	0.219682	0.0207428	0.0173474	0.14	0.321493	0.0107695	8.20E-196	0.0645202	0.0540021	0.2321763	0.7844533
ENSG00000071794	HLTF	3	148776127	C	A	0.338966	-0.0189314	0.0153932	0.08600031	-0.179322	0.00854182	7.54E-98	0.105572	0.0859884	0.2195409	0.2458439
ENSG00000071894	CPSF1	8	145626598	C	A	0.431412	0.00153397	0.014284	0.95	-0.82537	0.0107513	0	-0.00185852	0.0173062	0.914479	0.9442969
ENSG00000071909	MYO3B	2	171273168	A	G	0.250497	-0.0162244	0.0163968	0.4299995	0.136661	0.00901579	6.71E-52	-0.11872	0.120237	0.3234543	0.3242218

ENSG00000071967	CYBRD1	2	172396700	C	T	0.324056	0.00243855	0.0148787	0.95	-0.607106	0.00771178	0	-0.00401668	0.0245076	0.8698138	0.7788451
ENSG00000071994	PDCD2	6	170889081	C	G	0.50994	-0.0139402	0.0142499	0.25	-0.370898	0.00901911	0	0.037585	0.0384309	0.3280794	0.7904832
ENSG00000072042	RDH11	14	68153024	C	T	0.409543	0.007733	0.014675	0.9	-0.0780311	0.00807859	4.50E-22	-0.0991015	0.188346	0.5987714	0.7610029
ENSG00000072071	ADGRL1	19	14288874	A	G	0.335984	0.00942922	0.01525	0.5300002	-0.211304	0.0151133	2.02E-44	-0.0446239	0.0722413	0.5367693	0.5787494
ENSG00000072110	ACTN1	14	69393508	A	C	0.359841	0.00747053	0.0146636	0.6499995	0.107014	0.00811352	1.01E-39	0.0698088	0.137127	0.6106953	0.9842379
ENSG00000072121	ZFYVE26	14	68238699	C	T	0.409543	0.007733	0.014675	0.9	-0.104396	0.0080641	2.48E-38	-0.0740735	0.140687	0.5985307	0.6737524
ENSG00000072134	EPN2	17	19179478	G	A	0.0755467	0.013674	0.0280406	0.7800007	0.151668	0.014544	1.84E-25	0.0901574	0.185083	0.6261749	0.5357177
ENSG00000072135	PTPN18	2	131123281	G	A	0.244533	-0.00983218	0.0167987	0.56	-0.107712	0.00976163	2.61E-28	0.0912823	0.156179	0.5589025	0.9740286
ENSG00000072163	LIMS2	2	128417658	C	T	0.33499	0.00184139	0.0153215	0.81	-0.385019	0.0124002	1.16E-211	-0.0047826	0.0397945	0.9043386	0.8945215
ENSG00000072195	SPEG	2	220331288	A	C	0.413519	0.0170792	0.0146396	0.29	-0.125998	0.00809454	1.24E-54	-0.135551	0.116515	0.244675	0.1963971
ENSG00000072201	LNX1	4	54446520	C	T	0.291252	-0.0134442	0.0154834	0.4500005	-0.102982	0.00876441	7.06E-32	0.130549	0.150761	0.386525	0.03788131
ENSG00000072210	ALDH3A2	17	19566180	G	A	0.290258	0.00591881	0.0158041	0.8800001	-0.179729	0.0085815	2.14E-97	-0.0329318	0.0879469	0.7080687	0.9809223
ENSG00000072274	TFRC	3	195781557	G	A	0.227634	0.00724521	0.0178587	0.9199999	-0.336188	0.0105704	5.57E-222	-0.0215511	0.0531255	0.68499	0.8855414
ENSG00000072310	SREBF1	17	17727019	G	A	0.415507	0.0121077	0.0149045	0.4700002	-0.34167	0.00789454	0	-0.0354368	0.0436301	0.4166718	0.143667
ENSG00000072364	AFF4	5	132255198	C	G	0.274354	0.000720484	0.0162806	0.8700001	0.159655	0.00865273	5.08E-76	0.00451276	0.101974	0.9647019	0.2602428
ENSG00000072401	UBE2D1	10	60112069	A	G	0.467197	0.0397632	0.0142724	0.006299992	-0.839583	0.00956566	0	-0.0473607	0.017008	0.00535908	0.7918884
ENSG00000072415	PALS1	14	67755181	G	A	0.102386	0.0387316	0.0251649	0.2	-0.171028	0.0126648	1.48E-41	-0.226464	0.148092	0.1262119	0.006951203
ENSG00000072422	RHOBTB1	10	62695197	T	G	0.475149	0.0145576	0.0142684	0.32	-0.19277	0.00798419	8.63E-129	-0.0755179	0.0740837	0.3080326	0.4332367
ENSG00000072518	MARK2	11	63642445	A	G	0.0894632	-0.0515064	0.0267645	0.06199976	-0.137559	0.0162025	2.07E-17	0.374431	0.199503	0.06054294	0.8717456
ENSG00000072609	CHFR	12	133451354	A	G	0.497018	0.00127907	0.0144166	0.9699999	0.162758	0.00853573	4.68E-81	0.00785874	0.0885781	0.9293036	0.7999294
ENSG00000072682	P4HA2	5	131579269	A	G	0.419483	0.00472256	0.0142896	0.4799997	0.10359	0.00884687	1.14E-31	0.0455891	0.137999	0.7411297	0.979341
ENSG00000072694	FCGR2B	1	161599772	A	C	0.137177	0.0317528	0.0202375	0.25	0.87881	0.0134256	0	0.0361316	0.0230349	0.1167511	0.7453418
ENSG00000072736	NFATC3	16	68190908	G	A	0.150099	-0.0152342	0.0209764	0.29	0.256816	0.010999	1.41E-120	-0.0593196	0.0817183	0.4678981	0.3048866
ENSG00000072756	TRNT1	3	3180581	C	T	0.159046	0.000345346	0.0185154	0.8800001	0.465376	0.00975157	0	0.000742079	0.0397859	0.9851189	0.7093902
ENSG00000072778	ACADVL	17	7124518	A	G	0.416501	-0.00900178	0.0143775	0.7600007	0.321665	0.00795066	0	-0.027985	0.0447025	0.5312973	0.9565955
ENSG00000072786	STK10	5	171542233	T	C	0.0984095	-0.0183452	0.0218909	0.32	-0.254687	0.0120845	1.34E-98	0.0720305	0.0860202	0.4023864	0.7296182
ENSG00000072818	ACAP1	17	7247322	T	C	0.205765	0.00505854	0.01692	0.28	-0.6402	0.0142036	0	-0.0079015	0.0264298	0.7649694	0.4107173
ENSG00000072832	CRMP1	4	5822298	C	A	0.0864811	0.0283055	0.0258051	0.2099999	0.150747	0.0145691	4.32E-25	0.187769	0.172141	0.2753682	0.5935863
ENSG00000072840	EVC	4	5771848	C	A	0.0954274	0.0104892	0.025477	0.6700003	0.128697	0.014606	1.24E-18	0.0815032	0.198177	0.6808791	0.7107341
ENSG00000072858	SIDT1	3	113299784	A	G	0.32505	0.00810212	0.0148946	0.5300002	-0.414787	0.00897835	0	-0.0195332	0.0359115	0.5864925	0.6477731
ENSG00000072864	NDE1	16	15778667	C	T	0.214712	-0.0443615	0.0168834	0.001400006	0.175543	0.00998983	4.02E-69	-0.25271	0.0972473	0.009359613	0.03672577
ENSG00000072952	IRAG1	11	10655086	C	T	0.229622	0.026767	0.0166592	0.04799986	0.52721	0.00886332	0	0.050771	0.0316103	0.1082401	0.2263983
ENSG00000072954	TMEM38A	19	16786389	A	C	0.0238569	-0.0462991	0.0420064	0.2399999	-0.176835	0.0268706	4.67E-11	0.261821	0.240854	0.2770142	0.9226724
ENSG00000072958	AP1M1	19	16327274	T	C	0.215706	-0.00245088	0.0179405	0.9199999	-0.523704	0.00914149	0	0.00467989	0.034257	0.8913381	0.8943531
ENSG00000073008	PVR	19	45158263	T	C	0.0884692	-0.0480208	0.0258381	0.03799969	-0.225392	0.0151128	2.67E-50	0.213055	0.115523	0.06514497	0.4729511
ENSG00000073050	XRCC1	19	44065908	A	G	0.235586	-0.000794313	0.0164847	0.8499999	-0.252369	0.0136821	5.70E-76	0.00314743	0.0653202	0.961569	0.5593607
ENSG00000073060	SCARB1	12	125314308	C	A	0.400596	-0.0456692	0.0144305	0.000949992	0.316468	0.00785742	0	-0.144309	0.0457392	0.001604739	0.7561549
ENSG00000073111	MCM2	3	127329171	A	C	0.0119284	-0.0698842	0.0843484	0.3800004	0.613325	0.072662	3.15E-17	-0.113943	0.138187	0.4096239	0.3453828
ENSG00000073146	MOV10L1	22	50564213	A	G	0.138171	-0.0323837	0.0206791	0.0990011	0.118699	0.0126745	7.59E-21	-0.272822	0.176634	0.1224513	0.5998033
ENSG00000073150	PANX2	22	50613941	G	A	0.256461	-0.0196559	0.0159628	0.17	-0.277521	0.00920525	1.14E-199	0.0708268	0.0575672	0.2185732	0.1211114
ENSG00000073169	SELENOO	22	50647726	G	A	0.377734	-0.0313622	0.0144355	0.016	-0.218876	0.010717	1.04E-92	0.143288	0.066325	0.03074286	0.8159996
ENSG00000073282	TP63	3	189482136	G	A	0.0656064	0.0252719	0.0303229	0.6700003	-0.194758	0.0191578	2.81E-24	-0.129761	0.156218	0.4061773	0.682099
ENSG00000073331	ALPK1	4	113285220	A	G	0.505964	0.0221238	0.0141846	0.1299999	0.278192	0.00781632	1.92E-277	0.0795271	0.0510374	0.1191835	0.5257692
ENSG00000073350	LLGL2	17	73546225	T	G	0.370775	0.0151116	0.0146565	0.3800004	-0.135105	0.00953326	1.37E-45	-0.111851	0.108769	0.3037931	0.2200556
ENSG00000073417	PDE8A	15	85603023	T	G	0.163022	0.0422723	0.0199592	0.09299937	-0.166482	0.0109466	3.10E-52	-0.253915	0.121045	0.03593228	0.3644186
ENSG00000073536	NLE1	17	33462553	T	C	0.110338	-0.0257223	0.0229865	0.2399999	0.452094	0.0126255	8.29E-281	-0.056896	0.0508694	0.2633656	0.9721771
ENSG00000073578	SDHA	5	237585	G	A	0.143141	-0.0324927	0.0224392	0.2	0.177101	0.0125346	2.52E-45	-0.18347	0.127366	0.1497295	0.3424784
ENSG00000073584	SMARCE1	17	38792987	G	A	0.513917	-0.0148965	0.014248	0.1800002	0.666459	0.00705878	0	-0.0223517	0.02138	0.2958145	0.5919253
ENSG00000073598	FNDC8	17	33453174	T	C	0.110338	-0.026421	0.0229759	0.2300001	0.142591	0.0132195	3.99E-27	-0.185292	0.162044	0.2528459	0.9588625
ENSG00000073605	GSDMB	17	38068477	C	G	0.485089	0.00381947	0.0141934	0.83	0.833894	0.00642865	0	0.00458028	0.0170207	0.7878518	0.6920067

ENSG00000073614	KDM5A	12	443957	C	T	0.198807	0.00203249	0.0168636	0.5199996	-0.399384	0.00942717	0	-0.00508907	0.0422242	0.9040674	0.9996016
ENSG00000073670	ADAM11	17	42847806	T	C	0.146123	0.0013751	0.0199631	0.8800001	-0.0874837	0.0108835	9.12E-16	-0.0157184	0.228201	0.9450855	0.3385056
ENSG00000073711	PPP2R3A	3	135775624	T	C	0.222664	-0.031532	0.0159924	0.0530005	0.122249	0.00914342	9.03E-41	-0.257932	0.132233	0.05110574	0.55703
ENSG00000073737	DHRS9	2	169936988	A	C	0.522863	0.0232677	0.0142298	0.08600031	-0.522576	0.00738272	0	-0.044525	0.0272374	0.1021114	0.9400325
ENSG00000073756	PTGS2	1	186645241	A	G	0.027833	-0.018773	0.0466578	0.6300007	-0.774304	0.0264564	2.71E-188	0.024245	0.0602634	0.6874507	0.5263858
ENSG00000073792	IGF2BP2	3	185452185	C	T	0.0129225	-0.0289883	0.0669402	0.7300002	-0.353077	0.0573586	7.48E-10	0.082102	0.19006	0.6657559	0.9648662
ENSG00000073803	MAP3K13	3	185103807	A	G	0.161034	0.0147999	0.0193392	0.5199996	-0.191107	0.0115287	1.03E-61	-0.077443	0.101303	0.4445898	0.601084
ENSG00000073849	ST6GAL1	3	186722307	A	C	0.297217	-0.0156112	0.0157682	0.2200002	0.221668	0.00864708	6.22E-145	-0.070426	0.0711873	0.3225136	0.03240712
ENSG00000073910	FRY	13	32738115	T	C	0.027833	0.0468112	0.0373827	0.2599998	0.416619	0.0210095	1.64E-87	0.11236	0.0899075	0.2113995	0.35512
ENSG00000073921	PICALM	11	85724825	G	C	0.2167	0.0113462	0.0175927	0.29	0.185218	0.00972579	7.37E-81	0.0612587	0.0950383	0.5192069	0.8552348
ENSG00000073969	NSF	17	44751432	C	A	0.230616	0.00837895	0.0162367	0.4299995	-0.233548	0.00934019	5.45E-138	-0.0358768	0.0695368	0.6058962	0.4962518
ENSG00000074054	CLASP1	2	122251257	A	G	0.0298211	-0.0290556	0.0340027	0.3599996	0.386639	0.0214589	1.42E-72	-0.0751492	0.0880432	0.3933548	0.4679576
ENSG00000074071	MRPS34	16	1822523	G	A	0.430417	0.00270673	0.0144475	0.89	0.137722	0.00807742	3.48E-65	0.0196536	0.10491	0.8513956	0.3834948
ENSG00000074201	CLNS1A	11	77287415	C	A	0.0964215	-0.00856218	0.0232809	0.58	0.504813	0.0125358	0	-0.0169611	0.0461198	0.7130509	0.8294742
ENSG00000074219	TEAD2	19	49854783	G	T	0.5	0.000238174	0.014176	0.83	-0.35837	0.00769114	0	-0.000664604	0.0395569	0.9865952	0.9516979
ENSG00000074266	EED	11	85972720	C	G	0.423459	0.00554838	0.0144333	0.83	0.139935	0.00801157	2.57E-68	0.0396497	0.103168	0.7007397	0.5729815
ENSG00000074319	TSG101	11	18519331	C	T	0.362823	0.0217396	0.0150403	0.2099999	0.0735869	0.00830364	7.86E-19	0.295428	0.207089	0.1537032	0.176745
ENSG00000074356	NCBP3	17	3732002	T	A	0.198807	0.0145769	0.0174429	0.29	0.077291	0.0103891	1.01E-13	0.188598	0.227098	0.4062734	0.9691981
ENSG00000074370	ATP2A3	17	3847452	G	C	0.277336	0.0146112	0.0151617	0.1800002	-0.138683	0.0100884	5.32E-43	-0.105357	0.109594	0.3363852	0.9917161
ENSG00000074416	MGLL	3	127474980	G	A	0.279324	-0.0134539	0.0153375	0.3599996	-0.145648	0.00869706	5.98E-63	0.0923728	0.10545	0.381036	0.970399
ENSG00000074582	BCS1L	2	219525826	T	C	0.0258449	0.0515941	0.0399519	0.2099999	0.523256	0.0269203	3.74E-84	0.0986021	0.0765209	0.1975495	0.8728004
ENSG00000074590	NUAK1	12	106495464	T	C	0.0874751	0.0171968	0.0269906	0.6499995	0.322982	0.014788	9.51E-106	0.0532438	0.0836024	0.5242099	0.8845933
ENSG00000074603	DPP8	15	65772421	A	G	0.17992	0.0290171	0.0182983	0.1199999	-0.173696	0.00973655	3.48E-71	-0.167057	0.105762	0.1142093	0.004713907
ENSG00000074621	SLC24A1	15	65928518	T	C	0.0457256	-0.0216026	0.0295218	0.5099998	-0.219822	0.0182216	1.64E-33	0.0982732	0.134546	0.4651406	0.4714264
ENSG00000074657	ZNF532	18	56591772	G	A	0.32505	0.00550858	0.0150552	0.7300002	0.234927	0.0140038	3.66E-63	0.023448	0.0640998	0.7145104	0.442002
ENSG00000074695	LMAN1	18	57011124	G	A	0.292247	-0.00432706	0.0157895	0.7400005	0.446238	0.0107118	0	-0.00969676	0.0353844	0.7840533	0.8621447
ENSG00000074696	HACD3	15	65846721	C	T	0.220676	-0.0261049	0.0165266	0.08600031	0.233629	0.00931277	6.90E-139	-0.111736	0.0708786	0.1149228	0.9375266
ENSG00000074706	IPCEF1	6	154576778	A	G	0.166004	-0.016071	0.018981	0.2200002	-0.167356	0.0105869	2.75E-56	0.0960291	0.11358	0.3978446	0.02173918
ENSG00000074755	ZZEF1	17	3977026	A	G	0.307157	-0.0078878	0.0153539	0.6700003	0.127575	0.00876913	6.00E-48	-0.0618287	0.120427	0.6076627	0.9824245
ENSG00000074800	ENO1	1	8930184	T	G	0.284294	0.0104889	0.0152369	0.35	-0.283462	0.0128105	1.72E-108	-0.0370028	0.0537789	0.491418	0.7508362
ENSG00000074803	SLC12A1	15	48540068	A	C	0.16998	-0.0457956	0.0196601	0.01899984	1.66449	0.00859228	0	-0.0275132	0.0118123	0.01984851	0.03978846
ENSG00000074842	MYDGF	19	4655884	T	C	0.159046	0.0088385	0.019689	0.9599999	0.131739	0.0139555	3.73E-21	0.0670908	0.149623	0.6538654	0.9086297
ENSG00000074855	ANO8	19	17439835	A	G	0.0228628	0.00890367	0.0511517	0.7600007	0.336951	0.0318582	3.83E-26	0.0264242	0.151828	0.8618337	0.7820155
ENSG00000074935	TUBE1	6	112400348	T	C	0.246521	-0.0159104	0.0158202	0.2399999	-0.159312	0.00892392	2.78E-71	0.0998697	0.099461	0.3153258	0.723062
ENSG00000074964	ARHGEF10L	1	17945349	G	A	0.181909	-0.0127963	0.0185782	0.3900004	0.0616491	0.0104165	3.25E-09	-0.207567	0.303388	0.4938724	0.8315534
ENSG00000074966	TXK	4	48102341	A	G	0.107356	-0.043315	0.0232023	0.07299952	0.449095	0.0119279	0	-0.0964495	0.051728	0.06224447	0.4128423
ENSG00000075073	TACR2	10	71170141	A	C	0.44334	-0.0137359	0.0143015	0.3100002	-0.10744	0.00807794	2.30E-40	0.127847	0.133458	0.3380843	0.4368569
ENSG00000075089	ACTR6	12	100614271	A	G	0.0228628	0.0382266	0.0413423	0.2099999	-0.212018	0.0321064	4.01E-11	-0.180299	0.196897	0.3598224	0.6752677
ENSG00000075131	TIPIN	15	66653814	A	G	0.084493	-0.0189279	0.0253571	0.4	-0.359727	0.0141389	8.57E-143	0.0526173	0.0705201	0.4555873	0.5449193
ENSG00000075142	SRI	7	87845370	A	G	0.305169	0.00581771	0.015208	0.6800001	0.171616	0.00854704	1.13E-89	0.0338996	0.0886325	0.7021103	0.3208658
ENSG00000075151	EIF4G3	1	21318170	T	C	0.0974155	0.0341874	0.0256412	0.1199999	-0.219918	0.013995	1.21E-55	-0.155455	0.117013	0.1840044	0.6064223
ENSG00000075188	NUP37	12	102490934	T	A	0.0347913	-0.0215803	0.044884	0.6999999	0.252043	0.0254991	4.86E-23	-0.0856214	0.178291	0.6310611	0.6937236
ENSG00000075213	SEMA3A	7	83854849	A	G	0.0506958	-0.0727676	0.0298095	0.01199997	0.331297	0.0170312	2.78E-84	-0.219644	0.0906838	0.0154315	0.1911235
ENSG00000075223	SEMA3C	7	80461764	T	C	0.111332	-0.00804833	0.0218204	0.83	-0.282137	0.0121702	6.82E-119	0.0285263	0.0773495	0.7122786	0.7028138
ENSG00000075234	TTC38	22	46676881	A	G	0.0248509	-0.0110463	0.0407437	0.7199992	1.03401	0.0228439	0	-0.010683	0.0394045	0.786305	0.308764
ENSG00000075239	ACAT1	11	108005373	G	A	0.415507	-0.0133891	0.0144299	0.4299995	0.377586	0.0116896	6.77E-229	-0.0354597	0.0382319	0.3536725	0.4376861
ENSG00000075240	GRAMD4	22	47023798	G	A	0.380716	-0.0161246	0.0145054	0.1800002	-0.458076	0.00765752	0	0.0352007	0.0316714	0.2663809	0.438682
ENSG00000075275	CELSR1	22	46844899	G	C	0.0934394	-0.0013435	0.0247114	0.7300002	0.442376	0.0181183	1.16E-131	-0.00303701	0.0558608	0.9566424	0.7106894
ENSG00000075292	ZNF638	2	71582945	C	T	0.447316	0.0114739	0.0143423	0.29	0.21758	0.0087644	4.77E-136	0.0527343	0.0659517	0.4239489	0.2978558
ENSG00000075303	SLC25A40	7	87484277	A	G	0.0208748	0.0343208	0.0463537	0.5700002	-0.264467	0.0349279	3.68E-14	-0.129774	0.176108	0.4611853	0.8217463

ENSG00000075336	TIMM21	18	71820971	G	A	0.275348	-0.00951223	0.0154864	0.3599996	0.204703	0.00967663	2.51E-99	-0.0464684	0.0756848	0.5392335	0.08438991
ENSG00000075340	ADD2	2	70915053	T	A	0.39165	-0.00293703	0.0145886	0.9699999	-0.143143	0.00813957	3.15E-69	0.0205182	0.101923	0.8404558	0.4815302
ENSG00000075391	RASAL2	1	178255754	G	T	0.271372	-0.0056788	0.0162716	0.6300007	-0.0711324	0.00918952	9.89E-15	0.0798342	0.228983	0.7273547	0.9849339
ENSG00000075399	VPS9D1	16	89780468	C	G	0.286282	-0.0269496	0.0155741	0.04799986	0.421139	0.00934975	0	-0.0639922	0.0370082	0.08378478	0.1228046
ENSG00000075407	ZNF37A	10	38397770	G	A	0.17495	-0.00417068	0.019223	0.8700001	0.262971	0.0200081	1.86E-39	-0.0158598	0.0731093	0.8282602	0.5066476
ENSG00000075413	MARK3	14	103910948	C	T	0.282306	0.0219158	0.0161131	0.1800002	0.397333	0.00881065	0	0.0551573	0.0405716	0.1739866	0.2270245
ENSG00000075415	SLC25A3	12	98991657	A	G	0.0387674	0.0325901	0.0326732	0.2200002	0.481379	0.016804	1.76E-180	0.0677016	0.0679153	0.3188361	0.5027067
ENSG00000075420	FNDC3B	3	171938436	A	G	0.0407555	-0.0223889	0.0298554	0.4600002	-0.534007	0.0241645	3.25E-108	0.0419263	0.0559405	0.4535681	0.3903107
ENSG00000075426	FOSL2	2	28627747	C	T	0.484095	0.0209748	0.014368	0.05399953	-0.0572362	0.0079863	7.68E-13	-0.36646	0.256185	0.1525869	0.2745753
ENSG00000075568	TMEM131	2	98492593	A	C	0.0805169	0.00634018	0.0283337	0.91	0.137554	0.0175982	5.44E-15	0.0460922	0.206066	0.823009	0.5504883
ENSG00000075618	FSCN1	7	5639362	T	C	0.0954274	0.0568645	0.0269208	0.0329997	-0.359572	0.0143608	2.34E-138	-0.158145	0.0751349	0.03530768	0.8209165
ENSG00000075624	ACTB	7	5585098	C	T	0.307157	0.000895643	0.0149385	0.7600007	-0.103111	0.0186647	3.31E-08	-0.0086862	0.144886	0.952194	0.8908548
ENSG00000075643	MOCOS	18	33809801	A	C	0.110338	-0.0164869	0.0244466	0.4400003	0.346238	0.0219688	5.83E-56	-0.0476173	0.070671	0.5004461	0.8423206
ENSG00000075651	PLD1	3	171423467	C	T	0.185885	0.0173564	0.0194392	0.3800004	0.250389	0.0109691	2.48E-115	0.0693178	0.0776955	0.3723	0.022169
ENSG00000075702	WDR62	19	36570895	T	C	0.121272	-0.00228536	0.0200834	0.83	0.176378	0.0174687	5.71E-24	-0.0129572	0.113873	0.9094072	0.2767254
ENSG00000075711	DLG1	3	196897801	T	C	0.262425	-0.0114719	0.0158421	0.3599996	-0.13233	0.0165462	1.27E-15	0.0866915	0.120206	0.4707927	0.6604291
ENSG00000075785	RAB7A	3	128489302	C	G	0.337972	-0.0244521	0.0147767	0.1	-0.116981	0.00819587	3.21E-46	0.209026	0.127163	0.100225	0.07099683
ENSG00000075790	BCAP29	7	107245018	C	T	0.0248509	0.0165008	0.0511652	0.6300007	0.338329	0.0302441	4.74E-29	0.0487714	0.151292	0.747175	0.7784612
ENSG00000075826	SEC31B	10	102268013	G	A	0.190855	0.0176863	0.0171272	0.2599998	-0.624953	0.00900052	0	-0.0283002	0.0274086	0.301824	0.01560165
ENSG00000075856	SART3	12	108935766	T	A	0.132207	-0.0213611	0.0212224	0.29	0.0835727	0.012521	2.48E-11	-0.255599	0.256811	0.319599	0.7303213
ENSG00000075884	ARHGAP15	2	144187426	C	T	0.119284	-0.0200734	0.0215295	0.5500004	-0.504938	0.0122555	0	0.0397542	0.0426488	0.3512701	0.6439198
ENSG00000075914	EXOSC7	3	45047145	C	T	0.0318091	0.024492	0.038578	0.4500005	0.527914	0.032597	5.45E-59	0.0463939	0.0731324	0.5258313	0.6913444
ENSG00000075945	KIFAP3	1	169972408	T	C	0.0526839	-0.0158192	0.031953	0.6100002	-0.273791	0.0182095	4.29E-51	0.0577783	0.116769	0.620735	0.2136931
ENSG00000075975	MKRN2	3	12611862	G	C	0.0775348	-0.00947443	0.0256166	0.7600007	0.71531	0.0146095	0	-0.0132452	0.0358129	0.7114983	0.7644241
ENSG00000076003	MCM6	2	136615596	C	T	0.417495	-0.0227033	0.0168475	0.002100003	0.518119	0.0086321	0	-0.0438187	0.0325249	0.1779038	0.2482857
ENSG00000076043	REXO2	11	114315554	C	T	0.242545	0.0245075	0.0165446	0.06900014	-0.0958809	0.0092401	3.17E-25	-0.255604	0.174303	0.1425305	0.5632209
ENSG00000076053	RBM7	11	114277838	A	G	0.482107	-0.0028422	0.0142475	0.7300002	-0.0743669	0.0118441	3.41E-10	0.0382186	0.191681	0.84196	0.5064819
ENSG00000076067	RBMS2	12	56950229	G	T	0.475149	-0.000569097	0.0142122	0.7800007	-0.578761	0.00792144	0	0.000983302	0.0245562	0.968059	0.4745709
ENSG00000076108	BAZ2A	12	57009990	A	G	0.26839	0.0122755	0.0158065	0.3599996	0.0896604	0.00950698	4.06E-21	0.136911	0.17689	0.438936	0.9234371
ENSG00000076201	PTPN23	3	47438716	G	A	0.400596	0.0231812	0.014446	0.17	0.0596815	0.00820341	3.46E-13	0.388415	0.24787	0.1171114	0.9190794
ENSG00000076242	MLH1	3	37071101	A	G	0.244533	0.0145043	0.0176563	0.4600002	0.399402	0.0139347	1.12E-180	0.0363151	0.044225	0.4115648	0.0494994
ENSG00000076248	UNG	12	109542088	G	A	0.193837	0.00602406	0.0173563	0.6100002	-0.533526	0.0093422	0	-0.011291	0.0325319	0.7285345	0.7584269
ENSG00000076258	FMO4	1	171297285	A	G	0.475149	0.0185961	0.0142297	0.29	0.1652	0.00802706	4.11E-94	0.112567	0.0863096	0.1921571	0.38507
ENSG00000076321	KLHL20	1	173719960	A	G	0.33499	0.000790286	0.0154011	0.95	-0.155497	0.0086065	5.76E-73	-0.00508232	0.0990447	0.9590758	0.7928175
ENSG00000076351	SLC46A1	17	26727938	C	T	0.0168986	0.0150994	0.0406012	0.7300002	-0.195257	0.0256694	2.81E-14	-0.0773309	0.208186	0.7103011	0.532321
ENSG00000076356	PLXNA2	1	208306626	G	T	0.392644	-0.0201155	0.0146603	0.1299999	0.103889	0.00907326	2.35E-30	-0.193625	0.142125	0.1730843	0.5968387
ENSG00000076513	ANKRD13A	12	110457279	A	G	0.0646123	0.0356756	0.0294467	0.2700001	-0.115896	0.0155465	9.00E-14	-0.307824	0.257412	0.2317582	0.2569924
ENSG00000076554	TPD52	8	81007019	T	C	0.456262	0.00147786	0.0143282	0.9599999	0.434004	0.00762677	0	0.00340518	0.033014	0.9178493	0.04439965
ENSG00000076555	ACACB	12	109630215	G	A	0.192843	-0.00302022	0.0172864	0.7800007	-0.56872	0.00916456	0	0.00531055	0.0303954	0.8613029	0.9633755
ENSG00000076641	PAG1	8	81953414	C	T	0.151093	0.0305514	0.0199897	0.1299999	0.313799	0.0113902	4.45E-167	0.0973599	0.0638003	0.1270072	0.6144346
ENSG00000076650	GPATCH1	19	33596617	A	C	0.196819	-0.00849143	0.0182437	0.7899998	-0.45025	0.0158401	1.00E-177	0.0188594	0.0405245	0.6416578	0.8959137
ENSG00000076662	ICAM3	19	10447475	A	G	0.176938	-0.00750796	0.0191561	0.7400005	0.446716	0.0113474	0	-0.016807	0.0428842	0.6951201	0.1718854
ENSG00000076685	NT5C2	10	104899498	G	A	0.0874751	-0.0059981	0.0265055	0.84	0.560675	0.0131646	0	-0.010698	0.047275	0.8209734	0.000914846
ENSG00000076826	CAMSAP3	19	7671989	A	G	0.0675944	0.0141649	0.0333674	0.7300002	0.329246	0.0230904	3.94E-46	0.0430223	0.10139	0.6713287	0.4126469
ENSG00000076864	RAP1GAP	1	21959254	G	T	0.588469	-0.0182531	0.0143171	0.33	-0.701344	0.00693682	0	0.0260259	0.0204154	0.2023749	0.799857
ENSG00000076924	XAB2	19	7689431	A	G	0.164016	-0.00353808	0.0203835	0.9	-0.336794	0.0119093	6.09E-176	0.0105052	0.0605233	0.8622015	0.610163
ENSG00000076944	STXBP2	19	7707263	A	G	0.336978	-0.00798592	0.0146279	0.3800004	-0.236025	0.00957062	2.78E-134	0.0338351	0.0619913	0.5852008	0.8917308
ENSG00000077044	DGKD	2	234321951	C	G	0.358847	-0.0100572	0.0149313	0.59	0.130428	0.00841451	3.45E-54	-0.0771091	0.114587	0.5009919	0.9330017
ENSG00000077063	CTTNBP2	7	117432449	C	T	0.357853	0.00848528	0.0150341	0.7700005	0.0720246	0.00845121	1.56E-17	0.117811	0.209193	0.5733201	0.5116899
ENSG00000077092	RARB	3	25427623	A	T	0.306163	-0.00618427	0.0154961	0.7199992	0.0649167	0.00885485	2.28E-13	-0.0952647	0.239061	0.6902649	0.2558753

ENSG00000077097	TOP2B	3	25672936	G	A	0.305169	-0.00684206	0.0154955	0.6899999	-0.130127	0.00865829	4.73E-51	0.0525798	0.119131	0.6589517	0.1470655
ENSG00000077147	TM9SF3	10	98312537	G	T	0.16501	-0.0128301	0.0192192	0.3700002	-0.0586121	0.010516	2.50E-08	0.218898	0.330248	0.5074398	0.6743561
ENSG00000077157	PPP1R12B	1	202437762	C	T	0.204771	-0.00342401	0.0168978	0.8600001	-0.0725132	0.0105064	5.13E-12	0.0472191	0.233131	0.8394921	0.6959645
ENSG00000077232	DNAJC10	2	183612666	A	G	0.349901	-0.0197713	0.014703	0.14	-0.188569	0.00825869	2.17E-115	0.104849	0.0781066	0.1794709	0.9918712
ENSG00000077235	GTF3C1	16	27516055	G	C	0.150099	0.0161027	0.0204237	0.4199997	-0.0788081	0.0133302	3.38E-09	-0.204328	0.261452	0.4345008	0.05213878
ENSG00000077238	IL4R	16	27350544	A	G	0.497018	-0.0125886	0.0143028	0.3700002	0.231354	0.00788483	3.05E-189	-0.0544126	0.0618498	0.3789928	0.8672783
ENSG00000077254	USP33	1	78193604	A	G	0.272366	-0.0109416	0.0150826	0.7700005	-0.0916631	0.00861593	1.97E-26	0.119368	0.164926	0.4692103	0.2093827
ENSG00000077312	SNRPA	19	41263918	C	T	0.0725646	0.00389774	0.0273692	0.7700005	-0.284561	0.0150913	2.62E-79	-0.0136974	0.096183	0.8867567	0.915418
ENSG00000077348	EXOSC5	19	41897831	T	C	0.430417	-0.00130363	0.0142725	0.89	-0.0459286	0.00805455	1.18E-08	0.0283838	0.310794	0.9272329	0.7456953
ENSG00000077380	DYNC1I2	2	172574424	C	T	0.27336	-0.00331676	0.0158091	0.7400005	-0.149337	0.014819	6.95E-24	0.0222099	0.105885	0.8338587	0.9912385
ENSG00000077420	APBB1IP	10	26791932	C	T	0.347913	0.0103288	0.0151016	0.7099994	0.310363	0.00816857	0	0.0332797	0.0486657	0.4940742	0.4844729
ENSG00000077454	LRCH4	7	100176815	A	G	0.00497018	0.0650873	0.0884787	0.5500004	-1.09546	0.05206	2.70E-98	-0.0594157	0.0808181	0.4622313	0.9214775
ENSG00000077458	FAM76B	11	95512839	G	A	0.374751	0.00124537	0.0145786	0.91	-0.510015	0.00781538	0	-0.00244183	0.0285847	0.931924	0.3796642
ENSG00000077463	SIRT6	19	4178353	T	C	0.129225	0.0294585	0.0218471	0.1	0.16852	0.0134974	8.97E-36	0.174807	0.130394	0.1800508	0.01467782
ENSG00000077514	POLD3	11	74292529	C	G	0.212724	0.0191824	0.0174737	0.2700001	0.118429	0.00964688	1.21E-34	0.161974	0.148135	0.2742077	0.3205376
ENSG00000077549	CAPZB	1	19738666	A	G	0.054672	-0.00906005	0.02992	0.7199992	-0.192793	0.0176741	1.05E-27	0.0469938	0.155252	0.7621242	0.826806
ENSG00000077585	GPR137B	1	236345498	A	G	0.375746	-0.00806991	0.0146381	0.6800001	0.415312	0.00807826	0	-0.019431	0.0352481	0.5814533	0.5398259
ENSG00000077616	NAALAD2	11	89895372	A	T	0.158052	-0.00193188	0.0194134	0.7300002	0.131154	0.0109461	4.43E-33	-0.0147299	0.148025	0.9207339	0.7792734
ENSG00000077684	JADE1	4	129763579	A	G	0.315109	-0.0271788	0.0151427	0.08100093	-0.168034	0.00841237	9.16E-89	0.161746	0.0904801	0.07383367	0.1799039
ENSG00000077782	FGFR1	8	38297504	G	A	0.382704	0.0137266	0.0144672	0.35	0.273277	0.00796158	3.39E-258	0.0502297	0.05296	0.3429024	0.5221768
ENSG00000077809	NA	7	74123510	T	C	0.444334	-0.0122587	0.0143768	0.4	-0.37751	0.012827	2.21E-190	0.0324725	0.0380992	0.3940394	0.1373365
ENSG00000077935	SMC1B	22	45774722	A	G	0.507952	-0.0125921	0.0141816	0.3599996	-0.15177	0.00817138	5.28E-77	0.0829682	0.093548	0.3751299	0.3484228
ENSG00000077984	CST7	20	24935215	A	G	0.11829	0.024882	0.0214378	0.2099999	-0.581674	0.0124467	0	-0.0427765	0.0368667	0.2459257	0.5326085
ENSG00000078018	MAP2	2	210443812	G	A	0.38668	-0.00548937	0.0145285	0.95	-0.0518967	0.00815627	1.98E-10	0.105775	0.280444	0.7060471	0.7110728
ENSG00000078043	PIAS2	18	44444238	C	T	0.082505	0.029369	0.0264496	0.2200002	-0.67195	0.0172231	0	-0.0437071	0.0393784	0.2670307	0.356629
ENSG00000078053	AMPH	7	38547236	T	C	0.356859	0.0183304	0.0144375	0.2300001	-0.0879758	0.00817027	4.89E-27	-0.208357	0.165245	0.2073437	0.01399604
ENSG00000078070	MCCC1	3	182783434	A	G	0.34493	-0.0186715	0.0151501	0.1199999	-0.251575	0.00835506	3.53E-199	0.0742184	0.0602714	0.2181722	0.4868385
ENSG00000078081	LAMP3	3	182860814	C	T	0.10338	0.0111369	0.0230914	0.6100002	0.178531	0.0128469	6.62E-44	0.0623809	0.129419	0.6298015	0.8418242
ENSG00000078114	NEBL	10	21266009	C	T	0.106362	0.00608065	0.0239154	0.8700001	1.17242	0.0113487	0	0.00518642	0.0203984	0.7992979	0.2979598
ENSG00000078124	ACER3	11	76654876	T	C	0.259443	0.00544714	0.0167287	0.7400005	0.64812	0.00960241	0	0.00840452	0.0258114	0.7447174	0.0403938
ENSG00000078140	UBE2K	4	39742038	G	A	0.496024	-0.00600416	0.0141934	0.6700003	0.180905	0.00788608	1.86E-116	-0.0331895	0.078471	0.6723296	0.1221926
ENSG00000078142	PIK3C3	18	39601482	A	G	0.133201	0.00935333	0.0216192	0.5400003	-0.453681	0.0170438	4.15E-156	-0.0206165	0.0476592	0.6653179	0.9811267
ENSG00000078177	N4BP2	4	40109159	T	C	0.311133	0.0070554	0.0156599	0.4700002	-0.579258	0.00850228	0	-0.0121801	0.027035	0.6523287	0.9051138
ENSG00000078237	TIGAR	12	4446354	G	A	0.262425	0.0043934	0.0165127	0.95	-0.207217	0.00975807	4.50E-100	-0.021202	0.0796944	0.7902076	0.1625925
ENSG00000078246	TULP3	12	3018347	C	G	0.205765	-2.88E-05	0.0175789	0.8800001	-0.0837434	0.00974055	8.15E-18	0.000344335	0.209914	0.9986912	0.4644697
ENSG00000078269	SYNJ2	6	158461548	T	C	0.101392	0.00718699	0.0226207	0.5199996	0.871956	0.0131217	0	0.00824238	0.0259428	0.7507021	0.7764788
ENSG00000078304	PPP2R5C	14	102311230	T	C	0.0745527	0.0141481	0.0259026	0.7600007	0.0914908	0.01452	2.96E-10	0.15464	0.284179	0.5863292	0.7580332
ENSG00000078319	NA	7	99929073	C	A	0.177932	0.0355699	0.0180836	0.05399953	-0.143261	0.0118696	1.53E-33	-0.248287	0.127893	0.05221477	0.4911722
ENSG00000078399	HOXA9	7	27206085	A	G	0.218688	-0.0503476	0.0174561	0.004499974	-0.290852	0.0102932	1.18E-175	0.173104	0.060329	0.004113395	0.589089
ENSG00000078401	EDN1	6	12294011	T	C	0.465209	-0.00051182	0.0143806	0.9599999	-0.117934	0.00795419	9.86E-50	0.0043399	0.121938	0.9716086	0.8551197
ENSG00000078403	MLLT10	10	21927826	T	C	0.0258449	0.0853944	0.0456508	0.04300015	0.231726	0.031673	2.55E-13	0.368515	0.203341	0.06993966	0.5901847
ENSG00000078487	ZCWPW1	7	100012532	A	C	0.192843	0.0330681	0.0179304	0.06699926	-0.264006	0.0100468	3.45E-152	-0.125255	0.0680836	0.065809	0.3645831
ENSG00000078618	NRDC	1	52299670	A	C	0.363817	-0.00406634	0.0145352	0.9599999	-0.397737	0.0116484	1.58E-255	0.0102237	0.036546	0.7796703	0.2068105
ENSG00000078674	PCM1	8	17832913	A	G	0.237575	0.0042894	0.0174462	0.7199992	0.466843	0.00943961	0	0.00918809	0.037371	0.8057895	0.6635103
ENSG00000078687	TNRC6C	17	76052582	G	C	0.0526839	0.00371695	0.0273891	0.8200001	-0.140678	0.0153679	5.49E-20	-0.0264217	0.194715	0.892063	0.3568237
ENSG00000078699	CBFA2T2	20	32157861	T	C	0.415507	0.0155947	0.0143302	0.1299999	-0.0782642	0.00807264	3.17E-22	-0.199257	0.18425	0.2794976	0.04519969
ENSG00000078747	ITCH	20	33025119	A	G	0.493042	-0.0175803	0.0142133	0.35	-0.178163	0.00790366	1.62E-112	0.0986756	0.0798971	0.2168182	0.60429
ENSG00000078795	PKD2L2	5	137251046	C	T	0.49503	-0.00373872	0.014217	0.5199996	0.101064	0.00816021	3.15E-35	-0.0369935	0.140705	0.7926153	0.549606
ENSG00000078804	TP53INP2	20	33296668	T	C	0.129225	0.00358765	0.0183525	0.3900004	-0.191545	0.0106285	1.31E-72	-0.01873	0.0958185	0.8450218	0.8684989
ENSG00000078808	SDF4	1	1159849	A	G	0.0318091	-0.00670935	0.0368544	0.9400001	-0.812883	0.0293375	5.59E-169	0.00825377	0.0453389	0.8555464	0.8750926

ENSG00000078814	MYH7B	20	33576723	C	A	0.197813	-0.00872155	0.0183493	0.4299995	0.131464	0.00986154	1.53E-40	-0.0663417	0.139665	0.6347833	0.3202223
ENSG00000078900	TP73	1	3610924	T	C	0.227634	0.0488523	0.0170202	0.004	-0.0648989	0.011878	4.66E-08	-0.752745	0.296242	0.01105422	0.3201982
ENSG00000078902	TOLLIP	11	1313242	A	G	0.484095	0.0289498	0.0141982	0.02199986	-0.096237	0.00799191	2.14E-33	-0.300818	0.149634	0.044393	0.6133317
ENSG00000078967	UBE2D4	7	43979601	C	T	0.10835	-0.00547786	0.0232513	0.7899998	0.232272	0.0136401	5.04E-65	-0.0235838	0.100113	0.8137655	0.3635782
ENSG00000079134	THOC1	18	241285	A	G	0.212724	0.00826419	0.0168921	0.5400003	0.0807703	0.0104346	9.89E-15	0.102317	0.209555	0.6253659	0.8590742
ENSG00000079150	FKBP7	2	179335859	T	C	0.298211	0.00393354	0.0153009	0.7499995	-0.0516128	0.00863004	2.22E-09	-0.0762125	0.296729	0.7973009	0.791344
ENSG00000079156	OSBPL6	2	179161684	T	A	0.152087	0.00371445	0.0188112	0.84	-0.197191	0.0107136	1.18E-75	-0.0188368	0.0954013	0.8434769	0.6524729
ENSG00000079215	SLC1A3	5	36647446	A	T	0.333002	0.00643709	0.015485	0.8600001	0.189454	0.00857188	3.04E-108	0.033977	0.0817493	0.6776845	0.7588435
ENSG00000079246	XRCC5	2	217021606	A	G	0.510934	-0.00215136	0.0142162	0.8499999	0.156499	0.00792599	8.86E-87	-0.0137468	0.0908418	0.8797177	0.5979174
ENSG00000079257	LXN	3	158377046	T	A	0.384692	-0.0248038	0.0148953	0.06199976	0.477548	0.00778822	0	-0.0519399	0.0312027	0.09599336	0.4438014
ENSG00000079263	SP140	2	231157103	T	C	0.423459	0.00493096	0.0143271	0.4899999	-0.225912	0.00796337	4.89E-177	-0.0218269	0.0634236	0.7307374	0.6615395
ENSG00000079277	MKNK1	1	47052802	G	A	0.295229	-0.00864017	0.0160653	0.7199992	0.378497	0.0094145	0	-0.0228276	0.0424488	0.5907375	0.6742164
ENSG00000079308	TNS1	2	218766115	G	A	0.436382	-0.00849255	0.01475	0.7300002	0.286329	0.00804569	2.16E-277	-0.0296602	0.051521	0.5648242	0.8092987
ENSG00000079332	SAR1A	10	71920119	A	G	0.441352	0.0208603	0.0145114	0.1199999	0.25684	0.00796614	4.64E-228	0.0812192	0.056556	0.1509783	0.1313654
ENSG00000079335	CDC14A	1	100898208	T	A	0.383698	0.0112298	0.0145528	0.5300002	-0.539415	0.0075081	0	-0.0208185	0.0269804	0.4403428	0.06626587
ENSG00000079337	RAPGEF3	12	48146639	C	T	0.0775348	-0.0106932	0.027131	0.56	0.392533	0.0148915	3.98E-153	-0.0272415	0.0691255	0.693516	0.4328205
ENSG00000079387	SENP1	12	48468386	A	C	0.217694	-0.00162181	0.0171805	0.84	-0.151105	0.0110916	2.91E-42	0.010733	0.113702	0.9247946	0.2915963
ENSG00000079393	DUSP13B	10	76861583	C	T	0.44831	0.00193608	0.0144131	0.9400001	0.0470172	0.00806139	5.46E-09	0.0411781	0.306631	0.8931715	0.3040301
ENSG00000079435	LIPE	19	42918618	G	T	0.0705765	0.0539946	0.0318996	0.1	0.173422	0.0194023	3.96E-19	0.311348	0.187212	0.0962956	0.9687768
ENSG00000079459	FDFT1	8	11674950	C	A	0.240557	-0.0148938	0.0171052	0.4799997	-0.848823	0.00949746	0	0.0175464	0.0201526	0.3839309	0.2946626
ENSG00000079462	PAFAH1B3	19	42804441	G	A	0.0864811	0.0172616	0.0254811	0.4799997	0.18573	0.0156386	1.57E-32	0.0929394	0.137418	0.4988323	0.2999796
ENSG00000079616	KIF22	16	29809373	C	T	0.32008	0.0190997	0.0151356	0.1499999	-0.0637289	0.0113591	2.02E-08	-0.299702	0.243433	0.2182675	0.1099751
ENSG00000079691	CARMIL1	6	25450032	C	T	0.2833	0.0039848	0.0158629	0.6800001	0.678327	0.00871588	0	0.00587445	0.0233855	0.8016586	0.1717924
ENSG00000079739	PGM1	1	64092431	G	T	0.112326	0.00162196	0.0227281	0.9299999	-0.424334	0.012318	4.76E-260	-0.00382236	0.0535619	0.9431085	0.185246
ENSG00000079785	DDX1	2	15751268	G	A	0.422465	-0.017805	0.0145339	0.14	-0.170965	0.0120405	9.27E-46	0.104144	0.0853266	0.222263	0.667091
ENSG00000079805	DNM2	19	10886459	A	G	0.475149	0.00413466	0.014192	0.7099994	0.0812344	0.00897955	1.47E-19	0.0508979	0.174795	0.7709088	0.8948212
ENSG00000079819	EPB41L2	6	131272474	T	C	0.218688	-0.0164318	0.0168935	0.2700001	0.238506	0.00924372	8.42E-147	-0.0688946	0.0708807	0.3310607	0.7685463
ENSG00000079931	MOXD1	6	132669939	G	A	0.193837	0.0354056	0.0174798	0.06299992	-0.0965913	0.00972017	2.87E-23	-0.366551	0.184688	0.04717728	0.3377842
ENSG00000079950	STX7	6	132806695	A	C	0.442346	0.00877856	0.0143736	0.4899999	-0.164988	0.00808654	1.58E-92	-0.0532073	0.0871581	0.5415515	0.3788987
ENSG00000079974	RABL2B	22	51214010	A	G	0.0606362	-0.0326526	0.03161	0.3100002	0.593247	0.0272587	5.14E-105	-0.0550405	0.0533431	0.3021557	0.09414034
ENSG00000079999	KEAP1	19	10605606	T	G	0.116302	0.0327737	0.0241978	0.2300001	0.103758	0.0141029	1.88E-13	0.315868	0.237134	0.182852	0.1702976
ENSG00000080007	DDX43	6	74115881	C	G	0.415507	0.0200032	0.0144292	0.1499999	-0.203308	0.00797479	2.31E-143	-0.0983888	0.0710771	0.1662804	0.4849512
ENSG00000080166	DCT	13	95110747	A	G	0.277336	-0.000219785	0.0156449	0.9299999	0.1309	0.00892541	1.06E-48	-0.00167903	0.119518	0.9887914	0.1578878
ENSG00000080189	SLC35C2	20	44985605	A	G	0.267396	0.0117101	0.0163918	0.5300002	0.0786934	0.00899045	2.08E-18	0.148807	0.208992	0.4764523	0.5825871
ENSG00000080200	CRYBG3	3	97629814	G	A	0.284294	-0.00448737	0.0156314	0.7800007	0.18797	0.00960089	2.36E-85	-0.0238728	0.0831679	0.7740792	0.07461098
ENSG00000080298	RFX3	9	3372150	G	A	0.0586481	0.0435545	0.0277033	0.1199999	0.166151	0.0164266	4.75E-24	0.262138	0.168737	0.1202982	0.9755735
ENSG00000080345	RIF1	2	152315462	T	C	0.422465	-0.0219251	0.0145649	0.08999948	-0.145701	0.00806375	5.63E-73	0.15048	0.100311	0.1335779	0.692744
ENSG00000080371	RAB21	12	72166676	A	G	0.15507	0.0171384	0.0200201	0.5099998	0.277853	0.0108386	6.12E-145	0.0616815	0.072093	0.3922286	0.713611
ENSG00000080493	SLC4A4	4	72245403	G	A	0.0318091	0.00195314	0.0431621	0.81	0.184561	0.0219251	3.84E-17	0.0105826	0.233867	0.9639075	0.7326965
ENSG00000080503	SMARCA2	9	2104483	G	A	0.196819	0.0141689	0.0178855	0.5	-0.132425	0.0108684	3.76E-34	-0.106995	0.135346	0.4292176	0.09885143
ENSG00000080546	SESN1	6	109361831	T	G	0.137177	0.0147955	0.0216746	0.58	0.599575	0.0113848	0	0.0246766	0.036153	0.4948837	0.1691236
ENSG00000080573	COL5A3	19	10095692	T	C	0.498012	-0.0224376	0.0142508	0.09400046	-0.393056	0.00765288	0	0.0570851	0.0362735	0.1155474	0.2470202
ENSG00000080608	PUM3	9	2782355	C	G	0.45825	0.0143151	0.0142474	0.2200002	0.18172	0.0118232	2.61E-53	0.0787756	0.0785704	0.3160482	0.04904634
ENSG00000080802	CNOT4	7	135120711	A	G	0.122266	0.0415576	0.0205505	0.05600025	0.0946465	0.0115005	1.88E-16	0.439082	0.223588	0.04955304	0.09573822
ENSG00000080815	PSEN1	14	73646762	C	G	0.156064	-0.0103617	0.0180265	0.6800001	-0.359618	0.010446	1.02E-259	0.028813	0.0501337	0.5654782	0.7542399
ENSG00000080819	CPOX	3	98276271	A	T	0.295229	-0.00824406	0.0151403	0.8499999	-0.172601	0.00932334	1.63E-76	0.0477636	0.0877563	0.5862518	0.1678542
ENSG00000080822	CLDND1	3	98229333	A	G	0.394632	0.0120011	0.0145062	0.5	-0.101107	0.00815566	2.71E-35	-0.118697	0.143792	0.4091035	0.2089144
ENSG00000080823	MOK	14	102731187	T	C	0.0785288	-0.0408941	0.0267241	0.09499921	-0.32825	0.0157437	1.54E-96	0.124582	0.0816327	0.1269779	0.7552462
ENSG00000080824	HSP90AA1	14	102576555	G	A	0.16004	0.00377846	0.0197099	0.8700001	-0.276933	0.0110291	3.95E-139	-0.0136439	0.0711741	0.8479787	0.8385465
ENSG00000080839	RBL1	20	35674575	C	T	0.161034	-0.0169984	0.019588	0.4799997	0.16611	0.0109688	8.33E-52	-0.102332	0.118115	0.3862844	0.9158002

ENSG00000080854	IGSF9B	11	133806032	C	T	0.201789	0.0390935	0.0177967	0.01700004	-0.225698	0.0113712	1.14E-87	-0.173212	0.0793334	0.02901074	0.9746229
ENSG00000080947	CROCCP3	1	16809841	G	A	0.450298	-0.00614702	0.0142242	0.81	0.113388	0.0106861	2.65E-26	-0.0542123	0.125551	0.6658909	0.915229
ENSG00000080986	NDC80	18	2594072	A	G	0.130219	-0.0239633	0.0219933	0.35	0.225905	0.0176026	1.06E-37	-0.106077	0.0977068	0.2776263	0.2682854
ENSG00000081014	AP4E1	15	51249483	A	G	0.239563	-0.0163105	0.0174696	0.4	0.428881	0.00942247	0	-0.0380304	0.0407416	0.3505856	0.2197256
ENSG00000081019	RSBN1	1	114329776	G	A	0.0586481	-0.0145071	0.0306762	0.5400003	-0.244631	0.018891	2.36E-38	0.0593019	0.125481	0.6365018	0.9515805
ENSG00000081026	MAGI3	1	114080958	A	G	0.235586	-0.0125574	0.0171808	0.4	-0.0558787	0.00943483	3.17E-09	0.224726	0.309798	0.4682101	0.3442512
ENSG00000081052	COL4A4	2	227948128	G	C	0.407555	-0.00988723	0.0144961	0.4500005	-0.126555	0.00853626	1.00E-49	0.0781258	0.114665	0.4956559	0.4624761
ENSG00000081059	TCF7	5	133468979	C	T	0.154076	0.0304833	0.0212663	0.2099999	-0.0967355	0.0111828	5.13E-18	-0.31512	0.222837	0.157325	0.08752312
ENSG00000081087	OSTM1	6	108424835	T	C	0.2833	0.01263	0.0157776	0.4100001	0.337155	0.00907366	3.31E-302	0.0374605	0.0468071	0.4235276	0.5035452
ENSG00000081148	IMPG2	3	100992487	T	C	0.385686	-0.0185071	0.0146299	0.25	0.199918	0.0146957	3.80E-42	-0.0925737	0.0734954	0.2078192	0.141687
ENSG00000081154	PCNP	3	101303110	T	G	0.474155	0.020452	0.0142149	0.1499999	0.0980044	0.01184	1.26E-16	0.208684	0.147218	0.1563317	0.219199
ENSG00000081177	EXD2	14	69683651	T	C	0.403579	0.0119799	0.0144307	0.29	-0.114507	0.00800492	2.05E-46	-0.104621	0.126236	0.4072328	0.985129
ENSG00000081189	MEF2C	5	88106948	A	G	0.260437	-0.00216038	0.0163382	0.9400001	-0.28111	0.00903392	1.41E-212	0.00768517	0.0581208	0.8948043	0.2098256
ENSG00000081237	PTPRC	1	198667173	C	A	0.0139165	0.0773729	0.0514482	0.1299999	0.28589	0.0366685	6.36E-15	0.270639	0.183275	0.1397619	0.6889507
ENSG00000081307	UBA5	3	132385115	T	C	0.0149105	0.0268567	0.0610684	0.6899999	-0.336847	0.034963	5.72E-22	-0.0797296	0.181483	0.6604274	0.03673676
ENSG00000081320	STK17B	2	197019758	T	C	0.351889	0.00342856	0.0148369	0.9199999	0.194676	0.00828106	3.33E-122	0.0176116	0.076217	0.8172588	0.706541
ENSG00000081377	CDC14B	9	99317317	G	A	0.164016	0.0110115	0.0181905	0.5700002	-0.0779246	0.0105899	1.86E-13	-0.14131	0.234226	0.5463055	0.9137947
ENSG00000081386	ZNF510	9	99529279	T	C	0.0119284	-0.0276709	0.0796131	0.6700003	0.436603	0.0551726	2.50E-15	-0.0633778	0.182523	0.7284167	0.9373671
ENSG00000081665	ZNF506	19	19914604	C	T	0.457256	-0.0138646	0.0142336	0.35	0.14809	0.0098872	1.02E-50	-0.0936225	0.0963173	0.3310396	0.5132836
ENSG00000081692	JMJD4	1	227920619	A	G	0.247515	0.00025734	0.0167038	0.83	0.299854	0.00926088	5.49E-230	0.000858216	0.0557064	0.9877082	0.6096587
ENSG00000081721	DUSP12	1	161723288	G	T	0.0497018	0.0519772	0.0291914	0.0649995	-0.271587	0.0189937	2.23E-46	-0.191383	0.108315	0.07724184	0.5689133
ENSG00000081760	AACS	12	125588899	A	G	0.171968	-0.00292813	0.0217295	0.91	-0.212128	0.011404	3.14E-77	0.0138036	0.102438	0.8928094	0.8013679
ENSG00000081791	DELE1	5	141312492	C	T	0.307157	-0.0150418	0.0149703	0.3400001	-0.224381	0.00919068	1.21E-131	0.0670368	0.0667746	0.3154142	0.2362695
ENSG00000081870	IFT25	1	54399604	T	C	0.127237	0.0274612	0.0214853	0.25	-0.153321	0.0121788	2.42E-36	-0.179109	0.140853	0.2035145	0.1017514
ENSG00000081913	PHLPP1	18	60515169	A	G	0.416501	0.0175524	0.0144068	0.1800002	-0.275419	0.00793237	3.81E-264	-0.0637298	0.0523409	0.2233791	0.05003351
ENSG00000081923	ATP8B1	18	55391995	T	C	0.321074	0.0109798	0.0148151	0.29	-0.107381	0.0127633	3.99E-17	-0.102251	0.138503	0.4603544	0.7431158
ENSG00000081985	IL12RB2	1	67817815	C	T	0.163022	-0.0123633	0.019103	0.28	0.459752	0.0103857	0	-0.0268913	0.0415551	0.5175527	0.752403
ENSG00000082014	SMARCD3	7	150955416	T	C	0.0188867	0.100725	0.0623531	0.1199999	-0.372317	0.0321551	5.28E-31	-0.270535	0.169095	0.1096204	0.02355084
ENSG00000082068	WDR70	5	37566425	T	G	0.333002	-0.00818896	0.0152081	0.6200004	0.0946228	0.00854012	1.57E-28	-0.0865432	0.160913	0.5906974	0.9972423
ENSG00000082074	FYB1	5	39189984	G	C	0.23161	0.0116593	0.0177665	0.4299995	-0.0874297	0.00964646	1.26E-19	-0.133356	0.203741	0.512765	0.1642773
ENSG00000082146	STRADB	2	202299075	C	T	0.336978	0.0170851	0.0152132	0.29	-0.147155	0.0139101	3.73E-26	-0.116103	0.103963	0.2640929	0.8955049
ENSG00000082153	BZW1	2	201681943	G	A	0.110338	0.00155779	0.0238766	0.83	0.131214	0.0140739	1.13E-20	0.0118721	0.181971	0.9479815	0.9767756
ENSG00000082196	C1QTNF3	5	34055956	T	C	0.357853	0.0134817	0.0149599	0.4400003	-0.128753	0.00841952	8.62E-53	-0.10471	0.116392	0.3683181	0.6957617
ENSG00000082212	ME2	18	48440055	G	A	0.464215	0.0191029	0.0142481	0.1199999	-0.248638	0.00792098	2.79E-216	-0.0768302	0.0573568	0.1804041	0.8608998
ENSG00000082213	C5orf22	5	31543769	A	C	0.168986	-0.00959329	0.019053	0.6100002	-0.298689	0.0107596	1.31E-169	0.032118	0.0637992	0.6146671	0.7390937
ENSG00000082258	CCNT2	2	135696358	A	T	0.329026	-0.0321796	0.0173286	0.004799986	0.325033	0.00863627	58504599999999e-3	-0.0990042	0.0533783	0.06362964	0.6212648
ENSG00000082269	FAM135A	6	71196760	G	A	0.17495	0.0103009	0.018753	0.7099994	-0.231133	0.0102878	8.77E-112	-0.044567	0.0811594	0.5829167	0.1323451
ENSG00000082293	COL19A1	6	70748071	T	C	0.164016	0.00816113	0.0181124	0.5500004	-0.11271	0.0150372	6.61E-14	-0.0724082	0.160989	0.6528759	0.8016154
ENSG00000082397	EPB41L3	18	5511541	A	G	0.266402	0.00346384	0.0162601	0.9699999	-0.350574	0.00881493	0	-0.00988048	0.046382	0.8313083	0.9157945
ENSG00000082438	COBLL1	2	165605161	T	C	0.244533	0.0337614	0.0168153	0.04600023	0.156911	0.00951576	4.36E-61	0.215163	0.107956	0.046255	0.2702703
ENSG00000082512	TRAF5	1	211524122	T	C	0.402584	0.0156516	0.0145485	0.33	0.164961	0.00810731	4.92E-92	0.0948806	0.0883167	0.2826786	0.4462136
ENSG00000082515	MRPL22	5	154334800	G	A	0.114314	0.0152373	0.0226692	0.5199996	-0.247632	0.0129876	4.77E-81	-0.061532	0.0916007	0.5017483	0.179594
ENSG00000082516	GEMIN5	5	154292372	G	A	0.0208748	0.0366798	0.0584339	0.5400003	0.182116	0.0298346	1.03E-09	0.201409	0.322553	0.5323508	0.3751723
ENSG00000082641	NFE2L1	17	46132270	T	C	0.210736	-0.0173401	0.0178167	0.1900002	0.226835	0.0146057	2.15E-54	-0.0764436	0.0786987	0.3313768	0.5813858
ENSG00000082701	GSK3B	3	119676717	G	T	0.22167	-0.0037614	0.0164193	0.9400001	0.300277	0.0088799	1.17E-250	-0.0125264	0.0546817	0.8188077	0.2071191
ENSG00000082781	ITGB5	3	124543734	G	A	0.185885	-0.0145738	0.0176386	0.6300007	0.168488	0.00977625	1.47E-66	-0.0864976	0.104808	0.4092031	0.4180604
ENSG00000082805	ERC1	12	1352382	G	A	0.435388	0.00695062	0.0144819	0.81	-0.112758	0.0080871	3.47E-44	-0.0616417	0.128509	0.6314634	0.5215043
ENSG00000082898	XPO1	2	61735372	T	C	0.192843	-0.0397215	0.0167432	0.0129999	-0.0636818	0.00953664	2.43E-11	0.623749	0.27902	0.02538435	0.7602989
ENSG00000082996	RNF13	3	149605210	A	G	0.26839	-0.0128686	0.0160576	0.4700002	0.414949	0.00898873	0	-0.0310125	0.0387036	0.4229686	0.232684
ENSG00000083093	PALB2	16	23633559	T	G	0.26839	0.0252212	0.0162941	0.1100001	-0.088604	0.00890797	2.61E-23	-0.284651	0.186111	0.1261494	0.09194892

ENSG00000083097	DOP1A	6	83829227	A	G	0.143141	0.0170551	0.0214648	0.4	-0.81405	0.0108111	0	-0.0209509	0.0263694	0.4268945	0.834871
ENSG00000083099	LYRM2	6	90313167	T	C	0.474155	0.0214431	0.0141952	0.1199999	-0.0882682	0.00793465	9.54E-29	-0.242931	0.162295	0.1344321	0.2813497
ENSG00000083123	BCKDHB	6	80936175	A	C	0.517893	0.00166929	0.0142035	0.8	-0.133985	0.00793665	6.12E-64	-0.0124588	0.106011	0.906445	0.7101627
ENSG00000083168	KAT6A	8	41848252	G	A	0.349901	-0.0219941	0.0146655	0.1199999	0.181032	0.00921529	6.40E-86	-0.121493	0.0812461	0.134819	0.5641496
ENSG00000083223	TUT7	9	88936008	C	T	0.368787	-0.0072295	0.0150051	0.8	-0.132788	0.00836005	8.23E-57	0.0544441	0.113053	0.6301034	0.09170448
ENSG00000083290	ULK2	17	19722695	A	G	0.245527	-0.016016	0.0157315	0.33	-0.22555	0.00899862	1.20E-138	0.0710085	0.0698047	0.3090364	0.1249122
ENSG00000083312	TNPO1	5	72162349	C	T	0.294235	0.00924468	0.0156929	0.4700002	-0.10952	0.00877102	8.83E-36	-0.0844107	0.143447	0.5562336	0.3594949
ENSG00000083444	PLOD1	1	12014928	C	T	0.399602	0.00219644	0.0144258	0.8200001	0.633457	0.00732549	0	0.00346738	0.0227732	0.8789838	0.784996
ENSG00000083454	P2RX5	17	3587595	G	A	0.502982	-0.00164019	0.0143396	0.98	-0.0735649	0.00862831	1.51E-17	0.0222958	0.194942	0.9089433	0.9833222
ENSG00000083457	ITGAE	17	3661229	A	G	0.338966	0.0105108	0.0150483	0.5500004	-0.166879	0.00912315	9.62E-75	-0.0629846	0.0902408	0.4852008	0.818362
ENSG00000083520	DIS3	13	73342887	G	A	0.316103	-0.013239	0.0156639	0.29	0.09544	0.00862898	1.95E-28	-0.138715	0.164602	0.3993767	0.9669992
ENSG00000083535	PIBF1	13	73473394	G	A	0.0308151	0.00747668	0.0437899	1	-0.417702	0.0250097	1.28E-62	-0.0178996	0.104841	0.8644354	0.7690094
ENSG00000083544	TDRD3	13	61059301	G	A	0.407555	-0.0101491	0.0143614	0.4	0.0678897	0.00798984	1.95E-17	-0.149494	0.212271	0.48127	0.47449
ENSG00000083635	NUFIP1	13	45538501	T	C	0.0308151	-0.0401666	0.0410088	0.2999998	-0.502041	0.0481094	1.71E-25	0.0800066	0.0820432	0.3294725	0.6642157
ENSG00000083642	PDS5B	13	33256360	T	C	0.206759	-0.00212515	0.0176647	0.9400001	0.103115	0.00970491	2.28E-26	-0.0206096	0.171322	0.9042477	0.5803575
ENSG00000083720	OXCT1	5	41800394	T	C	0.251491	-0.00199756	0.0177385	0.99	0.142345	0.00974619	2.60E-48	-0.0140332	0.12462	0.9103412	0.542824
ENSG00000083799	CYLD	16	50805903	A	G	0.251491	0.0411674	0.0158948	0.0016	-0.156307	0.00914139	1.51E-65	-0.263376	0.10285	0.01044371	0.1122253
ENSG00000083807	SLC27A5	19	59007329	C	G	0.430417	-0.00167766	0.0142436	0.98	-0.0861545	0.00876801	8.70E-23	0.0194727	0.165338	0.906246	0.3628243
ENSG00000083812	ZNF324	19	58981684	C	T	0.0785288	0.0161062	0.0296166	0.6800001	-0.507179	0.0161665	4.85E-216	-0.0317564	0.0584036	0.5866192	0.4301151
ENSG00000083814	ZNF671	19	58235055	A	G	0.360835	0.0275802	0.0152304	0.08400014	-0.185346	0.008438	6.14E-107	-0.148804	0.0824516	0.07111498	0.4104081
ENSG00000083817	ZNF416	19	58086614	G	C	0.269384	-0.0157865	0.015983	0.2399999	-0.0555359	0.00919804	1.56E-09	0.284258	0.291621	0.3296846	0.2890927
ENSG00000083828	ZNF586	19	58306165	A	G	0.236581	-0.0229636	0.0175136	0.1299999	0.525685	0.00921443	0	-0.0436832	0.0333246	0.1899116	0.3814671
ENSG00000083838	ZNF446	19	58988990	G	T	0.220676	0.0232891	0.0182581	0.2200002	-0.150622	0.0101827	1.65E-49	-0.154619	0.121668	0.2037882	0.6266477
ENSG00000083844	ZNF264	19	57713796	C	G	0.0238569	-0.0586218	0.0451315	0.1800002	-0.781732	0.0782279	1.64E-23	0.0749897	0.0582184	0.1977198	0.689794
ENSG00000083845	RPS5	19	58901970	C	T	0.321074	0.00238944	0.0153052	0.8700001	-0.72087	0.0171608	0	-0.00331466	0.0212317	0.8759397	0.6187794
ENSG00000083937	CHMP2B	3	87290559	G	A	0.280318	0.00147066	0.0156299	0.8700001	0.345695	0.00857663	0	0.00425421	0.045213	0.9250357	0.7466098
ENSG00000084070	SMAP2	1	40849760	G	A	0.161034	0.0169408	0.0189836	0.3800004	-0.118748	0.0103832	2.74E-30	-0.142661	0.16035	0.373634	0.675567
ENSG00000084072	PPIE	1	40193720	A	G	0.356859	0.0183808	0.014669	0.1299999	0.83791	0.00677974	0	0.0219365	0.0175075	0.2102157	0.4254396
ENSG00000084073	ZMPSTE24	1	40741817	A	G	0.371769	-0.0312184	0.01434	0.0329997	-0.127856	0.00797824	8.47E-58	0.244168	0.113188	0.03098996	0.7871648
ENSG00000084090	STARD7	2	96862580	G	A	0.294235	-0.0158677	0.0157048	0.2700001	0.0595101	0.0105365	1.62E-08	-0.266639	0.268091	0.3199388	0.7771567
ENSG00000084092	NOA1	4	57837262	C	T	0.297217	0.0391354	0.0154698	0.01400006	-0.275053	0.00948014	4.43E-185	-0.142283	0.0564564	0.01172777	0.205079
ENSG00000084093	REST	4	57788042	A	G	0.33002	-0.0222246	0.015324	0.2300001	0.060457	0.00873933	4.59E-12	-0.36761	0.25898	0.1557667	0.02929139
ENSG00000084110	HAL	12	96378291	G	A	0.254473	0.00559476	0.0168882	0.8	-0.472695	0.00893844	0	-0.0118359	0.0357282	0.7404362	0.7162798
ENSG00000084112	SSH1	12	109213916	C	T	0.260437	-0.00819204	0.0161016	0.58	0.253944	0.00861693	6.92E-191	-0.0322593	0.0634157	0.6109653	0.9568777
ENSG00000084207	GSTP1	11	67352598	A	T	0.0347913	-0.0440833	0.0317454	0.2999998	-0.297939	0.0349816	1.64E-17	0.147961	0.107957	0.170514	0.2814307
ENSG00000084234	APLP2	11	129977215	T	C	0.101392	0.0415693	0.0217984	0.07699987	-0.143477	0.0123453	3.19E-31	-0.289729	0.153962	0.05985991	0.5553214
ENSG00000084444	FAM234B	12	13246336	A	G	0.45825	-0.00332184	0.0143418	0.89	0.16219	0.00872043	3.29E-77	-0.0204812	0.088433	0.8168475	0.5773165
ENSG00000084463	WBP11	12	14947942	T	C	0.452286	0.0100206	0.0142806	0.5400003	-0.169932	0.011789	4.20E-47	-0.0589683	0.0841367	0.4833884	0.4668346
ENSG00000084623	EIF3I	1	32692367	C	T	0.05666	0.0059692	0.0290584	0.89	-0.191968	0.0240561	1.46E-15	-0.0310947	0.151421	0.8372965	0.635076
ENSG00000084652	TXLNA	1	32654586	A	C	0.05666	0.00480086	0.028999	0.9299999	-0.151989	0.0150788	6.80E-24	-0.0315869	0.190823	0.8685266	0.8306521
ENSG00000084676	NCOA1	2	24854177	T	C	0.482107	0.0271329	0.0143006	0.05099998	0.164356	0.00803438	5.25E-93	0.165086	0.0873833	0.05886269	0.1044062
ENSG00000084693	AGBL5	2	27279361	T	C	0.428429	0.0041475	0.0143491	0.83	0.0483042	0.00881174	4.21E-08	0.0858621	0.29747	0.7728557	0.6225938
ENSG00000084710	EFR3B	2	25321621	G	A	0.00695825	-0.00655045	0.0701154	0.89	0.892703	0.094416	3.23E-21	-0.00733777	0.0785466	0.9255705	0.0880963
ENSG00000084731	KIF3C	2	26177544	G	T	0.281312	0.00736034	0.0162764	0.81	0.109898	0.00924805	1.44E-32	0.0669741	0.148211	0.6513538	0.3203559
ENSG00000084733	RAB10	2	26308649	A	G	0.111332	-0.00014885	0.0228799	0.8800001	0.217225	0.0210612	6.09E-25	-0.000685233	0.105328	0.9948092	0.07821862
ENSG00000084754	HADHA	2	26440549	G	A	0.208748	0.0110183	0.017676	0.5099998	-0.284086	0.0104752	5.77E-162	-0.0387851	0.0622371	0.533164	0.3496196
ENSG00000084764	MAPRE3	2	27221772	A	C	0.359841	-0.00339935	0.0148322	0.6700003	0.155559	0.0081825	1.38E-80	-0.0218525	0.0953546	0.8187364	0.9819863
ENSG00000084774	CAD	2	27453534	G	A	0.238569	-0.00985376	0.0165054	0.4899999	-0.186171	0.0136559	2.55E-42	0.0529285	0.0887421	0.5508876	0.1490812
ENSG00000085063	CD59	11	33738899	T	G	0.307157	-0.00759167	0.0151673	0.5199996	0.560349	0.0115823	0	-0.0135481	0.027069	0.6167217	0.2317387
ENSG00000085117	CD82	11	44613945	T	A	0.265408	0.00223311	0.0158996	0.95	-0.127297	0.00876874	9.43E-48	-0.0175425	0.124907	0.8883091	0.06389802

ENSG00000085231	AK6	5	68656325	A	G	0.449304	-0.00435779	0.0142342	0.8800001	-0.292537	0.0145399	4.97E-90	0.0148965	0.0486634	0.759518	0.7415004
ENSG00000085265	FCN1	9	137805620	A	C	0.335984	-0.0167918	0.0150859	0.2099999	0.697798	0.00744991	0	-0.024064	0.0216208	0.2657082	0.1468271
ENSG00000085276	MECOM	3	169091346	G	A	0.0715706	0.024753	0.025037	0.2300001	0.137927	0.0143441	6.87E-22	0.179465	0.182481	0.3253752	0.5749587
ENSG00000085365	SCAMP1	5	77716450	C	T	0.230616	0.00154874	0.0165731	0.89	0.172744	0.00917454	4.40E-79	0.00896553	0.0959415	0.9255478	0.1886086
ENSG00000085377	PREP	6	105788199	T	C	0.0666004	0.00285415	0.0307876	0.95	-0.218337	0.0165758	1.27E-39	-0.0130722	0.141013	0.9261401	0.3299435
ENSG00000085382	HACE1	6	105241881	C	T	0.483101	0.00247409	0.0141992	0.89	-0.0964788	0.00794012	5.68E-34	-0.0256439	0.147189	0.8616897	0.7528809
ENSG00000085415	SEH1L	18	12967333	A	G	0.207753	-0.0101335	0.0174562	0.5099998	-0.158778	0.00925315	5.35E-66	0.0638216	0.110003	0.5617945	0.7123383
ENSG00000085433	WDR47	1	109548843	C	T	0.212724	-0.012117	0.0182865	0.5400003	0.104382	0.0102698	2.87E-24	-0.116083	0.17556	0.5084736	0.3995922
ENSG00000085449	WDFY1	2	224765268	A	G	0.432406	0.010125	0.0142885	0.4899999	-0.240465	0.00788525	2.98E-204	-0.0421059	0.0594362	0.4786841	0.7007233
ENSG00000085465	OVGP1	1	111963667	T	C	0.393638	0.0213513	0.0144603	0.1299999	-0.424911	0.00775684	0	-0.0502489	0.0340437	0.1399412	0.2084305
ENSG00000085491	SLC25A24	1	108710064	C	G	0.549702	-0.027754	0.0142528	0.1299999	0.476475	0.00752299	0	-0.0582486	0.0299272	0.05161309	0.7299244
ENSG00000085511	MAP3K4	6	161475588	G	A	0.345924	0.00321444	0.0148545	0.4600002	-0.0623627	0.00828586	5.22E-14	-0.0515443	0.238294	0.8287495	0.5017616
ENSG00000085514	PILRA	7	99981436	A	G	0.26839	-0.0205208	0.0159514	0.2399999	-0.102388	0.0097546	8.97E-26	0.200422	0.156959	0.2016365	0.6846831
ENSG00000085552	IGSF9	1	159906107	C	T	0.0725646	-0.00326004	0.0313262	0.8200001	0.163296	0.016866	3.60E-22	-0.0199639	0.191848	0.9171205	0.5754838
ENSG00000085563	ABCB1	7	87237893	A	G	0.0596421	-0.00776866	0.0289033	0.64	0.191042	0.0157844	1.02E-33	-0.0406647	0.15133	0.7881491	0.8830864
ENSG00000085644	ZNF213	16	3186292	A	C	0.374751	-0.00356218	0.0144716	0.8700001	0.466058	0.0077541	0	-0.00764322	0.0310513	0.8055679	0.1476936
ENSG00000085662	AKR1B1	7	134135569	T	C	0.392644	-0.00321559	0.0144437	0.8600001	0.28106	0.00836027	8.97E-248	-0.0114409	0.0513911	0.8238279	0.8024811
ENSG00000085719	CPNE3	8	87535392	C	T	0.377734	0.00066028	0.0146481	0.9299999	-0.227589	0.00803789	2.29E-176	-0.0029012	0.0643622	0.9640467	0.2249095
ENSG00000085721	RRN3	16	15171026	A	C	0.408549	-0.0157048	0.014456	0.2700001	0.201672	0.00800183	3.69E-116	-0.0778731	0.0717474	0.2777541	0.3278232
ENSG00000085733	CTTN	11	70263600	C	T	0.257455	0.0163569	0.0165069	0.3599996	0.0996412	0.00948206	7.90E-26	0.164158	0.166398	0.3238699	0.6578697
ENSG00000085741	WNT11	11	75909586	C	T	0.341948	-0.013413	0.0151372	0.2200002	0.177459	0.0091219	2.69E-84	-0.0755835	0.085388	0.3760609	0.05432195
ENSG00000085760	MTIF2	2	55480107	C	T	0.0238569	-0.0540281	0.0499887	0.2599998	-0.290804	0.0278058	1.34E-25	0.185788	0.172814	0.2823386	0.7632419
ENSG00000085788	DDHD2	8	38107906	T	C	0.254473	-0.00327811	0.0165512	0.9	0.220048	0.00952665	4.83E-118	-0.0148972	0.0752189	0.8430048	0.1402503
ENSG00000085832	EPS15	1	51902467	T	A	0.423459	-0.00161843	0.014378	0.98	-0.118423	0.00793995	2.64E-50	0.0136665	0.121416	0.9103796	0.5774394
ENSG00000085871	MGST2	4	140624410	C	T	0.140159	0.00863965	0.0208904	0.8	0.688188	0.016086	0	0.0125542	0.0303571	0.6792029	0.4500435
ENSG00000085872	CHERP	19	16641020	C	T	0.0775348	-0.0284381	0.0280434	0.3800004	-0.121858	0.0154666	3.31E-15	0.23337	0.23203	0.3145224	0.7512315
ENSG00000085978	ATG16L1	2	234161508	A	C	0.486083	-0.00442118	0.0142145	0.8600001	-0.17949	0.00790858	4.95E-114	0.0246319	0.0792013	0.7557977	0.9125233
ENSG00000085982	USP40	2	234429797	C	T	0.150099	0.0215728	0.0194398	0.2300001	-0.844014	0.0193484	0	-0.0255598	0.02304	0.267273	0.8090484
ENSG00000085998	POMGNT1	1	46670165	G	C	0.183897	0.0116048	0.0188428	0.4500005	-0.295969	0.0111104	2.41E-156	-0.0392095	0.0636818	0.538086	0.5765405
ENSG00000085999	RAD54L	1	46728752	A	G	0.285288	0.00114983	0.0154262	0.8800001	-0.0537928	0.00864308	4.85E-10	-0.0213752	0.286791	0.940587	0.5544394
ENSG00000086015	MAST2	1	46377227	C	G	0.2833	0.020866	0.0154736	0.25	-0.146266	0.00912772	8.63E-58	-0.142658	0.106165	0.1790322	0.43916
ENSG00000086062	B4GALT1	9	33135717	T	C	0.442346	-0.0199283	0.0143768	0.17	0.252668	0.00794575	6.66E-222	-0.0788716	0.0569541	0.1661051	0.6682856
ENSG00000086102	NFX1	9	33330832	A	G	0.17992	0.00430715	0.0194932	0.7099994	0.0876962	0.0108387	5.92E-16	0.0491144	0.222364	0.8251904	0.4342156
ENSG00000086189	DIMT1	5	61691423	G	T	0.488072	0.00134882	0.0141824	0.7300002	0.170284	0.00878203	9.37E-84	0.00792102	0.0832879	0.9242322	0.7037569
ENSG00000086200	IPO11	5	61812104	G	A	0.407555	0.00448518	0.0144752	0.9599999	0.12061	0.0080889	2.81E-50	0.0371873	0.120042	0.7567238	0.4588387
ENSG00000086232	EIF2AK1	7	6080371	C	T	0.321074	-0.0185058	0.0147988	0.2	0.21154	0.00831474	8.74E-143	-0.0874812	0.0700418	0.2116704	0.7054346
ENSG00000086288	NME8	7	37914101	C	A	0.50994	0.0158433	0.0142592	0.2200002	-0.361439	0.00853031	0	-0.0438339	0.0394647	0.2666926	0.128459
ENSG00000086289	EPDR1	7	37857494	G	A	0.241551	-0.00221363	0.0167434	0.81	-0.497187	0.00924411	0	0.00445231	0.0336764	0.8948192	0.249183
ENSG00000086300	SNX10	7	26372745	T	A	0.116302	-0.0126711	0.0202464	0.81	0.16229	0.0119724	7.36E-42	-0.0780767	0.124887	0.5318538	0.2263011
ENSG00000086475	SEPHS1	10	13374860	A	G	0.275348	0.000523445	0.0156789	0.8600001	-0.08168	0.0133599	9.73E-10	-0.00640848	0.191958	0.9733677	0.281165
ENSG00000086504	MRPL28	16	418955	T	A	0.0705765	-0.00410708	0.0254431	0.8600001	-0.17677	0.0162451	1.41E-27	0.0232341	0.143949	0.8717749	0.1767637
ENSG00000086506	HBQ1	16	230816	G	A	0.0974155	-0.0093008	0.0240742	0.6899999	0.370364	0.0149086	3.12E-136	-0.0251126	0.0650093	0.6992803	0.07617527
ENSG00000086544	ITPKC	19	41234886	C	G	0.12326	-0.0127615	0.0210931	0.7300002	-0.129378	0.0120914	1.02E-26	0.0986374	0.163295	0.5458148	0.6695832
ENSG00000086548	CEACAM6	19	42265499	T	A	0.40159	-0.00711138	0.0144307	0.8600001	0.0711839	0.00808651	1.33E-18	-0.0999015	0.203042	0.6227018	0.3575672
ENSG00000086570	FAT2	5	150916079	G	A	0.379722	-0.015154	0.0146602	0.2200002	-0.1269	0.00830996	1.20E-52	0.119417	0.11579	0.3023894	0.04123254
ENSG00000086589	RBM22	5	150075512	G	A	0.115308	-0.00211668	0.0263179	0.81	-0.133608	0.0136762	1.52E-22	0.0158425	0.196985	0.9358995	0.5247503
ENSG00000086598	TMED2	12	124076097	T	C	0.248509	-0.00316519	0.0160808	0.83	0.172891	0.00930114	4.00E-77	-0.0183074	0.0930162	0.8439689	0.4976531
ENSG00000086619	ERO1B	1	236412087	T	C	0.400596	0.0175392	0.0144586	0.1499999	0.210883	0.00797784	5.63E-154	0.0831703	0.0686343	0.2255935	0.05364724
ENSG00000086666	ZFAND6	15	80391322	G	A	0.318091	0.00396712	0.0149877	0.64	0.0704354	0.0128036	3.77E-08	0.0563229	0.213033	0.7914826	0.2106051
ENSG00000086730	LAT2	7	73629071	G	A	0.0666004	0.0227879	0.0270573	0.4600002	-0.314692	0.0146427	1.87E-102	-0.0724132	0.0860461	0.4000326	0.393277

ENSG00000086848	ALG9	11	111697612	T	C	0.323062	0.0205777	0.0156426	0.2099999	-0.0766355	0.0133251	8.86E-09	-0.268514	0.209388	0.199712	0.4169515
ENSG00000087008	ACOX3	4	8405229	A	T	0.0805169	0.0662028	0.0282169	0.016	0.443598	0.0242456	8.91E-75	0.14924	0.06413	0.01995704	0.7847379
ENSG00000087053	MTMR2	11	95612262	A	C	0.424453	-0.0032667	0.014381	0.7499995	0.093994	0.0082893	8.39E-30	-0.0347543	0.15303	0.8203395	0.4393217
ENSG00000087074	PPP1R15A	19	49377481	T	C	0.110338	-0.0169024	0.0235596	0.3100002	-0.36504	0.0126701	1.55E-182	0.0463029	0.0645598	0.473245	0.8224382
ENSG00000087076	HSD17B14	19	49328104	C	T	0.267396	0.00311825	0.0165816	0.7099994	-0.392812	0.0092171	0	-0.00793828	0.042213	0.8508351	0.8222543
ENSG00000087077	TRIP6	7	100467918	A	G	0.182903	-0.00102616	0.0183431	0.9299999	-0.370859	0.0168625	3.35E-107	0.00276698	0.0494612	0.9553877	0.9898875
ENSG00000087086	FTL	19	49469346	A	G	0.383698	-0.00282677	0.0147293	0.8	-0.106357	0.0140401	3.59E-14	0.0265782	0.138534	0.8478572	0.4610002
ENSG00000087087	SRRT	7	100479509	C	A	0.0109344	-0.0869289	0.0852368	0.29	0.334731	0.0496663	1.59E-11	-0.259698	0.257542	0.3132759	0.542754
ENSG00000087088	BAX	19	49461563	A	G	0.383698	-0.00282677	0.0147293	0.8	-0.247683	0.00824452	2.77E-198	0.0114129	0.0594696	0.847812	0.1335883
ENSG00000087111	PIGS	17	26889645	T	A	0.178926	0.0249737	0.0189392	0.1800002	-0.115653	0.0108885	2.36E-26	-0.215936	0.165016	0.1906769	0.213898
ENSG00000087116	ADAMTS2	5	178655141	A	G	0.405567	0.0132057	0.0144832	0.29	-0.162192	0.00960453	5.61E-64	-0.0814203	0.0894269	0.3625757	0.9915916
ENSG00000087152	ATXN7L3	17	42273327	G	C	0.0228628	0.0287064	0.0428033	0.4400003	-0.212001	0.032136	4.20E-11	-0.135407	0.202942	0.5046319	0.7669972
ENSG00000087157	PGS1	17	76397958	A	G	0.170974	0.00928038	0.0183769	0.4500005	0.172205	0.0122369	5.60E-45	0.0538914	0.106784	0.6137853	0.8810368
ENSG00000087191	PSMC5	17	61906945	T	A	0.327038	-0.0101213	0.0150979	0.5300002	0.173774	0.0124379	2.33E-44	-0.0582439	0.0869821	0.503108	0.01369809
ENSG00000087206	UIMC1	5	176390820	C	T	0.489066	0.000939901	0.0142162	0.91	0.33326	0.00842658	0	0.00282033	0.0426581	0.9472865	0.9776856
ENSG00000087237	CETP	16	57006759	A	C	0.426441	-0.0239747	0.0143303	0.1100001	-0.130051	0.00805159	1.10E-58	0.184349	0.110779	0.09609148	0.1494456
ENSG00000087250	MT3	16	56623993	A	C	0.280318	-0.00357954	0.0155186	0.8800001	-0.149759	0.00881295	9.25E-65	0.0239021	0.103634	0.8175945	0.6900932
ENSG00000087253	LPCAT2	16	55581746	T	C	0.493042	0.0175427	0.0142897	0.2399999	0.661703	0.00703948	0	0.0265114	0.0215972	0.219619	0.5886275
ENSG00000087258	GNAO1	16	56308329	T	G	0.0457256	-0.0454354	0.0322351	0.1900002	-0.369674	0.0189468	8.82E-85	0.122907	0.0874259	0.1597725	0.3768704
ENSG00000087263	OGFOD1	16	56499207	T	C	0.178926	0.00532991	0.0187147	0.83	-0.29453	0.0100571	1.58E-188	-0.0180963	0.0635438	0.7758092	0.5460134
ENSG00000087266	SH3BP2	4	2818787	T	C	0.0606362	-0.0561728	0.0280898	0.03699985	-0.22603	0.0173596	9.37E-39	0.24852	0.125732	0.04808899	0.7500011
ENSG00000087269	NOP14	4	2952386	T	C	0.40159	-0.000544887	0.0146514	0.7800007	-0.0997268	0.00816416	2.58E-34	0.0054638	0.146916	0.9703336	0.3517358
ENSG00000087274	ADD1	4	2888693	C	T	0.302187	-0.000648254	0.015763	0.7800007	0.154157	0.00876051	2.61E-69	-0.00420516	0.102253	0.9671963	0.8295333
ENSG00000087299	L2HGDH	14	50741773	T	C	0.0198807	0.0134992	0.047488	0.6499995	0.200516	0.0355286	1.66E-08	0.0673223	0.237129	0.7764827	0.3968186
ENSG00000087301	TXNDC16	14	52958274	G	A	0.0626243	0.0070259	0.0268169	0.8	-0.282462	0.0183675	2.29E-53	-0.0248738	0.0949536	0.7933544	0.1586003
ENSG00000087302	RTRAF	14	52463806	A	G	0.0188867	-0.0603133	0.0497109	0.29	-0.25758	0.0326613	3.11E-15	0.234154	0.195263	0.2304607	0.32741
ENSG00000087303	NID2	14	52503616	C	A	0.148111	0.0129079	0.0207649	0.4	-0.112441	0.0119366	4.52E-21	-0.114797	0.185076	0.5350787	0.2432145
ENSG00000087338	GMCL1	2	70082651	G	A	0.258449	0.0081121	0.0162167	0.59	0.191289	0.00916848	1.14E-96	0.0424075	0.0848001	0.6170134	0.7709303
ENSG00000087365	SF3B2	11	65827489	T	C	0.27336	0.0102128	0.0157047	0.4500005	-0.296706	0.00880853	9.95E-249	-0.0344207	0.0529401	0.5155753	0.1057332
ENSG00000087448	KLHL42	12	27944463	T	C	0.208748	-0.0161657	0.017832	0.3800004	-0.330678	0.0107386	3.21E-208	0.0488865	0.0539489	0.3648496	0.1731659
ENSG00000087460	GNAS	20	57450510	T	C	0.474155	0.00979797	0.0142056	0.5700002	-0.221299	0.00797812	2.41E-169	-0.0442747	0.0642116	0.4905006	0.5870163
ENSG00000087470	DNM1L	12	32865310	T	C	0.136183	-0.00301593	0.0230336	0.8	-0.229524	0.0127091	6.61E-73	0.0131399	0.100356	0.8958286	0.9945043
ENSG00000087502	ERGIC2	12	29512203	A	T	0.16501	0.0141947	0.0202428	0.4899999	-0.253778	0.0116239	1.14E-105	-0.0559336	0.079807	0.4833899	0.8422025
ENSG00000087586	AURKA	20	54955919	A	G	0.0228628	0.0714923	0.0469664	0.07699987	-0.192558	0.0299208	1.23E-10	-0.371277	0.250638	0.1385192	0.9589271
ENSG00000087589	CASS4	20	55010782	A	T	0.0884692	0.0156062	0.0250512	0.4799997	-0.148792	0.0153021	2.39E-22	-0.104886	0.168709	0.5341408	0.9883411
ENSG00000087884	AAMDC	11	77580816	A	C	0.0994036	-0.0105327	0.0230704	0.5400003	-0.603949	0.032201	1.74E-78	0.0174397	0.0382106	0.6480946	0.8583433
ENSG00000087903	RFX2	19	6096379	A	G	0.100398	-0.00464584	0.0227423	0.6800001	0.188533	0.0129806	8.51E-48	-0.0246421	0.12064	0.838149	0.4906579
ENSG00000088002	SULT2B1	19	49079007	G	A	0.496024	-0.00462177	0.015799	0.9	0.201497	0.0103554	2.48E-84	-0.0229372	0.078417	0.7699024	0.3564917
ENSG00000088035	ALG6	1	63868747	G	A	0.0725646	0.000946599	0.0252881	0.8700001	-0.258233	0.013677	1.64E-79	-0.00366567	0.0979275	0.9701401	0.1903728
ENSG00000088038	CNOT3	19	54650431	G	A	0.359841	0.0365331	0.0146957	0.01199997	-0.115447	0.00940429	1.22E-34	-0.316449	0.129878	0.01482957	0.1136407
ENSG00000088053	GP6	19	55537352	A	G	0.248509	-0.00261206	0.0163153	0.9199999	0.339163	0.0177511	2.22E-81	-0.00770148	0.0481063	0.8728077	0.7076854
ENSG00000088179	PTPN4	2	120629300	T	C	0.424453	-0.000621815	0.0143768	0.9299999	-0.105405	0.00801386	1.64E-39	0.00589931	0.136397	0.9655014	0.2542766
ENSG00000088205	DDX18	2	118581090	G	A	0.139165	-0.0160033	0.0187132	0.2599998	-0.261372	0.0158954	9.38E-61	0.0612281	0.0716929	0.3930864	0.9089609
ENSG00000088247	KHSRP	19	6419082	T	C	0.10835	-0.023003	0.0254103	0.3800004	-0.10836	0.0151956	9.96E-13	0.212283	0.236381	0.369156	0.9261622
ENSG00000088256	GNA11	19	3109205	A	G	0.309145	-0.00160782	0.0159276	0.8800001	0.29048	0.0105286	1.48E-167	-0.00553504	0.0548323	0.9195942	0.8088354
ENSG00000088280	ASAP3	1	23782870	A	G	0.166004	0.0170154	0.0188678	0.4400003	-0.189021	0.0100127	1.72E-79	-0.0900185	0.0999322	0.3676974	0.5200271
ENSG00000088298	EDEM2	20	33784544	T	C	0.188867	0.00161622	0.0192058	0.8800001	-0.397893	0.0100386	0	-0.00406195	0.0482689	0.9329352	0.4751295
ENSG00000088305	DNMT3B	20	31373676	C	T	0.419483	0.00259117	0.0143031	0.6899999	-0.0725979	0.00797744	9.00E-20	-0.0356921	0.197057	0.856269	0.1149919
ENSG00000088340	FER1L4	20	34170995	T	C	0.101392	0.0380428	0.0235234	0.1199999	-0.172052	0.0146802	1.01E-31	-0.221112	0.138018	0.1091442	0.3705315
ENSG00000088356	PDRG1	20	30536326	C	T	0.420477	-0.00283301	0.0143981	0.7600007	0.0908029	0.00805504	1.79E-29	-0.0311996	0.158589	0.8440366	0.02036033

ENSG00000088387	DOCK9	13	99592310	A	G	0.247515	-0.011944	0.0173811	0.3100002	0.169849	0.00931169	2.47E-74	-0.0703213	0.102405	0.4922743	0.4801019
ENSG00000088448	ANKRD10	13	111549151	A	G	0.368787	0.0177583	0.0147045	0.1900002	0.393213	0.00807147	0	0.045162	0.0374072	0.2273139	0.7420187
ENSG00000088451	TGDS	13	95237409	G	A	0.350895	-0.00443735	0.0146557	0.8800001	-0.136466	0.00868483	1.23E-55	0.032516	0.107414	0.7621057	0.6222359
ENSG00000088538	DOCK3	3	51067150	C	T	0.117296	0.0221756	0.0211995	0.2200002	0.138932	0.0125239	1.35E-28	0.159614	0.153265	0.2976788	0.6406004
ENSG00000088543	C3orf18	3	50601960	G	A	0.115308	0.0182831	0.021052	0.2599998	0.115757	0.0118045	1.06E-22	0.157943	0.182575	0.3869909	0.9465038
ENSG00000088682	COQ9	16	57488262	A	C	0.0775348	-0.026897	0.0276016	0.4	-0.250599	0.0158156	1.52E-56	0.107331	0.110351	0.3307348	0.6535964
ENSG00000088726	TMEM40	3	12792990	G	C	0.323062	-0.00807989	0.0147387	0.4799997	-0.049503	0.00824584	1.93E-09	0.16322	0.298972	0.5851088	0.2664142
ENSG00000088766	CRLS1	20	6003717	C	T	0.341948	-0.0143135	0.0146381	0.3599996	0.400395	0.00791747	0	-0.0357484	0.036566	0.3282513	0.7038677
ENSG00000088808	PPP1R13B	14	104257008	T	C	0.0606362	-0.0219091	0.0320605	0.5	0.284628	0.0237368	3.96E-33	-0.0769744	0.112823	0.4950743	0.4521223
ENSG00000088812	ATRN	20	3541728	A	G	0.45328	-0.00180483	0.0143405	0.7700005	0.067303	0.00796862	3.01E-17	-0.0268165	0.213097	0.8998574	0.6283335
ENSG00000088826	SMOX	20	4135010	G	C	0.34493	0.0153358	0.0146239	0.2700001	-0.427078	0.00797252	0	-0.0359087	0.0342483	0.2944177	0.2671894
ENSG00000088832	FKBP1A	20	1361714	C	T	0.415507	-0.00582363	0.0145735	0.7600007	-0.351794	0.00896687	0	0.0165541	0.0414284	0.6894634	0.6951241
ENSG00000088833	NSFL1C	20	1435612	G	T	0.237575	0.000539046	0.0172586	0.7400005	-0.75077	0.00838066	0	-0.000717991	0.0229879	0.9750833	0.93752
ENSG00000088836	SLC4A11	20	3213975	G	A	0.529821	-0.00730645	0.0141801	0.64	0.0924878	0.00793873	2.29E-31	-0.0789991	0.153468	0.6067224	0.4731738
ENSG00000088854	DNAAF9	20	3309111	C	T	0.0984095	-0.0266131	0.026306	0.2700001	0.167747	0.0156914	1.13E-26	-0.15865	0.15752	0.313851	0.1568047
ENSG00000088876	ZNF343	20	2483905	A	G	0.420477	-0.0269337	0.014338	0.07299952	0.142507	0.00807945	1.25E-69	-0.188999	0.101182	0.06177319	0.7161994
ENSG00000088881	EBF4	20	2707117	T	C	0.101392	-0.0433716	0.0250103	0.04300015	-0.367093	0.0143004	2.52E-145	0.118149	0.0682859	0.08359348	0.3615027
ENSG00000088882	CPXM1	20	2777999	A	G	0.083499	0.00236458	0.0233864	0.7899998	-0.115248	0.0129074	4.31E-19	-0.0205174	0.202936	0.9194689	0.9652357
ENSG00000088888	MAVS	20	3838383	A	G	0.156064	0.00569451	0.0196353	0.84	0.350296	0.0110113	4.38E-222	0.0162563	0.0560557	0.7718148	0.5559762
ENSG00000088899	LZTS3	20	3148727	T	C	0.307157	-0.0100866	0.0160033	0.5199996	0.109133	0.00975426	4.66E-29	-0.0924251	0.146873	0.5291627	0.8167941
ENSG00000088930	XRN2	20	21327202	C	G	0.184891	-0.00136191	0.017924	0.7400005	0.704916	0.0104458	0	-0.00193202	0.0254272	0.9394331	0.1654007
ENSG00000088970	KIZ	20	21166942	A	C	0.375746	0.00855379	0.0146687	0.64	0.356326	0.00880374	0	0.0240055	0.0411708	0.5598451	0.1241788
ENSG00000088986	DYNLL1	12	120921974	A	G	0.350895	0.0193983	0.0150335	0.1900002	0.0633587	0.00891071	1.16E-12	0.306166	0.241151	0.2042263	0.9921313
ENSG00000088992	TESC	12	117507006	A	G	0.156064	-0.0154485	0.021303	0.28	0.424002	0.0112909	1.282507e-308	-0.0364349	0.050252	0.4684248	0.2317472
ENSG00000089006	SNX5	20	17935932	A	C	0.136183	0.0113074	0.0213342	0.33	-0.229657	0.012615	4.71E-74	-0.0492361	0.0929353	0.5962587	0.5013182
ENSG00000089012	SIRPG	20	1624111	G	A	0.249503	-0.0359046	0.0172087	0.007199959	-0.366343	0.00889132	0	0.0980082	0.0470345	0.03718278	0.3765404
ENSG00000089022	MAPKAPK5	12	112307062	T	C	0.0815109	-0.0020567	0.0260054	0.8700001	0.368808	0.0146049	1.07E-140	-0.00557662	0.0705124	0.9369634	0.3158992
ENSG00000089041	P2RX7	12	121597249	T	G	0.0367793	-0.0791367	0.0359962	0.03500016	-0.607794	0.0171572	7.07E-275	0.130203	0.0593383	0.02821728	0.7718172
ENSG00000089048	ESF1	20	13730250	G	A	0.332008	-0.00863485	0.0146772	0.6800001	0.0580464	0.00822652	1.71E-12	-0.148758	0.25373	0.5576854	0.6089127
ENSG00000089050	RBBP9	20	18472535	C	T	0.125249	0.0190311	0.0217425	0.59	0.206192	0.011611	1.48E-70	0.0922978	0.105576	0.381991	0.562506
ENSG00000089053	ANAPC5	12	121791873	C	A	0.351889	-0.0138202	0.0151876	0.1900002	-0.212533	0.00868722	3.48E-132	0.0650262	0.0715094	0.3631721	0.3880743
ENSG00000089057	SLC23A2	20	4911970	A	G	0.374751	-0.0257904	0.014632	0.08700015	-0.109399	0.00816433	6.08E-41	0.235747	0.134901	0.08054227	0.4504186
ENSG00000089060	SLC8B1	12	113766931	A	G	0.147117	-0.000993305	0.0208768	0.9199999	0.369499	0.0134475	3.29E-166	-0.00268825	0.0565004	0.9620515	0.1886305
ENSG00000089063	TMEM230	20	5087117	T	C	0.451292	-0.00386648	0.0142306	0.8200001	-0.633551	0.00854098	0	0.00610287	0.0224618	0.7858527	0.02440282
ENSG00000089091	DZANK1	20	18405920	T	C	0.427435	-0.0162416	0.0142976	0.3900004	0.0513166	0.00894672	9.71E-09	-0.316498	0.284027	0.2651405	0.7646153
ENSG00000089094	KDM2B	12	121943116	T	C	0.129225	-0.0106536	0.0199175	0.64	-0.0633491	0.0113655	2.49E-08	0.168173	0.315853	0.594421	0.2923397
ENSG00000089123	TASP1	20	13433148	C	T	0.0874751	0.0261928	0.0253527	0.3100002	-0.084168	0.0154295	4.90E-08	-0.311197	0.30657	0.3100622	0.9253696
ENSG00000089127	OAS1	12	113357286	C	T	0.345924	-0.00121579	0.0148211	0.7700005	0.666119	0.00763083	0	-0.00182518	0.0222499	0.9346221	0.6883499
ENSG00000089154	GCN1	12	120598760	G	A	0.424453	0.00831486	0.014514	0.6300007	-0.382704	0.00865371	0	-0.0217266	0.037928	0.5667548	0.9563649
ENSG00000089157	RPLP0	12	120636763	A	C	0.153082	0.0200244	0.0205078	0.5	0.0943275	0.0113944	1.25E-16	0.212286	0.218918	0.3321928	0.9073383
ENSG00000089159	PXN	12	120675912	C	A	0.156064	0.0182549	0.0204006	0.4899999	-0.125815	0.0161924	7.85E-15	-0.145093	0.16322	0.374032	0.8948506
ENSG00000089163	SIRT4	12	120745585	G	A	0.0536779	-0.0241009	0.0306239	0.4500005	0.514626	0.0178113	1.45E-183	-0.0468319	0.0595292	0.4314548	0.6893194
ENSG00000089169	RPH3A	12	113172435	A	G	0.422465	-0.0226897	0.0145028	0.14	0.769995	0.00688717	0	-0.0294673	0.0188368	0.117735	0.6566735
ENSG00000089177	KIF16B	20	16403413	A	C	0.12326	-0.00289311	0.0211114	0.6700003	-0.905806	0.00997682	0	0.00319396	0.0233068	0.8909992	0.7802642
ENSG00000089195	TRMT6	20	5924531	A	G	0.251491	0.0230512	0.0164079	0.2200002	0.148533	0.0093675	1.27E-56	0.155192	0.110899	0.1616924	0.07888389
ENSG00000089199	CHGB	20	5899041	A	G	0.251491	0.0230512	0.0164079	0.2200002	0.376072	0.010003	2.517563e-309	0.0612947	0.0436602	0.1603473	0.09857701
ENSG00000089220	PEBP1	12	118578526	A	G	0.5	-0.00256373	0.0141841	0.56	0.417017	0.0113218	5.43E-297	-0.00614779	0.0340137	0.8565681	0.8923582
ENSG00000089248	ERP29	12	112456187	G	A	0.0974155	0.0200635	0.0239991	0.4500005	-0.180855	0.01271	6.02E-46	-0.110937	0.132927	0.4039591	0.09946084
ENSG00000089327	FXYD5	19	35653209	G	A	0.462227	-0.00986914	0.0144368	0.6200004	-0.435462	0.00795724	0	0.0226636	0.0331554	0.4942552	0.9571454
ENSG00000089335	ZNF302	19	35172923	G	T	0.114314	-0.00918492	0.0231363	0.6200004	-0.563493	0.0122914	0	0.0163	0.0410603	0.6913845	0.121088

ENSG00000089351	GRAMD1A	19	35501531	A	G	0.341948	0.00554922	0.0154394	0.7600007	-0.352669	0.00882432	0	-0.0157349	0.0437805	0.7192922	0.2788503
ENSG00000089486	CDIP1	16	4574752	G	A	0.277336	0.0262691	0.016164	0.07799917	0.110537	0.0109345	5.04E-24	0.23765	0.14811	0.1085911	0.4161165
ENSG00000089505	CMTM1	16	66606667	A	G	0.0606362	0.022005	0.0371904	0.6100002	0.142553	0.0203577	2.52E-12	0.154364	0.261818	0.5554696	0.781066
ENSG00000089597	GANAB	11	62403201	T	G	0.161034	-0.00593634	0.0206011	0.6100002	-0.28793	0.0178885	2.73E-58	0.0206173	0.0715605	0.7732623	0.8041343
ENSG00000089639	GMIP	19	19747380	A	G	0.242545	0.0227175	0.0165231	0.1499999	-0.197124	0.00934371	8.47E-99	-0.115245	0.0839986	0.1700688	0.3481045
ENSG00000089685	BIRC5	17	76215992	A	C	0.274354	-0.00369967	0.0157516	0.9199999	-0.197155	0.00953241	4.97E-95	0.0187653	0.0798997	0.8143169	0.152147
ENSG00000089692	LAG3	12	6884649	A	G	0.173956	-0.0186699	0.0185401	0.2399999	-0.0852575	0.0105436	6.16E-16	0.218983	0.21914	0.317658	0.2291553
ENSG00000089693	MLF2	12	6866905	T	G	0.0357853	-0.0519716	0.0380799	0.1299999	-0.289984	0.0200495	2.06E-47	0.179222	0.131901	0.1742201	0.429197
ENSG00000089723	OTUB2	14	94503975	T	A	0.321074	0.0232072	0.0150426	0.07299952	0.0655951	0.00851067	1.28E-14	0.353795	0.233874	0.1303413	0.1217463
ENSG00000089737	DDX24	14	94532428	T	C	0.409543	0.0127878	0.0143641	0.28	-0.341953	0.00784295	0	-0.0373963	0.0420148	0.3734253	0.2936101
ENSG00000089775	ZBTB25	14	64943877	A	C	0.316103	-0.00329747	0.0152444	0.83	-0.475931	0.00826843	0	0.00692846	0.0320309	0.8287497	0.9404164
ENSG00000089818	NECAP1	12	8242587	A	G	0.309145	-0.00673578	0.0153976	0.8	-0.454928	0.00806658	0	0.0148062	0.0338472	0.6617899	0.9431924
ENSG00000089847	ANKRD24	19	4204081	C	T	0.129225	0.0255296	0.0217338	0.14	0.343306	0.0149976	5.74E-116	0.0743639	0.0633906	0.2407533	0.01907856
ENSG00000089876	DHX32	10	127554955	A	G	0.402584	-0.00681745	0.0143113	0.6899999	-0.671404	0.0070414	0	0.010154	0.0213157	0.6338162	0.3280385
ENSG00000089902	RCOR1	14	103127955	G	A	0.0934394	0.0296978	0.0216551	0.08400014	0.400517	0.019341	2.92E-95	0.0741486	0.0541863	0.1711862	0.5976777
ENSG00000089916	GPATCH2L	14	76669472	T	G	0.251491	-0.0120453	0.016281	0.5400003	0.184316	0.00993683	8.34E-77	-0.0653513	0.0884021	0.4597552	0.6259631
ENSG00000090006	LTBP4	19	41117257	T	G	0.227634	-0.00101571	0.017847	0.98	-0.288819	0.00962267	6.37E-198	0.00351677	0.0617932	0.9546153	0.5637688
ENSG00000090013	BLVRB	19	40962721	G	C	0.117296	-0.0189784	0.023591	0.3400001	-0.750845	0.0116266	0	0.0252761	0.0314217	0.421158	0.8300146
ENSG00000090020	SLC9A1	1	27459389	T	C	0.303181	-0.0176059	0.0161679	0.1900002	0.106273	0.00893099	1.19E-32	-0.165666	0.152771	0.2781829	0.877823
ENSG00000090054	SPTLC1	9	94835973	G	A	0.158052	0.0107349	0.0197334	0.6899999	0.0863181	0.0108911	2.27E-15	0.124364	0.22915	0.5873229	0.08206096
ENSG00000090060	PAPOLA	14	97000609	C	G	0.207753	0.0041749	0.0182158	0.9299999	-0.256733	0.0148164	2.91E-67	-0.0162616	0.0709585	0.8187359	0.7613749
ENSG00000090061	CCNK	14	99974443	G	A	0.449304	-0.00644278	0.0143637	0.3599996	-0.0753546	0.00795131	2.62E-21	0.0854995	0.190828	0.6541211	0.3401933
ENSG00000090097	PCBP4	3	51999751	C	G	0.0467197	0.0137446	0.0359544	0.7700005	0.126391	0.0190842	3.52E-11	0.108747	0.284944	0.7027259	0.7309871
ENSG00000090238	YPEL3	16	30105935	G	A	0.482107	0.0225083	0.0141952	0.07699987	0.247865	0.0078568	1.92E-218	0.0908087	0.0573422	0.1132783	0.7050371
ENSG00000090263	MRPS33	7	140710441	A	G	0.084493	-0.0347824	0.0241953	0.2	-0.35852	0.0142818	4.59E-139	0.0970167	0.0675972	0.1512253	0.4162986
ENSG00000090266	NDUFB2	7	140406583	G	A	0.154076	-0.00357759	0.0206395	0.6100002	-0.450437	0.0107747	0	0.00794249	0.0458215	0.8623877	0.03430788
ENSG00000090273	NUDC	1	27250041	G	A	0.0576541	0.0846416	0.0274188	0.001199997	-0.157831	0.0158762	2.75E-23	-0.536279	0.181905	0.003197031	0.6234721
ENSG00000090316	MAEA	4	1308787	C	G	0.130219	0.0121358	0.0214602	0.4500005	-0.219692	0.0134229	3.29E-60	-0.05524	0.0977413	0.5719613	0.5967257
ENSG00000090339	ICAM1	19	10389401	T	C	0.275348	0.0155874	0.0153881	0.2599998	0.181679	0.00996486	2.88E-74	0.0857962	0.0848299	0.311829	0.2897437
ENSG00000090372	STRN4	19	47236507	T	C	0.00994036	-0.167434	0.206632	0.29	-0.897863	0.071487	3.51E-36	0.186481	0.230616	0.4187341	0.761168
ENSG00000090376	IRAK3	12	66615530	T	C	0.407555	0.0350672	0.0144069	0.01199997	-0.270395	0.00794498	7.14E-254	-0.129689	0.0534171	0.01518843	0.06900446
ENSG00000090382	LYZ	12	69745067	C	T	0.469185	-0.00195498	0.0142293	0.7899998	-0.892818	0.00622741	0	0.00218967	0.0159375	0.8907217	0.4571103
ENSG00000090432	MUL1	1	20830298	A	C	0.119284	0.0285343	0.0206932	0.1299999	0.635123	0.0118342	0	0.0449272	0.0325921	0.1680593	0.2288564
ENSG00000090447	TFAP4	16	4315131	C	T	0.284294	-0.00458815	0.015911	0.6899999	-0.0848815	0.00886232	9.91E-22	0.0540536	0.187535	0.7731686	0.2795274
ENSG00000090470	PDCD7	15	65417945	C	T	0.0109344	0.0183171	0.0626034	0.9	0.706524	0.0470621	6.07E-51	0.0259257	0.0886244	0.7698784	0.1630776
ENSG00000090487	SPG21	15	65269005	T	C	0.371769	-0.0111685	0.0144618	0.4400003	-0.0992606	0.00815979	4.80E-34	0.112517	0.145989	0.4408704	0.06764722
ENSG00000090520	DNAJB11	3	186300126	T	C	0.314115	-0.0180847	0.0155915	0.2	0.0717103	0.00856427	5.61E-17	-0.252191	0.2195	0.2505823	0.09590947
ENSG00000090530	P3H2	3	189757371	T	C	0.515905	0.0088881	0.0142048	0.5199996	-0.106718	0.00864561	5.27E-35	-0.0832859	0.133277	0.532031	0.8726068
ENSG00000090554	FLT3LG	19	49984179	T	C	0.132207	-0.0195367	0.0234249	0.1800002	-0.0881459	0.0131971	2.40E-11	0.22164	0.267815	0.4079042	0.4112862
ENSG00000090565	RAB11FIP3	16	524315	G	T	0.0864811	0.00816016	0.0267073	0.83	-0.171762	0.015734	9.60E-28	-0.0475086	0.155551	0.7600457	0.2284399
ENSG00000090581	GNPTG	16	1407638	T	C	0.0695825	-0.00409884	0.0248474	0.8600001	0.339743	0.0178446	8.11E-81	-0.0120645	0.0731386	0.8689799	0.7315261
ENSG00000090612	ZNF268	12	133745634	T	G	0.237575	-0.0246977	0.016269	0.08500021	0.181882	0.0101759	1.89E-71	-0.13579	0.0897701	0.1303718	0.8720074
ENSG00000090615	GOLGA3	12	133375469	A	G	0.198807	0.0176316	0.0180929	0.2399999	0.244328	0.0107763	8.31E-114	0.0721636	0.07412	0.3302527	0.3976899
ENSG00000090621	PABPC4	1	40034475	T	G	0.187873	0.0234727	0.017879	0.25	-0.242163	0.0109889	1.27E-107	-0.0969293	0.0739613	0.190013	0.7310539
ENSG00000090659	CD209	19	7808671	C	T	0.260437	0.0362211	0.0164968	0.03799969	-0.200435	0.00910108	1.73E-107	-0.180713	0.0827131	0.02890257	0.04865036
ENSG00000090661	CERS4	19	8299462	G	A	0.512922	0.00417147	0.0143694	0.8499999	-0.352977	0.00934376	0	-0.011818	0.0407103	0.7715915	0.03334941
ENSG00000090674	MCOLN1	19	7593203	A	G	0.212724	0.0173293	0.0176004	0.2999998	0.137719	0.0103221	1.32E-40	0.125831	0.128147	0.3261359	0.4782184
ENSG00000090686	USP48	1	22057445	C	A	0.378728	-0.00280311	0.0150137	0.9	-0.359544	0.00807346	0	0.0077963	0.041758	0.8518944	0.7827746
ENSG00000090857	PDPR	16	70171366	C	T	0.404573	0.00400325	0.0145779	0.83	0.0808235	0.0097873	1.48E-16	0.0495307	0.180467	0.7837318	0.5655756
ENSG00000090861	AARS1	16	70304822	T	C	0.139165	0.0248022	0.022555	0.1800002	0.184067	0.0129738	1.09E-45	0.134745	0.122904	0.2729283	0.9775427

ENSG00000090863	GLG1	16	74563434	C	G	0.337972	0.0267564	0.014858	0.06199976	-0.197672	0.0082177	7.51E-128	-0.135358	0.0753753	0.07252914	0.9601812
ENSG00000090905	TNRC6A	16	24789984	G	A	0.138171	-0.0010231	0.022664	0.6600001	-0.303812	0.0114827	2.93E-154	0.00336754	0.0745988	0.9639941	0.2143504
ENSG00000090920	NA	19	40397248	A	C	0.271372	-0.0170414	0.0158451	0.3100002	0.567146	0.00840992	0	-0.0300476	0.0279419	0.2822123	0.277889
ENSG00000090924	PLEKHG2	19	39911139	T	C	0.388668	-0.00400457	0.0149811	0.5700002	-0.0819073	0.00819254	1.56E-23	0.0488915	0.182968	0.7893054	0.7865883
ENSG00000090971	NAT14	19	55997653	T	G	0.184891	-0.00421989	0.0185582	0.8600001	0.142434	0.012236	2.56E-31	-0.029627	0.130318	0.8201567	0.7912993
ENSG00000090975	PITPNM2	12	123551294	T	C	0.215706	-0.0141048	0.0175931	0.32	0.0648861	0.00968103	2.05E-11	-0.217378	0.273071	0.4260038	0.4305738
ENSG00000090989	EXOC1	4	56745491	T	C	0.0765408	-0.00316803	0.0247686	0.91	0.13279	0.0145399	6.67E-20	-0.0238574	0.186542	0.898234	0.5263948
ENSG00000091009	RBM27	5	145650963	T	C	0.00894632	-0.0734631	0.064838	0.2	-0.599674	0.0701061	1.19E-17	0.122505	0.109067	0.2613463	0.7070271
ENSG00000091039	OSBPL8	12	76849583	A	G	0.121272	-0.01122	0.0221459	0.6600001	0.91473	0.0158122	0	-0.0122659	0.0242112	0.612421	0.9759287
ENSG00000091073	DTX2	7	76113152	C	G	0.515905	-0.00655716	0.014206	0.89	-0.490623	0.00818762	0	0.013365	0.0289559	0.6443947	0.1591101
ENSG00000091106	NLRC4	2	32470222	T	C	0.352883	0.0115324	0.0149276	0.8600001	-0.240017	0.00809226	2.51E-193	-0.0480482	0.0622149	0.4399406	0.9005532
ENSG00000091127	PUS7	7	105121411	T	C	0.131213	-0.00319507	0.0216895	0.64	0.594722	0.011389	0	-0.00537237	0.0364701	0.8828882	0.4094017
ENSG00000091129	NRCAM	7	107942614	G	A	0.32505	0.00147218	0.0154738	0.8600001	0.0922696	0.008736	4.47E-26	0.0159552	0.167709	0.9242066	0.4387956
ENSG00000091140	DLD	7	107551795	T	G	0.126243	-0.0282575	0.0221934	0.16	0.132236	0.0119575	1.99E-28	-0.213691	0.168941	0.2059133	0.5967382
ENSG00000091157	WDR7	18	54508701	C	A	0.131213	-0.0107822	0.0209821	0.6300007	-0.133764	0.0114839	2.35E-31	0.080606	0.157011	0.6076878	0.2319404
ENSG00000091164	TXNL1	18	54291635	C	T	0.0606362	-0.0344673	0.0304374	0.2399999	-0.634526	0.0169302	2.03E-307	0.0543198	0.0479906	0.2576836	0.5063838
ENSG00000091262	ABCC6	16	16280082	T	C	0.202783	-0.0207849	0.0180981	0.25	-0.266628	0.0123004	3.43E-104	0.0779548	0.067973	0.251444	0.6888084
ENSG00000091317	CMTM6	3	32533852	C	A	0.348907	0.017372	0.0148118	0.2099999	-0.172481	0.00822922	1.53E-97	-0.100718	0.0860092	0.2415921	0.5000259
ENSG00000091409	ITGA6	2	173331631	G	A	0.0318091	-0.084719	0.0720447	0.1800002	0.200427	0.0281064	9.96E-13	-0.422693	0.364311	0.2459457	0.6328455
ENSG00000091436	MAP3K20	2	174036450	A	G	0.240557	-0.0415584	0.0172629	0.008	-0.369706	0.00918035	0	0.112409	0.0467769	0.01625708	0.3808138
ENSG00000091490	SEL1L3	4	25807218	A	G	0.431412	-0.00067898	0.0142395	0.8	0.278017	0.00781485	3.35E-277	-0.00244222	0.0512181	0.961969	0.03827079
ENSG00000091513	TF	3	133481325	C	T	0.10338	0.00538538	0.0238482	0.7199992	-0.205385	0.0120728	6.67E-65	-0.0262209	0.116125	0.8213576	0.9536318
ENSG00000091527	CDV3	3	133300839	G	A	0.125249	0.00638591	0.0203585	0.83	-0.281334	0.0115492	4.59E-131	-0.0226986	0.0723701	0.7537892	0.9419847
ENSG00000091536	MYO15A	17	18047568	G	A	0.399602	0.0195881	0.014642	0.14	-0.073532	0.00809325	1.03E-19	-0.266389	0.201271	0.1856587	0.189012
ENSG00000091542	ALKBH5	17	18099830	A	G	0.387674	-0.0241984	0.0143742	0.1299999	0.210409	0.0120285	1.63E-68	-0.115006	0.0686311	0.09379336	0.1912203
ENSG00000091592	NLRP1	17	5462745	C	T	0.0666004	-0.00256885	0.033354	0.8600001	-0.600238	0.0192597	3.13E-213	0.00427972	0.0555682	0.9386096	0.1245147
ENSG00000091622	PITPNM3	17	6407199	G	T	0.358847	0.0213858	0.0145105	0.08300036	0.122686	0.00814508	2.85E-51	0.174313	0.118838	0.1424275	0.9378258
ENSG00000091640	SPAG7	17	4866844	A	G	0.083499	-0.021565	0.0285242	0.25	0.93539	0.0141393	0	-0.0230546	0.0304964	0.449664	0.9772291
ENSG00000091831	ESR1	6	152214290	G	A	0.469185	-0.002518	0.0142873	0.7700005	-0.123822	0.00866495	2.53E-46	0.0203356	0.115394	0.8601156	0.7387097
ENSG00000091844	RGS17	6	153392120	T	G	0.484095	-0.0106699	0.0142117	0.4400003	0.331381	0.00790438	0	-0.0321983	0.0428932	0.4528558	0.04297538
ENSG00000091879	ANGPT2	8	6389051	G	C	0.0139165	0.0343479	0.0592524	0.5999997	0.346852	0.0385991	2.56E-19	0.0990277	0.171184	0.5629364	0.7781928
ENSG00000091947	TMEM101	17	42094935	G	A	0.0377734	-0.000871745	0.033256	0.9599999	-0.710257	0.0213085	1.32E-243	0.00122737	0.0468225	0.9790873	0.08950498
ENSG00000091972	CD200	3	112066787	G	A	0.243539	0.00717174	0.0170455	0.6200004	-0.121111	0.0090893	1.67E-40	-0.0592161	0.140813	0.6740973	0.9790058
ENSG00000092010	PSME1	14	24606771	C	T	0.323062	-0.00100221	0.0157563	0.9699999	-0.139464	0.00876659	5.52E-57	0.00718615	0.112978	0.9492836	0.5973548
ENSG00000092020	PPP2R3C	14	35573198	T	G	0.163022	-0.0213015	0.0190616	0.2999998	-0.90185	0.0092417	0	0.0236198	0.0211375	0.2638083	0.8314551
ENSG00000092036	HAUS4	14	23420903	T	C	0.187873	-0.00975863	0.0174378	0.4799997	0.828775	0.0088058	0	-0.0117748	0.0210408	0.575742	0.3269166
ENSG00000092067	CEBPE	14	23587669	A	G	0.449304	0.0184988	0.0143321	0.2599998	0.060371	0.00800124	4.52E-14	0.306419	0.240849	0.2032864	0.733818
ENSG00000092068	SLC7A8	14	23623693	A	G	0.116302	0.0223273	0.0230605	0.32	0.841751	0.0129898	0	0.0265248	0.0273989	0.3329958	0.7175103
ENSG00000092094	OSGEP	14	20918917	C	T	0.352883	0.011388	0.0150682	0.5099998	0.608766	0.00832685	0	0.0187067	0.0247534	0.449815	0.5960835
ENSG00000092096	SLC22A17	14	23818818	A	G	0.0318091	-0.00910284	0.0381316	0.81	0.446974	0.0188973	1.10E-123	-0.0203655	0.0853148	0.8113308	0.7667583
ENSG00000092108	SCFD1	14	31148168	T	C	0.364811	-0.00286118	0.0151376	0.5400003	-0.327053	0.00845198	0	0.00874838	0.0462854	0.8500856	0.2378904
ENSG00000092140	G2E3	14	31058799	G	A	0.0377734	0.0521553	0.0381396	0.2099999	-0.386222	0.0223131	4.01E-67	-0.13504	0.099058	0.172808	0.2666374
ENSG00000092148	HECTD1	14	31623164	A	G	0.028827	0.0175947	0.0355809	0.4799997	0.226382	0.0235393	6.76E-22	0.0777213	0.15738	0.6214149	0.8000117
ENSG00000092200	RPGRIP1	14	21787779	C	T	0.261431	-0.011621	0.0159779	0.3800004	-0.253968	0.0088707	2.84E-180	0.0457577	0.0629333	0.4671754	0.8868397
ENSG00000092203	TOX4	14	21956037	C	T	0.210736	0.00482422	0.0176201	0.7700005	-0.106635	0.0102522	2.45E-25	-0.0452405	0.165295	0.7843183	0.9276925
ENSG00000092208	GEMIN2	14	39594802	C	T	0.340954	-0.0106047	0.0150496	0.3800004	0.0896725	0.00933719	7.71E-22	-0.11826	0.16828	0.4822048	0.6024584
ENSG00000092295	TGM1	14	24725979	A	G	0.143141	0.0148914	0.0195616	0.3599996	0.333648	0.0112576	4.91E-193	0.044632	0.0586487	0.4466536	0.9217194
ENSG00000092421	SEMA6A	5	115844971	G	T	0.258449	-0.0261223	0.0157938	0.1100001	-0.134429	0.00874336	2.41E-53	0.19432	0.118166	0.1000789	0.7750861
ENSG00000092439	TRPM7	15	50911841	A	G	0.435388	-0.00593004	0.0142629	0.6200004	0.0779377	0.00801647	2.42E-22	-0.0760869	0.183171	0.6778585	0.878528
ENSG00000092529	CAPN3	15	42672408	T	C	0.0178926	-0.026574	0.0475411	0.6499995	1.10133	0.040514	1.01E-162	-0.024129	0.0431761	0.5762635	0.2925029

ENSG00000092531	SNAP23	15	42810489	A	G	0.222664	-0.0046158	0.0166328	0.98	0.177549	0.0104559	1.14E-64	-0.0259973	0.0936926	0.7814155	0.9995173
ENSG00000092621	PHGDH	1	120244629	A	G	0.112326	0.0670982	0.0236965	0.003899959	0.773856	0.012921	0	0.0867063	0.0306555	0.004678036	0.4700853
ENSG00000092758	COL9A3	20	61460053	A	G	0.217694	0.000839471	0.0174806	0.8700001	-0.775363	0.0145676	0	-0.00108268	0.0225451	0.9616979	0.2778551
ENSG00000092820	EZR	6	159213608	T	C	0.528827	0.00188892	0.0141549	0.95	-0.156436	0.00868636	1.64E-72	-0.0120747	0.090486	0.8938433	0.2332564
ENSG00000092841	MYL6	12	56554612	A	G	0.0954274	-0.0100764	0.0249962	0.7199992	0.197329	0.0147463	7.75E-41	-0.0510639	0.12673	0.6869964	0.7619283
ENSG00000092847	AGO1	1	36365310	A	G	0.0904573	-0.024823	0.0272463	0.3599996	0.122482	0.0159598	1.66E-14	-0.202667	0.224014	0.3656208	0.09517381
ENSG00000092850	TEKT2	1	36551776	T	C	0.171968	-0.0368825	0.0190491	0.02100003	0.0913843	0.0107037	1.37E-17	-0.403598	0.213744	0.05899466	0.193755
ENSG00000092871	RFFL	17	33374673	A	C	0.427435	0.0210465	0.0142949	0.09499921	0.271855	0.00806259	3.14E-249	0.0774181	0.0526329	0.1413163	0.5456693
ENSG00000092929	UNC13D	17	73832052	C	G	0.246521	0.0168373	0.0167562	0.25	-0.243633	0.00984891	4.28E-135	-0.0691094	0.0688332	0.3153728	0.2221181
ENSG00000092931	MFSD11	17	74754739	C	T	0.10338	0.0117168	0.0238923	0.6999999	-0.266506	0.0180189	1.69E-49	-0.0439644	0.0896993	0.624041	0.1615324
ENSG00000092964	DPYSL2	8	26443742	A	G	0.152087	-0.0306524	0.0203437	0.2	0.137244	0.0120538	4.91E-30	-0.223342	0.149522	0.1352527	0.08766363
ENSG00000092969	TGFB2	1	218568769	T	C	0.136183	0.00929264	0.0219934	0.8600001	0.0819862	0.0118893	5.36E-12	0.113344	0.268761	0.6732231	0.472321
ENSG00000092978	GPATCH2	1	217702379	C	T	0.388668	-0.0066035	0.014518	0.7099994	0.0554548	0.00820385	1.38E-11	-0.119079	0.262391	0.6499563	0.4778802
ENSG00000093000	NUP50	22	45571807	T	C	0.279324	0.012352	0.0165793	0.5199996	-0.266786	0.00879858	5.97E-202	-0.0462993	0.0621633	0.4563923	0.2258732
ENSG00000093009	CDC45	22	19487558	T	C	0.170974	0.0238466	0.017555	0.17	-0.0720794	0.00979739	1.88E-13	-0.330838	0.247668	0.18161	0.1833645
ENSG00000093010	COMT	22	19943314	G	A	0.483101	0.0135399	0.0142044	0.3800004	0.275823	0.00785518	4.19E-270	0.0490891	0.0515173	0.3406571	0.2212652
ENSG00000093072	ADA2	22	17681536	T	C	0.426441	0.0154958	0.0144075	0.29	0.38677	0.007772	0	0.0400647	0.0372596	0.2822467	0.7445174
ENSG00000093134	VNN3P	6	133049915	C	T	0.392644	-0.0198151	0.0145991	0.1100001	-0.669626	0.00719731	0	0.0295913	0.0218042	0.1747374	0.4665757
ENSG00000093144	ECHDC1	6	127637304	T	C	0.279324	0.030219	0.016495	0.09200046	0.283127	0.00894741	9.32E-220	0.106733	0.0583576	0.06740754	0.5970638
ENSG00000093167	LRRFIP2	3	37159648	C	T	0.439364	-0.0274766	0.0142441	0.04499974	-0.407236	0.00770596	0	0.0674709	0.0350008	0.05389278	0.03484103
ENSG00000093183	SEC22C	3	42616016	A	G	0.196819	0.00425307	0.0178507	0.91	-0.15788	0.00962716	1.93E-60	-0.0269387	0.113077	0.8117006	0.029962
ENSG00000093217	XYLB	3	38425545	T	G	0.323062	0.00848802	0.0150999	0.5099998	0.209838	0.00843738	1.57E-136	0.0404504	0.0719783	0.5741292	0.1401906
ENSG00000094880	CDC23	5	137536185	A	C	0.0407555	0.0366118	0.0352791	0.33	0.180341	0.0211395	1.45E-17	0.203014	0.197066	0.3029248	0.1951045
ENSG00000094916	CBX5	12	54649305	G	A	0.146123	-0.0351131	0.0212636	0.16	0.106165	0.0113223	6.81E-21	-0.330742	0.203371	0.1038865	0.5941565
ENSG00000094975	SUCO	1	172541230	T	C	0.33002	-0.00371464	0.0150553	0.81	-0.0747641	0.00930157	9.15E-16	0.0496848	0.201465	0.8052047	0.3735859
ENSG00000095002	MSH2	2	47709779	T	C	0.147117	0.0359761	0.0210689	0.05099998	0.761485	0.0108178	0	0.0472446	0.0276763	0.08781405	0.3183158
ENSG00000095015	MAP3K1	5	56151690	G	T	0.343936	0.0307588	0.0148836	0.05999983	0.200199	0.00898721	6.32E-110	0.153641	0.0746633	0.03961058	0.9620235
ENSG00000095059	DHPS	19	12789623	G	A	0.404573	0.000836763	0.0144988	0.7700005	-0.0919422	0.0081709	2.25E-29	-0.00910096	0.157697	0.9539782	0.7018523
ENSG00000095066	HOOK2	19	12928685	A	G	0.0815109	0.000789829	0.024026	0.7899998	0.203941	0.0156856	1.19E-38	0.00387284	0.117809	0.9737752	0.8205247
ENSG00000095139	ARCN1	11	118458426	T	C	0.131213	-0.00559756	0.0215212	0.7600007	-0.209001	0.0119615	2.31E-68	0.0267825	0.102983	0.7948126	0.9891264
ENSG00000095209	TMEM38B	9	108497859	G	A	0.311133	-0.0268406	0.0158875	0.03599979	0.0913082	0.00885405	6.18E-25	-0.293956	0.176318	0.09547605	0.3924504
ENSG00000095261	PSMD5	9	123591501	A	G	0.410537	-0.0157278	0.0143903	0.29	0.211117	0.00805333	1.80E-151	-0.0744979	0.0682218	0.2748349	0.8462817
ENSG00000095303	PTGS1	9	125145403	A	G	0.138171	-0.00446418	0.0221066	0.6899999	-0.155579	0.0122207	3.99E-37	0.028694	0.14211	0.8399844	0.2472419
ENSG00000095321	CRAT	9	131865278	C	A	0.265408	0.0163513	0.0154678	0.2200002	-0.60228	0.00861028	0	-0.027149	0.025685	0.2905125	0.2730358
ENSG00000095370	SH2D3C	9	130520808	T	C	0.383698	-0.00735819	0.0144098	0.4799997	-0.117892	0.00848819	7.39E-44	0.0624144	0.122311	0.6098461	0.03335563
ENSG00000095380	NANS	9	100832189	T	C	0.0208748	0.107646	0.0604625	0.07799917	-0.425578	0.0578404	1.87E-13	-0.252941	0.146171	0.08355202	0.4309798
ENSG00000095383	TBC1D2	9	100989613	C	T	0.294235	-0.0239881	0.0159002	0.14	0.242945	0.00871345	4.46E-171	-0.0987388	0.0655435	0.1319484	0.1873318
ENSG00000095397	WHRN	9	117216045	C	G	0.392644	-0.00476278	0.0147398	0.5400003	0.250167	0.00813625	1.33E-207	-0.0190384	0.0589231	0.746615	0.7732117
ENSG00000095464	PDE6C	10	95399056	A	G	0.119284	-0.0139544	0.0226122	0.5199996	0.0911799	0.0147131	5.75E-10	-0.153043	0.249222	0.5391618	0.7468021
ENSG00000095485	CWF19L1	10	102009746	C	G	0.427435	0.00979871	0.0142661	0.3700002	0.634581	0.00733182	0	0.0154412	0.0224818	0.4921892	0.2882389
ENSG00000095539	SEMA4G	10	102737451	C	G	0.182903	-0.014215	0.0174171	0.4400003	0.10943	0.00984493	1.06E-28	-0.1299	0.15959	0.4156674	0.7609893
ENSG00000095564	BTAF1	10	93736804	C	T	0.0785288	0.0280738	0.0247881	0.3800004	-0.232196	0.0136568	7.91E-65	-0.120905	0.106992	0.2584563	0.168661
ENSG00000095574	IKZF5	10	124759327	T	C	0.027833	0.0505616	0.0443271	0.4100001	-0.19231	0.0245512	4.76E-15	-0.262918	0.23293	0.2590064	0.8161082
ENSG00000095627	TDRD1	10	115965546	C	T	0.0109344	0.110565	0.0691039	0.09099971	0.383894	0.0644203	2.53E-09	0.28801	0.186383	0.1222846	NA
ENSG00000095637	SORBS1	10	97196349	C	T	0.303181	0.00090807	0.0160905	0.7899998	-0.0763174	0.00882568	5.28E-18	-0.0118986	0.210841	0.9549961	0.9937866
ENSG00000095739	BAMBI	10	28969069	G	C	0.264414	0.0152248	0.0160758	0.35	0.411766	0.00847196	0	0.0369744	0.0390485	0.3436981	0.2777124
ENSG00000095787	WAC	10	28865675	T	C	0.294235	0.0127469	0.0158647	0.32	0.250192	0.00884328	4.36E-176	0.0509484	0.0634356	0.421887	0.9873779
ENSG00000095794	CREM	10	35458802	C	G	0.184891	0.0167721	0.018689	0.29	-0.133348	0.0110216	1.07E-33	-0.125776	0.140537	0.3708015	0.9206035
ENSG00000095906	NUBP2	16	1836047	T	A	0.313121	0.00405141	0.015665	0.9400001	-0.101601	0.00861085	3.94E-32	-0.0398756	0.154218	0.7959701	0.3884647
ENSG00000095917	TPSD1	16	1307296	A	G	0.190855	-0.0184568	0.0181785	0.33	0.348395	0.0150152	4.27E-119	-0.0529766	0.0522277	0.3104212	0.7787706

ENSG00000095932	SMIM24	19	3477472	A	G	0.44831	-0.0170292	0.0143597	0.2200002	0.0850551	0.00975604	2.83E-18	-0.200214	0.170383	0.2399624	0.973165
ENSG00000095951	HIVEP1	6	12087113	A	G	0.188867	0.0192331	0.0194772	0.5300002	-0.15689	0.0102851	1.54E-52	-0.122589	0.124405	0.3244252	0.1401167
ENSG00000096006	CRISP3	6	49703623	G	A	0.238569	-0.0123117	0.0166259	0.4500005	-0.168268	0.00924131	4.44E-74	0.0731674	0.098888	0.4593597	0.4076976
ENSG00000096063	SRPK1	6	35844931	G	A	0.323062	-0.0327842	0.0153099	0.02100003	0.315206	0.008189	0	-0.104009	0.0486462	0.03251135	0.1071573
ENSG00000096070	BRPF3	6	36182544	T	G	0.405567	0.0268769	0.0146123	0.1100001	-0.0574611	0.00879229	6.34E-11	-0.46774	0.264178	0.07663588	0.08074868
ENSG00000096080	MRPS18A	6	43647284	C	T	0.027833	0.070059	0.0539233	0.2999998	0.194543	0.0319143	1.09E-09	0.36012	0.283405	0.2038379	0.985066
ENSG00000096092	TMEM14A	6	52543646	C	G	0.287276	-0.0110841	0.0160314	0.33	-0.190062	0.00885773	3.91E-102	0.0583183	0.084392	0.4895399	0.1696537
ENSG00000096093	EFHC1	6	52336499	G	T	0.311133	-0.00157947	0.0159452	0.7199992	-0.177635	0.00886818	2.98E-89	0.00889166	0.089765	0.9210947	0.04903125
ENSG00000096384	HSP90AB1	6	44218222	C	A	0.537773	0.00889784	0.014477	0.3700002	-0.0451942	0.00817344	3.21E-08	-0.19688	0.322301	0.5412943	0.9357283
ENSG00000096401	CDC5L	6	44386712	A	T	0.0785288	-0.0171586	0.0280721	0.5500004	-0.167862	0.0135931	4.93E-35	0.102219	0.167438	0.54154	0.1555886
ENSG00000096433	ITPR3	6	33626436	A	G	0.342942	-0.0197634	0.0146351	0.2200002	-0.474528	0.00789113	0	0.0416485	0.0308492	0.1769933	0.6443452
ENSG00000096654	ZNF184	6	27429709	G	A	0.286282	0.00452688	0.0159325	0.7800007	0.160282	0.00873298	3.09E-75	0.0282433	0.099415	0.7763379	0.04159641
ENSG00000096696	DSP	6	7564379	G	A	0.423459	0.0211229	0.0143276	0.1800002	-0.641702	0.00716339	0	-0.032917	0.0223305	0.1404598	0.1174439
ENSG00000096717	SIRT1	10	69661287	C	G	0.331014	-0.0261219	0.0150886	0.03899959	0.712669	0.0121184	0	-0.0366536	0.0211811	0.08354375	0.009064969
ENSG00000096746	HNRNPH3	10	70096939	A	G	0.138171	-0.0229421	0.0204123	0.2300001	0.133889	0.0131726	2.86E-24	-0.171352	0.153387	0.2639404	0.8109389
ENSG00000096872	IFT74	9	27004982	T	C	0.104374	-0.0549194	0.0246887	0.02300011	0.236282	0.0133551	4.82E-70	-0.232432	0.105311	0.02730721	0.02534544
ENSG00000096968	JAK2	9	5056608	T	C	0.5	0.00427352	0.0143521	0.8200001	0.200572	0.00793997	8.57E-141	0.0213067	0.0715609	0.7659001	0.8993455
ENSG00000096996	IL12RB1	19	18189782	C	T	0.199801	-0.00132055	0.0173064	0.9199999	-0.501916	0.00930845	0	0.00263102	0.0344807	0.9391772	0.5361854
ENSG00000097007	ABL1	9	133676197	A	G	0.0198807	-0.0315118	0.0513489	0.4400003	0.195752	0.0302797	1.01E-10	-0.160978	0.263495	0.5412434	0.83747
ENSG00000097021	ACOT7	1	6389392	G	C	0.0904573	0.00534278	0.0247922	0.83	0.33236	0.0148767	1.48E-110	0.0160753	0.0745979	0.8293834	0.150092
ENSG00000097033	SH3GLB1	1	87192063	T	C	0.0775348	-0.0139203	0.0313819	0.5300002	-0.341926	0.0170579	2.23E-89	0.0407115	0.0918023	0.6574267	0.3068308
ENSG00000097046	CDC7	1	91978864	T	G	0.491054	-0.00538025	0.0142169	0.8200001	-0.562981	0.00731721	0	0.00955672	0.0252532	0.7051068	0.3181548
ENSG00000097096	SYDE2	1	85644642	G	T	0.237575	-0.0197478	0.0162422	0.25	0.119936	0.0103911	8.08E-31	-0.164653	0.136174	0.2266079	0.2268902
ENSG00000099139	PCSK5	9	78741407	G	T	0.518887	-0.0321979	0.0142287	0.02199986	-0.182602	0.00793403	3.30E-117	0.176329	0.0782978	0.0243207	0.8169691
ENSG00000099194	SCD	10	102115736	G	C	0.398608	0.0151396	0.014319	0.1	0.122368	0.00813468	3.85E-51	0.123722	0.117304	0.2915596	0.303372
ENSG00000099203	TMED1	19	10945054	A	G	0.027833	0.0189395	0.0471617	0.83	-0.751061	0.0244614	5.05E-207	-0.025217	0.0627988	0.6880137	0.8874861
ENSG00000099204	ABLIM1	10	116317817	C	T	0.511928	-0.0141577	0.0142296	0.2200002	-0.13402	0.00790928	2.11E-64	0.105639	0.106358	0.3205948	0.4286618
ENSG00000099219	ERMP1	9	5799096	A	G	0.139165	0.037915	0.0210218	0.0530005	-0.691333	0.0130731	0	-0.0548433	0.0304253	0.071458	0.9025016
ENSG00000099246	RAB18	10	27812170	T	C	0.512922	-0.0109307	0.0142324	0.4199997	0.110118	0.00796402	1.75E-43	-0.0992631	0.129445	0.4431807	0.09965505
ENSG00000099250	NRP1	10	33545805	T	C	0.145129	0.00776199	0.0201053	0.6300007	-0.43159	0.0122104	1.15E-273	-0.0179846	0.0465871	0.6994642	0.0552524
ENSG00000099251	NA	10	38656369	C	T	0.420477	0.0104696	0.0142563	0.4299995	0.636864	0.0159477	0	0.0164393	0.0223889	0.4627903	0.6055388
ENSG00000099256	PRTFDC1	10	25189534	T	C	0.236581	-0.00937398	0.0172935	0.4299995	-0.0871729	0.00946441	3.24E-20	0.107533	0.198725	0.5884277	0.7067838
ENSG00000099282	TSPAN15	10	71239327	A	C	0.218688	-0.0214807	0.0166791	0.2	0.231301	0.00938798	4.95E-134	-0.092869	0.0722084	0.1983993	0.5563924
ENSG00000099284	MACROH2A2	10	71842294	G	T	0.412525	-0.0149672	0.0142774	0.35	-0.203893	0.00796196	1.23E-144	0.0734071	0.0700826	0.2948981	0.0430821
ENSG00000099290	WASHC2A	10	51860458	A	G	0.349901	-0.0300169	0.0146292	0.025	-0.762983	0.0156393	0	0.0393415	0.0191906	0.04036091	0.2346253
ENSG00000099308	MAST3	19	18235548	G	A	0.299205	0.00579328	0.0155797	0.6800001	-0.22446	0.00976373	5.98E-117	-0.0258098	0.0694187	0.7100422	0.1295534
ENSG00000099326	MZF1	19	59079120	C	T	0.263419	0.0120493	0.0172278	0.5099998	0.310055	0.0093469	2.73E-241	0.0388618	0.055576	0.4843928	0.07876886
ENSG00000099330	OCEL1	19	17338520	A	G	0.107356	-0.0128175	0.0220033	0.6499995	-0.56122	0.0123878	0	0.0228386	0.0392095	0.5602447	0.6763863
ENSG00000099331	MYO9B	19	17255968	A	G	0.139165	0.0172939	0.0212645	0.32	0.163942	0.0113888	5.56E-47	0.105488	0.129915	0.4168027	0.235199
ENSG00000099337	KCNK6	19	38815072	A	G	0.182903	-0.0257561	0.0199962	0.17	0.300084	0.0110581	3.61E-162	-0.0858298	0.0667105	0.1982333	0.367508
ENSG00000099338	CATSPERG	19	38844002	T	C	0.0387674	-0.0182155	0.0396135	0.4700002	0.534515	0.0222683	2.56E-127	-0.0340785	0.0741246	0.6456984	0.7723707
ENSG00000099341	PSMD8	19	38869820	A	G	0.0248509	-0.0557766	0.0445093	0.1499999	0.385672	0.0338119	3.88E-30	-0.144622	0.116102	0.2128929	0.5966209
ENSG00000099365	STX1B	16	31011263	A	G	0.242545	0.022743	0.0164465	0.1100001	-0.116114	0.0116477	2.09E-23	-0.195869	0.142998	0.1707707	0.6324285
ENSG00000099377	HSD3B7	16	30998496	G	A	0.382704	-0.0286056	0.0146743	0.025	0.197513	0.00818447	1.14E-128	-0.144829	0.0745375	0.05201152	0.4392789
ENSG00000099381	SETD1A	16	30982526	G	A	0.358847	0.00691431	0.0147547	0.5700002	0.124189	0.00888106	1.96E-44	0.0556756	0.118875	0.6395311	0.3858889
ENSG00000099385	BCL7C	16	30875614	A	C	0.0934394	-0.0152327	0.0224791	0.5300002	-0.160722	0.0134244	4.96E-33	0.0947766	0.140087	0.498688	0.169203
ENSG00000099622	CIRBP	19	1267131	C	T	0.208748	0.00246328	0.016306	0.4500005	-0.10508	0.0110039	1.30E-21	-0.0234419	0.155196	0.8799387	0.5929578
ENSG00000099624	ATP5F1D	19	1243286	C	G	0.256461	-0.0172298	0.0167173	0.28	-0.32457	0.0117312	1.73E-168	0.053085	0.0515417	0.303037	0.6583292
ENSG00000099769	IGFALS	16	1842693	A	G	0.172962	-0.031505	0.019991	0.1100001	-0.0955181	0.0109386	2.50E-18	0.329833	0.212671	0.1209248	0.1552895
ENSG00000099785	MARCHF2	19	8491027	A	G	0.292247	0.0167721	0.0151264	0.17	-0.305905	0.00921982	2.16E-241	-0.0548278	0.0494756	0.267785	0.9477026

ENSG00000099795	NDUFB7	19	14679882	A	G	0.454274	-0.00459932	0.0144052	0.7700005	0.0707188	0.0118807	2.64E-09	-0.0650367	0.20399	0.7498603	0.5653684
ENSG00000099797	TECR	19	14652344	C	T	0.45825	0.00136225	0.0142204	0.8800001	-0.12437	0.0110642	2.57E-29	-0.0109532	0.114343	0.9236858	0.8089993
ENSG00000099800	TIMM13	19	2426757	C	T	0.281312	-0.0209106	0.0155981	0.2300001	0.236266	0.00919666	1.50E-145	-0.0885045	0.0661091	0.1806472	0.4251844
ENSG00000099804	CDC34	19	536900	T	C	0.45328	-0.00860432	0.0142256	0.5199996	0.283867	0.0103526	1.59E-165	-0.0303111	0.0501259	0.545378	0.05704628
ENSG00000099810	MTAP	9	21867640	A	T	0.27336	0.00255232	0.016269	0.84	-0.203122	0.00860505	3.42E-123	-0.0125654	0.0800964	0.8753403	0.200585
ENSG00000099814	CEP170B	14	105347362	C	G	0.395626	0.00745468	0.0145155	0.5700002	0.373818	0.0115409	3.70E-230	0.019942	0.0388353	0.6076	0.7346239
ENSG00000099817	POLR2E	19	1091096	A	G	0.249503	-0.0283295	0.0170029	0.02300011	-0.308336	0.0124744	6.92E-135	0.0918786	0.0552691	0.09643571	0.7908583
ENSG00000099821	POLRMT	19	625395	G	A	0.158052	0.00454869	0.0197878	0.8700001	0.245686	0.019944	7.18E-35	0.0185143	0.0805552	0.8182212	0.2115526
ENSG00000099834	CDHR5	11	621321	A	G	0.149105	0.0258057	0.0212255	0.2399999	-0.106359	0.0162898	6.61E-11	-0.242627	0.202994	0.2319922	0.7071257
ENSG00000099840	IZUMO4	19	2097986	T	C	0.352883	0.011856	0.0168198	0.5199996	-0.0829517	0.0147543	1.89E-08	-0.142926	0.204354	0.4842971	NA
ENSG00000099849	RASSF7	11	562212	G	A	0.178926	0.00492909	0.0183328	0.7300002	0.10478	0.0102047	9.84E-25	0.0470422	0.175024	0.7881027	0.9931539
ENSG00000099860	GADD45B	19	2477188	T	C	0.055666	0.026894	0.0299795	0.5300002	-0.462786	0.0181171	6.37E-144	-0.0581133	0.0648205	0.3699714	0.2437149
ENSG00000099864	PALM	19	728641	G	C	0.0695825	0.0146964	0.0305709	0.6999999	0.649292	0.0203847	1.24E-222	0.0226345	0.0470888	0.6307461	0.918479
ENSG00000099866	MADCAM1	19	497261	A	G	0.110338	-0.0168414	0.0253193	0.4400003	-0.115842	0.0172456	1.85E-11	0.145382	0.219636	0.5080204	0.7845669
ENSG00000099875	MKNK2	19	2044356	T	C	0.289264	-0.0208474	0.0159902	0.2099999	-0.297739	0.0111597	8.08E-157	0.0700191	0.0537696	0.1928456	0.2026917
ENSG00000099889	ARVCF	22	19980875	G	A	0.314115	0.018589	0.0154379	0.2	-0.133152	0.00921185	2.35E-47	-0.139608	0.116344	0.2301556	0.1722681
ENSG00000099899	TRMT2A	22	20102152	C	T	0.0536779	-0.038673	0.0313197	0.2300001	0.133878	0.0175215	2.16E-14	-0.288867	0.236976	0.2228566	0.9102092
ENSG00000099901	RANBP1	22	20109169	C	T	0.0526839	-0.0357344	0.0313652	0.28	0.255729	0.0364369	2.24E-12	-0.139736	0.124256	0.260767	0.4459416
ENSG00000099904	ZDHHC8	22	20126254	C	T	0.0934394	-0.0108253	0.0258957	0.7400005	-0.264669	0.0143122	2.37E-76	0.0409013	0.0978669	0.6759998	0.4201733
ENSG00000099910	KLHL22	22	20816849	T	C	0.285288	-0.0184849	0.0156478	0.2999998	0.100866	0.00885971	4.98E-30	-0.183262	0.155967	0.2399942	0.1318104
ENSG00000099917	MED15	22	20896059	A	G	0.151093	-0.0445717	0.0200891	0.02800013	-0.274163	0.0117306	8.31E-121	0.162574	0.0736037	0.02719062	0.4644399
ENSG00000099940	SNAP29	22	21229386	C	T	0.402584	0.00302031	0.01472	0.9199999	0.639894	0.00739167	0	0.00472002	0.0230039	0.8374287	0.7778077
ENSG00000099942	CRKL	22	21289875	G	A	0.34493	0.00727252	0.0152837	0.84	-0.131031	0.00858713	1.43E-52	-0.0555023	0.116698	0.6343566	0.8969105
ENSG00000099949	LZTR1	22	21343539	G	C	0.484095	0.00365658	0.014237	0.81	0.410001	0.00863504	0	0.00891846	0.0347248	0.797308	0.8132111
ENSG00000099953	MMP11	22	24118458	A	G	0.101392	-0.0165861	0.0230016	0.5500004	-0.18695	0.0133296	1.09E-44	0.0887195	0.123199	0.4714428	0.9993329
ENSG00000099956	SMARCB1	22	24152926	C	T	0.12326	0.00226152	0.0218465	0.81	-0.562618	0.0112018	0	-0.00401964	0.0388302	0.9175515	0.6451793
ENSG00000099957	P2RX6	22	21373608	A	G	0.200795	0.0506655	0.0181932	0.007299952	-0.268913	0.0129486	8.46E-96	-0.188408	0.0682601	0.005777414	0.4946845
ENSG00000099958	DERL3	22	24179002	G	A	0.379722	-0.0191698	0.0147187	0.2599998	-0.128174	0.00881464	6.66E-48	0.149561	0.115294	0.1945561	0.7823649
ENSG00000099960	SLC7A4	22	21385068	G	A	0.205765	0.050501	0.0181575	0.007100027	-0.0945311	0.0122199	1.03E-14	-0.534226	0.204117	0.008863979	0.7988892
ENSG00000099968	BCL2L13	22	18162504	G	T	0.313121	-0.0116091	0.0156384	0.33	-0.51787	0.00822787	0	0.022417	0.0301996	0.4579091	0.243772
ENSG00000099974	DDTL	22	24311905	C	T	0.409543	0.00186157	0.0143492	0.9299999	-0.250222	0.0228722	7.42E-28	-0.00743967	0.0573498	0.8967846	0.8619031
ENSG00000099977	DDT	22	24318107	G	C	0.390656	0.00178233	0.0143884	0.89	-0.76458	0.00687521	0	-0.00233112	0.0188187	0.9014161	0.4883346
ENSG00000099991	CABIN1	22	24491119	C	G	0.0586481	0.00725732	0.030698	0.83	0.282232	0.0150032	6.09E-79	0.025714	0.108777	0.8131289	0.6606562
ENSG00000099992	TBC1D10A	22	30705507	T	C	0.0228628	-0.00282633	0.0476008	0.84	-0.132329	0.0236436	2.18E-08	0.0213584	0.359736	0.9526555	0.9762866
ENSG00000099994	SUSD2	22	24581152	A	G	0.187873	0.0147405	0.019791	0.56	0.324223	0.0104061	4.09E-213	0.045464	0.0610587	0.4565163	0.9180064
ENSG00000099995	SF3A1	22	30740457	A	C	0.154076	0.00576653	0.0177644	0.7300002	0.638883	0.00982944	0	0.00902595	0.0278057	0.7454781	0.1099785
ENSG00000099998	GGT5	22	24628366	G	A	0.0596421	0.00598341	0.0307655	0.9	0.170999	0.0146073	1.18E-31	0.0349909	0.179941	0.8458176	0.7997838
ENSG00000099999	RNF215	22	30795797	G	A	0.246521	-0.00342069	0.0156094	0.7499995	-0.141171	0.00990895	4.69E-46	0.0242308	0.110584	0.8265589	0.270485
ENSG00000100003	SEC14L2	22	30806724	G	A	0.182903	-0.00517991	0.0189579	0.7899998	0.14685	0.0100295	1.52E-48	-0.0352735	0.129119	0.7847112	0.6919341
ENSG00000100012	SEC14L3	22	30855991	G	T	0.387674	0.0130465	0.0144458	0.33	0.274809	0.00820393	5.29E-246	0.0474749	0.0525859	0.3666287	0.4559077
ENSG00000100014	SPECC1L	22	24740246	T	C	0.0427435	0.0207778	0.0322281	0.3800004	0.806063	0.0312665	1.47E-146	0.0257769	0.0399946	0.5192453	0.718796
ENSG00000100023	PPIL2	22	22029562	T	C	0.321074	-0.0176692	0.0151129	0.16	0.168369	0.00823322	6.01E-93	-0.104943	0.0899069	0.2431131	0.8954473
ENSG00000100024	UPB1	22	24893782	G	A	0.389662	-0.0087202	0.0145236	0.4700002	0.465541	0.00764686	0	-0.0187313	0.0311987	0.5482486	0.3311827
ENSG00000100027	YPEL1	22	22070978	A	G	0.253479	0.0134914	0.0165786	0.4500005	-0.11369	0.00961179	2.79E-32	-0.118668	0.146167	0.4168692	0.6478194
ENSG00000100028	SNRPD3	22	24978709	T	C	0.0238569	0.0324663	0.0439598	0.4700002	0.209845	0.036144	6.41E-09	0.154716	0.211175	0.4637773	0.4250964
ENSG00000100029	PES1	22	30987842	A	G	0.389662	0.00359352	0.0144605	0.6600001	0.150914	0.00808802	1.07E-77	0.0238117	0.0958278	0.80376	0.4328518
ENSG00000100030	MAPK1	22	22165379	G	T	0.028827	-0.0271611	0.0353293	0.58	0.261736	0.0187525	2.84E-44	-0.103773	0.135185	0.4427043	0.2104318
ENSG00000100031	GGT1	22	25002345	T	G	0.366799	-0.0051732	0.0149354	0.7099994	-0.220809	0.00972109	3.23E-114	0.0234284	0.0676474	0.7290936	0.9637647
ENSG00000100034	PPM1F	22	22290501	G	A	0.11829	0.000694316	0.0220557	0.98	-0.984628	0.0110498	0	-0.000705156	0.0224	0.9748867	0.3885366
ENSG00000100036	SLC35E4	22	31048321	T	C	0.0695825	-0.0170834	0.0283664	0.56	-0.216368	0.0159087	3.97E-42	0.0789551	0.131231	0.5474064	0.5449944

ENSG00000100038	TOP3B	22	22324305	G	A	0.11829	-0.00192855	0.0220508	0.8800001	-0.626518	0.0218969	4.74E-180	0.0030782	0.035196	0.9303067	0.2247791
ENSG00000100053	CRYBB3	22	25599573	T	C	0.478131	-0.00387806	0.0142734	0.7700005	0.0641144	0.00828458	1.00E-14	-0.0604866	0.222761	0.7859826	0.2182897
ENSG00000100055	CYTH4	22	37694725	G	A	0.407555	-0.0152815	0.0151895	0.2999998	-0.152011	0.00945635	3.82E-58	0.100529	0.100119	0.3153341	0.4909681
ENSG00000100056	ESS2	22	19124994	T	A	0.0775348	0.00354498	0.026925	0.83	-0.714777	0.0202389	3.22E-273	-0.00495956	0.0376694	0.8952529	0.6143807
ENSG00000100058	NA	22	25880446	A	G	0.0397614	-0.019703	0.0319789	0.3599996	0.689489	0.0399297	8.25E-67	-0.0285762	0.0464101	0.5380706	0.5925761
ENSG00000100060	MFNG	22	37873770	T	C	0.0785288	-0.0156535	0.0281667	0.4100001	0.33797	0.0155989	4.28E-104	-0.0463163	0.0833683	0.5785102	0.6053463
ENSG00000100068	NA	22	25774364	C	T	0.187873	-0.00664787	0.0182205	0.6499995	0.277103	0.0104409	3.33E-155	-0.0239906	0.0657598	0.7152442	0.6072147
ENSG00000100075	SLC25A1	22	19164719	A	G	0.374751	0.0274595	0.0147014	0.04700023	0.266471	0.00828412	5.19E-227	0.103049	0.0552636	0.06222707	0.8546747
ENSG00000100077	GRK3	22	26043037	C	T	0.0178926	-0.0437943	0.0529155	0.3599996	0.590205	0.0458234	5.83E-38	-0.0742018	0.089841	0.4088474	0.9296586
ENSG00000100079	LGALS2	22	37972439	G	A	0.439364	0.0325067	0.014509	0.04900044	1.02846	0.00649698	0	0.0316072	0.0141089	0.02507623	0.3163383
ENSG00000100083	GGA1	22	38017026	C	A	0.171968	0.003438	0.0188168	0.98	0.236454	0.011637	8.70E-92	0.0145398	0.0795824	0.8550321	0.5870553
ENSG00000100092	SH3BP1	22	38046800	T	A	0.398608	-0.00406671	0.0143502	0.7199992	-0.213032	0.0098112	1.54E-104	0.0190896	0.0673673	0.776896	0.2212374
ENSG00000100095	SEZ6L	22	26672501	T	C	0.026839	-0.011365	0.0380476	0.95	-0.390314	0.0332766	9.01E-32	0.0291175	0.0975109	0.7652392	0.2015988
ENSG00000100097	LGALS1	22	38073714	A	G	0.338966	0.000862334	0.0149577	0.9299999	-0.297678	0.00893676	2.82E-243	-0.00289687	0.0502479	0.9540263	0.5184538
ENSG00000100099	HPS4	22	26859596	T	C	0.0109344	0.0232727	0.0614828	0.6100002	-0.600103	0.0406227	2.20E-49	-0.0387812	0.102487	0.7051338	0.104975
ENSG00000100100	PIK3IP1	22	31683049	C	T	0.274354	-0.0310229	0.0156527	0.05399953	-0.514421	0.00877715	0	0.0603064	0.0304452	0.04761149	0.9372124
ENSG00000100104	SRRD	22	26883871	C	A	0.0914513	-0.0396383	0.0220987	0.0519996	0.266163	0.0128895	9.84E-95	-0.148925	0.0833395	0.07394317	0.7072775
ENSG00000100105	PATZ1	22	31732004	A	G	0.276342	-0.0309218	0.0156565	0.05499966	-0.0806727	0.00922122	2.16E-18	0.383299	0.198958	0.05403801	0.9239297
ENSG00000100106	TRIOBP	22	38132787	G	T	0.332008	-0.00696637	0.0155822	0.5400003	-0.46192	0.00811502	0	0.0150813	0.0337346	0.6548328	0.1144941
ENSG00000100109	TFIP11	22	26897831	A	G	0.143141	0.0081389	0.0208875	0.7400005	-0.991903	0.00999978	0	-0.00820534	0.0210582	0.6967946	0.05198859
ENSG00000100116	GCAT	22	38208547	A	G	0.125249	-0.0283829	0.020288	0.1199999	0.827012	0.010972	0	-0.0343198	0.0245359	0.1618854	0.781241
ENSG00000100122	CRYBB1	22	27004647	T	A	0.190855	-0.00239202	0.0181306	0.8200001	-0.0628268	0.0100739	4.47E-10	0.0380732	0.288645	0.8950608	0.3009531
ENSG00000100124	ANKRD54	22	38233650	G	A	0.0596421	0.0319558	0.0307406	0.28	0.474736	0.0210401	9.92E-113	0.0673128	0.0648217	0.2990702	0.008610471
ENSG00000100129	EIF3L	22	38265144	G	A	0.0477137	-0.0534324	0.0298569	0.0649995	0.230526	0.0188761	2.66E-34	-0.231784	0.130899	0.07660964	0.8377561
ENSG00000100138	SNU13	22	42078221	C	T	0.430417	-0.00767888	0.01425	0.4799997	0.224436	0.00788371	2.89E-178	-0.0342141	0.0635039	0.590044	0.01498357
ENSG00000100139	MICALL1	22	38320246	G	A	0.361829	-0.0100079	0.0149214	0.33	0.0905946	0.00833023	1.51E-27	-0.110469	0.165018	0.5032174	0.1966831
ENSG00000100147	CCDC134	22	42209493	G	C	0.420477	-0.0172828	0.0143109	0.17	-0.292142	0.00783852	5.03E-304	0.059159	0.0490119	0.2274193	0.01444419
ENSG00000100150	DEPDC5	22	32226472	A	C	0.486083	0.0150836	0.0142324	0.4299995	0.149032	0.00797382	5.95E-78	0.10121	0.0956521	0.2900064	0.1476099
ENSG00000100151	PICK1	22	38462013	T	C	0.318091	-0.0143679	0.0153206	0.4400003	-0.580224	0.00803131	0	0.0247627	0.0264068	0.3483797	0.03340558
ENSG00000100154	TTC28	22	28724928	C	T	0.112326	-0.00750269	0.0221436	0.6499995	0.251198	0.0116249	1.49E-103	-0.0298676	0.0881628	0.7347769	0.9600354
ENSG00000100162	CENPM	22	42338946	A	G	0.266402	-0.0220853	0.0158921	0.07100027	-0.137447	0.0089262	1.68E-53	0.160682	0.116093	0.1663347	0.1530513
ENSG00000100167	SEPTIN3	22	42383250	T	C	0.00795229	-0.00267187	0.066379	0.7400005	0.348873	0.0441366	2.69E-15	-0.00765857	0.190269	0.9678928	0.3667016
ENSG00000100181	NA	22	17131149	A	G	0.39165	0.00597456	0.0152841	0.7099994	0.22441	0.0111761	1.12E-89	0.0266234	0.0681209	0.6959256	0.3198524
ENSG00000100197	CYP2D6	22	42524704	T	C	0.462227	-0.0138451	0.0142352	0.3400001	-0.299306	0.00854005	4.29E-269	0.0462574	0.047579	0.3309402	0.2827351
ENSG00000100201	DDX17	22	38891555	T	C	0.304175	0.00688867	0.0155633	0.84	0.230509	0.00846148	2.06E-163	0.0298846	0.0675259	0.6580809	0.1734227
ENSG00000100207	TCF20	22	42647820	C	T	0.280318	0.0223808	0.015533	0.1499999	-0.084733	0.00892377	2.20E-21	-0.264133	0.185416	0.1542882	0.1150692
ENSG00000100209	HSCB	22	29145761	G	A	0.149105	-0.0114036	0.0197582	0.4700002	0.0785753	0.0114645	7.19E-12	-0.14513	0.252346	0.5652086	0.3962772
ENSG00000100211	CBY1	22	39061250	G	A	0.0178926	0.0671979	0.0490821	0.1800002	-0.228081	0.0261459	2.70E-18	-0.294623	0.21783	0.1762041	0.8648881
ENSG00000100216	TOMM22	22	39079385	C	A	0.295229	0.011286	0.0157	0.64	-0.115558	0.00899735	9.34E-38	-0.0976649	0.136075	0.4729237	0.4578825
ENSG00000100218	RSPH14	22	23444400	A	C	0.236581	-0.00646515	0.0162249	0.6999999	-0.114647	0.00970628	3.40E-32	0.0563919	0.141601	0.6904501	0.8427797
ENSG00000100219	XBP1	22	29193564	C	A	0.112326	-0.00592524	0.022206	0.7099994	0.572626	0.0113456	0	-0.0103475	0.0387798	0.7896025	0.5635191
ENSG00000100220	RTCB	22	32795905	C	T	0.148111	0.0218995	0.0180715	0.3400001	-0.164882	0.0110127	1.12E-50	-0.132819	0.109961	0.2270953	0.8313564
ENSG00000100221	JOSD1	22	39089554	A	T	0.293241	0.012665	0.0157085	0.5700002	-0.249097	0.00890719	4.24E-172	-0.0508437	0.0630881	0.420291	0.6685779
ENSG00000100225	FBXO7	22	32882740	T	G	0.498012	-0.0141009	0.0141911	0.5300002	0.120637	0.00791633	1.95E-52	-0.116887	0.117885	0.3214228	0.8638993
ENSG00000100227	POLDIP3	22	42995347	C	T	0.183897	0.00329804	0.0192343	0.7899998	0.165845	0.0106088	4.35E-55	0.0198863	0.115985	0.863865	0.326675
ENSG00000100228	RAB36	22	23497025	C	T	0.239563	-0.00471305	0.016207	0.7800007	-0.112295	0.00886628	9.20E-37	0.0419703	0.144363	0.7712603	0.4955929
ENSG00000100234	TIMP3	22	33228358	A	G	0.27833	0.0245997	0.0158987	0.1100001	0.0851361	0.00885857	7.21E-22	0.288945	0.189149	0.1266103	0.103731
ENSG00000100239	PPP6R2	22	50832623	T	C	0.266402	0.0130499	0.0157488	0.32	-0.112192	0.010409	4.35E-27	-0.116317	0.140788	0.4086962	0.3006042
ENSG00000100241	SBF1	22	50899319	A	G	0.083499	0.00135986	0.0261336	0.9199999	0.374159	0.0280353	1.25E-40	0.00363444	0.0698467	0.9585012	0.2737722
ENSG00000100242	SUN2	22	39160439	A	G	0.349901	0.00996568	0.0150767	0.7199992	-0.426503	0.00842246	0	-0.023366	0.0353526	0.5086492	0.3364978

ENSG00000100243	CYB5R3	22	43029710	A	G	0.112326	0.0339518	0.0245603	0.16	-0.462358	0.011758	0	-0.0734319	0.0531525	0.1671155	0.3600541
ENSG00000100246	DNAL4	22	39182358	T	G	0.0805169	0.0299545	0.0243541	0.1800002	-0.264812	0.0153764	1.82E-66	-0.113116	0.0922018	0.2198856	0.05926712
ENSG00000100258	LMF2	22	50943755	G	A	0.418489	0.0014481	0.0144879	0.8800001	-0.156614	0.0103745	1.72E-51	-0.00924629	0.092509	0.9203839	0.2767558
ENSG00000100263	RHBDD3	22	29660019	T	C	0.0129225	-0.0051354	0.067961	0.6999999	-0.525428	0.0443811	2.45E-32	0.00977375	0.129347	0.9397673	0.6888106
ENSG00000100266	PACSIN2	22	43321284	A	T	0.445328	-0.00958551	0.0142679	0.5	-0.168465	0.00791898	1.99E-100	0.056899	0.0847357	0.5019093	0.9932727
ENSG00000100271	TTLL1	22	43460478	T	C	0.478131	-0.0150789	0.0142093	0.2599998	-0.115311	0.00795663	1.35E-47	0.130768	0.123556	0.2898887	0.2187985
ENSG00000100276	RASL10A	22	29710333	T	C	0.360835	0.000675895	0.0149073	0.81	-0.0777755	0.00834752	1.19E-20	-0.00869033	0.191673	0.9638368	0.6699502
ENSG00000100280	AP1B1	22	29771418	C	T	0.282306	0.00226752	0.0158427	0.9299999	-0.176094	0.00861321	6.71E-93	-0.0128768	0.0899697	0.8861926	0.6975724
ENSG00000100281	HMGXB4	22	35672622	T	C	0.341948	-0.00999071	0.0148747	0.4500005	-0.172245	0.00902533	3.39E-81	0.058003	0.0864114	0.5020662	0.5737302
ENSG00000100284	TOM1	22	35719626	C	A	0.357853	-0.0060331	0.0148395	0.64	0.639717	0.00737588	0	-0.0094309	0.0231972	0.6843368	0.7949648
ENSG00000100285	NEFH	22	29881799	T	C	0.148111	-0.00984742	0.0189139	0.7700005	0.357743	0.011019	3.21E-231	-0.0275266	0.0528769	0.602661	0.8248295
ENSG00000100288	CHKB	22	51028631	C	T	0.474155	0.0150153	0.0141994	0.2200002	-0.176903	0.00930811	1.54E-80	-0.084879	0.080391	0.2910472	0.5298972
ENSG00000100290	BIK	22	43516236	A	C	0.267396	0.0103536	0.0162063	0.5199996	-0.146138	0.00980211	2.89E-50	-0.0708482	0.110999	0.5232934	0.7660198
ENSG00000100292	HMOX1	22	35783280	T	G	0.136183	0.0267098	0.0214407	0.2599998	-0.352072	0.0113321	6.42E-212	-0.0758645	0.0609475	0.2132229	0.5331037
ENSG00000100296	THOC5	22	29926536	G	A	0.242545	0.00631977	0.0168345	0.6899999	-0.223084	0.00965437	3.94E-118	-0.0283292	0.0754727	0.7073955	0.825024
ENSG00000100297	MCM5	22	35808739	T	G	0.136183	0.0267098	0.0214407	0.2599998	-0.612096	0.016361	2.49E-306	-0.0436366	0.0350477	0.2131089	0.3320958
ENSG00000100298	APOBEC3H	22	39496650	A	T	0.109344	-0.00888577	0.0218239	0.6999999	-0.621297	0.0119002	0	0.014302	0.0351274	0.6839012	0.4265633
ENSG00000100299	ARSA	22	51065026	C	T	0.0815109	-0.04733	0.026345	0.04799986	-0.903755	0.0150632	0	0.0523704	0.0291637	0.07253572	0.8511135
ENSG00000100300	TSPO	22	43553384	T	C	0.302187	-0.0222592	0.0156866	0.1800002	-0.29096	0.00963647	2.87E-200	0.0765027	0.0539728	0.1563572	0.6295942
ENSG00000100304	TTLL12	22	43572883	T	C	0.271372	-0.00294668	0.0162135	0.7400005	0.402234	0.00990017	0	-0.00732579	0.040309	0.8557863	0.2999113
ENSG00000100307	CBX7	22	39532425	T	C	0.422465	-0.00321259	0.0143999	0.8200001	-0.116679	0.00805774	1.61E-47	0.0275335	0.123429	0.8234798	0.2440886
ENSG00000100311	PDGFB	22	39630060	C	T	0.38171	0.0285272	0.0148188	0.08500021	-0.356257	0.00866145	0	-0.0800749	0.0416414	0.05448468	0.2026319
ENSG00000100316	RPL3	22	39712640	T	C	0.493042	-0.016257	0.0141892	0.25	0.142342	0.0109056	6.17E-39	-0.114211	0.100067	0.2537277	0.2808336
ENSG00000100319	ZMAT5	22	30144972	C	G	0.460239	0.00957292	0.0142691	0.5099998	-0.092942	0.00875803	2.62E-26	-0.102999	0.153833	0.503146	0.2489818
ENSG00000100320	RBFOX2	22	36279628	A	G	0.117296	0.0265204	0.0209746	0.2399999	-0.0892451	0.0121283	1.86E-13	-0.297163	0.238467	0.2127121	0.4100655
ENSG00000100321	SYNGR1	22	39763761	A	T	0.301193	0.0232564	0.015422	0.08300036	0.592433	0.00794075	0	0.0392558	0.026037	0.131633	0.4935741
ENSG00000100324	TAB1	22	39814405	C	T	0.475149	0.0115916	0.0142078	0.4600002	-0.0492604	0.00803596	8.79E-10	-0.235313	0.290966	0.4186703	0.3555352
ENSG00000100325	ASCC2	22	30209434	A	C	0.460239	0.0100143	0.0142774	0.4899999	0.264194	0.0078719	6.08E-247	0.037905	0.0540531	0.4831432	0.6975058
ENSG00000100330	MTMR3	22	30352999	C	T	0.152087	0.00757895	0.0192032	0.7400005	0.478519	0.0101282	0	0.0158383	0.0401319	0.6930957	0.2680764
ENSG00000100335	MIEF1	22	39904787	T	C	0.0656064	0.0950226	0.0305238	4.00E-04	-0.182745	0.0224073	3.47E-16	-0.519973	0.178784	0.003632959	0.7020402
ENSG00000100336	APOL4	22	36593029	T	G	0.270378	-0.000145114	0.0158389	0.9299999	0.135686	0.00873723	2.19E-54	-0.00106949	0.116732	0.99269	0.3257329
ENSG00000100342	APOL1	22	36656316	T	A	0.233598	-0.0256013	0.0177061	0.1900002	-0.155558	0.00972991	1.56E-57	0.164577	0.114288	0.1498601	0.6509075
ENSG00000100345	MYH9	22	36730695	G	C	0.173956	-0.0283685	0.0201121	0.08300036	-0.250631	0.010287	4.12E-131	0.113188	0.0803801	0.1590834	0.1850417
ENSG00000100346	CACNA1I	22	40026250	G	T	0.243539	0.0116887	0.0172412	0.4899999	-0.158295	0.0104269	4.70E-52	-0.0738413	0.109027	0.4982304	0.00665639
ENSG00000100347	SAMM50	22	44378856	T	G	0.130219	0.000741357	0.0229799	0.95	-0.844527	0.0112611	0	-0.000877837	0.0272104	0.9742638	0.3257142
ENSG00000100348	TXN2	22	36870580	C	T	0.384692	-0.0247352	0.0148015	0.1199999	0.110941	0.00814558	3.05E-42	-0.222958	0.134418	0.0971785	0.1219942
ENSG00000100350	FOXRED2	22	36893192	T	C	0.111332	-0.0361305	0.0232407	0.14	0.77147	0.0117047	0	-0.0468333	0.0301336	0.1201392	0.217088
ENSG00000100351	GRAP2	22	40333405	T	C	0.422465	-0.00451774	0.0143523	0.7499995	-0.0969081	0.00802937	1.54E-33	0.0466188	0.148153	0.753014	0.9333563
ENSG00000100353	EIF3D	22	36916190	C	G	0.136183	-0.00228744	0.0189165	0.9599999	0.215102	0.0110066	4.73E-85	-0.0106342	0.0879435	0.9037539	0.07754881
ENSG00000100354	TNRC6B	22	40586316	T	C	0.17495	0.00200887	0.0181483	0.5199996	0.0612038	0.0108702	1.80E-08	0.0328226	0.29658	0.9118776	0.9510678
ENSG00000100359	SGSM3	22	40786358	G	T	0.0377734	0.0328845	0.0308097	0.2099999	0.178717	0.0183495	2.04E-22	0.184004	0.173426	0.2886942	0.8104889
ENSG00000100360	IFT27	22	37163273	A	G	0.446322	-0.0109028	0.0144409	0.33	0.419084	0.0114676	2.15E-292	-0.0260158	0.0344656	0.4503489	0.08154196
ENSG00000100362	PVALB	22	37206125	C	T	0.331014	0.0087594	0.0153571	0.5300002	-0.607787	0.0121345	0	-0.014412	0.0252689	0.5684443	0.5112911
ENSG00000100364	KIAA0930	22	45612382	C	T	0.233598	-0.0275896	0.0162248	0.0530005	0.233106	0.0102172	3.25E-115	-0.118356	0.0697957	0.08993228	0.924224
ENSG00000100365	NCF4	22	37265543	G	T	0.142147	-0.0180328	0.0187516	0.5	-0.104657	0.0109885	1.66E-21	0.172304	0.180083	0.3386671	0.4322547
ENSG00000100368	CSF2RB	22	37323080	A	G	0.305169	-0.0213979	0.0157865	0.14	0.144344	0.00876597	6.40E-61	-0.148242	0.109737	0.1767318	0.3518673
ENSG00000100372	SLC25A17	22	41190518	G	A	0.478131	-0.0121234	0.0142081	0.6800001	0.131889	0.00796288	1.29E-61	-0.0919211	0.10787	0.3941343	0.6245515
ENSG00000100373	UPK3A	22	45686309	A	G	0.303181	0.00858782	0.0157804	0.5199996	-0.628477	0.0131013	0	-0.0136645	0.0251106	0.5863224	0.2286841
ENSG00000100376	FAM118A	22	45721342	G	A	0.134195	0.00231549	0.0215716	0.8200001	1.37933	0.00852233	0	0.00167871	0.0156392	0.9145195	0.6775889
ENSG00000100380	ST13	22	41236782	T	C	0.464215	-0.0138569	0.0142343	0.6100002	0.162561	0.0132086	8.28E-35	-0.0852413	0.0878364	0.3318195	0.3862724

ENSG00000100385	IL2RB	22	37546486	A	G	0.406561	-0.025542	0.0144012	0.07900053	-0.0682489	0.00902572	3.98E-14	0.374248	0.216737	0.08421447	0.3482729
ENSG00000100393	EP300	22	41531935	T	G	0.304175	0.015287	0.0157002	0.2	0.245407	0.00886317	9.63E-169	0.0622924	0.0640157	0.3305134	0.00024355
ENSG00000100395	L3MBTL2	22	41614242	T	G	0.366799	-0.0360878	0.0147139	0.01099993	-0.301203	0.00842847	1.08E-279	0.119812	0.0489653	0.01440999	0.5918515
ENSG00000100399	CHADL	22	41631227	C	T	0.0228628	-0.0705368	0.0456611	0.1299999	0.625787	0.0727254	7.65E-18	-0.112717	0.0741324	0.12839	0.4043708
ENSG00000100401	RANGAP1	22	41661935	G	A	0.435388	-0.0398147	0.0143085	0.002399993	0.10472	0.00795842	1.52E-39	-0.380201	0.139657	0.006481254	0.3231485
ENSG00000100410	PHF5A	22	41860225	C	T	0.222664	0.0400702	0.0176274	0.02900013	-0.157844	0.0145454	1.96E-27	-0.253859	0.1141	0.0260888	0.08926898
ENSG00000100413	POLR3H	22	41931209	C	T	0.296223	-0.0071324	0.0157497	0.56	0.0764418	0.009718	3.66E-15	-0.093305	0.206376	0.6511892	0.3102075
ENSG00000100416	TRMU	22	46740004	T	C	0.147117	0.0129836	0.0207201	0.4899999	0.0962317	0.0118766	5.38E-16	0.13492	0.215958	0.5321331	0.5860029
ENSG00000100417	PMM1	22	41979396	C	T	0.209742	0.040891	0.0180331	0.02900013	-0.306427	0.0101885	1.00E-198	-0.133444	0.0590165	0.02375089	0.2245544
ENSG00000100418	DESI1	22	42005566	T	C	0.416501	-0.0102722	0.0142717	0.4299995	-0.16972	0.00807551	4.62E-98	0.0605245	0.0841391	0.4719325	0.02219834
ENSG00000100422	CERK	22	47107233	C	A	0.50994	-0.010702	0.014199	0.4	0.348847	0.00874357	0	-0.0306782	0.04071	0.4511011	0.4638346
ENSG00000100425	BRD1	22	50194045	A	G	0.054672	-0.0463341	0.0300861	0.09400046	-0.284474	0.0168947	1.29E-63	0.162877	0.106202	0.1251161	0.314579
ENSG00000100426	ZBED4	22	50264790	T	C	0.141153	-0.0125774	0.0211989	0.5199996	0.278661	0.0116828	9.61E-126	-0.0451352	0.0760977	0.5531002	0.5417082
ENSG00000100427	MLC1	22	50511075	C	T	0.269384	-0.0113963	0.0155479	0.4	-0.180849	0.00914607	5.05E-87	0.0630156	0.0860308	0.4638775	0.2540261
ENSG00000100429	HDAC10	22	50686723	T	C	0.264414	-0.0127825	0.0157081	0.4100001	0.195032	0.0200977	2.89E-22	-0.0655406	0.0808239	0.4174196	0.5803044
ENSG00000100439	ABHD4	14	23074205	C	T	0.45328	-0.013212	0.0142071	0.4199997	-0.117569	0.0081311	2.19E-47	0.112376	0.12109	0.3533868	0.1778387
ENSG00000100441	KHNYN	14	24904516	C	T	0.137177	0.0048307	0.0206088	0.6700003	0.783806	0.0125491	0	0.00616313	0.0262934	0.8146758	0.7642134
ENSG00000100445	SDR39U1	14	24910541	T	G	0.0954274	-0.0381914	0.0239857	0.0649995	-0.414496	0.0154997	1.52E-157	0.0921393	0.0579696	0.1119606	0.09980544
ENSG00000100448	CTSG	14	25044097	A	G	0.11332	0.00878751	0.0227774	0.7899998	0.722807	0.0120277	0	0.0121575	0.0315131	0.699651	0.1717599
ENSG00000100450	GZMH	14	25077295	T	C	0.489066	-0.00910759	0.0142074	0.5400003	0.128142	0.00793487	1.15E-58	-0.0710743	0.11096	0.5218209	0.9209228
ENSG00000100453	GZMB	14	25101816	C	T	0.245527	-0.0118505	0.0170106	0.3900004	-0.44272	0.0100222	0	0.0267675	0.0384277	0.4860737	0.1211022
ENSG00000100461	RBM23	14	23379123	T	C	0.262425	0.0109056	0.0162278	0.5099998	-0.347767	0.00883835	0	-0.0313589	0.0466697	0.5016257	0.2332108
ENSG00000100462	PRMT5	14	23394257	G	A	0.224652	-0.0144064	0.0172109	0.64	-0.551549	0.00957272	0	0.0261199	0.0312079	0.4026136	0.3417107
ENSG00000100473	COCH	14	31353995	A	G	0.198807	-0.00527505	0.0178746	0.83	0.418687	0.00972944	0	-0.012599	0.0426931	0.7679119	0.9089764
ENSG00000100478	AP4S1	14	31528565	C	G	0.263419	0.00964652	0.0159452	0.6700003	0.094511	0.0100739	6.49E-21	0.102068	0.169063	0.5460261	0.8911748
ENSG00000100479	POLE2	14	50132706	T	C	0.225646	0.00558213	0.0168428	0.58	-0.0932127	0.010069	2.09E-20	-0.0598859	0.180808	0.7404835	0.9506192
ENSG00000100483	VCPKMT	14	50579334	T	A	0.145129	0.0058825	0.0191574	0.6899999	-0.501015	0.0113285	0	-0.0117412	0.0382381	0.7588022	0.5345648
ENSG00000100485	SOS2	14	50641061	G	A	0.243539	-0.00218151	0.0160852	0.9599999	0.187736	0.00933834	6.84E-90	-0.0116201	0.0856818	0.8921224	0.4205166
ENSG00000100490	CDKL1	14	50839744	C	T	0.524851	-0.00441343	0.0142251	0.7600007	-0.472741	0.011161	0	0.00933583	0.0300915	0.7563722	0.8110216
ENSG00000100503	NIN	14	51242160	G	C	0.50994	-0.00983362	0.0142055	0.4500005	0.161983	0.00791318	3.99E-93	-0.0607078	0.0877477	0.4890348	0.8450579
ENSG00000100504	PYGL	14	51368031	C	T	0.222664	0.0313627	0.0172445	0.06199976	0.259629	0.0094113	1.59E-167	0.120798	0.0665638	0.06955958	0.7100156
ENSG00000100505	TRIM9	14	51502379	C	T	0.026839	0.0103595	0.0362349	0.5	0.575945	0.021924	4.21E-152	0.017987	0.0629175	0.7749689	0.5226471
ENSG00000100519	PSMC6	14	53184597	G	T	0.388668	0.0208442	0.0145964	0.14	-0.258134	0.0152161	1.50E-64	-0.0807494	0.0567457	0.1547349	0.4825031
ENSG00000100526	CDKN3	14	54875251	G	A	0.282306	0.0280638	0.0158053	0.04600023	-0.115918	0.0089429	2.01E-38	-0.242101	0.137622	0.07854935	0.346318
ENSG00000100528	CNIH1	14	54900901	G	A	0.152087	0.0138434	0.0195734	0.6300007	0.197897	0.0117089	4.39E-64	0.0699526	0.0989936	0.4797919	0.2871458
ENSG00000100554	ATP6V1D	14	67794035	T	C	0.0715706	0.000124922	0.0302099	0.6200004	0.596388	0.0140156	0	0.000209464	0.0506548	0.9967006	0.1382956
ENSG00000100558	PLEK2	14	67866308	A	G	0.027833	-0.057827	0.0402588	0.16	-0.257638	0.0240536	9.04E-27	0.22445	0.15766	0.1545514	0.2712951
ENSG00000100564	PIGH	14	68057838	A	G	0.0964215	0.00617466	0.024895	0.9299999	0.157366	0.0149171	5.11E-26	0.0392376	0.158242	0.8041657	0.4569146
ENSG00000100568	VTI1B	14	68127670	A	G	0.116302	0.0174503	0.0204454	0.4600002	0.180847	0.0174106	2.84E-25	0.0964921	0.113435	0.3949691	0.7664387
ENSG00000100575	TIMM9	14	58884772	T	G	0.0705765	-0.0211814	0.0271734	0.3400001	-0.10144	0.0148556	8.59E-12	0.208808	0.269617	0.4386585	0.6297776
ENSG00000100577	GSTZ1	14	77792583	T	C	0.287276	0.00489097	0.0154644	0.6100002	0.320482	0.00857257	6.95E-306	0.0152613	0.0482553	0.751804	0.07847702
ENSG00000100578	KIAA0586	14	58954659	C	T	0.507952	-0.00543917	0.0142372	0.8499999	-0.131889	0.00810833	1.73E-59	0.0412407	0.107979	0.7025103	0.6728318
ENSG00000100580	TMED8	14	77822408	C	T	0.497018	0.0174123	0.0142318	0.2999998	0.129453	0.00872525	8.50E-50	0.134507	0.110311	0.2227153	0.2801286
ENSG00000100583	SAMD15	14	77850436	A	G	0.497018	0.0178604	0.0142343	0.28	0.0826827	0.00903636	5.69E-20	0.216011	0.173767	0.2138273	0.4128581
ENSG00000100591	AHSA1	14	77930015	A	C	0.121272	0.0453623	0.022479	0.05699936	0.0840404	0.0117071	7.04E-13	0.539768	0.277846	0.05205411	0.4145085
ENSG00000100592	DAAM1	14	59746743	A	C	0.17992	-0.00107741	0.01898	0.8499999	0.442082	0.0101672	0	-0.00243713	0.0429332	0.954732	0.305575
ENSG00000100596	SPTLC2	14	78027728	C	G	0.427435	-0.00742131	0.0143364	0.5300002	0.370514	0.0077674	0	-0.0200298	0.0386955	0.6047202	0.06778643
ENSG00000100599	RIN3	14	93067728	A	C	0.459245	-0.00279622	0.0143098	0.83	0.185582	0.00791827	1.79E-121	-0.0150673	0.0771103	0.8450805	0.5732793
ENSG00000100600	LGMN	14	93192599	A	C	0.392644	0.0174685	0.0144969	0.28	-0.195024	0.00809754	3.64E-128	-0.0895709	0.0744268	0.228792	0.9831608
ENSG00000100601	ALKBH1	14	78156555	G	A	0.270378	-0.0265221	0.0160934	0.1100001	0.236826	0.00891741	2.09E-155	-0.11199	0.0680853	0.1000013	0.9265619

ENSG00000100603	SNW1	14	78205746	T	C	0.0467197	0.046259	0.0300735	0.04900044	0.473501	0.0189862	2.80E-137	0.0976957	0.0636338	0.1247149	0.8745557
ENSG00000100605	ITPK1	14	93492962	A	G	0.0258449	0.0350088	0.0563424	0.5999997	-0.653606	0.0292643	1.70E-110	-0.0535625	0.0862357	0.5345222	0.06629228
ENSG00000100612	DHRS7	14	60623706	A	G	0.111332	0.00524196	0.0219787	0.7300002	-0.364199	0.0118883	4.17E-206	-0.0143931	0.0603499	0.8114975	0.04658609
ENSG00000100614	PPM1A	14	60739137	T	C	0.331014	-0.0065092	0.0148249	0.5500004	-0.0605816	0.00904716	2.14E-11	0.107445	0.245235	0.6612911	0.3927406
ENSG00000100628	ASB2	14	94421818	C	T	0.196819	0.0171827	0.0175208	0.1800002	0.0801448	0.0104426	1.66E-14	0.214396	0.220392	0.3306562	0.4194672
ENSG00000100629	CEP128	14	81184595	T	A	0.406561	-0.0428738	0.0143433	0.00259998	-0.319128	0.00888221	1.08E-282	0.134347	0.0451005	0.002893553	0.08046804
ENSG00000100632	ERH	14	69856096	G	C	0.0725646	0.0441325	0.0287541	0.09099971	0.127497	0.015918	1.15E-15	0.346146	0.229632	0.1317085	0.8699986
ENSG00000100644	HIF1A	14	62188603	C	G	0.055666	0.0075418	0.0364127	0.9	-0.305673	0.01933	2.52E-56	-0.0246728	0.119133	0.83593	0.9317976
ENSG00000100647	SUSD6	14	70130086	A	C	0.0208748	-0.016316	0.0665547	0.7800007	-0.751923	0.0366106	9.77E-94	0.021699	0.088519	0.8063525	0.1469366
ENSG00000100664	EIF5	14	103805621	G	A	0.282306	0.0108267	0.0158099	0.6499995	0.516251	0.00845058	0	0.0209718	0.0306264	0.4934941	0.1078989
ENSG00000100678	SLC8A3	14	70583360	A	G	0.0447316	-0.0121045	0.035649	0.7700005	-0.540749	0.0238592	1.01E-113	0.0223847	0.0659327	0.7342266	0.5683926
ENSG00000100697	DICER1	14	95588456	A	C	0.336978	-0.012951	0.0150282	0.3400001	-0.222784	0.00851897	9.48E-151	0.0581326	0.0674931	0.3890661	0.7737817
ENSG00000100711	ZFYVE21	14	104191036	T	G	0.264414	0.031619	0.0162822	0.04200007	-0.0744113	0.00938162	2.16E-15	-0.424922	0.225277	0.05926464	0.5302792
ENSG00000100714	MTHFD1	14	64890737	T	C	0.464215	-0.0115328	0.0143131	0.2399999	-0.234422	0.0117709	2.99E-88	0.0491968	0.061107	0.4207662	0.758215
ENSG00000100721	TCL1A	14	96178418	A	G	0.0467197	-0.0400935	0.0352243	0.2700001	-0.534224	0.0203343	4.01E-152	0.07505	0.0659974	0.2554683	0.8645833
ENSG00000100722	ZC3H14	14	89054553	G	A	0.491054	0.00670255	0.0142255	0.4799997	0.0645935	0.00895838	5.58E-13	0.103765	0.220701	0.6382395	0.468228
ENSG00000100726	TELO2	16	1551901	G	A	0.0656064	-0.00218646	0.0275477	0.8	-0.351468	0.0171202	1.18E-93	0.00622094	0.0783796	0.9367388	0.3703011
ENSG00000100731	PCNX1	14	71478110	G	T	0.170974	-0.00570458	0.0193315	0.4400003	-0.456845	0.0100254	0	0.0124869	0.0423161	0.767928	0.3829044
ENSG00000100744	GSKIP	14	96841719	C	T	0.427435	-0.00316785	0.0143821	0.83	0.441073	0.00776626	0	-0.00718215	0.0326074	0.8256673	0.4504923
ENSG00000100749	VRK1	14	97330850	A	G	0.499006	-0.0069577	0.014208	0.3599996	-0.243633	0.0116736	9.95E-97	0.0285582	0.0583334	0.6244395	0.6175556
ENSG00000100767	PAPLN	14	73722776	A	C	0.083499	-0.0120268	0.0239058	0.6999999	-0.22606	0.0139787	7.97E-59	0.0532018	0.105801	0.6150711	0.8362439
ENSG00000100784	RPS6KA5	14	91431889	C	T	0.0467197	-0.0107431	0.0370873	0.6800001	0.63158	0.0208754	4.52E-201	-0.0170099	0.0587241	0.7720784	0.5293726
ENSG00000100802	C14orf93	14	23467742	T	G	0.162028	0.00304259	0.0185448	0.89	0.100084	0.0106348	4.91E-21	0.0304004	0.185321	0.869698	0.8263199
ENSG00000100804	PSMB5	14	23495095	A	G	0.0626243	-0.0250473	0.0266694	0.4	-0.345282	0.016121	9.04E-102	0.0725416	0.0773137	0.3481027	0.2979344
ENSG00000100813	ACIN1	14	23546298	C	A	0.0725646	0.0255885	0.0250502	0.35	-0.140424	0.0145159	3.90E-22	-0.182223	0.179381	0.309705	0.4524712
ENSG00000100814	CCNB1IP1	14	20790499	C	T	0.50497	0.0140627	0.0142435	0.2399999	0.29429	0.00799349	1.02E-296	0.0477852	0.0484169	0.3236663	0.3651912
ENSG00000100815	TRIP11	14	92469788	C	A	0.223658	0.0128216	0.0165141	0.4500005	0.279395	0.00910899	1.33E-206	0.0458906	0.0591256	0.4376577	0.0862961
ENSG00000100823	APEX1	14	20924638	A	G	0.101392	0.0256647	0.0225981	0.1800002	-0.148768	0.0133449	7.32E-29	-0.172514	0.152687	0.2585379	0.6478687
ENSG00000100836	PABPN1	14	23792946	A	G	0.0616302	-0.0118066	0.0274511	0.6100002	-0.104407	0.0146224	9.32E-13	0.113083	0.263401	0.6676922	0.7892595
ENSG00000100852	ARHGAP5	14	32587127	C	A	0.373757	0.0100478	0.0145386	0.5199996	-0.194092	0.00818479	2.60E-124	-0.0517681	0.0749374	0.4896795	0.3839752
ENSG00000100865	CINP	14	102819104	T	C	0.298211	0.00603585	0.0157371	0.6600001	0.159027	0.00877806	2.37E-73	0.0379549	0.0989809	0.7013811	0.5920261
ENSG00000100883	SRP54	14	35474968	G	A	0.436382	0.00591326	0.0145026	0.6800001	-0.240509	0.00793735	1.12E-201	-0.0245864	0.0603051	0.6834932	0.7936018
ENSG00000100889	PCK2	14	24571534	G	A	0.471173	-0.00848061	0.014184	0.5400003	-0.385682	0.00814156	0	0.0219886	0.0367793	0.5499386	0.4039706
ENSG00000100897	DCAF11	14	24588927	G	C	0.0467197	-0.00043412	0.0387211	0.64	-0.136933	0.0213026	1.29E-10	0.00317031	0.282775	0.9910548	0.6491931
ENSG00000100902	PSMA6	14	35767269	C	T	0.100398	-0.00331806	0.0214761	0.8	0.169806	0.0147133	8.20E-31	-0.0195403	0.126486	0.8772263	0.339185
ENSG00000100906	NFKBIA	14	35872336	T	C	0.151093	-0.0334822	0.0185231	0.04600023	0.413327	0.0150032	4.51E-167	-0.0810065	0.0449109	0.07127628	0.2146998
ENSG00000100908	EMC9	14	24609485	C	T	0.275348	-0.0061748	0.0164315	0.8800001	-0.269671	0.00991545	7.05E-163	0.0228976	0.0609376	0.7070994	0.154586
ENSG00000100918	REC8	14	24645262	T	C	0.263419	0.00194949	0.0154067	0.6300007	0.167575	0.00888853	2.78E-79	0.0116335	0.091941	0.8993105	0.9648262
ENSG00000100926	TM9SF1	14	24670514	A	G	0.143141	0.00250568	0.0188981	0.5700002	0.096938	0.0122769	2.88E-15	0.0258483	0.194978	0.8945332	0.9930434
ENSG00000100938	GMPR2	14	24705038	C	A	0.0616302	-0.0134727	0.0310749	0.4700002	-0.212688	0.0183093	3.40E-31	0.0633449	0.146207	0.6648302	0.09709045
ENSG00000100941	PNN	14	39648404	T	A	0.102386	0.00429196	0.0235047	0.83	0.182698	0.0134169	3.17E-42	0.0234921	0.128665	0.8551247	0.4956624
ENSG00000100949	RABGGTA	14	24737844	C	A	0.153082	0.0025371	0.0190001	0.7700005	-0.147855	0.011044	7.13E-41	-0.0171594	0.128512	0.8937788	0.5994376
ENSG00000100968	NFATC4	14	24841844	G	C	0.15507	0.0164171	0.0186656	0.28	-0.0952107	0.0104902	1.12E-19	-0.172429	0.196963	0.3813363	0.3042157
ENSG00000100979	PLTP	20	44534096	G	C	0.450298	-0.0020343	0.0142574	0.9199999	0.57403	0.00729913	0	-0.00354389	0.0248374	0.8865401	0.8944534
ENSG00000100982	PCIF1	20	44569964	A	G	0.431412	0.00356128	0.0145321	0.83	0.0514732	0.00808589	1.94E-10	0.0691871	0.282533	0.8065481	0.8548081
ENSG00000100983	GSS	20	33529928	G	T	0.00795229	-0.00335287	0.0738061	0.7499995	-0.364292	0.0429853	2.35E-17	0.00920379	0.202604	0.9637666	0.4224004
ENSG00000100985	MMP9	20	44641373	A	G	0.17495	0.0147823	0.0203023	0.3100002	0.238272	0.0110944	2.57E-102	0.0620395	0.0852552	0.4668024	0.7505989
ENSG00000100991	TRPC4AP	20	33635440	C	T	0.32008	0.00508002	0.0145707	0.5199996	0.654538	0.00784014	0	0.00776123	0.0222612	0.7273572	0.7826058
ENSG00000100994	PYGB	20	25253677	G	C	0.457256	0.0116205	0.0141792	0.28	0.74915	0.0067536	0	0.0155116	0.0189276	0.412488	0.3099229
ENSG00000100997	ABHD12	20	25323499	A	G	0.248509	-0.00462419	0.0162413	0.6999999	0.191811	0.00899273	6.03E-101	-0.024108	0.0846809	0.7758797	0.4964433

ENSG00000101000	PROCR	20	33762520	G	T	0.442346	-0.00124056	0.0143821	0.7099994	-0.11743	0.00801887	1.47E-48	0.0105643	0.122476	0.9312631	0.2818074
ENSG00000101003	GINS1	20	25410813	C	T	0.49006	0.00830451	0.01419	0.4700002	0.0503224	0.00832794	1.52E-09	0.165026	0.283301	0.5602225	0.6771894
ENSG00000101004	NINL	20	25499747	T	C	0.157058	0.017247	0.0199508	0.5099998	0.339055	0.0109772	1.77E-209	0.0508679	0.0588655	0.387512	0.3277671
ENSG00000101017	CD40	20	44752706	A	G	0.247515	0.00566044	0.0160218	0.5400003	0.529103	0.00873393	0	0.0106982	0.0302816	0.7238709	0.3315611
ENSG00000101019	UQCC1	20	33945156	G	A	0.377734	-0.00250524	0.0146904	0.58	-0.456025	0.0083381	0	0.00549365	0.0322142	0.8645894	0.3987678
ENSG00000101040	ZMYND8	20	45911713	G	C	0.529821	0.0198574	0.0142609	0.1800002	-0.122124	0.00803857	3.98E-52	-0.162601	0.117264	0.1655563	0.7043206
ENSG00000101052	IFT52	20	42247753	A	G	0.0417495	0.0294083	0.0407892	0.5	-0.715139	0.0309541	4.30E-118	-0.0411225	0.0570645	0.4711361	0.9716099
ENSG00000101057	MYBL2	20	42320445	A	C	0.144135	0.00744825	0.0194408	0.7099994	0.34391	0.010609	1.58E-230	0.0216575	0.0565326	0.7016472	0.9379495
ENSG00000101079	NDRG3	20	35327325	C	T	0.0377734	-0.0645294	0.0358258	0.05999983	-0.54958	0.0228349	5.46E-128	0.117416	0.0653699	0.07246652	0.370463
ENSG00000101082	SLA2	20	35257670	G	A	0.414513	-0.0371744	0.0146249	0.0032	-0.051931	0.00822084	2.67E-10	0.715841	0.303566	0.01836817	0.3980478
ENSG00000101084	RAB5IF	20	35237548	C	T	0.0874751	-0.0410662	0.0246226	0.06699926	-0.23266	0.0150505	6.60E-54	0.176507	0.106445	0.0972766	0.4830115
ENSG00000101096	NFATC2	20	50091331	A	G	0.181909	0.00280325	0.0191183	0.81	0.0605636	0.0104136	6.03E-09	0.046286	0.315773	0.8834636	0.7551316
ENSG00000101104	PABPC1L	20	43563189	C	T	0.365805	-0.00260616	0.0152976	0.7600007	0.414695	0.00984532	0	-0.00628452	0.0368891	0.864725	0.6478659
ENSG00000101109	STK4	20	43651857	C	T	0.168986	-0.0342283	0.0184675	0.06100002	-0.221261	0.0110536	3.90E-89	0.154696	0.0838217	0.06495901	0.8907694
ENSG00000101115	SALL4	20	50409820	A	G	0.176938	0.0261863	0.0189144	0.14	0.190921	0.0118689	3.21E-58	0.137158	0.0994355	0.1677825	0.2389367
ENSG00000101126	ADNP	20	49526705	A	G	0.194831	0.00920142	0.0165818	0.4500005	-0.0597096	0.00934276	1.65E-10	-0.154103	0.278752	0.5803792	0.6925609
ENSG00000101138	CSTF1	20	54973472	C	T	0.122266	0.00925018	0.0218214	0.6100002	-0.189327	0.0124749	5.05E-52	-0.0488582	0.115303	0.6717563	0.3894124
ENSG00000101146	RAE1	20	55940166	A	C	0.0218688	0.0251721	0.0453222	0.5400003	-0.275453	0.0276592	2.31E-23	-0.0913844	0.164793	0.5792083	0.786678
ENSG00000101150	TPD52L2	20	62509770	A	G	0.298211	0.0149541	0.0156422	0.28	-0.154825	0.00886865	3.01E-68	-0.0965869	0.101183	0.3397903	0.2999695
ENSG00000101152	DNAJC5	20	62546951	G	A	0.182903	0.0181518	0.018785	0.28	-0.124858	0.0108104	7.40E-31	-0.14538	0.150977	0.3355836	0.4859579
ENSG00000101158	NELFCD	20	57563225	A	G	0.159046	-0.0173297	0.0206963	0.4299995	-0.706633	0.012352	0	0.0245243	0.0292917	0.4024557	0.6037062
ENSG00000101160	CTSZ	20	57576271	G	A	0.521869	0.00785	0.0142627	0.7300002	0.200401	0.00792349	3.91E-141	0.0391715	0.0711877	0.5821435	0.8807213
ENSG00000101161	PRPF6	20	62638470	C	A	0.172962	-0.00860101	0.0187765	0.6100002	0.348876	0.0100462	3.06E-264	-0.0246535	0.0538247	0.6469285	0.582994
ENSG00000101162	TUBB1	20	57598009	T	C	0.0477137	0.0786188	0.0332949	0.03099988	-0.103512	0.0167561	6.51E-10	-0.759511	0.344347	0.02740839	0.02029893
ENSG00000101181	MTG2	20	60768354	T	C	0.114314	0.0116239	0.0213898	0.6600001	0.204464	0.0147425	9.76E-44	0.0568506	0.104694	0.5871193	0.9981108
ENSG00000101182	PSMA7	20	60715143	C	T	0.0646123	0.0238119	0.0312474	0.5500004	-0.159225	0.0177473	2.92E-19	-0.149549	0.196954	0.4476671	0.7747489
ENSG00000101187	SLCO4A1	20	61295467	C	T	0.22167	-0.00029883	0.0166825	0.6800001	0.0993101	0.0107504	2.52E-20	-0.00300906	0.167984	0.9857085	0.7787225
ENSG00000101188	NTSR1	20	61367156	A	C	0.321074	-4.44E-05	0.0150461	0.7199992	-0.402399	0.00886379	0	0.000110294	0.037391	0.9976465	0.1304523
ENSG00000101189	MRGBP	20	61429875	A	G	0.0864811	0.0410477	0.0280995	0.1800002	-0.412793	0.0215414	7.57E-82	-0.0994389	0.0682691	0.1452345	0.902231
ENSG00000101190	TCFL5	20	61482791	A	G	0.217694	0.000839471	0.0174806	0.8700001	-0.41378	0.0121153	1.19E-255	-0.00202879	0.0422462	0.9616979	0.2311359
ENSG00000101191	DIDO1	20	61539197	G	A	0.189861	-0.0111064	0.0173571	0.6600001	-0.275654	0.0104554	3.47E-153	0.0402911	0.0629855	0.5223754	0.7296921
ENSG00000101193	GID8	20	61573233	A	G	0.197813	0.00715426	0.0180633	0.9	0.186684	0.0241774	1.15E-14	0.0383229	0.0968861	0.69244	0.7571098
ENSG00000101194	SLC17A9	20	61592000	C	A	0.287276	-0.0110072	0.0153428	0.5700002	0.202458	0.00854874	5.42E-124	-0.0543678	0.0758173	0.4733192	0.3744671
ENSG00000101197	BIRC7	20	61869547	A	C	0.440358	0.00606205	0.014218	0.8	0.334657	0.00891139	1.221496e-308	0.0181142	0.0424881	0.6698623	0.9787224
ENSG00000101199	ARFGAP1	20	61912639	A	G	0.400596	-0.00335954	0.0144809	0.95	-0.108945	0.00926863	6.72E-32	0.0308369	0.132945	0.8165746	0.8071347
ENSG00000101210	EEF1A2	20	62124935	C	T	0.141153	-0.00862385	0.0220685	0.7199992	0.50819	0.0141743	1.66E-281	-0.0169697	0.0434283	0.6959796	0.07740702
ENSG00000101213	PTK6	20	62164250	A	G	0.181909	0.00019768	0.0183866	0.91	-0.130869	0.0120426	1.65E-27	-0.00151052	0.140496	0.9914219	0.3614566
ENSG00000101216	GMEB2	20	62238674	A	G	0.21173	0.0175202	0.0166199	0.2700001	0.19155	0.0106482	2.38E-72	0.0914652	0.086914	0.2926323	0.8655984
ENSG00000101220	ADISSP	20	3741594	A	G	0.0367793	-0.0729876	0.0380221	0.04600023	1.08089	0.0235489	0	-0.0675257	0.0352075	0.05511946	0.3196254
ENSG00000101224	CDC25B	20	3777170	A	G	0.223658	0.0145923	0.0167974	0.5	-0.358069	0.00909811	0	-0.0407528	0.0469225	0.385114	0.02274375
ENSG00000101230	ISM1	20	13241858	G	C	0.132207	-0.0221116	0.0205934	0.2700001	-0.544665	0.0121049	0	0.0405967	0.0378201	0.283084	0.9112013
ENSG00000101236	RNF24	20	3954148	T	G	0.130219	-0.0142668	0.0200055	0.6800001	-0.28578	0.0114824	9.89E-137	0.0499223	0.0700319	0.4759378	0.2295245
ENSG00000101247	NDUFAF5	20	13782331	G	A	0.11829	-0.0172465	0.0202036	0.6300007	0.605741	0.0126132	0	-0.0284717	0.0333588	0.393382	0.3831877
ENSG00000101255	TRIB3	20	369732	C	T	0.442346	0.0196035	0.0147724	0.14	0.42953	0.00770763	0	0.0456394	0.0344018	0.1846214	0.120174
ENSG00000101265	RASSF2	20	4782480	G	A	0.452286	0.000119176	0.0142877	0.84	0.142786	0.00797631	1.15E-71	0.00083465	0.100064	0.9933448	0.05395828
ENSG00000101266	CSNK2A1	20	491799	T	C	0.0765408	-0.0329573	0.0279516	0.35	-0.113251	0.017573	1.16E-10	0.291012	0.250909	0.246117	0.8865964
ENSG00000101276	SLC52A3	20	744927	T	C	0.333996	0.000164669	0.0150974	0.8200001	0.108653	0.00956757	6.89E-30	0.00151555	0.13895	0.9912976	0.2946032
ENSG00000101278	RPS10P5	20	820355	A	G	0.483101	0.0336137	0.0142305	0.0329997	0.216555	0.0205304	5.19E-26	0.15522	0.0673407	0.02116657	0.3336316
ENSG00000101280	ANGPT4	20	875136	C	G	0.459245	0.0294612	0.0144326	0.07100027	0.0726114	0.00894317	4.69E-16	0.405738	0.204951	0.04773881	0.2302423
ENSG00000101282	RSPO4	20	961001	T	C	0.223658	-0.00282035	0.0177949	0.8800001	0.217521	0.0111697	1.82E-84	-0.0129659	0.0818103	0.8740732	0.2369682

ENSG00000101290	CDS2	20	5142982	T	G	0.459245	-0.0120503	0.0142288	0.35	-0.817058	0.0065345	0	0.0147484	0.0174151	0.3970645	0.430652
ENSG00000101294	HM13	20	30129800	T	C	0.157058	0.020881	0.0195913	0.25	0.525308	0.0104697	0	0.03975	0.0373033	0.2866091	0.3066731
ENSG00000101298	SNPH	20	1268466	A	G	0.192843	0.012257	0.0197665	0.4500005	-0.455566	0.0100724	0	-0.026905	0.0433929	0.5352372	0.9031099
ENSG00000101307	SIRPB1	20	1572437	T	C	0.477137	-0.0141787	0.0143656	0.3400001	-0.705548	0.00706058	0	0.020096	0.0203619	0.3236713	0.1699718
ENSG00000101310	SEC23B	20	18515098	A	G	0.126243	0.0246055	0.0215893	0.4	-0.495643	0.0113507	0	-0.0496436	0.043573	0.2545697	0.6368158
ENSG00000101335	MYL9	20	35174057	G	A	0.0497018	-0.0197285	0.0327083	0.4600002	-0.231387	0.0171321	1.44E-41	0.085262	0.141499	0.5467985	0.2014273
ENSG00000101336	HCK	20	30664825	C	A	0.115308	-0.0389741	0.0235846	0.06900014	-0.309654	0.0127377	1.54E-130	0.125863	0.0763401	0.09920534	0.3897369
ENSG00000101337	TM9SF4	20	30726185	C	G	0.408549	0.020403	0.0143808	0.17	0.0886241	0.00797524	1.09E-28	0.230219	0.163585	0.1593258	0.04170226
ENSG00000101342	TLDC2	20	35513581	C	T	0.162028	-0.0159277	0.0194424	0.5500004	0.0885097	0.0121363	3.03E-13	-0.179954	0.221046	0.4155849	0.9938497
ENSG00000101343	CRNKL1	20	20025851	G	C	0.456262	-0.000758394	0.0144677	0.99	0.349219	0.00880465	0	-0.00217169	0.0414288	0.9581942	0.8872327
ENSG00000101347	SAMHD1	20	35549439	G	A	0.349901	-0.00324397	0.0153418	0.9400001	0.182027	0.00860103	2.08E-99	-0.0178214	0.0842873	0.8325471	0.4437288
ENSG00000101350	KIF3B	20	30894140	C	G	0.408549	0.020403	0.0143808	0.17	0.0546914	0.00801222	8.73E-12	0.373057	0.268564	0.1648086	0.09017326
ENSG00000101353	MROH8	20	35768810	T	C	0.238569	-0.0032781	0.0168805	0.8700001	-0.0839266	0.0108086	8.18E-15	0.0390591	0.201197	0.8460711	0.5764182
ENSG00000101361	NOP56	20	2635915	C	G	0.180915	-0.0314652	0.0186242	0.09800089	0.390388	0.0107359	1.64E-289	-0.0805998	0.0477583	0.09147676	0.499503
ENSG00000101363	MANBAL	20	35931852	A	G	0.447316	-0.0240467	0.014348	0.06299992	-0.316335	0.00842676	2.098133e-308	0.0760165	0.0454021	0.09407283	0.009969689
ENSG00000101365	IDH3B	20	2641953	C	T	0.166004	0.0129939	0.0187024	0.7199992	-0.169869	0.0103039	4.64E-61	-0.0764938	0.110197	0.4875846	0.4420223
ENSG00000101367	MAPRE1	20	31422955	G	A	0.159046	0.0153817	0.0183324	0.4899999	0.22976	0.0166899	4.06E-43	0.0669469	0.0799376	0.4023169	0.4017337
ENSG00000101384	JAG1	20	10636470	T	C	0.10338	0.0293806	0.0217805	0.1299999	-0.102278	0.012893	2.14E-15	-0.287263	0.216011	0.1835674	0.3216785
ENSG00000101391	CDK5RAP1	20	31968006	C	T	0.326044	-0.00745482	0.015406	0.95	-0.0710194	0.00871125	3.56E-16	0.104969	0.217309	0.6290669	0.7453127
ENSG00000101400	SNTA1	20	32013729	A	G	0.0298211	0.131951	0.0570924	0.02300011	0.49884	0.0321202	2.16E-54	0.264516	0.115711	0.02225376	0.000338106
ENSG00000101407	TTI1	20	36636639	G	A	0.164016	-0.0184972	0.0192169	0.4100001	0.131692	0.0110921	1.64E-32	-0.140458	0.146402	0.3373569	0.4099546
ENSG00000101413	RPRD1B	20	36691358	A	G	0.164016	-0.019046	0.0192483	0.3900004	0.22893	0.0122593	8.06E-78	-0.0831957	0.0841973	0.3231018	0.8324398
ENSG00000101417	PXMP4	20	32301318	A	C	0.481113	0.00764225	0.0142174	0.3800004	-0.0718816	0.00794939	1.53E-19	-0.106317	0.198138	0.5915573	0.1258629
ENSG00000101421	CHMP4B	20	32420641	G	A	0.475149	0.0255788	0.0142293	0.016	0.363624	0.00769694	0	0.0703442	0.0391603	0.07244446	0.331243
ENSG00000101425	BPI	20	36927229	G	T	0.318091	0.00787456	0.0147584	0.7300002	0.34432	0.00823005	0	0.0228699	0.0428659	0.5936728	0.3869784
ENSG00000101439	CST3	20	23613822	G	A	0.232604	0.0122992	0.0171591	0.3900004	-0.574287	0.00901731	0	-0.0214165	0.0298808	0.4735415	0.4597194
ENSG00000101442	ACTR5	20	37388959	A	G	0.424453	0.0125537	0.0143301	0.3800004	-0.441482	0.00774598	0	-0.0284354	0.0324629	0.3810661	0.1217922
ENSG00000101444	AHCY	20	32883841	C	A	0.0129225	-0.0946763	0.0628961	0.1100001	0.273244	0.0494542	3.29E-08	-0.346489	0.238572	0.1464052	0.7469767
ENSG00000101445	PPP1R16B	20	37493007	G	C	0.402584	0.0142088	0.0147699	0.3700002	-0.136926	0.00822654	3.32E-62	-0.10377	0.108048	0.33685	0.05852999
ENSG00000101447	FAM83D	20	37568329	A	G	0.204771	0.0154007	0.0185281	0.4400003	-0.174321	0.0102725	1.38E-64	-0.088347	0.106415	0.4064187	0.6022161
ENSG00000101452	DHX35	20	37629654	G	T	0.347913	0.0238318	0.0153397	0.1100001	-0.285066	0.00839222	6.64E-253	-0.083601	0.0538673	0.1206669	0.3539465
ENSG00000101457	DNTTIP1	20	44430321	C	A	0.348907	0.00819679	0.0149539	0.6200004	-0.479766	0.00798019	0	-0.017085	0.0311705	0.5836133	0.2292289
ENSG00000101460	MAP1LC3A	20	33141403	G	T	0.11829	0.00358425	0.018561	0.3599996	-0.582393	0.0103008	0	-0.00615435	0.0318704	0.8468764	0.6825192
ENSG00000101464	PIGU	20	33206628	G	A	0.420477	-0.0167851	0.0146919	0.1499999	-0.0885792	0.00828881	1.18E-26	0.189493	0.166807	0.2559567	0.445463
ENSG00000101470	TNNC2	20	44457118	G	T	0.347913	0.00552806	0.0149801	0.7499995	0.368856	0.00808109	0	0.0149871	0.0406137	0.7121167	0.08999678
ENSG00000101473	ACOT8	20	44478202	A	G	0.0586481	-0.0226567	0.0329188	0.3900004	0.174331	0.0181214	6.57E-22	-0.129963	0.189311	0.4923944	0.6947847
ENSG00000101474	APMAP	20	24958588	G	T	0.521869	-0.013445	0.0141874	0.2999998	0.28803	0.00865932	1.35E-242	-0.0466792	0.0492767	0.3434923	0.7427213
ENSG00000101493	ZNF516	18	74138395	G	T	0.16004	-0.0153347	0.0188466	0.4	-0.375363	0.0106988	1.15E-269	0.040853	0.0502225	0.4159658	0.06840877
ENSG00000101544	ADNP2	18	77886160	A	G	0.207753	-0.00816752	0.0173067	0.95	0.0882136	0.00976224	1.62E-19	-0.092588	0.196458	0.6374366	0.6382136
ENSG00000101546	RBFA	18	77800377	C	A	0.316103	0.000537965	0.0154703	0.8499999	0.179251	0.00917295	4.90E-85	0.00300119	0.0863056	0.97226	0.6996099
ENSG00000101557	USP14	18	186502	T	C	0.280318	0.00217457	0.0161455	0.9199999	-0.228938	0.00968147	1.27E-123	-0.00949852	0.0705247	0.892862	0.4462232
ENSG00000101558	VAPA	18	9937008	C	T	0.358847	0.0158277	0.0149497	0.4299995	0.366164	0.00816344	0	0.0432258	0.0408393	0.289857	0.7039749
ENSG00000101574	METTL4	18	2554516	C	G	0.240557	0.0205967	0.0168129	0.25	0.232782	0.0092357	3.57E-140	0.0884806	0.0723112	0.2210998	0.9617046
ENSG00000101577	LPIN2	18	2965152	T	C	0.54175	0.0206194	0.014297	0.08199927	-0.61999	0.00727858	0	-0.0332576	0.0230634	0.149299	0.9932182
ENSG00000101596	SMCHD1	18	2730376	C	T	0.479125	0.0154908	0.0141921	0.3599996	0.0835173	0.00836478	1.78E-23	0.18548	0.170942	0.2779021	0.4532732
ENSG00000101605	MYOM1	18	3143455	A	C	0.207753	0.00259688	0.0179436	0.7800007	-0.711895	0.00953536	0	-0.00364784	0.0252055	0.8849285	0.9377302
ENSG00000101608	MYL12A	18	3251856	A	G	0.132207	1.08E-05	0.021118	0.9699999	0.286016	0.0121709	4.08E-122	3.78E-05	0.073835	0.9995918	0.9608546
ENSG00000101624	CEP76	18	12682304	T	C	0.326044	0.0105719	0.0148854	0.6200004	0.0642026	0.00914359	2.19E-12	0.164665	0.233033	0.4798064	0.1265957
ENSG00000101639	CEP192	18	13058206	T	G	0.380716	0.00792476	0.014531	0.4700002	0.826193	0.00674391	0	0.0095919	0.0175881	0.5855033	0.8220127
ENSG00000101654	RNMT	18	13745608	T	G	0.106362	0.00342646	0.0221261	0.83	0.607122	0.0122693	0	0.00564377	0.0364444	0.8769319	0.4968992

ENSG00000101665	SMAD7	18	46461652	G	A	0.377734	-0.00765087	0.0150439	0.5700002	0.0800883	0.00849888	4.37E-21	-0.0955304	0.188115	0.6115722	0.9565704
ENSG00000101695	RNF125	18	29625755	C	T	0.055666	0.0122094	0.0350549	0.7899998	-0.438417	0.0270347	3.84E-59	-0.0278489	0.0799764	0.7276799	0.2002032
ENSG00000101745	ANKRD12	18	9210716	C	T	0.37674	0.0237764	0.0148045	0.14	0.137366	0.00814128	7.13E-64	0.173088	0.108261	0.1098653	0.08792453
ENSG00000101751	POLI	18	51821705	T	C	0.428429	0.0235833	0.0144639	0.1199999	-0.503506	0.00757696	0	-0.0468382	0.028735	0.1031008	0.3476839
ENSG00000101752	MIB1	18	19367918	A	G	0.333996	0.0120372	0.0151873	0.5400003	-0.188589	0.00830655	4.13E-114	-0.0638278	0.0805804	0.4283019	0.8095895
ENSG00000101773	RBBP8	18	20492337	T	A	0.129225	-0.00690456	0.022635	0.8200001	0.577169	0.0117113	0	-0.0119628	0.039218	0.7603408	0.005806964
ENSG00000101782	RIOK3	18	21049677	G	A	0.279324	0.0164865	0.016952	0.4500005	-0.0982297	0.00910698	4.00E-27	-0.167836	0.173275	0.3327394	0.9167741
ENSG00000102189	EEA1	12	93243760	G	A	0.431412	-0.00214776	0.0144708	0.8200001	0.107405	0.00810741	4.65E-40	-0.0199968	0.134739	0.8820185	0.9130486
ENSG00000102445	RUBCNL	13	46964232	A	C	0.204771	0.00690135	0.0183222	0.8200001	0.506951	0.0109232	0	0.0136135	0.0361432	0.706431	0.5920344
ENSG00000102524	TNFSF13B	13	108932210	G	T	0.0208748	-0.0185529	0.0425721	0.6999999	-0.586547	0.0242818	6.49E-129	0.0316307	0.0725927	0.6630337	0.393781
ENSG00000102531	FNDC3A	13	49666981	A	T	0.026839	0.0612731	0.0504539	0.25	0.493558	0.0267991	9.60E-76	0.124146	0.102447	0.2255868	0.7906663
ENSG00000102543	CDADC1	13	49844832	T	C	0.316103	-0.0120547	0.0153007	0.4899999	-0.160744	0.00860588	7.42E-78	0.0749932	0.0952714	0.4311924	0.5202464
ENSG00000102547	CAB39L	13	49950524	G	C	0.302187	0.014102	0.0155851	0.3400001	0.223114	0.00877275	1.10E-142	0.0632054	0.0698969	0.3658541	0.6218464
ENSG00000102554	KLF5	13	73640395	T	C	0.215706	0.0157518	0.0167768	0.3100002	0.174384	0.00934062	8.78E-78	0.0903284	0.0963278	0.348389	0.9296022
ENSG00000102572	STK24	13	99166324	C	T	0.449304	-0.0131422	0.0142171	0.3599996	0.155478	0.00794836	3.32E-85	-0.0845278	0.0915433	0.3558179	0.6158803
ENSG00000102575	ACP5	19	11687649	C	T	0.345924	0.02946	0.0149917	0.09800089	0.696624	0.00744536	0	0.0422897	0.0215252	0.04945429	0.5402072
ENSG00000102580	DNAJC3	13	96388318	C	T	0.39165	0.0219151	0.0142826	0.1199999	0.0887585	0.00812539	8.89E-28	0.246907	0.162495	0.1286431	0.05996702
ENSG00000102595	UGGT2	13	96579785	C	T	0.0328032	-0.0143453	0.0405518	0.8600001	-0.812801	0.0230621	4.24E-272	0.0176492	0.0498939	0.7235377	0.2741713
ENSG00000102606	ARHGEF7	13	111862495	G	C	0.28827	0.0169484	0.0155334	0.2700001	0.371473	0.00838472	0	0.0456249	0.0418284	0.2753771	0.5721891
ENSG00000102678	FGF9	13	22262079	A	T	0.435388	0.00983838	0.014389	0.4100001	0.0493051	0.00804955	9.06E-10	0.199541	0.293649	0.4968068	0.04370669
ENSG00000102699	PARP4	13	25041006	T	G	0.23161	0.00736346	0.0165681	0.7499995	-0.365915	0.0147246	2.55E-136	-0.0201234	0.0452858	0.6567788	0.6621032
ENSG00000102710	SUPT20H	13	37608649	C	T	0.084493	0.0178816	0.0232941	0.35	-0.572441	0.0147043	0	-0.0312374	0.0407005	0.4427868	0.5172124
ENSG00000102738	MRPS31	13	41324370	A	C	0.307157	0.00752884	0.015515	0.5999997	-0.127511	0.00851187	9.86E-51	-0.0590446	0.121739	0.6276709	0.2532826
ENSG00000102743	SLC25A15	13	41373897	A	G	0.184891	-0.0146295	0.0183999	0.6200004	-0.118294	0.0150704	4.18E-15	0.123671	0.15634	0.4289218	0.8634552
ENSG00000102753	KPNA3	13	50320252	A	C	0.147117	0.0104524	0.0203191	0.6499995	-0.132309	0.0123046	5.75E-27	-0.0789998	0.153748	0.6073744	0.6696613
ENSG00000102755	FLT1	13	28971877	A	G	0.146123	0.000987241	0.0206659	0.8200001	-0.369673	0.0163762	7.84E-113	-0.00267058	0.0559034	0.9618984	0.4866845
ENSG00000102760	RGCC	13	42038356	T	C	0.454274	-0.00609449	0.0142393	0.9400001	-0.195303	0.00882225	1.37E-108	0.0312052	0.0729222	0.6687058	0.7607635
ENSG00000102763	VWA8	13	42338108	T	C	0.148111	-0.011726	0.0213685	0.4199997	-0.198331	0.0125135	1.42E-56	0.0591234	0.107806	0.5834014	0.8189733
ENSG00000102780	DGKH	13	42722445	A	G	0.16004	-0.0265482	0.0192019	0.32	0.0735106	0.010685	5.99E-12	-0.361148	0.266435	0.1752642	0.826552
ENSG00000102781	KATNAL1	13	30829194	T	C	0.446322	0.000330348	0.0143131	0.9599999	-0.14475	0.00795801	6.28E-74	-0.0022822	0.0988817	0.9815864	0.7559731
ENSG00000102786	INTS6	13	51978306	G	A	0.252485	0.00825692	0.0165452	0.59	0.28531	0.00907909	9.22E-217	0.0289401	0.0579975	0.6177869	0.7003054
ENSG00000102796	DHRS12	13	52360211	A	G	0.196819	0.0382051	0.0183783	0.03400008	0.313837	0.0105389	7.29E-195	0.121735	0.0587024	0.03810077	0.8327811
ENSG00000102804	TSC22D1	13	45079469	T	C	0.466203	0.013769	0.0144679	0.2999998	-0.0560297	0.00805557	3.52E-12	-0.245745	0.260624	0.3457282	0.5919716
ENSG00000102805	CLN5	13	77570723	T	C	0.00894632	-0.0564109	0.0560017	0.4299995	-1.1003	0.0327512	1.93E-247	0.0512686	0.0509196	0.3140047	0.4436233
ENSG00000102837	OLFM4	13	53614513	A	G	0.300199	0.00875315	0.0165877	0.7199992	-0.538876	0.00841042	0	-0.0162434	0.0307831	0.5977275	0.7662977
ENSG00000102854	MSLN	16	814813	A	C	0.0208748	0.0167864	0.061012	0.98	0.904893	0.0421088	1.96E-102	0.0185507	0.0674301	0.7832316	0.9651711
ENSG00000102858	MGRN1	16	4703734	T	G	0.457256	-0.0135859	0.0143391	0.25	-0.3456	0.00781076	0	0.039311	0.0414999	0.3435088	0.1202416
ENSG00000102871	TRADD	16	67191142	G	A	0.0715706	0.0221434	0.0270443	0.5400003	-0.509417	0.0146392	2.59E-265	-0.0434681	0.0531034	0.4130398	0.998015
ENSG00000102879	CORO1A	16	30197272	G	T	0.54672	-0.0180647	0.0141919	0.08999948	0.13145	0.00793549	1.25E-61	-0.137427	0.108283	0.2043888	0.9054802
ENSG00000102882	MAPK3	16	30130126	C	T	0.437376	-0.0243271	0.0145241	0.02900013	0.508772	0.00767103	0	-0.0478153	0.0285564	0.0940494	0.9250462
ENSG00000102886	GDPD3	16	30120654	C	G	0.437376	-0.0238932	0.0145296	0.032	0.177536	0.00811986	5.67E-106	-0.134582	0.0820715	0.1010429	0.9195266
ENSG00000102890	ELMO3	16	67235473	G	A	0.0596421	0.0216821	0.0288893	0.58	0.425891	0.0223767	9.12E-81	0.05091	0.0678854	0.4532904	0.7165409
ENSG00000102893	PHKB	16	47615234	A	G	0.0367793	-0.074379	0.0357845	0.03599979	-0.324605	0.0225925	8.24E-47	0.229137	0.111388	0.03967552	0.3427924
ENSG00000102897	LYRM1	16	20923759	G	T	0.173956	0.0266823	0.0192674	0.17	0.124944	0.0108865	1.72E-30	0.213554	0.155327	0.169172	0.9970286
ENSG00000102900	NUP93	16	56822404	G	A	0.219682	-0.0131547	0.0184871	0.3800004	0.20994	0.0148045	1.20E-45	-0.0626594	0.0881699	0.4772907	0.5796864
ENSG00000102901	CENPT	16	67871887	T	C	0.0347913	0.0138611	0.0352003	0.7099994	0.243318	0.0254879	1.34E-21	0.056967	0.144791	0.6939921	0.6495234
ENSG00000102904	TSNAXIP1	16	67853359	C	A	0.166004	-0.0108721	0.0198338	0.4199997	0.0996982	0.0111432	3.65E-19	-0.10905	0.199311	0.5842865	0.2981572
ENSG00000102908	NFAT5	16	69668783	A	G	0.450298	-0.00129871	0.0145372	0.9400001	-0.073129	0.0081112	1.95E-19	0.0177592	0.198798	0.9288176	0.03576067
ENSG00000102910	LONP2	16	48337620	C	T	0.16501	-0.00998754	0.0197709	0.4199997	-0.421495	0.0106605	0	0.0236955	0.0469104	0.6134729	0.9308397
ENSG00000102921	N4BP1	16	48613348	C	T	0.323062	0.00535131	0.0153287	0.89	-0.544478	0.00838936	0	-0.00982834	0.0281534	0.727015	0.6428986

ENSG00000102931	ARL2BP	16	57283263	G	A	0.471173	-0.0158016	0.0141821	0.2099999	-0.0843411	0.00794592	2.55E-26	0.187353	0.169076	0.2678165	0.7283912
ENSG00000102934	PLLP	16	57304301	A	C	0.0159046	0.0794951	0.0603264	0.2099999	-0.404747	0.041723	2.99E-22	-0.196407	0.150416	0.191635	0.279731
ENSG00000102967	DHODH	16	72050720	A	C	0.50994	-0.0118074	0.0141947	0.2700001	0.102017	0.00797372	1.77E-37	-0.115739	0.139434	0.406502	0.4799954
ENSG00000102974	CTCF	16	67634698	G	C	0.0367793	0.0379392	0.0347958	0.29	0.171701	0.0210893	3.90E-16	0.220961	0.204463	0.2798348	0.9246444
ENSG00000102977	ACD	16	67693064	G	A	0.125249	0.00187257	0.0211997	0.8499999	-0.220179	0.0120085	4.33E-75	-0.00850477	0.0962852	0.9296152	0.3599494
ENSG00000102978	POLR2C	16	57501110	G	A	0.257455	-0.00451872	0.0162699	0.7700005	0.190665	0.00979731	2.35E-84	-0.0236997	0.0853409	0.7812375	0.7107896
ENSG00000102981	PARD6A	16	67695765	C	A	0.166004	-0.0108721	0.0198338	0.4199997	-0.121456	0.0111354	1.06E-27	0.0895144	0.163506	0.5840564	0.5423207
ENSG00000102996	MMP15	16	58070137	A	G	0.239563	0.00847362	0.0160915	0.4500005	0.064079	0.00901785	1.20E-12	0.132237	0.251808	0.5994791	0.9626591
ENSG00000103005	USB1	16	58044486	T	C	0.214712	-0.0207212	0.0168942	0.25	-0.225584	0.0105318	8.81E-102	0.0918558	0.0750136	0.2207556	0.46799
ENSG00000103018	CYB5B	16	69479298	T	G	0.00497018	-0.0325258	0.115064	0.7700005	-1.02608	0.0521678	3.99E-86	0.0316992	0.112151	0.7774478	0.2546645
ENSG00000103024	NME3	16	1821009	G	C	0.328032	0.00752534	0.015472	0.64	0.197481	0.00849849	1.92E-119	0.0381066	0.0783639	0.6267701	0.5623541
ENSG00000103034	NDRG4	16	58522141	G	A	0.201789	-0.0228084	0.0178659	0.1900002	0.140611	0.00972132	2.04E-47	-0.162209	0.127553	0.2034795	0.7669786
ENSG00000103035	PSMD7	16	74335429	T	C	0.158052	0.0382921	0.01991	0.02699977	-0.0812473	0.0142195	1.10E-08	-0.471303	0.258564	0.06833813	0.609984
ENSG00000103037	SETD6	16	58551907	C	T	0.241551	0.012031	0.01642	0.4299995	-0.213139	0.00910506	3.48E-121	-0.0564468	0.0770768	0.4639578	0.08831294
ENSG00000103042	SLC38A7	16	58709010	C	A	0.0129225	-0.0575138	0.0671075	0.4299995	0.578924	0.0373827	4.29E-54	-0.0993461	0.116095	0.3921469	0.980555
ENSG00000103043	VAC14	16	70778203	G	A	0.428429	-0.02695	0.0143908	0.04700023	-0.063113	0.00803435	3.99E-15	0.427012	0.234406	0.06850465	0.3085649
ENSG00000103047	TANGO6	16	68998295	T	C	0.161034	-0.0100982	0.0197739	0.7199992	0.186595	0.013385	3.59E-44	-0.0541182	0.106043	0.6098126	0.5929236
ENSG00000103051	COG4	16	70535969	C	T	0.498012	0.00414626	0.0142228	0.8800001	-0.213856	0.00789502	1.39E-161	-0.0193881	0.0665104	0.770665	0.7087414
ENSG00000103056	SMPD3	16	68437411	A	G	0.357853	-0.000160036	0.0150569	0.84	-0.120589	0.00852873	2.18E-45	0.00132711	0.124861	0.9915196	0.1614372
ENSG00000103061	SLC7A6OS	16	68331627	A	G	0.411531	-0.0285732	0.0147178	0.01899984	-0.170641	0.0120571	1.79E-45	0.167446	0.0870576	0.05443077	0.2335557
ENSG00000103064	SLC7A6	16	68317077	A	T	0.451292	-0.0242666	0.0144031	0.03400008	-0.455726	0.00833632	0	0.0532482	0.0316198	0.09217814	0.5692509
ENSG00000103066	PLA2G15	16	68287084	C	G	0.316103	-0.0172874	0.0158065	0.2	0.0886964	0.0088514	1.24E-23	-0.194905	0.179267	0.276934	0.06155164
ENSG00000103067	ESRP2	16	68267509	G	T	0.507952	-0.0244884	0.0142862	0.032	-0.13136	0.0121684	3.63E-27	0.186422	0.110118	0.09047056	0.8756076
ENSG00000103091	WDR59	16	74970769	G	A	0.527833	-0.0182938	0.0143069	0.2200002	-0.126658	0.0079288	1.93E-57	0.144435	0.113318	0.2024533	0.0851897
ENSG00000103111	MON1B	16	77230517	C	G	0.435388	0.0107731	0.0142594	0.5500004	-0.460494	0.00752684	0	-0.0233947	0.0309678	0.4499784	0.6530974
ENSG00000103121	CMC2	16	81031786	T	C	0.229622	0.0275167	0.0169505	0.07399971	0.487843	0.0101209	0	0.0564049	0.0347655	0.1047099	0.6854548
ENSG00000103126	AXIN1	16	370056	A	G	0.196819	-0.0481047	0.0171908	0.00179999	-0.462126	0.0103587	0	0.104094	0.0372725	0.00522551	0.4706156
ENSG00000103145	HCFC1R1	16	3073454	A	T	0.44334	-0.019047	0.0145047	0.28	-0.0815737	0.00912009	3.74E-19	0.233494	0.179717	0.1938639	0.5401765
ENSG00000103148	NPRL3	16	161566	A	G	0.181909	0.0227822	0.0203174	0.2200002	0.374052	0.0122668	3.23E-204	0.0609065	0.0543538	0.2624763	0.09233835
ENSG00000103150	MLYCD	16	83941259	G	A	0.388668	0.00103566	0.0148758	1	-0.114484	0.00828824	2.13E-43	-0.00904635	0.12994	0.9444965	0.04316078
ENSG00000103152	MPG	16	131429	T	C	0.132207	0.0448739	0.0214229	0.0329997	-0.171333	0.0143117	5.01E-33	-0.26191	0.126936	0.03908185	0.5855858
ENSG00000103154	NECAB2	16	84019309	G	A	0.22167	0.00278812	0.0166476	0.6600001	0.468299	0.00928358	0	0.00595372	0.0355493	0.8669941	0.6241153
ENSG00000103160	HSDL1	16	84167341	T	A	0.210736	0.00678925	0.0174211	0.5099998	0.386368	0.00981592	0	0.017572	0.0450916	0.696762	0.06363813
ENSG00000103168	TAF1C	16	84216063	G	T	0.455268	0.00344937	0.0142607	0.95	-0.541994	0.00739952	0	-0.00636422	0.0263117	0.8088746	0.2184629
ENSG00000103174	NAGPA	16	5079493	G	A	0.466203	-0.0152131	0.014342	0.28	-0.49323	0.00747554	0	0.0308438	0.0290815	0.2888714	0.9209223
ENSG00000103184	SEC14L5	16	5038738	G	T	0.447316	-0.00555221	0.0144632	0.6300007	-0.4256	0.00766836	0	0.0130456	0.0339839	0.7010703	0.2109051
ENSG00000103187	COTL1	16	84625441	A	G	0.109344	-0.00150222	0.022546	0.8800001	0.262003	0.0190036	3.05E-43	-0.00573359	0.0860534	0.9468776	0.02282141
ENSG00000103194	USP10	16	84773556	G	T	0.0149105	-0.0656187	0.0483704	0.1800002	-0.73607	0.0471018	4.75E-55	0.0891474	0.0659616	0.1765337	0.9924633
ENSG00000103196	CRISPLD2	16	84903982	C	A	0.274354	0.00404852	0.0162571	0.7700005	-0.564167	0.00917011	0	-0.0071761	0.0288163	0.8033385	0.9292434
ENSG00000103199	ZNF500	16	4807932	C	T	0.256461	0.0235666	0.0158592	0.1100001	0.363297	0.0101865	1.40E-278	0.0648688	0.0436915	0.137623	0.6817375
ENSG00000103202	NME4	16	453546	T	C	0.294235	0.0166199	0.0151748	0.2099999	0.512426	0.00937247	0	0.0324338	0.0296196	0.273512	0.2813193
ENSG00000103222	ABCC1	16	16140182	C	G	0.177932	-3.77E-05	0.0189613	0.84	-0.274405	0.0109217	2.67E-139	0.000137378	0.0690997	0.9984137	0.5721709
ENSG00000103226	NOMO3	16	16357510	C	T	0.213718	-0.0228956	0.0176095	0.2	-0.709852	0.025271	1.31E-173	0.0322541	0.0248339	0.1940146	0.5310072
ENSG00000103227	LMF1	16	967476	G	A	0.337972	0.0160171	0.0150078	0.3100002	0.230649	0.00821849	2.65E-173	0.0694438	0.0651149	0.2862063	0.1130983
ENSG00000103245	CIAO3	16	785541	C	T	0.240557	0.00477513	0.0175187	0.6499995	0.48427	0.0105658	0	0.00986047	0.0361761	0.7851849	0.6625538
ENSG00000103248	MTHFSD	16	86576311	C	T	0.0487078	-0.0132328	0.0340428	0.4799997	-0.916029	0.0192632	0	0.0144458	0.0371647	0.6974998	0.4079506
ENSG00000103249	CLCN7	16	1510258	T	C	0.110338	0.0253782	0.0208047	0.1199999	-0.71499	0.0126044	0	-0.0354945	0.0291046	0.2226362	0.9667466
ENSG00000103253	HAGHL	16	781230	C	T	0.240557	0.00477513	0.0175187	0.6499995	0.457867	0.0121762	1.884426e-309	0.0104291	0.0382625	0.7851867	0.7354524
ENSG00000103254	ANTKMT	16	771591	T	C	0.240557	0.0168238	0.0173324	0.2700001	0.287213	0.0109856	1.14E-150	0.058576	0.0603884	0.3320526	0.7498055
ENSG00000103257	SLC7A5	16	87883361	T	C	0.26839	-0.00215887	0.0160226	0.7899998	-0.179359	0.0100286	1.55E-71	0.0120366	0.0893349	0.8928212	0.3216037

ENSG00000103260	METRN	16	767385	T	C	0.240557	0.0168238	0.0173324	0.2700001	0.386184	0.0148301	1.72E-149	0.0435642	0.0449124	0.3320552	0.8355952
ENSG00000103264	FBXO31	16	87393170	C	G	0.452286	-0.00536692	0.0143356	0.6700003	-0.230892	0.00791678	5.41E-187	0.0232443	0.062093	0.7081467	0.08152221
ENSG00000103269	RHBDL1	16	726967	A	G	0.285288	0.015465	0.0162965	0.3100002	0.0736089	0.0101451	4.00E-13	0.210097	0.223279	0.3467241	0.9430812
ENSG00000103274	NUBP1	16	10850425	G	A	0.0974155	0.0171394	0.0232479	0.4199997	0.103151	0.0124485	1.17E-16	0.166159	0.226268	0.462739	0.969616
ENSG00000103275	UBE2I	16	1366283	G	A	0.114314	-0.00123399	0.0213382	0.7600007	0.548911	0.0146371	8.77E-308	-0.00224807	0.0388738	0.953884	0.4562288
ENSG00000103313	MEFV	16	3299327	G	C	0.486083	-0.00964429	0.014213	0.4899999	-0.324978	0.0081229	0	0.0296768	0.0437416	0.4974823	0.1174173
ENSG00000103316	CRYM	16	21282299	C	G	0.432406	-0.00152008	0.0143624	0.7600007	-0.16223	0.00796323	2.94E-92	0.00936989	0.0885322	0.9157124	0.5903209
ENSG00000103326	CAPN15	16	591176	T	C	0.0914513	-0.00702529	0.0231231	0.7300002	-0.544691	0.0195824	2.83E-170	0.0128978	0.0424543	0.7612779	0.795294
ENSG00000103335	PIEZO1	16	88816685	G	C	0.22167	-0.0161788	0.0165132	0.4299995	0.32175	0.0125752	2.18E-144	-0.0502837	0.0513607	0.3275642	0.7044445
ENSG00000103342	GSPT1	16	11985962	C	G	0.0447316	0.0497897	0.0423838	0.3599996	-0.296536	0.0236096	3.50E-36	-0.167904	0.143553	0.2421501	0.3730507
ENSG00000103351	CLUAP1	16	3569986	T	G	0.301193	0.000364957	0.0155835	0.8800001	0.16842	0.00868753	1.01E-83	0.00216695	0.0925278	0.9813157	0.6221499
ENSG00000103353	UBFD1	16	23577051	G	A	0.180915	-0.0134354	0.0186321	0.4199997	0.189073	0.0110918	3.73E-65	-0.0710592	0.0986325	0.4712513	0.000119282
ENSG00000103355	PRSS33	16	2835951	A	G	0.248509	0.00472834	0.0166075	0.89	0.0879297	0.0106295	1.32E-16	0.0537741	0.188984	0.7759946	0.6700349
ENSG00000103356	EARS2	16	23551193	G	C	0.161034	0.0016341	0.0192576	0.9299999	-0.183227	0.0112672	1.84E-59	-0.00891846	0.105104	0.9323777	0.01027251
ENSG00000103363	ELOB	16	2824356	A	G	0.230616	0.00539368	0.0170875	0.8700001	0.199855	0.009677	9.25E-95	0.0269879	0.0855094	0.7522957	0.7865426
ENSG00000103365	GGA2	16	23504089	G	C	0.161034	0.0016341	0.0192576	0.9299999	-0.313828	0.011311	1.98E-169	-0.00520699	0.0613638	0.9323771	0.08178614
ENSG00000103381	CPPED1	16	12827396	T	C	0.135189	-0.00899232	0.0189999	0.6100002	0.317168	0.0103621	9.41E-206	-0.0283519	0.059912	0.636052	0.4884077
ENSG00000103404	USP31	16	23116659	A	G	0.026839	0.0344175	0.0532805	0.56	-0.214475	0.0299395	7.86E-13	-0.160473	0.249431	0.519992	0.1498835
ENSG00000103415	HMOX2	16	4542519	G	A	0.275348	0.0267016	0.0161644	0.07599937	0.115319	0.00900209	1.44E-37	0.231546	0.141332	0.1013566	0.6450626
ENSG00000103423	DNAJA3	16	4491291	C	T	0.0139165	-0.0307673	0.0563918	0.7800007	1.1325	0.0530734	5.00E-101	-0.0271676	0.0498103	0.5854643	0.4176158
ENSG00000103429	BFAR	16	14744882	G	C	0.0477137	-0.0207058	0.0320397	0.5999997	-0.20356	0.019173	2.48E-26	0.101718	0.157688	0.5188879	0.3725612
ENSG00000103472	RRN3P2	16	29107101	A	G	0.328032	0.00458856	0.0151034	0.64	0.556009	0.0171314	4.52E-231	0.00825267	0.0271651	0.761283	0.5414707
ENSG00000103479	RBL2	16	53496725	T	C	0.460239	0.00359633	0.0142067	0.8499999	-0.705718	0.00694376	0	-0.00509598	0.0201309	0.8001581	0.3607323
ENSG00000103485	QPRT	16	29700174	C	T	0.0974155	-0.0484333	0.027828	0.02999991	-0.295306	0.013695	4.00E-103	0.164011	0.0945411	0.08277443	0.003785763
ENSG00000103489	XYLT1	16	17380182	G	C	0.485089	0.0141665	0.0142118	0.2200002	0.615345	0.00720335	0	0.0230221	0.0230973	0.3188885	0.1377896
ENSG00000103496	STX4	16	31049253	G	A	0.367793	0.00995747	0.0146444	0.4299995	0.36301	0.00788017	0	0.0274303	0.0403459	0.4965826	0.05753348
ENSG00000103502	CDIPT	16	29872367	A	G	0.33002	0.0261437	0.0148862	0.06800017	0.0895525	0.00904041	3.93E-23	0.291937	0.168821	0.08376088	0.4995575
ENSG00000103507	BCKDK	16	31120769	T	C	0.393638	-0.0308901	0.0147364	0.01700004	0.253408	0.00818436	1.72E-210	-0.121899	0.0582859	0.03649316	0.4251786
ENSG00000103510	KAT8	16	31134894	A	G	0.296223	0.021914	0.0157079	0.14	0.244971	0.00933445	8.41E-152	0.0894553	0.0642119	0.1635813	0.4225928
ENSG00000103512	NOMO1	16	14958777	T	C	0.153082	-0.040656	0.0195841	0.0259998	0.429919	0.023664	9.32E-74	-0.0945667	0.0458495	0.03915559	0.9874591
ENSG00000103522	IL21R	16	27437799	T	C	0.23161	0.00812049	0.0169023	0.58	0.337674	0.009618	4.98E-270	0.0240483	0.0500597	0.6309487	0.1844525
ENSG00000103528	SYT17	16	19229472	A	G	0.478131	0.0102177	0.014187	0.28	0.0637679	0.00793883	9.56E-16	0.160233	0.223371	0.4731656	0.1901524
ENSG00000103534	TMC5	16	19466126	C	T	0.150099	0.00502311	0.0203386	0.7499995	-0.0788675	0.0113361	3.47E-12	-0.0636905	0.258046	0.8050485	0.5542922
ENSG00000103540	CCP110	16	19549931	A	G	0.158052	0.0117191	0.0196996	0.5300002	0.221389	0.0125944	3.61E-69	0.0529344	0.0890328	0.5521444	0.5924407
ENSG00000103544	VPS35L	16	19642338	A	G	0.262425	-0.00136932	0.0160473	0.8700001	-0.182538	0.00875248	1.36E-96	0.00750155	0.0879127	0.9319994	0.5164008
ENSG00000103549	RNF40	16	30780347	T	G	0.321074	0.0154316	0.0148607	0.2	-0.172082	0.00849941	3.82E-91	-0.0896756	0.0864715	0.2997109	0.111466
ENSG00000103550	KNOP1	16	19722229	T	C	0.163022	-0.0064473	0.0187851	0.8800001	0.388678	0.0124566	9.79E-214	-0.0165878	0.0483337	0.7314535	0.9142348
ENSG00000103569	AQP9	15	58454239	G	C	0.408549	0.014837	0.0142477	0.2599998	0.132451	0.00797036	5.16E-62	0.112018	0.10778	0.2986548	0.8364617
ENSG00000103591	AAGAB	15	67520452	T	C	0.261431	0.00742699	0.0166276	0.7800007	-0.535202	0.00933244	0	-0.013877	0.0310689	0.6551257	0.1389501
ENSG00000103642	LACTB	15	63424129	T	C	0.267396	-0.00892772	0.0171192	0.5099998	-0.228308	0.00935865	1.91E-131	0.0391039	0.0750001	0.6020991	0.3334136
ENSG00000103647	CORO2B	15	68945726	G	T	0.298211	0.0133635	0.0151016	0.4299995	-0.22021	0.00843277	2.55E-150	-0.0606852	0.0686175	0.3764811	0.2965305
ENSG00000103653	CSK	15	75084968	C	T	0.395626	-0.00318006	0.0150605	0.64	0.337667	0.00797334	0	-0.00941774	0.0446022	0.8327704	0.632469
ENSG00000103657	HERC1	15	64013479	G	C	0.363817	0.000337728	0.0151473	0.9599999	0.140995	0.00842942	8.39E-63	0.00239531	0.107431	0.9822117	0.5708358
ENSG00000103671	TRIP4	15	64713724	C	T	0.107356	-0.0116818	0.0223864	0.56	0.453468	0.0113887	0	-0.025761	0.0493714	0.6018229	0.7022749
ENSG00000103707	MTFMT	15	65308411	G	C	0.00397614	-0.355129	0.16385	0.01700004	-0.818443	0.0545629	7.34E-51	0.433908	0.202276	0.03194282	0.3561433
ENSG00000103723	AP3B2	15	83353349	A	T	0.185885	0.0237999	0.017773	0.17	0.441013	0.00968388	0	0.0539665	0.0403179	0.1807251	0.8787512
ENSG00000103740	ACSBG1	15	78498920	T	C	0.474155	-0.00344557	0.0142122	0.6200004	0.0978975	0.00795772	8.81E-35	-0.0351957	0.145203	0.8084777	0.8222795
ENSG00000103769	RAB11A	15	66101360	T	C	0.10338	-0.017807	0.0228711	0.5300002	0.13223	0.0135729	1.99E-22	-0.134667	0.173516	0.4376862	0.8771935
ENSG00000103811	CTSH	15	79227658	C	T	0.110338	0.00213809	0.0228114	0.8499999	-0.893564	0.011697	0	-0.00239277	0.0255286	0.9253245	0.01919495
ENSG00000103852	TTC23	15	99733978	C	G	0.472167	0.0121641	0.0142442	0.2599998	-0.36112	0.00772328	0	-0.0336844	0.0394511	0.3932013	0.008455133

ENSG00000103876	FAH	15	80462060	C	G	0.0626243	0.0363923	0.0254753	0.1900002	-0.412088	0.0145186	3.23E-177	-0.0883121	0.0618984	0.1536586	0.2892737
ENSG00000103888	CEMIP	15	81157900	T	C	0.135189	0.0204033	0.0207854	0.2399999	0.0978082	0.0130837	7.69E-14	0.208605	0.214336	0.330423	0.4793397
ENSG00000103932	RPAP1	15	41822920	T	C	0.517893	0.0259311	0.0141907	0.05099998	-0.400513	0.00766707	0	-0.0647447	0.035453	0.0678181	0.258668
ENSG00000103942	HOMER2	15	83582249	A	G	0.192843	0.00543027	0.0171982	0.91	-0.199707	0.00966119	6.32E-95	-0.0271913	0.0861274	0.7522229	0.6349216
ENSG00000103966	EHD4	15	42227863	T	C	0.189861	-0.00297387	0.0184628	0.7600007	0.468688	0.00997974	0	-0.00634509	0.0393927	0.8720362	0.03916974
ENSG00000103978	TMEM87A	15	42534295	T	C	0.0178926	-0.026574	0.0475411	0.6499995	-0.721552	0.0364487	3.20E-87	0.0368289	0.0659135	0.5763349	0.173886
ENSG00000103994	ZNF106	15	42744171	A	G	0.141153	-0.00109752	0.0197453	0.7700005	0.156039	0.0130671	7.20E-33	-0.0070336	0.126542	0.9556738	0.9214372
ENSG00000103995	CEP152	15	49054234	C	T	0.102386	0.0100113	0.021821	0.83	-0.451541	0.0131624	6.50E-258	-0.0221714	0.0483299	0.646413	0.7790241
ENSG00000104043	ATP8B4	15	50312724	G	T	0.225646	-0.00427356	0.0166929	0.95	-0.512426	0.00900999	0	0.00833986	0.0325766	0.7979446	0.7367091
ENSG00000104047	DTWD1	15	49925255	T	C	0.0119284	-0.0119848	0.05533	0.7899998	-0.459807	0.0478498	7.30E-22	0.0260648	0.120364	0.8285584	0.5528041
ENSG00000104064	GABPB1	15	50608497	G	A	0.418489	0.0218638	0.0143429	0.1299999	0.123418	0.00793962	1.73E-54	0.177152	0.116771	0.1292448	0.5453377
ENSG00000104081	BMF	15	40390592	A	G	0.184891	-0.0293727	0.0175817	0.14	-0.240625	0.0102805	3.71E-121	0.122068	0.0732527	0.09563356	0.515236
ENSG00000104093	DMXL2	15	51827469	C	G	0.265408	-0.0238552	0.0165585	0.1800002	0.274809	0.00907367	1.73E-201	-0.0868066	0.0603228	0.1501407	0.07863187
ENSG00000104129	DNAJC17	15	41079871	C	G	0.383698	-0.0069556	0.0148241	0.6200004	0.124367	0.00813207	8.46E-53	-0.0559281	0.119253	0.639078	0.7054379
ENSG00000104131	EIF3J	15	44842241	T	G	0.0188867	0.0449695	0.0453798	0.2700001	0.233207	0.0225967	5.70E-25	0.192831	0.195485	0.3239262	0.5098138
ENSG00000104133	SPG11	15	44905385	A	G	0.0328032	0.0674426	0.0409971	0.07299952	0.309137	0.0278974	1.55E-28	0.218164	0.134071	0.1036894	0.4873763
ENSG00000104147	OIP5	15	41613142	A	G	0.510934	-0.00691165	0.0142003	0.6300007	0.0990955	0.00796575	1.58E-35	-0.0697474	0.143409	0.6267165	0.7354564
ENSG00000104154	SLC30A4	15	45793407	A	T	0.276342	0.0167847	0.0162373	0.4100001	-0.0707541	0.00890168	1.89E-15	-0.237226	0.231422	0.3053255	0.8618332
ENSG00000104164	BLOC1S6	15	45893759	A	G	0.277336	0.016337	0.0162449	0.4400003	0.126931	0.00983973	4.51E-38	0.128708	0.128371	0.3160408	0.9911535
ENSG00000104177	MYEF2	15	48451169	C	T	0.16998	-0.0458341	0.0196591	0.01899984	0.355659	0.0117273	4.99E-202	-0.128871	0.0554382	0.02009413	0.01621006
ENSG00000104205	SGK3	8	67699455	T	G	0.0178926	-0.0291127	0.0610134	0.4700002	-0.280899	0.0334236	4.31E-17	0.103641	0.217558	0.6337999	0.2336319
ENSG00000104219	ZDHHC2	8	17047923	C	T	0.185885	-0.00750204	0.0198374	0.5999997	0.454681	0.0106353	0	-0.0164996	0.043631	0.7053104	0.4845243
ENSG00000104221	BRF2	8	37704104	C	T	0.292247	0.0257471	0.0156445	0.08100093	0.111203	0.00898002	3.21E-35	0.231532	0.141921	0.1028026	0.2065739
ENSG00000104228	TRIM35	8	27155620	A	T	0.440358	0.00504064	0.0142031	0.3700002	-0.326833	0.00778489	0	-0.0154227	0.0434583	0.7226761	0.6576463
ENSG00000104231	ZFAND1	8	82629353	C	G	0.15507	-0.0107026	0.0204485	0.5199996	-0.359688	0.0119426	2.81E-199	0.0297552	0.0568592	0.6007558	0.313849
ENSG00000104267	CA2	8	86384901	C	G	0.482107	-0.00645956	0.0142237	0.91	-0.350069	0.00774806	0	0.0184523	0.0406332	0.6497441	0.4326194
ENSG00000104290	FZD3	8	28391752	T	C	0.131213	0.0346525	0.0206583	0.05800027	-0.340532	0.01198	9.96E-178	-0.10176	0.0607704	0.09403241	0.06111394
ENSG00000104299	INTS9	8	28686468	T	G	0.17992	0.0150332	0.018512	0.4899999	-0.0907832	0.00978795	1.78E-20	-0.165595	0.204695	0.4185245	0.8435988
ENSG00000104312	RIPK2	8	90786633	T	C	0.510934	0.00783326	0.0142188	0.5700002	0.257986	0.00782819	3.46E-238	0.0303631	0.0551222	0.5817491	0.8224303
ENSG00000104320	NBN	8	90980510	C	A	0.33002	0.0200707	0.0146824	0.1499999	-0.351901	0.00799144	0	-0.057035	0.0417432	0.1718347	0.1463917
ENSG00000104324	CPQ	8	97909668	C	A	0.0387674	0.0367331	0.0435824	0.5700002	0.77215	0.0251222	1.89E-207	0.0475725	0.0564641	0.3994937	0.6223883
ENSG00000104325	DECR1	8	91038976	T	C	0.0745527	-0.0129286	0.0286735	0.84	-1.19954	0.0261742	0	0.0107779	0.0239048	0.6520844	0.9234996
ENSG00000104327	CALB1	8	91089269	G	T	0.319085	0.026986	0.015104	0.09099971	0.138528	0.00840072	4.33E-61	0.194806	0.10967	0.07568627	0.09135094
ENSG00000104331	BPNT2	8	57888447	T	C	0.127237	0.00618142	0.021537	0.7300002	0.182501	0.0123668	2.76E-49	0.0338705	0.118032	0.7741432	0.9992987
ENSG00000104332	SFRP1	8	41143248	A	G	0.370775	-0.00320284	0.0150012	0.7700005	-0.0691242	0.00869924	1.93E-15	0.0463346	0.217097	0.8309927	0.1833762
ENSG00000104341	LAPTM4B	8	98826263	T	C	0.144135	0.029481	0.0199036	0.09499921	-0.650068	0.011438	0	-0.0453506	0.0306281	0.1386901	0.8862144
ENSG00000104343	UBE2W	8	74741738	C	G	0.0149105	-0.111406	0.0582074	0.05600025	-0.264811	0.0354936	8.60E-14	0.4207	0.226925	0.06375088	0.2144192
ENSG00000104356	POP1	8	99150793	A	G	0.05666	-0.0148513	0.0391663	0.4899999	0.195645	0.0223049	1.76E-18	-0.0759094	0.200378	0.7048127	0.2455708
ENSG00000104361	NIPAL2	8	99254341	A	G	0.0616302	-0.0202598	0.0292943	0.3400001	-0.776273	0.015531	0	0.0260988	0.0377407	0.4892331	0.1282159
ENSG00000104365	IKBKB	8	42159396	A	C	0.126243	-0.0158957	0.0219314	0.5999997	0.182035	0.0118766	5.03E-53	-0.0873223	0.120614	0.4690763	0.7473536
ENSG00000104368	PLAT	8	42048739	G	A	0.412525	-0.0226127	0.0143942	0.1199999	-0.0722425	0.00803731	2.51E-19	0.313011	0.202269	0.1217424	0.306574
ENSG00000104375	STK3	8	99684343	T	G	0.331014	0.00495343	0.0148691	0.8800001	0.0511369	0.00842148	1.26E-09	0.0968661	0.291208	0.7394094	0.9519744
ENSG00000104381	GDAP1	8	75317236	A	G	0.457256	-0.0049785	0.0142045	0.91	0.114397	0.00797839	1.26E-46	-0.0435194	0.124205	0.7260514	0.5607034
ENSG00000104388	RAB2A	8	61482801	G	A	0.217694	-0.000857738	0.0166509	0.8200001	0.376765	0.00914562	0	-0.00227659	0.0441944	0.9589168	0.121037
ENSG00000104408	EIF3E	8	109330503	G	A	0.49006	-0.00373152	0.0142466	0.5999997	-0.0739772	0.0131853	2.02E-08	0.0504415	0.192791	0.7936001	0.3337322
ENSG00000104412	EMC2	8	109477487	C	G	0.357853	0.00226666	0.0146386	0.6899999	-0.142911	0.0091175	2.26E-55	-0.0158606	0.102436	0.8769524	0.907923
ENSG00000104419	NDRG1	8	134281839	C	T	0.289264	-0.00749104	0.0158762	0.5999997	-0.316972	0.00897716	4.32E-273	0.0236332	0.0500916	0.6370704	0.001777978
ENSG00000104427	ZC2HC1A	8	79604108	T	G	0.284294	0.0177397	0.0164209	0.4	-0.811398	0.00882966	0	-0.0218631	0.0202392	0.2800364	0.9858566
ENSG00000104432	IL7	8	79652868	A	C	0.284294	0.0178775	0.0164223	0.3900004	-0.0680224	0.00906288	6.11E-14	-0.262818	0.243951	0.2813291	0.9736596
ENSG00000104442	ARMC1	8	66530568	G	T	0.240557	-0.00404985	0.016846	0.9	0.136555	0.00965664	2.12E-45	-0.0296574	0.123382	0.8100438	0.6548524

ENSG00000104447	TRPS1	8	116621311	A	T	0.529821	0.0199121	0.0143113	0.2	0.125422	0.00793005	2.41E-56	0.158761	0.114546	0.1657462	0.2898675
ENSG00000104450	SPAG1	8	101220820	T	G	0.424453	0.0141715	0.0143215	0.32	0.368615	0.00778369	0	0.0384453	0.0388607	0.3225113	0.1031602
ENSG00000104472	CHRAC1	8	141524316	T	C	0.147117	-0.00235184	0.0210318	0.95	-0.330173	0.011697	2.72E-175	0.00712305	0.0636998	0.9109644	0.04816018
ENSG00000104490	NCALD	8	102917953	T	C	0.0944334	0.0261922	0.0219794	0.09499921	0.462567	0.0125414	8.62E-298	0.0566236	0.047541	0.2336344	0.9960744
ENSG00000104497	SNX16	8	82733458	C	T	0.406561	-0.0163628	0.0144605	0.2399999	0.664765	0.00718588	0	-0.0246144	0.0217544	0.2578587	0.06743654
ENSG00000104517	UBR5	8	103345154	C	T	0.148111	0.0163999	0.0198211	0.2700001	0.183148	0.0116653	1.51E-55	0.0895446	0.108375	0.4086626	0.2304016
ENSG00000104518	GSDMD	8	144640304	T	C	0.351889	-0.0165458	0.0149049	0.1900002	-0.289122	0.00919998	8.82E-217	0.0572277	0.0515844	0.2672579	0.6285569
ENSG00000104522	GFUS	8	144697503	G	C	0.0685885	-0.00777141	0.027619	0.84	-0.167483	0.0176016	1.81E-21	0.0464011	0.164978	0.7785139	0.7548466
ENSG00000104524	PYCR3	8	144689013	C	T	0.319085	-0.0215974	0.0153495	0.1100001	-0.0566114	0.00984889	9.03E-09	0.381503	0.279143	0.1717221	0.8710374
ENSG00000104529	EEF1D	8	144671789	A	G	0.240557	-0.0010597	0.0167083	0.9699999	0.139029	0.0106843	1.04E-38	-0.00762216	0.12018	0.9494298	0.8422641
ENSG00000104549	SQLE	8	126022632	A	G	0.084493	0.0320481	0.0243274	0.28	0.238088	0.014126	9.71E-64	0.134606	0.10249	0.1890614	0.6022229
ENSG00000104611	SH2D4A	8	19212428	A	G	0.296223	-0.0148772	0.0154594	0.1800002	-0.0756002	0.00866101	2.57E-18	0.196788	0.205728	0.3387973	0.1820814
ENSG00000104613	INTS10	8	19692122	T	C	0.456262	0.0133751	0.0144292	0.4	0.272632	0.00782442	5.29E-266	0.0490591	0.0529442	0.3541244	0.8107897
ENSG00000104626	ERI1	8	8916956	C	A	0.314115	0.00543195	0.0152113	0.5300002	-0.118239	0.00849162	4.52E-44	-0.0459404	0.128691	0.7211051	0.04097364
ENSG00000104635	SLC39A14	8	22258202	T	G	0.492048	-0.00541866	0.0142414	0.58	-0.070585	0.00867991	4.22E-16	0.0767678	0.201983	0.703893	0.544197
ENSG00000104643	MTMR9	8	11163785	C	T	0.222664	0.0199104	0.017159	0.1499999	0.140065	0.00963443	6.96E-48	0.142151	0.122897	0.2474076	0.2594687
ENSG00000104660	LEPROTL1	8	29993819	A	G	0.0407555	-0.032698	0.0339917	0.28	0.636774	0.0185403	1.66E-258	-0.0513495	0.0534021	0.3362688	0.005258373
ENSG00000104671	DCTN6	8	30027484	A	G	0.00994036	-0.0634573	0.0862376	0.2999998	0.985552	0.0692616	6.02E-46	-0.0643876	0.0876188	0.4624244	0.5844781
ENSG00000104679	R3HCC1	8	23140712	A	G	0.258449	0.01402	0.0156685	0.4	0.142494	0.00940555	7.58E-52	0.0983901	0.110151	0.3717328	0.7645311
ENSG00000104687	GSR	8	30560513	A	C	0.406561	-0.00429175	0.0147282	0.7400005	0.394374	0.00798361	0	-0.0108824	0.0373464	0.7707518	0.9920412
ENSG00000104689	TNFRSF10A	8	23065302	T	G	0.223658	0.000906574	0.0171867	0.8499999	0.298427	0.0101817	7.73E-189	0.00303784	0.0575911	0.9579323	0.4448925
ENSG00000104691	UBXN8	8	30607143	T	G	0.156064	0.00981484	0.0192481	0.5999997	0.0640968	0.0102755	4.44E-10	0.153125	0.301299	0.6113012	0.9758129
ENSG00000104695	PPP2CB	8	30651901	A	G	0.0337972	0.038486	0.0315794	0.14	0.870003	0.0185767	0	0.0442366	0.0363103	0.2231125	0.986816
ENSG00000104714	ERICH1	8	626426	A	G	0.206759	0.0078623	0.0170411	0.64	0.535834	0.00894287	0	0.014673	0.0318039	0.644541	0.7855839
ENSG00000104722	NEFM	8	24773566	G	T	0.213718	0.0137639	0.0180158	0.4299995	-0.146362	0.0146577	1.77E-23	-0.0940398	0.12345	0.4462008	0.2001407
ENSG00000104723	TUSC3	8	15449441	G	A	0.0228628	0.00945933	0.0442796	0.7600007	0.347491	0.0270623	9.74E-38	0.0272218	0.127444	0.8308608	0.1732221
ENSG00000104725	NA	8	24811546	C	T	0.355865	-0.00668446	0.0150534	0.5199996	-0.197982	0.00818898	3.92E-129	0.033763	0.0760472	0.6570607	0.1742721
ENSG00000104728	ARHGEF10	8	1839474	A	G	0.292247	0.0164683	0.0153895	0.2099999	0.523532	0.00894233	0	0.0314561	0.0294004	0.2846549	0.3821006
ENSG00000104731	KLHDC4	16	87764844	A	C	0.215706	-0.00887672	0.0177085	0.64	-0.691469	0.00873649	0	0.0128375	0.0256105	0.616189	0.9142981
ENSG00000104738	MCM4	8	48881732	T	C	0.296223	-0.0259394	0.0160529	0.0519996	-0.12931	0.0086102	5.58E-51	0.200599	0.12486	0.1081427	0.3755752
ENSG00000104756	KCTD9	8	25300679	A	T	0.220676	-0.0296829	0.0175619	0.04200007	-0.133806	0.0103048	1.49E-38	0.221835	0.132356	0.09372879	0.8823878
ENSG00000104763	ASAH1	8	17928214	T	G	0.464215	-0.0131504	0.0142258	0.3599996	0.526311	0.00740477	0	-0.024986	0.0270316	0.3553162	0.975424
ENSG00000104765	BNIP3L	8	26301783	T	C	0.0308151	-0.03169	0.042662	0.33	0.186343	0.0219454	2.05E-17	-0.170063	0.229818	0.4593064	0.9793111
ENSG00000104774	MAN2B1	19	12767440	G	A	0.0974155	0.01003	0.0229085	0.56	-1.0809	0.0311734	1.96E-263	-0.00927931	0.0211956	0.6615357	0.3562343
ENSG00000104783	KCNN4	19	44278047	C	T	0.341948	-0.0087202	0.0146773	0.59	0.631845	0.00747801	0	-0.0138012	0.0232298	0.5524361	0.6813361
ENSG00000104804	TULP2	19	49393106	T	C	0.110338	-0.0169024	0.0235596	0.3100002	0.0843912	0.0134364	3.37E-10	-0.200286	0.280987	0.4759717	0.85119
ENSG00000104805	NUCB1	19	49414968	C	G	0.128231	-0.012514	0.0220399	0.4199997	-0.784739	0.0125085	0	0.0159467	0.0280868	0.5701944	0.9220214
ENSG00000104808	DHDH	19	49442582	C	T	0.205765	0.000455117	0.0190283	0.9699999	0.189844	0.0105694	3.89E-72	0.00239733	0.100232	0.9809181	0.2529242
ENSG00000104812	GYS1	19	49483974	C	A	0.0954274	-0.0286629	0.0273514	0.3100002	0.25238	0.015921	1.36E-56	-0.113571	0.108611	0.2957145	0.09268871
ENSG00000104814	MAP4K1	19	39093901	G	C	0.163022	0.000925009	0.0211041	0.9400001	0.136084	0.0109963	3.55E-35	0.00679736	0.155083	0.9650395	0.9987804
ENSG00000104823	ECH1	19	39314353	C	T	0.408549	0.0111171	0.0142998	0.6600001	-0.307219	0.0115896	7.80E-155	-0.0361863	0.046566	0.4371019	0.3210157
ENSG00000104833	TUBB4A	19	6498594	A	G	0.0934394	-0.0199367	0.0272095	0.5	-1.07203	0.0450162	2.37E-125	0.0185971	0.0253932	0.4639462	0.998128
ENSG00000104835	SARS2	19	39423200	A	G	0.331014	0.0251218	0.0145703	0.03899959	0.593355	0.00755272	0	0.0423385	0.0245617	0.08475054	0.1994105
ENSG00000104852	SNRNP70	19	49600269	T	C	0.229622	-0.00429274	0.0164461	0.81	-0.151436	0.010049	2.56E-51	0.0283468	0.108617	0.7941083	0.7424763
ENSG00000104853	CLPTM1	19	45477220	T	C	0.0168986	-0.136057	0.0649177	0.03799969	0.380599	0.0399831	1.75E-21	-0.357481	0.174652	0.04067635	NA
ENSG00000104856	RELB	19	45523070	G	A	0.50497	0.0213781	0.0142061	0.09200046	-0.0495601	0.00794054	4.34E-10	-0.431357	0.294858	0.1434861	0.7398289
ENSG00000104859	CLASRP	19	45558256	C	T	0.463221	0.0201378	0.0143454	0.09400046	-0.123806	0.0147297	4.27E-17	-0.162656	0.117475	0.1661739	0.7377673
ENSG00000104863	LIN7B	19	49619649	G	T	0.240557	-0.00119823	0.0162622	0.9599999	0.165701	0.0147918	3.98E-29	-0.00723128	0.0981439	0.9412648	0.9556046
ENSG00000104866	PPP1R37	19	45622994	G	C	0.149105	0.032831	0.019162	0.1299999	0.396103	0.0200498	7.12E-87	0.0828849	0.0485578	0.08783473	0.04695603
ENSG00000104870	FCGRT	19	50019831	T	C	0.0914513	-0.0419388	0.025757	0.04399973	-0.442984	0.0156233	7.45E-177	0.0946733	0.0582401	0.104041	0.9653883

ENSG00000104872	PIH1D1	19	49953154	T	G	0.246521	0.0026843	0.0168257	0.9199999	0.267005	0.0104496	5.27E-144	0.0100534	0.0630177	0.8732493	0.1862303
ENSG00000104879	CKM	19	45817953	G	A	0.319085	-0.0128454	0.015566	0.5099998	0.277959	0.00858524	5.90E-230	-0.0462133	0.0560192	0.4093979	0.6660805
ENSG00000104880	ARHGEF18	19	7498681	A	G	0.0337972	-0.041985	0.0386452	0.2700001	-0.805016	0.0330333	3.58E-131	0.0521543	0.0480532	0.2777693	0.6645738
ENSG00000104881	PPP1R13L	19	45896249	G	A	0.418489	0.00420932	0.0145762	0.8200001	0.0573267	0.00888289	1.09E-10	0.0734269	0.25452	0.7729704	0.3483049
ENSG00000104883	PEX11G	19	7552048	T	G	0.21173	0.0180926	0.0176054	0.2700001	0.477546	0.0100166	0	0.0378866	0.036875	0.3042158	0.559728
ENSG00000104884	ERCC2	19	45863635	C	A	0.452286	-0.0186701	0.0142741	0.28	0.239933	0.0117391	7.56E-93	-0.077814	0.0596138	0.1917906	0.7316853
ENSG00000104885	DOT1L	19	2198362	C	A	0.0914513	-0.0175237	0.0273914	0.3900004	0.176064	0.022617	6.99E-15	-0.0995302	0.156101	0.5237325	0.7184152
ENSG00000104886	PLEKHJ1	19	2233898	T	C	0.0477137	-0.0131357	0.0368879	0.6600001	0.44128	0.0218728	1.63E-90	-0.0297673	0.083606	0.7218089	0.9403322
ENSG00000104889	RNASEH2A	19	12920923	C	G	0.439364	-0.0046507	0.0144729	0.5700002	0.162421	0.00961937	5.82E-64	-0.0286336	0.0891234	0.747998	0.6045881
ENSG00000104892	KLC3	19	45845735	G	A	0.333996	0.0189594	0.015155	0.32	0.213476	0.00834313	2.13E-144	0.088813	0.0710765	0.2114676	0.4490605
ENSG00000104894	CD37	19	49842510	A	G	0.235586	0.00348275	0.0163474	0.8800001	-0.384109	0.00881442	0	-0.00906709	0.0425598	0.831293	0.9557397
ENSG00000104897	SF3A2	19	2242616	C	T	0.161034	0.0258538	0.0207031	0.1800002	0.134083	0.0131789	2.59E-24	0.19282	0.155564	0.2151653	0.05191054
ENSG00000104899	AMH	19	2250369	C	T	0.161034	0.0258538	0.0207031	0.1800002	-0.36573	0.0129934	2.59E-174	-0.070691	0.0566633	0.2121916	0.5797804
ENSG00000104915	STX10	19	13258034	G	A	0.0318091	0.0481117	0.0477178	0.28	-0.360121	0.0347145	3.26E-25	-0.133599	0.133129	0.3156071	0.3186642
ENSG00000104918	RETN	19	7734632	A	G	0.00994036	0.0610955	0.0872929	0.5700002	0.701934	0.047355	1.04E-49	0.0870389	0.124499	0.4844821	0.5498525
ENSG00000104921	FCER2	19	7760338	C	T	0.498012	0.00757116	0.0142148	0.6899999	-0.251413	0.00791155	1.30E-221	-0.0301144	0.0565475	0.5943449	0.292801
ENSG00000104936	DMPK	19	46279392	A	G	0.466203	0.0193061	0.0142121	0.1900002	0.281675	0.00778892	2.27E-286	0.0685402	0.0504912	0.174632	0.60001
ENSG00000104946	TBC1D17	19	50386343	T	C	0.406561	-0.0185934	0.0144857	0.2200002	0.0895892	0.00912276	9.20E-23	-0.207541	0.163066	0.203109	0.3019286
ENSG00000104951	IL4I1	19	50412853	G	T	0.450298	-0.00795716	0.0142385	0.7899998	0.0605097	0.00876519	5.08E-12	-0.131502	0.236079	0.5775097	0.523869
ENSG00000104953	TLE6	19	2986310	G	C	0.220676	-0.0276186	0.0172574	0.05999983	-0.115259	0.0131251	1.61E-18	0.239622	0.152193	0.1153813	0.253361
ENSG00000104957	YJU2B	19	13858342	T	C	0.415507	-0.00930621	0.0143299	0.32	0.247005	0.00794419	3.04E-212	-0.0376762	0.0580273	0.5161546	0.2288169
ENSG00000104960	PTOV1	19	50358996	C	T	0.0208748	-0.0224769	0.0401392	0.5	-0.370709	0.022955	1.15E-58	0.0606322	0.108342	0.5757267	0.6380519
ENSG00000104964	TLE5	19	3058006	C	T	0.402584	-0.00782977	0.0149618	0.58	-0.192899	0.0105643	1.74E-74	0.0405899	0.0775946	0.6009029	0.8374696
ENSG00000104969	SGTA	19	2769040	C	T	0.28827	0.0312367	0.0155003	0.02100003	-0.160543	0.0112512	3.41E-46	-0.194569	0.0975074	0.04599606	0.6898557
ENSG00000104970	NA	19	55050481	A	G	0.4334	0.00368719	0.0144306	0.6100002	0.184195	0.00799235	1.60E-117	0.0200179	0.078349	0.7983399	0.4150449
ENSG00000104972	LILRB1	19	55117162	A	G	0.140159	0.0285633	0.0235254	0.2	0.706696	0.0139119	0	0.0404181	0.0332988	0.2248239	0.3478188
ENSG00000104973	MED25	19	50331806	A	G	0.415507	0.00232338	0.014247	0.8	0.105032	0.0119743	1.76E-18	0.0221208	0.135668	0.8704788	0.1391478
ENSG00000104974	LILRA1	19	55109301	G	T	0.0934394	0.027015	0.0246779	0.28	-0.803789	0.0183573	0	-0.0336096	0.0307116	0.2737962	0.1179112
ENSG00000104976	SNAPC2	19	7986668	G	T	0.291252	0.0191084	0.0164269	0.16	-0.0953544	0.011004	4.50E-18	-0.200393	0.173817	0.2489527	0.05002137
ENSG00000104979	C19orf53	19	13887129	A	G	0.253479	0.0200135	0.0164125	0.2300001	-0.433684	0.00934804	0	-0.0461477	0.0378575	0.2228499	0.2014008
ENSG00000104980	TIMM44	19	8000204	G	A	0.083499	0.0101277	0.0276473	0.6800001	0.393375	0.0160757	3.07E-132	0.0257457	0.0702902	0.7141585	0.187042
ENSG00000104983	CCDC61	19	46511457	A	G	0.362823	-0.00762463	0.015511	0.5300002	0.138486	0.0117053	2.70E-32	-0.0550571	0.112101	0.6233281	0.1294067
ENSG00000104998	IL27RA	19	14153151	A	G	0.0477137	0.0170046	0.0324964	0.58	-0.565202	0.0305086	1.27E-76	-0.0300859	0.0575181	0.6009271	0.4668403
ENSG00000105011	ASF1B	19	14239044	A	G	0.084493	0.00344522	0.0252851	0.7199992	-0.16334	0.0189565	6.90E-18	-0.0210923	0.154819	0.8916332	0.8064133
ENSG00000105048	TNNT1	19	55652442	A	C	0.141153	-0.0218852	0.0199938	0.29	0.920013	0.0153297	0	-0.0237879	0.0217357	0.273772	0.1265292
ENSG00000105053	VRK3	19	50504463	C	G	0.254473	-0.0117569	0.0157481	0.3700002	-0.501775	0.0085478	0	0.0234306	0.0313873	0.4553657	0.3532115
ENSG00000105058	FAM32A	19	16299524	C	T	0.215706	-0.000895876	0.0179378	0.99	-0.107636	0.0105027	1.20E-24	0.00832318	0.166654	0.9601679	0.8292252
ENSG00000105063	PPP6R1	19	55755755	A	G	0.0954274	-0.00747715	0.0271109	0.6800001	-0.243694	0.0164292	8.96E-50	0.0306826	0.111269	0.7827393	0.96905
ENSG00000105072	C19orf44	19	16619642	G	A	0.159046	-0.0171569	0.0209832	0.4	-0.346112	0.0114147	5.95E-202	0.0495704	0.0606476	0.4137272	0.1493444
ENSG00000105122	RASAL3	19	15568907	A	G	0.0218688	-0.0212413	0.0518672	0.5	-0.395368	0.0539649	2.36E-13	0.0537254	0.131392	0.6826174	0.3471509
ENSG00000105127	AKAP8	19	15477404	A	G	0.262425	0.0199986	0.0164924	0.2099999	-0.0791624	0.00935571	2.64E-17	-0.252628	0.210465	0.2300106	0.2356774
ENSG00000105135	ILVBL	19	15231195	A	G	0.135189	0.0114597	0.0205915	0.4100001	0.311534	0.0120652	5.18E-147	0.0367848	0.0661125	0.5779398	0.4825691
ENSG00000105136	ZNF419	19	58002563	G	A	0.0238569	-0.0366895	0.0632774	0.4500005	0.644429	0.0339932	3.82E-80	-0.0569334	0.0982374	0.5622188	0.5596748
ENSG00000105137	SYDE1	19	15222006	A	G	0.135189	0.0118514	0.0205674	0.4	0.115002	0.0125955	6.82E-20	0.103054	0.1792	0.5652382	0.4194818
ENSG00000105146	AURKC	19	57744646	G	C	0.351889	-0.00489721	0.0145321	0.84	0.100694	0.00819873	1.14E-34	-0.0486348	0.144374	0.7362181	0.2874387
ENSG00000105171	POP4	19	30101534	G	C	0.473161	-0.00308849	0.0142679	0.7400005	0.0699871	0.00796738	1.57E-18	-0.0441294	0.203927	0.8286771	0.9094631
ENSG00000105173	CCNE1	19	30309010	T	C	0.356859	0.00999504	0.015106	0.6499995	0.118821	0.00844694	6.08E-45	0.0841182	0.127273	0.5086575	0.127391
ENSG00000105176	URI1	19	30460581	T	C	0.0109344	-0.0587821	0.0663044	0.32	-0.648669	0.0689443	5.03E-21	0.0906195	0.102669	0.3774312	0.9884576
ENSG00000105185	PDCD5	19	33075166	C	A	0.374751	-0.000294771	0.0147488	0.9299999	0.337737	0.00811846	0	-0.000872783	0.0436695	0.9840545	0.2807282
ENSG00000105186	ANKRD27	19	33127470	G	A	0.0357853	-0.0369907	0.0379605	0.29	0.77726	0.02187	1.19E-276	-0.0475912	0.0488572	0.3300137	0.6514746

ENSG00000105193	RPS16	19	39925217	A	G	0.446322	0.0114609	0.0141974	0.3400001	-0.169206	0.00801681	6.95E-99	-0.0677334	0.0839673	0.4198603	0.6372447
ENSG00000105197	TIMM50	19	39977737	T	C	0.362823	0.00373845	0.0145907	0.7899998	-0.0520697	0.00821056	2.27E-10	-0.071797	0.280443	0.7979411	0.5704425
ENSG00000105202	FBL	19	40331076	T	C	0.314115	0.0303757	0.015011	0.0259998	-0.162463	0.0124619	7.56E-39	-0.18697	0.0935029	0.04554156	0.3890655
ENSG00000105204	DYRK1B	19	40320415	C	T	0.437376	0.00307266	0.0142159	0.7700005	0.112955	0.00793044	4.94E-46	0.0272025	0.125869	0.8288961	0.02448321
ENSG00000105205	CLC	19	40225279	A	G	0.304175	0.0147277	0.0160784	0.32	0.0569972	0.00893681	1.80E-10	0.258394	0.284986	0.3645711	0.6227648
ENSG00000105220	GPI	19	34871723	T	C	0.0228628	-0.0197485	0.0458377	0.5199996	0.389452	0.0283053	4.50E-43	-0.0507084	0.117756	0.6667416	0.9477228
ENSG00000105221	AKT2	19	40763833	T	A	0.271372	-0.00345077	0.0155353	0.7600007	-0.0841543	0.00864646	2.18E-22	0.0410053	0.184653	0.824262	0.6081142
ENSG00000105223	PLD3	19	40870354	C	T	0.293241	-0.0042914	0.0152173	0.7700005	-0.19003	0.00844999	5.34E-112	0.0225827	0.0800847	0.7779544	0.3005923
ENSG00000105227	PRX	19	40909474	A	G	0.026839	0.0492877	0.0392244	0.3599996	0.585876	0.0219489	5.72E-157	0.0841264	0.0670241	0.2094181	0.1276467
ENSG00000105229	PIAS4	19	4023514	G	A	0.184891	-0.00776829	0.0192886	0.4799997	0.23553	0.0115429	1.52E-92	-0.0329821	0.0819103	0.6871972	0.4161768
ENSG00000105245	NUMBL	19	41184736	T	C	0.111332	-0.0212401	0.0259965	0.58	0.115614	0.0146655	3.19E-15	-0.183716	0.22606	0.4163993	0.972021
ENSG00000105248	YJU2	19	4258081	G	A	0.309145	-0.00190859	0.0156768	0.9400001	-0.24614	0.00924552	3.72E-156	0.00775409	0.0636913	0.9031011	0.6444927
ENSG00000105251	SHD	19	4284659	T	C	0.531809	0.00477043	0.0143532	0.7600007	-0.221188	0.00909863	1.54E-130	-0.0215673	0.0648976	0.7396409	0.7150433
ENSG00000105254	TBCB	19	36611020	T	G	0.0506958	0.0338154	0.036034	0.4500005	0.752087	0.0226529	1.06E-241	0.0449621	0.0479312	0.3482158	0.08359415
ENSG00000105255	FSD1	19	4314218	A	G	0.464215	0.00114883	0.0143172	0.99	-0.14795	0.00917727	1.80E-58	-0.00776496	0.0967714	0.9360462	0.2484489
ENSG00000105258	POLR2I	19	36605430	C	T	0.412525	-0.0104324	0.0143083	0.6499995	0.271925	0.0146489	6.43E-77	-0.038365	0.0526592	0.4662748	0.3309735
ENSG00000105270	CLIP3	19	36514903	T	C	0.139165	-0.00154817	0.0227821	0.6700003	0.764723	0.011761	0	-0.00202448	0.0297913	0.9458211	0.5034367
ENSG00000105278	ZFR2	19	3836526	A	G	0.434394	-0.0292324	0.0142667	0.04300015	0.0967718	0.0087708	2.64E-28	-0.302076	0.149947	0.0439517	0.07437342
ENSG00000105281	SLC1A5	19	47284995	C	A	0.474155	-0.0169236	0.0143678	0.2300001	0.152534	0.00864383	1.08E-69	-0.110949	0.0944035	0.2398872	0.7299433
ENSG00000105287	PRKD2	19	47198958	G	A	0.172962	-0.000505812	0.0191951	0.9	0.438172	0.010499	0	-0.00115437	0.0438073	0.9789773	0.7499397
ENSG00000105289	TJP3	19	3729459	A	G	0.440358	-0.0293178	0.0142709	0.04099964	0.117858	0.00794019	7.70E-50	-0.248756	0.12224	0.0418531	0.4590014
ENSG00000105321	CCDC9	19	47767223	C	T	0.429423	-0.00204161	0.0143696	0.7400005	0.134047	0.00806087	4.27E-62	-0.0152305	0.107202	0.8870222	0.194395
ENSG00000105323	HNRNPUL1	19	41790952	G	A	0.185885	-0.020727	0.0188733	0.2399999	0.243576	0.0119988	1.29E-91	-0.0850947	0.0775976	0.2728099	0.1628287
ENSG00000105327	BBC3	19	47730052	G	A	0.485089	-0.0272149	0.0142416	0.07000032	0.0554615	0.00813862	9.45E-12	-0.490699	0.266689	0.06577265	0.1931937
ENSG00000105339	DENND3	8	142166642	T	C	0.397614	-0.0142288	0.0146899	0.32	-0.310197	0.00870981	8.30E-278	0.0458702	0.0473742	0.332918	0.4870308
ENSG00000105341	DMAC2	19	41941922	C	A	0.408549	-0.00221226	0.0145028	0.7400005	0.210031	0.00800892	1.39E-151	-0.010533	0.0690518	0.8787629	0.7083663
ENSG00000105352	CEACAM4	19	42129393	T	C	0.280318	0.0119298	0.0154969	0.4799997	-0.663579	0.0122219	0	-0.017978	0.0233559	0.4414535	0.3481717
ENSG00000105355	PLIN3	19	4853066	A	G	0.0815109	-0.0100717	0.02735	0.7099994	-0.259864	0.0171709	9.66E-52	0.0387576	0.105279	0.7127664	0.04484528
ENSG00000105364	MRPL4	19	10366649	A	T	0.149105	-0.00327545	0.0199097	0.98	0.280195	0.0134796	5.72E-96	-0.0116899	0.0710588	0.8693296	0.5442509
ENSG00000105370	LIM2	19	51887188	C	T	0.292247	-0.0233801	0.0160027	0.1800002	0.209136	0.0132282	2.66E-56	-0.111794	0.0768442	0.1457216	0.8830314
ENSG00000105372	RPS19	19	42370491	T	C	0.462227	0.00161554	0.0142027	0.5999997	0.143741	0.0118305	5.74E-34	0.0112393	0.098812	0.9094409	0.4223034
ENSG00000105373	NOP53	19	48254547	A	G	0.321074	-0.00299395	0.0158137	0.6800001	0.331751	0.0086068	0	-0.00902469	0.047668	0.8498388	0.5767079
ENSG00000105374	NKG7	19	51875414	C	T	0.293241	-0.0235169	0.015987	0.17	0.268768	0.00949178	2.20E-176	-0.0874988	0.0595627	0.1418272	0.2594548
ENSG00000105376	ICAM5	19	10404055	G	A	0.203777	-0.0101934	0.0172224	0.4400003	-0.180571	0.0106055	5.25E-65	0.0564509	0.0954349	0.5541778	0.7303262
ENSG00000105379	ETFB	19	51859047	T	C	0.427435	0.00774885	0.0144211	0.5	-0.194179	0.00799782	3.27E-130	-0.0399057	0.0742852	0.5911324	0.1643323
ENSG00000105383	CD33	19	51737717	C	A	0.449304	-0.00846682	0.0143609	0.59	0.247341	0.00794617	1.04E-212	-0.0342313	0.0580715	0.5555469	0.01296481
ENSG00000105393	BABAM1	19	17385108	A	G	0.479125	0.00644543	0.0143182	0.4600002	-0.249516	0.0102315	2.35E-131	-0.0258317	0.0573936	0.6526529	0.9421622
ENSG00000105397	TYK2	19	10476280	G	A	0.263419	0.0180168	0.0156622	0.2300001	0.248384	0.00894155	7.87E-170	0.0725361	0.0631105	0.2504111	0.361034
ENSG00000105401	CDC37	19	10516303	C	A	0.303181	0.0213557	0.0151303	0.1199999	0.137487	0.00846836	2.83E-59	0.155329	0.110464	0.15968	0.3438043
ENSG00000105402	NAPA	19	48004695	T	G	0.0337972	0.0215869	0.039547	0.6100002	-0.260815	0.0242623	5.94E-27	-0.082767	0.151824	0.585649	0.7926272
ENSG00000105404	RABAC1	19	42462187	C	T	0.354871	-0.00181625	0.0149268	0.7600007	-0.454484	0.00834172	0	0.00399629	0.0328435	0.903155	0.4439539
ENSG00000105419	MEIS3	19	47914548	G	A	0.181909	-0.0119622	0.0191443	0.59	0.0784209	0.0122537	1.56E-10	-0.152538	0.245283	0.5340162	0.9421451
ENSG00000105426	PTPRS	19	5249660	T	G	0.10835	0.0434775	0.0221035	0.05800027	0.145417	0.013275	6.34E-28	0.298985	0.154432	0.05286386	0.7545026
ENSG00000105427	CNFN	19	42892805	G	A	0.0606362	0.0547679	0.0321326	0.08100093	-0.181462	0.0183332	4.25E-23	-0.301814	0.179682	0.09301337	0.7684816
ENSG00000105438	KDELR1	19	48890318	G	A	0.482107	-0.00794886	0.0142611	0.7199992	0.0804496	0.00796468	5.48E-24	-0.0988054	0.177537	0.5778459	0.9793036
ENSG00000105443	CYTH2	19	48978930	G	C	0.135189	0.00729634	0.0217852	0.7899998	-0.283228	0.012255	3.57E-118	-0.0257614	0.0769256	0.7377105	0.07724326
ENSG00000105447	GRWD1	19	48954654	T	C	0.115308	-0.00452959	0.024521	0.9699999	-0.430602	0.0130426	4.93E-239	0.0105192	0.0569467	0.8534487	0.01925119
ENSG00000105464	GRIN2D	19	48923160	T	C	0.473161	-0.00759769	0.0142434	0.7300002	0.0585731	0.00796219	1.89E-13	-0.129713	0.243812	0.5947111	0.5951871
ENSG00000105472	CLEC11A	19	51227780	A	G	0.0357853	-0.140913	0.0425684	0.001099993	1.0826	0.0332279	7.62E-233	-0.130162	0.0395231	0.000990125	0.8192369
ENSG00000105479	ODAD1	19	48812422	C	T	0.202783	0.0487092	0.0179305	0.01899984	-0.183132	0.00953604	3.41E-82	-0.265978	0.0988849	0.007149961	0.2061866

ENSG00000105483	CARD8	19	48721615	T	C	0.480119	0.00851786	0.0143247	0.6999999	-0.602751	0.00730845	0	-0.0141316	0.0237662	0.5521029	0.1076139
ENSG00000105486	LIG1	19	48646281	A	G	0.384692	0.02024	0.0147986	0.2	0.252515	0.00841119	5.18E-198	0.0801537	0.0586657	0.1718511	0.6897829
ENSG00000105492	SIGLEC6	19	52028944	G	A	0.415507	-0.00134222	0.0143688	0.9299999	-0.128246	0.00812808	4.40E-56	0.0104659	0.112043	0.9255775	0.5285827
ENSG00000105497	ZNF175	19	52083771	A	G	0.246521	0.000587201	0.0160561	0.5700002	-0.38601	0.00897844	0	-0.00152121	0.0415951	0.9708264	0.1628396
ENSG00000105499	PLA2G4C	19	48582587	C	T	0.465209	-0.011777	0.0143809	0.4600002	-0.644936	0.00746312	0	0.0182607	0.0222992	0.4128454	0.3569261
ENSG00000105501	NA	19	52132466	G	A	0.0964215	-0.0280512	0.0220447	0.1800002	-0.51067	0.0136201	1.16E-307	0.0549302	0.043193	0.2034666	0.2697681
ENSG00000105507	CABP5	19	48540260	T	C	0.0646123	0.0097074	0.026947	0.8499999	0.282465	0.0142793	4.31E-87	0.0343667	0.0954152	0.718712	0.06645361
ENSG00000105509	HAS1	19	52221806	A	G	0.181909	0.0178232	0.0173546	0.2099999	0.252382	0.00974803	8.50E-148	0.0706198	0.0688172	0.3047999	0.5770601
ENSG00000105514	RAB3D	19	11444834	A	G	0.185885	-0.00592226	0.0195122	0.64	0.229409	0.010879	1.04E-98	-0.0258153	0.085063	0.7615208	0.04352382
ENSG00000105518	TMEM205	19	11455323	A	G	0.185885	-0.00592226	0.0195122	0.64	0.368639	0.0107379	2.74E-258	-0.0160652	0.0529324	0.7615059	0.04461695
ENSG00000105519	CAPS	19	5913802	G	A	0.362823	-0.00982253	0.0151932	0.3900004	-0.397893	0.00811568	0	0.0246864	0.0381875	0.5179866	0.2700807
ENSG00000105520	PLPPR2	19	11471218	A	G	0.185885	-0.00592226	0.0195122	0.64	0.422091	0.0108497	0	-0.0140308	0.0462289	0.7615039	0.03090707
ENSG00000105538	RASIP1	19	49233911	A	G	0.45825	0.0325776	0.0141824	0.0016	0.0813307	0.00803536	4.43E-24	0.400557	0.178814	0.0250856	0.2049945
ENSG00000105552	BCAT2	19	49306321	C	T	0.497018	0.0239832	0.0142965	0.05600025	-0.238613	0.00839209	7.89E-178	-0.100511	0.0600192	0.09400386	0.8836349
ENSG00000105556	MIER2	19	325186	G	A	0.375746	-0.002804	0.0144081	0.9699999	0.0857305	0.00813028	5.38E-26	-0.0327071	0.168091	0.8457221	0.458453
ENSG00000105559	PLEKHA4	19	49356121	G	T	0.171968	0.00605641	0.0194962	0.5300002	-0.152265	0.0114926	4.57E-40	-0.0397753	0.128076	0.7561349	0.9356518
ENSG00000105568	PPP2R1A	19	52711989	C	T	0.284294	-0.00600127	0.0161605	0.5400003	0.156677	0.00883466	2.28E-70	-0.0383034	0.103168	0.7104343	0.8632136
ENSG00000105576	TNPO2	19	12822416	T	C	0.00795229	0.130258	0.0675844	0.04	0.569998	0.0473633	2.34E-33	0.228524	0.12008	0.05702911	0.5244054
ENSG00000105583	WDR83OS	19	12780527	A	G	0.0248509	-0.0189649	0.0470419	0.6800001	-0.771699	0.0310293	1.57E-136	0.0245755	0.0609669	0.6868775	0.8100674
ENSG00000105607	GCDH	19	13013430	A	G	0.478131	0.01244	0.0140568	0.4500005	-0.458059	0.00775151	0	-0.0271581	0.0306912	0.3762204	0.4960369
ENSG00000105609	LILRB5	19	54757715	G	C	0.105368	0.00865783	0.0219628	0.4799997	0.133973	0.0133957	1.51E-23	0.0646237	0.164062	0.693656	0.8192529
ENSG00000105610	KLF1	19	12996616	C	G	0.229622	0.0199986	0.0170104	0.14	-0.0580916	0.0092329	3.14E-10	-0.34426	0.297889	0.2478175	0.8875986
ENSG00000105612	DNASE2	19	12989153	A	G	0.243539	0.0269551	0.016738	0.05	-0.53859	0.00854904	0	-0.0500476	0.0310876	0.1074224	0.6023441
ENSG00000105618	PRPF31	19	54626988	C	T	0.105368	-0.015817	0.0231433	0.6499995	-0.594189	0.0150984	0	0.0266195	0.0389553	0.4943955	0.5720182
ENSG00000105619	TFPT	19	54614687	G	A	0.124254	0.00586539	0.0206424	0.6300007	-0.585632	0.0171305	3.83E-256	-0.0100155	0.0352493	0.7763084	0.09150218
ENSG00000105639	JAK3	19	17947234	T	C	0.116302	0.012682	0.021851	0.4799997	-0.0928101	0.0156509	3.03E-09	-0.136645	0.236563	0.5635171	0.9187982
ENSG00000105640	RPL18A	19	17972823	A	C	0.0139165	0.0545166	0.0610745	0.33	-0.315637	0.0498558	2.44E-10	-0.172719	0.19541	0.3767597	0.3307761
ENSG00000105641	SLC5A5	19	17994382	T	C	0.054672	0.00651214	0.0296876	0.84	0.117104	0.0170313	6.16E-12	0.0556099	0.253644	0.82646	0.5223479
ENSG00000105643	ARRDC2	19	18118426	T	C	0.253479	0.015619	0.0170214	0.3100002	-0.260054	0.00974735	8.16E-157	-0.0600607	0.0654921	0.3591074	0.220376
ENSG00000105649	RAB3A	19	18311239	A	G	0.241551	-0.00190456	0.0172239	0.81	0.0598532	0.00950292	3.01E-10	-0.0318205	0.287813	0.9119656	0.1556331
ENSG00000105655	ISYNA1	19	18547154	G	A	0.371769	-0.00182793	0.014725	0.9199999	0.048344	0.00830738	5.91E-09	-0.0378109	0.304657	0.9012285	0.5715388
ENSG00000105656	ELL	19	18593205	A	G	0.343936	0.00463748	0.0149392	0.5500004	0.441471	0.00800387	0	0.0105046	0.0338402	0.7562426	0.4748727
ENSG00000105662	CRTC1	19	18843745	A	G	0.0178926	0.0896039	0.0512636	0.08600031	-0.265603	0.0456033	5.74E-09	-0.337361	0.201513	0.09410343	0.1428057
ENSG00000105663	NA	19	36219350	G	C	0.351889	-0.0363812	0.0150693	0.002999991	0.121986	0.00924126	8.75E-40	-0.29824	0.125582	0.01755534	0.50836
ENSG00000105669	COPE	19	19020264	G	C	0.343936	0.00259935	0.0149311	0.7899998	-0.201611	0.0123495	6.51E-60	-0.0128929	0.0740632	0.8618028	0.1190153
ENSG00000105671	DDX49	19	19034960	C	T	0.487078	0.0140982	0.014209	0.4400003	-0.125059	0.0118297	4.04E-26	-0.112732	0.114117	0.3232209	0.3433186
ENSG00000105672	ETV2	19	36134210	A	G	0.444334	0.0292277	0.0143469	0.02399993	-0.078151	0.00875092	4.24E-19	-0.37399	0.188295	0.04701209	0.5949339
ENSG00000105676	ARMC6	19	19157473	G	A	0.161034	-0.0264992	0.0184967	0.08999948	-0.123454	0.0102499	2.08E-33	0.214648	0.150882	0.1548474	0.9606296
ENSG00000105677	TMEM147	19	36037462	A	G	0.380716	-0.0133986	0.0146888	0.2300001	-0.201443	0.00878106	1.83E-116	0.0665132	0.0729756	0.3620614	0.5934788
ENSG00000105695	MAG	19	35793867	G	T	0.194831	0.0147131	0.0173473	0.3100002	0.103988	0.0104594	2.73E-23	0.141489	0.167427	0.3980663	0.2518913
ENSG00000105698	USF2	19	35765299	G	A	0.247515	0.00666712	0.0166489	0.84	-0.144237	0.00921781	3.45E-55	-0.0462235	0.115466	0.6889186	0.2462511
ENSG00000105699	LSR	19	35749049	C	T	0.363817	0.0139111	0.0149347	0.5099998	-0.194065	0.0082384	1.08E-122	-0.0716825	0.0770172	0.351991	0.1952187
ENSG00000105700	KXD1	19	18674384	G	T	0.404573	0.00979251	0.0144769	0.6200004	0.0997604	0.00986204	4.71E-24	0.0981603	0.145441	0.4997292	0.7626967
ENSG00000105705	SUGP1	19	19409475	T	C	0.16998	-0.044022	0.018961	0.02	0.0827535	0.0129262	1.53E-10	-0.531965	0.243728	0.02906393	0.163651
ENSG00000105707	HPN	19	35544442	G	A	0.374751	0.013431	0.0145895	0.32	0.052008	0.00825235	2.93E-10	0.258249	0.283501	0.3623342	0.06001559
ENSG00000105708	ZNF14	19	19832593	G	A	0.237575	0.0426925	0.0161686	0.002900013	0.0725804	0.00911197	1.65E-15	0.588209	0.234689	0.01219883	0.169268
ENSG00000105717	PBX4	19	19701120	A	C	0.258449	0.0223725	0.0161773	0.1199999	-0.14081	0.00908396	3.42E-54	-0.158884	0.115343	0.1683623	0.2875265
ENSG00000105726	ATP13A1	19	19765254	G	A	0.082505	-0.0195967	0.0254809	0.3800004	0.7374	0.0143534	0	-0.0265754	0.0345589	0.4419005	0.4264478
ENSG00000105732	ZNF574	19	42579165	A	G	0.10338	0.0268795	0.0225442	0.2300001	0.109254	0.0132748	1.87E-16	0.246028	0.208501	0.2380065	0.9094593
ENSG00000105750	ZNF85	19	21119765	C	A	0.130219	-0.0113409	0.0214779	0.4299995	-0.476436	0.0121571	0	0.0238036	0.0450845	0.5975142	0.05455574

ENSG00000105755	ETHE1	19	44021133	T	C	0.170974	0.00831696	0.0181569	0.5700002	0.15185	0.011228	1.13E-41	0.0547709	0.11964	0.6470975	0.8130946
ENSG00000105767	CADM4	19	44135255	G	A	0.149105	-0.0343661	0.0195864	0.04	0.134159	0.0118202	7.42E-30	-0.256159	0.147728	0.08291935	0.8157834
ENSG00000105771	SMG9	19	44247221	G	A	0.238569	0.0115452	0.0172831	0.58	0.156306	0.0107637	8.84E-48	0.0738629	0.110689	0.5045808	0.9053801
ENSG00000105778	AVL9	7	32806777	G	A	0.257455	-0.00661785	0.0159653	0.5400003	-0.278975	0.00941776	7.74E-193	0.023722	0.0572341	0.678527	0.5865987
ENSG00000105784	RUNDC3B	7	87359237	A	G	0.0308151	0.0591597	0.0488272	0.16	0.454197	0.028554	5.70E-57	0.130251	0.107814	0.2270034	0.08147953
ENSG00000105793	GTPBP10	7	89992653	A	G	0.254473	-0.0503038	0.0175319	0.003799969	-0.272873	0.00979944	1.21E-170	0.184349	0.0645894	0.004315072	0.6275238
ENSG00000105808	RASA4	7	102251704	C	T	0.217694	0.0207167	0.0169802	0.25	-0.315244	0.0155612	3.00E-91	-0.0657163	0.0539612	0.2232833	0.03926787
ENSG00000105810	CDK6	7	92350071	T	C	0.0815109	0.0309299	0.0273319	0.35	-0.0739475	0.0134778	4.10E-08	-0.418268	0.377392	0.2677271	0.4930301
ENSG00000105819	PMPCB	7	102953913	C	G	0.186879	0.00100954	0.0176907	0.8	0.0675035	0.00959784	2.02E-12	0.0149554	0.262079	0.954494	0.92256
ENSG00000105821	DNAJC2	7	102969120	C	G	0.281312	-0.000342852	0.0155739	0.7099994	0.0891764	0.0086739	8.58E-25	-0.00384465	0.174642	0.9824364	0.9449796
ENSG00000105829	BET1	7	93612884	G	A	0.332008	0.00244266	0.0146598	0.8499999	-0.247147	0.00863707	4.41E-180	-0.00988343	0.0593171	0.8676689	0.8211256
ENSG00000105835	NAMPT	7	105907751	A	T	0.326044	-0.00638181	0.0146541	0.6800001	0.0850761	0.00824988	6.19E-25	-0.075013	0.172401	0.6634835	0.3964346
ENSG00000105849	POLR1F	7	19741897	T	C	0.083499	-0.0234185	0.0248025	0.33	-0.159296	0.0130826	4.16E-34	0.147013	0.156168	0.3465137	0.3259103
ENSG00000105851	PIK3CG	7	106526656	A	G	0.428429	0.0298569	0.0143977	0.04700023	0.10421	0.00806691	3.55E-38	0.286507	0.139929	0.04060769	0.2005685
ENSG00000105854	PON2	7	95049342	G	T	0.129225	-0.00756068	0.0208216	0.89	0.646868	0.0120305	0	-0.0116881	0.0321891	0.7165236	0.7997737
ENSG00000105856	HBP1	7	106826190	G	A	0.361829	-0.0138933	0.01499	0.3700002	-0.151043	0.00840662	3.52E-72	0.0919822	0.0993749	0.3546502	0.4612139
ENSG00000105865	DUS4L	7	107211417	A	G	0.054672	-0.0336189	0.030621	0.2999998	0.230556	0.0177046	9.13E-39	-0.145817	0.133285	0.2739453	0.1680774
ENSG00000105866	SP4	7	21511046	G	T	0.261431	0.0105136	0.015922	0.4899999	-0.176929	0.00902637	1.50E-85	-0.0594226	0.0900417	0.5092893	0.5078879
ENSG00000105875	WDR91	7	134882453	C	T	0.331014	-0.0328261	0.0152897	0.01400006	-0.140609	0.00843919	2.50E-62	0.233457	0.109638	0.0332264	0.7591249
ENSG00000105877	DNAH11	7	21762145	C	T	0.332008	-0.0171755	0.0152751	0.28	-0.0583067	0.0089999	9.26E-11	0.294571	0.265895	0.2679266	0.1225146
ENSG00000105879	CBLL1	7	107392136	A	G	0.264414	-0.022611	0.0164678	0.09099971	-0.170492	0.00888636	4.86E-82	0.132622	0.0968369	0.1708304	0.4611027
ENSG00000105887	MTPN	7	135636805	G	A	0.100398	0.00618925	0.0249466	0.81	0.145002	0.013888	1.61E-25	0.0426839	0.172092	0.8041113	0.3984501
ENSG00000105889	STEAP1B	7	22565803	G	T	0.229622	-0.0125262	0.017063	0.5	-0.185633	0.0193268	7.62E-22	0.0674781	0.0921858	0.4641808	0.7975406
ENSG00000105926	PALS2	7	24671023	A	G	0.132207	-0.0163984	0.0211766	0.3400001	0.182449	0.0178421	1.52E-24	-0.0898792	0.116401	0.4400236	0.08050303
ENSG00000105928	GSDME	7	24773608	G	C	0.473161	0.0236136	0.0143182	0.04	0.496595	0.00750669	0	0.047551	0.0288417	0.09921127	0.7453594
ENSG00000105939	ZC3HAV1	7	138761365	G	A	0.0616302	-0.0199042	0.0341078	0.3900004	-0.35802	0.017369	2.12E-94	0.0555951	0.0953059	0.5596687	0.9371333
ENSG00000105948	IFT56	7	138847611	T	C	0.159046	0.0143843	0.0184334	0.2999998	0.28345	0.0103231	5.61E-166	0.0507472	0.0650585	0.4353767	0.7427479
ENSG00000105953	OGDH	7	44697418	T	G	0.429423	-0.00371792	0.0143062	0.9299999	-0.116705	0.00807503	2.41E-47	0.0318574	0.122604	0.7949874	0.8903996
ENSG00000105963	ADAP1	7	966291	A	G	0.356859	0.00866226	0.0149552	0.6100002	0.306398	0.0113213	2.63E-161	0.0282713	0.0488209	0.5625343	0.7129994
ENSG00000105967	TFEC	7	115687576	A	G	0.222664	0.00907217	0.0164749	0.5999997	-0.113124	0.00941071	2.76E-33	-0.0801965	0.145788	0.5822581	0.3916732
ENSG00000105968	H2AZ2	7	44877036	C	T	0.167992	-0.00372769	0.0190083	0.8	-0.103732	0.010386	1.73E-23	0.0359359	0.18328	0.8445548	0.01861532
ENSG00000105971	CAV2	7	116038014	T	G	0.0715706	0.0395832	0.0251428	0.08999948	0.462266	0.0136606	5.20E-251	0.0856286	0.0544492	0.1158035	0.03035036
ENSG00000105974	CAV1	7	116183036	T	A	0.173956	-0.0230491	0.0183001	0.2200002	0.0920863	0.0104073	8.89E-19	-0.250299	0.200731	0.2124205	0.000749199
ENSG00000105982	RNF32	7	156451399	C	T	0.158052	-0.0239285	0.0199452	0.28	-0.150077	0.0108988	3.86E-43	0.159442	0.133403	0.2320149	0.5172458
ENSG00000105983	LMBR1	7	156579747	C	T	0.158052	-0.0225962	0.0199909	0.32	-0.461469	0.0115808	0	0.0489658	0.0433375	0.2585311	0.3031655
ENSG00000105991	HOXA1	7	27134113	C	T	0.510934	-0.00976203	0.0142234	0.56	0.179294	0.00789869	4.56E-114	-0.0544471	0.0793664	0.492699	0.5756829
ENSG00000105993	DNAJB6	7	157169104	C	T	0.468191	-0.015201	0.0144458	0.32	0.475731	0.00758834	0	-0.0319529	0.0303698	0.2927399	0.2877281
ENSG00000105996	HOXA2	7	27141075	C	T	0.50994	-0.0113182	0.0142256	0.4899999	0.353394	0.00776388	0	-0.0320271	0.0402604	0.4263224	0.8365156
ENSG00000105997	HOXA3	7	27169001	C	T	0.50994	-0.0113182	0.0142256	0.4899999	0.107562	0.00875326	1.05E-34	-0.105225	0.132532	0.427219	0.8906499
ENSG00000106003	LFNG	7	2560487	G	C	0.0695825	-0.00528619	0.0238724	0.91	-0.491017	0.0154483	1.06E-221	0.0107658	0.0486195	0.8247578	0.4611647
ENSG00000106004	HOXA5	7	27181979	C	T	0.50994	-0.0113182	0.0142256	0.4899999	0.218388	0.00805299	5.89E-162	-0.0518261	0.0651671	0.4264508	0.8734394
ENSG00000106009	BRAT1	7	2586436	C	T	0.152087	0.010753	0.018563	0.5099998	-0.283661	0.0118885	7.94E-126	-0.0379079	0.06546	0.5625217	0.4618955
ENSG00000106012	IQCE	7	2626500	G	A	0.0695825	-0.00513363	0.0236044	0.91	-0.248034	0.0156016	6.54E-57	0.0206973	0.0951748	0.8278454	0.3905338
ENSG00000106018	VIPR2	7	158879257	C	T	0.496024	0.00444216	0.0141958	0.6300007	-0.171352	0.00945309	1.97E-73	-0.0259241	0.082858	0.7543763	0.4521086
ENSG00000106034	CPED1	7	120783114	C	T	0.446322	-0.0194457	0.0143183	0.2300001	-0.521041	0.00830438	0	0.0373208	0.0274866	0.1745325	0.9395618
ENSG00000106049	HIBADH	7	27633837	G	A	0.248509	0.00387227	0.0163655	0.5500004	0.382505	0.00892882	0	0.0101234	0.0427857	0.8129608	0.3059091
ENSG00000106052	TAX1BP1	7	27829944	A	C	0.508946	0.0215597	0.0141925	0.1900002	-0.0854432	0.0079454	5.69E-27	-0.252328	0.167754	0.1325408	0.2364447
ENSG00000106066	CPVL	7	29134957	A	G	0.489066	0.0368067	0.0142848	0.005800027	0.539428	0.00737966	0	0.0682328	0.0264978	0.01002301	0.5891064
ENSG00000106069	CHN2	7	29357950	A	G	0.213718	-0.0109053	0.0171526	0.4899999	-0.54407	0.00938275	0	0.0200439	0.0315284	0.524944	0.02112641
ENSG00000106070	GRB10	7	50759459	A	C	0.0765408	-0.02309	0.0351297	0.4299995	0.258937	0.0164098	4.31E-56	-0.0891722	0.135786	0.511368	0.1965039

ENSG00000106077	ABHD11	7	73151810	A	G	0.355865	0.0261584	0.0149392	0.1199999	-0.450665	0.00796843	0	-0.058044	0.0331651	0.08009207	0.146277
ENSG00000106078	COBL	7	51234212	T	C	0.539761	-0.0193736	0.0142013	0.1199999	-0.161	0.00802935	1.96E-89	0.120333	0.0884109	0.1734926	0.4170583
ENSG00000106086	PLEKHA8	7	30118558	A	G	0.223658	0.00065333	0.0170829	0.95	0.106698	0.0095361	4.63E-29	0.00612319	0.160107	0.9694928	0.3779851
ENSG00000106089	STX1A	7	73123769	C	T	0.350895	-0.0138438	0.0150088	0.2399999	-0.166378	0.0121071	5.67E-43	0.083207	0.0904121	0.3574118	0.04988225
ENSG00000106100	NOD1	7	30491271	C	A	0.267396	-0.00191619	0.0160592	0.8499999	0.140795	0.00902535	7.28E-55	-0.0136097	0.114064	0.9050243	0.5604124
ENSG00000106105	GARS1	7	30653973	G	C	0.0656064	-0.0309643	0.0263029	0.2099999	-0.171913	0.0140172	1.41E-34	0.180116	0.153705	0.2412637	0.4016674
ENSG00000106113	CRHR2	7	30715972	G	A	0.352883	-0.0125578	0.0148882	0.4299995	0.116356	0.00857148	5.65E-42	-0.107926	0.1282	0.399872	0.02877206
ENSG00000106123	EPHB6	7	142560819	T	A	0.110338	-0.0355822	0.0245092	0.17	0.362208	0.019296	1.30E-78	-0.0982368	0.0678681	0.1477662	0.7978987
ENSG00000106133	NA	7	72421724	T	C	0.322068	-0.0188098	0.0152005	0.1900002	0.581128	0.019597	3.02E-193	-0.0323678	0.0261797	0.216321	0.9606805
ENSG00000106144	CASP2	7	142995048	A	G	0.0258449	0.0535452	0.0484048	0.3700002	0.317954	0.030549	2.28E-25	0.168405	0.153096	0.2713318	0.7460133
ENSG00000106153	CHCHD2	7	56171765	C	T	0.222664	0.0267223	0.0172085	0.089	1.07329	0.0090931	0	0.0248977	0.0160349	0.12049	0.8052015
ENSG00000106211	HSPB1	7	75932736	G	C	0.257455	0.0418044	0.0165654	0.01	-0.124305	0.0136593	9.00E-20	-0.336304	0.138293	0.01502289	0.1063597
ENSG00000106236	NPTX2	7	98252894	T	C	0.0347913	0.0264768	0.036073	0.4899999	0.173691	0.0201704	7.23E-18	0.152436	0.208438	0.4645797	0.4110814
ENSG00000106244	PDAP1	7	98998061	T	C	0.136183	-0.0028449	0.0214222	0.8200001	-0.0796547	0.0118319	1.67E-11	0.0357154	0.268991	0.8943708	0.7003462
ENSG00000106245	BUD31	7	99011751	T	C	0.0198807	-0.080289	0.0476451	0.1100001	0.330655	0.0220355	6.75E-51	-0.242818	0.144999	0.09400874	0.5664666
ENSG00000106246	PTCD1	7	99039091	T	C	0.028827	0.010639	0.0433875	0.7099994	-0.532158	0.0363	1.16E-48	-0.0199922	0.0815427	0.8063213	0.9522354
ENSG00000106261	ZKSCAN1	7	99626258	T	C	0.393638	-0.0110968	0.0142963	0.4500005	0.202105	0.00796863	6.53E-142	-0.0549061	0.0707701	0.4378449	0.01310951
ENSG00000106263	EIF3B	7	2407050	A	G	0.215706	-0.00791544	0.0174315	0.58	0.237736	0.0102113	6.81E-120	-0.033295	0.0733367	0.6498274	0.2865956
ENSG00000106266	SNX8	7	2342679	A	G	0.326044	-0.00023936	0.0155866	0.95	-0.0850806	0.00880078	4.15E-22	0.00281333	0.183198	0.9877476	0.2081624
ENSG00000106268	NUDT1	7	2286319	A	G	0.329026	-0.0226271	0.0153756	0.0519996	0.221166	0.00829573	1.36E-156	-0.102308	0.0696266	0.1417279	0.1210869
ENSG00000106290	TAF6	7	99711078	G	C	0.394632	-0.0101638	0.0142998	0.5	0.0922774	0.00804665	1.91E-30	-0.110144	0.155263	0.4780738	0.05023094
ENSG00000106299	WASL	7	123355555	G	A	0.267396	-0.00414363	0.0168808	0.7899998	-0.195434	0.00926704	9.99E-99	0.0212021	0.0863816	0.80611	0.1054269
ENSG00000106305	AIMP2	7	6056170	G	T	0.43837	0.0117982	0.0145027	0.4400003	0.264439	0.00849869	1.50E-212	0.0446159	0.0548619	0.4160807	0.1729349
ENSG00000106327	TFR2	7	100229220	T	C	0.198807	0.0340102	0.0174465	0.03899959	-0.102981	0.00970186	2.55E-26	-0.330256	0.172247	0.05519557	0.7815144
ENSG00000106330	MOSPD3	7	100211366	A	C	0.186879	0.0340197	0.0179123	0.03400008	-0.310945	0.00971736	1.13E-224	-0.109407	0.0577074	0.05797322	0.9702298
ENSG00000106333	PCOLCE	7	100202799	A	G	0.186879	0.031878	0.0178226	0.04600023	0.0864737	0.010056	8.03E-18	0.368644	0.210516	0.07992035	0.9132213
ENSG00000106341	PPP1R17	7	31737199	C	T	0.168986	0.00147196	0.0181456	0.8	-0.483134	0.0109778	0	-0.00304669	0.0375582	0.9353471	0.7163091
ENSG00000106344	RBM28	7	127967199	G	A	0.054672	0.0108588	0.0271461	0.4400003	0.370337	0.0160377	5.62E-118	0.0293214	0.0733121	0.6891909	0.6252377
ENSG00000106346	USP42	7	6172855	G	C	0.0954274	-0.0295214	0.0254252	0.16	0.115123	0.0143549	1.06E-15	-0.256433	0.223155	0.2505037	0.3411111
ENSG00000106348	IMPDH1	7	128041318	T	A	0.0477137	-0.00321335	0.0314325	0.81	0.275722	0.0179431	2.75E-53	-0.0116543	0.114003	0.9185758	0.30587
ENSG00000106351	AGFG2	7	100151338	T	C	0.0646123	-0.0614614	0.0352161	0.07000032	0.134782	0.0217096	5.35E-10	-0.456007	0.27141	0.09293007	0.09961483
ENSG00000106355	LSM5	7	32529923	T	G	0.379722	0.022247	0.0147547	0.14	0.0657502	0.00829006	2.17E-15	0.338357	0.228425	0.1385371	0.9312963
ENSG00000106366	SERPINE1	7	100776458	G	A	0.209742	0.0030828	0.0178843	0.9299999	-0.0909017	0.0097321	9.60E-21	-0.0339135	0.196777	0.8631661	0.4306008
ENSG00000106367	AP1S1	7	100801277	A	G	0.186879	-0.0350302	0.0200523	0.08500021	0.244551	0.0112545	1.09E-104	-0.143243	0.0822608	0.08162693	0.5178857
ENSG00000106392	C1GALT1	7	7242408	G	A	0.358847	0.0273152	0.0149671	0.05600025	0.475629	0.00778767	0	0.0574296	0.0314821	0.06812155	0.6894507
ENSG00000106397	PLOD3	7	100855479	C	T	0.250497	0.00561467	0.0169192	0.95	0.0631085	0.00960906	5.11E-11	0.0889686	0.268439	0.7403203	0.09109533
ENSG00000106399	RPA3	7	7717193	A	G	0.0914513	-0.0122331	0.0246216	0.84	0.254536	0.0198956	1.78E-37	-0.0480604	0.0968043	0.6195629	0.4827595
ENSG00000106400	ZNHIT1	7	100864210	C	T	0.250497	0.00561467	0.0169192	0.95	0.293669	0.0140696	9.50E-97	0.0191191	0.0576205	0.7400327	0.0463952
ENSG00000106404	CLDN15	7	100878737	T	C	0.0596421	0.0653031	0.0314145	0.03400008	0.542719	0.0182155	4.63E-195	0.120326	0.0580243	0.03810575	0.2054029
ENSG00000106415	GLCCI1	7	8071163	C	A	0.0695825	-0.073038	0.0301289	0.009200046	-0.258192	0.0196484	1.93E-39	0.282882	0.118661	0.01712761	0.5101897
ENSG00000106443	PHF14	7	11111374	C	T	0.112326	-0.0047846	0.0224967	0.5	-0.251439	0.0125216	1.10E-89	0.0190289	0.0894769	0.8315858	0.07007717
ENSG00000106459	NRF1	7	129324238	T	C	0.0695825	0.0062299	0.0298991	0.91	0.148049	0.0173497	1.42E-17	0.04208	0.202014	0.8349931	0.7449752
ENSG00000106460	TMEM106B	7	12263876	A	G	0.418489	0.0136289	0.0143295	0.2099999	0.112307	0.00801883	1.44E-44	0.121354	0.127886	0.3426602	0.4246169
ENSG00000106462	EZH2	7	148542944	T	A	0.331014	0.0101846	0.0153502	0.3900004	-0.192849	0.00899986	7.33E-102	-0.0528113	0.0796351	0.5072246	0.5058246
ENSG00000106477	CEP41	7	130059324	A	G	0.185885	0.0141397	0.018063	0.5199996	0.230395	0.0111182	2.18E-95	0.0613714	0.0784559	0.4340729	0.2019135
ENSG00000106479	ZNF862	7	149550012	G	T	0.147117	0.016124	0.0196715	0.5500004	-0.143829	0.0120535	8.00E-33	-0.112105	0.137092	0.4135079	0.9679274
ENSG00000106484	MEST	7	130136089	G	A	0.193837	-0.0216713	0.0178715	0.2300001	-0.167309	0.00977132	1.01E-65	0.129529	0.107085	0.2264369	0.05131207
ENSG00000106524	ANKMY2	7	16662421	T	C	0.194831	0.00287681	0.0182751	0.98	-0.166276	0.0100913	5.35E-61	-0.0173014	0.109913	0.8749219	0.5522227
ENSG00000106526	ACTR3C	7	149980909	G	A	0.0964215	-0.0286443	0.0254217	0.3800004	-0.139058	0.0157912	1.30E-18	0.205989	0.184305	0.263715	0.3963233
ENSG00000106537	TSPAN13	7	16808660	C	T	0.522863	-0.00669374	0.0142922	0.98	-0.0773313	0.00795344	2.41E-22	0.0865593	0.185032	0.6399228	0.7662292

ENSG00000106538	RARRES2	7	150037085	C	T	0.244533	-0.000427921	0.0163773	0.9299999	0.0722812	0.00951218	2.99E-14	-0.00592023	0.226579	0.9791546	0.1006719
ENSG00000106546	AHR	7	17362011	G	A	0.0755467	0.00703424	0.0304942	0.8499999	0.477504	0.0164998	3.75E-184	0.0147313	0.0638637	0.8175732	0.5213698
ENSG00000106554	CHCHD3	7	132618238	G	T	0.395626	0.0130203	0.0147023	0.4899999	0.102233	0.012086	2.70E-17	0.127359	0.144598	0.3784358	0.503516
ENSG00000106560	GIMAP2	7	150386757	C	G	0.336978	-0.00853641	0.0150203	0.4899999	-0.343127	0.00823936	0	0.0248783	0.0437788	0.5698503	0.6037314
ENSG00000106565	TMEM176B	7	150493410	G	A	0.373757	-0.000126326	0.0147444	0.9699999	1.05108	0.0106834	0	-0.000120187	0.0140279	0.993164	0.3511479
ENSG00000106571	GLI3	7	42139008	G	A	0.450298	-0.0125175	0.0143694	0.3100002	-0.0938466	0.00814189	9.71E-31	0.133383	0.153552	0.3850415	0.5846996
ENSG00000106588	PSMA2	7	42964141	A	G	0.082505	0.0446688	0.0238154	0.04499974	0.461732	0.0155999	1.57E-192	0.0967418	0.0516818	0.06122431	0.3709049
ENSG00000106591	MRPL32	7	42980178	C	T	0.127237	0.0289339	0.0210502	0.1499999	0.283779	0.0136612	7.66E-96	0.101959	0.0743405	0.170213	0.472686
ENSG00000106603	COA1	7	43708685	A	G	0.195825	0.0087065	0.0181539	0.8200001	0.4124	0.0112337	4.86E-295	0.0211118	0.0440239	0.6315453	0.2174485
ENSG00000106605	BLVRA	7	43822609	G	A	0.253479	-0.0240542	0.0166643	0.1199999	-0.61591	0.00880663	0	0.0390547	0.0270621	0.1489783	0.3961537
ENSG00000106608	URGCP	7	43940751	C	T	0.0656064	0.0195651	0.0330664	0.6200004	0.336396	0.018797	1.26E-71	0.058161	0.0983499	0.5542738	0.8940289
ENSG00000106609	TMEM248	7	66404875	G	A	0.114314	-0.0254323	0.0226574	0.17	-0.181178	0.0161107	2.43E-29	0.140372	0.125677	0.2640276	NA
ENSG00000106610	NA	7	66777060	T	A	0.392644	-0.03976	0.0146785	0.00329997	-0.613053	0.00825931	0	0.0648557	0.0239592	0.006791026	0.8846502
ENSG00000106615	RHEB	7	151190152	C	T	0.536779	-0.00694246	0.0141996	0.6499995	0.106152	0.0118104	2.52E-19	-0.0654011	0.133964	0.6254099	0.7865727
ENSG00000106617	PRKAG2	7	151413703	C	T	0.32008	0.00185658	0.0149859	0.9299999	0.201831	0.00880641	3.03E-116	0.0091987	0.074251	0.901405	0.6550236
ENSG00000106624	AEBP1	7	44149060	A	G	0.413519	-0.0148858	0.0143826	0.2099999	-0.202967	0.00917495	1.95E-108	0.0733412	0.0709394	0.3012036	0.3741664
ENSG00000106628	POLD2	7	44159121	T	C	0.0159046	0.0213205	0.054291	0.64	-0.60956	0.0523084	2.21E-31	-0.0349769	0.0891164	0.6946999	0.6337285
ENSG00000106633	GCK	7	44210820	A	G	0.531809	0.0112632	0.0143535	0.3400001	-0.102395	0.00933184	5.18E-28	-0.109998	0.140536	0.4338023	0.4102021
ENSG00000106635	BCL7B	7	72961509	G	A	0.243539	0.00237353	0.0159001	0.7400005	-0.0983506	0.00918255	9.08E-27	-0.0241334	0.161683	0.881346	0.8171716
ENSG00000106636	YKT6	7	44247230	G	T	0.0775348	-0.0394497	0.0315515	0.14	0.0973056	0.0157959	7.27E-10	-0.40542	0.330863	0.2204463	0.3823146
ENSG00000106638	TBL2	7	72988555	A	G	0.0437376	0.0271214	0.0352546	0.4100001	0.6375	0.0227071	1.98E-173	0.0425434	0.0553221	0.441886	0.7386321
ENSG00000106665	CLIP2	7	73762039	C	T	0.0666004	0.0218619	0.0270467	0.4899999	-0.188777	0.0145857	2.59E-38	-0.115808	0.143552	0.4198219	0.2968352
ENSG00000106682	EIF4H	7	73600003	A	C	0.27833	-0.0371477	0.0155828	0.01499996	-0.183529	0.0143996	3.31E-37	0.202408	0.086379	0.01911628	0.7209488
ENSG00000106683	LIMK1	7	73517059	C	G	0.143141	-0.0292798	0.0187284	0.1199999	-0.703835	0.0147358	0	0.0416004	0.0266233	0.1181576	0.1671614
ENSG00000106686	SPATA6L	9	4610030	A	G	0.176938	0.0204059	0.0186251	0.14	0.168911	0.0117752	1.15E-46	0.120809	0.110587	0.2746436	0.1463245
ENSG00000106692	FKTN	9	108361905	G	A	0.275348	-0.00829421	0.0151891	0.7600007	-0.118233	0.00866749	2.28E-42	0.0701512	0.12857	0.5853228	0.9857572
ENSG00000106701	FSD1L	9	108262395	T	A	0.461233	0.000754348	0.0143193	0.95	0.0918146	0.00874908	9.19E-26	0.00821599	0.155961	0.957987	0.491096
ENSG00000106714	CNTNAP3	9	39180538	G	A	0.21173	-0.0222319	0.0186999	0.3400001	-0.308983	0.035667	4.60E-18	0.0719518	0.061088	0.2388611	0.6355698
ENSG00000106733	NMRK1	9	77689311	T	A	0.368787	-0.01002	0.0144651	0.5500004	-0.829753	0.00751485	0	0.0120759	0.0174334	0.4885053	0.6532473
ENSG00000106771	TMEM245	9	111829828	A	G	0.454274	0.00970633	0.0142663	0.3700002	0.413468	0.0085626	0	0.0234754	0.0345075	0.4963144	0.209401
ENSG00000106772	PRUNE2	9	79373647	A	C	0.228628	0.00280876	0.0165604	0.8700001	1.06353	0.00725968	0	0.00264099	0.0155712	0.8653193	0.9695837
ENSG00000106780	MEGF9	9	123419919	T	C	0.302187	-0.0206637	0.0154929	0.2300001	-0.449961	0.0083192	0	0.0459233	0.0344421	0.1824178	0.9574695
ENSG00000106785	TRIM14	9	100856525	C	A	0.232604	0.00751733	0.0166323	0.5700002	0.220975	0.0105877	9.87E-97	0.034019	0.0752855	0.6513656	0.7010303
ENSG00000106789	CORO2A	9	100919089	G	A	0.129225	0.00456596	0.0218125	0.7700005	-0.526202	0.0122892	0	-0.0086772	0.0414532	0.8341944	0.2776335
ENSG00000106799	TGFBR1	9	101891397	T	C	0.0347913	-0.00736794	0.039573	0.9400001	0.43106	0.026181	6.59E-61	-0.0170926	0.0918098	0.8523082	0.1636546
ENSG00000106804	C5	9	123763585	T	C	0.45825	0.00496317	0.0142983	0.6200004	0.558511	0.00734675	0	0.00888644	0.025601	0.7285068	0.9145525
ENSG00000106829	TLE4	9	82264173	C	T	0.240557	0.00252535	0.0174482	0.9599999	0.171127	0.00986889	2.35E-67	0.0147572	0.101964	0.8849248	0.4808181
ENSG00000106853	PTGR1	9	114337068	T	C	0.275348	0.0110074	0.0156048	0.6100002	-0.224199	0.00927537	4.45E-129	-0.0490966	0.0696322	0.4807571	0.828515
ENSG00000106868	SUSD1	9	114870376	A	C	0.328032	0.00674888	0.0155108	0.83	-0.69727	0.00758585	0	-0.009679	0.0222453	0.6634873	0.9541513
ENSG00000106948	AKNA	9	117126560	T	C	0.409543	-0.0135977	0.0144856	0.4500005	-0.177471	0.0081037	2.60E-106	0.0766192	0.0816973	0.3483248	0.8504571
ENSG00000106952	TNFSF8	9	117674350	G	A	0.324056	0.000882778	0.0153402	0.95	0.309346	0.00849138	1.40E-290	0.00285369	0.0495892	0.9541098	0.6727245
ENSG00000106976	DNM1	9	130991592	G	A	0.0119284	-0.0284074	0.0620832	0.4500005	0.459951	0.0285737	2.68E-58	-0.0617618	0.135032	0.6473943	0.2744415
ENSG00000106991	ENG	9	130597163	C	T	0.0854871	-0.0125601	0.026443	0.4100001	-0.542477	0.0141698	0	0.0231532	0.0487487	0.6348218	0.06638347
ENSG00000106992	AK1	9	130634390	G	A	0.172962	-0.00291185	0.0190014	0.8800001	-0.431355	0.0162653	5.71E-155	0.00675048	0.0440513	0.8782079	0.01448939
ENSG00000106993	CDC37L1	9	4693978	A	C	0.314115	0.0481098	0.014975	0.000530005	0.0649225	0.00838662	9.85E-15	0.741035	0.249735	0.003004393	0.3142653
ENSG00000107014	RLN2	9	5302418	G	C	0.256461	0.00838664	0.0170015	0.81	0.157834	0.0139479	1.09E-29	0.0531358	0.10782	0.6221396	0.541057
ENSG00000107018	RLN1	9	5337421	T	C	0.260437	0.0112836	0.0168545	0.6600001	-0.209961	0.0102929	1.72E-92	-0.0537415	0.0803178	0.5034248	0.741629
ENSG00000107020	PLGRKT	9	5397925	A	G	0.353877	-0.0141281	0.0147602	0.4199997	0.654003	0.00823307	0	-0.0216025	0.0225707	0.3385138	0.3917103
ENSG00000107021	TBC1D13	9	131561097	G	A	0.289264	-0.0068084	0.0157639	0.8	0.0971061	0.00901668	4.79E-27	-0.070113	0.162467	0.6660676	0.7679766
ENSG00000107036	RIC1	9	5702791	C	T	0.0407555	0.020294	0.0384957	0.6499995	0.161036	0.0183512	1.71E-18	0.126022	0.239482	0.5987305	0.9608281

ENSG00000107077	KDM4C	9	6948255	A	G	0.205765	-0.0285794	0.0177521	0.07599937	0.128143	0.0096322	2.21E-40	-0.223028	0.139544	0.1099859	0.3645445
ENSG00000107099	DOCK8	9	340056	G	A	0.144135	0.00773062	0.0200297	0.7899998	0.577631	0.0113511	0	0.0133833	0.0346766	0.6995361	0.6300261
ENSG00000107104	KANK1	9	608198	G	A	0.472167	-0.0183629	0.0152443	0.2200002	0.387419	0.00811488	0	-0.047398	0.0393609	0.2285155	0.9573182
ENSG00000107140	TESK1	9	35607702	T	C	0.0168986	-0.0772279	0.0611025	0.2399999	-0.265177	0.0373604	1.27E-12	0.291232	0.234047	0.213377	0.3094948
ENSG00000107147	KCNT1	9	138639511	A	G	0.22167	0.021177	0.017598	0.1800002	0.300836	0.0129701	5.16E-119	0.0703939	0.0585758	0.2294569	0.07667906
ENSG00000107164	FUBP3	9	133484045	A	G	0.0994036	0.0153809	0.0271715	0.4400003	-0.0940354	0.013883	1.26E-11	-0.163565	0.289957	0.5726855	0.674851
ENSG00000107175	CREB3	9	35734666	A	G	0.4334	0.0144565	0.0143954	0.4700002	-0.072643	0.00799294	1.01E-19	-0.199008	0.199373	0.3181973	0.07687212
ENSG00000107185	RGP1	9	35753887	T	C	0.434394	0.0137971	0.01439	0.5099998	-0.264517	0.00788931	1.85E-246	-0.0521596	0.0544233	0.3378581	0.09449246
ENSG00000107201	RIGI	9	32490811	T	C	0.431412	0.0359036	0.0143687	0.005099998	-0.194472	0.0079967	1.23E-130	-0.184621	0.0742748	0.01293149	0.09047393
ENSG00000107223	EDF1	9	139758654	T	C	0.110338	0.0114579	0.0206469	0.3800004	-0.0792283	0.0137277	7.86E-09	-0.144619	0.261802	0.580676	0.6974533
ENSG00000107242	PIP5K1B	9	71472333	T	A	0.274354	-0.0100414	0.0172717	0.5999997	0.164517	0.00965689	4.43E-65	-0.0610357	0.105046	0.5612131	0.5178035
ENSG00000107249	GLIS3	9	4086259	C	G	0.419483	0.0156	0.0142754	0.1199999	-0.129779	0.00808503	5.55E-58	-0.120204	0.110252	0.2755963	0.006430565
ENSG00000107262	BAG1	9	33256289	A	G	0.0666004	-0.0627129	0.027804	0.00990011	0.358673	0.0148419	5.05E-129	-0.174847	0.077856	0.02471845	0.19565
ENSG00000107263	RAPGEF1	9	134533809	T	C	0.267396	1.58E-05	0.0152081	0.9400001	-0.665773	0.00787484	0	-2.37E-05	0.0228428	0.9991722	0.8475307
ENSG00000107281	NPDC1	9	139937288	T	C	0.10835	-0.017563	0.0243522	0.5500004	-0.696307	0.0231353	5.27E-199	0.0252231	0.0349834	0.4709089	0.1121424
ENSG00000107282	APBA1	9	72166213	G	A	0.447316	-0.00198878	0.0142615	0.9400001	0.0453515	0.00807628	1.96E-08	-0.0438526	0.314563	0.8891278	0.7965505
ENSG00000107290	SETX	9	135183557	A	G	0.0149105	0.107812	0.0539731	0.03400008	0.426356	0.0321783	4.52E-40	0.252868	0.128022	0.04824618	0.9599137
ENSG00000107317	PTGDS	9	139875921	G	A	0.435388	0.0299979	0.0143156	0.05499966	0.301629	0.0116077	7.28E-149	0.099453	0.0476151	0.03673573	0.5840755
ENSG00000107331	ABCA2	9	139912526	A	G	0.272366	0.0231388	0.0158197	0.08799946	-0.314593	0.0104477	3.45E-199	-0.0735514	0.0503455	0.1440333	0.05638727
ENSG00000107338	SHB	9	37994170	A	G	0.451292	0.0104782	0.0143061	0.6499995	0.08478	0.00806185	7.27E-26	0.123593	0.169153	0.4649876	0.5896165
ENSG00000107341	UBE2R2	9	33868983	C	G	0.316103	0.00223267	0.0154302	0.8	0.074454	0.0088155	3.02E-17	0.0299873	0.207275	0.8849686	0.7513222
ENSG00000107362	ABHD17B	9	74501607	T	A	0.154076	-0.0335161	0.0205672	0.05699936	0.089857	0.0134751	2.59E-11	-0.372994	0.235623	0.1134198	0.4413171
ENSG00000107371	EXOSC3	9	37784204	A	G	0.483101	0.020501	0.0141857	0.09200046	-0.193128	0.0078853	1.79E-132	-0.106152	0.0735799	0.1491111	0.729203
ENSG00000107372	ZFAND5	9	74973252	C	T	0.410537	0.0101824	0.0145512	0.3900004	-0.116478	0.00816472	3.56E-46	-0.0874191	0.125077	0.4846	0.5009302
ENSG00000107438	PDLIM1	10	97024055	A	T	0.201789	-0.00946492	0.0183765	0.4199997	-0.235428	0.00981472	3.78E-127	0.0402031	0.0780738	0.6065971	0.3696434
ENSG00000107443	CCNJ	10	97811889	A	G	0.496024	0.0219764	0.0142263	0.1	0.0619835	0.00793968	5.87E-15	0.354553	0.233968	0.1296736	0.02244382
ENSG00000107447	DNTT	10	98081203	G	A	0.0308151	0.00128583	0.037111	0.98	0.26458	0.0190699	9.08E-44	0.0048599	0.140264	0.9723603	0.2151274
ENSG00000107485	GATA3	10	8106364	A	G	0.290258	0.0254913	0.0158916	0.08700015	0.057808	0.00896359	1.12E-10	0.440965	0.283279	0.1195545	0.5432932
ENSG00000107521	HPS1	10	100191319	C	G	0.510934	-0.00793297	0.0141936	0.4500005	0.303767	0.00778824	0	-0.0261153	0.0467301	0.5762611	0.0705288
ENSG00000107537	PHYH	10	13332104	A	G	0.291252	0.00549809	0.0155528	0.81	-0.365766	0.00867504	0	-0.0150317	0.0425226	0.7237145	0.0313983
ENSG00000107551	RASSF4	10	45472513	A	G	0.112326	0.0138414	0.0231813	0.7099994	-0.476888	0.0129181	2.52E-298	-0.0290244	0.0486159	0.5504972	0.3697554
ENSG00000107554	DNMBP	10	101702505	T	C	0.0208748	0.00633095	0.0571498	0.9199999	-0.406341	0.0282562	6.85E-47	-0.0155804	0.140649	0.9117949	0.7786283
ENSG00000107560	RAB11FIP2	10	119785270	G	T	0.187873	-0.0155451	0.0185143	0.33	-0.13562	0.0100544	1.83E-41	0.114622	0.13678	0.402028	0.7782188
ENSG00000107566	ERLIN1	10	101928971	A	G	0.026839	0.0188692	0.0578219	0.9699999	-0.405533	0.0241671	3.40E-63	-0.0465293	0.142609	0.744219	0.8659602
ENSG00000107581	EIF3A	10	120817376	T	C	0.400596	0.00289332	0.0143606	0.6800001	-0.0945639	0.0120457	4.15E-15	-0.0305965	0.151911	0.840378	0.4915147
ENSG00000107593	PKD2L1	10	102069073	A	C	0.0576541	-0.044834	0.0297701	0.2099999	0.839483	0.0172286	0	-0.0534067	0.0354793	0.1322498	0.4629383
ENSG00000107611	CUBN	10	17018896	A	G	0.341948	-0.000570896	0.0154319	0.8800001	0.586786	0.00802677	0	-0.000972921	0.026299	0.9704894	0.7965123
ENSG00000107614	TRDMT1	10	17214153	A	G	0.151093	0.00956691	0.0197351	0.4899999	-0.237026	0.0113288	3.35E-97	-0.0403623	0.0832838	0.6279345	0.6491725
ENSG00000107623	NA	10	48432395	T	C	0.210736	-0.0232204	0.0183847	0.1499999	-0.117457	0.0150159	5.19E-15	0.197692	0.15855	0.2124419	0.4387468
ENSG00000107625	DDX50	10	70683818	A	G	0.350895	-0.0111229	0.0149762	0.4199997	-0.0867083	0.00871405	2.51E-23	0.128279	0.1732	0.4589088	0.7547926
ENSG00000107643	MAPK8	10	49581050	T	C	0.411531	0.00557766	0.0143304	0.5	-0.231227	0.00791473	1.26E-187	-0.024122	0.0619809	0.6971399	0.08000236
ENSG00000107651	SEC23IP	10	121677118	C	T	0.301193	-0.0443486	0.0162717	0.00179999	-0.0961127	0.00869956	2.24E-28	0.461423	0.174374	0.008140836	0.7461339
ENSG00000107669	ATE1	10	123594127	C	A	0.0755467	-0.019756	0.0267428	0.6200004	0.176808	0.0141345	6.67E-36	-0.111737	0.151517	0.4608451	0.7282455
ENSG00000107672	NSMCE4A	10	123725667	T	C	0.411531	-0.00714303	0.0144563	0.4700002	0.122884	0.00794512	5.83E-54	-0.0581282	0.117702	0.6214051	0.6386881
ENSG00000107679	PLEKHA1	10	124163039	C	T	0.468191	-0.0174563	0.0142123	0.2300001	-0.391313	0.00764978	0	0.0446096	0.03633	0.2194849	0.3473083
ENSG00000107719	PALD1	10	72283391	A	G	0.204771	0.00630793	0.0184664	0.64	-0.179655	0.011129	1.27E-58	-0.0351114	0.102811	0.7327173	0.09414434
ENSG00000107731	UNC5B	10	73017474	C	T	0.33002	-0.0117973	0.0151223	0.5400003	0.418761	0.0089359	0	-0.0281719	0.036117	0.4353803	0.3693616
ENSG00000107736	CDH23	10	73366196	G	A	0.440358	0.00606913	0.0142615	0.6300007	0.470521	0.0075151	0	0.0128987	0.0303107	0.6704353	0.7307828
ENSG00000107738	VSIR	10	73520285	A	G	0.207753	0.0173889	0.0183304	0.6499995	-0.141304	0.00996208	1.15E-45	-0.12306	0.130013	0.343882	0.9006898
ENSG00000107742	SPOCK2	10	73833791	T	C	0.442346	0.00268341	0.0142664	0.8	0.187588	0.00795875	7.83E-123	0.0143048	0.0760543	0.8508084	0.0724437

ENSG00000107745	MICU1	10	74256498	C	T	0.392644	0.00272104	0.0149448	0.99	0.240682	0.00947389	2.23E-142	0.0113055	0.0620951	0.8555293	0.2744606
ENSG00000107771	CCSER2	10	86183307	T	C	0.111332	-0.00929749	0.0211451	0.4799997	-0.942251	0.0116173	0	0.00986732	0.0224414	0.6601594	0.9340515
ENSG00000107779	BMPR1A	10	88600676	C	G	0.373757	0.0103053	0.0146947	0.7300002	-0.153445	0.0133092	9.40E-31	-0.0671596	0.0959424	0.4839274	0.513083
ENSG00000107789	MINPP1	10	89288924	C	A	0.00198807	-0.0326752	0.091743	0.7700005	0.5101	0.0648536	3.68E-15	-0.0640565	0.180037	0.7219937	NA
ENSG00000107796	ACTA2	10	90722989	A	G	0.531809	-0.0421434	0.0141804	0.001899984	0.891366	0.00616892	0	-0.0472795	0.015912	0.002965238	0.01971625
ENSG00000107798	LIPA	10	91073820	T	C	0.360835	0.0150205	0.014965	0.1499999	0.904107	0.00661267	0	0.0166136	0.0165527	0.3155321	0.01142082
ENSG00000107815	TWNK	10	102750641	A	G	0.182903	-0.0134292	0.0174198	0.4600002	-0.13469	0.00982189	8.46E-43	0.0997049	0.129537	0.4414775	0.7893135
ENSG00000107816	LZTS2	10	102761984	T	G	0.316103	-0.000954878	0.0150649	0.83	-0.100491	0.00859514	1.41E-31	0.00950213	0.149915	0.9494612	0.6485114
ENSG00000107819	SFXN3	10	102795994	A	C	0.217694	0.01512	0.0186031	0.2	0.258114	0.0120663	1.61E-101	0.0585787	0.0721252	0.4166874	0.3761235
ENSG00000107833	NPM3	10	103542126	C	G	0.372763	-0.00315226	0.014481	0.9199999	-0.183831	0.00820799	4.25E-111	0.0171476	0.0787771	0.8276845	0.34166
ENSG00000107854	TNKS2	10	93591551	C	G	0.501988	0.0106735	0.0141845	0.35	0.239664	0.0118932	2.62E-90	0.0445353	0.0592262	0.4520798	0.1761652
ENSG00000107863	ARHGAP21	10	24942567	C	T	0.488072	0.00706281	0.0142224	0.5199996	-0.169913	0.0117807	3.71E-47	-0.0415671	0.0837535	0.6196803	0.3693908
ENSG00000107864	CPEB3	10	93929621	G	C	0.374751	-0.0102518	0.0144723	0.5500004	-0.0928735	0.00810796	2.23E-30	0.110385	0.156126	0.479552	0.09974203
ENSG00000107872	FBXL15	10	104180919	C	G	0.0168986	0.00117225	0.0518723	0.9699999	0.771335	0.0352652	4.76E-106	0.00151977	0.0672501	0.9819703	0.7323654
ENSG00000107874	CUEDC2	10	104187710	A	G	0.244533	-0.00689039	0.0164584	0.9	0.0812555	0.00960445	2.67E-17	-0.084799	0.202799	0.6758424	0.7595486
ENSG00000107882	SUFU	10	104328518	G	A	0.427435	0.00929129	0.014235	0.6100002	0.0903266	0.00807222	4.57E-29	0.102863	0.157863	0.5146594	0.4316718
ENSG00000107890	ANKRD26	10	27335132	C	T	0.0526839	-0.00242053	0.033236	0.9400001	0.593618	0.0177757	1.63E-244	-0.00407759	0.055989	0.9419427	0.1566508
ENSG00000107897	ACBD5	10	27507602	A	G	0.309145	-0.00656565	0.015603	0.5999997	-0.0807714	0.0089758	2.28E-19	0.0812868	0.193386	0.6742409	0.5959958
ENSG00000107902	LHPP	10	126228430	A	G	0.370775	0.0163515	0.0144399	0.25	-0.373235	0.00846526	0	-0.0438102	0.0387013	0.2576303	0.8636272
ENSG00000107929	LARP4B	10	916524	T	C	0.480119	-0.0176737	0.0142103	0.2700001	-0.169005	0.00789579	1.22E-101	0.104575	0.0842242	0.214373	0.7531412
ENSG00000107937	GTPBP4	10	1050107	T	C	0.50497	0.00283901	0.0141969	0.6800001	-0.274063	0.00780118	2.28E-270	-0.010359	0.0518025	0.8415035	0.6899477
ENSG00000107949	BCCIP	10	127527189	T	C	0.558648	-0.0062567	0.0142298	0.56	-0.176116	0.00849783	2.07E-95	0.0355261	0.0808162	0.6602333	0.02362325
ENSG00000107951	MTPAP	10	30631053	A	T	0.439364	-0.022227	0.0142329	0.08799946	-0.271114	0.0087527	1.17E-210	0.081984	0.0525645	0.1188349	0.3752117
ENSG00000107954	NEURL1	10	105303022	T	C	0.16501	-0.0322344	0.0190516	0.025	-0.0620812	0.0109641	1.49E-08	0.519229	0.32029	0.104991	0.8852815
ENSG00000107957	SH3PXD2A	10	105481793	A	G	0.309145	-0.025157	0.0147952	0.09499921	-0.238653	0.00818984	1.11E-186	0.105412	0.0620999	0.08960945	0.9192223
ENSG00000107959	PITRM1	10	3197461	A	G	0.242545	-0.0129802	0.0156424	0.5	0.579708	0.0084866	0	-0.0223909	0.0269852	0.4066816	0.1853196
ENSG00000107960	STN1	10	105660131	A	G	0.537773	0.0144699	0.0142056	0.2300001	0.20687	0.0117194	9.82E-70	0.0699467	0.0687833	0.3091945	0.6286299
ENSG00000107968	MAP3K8	10	30736814	T	C	0.195825	-0.000330337	0.0189295	0.98	0.277279	0.0110016	3.66E-140	-0.00119135	0.0682688	0.9860769	0.6053834
ENSG00000108010	GLRX3	10	131958724	A	T	0.0994036	-0.0236256	0.0227824	0.1900002	-0.185796	0.0144352	6.55E-38	0.127159	0.123018	0.3012945	0.8182849
ENSG00000108021	TASOR2	10	5766252	A	G	0.115308	-0.027868	0.0227367	0.17	0.215832	0.0234825	3.88E-20	-0.129119	0.106277	0.2243934	0.8421058
ENSG00000108039	XPNPEP1	10	111653917	C	T	0.437376	-0.0183089	0.0144778	0.2300001	0.372421	0.00775142	0	-0.0491618	0.0388883	0.206165	0.324829
ENSG00000108055	SMC3	10	112345921	A	G	0.0994036	0.024679	0.0243433	0.3900004	-0.16147	0.0183685	1.49E-18	-0.15284	0.15176	0.3138795	0.1988056
ENSG00000108061	SHOC2	10	112726363	T	G	0.0596421	-0.0236139	0.0318282	0.35	-0.138671	0.0172937	1.07E-15	0.170287	0.230503	0.4600515	0.3890355
ENSG00000108064	TFAM	10	60151881	A	G	0.467197	0.0397632	0.0142724	0.006299992	-0.575466	0.0072792	0	-0.0690974	0.0248169	0.005364469	0.7338087
ENSG00000108091	CCDC6	10	61607467	T	C	0.55169	-0.0163066	0.0142098	0.2099999	-0.186656	0.011742	6.71E-57	0.0873616	0.0763263	0.2523825	0.09719765
ENSG00000108094	CUL2	10	35338524	G	A	0.360835	0.015517	0.0149421	0.3400001	-0.177959	0.00862386	1.31E-94	-0.0871945	0.0840702	0.29966	0.7480668
ENSG00000108100	CCNY	10	35698402	T	C	0.308151	0.0156292	0.0153736	0.2599998	-0.101575	0.00905931	3.55E-29	-0.153869	0.151973	0.3113122	0.8726416
ENSG00000108106	UBE2S	19	55915898	A	G	0.0377734	0.0441447	0.0361305	0.2999998	-0.350181	0.0417837	5.26E-17	-0.126063	0.104267	0.2266506	0.750878
ENSG00000108107	RPL28	19	55905662	G	A	0.37674	0.0105088	0.014752	0.3800004	-0.567715	0.008303	0	-0.0185107	0.0259863	0.4762631	0.09963992
ENSG00000108175	ZMIZ1	10	80952534	A	G	0.326044	0.0223722	0.0147305	0.07599937	0.144465	0.00957619	2.01E-51	0.154863	0.102482	0.1307557	0.8869565
ENSG00000108179	PPIF	10	81111159	T	C	0.462227	0.0350536	0.0142066	0.002900013	0.137536	0.00793191	2.36E-67	0.254868	0.104334	0.01457348	0.2091786
ENSG00000108187	PBLD	10	70067550	G	A	0.00497018	-0.133546	0.0749346	0.04499974	0.517752	0.0890833	6.17E-09	-0.257934	0.151382	0.08840662	0.6327219
ENSG00000108219	TSPAN14	10	82248154	T	C	0.299205	-0.00828839	0.0156788	0.4400003	0.220032	0.00871164	9.44E-141	-0.0376691	0.0712726	0.5971378	0.3015049
ENSG00000108239	TBC1D12	10	96228974	T	C	0.441352	0.0111562	0.0142984	0.4	-0.293099	0.0145125	1.05E-90	-0.0380629	0.0488199	0.4355913	0.6345612
ENSG00000108244	KRT23	17	39086417	A	G	0.382704	0.016799	0.0147495	0.3800004	0.479552	0.00843257	0	0.0350306	0.030763	0.2548177	0.0258899
ENSG00000108256	NUFIP2	17	27601995	T	C	0.172962	-0.0339534	0.0187218	0.04200007	-0.292239	0.0100648	2.33E-185	0.116184	0.0641882	0.0702883	0.2867408
ENSG00000108262	GIT1	17	27910779	T	C	0.429423	-0.0133679	0.0143566	0.16	-0.370038	0.00773324	0	0.0361258	0.038805	0.3518761	0.1840183
ENSG00000108264	NA	17	35803400	G	A	0.336978	-0.00118566	0.0147769	0.9299999	0.360601	0.00813369	0	-0.00328802	0.0409787	0.9360486	0.9559881
ENSG00000108270	NA	17	35360173	C	T	0.341948	-0.0251997	0.01533	0.05499966	0.154197	0.00876396	2.72E-69	-0.163426	0.0998515	0.1016956	0.04928185
ENSG00000108272	NA	17	34952731	C	T	0.466203	-0.0237878	0.0142018	0.1	-0.297333	0.00779179	0	0.0800039	0.04781	0.09425377	0.843858

ENSG00000108278	NA	17	34848813	G	A	0.450298	0.0296901	0.0142469	0.03899959	-0.25841	0.00783674	1.90E-238	-0.114895	0.0552429	0.03754208	0.9609823
ENSG00000108292	NA	17	36873925	T	C	0.138171	-0.00435852	0.0202225	0.9299999	0.216161	0.0173889	1.77E-35	-0.0201633	0.0935669	0.8293807	0.922982
ENSG00000108294	NA	17	36914736	A	G	0.4334	-0.00989748	0.0142991	0.2999998	0.311249	0.0115878	6.45E-159	-0.0317993	0.0459563	0.4889717	0.6930549
ENSG00000108296	NA	17	36969537	G	A	0.237575	-0.0247993	0.0170095	0.08999948	0.235464	0.00931999	7.87E-141	-0.105321	0.0723584	0.1455182	0.966383
ENSG00000108306	FBXL20	17	37487080	C	T	0.251491	0.0202864	0.0163143	0.4	0.14933	0.0103647	4.64E-47	0.135849	0.109656	0.2153944	0.6215825
ENSG00000108309	RUNDC3A	17	42390910	C	A	0.403579	-0.0172924	0.0146557	0.17	-0.322508	0.00823201	0	0.0536185	0.0454635	0.238249	0.4609673
ENSG00000108312	UBTF	17	42290697	A	C	0.287276	-0.0113229	0.0155658	0.4400003	0.117571	0.0091394	7.16E-38	-0.0963068	0.132606	0.4676785	0.6757832
ENSG00000108344	PSMD3	17	38145631	A	G	0.247515	0.0214845	0.0167071	0.3599996	0.159732	0.00924726	7.46E-67	0.134504	0.104884	0.1997019	0.5332278
ENSG00000108349	CASC3	17	38312503	C	T	0.0596421	0.0258064	0.0309998	0.33	-0.422496	0.0197837	3.44E-101	-0.0610808	0.0734287	0.4055003	0.995905
ENSG00000108352	RAPGEFL1	17	38342585	A	G	0.0178926	0.077812	0.0536063	0.08999948	1.42204	0.0368495	0	0.0547185	0.0377233	0.1469135	0.6070115
ENSG00000108370	RGS9	17	63178685	T	C	0.0328032	0.0348997	0.0329565	0.5	-0.165571	0.0202049	2.51E-16	-0.210784	0.200703	0.2936125	0.9178435
ENSG00000108375	RNF43	17	56462408	G	A	0.213718	0.0193679	0.0181475	0.2599998	0.0913336	0.0106793	1.21E-17	0.212057	0.200236	0.2895839	0.2938457
ENSG00000108379	WNT3	17	44875196	G	A	0.257455	0.00275443	0.0165753	0.91	0.146261	0.00999732	1.81E-48	0.0188323	0.113334	0.8680263	0.5084855
ENSG00000108384	RAD51C	17	56790818	A	G	0.181909	0.00394262	0.0171721	0.7300002	0.878078	0.00826968	0	0.00449006	0.0195565	0.8184073	0.2846915
ENSG00000108387	SEPTIN4	17	56607895	T	C	0.384692	0.00738998	0.0145854	0.6999999	0.161622	0.00812589	4.99E-88	0.0457238	0.090273	0.6125019	0.6132624
ENSG00000108389	MTMR4	17	56581082	T	G	0.299205	-0.00927419	0.0154294	0.5999997	0.0697903	0.0087988	2.16E-15	-0.132886	0.221716	0.5489367	0.5096211
ENSG00000108395	TRIM37	17	57122140	G	A	0.362823	0.00854303	0.0146825	0.6100002	-0.628034	0.0074766	0	-0.0136028	0.0233791	0.5606763	0.07723036
ENSG00000108405	P2RX1	17	3809840	G	C	0.277336	0.0146112	0.0151617	0.1800002	-0.891622	0.00826767	0	-0.0163872	0.0170053	0.33522	0.935957
ENSG00000108423	TUBD1	17	57953577	A	G	0.15507	0.00192161	0.0192844	0.98	-0.159287	0.0125232	4.61E-37	-0.0120638	0.121071	0.9206279	0.05218512
ENSG00000108424	KPNB1	17	45744856	T	C	0.0536779	-0.0412669	0.0303917	0.1199999	-0.203762	0.0267123	2.38E-14	0.202525	0.151498	0.1812813	0.3666613
ENSG00000108433	GOSR2	17	45052743	A	C	0.0228628	0.0115612	0.0455487	0.9699999	-0.276802	0.0292102	2.64E-21	-0.0417671	0.164613	0.7997044	0.9601496
ENSG00000108439	PNPO	17	46022263	T	C	0.162028	-0.00372951	0.0188955	0.8700001	0.321352	0.0104747	1.08E-206	-0.0116057	0.0588012	0.8435368	0.2718083
ENSG00000108443	RPS6KB1	17	57999186	G	A	0.291252	0.0248914	0.0155613	0.1900002	0.100983	0.00853315	2.60E-32	0.246491	0.1555	0.1129309	0.007964519
ENSG00000108448	NA	17	18620371	T	C	0.468191	-0.00266244	0.0143233	0.6200004	0.397217	0.0127089	1.91E-214	-0.00670273	0.0360598	0.8525401	0.2515601
ENSG00000108465	CDK5RAP3	17	46052158	G	T	0.136183	0.0207565	0.0229929	0.4700002	-0.277033	0.0131849	5.16E-98	-0.0749244	0.0830736	0.3671083	0.2453315
ENSG00000108468	CBX1	17	46163148	C	T	0.329026	-0.0343479	0.015068	0.016	0.228644	0.0138135	1.54E-61	-0.150224	0.0665236	0.02393244	0.6529193
ENSG00000108469	RECQL5	17	73643097	C	T	0.0328032	0.0253643	0.0388439	0.6700003	0.470642	0.0317153	8.12E-50	0.053893	0.0826138	0.514176	0.6966709
ENSG00000108474	PIGL	17	16186319	G	A	0.506958	-0.00488244	0.0142025	0.6999999	0.20276	0.0079752	1.38E-142	-0.0240799	0.0700523	0.7310404	0.3447187
ENSG00000108479	GALK1	17	73754733	A	G	0.194831	0.0280119	0.018083	0.1	-0.26016	0.0104064	6.11E-138	-0.107672	0.0696406	0.1220784	0.2088337
ENSG00000108506	INTS2	17	59974054	A	G	0.362823	0.00351839	0.0148371	0.4700002	-0.0520392	0.00831935	3.97E-10	-0.0676104	0.285319	0.8126844	0.9609649
ENSG00000108509	CAMTA2	17	4881123	G	T	0.124254	-0.0148916	0.0219513	0.4199997	0.289478	0.0114992	7.76E-140	-0.051443	0.0758582	0.4976789	0.3805146
ENSG00000108510	MED13	17	60081304	C	A	0.227634	-0.012545	0.017191	0.29	0.0570643	0.00905581	2.95E-10	-0.21984	0.30327	0.4685148	0.7058845
ENSG00000108515	ENO3	17	4855906	A	C	0.0904573	0.0276761	0.0226931	0.2099999	0.177801	0.0138667	1.23E-37	0.155658	0.128208	0.2247085	0.9266489
ENSG00000108518	PFN1	17	4850651	A	G	0.0109344	-0.00878378	0.0678733	0.7499995	0.855369	0.0778287	4.25E-28	-0.010269	0.0793552	0.8970369	0.3694789
ENSG00000108523	RNF167	17	4845910	A	G	0.204771	-0.00232878	0.0184301	0.95	0.544938	0.0102478	0	-0.00427347	0.0338206	0.8994493	0.6439603
ENSG00000108528	SLC25A11	17	4841985	C	T	0.43837	-0.0160034	0.0144322	0.2	-0.213933	0.00864537	3.47E-135	0.0748056	0.067529	0.2679672	0.9425844
ENSG00000108551	RASD1	17	17398730	T	C	0.442346	-0.0190858	0.01424	0.1800002	0.137746	0.00803849	8.02E-66	-0.138558	0.103694	0.1814794	0.2512519
ENSG00000108556	CHRNE	17	4803719	A	G	0.0944334	0.0064487	0.024826	0.8499999	0.344338	0.0133775	4.17E-146	0.0187278	0.0721015	0.7950623	0.4889015
ENSG00000108557	RAI1	17	17649777	A	G	0.00695825	0.140665	0.0843694	0.09400046	0.934856	0.0886792	5.53E-26	0.150467	0.0913702	0.09960254	0.6504606
ENSG00000108559	NUP88	17	5293869	T	C	0.440358	0.00618981	0.014571	0.5400003	-0.400129	0.00846713	0	-0.0154695	0.0364173	0.6709925	0.1307515
ENSG00000108561	C1QBP	17	5344123	T	C	0.0596421	-0.0316864	0.0330227	0.2099999	-0.114682	0.0183962	4.55E-10	0.276298	0.291341	0.3429433	0.2984976
ENSG00000108578	BLMH	17	28597146	A	T	0.494036	0.0222336	0.0142563	0.0649995	-0.322236	0.00789987	0	-0.0689978	0.0442741	0.1191327	0.5169938
ENSG00000108582	CPD	17	28751465	A	C	0.322068	0.0130102	0.015221	0.4	-0.475184	0.0121139	0	-0.0273793	0.0320394	0.3928001	0.4644527
ENSG00000108587	GOSR1	17	28829495	C	T	0.493042	0.0245435	0.0141896	0.0519996	0.0743274	0.00797812	1.20E-20	0.330208	0.194169	0.08901422	0.6513889
ENSG00000108588	CCDC47	17	61838160	C	T	0.460239	0.0325274	0.0142684	0.008600031	-0.124022	0.00800026	3.35E-54	-0.262272	0.116285	0.02410671	0.4611436
ENSG00000108590	MED31	17	6550794	C	G	0.110338	-0.0331064	0.0230234	0.16	0.465146	0.0138677	1.19E-246	-0.0711742	0.0495426	0.1508242	0.6195129
ENSG00000108591	DRG2	17	18001242	A	G	0.0904573	-0.0306181	0.0261014	0.1900002	-0.373047	0.0144796	2.27E-146	0.0820756	0.0700405	0.2412652	0.01840382
ENSG00000108592	FTSJ3	17	61902082	A	G	0.400596	0.0423094	0.0145475	0.00129999	-0.411473	0.00794612	0	-0.102824	0.0354104	0.003686801	0.4477131
ENSG00000108599	AKAP10	17	19844635	C	A	0.394632	0.00119266	0.014494	0.7199992	0.621242	0.00744116	0	0.0019198	0.0233307	0.934419	0.8735543
ENSG00000108604	SMARCD2	17	61914934	T	C	0.26839	-0.0348173	0.0157672	0.009800089	0.333303	0.00871331	0	-0.104461	0.0473846	0.02748636	0.4766041

ENSG00000108622	ICAM2	17	62088974	G	A	0.380716	-0.00663006	0.0146413	0.8499999	-0.207645	0.00902696	4.37E-117	0.0319297	0.0705248	0.6507324	0.08997428
ENSG00000108639	SYNGR2	17	76167123	T	C	0.309145	0.00219625	0.0151993	0.6800001	-0.123664	0.00977995	1.20E-36	-0.0177599	0.122917	0.8851156	0.8553288
ENSG00000108651	UTP6	17	30208353	G	A	0.0119284	0.0881552	0.0565503	0.1800002	0.407555	0.0481881	2.73E-17	0.216302	0.141092	0.1252617	0.6454421
ENSG00000108654	DDX5	17	62500025	G	A	0.358847	0.0193737	0.0153986	0.2200002	-0.584066	0.011711	0	-0.0331704	0.0263729	0.2084834	0.598715
ENSG00000108666	C17orf75	17	30664313	T	G	0.362823	0.0164966	0.0146363	0.3400001	0.442892	0.00818948	0	0.0372474	0.0330543	0.2598029	0.2916203
ENSG00000108669	CYTH1	17	76724254	G	A	0.234592	-0.0185027	0.017053	0.1900002	-0.13317	0.00948059	8.08E-45	0.13894	0.128436	0.2793463	0.4351954
ENSG00000108671	PSMD11	17	30790807	A	G	0.485089	-0.0187072	0.0142038	0.2700001	0.0538425	0.00797761	1.49E-11	-0.347443	0.268779	0.1961242	0.6115041
ENSG00000108679	LGALS3BP	17	76971755	T	C	0.0497018	0.0154506	0.0324589	0.6800001	-0.292957	0.0193519	9.04E-52	-0.0527402	0.110852	0.6342383	0.02200263
ENSG00000108691	CCL2	17	32583229	C	G	0.0705765	-0.00845679	0.0271359	0.81	0.105558	0.0160232	4.46E-11	-0.0801152	0.257359	0.7555743	0.8999063
ENSG00000108700	CCL8	17	32647238	C	A	0.255467	-0.00313286	0.0156616	0.7800007	0.0484822	0.00889255	4.98E-08	-0.0646188	0.323256	0.8415588	0.5889827
ENSG00000108733	PEX12	17	33903848	C	A	0.467197	0.0169325	0.0141817	0.1199999	0.0992674	0.00793174	6.17E-36	0.170575	0.143512	0.2346083	0.9895981
ENSG00000108771	DHX58	17	40259086	G	C	0.177932	-0.0229433	0.0189044	0.2	-0.536463	0.0155561	1.31E-260	0.0427677	0.0352608	0.2251689	0.1488361
ENSG00000108773	KAT2A	17	40269251	C	G	0.235586	-0.00959907	0.0169362	0.5500004	-0.138186	0.0153141	1.82E-19	0.069465	0.122803	0.5716226	0.6579649
ENSG00000108774	RAB5C	17	40292014	C	G	0.235586	-0.00959907	0.0169362	0.5500004	0.115342	0.0153311	5.34E-14	-0.0832225	0.14725	0.5719536	0.6657644
ENSG00000108784	NAGLU	17	40692328	T	C	0.244533	0.022101	0.0159529	0.1199999	-0.183643	0.00884411	9.09E-96	-0.120348	0.0870624	0.1668742	0.2495295
ENSG00000108785	NA	17	40699747	C	A	0.475149	0.0226219	0.0143002	0.2300001	0.303755	0.0138605	1.86E-106	0.0744742	0.0472006	0.1146061	0.6742592
ENSG00000108786	HSD17B1	17	40704231	C	T	0.475149	0.0219759	0.0142894	0.25	0.160813	0.0088383	5.65E-74	0.136655	0.0891741	0.125411	0.04357492
ENSG00000108788	MLX	17	40722171	A	G	0.265408	0.0108129	0.0157183	0.3599996	0.147913	0.00883589	6.70E-63	0.0731032	0.106357	0.4918699	0.1085733
ENSG00000108797	CNTNAP1	17	40843231	A	G	0.0477137	0.0234661	0.0320794	0.56	0.204942	0.0174677	8.68E-32	0.114501	0.156833	0.4653395	0.4143842
ENSG00000108798	ABI3	17	47294088	A	G	0.186879	0.0178142	0.0186246	0.32	-0.0850522	0.0103116	1.61E-16	-0.20945	0.220446	0.342051	0.7657852
ENSG00000108799	EZH1	17	40874682	C	T	0.501988	0.0250358	0.0142366	0.14	-0.060648	0.00806188	5.36E-14	-0.412805	0.24107	0.0868253	0.4946237
ENSG00000108813	DLX4	17	48049327	C	T	0.191849	-0.0123	0.0179551	0.3599996	-0.0913762	0.00963945	2.56E-21	0.134608	0.197009	0.4944424	0.4336526
ENSG00000108821	COL1A1	17	48269821	T	C	0.206759	-0.00466602	0.0176592	0.8200001	0.0840469	0.010457	9.18E-16	-0.0555169	0.210225	0.7917157	0.1997604
ENSG00000108823	SGCA	17	48247433	G	T	0.442346	0.0244325	0.0141916	0.0659994	0.351747	0.0115102	4.22E-205	0.0694604	0.04041	0.08563413	0.3750313
ENSG00000108826	MRPL27	17	48447896	G	A	0.446322	0.0186171	0.0141807	0.17	-0.165867	0.00804548	1.96E-94	-0.112241	0.0856674	0.1901303	0.8130941
ENSG00000108828	VAT1	17	41171881	G	A	0.361829	-0.0214476	0.0150611	0.1100001	-0.305353	0.00890997	2.13E-257	0.0702388	0.0493662	0.1547908	0.5391973
ENSG00000108839	ALOX12	17	6906719	G	C	0.383698	-0.0100852	0.0144974	0.6600001	0.115695	0.00815534	1.11E-45	-0.0871706	0.125458	0.4871676	0.843295
ENSG00000108846	ABCC3	17	48740875	T	C	0.346918	-0.00604093	0.0147322	0.7800007	-0.594049	0.00762551	0	0.0101691	0.0248	0.6817739	0.2447683
ENSG00000108852	MPP2	17	41969896	A	G	0.342942	-0.00606382	0.0149137	0.83	0.185659	0.00823607	1.60E-112	-0.032661	0.0803415	0.6843548	0.8047422
ENSG00000108854	SMURF2	17	62598299	T	C	0.0159046	0.0481834	0.0535998	0.3700002	0.691355	0.0739433	8.78E-21	0.0696942	0.0778862	0.3708828	0.5834936
ENSG00000108861	DUSP3	17	41849922	C	A	0.0606362	-0.0169381	0.0267034	0.5300002	0.528567	0.0145377	1.94E-289	-0.0320453	0.0505281	0.5259448	0.09126614
ENSG00000108883	EFTUD2	17	42952170	C	A	0.407555	0.00598341	0.0147138	0.6499995	-0.247605	0.00809023	1.04E-205	-0.0241652	0.0594298	0.6842893	0.6595143
ENSG00000108924	HLF	17	53372399	T	C	0.286282	-0.0122081	0.0153445	0.5999997	0.0897709	0.00872053	7.48E-25	-0.135992	0.171439	0.4276406	0.7935136
ENSG00000108932	SLC16A6	17	66275287	G	T	0.360835	0.00525421	0.0148109	0.5700002	0.275657	0.00810961	2.98E-253	0.0190607	0.0537324	0.7227892	0.6497323
ENSG00000108946	PRKAR1A	17	66478612	T	C	0.252485	0.0221168	0.0164237	0.1100001	0.0856097	0.00938312	7.25E-20	0.258345	0.193922	0.1827923	0.9700109
ENSG00000108950	FAM20A	17	66564392	A	G	0.183897	-0.00415707	0.0179279	0.84	-0.153252	0.0101278	9.99E-52	0.0271257	0.116997	0.8166546	0.9923167
ENSG00000108953	YWHAE	17	1275619	G	A	0.0815109	0.0206163	0.0281333	0.6100002	-0.096268	0.0159648	1.64E-09	-0.214155	0.29439	0.4669469	0.5202786
ENSG00000108960	MMD	17	53484663	C	T	0.40159	-0.00201273	0.0146392	0.9199999	0.157	0.00818081	4.38E-82	-0.01282	0.093246	0.890647	0.9156483
ENSG00000108961	RANGRF	17	8192612	A	G	0.0238569	-0.0087326	0.052826	0.7099994	-0.211173	0.0290415	3.56E-13	0.0413529	0.25022	0.8687346	0.4423121
ENSG00000108963	DPH1	17	1940064	G	T	0.331014	-0.00152702	0.0153578	0.9699999	0.132114	0.00850973	2.35E-54	-0.0115584	0.116249	0.9207988	0.05521812
ENSG00000109016	DHRS7B	17	21061627	T	C	0.435388	0.0165111	0.0143474	0.3100002	-0.131884	0.007992	3.55E-61	-0.125194	0.109052	0.2509605	0.4442631
ENSG00000109046	WSB1	17	25630879	A	G	0.387674	0.0119403	0.014395	0.4899999	-0.224513	0.00798289	4.94E-174	-0.053183	0.0641444	0.4070393	0.483463
ENSG00000109047	RCVRN	17	9804287	C	T	0.44334	0.016974	0.0144854	0.2999998	0.59847	0.00743304	0	0.0283623	0.0242066	0.241327	0.1335767
ENSG00000109062	NHERF1	17	72755141	T	C	0.45825	0.0132513	0.014257	0.5	-0.223118	0.0078527	1.40E-177	-0.0593914	0.0639331	0.3529086	0.7339378
ENSG00000109063	MYH3	17	10546234	C	G	0.471173	-0.00883557	0.0143694	0.5	0.265195	0.0130679	1.46E-91	-0.0333172	0.054209	0.5388151	0.4599242
ENSG00000109065	NAT9	17	72769596	C	G	0.439364	-0.00754767	0.0147698	0.81	-0.105552	0.0101963	4.10E-25	0.0715065	0.140099	0.609772	0.5782079
ENSG00000109066	TMEM104	17	72804270	A	C	0.440358	0.00725015	0.0143196	0.9599999	-0.0619043	0.00795195	6.98E-15	-0.117119	0.231807	0.6133888	0.3183287
ENSG00000109079	TNFAIP1	17	26668331	G	A	0.00795229	0.0639402	0.0753979	0.3800004	-0.518871	0.06747	1.47E-14	-0.123229	0.146192	0.3992691	0.6080763
ENSG00000109084	TMEM97	17	26650914	G	A	0.429423	-0.0243492	0.0144299	0.0649995	-0.463058	0.00767693	0	0.0525835	0.0311744	0.09165085	0.5220334
ENSG00000109099	PMP22	17	15150869	A	T	0.198807	0.00578853	0.0172246	0.7400005	0.300717	0.00990101	1.28E-202	0.0192491	0.057282	0.7368398	0.2737848

ENSG00000109107	ALDOC	17	26902207	C	A	0.303181	0.0131095	0.0156219	0.5199996	-0.264069	0.0086689	8.41E-204	-0.0496443	0.0591809	0.40155	0.1691263
ENSG00000109113	RAB34	17	27043373	C	A	0.0725646	0.00108696	0.025119	0.7800007	-0.345425	0.0134226	4.79E-146	-0.00314673	0.0727191	0.9654844	0.6274591
ENSG00000109133	TMEM33	4	41949863	T	G	0.116302	0.0413564	0.0214081	0.0329997	0.194231	0.0137217	1.74E-45	0.212924	0.111242	0.05561149	0.1880521
ENSG00000109171	SLAIN2	4	48385784	T	C	0.270378	0.00823133	0.0154502	0.4199997	-0.192576	0.00937494	9.15E-94	-0.0427432	0.0802559	0.5943201	0.05951013
ENSG00000109180	OCIAD1	4	48835531	C	T	0.518887	-0.0151962	0.0142161	0.2200002	0.212476	0.00790207	2.99E-159	-0.0715198	0.0669598	0.2854757	0.1754772
ENSG00000109184	DCUN1D4	4	52746084	T	A	0.255467	0.0105096	0.0155759	0.29	-0.0589809	0.0105004	1.94E-08	-0.178186	0.265982	0.5029097	0.3407897
ENSG00000109189	USP46	4	53492921	T	C	0.131213	0.0336022	0.0225509	0.2099999	0.140242	0.0122536	2.49E-30	0.239602	0.162157	0.1395175	0.5207648
ENSG00000109270	LAMTOR3	4	100807570	C	T	0.256461	0.000778097	0.0172763	0.7700005	-0.135979	0.00943302	4.15E-47	-0.00572219	0.127052	0.9640769	0.814332
ENSG00000109272	PF4V1	4	74719389	T	C	0.286282	-0.0310034	0.0156257	0.05699936	0.758779	0.00775406	0	-0.0408596	0.0205974	0.04728695	0.9907814
ENSG00000109320	NFKB1	4	103480472	T	C	0.398608	0.019972	0.0144549	0.08799946	0.18313	0.00821925	5.70E-110	0.109059	0.0790842	0.1678862	0.924987
ENSG00000109323	MANBA	4	103617405	T	C	0.332008	0.0153199	0.0150838	0.4100001	0.242721	0.00881649	7.60E-167	0.0631172	0.0621868	0.3101239	0.9631371
ENSG00000109381	ELF2	4	140023819	G	C	0.212724	-0.02906	0.0172159	0.06900014	-0.217708	0.00943969	1.09E-117	0.133482	0.0792896	0.09228384	0.8794542
ENSG00000109390	NDUFC1	4	140205869	T	C	0.198807	-0.00394317	0.0182448	0.7899998	0.257386	0.0152274	4.29E-64	-0.0153201	0.0708908	0.8289035	0.7270298
ENSG00000109436	TBC1D9	4	141609596	A	G	0.303181	-0.0205982	0.0154145	0.1499999	-0.301342	0.00932878	6.47E-229	0.0683549	0.0511966	0.1818288	0.6248809
ENSG00000109445	ZNF330	4	142148946	T	G	0.243539	0.00415954	0.0165055	0.95	-0.256101	0.00908785	1.01E-174	-0.0162418	0.0644518	0.8010416	0.3969313
ENSG00000109452	INPP4B	4	143356449	T	G	0.264414	-0.01708	0.0169225	0.5099998	-0.32545	0.00947867	2.36E-258	0.0524811	0.0520197	0.3130363	0.7135606
ENSG00000109458	GAB1	4	144326818	C	G	0.365805	0.00922454	0.0147545	0.5300002	0.0650771	0.00814544	1.36E-15	0.141748	0.227416	0.5330898	0.2378981
ENSG00000109466	KLHL2	4	166186539	T	C	0.0874751	0.00709344	0.0258661	0.7199992	0.135949	0.0141774	8.88E-22	0.052177	0.19034	0.7839887	0.6071452
ENSG00000109501	WFS1	4	6288284	C	T	0.105368	-0.0113462	0.0211908	0.59	-0.277939	0.0121435	6.12E-116	0.0408226	0.0762634	0.5924535	0.4415358
ENSG00000109519	GRPEL1	4	7065278	G	A	0.238569	0.0125132	0.0172849	0.5099998	-0.111658	0.00982161	5.99E-30	-0.112067	0.155116	0.4700025	0.1835318
ENSG00000109536	FRG1	4	190873151	A	G	0.323062	0.00243895	0.0152497	0.99	-0.105497	0.0126647	8.08E-17	-0.0231186	0.144577	0.872956	0.1756961
ENSG00000109572	CLCN3	4	170589304	G	A	0.271372	0.00318867	0.0160046	0.84	-0.0820269	0.00892975	4.08E-20	-0.0388735	0.19516	0.8421159	0.2612837
ENSG00000109586	GALNT7	4	174167511	A	G	0.503976	-0.00228371	0.0142492	0.98	0.0817133	0.00793241	6.96E-25	-0.0279478	0.174402	0.8726842	0.5562419
ENSG00000109606	DHX15	4	24552618	T	C	0.38668	-0.00259794	0.0147079	0.91	0.0664602	0.0082318	6.83E-16	-0.0390901	0.221357	0.8598278	0.7781631
ENSG00000109618	SEPSECS	4	25141915	A	G	0.106362	0.000710728	0.0228194	0.8499999	0.162122	0.0132409	1.81E-34	0.00438391	0.140755	0.9751534	0.08490093
ENSG00000109654	TRIM2	4	154166983	G	C	0.431412	0.00622421	0.0145218	0.58	-0.112324	0.00814566	2.95E-43	-0.0554131	0.129348	0.6683566	0.532375
ENSG00000109667	SLC2A9	4	9914668	C	T	0.434394	0.0294387	0.0143528	0.03899959	-0.251443	0.00859775	5.17E-188	-0.117079	0.057222	0.04075132	0.02528648
ENSG00000109670	FBXW7	4	153349291	G	C	0.300199	0.00800689	0.015746	0.7099994	0.0926191	0.00874623	3.33E-26	0.0864496	0.170204	0.6115109	0.8936635
ENSG00000109680	TBC1D19	4	26667516	C	T	0.0785288	0.0226201	0.0295553	0.5	0.101018	0.0183462	3.67E-08	0.223921	0.295387	0.4484148	0.2382406
ENSG00000109684	CLNK	4	10587254	T	C	0.476143	-0.029379	0.0142394	0.04600023	0.0597267	0.00808013	1.45E-13	-0.491891	0.247522	0.04689384	0.09760417
ENSG00000109689	STIM2	4	26943151	A	G	0.232604	0.00163306	0.0165568	0.7600007	0.290241	0.00905803	2.80E-225	0.00562656	0.0570452	0.9214294	0.4108321
ENSG00000109736	MFSD10	4	2934437	C	G	0.307157	-0.000849674	0.0157605	0.7499995	-0.166389	0.00869496	1.26E-81	0.00510656	0.0947214	0.9570058	0.7811223
ENSG00000109743	BST1	4	15722254	G	T	0.437376	-0.0270626	0.0142868	0.04499974	0.482733	0.00752411	0	-0.0560613	0.0296086	0.05830297	0.551828
ENSG00000109756	RAPGEF2	4	160153325	G	A	0.370775	-0.0151985	0.0148471	0.32	-0.426662	0.0085957	0	0.0356219	0.0348057	0.306095	0.2023144
ENSG00000109762	SNX25	4	186208365	C	T	0.422465	0.00520196	0.0143438	0.5	0.390191	0.0077138	0	0.0133318	0.0367619	0.7168639	0.2309524
ENSG00000109771	LRP2BP	4	186301042	A	G	0.422465	0.00585421	0.0143145	0.4700002	0.121353	0.00886801	1.26E-42	0.048241	0.11801	0.6826952	0.3678426
ENSG00000109787	KLF3	4	38684240	A	C	0.237575	0.00559989	0.0168272	0.3900004	-0.0705418	0.00953138	1.35E-13	-0.079384	0.238783	0.7395479	0.3460618
ENSG00000109790	KLHL5	4	39087568	C	T	0.40159	0.0152342	0.014531	0.2200002	-0.440891	0.00776147	0	-0.0345532	0.0329639	0.2945398	0.2617398
ENSG00000109794	FAM149A	4	187059697	G	C	0.523857	0.00191482	0.0142804	0.7300002	0.30609	0.00918619	1.94E-243	0.00625574	0.0466546	0.8933344	0.5953083
ENSG00000109814	UGDH	4	39515153	T	G	0.309145	0.0153912	0.0151967	0.1299999	-0.648955	0.00796677	0	-0.0237169	0.023419	0.3111937	0.900964
ENSG00000109819	PPARGC1A	4	23831188	G	A	0.178926	-0.0125065	0.0182956	0.5300002	0.499826	0.0096468	0	-0.0250217	0.0366071	0.4942782	0.2875708
ENSG00000109854	HTATIP2	11	20395280	C	T	0.33996	-0.000158754	0.0149099	0.9400001	-0.487375	0.00784374	0	0.000325733	0.0305923	0.9915046	0.9743754
ENSG00000109861	CTSC	11	88048857	A	G	0.196819	-0.0321601	0.0184841	0.03799969	-0.541085	0.00912139	0	0.0594363	0.0341758	0.08201168	0.1731879
ENSG00000109881	CCDC34	11	27368894	A	T	0.28827	0.00888432	0.0159012	0.6700003	0.0973785	0.00873162	6.97E-29	0.0912349	0.163497	0.5768306	0.9484336
ENSG00000109917	ZPR1	11	116653601	A	G	0.0666004	-0.0276343	0.0293419	0.25	-0.311621	0.0300792	3.77E-25	0.0886792	0.0945472	0.3482775	0.6513752
ENSG00000109919	MTCH2	11	47651521	A	G	0.0636183	0.0197659	0.030207	0.4100001	-0.23929	0.0197353	7.79E-34	-0.0826021	0.126419	0.5135	0.7672834
ENSG00000109920	FNBP4	11	47763533	C	T	0.430417	0.00843773	0.0142437	0.6999999	-0.1343	0.0079946	2.49E-63	-0.0628276	0.106125	0.5538403	0.8584625
ENSG00000109927	TECTA	11	121017042	T	C	0.244533	0.00290597	0.0170311	0.91	0.0704427	0.00956166	1.74E-13	0.041253	0.241837	0.8645528	0.5783914
ENSG00000109929	SC5D	11	121171282	A	G	0.0735586	0.026065	0.0274335	0.4299995	0.541257	0.0162956	6.56E-242	0.0481565	0.0507056	0.3422509	0.9639904
ENSG00000109943	CRTAM	11	122726277	A	G	0.333002	-0.0186893	0.0150155	0.14	-0.264577	0.00816199	1.64E-230	0.0706383	0.0567946	0.2135913	0.09865234

ENSG00000109944	JHY	11	122791948	T	C	0.100398	-0.0263408	0.0241744	0.3100002	0.516931	0.0133707	0	-0.0509561	0.0467838	0.2760732	0.1397872
ENSG00000109956	B3GAT1	11	134265105	G	T	0.121272	-0.00691971	0.0201188	0.9400001	-0.397534	0.0119196	7.00E-244	0.0174066	0.0506117	0.7309033	0.4222231
ENSG00000109971	HSPA8	11	122931067	G	A	0.171968	-0.0247804	0.0183735	0.17	0.112495	0.00998376	1.89E-29	-0.22028	0.164493	0.1805236	0.7596257
ENSG00000110002	VWA5A	11	124002248	G	A	0.151093	-0.0329397	0.0212425	0.06699926	-0.362274	0.0114387	3.92E-220	0.0909249	0.0587068	0.1214309	0.7309694
ENSG00000110011	DNAJC4	11	63999754	T	G	0.335984	0.0210365	0.014763	0.09599973	-0.0601274	0.00911269	4.16E-11	-0.349866	0.251189	0.1636689	0.1151821
ENSG00000110013	SIAE	11	124534306	C	A	0.0178926	0.0131175	0.0482184	0.4500005	0.367926	0.0353293	2.14E-25	0.0356525	0.131099	0.7856599	0.5036021
ENSG00000110025	SNX15	11	64794849	C	T	0.298211	0.0168451	0.0157623	0.3900004	-0.178306	0.00869436	1.82E-93	-0.0944732	0.0885204	0.2858601	0.1860804
ENSG00000110031	LPXN	11	58320018	A	G	0.0149105	0.0400388	0.0622668	0.4600002	-1.36623	0.0339428	0	-0.029306	0.0455814	0.5202643	0.589278
ENSG00000110042	DTX4	11	58957481	A	C	0.489066	0.012436	0.0142059	0.6600001	0.0599224	0.0088225	1.11E-11	0.207535	0.239033	0.385269	0.9382808
ENSG00000110047	EHD1	11	64637441	A	G	0.122266	-0.0314676	0.0229626	0.1100001	0.257846	0.0128279	7.32E-90	-0.12204	0.0892623	0.1715591	0.9681564
ENSG00000110048	OSBP	11	59362744	A	G	0.157058	-0.011894	0.0200072	0.4199997	-0.0617462	0.0109421	1.67E-08	0.192627	0.325816	0.5543769	0.5009516
ENSG00000110057	UNC93B1	11	67765513	C	G	0.15507	0.0141993	0.0175057	0.4600002	0.114827	0.0117735	1.79E-22	0.123658	0.152979	0.4188985	0.3962668
ENSG00000110060	PUS3	11	125768248	A	G	0.414513	-0.0207575	0.0144379	0.1100001	-0.292055	0.00784754	3.73E-303	0.0710738	0.0494723	0.150821	0.1637601
ENSG00000110063	DCPS	11	126194645	A	G	0.0984095	0.0145843	0.022704	0.4500005	-0.503379	0.0124491	0	-0.0289728	0.0451089	0.5206875	0.1590929
ENSG00000110074	FOXRED1	11	126143488	T	C	0.223658	0.0190268	0.0171343	0.3100002	-0.800458	0.00865373	0	-0.0237699	0.0214072	0.2668392	0.3668816
ENSG00000110075	PPP6R3	11	68305494	A	G	0.27336	-0.00574383	0.0158817	0.6700003	0.159841	0.00930291	3.63E-66	-0.0359347	0.0993816	0.7176634	0.8833695
ENSG00000110077	MS4A6A	11	59945610	G	A	0.404573	0.00840095	0.0144789	0.56	-0.408146	0.00788745	0	-0.0205832	0.035477	0.5617907	0.7487818
ENSG00000110079	MS4A4A	11	60062229	C	A	0.40159	0.00581076	0.014423	0.64	-0.251664	0.00807237	2.26E-213	-0.0230894	0.0573154	0.6870601	0.9469459
ENSG00000110080	ST3GAL4	11	126267887	T	G	0.176938	-0.0201998	0.0181063	0.2399999	-0.16678	0.00992198	2.09E-63	0.121116	0.108803	0.2656338	0.5066526
ENSG00000110090	CPT1A	11	68566983	A	G	0.153082	0.00983922	0.0180312	0.56	-0.369261	0.0106313	2.48E-264	-0.0266457	0.0488366	0.5853339	0.04931109
ENSG00000110092	CCND1	11	69462548	G	T	0.358847	0.012376	0.0149801	0.4299995	-0.169161	0.00836619	6.58E-91	-0.0731611	0.0886292	0.4091026	0.240784
ENSG00000110104	CCDC86	11	60614049	G	A	0.104374	-0.0264452	0.0225001	0.1800002	-0.163424	0.0123986	1.13E-39	0.16182	0.138226	0.2417226	0.2786092
ENSG00000110107	PRPF19	11	60666131	C	G	0.389662	-0.010297	0.0144596	0.5300002	-0.152684	0.00820078	2.29E-77	0.06744	0.0947722	0.4767114	0.7134574
ENSG00000110108	TMEM109	11	60686130	A	G	0.388668	-0.0101957	0.0144621	0.5400003	-0.253394	0.00811446	4.53E-214	0.0402366	0.0570882	0.4809255	0.7401938
ENSG00000110169	HPX	11	6458063	G	A	0.208748	0.026084	0.0174096	0.1	-0.0896275	0.00980265	6.06E-20	-0.291027	0.196834	0.1392647	0.8273337
ENSG00000110171	TRIM3	11	6482766	A	G	0.184891	0.0117142	0.0182195	0.3800004	0.17992	0.0104577	2.45E-66	0.0651078	0.101335	0.5205486	0.7857273
ENSG00000110172	CHORDC1	11	89945430	G	A	0.361829	-0.00971006	0.0150967	0.4100001	-0.0859065	0.0125162	6.71E-12	0.113031	0.176504	0.5219222	0.4349786
ENSG00000110200	ANAPC15	11	71820625	A	G	0.506958	-0.0101073	0.0142083	0.5999997	0.161607	0.0157828	1.32E-24	-0.0625425	0.0881309	0.4779179	0.8584516
ENSG00000110203	FOLR3	11	71838425	T	C	0.0974155	0.0158799	0.0245914	0.5099998	1.26233	0.0119559	0	0.0125798	0.0194813	0.518449	0.5757187
ENSG00000110218	PANX1	11	93888616	C	T	0.481113	0.00767749	0.0142002	0.64	-0.421779	0.00760807	0	-0.0182026	0.033669	0.5887592	0.3946787
ENSG00000110237	ARHGEF17	11	73049735	A	G	0.10835	-0.00471454	0.0224321	0.8700001	0.668126	0.0123764	0	-0.00705637	0.0335749	0.8335367	0.8908364
ENSG00000110274	CEP164	11	117234628	G	C	0.375746	-0.0139459	0.0147049	0.4199997	0.170059	0.00820992	2.60E-95	-0.0820064	0.0865602	0.343439	0.1590464
ENSG00000110315	RNF141	11	10548001	A	C	0.451292	-0.0031925	0.0142269	0.6200004	0.199387	0.00787462	1.91E-141	-0.0160116	0.071356	0.8224538	0.09901397
ENSG00000110318	CEP126	11	101828767	A	G	0.0685885	-0.0411759	0.0283943	0.16	0.255026	0.0180098	1.61E-45	-0.161458	0.111921	0.1491327	0.1168475
ENSG00000110321	EIF4G2	11	10824627	A	G	0.432406	-0.00164147	0.0143761	0.8200001	0.102255	0.00810274	1.64E-36	-0.0160527	0.140596	0.9090984	0.5495687
ENSG00000110324	IL10RA	11	117864629	A	T	0.055666	0.0289302	0.0310225	0.35	-0.390607	0.0160308	3.92E-131	-0.0740647	0.0794794	0.3514021	0.9737316
ENSG00000110330	BIRC2	11	102233671	T	G	0.084493	0.00583132	0.0231826	0.6700003	-0.0960918	0.0149806	1.41E-10	-0.0606849	0.24144	0.801547	0.5644608
ENSG00000110344	UBE4A	11	118250113	T	G	0.0944334	-0.0106676	0.0226274	0.8	0.307057	0.0128237	1.05E-126	-0.0347414	0.0737054	0.6373874	0.1410427
ENSG00000110395	CBL	11	119127201	T	C	0.324056	-0.0279873	0.0159663	0.064	-0.31474	0.0127292	5.65E-135	0.0889221	0.0508559	0.08037577	0.1570329
ENSG00000110400	NECTIN1	11	119546957	G	A	0.435388	0.00116593	0.0147824	0.98	-0.0564693	0.00926272	1.08E-09	-0.0206472	0.2618	0.937139	0.7864943
ENSG00000110422	HIPK3	11	33328393	C	T	0.483101	0.000677344	0.014223	0.9599999	-0.0969299	0.00800597	9.67E-34	-0.00698798	0.146736	0.9620169	0.00590325
ENSG00000110429	FBXO3	11	33779287	T	C	0.388668	0.0240913	0.0148364	0.1199999	0.227252	0.0083314	8.04E-164	0.106011	0.0654016	0.1050333	0.3336793
ENSG00000110435	PDHX	11	34989757	T	C	0.328032	-0.0106203	0.0148399	0.7499995	0.13745	0.00860286	1.84E-57	-0.0772669	0.108074	0.4746451	0.7060621
ENSG00000110442	COMMD9	11	36303025	G	C	0.195825	-0.0076958	0.0166293	0.5700002	0.0890323	0.00947535	5.66E-21	-0.0864383	0.187005	0.6439198	0.2062147
ENSG00000110446	SLC15A3	11	60712279	G	C	0.196819	-0.0060242	0.0181799	0.9400001	-0.244857	0.00950901	3.22E-146	0.0246029	0.0742532	0.7403888	0.577461
ENSG00000110448	CD5	11	60882595	T	C	0.409543	0.0229252	0.014511	0.1100001	-0.203527	0.00822419	3.31E-135	-0.112639	0.0714427	0.1148783	0.8174588
ENSG00000110455	ACCS	11	44096623	T	G	0.120278	0.0254848	0.0233739	0.3599996	1.31917	0.0131363	0	0.0193188	0.0177197	0.2756049	0.8325781
ENSG00000110514	MADD	11	47321147	T	C	0.0516899	-0.0378712	0.0292386	0.14	-0.626283	0.0170197	2.02E-296	0.0604698	0.0467148	0.1955117	0.4184743
ENSG00000110536	PTPMT1	11	47590997	A	G	0.503976	-0.0119937	0.0141779	0.4799997	-0.094715	0.00873754	2.22E-27	0.126629	0.150145	0.3990165	0.9775498
ENSG00000110583	NAA40	11	63715615	A	G	0.394632	0.0192801	0.0144759	0.09699961	0.112733	0.00826757	2.46E-42	0.171024	0.12902	0.184983	0.3189756

ENSG00000110619	CARS1	11	3050497	A	G	0.394632	0.0275704	0.0144379	0.05399953	0.208206	0.00806263	4.82E-147	0.132419	0.0695336	0.05686	0.01366496
ENSG00000110628	SLC22A18	11	2933713	G	A	0.430417	-0.00164732	0.0142955	0.89	-0.424941	0.00941739	0	0.00387659	0.0336413	0.9082604	0.03286101
ENSG00000110651	CD81	11	2408028	A	C	0.358847	-0.0146862	0.014761	0.2599998	-0.22718	0.00842656	4.35E-160	0.0646456	0.0650191	0.3200984	0.3007472
ENSG00000110660	SLC35F2	11	107730368	C	T	0.126243	-0.0291871	0.0219505	0.17	-0.319644	0.0122222	9.16E-151	0.0913114	0.0687605	0.1841904	0.3061196
ENSG00000110665	C11orf21	11	2320577	T	C	0.357853	-0.0157168	0.0148764	0.2399999	-0.558045	0.00858186	0	0.028164	0.0266616	0.2908071	0.3433813
ENSG00000110693	SOX6	11	16374566	T	G	0.194831	-0.0386218	0.0184778	0.04300015	0.0620119	0.0102054	1.23E-09	-0.622813	0.315108	0.04809732	0.6644145
ENSG00000110696	C11orf58	11	16706553	G	A	0.432406	0.0230218	0.0144517	0.08600031	0.171572	0.00855226	1.60E-89	0.134181	0.0844962	0.1122822	0.4783467
ENSG00000110697	PITPNM1	11	67266486	A	G	0.0159046	-0.046834	0.0947881	0.9599999	-0.869286	0.0680022	2.04E-37	0.0538764	0.109123	0.6215019	0.6705297
ENSG00000110700	RPS13	11	17097635	A	G	0.00994036	-0.0614521	0.0628299	0.25	0.33404	0.0394194	2.37E-17	-0.183966	0.18934	0.3312394	0.4068369
ENSG00000110711	AIP	11	67254543	G	C	0.40159	-0.012258	0.0143548	0.4799997	-0.124687	0.00817654	1.66E-52	0.0983099	0.115307	0.3938841	0.9666823
ENSG00000110713	NUP98	11	3755667	G	A	0.43837	-0.000183707	0.0143595	0.83	0.0820233	0.00802953	1.70E-24	-0.00223969	0.175066	0.9897926	0.505966
ENSG00000110717	NDUFS8	11	67801097	C	G	0.2167	0.00811592	0.0166594	0.6100002	0.371886	0.00936546	0	0.0218237	0.0448005	0.6261655	0.1389191
ENSG00000110719	TCIRG1	11	67812422	C	A	0.281312	0.0251022	0.0162307	0.05399953	-0.389511	0.00886662	0	-0.0644453	0.0416952	0.1221939	0.01895022
ENSG00000110721	CHKA	11	67854618	A	G	0.39165	-0.00683879	0.0146128	0.83	0.0807321	0.00820982	8.07E-23	-0.0847097	0.181209	0.640163	0.3873132
ENSG00000110723	EXPH5	11	108420311	C	T	0.49503	-0.00561252	0.014168	0.8800001	-0.223582	0.00789984	3.26E-176	0.0251027	0.0633744	0.6920299	0.1370042
ENSG00000110756	HPS5	11	18321984	G	A	0.127237	-0.00144598	0.0250171	0.7300002	-0.510249	0.0122599	0	0.00283387	0.0490292	0.9539082	0.9128802
ENSG00000110768	GTF2H1	11	18366216	C	T	0.416501	-0.00644837	0.0146599	0.4299995	-0.322786	0.00784905	0	0.0199772	0.0454194	0.6600535	0.9896077
ENSG00000110777	POU2AF1	11	111274666	A	C	0.293241	0.000278809	0.0154973	0.6999999	0.0607327	0.00838571	4.41E-13	0.00459076	0.255173	0.9856462	0.8245663
ENSG00000110799	VWF	12	6145988	C	T	0.527833	0.00228727	0.0141955	0.8700001	0.311101	0.0078027	0	0.00735219	0.0456303	0.8719949	0.4889575
ENSG00000110801	PSMD9	12	122341420	T	C	0.313121	-0.013732	0.0155921	0.4899999	-0.186868	0.00858494	4.77E-105	0.0734849	0.0835072	0.3788688	0.4276133
ENSG00000110811	P3H3	12	6943291	C	A	0.418489	0.00575623	0.0143752	0.7700005	0.135892	0.00931599	3.40E-48	0.0423587	0.105824	0.6889525	0.9450929
ENSG00000110841	PPFIBP1	12	27762430	T	G	0.192843	-0.000482888	0.018079	0.7700005	0.119322	0.00984116	7.81E-34	-0.00404693	0.151515	0.9786912	0.3313884
ENSG00000110848	CD69	12	9909289	G	A	0.304175	-0.0055358	0.0155345	0.5199996	0.14357	0.00861041	2.03E-62	-0.0385582	0.108226	0.7216355	0.1712751
ENSG00000110851	PRDM4	12	108140846	T	C	0.192843	-0.0174906	0.0173012	0.5	0.333055	0.0094629	2.31E-271	-0.0525156	0.0519684	0.3122412	0.6586919
ENSG00000110852	CLEC2B	12	10014159	G	C	0.428429	0.0125389	0.0142843	0.25	0.263959	0.0117572	1.26E-111	0.0475031	0.0541569	0.3804108	0.07033216
ENSG00000110871	COQ5	12	120956657	C	A	0.279324	0.00565359	0.0150615	0.4199997	0.770315	0.00860402	0	0.00733932	0.0195526	0.70739	0.2439962
ENSG00000110876	SELPLG	12	109021710	A	G	0.0705765	-0.0191649	0.0298085	0.32	-0.415982	0.015802	1.00E-152	0.0460715	0.0716795	0.5203913	0.887089
ENSG00000110880	CORO1C	12	109082128	T	C	0.281312	-0.00192841	0.0155912	0.98	-0.396116	0.00831456	0	0.00486829	0.0393603	0.9015644	0.9534366
ENSG00000110881	ASIC1	12	50464362	T	C	0.348907	-0.00279463	0.0149079	0.9199999	-0.134852	0.00976102	2.06E-43	0.0207236	0.11056	0.8513138	0.7667384
ENSG00000110888	CAPRIN2	12	30885185	G	T	0.11332	-0.0241083	0.0235182	0.2399999	-0.338521	0.0126173	1.45E-158	0.0712165	0.069524	0.3056729	0.5902351
ENSG00000110906	KCTD10	12	109900905	A	G	0.233598	0.0157371	0.0164618	0.4199997	0.697497	0.00842795	0	0.0225622	0.0236028	0.3391161	0.2607015
ENSG00000110911	SLC11A2	12	51397766	G	C	0.0705765	0.0338931	0.0289309	0.2399999	0.359075	0.0180488	4.53E-88	0.0943902	0.0807103	0.242205	0.3361613
ENSG00000110917	MLEC	12	121132169	C	T	0.470179	-0.0117106	0.0142958	0.3400001	-0.216797	0.0086707	5.61E-138	0.0540164	0.0659763	0.4129437	0.8458354
ENSG00000110921	MVK	12	110023063	A	G	0.260437	0.00923666	0.0161669	0.64	-0.106363	0.00914541	2.89E-31	-0.086841	0.152181	0.5682414	0.7306256
ENSG00000110925	CSRNP2	12	51466218	A	G	0.378728	-0.0184877	0.0147766	0.3100002	0.126875	0.00821429	8.08E-54	-0.145716	0.116848	0.2123746	0.721237
ENSG00000110931	CAMKK2	12	121705804	C	G	0.326044	-0.00531034	0.0148822	0.6300007	0.436288	0.00808657	0	-0.0121716	0.0341117	0.7212287	0.4074948
ENSG00000110934	BIN2	12	51696637	A	G	0.243539	0.00306243	0.0161465	0.98	0.370244	0.0129328	2.99E-180	0.00827138	0.0436114	0.8495747	0.4924547
ENSG00000110944	IL23A	12	56733428	C	T	0.0695825	0.0630322	0.0282033	0.01400006	0.134781	0.0163031	1.37E-16	0.467664	0.216764	0.03096852	0.2858255
ENSG00000110958	PTGES3	12	57069643	A	G	0.33996	0.014668	0.0150152	0.32	0.0963568	0.00936885	8.25E-25	0.152226	0.156531	0.330802	0.5766105
ENSG00000110987	BCL7A	12	122478638	A	G	0.531809	-0.00879893	0.0142222	0.5700002	0.184953	0.00869768	2.42E-100	-0.0475739	0.0769289	0.5363024	0.5454969
ENSG00000111011	RSRC2	12	123000368	C	T	0.454274	0.0327492	0.0142585	0.01700004	0.0645278	0.00803344	9.56E-16	0.507521	0.229823	0.02722249	0.236758
ENSG00000111052	LIN7A	12	81259001	A	G	0.0506958	-0.0128712	0.0402168	0.8700001	-0.97343	0.0200012	0	0.0132225	0.0413154	0.7489392	0.1671845
ENSG00000111057	KRT18	12	53344670	C	A	0.245527	0.0173537	0.0156643	0.14	0.108337	0.0102681	5.04E-26	0.160183	0.145384	0.2705516	0.2056094
ENSG00000111058	ACSS3	12	81491063	G	A	0.05666	0.0187574	0.0335553	0.6800001	0.209107	0.0166375	3.15E-36	0.0897025	0.160628	0.5765388	0.4409961
ENSG00000111077	TNS2	12	53449454	T	C	0.082505	-0.0218528	0.0255588	0.4600002	-0.236682	0.0168069	4.87E-45	0.0923298	0.108187	0.3934212	0.9504131
ENSG00000111087	GLI1	12	57859981	T	C	0.329026	-0.00748908	0.0152255	0.9400001	-0.0708189	0.00843865	4.77E-17	0.10575	0.215361	0.6234019	0.6045953
ENSG00000111110	PPM1H	12	63183289	G	A	0.430417	-0.00601036	0.0142172	0.5999997	0.212511	0.007957	3.86E-157	-0.0282826	0.0669094	0.6725143	0.9259182
ENSG00000111142	METAP2	12	95888455	G	C	0.0884692	0.00434722	0.021613	0.5099998	0.260539	0.0124687	5.90E-97	0.0166855	0.0829587	0.8405971	0.4048007
ENSG00000111144	LTA4H	12	96415952	C	T	0.10338	-0.000802666	0.0265583	0.9400001	-0.926957	0.0128653	0	0.000865915	0.0286511	0.9758894	0.9035435
ENSG00000111145	ELK3	12	96625886	G	C	0.234592	0.0098594	0.0168547	0.5199996	-0.198721	0.0175561	1.05E-29	-0.0496143	0.084929	0.5590959	0.9461185

ENSG00000111181	SLC6A12	12	311489	A	G	0.480119	-0.0163148	0.0143115	0.3900004	-0.229826	0.00800357	2.44E-181	0.0709875	0.06232	0.2546693	0.9359387
ENSG00000111186	WNT5B	12	1697733	G	A	0.237575	-0.00168242	0.0164243	0.8800001	0.283845	0.00906475	3.10E-215	-0.00592726	0.057864	0.918412	0.2101352
ENSG00000111196	MAGOHB	12	10762417	G	A	0.476143	0.00457654	0.0143103	0.8600001	-0.154595	0.0117742	2.22E-39	-0.0296034	0.0925937	0.7491862	0.4345823
ENSG00000111199	TRPV4	12	110246051	C	T	0.0656064	0.0209996	0.0296702	0.33	0.26746	0.0180553	1.20E-49	0.0785148	0.11106	0.4795908	0.3709877
ENSG00000111203	ITFG2	12	2945372	T	C	0.367793	-0.00986565	0.015675	0.3599996	0.227207	0.00855471	2.01E-155	-0.0434214	0.0690093	0.529211	0.579261
ENSG00000111215	PRH1	12	11150885	C	T	0.342942	-0.00650598	0.0144425	0.8	0.434838	0.00778315	0	-0.0149618	0.0332146	0.6523788	0.02979245
ENSG00000111224	PARP11	12	3941410	A	C	0.16004	0.0193743	0.0197645	0.35	-0.161078	0.010613	4.99E-52	-0.120279	0.122957	0.3279658	0.6462614
ENSG00000111229	ARPC3	12	110880428	T	C	0.384692	-0.00540025	0.0145681	0.9599999	-0.236455	0.0151099	3.37E-55	0.0228384	0.0616277	0.7109458	0.4737032
ENSG00000111231	GPN3	12	110898654	C	G	0.384692	-0.00647555	0.0145611	0.91	-0.711491	0.00794718	0	0.00910138	0.0204659	0.6565292	0.5051671
ENSG00000111237	VPS29	12	110934412	A	T	0.200795	-0.0148646	0.016837	0.4199997	0.388907	0.00943172	0	-0.0382214	0.043303	0.3774249	0.6075356
ENSG00000111247	RAD51AP1	12	4658582	A	C	0.0168986	0.0478944	0.0483531	0.4700002	0.33403	0.0285877	1.53E-31	0.143384	0.145276	0.3236556	0.6219786
ENSG00000111252	SH2B3	12	111866589	C	T	0.202783	-0.0114583	0.0173751	0.5	-0.227639	0.00953532	5.82E-126	0.0503355	0.0763567	0.5097576	0.7959348
ENSG00000111254	AKAP3	12	4741443	T	C	0.477137	-0.0111801	0.0142078	0.2700001	-0.182869	0.00788441	5.26E-119	0.0611371	0.0777384	0.4316061	0.2293421
ENSG00000111261	MANSC1	12	12492836	A	G	0.153082	0.00801519	0.0213546	0.9699999	-0.215895	0.0112563	5.44E-82	-0.0371253	0.0989307	0.7074626	0.05973755
ENSG00000111266	DUSP16	12	12672073	T	C	0.083499	-0.0215862	0.0243644	0.3700002	-0.482897	0.0139163	7.85E-264	0.0447015	0.0504711	0.3757877	0.7818261
ENSG00000111269	CREBL2	12	12781401	T	C	0.315109	-0.00711245	0.0149505	0.7700005	0.111681	0.00864416	3.48E-38	-0.0636855	0.133959	0.634494	0.1756945
ENSG00000111271	ACAD10	12	112159380	G	A	0.16998	-0.00345536	0.0186307	0.6800001	0.104784	0.0104402	1.05E-23	-0.0329759	0.177831	0.8528884	0.7845268
ENSG00000111275	ALDH2	12	112226236	A	T	0.0894632	-0.00947084	0.0232813	0.6600001	0.450267	0.0131472	4.65E-257	-0.0210338	0.0517092	0.6841757	0.9982601
ENSG00000111276	CDKN1B	12	12871648	G	T	0.286282	-0.00981153	0.0156961	0.6899999	-0.127628	0.00900807	1.44E-45	0.0768759	0.123103	0.5323085	0.392228
ENSG00000111300	NAA25	12	112505663	T	C	0.082505	-0.00307402	0.026025	0.8499999	0.297303	0.0140367	1.45E-99	-0.0103397	0.0875384	0.9059756	0.3934153
ENSG00000111319	SCNN1A	12	6471452	G	A	0.412525	-0.0239865	0.0144274	0.04600023	-0.0882939	0.00809085	1.00E-27	0.271667	0.165287	0.1002587	0.2420182
ENSG00000111321	LTBR	12	6492472	C	A	0.265408	-0.00291566	0.0163409	0.83	0.294955	0.00963776	1.08E-205	-0.0098851	0.0554023	0.8583899	0.127212
ENSG00000111325	OGFOD2	12	123461858	G	T	0.311133	-0.0076656	0.0156431	0.5	0.0604286	0.00869102	3.58E-12	-0.126854	0.259511	0.6249704	0.96489
ENSG00000111328	CDK2AP1	12	123751204	A	G	0.10338	0.00203043	0.0244864	0.7499995	0.737404	0.0135602	0	0.00275348	0.0332063	0.9339147	0.7259313
ENSG00000111331	OAS3	12	113393605	C	T	0.364811	0.00294151	0.0147247	0.5199996	-0.251332	0.0088907	8.29E-176	-0.0117037	0.0585881	0.8416667	0.4955857
ENSG00000111335	OAS2	12	113432864	A	G	0.477137	0.00935823	0.0142355	0.58	0.114754	0.0079228	1.53E-47	0.0815504	0.12418	0.5113669	0.4605812
ENSG00000111341	MGP	12	15036487	T	G	0.39165	-0.00299391	0.0145331	0.7899998	0.200261	0.00823122	9.58E-131	-0.0149501	0.0725735	0.8367918	0.4355068
ENSG00000111344	RASAL1	12	113555334	G	A	0.083499	0.00810972	0.0249252	0.4799997	0.229107	0.015105	5.80E-52	0.035397	0.108818	0.7449635	0.2875812
ENSG00000111348	ARHGDIB	12	15104806	T	A	0.285288	0.0116242	0.01596	0.4299995	-0.104638	0.00906017	7.45E-31	-0.11109	0.152829	0.4672937	0.6596917
ENSG00000111358	GTF2H3	12	124132764	T	C	0.0159046	0.134901	0.0524042	0.01199997	0.547404	0.0330056	8.92E-62	0.246438	0.0968786	0.01096597	0.8136072
ENSG00000111361	EIF2B1	12	124111633	G	A	0.270378	0.00582931	0.0156145	0.7199992	0.411795	0.00870645	0	0.0141558	0.0379193	0.7089139	0.4585995
ENSG00000111364	DDX55	12	124096056	G	C	0.114314	0.0143733	0.0203438	0.25	-0.635494	0.01183	0	-0.0226175	0.0320153	0.4799029	0.08652989
ENSG00000111371	SLC38A1	12	46620323	A	G	0.275348	-0.00215275	0.0160009	0.98	-0.391303	0.00862883	0	0.00550149	0.0408915	0.8929766	0.2599167
ENSG00000111405	ENDOU	12	48111433	T	C	0.0775348	-0.0146927	0.0270745	0.4700002	0.276506	0.015783	1.02E-68	-0.053137	0.0979635	0.5875316	0.1617158
ENSG00000111412	SPRING1	12	117164734	G	A	0.265408	0.0174305	0.0165277	0.2999998	0.14678	0.0089951	7.37E-60	0.118752	0.112837	0.2926034	0.7476455
ENSG00000111424	VDR	12	48286075	C	A	0.445328	0.00418827	0.0142124	0.7499995	0.136127	0.00864109	6.51E-56	0.0307675	0.104424	0.7682691	0.09001434
ENSG00000111445	RFC5	12	118461164	G	A	0.398608	-0.00734001	0.0147488	0.56	0.181831	0.00805195	6.48E-113	-0.0403673	0.0811325	0.618803	0.4134291
ENSG00000111450	STX2	12	131298978	C	T	0.445328	-0.0333343	0.014375	0.007599937	-0.726809	0.00694841	0	0.0458639	0.0197831	0.02043095	0.5548068
ENSG00000111452	ADGRD1	12	131532233	C	G	0.138171	-0.0137242	0.0210961	0.4700002	0.41723	0.0181417	4.84E-117	-0.0328936	0.0505826	0.5155009	0.2972602
ENSG00000111481	COPZ1	12	54720309	A	G	0.127237	-0.0150512	0.0221631	0.7700005	0.15183	0.0124213	2.33E-34	-0.099132	0.146198	0.4977304	0.6376202
ENSG00000111490	TBC1D30	12	65224700	A	G	0.408549	-0.00109916	0.0143693	0.98	-0.458366	0.0142961	1.48E-225	0.002398	0.0313491	0.9390266	0.6964266
ENSG00000111530	CAND1	12	67688396	G	T	0.409543	-0.00986798	0.014496	0.33	0.077536	0.00803965	5.20E-22	-0.12727	0.187424	0.497107	0.9904084
ENSG00000111537	IFNG	12	68551037	C	T	0.28827	-0.00317935	0.0153851	0.6499995	-0.0915546	0.00850848	5.29E-27	0.0347263	0.168074	0.836312	0.424278
ENSG00000111554	MDM1	12	68696192	A	G	0.280318	-0.00818808	0.0157025	0.7400005	0.234354	0.00860765	3.17E-163	-0.0349389	0.0670155	0.6021194	0.8314622
ENSG00000111581	NUP107	12	69108649	C	T	0.34493	0.0140269	0.0152676	0.4100001	-0.446549	0.00811266	0	-0.0314118	0.034195	0.3583004	0.6863545
ENSG00000111596	CNOT2	12	70692773	T	C	0.161034	-0.000552321	0.0211392	0.6899999	-0.155617	0.0175382	7.12E-19	0.00354924	0.135842	0.9791555	0.1761597
ENSG00000111602	TIMELESS	12	56827045	G	T	0.4334	-0.0345446	0.0143638	0.00649995	-0.076482	0.00871928	1.76E-18	0.451669	0.194737	0.02037454	0.1109391
ENSG00000111615	KRR1	12	75898050	T	A	0.365805	-0.00142418	0.0147787	1	0.122079	0.00822682	8.17E-50	-0.011666	0.121061	0.9232307	0.9373066
ENSG00000111639	MRPL51	12	6602078	C	T	0.309145	0.00562115	0.0159245	0.9	-0.196189	0.00873785	1.20E-111	-0.0286517	0.0811791	0.7241305	0.3187486
ENSG00000111640	GAPDH	12	6645315	G	A	0.220676	-0.0156404	0.0173627	0.4199997	-0.395194	0.0112926	2.60E-268	0.0395765	0.0439492	0.3678509	0.3531244

ENSG00000111641	NOP2	12	6671943	A	C	0.028827	0.0201679	0.0620137	0.89	0.157669	0.0265446	2.85E-09	0.127913	0.393904	0.7453851	0.6008271
ENSG00000111642	CHD4	12	6697945	A	G	0.134195	0.0199355	0.0192086	0.2700001	-0.148466	0.0109428	6.25E-42	-0.134277	0.129759	0.3007534	0.38499
ENSG00000111644	ACRBP	12	6751933	T	C	0.0328032	-0.0353357	0.0371125	0.33	-0.215875	0.0221687	2.08E-22	0.163686	0.172737	0.3433305	0.9976109
ENSG00000111647	BLTP3B	12	100479429	G	T	0.0685885	-0.0264179	0.0292091	0.17	-0.524955	0.0160943	2.32E-233	0.0503241	0.0556625	0.365946	0.9967309
ENSG00000111652	COPS7A	12	6836974	A	G	0.192843	0.0108755	0.0181314	0.7199992	0.205768	0.0103768	1.65E-87	0.0528532	0.0881561	0.5488118	0.4272763
ENSG00000111653	ING4	12	6765880	A	G	0.0198807	0.011895	0.0486477	0.7199992	-0.211198	0.0271044	6.60E-15	-0.0563216	0.230455	0.8069266	0.2917516
ENSG00000111664	GNB3	12	6952837	A	G	0.354871	0.0108758	0.0148348	0.5300002	0.130427	0.00898433	9.43E-48	0.0833859	0.113885	0.4640499	0.792959
ENSG00000111666	CHPT1	12	102114321	G	C	0.394632	-0.00922086	0.0144041	0.7199992	-0.649547	0.00722255	0	0.0141958	0.0221762	0.5220822	0.301341
ENSG00000111667	USP5	12	6968544	G	A	0.00795229	-0.103384	0.183751	0.5300002	-0.776099	0.127841	1.27E-09	0.13321	0.237777	0.5753225	NA
ENSG00000111669	TPI1	12	6978197	C	A	0.309145	-0.0126362	0.0157343	0.32	-0.201548	0.00963163	3.13E-97	0.0626958	0.0781248	0.4222591	0.8185001
ENSG00000111670	GNPTAB	12	102181995	C	T	0.219682	-0.00473197	0.017736	0.6999999	-0.19723	0.00974956	5.37E-91	0.0239922	0.0899335	0.7896408	0.1397035
ENSG00000111671	SPSB2	12	6989310	G	A	0.303181	-0.0161291	0.0158113	0.2300001	0.777287	0.00766642	0	-0.0207505	0.0203427	0.3077057	0.6594308
ENSG00000111674	ENO2	12	7027885	C	A	0.16501	0.0121718	0.0187697	0.3400001	0.07462	0.0115618	1.09E-10	0.163117	0.252804	0.5187766	0.7053565
ENSG00000111676	ATN1	12	7042555	A	G	0.349901	0.0119616	0.0151485	0.6200004	-0.104594	0.00882158	1.99E-32	-0.114362	0.145152	0.4307684	0.9930365
ENSG00000111678	C12orf57	12	7053653	A	G	0.0506958	0.0397756	0.0311579	0.2200002	0.363257	0.0232857	7.28E-55	0.109497	0.0860604	0.2032564	0.967275
ENSG00000111684	LPCAT3	12	7105581	T	C	0.084493	-0.00934522	0.0260719	0.7600007	-0.22654	0.0135729	1.53E-62	0.0412519	0.115114	0.7200757	0.637405
ENSG00000111696	NT5DC3	12	104199603	A	C	0.168986	0.015997	0.0202605	0.5500004	1.10638	0.00919168	0	0.0144589	0.0183129	0.4297913	0.05258305
ENSG00000111707	SUDS3	12	118835012	T	C	0.0218688	-0.0192462	0.0558199	0.6200004	0.238176	0.0232221	1.11E-24	-0.0808068	0.234497	0.7303978	0.9388434
ENSG00000111711	GOLT1B	12	21663028	C	T	0.292247	0.0151415	0.0158611	0.4	0.263404	0.00880774	1.64E-196	0.0574839	0.0602465	0.3400102	0.2860794
ENSG00000111716	LDHB	12	21849533	A	G	0.0248509	0.0430007	0.053727	0.4600002	-0.304299	0.0463234	5.07E-11	-0.141311	0.177866	0.4269158	0.3881254
ENSG00000111725	PRKAB1	12	120112496	A	G	0.0675944	-0.0359817	0.0271954	0.2399999	0.809455	0.0146735	0	-0.0444517	0.0336068	0.1859352	0.5974989
ENSG00000111726	CMAS	12	22208858	T	G	0.148111	0.00307486	0.0193255	0.81	0.0704712	0.0101011	3.02E-12	0.0436328	0.274304	0.8736156	0.3785489
ENSG00000111728	ST8SIA1	12	22403341	G	A	0.0616302	0.0131773	0.0315017	0.81	0.294087	0.0168177	1.81E-68	0.0448074	0.107147	0.675812	0.8266541
ENSG00000111729	CLEC4A	12	8283715	A	G	0.37674	-0.0102636	0.0147968	0.5	0.244228	0.0080538	5.43E-202	-0.0420246	0.0606018	0.488024	0.8923295
ENSG00000111731	C2CD5	12	22649498	T	C	0.0596421	-0.0239197	0.0323074	0.35	0.682089	0.0186904	1.38E-291	-0.0350683	0.0473751	0.4591628	0.9396251
ENSG00000111737	RAB35	12	120544102	T	C	0.0109344	0.00934811	0.0565592	0.7700005	0.181811	0.0317141	9.88E-09	0.0514167	0.311218	0.8687776	0.7361346
ENSG00000111752	PHC1	12	9080277	T	G	0.112326	0.00954472	0.0228168	0.59	0.312585	0.0195129	9.35E-58	0.0305348	0.0730189	0.6758175	0.008332379
ENSG00000111785	RIC8B	12	107225731	C	G	0.230616	0.00578685	0.0167889	0.58	0.071052	0.00961566	1.48E-13	0.0814452	0.236547	0.7306141	0.6278424
ENSG00000111786	SRSF9	12	120903533	A	C	0.372763	-0.0258493	0.0150079	0.02999991	0.113064	0.012326	4.61E-20	-0.228625	0.135057	0.0904943	0.8581127
ENSG00000111788	NA	12	9448257	G	A	0.462227	-0.0124724	0.0141954	0.5400003	-1.01796	0.0132407	0	0.0122524	0.0139459	0.379637	0.5841566
ENSG00000111790	FGFR1OP2	12	27105449	T	C	0.111332	0.00372769	0.0227741	0.9699999	0.107346	0.0132056	4.34E-16	0.034726	0.212199	0.8700083	0.944577
ENSG00000111796	KLRB1	12	9753814	C	T	0.422465	0.0167616	0.0144289	0.25	-0.218386	0.00806649	2.04E-161	-0.0767523	0.0661315	0.2458043	0.5179283
ENSG00000111801	BTN3A3	6	26447171	C	T	0.00397614	0.147171	0.213099	0.4400003	-1.45074	0.0765285	3.88E-80	-0.101446	0.146988	0.4900905	0.5387863
ENSG00000111802	TDP2	6	24658733	T	G	0.324056	-0.000373268	0.0154839	0.89	0.168802	0.00857628	3.05E-86	-0.00221128	0.0917284	0.9807674	0.1284729
ENSG00000111817	DSE	6	116667389	G	A	0.358847	-0.000666097	0.0148401	0.9699999	-0.524583	0.00786362	0	0.00126977	0.0282893	0.964199	0.2541182
ENSG00000111832	RWDD1	6	116905684	A	G	0.0298211	-0.0195128	0.0411389	0.6100002	0.571243	0.0249321	3.53E-116	-0.0341585	0.0720318	0.6353477	0.9573392
ENSG00000111834	RSPH4A	6	116945895	C	T	0.0298211	-0.038195	0.0412367	0.33	-0.484368	0.0274225	8.05E-70	0.0788553	0.085252	0.354983	0.8037117
ENSG00000111837	MAK	6	10800860	C	T	0.353877	-0.00422172	0.0151701	0.9400001	-0.237212	0.0085646	7.63E-169	0.0177972	0.0639549	0.7807989	0.1596191
ENSG00000111843	TMEM14C	6	10727255	C	A	0.135189	0.0347453	0.0204591	0.1299999	0.578824	0.0130422	0	0.0600274	0.0353718	0.0896893	0.5969164
ENSG00000111845	PAK1IP1	6	10702471	C	T	0.0308151	0.0142835	0.0439097	0.81	-0.326036	0.0293885	1.34E-28	-0.0438096	0.134735	0.7450654	0.5508148
ENSG00000111846	GCNT2	6	10561028	T	A	0.488072	0.00879417	0.0142007	0.5099998	0.502875	0.00752084	0	0.0174878	0.0282402	0.5357512	0.5767736
ENSG00000111850	SMIM8	6	88042174	A	G	0.115308	0.0343798	0.0223023	0.07599937	0.330999	0.013688	3.47E-129	0.103867	0.0675155	0.1239476	0.839802
ENSG00000111859	NEDD9	6	11283056	T	C	0.172962	0.0220936	0.0188089	0.2999998	0.195438	0.0104177	1.60E-78	0.113047	0.0964283	0.2410607	0.08643261
ENSG00000111860	CEP85L	6	118906586	A	G	0.310139	0.00839685	0.0162397	0.64	-0.225311	0.00948472	9.71E-125	-0.0372678	0.0720938	0.6052019	0.6389283
ENSG00000111863	ADTRP	6	11759783	G	A	0.342942	0.00057132	0.0151338	0.83	-0.325368	0.0162385	2.63E-89	-0.00175592	0.0465129	0.9698861	0.4797639
ENSG00000111875	ASF1A	6	119222858	T	C	0.195825	0.012308	0.0191928	0.6200004	0.474959	0.00964704	0	0.0259138	0.0404128	0.5213747	0.6177205
ENSG00000111877	MCM9	6	119195466	T	C	0.457256	-0.0138003	0.0142626	0.3800004	-0.145778	0.00870432	5.88E-63	0.0946664	0.0980008	0.3340563	0.5247836
ENSG00000111879	FAM184A	6	119375740	C	T	0.510934	-0.000275981	0.0143742	0.9	0.0654746	0.00795193	1.81E-16	-0.00421509	0.219539	0.9846818	0.4035172
ENSG00000111880	RNGTT	6	89496666	G	C	0.16501	-0.0299814	0.0198853	0.1299999	-0.526127	0.0107212	0	0.0569851	0.0378134	0.1318092	0.4093613
ENSG00000111885	MAN1A1	6	119584650	C	T	0.382704	0.0240139	0.0146506	0.06699926	-0.30605	0.00816411	1.48E-307	-0.0784639	0.0479156	0.1015172	0.03548409

ENSG00000111886	GABRR2	6	89995972	T	C	0.456262	-0.00921191	0.0142255	0.4899999	0.14218	0.00791087	3.18E-72	-0.0647903	0.100117	0.5175392	0.8112564
ENSG00000111897	SERINC1	6	122835881	C	A	0.153082	0.00330847	0.0192476	0.8499999	-0.3548	0.0110258	3.46E-227	-0.0093249	0.05425	0.8635259	0.328767
ENSG00000111906	HDDC2	6	125609889	T	C	0.473161	-0.00696385	0.0142569	0.4400003	-0.702564	0.00690703	0	0.00991205	0.0202929	0.6252318	0.8375139
ENSG00000111907	TPD52L1	6	125512874	T	A	0.161034	-0.0213896	0.019047	0.2399999	-0.175802	0.0111653	7.39E-56	0.121669	0.108619	0.2626519	0.6860989
ENSG00000111912	NCOA7	6	126177286	G	T	0.312127	-0.00532688	0.0153355	0.8600001	0.252656	0.008434	3.59E-197	-0.0210835	0.0607013	0.7283413	0.4144657
ENSG00000111913	RIPOR2	6	24919919	A	C	0.367793	-0.017099	0.01492	0.16	-0.245783	0.00822377	2.91E-196	0.0695695	0.0607486	0.2521247	0.8696314
ENSG00000111961	SASH1	6	148733313	C	A	0.230616	0.0116578	0.0174608	0.5300002	0.313093	0.00948879	9.24E-239	0.0372343	0.0557801	0.5044407	0.5556883
ENSG00000111962	UST	6	149233295	G	A	0.10338	-0.0141386	0.0225207	0.5700002	-0.413129	0.0129313	5.78E-224	0.0342232	0.0545231	0.5302113	0.6819681
ENSG00000112029	FBXO5	6	153298189	T	C	0.243539	-0.0242986	0.0164308	0.08300036	0.0952279	0.0089008	1.03E-26	-0.255163	0.174182	0.1429437	0.1044753
ENSG00000112031	MTRF1L	6	153316158	T	G	0.308151	-0.0441159	0.0154639	0.001899984	0.854771	0.0157258	0	-0.0516114	0.0181162	0.004386927	0.4560584
ENSG00000112033	PPARD	6	35353151	A	G	0.0208748	0.0611976	0.0506133	0.2	0.224243	0.0324736	5.01E-12	0.272908	0.229141	0.2336531	0.4001628
ENSG00000112039	FANCE	6	35427509	C	A	0.317097	0.00269081	0.0150989	0.9199999	0.08041	0.00862454	1.13E-20	0.0334636	0.187808	0.8585818	0.2946323
ENSG00000112053	SLC26A8	6	35951968	T	C	0.474155	-0.0449573	0.0141771	0.001899984	0.282794	0.00784704	2.10E-284	-0.158975	0.050326	0.001583576	0.1424507
ENSG00000112062	MAPK14	6	36037250	T	A	0.167992	0.0295409	0.018178	0.1299999	0.202765	0.00984247	2.69E-94	0.145691	0.0899292	0.1052198	0.002729601
ENSG00000112078	KCTD20	6	36434732	G	T	0.322068	0.0110292	0.0159552	0.5700002	-0.230443	0.00913621	2.24E-140	-0.047861	0.0692632	0.4895649	0.2541235
ENSG00000112079	STK38	6	36488458	T	C	0.222664	0.0199142	0.0181747	0.2999998	-0.162206	0.0105901	5.91E-53	-0.122771	0.112333	0.2744302	0.8256728
ENSG00000112081	SRSF3	6	36566677	T	C	0.253479	0.0299997	0.0170924	0.08	0.257263	0.00944322	2.00E-163	0.116611	0.066577	0.07985684	0.8802385
ENSG00000112096	NA	6	160141828	C	T	0.0596421	0.0607669	0.0322858	0.0329997	0.992312	0.018108	0	0.0612377	0.0325551	0.0599656	0.08808834
ENSG00000112110	MRPL18	6	160215156	G	A	0.242545	-0.0295737	0.0167985	0.08799946	0.606732	0.0095833	0	-0.0487426	0.0276975	0.07843897	0.1552618
ENSG00000112118	MCM3	6	52139243	C	T	0.516899	-0.00401279	0.0142199	0.6899999	-0.136937	0.00798552	6.48E-66	0.0293039	0.103857	0.777823	0.7768081
ENSG00000112130	RNF8	6	37342131	G	A	0.472167	0.0299758	0.014266	0.02999991	0.129103	0.00846788	1.74E-52	0.232185	0.111545	0.03738538	0.5355051
ENSG00000112137	PHACTR1	6	13003269	C	T	0.217694	-0.0133786	0.0166558	0.35	0.395533	0.0135137	2.57E-188	-0.0338242	0.0421256	0.4220109	0.1725432
ENSG00000112139	MDGA1	6	37632768	T	C	0.501988	-0.00796947	0.0142349	0.5300002	0.676262	0.00702331	0	-0.0117846	0.0210497	0.5755853	0.4106257
ENSG00000112144	CILK1	6	52896338	T	C	0.00497018	0.091185	0.103568	0.4100001	-0.408442	0.0505147	6.19E-16	-0.223251	0.255067	0.3814312	0.3480399
ENSG00000112149	CD83	6	14127510	G	A	0.299205	0.00485116	0.0159517	0.5400003	-0.262031	0.00893997	7.73E-189	-0.0185137	0.0608805	0.7610523	0.2890034
ENSG00000112159	MDN1	6	90440830	G	A	0.162028	-0.0195344	0.0190866	0.2399999	0.124914	0.0107871	5.20E-31	-0.156382	0.153393	0.3079716	0.4160655
ENSG00000112164	GLP1R	6	39036046	A	G	0.170974	-0.0246261	0.0201834	0.09599973	0.101336	0.0115312	1.52E-18	-0.243014	0.201083	0.2268457	0.1189594
ENSG00000112167	SAYSD1	6	39077402	G	A	0.457256	-0.0100065	0.0143272	0.4299995	0.579208	0.00819521	0	-0.0172762	0.0247371	0.4849317	0.9807089
ENSG00000112182	BACH2	6	90821437	A	G	0.17992	0.0174768	0.0185254	0.3400001	-0.236207	0.0113153	9.04E-97	-0.0739894	0.0785087	0.3459701	0.7114762
ENSG00000112195	TREML2	6	41163473	A	C	0.0258449	-0.015714	0.0417232	0.4100001	-0.498747	0.0206344	4.54E-129	0.031507	0.0836663	0.7064863	0.5386845
ENSG00000112208	BAG2	6	57043429	A	G	0.147117	0.0163606	0.0202196	0.5	0.204659	0.0191281	1.02E-26	0.0799407	0.0990785	0.4197577	0.2850126
ENSG00000112210	RAB23	6	57070329	C	T	0.15507	0.0146355	0.0200549	0.56	0.787223	0.010694	0	0.0185913	0.0254768	0.4655516	0.4597574
ENSG00000112232	KHDRBS2	6	62693135	A	C	0.15507	-0.0400472	0.0202601	0.04499974	0.142381	0.010947	1.13E-38	-0.281268	0.143929	0.05067584	0.06481919
ENSG00000112234	FBXL4	6	99358591	C	T	0.389662	0.00516705	0.0144843	0.7199992	-0.13932	0.00812732	7.19E-66	-0.0370876	0.103987	0.7213484	0.8641572
ENSG00000112237	CCNC	6	100003552	C	G	0.392644	-0.0185003	0.0143535	0.2700001	-0.0617239	0.00801401	1.34E-14	0.299727	0.235777	0.2036476	0.290609
ENSG00000112242	E2F3	6	20448169	G	A	0.399602	-0.00949037	0.0144067	0.5999997	0.0993244	0.0119786	1.12E-16	-0.0955492	0.145504	0.5113881	0.552151
ENSG00000112245	PTP4A1	6	64262579	A	G	0.0248509	0.0153619	0.0430176	0.5999997	-0.671912	0.0262265	9.23E-145	-0.022863	0.0640289	0.7210374	0.855598
ENSG00000112249	ASCC3	6	101142659	C	T	0.464215	-0.0153519	0.0142702	0.28	0.165061	0.00799813	1.27E-94	-0.0930077	0.0865717	0.2826692	0.03542954
ENSG00000112282	MED23	6	131922237	C	T	0.166998	0.0413881	0.0191271	0.025	-0.300435	0.0104679	3.75E-181	-0.13776	0.0638453	0.03094943	0.6600838
ENSG00000112290	WASF1	6	110461114	G	A	0.140159	0.01208	0.0198862	0.5099998	-0.190646	0.0109354	4.57E-68	-0.0633634	0.104373	0.5437925	0.08173189
ENSG00000112293	GPLD1	6	24460113	A	T	0.171968	0.00596444	0.0213512	0.8200001	-0.105837	0.0129954	3.82E-16	-0.0563548	0.201855	0.7801029	0.5612839
ENSG00000112294	ALDH5A1	6	24516257	A	G	0.154076	0.00669458	0.0218636	0.7800007	-0.505768	0.0117598	0	-0.0132365	0.0432296	0.7594602	0.41655
ENSG00000112297	CRYBG1	6	106989028	A	G	0.44831	-0.00224492	0.0144355	0.6999999	0.16117	0.00878369	3.37E-75	-0.0139289	0.0895701	0.8764206	0.6440437
ENSG00000112299	VNN1	6	133018958	C	T	0.343936	-0.0104648	0.0149754	0.5099998	0.790637	0.00698074	0	-0.0132359	0.0189413	0.4846857	0.4611645
ENSG00000112303	VNN2	6	133074803	A	C	0.33996	-0.0108437	0.0150716	0.3800004	-0.736367	0.00798691	0	0.014726	0.0204681	0.4718593	0.1606331
ENSG00000112304	ACOT13	6	24686278	C	A	0.126243	0.00755993	0.0212597	0.56	0.320769	0.0122208	7.55E-152	0.0235681	0.0662833	0.7221653	0.4690698
ENSG00000112305	SMAP1	6	71474598	T	G	0.530815	-0.00981367	0.0141956	0.58	-0.447929	0.00826177	0	0.021909	0.0316942	0.4894012	0.9814954
ENSG00000112306	RPS12	6	133137141	T	C	0.434394	0.00503318	0.0143597	0.8700001	0.240265	0.0176059	2.11E-42	0.0209485	0.0597859	0.7260447	0.952657
ENSG00000112308	C6orf62	6	24713076	C	A	0.149105	0.00143982	0.0178578	0.95	0.533617	0.00999835	0	0.00269823	0.0334656	0.9357387	0.1577412
ENSG00000112309	B3GAT2	6	71616561	T	C	0.468191	0.00913085	0.0142016	0.6200004	0.13116	0.00898273	2.75E-48	0.0696159	0.108382	0.5206636	0.9115065

ENSG00000112312	GMNN	6	24780743	T	G	0.116302	-0.0137283	0.0222635	0.6499995	0.154563	0.0127204	5.68E-34	-0.0888199	0.144227	0.538003	0.1833799
ENSG00000112320	SOBP	6	107896259	G	A	0.2833	0.00300323	0.0160921	0.81	-0.0842874	0.00864965	1.95E-22	-0.0356308	0.190954	0.8519795	0.7148434
ENSG00000112335	SNX3	6	108557445	T	C	0.0238569	-0.0796648	0.0464981	0.04499974	0.170124	0.0253357	1.88E-11	-0.468274	0.282075	0.09689329	0.6861166
ENSG00000112339	HBS1L	6	135352855	T	C	0.491054	0.0119112	0.0141806	0.4600002	-0.207638	0.00786642	1.54E-153	-0.0573652	0.0683294	0.4011666	0.1790718
ENSG00000112343	TRIM38	6	25974189	G	A	0.437376	0.0416576	0.0142914	0.000959997	0.261853	0.00798509	7.48E-236	0.159088	0.0547932	0.003691061	0.2384585
ENSG00000112357	PEX7	6	137189396	G	A	0.0984095	0.0214758	0.0209387	0.4	-0.36834	0.0181604	1.83E-91	-0.0583043	0.0569188	0.3056737	0.05134474
ENSG00000112365	ZBTB24	6	109794118	T	C	0.355865	-0.00207107	0.0146116	0.9699999	0.0988916	0.00913126	2.48E-27	-0.0209428	0.147766	0.8872938	0.9677536
ENSG00000112367	FIG4	6	110079565	C	T	0.410537	0.00440246	0.0143018	0.6200004	0.217381	0.00802381	1.22E-161	0.0202523	0.0657956	0.7582302	0.8002676
ENSG00000112378	PERP	6	138420285	C	G	0.116302	0.0133794	0.0254075	0.6999999	-0.153447	0.0139685	4.50E-28	-0.0871924	0.165769	0.5988962	0.9710122
ENSG00000112394	SLC16A10	6	111476502	T	A	0.0149105	0.0236323	0.061301	0.7899998	-0.273456	0.0364142	5.93E-14	-0.0864208	0.224466	0.7002334	0.3599201
ENSG00000112406	HECA	6	139479094	C	T	0.0467197	0.0591471	0.0340533	0.08600031	0.150982	0.0184367	2.63E-16	0.39175	0.230563	0.0893001	0.4977388
ENSG00000112414	ADGRG6	6	142695197	C	A	0.227634	0.004595	0.0179866	0.7199992	-0.24922	0.0100404	5.22E-136	-0.0184375	0.0721755	0.7983721	0.3061656
ENSG00000112419	PHACTR2	6	144005152	G	A	0.192843	-0.0217217	0.0176668	0.28	-0.166475	0.00990866	2.40E-63	0.13048	0.106406	0.2201077	0.8566518
ENSG00000112425	EPM2A	6	145939939	A	C	0.481113	0.0174103	0.0142246	0.2399999	-0.245567	0.00793489	2.73E-210	-0.0708985	0.0579709	0.2213293	0.2222148
ENSG00000112486	CCR6	6	167539239	A	G	0.423459	-0.0258443	0.0142988	0.04700023	0.208679	0.0080536	4.98E-148	-0.123847	0.0686872	0.07137816	0.2103686
ENSG00000112511	PHF1	6	33381203	G	T	0.028827	0.113078	0.0545409	0.04900044	-0.295338	0.044033	1.98E-11	-0.382876	0.193294	0.04761413	0.4092596
ENSG00000112514	CUTA	6	33385156	A	G	0.115308	0.00825263	0.0205454	0.4899999	-0.37885	0.018161	1.22E-96	-0.0217834	0.054241	0.687976	0.9455091
ENSG00000112531	QKI	6	163917330	A	G	0.0755467	-0.00810096	0.0255123	0.81	-0.728709	0.0146433	0	0.0111169	0.035011	0.7508452	0.9155102
ENSG00000112561	TFEB	6	41677856	C	T	0.455268	0.0208651	0.0142294	0.17	0.0485634	0.00795886	1.05E-09	0.429647	0.301349	0.1539415	0.7197913
ENSG00000112576	CCND3	6	41960383	A	G	0.242545	0.036679	0.0175479	0.05	-0.180754	0.00971953	3.40E-77	-0.202922	0.0976929	0.03778839	0.9674307
ENSG00000112578	BYSL	6	41894855	T	C	0.246521	0.0161413	0.0161476	0.1900002	0.325869	0.00988628	2.88E-238	0.0495331	0.0495753	0.3177218	0.01182153
ENSG00000112584	FAM120B	6	170657972	C	T	0.198807	-0.00646235	0.0167728	0.56	-0.638095	0.00898186	0	0.0101276	0.0262861	0.7000286	0.8333117
ENSG00000112592	TBP	6	170872674	A	G	0.0447316	0.00475882	0.0368592	0.9	0.274875	0.0198248	1.03E-43	0.0173127	0.1341	0.8972766	0.8919279
ENSG00000112599	GUCA1B	6	42157396	A	G	0.159046	-0.0208949	0.0191389	0.3700002	-0.362158	0.0181035	5.00E-89	0.0576956	0.0529255	0.2756567	0.8996901
ENSG00000112619	PRPH2	6	42677326	C	G	0.451292	-0.00960378	0.0141861	0.4899999	-0.111573	0.00812518	6.55E-43	0.0860759	0.1273	0.4989365	0.06487582
ENSG00000112624	BICRAL	6	42793031	C	T	0.515905	0.0118283	0.0142636	0.2999998	0.173721	0.00904648	3.48E-82	0.0680878	0.0821827	0.4073916	0.04808328
ENSG00000112640	PPP2R5D	6	42966158	C	T	0.122266	-0.0094577	0.0207758	0.7600007	-0.170881	0.0117506	6.53E-48	0.0553466	0.12164	0.6491064	0.659244
ENSG00000112651	MRPL2	6	43024655	C	T	0.281312	-0.0222349	0.015918	0.1299999	0.204705	0.00901626	4.09E-114	-0.108619	0.0779076	0.163256	0.04205919
ENSG00000112655	PTK7	6	43086731	C	T	0.0894632	0.00358537	0.0210314	0.7199992	-0.229352	0.0119889	1.41E-81	-0.0156326	0.0917029	0.8646403	0.09820179
ENSG00000112658	SRF	6	43144140	A	G	0.329026	-0.00618417	0.0156633	0.59	-0.0553765	0.00871001	2.05E-10	0.111675	0.283396	0.6935367	0.08781312
ENSG00000112659	CUL9	6	43171119	C	T	0.120278	0.013133	0.0195972	0.35	-0.146164	0.0110311	4.50E-40	-0.0898514	0.134249	0.5033097	0.2329304
ENSG00000112664	NA	6	34308224	T	C	0.126243	-0.0669434	0.0228164	0.001400006	0.324056	0.0296358	7.88E-28	-0.20658	0.0728994	0.004600316	0.6585319
ENSG00000112667	DNPH1	6	43195294	A	G	0.0705765	-0.00843055	0.0231304	0.8600001	-0.59151	0.0144834	0	0.0142526	0.0391055	0.715511	0.4191004
ENSG00000112679	DUSP22	6	321726	C	T	0.137177	0.0372458	0.0214517	0.1299999	0.0971007	0.0119866	5.46E-16	0.383579	0.22594	0.08956322	0.5734854
ENSG00000112685	EXOC2	6	589125	C	G	0.232604	0.0193576	0.0167836	0.5199996	0.431155	0.00908337	0	0.0448971	0.0389386	0.2489007	0.02613934
ENSG00000112697	TMEM30A	6	75978662	A	G	0.0497018	-0.0388164	0.0331635	0.17	-0.486035	0.0163283	1.06E-194	0.0798633	0.0682854	0.2421814	0.9850136
ENSG00000112699	GMDS	6	1934983	T	C	0.00994036	0.0171705	0.0599115	0.7800007	0.910681	0.0404183	2.04E-112	0.0188546	0.0657929	0.7744381	0.6486875
ENSG00000112701	SENP6	6	76369611	C	T	0.524851	-0.00482361	0.014185	0.6499995	0.39793	0.00766421	0	-0.0121217	0.0356477	0.7338243	0.4738076
ENSG00000112715	VEGFA	6	43746072	G	T	0.414513	0.0334995	0.014544	0.025	0.236327	0.0080003	8.88E-192	0.14175	0.0617286	0.02165583	0.4366469
ENSG00000112739	PRPF4B	6	4043359	C	T	0.430417	0.00762906	0.0143216	0.5400003	-0.0440102	0.00799692	3.73E-08	-0.173347	0.326936	0.5959608	0.8481833
ENSG00000112742	TTK	6	80732924	T	C	0.436382	-0.00903772	0.0143753	0.4299995	0.0487087	0.00799605	1.12E-09	-0.185546	0.296696	0.5317243	0.1874078
ENSG00000112759	SLC29A1	6	44194565	A	T	0.196819	0.0191198	0.0200193	0.29	0.149367	0.011288	5.70E-40	0.128006	0.134376	0.3407977	0.701131
ENSG00000112763	BTN2A1	6	26467499	C	G	0.243539	-0.00622076	0.0170793	0.58	0.561468	0.00888882	0	-0.0110795	0.0304195	0.7156922	0.08452614
ENSG00000112773	TENT5A	6	82331823	T	C	0.22167	-0.0127641	0.0177409	0.4700002	0.109239	0.0101039	3.03E-27	-0.116845	0.162763	0.472828	0.2227031
ENSG00000112782	CLIC5	6	45958088	C	T	0.45328	0.00718657	0.0144917	0.8	0.136368	0.00795745	7.83E-66	0.0526997	0.106313	0.6201048	0.5620563
ENSG00000112787	FBRSL1	12	133113955	G	A	0.308151	0.012953	0.0156101	0.4600002	-0.250694	0.0101872	1.02E-133	-0.0516686	0.0623029	0.4069279	0.5127104
ENSG00000112796	ENPP5	6	46132816	A	G	0.465209	0.0013237	0.0142134	0.84	0.0957852	0.00794423	1.78E-33	0.0138195	0.148393	0.9258022	0.2422424
ENSG00000112799	LY86	6	6621778	G	A	0.462227	-0.00812855	0.014354	0.64	0.545657	0.0074252	0	-0.0148968	0.0263067	0.571207	0.2286195
ENSG00000112812	PRSS16	6	27219941	T	G	0.172962	-0.00661268	0.0175155	0.7700005	0.33233	0.0150085	1.22E-108	-0.0198979	0.0527127	0.7058183	0.5768837
ENSG00000112851	ERBIN	5	65300340	G	A	0.00994036	-0.0339555	0.0474311	0.4400003	-0.196341	0.0274104	7.89E-13	0.172942	0.242779	0.4762529	0.7382143

ENSG00000112855	HARS2	5	140074950	A	G	0.425447	0.00203895	0.0143298	0.98	0.112711	0.00797602	2.44E-45	0.0180901	0.127144	0.8868587	0.4750513
ENSG00000112874	NUDT12	5	102891525	C	A	0.162028	0.00445194	0.0197136	0.7499995	-0.454984	0.0109238	0	-0.00978482	0.0433287	0.8213355	0.393504
ENSG00000112877	CEP72	5	639835	G	A	0.436382	0.0113228	0.0143855	0.58	-0.125502	0.00963033	8.05E-39	-0.0902197	0.114832	0.4320629	0.3322095
ENSG00000112893	MAN2A1	5	109115196	A	G	0.16004	0.0176135	0.0187931	0.29	-0.175813	0.0106804	6.97E-61	-0.100183	0.107066	0.349419	0.8659864
ENSG00000112902	SEMA5A	5	9290662	G	C	0.0815109	-0.00889807	0.0240051	0.6600001	0.420445	0.0148941	2.58E-175	-0.0211634	0.0570994	0.710904	0.08295167
ENSG00000112941	TENT4A	5	6735939	T	C	0.0218688	-0.0352041	0.0477849	0.3400001	-0.590537	0.0260762	1.51E-113	0.0596137	0.0809605	0.4615295	0.184931
ENSG00000112972	HMGCS1	5	43301555	T	C	0.543738	-0.000880848	0.0142106	0.9299999	0.0476821	0.00796295	2.12E-09	-0.0184733	0.298044	0.9505772	0.9507536
ENSG00000112977	DAP	5	10720363	A	G	0.33996	0.00255934	0.0153127	0.89	-0.431926	0.00813816	0	-0.00592541	0.0354523	0.8672618	0.63076
ENSG00000112992	NNT	5	43655150	G	A	0.120278	0.0043369	0.0221484	0.81	0.654686	0.0113501	0	0.0066244	0.0338308	0.844759	0.8151078
ENSG00000112996	MRPS30	5	44814778	C	T	0.371769	0.0287664	0.014688	0.03699985	0.0706303	0.00816725	5.24E-18	0.407281	0.213222	0.05611698	0.499989
ENSG00000113068	PFDN1	5	139653665	T	A	0.236581	0.00573325	0.0169408	0.81	0.221969	0.00992104	7.12E-111	0.025829	0.0763292	0.7350694	0.3180386
ENSG00000113070	HBEGF	5	139719322	T	C	0.237575	0.00523813	0.0169266	0.83	0.167283	0.00952377	4.58E-69	0.031313	0.101201	0.7570071	0.5442733
ENSG00000113083	LOX	5	121406435	T	G	0.559642	0.0173676	0.0142144	0.2300001	0.0549082	0.0080617	9.69E-12	0.316302	0.263008	0.2291181	0.2869997
ENSG00000113088	GZMK	5	54325239	C	T	0.455268	0.00849292	0.0143179	0.59	-0.174961	0.00797909	1.42E-106	-0.0485419	0.0818649	0.5532139	0.7446628
ENSG00000113108	APBB3	5	139955595	C	A	0.0497018	-0.0302901	0.0292061	0.32	0.404471	0.019898	7.39E-92	-0.0748881	0.072302	0.3003103	0.261964
ENSG00000113119	TMCO6	5	140022002	C	T	0.253479	-0.0141753	0.0166677	0.3599996	0.170988	0.00916961	1.33E-77	-0.0829025	0.0975803	0.3955575	0.8666173
ENSG00000113140	SPARC	5	151053691	A	C	0.529821	-0.0195853	0.0141759	0.33	0.156818	0.00791529	2.35E-87	-0.124892	0.0906168	0.1681279	0.6628387
ENSG00000113141	IK	5	140034353	C	T	0.403579	0.00264178	0.0143997	0.99	0.224989	0.0079966	3.60E-174	0.0117418	0.0640032	0.8544396	0.7407701
ENSG00000113161	HMGCR	5	74645041	G	A	0.289264	-0.00274854	0.0156428	0.6800001	0.127763	0.00853654	1.21E-50	-0.0215128	0.122444	0.8605341	0.5468604
ENSG00000113163	CERT1	5	74736137	A	G	0.100398	0.0351257	0.0243134	0.1900002	-0.119116	0.0142993	8.07E-17	-0.294886	0.207162	0.1546038	0.9557183
ENSG00000113194	FAF2	5	175905852	C	T	0.326044	-0.0155246	0.0154998	0.28	0.0671922	0.00870229	1.15E-14	-0.231048	0.232611	0.3205746	0.2171903
ENSG00000113231	PDE8B	5	76615953	A	G	0.449304	0.00261801	0.0142158	0.8499999	-0.249221	0.00788748	4.05E-219	-0.0105048	0.0570418	0.8538888	0.2056953
ENSG00000113240	CLK4	5	178043640	A	G	0.252485	-0.00068291	0.0170975	0.98	-0.464965	0.0095958	0	0.00146874	0.0367716	0.9681393	0.2000821
ENSG00000113249	HAVCR1	5	156471277	T	G	0.357853	0.02354	0.0147765	0.07900053	0.098251	0.00901336	1.14E-27	0.23959	0.151993	0.1149509	0.1505094
ENSG00000113263	ITK	5	156626072	A	G	0.227634	0.0116848	0.0167306	0.58	0.225609	0.00887064	1.08E-142	0.0517923	0.0741855	0.4850861	0.9553583
ENSG00000113269	RNF130	5	179422514	T	C	0.0347913	0.0907016	0.0345092	0.004399973	0.5038	0.0232153	2.00E-104	0.180035	0.0689983	0.009073738	0.587695
ENSG00000113272	THG1L	5	157163330	C	T	0.348907	0.0273607	0.0150358	0.14	-0.451836	0.00798442	0	-0.0605545	0.0332943	0.06894751	0.9082325
ENSG00000113273	ARSB	5	78177471	C	T	0.17992	0.0286032	0.018468	0.2	-0.475088	0.0107154	0	-0.0602061	0.0388965	0.1216575	0.4917074
ENSG00000113282	CLINT1	5	157249467	C	T	0.424453	-0.0012785	0.014413	0.81	-0.0659503	0.00799494	1.60E-16	0.0193858	0.218556	0.9293207	0.1344876
ENSG00000113296	THBS4	5	79333122	C	T	0.286282	0.011561	0.0160022	0.3900004	-0.0627144	0.0091626	7.67E-12	-0.184344	0.256578	0.4724664	0.6483362
ENSG00000113300	CNOT6	5	179963408	C	T	0.0934394	-0.00340485	0.0225428	0.9699999	0.752348	0.0123887	0	-0.00452563	0.0299634	0.879945	0.2319524
ENSG00000113303	BTNL8	5	180351991	C	T	0.293241	-0.0142114	0.0156811	0.32	-0.644924	0.00809789	0	0.0220358	0.0243162	0.364821	0.889952
ENSG00000113318	MSH3	5	80061373	C	T	0.245527	-0.0127339	0.016567	0.3700002	0.858351	0.0114426	0	-0.0148353	0.019302	0.4421367	0.6005143
ENSG00000113319	RASGRF2	5	80391233	A	G	0.27336	0.00874374	0.015779	0.4299995	0.179026	0.00900569	6.16E-88	0.0488407	0.0881723	0.5796318	0.01394689
ENSG00000113328	CCNG1	5	162868866	T	C	0.300199	0.0219344	0.0155446	0.1499999	0.130856	0.00902144	1.13E-47	0.167622	0.119352	0.1601901	0.8572503
ENSG00000113356	POLR3G	5	89788967	C	A	0.408549	-0.0106252	0.0143427	0.5199996	0.117488	0.00987116	1.15E-32	-0.0904361	0.122314	0.4596774	0.7174817
ENSG00000113360	DROSHA	5	31466453	C	T	0.0357853	0.0199892	0.0391514	0.7499995	0.269275	0.0234495	1.60E-30	0.0742333	0.145539	0.6100111	0.8204867
ENSG00000113368	LMNB1	5	126142513	A	G	0.261431	0.00726926	0.0158719	0.5099998	0.335987	0.0130712	1.04E-145	0.0216356	0.0472472	0.6470078	0.1364753
ENSG00000113369	ARRDC3	5	90671858	C	T	0.144135	5.93E-05	0.0196758	0.99	-0.104797	0.0110338	2.14E-21	-0.000565953	0.187751	0.9975949	0.741315
ENSG00000113384	GOLPH3	5	32149633	A	C	0.027833	0.0861467	0.0540426	0.1199999	-0.760142	0.0256463	4.63E-193	-0.11333	0.0711981	0.1114406	0.7432752
ENSG00000113387	SUB1	5	32567962	C	T	0.413519	0.0184894	0.0144616	0.2200002	0.0723645	0.00808633	3.59E-19	0.255504	0.201873	0.2056329	0.8838322
ENSG00000113391	ARB2A	5	93200589	A	T	0.146123	-0.00137405	0.020441	0.84	0.191919	0.0120033	1.53E-57	-0.00715955	0.10651	0.9464068	0.8217787
ENSG00000113407	TARS1	5	33455223	A	C	0.116302	-0.00851977	0.0225609	0.5999997	0.18767	0.0118216	9.41E-57	-0.0453976	0.12025	0.7057816	0.2954845
ENSG00000113441	LNPEP	5	96322158	A	G	0.405567	-0.00347516	0.0144129	0.7600007	0.312487	0.00868432	1.55E-283	-0.011121	0.0461242	0.8094705	0.08148221
ENSG00000113448	PDE4D	5	59041406	A	G	0.479125	-0.010205	0.0142161	0.5	-0.209825	0.0117112	8.75E-72	0.0486358	0.0678066	0.4732059	0.2945822
ENSG00000113456	RAD1	5	34912231	A	G	0.286282	-0.0120832	0.015648	0.6999999	-0.0781166	0.00897315	3.16E-18	0.154681	0.201102	0.4417934	0.8484221
ENSG00000113494	PRLR	5	35139827	A	G	0.382704	-0.0121225	0.01452	0.2599998	0.162094	0.00800221	3.13E-91	-0.0747867	0.0896535	0.4041826	0.3657562
ENSG00000113504	SLC12A7	5	1081324	C	T	0.442346	0.0347126	0.0143638	0.0179999	0.648862	0.00955072	0	0.0534976	0.0221509	0.01572895	0.8402827
ENSG00000113520	IL4	5	132014023	T	C	0.167992	-0.00784841	0.0209338	0.59	-0.171233	0.01083	2.61E-56	0.0458347	0.122288	0.7078015	0.6887133
ENSG00000113522	RAD50	5	131936012	A	G	0.263419	-0.0194878	0.016292	0.2700001	-0.167731	0.00995931	1.21E-63	0.116185	0.0973765	0.2328098	0.03467541

ENSG00000113532	ST8SIA4	5	100192163	C	T	0.0984095	0.0161256	0.0240744	0.4199997	-0.419018	0.0136665	1.93E-206	-0.0384843	0.057468	0.5030722	0.8801822
ENSG00000113552	GNPDA1	5	141381960	G	A	0.163022	-0.00572206	0.0199277	0.56	0.218347	0.0108136	1.15E-90	-0.0262062	0.0912752	0.7740266	0.6280413
ENSG00000113555	PCDH12	5	141336227	A	G	0.423459	0.0017084	0.0142571	0.89	-0.136945	0.00808748	2.57E-64	-0.0124751	0.104111	0.9046218	0.1842259
ENSG00000113558	SKP1	5	133526922	C	T	0.167992	0.0406885	0.0206988	0.0530005	-0.261113	0.0130691	8.32E-89	-0.155827	0.0796542	0.05043029	0.1225409
ENSG00000113569	NUP155	5	37329761	G	C	0.0487078	-0.0148846	0.0349251	0.64	0.102302	0.0185733	3.63E-08	-0.145497	0.342413	0.6708977	0.9451109
ENSG00000113575	PPP2CA	5	133545929	T	A	0.209742	0.00607147	0.0188234	0.7899998	-0.0746898	0.0106886	2.79E-12	-0.0812891	0.252289	0.7472966	0.2640522
ENSG00000113580	NR3C1	5	142736286	G	A	0.234592	-0.000380841	0.0174296	0.8800001	0.0728593	0.00980424	1.07E-13	-0.00522708	0.239224	0.9825675	0.4770731
ENSG00000113593	PPWD1	5	64871219	G	A	0.399602	-0.0183855	0.0148304	0.1900002	0.362278	0.00833274	0	-0.0507497	0.0409531	0.2152665	0.9674474
ENSG00000113595	TRIM23	5	64903654	A	G	0.379722	-0.0135951	0.0150084	0.3700002	0.158653	0.00829482	1.51E-81	-0.0856905	0.0947047	0.3655615	0.7915095
ENSG00000113597	TRAPPC13	5	64941301	C	A	0.459245	-0.0199819	0.0142937	0.2200002	0.186104	0.00880747	4.20E-99	-0.10737	0.0769729	0.1630454	0.9020637
ENSG00000113615	SEC24A	5	134023996	C	T	0.282306	0.0146771	0.0159998	0.35	-0.0521334	0.00884997	3.84E-09	-0.28153	0.3106	0.3647209	0.26498
ENSG00000113621	TXNDC15	5	134223354	C	T	0.148111	0.0399003	0.0208873	0.03099988	0.582107	0.011312	0	0.0685446	0.0359069	0.05626839	0.9403566
ENSG00000113638	TTC33	5	40735327	C	T	0.340954	0.007213	0.0152525	0.7600007	0.185059	0.0084175	4.01E-107	0.0389768	0.0824388	0.6363582	0.2081717
ENSG00000113643	RARS1	5	167929877	T	C	0.17992	-0.0395902	0.0181159	0.03099988	0.196534	0.0106922	1.87E-75	-0.201442	0.0928261	0.02999927	0.8375985
ENSG00000113645	WWC1	5	167808982	A	C	0.138171	-0.0310762	0.02155	0.1900002	0.268085	0.0111266	2.89E-128	-0.115919	0.0805289	0.1500159	0.5723475
ENSG00000113648	MACROH2A1	5	134702597	T	C	0.0188867	0.0290002	0.0470545	0.4100001	0.323982	0.0376321	7.36E-18	0.0895117	0.14561	0.538728	0.9768247
ENSG00000113649	TCERG1	5	145859199	T	C	0.140159	-0.0426115	0.0210731	0.02	-0.184652	0.0122375	1.91E-51	0.230767	0.115144	0.04505247	0.5281217
ENSG00000113658	SMAD5	5	135496484	C	T	0.326044	0.00465756	0.0153909	0.8600001	0.656036	0.00803136	0	0.00709955	0.0234606	0.7621827	0.06492314
ENSG00000113712	CSNK1A1	5	148901383	T	G	0.264414	0.0470699	0.0160595	0.0032	-0.0513601	0.00919542	2.33E-08	-0.916468	0.353121	0.009449867	0.7719651
ENSG00000113716	HMGXB3	5	149406135	G	A	0.279324	-0.00439413	0.0160268	0.8	-0.50556	0.0191212	4.78E-154	0.00869161	0.0317028	0.7839623	0.09050418
ENSG00000113719	ERGIC1	5	172320483	T	C	0.480119	-0.023388	0.0142414	0.05999983	-0.110059	0.00856653	8.87E-38	0.212503	0.13045	0.1033131	0.4286203
ENSG00000113732	ATP6V0E1	5	172436604	A	C	0.190855	0.0082008	0.016704	0.58	0.0884892	0.00925329	1.14E-21	0.0926757	0.189017	0.6239198	0.55383
ENSG00000113734	BNIP1	5	172581417	A	C	0.116302	0.0213236	0.0210468	0.4500005	-0.449864	0.0116183	0	-0.0474001	0.0468008	0.3111533	0.2738927
ENSG00000113742	CPEB4	5	173352131	G	A	0.287276	0.0254244	0.0154519	0.09699961	0.670265	0.00780309	0	0.0379319	0.0230577	0.09995164	0.2446908
ENSG00000113758	DBN1	5	176892505	C	A	0.476143	0.00303839	0.0143032	0.9	0.340002	0.00829927	0	0.00893639	0.0420686	0.8317759	0.6126099
ENSG00000113761	ZNF346	5	176478943	G	T	0.0347913	0.0397004	0.0476758	0.7199992	0.296321	0.0344159	7.31E-18	0.133978	0.161643	0.4071901	0.3756277
ENSG00000113763	UNC5A	5	176272687	G	A	0.0576541	-0.00200322	0.0282795	0.9699999	0.227483	0.0229313	3.40E-23	-0.00880602	0.124318	0.9435293	0.6423091
ENSG00000113790	EHHADH	3	184954095	T	C	0.16004	0.0153678	0.0193595	0.5	-0.279253	0.0106752	7.79E-151	-0.0550319	0.069358	0.4275177	0.2976196
ENSG00000113810	SMC4	3	160134906	C	T	0.484095	0.0166772	0.0142306	0.2	-0.177532	0.00866349	2.54E-93	-0.093939	0.0802888	0.2419955	0.8880155
ENSG00000113811	SELENOK	3	53922226	G	A	0.452286	0.0148981	0.0142508	0.1900002	-0.108975	0.00797557	1.67E-42	-0.136711	0.131153	0.2972377	0.9625127
ENSG00000113812	ACTR8	3	53908661	A	T	0.374751	0.00744275	0.0146765	0.4199997	-0.0866648	0.00830329	1.67E-25	-0.0858798	0.169548	0.6124898	0.5494938
ENSG00000113845	TIMMDC1	3	119230658	C	T	0.0208748	-0.0272966	0.0481434	0.6899999	-0.250226	0.0251176	2.23E-23	0.109088	0.192711	0.5713467	0.7079628
ENSG00000113851	CRBN	3	3206035	G	C	0.324056	-0.00156252	0.0148918	0.99	-0.253486	0.00869758	9.84E-187	0.00616413	0.0587484	0.916436	0.3828601
ENSG00000113916	BCL6	3	187451340	G	T	0.2833	0.0089974	0.0160437	0.4899999	0.115605	0.00909133	4.82E-37	0.0778286	0.138915	0.5753013	0.2478533
ENSG00000113924	HGD	3	120374219	A	G	0.270378	-0.0167525	0.0153206	0.2200002	-0.0671563	0.00944241	1.14E-12	0.249455	0.230814	0.2798022	0.4081204
ENSG00000113971	NPHP3	3	132359144	T	C	0.370775	0.0169127	0.0154045	0.29	-0.678816	0.00763423	0	-0.024915	0.0226949	0.2722819	0.7004814
ENSG00000114013	CD86	3	121807098	C	T	0.0785288	0.00995281	0.0274639	0.7499995	0.324546	0.0148735	1.49E-105	0.0306669	0.0846343	0.7170934	0.8850639
ENSG00000114021	NIT2	3	100063997	T	C	0.346918	0.0156953	0.0150141	0.2300001	-0.285626	0.0123037	3.24E-119	-0.0549506	0.0526189	0.2963409	0.5080252
ENSG00000114023	FAM162A	3	122117102	A	G	0.408549	-0.0112434	0.0143531	0.3599996	-0.105949	0.00842293	2.77E-36	0.106121	0.135735	0.4343154	0.7137423
ENSG00000114026	OGG1	3	9810765	A	G	0.0119284	0.0371735	0.0923256	0.8600001	-0.342282	0.0404321	2.55E-17	-0.108605	0.270041	0.6875514	0.9581096
ENSG00000114030	KPNA1	3	122187294	T	C	0.154076	0.0152319	0.0202594	0.5400003	0.222187	0.0113403	1.78E-85	0.0685543	0.0912487	0.4524778	0.8430563
ENSG00000114054	PCCB	3	136012943	C	T	0.447316	0.01283	0.0144051	0.4299995	0.441175	0.00759738	0	0.0290814	0.0326555	0.3731695	0.687762
ENSG00000114098	ARMC8	3	137961670	A	G	0.0367793	-0.00730403	0.0323352	0.8800001	0.13153	0.018819	2.76E-12	-0.0555313	0.245967	0.8213827	0.06568731
ENSG00000114107	CEP70	3	138263283	A	G	0.026839	-0.0762994	0.0503933	0.06800017	0.378415	0.0223051	1.48E-64	-0.201629	0.133699	0.1315329	0.9526366
ENSG00000114120	SLC25A36	3	140679723	A	G	0.055666	-0.0432064	0.0351937	0.2399999	0.181334	0.0188371	6.19E-22	-0.23827	0.195654	0.2232959	0.9863382
ENSG00000114124	GRK7	3	141517161	C	T	0.116302	0.00691911	0.0233261	0.8800001	0.0974998	0.0138762	2.12E-12	0.0709654	0.239456	0.7669542	0.2501651
ENSG00000114125	RNF7	3	141461724	T	C	0.152087	-0.00150414	0.0202873	0.9199999	-0.26981	0.011333	2.80E-125	0.0055748	0.0751913	0.9408977	0.007013433
ENSG00000114126	TFDP2	3	141765831	G	A	0.0795229	0.00932681	0.0227117	0.64	0.252811	0.0146325	6.96E-67	0.0368924	0.089862	0.6814061	0.2632635
ENSG00000114127	XRN1	3	142096176	T	C	0.148111	0.00180626	0.0208094	0.7600007	0.190568	0.0114505	3.41E-62	0.00947827	0.109198	0.9308313	0.05321611
ENSG00000114166	KAT2B	3	20138705	T	C	0.147117	-0.0171943	0.0188732	0.3100002	0.28563	0.0117913	1.25E-129	-0.0601977	0.0661223	0.3626118	0.1678373

ENSG00000114209	PDCD10	3	167426906	T	G	0.105368	-0.0107759	0.0240511	0.6499995	0.106775	0.0139425	1.89E-14	-0.100922	0.225636	0.654675	0.8573504
ENSG00000114268	PFKFB4	3	48577282	A	G	0.0318091	-0.0118422	0.0415835	0.4799997	0.496075	0.0207695	4.41E-126	-0.0238718	0.083831	0.7758273	0.1763152
ENSG00000114270	COL7A1	3	48617103	A	G	0.336978	-0.00532208	0.0153766	0.6999999	0.061696	0.0086093	7.71E-13	-0.086263	0.249522	0.7295588	0.4329792
ENSG00000114302	PRKAR2A	3	48833654	G	A	0.11332	-0.0237122	0.0231623	0.1800002	-0.186165	0.0197447	4.16E-21	0.127372	0.12515	0.3087923	0.7164672
ENSG00000114315	HES1	3	193855227	C	A	0.0675944	-0.0660196	0.0276883	0.02199986	-0.221734	0.0169449	3.98E-39	0.297743	0.126928	0.01898823	0.1250916
ENSG00000114316	USP4	3	49346704	A	G	0.354871	0.0188363	0.0146638	0.3400001	0.0763625	0.00822765	1.68E-20	0.24667	0.193859	0.2032256	0.05288525
ENSG00000114331	ACAP2	3	195079636	G	A	0.166004	0.0321188	0.0183388	0.09099971	-0.114433	0.012144	4.38E-21	-0.280679	0.163003	0.08508364	0.5914451
ENSG00000114353	GNAI2	3	50280255	T	C	0.101392	0.036604	0.0239986	0.05	-0.114887	0.0147234	6.05E-15	-0.318609	0.212842	0.1344125	0.706906
ENSG00000114354	TFG	3	100448007	C	T	0.354871	-0.0142297	0.0148991	0.6300007	0.568363	0.00800029	0	-0.0250363	0.0262164	0.3395854	0.04464948
ENSG00000114383	TUSC2	3	50361570	G	A	0.11332	-0.00305425	0.0234149	0.7300002	-0.119509	0.0146267	3.07E-16	0.0255567	0.195951	0.8962311	0.128924
ENSG00000114395	CYB561D2	3	50392008	T	C	0.112326	0.0251155	0.0238814	0.16	-0.118366	0.0155617	2.82E-14	-0.212186	0.203679	0.2975204	0.2398062
ENSG00000114405	CEP15	3	62313268	A	C	0.395626	-0.00326224	0.0144238	0.64	-0.214342	0.0079251	4.27E-161	0.0152198	0.0672959	0.8210749	0.2659743
ENSG00000114423	CBLB	3	105481350	G	A	0.274354	-0.0222391	0.0158976	0.28	-0.359956	0.00925536	0	0.0617829	0.044194	0.1621153	0.4915025
ENSG00000114439	BBX	3	107385977	A	G	0.104374	0.00182157	0.0216772	0.59	-0.271839	0.0127104	1.76E-101	-0.0067009	0.0797433	0.9330318	0.2090933
ENSG00000114446	IFT57	3	107910538	T	A	0.106362	-0.0411003	0.0231088	0.06100002	0.249928	0.0131353	1.02E-80	-0.164448	0.0928648	0.07658785	0.7217838
ENSG00000114450	GNB4	3	179143184	A	G	0.229622	0.0039906	0.016838	0.5999997	0.117146	0.00977193	4.11E-33	0.0340652	0.143763	0.812693	0.9648798
ENSG00000114473	IQCG	3	197651479	G	C	0.181909	-0.00615262	0.0191429	0.7099994	0.454828	0.011216	0	-0.0135273	0.0420895	0.7479113	0.08642281
ENSG00000114480	GBE1	3	81675081	T	A	0.331014	-0.00088426	0.0149011	0.95	-0.155264	0.00824703	4.57E-79	0.00569522	0.0959734	0.95268	0.1685141
ENSG00000114491	UMPS	3	124456626	A	G	0.426441	0.00479136	0.0143914	0.7099994	-0.200263	0.00801995	1.28E-137	-0.0239254	0.0718689	0.7392078	0.1712177
ENSG00000114503	NCBP2	3	196665870	T	A	0.532803	0.0154748	0.0141829	0.3900004	-0.0855992	0.00874033	1.20E-22	-0.180782	0.166715	0.2781965	0.4649817
ENSG00000114520	SNX4	3	125202268	C	A	0.109344	0.0112239	0.0233513	0.5999997	0.180686	0.0123754	2.79E-48	0.0621182	0.129307	0.6309473	0.3153229
ENSG00000114529	C3orf52	3	111827516	A	G	0.188867	-0.0140311	0.0178625	0.2700001	-0.124894	0.0101543	9.10E-35	0.112344	0.143312	0.4330936	0.9301463
ENSG00000114541	FRMD4B	3	69405437	A	G	0.365805	-0.00543372	0.0151083	0.4199997	0.435914	0.00884375	0	-0.0124651	0.0346598	0.7191151	0.2318428
ENSG00000114544	SLC41A3	3	125772801	C	A	0.341948	0.00299744	0.0148344	0.8600001	0.641296	0.00761593	0	0.00467403	0.023132	0.8398702	0.6084209
ENSG00000114554	PLXNA1	3	126731836	T	C	0.120278	0.0188985	0.0231078	0.4400003	-0.0988801	0.0124299	1.79E-15	-0.191125	0.234927	0.4159013	0.3776646
ENSG00000114573	ATP6V1A	3	113498384	T	C	0.191849	-0.0299533	0.0180185	0.07299952	-0.254121	0.0164727	1.08E-53	0.11787	0.0713156	0.09837251	0.5485108
ENSG00000114626	ABTB1	3	127395773	C	G	0.223658	0.0112113	0.0176955	0.4199997	0.513084	0.00944742	0	0.0218508	0.0344909	0.526391	0.4344586
ENSG00000114631	PODXL2	3	127369838	T	C	0.215706	0.0124095	0.0178371	0.3900004	0.242129	0.00988231	1.43E-132	0.0512517	0.0736976	0.4867843	0.3315182
ENSG00000114648	KLHL18	3	47356356	T	C	0.45328	0.0298943	0.0142472	0.05999983	0.0899242	0.00803914	4.79E-29	0.332439	0.161199	0.03918027	0.8264058
ENSG00000114650	SCAP	3	47486909	A	G	0.395626	0.0198043	0.0144968	0.2599998	-0.307795	0.00795586	0	-0.0643424	0.0471282	0.17217	0.8443138
ENSG00000114656	CFAP92	3	128675121	C	T	0.184891	-0.0331319	0.0183265	0.05099998	0.0833442	0.0127332	5.93E-11	-0.397531	0.228123	0.08140023	0.5328012
ENSG00000114670	NEK11	3	130907501	C	T	0.203777	-0.00118368	0.0175661	0.83	0.349988	0.0100408	3.39E-266	-0.00338206	0.0501907	0.9462758	0.7787156
ENSG00000114735	HEMK1	3	50614474	A	G	0.124254	-0.00374363	0.0214247	0.7499995	-0.215387	0.0123763	7.80E-68	0.017381	0.0994758	0.8612953	0.5715317
ENSG00000114738	MAPKAPK3	3	50667835	A	G	0.10835	0.0124264	0.022097	0.4299995	-0.520196	0.0118828	0	-0.0238879	0.0424817	0.5739045	0.417521
ENSG00000114739	ACVR2B	3	38510145	T	G	0.323062	0.00848802	0.0150999	0.5099998	0.411281	0.012214	1.45E-248	0.020638	0.0367194	0.5740847	0.2598301
ENSG00000114742	WDR48	3	39115822	T	C	0.276342	-0.0146374	0.0160603	0.25	0.712719	0.00876908	0	-0.0205374	0.0225352	0.3621134	0.1954507
ENSG00000114744	COMMD2	3	149463271	C	T	0.0516899	-0.0237303	0.0298293	0.3100002	-0.874146	0.0159673	0	0.0271468	0.0341275	0.4263503	0.03373506
ENSG00000114745	GORASP1	3	39144002	A	G	0.027833	0.0976324	0.0531891	0.07299952	-0.381353	0.0280093	3.25E-42	-0.256016	0.140737	0.06889403	0.387948
ENSG00000114770	ABCC5	3	183686762	A	G	0.497018	-0.0052407	0.0141949	0.5500004	-0.723481	0.00682673	0	0.00724373	0.0196204	0.7119833	0.9413849
ENSG00000114779	ABHD14B	3	52009975	T	C	0.0447316	0.00196853	0.0372495	0.9699999	-0.414956	0.0197088	2.08E-98	-0.00474395	0.0897676	0.9578538	0.8622864
ENSG00000114784	EIF1B	3	40352545	A	G	0.314115	0.00609407	0.0151963	0.8600001	-0.0919191	0.0125418	2.32E-13	-0.0662982	0.16557	0.688844	0.5667086
ENSG00000114796	KLHL24	3	183376044	C	T	0.265408	-0.00329089	0.0161598	0.7899998	-0.491666	0.0161883	1.30E-202	0.00669335	0.0328682	0.8386332	0.6068967
ENSG00000114805	PLCH1	3	155278112	C	T	0.342942	-0.0104782	0.0149788	0.4	0.128801	0.00949075	5.93E-42	-0.0813519	0.116449	0.4847971	0.7404762
ENSG00000114812	VIPR1	3	42554925	C	T	0.345924	0.00389504	0.0146946	0.7499995	-0.407069	0.00796127	0	-0.00956849	0.036099	0.7909613	0.5517998
ENSG00000114841	DNAH1	3	52392421	G	A	0.104374	-0.0431152	0.0210152	0.08799946	-0.248833	0.0128772	3.40E-83	0.17327	0.0849297	0.04133506	0.295313
ENSG00000114850	SSR3	3	156265451	T	C	0.243539	-0.0275512	0.0169981	0.07499978	0.126479	0.0094379	5.95E-41	-0.217832	0.135374	0.1075915	0.2560096
ENSG00000114853	ZBTB47	3	42702124	G	T	0.140159	-0.0448055	0.0210306	0.032	-0.343219	0.0124807	1.76E-166	0.130545	0.0614583	0.03365962	0.7503468
ENSG00000114854	TNNC1	3	52486602	T	G	0.0576541	0.0219085	0.0324096	0.3900004	0.255418	0.0186725	1.36E-42	0.0857752	0.127043	0.4995716	0.4115692
ENSG00000114857	NKTR	3	42666166	G	A	0.207753	-0.000668143	0.0174716	0.8800001	-0.365941	0.00920594	0	0.00182582	0.0477444	0.969495	0.0847549
ENSG00000114859	CLCN2	3	184071706	T	C	0.2833	0.0174407	0.0157815	0.2399999	-0.18508	0.0138983	1.85E-40	-0.0942331	0.0855615	0.2707445	0.1753121

ENSG00000114867	EIF4G1	3	184042714	A	T	0.0208748	-0.152557	0.0464891	0.000940005	0.158374	0.0266856	2.94E-09	-0.963272	0.335425	0.004081517	0.7142716
ENSG00000114902	SPCS1	3	52740576	A	G	0.429423	-0.00333543	0.0144193	0.5999997	0.195031	0.00875095	4.96E-110	-0.0171021	0.0739375	0.8170782	0.1005439
ENSG00000114904	NEK4	3	52774882	A	C	0.513917	0.00869797	0.0142065	0.7400005	-0.0630488	0.00803333	4.21E-15	-0.137956	0.22601	0.541598	0.2227297
ENSG00000114942	EEF1B2	2	207025980	C	A	0.0178926	-0.0197649	0.0661077	0.6499995	0.192501	0.0299864	1.37E-10	-0.102674	0.343788	0.7652021	0.9261505
ENSG00000114948	ADAM23	2	207397057	G	A	0.199801	0.00384875	0.018198	0.8200001	0.126938	0.00982053	3.22E-38	0.0303199	0.14338	0.8325247	0.7975845
ENSG00000114956	DGUOK	2	74170020	A	T	0.390656	-0.00805062	0.0145366	0.64	-0.214435	0.00880942	7.12E-131	0.0375433	0.0678077	0.579802	0.9052213
ENSG00000114978	MOB1A	2	74394095	G	A	0.100398	-0.0160752	0.0232367	0.32	0.148516	0.015656	2.40E-21	-0.108239	0.156875	0.4902131	0.9631512
ENSG00000114982	KANSL3	2	97283715	G	C	0.340954	0.00365542	0.0145907	0.9199999	0.0831252	0.0101947	3.53E-16	0.0439749	0.17561	0.8022683	0.01858436
ENSG00000114988	LMAN2L	2	97388733	A	G	0.311133	-0.0302711	0.0151386	0.02300011	0.168791	0.00843289	4.01E-89	-0.179341	0.0901349	0.04662477	0.7480563
ENSG00000114993	RTKN	2	74661256	T	G	0.176938	-0.0186899	0.0184374	0.33	0.0673467	0.0107841	4.24E-10	-0.277518	0.277351	0.3170208	0.7010592
ENSG00000114999	TTL	2	113264979	T	C	0.255467	-0.0289416	0.0171261	0.2300001	-0.128009	0.00930418	4.55E-43	0.226091	0.134794	0.09348258	0.7227329
ENSG00000115020	PIKFYVE	2	209177233	G	A	0.441352	0.0138636	0.0144447	0.4500005	0.0469509	0.00808188	6.27E-09	0.295279	0.311826	0.343672	0.3897299
ENSG00000115053	NCL	2	232333297	G	C	0.102386	-0.00365818	0.023249	0.9199999	-0.433408	0.0129583	2.91E-245	0.00844049	0.0536428	0.8749721	0.3406228
ENSG00000115073	ACTR1B	2	98276500	A	G	0.083499	-0.0397771	0.0268396	0.06699926	0.457315	0.0132274	6.37E-262	-0.0869797	0.0587435	0.1386944	0.258654
ENSG00000115084	SLC35F5	2	114488494	T	A	0.45825	0.0142245	0.0142187	0.2700001	0.205217	0.0079125	2.63E-148	0.0693145	0.0693377	0.3174729	0.122306
ENSG00000115085	ZAP70	2	98343174	G	A	0.487078	0.0245321	0.0142436	0.05499966	-0.223141	0.00785857	2.36E-177	-0.10994	0.0639496	0.08558439	0.8738088
ENSG00000115091	ACTR3	2	114683855	T	C	0.083499	-0.00607394	0.0223829	0.83	0.11365	0.0183449	5.82E-10	-0.053444	0.197134	0.7863101	0.916678
ENSG00000115107	STEAP3	2	120002306	T	C	0.464215	0.00576606	0.014261	0.6499995	-0.530184	0.00798705	0	-0.0108756	0.0268987	0.6859802	0.6134204
ENSG00000115109	EPB41L5	2	120853638	A	C	0.214712	0.0210566	0.017341	0.2099999	-0.150319	0.00943494	3.79E-57	-0.140079	0.115696	0.2259892	0.5962497
ENSG00000115112	TFCP2L1	2	122008473	C	T	0.378728	-0.000631702	0.0147976	0.95	0.182578	0.0088121	2.33E-95	-0.0034599	0.0810483	0.9659491	0.20193
ENSG00000115129	TP53I3	2	24304517	A	G	0.156064	0.0108243	0.0194004	0.6100002	-0.613375	0.00996134	0	-0.0176471	0.0316302	0.5768996	0.8367272
ENSG00000115137	DNAJC27	2	25180734	A	G	0.110338	0.0164779	0.0217536	0.2599998	-0.28779	0.0119562	5.11E-128	-0.0572568	0.075626	0.4489873	0.9520368
ENSG00000115138	POMC	2	25387747	C	A	0.0785288	0.0145347	0.0278557	0.6300007	0.582229	0.0157125	1.48E-300	0.0249639	0.0478479	0.6018557	0.02857601
ENSG00000115145	STAM2	2	153002910	T	A	0.181909	-0.010378	0.0180141	0.5	0.295586	0.0105589	1.92E-172	-0.03511	0.0609567	0.5646271	0.353651
ENSG00000115155	OTOF	2	26730818	T	C	0.219682	-0.000970572	0.017115	0.95	-0.227824	0.00965845	5.12E-123	0.00426019	0.0751241	0.9547773	0.326168
ENSG00000115159	GPD2	2	157381024	G	A	0.457256	-0.00679983	0.0143414	0.6100002	0.389822	0.00779097	0	-0.0174434	0.0367913	0.6354153	0.8387139
ENSG00000115165	CYTIP	2	158308302	T	C	0.00596421	-0.114405	0.0804074	0.1199999	1.16734	0.0607653	3.02E-82	-0.098005	0.0690696	0.1559193	0.7572408
ENSG00000115170	ACVR1	2	158662666	T	C	0.0755467	0.00759247	0.0268635	0.6300007	-0.309597	0.0149719	5.40E-95	-0.0245237	0.0867773	0.7774796	0.2540683
ENSG00000115211	EIF2B4	2	27590286	C	A	0.374751	0.00935827	0.0145345	0.58	0.0924255	0.00888672	2.47E-25	0.101252	0.157558	0.5204605	0.01861536
ENSG00000115216	NRBP1	2	27657891	T	C	0.411531	0.0140775	0.0142876	0.3400001	-0.238246	0.00788786	2.09E-200	-0.059088	0.0600018	0.3247365	0.09117096
ENSG00000115232	ITGA4	2	182361421	G	A	0.462227	0.0191313	0.0143421	0.1800002	0.481623	0.00824985	0	0.0397226	0.0297864	0.1823422	0.6100398
ENSG00000115234	SNX17	2	27596692	G	A	0.374751	0.00971859	0.014541	0.5700002	-0.2287	0.00957804	5.23E-126	-0.0424948	0.0636059	0.5040723	0.02246206
ENSG00000115239	ASB3	2	53923553	G	A	0.206759	0.0150098	0.0176727	0.29	-0.152407	0.00993045	3.69E-53	-0.0984852	0.116135	0.3964248	0.4233873
ENSG00000115241	PPM1G	2	27618307	T	C	0.365805	0.00932499	0.0145644	0.56	-0.143413	0.0081286	1.15E-69	-0.0650219	0.101623	0.522278	0.0332439
ENSG00000115255	REEP6	19	1494545	T	C	0.055666	-0.0228929	0.0305825	0.32	-0.421565	0.0239967	4.36E-69	0.0543045	0.0726109	0.4545305	0.7201999
ENSG00000115257	PCSK4	19	1486089	A	G	0.370775	-0.00678086	0.0149608	0.5400003	0.300932	0.0102596	4.09E-189	-0.0225328	0.0497208	0.6504136	0.6500203
ENSG00000115266	APC2	19	1459771	T	C	0.00497018	-0.0690363	0.0955712	0.4199997	-1.30063	0.148528	2.01E-18	0.0530791	0.0737303	0.4715808	0.2032548
ENSG00000115267	IFIH1	2	163149401	T	C	0.0119284	0.0107647	0.0488877	0.6999999	-0.440444	0.060919	4.83E-13	-0.0244406	0.111048	0.8258008	0.9494358
ENSG00000115268	RPS15	19	1439712	G	A	0.0477137	0.0307283	0.0350102	0.4299995	0.162567	0.0257178	2.60E-10	0.189019	0.217424	0.3846528	0.3135543
ENSG00000115271	GCA	2	163201727	T	C	0.00994036	0.00968577	0.0717366	0.95	-0.362595	0.0385821	5.56E-21	-0.0267124	0.197863	0.8926082	0.9894707
ENSG00000115274	INO80B	2	74685080	A	G	0.159046	-0.01363	0.0203352	0.4799997	-0.348682	0.0117286	3.23E-194	0.03909	0.058335	0.5027966	0.6337569
ENSG00000115275	MOGS	2	74690360	T	C	0.153082	-0.000715492	0.0209255	1	0.142326	0.0116287	1.92E-34	-0.00502712	0.147025	0.9727239	0.1315049
ENSG00000115282	TTC31	2	74716106	T	C	0.154076	-0.00379899	0.0209246	0.9	0.166704	0.0116504	1.93E-46	-0.0227889	0.12553	0.8559425	0.2859022
ENSG00000115286	NDUFS7	19	1389554	C	G	0.161034	-0.00293243	0.018635	0.7600007	0.0704102	0.0125432	1.98E-08	-0.0416478	0.264767	0.8750087	0.2179759
ENSG00000115290	GRB14	2	165413840	G	A	0.0924453	0.014117	0.0243394	0.58	-0.34797	0.0139385	1.48E-137	-0.0405696	0.0699657	0.562016	0.1225996
ENSG00000115295	CLIP4	2	29366540	T	C	0.491054	0.0119328	0.0142779	0.3900004	0.172805	0.00789171	2.77E-106	0.0690537	0.0826846	0.403636	0.06329606
ENSG00000115306	SPTBN1	2	54790117	A	G	0.248509	0.00657077	0.016123	0.5999997	0.363478	0.00912913	0	0.0180775	0.0443599	0.683627	0.2208121
ENSG00000115307	AUP1	2	74755419	T	C	0.158052	-0.00785536	0.0204424	0.7400005	0.172226	0.0114761	6.57E-51	-0.0456108	0.118734	0.7008731	0.1729502
ENSG00000115310	RTN4	2	55269541	T	C	0.409543	0.0168916	0.0143065	0.1900002	0.291785	0.00791152	9.32E-298	0.0578906	0.0490561	0.2379646	0.4060919
ENSG00000115317	HTRA2	2	74758488	T	G	0.027833	-0.0617592	0.0443924	0.16	-0.296583	0.0291436	2.52E-24	0.208236	0.151072	0.1680827	0.2320955

ENSG00000115318	LOXL3	2	74771179	C	T	0.0159046	0.0770629	0.0472367	0.07299952	-0.624928	0.0325687	4.66E-82	-0.123315	0.0758601	0.1040443	0.0807366
ENSG00000115325	DOK1	2	74780417	T	A	0.140159	0.00126201	0.0215432	0.95	-0.101772	0.0118629	9.57E-18	-0.0124004	0.211687	0.9532874	0.02877601
ENSG00000115339	GALNT3	2	166627646	G	A	0.117296	-0.00155968	0.0213036	0.84	0.130063	0.0179288	4.03E-13	-0.0119917	0.163803	0.9416404	0.8412637
ENSG00000115350	POLE4	2	75191437	C	G	0.252485	0.00955805	0.0157927	0.5400003	-0.111815	0.00932199	3.78E-33	-0.0854806	0.141419	0.5455441	0.8551372
ENSG00000115355	CCDC88A	2	55581017	T	C	0.0924453	0.00195495	0.0231923	0.84	0.307892	0.0135577	3.58E-114	0.00634946	0.0753265	0.9328239	0.1894399
ENSG00000115364	MRPL19	2	75895943	A	C	0.242545	-0.0109722	0.0163536	0.4799997	-0.324255	0.00892133	3.04E-289	0.0338382	0.050443	0.5023349	0.008342706
ENSG00000115365	LANCL1	2	211319174	A	G	0.451292	0.0072845	0.0142052	0.6200004	0.184164	0.00792336	1.67E-119	0.0395544	0.0771521	0.6081749	0.01727389
ENSG00000115368	WDR75	2	190323225	G	A	0.0367793	-0.00692587	0.0381232	0.91	-0.489364	0.0211151	7.95E-119	0.0141528	0.077906	0.8558455	0.6089481
ENSG00000115392	FANCL	2	58427442	T	G	0.0129225	0.0630145	0.061838	0.35	0.421297	0.0317855	4.26E-40	0.149572	0.147213	0.3096165	0.4650827
ENSG00000115414	FN1	2	216263029	C	T	0.375746	-0.00477187	0.0145635	0.7499995	0.14333	0.00810289	5.12E-70	-0.0332928	0.101625	0.7432116	0.535974
ENSG00000115415	STAT1	2	191857385	G	C	0.342942	-0.0283766	0.0152506	0.0990011	-0.0668097	0.00873466	2.03E-14	0.424738	0.234927	0.07061263	0.7968903
ENSG00000115419	GLS	2	191787915	G	C	0.428429	0.0124566	0.0145193	0.4	0.224792	0.0080654	5.98E-171	0.055414	0.0646207	0.3911533	0.037813
ENSG00000115423	DNAH6	2	84895146	G	A	0.394632	-0.0142712	0.0144067	0.32	-0.224161	0.00805117	1.34E-170	0.0636648	0.06431	0.3221896	0.5533336
ENSG00000115425	PECR	2	216904365	C	A	0.235586	0.00161313	0.0159331	0.98	-0.147691	0.00918502	3.55E-58	-0.0109223	0.107883	0.9193584	0.9617524
ENSG00000115446	UNC50	2	99230010	G	C	0.270378	0.00153152	0.0163068	0.99	-0.255505	0.00921143	2.45E-169	-0.00599409	0.0638222	0.9251738	0.5173445
ENSG00000115457	IGFBP2	2	217513355	C	T	0.32505	-0.0103769	0.0149361	0.3900004	0.357667	0.0086188	0	-0.0290127	0.0417656	0.4872711	0.3001591
ENSG00000115459	ELMOD3	2	85600196	T	G	0.384692	-0.00563019	0.0145634	0.7099994	-0.0943286	0.00817845	8.91E-31	0.059687	0.154477	0.6992141	0.9818604
ENSG00000115464	USP34	2	61556251	A	G	0.285288	0.0159941	0.0158071	0.1900002	0.157077	0.00890955	1.44E-69	0.101823	0.100798	0.3124149	0.526796
ENSG00000115468	EFHD1	2	233509129	A	C	0.0218688	0.00664041	0.0443061	0.7899998	0.518301	0.0348808	6.06E-50	0.0128119	0.0854876	0.8808687	0.1073504
ENSG00000115484	CCT4	2	62105581	A	G	0.171968	0.0118929	0.0200896	0.64	0.27856	0.0111236	2.12E-138	0.0426942	0.0721396	0.5539661	0.7187602
ENSG00000115486	GGCX	2	85781706	A	G	0.44334	-0.0278894	0.0143736	0.06800017	0.300561	0.00787632	0	-0.0927911	0.0478844	0.05264546	0.07495309
ENSG00000115504	EHBP1	2	63087304	T	C	0.128231	0.0296274	0.0226834	0.2200002	0.0925528	0.0125495	1.64E-13	0.320113	0.2489	0.1984036	0.954109
ENSG00000115507	OTX1	2	63281081	T	A	0.10338	-0.0163259	0.020851	0.4500005	0.5167	0.0114404	0	-0.0315965	0.0403602	0.4337084	0.2128126
ENSG00000115520	COQ10B	2	198329089	C	T	0.212724	0.0276066	0.0180869	0.16	-0.10404	0.0112783	2.84E-20	-0.265345	0.176209	0.1321037	0.176421
ENSG00000115523	GNLY	2	85919137	T	C	0.236581	-0.0021918	0.0169461	0.89	0.868951	0.00817998	0	-0.00252235	0.0195018	0.8970891	0.08554911
ENSG00000115524	SF3B1	2	198278256	T	A	0.27833	0.00911533	0.0150741	0.3599996	0.130373	0.00837722	1.30E-54	0.0699173	0.11571	0.5456804	0.399651
ENSG00000115525	ST3GAL5	2	86091202	A	G	0.193837	-0.0199721	0.0178643	0.2399999	-0.418504	0.00919316	0	0.0477226	0.042699	0.2637154	0.1191346
ENSG00000115526	CHST10	2	101021222	T	G	0.291252	0.0125384	0.0152087	0.2999998	0.110473	0.00853588	2.60E-38	0.113497	0.137948	0.4106472	0.9776612
ENSG00000115539	PDCL3	2	101186174	T	C	0.204771	0.00466359	0.0171859	0.7099994	0.163794	0.0106873	5.13E-53	0.0284723	0.10494	0.7861458	0.6392824
ENSG00000115540	MOB4	2	198399359	T	C	0.0457256	0.00877049	0.0395415	0.8200001	0.20758	0.0299391	4.11E-12	0.0422511	0.190585	0.8245542	0.3178266
ENSG00000115541	HSPE1	2	198366449	T	C	0.27336	0.00951307	0.0151342	0.35	0.134256	0.0139655	7.02E-22	0.0708579	0.112968	0.5305009	0.111385
ENSG00000115548	KDM3A	2	86693804	C	T	0.333002	-0.00591443	0.015291	0.81	0.104152	0.00876608	1.48E-32	-0.0567868	0.146893	0.6990622	0.7378838
ENSG00000115561	CHMP3	2	86839399	C	A	0.400596	-0.00288103	0.0146514	0.95	-0.213336	0.00916312	6.75E-120	0.0135047	0.0686801	0.8441159	0.6740144
ENSG00000115568	ZNF142	2	219513508	G	A	0.0258449	0.0510403	0.039651	0.2200002	0.632142	0.0226492	2.01E-171	0.0807418	0.0627915	0.1984878	0.9739048
ENSG00000115590	IL1R2	2	102626656	A	G	0.292247	-0.0220448	0.0161567	0.1100001	0.306007	0.0085661	1.74E-279	-0.0720402	0.052837	0.172743	0.6822462
ENSG00000115592	PRKAG3	2	219691957	T	G	0.467197	0.0173796	0.0141961	0.1499999	0.127365	0.00825151	9.46E-54	0.136455	0.11181	0.2223061	0.3391263
ENSG00000115594	IL1R1	2	102738669	T	C	0.200795	0.0135677	0.0192632	0.5400003	0.16004	0.0103141	2.68E-54	0.0847769	0.120489	0.4816769	0.455822
ENSG00000115602	IL1RL1	2	102948229	G	A	0.220676	-0.00498536	0.0169839	0.4600002	-0.236888	0.00911948	9.25E-149	0.0210452	0.0717006	0.769128	0.06261652
ENSG00000115604	IL18R1	2	102971603	T	C	0.387674	0.0246307	0.0146554	0.1800002	-0.314116	0.0080124	0	-0.0784129	0.0466989	0.09312969	0.1056765
ENSG00000115607	IL18RAP	2	103052087	T	C	0.528827	0.0285601	0.0141997	0.02399993	0.787025	0.00663576	0	0.0362887	0.0180449	0.0443225	0.2407446
ENSG00000115641	FHL2	2	106014564	G	A	0.0725646	-0.0033926	0.0280876	0.6499995	0.637815	0.0153831	0	-0.0053191	0.0440374	0.9038606	0.6595149
ENSG00000115649	CNPPD1	2	220039723	A	G	0.383698	-0.000369845	0.014507	0.9599999	-0.183583	0.00904457	1.35E-91	0.00201459	0.0790215	0.9796608	0.105426
ENSG00000115652	UXS1	2	106760277	A	G	0.0924453	0.00375518	0.0265404	0.9299999	0.154732	0.0136571	9.34E-30	0.024269	0.171539	0.8874922	0.4042322
ENSG00000115677	HDLBP	2	242211577	T	A	0.192843	0.00218601	0.0182508	0.89	-0.184472	0.0099731	2.18E-76	-0.0118501	0.0989372	0.9046625	0.906318
ENSG00000115685	PPP1R7	2	242106029	G	A	0.21173	0.0241301	0.0167257	0.09400046	0.156579	0.0145701	6.15E-27	0.154108	0.107778	0.1527547	0.07409834
ENSG00000115687	PASK	2	242067596	G	A	0.167992	-0.00015107	0.0194532	0.89	-0.950814	0.0140358	0	0.000158885	0.0204595	0.9938038	0.02222166
ENSG00000115694	STK25	2	242440617	G	A	0.228628	0.00930015	0.0172533	0.7300002	-0.114978	0.00960387	4.98E-33	-0.0808867	0.15021	0.5902385	0.8057896
ENSG00000115705	TPO	2	1462739	C	A	0.402584	-0.00263117	0.0144734	0.98	0.103932	0.00815461	3.32E-37	-0.0253162	0.139272	0.8557593	0.6139335
ENSG00000115718	PROC	2	128181412	T	C	0.321074	-0.0128282	0.0153514	0.5300002	-0.175659	0.00850087	7.37E-95	0.0730291	0.0874648	0.4037432	0.6331123
ENSG00000115738	ID2	2	8821779	T	A	0.479125	-0.00224028	0.0142554	1	0.118168	0.00831077	7.02E-46	-0.0189585	0.120645	0.8751318	0.3962699

ENSG00000115750	TAF1B	2	10029014	T	C	0.478131	-0.0143066	0.0142928	0.2200002	-0.510665	0.00756204	0	0.0280156	0.0279917	0.3168966	0.2552299
ENSG00000115756	HPCAL1	2	10505379	T	C	0.282306	-0.00266493	0.0157686	0.9	0.37877	0.00985516	0	-0.00703574	0.0416315	0.8657961	0.3874537
ENSG00000115758	ODC1	2	10584362	G	A	0.358847	-0.00823152	0.0150796	0.7700005	-0.11749	0.00889524	7.87E-40	0.0700614	0.128457	0.5854745	0.2963883
ENSG00000115760	BIRC6	2	32713031	A	G	0.11829	0.0127973	0.0223349	0.5199996	0.140765	0.0139482	5.99E-24	0.0909126	0.158924	0.5672867	0.9002466
ENSG00000115761	NOL10	2	10770496	G	A	0.32008	-0.0184641	0.0150498	0.3100002	0.0749107	0.00848519	1.06E-18	-0.246482	0.202834	0.224294	0.825405
ENSG00000115762	PLEKHB2	2	131986851	C	T	0.109344	0.0142182	0.0212438	0.4	0.254428	0.0118074	5.50E-103	0.055883	0.0835366	0.5035182	0.7259617
ENSG00000115806	GORASP2	2	171804306	G	T	0.282306	0.0029253	0.0157311	0.9	0.098311	0.00890321	2.39E-28	0.0297556	0.160036	0.8524996	0.1765427
ENSG00000115808	STRN	2	37132199	C	T	0.255467	0.000694815	0.0164531	0.6700003	0.134287	0.00906539	1.20E-49	0.00517409	0.122522	0.9663155	0.9572507
ENSG00000115816	CEBPZ	2	37443805	A	G	0.423459	-0.024535	0.0145647	0.0259998	-0.407864	0.00780434	0	0.0601548	0.0357282	0.09224393	0.3088062
ENSG00000115825	PRKD3	2	37514798	G	C	0.311133	0.0190352	0.0146256	0.1299999	0.323878	0.00808543	0	0.0587727	0.0451816	0.1933227	0.3490656
ENSG00000115827	DCAF17	2	172316144	C	T	0.227634	0.0169026	0.0170798	0.2599998	0.102672	0.00941735	1.12E-27	0.164628	0.167037	0.3243426	0.5347301
ENSG00000115828	QPCT	2	37586091	G	A	0.353877	0.0181577	0.0145098	0.16	0.155743	0.00807947	8.48E-83	0.116587	0.093361	0.2117453	0.002161372
ENSG00000115839	RAB3GAP1	2	135871899	T	C	0.204771	0.0033693	0.0229879	0.35	-0.290333	0.0127948	5.45E-114	-0.011605	0.0791794	0.8834751	0.7849189
ENSG00000115840	SLC25A12	2	172752823	C	T	0.276342	-0.00502316	0.0158113	0.6600001	0.203034	0.0092996	1.14E-105	-0.0247404	0.0778832	0.7507423	0.962468
ENSG00000115841	RMDN2	2	38222307	G	A	0.247515	-0.0148052	0.0166665	0.4299995	0.123029	0.01031	7.96E-33	-0.120339	0.135843	0.3756878	0.5767504
ENSG00000115866	DARS1	2	136703958	A	G	0.210736	-0.0203306	0.0220492	0.03799969	0.647552	0.0105252	0	-0.0313961	0.0340539	0.3565533	0.4234765
ENSG00000115875	SRSF7	2	38974688	C	G	0.286282	-0.00030212	0.0153856	0.7400005	0.0485041	0.00868564	2.35E-08	-0.00622875	0.317204	0.9843334	0.3228037
ENSG00000115896	PLCL1	2	199053365	A	C	0.449304	0.0136246	0.0142211	0.2999998	0.482041	0.00747925	0	0.0282644	0.0295051	0.3380882	0.03490492
ENSG00000115902	SLC1A4	2	65233305	A	G	0.143141	-0.0238803	0.0212906	0.25	0.518008	0.0117815	0	-0.0461002	0.0411143	0.2621725	0.6070073
ENSG00000115904	SOS1	2	39280011	G	C	0.0775348	0.00946035	0.0271671	0.6499995	0.472709	0.0144248	1.55E-235	0.0200131	0.0574743	0.7276836	0.4626955
ENSG00000115919	KYNU	2	143717478	G	A	0.0636183	-0.00605795	0.0331085	0.9299999	0.910062	0.0180429	0	-0.00665664	0.0363807	0.8548204	0.8276702
ENSG00000115935	WIPF1	2	175485972	C	T	0.0536779	-0.038947	0.0305544	0.17	-0.261168	0.0169175	9.12E-54	0.149126	0.117389	0.2039585	0.7869607
ENSG00000115942	ORC2	2	201801049	G	A	0.0218688	-0.0205831	0.0405399	0.5500004	0.190877	0.0245849	8.23E-15	-0.107834	0.212841	0.6124058	0.5275537
ENSG00000115944	COX7A2L	2	42578418	A	G	0.410537	-0.00275787	0.0143453	0.7800007	0.36114	0.00860332	0	-0.00763657	0.0397227	0.8475487	0.9632097
ENSG00000115946	PNO1	2	68394173	C	G	0.217694	-0.0218869	0.0160746	0.1499999	-0.205051	0.0101456	7.87E-91	0.106739	0.078571	0.174304	0.7367177
ENSG00000115947	ORC4	2	148735439	C	G	0.54175	-0.0236188	0.0141946	0.0530005	0.108211	0.0093492	5.56E-31	-0.218265	0.132523	0.09955876	0.2065148
ENSG00000115956	PLEK	2	68608445	C	T	0.27833	-0.00594654	0.016195	0.8200001	0.49367	0.00851336	0	-0.0120456	0.032806	0.7134875	0.2763643
ENSG00000115970	THADA	2	43608492	A	G	0.0149105	0.0566305	0.0506096	0.3599996	-0.266349	0.0284136	6.98E-21	-0.212618	0.191361	0.2665341	0.7633088
ENSG00000115993	TRAK2	2	202279116	T	C	0.496024	0.021715	0.0143364	0.14	-0.0515651	0.00803971	1.42E-10	-0.421118	0.285673	0.1404475	0.5613818
ENSG00000115998	C2orf42	2	70426379	G	T	0.0695825	-0.0188452	0.0284839	0.5	-0.537784	0.0158264	4.35E-253	0.0350423	0.0529753	0.5083023	0.3546989
ENSG00000116001	TIA1	2	70456184	C	T	0.0685885	-0.0152561	0.0285564	0.59	0.393679	0.0159485	1.57E-134	-0.0387527	0.0725543	0.5932589	0.824804
ENSG00000116005	PCYOX1	2	70496420	T	C	0.215706	0.00545674	0.0166579	0.5199996	-0.160609	0.00964883	3.27E-62	-0.0339754	0.103737	0.7432797	0.8349901
ENSG00000116014	KISS1R	19	919151	A	G	0.483101	-0.020292	0.0142358	0.1199999	-0.32917	0.00965293	7.25E-255	0.061646	0.0432854	0.1543953	0.2407424
ENSG00000116016	EPAS1	2	46567321	C	G	0.479125	0.0082888	0.0142274	0.4500005	0.227698	0.00791474	5.26E-182	0.0364025	0.0624963	0.560247	0.3371049
ENSG00000116017	ARID3A	19	950860	G	A	0.208748	0.00692754	0.0179554	0.4899999	0.0877676	0.0132615	3.64E-11	0.0789305	0.204926	0.7001149	0.6907242
ENSG00000116031	CD207	2	71060149	A	G	0.317097	-0.0245343	0.0148937	0.14	0.105481	0.00839025	3.02E-36	-0.232595	0.142405	0.1023992	0.8528591
ENSG00000116032	GRIN3B	19	1005074	T	C	0.0874751	0.0184823	0.0256653	0.4799997	-0.288052	0.0214194	3.15E-41	-0.064163	0.0892271	0.4720807	0.9451831
ENSG00000116044	NFE2L2	2	178174874	T	C	0.267396	-0.0186449	0.0163135	0.2099999	0.0653408	0.00939533	3.54E-12	-0.285349	0.253017	0.2594109	0.9616627
ENSG00000116062	MSH6	2	48022156	C	T	0.17992	-0.026509	0.018004	0.1900002	0.508708	0.0151357	1.21E-247	-0.0521104	0.0354255	0.1412954	0.9830812
ENSG00000116095	PLEKHA3	2	179357644	T	C	0.298211	0.00372183	0.0153278	0.7600007	-0.231291	0.0140032	2.77E-61	-0.0160915	0.0662777	0.8081685	0.5369709
ENSG00000116106	EPHA4	2	222360834	T	G	0.463221	-0.00946432	0.0142363	0.6600001	-0.506765	0.00744088	0	0.018676	0.0280938	0.5061974	0.7276468
ENSG00000116117	PARD3B	2	205947701	T	C	0.147117	-0.0147116	0.0204557	0.4899999	0.274375	0.0118277	4.81E-119	-0.0536185	0.0745896	0.4722347	0.6784148
ENSG00000116120	FARSB	2	223478155	C	G	0.257455	0.0195998	0.015666	0.1299999	-0.145307	0.00962733	1.80E-51	-0.134885	0.108183	0.2124605	0.03958846
ENSG00000116127	ALMS1	2	73725403	A	G	0.280318	-0.00960728	0.0155428	0.4500005	0.434377	0.00840933	0	-0.0221174	0.0357844	0.5365269	0.8421029
ENSG00000116128	BCL9	1	147055599	C	T	0.0298211	-0.0352932	0.0364201	0.2700001	0.521811	0.0267417	8.51E-85	-0.067636	0.0698816	0.3331116	0.3876369
ENSG00000116133	DHCR24	1	55334098	C	T	0.412525	0.0182184	0.0143805	0.2300001	-0.240297	0.00798304	4.70E-199	-0.075816	0.0598976	0.2055984	0.9380225
ENSG00000116138	DNAJC16	1	15886091	G	A	0.298211	0.00594412	0.0152426	0.6999999	0.0798716	0.00857526	1.23E-20	0.0744209	0.191006	0.6968129	0.03968331
ENSG00000116151	MORN1	1	2287919	C	T	0.352883	-0.0301721	0.0147378	0.03400008	-0.159328	0.0087852	1.66E-73	0.18937	0.093087	0.04191743	0.4753476
ENSG00000116157	GPX7	1	53071383	G	A	0.418489	-0.0399632	0.0142364	0.006100002	0.612237	0.00719435	0	-0.0652741	0.0232657	0.005022483	0.03204933
ENSG00000116161	CACYBP	1	174974575	C	T	0.390656	0.0122063	0.014528	0.3800004	-0.0558677	0.00819848	9.47E-12	-0.218486	0.262012	0.4043503	0.622992

ENSG00000116171	SCP2	1	53455138	C	T	0.349901	0.029817	0.0148754	0.08300036	-0.721517	0.00745799	0	-0.0413254	0.0206213	0.0450679	0.3011415
ENSG00000116191	RALGPS2	1	178791769	T	C	0.106362	-0.0187868	0.0239135	0.33	-0.120751	0.0125982	9.26E-22	0.155583	0.198704	0.4336335	0.745936
ENSG00000116198	CEP104	1	3751211	T	C	0.429423	0.00813494	0.0142882	0.6700003	0.726915	0.00771562	0	0.011191	0.0196563	0.5691279	0.267908
ENSG00000116199	FAM20B	1	179020318	C	A	0.397614	-0.0154205	0.0145017	0.2300001	-0.184606	0.00806083	4.48E-116	0.083532	0.0786396	0.2881387	0.6683948
ENSG00000116205	TCEANC2	1	54548726	G	A	0.026839	0.100047	0.0461995	0.03599979	-0.361866	0.0257585	7.88E-45	-0.276475	0.129178	0.03233344	0.141675
ENSG00000116209	TMEM59	1	54508262	T	C	0.0119284	-0.0611043	0.0740555	0.3100002	-0.542873	0.0417248	1.06E-38	0.112557	0.136688	0.4102461	0.07848343
ENSG00000116212	LRRC42	1	54422795	A	G	0.145129	0.0144805	0.0199479	0.5500004	0.171824	0.0114072	2.84E-51	0.0842753	0.11623	0.4684063	0.2262361
ENSG00000116213	WRAP73	1	3558328	C	T	0.510934	0.0431454	0.0142004	0.00179999	-0.512752	0.0110488	0	-0.0841448	0.0277538	0.002430744	0.2339985
ENSG00000116221	MRPL37	1	54668158	G	C	0.125249	-0.0442672	0.0202942	0.02900013	-0.30053	0.012231	2.57E-133	0.147297	0.0677935	0.02980073	0.05457128
ENSG00000116237	ICMT	1	6288642	A	G	0.0387674	-0.040366	0.0358878	0.3400001	-0.885842	0.0235393	34906400000002e-3	0.045568	0.0405308	0.2608937	0.8088814
ENSG00000116251	RPL22	1	6255389	A	G	0.0367793	0.0978137	0.0344961	0.001	-0.231685	0.0229696	6.33E-24	-0.422183	0.154663	0.006339341	0.3585627
ENSG00000116254	CHD5	1	6201018	A	G	0.400596	-0.00684341	0.0142502	0.8499999	0.0702964	0.00936362	6.03E-14	-0.0973508	0.20313	0.6317586	0.3473045
ENSG00000116260	QSOX1	1	180148567	C	T	0.261431	0.0320293	0.0166307	0.05	0.498837	0.00879828	0	0.064208	0.0333582	0.05425389	0.3847613
ENSG00000116266	STXBP3	1	109320722	G	A	0.230616	-0.00812058	0.0160867	0.6700003	-0.183993	0.0134308	1.02E-42	0.0441352	0.0874902	0.6139392	0.2505214
ENSG00000116273	PHF13	1	6678919	T	C	0.354871	0.0181754	0.0150421	0.2300001	-0.181777	0.00849418	1.33E-101	-0.0999872	0.082882	0.2276708	0.7726394
ENSG00000116285	ERRFI1	1	8075416	A	G	0.407555	-0.00632462	0.0143784	0.7099994	0.0503458	0.00808093	4.66E-10	-0.125624	0.286304	0.6608229	0.2852353
ENSG00000116288	PARK7	1	8029958	T	G	0.16004	-0.027032	0.0188747	0.09200046	0.409378	0.0107042	0	-0.0660319	0.0461381	0.152379	0.2823385
ENSG00000116299	ELAPOR1	1	109702851	A	G	0.399602	-0.016635	0.0147528	0.2	-0.936657	0.00623621	0	0.01776	0.0157509	0.2595097	0.7734446
ENSG00000116350	SRSF4	1	29491377	G	C	0.292247	-0.0195643	0.0160873	0.16	0.0974312	0.00963216	4.73E-24	-0.200801	0.166304	0.2272636	0.5090312
ENSG00000116353	MECR	1	29538419	G	A	0.0795229	-0.0127528	0.0250435	0.6499995	-0.151175	0.0143365	5.37E-26	0.0843576	0.165852	0.6110094	0.2355861
ENSG00000116396	KCNC4	1	110789843	A	G	0.424453	-0.00122872	0.0146412	0.5400003	0.0482886	0.00849285	1.30E-08	-0.0254453	0.303235	0.9331256	0.3348667
ENSG00000116406	EDEM3	1	184691706	T	A	0.408549	0.0219639	0.0143624	0.089	-0.230291	0.00798946	1.06E-182	-0.0953747	0.0624541	0.1267321	0.0517352
ENSG00000116455	WDR77	1	111987255	G	A	0.291252	0.0191137	0.0150711	0.1299999	-0.125772	0.0124465	5.25E-24	-0.151971	0.120769	0.2082604	0.1895024
ENSG00000116473	RAP1A	1	112172076	A	G	0.16004	-0.00772787	0.0189249	0.6100002	-0.182787	0.0104926	5.76E-68	0.042278	0.103564	0.6831034	0.2972061
ENSG00000116478	HDAC1	1	32778461	C	T	0.0248509	0.0609566	0.0416239	0.1499999	0.347874	0.0440681	2.93E-15	0.175226	0.121694	0.1498976	0.6868782
ENSG00000116489	CAPZA1	1	113188018	A	G	0.244533	-0.0111045	0.016239	0.3900004	0.0965549	0.0135148	9.04E-13	-0.115007	0.168953	0.4960576	0.3798312
ENSG00000116497	S100PBP	1	33303422	T	C	0.299205	0.0286028	0.0159227	0.0990011	-0.349018	0.00835787	0	-0.0819522	0.0456636	0.07270248	0.1920518
ENSG00000116514	RNF19B	1	33416166	T	C	0.104374	0.0151863	0.0245274	0.5500004	0.249708	0.0133877	1.22E-77	0.0608162	0.0982784	0.5360378	0.2804254
ENSG00000116574	RHOU	1	228876620	C	A	0.393638	0.0255409	0.0146342	0.07499978	0.251582	0.00887275	7.39E-177	0.101521	0.0582789	0.08150992	0.610036
ENSG00000116584	ARHGEF2	1	155941387	A	G	0.101392	-0.0413795	0.0283686	0.09699961	-0.158283	0.0229996	5.90E-12	0.261427	0.183208	0.1535976	0.5166045
ENSG00000116586	LAMTOR2	1	156026422	T	C	0.0656064	-0.0106839	0.0346188	0.6899999	0.157001	0.0246655	1.95E-10	-0.06805	0.22076	0.7578895	0.8059428
ENSG00000116641	DOCK7	1	63037184	A	G	0.301193	-0.010588	0.0149064	0.5700002	-0.254706	0.00840064	6.27E-202	0.0415694	0.0585399	0.4776396	0.5030262
ENSG00000116649	SRM	1	11117361	A	G	0.0208748	-0.00796033	0.0533299	0.7899998	0.170168	0.0277218	8.34E-10	-0.0467794	0.313489	0.8813787	0.1685771
ENSG00000116661	FBXO2	1	11712133	T	G	0.202783	0.00705354	0.0176447	0.6899999	0.71808	0.0144553	0	0.00982277	0.0245728	0.6893472	0.9154753
ENSG00000116663	FBXO6	1	11729296	A	G	0.393638	0.0088399	0.0145847	0.6100002	-0.222855	0.00933035	4.39E-126	-0.0396665	0.0654658	0.5445727	0.9894356
ENSG00000116667	C1orf21	1	184477173	T	C	0.189861	-0.00771287	0.0175436	0.6800001	0.122703	0.00977788	4.03E-36	-0.0628582	0.143064	0.6603931	0.4259804
ENSG00000116670	MAD2L2	1	11743122	G	T	0.149105	-0.00131648	0.0200479	0.99	-0.208876	0.0120534	2.83E-67	0.00630269	0.0959807	0.9476435	0.3309614
ENSG00000116678	LEPR	1	65996745	C	T	0.439364	0.00131224	0.0142626	0.98	0.427014	0.00800224	0	0.00307306	0.0334008	0.9266936	0.9685385
ENSG00000116679	IVNS1ABP	1	185275990	A	G	0.224652	0.0401096	0.0167837	0.02300011	0.217153	0.00925458	9.42E-122	0.184707	0.0776895	0.01743064	0.9073146
ENSG00000116685	KIAA2013	1	11983066	G	T	0.342942	0.00970852	0.0148391	0.5099998	0.819385	0.015529	0	0.0118485	0.0181114	0.5129815	0.8748507
ENSG00000116688	MFN2	1	12056904	G	A	0.473161	-0.00149983	0.0142104	0.9199999	0.879647	0.00624334	0	-0.00170504	0.0161547	0.9159438	0.8493615
ENSG00000116690	PRG4	1	186274549	A	T	0.150099	0.0150412	0.0188634	0.3599996	-0.247661	0.0107235	5.17E-118	-0.0607329	0.0762115	0.4255091	0.1113556
ENSG00000116691	MIIP	1	12085812	G	A	0.473161	-0.00149983	0.0142104	0.9199999	0.137833	0.00795168	2.61E-67	-0.0108815	0.103101	0.9159453	0.7565954
ENSG00000116698	SMG7	1	183504366	A	G	0.335984	0.0238087	0.0151151	0.08500021	-0.655793	0.00773434	0	-0.0363052	0.0230526	0.1152823	0.8365943
ENSG00000116701	NCF2	1	183542354	T	C	0.452286	-0.0176598	0.014237	0.25	0.146211	0.00800365	1.49E-74	-0.120783	0.0975973	0.215877	0.6403598
ENSG00000116704	SLC35D1	1	67492398	A	G	0.442346	-0.0210259	0.0143184	0.2	0.351982	0.0115726	3.48E-203	-0.0597358	0.0407268	0.1424455	0.8428407
ENSG00000116711	PLA2G4A	1	186878099	G	A	0.54175	0.0193697	0.0142205	0.2	0.147062	0.00794416	1.65E-76	0.131711	0.0969586	0.1743289	0.4568359
ENSG00000116717	GADD45A	1	68152382	T	C	0.0795229	0.0208047	0.0294892	0.4600002	-0.119833	0.0151726	2.83E-15	-0.173614	0.247065	0.48224	0.7573033
ENSG00000116729	WLS	1	68631472	A	C	0.159046	0.00714546	0.0191973	0.7300002	0.769124	0.00970531	0	0.00929039	0.0249602	0.7097383	0.1667862
ENSG00000116741	RGS2	1	192779786	A	G	0.459245	0.0186827	0.0142672	0.16	-0.143425	0.00794739	8.36E-73	-0.130261	0.0997366	0.1915347	0.495367

ENSG00000116747	RO60	1	193044729	A	G	0.38171	-0.0137031	0.0142628	0.29	-0.0838136	0.00802198	1.50E-25	0.163495	0.170891	0.3387078	0.5272107
ENSG00000116750	UCHL5	1	193007063	C	T	0.269384	0.0125637	0.0170648	0.4700002	0.0884017	0.00946384	9.54E-21	0.142121	0.193636	0.462974	0.3511881
ENSG00000116761	CTH	1	70891217	G	A	0.421471	1.75E-06	0.0146082	0.8800001	-0.383344	0.0077892	0	-4.57E-06	0.0381073	0.9999043	0.2150797
ENSG00000116771	AGMAT	1	15905226	A	G	0.0755467	-0.0945579	0.0276665	0.000179999	0.116708	0.0137904	2.61E-17	-0.810209	0.255659	0.00152911	0.7127082
ENSG00000116786	PLEKHM2	1	16036045	A	G	0.374751	0.0146085	0.0147755	0.2999998	-0.207598	0.00827558	7.14E-139	-0.0703692	0.0712289	0.3231867	0.3746587
ENSG00000116791	CRYZ	1	75185131	C	G	0.314115	-0.0236047	0.0152934	0.1199999	0.864808	0.00773749	0	-0.0272947	0.0176858	0.1227557	0.6493658
ENSG00000116793	PHTF1	1	114270782	G	A	0.467197	0.01826	0.0143249	0.1499999	0.177644	0.00800747	4.82E-109	0.10279	0.0807712	0.2031584	0.1087585
ENSG00000116809	ZBTB17	1	16285495	C	T	0.449304	0.00622925	0.0143476	0.6800001	0.0634351	0.00802752	2.74E-15	0.0981988	0.226519	0.6646422	0.5454225
ENSG00000116815	CD58	1	117085409	C	T	0.122266	-0.0408051	0.0234023	0.1100001	0.100322	0.0124602	8.19E-16	-0.406742	0.23868	0.08835631	0.9267734
ENSG00000116824	CD2	1	117304428	G	A	0.101392	0.0412121	0.0260225	0.1100001	-0.121947	0.0138844	1.59E-18	-0.337951	0.216834	0.1190969	0.4462031
ENSG00000116830	TTF2	1	117624208	A	G	0.0526839	-0.0210483	0.0323679	0.4400003	1.06711	0.0161382	0	-0.0197246	0.0303338	0.5155296	0.1048575
ENSG00000116852	KIF21B	1	200965674	C	A	0.0646123	-0.0160521	0.0321029	0.64	-0.146418	0.0171647	1.46E-17	0.109632	0.219631	0.6176643	0.6045949
ENSG00000116857	TMEM9	1	201122301	C	G	0.384692	0.0265943	0.0146401	0.05699936	0.685665	0.00721461	0	0.0387862	0.0213556	0.06933872	0.5966434
ENSG00000116871	MAP7D1	1	36633815	G	A	0.33996	-0.00583519	0.0153582	0.6700003	0.406876	0.0089082	0	-0.0143415	0.037748	0.7039999	0.5385994
ENSG00000116874	WARS2	1	119628566	A	G	0.249503	0.00269239	0.0163037	0.8800001	0.643959	0.00873126	0	0.004181	0.025318	0.8688343	0.9091824
ENSG00000116883	NA	1	36792076	G	A	0.421471	-0.0127992	0.0145624	0.2599998	0.461053	0.0152327	3.09E-201	-0.0277608	0.0315984	0.3796449	0.6057068
ENSG00000116885	OSCP1	1	36898757	C	A	0.297217	0.0189419	0.0152669	0.1800002	0.130374	0.00856879	2.81E-52	0.145289	0.117489	0.2162311	0.125147
ENSG00000116898	MRPS15	1	36925678	G	T	0.0904573	-0.0154466	0.0238706	0.4299995	0.230559	0.0133068	2.97E-67	-0.0669962	0.103606	0.5178608	0.693004
ENSG00000116903	EXOC8	1	231471039	A	G	0.336978	0.0295696	0.014755	0.02	0.244284	0.00968857	2.84E-140	0.121046	0.0605915	0.04574497	0.1921155
ENSG00000116906	GNPAT	1	231395336	C	T	0.481113	-0.0123065	0.0142532	0.3100002	0.192353	0.00787409	8.50E-132	-0.0639787	0.0741454	0.3882027	0.4920718
ENSG00000116918	TSNAX	1	231683334	T	C	0.300199	-0.0285139	0.0155443	0.04600023	-0.114052	0.00894116	2.89E-37	0.250009	0.137694	0.06941865	0.9919002
ENSG00000116922	AIRIM	1	38152578	A	C	0.173956	0.00395352	0.0186527	0.9299999	0.288826	0.00986148	1.46E-188	0.0136882	0.0645828	0.832147	0.07377249
ENSG00000116954	RRAGC	1	39314682	G	A	0.0715706	-0.00961501	0.0286088	0.83	0.310366	0.0165252	1.07E-78	-0.0309796	0.0921924	0.7368468	0.06019856
ENSG00000116957	NA	1	235571479	T	G	0.481113	0.00328691	0.0141607	0.6999999	0.261018	0.00785449	3.75E-242	0.0125927	0.0542531	0.8164531	0.8838056
ENSG00000116962	NID1	1	236183796	T	C	0.210736	0.0270822	0.0171802	0.08500021	-0.135249	0.00945852	2.21E-46	-0.200239	0.127796	0.1171454	0.9237152
ENSG00000116977	LGALS8	1	236698790	T	A	0.0318091	-0.00782292	0.0398921	0.58	-1.17204	0.019884	0	0.0066746	0.0340366	0.8445312	0.6898125
ENSG00000116983	HPCAL4	1	40150840	T	C	0.476143	-0.0109624	0.0143224	0.5999997	0.592943	0.00722992	0	-0.0184881	0.0241558	0.4440518	0.002546163
ENSG00000116984	MTR	1	237012945	C	A	0.0198807	-0.00052977	0.0583248	0.7800007	0.501125	0.0281155	4.62E-71	-0.00105716	0.116388	0.9927528	0.7607021
ENSG00000116985	BMP8B	1	40238693	G	A	0.0487078	-0.00795259	0.0321273	0.6600001	0.263619	0.0183334	6.99E-47	-0.030167	0.121888	0.8045236	0.3154289
ENSG00000116990	MYCL	1	40364513	C	G	0.394632	0.0163076	0.014457	0.17	-0.0735613	0.00889733	1.36E-16	-0.221687	0.198351	0.2637152	0.7631668
ENSG00000116991	SIPA1L2	1	232615507	T	C	0.228628	0.00100149	0.016793	0.9699999	0.446967	0.00888223	0	0.00224063	0.037571	0.9524445	0.5874551
ENSG00000117000	RLF	1	40666819	T	C	0.110338	-0.009265	0.0230764	0.4799997	-0.08763	0.0123649	1.37E-12	0.105729	0.263761	0.6885308	0.4547941
ENSG00000117009	KMO	1	241727189	C	T	0.0318091	-0.0871903	0.0477136	0.05899973	-0.599324	0.0230235	2.21E-149	0.145481	0.0798083	0.06832129	0.8298155
ENSG00000117010	ZNF684	1	41005537	T	C	0.355865	0.0128089	0.0153023	0.3700002	0.0537539	0.00918306	4.81E-09	0.238288	0.287569	0.407315	0.7733316
ENSG00000117013	KCNQ4	1	41277904	G	A	0.434394	0.0133008	0.0144063	0.2399999	0.0555105	0.00876638	2.42E-10	0.239609	0.262268	0.3609256	0.3284379
ENSG00000117016	RIMS3	1	41108840	G	A	0.258449	-0.00680514	0.0165236	0.5199996	0.335317	0.00891186	10902699999999e-3	-0.0202947	0.0492806	0.6804721	0.3289939
ENSG00000117020	AKT3	1	243832958	T	C	0.207753	0.00187275	0.0171129	0.95	0.116648	0.00960447	6.08E-34	0.0160547	0.146711	0.912861	0.6743531
ENSG00000117036	ETV3	1	157099624	A	G	0.514911	-0.00339412	0.0141883	0.7199992	0.0440013	0.00799704	3.75E-08	-0.0771368	0.322756	0.8111103	0.1584286
ENSG00000117054	ACADM	1	76221648	C	A	0.305169	0.0116943	0.0153816	0.4799997	0.360913	0.00868509	0	0.032402	0.0426258	0.4471641	0.2304725
ENSG00000117090	SLAMF1	1	160597487	C	T	0.355865	0.0182211	0.0148398	0.1499999	-0.159759	0.00841129	1.93E-80	-0.114054	0.0930825	0.2204638	0.4689703
ENSG00000117091	CD48	1	160665088	A	G	0.185885	0.0185007	0.0175369	0.32	-0.327493	0.00992919	1.43E-238	-0.0564918	0.0535763	0.291691	0.7595546
ENSG00000117115	PADI2	1	17419602	G	T	0.372763	-0.000313014	0.0146726	0.9299999	-0.807604	0.00676284	0	0.000387583	0.0181681	0.9829798	0.373488
ENSG00000117118	SDHB	1	17362941	A	C	0.00795229	-0.0405541	0.0829403	0.5700002	-1.10183	0.0636268	3.50E-67	0.0368063	0.0753053	0.6250113	0.1634496
ENSG00000117133	RPF1	1	84954207	T	C	0.026839	-0.0287174	0.0391307	0.4899999	-0.533489	0.0248264	1.98E-102	0.0538295	0.0733915	0.46328	0.9827726
ENSG00000117139	KDM5B	1	202737562	T	C	0.028827	-0.00962322	0.0389923	0.9699999	0.395246	0.0213312	1.21E-76	-0.0243474	0.0986619	0.8050815	0.8318172
ENSG00000117143	UAP1	1	162550474	G	A	0.482107	0.0092925	0.0143009	0.4799997	0.166585	0.00789392	7.46E-99	0.0557822	0.085888	0.5160305	0.8582292
ENSG00000117151	CTBS	1	85029483	G	T	0.0894632	0.0062767	0.0239048	0.8499999	-0.433582	0.0129222	8.03E-247	-0.0144764	0.055135	0.7928877	0.3579109
ENSG00000117153	KLHL12	1	202878996	T	C	0.0576541	0.0464011	0.0284019	0.04300015	-0.486447	0.0177149	5.32E-166	-0.0953878	0.0584897	0.1029223	0.127852
ENSG00000117155	SSX2IP	1	85132938	T	C	0.0775348	-0.0281102	0.0249823	0.2200002	-0.298843	0.0137151	2.92E-105	0.0940634	0.0837081	0.2611372	0.4075737
ENSG00000117174	ZNHIT6	1	86144611	G	A	0.515905	-0.00665631	0.0142035	0.4700002	0.279351	0.00780734	2.22E-280	-0.0238278	0.0508491	0.6393566	0.7537223

ENSG00000117222	RBBP5	1	205073206	A	G	0.392644	0.00124881	0.0147473	0.83	0.0535588	0.00816869	5.50E-11	0.0233166	0.275371	0.9325209	0.1699558
ENSG00000117226	GBP3	1	89480463	G	A	0.32008	0.00480711	0.0151194	0.8700001	-0.821571	0.00736474	0	-0.00585112	0.0184031	0.750529	0.754455
ENSG00000117228	GBP1	1	89524522	G	A	0.378728	0.00869396	0.0146147	0.6700003	-0.0644191	0.00831644	9.48E-15	-0.134959	0.227537	0.5530933	0.4007672
ENSG00000117242	PINK1-AS	1	20973918	C	T	0.487078	-0.000158881	0.0142201	0.84	-0.334239	0.015435	5.50E-104	0.000475352	0.0425447	0.9910854	0.222981
ENSG00000117245	KIF17	1	21017508	C	T	0.394632	0.00843451	0.0150942	0.4500005	-0.0774847	0.00824938	5.84E-21	-0.108854	0.195147	0.5769774	0.3901705
ENSG00000117262	GPR89A	1	145795757	C	T	0.0377734	-0.0209486	0.0392764	0.5199996	0.69743	0.0550301	8.28E-37	-0.0300369	0.0563658	0.5941084	0.696337
ENSG00000117266	CDK18	1	205487822	A	G	0.194831	0.0171944	0.0180828	0.3900004	-0.0740631	0.0103299	7.51E-13	-0.232159	0.246292	0.3458769	0.3057182
ENSG00000117280	RAB29	1	205740851	A	G	0.32505	0.00287748	0.0150376	0.7400005	-0.341477	0.00910822	1.29E-307	-0.00842657	0.0440375	0.8482515	0.642494
ENSG00000117281	CD160	1	145705706	C	T	0.496024	0.00631208	0.0143914	0.7199992	-0.235495	0.0118598	9.66E-88	-0.0268034	0.0611261	0.6610283	0.816752
ENSG00000117298	ECE1	1	21607868	A	C	0.365805	0.00957059	0.0148447	0.4500005	0.32479	0.00890808	4.76E-291	0.029467	0.0457127	0.5191777	0.528267
ENSG00000117305	HMGCL	1	24146742	C	T	0.0765408	0.0436531	0.0254809	0.08500021	0.326631	0.0142518	3.03E-116	0.133646	0.0782288	0.08756156	0.886405
ENSG00000117308	GALE	1	24124680	C	A	0.412525	0.0103609	0.0144163	0.33	-0.0727604	0.00824518	1.10E-18	-0.142397	0.19879	0.4737927	0.3740921
ENSG00000117318	ID3	1	23885347	C	T	0.480119	0.00606205	0.0148115	0.8600001	0.142311	0.00870364	4.29E-60	0.0425971	0.104111	0.6824283	0.2805126
ENSG00000117322	CR2	1	207645407	C	T	0.200795	-0.000426137	0.0172776	0.9599999	0.131591	0.0103703	6.79E-37	-0.00323833	0.131298	0.9803229	0.3422763
ENSG00000117335	CD46	1	207947130	G	T	0.200795	0.0299386	0.0171723	0.08999948	-0.0745897	0.00964662	1.06E-14	-0.401377	0.236003	0.08899407	0.1397642
ENSG00000117362	APH1A	1	150239892	T	C	0.140159	0.0102532	0.0214832	0.4600002	0.130478	0.0207306	3.09E-10	0.0785818	0.165123	0.6341458	0.6867759
ENSG00000117385	P3H1	1	43222380	C	A	0.398608	0.005405	0.0144227	0.59	0.235607	0.00800599	2.34E-190	0.0229407	0.0612199	0.7078642	0.1819498
ENSG00000117394	SLC2A1	1	43407791	A	G	0.403579	-0.00716592	0.0144765	0.4500005	-0.0826125	0.00806464	1.26E-24	0.0867413	0.175438	0.6210049	0.6223377
ENSG00000117395	EBNA1BP2	1	43678717	G	A	0.28827	-0.0216234	0.016198	0.1299999	0.289124	0.00979267	1.40E-191	-0.0747895	0.0560817	0.1823415	0.2966593
ENSG00000117399	CDC20	1	43826750	A	T	0.370775	0.000670963	0.0145533	0.9699999	-0.0777738	0.0138827	2.12E-08	-0.0086271	0.18713	0.9632287	0.4556069
ENSG00000117400	MPL	1	43810960	A	T	0.370775	0.000670963	0.0145533	0.9699999	-0.197584	0.0137781	1.22E-46	-0.00339584	0.0736568	0.9632277	0.5952606
ENSG00000117408	IPO13	1	44423152	A	C	0.125249	-0.0200489	0.0233105	0.4100001	0.118148	0.012918	5.91E-20	-0.169693	0.198169	0.3918306	0.03721689
ENSG00000117410	ATP6V0B	1	44442063	T	C	0.0775348	-0.0190844	0.0283581	0.5700002	0.39508	0.0160791	2.58E-133	-0.0483052	0.0718051	0.5011213	0.05891152
ENSG00000117425	PTCH2	1	45297125	C	T	0.365805	-0.000631637	0.0145745	0.6999999	-0.061185	0.00814324	5.75E-14	0.0103234	0.238208	0.9654323	0.1309085
ENSG00000117448	AKR1A1	1	46025968	G	A	0.480119	0.0054126	0.0143598	0.59	-0.0970596	0.00795792	3.24E-34	-0.0557658	0.148019	0.7063613	0.6668734
ENSG00000117450	PRDX1	1	45982713	A	C	0.082505	0.0264557	0.0239198	0.2099999	-0.373576	0.0138669	7.43E-160	-0.0708174	0.0640832	0.2691224	0.8128613
ENSG00000117461	PIK3R3	1	46573986	G	A	0.460239	-0.004905	0.0142346	0.8800001	0.255361	0.00785015	4.08E-232	-0.0192081	0.0557461	0.7304225	0.7368416
ENSG00000117475	BLZF1	1	169351493	A	G	0.22664	-0.0355313	0.0174419	0.02	-0.0683928	0.00954247	7.65E-13	0.519518	0.265127	0.0500531	0.323152
ENSG00000117479	SLC19A2	1	169444194	T	G	0.0616302	0.00589317	0.030046	0.89	0.172662	0.0164093	6.83E-26	0.0341312	0.174046	0.8445286	0.953777
ENSG00000117480	FAAH	1	46869728	G	A	0.272366	0.0048197	0.015522	0.89	0.36926	0.00898507	0	0.0130523	0.0420366	0.7561817	0.3756299
ENSG00000117481	NSUN4	1	46818336	C	T	0.277336	0.00453279	0.0154662	0.91	0.663303	0.00798164	0	0.00683366	0.0233171	0.7694646	0.2743992
ENSG00000117500	TMED5	1	93630792	T	C	0.342942	-0.00142722	0.0148615	0.89	-0.442324	0.00801918	0	0.00322664	0.0335987	0.9234931	0.8561153
ENSG00000117505	DR1	1	93819797	T	C	0.363817	0.00738094	0.0146941	0.5099998	0.490599	0.00785837	0	0.0150448	0.0299523	0.6154635	0.174283
ENSG00000117519	CNN3	1	95377670	C	A	0.0258449	-0.0321768	0.0431656	0.5	-0.528091	0.0287106	1.48E-75	0.0609305	0.0818061	0.4563832	0.9375862
ENSG00000117528	ABCD3	1	94934077	A	G	0.393638	0.000566016	0.0144562	0.98	0.29954	0.0078533	0	0.00188962	0.0482614	0.9687677	0.5685762
ENSG00000117533	VAMP4	1	171690343	C	G	0.173956	-0.010258	0.01783	0.59	0.202141	0.0102734	3.45E-86	-0.0507467	0.0882434	0.5652394	0.9556641
ENSG00000117560	FASLG	1	172632084	G	A	0.158052	0.0133749	0.0196788	0.6600001	-0.199079	0.0106049	1.27E-78	-0.0671841	0.0989142	0.4970014	0.05311996
ENSG00000117569	PTBP2	1	97233785	A	G	0.535785	0.00702221	0.0141942	0.6700003	0.346746	0.00774998	0	0.0202518	0.040938	0.6208164	0.7387471
ENSG00000117586	TNFSF4	1	173164662	C	T	0.193837	-0.0285777	0.0178465	0.09400046	-0.390882	0.00986888	0	0.0731107	0.0456942	0.1095989	0.3226658
ENSG00000117592	PRDX6	1	173452175	G	A	0.166004	0.00848719	0.0182158	0.7099994	0.327406	0.0101102	4.66E-230	0.0259225	0.0556425	0.641304	0.8908987
ENSG00000117593	DARS2	1	173810662	T	C	0.271372	0.00522429	0.0157497	0.81	-0.167662	0.00887842	1.54E-79	-0.0311597	0.0939517	0.7401489	0.6714005
ENSG00000117595	IRF6	1	209969250	T	C	0.411531	-0.00225244	0.0144249	0.7600007	0.39482	0.00780969	0	-0.00570498	0.0365356	0.8759158	0.01700102
ENSG00000117597	UTP25	1	210016131	A	C	0.477137	-0.0349847	0.0142935	0.01499996	0.512965	0.00828393	0	-0.0682009	0.0278862	0.01445771	0.05537005
ENSG00000117601	SERPINC1	1	173879731	A	G	0.116302	0.0260317	0.0222304	0.28	0.439698	0.0126086	1.91E-266	0.0592035	0.0505868	0.2418658	0.6848163
ENSG00000117602	RCAN3	1	24846446	A	G	0.0596421	0.0236545	0.0301965	0.4500005	0.205256	0.016578	3.30E-35	0.115244	0.147411	0.4343385	0.4099212
ENSG00000117616	RSRP1	1	25616716	A	G	0.388668	0.00647447	0.0144501	0.6200004	-0.177569	0.00972545	1.78E-74	-0.0364617	0.0814018	0.6542093	0.1727862
ENSG00000117620	SLC35A3	1	100463940	G	A	0.154076	0.0171022	0.0193629	0.5	-0.0753331	0.0112835	2.45E-11	-0.227021	0.25927	0.3812385	0.4277141
ENSG00000117625	RCOR3	1	211460723	G	C	0.0447316	-0.0225415	0.0377864	0.5700002	-0.135201	0.021244	1.96E-10	0.166726	0.280709	0.5525476	0.8623722
ENSG00000117632	STMN1	1	26222077	G	T	0.198807	-0.0461747	0.0174925	0.005600025	-0.204226	0.0110069	7.51E-77	0.226096	0.0865149	0.008965399	0.002970089
ENSG00000117640	MTFR1L	1	26152281	G	T	0.22664	0.0297579	0.0174558	0.08300036	0.100457	0.013248	3.38E-14	0.296225	0.178101	0.09626397	0.05492703

ENSG00000117643	MAN1C1	1	26028328	C	T	0.298211	0.00161707	0.0152817	0.9	0.111965	0.00935273	5.02E-33	0.0144426	0.136491	0.9157305	0.2518095
ENSG00000117676	RPS6KA1	1	26878886	T	C	0.222664	-0.00890758	0.017606	0.4700002	0.247067	0.00958056	1.20E-146	-0.0360533	0.0712737	0.6129671	0.5212399
ENSG00000117682	DHDDS	1	26778279	A	G	0.234592	-0.016462	0.0174171	0.25	-0.132878	0.00952534	3.15E-44	0.123888	0.131376	0.3456803	0.7159967
ENSG00000117691	NENF	1	212612971	A	G	0.380716	-0.00836818	0.0144503	0.4199997	-0.186847	0.00808624	3.96E-118	0.0447862	0.0773618	0.5626438	0.3432429
ENSG00000117697	NSL1	1	212932309	A	C	0.460239	0.0118227	0.014195	0.3100002	0.260143	0.00786928	1.19E-239	0.0454468	0.0545834	0.4050627	0.07524937
ENSG00000117713	ARID1A	1	27065559	G	A	0.0715706	-0.0396226	0.0261064	0.16	-0.849727	0.0153505	0	0.0466298	0.0307348	0.129225	0.1495717
ENSG00000117724	CENPF	1	214807234	C	A	0.028827	-0.0179022	0.0512678	0.6800001	0.390924	0.0286027	1.59E-42	-0.0457946	0.131188	0.7270322	0.5307051
ENSG00000117748	RPA2	1	28229646	A	G	0.0626243	-0.000582167	0.0284018	0.9199999	0.866754	0.0202137	0	-0.000671664	0.032768	0.9836465	0.9051203
ENSG00000117791	MTARC2	1	220939858	G	A	0.141153	-0.0264559	0.0216576	0.1499999	-0.217994	0.0219297	2.77E-23	0.121361	0.100097	0.2253468	0.620396
ENSG00000117834	SLC5A9	1	48701336	A	G	0.117296	-0.0168524	0.0232988	0.3900004	-0.0885482	0.0139393	2.12E-10	0.190319	0.26482	0.4723428	0.5203046
ENSG00000117859	OSBPL9	1	52148870	T	C	0.0457256	0.0183318	0.0327725	0.7300002	0.222378	0.0178439	1.20E-35	0.0824354	0.147522	0.5762964	0.602176
ENSG00000117862	TXNDC12	1	52503823	C	T	0.0735586	-0.0447259	0.0281954	0.064	-0.707527	0.0149668	0	0.0632144	0.039873	0.1128775	0.09533799
ENSG00000117868	ESYT2	7	158573315	T	C	0.469185	0.00298237	0.0142841	0.8499999	-0.367075	0.00773161	0	-0.0081247	0.0389137	0.8346141	0.5517361
ENSG00000117877	POLR1G	19	45911745	A	G	0.132207	0.00550881	0.0205702	0.8200001	-0.152973	0.0123733	4.13E-35	-0.0360116	0.134501	0.7888975	0.9836792
ENSG00000117899	MESD	15	81260943	A	C	0.207753	0.0638843	0.0181359	0.000580003	-0.116197	0.0104014	5.64E-29	-0.549795	0.163655	0.000780904	0.4908657
ENSG00000117906	RCN2	15	77233280	C	T	0.127237	0.00919177	0.0225117	0.58	-0.120968	0.0125634	6.05E-22	-0.0759853	0.186264	0.6833147	0.84653
ENSG00000117984	CTSD	11	1779602	A	G	0.0258449	0.0580923	0.0490639	0.2399999	-0.555971	0.0380348	2.18E-48	-0.104488	0.0885381	0.2379414	0.3063557
ENSG00000118004	COLEC11	2	3667237	A	G	0.110338	-0.0114609	0.0220088	0.5	0.232365	0.0129825	1.21E-71	-0.0493228	0.0947565	0.6027002	0.1147096
ENSG00000118007	STAG1	3	136263148	A	G	0.101392	0.00730892	0.0239614	0.7800007	0.280008	0.0145658	2.35E-82	0.0261025	0.0855848	0.7603734	0.03848327
ENSG00000118046	STK11	19	1208917	T	G	0.219682	-0.00548644	0.0172161	0.7099994	-0.318075	0.0117361	9.25E-162	0.0172489	0.0541297	0.7499853	0.1897276
ENSG00000118096	IFT46	11	118429464	T	C	0.133201	-5.91E-05	0.0211258	0.9699999	-0.214749	0.0113091	2.10E-80	0.000274975	0.0983744	0.9977698	0.8945609
ENSG00000118113	MMP8	11	102590153	G	C	0.444334	-0.0207643	0.0142412	0.2200002	-0.0869291	0.00807216	4.82E-27	0.238865	0.16532	0.1484968	0.3722473
ENSG00000118162	KPTN	19	47982963	T	C	0.0188867	0.158681	0.0652649	0.007799917	0.464759	0.0446077	2.04E-25	0.341427	0.1442	0.01789796	0.5482343
ENSG00000118181	RPS25	11	118887911	C	T	0.257455	0.0126505	0.0161444	0.35	-0.184419	0.0136298	1.03E-41	-0.0685964	0.0876885	0.4340537	0.7623831
ENSG00000118197	DDX59	1	200616060	G	C	0.426441	0.00953726	0.0143777	0.4199997	-0.618034	0.00726323	0	-0.0154316	0.0232643	0.5071277	0.8471878
ENSG00000118200	CAMSAP2	1	200769259	A	C	0.233598	0.00375069	0.017728	0.98	-0.27145	0.0102306	4.02E-155	-0.0138172	0.0653105	0.8324488	0.5354174
ENSG00000118217	ATF6	1	161834972	A	G	0.100398	-0.0215752	0.0231669	0.1900002	0.564033	0.0118447	0	-0.0382516	0.0410815	0.3517935	0.06296692
ENSG00000118242	MREG	2	216854016	T	G	0.340954	0.000823669	0.0149202	0.81	-0.0642529	0.0096236	2.45E-11	-0.0128192	0.232218	0.9559767	0.9391061
ENSG00000118246	FASTKD2	2	207643657	G	A	0.0467197	-0.0390748	0.0279949	0.2999998	-0.160581	0.017448	3.47E-20	0.243334	0.176329	0.1675858	0.5990989
ENSG00000118257	NRP2	2	206604785	G	A	0.0626243	0.0645648	0.0320003	0.06699926	0.134258	0.0182005	1.62E-13	0.480902	0.247105	0.05163715	0.4273794
ENSG00000118260	CREB1	2	208431308	C	G	0.163022	-0.0188563	0.018864	0.3100002	0.16986	0.0108817	6.26E-55	-0.111011	0.111284	0.3184983	0.6254412
ENSG00000118263	KLF7	2	207985426	A	G	0.416501	-0.0131484	0.0144542	0.2200002	-0.0876386	0.00803935	1.14E-27	0.15003	0.165503	0.3646666	0.5006655
ENSG00000118276	B4GALT6	18	29234004	T	C	0.43837	-0.00845185	0.0143389	0.5099998	-0.139314	0.00800378	7.43E-68	0.0606677	0.102984	0.555796	0.4950637
ENSG00000118292	C1orf54	1	150246963	A	G	0.212724	-0.0231414	0.0171005	0.14	-0.141474	0.0145895	3.10E-22	0.163574	0.122045	0.1801574	0.3004124
ENSG00000118298	CA14	1	150233823	T	C	0.140159	0.0106252	0.0214635	0.4400003	0.256246	0.031319	2.80E-16	0.0414648	0.0839144	0.6212129	0.5546284
ENSG00000118307	DNAI7	12	25304725	G	T	0.475149	-0.0222429	0.0142299	0.17	0.119467	0.00805663	9.60E-50	-0.186185	0.119772	0.1200659	0.1448209
ENSG00000118308	IRAG2	12	25217602	T	C	0.390656	0.0198909	0.0145137	0.17	0.168026	0.00815487	2.51E-94	0.11838	0.0865685	0.1714781	0.0539527
ENSG00000118363	SPCS2	11	74675184	G	C	0.240557	-0.02034	0.0170627	0.2	-0.182244	0.0173768	9.83E-26	0.111609	0.0942284	0.236236	0.001825359
ENSG00000118369	USP35	11	77912807	G	A	0.195825	0.00769613	0.0189786	0.58	0.368467	0.0116514	1.71E-219	0.0208869	0.0515112	0.6851226	0.1839635
ENSG00000118402	ELOVL4	6	80640913	A	G	0.43837	-0.0100287	0.0143844	0.4	0.230991	0.00788909	1.88E-188	-0.043416	0.0622902	0.4858058	0.4140159
ENSG00000118412	CASP8AP2	6	90561884	C	T	0.149105	0.00969328	0.0211355	0.6100002	-0.123843	0.0178243	3.71E-12	-0.0782706	0.171035	0.6472185	0.1831478
ENSG00000118418	HMGN3	6	79927684	G	T	0.0646123	0.0147114	0.0263112	0.4299995	-0.21177	0.0154006	5.04E-43	-0.0694688	0.124347	0.5763876	0.7758009
ENSG00000118420	UBE3D	6	83688838	C	T	0.319085	0.0104188	0.0152797	0.3900004	0.0888706	0.00973071	6.66E-20	0.117236	0.172411	0.4965174	0.8463562
ENSG00000118432	CNR1	6	88862830	G	A	0.363817	0.0292877	0.0143827	0.02199986	0.328517	0.0080417	0	0.0891512	0.043835	0.04197284	0.08005627
ENSG00000118454	ANKRD13C	1	70773344	G	T	0.105368	0.0260322	0.0249354	0.3400001	-0.310484	0.0143494	7.99E-104	-0.0838441	0.0804049	0.2970536	0.6666636
ENSG00000118473	SGIP1	1	67106524	C	T	0.143141	0.00303123	0.019139	0.6300007	-0.268037	0.0106331	3.29E-140	-0.011309	0.0714056	0.87416	0.5061551
ENSG00000118482	PHF3	6	64417477	C	A	0.0695825	-0.0297433	0.0263373	0.2700001	-0.129553	0.0153165	2.71E-17	0.229584	0.205098	0.2629742	0.9208391
ENSG00000118495	PLAGL1	6	144323586	A	G	0.0347913	0.0301497	0.033964	0.32	1.07131	0.0189322	0	0.0281429	0.0317072	0.3747632	0.1373458
ENSG00000118496	FBXO30	6	146127580	G	T	0.0775348	-0.00113974	0.0244938	0.98	-0.188928	0.0137618	6.86E-43	0.00603268	0.129647	0.9628866	0.1329012
ENSG00000118503	TNFAIP3	6	138196400	C	T	0.384692	0.00609981	0.0147071	0.7300002	-0.0568489	0.00818016	3.66E-12	-0.107299	0.259165	0.6788623	0.4661191

ENSG00000118507	AKAP7	6	131530740	C	G	0.378728	-0.0043419	0.0147859	0.84	-0.381221	0.00814117	0	0.0113895	0.0387864	0.7690287	0.005055604
ENSG00000118508	RAB32	6	146870465	G	A	0.0308151	0.0135317	0.0456638	0.7099994	0.312663	0.0265234	4.49E-32	0.0432789	0.146094	0.7670471	0.4564813
ENSG00000118513	MYB	6	135521382	T	C	0.263419	-0.00650836	0.0161967	0.6700003	-0.0510905	0.00901226	1.44E-08	0.127389	0.317815	0.6885477	0.7692828
ENSG00000118514	ALDH8A1	6	135254894	T	C	0.491054	0.0119112	0.0141806	0.4600002	-0.364236	0.00777702	0	-0.0327019	0.0389387	0.401004	0.1564186
ENSG00000118515	SGK1	6	134564817	G	T	0.0715706	0.0119871	0.0323307	0.6700003	0.631131	0.019214	1.24E-236	0.018993	0.0512299	0.71083	0.9886485
ENSG00000118518	RNF146	6	127598733	G	A	0.34493	-0.0119227	0.0145435	0.5500004	-0.122292	0.00848516	4.32E-47	0.0974941	0.119117	0.4130873	0.1352355
ENSG00000118520	ARG1	6	131899878	C	T	0.170974	0.0426358	0.0190628	0.02199986	-0.669133	0.00989284	0	-0.063718	0.0285044	0.02539273	0.7710467
ENSG00000118523	CCN2	6	132270914	C	T	0.422465	0.0155292	0.0143693	0.1900002	-0.197232	0.00807304	8.01E-132	-0.0787356	0.0729259	0.2802914	0.2198873
ENSG00000118564	FBXL5	4	15644732	C	T	0.267396	0.0311215	0.0156001	0.03500016	-0.199768	0.0087117	2.28E-116	-0.155788	0.0783861	0.04687276	0.581219
ENSG00000118579	MED28	4	17621865	G	A	0.373757	0.0220595	0.0144404	0.14	-0.245273	0.00823981	1.05E-194	-0.0899384	0.0589522	0.1271055	0.8279447
ENSG00000118596	SLC16A7	12	60083121	T	C	0.285288	-0.00147685	0.0165956	0.95	0.156388	0.0102465	1.36E-52	-0.00944351	0.10612	0.9290907	0.9277741
ENSG00000118600	RXYLT1	12	64188460	A	G	0.104374	-0.0288033	0.0233919	0.2099999	-0.177036	0.0132685	1.31E-40	0.162697	0.132692	0.2201512	0.5360852
ENSG00000118620	ZNF430	19	21223141	A	G	0.441352	0.00543409	0.0143462	0.7700005	0.183593	0.0080184	5.05E-116	0.0295986	0.0781522	0.7048878	0.3120213
ENSG00000118640	VAMP8	2	85798919	G	A	0.431412	-0.029512	0.0143583	0.0519996	-0.464511	0.00764528	0	0.0635334	0.0309282	0.03995427	0.2281139
ENSG00000118655	DCLRE1B	1	114452235	G	A	0.236581	-0.0357162	0.0167597	0.01899984	0.288109	0.00948106	7.94E-203	-0.123967	0.0583142	0.03351532	0.8306767
ENSG00000118680	MYL12B	18	3270094	A	C	0.284294	0.00557933	0.0157943	0.6700003	-0.115075	0.00912671	1.89E-36	-0.0484843	0.137306	0.7240052	0.9658955
ENSG00000118690	ARMC2	6	109232402	G	A	0.228628	0.0305119	0.017002	0.07799917	0.170546	0.00941149	2.18E-73	0.178907	0.100179	0.07412029	0.4185195
ENSG00000118705	RPN2	20	35838417	T	C	0.199801	-0.00958467	0.0181314	0.6499995	-0.136351	0.0146324	1.18E-20	0.0702943	0.13319	0.5976561	0.7788937
ENSG00000118762	PKD2	4	88963874	G	A	0.0248509	-0.0610848	0.0486202	0.2300001	1.36638	0.0263172	0	-0.0447055	0.0355936	0.2091163	0.8929114
ENSG00000118777	ABCG2	4	89081945	G	A	0.140159	0.00990914	0.0198159	0.6300007	0.404589	0.0116111	5.05E-266	0.0244919	0.0489829	0.617069	0.2205609
ENSG00000118785	SPP1	4	88900690	T	C	0.33499	0.0106914	0.0155603	0.6600001	-0.471442	0.00829515	0	-0.0226781	0.0330082	0.4920547	0.9824674
ENSG00000118804	STBD1	4	77202819	T	C	0.130219	0.056603	0.0211141	0.01199997	-0.300449	0.0115627	7.43E-149	-0.188395	0.0706482	0.007660898	0.317499
ENSG00000118816	CCNI	4	77982734	T	G	0.0497018	0.0193356	0.0378434	0.6999999	-0.26804	0.0195024	5.53E-43	-0.0721369	0.141283	0.609642	0.3906668
ENSG00000118855	MFSD1	3	158498747	T	C	0.429423	0.0169861	0.014249	0.2	0.209496	0.00786791	3.34E-156	0.0810809	0.0680839	0.2336937	0.03721296
ENSG00000118873	RAB3GAP2	1	220383715	C	T	0.311133	0.0202323	0.0147944	0.1199999	-0.201441	0.0089236	7.81E-113	-0.100438	0.0735774	0.1722335	0.03314325
ENSG00000118894	EEF2KMT	16	5141057	A	G	0.478131	0.00126029	0.0144187	0.9299999	0.145881	0.0100794	1.79E-47	0.00863917	0.0988406	0.9303496	0.8253534
ENSG00000118898	PPL	16	4971625	G	A	0.470179	0.0168579	0.0142983	0.2099999	-0.234443	0.00806025	5.35E-186	-0.0719063	0.0610385	0.2387779	0.8376908
ENSG00000118900	UBN1	16	4914513	G	A	0.496024	0.0269254	0.0142475	0.03799969	-0.0863333	0.0080304	5.87E-27	-0.311877	0.167559	0.06270258	0.687249
ENSG00000118922	KLF12	13	74484310	T	C	0.15507	0.024438	0.0197388	0.17	-0.291102	0.0111879	2.99E-149	-0.0839498	0.0678838	0.2162096	0.9187384
ENSG00000118939	UCHL3	13	76151852	T	C	0.215706	0.000331554	0.0186806	0.7600007	0.109846	0.0147404	9.19E-14	0.00301836	0.170063	0.9858395	0.86592
ENSG00000118960	HS1BP3	2	20805528	T	C	0.367793	0.0110956	0.014699	0.2700001	-0.0994696	0.00898779	1.81E-28	-0.111548	0.148117	0.4513872	0.9110022
ENSG00000118961	LDAH	2	20953335	C	T	0.249503	0.00823059	0.0167294	0.56	-0.585268	0.0085732	0	-0.0140629	0.0285849	0.62274	0.4090023
ENSG00000118965	WDR35	2	20149956	C	T	0.2833	0.00246426	0.0156002	0.91	-0.141405	0.00856533	3.16E-61	-0.017427	0.110328	0.8744914	0.3430314
ENSG00000118971	CCND2	12	4398727	G	A	0.262425	0.0043934	0.0165127	0.95	-0.107408	0.00902525	1.17E-32	-0.040904	0.153777	0.7902427	0.3807971
ENSG00000118985	ELL2	5	95259288	C	G	0.289264	-0.00786585	0.0158046	0.7400005	-0.167414	0.0130521	1.16E-37	0.0469843	0.0944752	0.6189638	0.4680658
ENSG00000118997	DNAH7	2	196767981	T	C	0.358847	0.0147259	0.0149149	0.5199996	0.158023	0.00848401	1.98E-77	0.0931882	0.0945168	0.3241606	0.7096473
ENSG00000119041	GTF3C3	2	197646102	T	C	0.0636183	-0.00853855	0.029409	0.9400001	-0.658145	0.0222373	1.66E-192	0.0129737	0.0446868	0.7715684	0.1790629
ENSG00000119042	SATB2	2	200235106	A	G	0.0417495	-0.000764994	0.0377109	0.89	-0.16994	0.0231267	2.01E-13	0.00450156	0.221908	0.9838155	0.2654811
ENSG00000119048	UBE2B	5	133717276	C	T	0.0755467	-0.0135377	0.0264568	0.5099998	-0.367039	0.0204369	4.04E-72	0.0368836	0.0721111	0.6090127	0.4410621
ENSG00000119121	TRPM6	9	77420210	G	A	0.390656	-0.000812063	0.0147514	0.7700005	-0.397838	0.0078685	0	0.00204119	0.0370789	0.9560987	0.07147788
ENSG00000119138	KLF9	9	73014521	C	T	0.417495	-0.00929055	0.014262	0.7400005	-0.156241	0.00810849	9.82E-83	0.059463	0.0913343	0.5150156	0.8570304
ENSG00000119139	TJP2	9	71803174	T	C	0.399602	-0.0293733	0.0143804	0.04	-0.666967	0.00715915	0	0.0440401	0.0215661	0.04114142	0.9808766
ENSG00000119147	ECRG4	2	106687158	C	G	0.126243	-6.07E-05	0.0219291	0.9400001	-0.425654	0.0118612	4.98E-282	0.000142707	0.0515186	0.9977898	0.02322948
ENSG00000119185	ITGB1BP1	2	9553640	C	G	0.321074	0.0182026	0.015051	0.16	0.160961	0.00860378	4.24E-78	0.113087	0.0937022	0.2274801	0.02377664
ENSG00000119203	CPSF3	2	9588463	C	A	0.0735586	-0.000557276	0.0255256	0.99	0.281169	0.0214594	3.19E-39	-0.001982	0.090784	0.982582	0.01348971
ENSG00000119227	PIGZ	3	196684572	G	A	0.488072	0.0136275	0.0142717	0.3800004	0.295672	0.00853206	3.85E-263	0.0460898	0.0482869	0.3398309	0.5307655
ENSG00000119231	SENP5	3	196628156	G	A	0.528827	0.0167808	0.0142018	0.3400001	-0.104872	0.00865083	8.00E-34	-0.160012	0.136062	0.2395854	0.6014662
ENSG00000119242	CCDC92	12	124430292	T	C	0.222664	-0.0136476	0.0181939	0.4400003	0.278873	0.0102516	6.02E-163	-0.0489384	0.0652656	0.4533544	0.4248325
ENSG00000119280	C1orf198	1	230989100	T	C	0.151093	0.0203102	0.0189694	0.2399999	0.27983	0.0104737	2.96E-157	0.0725805	0.0678435	0.2846987	0.6505823
ENSG00000119285	HEATR1	1	236740054	T	C	0.384692	-0.00192837	0.0147843	0.7499995	-0.118656	0.00831009	2.98E-46	0.0162517	0.124603	0.8962277	0.9720606

ENSG00000119314	PTBP3	9	115038331	C	T	0.472167	-0.0109498	0.0141922	0.5999997	-0.204359	0.00876368	2.86E-120	0.0535813	0.0694855	0.4406391	0.9910759
ENSG00000119318	RAD23B	9	110069946	C	T	0.286282	-0.00125527	0.0151139	0.9699999	0.188029	0.00839039	3.14E-111	-0.00667595	0.0803813	0.933809	0.4200823
ENSG00000119321	FKBP15	9	115953463	T	C	0.0616302	-0.0314992	0.027184	0.1900002	0.465849	0.01776	1.20E-151	-0.0676167	0.0584105	0.2470226	0.2312389
ENSG00000119326	CTNNAL1	9	111740330	A	T	0.0248509	-0.0531057	0.0456529	0.1499999	1.18806	0.0237839	0	-0.0446995	0.0384369	0.2448563	0.5367377
ENSG00000119328	ABITRAM	9	111704742	C	A	0.359841	0.0167506	0.0147379	0.2300001	-0.354389	0.0157129	1.23E-112	-0.0472662	0.0416396	0.2563226	0.295977
ENSG00000119383	PTPA	9	131892227	C	A	0.265408	0.0163513	0.0154678	0.2200002	-0.545277	0.0087095	0	-0.0299872	0.0283709	0.2905262	0.3175707
ENSG00000119392	GLE1	9	131285773	G	C	0.0516899	0.00749775	0.036089	0.9400001	-0.416508	0.0189139	1.80E-107	-0.0180015	0.0866505	0.8354258	0.6107126
ENSG00000119396	RAB14	9	123962853	A	G	0.145129	0.00844393	0.0199418	0.5400003	-0.392189	0.0123566	4.46E-221	-0.0215302	0.0508519	0.67201	0.8945452
ENSG00000119397	CNTRL	9	123888514	T	C	0.450298	0.00726017	0.014329	0.5300002	0.210123	0.00886214	2.83E-124	0.034552	0.0682089	0.6124629	0.8678051
ENSG00000119401	TRIM32	9	119456580	G	A	0.310139	-0.0134938	0.0151076	0.4899999	0.170184	0.00843097	1.31E-90	-0.0792894	0.0888589	0.3722287	0.8251708
ENSG00000119402	FBXW2	9	123534973	A	G	0.405567	-0.0156901	0.0144259	0.28	-0.110076	0.0082092	5.37E-41	0.142539	0.131485	0.2783322	0.748911
ENSG00000119403	PHF19	9	123628791	C	T	0.435388	-0.00417424	0.0143069	0.64	-0.241835	0.0079963	6.37E-201	0.0172607	0.0591624	0.7704774	0.952667
ENSG00000119408	NEK6	9	127067735	G	A	0.187873	-0.0228735	0.0177193	0.2399999	-0.625658	0.00935626	0	0.0365591	0.0283263	0.1968285	0.3755283
ENSG00000119411	BSPRY	9	116122667	T	C	0.176938	-0.0113522	0.0181187	0.5500004	-0.403128	0.0105505	0	0.0281603	0.0449513	0.5310121	0.1575106
ENSG00000119414	PPP6C	9	127930535	T	C	0.309145	-0.0100503	0.0152848	0.5199996	-0.156674	0.00874245	8.09E-72	0.0641481	0.0976239	0.5111208	0.4205425
ENSG00000119421	NDUFA8	9	124908421	T	C	0.05666	0.0240236	0.0311493	0.58	0.345589	0.0178075	6.73E-84	0.0695149	0.090205	0.440925	0.2542831
ENSG00000119431	HDHD3	9	116137489	T	C	0.180915	0.00947802	0.0185156	0.4	-0.598343	0.0105473	0	-0.0158405	0.0309461	0.60874	0.934477
ENSG00000119446	RBM18	9	125013510	C	G	0.417495	-0.00457105	0.014444	0.9400001	0.161746	0.0080647	1.79E-89	-0.0282607	0.0893118	0.7516779	0.3766331
ENSG00000119457	SLC46A2	9	115647196	C	T	0.237575	0.00458426	0.0159732	0.8800001	-0.42458	0.0085535	0	-0.0107972	0.0376218	0.7741183	0.5568144
ENSG00000119471	HSDL2	9	115188453	T	C	0.28827	0.0110897	0.0158816	0.58	-0.423141	0.00850289	0	-0.0262081	0.0375364	0.4850492	0.4280972
ENSG00000119487	MAPKAP1	9	128334592	G	A	0.477137	0.0203838	0.0142445	0.1499999	0.104654	0.00797653	2.52E-39	0.194774	0.136918	0.1548643	0.1679479
ENSG00000119509	INVS	9	102962410	C	T	0.32505	0.0179181	0.0152391	0.3400001	0.255705	0.00803386	2.62E-222	0.0700734	0.0596371	0.2399962	0.7780187
ENSG00000119514	GALNT12	9	101591172	C	T	0.0685885	0.0299491	0.0317161	0.4799997	-0.241551	0.018423	2.83E-39	-0.123987	0.131642	0.3462707	0.1353419
ENSG00000119522	DENND1A	9	126417182	T	C	0.083499	0.0118443	0.0273877	0.8499999	-0.40817	0.0130157	7.18E-216	-0.0290181	0.0671052	0.6654313	0.9613698
ENSG00000119535	CSF3R	1	36940261	T	C	0.0119284	0.10182	0.0664102	0.1199999	-0.522563	0.039365	3.24E-40	-0.194847	0.12793	0.1277404	0.7972588
ENSG00000119537	KDSR	18	61014851	G	T	0.208748	0.0105088	0.0174459	0.5999997	0.438035	0.00996689	0	0.0239908	0.0398314	0.5469688	0.06215231
ENSG00000119541	VPS4B	18	61073069	T	G	0.0188867	-0.0872067	0.046938	0.0649995	0.397645	0.0260747	1.64E-52	-0.219308	0.118913	0.06514287	0.1295437
ENSG00000119559	C19orf25	19	1470348	T	C	0.0506958	-0.0107975	0.0317624	0.5500004	0.449822	0.022994	3.22E-85	-0.0240039	0.0706217	0.7339359	0.7487447
ENSG00000119599	DCAF4	14	73409725	A	G	0.184891	0.0222972	0.0179742	0.2099999	0.759759	0.00891363	0	0.0293477	0.0236603	0.2148349	0.9277166
ENSG00000119608	PROX2	14	75325136	T	C	0.156064	0.0461255	0.020318	0.01400006	0.136881	0.0146963	1.23E-20	0.336974	0.152781	0.02741143	0.411661
ENSG00000119616	FCF1	14	75191620	G	A	0.227634	0.0365889	0.0169794	0.02300011	-0.102829	0.0094934	2.44E-27	-0.355824	0.168359	0.03455969	0.4661073
ENSG00000119630	PGF	14	75415512	C	T	0.170974	0.0365319	0.0191034	0.04600023	0.187789	0.0106927	4.78E-69	0.194537	0.102329	0.05729054	0.30837
ENSG00000119632	IFI27L2	14	94595353	C	T	0.16501	-0.00691123	0.0173589	0.5700002	-0.49073	0.0109014	0	0.0140836	0.035375	0.6905403	0.06795967
ENSG00000119636	BBOF1	14	74517811	T	C	0.154076	-0.00449329	0.0205671	0.7099994	0.196668	0.0126917	3.70E-54	-0.0228471	0.104588	0.82708	0.3764975
ENSG00000119638	NEK9	14	75571434	A	G	0.0477137	0.0604942	0.0302544	0.02999991	0.117364	0.0180766	8.44E-11	0.515441	0.26973	0.05601123	0.4091198
ENSG00000119640	ACYP1	14	75528055	G	A	0.508946	0.0171789	0.0141885	0.2099999	0.091778	0.00794808	7.63E-31	0.187179	0.155443	0.2285274	0.1539884
ENSG00000119655	NPC2	14	74951887	T	A	0.456262	0.0210652	0.0143387	0.14	-0.282857	0.00788668	1.07E-281	-0.0744731	0.050735	0.1421357	0.573601
ENSG00000119669	IRF2BPL	14	77492961	T	C	0.0884692	-0.00485419	0.0257044	0.9199999	-0.952219	0.0174815	0	0.00509776	0.0269944	0.8502139	0.8000241
ENSG00000119673	ACOT2	14	74038340	T	C	0.393638	-0.00131542	0.0146881	0.91	0.227355	0.00950091	1.50E-126	-0.00578575	0.0646047	0.9286399	0.587717
ENSG00000119681	LTBP2	14	75022089	G	A	0.5	-0.00480571	0.0141934	0.6600001	0.143825	0.0086646	7.06E-62	-0.0334135	0.0987055	0.7349734	0.7962551
ENSG00000119684	MLH3	14	75499351	C	T	0.452286	-0.0147632	0.0142211	0.32	-0.490795	0.00747977	0	0.0300802	0.0289793	0.2992749	0.4532031
ENSG00000119685	TTLL5	14	76260694	T	C	0.310139	0.0150535	0.0153611	0.3100002	-0.311989	0.00844592	1.07E-298	-0.0482501	0.0492534	0.3272684	0.3845489
ENSG00000119686	FLVCR2	14	76087258	A	C	0.255467	-0.00792616	0.0159356	0.4899999	0.389463	0.00894681	0	-0.0203515	0.0409196	0.6189391	0.5292579
ENSG00000119688	ABCD4	14	74760942	G	A	0.50497	0.00272075	0.0141831	0.9199999	0.0572624	0.00815355	2.17E-12	0.0475137	0.247778	0.8479311	0.9095826
ENSG00000119689	DLST	14	75359521	A	G	0.00596421	-0.15731	0.100055	0.09099971	1.05073	0.0928815	1.14E-29	-0.149715	0.0961394	0.119407	0.562006
ENSG00000119698	PPP4R4	14	94679268	A	T	0.33002	-0.00212836	0.0147703	0.84	0.289332	0.0083602	1.90E-262	-0.00735611	0.0510501	0.8854246	0.1800749
ENSG00000119699	TGFB3	14	76436888	G	A	0.0616302	0.0290829	0.0304265	0.3100002	-0.445802	0.0174029	9.97E-145	-0.0652373	0.0682986	0.3394885	0.3180527
ENSG00000119707	RBM25	14	73556633	G	A	0.054672	-0.00985143	0.0287781	0.7700005	-0.216655	0.017772	3.48E-34	0.0454707	0.132882	0.7322087	0.5904465
ENSG00000119711	ALDH6A1	14	74537374	A	G	0.190855	0.0251777	0.0182846	0.1900002	0.0962227	0.0114764	5.10E-17	0.261661	0.192569	0.1742142	0.7937264
ENSG00000119714	GPR68	14	91709572	A	C	0.180915	0.000259884	0.0179982	0.99	-0.232479	0.00992399	2.32E-121	-0.00111788	0.0774185	0.9884794	0.3058733

ENSG00000119718	EIF2B2	14	75472953	T	C	0.461233	-0.0160112	0.0142049	0.29	-0.590569	0.00723517	0	0.0271115	0.0240552	0.2597201	0.3050878
ENSG00000119720	NRDE2	14	90770530	C	T	0.39662	0.0034923	0.0144328	0.64	-0.377262	0.00876647	0	-0.00925695	0.0382573	0.8088066	0.7366452
ENSG00000119723	COQ6	14	74423501	C	T	0.129225	-0.0176792	0.0198487	0.4899999	-0.400219	0.0109908	2.57E-290	0.0441738	0.0496095	0.3732341	0.7842993
ENSG00000119725	ZNF410	14	74376267	G	A	0.102386	0.0283876	0.0240163	0.2999998	0.161473	0.012639	2.24E-37	0.175804	0.149368	0.2392006	0.2131037
ENSG00000119729	RHOQ	2	46789602	T	C	0.227634	0.0116859	0.0166051	0.7899998	0.169577	0.0105574	4.68E-58	0.0689119	0.0980144	0.4820064	0.3848698
ENSG00000119737	GPR75	2	54083588	G	A	0.184891	-0.00646291	0.018855	0.7600007	-0.0879942	0.0114296	1.37E-14	0.073447	0.214488	0.7320275	0.6903793
ENSG00000119760	SUPT7L	2	27880177	G	C	0.225646	0.0171861	0.0172148	0.2300001	0.093579	0.00962153	2.34E-22	0.183653	0.184927	0.320654	0.8780873
ENSG00000119771	KLHL29	2	23769784	A	G	0.440358	0.0213347	0.0144355	0.1499999	-0.0862873	0.00814816	3.32E-26	-0.247252	0.168917	0.1432632	0.8898097
ENSG00000119772	DNMT3A	2	25510652	A	T	0.329026	-0.00219472	0.0157228	0.8200001	-0.116509	0.00883755	1.09E-39	0.0188373	0.134957	0.8889914	0.5045127
ENSG00000119777	TMEM214	2	27260170	A	G	0.380716	-0.00625414	0.0146542	0.5199996	-0.0481395	0.00816671	3.76E-09	0.129917	0.305208	0.6703504	0.9840339
ENSG00000119782	FKBP1B	2	24279561	C	T	0.140159	0.00278291	0.0202458	0.8600001	0.250209	0.0205507	4.21E-34	0.0111223	0.0809207	0.8906772	0.5291665
ENSG00000119787	ATL2	2	38563224	A	C	0.0626243	-0.041359	0.0273822	0.14	0.215833	0.015046	1.15E-46	-0.191625	0.127569	0.1330636	0.7988711
ENSG00000119801	YPEL5	2	30376603	A	G	0.0218688	0.0780492	0.068669	0.35	0.298681	0.0329721	1.32E-19	0.261313	0.23171	0.2594223	0.637668
ENSG00000119812	FAM98A	2	33816587	C	T	0.275348	-0.0092987	0.0160753	0.64	-0.061836	0.00905015	8.34E-12	0.150377	0.260896	0.5643553	0.08207972
ENSG00000119820	YIPF4	2	32519990	C	T	0.535785	0.0130242	0.0142183	0.6600001	0.0660176	0.00797449	1.25E-16	0.197284	0.216686	0.3625799	0.9311367
ENSG00000119844	AFTPH	2	64785802	G	A	0.337972	0.0212718	0.0151224	0.1499999	-0.108831	0.00929865	1.22E-31	-0.195456	0.139952	0.1625364	0.8492842
ENSG00000119862	LGALSL	2	64684809	T	G	0.128231	-0.0106045	0.0205879	0.7800007	0.525403	0.0125159	0	-0.0201836	0.0391879	0.6065213	0.1798039
ENSG00000119865	CNRIP1	2	68529243	G	A	0.419483	0.000427155	0.0145533	0.8499999	0.181656	0.00876109	1.70E-95	0.00235145	0.0801147	0.9765846	0.08171144
ENSG00000119866	BCL11A	2	60729502	A	G	0.486083	-0.0161437	0.0142858	0.4600002	-0.0512107	0.00803003	1.80E-10	0.315241	0.283307	0.2658294	0.7426078
ENSG00000119878	CRIPT	2	46848218	T	C	0.422465	0.0220721	0.0143074	0.1299999	0.530644	0.00744593	0	0.0415949	0.0269686	0.1229901	0.2010782
ENSG00000119899	SLC17A5	6	74333490	T	C	0.0725646	0.0172402	0.0285489	0.7700005	0.228018	0.0171711	3.06E-40	0.0756088	0.125334	0.5463359	0.416156
ENSG00000119900	OGFRL1	6	72005239	C	T	0.156064	-0.00480571	0.0194176	0.9299999	-0.42369	0.0110649	0	0.0113425	0.0458307	0.8045311	0.8487576
ENSG00000119906	SLF2	10	102698806	T	C	0.513917	-0.0147096	0.0142078	0.3800004	0.201147	0.00789095	2.49E-143	-0.0731288	0.0706923	0.3009185	0.3467046
ENSG00000119912	IDE	10	94272637	A	C	0.0159046	0.0296279	0.0580335	0.6100002	0.631689	0.0747791	2.98E-17	0.0469027	0.092038	0.6103309	0.7590733
ENSG00000119917	IFIT3	10	91094189	T	C	0.473161	-0.00233831	0.0142337	0.9400001	0.105302	0.00793758	3.64E-40	-0.0222058	0.135181	0.8695208	0.8892504
ENSG00000119922	IFIT2	10	91065372	G	A	0.0437376	-0.00583677	0.0453989	0.8200001	0.279126	0.0248302	2.55E-29	-0.0209109	0.162657	0.8977075	0.8641981
ENSG00000119927	GPAM	10	113942379	G	T	0.05666	-0.044102	0.0326372	0.1299999	0.175499	0.0183025	8.91E-22	-0.251295	0.187805	0.1808772	0.9656776
ENSG00000119929	CUTC	10	101489103	A	G	0.0248509	-0.0687096	0.0400388	0.1	-0.144979	0.0265142	4.55E-08	0.473926	0.28945	0.1015607	0.8858209
ENSG00000119943	PYROXD2	10	100159131	G	A	0.319085	-0.00417684	0.0151662	0.6700003	-0.122742	0.00833813	4.75E-49	0.0340293	0.123583	0.7830425	0.1460161
ENSG00000119950	MXI1	10	112007243	T	C	0.484095	0.00888483	0.0142299	0.5700002	0.244253	0.007865	9.56E-212	0.0363755	0.0582706	0.5324625	0.8882488
ENSG00000119953	SMNDC1	10	112058753	C	T	0.451292	0.00452668	0.0142942	0.7899998	-0.0847511	0.00800082	3.22E-26	-0.0534114	0.168736	0.7515941	0.9397393
ENSG00000119965	C10orf88	10	124702169	T	C	0.398608	0.00754119	0.0143538	0.5500004	0.357772	0.00778633	0	0.0210782	0.0401226	0.5993434	0.4222957
ENSG00000119969	HELLS	10	96339604	A	G	0.442346	0.0108541	0.0142715	0.4	0.333803	0.00778996	0	0.0325165	0.042761	0.4470012	0.4015868
ENSG00000119977	TCTN3	10	97438526	A	G	0.0536779	0.00403899	0.0336194	0.9699999	0.697481	0.0192449	1.31E-287	0.00579082	0.0482014	0.9043738	0.9299962
ENSG00000119979	DENND10	10	120880547	T	C	0.402584	0.00133276	0.0143616	0.7700005	-0.107114	0.0140297	2.26E-14	-0.0124424	0.134087	0.9260677	0.8701336
ENSG00000119986	AVPI1	10	99442130	A	G	0.0357853	-0.0400951	0.0313262	0.2999998	-0.399215	0.0208137	5.40E-82	0.100435	0.0786439	0.2015733	0.2504506
ENSG00000120008	WDR11	10	122639865	A	C	0.333002	0.00420376	0.0149904	0.7499995	-0.657991	0.00816478	0	-0.00638878	0.0227822	0.7791488	0.5782028
ENSG00000120029	ARMH3	10	103710653	A	G	0.0626243	0.0124639	0.0320646	0.56	0.323155	0.0183415	1.77E-69	0.0385694	0.0992477	0.697559	0.03042847
ENSG00000120049	KCNIP2	10	103594704	T	G	0.467197	-0.00188768	0.0142546	0.8499999	0.0664493	0.00802121	1.19E-16	-0.0284078	0.214546	0.8946607	0.7533985
ENSG00000120051	CFAP58	10	106164185	C	T	0.27833	-0.0210393	0.0158855	0.1100001	0.152711	0.00973486	1.86E-55	-0.137772	0.104393	0.1869222	0.3134385
ENSG00000120053	GOT1	10	101173504	A	G	0.12326	-0.0135091	0.0225144	0.4100001	0.165834	0.0125101	4.17E-40	-0.0814615	0.135904	0.5489017	0.8438338
ENSG00000120057	SFRP5	10	99529108	A	G	0.147117	-0.00388144	0.0184509	0.9	0.18441	0.0151104	2.95E-34	-0.0210479	0.100069	0.8334065	0.880544
ENSG00000120063	GNA13	17	63029895	G	A	0.127237	-0.00958238	0.0242223	0.5400003	-0.370981	0.0126845	4.93E-188	0.0258298	0.0652985	0.6924257	0.8131263
ENSG00000120071	KANSL1	17	44205007	G	C	0.166004	-0.00777327	0.0193212	0.91	-0.44087	0.0318755	1.66E-43	0.0176317	0.0438437	0.687575	0.6269755
ENSG00000120087	NA	17	46686486	C	A	0.159046	0.00371503	0.0183782	0.99	-0.131673	0.0109957	4.80E-33	-0.028214	0.139594	0.8398272	0.20959
ENSG00000120093	HOXB3	17	46654253	C	T	0.515905	-0.00625665	0.0142062	0.89	0.286604	0.00779543	6.55E-296	-0.0218303	0.049571	0.6596581	0.2915791
ENSG00000120129	DUSP1	5	172196645	C	T	0.157058	0.00405689	0.0197417	0.9599999	0.107787	0.0105017	1.03E-24	0.0376379	0.183191	0.8372148	0.9111514
ENSG00000120137	PANK3	5	167991052	A	G	0.182903	-0.0302748	0.0182255	0.14	0.114344	0.0111719	1.38E-24	-0.26477	0.161478	0.1010741	0.5399784
ENSG00000120156	TEK	9	27169656	G	A	0.233598	0.00788612	0.0163496	0.4	0.522502	0.0089185	0	0.015093	0.031292	0.6295737	0.09217644
ENSG00000120158	RCL1	9	4826966	C	T	0.362823	-0.00759787	0.0148587	0.56	0.144349	0.00837709	1.54E-66	-0.0526354	0.102981	0.6092695	0.6192278

ENSG00000120159	CAAP1	9	26866742	T	C	0.385686	-0.00619196	0.0145741	0.6600001	-0.0735032	0.00906709	5.21E-16	0.0842406	0.19855	0.6713629	0.1582668
ENSG00000120162	MOB3B	9	27427493	G	A	0.316103	-0.0111789	0.0150879	0.4500005	-0.0935605	0.00940175	2.49E-23	0.119483	0.16171	0.459984	0.9718641
ENSG00000120217	CD274	9	5460534	A	C	0.10338	-0.0249574	0.0256648	0.3700002	0.299232	0.0141641	4.59E-99	-0.0834049	0.0858598	0.3313449	0.01592115
ENSG00000120253	NUP43	6	150058126	T	G	0.359841	-0.0198505	0.0148954	0.1499999	-0.6216	0.00749942	0	0.0319345	0.0239661	0.1827	0.03408957
ENSG00000120254	MTHFD1L	6	151304854	G	A	0.0904573	-0.0166864	0.0255806	0.5300002	0.452701	0.0249065	8.00E-74	-0.0368596	0.0565429	0.514474	0.7046578
ENSG00000120262	CCDC170	6	151878746	A	G	0.0477137	-0.0221814	0.031871	0.3800004	0.488362	0.020034	3.03E-131	-0.04542	0.0652876	0.4866224	0.9519023
ENSG00000120265	PCMT1	6	150101567	G	A	0.366799	-0.0205038	0.0148372	0.1299999	0.252954	0.00808601	7.97E-215	-0.0810574	0.0587129	0.1674106	0.07560192
ENSG00000120278	PLEKHG1	6	151042899	T	G	0.244533	-0.00534893	0.0166101	0.7800007	0.0670709	0.0104733	1.51E-10	-0.0797503	0.247963	0.7477388	0.9665348
ENSG00000120279	MYCT1	6	153032366	A	G	0.201789	0.0148021	0.0177911	0.4	0.079643	0.0110171	4.87E-13	0.185856	0.22486	0.4084987	0.7880097
ENSG00000120306	CYSTM1	5	139607932	T	C	0.217694	-0.00410985	0.0174467	0.7099994	-0.16654	0.0105206	1.93E-56	0.0246778	0.104771	0.8137897	0.2424595
ENSG00000120314	WDR55	5	140048985	T	C	0.43837	-0.00629996	0.014297	0.5199996	0.376351	0.00767422	0	-0.0167396	0.03799	0.6594799	0.8819529
ENSG00000120318	ARAP3	5	141047378	T	C	0.195825	-0.023259	0.0174891	0.1499999	-0.466704	0.00919584	0	0.0498367	0.0374865	0.183697	0.2268171
ENSG00000120333	MRPS14	1	174986243	T	C	0.507952	0.00669406	0.014213	0.5099998	0.055959	0.00795437	1.99E-12	0.119624	0.254558	0.6384054	0.8589454
ENSG00000120370	GORAB	1	170511928	T	C	0.104374	0.010662	0.0229497	0.64	-0.54902	0.0119558	0	-0.0194201	0.0418033	0.6422484	0.4242007
ENSG00000120437	ACAT2	6	160190752	C	T	0.457256	-0.0237137	0.0143624	0.09699961	0.2268	0.00804534	7.71E-175	-0.104558	0.0634349	0.09929703	0.1648292
ENSG00000120438	TCP1	6	160205155	C	T	0.242545	-0.0270208	0.0167679	0.1199999	0.624256	0.00940335	0	-0.0432848	0.0268685	0.1071829	0.2271534
ENSG00000120440	TTLL2	6	167755782	C	G	0.0954274	0.0210169	0.0225971	0.28	0.224472	0.0135541	1.33E-61	0.0936282	0.100826	0.3530926	0.6495886
ENSG00000120451	SNX19	11	130765867	C	T	0.198807	0.00841498	0.0177507	0.5099998	0.771896	0.00895569	0	0.0109017	0.0229966	0.6354589	0.61107
ENSG00000120519	SLC10A7	4	147309125	G	A	0.440358	0.0148385	0.0142491	0.1900002	0.301074	0.00780915	0	0.0492852	0.0473448	0.2978828	0.04899089
ENSG00000120533	ENY2	8	110352367	T	A	0.460239	0.0218344	0.0142989	0.2	0.144594	0.0118531	3.15E-34	0.151005	0.0996617	0.1297283	0.1860329
ENSG00000120539	MASTL	10	27459800	C	A	0.397614	0.00257732	0.0148065	0.9299999	-0.690736	0.00739099	0	-0.00373126	0.0214359	0.8618132	0.8854346
ENSG00000120594	PLXDC2	10	20337227	T	C	0.224652	0.0256117	0.0174064	0.1499999	0.133812	0.00961582	5.08E-44	0.191401	0.130806	0.143402	0.4168003
ENSG00000120616	EPC1	10	32612202	T	C	0.153082	-0.0054099	0.018938	0.9599999	-0.115656	0.0105704	7.31E-28	0.046776	0.163801	0.7752102	0.4064663
ENSG00000120645	IQSEC3	12	231778	C	G	0.338966	0.0182949	0.0148462	0.2200002	0.580201	0.0143629	0	0.031532	0.0255999	0.2180529	0.5882372
ENSG00000120647	CCDC77	12	525125	T	G	0.186879	0.00351381	0.0171493	0.4500005	-0.655271	0.00938966	0	-0.00536238	0.0261714	0.8376547	0.9925225
ENSG00000120662	MTRF1	13	41814123	T	G	0.277336	-0.0101131	0.0161746	0.32	-0.217027	0.00927876	5.45E-121	0.0465985	0.0745548	0.5319563	0.4143037
ENSG00000120675	DNAJC15	13	43640192	T	G	0.253479	0.0156944	0.0167748	0.4700002	-0.924616	0.0114502	0	-0.016974	0.0181437	0.3495147	0.9980196
ENSG00000120685	PROSER1	13	39598127	T	C	0.162028	0.0235446	0.0203732	0.2399999	0.196256	0.012465	7.48E-56	0.119969	0.104089	0.2490891	0.6968997
ENSG00000120688	WBP4	13	41646773	A	G	0.0755467	0.00115774	0.027072	0.98	0.285152	0.0166491	9.30E-66	0.00406007	0.094939	0.9658888	0.1097992
ENSG00000120690	ELF1	13	41570816	C	T	0.432406	-0.00747109	0.0146507	0.5199996	0.098673	0.00828516	1.05E-32	-0.0757157	0.148613	0.6104152	0.2012788
ENSG00000120694	HSPH1	13	31723643	A	G	0.241551	0.0150488	0.0168547	0.4500005	0.216261	0.00918492	1.41E-122	0.0695863	0.0779929	0.3722785	0.06695864
ENSG00000120696	KBTBD7	13	41766335	T	C	0.16501	-0.00327704	0.0197109	0.8200001	0.259326	0.0110833	4.50E-121	-0.0126368	0.0760102	0.8679594	0.0301515
ENSG00000120697	ALG5	13	37549155	C	A	0.380716	0.0132005	0.0145515	0.29	-0.147004	0.00805446	2.02E-74	-0.0897971	0.0991095	0.3649153	0.07015545
ENSG00000120699	EXOSC8	13	37578351	T	C	0.482107	-0.0269906	0.0142458	0.03599979	0.375905	0.0114756	2.42E-235	-0.0718016	0.0379606	0.05856105	0.2638196
ENSG00000120705	ETF1	5	137860386	T	C	0.44831	-0.00790206	0.0145676	0.58	-0.103475	0.00804851	7.92E-38	0.076367	0.140909	0.587847	0.7967024
ENSG00000120708	TGFBI	5	135382045	A	G	0.156064	-0.0238174	0.0201012	0.2099999	-0.609241	0.0102528	0	0.0390935	0.0330004	0.2361602	0.1207518
ENSG00000120709	FAM53C	5	137676520	T	C	0.261431	0.00709712	0.0161835	0.4299995	0.128517	0.00923682	5.24E-44	0.055223	0.125987	0.6611523	0.9757026
ENSG00000120725	SIL1	5	138455827	T	C	0.261431	0.0149597	0.0157142	0.32	0.157137	0.00860374	1.61E-74	0.0952016	0.100139	0.341759	0.7579933
ENSG00000120733	KDM3B	5	137730501	T	C	0.119284	0.0248552	0.0214668	0.2999998	-0.461886	0.0138642	2.35E-243	-0.0538125	0.0465045	0.2472131	0.3667209
ENSG00000120742	SERP1	3	150290398	G	A	0.251491	-0.0386578	0.0166361	0.01499996	0.134997	0.0091961	8.68E-49	-0.286361	0.124768	0.02172438	0.6720011
ENSG00000120756	PLS1	3	142373867	A	G	0.0238569	-0.0343156	0.0502791	0.7099994	0.575148	0.0331245	1.57E-67	-0.059664	0.0874869	0.4952544	0.8718581
ENSG00000120784	ZFP30	19	38143944	A	G	0.383698	-0.000706524	0.0146656	0.81	-0.313895	0.0079045	0	0.00225083	0.0467214	0.9615763	0.5007183
ENSG00000120798	NR2C1	12	95441574	G	A	0.147117	0.00144097	0.0183289	0.8	0.204007	0.0106213	3.21E-82	0.00706334	0.0898453	0.9373375	0.8547348
ENSG00000120800	UTP20	12	101727140	G	A	0.208748	0.0165949	0.0180059	0.3599996	0.0811843	0.0101844	1.57E-15	0.20441	0.223268	0.3599093	0.3704359
ENSG00000120802	TMPO	12	98926723	T	G	0.266402	-0.0153725	0.0160522	0.4199997	0.095195	0.00906326	8.33E-26	-0.161484	0.169324	0.340235	0.08532754
ENSG00000120805	ARL1	12	101794248	C	A	0.115308	-0.0332912	0.0228052	0.2099999	0.265475	0.0125872	9.66E-99	-0.125402	0.0861089	0.1453033	0.7543715
ENSG00000120832	MTERF2	12	107376006	A	G	0.0318091	-0.0212264	0.0399695	0.64	-0.320804	0.0252781	6.63E-37	0.0661662	0.124701	0.5956956	0.5356932
ENSG00000120833	SOCS2	12	93970426	G	C	0.502982	-0.0277423	0.0142663	0.025	0.247772	0.0078651	7.98E-218	-0.111967	0.057688	0.05226926	0.2758873
ENSG00000120837	NFYB	12	104521461	A	T	0.228628	-0.00890763	0.0172385	0.81	-0.0521748	0.0093789	2.65E-08	0.170727	0.331821	0.6068927	0.3973757
ENSG00000120860	WASHC3	12	102431316	T	C	0.215706	0.0159	0.0172438	0.17	-0.643981	0.0148544	0	-0.0246902	0.0267829	0.3566005	0.9445248

ENSG00000120868	APAF1	12	99084061	T	A	0.182903	0.00641765	0.0185537	0.9599999	0.344441	0.0105049	8.59E-236	0.0186321	0.0538691	0.7294354	0.1302833
ENSG00000120875	DUSP4	8	29199383	G	T	0.128231	-0.00243615	0.0199612	0.7099994	-0.0830155	0.0114356	3.89E-13	0.0293457	0.240485	0.9028776	0.7139691
ENSG00000120885	CLU	8	27463491	G	C	0.271372	0.00937911	0.0160356	0.5099998	0.0775688	0.00924121	4.71E-17	0.120913	0.207229	0.5595715	0.8501433
ENSG00000120889	TNFRSF10B	8	22902169	G	A	0.230616	-0.0200603	0.0173322	0.1800002	0.48875	0.00965733	0	-0.0410441	0.0354716	0.2472323	0.4437275
ENSG00000120896	SORBS3	8	22417900	C	T	0.201789	0.0188165	0.0187278	0.1800002	-0.128302	0.0129112	2.87E-23	-0.146658	0.146711	0.3174849	0.09786994
ENSG00000120899	PTK2B	8	27242951	A	G	0.34493	-0.00287928	0.0147215	0.9199999	0.436579	0.00791937	0	-0.0065951	0.0337203	0.8449373	0.9235762
ENSG00000120903	CHRNA2	8	27327339	C	T	0.515905	0.0157624	0.0143501	0.5400003	0.0931448	0.00793046	7.47E-32	0.169225	0.154734	0.2741106	0.462079
ENSG00000120910	PPP3CC	8	22348492	G	A	0.0526839	-0.0363697	0.0317799	0.2200002	-0.185381	0.0158809	1.75E-31	0.196189	0.172252	0.2547181	0.6629819
ENSG00000120913	PDLIM2	8	22445665	T	C	0.0904573	-0.0515395	0.0242775	0.02800013	-0.172238	0.0148522	4.28E-31	0.299235	0.143296	0.036777	0.3065044
ENSG00000120915	EPHX2	8	27375688	C	A	0.236581	-0.010189	0.0160284	0.7899998	0.537341	0.00851975	0	-0.0189619	0.0298306	0.5250034	0.6334795
ENSG00000120925	RNF170	8	42728606	T	G	0.00795229	0.032585	0.0603795	0.5300002	0.509081	0.0556202	5.55E-20	0.0640075	0.118811	0.5900702	0.9875648
ENSG00000120942	UBIAD1	1	11344684	A	G	0.0208748	-0.00796033	0.0533299	0.7899998	-0.182437	0.0277175	4.64E-11	0.0436334	0.292395	0.8813742	0.6602223
ENSG00000120949	TNFRSF8	1	12163849	A	G	0.505964	0.00642157	0.0142872	0.58	0.0713679	0.00795877	3.04E-19	0.0899784	0.200442	0.6535041	0.2901187
ENSG00000120963	ZNF706	8	102204263	A	G	0.0337972	0.0348705	0.0399875	0.3100002	-0.127789	0.0229795	2.68E-08	-0.272876	0.316742	0.3889588	0.5871432
ENSG00000120992	LYPLA1	8	54986757	C	T	0.0238569	-0.0372562	0.0477733	0.4600002	-0.359759	0.0584518	7.52E-10	0.103559	0.133854	0.439127	0.5294465
ENSG00000121039	RDH10	8	74222181	A	G	0.178926	-0.00632942	0.0179115	0.8499999	-0.122686	0.0103438	1.89E-32	0.0515903	0.146059	0.7239272	0.1662826
ENSG00000121057	AKAP1	17	55180581	A	G	0.485089	0.00605375	0.0142444	0.5999997	0.0999103	0.00796276	4.12E-36	0.0605918	0.142654	0.6710204	0.4924589
ENSG00000121060	TRIM25	17	54978334	T	G	0.360835	-0.014026	0.0148587	0.4100001	-0.0466926	0.00822633	1.38E-08	0.30039	0.322594	0.3517659	0.9170976
ENSG00000121064	SCPEP1	17	55069797	G	T	0.482107	0.00999187	0.0143601	0.6899999	0.15003	0.00794358	1.46E-79	0.066599	0.0957796	0.4868447	0.5769475
ENSG00000121067	SPOP	17	47715921	T	C	0.0695825	-0.0271915	0.0257215	0.2599998	0.0821001	0.0149164	3.71E-08	-0.331199	0.319021	0.2991888	0.120498
ENSG00000121073	SLC35B1	17	47782340	T	G	0.0954274	-0.0216643	0.0247097	0.4100001	0.106681	0.0149493	9.59E-13	-0.203076	0.233364	0.3841858	0.08713481
ENSG00000121083	NA	17	56164684	A	G	0.321074	0.0262044	0.015216	0.07900053	-0.25175	0.00911205	5.09E-168	-0.104089	0.0605581	0.08564625	0.3077933
ENSG00000121101	TEX14	17	56701727	G	A	0.44334	-0.000119245	0.0142865	0.91	-0.109213	0.00800837	2.40E-42	0.00109185	0.130813	0.9933404	0.9638799
ENSG00000121104	FAM117A	17	47827118	C	T	0.055666	-0.0374719	0.0311758	0.1800002	0.112398	0.0160867	2.81E-12	-0.333387	0.281445	0.2361934	0.01477826
ENSG00000121210	TMEM131L	4	154472680	G	A	0.0308151	0.0475779	0.0454192	0.2099999	0.609205	0.025842	7.09E-123	0.0780983	0.0746284	0.2953322	0.2680715
ENSG00000121211	MND1	4	154301035	A	G	0.291252	0.0202788	0.0157811	0.25	-0.0588655	0.0089058	3.85E-11	-0.344494	0.273106	0.2071686	0.9366976
ENSG00000121236	TRIM6	11	5625763	T	C	0.255467	0.00867872	0.0163698	0.5400003	0.227125	0.0101579	9.78E-111	0.0382113	0.0720943	0.5960998	0.9203548
ENSG00000121274	TENT4B	16	50228025	T	C	0.157058	0.00750661	0.0192632	0.6499995	0.254126	0.0105218	7.05E-129	0.0295389	0.0758115	0.6968058	0.2407338
ENSG00000121281	ADCY7	16	50316047	T	C	0.157058	0.00750661	0.0192632	0.6499995	0.559315	0.0101426	0	0.0134211	0.0344416	0.6967755	0.1712488
ENSG00000121289	CEP89	19	33416402	C	T	0.33996	0.0190104	0.0146495	0.1499999	-0.225347	0.0100563	3.24E-111	-0.0843604	0.0651174	0.1951437	0.8032797
ENSG00000121297	TSHZ3	19	31803152	T	C	0.143141	0.00310754	0.0211356	0.6999999	0.273546	0.0125753	6.53E-105	0.0113602	0.0772669	0.883112	0.1455863
ENSG00000121310	ECHDC2	1	53377270	C	T	0.349901	0.029817	0.0148754	0.08300036	-0.56109	0.00788291	0	-0.0531412	0.0265221	0.04510693	0.3123689
ENSG00000121316	PLBD1	12	14688939	T	C	0.315109	0.0390927	0.0154829	0.004799986	-0.238613	0.00950421	4.28E-139	-0.163833	0.0652145	0.01199734	0.3636327
ENSG00000121350	PYROXD1	12	21606924	C	A	0.44831	0.0156476	0.0141809	0.2099999	0.281666	0.0107896	3.18E-150	0.0555538	0.0503915	0.2702689	0.07589095
ENSG00000121380	BCL2L14	12	12283398	T	C	0.251491	-0.000920758	0.0159176	1	0.226874	0.0089185	9.44E-143	-0.00405845	0.0701607	0.953872	0.8044836
ENSG00000121390	PSPC1	13	20303019	T	C	0.236581	-0.0107353	0.0174083	0.2999998	-0.189052	0.00992494	6.80E-81	0.0567848	0.0921302	0.5376608	0.5326737
ENSG00000121413	ZSCAN18	19	58612499	G	A	0.227634	-0.0315027	0.017194	0.07799917	-0.323994	0.00936039	1.59E-262	0.0972324	0.0531432	0.06730528	0.3139356
ENSG00000121417	ZNF211	19	58147954	G	C	0.121272	0.0442601	0.0225645	0.05099998	0.533275	0.0128156	0	0.0829968	0.0423601	0.05007568	0.1784531
ENSG00000121454	LHX4	1	180224400	C	A	0.0705765	-0.00768527	0.0253511	0.7800007	-0.113889	0.014408	2.69E-15	0.0674802	0.222758	0.7619427	0.5334009
ENSG00000121481	RNF2	1	185043118	A	T	0.0606362	0.0464662	0.0305559	0.07499978	-0.212209	0.0164406	4.08E-38	-0.218965	0.144986	0.1309797	0.5365899
ENSG00000121486	TRMT1L	1	185106712	C	A	0.0626243	0.0447457	0.0305266	0.08500021	-0.286272	0.0180385	1.02E-56	-0.156305	0.107089	0.1444052	0.3263063
ENSG00000121542	SEC22A	3	122956875	G	A	0.411531	-0.00950906	0.0143728	0.5300002	-0.161532	0.00801331	2.29E-90	0.0588678	0.0890258	0.5084552	0.205365
ENSG00000121552	CSTA	3	122052455	T	C	0.156064	0.0136943	0.0210295	0.6100002	-0.538016	0.0112828	0	-0.0254533	0.0390908	0.51496	0.9407234
ENSG00000121570	DPPA4	3	109050703	A	G	0.0666004	0.0257507	0.028338	0.33	0.37195	0.0149368	7.15E-137	0.0692317	0.0762385	0.3638285	0.9601711
ENSG00000121577	POPDC2	3	119369737	C	G	0.4334	-0.00755005	0.0144675	0.56	0.347793	0.00789641	0	-0.0217085	0.041601	0.6017914	0.13102
ENSG00000121578	B4GALT4	3	118945264	A	G	0.162028	0.0148749	0.0177148	0.32	0.62646	0.00913398	0	0.0237444	0.0282798	0.4011188	0.08967058
ENSG00000121644	DESI2	1	244844286	T	C	0.511928	-0.0156867	0.014356	0.29	0.0663456	0.0088733	7.60E-14	-0.236439	0.21868	0.2796045	0.6435676
ENSG00000121653	MAPK8IP1	11	45917609	G	C	0.237575	-0.0179882	0.0164098	0.33	0.585398	0.0127231	0	-0.0307282	0.0280398	0.2731333	0.5984519
ENSG00000121671	CRY2	11	45886733	C	T	0.251491	-0.00679964	0.0176402	0.7199992	0.42026	0.010353	0	-0.0161796	0.0419764	0.6999075	0.1223093
ENSG00000121680	PEX16	11	45935791	A	G	0.346918	-0.00855431	0.0149405	0.5400003	-0.0536931	0.00852813	3.05E-10	0.159319	0.279405	0.5685383	0.7270038

ENSG00000121690	DEPDC7	11	33046269	C	T	0.341948	-0.00191956	0.0151679	0.91	0.242649	0.00828045	9.28E-189	-0.00791086	0.0625103	0.8992943	0.0416179
ENSG00000121691	CAT	11	34477040	G	T	0.349901	0.00322165	0.0147807	0.8800001	0.681117	0.0074574	0	0.00472995	0.0217008	0.8274582	0.07683182
ENSG00000121716	PILRB	7	99949578	G	A	0.177932	0.0364538	0.0181146	0.04700023	1.02405	0.00855318	0	0.0355978	0.0176918	0.04420785	0.4595966
ENSG00000121741	ZMYM2	13	20599389	C	G	0.186879	0.0106796	0.0209508	0.6899999	-0.0877096	0.0113472	1.08E-14	-0.121761	0.239384	0.6110033	0.8835976
ENSG00000121742	GJB6	13	20801322	T	C	0.311133	0.00511722	0.0155876	0.6600001	0.37352	0.00855452	0	0.0137	0.0417328	0.742701	0.906646
ENSG00000121743	GJA3	13	20723791	G	A	0.337972	0.0033096	0.0152275	0.8700001	0.138826	0.00863262	3.43E-58	0.0238399	0.109697	0.827956	0.2912482
ENSG00000121749	TBC1D15	12	72277058	T	C	0.0745527	-0.0237275	0.0238888	0.4199997	0.394819	0.0130872	6.15E-200	-0.0600971	0.0605385	0.3208513	0.2664918
ENSG00000121766	ZCCHC17	1	31803812	T	C	0.210736	0.0252375	0.0177101	0.1299999	-0.158587	0.00984032	1.97E-58	-0.15914	0.11211	0.1557545	0.7927207
ENSG00000121769	FABP3	1	31844084	G	T	0.210736	0.0245243	0.017723	0.14	-0.149714	0.016695	3.03E-19	-0.163808	0.11978	0.1714467	0.7386552
ENSG00000121775	TMEM39B	1	32553049	G	A	0.05666	0.00998041	0.0291217	0.7700005	-0.323109	0.0152433	1.02E-99	-0.0308887	0.0901415	0.7318474	0.5577124
ENSG00000121797	CCRL2	3	46449834	T	C	0.357853	-0.0100582	0.0147649	0.5199996	-0.139381	0.00903521	1.09E-53	0.0721634	0.106035	0.4961496	0.748081
ENSG00000121807	CCR2	3	46398822	A	G	0.215706	0.0245503	0.0168501	0.1100001	0.255641	0.00975214	1.85E-151	0.0960344	0.066015	0.145742	0.9587322
ENSG00000121848	NA	1	145650848	T	G	0.358847	0.0101346	0.0147135	0.4799997	0.144288	0.012249	4.97E-32	0.0702387	0.102147	0.4916912	0.2684176
ENSG00000121858	TNFSF10	3	172232297	A	G	0.309145	0.0024656	0.0151215	0.8	0.184241	0.00847975	1.14E-104	0.0133825	0.0820768	0.8704806	0.6806102
ENSG00000121864	ZNF639	3	179047051	G	A	0.5	-0.00286315	0.0142384	0.9599999	-0.0854897	0.00799881	1.16E-26	0.0334912	0.166581	0.8406588	0.8831636
ENSG00000121879	PIK3CA	3	178911891	G	T	0.419483	-0.0198763	0.0143411	0.1499999	-0.0930482	0.00803803	5.45E-31	0.213613	0.155226	0.1687785	0.7155667
ENSG00000121892	PDS5A	4	39902029	C	T	0.323062	0.00283598	0.0147028	0.99	0.167939	0.00826913	1.07E-91	0.0168869	0.0875522	0.8470542	0.5201658
ENSG00000121895	TMEM156	4	39001453	A	G	0.341948	-0.0377179	0.0150631	0.008	0.318304	0.0082356	0	-0.118496	0.0474221	0.01246306	0.4475445
ENSG00000121897	LIAS	4	39469946	A	G	0.406561	0.0130334	0.0144802	0.2700001	0.394543	0.00862173	0	0.0330342	0.0367083	0.368168	0.8190357
ENSG00000121931	LRIF1	1	111498254	T	C	0.256461	-0.00369334	0.016536	0.8700001	-0.368468	0.0100058	7.19E-297	0.0100235	0.0448785	0.8232652	0.6386446
ENSG00000121933	TMIGD3	1	112066277	C	T	0.442346	0.00643909	0.0142693	0.6999999	0.396325	0.00816946	0	0.016247	0.0360056	0.65182	0.8141657
ENSG00000121940	CLCC1	1	109489120	T	C	0.0526839	0.0525943	0.0343953	0.1100001	0.187097	0.0202154	2.14E-20	0.281107	0.186329	0.1313854	0.07124285
ENSG00000121957	GPSM2	1	109445508	T	C	0.222664	0.0278359	0.017505	0.1299999	0.123133	0.010699	1.19E-30	0.226063	0.143514	0.1152095	0.2870177
ENSG00000121964	GTDC1	2	144896728	T	G	0.0685885	-0.000502735	0.0263482	0.9699999	-0.144516	0.0152379	2.45E-21	0.00347874	0.18232	0.984777	0.3070001
ENSG00000121966	CXCR4	2	136873827	A	G	0.477137	-0.0332864	0.0143517	0.01199997	-0.053066	0.00798995	3.10E-11	0.627264	0.286466	0.02854889	0.9581899
ENSG00000121988	ZRANB3	2	136091646	A	G	0.131213	9.43E-05	0.0270664	0.35	-0.145907	0.0122341	8.64E-33	-0.000646613	0.185505	0.9972188	0.8849262
ENSG00000121989	ACVR2A	2	148645239	A	G	0.327038	-0.00429063	0.0154395	0.5199996	0.235626	0.00838003	5.97E-174	-0.0182095	0.0655285	0.7810999	0.04348322
ENSG00000122025	FLT3	13	28626070	C	T	0.152087	0.0100766	0.0207464	0.6300007	-0.0950571	0.0108528	1.97E-18	-0.106006	0.218587	0.6277058	0.4072007
ENSG00000122026	RPL21	13	27828137	A	C	0.054672	-0.0187136	0.0309761	0.4400003	-0.175166	0.0231261	3.61E-14	0.106833	0.1774	0.5470292	0.08261799
ENSG00000122033	MTIF3	13	28017257	T	C	0.447316	0.0185397	0.0142807	0.1800002	0.217774	0.011782	2.80E-76	0.0851328	0.0657373	0.195305	0.944005
ENSG00000122034	GTF3A	13	28004319	T	C	0.230616	0.00769361	0.0175929	0.89	-0.450558	0.00923887	0	-0.0170757	0.0390485	0.6618969	0.779182
ENSG00000122035	RASL11A	13	27846145	A	G	0.191849	0.0100518	0.0185395	0.5700002	-0.174378	0.0101528	4.06E-66	-0.0576437	0.106371	0.5878785	0.2104629
ENSG00000122042	UBL3	13	30381664	A	C	0.247515	0.000616725	0.0171184	0.8700001	0.166616	0.00950823	9.50E-69	0.00370146	0.102742	0.9712609	0.1829364
ENSG00000122043	NA	13	30517333	A	C	0.28827	0.0183965	0.0156461	0.17	0.11432	0.0109191	1.19E-25	0.16092	0.137722	0.2426281	0.9394683
ENSG00000122068	FYTTD1	3	197487683	G	A	0.0725646	-0.0107483	0.0278837	0.6499995	0.152547	0.0171494	5.83E-19	-0.0704589	0.182959	0.7001578	0.5969595
ENSG00000122085	MTERF4	2	242028108	A	G	0.0685885	-0.0420329	0.0285844	0.1	-0.412174	0.0179228	4.97E-117	0.101979	0.069492	0.1422436	0.06236106
ENSG00000122188	LAX1	1	203739832	A	G	0.331014	-0.000487628	0.0151108	0.8499999	-0.583182	0.0078335	0	0.000836151	0.025911	0.9742566	0.7215151
ENSG00000122203	KIAA1191	5	175781017	T	C	0.0576541	0.0594585	0.0321792	0.1199999	-0.391153	0.0170526	1.94E-116	-0.152008	0.0825341	0.06550953	0.81112
ENSG00000122218	COPA	1	160286126	T	A	0.4334	-0.0125274	0.0142724	0.35	0.196362	0.00793407	3.16E-135	-0.0637975	0.0727299	0.3803864	0.8989197
ENSG00000122223	CD244	1	160816321	C	T	0.196819	-0.00120098	0.018056	0.9199999	0.364938	0.009523	0	-0.00329091	0.0494769	0.9469685	0.3914303
ENSG00000122224	LY9	1	160781970	A	G	0.232604	0.024908	0.017235	0.1800002	-0.246702	0.00955734	6.37E-147	-0.100964	0.0699711	0.1490377	0.6629526
ENSG00000122257	RBBP6	16	24566599	A	G	0.238569	0.0016736	0.0169475	0.7899998	-0.147894	0.0093105	8.10E-57	-0.0113162	0.114595	0.9213367	0.01072943
ENSG00000122299	ZC3H7A	16	11867782	T	G	0.192843	0.000164173	0.0165037	0.6600001	0.156125	0.00993756	1.28E-55	0.00105155	0.105708	0.9920631	0.5282105
ENSG00000122335	SERAC1	6	158559924	A	G	0.430417	-0.0105137	0.0142972	0.3900004	0.377976	0.00780289	0	-0.0278158	0.0378301	0.4621671	0.8219781
ENSG00000122359	ANXA11	10	81937986	A	G	0.237575	0.0152776	0.0164415	0.29	-0.326838	0.00919865	1.63E-276	-0.0467436	0.0503219	0.352945	0.7219012
ENSG00000122376	SHLD2	10	88903089	A	C	0.107356	-0.00447556	0.0244376	0.84	0.469328	0.0300901	7.58E-55	-0.00953611	0.0520729	0.8546962	0.2079069
ENSG00000122378	PRXL2A	10	82180169	T	C	0.537773	-0.00565317	0.0141744	0.8499999	0.361086	0.00855787	0	-0.015656	0.0392566	0.6900319	0.662641
ENSG00000122386	ZNF205	16	3166539	G	C	0.415507	0.0171295	0.0146316	0.3800004	0.417943	0.00775079	0	0.0409853	0.0350169	0.2418225	0.9324042
ENSG00000122390	NAA60	16	3515287	G	A	0.274354	0.00396341	0.0160139	0.9699999	-0.313947	0.00989206	4.75E-221	-0.0126244	0.0510098	0.8045286	0.6575535
ENSG00000122417	ODF2L	1	86837266	C	T	0.34493	0.00184399	0.014911	0.7099994	0.585729	0.00785926	0	0.00314819	0.0254572	0.9015795	0.2246564

ENSG00000122420	PTGFR	1	78887501	C	T	0.249503	0.0213479	0.0157257	0.2	-0.170823	0.00899068	1.71E-80	-0.124971	0.0922932	0.1757157	0.9187644
ENSG00000122432	SPATA1	1	85001925	C	T	0.228628	0.000406807	0.0162861	0.98	-0.123579	0.0100954	1.87E-34	-0.00329187	0.131787	0.9800719	0.8221029
ENSG00000122477	LRRC39	1	100629090	C	T	0.0467197	0.012893	0.0338558	0.56	0.399081	0.0218629	1.93E-74	0.0323067	0.0848528	0.7033975	0.9672211
ENSG00000122481	RWDD3	1	95706246	A	G	0.209742	-0.00498023	0.0172728	0.7700005	-0.335808	0.00930749	4.79E-285	0.0148306	0.0514382	0.7731028	0.04394806
ENSG00000122482	ZNF644	1	91434344	G	A	0.23161	-0.0115421	0.0175122	0.3100002	-0.0540594	0.00937782	8.19E-09	0.213508	0.326054	0.5125817	0.4117216
ENSG00000122483	CCDC18	1	93694881	G	A	0.115308	0.0217449	0.0205925	0.2099999	-0.178558	0.0112877	2.31E-56	-0.121781	0.115583	0.2920582	0.6110877
ENSG00000122484	RPAP2	1	92809126	C	G	0.342942	-0.0182029	0.0147343	0.29	0.204703	0.00823356	1.92E-136	-0.0889236	0.0720678	0.2172448	0.9302901
ENSG00000122490	SLC66A2	18	77687042	T	C	0.136183	0.00825632	0.0220991	0.7899998	-0.61983	0.0116045	0	-0.0133203	0.0356544	0.7087055	0.7918724
ENSG00000122497	NA	1	148014752	G	T	0.210736	0.00258442	0.0178525	0.99	-0.153695	0.0221814	4.24E-12	-0.0168153	0.116181	0.8849212	0.560674
ENSG00000122507	BBS9	7	33407268	G	T	0.300199	0.00883258	0.0150893	0.35	-0.155624	0.00849607	6.03E-75	-0.0567559	0.0970094	0.5585106	0.4681067
ENSG00000122512	PMS2	7	6030813	C	G	0.203777	0.0194539	0.0177141	0.2399999	-0.174964	0.0095922	2.48E-74	-0.111188	0.101428	0.272978	0.307349
ENSG00000122515	ZMIZ2	7	44798828	G	A	0.468191	-0.0371934	0.0141947	0.01	-0.45541	0.00753142	0	0.0816701	0.0311983	0.008850516	0.02145408
ENSG00000122543	OCM	7	5922725	A	G	0.0337972	0.0472428	0.042103	0.1900002	0.965219	0.0606705	5.47E-57	0.0489451	0.0437285	0.2630138	0.1847216
ENSG00000122547	EEPD1	7	36266955	C	T	0.182903	0.00135213	0.0182079	0.83	-0.237463	0.00980969	1.88E-129	-0.00569406	0.0766771	0.9408033	0.01056476
ENSG00000122550	KLHL7	7	23180196	G	T	0.407555	-0.0150748	0.0143588	0.2300001	0.210864	0.00796613	2.15E-154	-0.0714908	0.0681488	0.2941597	0.9023235
ENSG00000122557	HERPUD2	7	35703507	T	C	0.489066	-0.00363913	0.0141964	0.8700001	0.223047	0.00786525	6.58E-177	-0.0163155	0.0636502	0.797695	0.2429483
ENSG00000122565	CBX3	7	26246879	T	C	0.210736	-0.00514617	0.0174391	0.7800007	-0.164996	0.00997327	1.77E-61	0.0311897	0.105711	0.7679581	0.3103563
ENSG00000122591	HYCC1	7	23017313	T	C	0.0119284	0.105458	0.0541018	0.06100002	0.530632	0.0305593	1.55E-67	0.19874	0.102598	0.05273508	0.3551447
ENSG00000122642	FKBP9	7	33021780	T	C	0.227634	-0.0300636	0.0167297	0.06699926	-0.290619	0.0104555	4.88E-170	0.103447	0.0576859	0.07292882	0.2349086
ENSG00000122643	NT5C3A	7	33078075	G	A	0.291252	0.0191861	0.0156225	0.33	0.226471	0.0160524	3.38E-45	0.0847178	0.0692433	0.2211484	0.6228019
ENSG00000122644	ARL4A	7	12728520	G	A	0.414513	0.0180918	0.0143734	0.1499999	-0.389483	0.0078473	0	-0.0464508	0.0369157	0.2082849	0.8986085
ENSG00000122674	CCZ1	7	5951980	A	G	0.451292	0.014071	0.0143176	0.3700002	0.674091	0.015762	0	0.020874	0.0212455	0.3258452	0.7637263
ENSG00000122678	POLM	7	44116992	C	T	0.084493	0.0280491	0.0245375	0.17	0.727728	0.0134248	0	0.0385434	0.0337255	0.2530978	0.1708888
ENSG00000122679	RAMP3	7	45211645	T	C	0.222664	0.0203744	0.0178502	0.33	0.307519	0.0096318	1.11E-223	0.0662541	0.0580829	0.2540025	0.5379169
ENSG00000122687	MRM2	7	2277853	G	A	0.385686	-0.0127043	0.0143933	0.4400003	0.194341	0.00802941	2.04E-129	-0.0653713	0.0741114	0.3777404	0.3429864
ENSG00000122692	SMU1	9	33059213	G	A	0.414513	-0.00649536	0.0144195	0.59	-0.221104	0.00800636	7.15E-168	0.029377	0.0652247	0.6524244	0.06166752
ENSG00000122694	GLIPR2	9	36150321	A	G	0.291252	-0.0422438	0.0154336	0.007199959	-0.210814	0.00882272	3.50E-126	0.200385	0.0736885	0.006541065	0.1973654
ENSG00000122696	SLC25A51	9	37891875	A	G	0.162028	0.0194307	0.0190703	0.2399999	0.191205	0.0121048	3.33E-56	0.101622	0.0999447	0.3092555	0.4300445
ENSG00000122705	CLTA	9	36247815	C	T	0.0497018	0.0266683	0.0308302	0.4600002	0.150813	0.0162427	1.62E-20	0.17683	0.205312	0.3890859	0.5561531
ENSG00000122707	RECK	9	36080439	C	T	0.518887	0.00697537	0.014216	0.59	-0.0689861	0.00794058	3.70E-18	-0.101113	0.206399	0.624212	0.4541907
ENSG00000122729	ACO1	9	32417726	G	C	0.131213	-0.0089968	0.0204197	0.7800007	-0.241751	0.0106979	4.53E-113	0.0372152	0.0844819	0.6595675	0.4163225
ENSG00000122733	PHF24	9	34970012	C	T	0.0168986	0.0131477	0.0623382	0.9	-0.386002	0.0438032	1.23E-18	-0.0340612	0.161543	0.8330052	0.8261478
ENSG00000122741	DCAF10	9	37834081	T	G	0.00695825	-0.0247341	0.0609053	0.6100002	-0.508669	0.0609389	6.99E-17	0.0486251	0.119876	0.6850161	NA
ENSG00000122779	TRIM24	7	138209908	A	G	0.306163	-0.00928225	0.0154323	0.2399999	0.441947	0.00831999	0	-0.0210031	0.0349211	0.5475447	0.2585625
ENSG00000122783	CYREN	7	134816331	A	G	0.270378	0.0170393	0.0156963	0.32	0.254972	0.00935623	1.59E-163	0.066828	0.0616096	0.278054	0.4805293
ENSG00000122861	PLAU	10	75673095	G	T	0.242545	-0.00325227	0.0157781	0.95	0.237734	0.00859183	1.61E-168	-0.0136803	0.0663705	0.8366973	0.3080808
ENSG00000122862	SRGN	10	70856214	T	C	0.224652	-0.0181501	0.0173294	0.4100001	0.315101	0.00997437	4.89E-219	-0.0576008	0.0550265	0.2951994	0.7104866
ENSG00000122870	BICC1	10	60432047	G	A	0.486083	0.0059238	0.0142921	0.6800001	-0.156216	0.00807359	2.08E-83	-0.0379206	0.0915104	0.6785917	0.9294424
ENSG00000122873	CISD1	10	60039082	C	T	0.209742	0.0144657	0.0173865	0.35	-0.874325	0.00832902	0	-0.016545	0.0198862	0.4054194	0.8069843
ENSG00000122877	EGR2	10	64625708	C	T	0.428429	0.018337	0.014336	0.17	-0.0747499	0.0119627	4.14E-10	-0.245311	0.195763	0.2101681	0.561756
ENSG00000122882	ECD	10	74908883	C	T	0.0596421	0.0376293	0.028621	0.2399999	-0.564379	0.0147498	0	-0.0666738	0.0507423	0.1888566	0.9080074
ENSG00000122884	P4HA1	10	74811853	G	A	0.0596421	0.0416222	0.0287115	0.1900002	0.183357	0.0155219	3.35E-32	0.227001	0.157763	0.1501858	0.9418194
ENSG00000122912	SLC25A16	10	70264876	C	A	0.136183	-0.0193984	0.0210026	0.25	0.59115	0.0105246	0	-0.0328147	0.0355332	0.3557498	0.362925
ENSG00000122952	ZWINT	10	58119012	A	G	0.0506958	0.00991482	0.0347666	0.8200001	-0.210906	0.0231374	7.84E-20	-0.0470105	0.164924	0.7756113	0.91357
ENSG00000122958	VPS26A	10	70907942	C	G	0.253479	-0.00696894	0.0168904	0.5999997	-0.201688	0.0101306	3.41E-88	0.0345531	0.0837634	0.6799663	0.5987266
ENSG00000122965	RBM19	12	114329359	T	G	0.026839	-0.0582153	0.0518728	0.1299999	-0.252773	0.0262294	5.58E-22	0.230307	0.206602	0.2649632	0.716598
ENSG00000122971	ACADS	12	121170674	A	G	0.0626243	-0.0378338	0.0306823	0.16	-0.10558	0.0168115	3.38E-10	0.358343	0.296156	0.2262867	0.4911873
ENSG00000122986	HVCN1	12	111104200	C	T	0.112326	0.0146194	0.0247526	0.32	-0.319939	0.0147055	6.01E-105	-0.0456944	0.0773952	0.5549203	0.5732891
ENSG00000123064	DDX54	12	113609131	C	T	0.291252	0.0191816	0.0154842	0.2599998	-0.0516673	0.00842997	8.84E-10	-0.371252	0.305751	0.2246592	0.52042
ENSG00000123091	RNF11	1	51720535	C	T	0.0298211	-0.107259	0.0502106	0.0129999	-0.187795	0.0274916	8.43E-12	0.57115	0.280138	0.04146837	0.2496987

ENSG00000123095	BHLHE41	12	26275509	G	C	0.185885	-0.00723122	0.0175106	0.7099994	0.0678056	0.00988592	6.94E-12	-0.106646	0.258715	0.6801812	0.6155777
ENSG00000123096	SSPN	12	26363573	C	G	0.235586	0.00365397	0.0164403	0.7600007	0.100631	0.0100697	1.63E-23	0.0363107	0.163413	0.8241567	0.8378723
ENSG00000123104	ITPR2	12	26738236	A	G	0.106362	-0.01222	0.0235386	0.7499995	-0.577532	0.0130474	0	0.021159	0.04076	0.6036829	0.2746071
ENSG00000123106	CCDC91	12	28509532	A	G	0.0467197	0.0114477	0.0367783	0.7899998	0.413577	0.0224658	1.11E-75	0.0276797	0.0889401	0.755635	0.5393655
ENSG00000123124	WWP1	8	87422808	T	C	0.0308151	0.00448315	0.0503845	0.91	-1.23241	0.0504453	8.09E-132	-0.00363771	0.0408832	0.9290993	0.03872515
ENSG00000123143	PKN1	19	14563272	A	G	0.0924453	-0.00705517	0.0245534	0.7700005	-0.260415	0.0140966	3.37E-76	0.027092	0.0942969	0.7738787	0.571975
ENSG00000123146	ADGRE5	19	14505425	A	G	0.249503	0.00953601	0.0157784	0.4799997	0.135552	0.0109295	2.54E-35	0.0703492	0.116539	0.5460731	0.7228511
ENSG00000123154	WDR83	19	12782130	C	G	0.0646123	-0.00869536	0.0296374	0.6999999	0.17166	0.0173065	3.45E-23	-0.0506546	0.172727	0.769321	0.08131273
ENSG00000123159	GIPC1	19	14597758	A	C	0.150099	-0.00932961	0.0202128	0.5400003	-0.26964	0.0114478	1.14E-122	0.0346002	0.0749765	0.6444537	0.9609913
ENSG00000123178	SPRYD7	13	50498734	C	T	0.0526839	0.0140135	0.032092	0.7499995	-0.215548	0.0178452	1.37E-33	-0.0650133	0.148983	0.66256	0.4195117
ENSG00000123179	EBPL	13	50250241	C	T	0.215706	0.00585691	0.0168283	0.83	-0.657426	0.0125221	0	-0.00890886	0.0255978	0.7278164	0.3728503
ENSG00000123191	ATP7B	13	52546219	G	A	0.120278	-0.0315932	0.0217385	0.1100001	-0.334651	0.0118938	3.50E-174	0.0944063	0.0650453	0.14667	0.5600494
ENSG00000123200	ZC3H13	13	46577747	C	T	0.40159	-0.00200292	0.014596	0.9400001	0.0546421	0.00826433	3.80E-11	-0.0366552	0.267177	0.8908772	0.1203208
ENSG00000123201	GUCY1B2	13	51611822	T	C	0.206759	0.00212866	0.0168318	0.9400001	0.469224	0.010362	0	0.00453655	0.0358717	0.899363	0.446621
ENSG00000123213	NLN	5	65092788	A	C	0.23161	0.00277981	0.0164106	0.6700003	0.517467	0.00896447	0	0.00537196	0.0317135	0.8654896	0.4472813
ENSG00000123219	CENPK	5	64836295	G	C	0.400596	-0.0175245	0.0148345	0.2099999	0.967579	0.00612133	0	-0.0181117	0.015332	0.2374831	0.97114
ENSG00000123240	OPTN	10	13160878	T	A	0.276342	0.000138467	0.0157034	0.84	-0.430845	0.00865416	0	-0.000321385	0.0364479	0.9929646	0.04596804
ENSG00000123268	ATF1	12	51186199	T	C	0.335984	0.00924449	0.0152845	0.7400005	0.328374	0.00835424	0	0.0281523	0.0465515	0.545341	0.2077573
ENSG00000123297	TSFM	12	58189113	A	G	0.318091	-0.0426971	0.0151434	0.008199927	-0.284544	0.0087018	1.59E-234	0.150055	0.0534174	0.00496809	0.8301708
ENSG00000123329	ARHGAP9	12	57874317	A	G	0.0357853	-0.0242348	0.0433125	0.4199997	-0.918154	0.023862	0	0.0263951	0.0471784	0.5758379	0.6643613
ENSG00000123338	NCKAP1L	12	54914610	A	C	0.33002	0.0175781	0.0154377	0.35	0.187496	0.00883482	5.93E-100	0.0937518	0.0824546	0.2555332	0.5509835
ENSG00000123342	MMP19	12	56232983	T	C	0.0675944	-0.0104662	0.0306972	0.7899998	-0.147682	0.0208998	1.59E-12	0.0708698	0.208102	0.7334398	0.744379
ENSG00000123352	SPATS2	12	49840786	T	C	0.300199	-0.0141325	0.0155885	0.4700002	0.0623492	0.00898274	3.89E-12	-0.226667	0.252143	0.3686729	0.7351307
ENSG00000123353	ORMDL2	12	56213683	T	C	0.0367793	-0.0669537	0.0405818	0.05099998	0.225821	0.0209785	5.07E-27	-0.29649	0.181806	0.1029321	0.1086886
ENSG00000123360	PDE1B	12	54958078	A	T	0.308151	-0.000406366	0.0157208	0.9199999	0.428966	0.0089412	0	-0.000947314	0.0366481	0.9793778	0.671513
ENSG00000123374	CDK2	12	56363560	G	C	0.0675944	0.051938	0.0295606	0.03500016	0.113864	0.0184395	6.62E-10	0.456141	0.269918	0.09104248	0.1147464
ENSG00000123384	LRP1	12	57564705	T	C	0.0606362	0.00258413	0.0292783	0.8600001	0.111336	0.017867	4.62E-10	0.0232101	0.262998	0.9296763	0.8390918
ENSG00000123395	ATG101	12	52467154	T	C	0.22664	-0.0360278	0.0180314	0.05399953	-0.190226	0.0110335	1.31E-66	0.189395	0.0954239	0.04716953	0.2333686
ENSG00000123405	NFE2	12	54690400	T	C	0.0854871	-0.0123788	0.0277159	0.6800001	-0.979775	0.016705	0	0.0126343	0.0282888	0.6551503	0.5054851
ENSG00000123411	IKZF4	12	56416831	G	A	0.0765408	0.00712425	0.0254625	0.8499999	0.142224	0.0142486	1.83E-23	0.0500918	0.179101	0.77972	0.8540118
ENSG00000123415	SMUG1	12	54570653	A	C	0.477137	0.00155153	0.0141992	0.6899999	-0.343977	0.0078226	0	-0.00451056	0.0412796	0.9129895	0.3242459
ENSG00000123416	TUBA1B	12	49523372	C	T	0.34493	-0.0299133	0.0148993	0.0659994	0.0886558	0.00895368	4.10E-23	-0.337409	0.171478	0.04910752	0.9571299
ENSG00000123427	EEF1AKMT3	12	58170799	G	A	0.32008	-0.0427389	0.0151095	0.008600031	0.911623	0.00736293	0	-0.0468822	0.0165786	0.004685836	0.9537303
ENSG00000123453	SARDH	9	136566879	A	C	0.383698	-0.00606214	0.0150815	0.64	0.245621	0.00915187	1.16E-158	-0.0246809	0.0614085	0.6877476	0.3394253
ENSG00000123454	DBH	9	136512974	G	C	0.419483	0.00990858	0.014926	0.4899999	-0.164557	0.0110797	6.73E-50	-0.0602136	0.0907945	0.5072122	0.5305184
ENSG00000123472	ATPAF1	1	47118974	A	G	0.215706	0.0102685	0.0167165	0.5999997	0.14869	0.0102942	2.73E-47	0.0690598	0.112527	0.5394013	0.6458179
ENSG00000123473	STIL	1	47747815	T	C	0.289264	0.00658742	0.0151646	0.5199996	0.102158	0.0131271	7.13E-15	0.0644826	0.148674	0.6644922	0.5167254
ENSG00000123485	HJURP	2	234752637	G	A	0.484095	-0.0196974	0.0141767	0.1499999	-0.0635645	0.00795213	1.31E-15	0.30988	0.226373	0.1710322	0.2658794
ENSG00000123500	COL10A1	6	116459998	G	A	0.0308151	0.0179886	0.0478749	0.7899998	0.384394	0.0235764	9.21E-60	0.0467973	0.124579	0.707183	0.9037544
ENSG00000123505	AMD1	6	111206444	A	G	0.167992	-0.0255744	0.0194639	0.1199999	-0.132192	0.00994402	2.52E-40	0.193464	0.147957	0.1910197	0.1839683
ENSG00000123545	NDUFAF4	6	97341473	T	C	0.00994036	0.0724926	0.0990261	0.3800004	-0.271451	0.0408528	3.04E-11	-0.267056	0.367011	0.4668262	0.6968876
ENSG00000123552	USP45	6	99924897	A	G	0.393638	-0.017601	0.0143478	0.2999998	0.136329	0.00800054	4.15E-65	-0.129107	0.105516	0.2211138	0.3579696
ENSG00000123600	METTL8	2	172235536	C	T	0.239563	0.00605571	0.0165929	0.5400003	-0.2796	0.0091722	4.33E-204	-0.0216585	0.0593494	0.7151624	0.2659524
ENSG00000123607	TTC21B	2	166762169	G	A	0.372763	0.0188873	0.0148607	0.17	0.157978	0.0121673	1.51E-38	0.119557	0.0945179	0.2059034	0.1186944
ENSG00000123609	NMI	2	152136775	A	G	0.437376	0.0251937	0.0142092	0.05699936	0.299984	0.0078111	0	0.0839835	0.047417	0.07653262	0.9398397
ENSG00000123610	TNFAIP6	2	152225333	G	T	0.413519	-0.0220198	0.0146083	0.09200046	0.374827	0.00784484	0	-0.0587465	0.0389928	0.1319135	0.6186078
ENSG00000123612	ACVR1C	2	158437202	G	A	0.0656064	0.025354	0.0269074	0.2599998	0.175481	0.0171932	1.86E-24	0.144483	0.153988	0.3481013	0.4328711
ENSG00000123636	BAZ2B	2	160324346	G	A	0.371769	0.0141093	0.0146926	0.35	0.564846	0.00749011	0	0.024979	0.0260138	0.3369436	0.9372523
ENSG00000123643	SLC36A1	5	150844274	G	T	0.385686	-0.0289454	0.0145563	0.04799986	-0.566252	0.00747416	0	0.0511176	0.0257153	0.04683061	0.556245
ENSG00000123684	LPGAT1	1	211960456	C	A	0.316103	0.0436703	0.0157201	0.0129999	-0.248975	0.00853444	4.27E-187	-0.1754	0.0634248	0.005683888	0.000418196

ENSG00000123685	BATF3	1	212866543	T	C	0.124254	0.0106053	0.0201112	0.4	-0.458526	0.011154	0	-0.0231291	0.0438641	0.5979925	0.01479839
ENSG00000123689	G0S2	1	209849249	T	C	0.267396	0.0154498	0.0165764	0.35	-0.151386	0.00928201	8.43E-60	-0.102056	0.109676	0.3521031	0.277556
ENSG00000123700	KCNJ2	17	68170501	G	A	0.12326	-0.0174313	0.0221694	0.3900004	-0.25597	0.0124572	8.02E-94	0.0680991	0.0866728	0.4320418	0.08824473
ENSG00000123737	EXOSC9	4	122730324	A	G	0.342942	0.00779127	0.0152155	0.6499995	0.32162	0.00886999	7.09E-288	0.0242251	0.0473137	0.6086438	0.2210543
ENSG00000123739	PLA2G12A	4	110641189	C	A	0.400596	-0.0141805	0.0145689	0.28	-0.159752	0.00820848	2.32E-84	0.0887659	0.0913111	0.3309881	0.4061124
ENSG00000123810	B9D2	19	41865202	T	C	0.38171	-0.0114681	0.0146496	0.32	0.137899	0.00813495	1.88E-64	-0.0831631	0.106348	0.4342184	0.961282
ENSG00000123815	COQ8B	19	41210773	C	A	0.145129	0.000282046	0.0206613	0.81	-0.138963	0.011274	6.56E-35	-0.00202965	0.148682	0.9891085	0.9692881
ENSG00000123836	PFKFB2	1	207238585	G	A	0.558648	0.00287775	0.0142263	0.9400001	0.0713972	0.00795034	2.70E-19	0.0403062	0.199306	0.839735	0.842351
ENSG00000123838	C4BPA	1	207297962	T	C	0.0715706	-0.0472428	0.0281894	0.06900014	2.03105	0.0153157	0	-0.0232603	0.0138803	0.09378272	0.7303161
ENSG00000123843	C4BPB	1	207267762	C	T	0.528827	0.0179981	0.0142155	0.17	-0.252477	0.00793794	5.30E-222	-0.0712862	0.0563488	0.2058398	0.2614041
ENSG00000123870	NA	19	53096209	T	A	0.243539	0.00966214	0.0167932	0.3900004	-0.122001	0.0126245	4.30E-22	-0.0791972	0.137892	0.5657356	0.7486516
ENSG00000123892	RAB38	11	87877522	G	C	0.336978	-0.0348629	0.0154079	0.01400006	0.529028	0.00860213	0	-0.0658999	0.0291446	0.02375095	0.6401563
ENSG00000123901	GPR83	11	94122531	A	G	0.329026	-0.0174568	0.0149453	0.33	0.273776	0.00859975	2.10E-222	-0.063763	0.0546262	0.2431051	0.2685592
ENSG00000123908	AGO2	8	141593491	T	C	0.122266	0.0252516	0.0215219	0.17	0.259192	0.0135744	2.82E-81	0.0974244	0.0831913	0.241563	0.4699505
ENSG00000123965	NA	7	74336604	T	C	0.0636183	0.0381941	0.0305639	0.28	-1.0809	0.038542	4.65E-173	-0.0353356	0.0283045	0.2118814	0.4128329
ENSG00000123975	CKS2	9	91928865	T	C	0.0854871	0.0127093	0.0250498	0.4299995	-0.565087	0.0158023	4.68E-280	-0.0224909	0.0443335	0.6119371	0.1073016
ENSG00000123983	ACSL3	2	223767504	A	C	0.0487078	-0.0241904	0.0492122	0.4299995	0.469428	0.0255947	3.91E-75	-0.0515317	0.104872	0.6231602	0.1670306
ENSG00000123989	CHPF	2	220406089	T	C	0.262425	-0.0293611	0.0158051	0.1199999	0.128061	0.0105985	1.30E-33	-0.229274	0.124868	0.06633933	0.6907268
ENSG00000123992	DNPEP	2	220251506	A	G	0.0129225	-0.0944828	0.0677256	0.2	1.01899	0.0457428	6.26E-110	-0.0927224	0.066594	0.163815	0.1925809
ENSG00000124006	OBSL1	2	220426016	A	G	0.463221	0.00565447	0.0142702	0.8200001	-0.0791094	0.00912365	4.29E-18	-0.0714766	0.180574	0.6922304	0.620766
ENSG00000124019	FAM124B	2	225255108	G	A	0.0685885	-0.00338833	0.0294278	0.7300002	0.366622	0.0167339	2.13E-106	-0.00924202	0.0802685	0.9083351	0.525562
ENSG00000124067	SLC12A4	16	67990440	A	G	0.149105	-0.00890572	0.0204579	0.4500005	0.154676	0.0110847	2.97E-44	-0.0575768	0.132328	0.6634843	0.2483547
ENSG00000124074	ENKD1	16	67699008	A	G	0.0387674	0.0577758	0.0413333	0.1100001	-0.237611	0.0401954	3.39E-09	-0.243153	0.17875	0.1737378	0.3063679
ENSG00000124098	FAM210B	20	54938845	C	G	0.185885	0.0201488	0.0193117	0.2999998	0.257619	0.0119775	1.29E-102	0.0782116	0.0750504	0.2973554	0.2790355
ENSG00000124102	PI3	20	43804351	T	C	0.161034	0.0565606	0.0184692	0.001700004	-0.44312	0.0104524	0	-0.127642	0.0417885	0.002254592	0.7750066
ENSG00000124103	FAM209A	20	55096720	A	G	0.444334	0.0187847	0.0142487	0.1299999	-0.165859	0.00886654	4.41E-78	-0.113257	0.0861214	0.188482	0.9177416
ENSG00000124104	SNX21	20	44467181	T	C	0.0208748	-0.0156706	0.0437111	0.64	-0.450393	0.0272774	3.03E-61	0.0347932	0.0970739	0.7200295	0.5731292
ENSG00000124107	SLPI	20	43882042	A	C	0.0904573	-0.0207571	0.0228843	0.3800004	0.25518	0.0129579	2.48E-86	-0.081343	0.0897742	0.3648909	0.07131296
ENSG00000124116	WFDC3	20	44398577	A	G	0.343936	0.0122679	0.0150188	0.4500005	-0.296192	0.00862636	2.30E-258	-0.0414187	0.0507206	0.4141541	0.3511715
ENSG00000124120	TTPAL	20	43113885	T	G	0.0377734	-0.0299686	0.0357429	0.4899999	0.496656	0.0229729	1.19E-103	-0.0603408	0.0720212	0.4021323	0.827433
ENSG00000124126	PREX1	20	47342605	C	T	0.0884692	-0.028466	0.024357	0.2399999	-0.316378	0.0138801	5.31E-115	0.0899745	0.0770881	0.2431434	0.9936623
ENSG00000124134	KCNS1	20	43725352	C	G	0.291252	0.00193688	0.016029	0.8499999	0.0907053	0.0091108	2.38E-23	0.0213535	0.176728	0.903828	0.003043062
ENSG00000124145	SDC4	20	43965496	T	C	0.39165	-0.0165951	0.0146688	0.2700001	-0.0931068	0.00833081	5.33E-29	0.178237	0.158353	0.2603486	0.7528405
ENSG00000124151	NCOA3	20	46208111	A	C	0.082505	-0.0265693	0.0259512	0.2700001	0.347936	0.0138291	1.11E-139	-0.0763627	0.074648	0.3063215	0.133119
ENSG00000124155	PIGT	20	44049795	G	C	0.145129	0.00011237	0.0191188	0.98	0.185128	0.0104804	7.91E-70	0.000606987	0.103274	0.9953105	0.09631219
ENSG00000124160	NCOA5	20	44704107	C	T	0.447316	0.0271051	0.0142392	0.0649995	0.0575877	0.00808293	1.04E-12	0.470675	0.255935	0.06590844	0.757863
ENSG00000124164	VAPB	20	56995167	T	G	0.39165	-0.0123057	0.0144272	0.28	0.140186	0.00796286	2.26E-69	-0.0877812	0.103035	0.3942407	0.2783538
ENSG00000124172	ATP5F1E	20	57603979	T	C	0.0119284	0.0898472	0.0731359	0.3599996	-0.694977	0.0426094	8.32E-60	-0.129281	0.105533	0.2205652	0.3048697
ENSG00000124177	CHD6	20	40138937	G	C	0.172962	-0.00606517	0.019749	0.5700002	-0.121558	0.0103907	1.29E-31	0.0498951	0.162521	0.758838	0.1030766
ENSG00000124181	PLCG1	20	39795513	G	A	0.00994036	-0.168384	0.0657483	0.01199997	-0.470766	0.0506549	1.49E-20	0.357681	0.144868	0.01354883	0.02650463
ENSG00000124191	TOX2	20	42620874	C	T	0.367793	-0.0023312	0.0164798	0.9	0.264539	0.00907193	6.20E-187	-0.0088123	0.062297	0.8875094	0.6581369
ENSG00000124193	SRSF6	20	42089406	C	T	0.428429	-0.00393359	0.014304	0.6700003	-0.0810605	0.00885114	5.28E-20	0.0485266	0.17654	0.783412	0.4008147
ENSG00000124196	GTSF1L	20	42355221	C	G	0.147117	0.00722605	0.0192662	0.7499995	-0.0846237	0.0107434	3.36E-15	-0.0853904	0.227927	0.7079289	0.9634033
ENSG00000124198	ARFGEF2	20	47595828	C	A	0.207753	-0.00532409	0.0166409	0.84	-0.20038	0.00985948	7.95E-92	0.0265699	0.0830569	0.7490438	0.9259412
ENSG00000124201	ZNFX1	20	47874723	A	G	0.203777	-0.00358734	0.0171919	0.8700001	-0.240039	0.00955751	3.39E-139	0.0149448	0.0716238	0.8347157	0.8362236
ENSG00000124203	ZNF831	20	57800121	T	C	0.0944334	0.0223219	0.0232639	0.3800004	0.332117	0.0139885	1.32E-124	0.067211	0.0701045	0.3376971	0.411358
ENSG00000124205	EDN3	20	57888264	A	C	0.439364	0.00835168	0.0142658	0.56	-0.231305	0.00789267	8.62E-189	-0.0361068	0.0616876	0.5583346	0.8578265
ENSG00000124207	CSE1L	20	47688169	T	G	0.296223	-0.00791498	0.0151847	0.7099994	-0.269879	0.00851167	1.25E-220	0.0293279	0.0562725	0.6022437	0.8884912
ENSG00000124209	RAB22A	20	56913657	A	T	0.137177	-0.0199283	0.0207904	0.28	-0.0784464	0.0106475	1.74E-13	0.254037	0.26726	0.3418465	0.3728184
ENSG00000124214	STAU1	20	47767391	A	G	0.422465	0.00669117	0.0144856	0.7700005	-0.0922929	0.00843906	7.72E-28	-0.0724993	0.157092	0.6444347	0.473347

ENSG00000124215	CDH26	20	58571268	G	A	0.449304	-0.00282801	0.014215	0.9199999	-0.0776373	0.00808234	7.56E-22	0.0364259	0.183134	0.8423388	0.4466649
ENSG00000124217	MOCS3	20	49576591	G	A	0.084493	0.0419382	0.0246434	0.1100001	-0.146856	0.0139005	4.34E-26	-0.285574	0.16997	0.09292924	0.887306
ENSG00000124222	STX16	20	57240455	A	G	0.404573	0.0299974	0.0146628	0.04	0.189206	0.00816478	8.45E-119	0.158544	0.0777981	0.04156089	0.4571532
ENSG00000124224	NA	20	56845341	T	C	0.415507	0.00995542	0.0144074	0.4	0.0589865	0.00883164	2.41E-11	0.168774	0.245553	0.491878	0.03405905
ENSG00000124225	PMEPA1	20	56255020	T	C	0.270378	0.0171873	0.0159039	0.3100002	-0.155652	0.0103728	6.73E-51	-0.110421	0.102441	0.281076	0.174682
ENSG00000124226	RNF114	20	48561688	T	C	0.16501	-0.0515647	0.0188935	0.008	0.322935	0.0118454	1.18E-163	-0.159675	0.0587981	0.006614469	0.699408
ENSG00000124228	DDX27	20	47848249	T	C	0.0864811	-0.0125581	0.0266026	0.6899999	-0.0955784	0.0149491	1.62E-10	0.131391	0.27909	0.6377968	0.9585463
ENSG00000124233	SEMG1	20	43837025	G	T	0.167992	0.0449822	0.0181103	0.01099993	-0.306273	0.0104405	3.69E-189	-0.146869	0.0593428	0.01332602	0.2706827
ENSG00000124243	BCAS4	20	49452572	A	G	0.299205	0.0163212	0.0152688	0.2399999	-0.409473	0.0081169	0	-0.039859	0.0372972	0.2852116	0.4992068
ENSG00000124251	TP53TG5	20	44019527	A	G	0.225646	-0.00786939	0.0169001	0.7700005	-0.103567	0.00964619	6.85E-27	0.0759834	0.163333	0.6417851	0.735323
ENSG00000124256	ZBP1	20	56187267	A	G	0.0516899	0.0304133	0.0333996	0.3900004	-0.392839	0.0183631	1.56E-101	-0.0774192	0.0850981	0.3629465	0.03073148
ENSG00000124275	MTRR	5	7878718	G	A	0.2167	0.0125563	0.0180175	0.6700003	0.737212	0.00859963	0	0.0170321	0.0244408	0.4858831	0.5316187
ENSG00000124279	FASTKD3	5	7864211	A	G	0.17992	0.00162978	0.0188465	0.7899998	-0.289121	0.0102679	1.93E-174	-0.00563702	0.0651858	0.9310879	0.3800707
ENSG00000124299	PEPD	19	33945278	T	C	0.519881	-0.00392451	0.0142287	0.5700002	0.15526	0.0079127	1.01E-85	-0.025277	0.0916534	0.78271	0.7294291
ENSG00000124302	CHST8	19	34188637	C	A	0.2833	-0.00766336	0.0160629	0.59	0.159494	0.00888627	4.94E-72	-0.0480479	0.100747	0.6334219	0.8086346
ENSG00000124356	STAMBP	2	74078436	A	G	0.0675944	0.0157636	0.0286471	0.8200001	0.315746	0.0156287	9.21E-91	0.0499249	0.0907619	0.5822751	0.66411
ENSG00000124357	NAGK	2	71299204	C	A	0.143141	0.0351845	0.0196851	0.09699961	-1.11524	0.0138349	0	-0.0315487	0.0176553	0.07394914	0.5130657
ENSG00000124370	MCEE	2	71347091	C	T	0.39662	-0.00428853	0.0147579	0.8600001	0.238017	0.00814392	8.99E-188	-0.0180178	0.0620067	0.7713742	0.4531459
ENSG00000124374	PAIP2B	2	71432041	A	C	0.110338	0.00741795	0.0223777	0.7700005	-0.134481	0.012829	1.04E-25	-0.0551598	0.166484	0.7404006	0.1378881
ENSG00000124380	SNRNP27	2	70126699	C	T	0.244533	0.00967337	0.0165474	0.5300002	0.496072	0.00903678	0	0.0194999	0.0333588	0.5588488	0.7678731
ENSG00000124383	MPHOSPH10	2	71367337	T	C	0.397614	-0.00356136	0.0147542	0.9	0.0859276	0.00824451	1.96E-25	-0.0414461	0.171751	0.8093112	0.3751187
ENSG00000124406	ATP8A1	4	42534756	C	T	0.0337972	0.0182222	0.0386238	0.7400005	0.444373	0.0183121	4.42E-130	0.0410065	0.0869339	0.6371426	0.1259223
ENSG00000124422	USP22	17	20924991	G	C	0.214712	0.0106392	0.0176371	0.7800007	0.331424	0.0103327	9.78E-226	0.0321014	0.0532255	0.5464277	0.3057095
ENSG00000124444	ZNF576	19	44102926	C	T	0.518887	-0.00154641	0.0143888	0.8200001	-0.0809593	0.00835045	3.16E-22	0.0191011	0.17774	0.9144188	0.7650051
ENSG00000124466	LYPD3	19	43967375	C	T	0.306163	0.000479066	0.0156897	0.95	0.103321	0.00871699	2.08E-32	0.00463669	0.151855	0.9756414	0.8355968
ENSG00000124490	CRISP2	6	49670673	T	C	0.499006	-0.00106657	0.0142607	0.8200001	0.118378	0.00802919	3.39E-49	-0.0090099	0.120469	0.9403819	0.4371972
ENSG00000124491	F13A1	6	6232782	G	T	0.360835	0.00850774	0.0146211	0.4400003	0.338112	0.00803234	0	0.0251625	0.0432475	0.5606843	0.5128355
ENSG00000124496	TRERF1	6	42306229	T	C	0.222664	0.0168773	0.0174523	0.29	0.202211	0.00965042	1.74E-97	0.0834638	0.0863992	0.3340318	0.07537455
ENSG00000124507	PACSIN1	6	34468461	G	T	0.493042	0.0121303	0.014248	0.33	0.0801889	0.00794327	5.80E-24	0.151272	0.178311	0.3962391	0.2713782
ENSG00000124508	BTN2A2	6	26389213	G	A	0.225646	-0.0239629	0.0166982	0.14	0.31859	0.00925581	1.24E-259	-0.0752155	0.0524584	0.1516256	0.8176828
ENSG00000124523	SIRT5	6	13593675	C	T	0.305169	-0.0134886	0.0153276	0.4400003	-0.306697	0.00840948	3.27E-291	0.0439802	0.0499909	0.378987	0.8590244
ENSG00000124532	MRS2	6	24414481	A	G	0.309145	0.0268494	0.0162661	0.1199999	0.278613	0.00877355	2.62E-221	0.0963681	0.0584613	0.09926856	0.7572373
ENSG00000124535	WRNIP1	6	2776417	C	A	0.301193	0.00352473	0.0156348	0.95	-0.0985891	0.00866946	5.77E-30	-0.0357517	0.158617	0.8216704	0.7722794
ENSG00000124541	RRP36	6	42993359	A	G	0.115308	-0.0186814	0.0203489	0.4500005	-0.165758	0.0113832	4.93E-48	0.112703	0.123006	0.3595434	0.1472575
ENSG00000124549	NA	6	26427115	A	G	0.244533	0.00389371	0.0173964	0.9699999	-0.105069	0.0106598	6.42E-23	-0.0370586	0.165614	0.8229401	0.4264841
ENSG00000124562	SNRPC	6	34733377	A	C	0.335984	0.0388638	0.0150925	0.02399993	-0.285074	0.00827942	8.49E-260	-0.136329	0.0530903	0.01023257	0.3955
ENSG00000124564	SLC17A3	6	25857904	T	C	0.277336	-0.0200598	0.0158967	0.16	-0.331445	0.00882918	2.074506e-308	0.0605223	0.0479889	0.2072468	0.08431906
ENSG00000124570	SERPINB6	6	2960241	T	C	0.327038	-0.0091157	0.0151288	0.5500004	-0.687945	0.007494	0	0.0132506	0.0219918	0.5468237	0.8416061
ENSG00000124571	XPO5	6	43516942	C	T	0.0656064	0.0192113	0.029544	0.5099998	0.101073	0.0179373	1.75E-08	0.190073	0.294242	0.5182965	0.5503732
ENSG00000124574	ABCC10	6	43406636	A	G	0.286282	-0.0153393	0.0154558	0.2700001	0.0903805	0.0087186	3.53E-25	-0.169719	0.17179	0.3231796	0.550795
ENSG00000124587	PEX6	6	42939283	A	G	0.411531	0.0315352	0.0143978	0.04	-0.91766	0.00614326	0	-0.0343648	0.0156914	0.02852107	0.1054135
ENSG00000124588	NQO2	6	3004108	A	G	0.257455	0.00580871	0.0165351	0.7899998	0.856452	0.00754872	0	0.00678229	0.0193066	0.7253678	0.9517456
ENSG00000124593	NA	6	41752983	T	C	0.246521	0.0161413	0.0161476	0.1900002	-0.101805	0.0112991	2.06E-19	-0.158552	0.159587	0.3204595	0.008182236
ENSG00000124596	OARD1	6	41033446	A	G	0.0198807	-0.0200082	0.0570826	0.4600002	-0.256495	0.0369121	3.68E-12	0.0780062	0.222832	0.7262876	0.8573557
ENSG00000124602	UNC5CL	6	41000850	G	T	0.152087	-0.00870211	0.0209838	0.5999997	0.148901	0.0117865	1.39E-36	-0.0584422	0.141	0.6785205	0.6482161
ENSG00000124608	AARS2	6	44274227	C	G	0.237575	0.0201029	0.01639	0.1299999	-0.0957154	0.00934794	1.32E-24	-0.210028	0.172461	0.2232893	0.4617616
ENSG00000124613	ZNF391	6	27357038	A	C	0.255467	0.00233596	0.0166565	0.8499999	-0.312608	0.00977028	1.25E-224	-0.00747249	0.0532829	0.8884689	0.02368089
ENSG00000124614	RPS10	6	34389566	G	A	0.083499	0.0387554	0.0260976	0.1800002	0.476423	0.0293281	2.44E-59	0.0813466	0.0550066	0.1391801	0.384131
ENSG00000124615	MOCS1	6	39884822	T	C	0.0178926	-0.019784	0.0615462	0.6800001	0.635867	0.0388986	4.58E-60	-0.0311134	0.0968097	0.7479168	0.3043087
ENSG00000124635	H2BC11	6	27097299	T	C	0.110338	-0.00867438	0.0244014	0.59	0.329426	0.029614	9.59E-29	-0.0263318	0.0741102	0.7223607	0.5945304

ENSG00000124641	MED20	6	41880984	A	G	0.496024	0.0240232	0.0141762	0.1299999	0.144371	0.00864365	1.25E-62	0.166399	0.0986966	0.09180356	0.2317545
ENSG00000124657	OR2B6	6	27925489	C	T	0.0775348	0.0211016	0.0251546	0.1800002	-0.100399	0.0173659	7.41E-09	-0.210178	0.25317	0.4064355	0.6768835
ENSG00000124659	TBCC	6	42713388	C	A	0.0745527	0.0647298	0.0280173	0.01400006	0.133798	0.0158187	2.72E-17	0.483788	0.217072	0.02583359	0.465202
ENSG00000124688	MAD2L1BP	6	43602983	T	C	0.327038	0.00508455	0.015077	0.7800007	0.425246	0.00813817	0	0.0119567	0.0354555	0.7359422	0.8329222
ENSG00000124693	NA	6	26032052	T	C	0.33002	-0.0317623	0.0155268	0.0129999	-0.141448	0.00866884	7.50E-60	0.224551	0.11063	0.04238152	0.01055709
ENSG00000124701	APOBEC2	6	41026646	C	A	0.192843	0.0124617	0.0165441	0.4199997	0.0935088	0.00911926	1.14E-24	0.133268	0.177402	0.4525219	0.9177879
ENSG00000124702	KLHDC3	6	42985493	T	C	0.0586481	-0.0405009	0.0309658	0.1800002	-0.1849	0.018783	7.28E-23	0.219043	0.168945	0.194793	0.5422703
ENSG00000124713	GNMT	6	42930057	A	G	0.411531	0.0315352	0.0143978	0.04	0.434291	0.0113865	0	0.072613	0.033207	0.02876615	0.2325208
ENSG00000124721	DNAH8	6	38840709	G	A	0.37674	-0.000561499	0.0143227	0.8	-0.149503	0.00798488	3.20E-78	0.00375578	0.0958026	0.9687283	0.4341527
ENSG00000124731	TREM1	6	41245060	T	C	0.111332	0.0379462	0.0227258	0.08600031	-0.822647	0.0120492	0	-0.046127	0.0276335	0.09506945	0.3181331
ENSG00000124733	MEA1	6	42980769	A	C	0.028827	-0.0364458	0.0458123	0.3599996	0.257413	0.0224607	2.08E-30	-0.141585	0.1784	0.4274071	0.02761735
ENSG00000124743	KLHL31	6	53521602	C	T	0.0675944	0.029668	0.0270126	0.3400001	-0.0871599	0.0144654	1.69E-09	-0.340386	0.315027	0.2799201	0.4939936
ENSG00000124762	CDKN1A	6	36649710	A	C	0.208748	-0.0070458	0.0166953	0.7199992	-0.208691	0.00919676	5.40E-114	0.0337619	0.0800139	0.6730615	0.9434597
ENSG00000124766	SOX4	6	21596409	G	A	0.311133	0.00535886	0.0152954	0.4899999	-0.0803967	0.00863608	1.29E-20	-0.0666552	0.190384	0.7262563	0.4205991
ENSG00000124767	GLO1	6	38657309	T	G	0.491054	0.0109856	0.014223	0.4700002	-0.325639	0.00773387	0	-0.0337355	0.0436845	0.4399647	0.3860357
ENSG00000124772	CPNE5	6	36758165	G	A	0.250497	-0.00509531	0.0166634	0.7400005	0.188573	0.00912249	6.29E-95	-0.0270204	0.0883755	0.7597987	0.1806552
ENSG00000124780	KCNK17	6	39274553	G	A	0.493042	-0.0117416	0.0141825	0.3700002	-0.649203	0.00705904	0	0.0180862	0.0218469	0.4077498	0.1573562
ENSG00000124782	RREB1	6	7180021	G	T	0.460239	0.0252104	0.0142179	0.04300015	-0.172222	0.00872927	1.21E-86	-0.146383	0.0828886	0.07739131	0.456564
ENSG00000124783	SSR1	6	7308109	C	A	0.12326	-0.00904518	0.0234043	0.91	-0.396901	0.0123688	6.32E-226	0.0227895	0.0589719	0.6991656	0.02615185
ENSG00000124784	RIOK1	6	7403999	G	A	0.420477	0.0180918	0.0143948	0.2599998	-0.234976	0.00804838	2.22E-187	-0.0769943	0.0613175	0.2092371	0.1091786
ENSG00000124785	NRN1	6	6002716	A	G	0.292247	-0.0155582	0.0158313	0.29	-0.270843	0.00879248	2.32E-208	0.0574437	0.0584817	0.3259769	0.848762
ENSG00000124786	SLC35B3	6	8424547	T	C	0.203777	0.00121826	0.0184158	0.7600007	-0.308679	0.00983644	3.65E-216	-0.00394668	0.0596601	0.9472562	0.2685299
ENSG00000124787	RPP40	6	4999631	T	C	0.0318091	0.0627539	0.0386701	0.09499921	-0.571249	0.0220793	1.35E-147	-0.109854	0.067827	0.1053142	0.9928076
ENSG00000124788	ATXN1	6	16530532	A	G	0.17992	-0.00493977	0.0188379	0.5999997	-0.346649	0.0101891	1.07E-253	0.0142501	0.0543444	0.7931539	0.7519491
ENSG00000124789	NUP153	6	17660962	C	T	0.209742	0.0376839	0.0160043	0.01199997	0.0856563	0.00990384	5.20E-18	0.439943	0.193644	0.02309134	0.4121668
ENSG00000124795	DEK	6	18244576	C	G	0.0407555	0.0241024	0.036306	0.6200004	-0.297013	0.0414209	7.47E-13	-0.0811494	0.12276	0.5085859	0.92302
ENSG00000124802	EEF1E1	6	8088202	G	A	0.256461	-0.00743375	0.0161189	0.58	-0.0597781	0.00988983	1.50E-09	0.124356	0.270429	0.645627	0.9976306
ENSG00000124813	RUNX2	6	45463990	T	G	0.225646	0.00399621	0.0169824	0.6899999	-0.526279	0.00915842	0	-0.00759333	0.0322691	0.8139659	0.3905732
ENSG00000124831	LRRFIP1	2	238629272	C	G	0.217694	0.0113751	0.0165643	0.4299995	0.446676	0.0133939	7.48E-244	0.0254661	0.0370913	0.4923485	0.9734824
ENSG00000124875	CXCL6	4	74708497	G	A	0.398608	-0.000638288	0.0143274	0.7600007	0.0789181	0.00804155	9.82E-23	-0.00808798	0.181549	0.9644662	0.3466615
ENSG00000124882	EREG	4	75242664	C	G	0.285288	-0.0147827	0.0164886	0.32	-0.167204	0.00911772	4.09E-75	0.088411	0.0987313	0.3705353	0.1793253
ENSG00000124920	MYRF	11	61538052	A	G	0.494036	0.00199374	0.0141808	0.6999999	-0.23351	0.00954623	3.84E-132	-0.00853813	0.0607298	0.8881923	0.3511078
ENSG00000124942	AHNAK	11	62262361	C	T	0.279324	0.0108069	0.0161853	0.6200004	0.149647	0.008925	4.24E-63	0.0722158	0.108242	0.5046638	0.9520531
ENSG00000125089	SH3TC1	4	8213314	A	G	0.352883	-0.000604974	0.0152428	0.8800001	-0.284555	0.00977092	1.86E-186	0.00212604	0.0535673	0.968341	0.9715288
ENSG00000125107	CNOT1	16	58608822	C	T	0.241551	0.0122182	0.0164366	0.4199997	0.138783	0.00910963	2.08E-52	0.0880379	0.118574	0.4578027	0.41128
ENSG00000125122	FBXL9P	16	67250996	T	C	0.0656064	0.00828997	0.0284306	0.9699999	-0.136876	0.0151882	2.02E-19	-0.0605654	0.207819	0.7707197	0.840363
ENSG00000125124	BBS2	16	56527471	A	C	0.213718	-0.0153519	0.0163682	0.5	-0.926123	0.00790098	0	0.0165765	0.0176745	0.348306	0.2998406
ENSG00000125148	MT2A	16	56642760	A	C	0.280318	-0.00357954	0.0155186	0.8800001	-0.588207	0.0081061	0	0.00608551	0.026383	0.8175789	0.612624
ENSG00000125149	PHAF1	16	67163151	T	G	0.0308151	0.0110705	0.0417889	0.9699999	-0.242954	0.0217319	5.13E-29	-0.0455663	0.172052	0.7911323	0.5374363
ENSG00000125166	GOT2	16	58754648	A	C	0.38171	-0.00675457	0.0144551	0.83	0.23992	0.00798426	2.24E-198	-0.0281535	0.060257	0.6403404	0.4157387
ENSG00000125170	DOK4	16	57513551	C	G	0.312127	-0.0145207	0.0155624	0.2599998	0.250505	0.00852267	6.79E-190	-0.0579656	0.0621553	0.35103	0.7472825
ENSG00000125245	GPR18	13	99910483	T	C	0.0516899	-0.0300985	0.0365022	0.3700002	0.345216	0.0214313	2.24E-58	-0.0871874	0.105876	0.4102302	0.4019006
ENSG00000125246	CLYBL	13	100404153	C	G	0.10835	-0.0479742	0.0237355	0.05600025	-0.48704	0.0127476	0	0.0985015	0.0488023	0.04355192	0.1634341
ENSG00000125247	TMTC4	13	101291764	C	T	0.305169	-0.0038045	0.0155895	0.84	0.495178	0.00804012	0	-0.00768309	0.0314829	0.8071994	0.1650843
ENSG00000125249	RAP2A	13	98103929	T	C	0.287276	0.00610931	0.0155581	0.5400003	-0.225067	0.00870962	3.06E-147	-0.0271444	0.0691345	0.6945915	0.3063242
ENSG00000125257	ABCC4	13	95812885	G	A	0.415507	-0.00627623	0.0146805	0.8	0.500614	0.00777976	0	-0.0125371	0.0293257	0.6690064	0.4065111
ENSG00000125266	EFNB2	13	107164770	T	C	0.415507	0.021198	0.0144145	0.14	-0.0502498	0.0080174	3.67E-10	-0.421852	0.294647	0.1522241	0.4047538
ENSG00000125304	TM9SF2	13	100184965	T	C	0.15507	-0.0438449	0.0198515	0.0179999	-0.191485	0.0110442	2.43E-67	0.228974	0.104509	0.02845621	0.5504695
ENSG00000125319	HROB	17	42229559	C	T	0.302187	-0.00197188	0.0156597	0.8600001	-0.135461	0.00894997	9.46E-52	0.0145568	0.115607	0.8997982	0.8724548
ENSG00000125347	IRF1	5	131821895	A	G	0.379722	0.010574	0.0144329	0.25	0.0801856	0.00821321	1.62E-22	0.131869	0.1805	0.4650372	0.9484704

ENSG00000125375	DMAC2L	14	50790660	C	A	0.439364	-0.0102577	0.0143387	0.4500005	0.232592	0.00794634	2.48E-188	-0.0441017	0.0616659	0.4745027	0.7709568
ENSG00000125378	BMP4	14	54420966	C	G	0.515905	0.00609286	0.0142105	0.8200001	0.0832066	0.00803044	3.71E-25	0.0732257	0.170932	0.6683665	0.7847345
ENSG00000125384	PTGER2	14	52788173	A	G	0.158052	-0.0256864	0.0199957	0.1800002	-0.355327	0.0102222	9.69E-265	0.0722895	0.0563125	0.19924	0.4356561
ENSG00000125386	FAM193A	4	2680640	G	C	0.304175	-0.00450166	0.0153059	0.8	0.0923179	0.00874157	4.53E-26	-0.0487626	0.16586	0.7687589	0.2136404
ENSG00000125388	GRK4	4	3003904	G	T	0.349901	0.00528302	0.015142	0.8600001	-0.354541	0.00839409	0	-0.014901	0.0427102	0.7271745	0.6446179
ENSG00000125430	HS3ST3B1	17	14228560	G	A	0.10835	0.0226727	0.0228049	0.2599998	0.194781	0.0136633	4.13E-46	0.116401	0.117364	0.321298	0.4817797
ENSG00000125434	SLC25A35	17	8194871	T	C	0.0497018	0.0396404	0.0378113	0.4600002	-0.231959	0.0197735	8.86E-32	-0.170894	0.163658	0.296387	0.8055849
ENSG00000125445	MRPS7	17	73260104	G	A	0.202783	0.0195654	0.0189548	0.5199996	-0.699071	0.0110109	0	-0.0279877	0.0271178	0.302036	0.0118228
ENSG00000125447	GGA3	17	73245569	C	G	0.115308	-0.0309313	0.0211308	0.1299999	-0.090827	0.011387	1.51E-15	0.340552	0.236534	0.1499358	0.9350198
ENSG00000125449	ARMC7	17	73116203	C	T	0.384692	0.00777388	0.014874	0.5700002	-0.207079	0.008583	1.31E-128	-0.0375407	0.0718446	0.6013039	0.2479145
ENSG00000125450	NUP85	17	73216803	C	T	0.243539	0.00518756	0.0171773	0.9699999	0.657288	0.00973426	0	0.00789237	0.0261339	0.7626537	0.09755059
ENSG00000125454	SLC25A19	17	73277332	C	T	0.240557	0.00287608	0.0172765	0.95	0.105354	0.0104534	6.88E-24	0.0272993	0.164008	0.8678022	0.4324777
ENSG00000125457	MIF4GD	17	73264808	G	C	0.314115	-0.00501696	0.0155202	0.5199996	-0.365653	0.00848076	0	0.0137206	0.0424464	0.7465101	0.06733565
ENSG00000125458	NT5C	17	73127105	C	G	0.328032	0.0163244	0.0157599	0.28	0.287543	0.00863203	2.66E-243	0.0567719	0.0548353	0.3005203	0.4061466
ENSG00000125459	MSTO1	1	155649066	T	C	0.027833	0.0805565	0.053844	0.1100001	-0.421159	0.0291625	2.82E-47	-0.191273	0.128531	0.1367127	0.02374364
ENSG00000125482	TTF1	9	135266608	T	C	0.130219	-0.0181456	0.022331	0.28	0.0995973	0.0118087	3.33E-17	-0.18219	0.225251	0.4186133	0.1235771
ENSG00000125484	GTF3C4	9	135557882	C	T	0.403579	0.0168133	0.0145291	0.2	-0.0544505	0.00821175	3.34E-11	-0.308781	0.270864	0.2542925	0.1429082
ENSG00000125485	DDX31	9	135507086	T	C	0.259443	0.0292196	0.0167076	0.07000032	0.367548	0.00893981	0	0.0794988	0.0454981	0.0805852	0.2145087
ENSG00000125498	KIR2DL1	19	55288518	T	A	0.303181	0.0221961	0.0154856	0.1499999	0.240857	0.0159645	1.97E-51	0.0921547	0.0645832	0.153605	0.292532
ENSG00000125503	PPP1R12C	19	55615604	C	T	0.122266	0.00167208	0.0216801	0.8700001	-0.216131	0.0210101	8.07E-25	-0.00773644	0.100313	0.9385257	0.9840835
ENSG00000125505	MBOAT7	19	54685420	T	A	0.276342	-0.00907217	0.0158423	0.2200002	-0.155023	0.0084551	4.37E-75	0.0585216	0.102243	0.5670674	0.6147467
ENSG00000125510	OPRL1	20	62721761	T	C	0.483101	-0.00913206	0.0142306	0.4899999	-0.436714	0.00873878	0	0.0209108	0.0325883	0.5210894	0.02340282
ENSG00000125520	SLC2A4RG	20	62372240	A	C	0.237575	0.0101994	0.0160134	0.4	0.192154	0.0136731	7.34E-45	0.0530794	0.0834219	0.5245966	0.9187136
ENSG00000125531	FNDC11	20	62186217	T	C	0.0447316	-0.150162	0.045613	0.000549997	0.20085	0.0258262	7.43E-15	-0.747631	0.246609	0.002432206	0.2590679
ENSG00000125534	PPDPF	20	62152818	T	C	0.135189	-0.0176749	0.0204459	0.4	-0.618784	0.0126448	0	0.0285639	0.0330472	0.3874029	0.3616838
ENSG00000125538	IL1B	2	113590904	G	A	0.5	-0.0115367	0.0142023	0.58	-0.0653327	0.00804194	4.51E-16	0.176584	0.218468	0.4189285	0.6138548
ENSG00000125551	PLGLB2	2	88052959	A	G	0.369781	-0.0228047	0.0152923	0.1100001	0.581192	0.0200572	1.29E-184	-0.0392378	0.0263468	0.1364129	0.4622085
ENSG00000125611	CHCHD5	2	113344334	C	G	0.255467	0.00588753	0.0168732	0.81	0.128529	0.0102265	3.16E-36	0.0458072	0.13133	0.7272443	0.7588711
ENSG00000125618	PAX8	2	114005050	T	C	0.393638	-0.0117791	0.0147757	0.2399999	-0.541301	0.00759039	0	0.0217607	0.0272983	0.4253673	0.5913758
ENSG00000125629	INSIG2	2	118857300	A	G	0.239563	-0.015578	0.0162063	0.33	-0.189777	0.00908982	8.49E-97	0.0820857	0.0854869	0.3369475	0.9720264
ENSG00000125630	POLR1B	2	113317063	G	C	0.0695825	-0.0270104	0.025534	0.5500004	0.905412	0.0211348	0	-0.0298322	0.0282101	0.2902841	0.4900333
ENSG00000125633	CCDC93	2	118722381	C	T	0.0904573	0.0111205	0.0275583	0.5	-0.117277	0.0154919	3.73E-14	-0.0948225	0.235318	0.6869819	0.6252786
ENSG00000125637	PSD4	2	113937858	G	A	0.452286	-0.00999933	0.0144259	0.32	0.664003	0.00715405	0	-0.0150592	0.0217263	0.4882268	0.4599395
ENSG00000125648	SLC25A23	19	6450652	C	G	0.469185	0.00262326	0.0142183	0.58	-0.2603	0.0130527	1.75E-88	-0.0100778	0.054625	0.8536283	0.6801172
ENSG00000125651	GTF2F1	19	6386786	C	T	0.178926	0.0216773	0.0189672	0.2	-0.226548	0.0110795	6.34E-93	-0.0956854	0.0838535	0.2538273	0.1646851
ENSG00000125652	ALKBH7	19	6373743	G	A	0.0894632	0.025186	0.0237859	0.17	0.32916	0.0263328	7.47E-36	0.0765161	0.0725213	0.2913869	0.02133253
ENSG00000125656	CLPP	19	6365191	T	G	0.026839	0.0409468	0.0408521	0.3100002	0.630672	0.0257211	9.13E-133	0.0649257	0.0648296	0.316594	0.7562107
ENSG00000125686	MED1	17	37584038	G	A	0.250497	0.0172617	0.0163507	0.5300002	0.166035	0.00915306	1.55E-73	0.103964	0.0986442	0.2919136	0.3141214
ENSG00000125703	ATG4C	1	63290495	T	C	0.417495	-0.0121759	0.0143983	0.3400001	-0.215509	0.00795639	1.43E-161	0.0564983	0.0668432	0.3979787	0.6174978
ENSG00000125726	CD70	19	6593654	C	T	0.399602	0.0152398	0.0146236	0.3100002	-0.0979867	0.00814412	2.42E-33	-0.155529	0.149799	0.2991537	0.5041712
ENSG00000125730	C3	19	6704144	G	T	0.183897	0.0100147	0.017342	0.64	-0.0919391	0.0105965	4.08E-18	-0.108928	0.189042	0.5644749	0.09206621
ENSG00000125731	SH2D3A	19	6759885	C	T	0.27833	-0.0104076	0.0159615	0.4199997	-0.0759846	0.00868098	2.08E-18	0.13697	0.210644	0.5155357	0.6028791
ENSG00000125733	TRIP10	19	6744736	C	T	0.181909	0.00578154	0.0186846	0.8200001	-0.234886	0.0104433	5.02E-112	-0.0246142	0.079555	0.7570182	0.2497986
ENSG00000125734	GPR108	19	6733769	G	A	0.471173	0.0395616	0.0143224	0.005600025	-0.193501	0.00990158	4.79E-85	-0.204452	0.074753	0.006237403	0.05220151
ENSG00000125735	TNFSF14	19	6666873	A	G	0.195825	0.0179894	0.0191012	0.25	0.305469	0.0102981	2.32E-193	0.058891	0.0625621	0.3465409	0.2650288
ENSG00000125740	FOSB	19	45974845	G	A	0.406561	-0.0066	0.0144581	0.7499995	-0.0710765	0.00966581	1.93E-13	0.0928577	0.203808	0.6486673	0.8716256
ENSG00000125743	SNRPD2	19	46193269	G	T	0.200795	-0.012775	0.0180179	0.4600002	-0.319814	0.0101167	2.49E-219	0.0399451	0.0563529	0.4784245	0.3399414
ENSG00000125744	RTN2	19	45994433	T	A	0.0984095	-0.017348	0.0226786	0.4199997	-0.245073	0.0126038	3.25E-84	0.0707872	0.0926098	0.4446534	0.8713145
ENSG00000125746	EML2	19	46129569	A	G	0.10835	0.0162134	0.021451	0.3599996	-0.0970599	0.0140626	5.13E-12	-0.167045	0.222329	0.4524466	0.8587567
ENSG00000125753	VASP	19	46020039	A	G	0.297217	0.00163201	0.0161724	0.83	0.626448	0.00910127	0	0.00260518	0.0258161	0.9196194	0.6776168

ENSG00000125755	SYMPK	19	46342608	G	A	0.158052	0.00352945	0.0194816	0.8200001	-0.186651	0.010981	8.56E-65	-0.0189094	0.10438	0.8562435	0.2920109
ENSG00000125772	GPCPD1	20	5558378	A	G	0.0119284	0.00992769	0.0474097	0.7400005	0.67085	0.0292325	1.51E-116	0.0147987	0.070674	0.8341412	0.3492454
ENSG00000125775	SDCBP2	20	1300251	T	C	0.131213	-0.0345541	0.0211108	0.16	0.188785	0.0122017	5.36E-54	-0.183034	0.112448	0.1035855	0.3944372
ENSG00000125779	PANK2	20	3888545	C	T	0.187873	0.0114787	0.0186922	0.5400003	-0.312609	0.0105337	1.52E-193	-0.036719	0.0598069	0.5392425	0.7440134
ENSG00000125780	TGM3	20	2299185	C	T	0.163022	-0.016183	0.0184645	0.3700002	-0.397985	0.0103111	0	0.0406623	0.0464069	0.3809144	0.06978793
ENSG00000125810	CD93	20	23063481	T	A	0.110338	-0.00801309	0.024096	0.7700005	-0.88521	0.012376	0	0.00905219	0.0272209	0.7394778	0.1445213
ENSG00000125812	GZF1	20	23348243	C	G	0.292247	0.0105992	0.0153653	0.4899999	0.0724497	0.00926822	5.41E-15	0.146297	0.212907	0.4919921	0.8266758
ENSG00000125814	NAPB	20	23378642	A	C	0.0795229	-0.0237928	0.0266634	0.4400003	0.730011	0.0141531	0	-0.0325924	0.0365301	0.3722826	0.8175894
ENSG00000125817	CENPB	20	3765917	A	G	0.0815109	0.039006	0.0248929	0.064	0.259319	0.0149908	4.82E-67	0.150417	0.0963862	0.1186268	0.08656012
ENSG00000125818	PSMF1	20	1121464	C	T	0.12326	0.00919998	0.0220592	0.4199997	-0.280409	0.0130036	3.91E-103	-0.0328091	0.0786826	0.6766926	0.625117
ENSG00000125821	DTD1	20	18656549	A	G	0.028827	0.00728897	0.0451619	0.7899998	0.685411	0.0243267	1.18E-174	0.0106345	0.0658914	0.8717833	0.3091341
ENSG00000125826	RBCK1	20	399876	G	A	0.10338	-0.0324303	0.0214326	0.14	0.540368	0.0118719	0	-0.0600152	0.0396849	0.1304591	0.4605906
ENSG00000125827	TMX4	20	7979235	T	C	0.0755467	-0.0183924	0.028926	0.4299995	0.774404	0.0151818	0	-0.0237504	0.0373555	0.5249108	0.3261285
ENSG00000125834	STK35	20	2119970	T	G	0.434394	-0.0159005	0.0143661	0.17	0.108173	0.00802112	1.89E-41	-0.146992	0.133253	0.2699845	0.9445877
ENSG00000125835	SNRPB	20	2446889	T	G	0.101392	-0.00686285	0.0228983	0.6499995	0.333089	0.0121092	1.44E-166	-0.0206036	0.0687493	0.7644119	0.1726308
ENSG00000125843	AP5S1	20	3803563	G	A	0.38668	0.0145144	0.0144587	0.5700002	-0.0886974	0.00989462	3.13E-19	-0.16364	0.164031	0.3184654	0.1967009
ENSG00000125844	RRBP1	20	17628631	A	G	0.437376	0.00185959	0.0143479	0.9599999	0.255327	0.00797201	4.46E-225	0.00728318	0.0561947	0.8968781	0.920551
ENSG00000125845	BMP2	20	6754619	A	G	0.154076	0.0236256	0.0208484	0.3100002	0.260354	0.0110159	1.71E-123	0.0907441	0.0801691	0.2576727	0.954511
ENSG00000125846	ZNF133	20	18283380	A	G	0.209742	0.00691067	0.0174717	0.9599999	0.0798277	0.00962546	1.10E-16	0.0865698	0.219116	0.6927794	0.7872304
ENSG00000125863	MKKS	20	10400351	T	G	0.444334	0.0280739	0.0143245	0.07199959	0.467632	0.00787866	0	0.0600342	0.0306487	0.05013803	0.05077788
ENSG00000125864	BFSP1	20	17512207	A	G	0.290258	-0.000315348	0.0156304	0.7499995	0.217849	0.00877999	6.65E-136	-0.00144755	0.0717487	0.9839035	0.324173
ENSG00000125868	DSTN	20	17569697	G	A	0.440358	0.00264539	0.0143606	0.9199999	0.380465	0.00779679	0	0.00695305	0.0377452	0.8538484	0.9027783
ENSG00000125869	LAMP5	20	9503089	A	C	0.129225	-0.0075177	0.0206737	0.5700002	0.388426	0.0124713	5.84E-213	-0.0193543	0.0532279	0.7161484	0.3110299
ENSG00000125870	SNRPB2	20	16716513	T	G	0.0725646	-0.0201832	0.0305371	0.3599996	-0.535606	0.0156287	2.14E-257	0.0376829	0.0570247	0.5087294	0.3370957
ENSG00000125871	MGME1	20	17960660	A	C	0.136183	0.011047	0.0212777	0.3400001	-0.934625	0.0127774	0	-0.0118197	0.0227666	0.6036429	0.2933082
ENSG00000125875	TBC1D20	20	429660	T	C	0.519881	-0.0195614	0.014249	0.1499999	0.14104	0.00867989	2.27E-59	-0.138694	0.101388	0.1713269	0.1889625
ENSG00000125877	ITPA	20	3197015	C	A	0.129225	-0.0200793	0.02062	0.2700001	-0.658844	0.0121177	0	0.0304765	0.0313022	0.3302444	0.5488933
ENSG00000125885	MCM8	20	5953575	C	T	0.172962	0.0194838	0.0188579	0.29	-0.538047	0.0108555	0	-0.0362121	0.0350564	0.3016198	0.6637983
ENSG00000125898	FAM110A	20	820640	A	G	0.483101	0.0336137	0.0142305	0.0329997	0.303175	0.00839831	2.30E-285	0.110872	0.0470385	0.01842086	0.3075886
ENSG00000125900	SIRPD	20	1527193	T	C	0.308151	0.00116055	0.0152129	0.98	0.224521	0.00927344	1.70E-129	0.005169	0.0677574	0.9391909	0.06283378
ENSG00000125901	MRPS26	20	3027745	T	G	0.481113	0.00310102	0.0142042	0.7600007	0.11779	0.00793972	8.62E-50	0.0263266	0.120602	0.8272005	0.5187468
ENSG00000125910	S1PR4	19	3176336	T	C	0.302187	0.0199889	0.0156071	0.17	-0.10276	0.0100285	1.22E-24	-0.194521	0.153061	0.2037756	0.4809046
ENSG00000125912	NCLN	19	3197567	G	C	0.441352	-0.0168793	0.0142726	0.2	0.365494	0.010013	1.03E-291	-0.0461822	0.0390707	0.2371992	0.2973174
ENSG00000125945	ZNF436	1	23690938	C	A	0.275348	0.0237144	0.0161115	0.1	0.0782738	0.00895213	2.26E-18	0.302967	0.208731	0.1466489	0.8660837
ENSG00000125952	MAX	14	65521152	T	C	0.39165	0.018517	0.0143992	0.1499999	-0.197013	0.00805581	4.34E-132	-0.0939888	0.0731886	0.199072	0.9290858
ENSG00000125966	MMP24	20	33839629	C	G	0.196819	0.028214	0.0176902	0.1800002	-0.20117	0.0103434	2.96E-84	-0.140249	0.0882316	0.1119343	0.02780129
ENSG00000125970	RALY	20	32638783	T	G	0.0735586	-0.0235332	0.0290874	0.25	-0.0914518	0.0146619	4.45E-10	0.257329	0.320727	0.4223621	0.9544251
ENSG00000125971	DYNLRB1	20	33116488	T	G	0.0735586	-0.0235332	0.0290874	0.25	-0.0995278	0.0147157	1.35E-11	0.236448	0.294337	0.4217874	0.9735743
ENSG00000125977	EIF2S2	20	32688121	G	A	0.358847	-0.00146463	0.0144495	0.89	-0.272165	0.00878254	7.51E-211	0.00538141	0.0530913	0.9192635	0.7556963
ENSG00000125991	ERGIC3	20	34137587	G	A	0.235586	0.0111052	0.017361	0.7199992	-0.124014	0.00929265	1.26E-40	-0.0895478	0.140153	0.5228684	0.1229453
ENSG00000126001	CEP250	20	34071394	T	C	0.250497	0.00967869	0.0169501	0.8499999	-0.17733	0.00894783	2.07E-87	-0.0545802	0.0956248	0.5681529	0.4851302
ENSG00000126003	PLAGL2	20	30787950	T	G	0.105368	-0.0211445	0.0204629	0.4600002	0.146975	0.0118372	2.13E-35	-0.143864	0.139708	0.3031277	0.07960356
ENSG00000126005	MMP24OS	20	33835284	T	A	0.200795	0.0332592	0.0175711	0.1100001	-0.304472	0.0109491	3.48E-170	-0.109236	0.0578436	0.05896348	0.05427306
ENSG00000126067	PSMB2	1	36087315	G	A	0.0119284	-0.21515	0.185736	0.1800002	-0.58636	0.0514874	4.78E-30	0.366925	0.318396	0.2491491	0.2864169
ENSG00000126088	UROD	1	45479533	T	C	0.212724	0.0214182	0.0162465	0.07699987	-0.442125	0.00906863	0	-0.0484438	0.0367598	0.1875554	0.5317334
ENSG00000126091	ST3GAL3	1	44284163	G	C	0.393638	-0.01767	0.0143819	0.3599996	-0.0873247	0.00807321	2.87E-27	0.202348	0.165754	0.2221702	0.664977
ENSG00000126106	TMEM53	1	45120568	A	G	0.411531	-0.00304355	0.0145757	0.6800001	-0.0793571	0.00807772	8.86E-23	0.0383526	0.183714	0.8346335	0.800857
ENSG00000126107	HECTD3	1	45472606	A	G	0.0129225	-0.0030674	0.0791311	0.91	-1.43195	0.126789	1.40E-29	0.00214211	0.0552612	0.9690791	0.986221
ENSG00000126214	KLC1	14	104098060	A	G	0.310139	-0.0049449	0.0150209	0.7899998	0.374979	0.0081414	0	-0.0131871	0.040059	0.7420099	0.5168877
ENSG00000126215	XRCC3	14	104172893	C	T	0.215706	0.0375352	0.0178505	0.03799969	-0.333319	0.0105884	1.65E-217	-0.112611	0.0536732	0.03589826	0.4373275

ENSG00000126216	TUBGCP3	13	113190903	G	A	0.387674	0.031086	0.0145532	0.05999983	-0.198443	0.00808862	6.47E-133	-0.15665	0.0736144	0.03333917	0.2592021
ENSG00000126217	MCF2L	13	113651372	A	G	0.0854871	-0.0266629	0.0245847	0.3100002	0.438739	0.0204375	3.14E-102	-0.0607716	0.0561063	0.2787415	0.02636526
ENSG00000126218	F10	13	113790485	T	C	0.171968	-0.0286276	0.0191392	0.1199999	-0.0728078	0.0100929	5.44E-13	0.393194	0.268464	0.1430287	0.9889455
ENSG00000126226	PCID2	13	113847460	T	C	0.171968	-0.0286276	0.0191392	0.1199999	-0.14789	0.0105857	2.35E-44	0.193573	0.130155	0.1369467	0.4553789
ENSG00000126231	PROZ	13	113819831	A	T	0.225646	-0.0288314	0.0171215	0.08400014	-0.190758	0.00914356	1.17E-96	0.151141	0.0900468	0.09325521	0.6552932
ENSG00000126243	LRFN3	19	36431464	A	G	0.0248509	-0.00903865	0.0397103	0.8	0.578727	0.0248199	2.98E-120	-0.0156182	0.06862	0.8199542	0.7050338
ENSG00000126246	IGFLR1	19	36231706	T	C	0.157058	-0.0569209	0.0202848	0.000749998	-0.901085	0.0121283	0	0.0631693	0.0225276	0.005045928	0.09635281
ENSG00000126247	CAPNS1	19	36635866	C	T	0.377734	-0.0104247	0.0145344	0.6800001	-0.186019	0.00818697	2.75E-114	0.056041	0.0781727	0.4734444	0.1892646
ENSG00000126249	PDCD2L	19	34906288	A	C	0.0318091	-0.0687077	0.0354053	0.04	0.263255	0.0200747	2.74E-39	-0.260993	0.135955	0.05489588	0.1487712
ENSG00000126251	GPR42	19	35862843	A	G	0.346918	0.0248993	0.0147472	0.05499966	0.292072	0.0214459	3.09E-42	0.0852505	0.0508782	0.09382026	0.8576942
ENSG00000126254	RBM42	19	36124260	G	T	0.0417495	-0.0171528	0.0327152	0.7499995	0.208838	0.0205601	3.07E-24	-0.0821346	0.156862	0.6005501	0.475428
ENSG00000126261	UBA2	19	34940058	T	C	0.10835	-0.0257059	0.02208	0.1900002	0.149515	0.017763	3.86E-17	-0.171928	0.149083	0.2488129	0.1214337
ENSG00000126262	FFAR2	19	35938738	T	C	0.0198807	0.063255	0.0492047	0.1900002	0.313877	0.0276009	5.77E-30	0.201528	0.157763	0.2014569	0.9223153
ENSG00000126264	HCST	19	36394330	G	A	0.00894632	0.008866	0.0689692	0.9699999	-0.431967	0.0492237	1.70E-18	-0.0205247	0.15968	0.8977245	0.6421144
ENSG00000126267	COX6B1	19	36144444	T	C	0.5	0.0329862	0.0142128	0.005099998	0.349669	0.00775392	0	0.0943356	0.0407003	0.02045953	0.322902
ENSG00000126337	KRT36	17	39645593	C	T	0.102386	-0.0314257	0.0232857	0.1100001	0.148855	0.0226278	4.76E-11	-0.211117	0.159691	0.1861558	0.9500857
ENSG00000126351	THRA	17	38232331	A	G	0.150099	-0.00501481	0.021712	0.9	0.445407	0.0109122	0	-0.0112589	0.0487472	0.8173414	0.327229
ENSG00000126353	CCR7	17	38715872	C	T	0.522863	-0.0107956	0.0141874	0.29	-0.0825997	0.00793374	2.20E-25	0.130698	0.172219	0.4479097	0.6412458
ENSG00000126368	NR1D1	17	38253009	A	G	0.150099	-0.00501481	0.021712	0.9	0.303966	0.0120736	7.30E-140	-0.0164979	0.071432	0.817346	0.285411
ENSG00000126432	PRDX5	11	64087421	G	C	0.161034	0.0351672	0.0192121	0.03899959	0.841784	0.0101932	0	0.041777	0.0228287	0.06724666	0.4103039
ENSG00000126456	IRF3	19	50165979	C	T	0.256461	-0.0250656	0.0165496	0.07599937	0.292491	0.00888771	1.60E-237	-0.085697	0.0566415	0.1302865	0.4157941
ENSG00000126458	RRAS	19	50141003	G	C	0.333002	-0.0334403	0.0152038	0.01499996	0.0523575	0.00884566	3.24E-09	-0.638692	0.309785	0.03923375	0.679974
ENSG00000126460	PRRG2	19	50089087	G	A	0.125249	-0.0101204	0.0211491	0.4700002	0.106481	0.0108292	8.13E-23	-0.095044	0.198853	0.6326782	0.2187572
ENSG00000126464	PRR12	19	50112298	C	T	0.218688	-0.0142362	0.0168919	0.16	0.0768531	0.0100041	1.56E-14	-0.185239	0.221113	0.4021676	0.4441321
ENSG00000126467	TSKS	19	50254798	T	G	0.275348	0.000354596	0.0168401	0.9400001	0.387879	0.0100101	0	0.000914192	0.0434158	0.9832005	0.04206208
ENSG00000126500	FLRT1	11	63878652	A	G	0.527833	-0.00599418	0.014202	0.5300002	0.0560321	0.00877367	1.70E-10	-0.106978	0.254015	0.6736476	0.1044991
ENSG00000126522	ASL	7	65549665	C	G	0.372763	-0.00629245	0.0146938	0.6700003	-0.189758	0.0080934	1.45E-121	0.0331604	0.0774474	0.6685295	0.4680944
ENSG00000126524	SBDS	7	66456626	T	C	0.0437376	-0.0164886	0.035739	0.7899998	-0.257628	0.0369582	3.15E-12	0.0640015	0.139027	0.6452619	0.5835707
ENSG00000126561	STAT5A	17	40451763	A	G	0.199801	0.0332003	0.0181884	0.14	-0.164658	0.00991391	6.02E-62	-0.201632	0.111127	0.06961145	0.5399702
ENSG00000126581	BECN1	17	40973759	T	C	0.110338	-0.00849837	0.0219546	0.7700005	0.460707	0.0123421	5.73E-305	-0.0184464	0.0476568	0.6987062	0.9507712
ENSG00000126602	TRAP1	16	3734619	C	T	0.284294	0.0077324	0.015605	0.5300002	-0.587983	0.0125114	0	-0.0131507	0.0265413	0.6202611	0.4700348
ENSG00000126698	DNAJC8	1	28543302	C	T	0.287276	0.0151783	0.0155906	0.3100002	-0.264029	0.0144041	4.75E-75	-0.0574872	0.059132	0.3309589	0.3898302
ENSG00000126746	ZNF384	12	6787190	T	C	0.0964215	-0.0292049	0.0236905	0.25	0.324505	0.0156041	4.68E-96	-0.0899982	0.0731331	0.2184695	0.2263383
ENSG00000126749	EMG1	12	7087932	A	G	0.0924453	0.00129052	0.0247192	0.9199999	0.34118	0.0131357	9.90E-149	0.00378252	0.0724522	0.9583637	0.9677358
ENSG00000126773	PCNX4	14	60597240	G	A	0.150099	-0.0426382	0.0200008	0.016	0.369621	0.011337	3.72E-233	-0.115357	0.0542272	0.03339666	0.6136473
ENSG00000126775	ATG14	14	55855843	A	G	0.154076	0.0200021	0.0195558	0.1299999	0.105852	0.0111619	2.46E-21	0.188962	0.185817	0.3091894	0.9367154
ENSG00000126777	KTN1	14	56097017	T	A	0.0606362	-0.0301099	0.024807	0.1800002	-0.714911	0.0146981	0	0.042117	0.0347102	0.2249813	0.3698723
ENSG00000126787	DLGAP5	14	55636613	T	C	0.326044	0.00728566	0.0151443	0.33	0.0727499	0.00937306	8.39E-15	0.100147	0.208569	0.6311128	0.6921645
ENSG00000126790	L3HYPDH	14	59939114	C	T	0.469185	0.00666372	0.0142573	0.7099994	-0.263115	0.008757	2.45E-198	-0.0253263	0.0541932	0.6402613	0.7789359
ENSG00000126804	ZBTB1	14	64985419	C	T	0.450298	0.00260278	0.0142375	0.7199992	0.0543053	0.00814221	2.57E-11	0.0479286	0.262274	0.8549997	0.4999973
ENSG00000126814	TRMT5	14	61443122	G	C	0.0516899	0.0141565	0.0375348	0.7499995	0.266783	0.0287018	1.47E-20	0.0530638	0.14081	0.7062876	0.2694531
ENSG00000126821	SGPP1	14	64172844	T	C	0.0328032	-0.0942181	0.0461499	0.02699977	0.169488	0.0243154	3.16E-12	-0.555899	0.283729	0.05008257	0.6238707
ENSG00000126822	PLEKHG3	14	65192215	T	C	0.149105	-0.0119903	0.0207478	0.5099998	0.461362	0.0110367	0	-0.0259889	0.0449751	0.5633642	0.8632073
ENSG00000126838	PZP	12	9331201	T	C	0.263419	0.00381047	0.0154094	0.6800001	-0.78603	0.0116106	0	-0.00484774	0.0196042	0.8046911	0.08068236
ENSG00000126858	RHOT1	17	30524933	C	T	0.188867	0.0271759	0.0189744	0.1900002	-0.393825	0.0103052	0	-0.069005	0.0482136	0.1523627	0.500974
ENSG00000126860	EVI2A	17	29646740	G	A	0.379722	-0.00957749	0.014571	0.56	-0.368562	0.0078596	0	0.0259861	0.0395386	0.5110308	0.1723867
ENSG00000126861	OMG	17	29611794	A	T	0.286282	-0.000923168	0.0154869	0.9400001	0.0704595	0.00863325	3.31E-16	-0.0131021	0.219805	0.9524678	0.4969267
ENSG00000126870	DYNC2I1	7	158699353	T	C	0.237575	0.0190956	0.0169669	0.4100001	-0.490578	0.00885722	0	-0.0389247	0.0345926	0.2604917	0.9076268
ENSG00000126878	AIF1L	9	133985201	A	T	0.50497	-0.00471156	0.0142037	0.7099994	-0.169985	0.00962817	9.31E-70	0.0277174	0.0835731	0.7401498	0.07038746
ENSG00000126882	FAM78A	9	134142698	T	C	0.026839	0.0323366	0.0431107	0.5099998	0.156757	0.0236351	3.30E-11	0.206285	0.276769	0.4560713	0.8845455

ENSG00000126883	NUP214	9	134055502	G	A	0.026839	0.0346363	0.0428154	0.4899999	0.651523	0.0337629	5.69E-83	0.0531621	0.0657736	0.4189419	0.8372087
ENSG00000126934	MAP2K2	19	4107222	T	G	0.476143	0.00594468	0.0144069	0.64	0.237187	0.0103385	1.76E-116	0.0250632	0.0607504	0.6799286	0.4852598
ENSG00000127022	CANX	5	179131777	C	T	0.510934	0.019526	0.0141789	0.1199999	0.252415	0.0116562	5.45E-104	0.0773567	0.0562864	0.169336	0.04126711
ENSG00000127054	INTS11	1	1253518	C	T	0.0775348	0.00830969	0.028407	0.7400005	0.283561	0.0176612	5.22E-58	0.0293048	0.100196	0.7699242	0.9451492
ENSG00000127080	IPPK	9	95404006	A	G	0.32505	0.0217812	0.0151982	0.14	0.085222	0.00863988	5.97E-23	0.255582	0.180209	0.1561173	0.4047031
ENSG00000127081	ZNF484	9	95624089	A	G	0.0258449	0.0603129	0.056967	0.2599998	0.435864	0.0311924	2.27E-44	0.138375	0.131074	0.2911016	0.1200117
ENSG00000127084	FGD3	9	95754125	T	C	0.0427435	-0.0680817	0.0364452	0.04700023	0.863071	0.0189897	0	-0.0788831	0.042263	0.06197413	0.2901113
ENSG00000127124	HIVEP3	1	42236816	C	T	0.431412	0.0184546	0.0144486	0.2399999	0.27973	0.00797341	1.23E-269	0.065973	0.0516862	0.2018095	0.2411397
ENSG00000127125	PPCS	1	42935099	T	C	0.33499	-0.0194217	0.0151149	0.1900002	0.050173	0.00844634	2.85E-09	-0.387095	0.308223	0.2091554	0.6026302
ENSG00000127191	TRAF2	9	139798711	G	A	0.371769	-0.016568	0.0144984	0.3900004	0.165189	0.00831933	9.78E-88	-0.100298	0.087914	0.2539286	0.4321379
ENSG00000127220	ABHD8	19	17411992	T	C	0.277336	0.000987199	0.0157846	0.8800001	0.188058	0.00941856	1.07E-88	0.00524943	0.0839349	0.9501315	0.8548765
ENSG00000127249	ATP13A4	3	193215383	A	C	0.460239	-0.0293478	0.0142306	0.03400008	-0.256875	0.00852965	3.03E-199	0.114249	0.0555286	0.03963971	0.7221221
ENSG00000127252	PLAAT1	3	192977220	G	A	0.163022	0.00929847	0.0179015	0.7700005	-0.234215	0.0187849	1.11E-35	-0.0397007	0.0764984	0.6037789	0.8222397
ENSG00000127311	HELB	12	66716874	G	A	0.483101	-0.00738192	0.0141991	0.7899998	-0.091152	0.00794685	1.86E-30	0.0809848	0.155934	0.6035144	0.3333324
ENSG00000127314	RAP1B	12	69029495	A	C	0.225646	0.00835392	0.0182567	0.99	0.0538356	0.00971621	3.01E-08	0.155175	0.340274	0.6483694	0.9250955
ENSG00000127325	BEST3	12	70065198	G	A	0.501988	-0.0229081	0.0141809	0.1800002	0.0453249	0.00819944	3.24E-08	-0.50542	0.325959	0.1210059	0.06396909
ENSG00000127328	RAB3IP	12	70174722	G	A	0.338966	-0.0222612	0.015233	0.08799946	0.320054	0.00828595	0	-0.0695546	0.0476292	0.144198	0.1204624
ENSG00000127329	PTPRB	12	70970925	G	A	0.170974	0.00987264	0.0188078	0.3599996	-0.258591	0.0158191	4.58E-60	-0.0381786	0.0727693	0.599825	0.5571486
ENSG00000127334	DYRK2	12	68050652	A	G	0.506958	-0.0193052	0.0143706	0.1299999	-0.0475735	0.0079525	2.20E-09	0.405797	0.309594	0.189946	0.778366
ENSG00000127337	YEATS4	12	69769029	T	G	0.447316	0.0031779	0.014217	0.5500004	-0.697165	0.00698565	0	-0.00455832	0.0203927	0.8231249	0.4710385
ENSG00000127364	TAS2R4	7	141478738	A	T	0.466203	0.000367727	0.014213	0.84	0.052993	0.00839479	2.74E-10	0.00693917	0.268208	0.9793591	0.4697619
ENSG00000127366	TAS2R5	7	141490591	C	G	0.472167	-0.000308807	0.0142061	0.9	-0.270639	0.00784599	9.90E-261	0.00114103	0.052491	0.9826572	0.4423142
ENSG00000127377	CRYGN	7	151131910	C	G	0.364811	0.0454089	0.0151377	0.002999991	0.22747	0.00841174	4.76E-161	0.199626	0.0669564	0.002869009	0.1821951
ENSG00000127399	LRRC61	7	150027483	C	T	0.26839	-0.00519539	0.0162161	0.6999999	0.429402	0.00865121	0	-0.0120991	0.0377652	0.7486818	0.297551
ENSG00000127412	TRPV5	7	142618086	A	G	0.197813	0.0531952	0.0186274	0.007699987	-0.573768	0.0182792	2.85E-216	-0.092712	0.0325991	0.004455046	0.2508605
ENSG00000127415	IDUA	4	989550	A	G	0.441352	-0.00567013	0.0142741	0.7800007	0.288681	0.0118808	2.05E-130	-0.0196415	0.0494525	0.6912355	0.2856867
ENSG00000127418	FGFRL1	4	1012204	A	G	0.212724	-0.00268268	0.0172661	1	-0.224903	0.0110693	8.95E-92	0.0119282	0.0767736	0.8765311	0.9335105
ENSG00000127419	TMEM175	4	939309	T	C	0.176938	-0.00525426	0.0194726	0.5700002	-0.139146	0.0114112	3.35E-34	0.0377608	0.139978	0.7873431	0.8523788
ENSG00000127445	PIN1	19	9953145	T	C	0.228628	0.00598197	0.0170122	0.6800001	0.0895971	0.00950188	4.12E-21	0.0667652	0.190006	0.7253003	0.3357004
ENSG00000127452	FBXL12	19	9929717	A	G	0.214712	0.0198605	0.018004	0.35	-0.125442	0.00978133	1.19E-37	-0.158325	0.144055	0.2717429	0.4934448
ENSG00000127463	EMC1	1	19560102	G	C	0.484095	0.00226913	0.0142915	0.95	0.268671	0.00873951	1.56E-207	0.00844576	0.053194	0.873848	0.1013055
ENSG00000127481	UBR4	1	19468885	G	A	0.441352	0.0206135	0.0142425	0.09299937	-0.0989057	0.00794704	1.48E-35	-0.208416	0.144971	0.1505377	0.4419235
ENSG00000127483	HP1BP3	1	21091485	G	A	0.125249	-0.00390745	0.0223532	0.8700001	-0.140961	0.0122684	1.48E-30	0.02772	0.158595	0.8612488	0.2148015
ENSG00000127507	ADGRE2	19	14866279	T	G	0.409543	-0.0024255	0.0143028	0.84	0.678814	0.00717868	0	-0.00357315	0.0210703	0.865339	0.2975182
ENSG00000127511	SIN3B	19	16965687	T	C	0.0785288	0.0155641	0.028877	0.6300007	-0.439502	0.0157784	9.45E-171	-0.035413	0.0657162	0.5899709	0.4735984
ENSG00000127526	SLC35E1	19	16671917	G	A	0.329026	-0.0334825	0.0152757	0.03799969	0.130352	0.00957019	3.02E-42	-0.256863	0.118696	0.03046139	0.1076925
ENSG00000127527	EPS15L1	19	16524473	G	A	0.324056	-0.0347266	0.0152942	0.02999991	0.0913256	0.0086599	5.31E-26	-0.380251	0.171307	0.02643867	0.4255348
ENSG00000127528	KLF2	19	16437156	A	G	0.416501	0.00917779	0.0146105	0.56	0.0903729	0.00829688	1.25E-27	0.101555	0.161938	0.5305793	0.6105632
ENSG00000127533	F2RL3	19	17001544	C	T	0.439364	0.00477961	0.0144294	0.6999999	0.114179	0.00798926	2.47E-46	0.0418605	0.126409	0.7405296	0.3230307
ENSG00000127554	GFER	16	2035979	G	A	0.191849	0.0252088	0.0178758	0.14	-0.0826463	0.0127943	1.05E-10	-0.30502	0.221387	0.1682747	0.5553473
ENSG00000127561	SYNGR3	16	2041968	T	C	0.175944	-0.015085	0.0183533	0.5400003	0.35823	0.0125537	4.20E-179	-0.0421098	0.0512545	0.4113147	0.9088318
ENSG00000127578	WFIKKN1	16	681677	A	G	0.254473	-0.00699569	0.0171773	0.6200004	0.385412	0.0100679	0	-0.0181512	0.0445711	0.6838313	0.2651634
ENSG00000127580	WDR24	16	737533	C	A	0.263419	0.0147809	0.0170453	0.3400001	0.286139	0.01172	1.20E-131	0.0516564	0.0596076	0.3861571	0.6192682
ENSG00000127585	FBXL16	16	749164	C	T	0.267396	0.0108549	0.0170469	0.4899999	-0.144564	0.0104094	7.51E-44	-0.0750874	0.118044	0.5247122	0.8275272
ENSG00000127586	CHTF18	16	844391	C	T	0.168986	0.0134581	0.0185285	0.4400003	-0.135403	0.0152443	6.56E-19	-0.0993931	0.137297	0.4691091	0.9738256
ENSG00000127603	MACF1	1	39749918	T	C	0.212724	-0.0393892	0.016726	0.02900013	-0.362042	0.00911101	0	0.108797	0.0462801	0.01873091	0.203707
ENSG00000127616	SMARCA4	19	11123834	C	G	0.313121	-0.00201907	0.0152412	0.7800007	-0.166156	0.00864001	2.04E-82	0.0121517	0.0917304	0.8946115	0.3466902
ENSG00000127663	KDM4B	19	5061365	T	C	0.190855	0.00014329	0.0192485	0.7199992	0.281934	0.0100907	8.70E-172	0.000508239	0.068273	0.9940604	0.4909267
ENSG00000127666	TICAM1	19	4823830	T	C	0.375746	-0.0109287	0.0146622	0.4100001	0.204344	0.00814141	5.05E-139	-0.0534818	0.071784	0.4562492	0.05571625
ENSG00000127688	NA	16	81381248	G	A	0.269384	-0.00812851	0.0163143	0.4	-0.0599121	0.0109281	4.20E-08	0.135674	0.273426	0.6197536	0.1796763

ENSG00000127720	METTL25	12	82812645	T	G	0.124254	-0.0331997	0.0208509	0.1199999	0.29898	0.0124633	3.64E-127	-0.111043	0.0698936	0.1121175	0.7919043
ENSG00000127804	METTL16	17	2367264	A	G	0.353877	-0.0166693	0.01508	0.17	-0.110522	0.00940905	7.37E-32	0.150823	0.137046	0.2711017	0.4516569
ENSG00000127824	TUBA4A	2	220128662	C	T	0.0924453	0.0153355	0.0237808	0.4700002	-0.103219	0.0143735	6.91E-13	-0.148573	0.23132	0.5206885	0.8421417
ENSG00000127831	VIL1	2	219300916	A	T	0.417495	0.00820635	0.0143288	0.4600002	-0.316584	0.0130341	2.57E-130	-0.0259216	0.0452733	0.566944	0.6923173
ENSG00000127837	AAMP	2	219131915	C	T	0.418489	-0.00730091	0.0143134	0.7600007	-0.0922917	0.0080248	1.31E-30	0.0791069	0.155241	0.6103498	0.885059
ENSG00000127838	PNKD	2	219173315	C	T	0.389662	-0.00373003	0.0144294	0.9	-0.531465	0.00760247	0	0.00701839	0.0271504	0.7960208	0.6863721
ENSG00000127863	TNFRSF19	13	24197370	A	G	0.406561	-0.0114026	0.0143315	0.5400003	0.0456908	0.00823436	2.88E-08	-0.24956	0.316871	0.4309443	0.7192139
ENSG00000127870	RNF6	13	26751522	A	C	0.39662	0.000404284	0.014452	0.9	0.0573301	0.00813701	1.85E-12	0.00705186	0.252086	0.9776829	0.7350511
ENSG00000127884	ECHS1	10	135181588	A	G	0.232604	0.0183205	0.0161589	0.1900002	0.250181	0.0118365	3.68E-99	0.0732289	0.0646816	0.257574	0.1280971
ENSG00000127903	ZNF835	19	57178585	C	T	0.241551	0.018154	0.0161165	0.1499999	-0.151604	0.00973893	1.22E-54	-0.119746	0.106585	0.2612312	0.8079231
ENSG00000127914	AKAP9	7	91655084	G	T	0.385686	1.67E-05	0.0145542	0.9299999	-0.0747716	0.00817998	6.20E-20	-0.000223021	0.194649	0.9990858	0.5342807
ENSG00000127920	GNG11	7	93553421	G	T	0.0745527	0.00710616	0.0235513	0.7300002	-0.21339	0.0131589	3.86E-59	-0.0333014	0.110387	0.7628969	0.7260685
ENSG00000127946	HIP1	7	75265450	T	C	0.436382	0.00631119	0.0143518	0.5400003	-0.862639	0.00700202	0	-0.00731614	0.0166372	0.6601209	0.8054344
ENSG00000127947	PTPN12	7	77217990	T	C	0.350895	-0.00709493	0.0150244	0.4400003	-0.393427	0.00825264	0	0.0180337	0.0381904	0.6367812	0.5704795
ENSG00000127948	POR	7	75572345	T	C	0.176938	-0.0054855	0.0190594	0.9	0.403317	0.0100179	0	-0.013601	0.0472579	0.7734972	0.7343641
ENSG00000127951	FGL2	7	76825915	C	T	0.166998	0.0257176	0.0191491	0.16	-0.191715	0.0112005	1.12E-65	-0.134145	0.10019	0.1806021	0.6723007
ENSG00000127952	STYXL1	7	75651489	A	G	0.10835	-0.0145914	0.0237195	0.3700002	1.33059	0.0116906	0	-0.0109661	0.0178266	0.5384519	0.8579315
ENSG00000127954	STEAP4	7	87920975	C	T	0.189861	0.00432813	0.0177465	0.84	-0.89256	0.00904323	0	-0.00484912	0.0198828	0.8073194	0.9943426
ENSG00000127955	GNAI1	7	79805994	G	T	0.121272	0.0167674	0.0222401	0.3599996	0.171094	0.0134575	4.97E-37	0.0980013	0.130216	0.4516877	0.5934696
ENSG00000127957	NA	7	75144866	A	T	0.445328	-0.0146869	0.0143915	0.3100002	0.765195	0.0150094	0	-0.0191937	0.0188114	0.307576	0.02383129
ENSG00000127980	PEX1	7	92137089	C	T	0.0357853	-0.0597111	0.0345531	0.06199976	0.553288	0.024694	3.46E-111	-0.10792	0.0626359	0.0848922	0.6656561
ENSG00000127990	SGCE	7	94250031	C	T	0.500994	-0.0107407	0.0141976	0.2999998	-0.255624	0.00781526	1.19E-234	0.0420176	0.0555558	0.4494616	0.9964444
ENSG00000127993	RBM48	7	92162703	G	A	0.0188867	0.0278855	0.0531737	0.59	-0.283	0.0337653	5.23E-17	-0.0985352	0.18826	0.6006965	0.2338867
ENSG00000127995	CASD1	7	94162431	T	G	0.106362	-0.00875847	0.0253738	0.56	-0.136112	0.0131076	2.93E-25	0.0643476	0.186522	0.730104	0.7893361
ENSG00000128000	ZNF780B	19	40548141	T	C	0.0119284	-0.0402151	0.0599143	0.59	-0.402306	0.0674219	2.42E-09	0.0999614	0.149866	0.5047698	0.9392211
ENSG00000128011	LRFN1	19	39801592	G	A	0.124254	-0.0117142	0.0225743	0.59	-0.209738	0.0164325	2.62E-37	0.0558516	0.10772	0.604117	0.06296938
ENSG00000128039	SRD5A3	4	56225769	T	C	0.152087	0.04713	0.0189331	0.004600023	0.657914	0.0114141	0	0.0716356	0.0288043	0.01288351	0.4603072
ENSG00000128040	SPINK2	4	57681967	A	G	0.249503	0.0137806	0.0162663	0.4299995	0.226418	0.00986504	1.42E-116	0.0608634	0.0718907	0.3972122	0.5566979
ENSG00000128050	PAICS	4	57314720	A	G	0.15507	-0.00450881	0.0194449	0.8600001	-0.138679	0.0109768	1.37E-36	0.0325125	0.140239	0.8166645	0.6227457
ENSG00000128059	PPAT	4	57280654	T	C	0.454274	-0.0261832	0.0142985	0.03400008	-0.186016	0.0146601	6.84E-37	0.140758	0.0776633	0.06992312	0.2596298
ENSG00000128159	TUBGCP6	22	50669769	A	G	0.397614	0.0138994	0.01444	0.2200002	0.104938	0.00934546	2.94E-29	0.132453	0.138109	0.337536	0.305115
ENSG00000128165	ADM2	22	50922427	T	G	0.137177	0.0318438	0.022092	0.1199999	0.142614	0.0138649	8.15E-25	0.223286	0.156421	0.1534445	0.03149253
ENSG00000128185	DGCR6L	22	20304701	A	G	0.427435	0.00805038	0.0142926	0.5700002	-0.478963	0.0172418	7.74E-170	-0.016808	0.0298469	0.57334	0.1363553
ENSG00000128191	DGCR8	22	20083577	A	G	0.054672	-0.0368718	0.0313372	0.2599998	1.00532	0.0169039	0	-0.0366768	0.0311776	0.2394414	0.8719896
ENSG00000128203	ASPHD2	22	26833110	A	G	0.4334	4.96E-05	0.0142178	0.9299999	-0.206611	0.00790836	1.87E-150	-0.000240254	0.0688144	0.9972143	0.135074
ENSG00000128218	VPREB3	22	24095792	C	A	0.107356	0.0038032	0.0234056	0.84	0.27515	0.0126519	7.25E-105	0.0138223	0.0850671	0.8709228	0.8856071
ENSG00000128228	SDF2L1	22	21997568	T	C	0.0318091	-0.0986746	0.0433981	0.05999983	-0.274248	0.0303486	1.62E-19	0.359801	0.163176	0.02745534	0.3514429
ENSG00000128242	GAL3ST1	22	30960598	G	A	0.347913	-0.0176727	0.0145938	0.1499999	-0.0539778	0.00847109	1.87E-10	0.327407	0.275206	0.2341724	0.8997969
ENSG00000128245	YWHAH	22	32347018	C	A	0.250497	-0.0369709	0.0164507	0.025	0.41291	0.00886965	0	-0.0895373	0.0398872	0.02478346	0.9560002
ENSG00000128253	RFPL2	22	32593570	G	T	0.493042	0.0064693	0.0142239	0.5500004	-0.160993	0.0079641	7.25E-91	-0.0401838	0.0883736	0.6493223	0.2081027
ENSG00000128266	GNAZ	22	23439882	A	G	0.445328	0.0133877	0.0142678	0.1900002	0.146609	0.00795542	7.72E-76	0.0913158	0.0974449	0.3487062	0.8867966
ENSG00000128268	MGAT3	22	39870774	C	A	0.251491	-0.0220405	0.0167635	0.07399971	0.381362	0.00929499	0	-0.0577942	0.0439795	0.188807	0.8505498
ENSG00000128271	ADORA2A	22	24826087	C	T	0.412525	-0.00202382	0.0143894	0.7499995	-0.13974	0.00807589	4.43E-67	0.0144827	0.102976	0.8881528	0.06043829
ENSG00000128272	ATF4	22	39917195	A	G	0.327038	0.00393948	0.0153811	0.9599999	0.135449	0.0088479	6.70E-53	0.0290846	0.113572	0.7978822	0.365377
ENSG00000128274	A4GALT	22	43102715	G	A	0.114314	0.0175257	0.0216641	0.4700002	0.606509	0.012059	0	0.028896	0.035724	0.4185901	0.5433061
ENSG00000128283	CDC42EP1	22	37960933	G	A	0.436382	0.0324156	0.0144597	0.04799986	0.555232	0.00916794	0	0.0583821	0.0260605	0.02507437	0.3994018
ENSG00000128284	APOL3	22	36549298	A	T	0.503976	-0.00270704	0.0142152	0.8499999	-0.181925	0.0078983	2.16E-117	0.01488	0.0781405	0.8489751	0.8053945
ENSG00000128285	MCHR1	22	41076786	T	G	0.454274	-0.00907953	0.0142697	0.8499999	0.27015	0.00816202	3.13E-240	-0.0336092	0.0528312	0.5246703	0.5210836
ENSG00000128294	TPST2	22	26957069	G	A	0.467197	0.00788216	0.0143558	0.64	-0.155339	0.00796244	9.21E-85	-0.0507416	0.0924523	0.5831151	0.7454123
ENSG00000128298	BAIAP2L2	22	38493786	C	A	0.152087	0.0181233	0.0195677	0.4299995	-0.221339	0.0106496	6.07E-96	-0.0818803	0.0884938	0.3548274	0.7048128

ENSG00000128309	MPST	22	37420769	A	G	0.305169	-0.0213979	0.0157865	0.14	0.0792236	0.00879246	2.05E-19	-0.270095	0.201507	0.1801237	0.3986817
ENSG00000128311	TST	22	37411290	A	G	0.2833	0.0258505	0.015967	0.16	0.230357	0.00886198	5.82E-149	0.112219	0.0694486	0.1061237	0.6820804
ENSG00000128322	IGLL1	22	23918903	A	G	0.360835	-0.00873507	0.0146983	0.58	-0.0716899	0.0085272	4.20E-17	0.121845	0.205538	0.553307	0.2667934
ENSG00000128335	APOL2	22	36629128	C	G	0.340954	0.010336	0.0153959	0.5099998	0.164082	0.00931979	2.22E-69	0.0629927	0.0938985	0.5023097	0.5790739
ENSG00000128340	RAC2	22	37630894	A	G	0.279324	0.0180698	0.0156738	0.25	-0.252532	0.00859408	8.69E-190	-0.0715545	0.0621144	0.2493292	0.1567125
ENSG00000128383	APOBEC3A	22	39353967	C	A	0.0715706	-0.0248727	0.026044	0.33	-0.744595	0.0157812	0	0.0334044	0.0349846	0.3396634	0.1458856
ENSG00000128394	APOBEC3F	22	39443262	T	C	0.0536779	-0.0408775	0.0273946	0.17	-0.323958	0.0268738	1.83E-33	0.126182	0.0852077	0.1386408	0.3366054
ENSG00000128408	RIBC2	22	45818974	A	G	0.507952	-0.0125921	0.0141816	0.3599996	-0.0900166	0.00821229	5.87E-28	0.139886	0.15806	0.3761463	0.3620561
ENSG00000128438	NA	17	16832191	A	G	0.166998	0.0200348	0.018018	0.2200002	0.115724	0.012356	7.55E-21	0.173125	0.156791	0.2695159	0.5487762
ENSG00000128463	EMC4	15	34519778	A	C	0.205765	0.00340845	0.0186627	0.9	-0.22684	0.020659	4.76E-28	-0.0150258	0.082284	0.8551048	0.2110852
ENSG00000128482	RNF112	17	19317513	G	T	0.220676	0.0072865	0.0173296	0.7700005	0.316803	0.00925338	6.93E-257	0.0230001	0.0547056	0.6741685	0.9230642
ENSG00000128487	SPECC1	17	20067498	T	C	0.112326	0.0163094	0.0210309	0.3700002	0.351421	0.0145013	9.80E-130	0.0464099	0.059876	0.4382804	0.6204855
ENSG00000128512	DOCK4	7	111606316	C	T	0.119284	0.0235287	0.0205719	0.2	-0.0837757	0.0127536	5.07E-11	-0.280854	0.249254	0.2598365	0.3138065
ENSG00000128534	LSM8	7	117828482	G	C	0.382704	-0.00868128	0.0147904	0.58	0.397951	0.00879015	0	-0.0218149	0.0371695	0.5572679	0.2498723
ENSG00000128536	CDHR3	7	105597059	A	G	0.498012	-0.00238786	0.0142193	0.7800007	-0.108959	0.0080597	1.21E-41	0.0219152	0.130511	0.8666478	0.3929468
ENSG00000128563	PRKRIP1	7	102035721	A	T	0.0705765	-0.00731792	0.0267175	0.91	0.560804	0.0167766	5.43E-245	-0.013049	0.047643	0.7841686	0.2002128
ENSG00000128567	PODXL	7	131213998	G	C	0.213718	0.00495879	0.0179744	0.9299999	0.120558	0.00984417	1.75E-34	0.0411321	0.149132	0.7826934	0.772179
ENSG00000128585	MKLN1	7	130988125	G	A	0.513917	-0.00726255	0.0142223	0.3700002	-0.0468291	0.00794927	3.84E-09	0.155086	0.304845	0.6109362	0.5005158
ENSG00000128590	DNAJB9	7	108212653	G	A	0.502982	-0.0166246	0.0141967	0.1299999	0.0741762	0.0079416	9.62E-21	-0.224123	0.19289	0.245267	0.04155026
ENSG00000128594	LRRC4	7	127669642	C	T	0.0775348	-0.0216948	0.027835	0.2599998	0.241635	0.0141667	3.12E-65	-0.0897834	0.115315	0.4362185	0.04869705
ENSG00000128595	CALU	7	128395603	A	G	0.43837	-0.0031458	0.0143093	0.83	0.191266	0.00791159	4.04E-129	-0.0164473	0.0748167	0.8260005	0.3468015
ENSG00000128596	CCDC136	7	128446498	T	C	0.109344	0.0161892	0.0225446	0.6200004	-0.285083	0.0117701	1.34E-129	-0.0567877	0.0791156	0.4728922	0.8124884
ENSG00000128602	SMO	7	128841049	G	A	0.202783	-0.0251706	0.0176627	0.1499999	-0.0913166	0.0103391	1.03E-18	0.275641	0.195924	0.1594643	0.9705065
ENSG00000128604	IRF5	7	128583877	C	T	0.468191	0.00525282	0.014181	0.6100002	0.608294	0.00777248	0	0.00863533	0.023313	0.7110782	0.4632921
ENSG00000128606	LRRC17	7	102569417	G	C	0.0248509	0.0110322	0.0387669	0.7199992	0.693802	0.0471237	4.59E-49	0.0159011	0.0558864	0.7760083	0.9924177
ENSG00000128607	KLHDC10	7	129741973	T	G	0.436382	0.00915803	0.0144442	0.5700002	-0.352764	0.00856709	0	-0.0259608	0.0409506	0.5261115	0.1814911
ENSG00000128609	NDUFA5	7	123187680	G	A	0.297217	0.0349819	0.0156815	0.03899959	-0.110188	0.0184817	2.49E-09	-0.317475	0.151952	0.03667961	0.4652337
ENSG00000128617	OPN1SW	7	128414194	T	C	0.00497018	-0.0745496	0.088571	0.29	1.37212	0.0680468	2.01E-90	-0.0543316	0.0646066	0.4003699	0.9795819
ENSG00000128626	MRPS12	19	39422495	T	C	0.264414	-0.00981027	0.0159727	0.4799997	-0.0614982	0.0100082	8.01E-10	0.159521	0.26102	0.5411039	0.733789
ENSG00000128641	MYO1B	2	192200013	C	T	0.333002	0.0308734	0.0151652	0.04099964	-0.0932	0.00863139	3.53E-27	-0.33126	0.165583	0.04543991	0.9493352
ENSG00000128654	MTX2	2	177168438	C	T	0.201789	0.00498843	0.016607	0.5199996	0.168676	0.00940756	6.90E-72	0.0295741	0.098469	0.7639186	0.2548664
ENSG00000128656	CHN1	2	175767094	T	C	0.442346	-0.0275534	0.0143887	0.04900044	-0.430521	0.00774623	0	0.0640001	0.0334414	0.05564602	0.1443926
ENSG00000128683	GAD1	2	171693692	C	T	0.0109344	0.0235244	0.049352	0.5400003	0.841121	0.0322056	2.33E-150	0.0279679	0.0586839	0.6336568	0.9776531
ENSG00000128699	ORMDL1	2	190642073	C	T	0.0437376	0.0242752	0.0326964	0.5	-0.23659	0.0176676	6.81E-41	-0.102604	0.138411	0.4585091	0.5241874
ENSG00000128708	HAT1	2	172813778	C	T	0.321074	0.00107584	0.0152158	0.95	-0.218318	0.00842478	4.66E-148	-0.00492785	0.0696958	0.9436324	0.8835566
ENSG00000128731	HERC2	15	28461742	G	C	0.0884692	-0.04905	0.0329608	0.05899973	1.25951	0.0165193	0	-0.0389436	0.0261744	0.136791	0.7077033
ENSG00000128789	PSMG2	18	12714082	T	A	0.4334	-0.00821371	0.0142404	0.3700002	-0.169021	0.0141145	4.80E-33	0.0485959	0.08435	0.5645331	0.3146705
ENSG00000128791	TWSG1	18	9368591	G	A	0.389662	0.000567708	0.0144956	0.91	0.149142	0.0081609	1.30E-74	0.00380649	0.0971934	0.9687596	0.7593298
ENSG00000128805	ARHGAP22	10	49759193	T	G	0.497018	0.0216946	0.0141789	0.09099971	0.359181	0.00771635	0	0.0604001	0.0394969	0.1262059	0.8880473
ENSG00000128815	WDFY4	10	50041961	C	T	0.0815109	-0.0120845	0.0289508	0.7300002	0.915581	0.0158874	0	-0.0131987	0.031621	0.6763828	0.09728495
ENSG00000128829	EIF2AK4	15	40277072	G	T	0.531809	0.00640735	0.0142498	0.5700002	-0.20132	0.00786646	1.86E-144	-0.0318267	0.0707928	0.6530165	0.2625364
ENSG00000128833	MYO5C	15	52536257	A	G	0.026839	0.00455751	0.03416	0.8	0.786057	0.0208388	0	0.00579794	0.0434577	0.8938646	0.5331555
ENSG00000128872	TMOD2	15	52076161	G	C	0.415507	-0.0124641	0.0145086	0.29	0.56896	0.0082031	0	-0.0219068	0.0255022	0.3903308	0.4181727
ENSG00000128881	TTBK2	15	43121969	A	C	0.276342	-0.00532381	0.0154948	0.7099994	-0.10475	0.00871245	2.69E-33	0.050824	0.147982	0.7312625	0.4407891
ENSG00000128891	CCDC32	15	40839069	G	A	0.395626	0.0066379	0.0145452	0.6300007	0.468145	0.00834792	0	0.0141792	0.0310709	0.6481394	0.9532555
ENSG00000128908	INO80	15	41339815	T	C	0.455268	0.00702408	0.0142753	0.6100002	0.0557783	0.00883743	2.76E-10	0.125929	0.256706	0.6237406	0.9020215
ENSG00000128915	ICE2	15	60741583	G	T	0.2833	-0.00957949	0.0156114	0.3700002	0.625985	0.0100352	0	-0.0153031	0.0249401	0.5394851	0.258332
ENSG00000128918	ALDH1A2	15	58517843	G	C	0.241551	-0.0134943	0.0172436	0.4100001	-0.06341	0.0105479	1.84E-09	0.21281	0.274233	0.4377366	0.5039774
ENSG00000128923	MINDY2	15	59108745	C	G	0.421471	-0.00603278	0.0146385	0.6200004	0.225899	0.00797793	2.23E-176	-0.0267057	0.0648079	0.6802854	0.8697922
ENSG00000128928	IVD	15	40712916	A	G	0.467197	0.00340421	0.0142133	0.6899999	-0.430107	0.00773247	0	-0.0079148	0.0330463	0.8107128	0.8323811

ENSG00000128944	KNSTRN	15	40680684	C	T	0.0984095	0.0089748	0.0211329	0.5400003	0.977534	0.011939	0	0.00918106	0.0216189	0.6710714	0.9323991
ENSG00000128951	DUT	15	48629389	A	C	0.16998	-0.0457956	0.0196601	0.01899984	0.314482	0.0107876	7.83E-187	-0.145622	0.0627151	0.02023471	0.003789058
ENSG00000128973	CLN6	15	68524439	G	A	0.441352	-0.0123731	0.014281	0.4299995	-0.593786	0.00724469	0	0.0208376	0.0240521	0.3862958	0.8997314
ENSG00000128989	ARPP19	15	52850661	G	A	0.120278	0.0319237	0.0214896	0.1499999	-0.482536	0.0132654	1.04E-289	-0.0661582	0.0445719	0.1377281	0.09651894
ENSG00000129003	VPS13C	15	62248630	A	G	0.474155	0.0107313	0.0142137	0.5999997	0.265715	0.00782058	4.97E-253	0.0403865	0.0535054	0.4503626	0.6642951
ENSG00000129007	CALML4	15	68490730	G	A	0.440358	-0.0180699	0.0141193	0.2099999	-0.32603	0.00778574	0	0.055424	0.0433269	0.2008252	0.5323869
ENSG00000129028	THAP10	15	71179402	T	C	0.0188867	0.00977391	0.0481289	0.98	-0.251316	0.0289828	4.28E-18	-0.0388909	0.19156	0.8391178	0.9551624
ENSG00000129055	ANAPC13	3	134201053	C	G	0.308151	-0.00508044	0.0151811	0.7300002	-0.647969	0.00807275	0	0.00784056	0.0234289	0.7378871	0.7894889
ENSG00000129071	MBD4	3	129154332	A	G	0.416501	-0.0100567	0.0143259	0.58	-0.0699803	0.0080749	4.46E-18	0.143708	0.205384	0.4841131	0.7658165
ENSG00000129083	COPB1	11	14493279	C	T	0.349901	0.0341981	0.0146142	0.01400006	-0.506264	0.00775312	0	-0.0675499	0.0288853	0.0193583	0.7179386
ENSG00000129103	SUMF2	7	56140029	T	C	0.206759	0.017379	0.0170507	0.3900004	0.311661	0.00901947	1.23E-261	0.0557624	0.0547329	0.3082926	0.4152203
ENSG00000129116	PALLD	4	169633912	T	G	0.217694	-0.0215135	0.0163724	0.2	-0.108342	0.00950151	4.06E-30	0.19857	0.152118	0.1917665	0.8307557
ENSG00000129128	SPCS3	4	177247255	C	G	0.141153	0.0134932	0.0203681	0.3700002	-0.244305	0.0117122	1.26E-96	-0.0552309	0.0834135	0.5078857	0.3767672
ENSG00000129158	SERGEF	11	17922152	C	T	0.417495	-0.0246085	0.0145601	0.1	-0.126849	0.00812188	5.47E-55	0.193998	0.115453	0.09289459	0.7862271
ENSG00000129167	TPH1	11	18051542	G	A	0.104374	0.00868342	0.0239287	0.59	-0.228707	0.0207369	2.77E-28	-0.0379674	0.104682	0.7168357	0.2835827
ENSG00000129187	DCTD	4	183825151	A	C	0.0487078	-0.00765329	0.029118	0.9	0.641856	0.0182225	8.80E-272	-0.0119237	0.0453666	0.7926818	0.6840698
ENSG00000129194	SOX15	17	7492492	A	G	0.0994036	-0.0256985	0.021655	0.32	-0.0950464	0.0130785	3.66E-13	0.270379	0.230854	0.2415139	0.7728289
ENSG00000129195	PIMREG	17	6351262	G	A	0.430417	-0.00117086	0.0146354	0.7899998	-0.0985737	0.0121489	4.91E-16	0.011878	0.148479	0.9362388	0.774234
ENSG00000129197	RPAIN	17	5329578	C	T	0.440358	0.00320637	0.0147317	0.6800001	-0.125752	0.0087813	1.63E-46	-0.0254976	0.117163	0.8277206	0.08973949
ENSG00000129204	USP6	17	5049031	T	C	0.084493	0.0189063	0.0237185	0.4600002	0.353726	0.0167725	9.91E-99	0.0534491	0.0671013	0.4257164	0.695
ENSG00000129219	PLD2	17	4718560	T	G	0.16004	0.025202	0.0204381	0.25	0.553273	0.0116116	0	0.0455507	0.0369527	0.2176965	0.8007718
ENSG00000129226	CD68	17	7484107	A	G	0.296223	0.0155147	0.0155853	0.2599998	-0.184151	0.00867867	6.40E-100	-0.0842499	0.0847264	0.3200397	0.9742223
ENSG00000129235	TXNDC17	17	6545969	C	T	0.343936	0.0230606	0.0146539	0.09200046	-0.290815	0.0149511	2.86E-84	-0.0792965	0.0505538	0.1167509	0.7490939
ENSG00000129244	ATP1B2	17	7555515	C	T	0.129225	0.00826727	0.0218231	0.6700003	0.301159	0.0115684	2.11E-149	0.0274516	0.0724715	0.704843	0.7356855
ENSG00000129245	FXR2	17	7506368	A	G	0.17992	-0.00600285	0.0177868	0.9599999	0.0613951	0.0105157	5.27E-09	-0.0977742	0.290194	0.7361722	0.4120406
ENSG00000129250	KIF1C	17	4916469	C	T	0.124254	-0.00643634	0.0216706	0.6899999	0.150141	0.0116143	3.16E-38	-0.0428687	0.144373	0.7665203	0.527073
ENSG00000129255	MPDU1	17	7491477	A	G	0.0994036	-0.0256985	0.021655	0.32	0.208718	0.0130895	3.07E-57	-0.123126	0.10404	0.2366306	0.9129827
ENSG00000129270	NA	17	34102989	C	A	0.16004	0.00278918	0.0186734	0.91	-0.123167	0.0102562	3.19E-33	-0.0226456	0.151623	0.8812736	0.7549712
ENSG00000129277	NA	17	34431998	T	A	0.241551	-0.00905082	0.0170084	0.4299995	0.389813	0.0136156	2.86E-180	-0.0232184	0.0436397	0.5946939	0.8848709
ENSG00000129282	NA	17	34961704	G	A	0.445328	-0.0204345	0.0142665	0.16	-0.0769121	0.0119544	1.24E-10	0.265686	0.190032	0.1620791	0.5130765
ENSG00000129292	PHF20L1	8	133824335	G	T	0.421471	0.0213773	0.0144128	0.1499999	-0.177554	0.00797443	8.00E-110	-0.120399	0.0813542	0.1388903	0.904464
ENSG00000129295	DNAAF11	8	133636079	T	G	0.451292	-0.0185947	0.0142545	0.17	-0.884967	0.00623542	0	0.0210117	0.0161081	0.192089	0.6073466
ENSG00000129315	CCNT1	12	49096464	G	A	0.315109	-0.0153849	0.0158302	0.2099999	0.151884	0.0113529	8.09E-41	-0.101294	0.1045	0.3323876	0.7172219
ENSG00000129317	PUS7L	12	44137515	G	A	0.0675944	-0.0141747	0.0284089	0.5500004	0.449309	0.0162706	7.39E-168	-0.0315478	0.0632383	0.61787	0.7189896
ENSG00000129347	KRI1	19	10670237	T	C	0.222664	-0.00316051	0.0165346	0.7099994	-0.248828	0.0108168	4.27E-117	0.0127016	0.0664523	0.848417	0.5254796
ENSG00000129351	ILF3	19	10784015	A	G	0.360835	-0.0141031	0.0148873	0.2999998	0.05054	0.00858413	3.92E-09	-0.279049	0.298354	0.3496365	0.7878299
ENSG00000129353	SLC44A2	19	10734184	C	A	0.0914513	0.0235898	0.0247457	0.25	-0.775065	0.0149054	0	-0.0304359	0.0319326	0.3405249	0.4060762
ENSG00000129354	AP1M2	19	10690669	C	G	0.176938	-0.00812562	0.018052	0.5400003	0.325243	0.0107647	1.56E-200	-0.0249832	0.0555092	0.6526578	0.7978576
ENSG00000129355	CDKN2D	19	10678436	C	T	0.49503	0.000237542	0.0142116	0.6499995	0.161489	0.00851769	3.71E-80	0.00147095	0.0880038	0.9866643	0.7189388
ENSG00000129422	MTUS1	8	17579865	C	T	0.0248509	-0.0210858	0.0364382	0.6499995	1.10498	0.0198513	0	-0.0190825	0.032978	0.562831	0.1634016
ENSG00000129437	KLK14	19	51584127	A	G	0.474155	0.0312321	0.014339	0.03099988	0.0566853	0.00805143	1.92E-12	0.550974	0.264787	0.03745063	0.002610616
ENSG00000129450	SIGLEC9	19	51634036	T	G	0.285288	-0.0661361	0.0151688	1.30E-05	0.223788	0.0119696	5.30E-78	-0.29553	0.0696006	2.18E-05	0.02733088
ENSG00000129460	NGDN	14	23958984	A	T	0.156064	-0.0134478	0.018773	0.3800004	-0.137827	0.0111815	6.53E-35	0.0975701	0.136437	0.4745283	0.9553026
ENSG00000129465	RIPK3	14	24807239	G	A	0.0815109	0.0568067	0.0279622	0.064	-0.161033	0.0143069	2.17E-29	-0.352765	0.176449	0.04558143	0.4862782
ENSG00000129467	ADCY4	14	24795927	A	G	0.083499	-0.0430902	0.0290164	0.0990011	0.119843	0.015543	1.25E-14	-0.359557	0.246571	0.1447775	0.09996581
ENSG00000129472	RAB2B	14	21936155	C	T	0.142147	0.00221857	0.0209678	0.99	-0.367784	0.0122159	3.92E-199	-0.00603226	0.0570115	0.9157349	0.9666761
ENSG00000129473	BCL2L2	14	23774483	C	T	0.0298211	0.0344094	0.0437213	0.4400003	-0.275703	0.0397519	4.05E-12	-0.124806	0.159599	0.4342156	0.5473435
ENSG00000129480	DTD2	14	31920979	C	T	0.455268	0.00848812	0.0142284	0.3400001	0.130316	0.00953732	1.67E-42	0.0651349	0.109288	0.5511792	0.8221524
ENSG00000129484	PARP2	14	20818902	C	G	0.256461	0.00412016	0.0163519	0.83	0.267037	0.00891229	3.00E-197	0.0154292	0.0612367	0.8010725	0.4888144
ENSG00000129493	HEATR5A	14	31825391	G	T	0.0318091	-0.00177912	0.031207	0.81	0.187498	0.0185914	6.42E-24	-0.00948876	0.166442	0.9545377	0.9624079

ENSG00000129515	SNX6	14	35064844	T	C	0.405567	0.00906303	0.0143856	0.59	-0.370853	0.00774719	0	-0.0244384	0.038794	0.5287256	0.9134912
ENSG00000129518	EAPP	14	34997025	T	C	0.0238569	0.0613108	0.0440363	0.1900002	-0.627831	0.0272961	4.58E-117	-0.0976549	0.0702687	0.1646094	0.5724529
ENSG00000129521	EGLN3	14	34662708	T	C	0.259443	0.0191781	0.0164756	0.2300001	-0.200203	0.00909997	2.85E-107	-0.0957933	0.0824096	0.2450712	0.9446419
ENSG00000129534	MIS18BP1	14	45697568	T	C	0.124254	-0.0140899	0.0227327	0.56	-0.295086	0.0145477	1.78E-91	0.0477485	0.0770735	0.5355749	0.2456822
ENSG00000129538	RNASE1	14	21270412	A	G	0.0586481	-0.0194928	0.0284181	0.5300002	0.351569	0.0163993	5.91E-102	-0.0554451	0.0808735	0.4929791	0.0296618
ENSG00000129559	NEDD8	14	24693859	C	T	0.186879	0.0117505	0.018237	0.3599996	-0.0718089	0.0105657	1.07E-11	-0.163636	0.255105	0.521233	0.7912042
ENSG00000129562	DAD1	14	23045990	A	G	0.176938	0.0177467	0.0188267	0.3599996	0.492898	0.0111314	0	0.0360048	0.0382046	0.3459771	0.5207675
ENSG00000129566	TEP1	14	20857707	C	T	0.463221	-0.018617	0.014272	0.2200002	-0.19762	0.00808466	5.86E-132	0.0942062	0.0723223	0.1927152	0.194334
ENSG00000129595	EPB41L4A	5	111616575	A	G	0.0894632	0.010202	0.0236924	0.5700002	0.286493	0.0133358	2.25E-102	0.03561	0.0827147	0.6668205	0.7334851
ENSG00000129596	CDO1	5	115146540	A	G	0.151093	-0.00566473	0.019788	0.7899998	0.680547	0.010098	0	-0.00832379	0.0290769	0.7746726	0.1776994
ENSG00000129625	REEP5	5	112235160	T	C	0.405567	0.0293488	0.0144421	0.04799986	-0.27767	0.00863342	5.96E-227	-0.105697	0.0521155	0.04254745	0.3357091
ENSG00000129636	ITFG1	16	47343179	C	A	0.408549	-0.0149872	0.0145142	0.35	-0.11314	0.0121356	1.13E-20	0.132466	0.12907	0.3047441	0.2092142
ENSG00000129646	QRICH2	17	74286945	G	A	0.0308151	0.06095	0.0442883	0.17	0.85389	0.0382197	1.46E-110	0.0713792	0.0519648	0.1695639	0.7027345
ENSG00000129654	FOXJ1	17	74134897	C	T	0.0347913	-0.0232578	0.0521138	0.7600007	-0.190407	0.0306584	5.28E-10	0.122148	0.274403	0.6562179	0.8976354
ENSG00000129657	SEC14L1	17	75147988	T	C	0.354871	-0.0115149	0.0150827	0.2999998	-0.147784	0.00857147	1.30E-66	0.077917	0.102159	0.4456403	0.1337812
ENSG00000129667	RHBDF2	17	74482422	T	C	0.493042	-0.00254575	0.0142423	0.8200001	-0.10919	0.0095394	2.46E-30	0.0233149	0.130452	0.8581545	0.5469738
ENSG00000129673	AANAT	17	74457816	T	C	0.327038	-0.0100987	0.0150623	0.5	0.206236	0.00958847	1.29E-102	-0.0489666	0.0730696	0.5027708	0.551301
ENSG00000129691	ASH2L	8	37982177	C	T	0.0168986	0.0722964	0.222464	0.9400001	-0.551976	0.0450615	1.69E-34	-0.130977	0.403174	0.7452826	0.09357789
ENSG00000129696	TTI2	8	33351011	A	T	0.34493	-0.011659	0.0148257	0.32	-0.101613	0.0159508	1.89E-10	0.114739	0.147011	0.4351082	0.8537394
ENSG00000129749	CHRNA10	11	3689715	T	C	0.43837	0.000313169	0.014387	0.8600001	-0.0845674	0.00804286	7.40E-26	-0.00370319	0.170125	0.9826335	0.6543667
ENSG00000129757	CDKN1C	11	2905777	G	A	0.261431	-0.0167603	0.0162597	0.4400003	-0.115824	0.0138852	7.33E-17	0.144704	0.14145	0.3063047	0.06702058
ENSG00000129925	PGAP6	16	428943	T	C	0.417495	0.00173933	0.0141963	0.6200004	-0.792772	0.00714529	0	-0.00219399	0.0179072	0.9024873	0.04211041
ENSG00000129933	MAU2	19	19450527	A	G	0.152087	0.0121289	0.0187235	0.6200004	-0.940509	0.0104235	0	-0.0128961	0.0199083	0.5171314	0.1034585
ENSG00000129946	SHC2	19	438789	A	G	0.271372	0.028626	0.0159238	0.04799986	0.159633	0.0122002	4.04E-39	0.179324	0.10069	0.07491964	0.3638376
ENSG00000129990	SYT5	19	55687188	T	C	0.17495	-0.011724	0.0188175	0.6100002	0.102575	0.0108729	3.95E-21	-0.114297	0.183851	0.5341505	0.235715
ENSG00000129993	CBFA2T3	16	88992439	T	C	0.190855	-0.0313364	0.0201722	0.06800017	0.208998	0.01321	2.22E-56	-0.149936	0.0969828	0.1221021	0.559941
ENSG00000130005	GAMT	19	1399330	T	C	0.027833	-0.128517	0.0436542	0.002100003	0.500615	0.022438	2.89E-110	-0.256718	0.0879571	0.003515185	0.6513259
ENSG00000130023	ERMARD	6	170166699	T	C	0.124254	-0.0126284	0.0231264	0.6999999	0.272925	0.0272598	1.35E-23	-0.0462706	0.0848614	0.5855819	0.5769321
ENSG00000130024	PHF10	6	170114076	G	T	0.378728	-0.0126634	0.0149042	0.4199997	0.395037	0.0147857	2.94E-157	-0.0320562	0.0377477	0.3957568	0.4978114
ENSG00000130038	CRACR2A	12	3794892	G	A	0.257455	0.00348166	0.0159284	0.8	0.21523	0.00906428	1.24E-124	0.0161765	0.0740097	0.826983	0.8952959
ENSG00000130147	SH3BP4	2	235912487	C	T	0.144135	-0.0230826	0.0198159	0.29	0.114477	0.0116766	1.08E-22	-0.201635	0.174317	0.2473882	0.5857328
ENSG00000130158	DOCK6	19	11341564	A	G	0.185885	-0.00592226	0.0195122	0.64	0.109531	0.0109472	1.44E-23	-0.054069	0.178224	0.7616032	0.06418311
ENSG00000130159	ECSIT	19	11628360	A	G	0.083499	-0.0248392	0.0288174	0.4799997	0.215889	0.0176015	1.39E-34	-0.115055	0.133812	0.3898823	0.8759802
ENSG00000130164	LDLR	19	11222265	G	T	0.161034	0.000199947	0.0207808	0.8700001	0.0915967	0.0120509	2.94E-14	0.00218291	0.226873	0.9923231	0.975798
ENSG00000130165	ELOF1	19	11666006	A	G	0.170974	-0.0148151	0.0201447	0.5400003	0.306831	0.0110164	1.01E-170	-0.0482842	0.0656769	0.4622312	0.4859061
ENSG00000130167	TSPAN16	19	11422248	A	G	0.185885	-0.00592226	0.0195122	0.64	-0.23232	0.0121943	6.37E-81	0.0254918	0.083999	0.7615262	0.0338986
ENSG00000130175	PRKCSH	19	11553946	G	A	0.0994036	0.0142271	0.0284642	0.81	0.256005	0.0154384	9.34E-62	0.0555735	0.111237	0.6173586	0.1868459
ENSG00000130177	CDC16	13	115019280	T	C	0.257455	0.0186437	0.0167365	0.2700001	1.01144	0.00765128	0	0.0184328	0.0165477	0.2653157	0.2369097
ENSG00000130193	THEM6	8	143813483	C	A	0.0168986	-0.0669322	0.0530726	0.3100002	0.941526	0.0345873	3.60E-163	-0.0710891	0.0564292	0.2077441	0.7014628
ENSG00000130201	NA	19	45726674	T	C	0.261431	0.00918623	0.0162489	0.6800001	0.0747114	0.00904321	1.44E-16	0.122956	0.217998	0.5727367	0.2415222
ENSG00000130202	NECTIN2	19	45370958	G	A	0.0606362	-0.0284017	0.0291139	0.4400003	1.53167	0.0147568	0	-0.0185429	0.0190087	0.3293151	0.05440922
ENSG00000130222	GADD45G	9	92220699	C	T	0.418489	0.00567605	0.0143172	0.6700003	-0.123932	0.0119069	2.27E-25	-0.0457998	0.115609	0.6919852	0.2591124
ENSG00000130227	XPO7	8	21820638	A	G	0.427435	-0.0240283	0.0143522	0.09299937	-0.119331	0.00808115	2.41E-49	0.201358	0.121043	0.09620605	0.210437
ENSG00000130244	FAM98C	19	38896751	T	C	0.0119284	0.0366808	0.0521389	0.4799997	0.581973	0.0371166	2.09E-55	0.0630283	0.08968	0.4821719	0.5904711
ENSG00000130254	SAFB2	19	5605533	T	C	0.0427435	-0.0223592	0.0349628	0.3599996	0.212875	0.019959	1.47E-26	-0.105034	0.164536	0.5232345	0.3367967
ENSG00000130255	RPL36	19	5683422	A	G	0.0606362	0.0399006	0.0297552	0.16	0.194562	0.0173335	3.09E-29	0.20508	0.154022	0.1830264	0.456557
ENSG00000130270	ATP8B3	19	1797174	T	C	0.314115	-0.017747	0.0155633	0.25	0.169611	0.0100106	2.16E-64	-0.104633	0.0919663	0.2552303	0.8147204
ENSG00000130299	GTPBP3	19	17449636	G	A	0.342942	-0.00680351	0.0150457	0.99	0.165277	0.00944049	1.26E-68	-0.0411644	0.0910639	0.651241	0.8146028
ENSG00000130300	PLVAP	19	17475208	A	G	0.277336	0.00620151	0.0158574	0.95	0.122964	0.00949133	2.19E-38	0.0504335	0.129018	0.6958698	0.9522029
ENSG00000130303	BST2	19	17515102	C	T	0.0168986	-0.00846929	0.0473747	0.6999999	0.232965	0.0304266	1.91E-14	-0.0363544	0.203411	0.8581546	0.794466

ENSG00000130304	SLC27A1	19	17598277	T	C	0.119284	0.0112861	0.0239592	0.6800001	-0.252988	0.0120312	3.67E-98	-0.0446112	0.0947287	0.6376859	0.1670886
ENSG00000130309	COLGALT1	19	17680187	C	T	0.463221	0.00645317	0.0142541	0.59	-0.369076	0.00848191	0	-0.0174847	0.0386231	0.6507653	0.6110305
ENSG00000130311	DDA1	19	17427218	A	C	0.444334	-0.00616497	0.0142919	0.9199999	0.148487	0.0119167	1.23E-35	-0.0415187	0.0963081	0.6663936	0.7633454
ENSG00000130312	MRPL34	19	17410535	A	G	0.426441	-0.00168137	0.0144191	0.8700001	0.50404	0.00876183	0	-0.00333579	0.0286071	0.9071716	0.932395
ENSG00000130313	PGLS	19	17627267	G	A	0.441352	0.0186876	0.0144622	0.16	-0.115833	0.00927511	8.62E-36	-0.161332	0.12552	0.1986851	0.6250943
ENSG00000130332	LSM7	19	2325067	C	T	0.380716	0.00468503	0.0145023	0.7600007	-0.534053	0.011422	0	-0.0087726	0.0271558	0.74666	0.05003244
ENSG00000130338	TULP4	6	158833276	A	C	0.254473	-0.008928	0.016183	0.5199996	-0.200544	0.00881714	1.62E-114	0.0445189	0.0807192	0.5812719	0.5116207
ENSG00000130340	SNX9	6	158305202	G	C	0.133201	0.00606564	0.0214022	0.8499999	0.0980912	0.0125684	5.97E-15	0.0618367	0.218331	0.7770041	0.7932312
ENSG00000130347	RTN4IP1	6	107048138	A	G	0.383698	-0.00846146	0.0142979	0.6700003	0.3168	0.011776	2.07E-159	-0.0267091	0.0451431	0.5540821	0.3649265
ENSG00000130348	QRSL1	6	107096872	C	A	0.38171	-0.0208509	0.0144037	0.2200002	-0.41709	0.00774169	0	0.0499914	0.0345463	0.1478731	0.8341339
ENSG00000130349	MTRES1	6	107360976	A	G	0.0815109	-0.0182842	0.0295365	0.4400003	-0.171587	0.0244991	2.49E-12	0.106559	0.172808	0.5374765	NA
ENSG00000130363	RSPH3	6	159409265	T	C	0.026839	0.0666176	0.0454778	0.1100001	-1.35449	0.027494	0	-0.0491827	0.0335904	0.1431419	0.7319524
ENSG00000130382	MLLT1	19	6246462	C	T	0.235586	0.0109957	0.0167583	0.4199997	0.0826716	0.00949092	3.02E-18	0.133005	0.203283	0.5129308	0.1556402
ENSG00000130396	AFDN	6	168300152	A	G	0.286282	-0.0262043	0.0155123	0.08500021	0.39504	0.00989904	0	-0.0663333	0.0393028	0.0914596	0.1147528
ENSG00000130402	ACTN4	19	39180256	G	C	0.0636183	-0.0211674	0.0280349	0.4100001	-0.286974	0.0161982	3.13E-70	0.0737606	0.09778	0.4506375	0.3805238
ENSG00000130413	STK33	11	8514627	T	C	0.0616302	0.0560548	0.0275171	0.04399973	-0.305731	0.0168692	2.08E-73	-0.183347	0.0905711	0.04293494	0.04233157
ENSG00000130414	NDUFA10	2	240898343	A	G	0.343936	0.00630159	0.0151812	0.7400005	-0.527968	0.00792437	0	-0.0119355	0.0287546	0.6780807	0.9325548
ENSG00000130429	ARPC1B	7	98982148	C	T	0.116302	0.0177227	0.0234161	0.5300002	0.293489	0.0128149	4.42E-116	0.0603863	0.0798289	0.4493815	0.6227732
ENSG00000130433	CACNG6	19	54505732	G	A	0.579523	0.00382789	0.0145593	0.59	-0.0979562	0.00797403	1.10E-34	-0.0390776	0.148665	0.7926608	0.06277165
ENSG00000130475	FCHO1	19	17878952	G	C	0.227634	0.00639654	0.0175268	0.6999999	-0.440887	0.0143991	6.82E-206	-0.0145084	0.0397563	0.7151624	0.01921958
ENSG00000130479	MAP1S	19	17837688	G	C	0.026839	0.00876397	0.0466049	0.84	0.43009	0.0454199	2.82E-21	0.0203771	0.108382	0.8508679	0.2893518
ENSG00000130487	KLHDC7B	22	50987956	T	C	0.0854871	0.00078507	0.0264527	0.9	-0.202781	0.0145412	3.36E-44	-0.00387153	0.13045	0.9763237	0.2122285
ENSG00000130489	NA	22	50963432	T	G	0.220676	-0.00787013	0.0174063	0.6100002	0.595244	0.0111211	0	-0.0132217	0.0292433	0.6511779	0.1622229
ENSG00000130508	PXDN	2	1692141	C	G	0.491054	-0.0121335	0.0142511	0.5099998	0.589836	0.00890783	0	-0.020571	0.0241631	0.3945819	0.3742879
ENSG00000130511	SSBP4	19	18537523	G	T	0.206759	-0.00635608	0.0175024	0.9400001	-0.190103	0.0164347	6.04E-31	0.0334349	0.0921132	0.7166229	0.8632726
ENSG00000130513	GDF15	19	18492764	A	G	0.246521	-0.0166614	0.0169384	0.2	0.216695	0.0104496	1.60E-95	-0.0768887	0.0782548	0.325833	0.9493452
ENSG00000130517	PGPEP1	19	18466078	T	C	0.285288	0.0131691	0.0161004	0.4199997	-0.172809	0.00966462	1.67E-71	-0.076206	0.093266	0.4138817	0.5862354
ENSG00000130518	IQCN	19	18376612	A	G	0.358847	0.0172032	0.0147339	0.1900002	0.623473	0.00815908	0	0.0275925	0.0236347	0.2430259	0.060494
ENSG00000130520	LSM4	19	18425562	A	G	0.082505	0.00240809	0.0297532	0.99	-0.449203	0.0151023	2.07E-194	-0.00536081	0.0662358	0.9354935	0.6443993
ENSG00000130522	JUND	19	18391497	G	A	0.244533	-0.025331	0.0164582	0.1100001	-0.0943373	0.00970288	2.42E-22	0.268515	0.176634	0.1284654	0.09667295
ENSG00000130529	TRPM4	19	49688045	A	G	0.335984	-0.00593587	0.0151999	0.7300002	0.263865	0.0089848	1.41E-189	-0.0224959	0.05761	0.6961773	0.8130321
ENSG00000130544	ZNF557	19	7078717	G	C	0.301193	0.0116482	0.0156555	0.4199997	-0.10491	0.00958046	6.62E-28	-0.111031	0.149572	0.4578929	0.7198306
ENSG00000130545	CRB3	19	6465510	T	G	0.105368	-0.0020755	0.0259714	0.81	-0.182905	0.0161995	1.46E-29	0.0113474	0.141997	0.9363066	0.2735337
ENSG00000130558	OLFM1	9	137990146	A	C	0.335984	-0.0167918	0.0150859	0.2099999	-0.581415	0.00775887	0	0.0288809	0.0259498	0.2657272	0.738494
ENSG00000130559	CAMSAP1	9	138749703	T	C	0.109344	0.0121657	0.0239118	0.56	-0.312331	0.012986	8.07E-128	-0.0389513	0.0765763	0.6109905	0.7937151
ENSG00000130560	UBAC1	9	138839020	C	T	0.17495	0.000203147	0.0189623	0.8700001	-0.304845	0.0120239	8.28E-142	-0.000666395	0.0622032	0.9914523	0.4151508
ENSG00000130561	SAG	2	234236081	T	C	0.488072	-0.00561867	0.014247	0.7400005	0.116334	0.00819194	9.03E-46	-0.0482979	0.122514	0.6934159	0.7261943
ENSG00000130584	ZBTB46	20	62418808	T	A	0.193837	-0.0284322	0.0182699	0.1199999	0.0972737	0.0110619	1.45E-18	-0.292291	0.190738	0.1254189	0.666082
ENSG00000130589	HELZ2	20	62197515	C	T	0.138171	-0.0148737	0.0220552	0.4899999	-0.170872	0.0144359	2.52E-32	0.0870458	0.129284	0.5007607	0.3389161
ENSG00000130590	SAMD10	20	62608413	T	C	0.173956	-0.0102782	0.018778	0.5500004	0.465296	0.010298	0	-0.0220896	0.04036	0.5841636	0.7281218
ENSG00000130592	LSP1	11	1893848	T	C	0.385686	-0.00528232	0.0147034	0.7499995	0.193746	0.0100382	5.29E-83	-0.0272641	0.0759032	0.7194483	0.1862881
ENSG00000130595	TNNT3	11	1950364	A	G	0.372763	-0.00292829	0.0148851	0.56	-0.422828	0.00902687	0	0.00692548	0.035204	0.8440431	0.5803595
ENSG00000130598	TNNI2	11	1861564	T	C	0.267396	-0.0175324	0.0170075	0.2	0.29389	0.012657	2.89E-119	-0.0596563	0.0579273	0.3030812	0.2940311
ENSG00000130635	COL5A1	9	137635153	A	G	0.119284	0.0148634	0.0213876	0.3400001	-0.140113	0.0134615	2.27E-25	-0.106082	0.152986	0.4880522	0.9854281
ENSG00000130638	ATXN10	22	46154432	A	C	0.166004	0.000415125	0.0190404	0.99	0.306858	0.0106365	5.14E-183	0.00135282	0.0620495	0.9826056	0.1082944
ENSG00000130640	TUBGCP2	10	135109488	C	A	0.105368	0.0499864	0.0227178	0.01899984	-0.506869	0.0213372	9.71E-125	-0.0986181	0.0450118	0.02845611	0.8619759
ENSG00000130643	CALY	10	135144701	C	A	0.0636183	-0.0263302	0.0311105	0.3400001	0.152778	0.0245474	4.85E-10	-0.172343	0.205506	0.4016792	0.6493827
ENSG00000130649	CYP2E1	10	135354317	A	C	0.22167	0.0388524	0.0175133	0.0179999	-0.1495	0.01037	4.07E-47	-0.259883	0.118525	0.02833314	0.5779134
ENSG00000130653	PNPLA7	9	140399695	C	T	0.447316	-0.00762962	0.0145105	0.5700002	0.548241	0.00837695	0	-0.0139166	0.0264682	0.599039	0.1718383
ENSG00000130656	HBZ	16	203594	C	A	0.374751	0.00508236	0.0145957	0.7800007	0.754558	0.00901722	0	0.00673555	0.0193436	0.7276852	0.1479133

ENSG00000130669	PAK4	19	39644933	T	C	0.346918	0.00618268	0.0148971	0.83	-0.215866	0.0082748	5.09E-150	-0.0286412	0.0690195	0.6781615	0.01276912
ENSG00000130684	ZNF337	20	25666164	T	C	0.0606362	0.036552	0.0346206	0.3100002	-0.924066	0.0197861	0	-0.0395556	0.0374751	0.2911885	0.6496982
ENSG00000130695	CEP85	1	26582995	T	A	0.170974	0.0032322	0.0189379	0.84	0.366974	0.0113067	4.43E-231	0.00880772	0.0516063	0.8644823	0.02547933
ENSG00000130699	TAF4	20	60584695	A	G	0.305169	0.0187808	0.0153155	0.1800002	-0.100457	0.00841307	7.27E-33	-0.186953	0.15326	0.2225236	0.7750363
ENSG00000130702	LAMA5	20	60912689	T	C	0.084493	-0.0413556	0.0278305	0.1199999	-0.429962	0.0136839	1.04E-216	0.0961844	0.0648002	0.1377235	0.9244717
ENSG00000130703	OSBPL2	20	60842424	C	G	0.0616302	-0.0401591	0.033655	0.1800002	-0.447485	0.0155348	1.84E-182	0.0897441	0.0752738	0.2331689	0.5074199
ENSG00000130706	ADRM1	20	60880533	C	T	0.027833	0.0541316	0.0460667	0.2300001	-0.271656	0.0277631	1.31E-22	-0.199265	0.170796	0.2433367	0.6961099
ENSG00000130713	EXOSC2	9	133574678	C	T	0.310139	0.0248819	0.0150857	0.05800027	-0.109388	0.00862709	7.67E-37	-0.227465	0.139072	0.1019254	0.905729
ENSG00000130714	POMT1	9	134388741	T	C	0.0596421	0.0301274	0.0338351	0.2599998	0.639926	0.0210138	1.11E-203	0.0470795	0.052896	0.3734454	0.2321291
ENSG00000130717	UCK1	9	134402921	C	T	0.0695825	-0.000775225	0.031637	0.9400001	0.100831	0.0168613	2.23E-09	-0.00768839	0.313767	0.980451	0.1579537
ENSG00000130720	FIBCD1	9	133796249	G	A	0.413519	-0.00420448	0.0148705	0.5999997	0.104036	0.00850701	2.17E-34	-0.0404139	0.142975	0.7774343	0.3301705
ENSG00000130723	NA	9	134322532	G	A	0.0695825	0.000828326	0.031783	0.9	0.348542	0.0207832	4.02E-63	0.00237654	0.0911884	0.979208	0.1293957
ENSG00000130724	CHMP2A	19	59064712	C	T	0.263419	0.0120493	0.0172278	0.5099998	0.0819961	0.00949688	5.92E-18	0.14695	0.210793	0.4857235	0.166526
ENSG00000130725	UBE2M	19	59068703	C	T	0.263419	0.0120493	0.0172278	0.5099998	0.159618	0.0157151	3.08E-24	0.0754884	0.108187	0.4853286	0.1068663
ENSG00000130726	TRIM28	19	59058773	C	T	0.263419	0.0113903	0.0172266	0.5300002	0.149997	0.0094815	2.26E-56	0.0759367	0.114946	0.5088513	0.08179076
ENSG00000130731	METTL26	16	685393	A	C	0.129225	-0.00649904	0.0220928	0.5999997	0.628351	0.0143592	0	-0.010343	0.0351608	0.768633	0.6385816
ENSG00000130733	YIPF2	19	11036400	A	G	0.475149	0.00413466	0.014192	0.7099994	0.0782688	0.0100193	5.64E-15	0.0528264	0.18145	0.7709481	0.8010323
ENSG00000130734	ATG4D	19	10659332	G	A	0.422465	0.00900645	0.0143636	0.4700002	0.0648083	0.00868045	8.27E-14	0.138971	0.222413	0.5320806	0.7539032
ENSG00000130748	TMEM160	19	47550526	A	T	0.229622	-0.0244683	0.0167019	0.1299999	0.134475	0.00973768	2.22E-43	-0.181954	0.124897	0.1451643	0.3729522
ENSG00000130749	ZC3H4	19	47592226	T	C	0.312127	-0.0226375	0.0156756	0.16	0.115672	0.0100002	6.06E-31	-0.195704	0.13657	0.1518583	0.347009
ENSG00000130755	GMFG	19	39826002	G	A	0.112326	-0.0170822	0.0234709	0.4700002	0.311473	0.0254742	2.23E-34	-0.0548432	0.0754879	0.4675216	0.1225215
ENSG00000130758	MAP3K10	19	40709566	A	G	0.333002	-0.0122063	0.0152244	0.5199996	-0.1751	0.00857526	1.13E-92	0.0697104	0.0870139	0.4230496	0.2620221
ENSG00000130764	LRRC47	1	3704926	A	C	0.0934394	0.00449975	0.0266576	0.9299999	0.230897	0.0127867	6.87E-73	0.0194881	0.115457	0.8659613	0.8361296
ENSG00000130766	SESN2	1	28597520	C	T	0.190855	-0.000409003	0.0181908	0.95	-0.117403	0.0102491	2.22E-30	0.00348374	0.154943	0.9820619	0.8896534
ENSG00000130768	SMPDL3B	1	28273586	G	A	0.38171	-0.00753821	0.0147073	0.5199996	-0.156125	0.00817655	2.82E-81	0.0482833	0.0942362	0.6083961	0.8594418
ENSG00000130770	ATP5IF1	1	28568018	G	C	0.286282	0.0149668	0.0155971	0.3100002	-0.282633	0.00867729	1.04E-232	-0.0529549	0.0552089	0.3374719	0.3572275
ENSG00000130775	THEMIS2	1	28206125	A	G	0.367793	-0.00451847	0.0147936	0.7199992	0.362466	0.008853	0	-0.0124659	0.0408149	0.760042	0.8893567
ENSG00000130779	CLIP1	12	122831579	T	C	0.104374	-0.0159736	0.0229487	0.4600002	-0.11003	0.0127651	6.72E-18	0.145175	0.209246	0.4878086	0.2066215
ENSG00000130783	CCDC62	12	123285474	G	A	0.054672	-0.0288913	0.0329162	0.1900002	-0.189036	0.0171108	2.25E-28	0.152835	0.174675	0.3815925	0.8916954
ENSG00000130787	HIP1R	12	123333253	T	C	0.0904573	0.00996209	0.0279627	0.9400001	0.158931	0.0137254	5.24E-31	0.0626817	0.176025	0.7217694	0.7686779
ENSG00000130803	ZNF317	19	9262578	G	T	0.406561	-0.00991184	0.0144131	0.5	0.254622	0.00836359	1.43E-203	-0.0389277	0.0566203	0.4917543	0.1431712
ENSG00000130810	PPAN	19	10221189	G	T	0.400596	0.0176697	0.0143145	0.2399999	-0.0863022	0.0119176	4.43E-13	-0.204742	0.168257	0.2236651	0.3799909
ENSG00000130811	EIF3G	19	10228144	G	A	0.40159	0.0189636	0.0143007	0.2099999	-0.371303	0.0115098	2.58E-228	-0.0510732	0.0385475	0.1851904	0.2966952
ENSG00000130812	ANGPTL6	19	10208243	G	A	0.40159	0.0189636	0.0143007	0.2099999	-0.189611	0.00944477	1.20E-89	-0.100013	0.0755855	0.1857763	0.4500922
ENSG00000130813	SHFL	19	10200363	A	T	0.0864811	-0.0419178	0.0243689	0.04399973	0.393927	0.0145562	2.71E-161	-0.10641	0.0619862	0.08603932	0.9205187
ENSG00000130816	DNMT1	19	10292991	T	A	0.460239	0.00899698	0.0141975	0.6100002	0.203968	0.00791882	2.66E-146	0.0441097	0.0696275	0.5264006	0.7800942
ENSG00000130818	ZNF426	19	9643985	T	A	0.427435	-0.0052648	0.0144139	0.6899999	-0.0569084	0.00805588	1.62E-12	0.0925136	0.253621	0.7152822	0.790199
ENSG00000130844	ZNF331	19	54053879	A	G	0.481113	-0.00210547	0.0141949	0.98	-0.255086	0.00785915	4.29E-231	0.00825396	0.0556481	0.8820869	0.8331946
ENSG00000130856	ZNF236	18	74608623	T	G	0.338966	-0.0122819	0.0148373	0.4500005	-0.155791	0.0082362	8.52E-80	0.0788358	0.0953297	0.4082482	0.03980512
ENSG00000130876	SLC7A10	19	33708163	T	C	0.241551	-0.00862381	0.0170506	0.5700002	0.0828055	0.00927876	4.49E-19	-0.104145	0.206242	0.6135819	0.3592294
ENSG00000130881	LRP3	19	33683601	T	C	0.241551	-0.00862381	0.0170506	0.5700002	0.358958	0.00909946	0	-0.0240245	0.0475041	0.613042	0.7997633
ENSG00000130921	MTRFR	12	123729984	A	C	0.0506958	-0.0212789	0.0385628	0.5400003	0.217354	0.0216195	8.86E-24	-0.0978998	0.177687	0.5816547	0.2907808
ENSG00000130939	UBE4B	1	10167093	G	A	0.139165	-0.0127273	0.0217054	0.4700002	-0.280268	0.0112279	1.59E-137	0.0454112	0.0774666	0.5577385	0.6640408
ENSG00000130940	CASZ1	1	10776684	G	A	0.206759	0.0458101	0.0182433	0.008300036	-0.140525	0.0100654	2.69E-44	-0.325993	0.131906	0.01345822	0.02749774
ENSG00000130943	PKDREJ	22	46655389	G	A	0.161034	0.0174221	0.0200923	0.4400003	-0.151345	0.0116169	8.48E-39	-0.115115	0.133052	0.386935	0.4782111
ENSG00000130948	HSD17B3	9	99031011	C	T	0.280318	-0.0147344	0.0162321	0.4899999	0.129299	0.00882151	1.21E-48	-0.113956	0.12578	0.3649377	0.7159385
ENSG00000130956	HABP4	9	99233050	T	C	0.379722	0.012384	0.0145209	0.4	-0.193698	0.00811592	6.84E-126	-0.0639346	0.0750146	0.39405	0.7311398
ENSG00000130958	SLC35D2	9	99114490	G	T	0.0347913	0.00105162	0.0400677	0.9699999	-0.410981	0.0197642	4.88E-96	-0.0025588	0.0974929	0.9790611	0.8447737
ENSG00000130997	POLN	4	2158746	A	G	0.0675944	-0.0396225	0.0318463	0.0990011	0.276781	0.0160809	2.17E-66	-0.143155	0.11536	0.2146275	0.4878996
ENSG00000131015	ULBP2	6	150266753	G	T	0.217694	-0.0313348	0.0187776	0.04700023	-0.0972948	0.0159762	1.13E-09	0.32206	0.200111	0.1075273	0.7764792

ENSG00000131016	AKAP12	6	151620413	A	G	0.0487078	-0.0596374	0.0366477	0.08199927	0.232388	0.0196506	2.86E-32	-0.256628	0.159186	0.1069347	0.4517537
ENSG00000131018	SYNE1	6	152700877	G	T	0.143141	-0.00749682	0.0215201	0.7600007	0.119921	0.0121132	4.16E-23	-0.0625149	0.179564	0.7277289	0.952224
ENSG00000131037	EPS8L1	19	55591339	A	G	0.298211	0.0018692	0.0156206	0.9599999	0.244796	0.0103156	1.74E-124	0.00763575	0.0638115	0.9047517	0.974106
ENSG00000131042	LILRB2	19	54781357	C	T	0.316103	0.00319984	0.0150621	0.6800001	-0.206796	0.00939758	2.57E-107	-0.0154734	0.0728391	0.831769	0.2499698
ENSG00000131043	AAR2	20	34841610	G	T	0.0318091	0.111573	0.0387319	0.00179999	-1.01929	0.0258456	0	-0.109461	0.0381001	0.004066008	0.8755116
ENSG00000131044	TTLL9	20	30495634	C	T	0.418489	-0.00269359	0.0144589	0.7600007	-0.100164	0.00923412	2.06E-27	0.0268917	0.144373	0.8522366	0.03017838
ENSG00000131051	RBM39	20	34310882	G	A	0.215706	0.00639696	0.0177746	0.8700001	0.123397	0.00958038	5.82E-38	0.0518404	0.1441	0.7190319	0.3415751
ENSG00000131061	ZNF341	20	32349769	T	C	0.176938	0.0231133	0.0190136	0.32	0.11501	0.0104581	3.94E-28	0.200968	0.166328	0.2269473	0.8221522
ENSG00000131067	GGT7	20	33446593	G	C	0.428429	9.82E-05	0.0146295	0.83	0.206454	0.00820917	1.44E-139	0.000475656	0.0708608	0.9946442	0.2732628
ENSG00000131069	ACSS2	20	33487859	T	G	0.112326	-0.00768783	0.0233905	0.4199997	-0.100652	0.0115042	2.15E-18	0.07638	0.232553	0.7425779	0.5808789
ENSG00000131095	GFAP	17	42988340	A	C	0.0854871	0.0233032	0.0232946	0.32	0.104956	0.0127973	2.38E-16	0.222029	0.223592	0.3207053	0.7456824
ENSG00000131097	HIGD1B	17	42925784	A	G	0.198807	-0.025861	0.0171872	0.14	0.0613741	0.0104247	3.92E-09	-0.421367	0.289041	0.1448933	0.482985
ENSG00000131100	ATP6V1E1	22	18093243	C	T	0.139165	0.0122846	0.0184735	0.35	-0.457181	0.0102559	0	-0.0268703	0.0404119	0.5061074	0.6710044
ENSG00000131115	ZNF227	19	44726560	G	A	0.455268	-0.00787302	0.0142382	0.6700003	0.0975364	0.00799715	3.25E-34	-0.0807188	0.146128	0.5806859	0.4725651
ENSG00000131116	ZNF428	19	44117698	A	G	0.147117	0.0127918	0.0196947	0.3400001	0.10211	0.0111248	4.37E-20	0.125275	0.193359	0.5170596	0.485545
ENSG00000131126	TEX101	19	43914205	T	C	0.207753	-0.00361655	0.0169669	0.8800001	0.244942	0.0101475	9.93E-129	-0.0147649	0.0692718	0.8312139	0.308726
ENSG00000131127	ZNF141	4	355128	G	A	0.27336	-0.00648757	0.0158872	0.83	0.0740162	0.0117887	3.42E-10	-0.0876506	0.215098	0.6836474	0.693996
ENSG00000131142	CCL25	19	8122592	G	A	0.373757	0.000380825	0.0146458	0.91	0.0538804	0.00858843	3.53E-10	0.00706797	0.271823	0.9792557	0.1094017
ENSG00000131143	COX4I1	16	85836444	C	T	0.198807	0.00735554	0.0180038	0.89	0.207191	0.0105277	3.16E-86	0.0355012	0.0869133	0.682931	0.2893915
ENSG00000131148	EMC8	16	85819289	C	G	0.0775348	-0.0267543	0.0282981	0.29	-0.409408	0.0159108	5.21E-146	0.0653487	0.0691662	0.3447571	0.1624425
ENSG00000131149	GSE1	16	85677412	G	A	0.124254	0.0122528	0.0221042	0.5999997	-0.121743	0.0168153	4.49E-13	-0.100645	0.182096	0.5804672	0.8306367
ENSG00000131153	GINS2	16	85716741	A	G	0.292247	-0.036783	0.0156949	0.01	0.351501	0.00884529	0	-0.104646	0.0447287	0.01930635	0.9969744
ENSG00000131165	CHMP1A	16	89717546	T	C	0.416501	-0.0263361	0.0144467	0.05	0.185867	0.0092017	9.95E-91	-0.141693	0.078042	0.06943104	0.5423868
ENSG00000131187	F12	5	176832859	T	C	0.224652	0.000366558	0.0163171	0.6700003	-0.0992909	0.0101053	8.74E-23	-0.00369176	0.164337	0.9820774	0.3034178
ENSG00000131196	NFATC1	18	77222590	A	G	0.172962	-0.0218959	0.0185181	0.2099999	0.264584	0.0134312	2.19E-86	-0.082756	0.0701155	0.2378884	0.525551
ENSG00000131203	IDO1	8	39772878	T	C	0.322068	0.00783965	0.0149472	0.6600001	0.293431	0.00910758	9.78E-228	0.0267172	0.0509462	0.5999864	0.6565619
ENSG00000131236	CAP1	1	40522113	A	G	0.23161	-0.00429555	0.0159604	0.7899998	0.224109	0.0132552	3.98E-64	-0.0191673	0.0712263	0.7878497	0.1016864
ENSG00000131238	PPT1	1	40550877	C	T	0.110338	-0.0112952	0.0230645	0.4299995	-1.20536	0.0102914	0	0.00937077	0.019135	0.6243334	0.6951148
ENSG00000131242	RAB11FIP4	17	29791939	G	T	0.0427435	-0.0517493	0.0328616	0.1100001	-0.870348	0.0184489	0	0.0594582	0.0377779	0.1155132	0.3340379
ENSG00000131323	TRAF3	14	103310825	A	G	0.417495	-0.0150332	0.0144678	0.1800002	0.20536	0.00807645	1.27E-142	-0.0732042	0.0705098	0.2991707	0.4346333
ENSG00000131351	HAUS8	19	17173487	T	C	0.399602	-0.0193336	0.0144532	0.1800002	-0.295598	0.00864359	2.55E-256	0.0654051	0.0489322	0.1813379	0.0318092
ENSG00000131355	ADGRE3	19	14765384	T	C	0.0964215	-0.00637617	0.0238747	0.9400001	-0.56696	0.0141115	0	0.0112462	0.0421109	0.7894213	0.4635406
ENSG00000131368	MRPS25	3	15095404	A	C	0.198807	-0.00103976	0.0177545	0.9400001	-0.382811	0.0108818	4.20E-271	0.00271612	0.0463793	0.9533001	0.4386582
ENSG00000131370	SH3BP5	3	15339617	T	G	0.390656	-0.00817213	0.0149322	0.59	0.10505	0.00810859	2.19E-38	-0.0777927	0.14227	0.5845198	0.5047105
ENSG00000131373	HACL1	3	15622774	G	T	0.112326	-0.0225119	0.0225648	0.2599998	-0.165656	0.0181624	7.46E-20	0.135896	0.137027	0.3213242	0.1158538
ENSG00000131374	TBC1D5	3	17843104	A	G	0.335984	0.0255567	0.015392	0.08799946	0.0778129	0.00900779	5.70E-18	0.328438	0.201429	0.1029871	0.3604673
ENSG00000131375	CAPN7	3	15271042	A	G	0.156064	0.00847502	0.019543	0.6999999	0.0748253	0.0112516	2.93E-11	0.113264	0.261736	0.6652029	0.6974512
ENSG00000131378	RFTN1	3	16456442	G	A	0.397614	-0.00337287	0.0144698	0.8700001	-0.164252	0.00801009	1.92E-93	0.0205348	0.088101	0.8156976	0.8195661
ENSG00000131379	C3orf20	3	14765573	A	G	0.351889	0.0245348	0.0149684	0.04099964	-0.178733	0.00850803	5.59E-98	-0.13727	0.0840016	0.1022295	0.8411462
ENSG00000131381	RBSN	3	15126125	T	C	0.0328032	-0.041485	0.0341578	0.2099999	0.337522	0.0198741	1.10E-64	-0.122911	0.10146	0.2257358	0.9180945
ENSG00000131389	SLC6A6	3	14487466	C	G	0.148111	-0.0181617	0.0195918	0.35	0.450199	0.0105302	0	-0.0403415	0.0435283	0.354037	0.7585325
ENSG00000131398	KCNC3	19	50825983	G	A	0.331014	-0.001913	0.0151082	0.7899998	0.0983406	0.00856312	1.58E-30	-0.0194528	0.153641	0.8992473	0.8514361
ENSG00000131400	NAPSA	19	50865410	C	T	0.324056	0.00402268	0.015123	0.9	-0.223222	0.012629	6.49E-70	-0.018021	0.0677563	0.7902643	0.6494366
ENSG00000131401	NA	19	50842538	G	A	0.306163	-0.000806022	0.0154977	0.8700001	-0.917984	0.00770307	0	0.000878035	0.0168823	0.9585214	0.7184583
ENSG00000131408	NR1H2	19	50859574	T	C	0.0874751	0.0252071	0.0248332	0.32	0.134503	0.0157856	1.59E-17	0.187409	0.185935	0.3134883	0.5069565
ENSG00000131409	LRRC4B	19	51045725	G	A	0.315109	0.0279795	0.0152405	0.07000032	0.125712	0.00956146	1.75E-39	0.222568	0.122409	0.06902971	0.3146268
ENSG00000131437	KIF3A	5	132050825	C	A	0.105368	-0.00737908	0.025338	0.7400005	-0.169005	0.0130941	4.11E-38	0.0436619	0.149963	0.7709353	0.9697669
ENSG00000131446	MGAT1	5	180230096	T	C	0.224652	0.00446808	0.0184192	0.8700001	0.398089	0.0101253	0	0.0112238	0.0462699	0.8083364	0.4175418
ENSG00000131459	GFPT2	5	179754038	G	A	0.200795	-0.0326963	0.0183305	0.03799969	0.110205	0.0107272	9.29E-25	-0.296687	0.16882	0.0788466	0.06305834
ENSG00000131470	PSMC3IP	17	40727091	G	C	0.266402	0.0120152	0.0157297	0.32	0.0901918	0.00896325	8.10E-24	0.133218	0.174904	0.4462612	0.07826272

ENSG00000131473	ACLY	17	40054978	A	G	0.161034	0.013298	0.019223	0.4100001	-0.195424	0.0108784	3.70E-72	-0.068047	0.0984386	0.4894002	0.7149106
ENSG00000131475	VPS25	17	40928535	A	G	0.00497018	0.176702	0.171254	0.4400003	-0.383814	0.0523264	2.22E-13	-0.460384	0.450583	0.306898	0.4597142
ENSG00000131484	NA	17	43596351	T	C	0.242545	0.0186779	0.0171994	0.4299995	-0.610969	0.0271795	6.66E-112	-0.030571	0.0281839	0.2780558	0.4614785
ENSG00000131495	NDUFA2	5	140022847	C	T	0.403579	0.00263956	0.0143997	0.99	-0.33236	0.00948257	3.95E-269	-0.00794187	0.0433262	0.8545594	0.7205588
ENSG00000131504	DIAPH1	5	140946602	C	T	0.112326	-0.0395791	0.020712	0.02199986	-0.0931236	0.0112667	1.39E-16	0.425017	0.228281	0.06262913	0.3931386
ENSG00000131507	NDFIP1	5	141511039	C	A	0.493042	-0.00749589	0.0141963	0.7199992	0.236445	0.00783351	3.86E-200	-0.0317024	0.0600497	0.5975435	0.6618958
ENSG00000131558	EXOC4	7	133344585	A	G	0.0347913	-0.0563485	0.0383139	0.08100093	-0.390171	0.0187936	9.79E-96	0.14442	0.0984439	0.1423679	0.2422944
ENSG00000131584	ACAP3	1	1236372	C	T	0.084493	-0.00469683	0.0286742	0.8800001	-0.104248	0.0177444	4.23E-09	0.0450542	0.275163	0.8699388	0.8587505
ENSG00000131591	C1orf159	1	1034469	A	G	0.166004	0.0195754	0.0193937	0.3700002	-0.192026	0.0110138	4.48E-68	-0.101941	0.101164	0.3136073	0.9180599
ENSG00000131626	PPFIA1	11	70173657	C	G	0.350895	0.0255851	0.0148622	0.08199927	-0.493134	0.00790407	0	-0.0518826	0.0301497	0.08528111	0.7833147
ENSG00000131634	TMEM204	16	1592135	C	T	0.12326	-0.0128612	0.021002	0.3100002	1.0578	0.0103141	0	-0.0121584	0.0198547	0.5402937	0.1685433
ENSG00000131650	KREMEN2	16	3016164	A	G	0.287276	-0.0134041	0.0153393	0.3599996	0.0598145	0.00873306	7.43E-12	-0.224095	0.258527	0.3860439	0.1421248
ENSG00000131652	THOC6	16	3075892	C	G	0.365805	0.0301334	0.0144997	0.05399953	0.0768297	0.0106256	4.81E-13	0.39221	0.196366	0.04578765	0.294895
ENSG00000131669	NINJ1	9	95890170	C	T	0.187873	-0.00151589	0.0178453	0.9699999	-0.517287	0.00984246	0	0.00293046	0.0344979	0.9323043	0.8064016
ENSG00000131686	CA6	1	9020536	G	A	0.0646123	-0.0123264	0.0296065	0.5199996	0.619622	0.0156875	0	-0.0198934	0.0477842	0.6771774	0.5327614
ENSG00000131697	NPHP4	1	5987702	A	G	0.452286	0.00998246	0.014391	0.3800004	0.314292	0.00787531	0	0.0317617	0.0457955	0.4879612	0.5775321
ENSG00000131711	MAP1B	5	71454228	A	C	0.400596	-0.011627	0.0148371	0.3400001	-0.0614913	0.0082201	7.40E-14	0.189084	0.242608	0.4357565	0.3828732
ENSG00000131730	CKMT2	5	80545660	T	C	0.112326	-0.0133571	0.0241501	0.4400003	-0.248977	0.0135955	6.49E-75	0.053648	0.0970417	0.5803766	0.5873067
ENSG00000131732	ZCCHC9	5	80603262	C	T	0.210736	0.0155757	0.0170551	0.3599996	0.0972776	0.0101135	6.67E-22	0.160116	0.176113	0.3632611	0.6908729
ENSG00000131746	TNS4	17	38644964	C	T	0.522863	0.001212	0.0142416	0.9699999	0.0651417	0.00864499	4.88E-14	0.0186056	0.218639	0.932184	0.3509772
ENSG00000131748	STARD3	17	37806527	G	T	0.0109344	0.0487959	0.0562203	0.4700002	-0.554114	0.0368045	3.17E-51	-0.0880612	0.101628	0.3862148	0.628446
ENSG00000131759	RARA	17	38489269	C	T	0.126243	0.00533579	0.0213207	0.9199999	0.13223	0.0114171	5.10E-31	0.0403523	0.161277	0.8024289	0.9550869
ENSG00000131771	PPP1R1B	17	37787936	A	C	0.196819	-0.00195601	0.0183712	0.7600007	0.155617	0.0106286	1.53E-48	-0.0125694	0.118057	0.9152105	0.07211605
ENSG00000131773	KHDRBS3	8	136569332	T	C	0.0168986	0.0554004	0.0470657	0.2300001	0.303404	0.0303477	1.56E-23	0.182596	0.156197	0.2423988	0.7539115
ENSG00000131778	CHD1L	1	146740867	A	T	0.315109	-0.0080584	0.0152108	0.6200004	0.261091	0.00854117	3.21E-205	-0.0308644	0.0582674	0.5963186	0.9619352
ENSG00000131781	FMO5	1	146680815	A	G	0.0407555	0.0105987	0.0369267	0.5199996	0.68734	0.0376996	2.88E-74	0.0154199	0.0537308	0.7741243	0.3993176
ENSG00000131788	PIAS3	1	145580889	C	T	0.43837	0.010471	0.0144946	0.4899999	-0.0556755	0.00884187	3.04E-10	-0.188072	0.262048	0.4729428	0.5976958
ENSG00000131791	PRKAB2	1	146635407	A	G	0.434394	-0.0031177	0.0144486	0.95	-0.349942	0.0117366	2.41E-195	0.00890918	0.0412896	0.8291647	0.8429731
ENSG00000131795	NA	1	145509521	A	G	0.0318091	-0.0487185	0.0413567	0.2999998	-0.530113	0.0478649	1.66E-28	0.0919021	0.0784549	0.2414381	0.4562171
ENSG00000131797	NA	16	31716504	C	T	0.32505	-0.0418989	0.0156276	0.002900013	0.804132	0.00842847	0	-0.0521045	0.0194418	0.007361656	0.6571634
ENSG00000131844	MCCC2	5	70918823	C	G	0.45825	0.0112061	0.0142285	0.33	0.191352	0.0117838	2.69E-59	0.0585628	0.0744452	0.4314829	0.1808779
ENSG00000131845	ZNF304	19	57866970	T	C	0.289264	0.0198976	0.0159737	0.3100002	-0.155895	0.00887927	5.24E-69	-0.127635	0.102722	0.2140432	0.5417664
ENSG00000131848	ZSCAN5A	19	56806216	T	C	0.0218688	-0.00282722	0.054842	0.8	-0.217709	0.0338331	1.24E-10	0.0129862	0.251913	0.9588869	0.5691499
ENSG00000131849	ZNF132	19	58947885	T	C	0.498012	0.00360526	0.0142043	0.8	0.462433	0.00750918	0	0.00779628	0.0307167	0.7996404	0.2642806
ENSG00000131871	SELENOS	15	101814363	A	G	0.315109	-0.000208899	0.0151057	0.98	0.140516	0.00934393	4.13E-51	-0.00148666	0.107502	0.9889663	0.7489595
ENSG00000131873	CHSY1	15	101754032	T	C	0.0984095	-0.0307789	0.0229418	0.3700002	-0.8327	0.0120956	0	0.0369628	0.0275563	0.1798058	0.5344029
ENSG00000131876	SNRPA1	15	101828601	A	G	0.33002	-0.00247091	0.0151274	0.8700001	-0.0822967	0.0125813	6.10E-11	0.0300244	0.183873	0.8702908	0.3842624
ENSG00000131885	NA	17	16746627	A	G	0.0367793	-0.0416284	0.0391279	0.2200002	0.695494	0.0485938	1.83E-46	-0.0598544	0.0564144	0.2886996	0.2391067
ENSG00000131899	LLGL1	17	18138545	G	A	0.0984095	-0.0226594	0.0276163	0.2999998	0.109142	0.0154077	1.40E-12	-0.207613	0.254722	0.4150388	0.1484358
ENSG00000131931	THAP1	8	42695142	A	G	0.0198807	-0.0796923	0.0512706	0.17	0.243235	0.030134	6.93E-16	-0.327634	0.214658	0.1269337	0.4156351
ENSG00000131941	RHPN2	19	33512646	A	C	0.196819	-0.00849143	0.0182437	0.7899998	-0.217178	0.0108471	3.56E-89	0.0390989	0.084026	0.6417032	0.9996425
ENSG00000131943	C19orf12	19	30199042	C	T	0.240557	0.00718843	0.0166724	0.7800007	-0.557158	0.0085016	0	-0.012902	0.0299246	0.6663607	0.3842653
ENSG00000131944	FAAP24	19	33466121	G	A	0.263419	0.00223251	0.015447	0.9299999	0.43698	0.00828383	0	0.00510895	0.0353496	0.8850847	0.7055764
ENSG00000131966	ACTR10	14	58684274	A	G	0.0427435	-0.0269939	0.0387877	0.4400003	-1.00414	0.035085	3.78E-180	0.0268827	0.0386393	0.4865954	0.6231172
ENSG00000131969	ABHD12B	14	51355283	G	A	0.203777	0.0134448	0.0179606	0.3400001	-0.415196	0.00990014	0	-0.0323818	0.043265	0.4541872	0.3111501
ENSG00000131979	GCH1	14	55339148	T	C	0.082505	-0.0239239	0.0225264	0.2999998	-0.481874	0.0122779	0	0.0496477	0.0467646	0.2883947	0.4329765
ENSG00000131981	LGALS3	14	55601477	G	C	0.0974155	-0.0167888	0.0246173	0.3700002	-0.103	0.013869	1.11E-13	0.162999	0.240009	0.4970527	0.5314795
ENSG00000132002	DNAJB1	19	14633082	T	C	0.148111	-0.0107413	0.0199994	0.4899999	0.230559	0.0169406	3.50E-42	-0.0465882	0.0868107	0.5915001	0.8303903
ENSG00000132003	ZSWIM4	19	13924659	A	G	0.203777	-0.00603096	0.0179023	0.6499995	0.159206	0.0116813	2.69E-42	-0.0378815	0.112482	0.7362835	0.3112075
ENSG00000132004	FBXW9	19	12803162	T	C	0.0536779	-0.0554843	0.0302629	0.06100002	0.1547	0.0177478	2.87E-18	-0.358657	0.199903	0.07278893	0.03625817

ENSG00000132010	ZNF20	19	12227440	G	C	0.127237	-0.0080962	0.0229334	0.6499995	0.14821	0.0126803	1.46E-31	-0.0546264	0.154806	0.7241859	0.3028818
ENSG00000132016	BRME1	19	14005213	A	C	0.21173	-0.00997211	0.0171132	0.56	-0.0895313	0.0107038	6.04E-17	0.111381	0.191605	0.5610345	0.5329306
ENSG00000132024	CC2D1A	19	14029353	A	G	0.212724	-0.0140151	0.0174384	0.3900004	-0.112954	0.0107502	8.00E-26	0.124078	0.154836	0.4229284	0.9517427
ENSG00000132109	TRIM21	11	4410526	T	C	0.12326	-0.015003	0.0210532	0.4600002	-0.177817	0.0110915	7.66E-58	0.084373	0.118515	0.4765141	0.2227442
ENSG00000132122	SPATA6	1	48849444	T	C	0.0188867	0.0458996	0.0464535	0.35	-0.39379	0.0268403	9.80E-49	-0.116559	0.118232	0.3242101	0.9685979
ENSG00000132128	LRRC41	1	46748074	T	C	0.0159046	0.0490374	0.0478613	0.3100002	-0.247253	0.0306241	6.81E-16	-0.198329	0.195125	0.3094287	0.7451293
ENSG00000132141	CCT6B	17	33281487	C	T	0.422465	0.0176257	0.0143079	0.1499999	0.215769	0.0119418	5.65E-73	0.0816879	0.0664652	0.2190596	0.5240046
ENSG00000132142	NA	17	35604416	T	C	0.403579	-0.0086205	0.0143636	0.4400003	-0.13121	0.00806922	1.88E-59	0.0656999	0.109545	0.5486698	0.4332048
ENSG00000132155	RAF1	3	12665412	G	A	0.432406	-0.0102598	0.0143159	0.6700003	0.153027	0.00810496	1.65E-79	-0.0670459	0.0936191	0.4738951	0.9143496
ENSG00000132170	PPARG	3	12402361	A	G	0.426441	0.0080337	0.0144096	0.6600001	0.246549	0.0078689	1.71E-215	0.0325846	0.0584544	0.5772296	0.824775
ENSG00000132182	NUP210	3	13409773	G	T	0.512922	-0.00992088	0.0142381	0.4500005	0.312402	0.00846341	2.97E-298	-0.0317568	0.0455844	0.4860157	0.4359687
ENSG00000132185	FCRLA	1	161680452	C	T	0.147117	-0.0602961	0.0212184	0.0016	0.184227	0.011666	3.54E-56	-0.327293	0.117025	0.005161643	0.4624088
ENSG00000132196	HSD17B7	1	162771549	C	T	0.218688	-0.0134403	0.017365	0.4	0.405232	0.0218078	4.49E-77	-0.0331669	0.0428891	0.4393346	0.4025381
ENSG00000132199	ENOSF1	18	692609	T	C	0.457256	0.000953214	0.0142515	0.8	0.43311	0.00832593	0	0.00220086	0.0329051	0.9466732	0.1111044
ENSG00000132204	NA	18	1331364	G	A	0.465209	-0.0267601	0.0143181	0.07699987	0.0553507	0.00908818	1.13E-09	-0.483465	0.270586	0.07398048	0.2510859
ENSG00000132205	EMILIN2	18	2881509	T	C	0.297217	0.015017	0.0156182	0.3700002	-0.218115	0.00927912	3.54E-122	-0.068849	0.0716652	0.3367015	0.04821203
ENSG00000132256	TRIM5	11	5822137	C	T	0.135189	-0.00691533	0.0224099	0.6899999	-0.529887	0.0131674	0	0.0130506	0.0422931	0.7576451	0.1939905
ENSG00000132259	CNGA4	11	6260827	G	A	0.0188867	0.0430467	0.0470764	0.3800004	0.472748	0.032703	2.30E-47	0.0910564	0.0997795	0.3614651	0.3839146
ENSG00000132274	TRIM22	11	5734619	G	T	0.340954	7.92E-05	0.0146818	0.99	0.160409	0.00806366	4.70E-88	0.000493489	0.0915274	0.9956981	0.3997994
ENSG00000132275	RRP8	11	6620577	A	G	0.39662	-0.0210787	0.0150457	0.07399971	-0.24753	0.00828729	5.03E-196	0.0851562	0.0608502	0.1616811	0.4633292
ENSG00000132286	TIMM10B	11	6504293	G	A	0.354871	-0.0032475	0.0151164	0.6999999	-0.171376	0.00929048	5.57E-76	0.0189496	0.0882121	0.829909	0.3518558
ENSG00000132294	EFR3A	8	132971112	A	G	0.481113	0.00326366	0.0142389	0.8200001	-0.114464	0.00873572	3.17E-39	-0.0285125	0.124415	0.818735	0.6666726
ENSG00000132305	IMMT	2	86396974	G	A	0.522863	-0.00417581	0.0141755	0.6499995	0.533306	0.00737329	0	-0.00783005	0.0265807	0.7683171	0.787541
ENSG00000132313	MRPL35	2	86433748	C	T	0.0755467	0.02806	0.026899	0.28	0.610989	0.0159946	0	0.0459256	0.0440418	0.2970536	0.7186182
ENSG00000132323	ILKAP	2	239095706	C	T	0.248509	-0.0194931	0.0165034	0.1900002	0.132122	0.00965	1.14E-42	-0.147539	0.125374	0.2392811	0.973644
ENSG00000132326	PER2	2	239175711	A	G	0.233598	-0.0299972	0.0185251	0.07499978	-0.0608242	0.0103109	3.66E-09	0.493178	0.315834	0.1184031	0.1128614
ENSG00000132329	RAMP1	2	238794146	T	G	0.447316	-0.0155724	0.0142882	0.2399999	-0.207868	0.00788737	4.56E-153	0.074915	0.0687958	0.276177	0.9834109
ENSG00000132330	SCLY	2	238988792	A	G	0.298211	0.0220455	0.0151657	0.1	0.0925416	0.00919568	8.00E-24	0.238223	0.165581	0.1502325	0.5466402
ENSG00000132334	PTPRE	10	129794722	G	C	0.326044	0.00181589	0.0151719	0.9	-0.32535	0.00830369	0	-0.00558134	0.0466328	0.904731	0.4699372
ENSG00000132341	RAN	12	131359323	G	A	0.140159	-0.0474325	0.0222394	0.02199986	0.138116	0.012429	1.09E-28	-0.343426	0.163959	0.03620806	0.5935773
ENSG00000132356	PRKAA1	5	40778978	T	C	0.28827	0.00758631	0.0160507	0.64	0.0918401	0.00902534	2.54E-24	0.0826035	0.174956	0.6368285	0.6079344
ENSG00000132357	CARD6	5	40850780	G	A	0.0318091	-0.011152	0.0460505	0.6200004	0.359056	0.0268734	1.02E-40	-0.0310593	0.128276	0.8086801	0.2475348
ENSG00000132359	RAP1GAP2	17	2810691	C	T	0.49006	-0.00603693	0.0146931	0.6200004	0.12226	0.00825155	1.14E-49	-0.0493778	0.120225	0.6812848	0.2747057
ENSG00000132361	CLUH	17	2604318	C	T	0.0695825	-0.00230924	0.0264371	0.8800001	0.135884	0.0223884	1.28E-09	-0.0169942	0.194577	0.9304017	0.9223352
ENSG00000132376	INPP5K	17	1409023	T	C	0.0646123	-0.00863387	0.0266688	0.9400001	-0.648484	0.0170888	0	0.0133139	0.0411263	0.7461406	0.4099474
ENSG00000132382	MYBBP1A	17	4450559	C	A	0.199801	-0.00176258	0.0174173	0.8800001	-0.160735	0.0103767	4.05E-54	0.0109657	0.108362	0.9193957	0.9323537
ENSG00000132383	RPA1	17	1768186	C	T	0.377734	-0.00557966	0.0144012	0.7099994	-0.510437	0.00756433	0	0.0109311	0.0282139	0.6984322	0.9028057
ENSG00000132386	SERPINF1	17	1673060	C	T	0.265408	0.00819633	0.0160392	0.8700001	-0.130894	0.00898379	4.36E-48	-0.0626182	0.122611	0.6095579	0.6192868
ENSG00000132388	UBE2G1	17	4221238	A	G	0.0238569	-0.0430614	0.0463035	0.2999998	0.320431	0.0305924	1.13E-25	-0.134386	0.145072	0.3542707	0.3467713
ENSG00000132394	EEFSEC	3	127999891	T	A	0.263419	-0.0122484	0.0160051	0.33	-0.15086	0.00875321	1.46E-66	0.0811906	0.106197	0.4445528	0.1576851
ENSG00000132405	TBC1D14	4	6972907	T	C	0.27336	0.0252503	0.0159292	0.16	-0.41032	0.00854048	0	-0.0615381	0.0388425	0.1131264	0.5165606
ENSG00000132406	TMEM128	4	4243609	C	T	0.342942	0.00638689	0.0148527	0.4400003	0.184115	0.0138518	2.58E-40	0.0346897	0.0807131	0.667348	0.9041176
ENSG00000132423	COQ3	6	99829678	A	T	0.0675944	-0.0245487	0.0311792	0.4899999	0.107648	0.0171886	3.78E-10	-0.228045	0.291919	0.4346889	0.5439565
ENSG00000132434	LANCL2	7	55467288	A	G	0.298211	-0.00666554	0.015441	0.6800001	0.148478	0.00852432	6.00E-68	-0.0448924	0.104027	0.6660718	0.7804728
ENSG00000132436	FIGNL1	7	50514959	C	T	0.542744	-0.00906238	0.0141796	0.6600001	-0.457075	0.00753049	0	0.0198269	0.0310242	0.5227719	0.9967759
ENSG00000132463	GRSF1	4	71693580	A	G	0.0149105	-0.0191102	0.0573977	0.7199992	-0.221212	0.0375342	3.78E-09	0.0863887	0.259883	0.7395769	0.561271
ENSG00000132466	ANKRD17	4	74031804	T	G	0.415507	0.0240748	0.0143893	0.07100027	0.0498227	0.0081261	8.72E-10	0.483209	0.29937	0.1065087	0.3002491
ENSG00000132471	WBP2	17	73847184	T	C	0.0178926	-0.0384166	0.0576	0.5	-0.638266	0.0344446	1.18E-76	0.060189	0.0903029	0.5050767	0.1023298
ENSG00000132475	H3-3B	17	73777244	C	G	0.311133	0.0243616	0.015532	0.14	-0.119711	0.00899312	1.99E-40	-0.203503	0.130643	0.1193039	0.1445152
ENSG00000132478	UNK	17	73801283	A	G	0.0387674	0.0147785	0.0366254	0.6800001	-0.302104	0.0225802	8.00E-41	-0.0489185	0.121289	0.6867123	0.7665967

ENSG00000132481	TRIM47	17	73872449	T	C	0.135189	0.00487414	0.020317	0.9599999	-0.270103	0.0110749	2.25E-131	-0.0180455	0.0752232	0.8104133	0.8198313
ENSG00000132485	ZRANB2	1	71537977	T	C	0.450298	-0.001209	0.0142211	0.9599999	0.0804328	0.00848877	2.66E-21	-0.0150312	0.176814	0.9322526	0.1866796
ENSG00000132507	EIF5A	17	7213046	G	A	0.441352	-0.0108584	0.0143861	0.2300001	0.286253	0.0117469	3.70E-131	-0.0379329	0.0502807	0.4505957	0.2485984
ENSG00000132510	KDM6B	17	7750668	G	A	0.0695825	0.0122798	0.0268764	0.5199996	-0.247999	0.0167192	8.94E-50	-0.0495155	0.108424	0.6478993	0.7921971
ENSG00000132514	CLEC10A	17	6980741	A	G	0.291252	-0.00545791	0.0155838	0.6499995	0.313032	0.00868984	3.68E-284	-0.0174356	0.0497857	0.7261787	0.4073151
ENSG00000132518	GUCY2D	17	7914784	T	C	0.511928	0.000840659	0.0147831	0.98	0.0754246	0.00797789	3.26E-21	0.0111457	0.196002	0.9546526	0.4466613
ENSG00000132530	XAF1	17	6668866	G	A	0.361829	0.0109052	0.0147269	0.4500005	0.103389	0.0081072	3.01E-37	0.105477	0.142681	0.4597551	0.1712423
ENSG00000132541	RIDA	8	99122020	A	G	0.40159	-0.0177075	0.0143964	0.28	0.222799	0.0079142	2.28E-174	-0.0794774	0.0646777	0.2191383	0.1977138
ENSG00000132549	VPS13B	8	100457651	T	C	0.197813	-0.00192769	0.0180565	0.98	-0.0674015	0.0100035	1.61E-11	0.0286001	0.267928	0.914991	0.7512953
ENSG00000132561	MATN2	8	98965006	C	T	0.152087	0.0155873	0.0193644	0.3599996	-0.368847	0.0110062	3.14E-246	-0.0422595	0.052515	0.4209847	0.2427469
ENSG00000132570	PCBD2	5	134292122	G	A	0.138171	0.0357383	0.0230683	0.0659994	0.0867312	0.0136606	2.17E-10	0.412058	0.273778	0.1323039	0.9797483
ENSG00000132589	FLOT2	17	27215525	C	T	0.324056	0.0266158	0.0150941	0.1299999	0.0910066	0.00841454	2.91E-27	0.29246	0.168047	0.08179829	0.7981347
ENSG00000132591	ERAL1	17	27185020	T	C	0.193837	0.000128178	0.0176055	0.99	-0.21736	0.00975022	4.34E-110	-0.000589704	0.0809971	0.994191	0.2071068
ENSG00000132600	PRMT7	16	68368671	T	C	0.11332	-0.0392407	0.0239601	0.0530005	0.420251	0.0130767	1.32E-226	-0.0933744	0.0570878	0.1019172	0.9782434
ENSG00000132603	NIP7	16	69375173	A	G	0.286282	0.0228163	0.0151681	0.08400014	-0.315205	0.00848711	6.53E-302	-0.0723856	0.0481608	0.1328398	0.07081306
ENSG00000132604	TERF2	16	69415969	T	C	0.247515	0.0256033	0.0157006	0.06800017	-0.192535	0.00909849	2.18E-99	-0.13298	0.0817886	0.1039708	0.2454727
ENSG00000132612	VPS4A	16	69346255	G	A	0.286282	0.0257662	0.0151676	0.04900044	0.0941452	0.00865194	1.41E-27	0.273686	0.16306	0.09326217	0.1684131
ENSG00000132622	HSPA12B	20	3723536	C	T	0.0596421	-0.00503947	0.0304536	0.9199999	0.433895	0.0178855	5.24E-130	-0.0116145	0.0701881	0.868569	0.9968787
ENSG00000132623	ANKEF1	20	10026549	G	C	0.191849	0.00497165	0.018299	0.81	-0.24543	0.0113621	1.77E-103	-0.0202569	0.074565	0.7858771	0.5789841
ENSG00000132635	PCED1A	20	2818898	T	G	0.499006	-0.00203928	0.0141924	0.83	0.160614	0.00876821	5.97E-75	-0.0126968	0.088366	0.8857502	0.5931304
ENSG00000132640	BTBD3	20	11889314	T	C	0.317097	0.00964531	0.0150612	0.3800004	0.0951296	0.00836996	6.20E-30	0.101391	0.158574	0.5225669	0.898054
ENSG00000132646	PCNA	20	5101435	T	G	0.0924453	0.00987665	0.0226064	0.6499995	-0.186617	0.0143866	1.77E-38	-0.0529246	0.121206	0.6623662	0.3218887
ENSG00000132661	NXT1	20	23333393	C	G	0.154076	0.00283772	0.0221076	0.99	-0.142895	0.0119069	3.51E-33	-0.0198588	0.154721	0.8978703	0.3043882
ENSG00000132664	POLR3F	20	18456529	T	C	0.0745527	0.0114719	0.0290111	0.8	-0.107735	0.0163081	3.94E-11	-0.106483	0.269764	0.6930459	0.6224296
ENSG00000132669	RIN2	20	19925133	A	G	0.0536779	-0.0654448	0.0335018	0.06900014	-0.226779	0.0191426	2.24E-32	0.288585	0.149724	0.05392443	0.07452704
ENSG00000132670	PTPRA	20	2932276	A	G	0.476143	-0.000920949	0.014221	0.9299999	0.182945	0.00795386	4.58E-117	-0.00503402	0.077734	0.9483655	0.5010985
ENSG00000132676	DAP3	1	155683277	T	C	0.297217	0.00760193	0.015526	0.5400003	-0.20289	0.00870452	3.63E-120	-0.0374682	0.076541	0.6244752	0.4360967
ENSG00000132680	KHDC4	1	155893512	A	G	0.101392	-0.0413795	0.0283686	0.09699961	-0.0973072	0.0154751	3.22E-10	0.425246	0.299277	0.1553435	0.6129965
ENSG00000132681	ATP1A4	1	160139063	G	A	0.286282	0.00735092	0.0158172	0.64	0.44741	0.00836729	0	0.0164299	0.0353542	0.6421287	0.9838329
ENSG00000132694	ARHGEF11	1	156959897	A	G	0.17495	0.00320746	0.0196903	0.8200001	0.183831	0.0107506	1.49E-65	0.0174479	0.107116	0.8706066	0.2248726
ENSG00000132698	RAB25	1	156035623	C	A	0.206759	-0.00214458	0.0171221	0.99	-0.0957695	0.00974852	8.87E-23	0.0223931	0.178799	0.900332	0.7178343
ENSG00000132704	FCRL2	1	157731222	C	G	0.240557	-0.0205334	0.0164877	0.1499999	-0.125897	0.00930364	1.01E-41	0.163097	0.131515	0.2149251	0.5596105
ENSG00000132716	DCAF8	1	160220212	T	A	0.427435	-0.00918935	0.0143095	0.4799997	0.109926	0.0087751	5.31E-36	-0.083596	0.130345	0.5212998	0.8951879
ENSG00000132718	SYT11	1	155842145	A	T	0.269384	0.0073044	0.0156462	0.5400003	-0.182504	0.00883729	9.44E-95	-0.0400232	0.0857525	0.6406938	0.4587922
ENSG00000132740	IGHMBP2	11	68689690	C	G	0.299205	0.00926179	0.0156687	0.6100002	0.655335	0.00761802	0	0.0141329	0.02391	0.5544619	0.03564702
ENSG00000132744	ACY3	11	67414078	A	G	0.11332	-0.024162	0.0231426	0.3100002	0.351561	0.0154978	6.36E-114	-0.0687277	0.0658977	0.2969739	0.2463652
ENSG00000132749	TESMIN	11	68496970	T	C	0.266402	0.0253647	0.0154684	0.08999948	-0.355613	0.00884555	0	-0.0713266	0.043534	0.1013356	0.6378277
ENSG00000132763	MMACHC	1	45971232	A	G	0.217694	0.0014957	0.0168703	0.95	0.194184	0.00976544	5.51E-88	0.00770249	0.0868788	0.9293538	0.3456601
ENSG00000132768	DPH2	1	44437356	G	A	0.150099	0.0145534	0.020856	0.6100002	0.121194	0.0115676	1.10E-25	0.120084	0.172469	0.4862652	0.287548
ENSG00000132780	NASP	1	46067042	A	G	0.476143	-0.00539079	0.0142394	0.8499999	0.0900619	0.0120208	6.77E-14	-0.0598565	0.158309	0.7053561	0.5871422
ENSG00000132781	MUTYH	1	45800488	T	C	0.470179	0.00238374	0.0142684	0.95	-0.25139	0.00790331	4.99E-222	-0.00948223	0.0567588	0.8673213	0.8274169
ENSG00000132792	CTNNBL1	20	36411469	T	C	0.0606362	-0.0173622	0.0337002	0.5199996	-0.255836	0.0170244	4.84E-51	0.0678647	0.131803	0.6066276	0.1880596
ENSG00000132793	LPIN3	20	39979391	C	A	0.298211	0.00366457	0.0152379	0.91	0.0510504	0.00867762	4.03E-09	0.0717834	0.298737	0.8101056	0.1139714
ENSG00000132801	ZSWIM3	20	44497008	A	G	0.294235	0.0135422	0.0159072	0.3800004	0.0518315	0.00881818	4.16E-09	0.261273	0.310104	0.3994892	0.7807018
ENSG00000132819	RBM38	20	55975426	T	C	0.129225	-0.0168262	0.022748	0.4299995	0.0841345	0.0120254	2.63E-12	-0.199992	0.271883	0.4619871	0.4283191
ENSG00000132823	OSER1	20	42832283	G	A	0.461233	-0.00381774	0.0142243	0.6100002	0.485452	0.00830147	0	-0.00786429	0.0293014	0.788397	0.1075283
ENSG00000132824	SERINC3	20	43137806	A	G	0.055666	0.0512501	0.0314524	0.1100001	-0.67201	0.0214219	5.16E-216	-0.0762639	0.0468666	0.1036828	0.9292868
ENSG00000132825	PPP1R3D	20	58513623	T	C	0.257455	0.0103969	0.0167004	0.5199996	-0.100274	0.00925759	2.44E-27	-0.103684	0.166822	0.5342521	0.8745358
ENSG00000132832	NA	20	43304914	G	C	0.0646123	0.0288747	0.0258382	0.1800002	-1.01054	0.0353208	5.00E-180	-0.0285736	0.0255882	0.2641362	0.8769086
ENSG00000132842	AP3B1	5	77443464	A	G	0.286282	-0.0097115	0.015449	0.5400003	-0.562535	0.00828337	0	0.0172638	0.0274643	0.5296176	0.5625023

ENSG00000132846	ZBED3	5	76375522	C	T	0.408549	-0.00384158	0.0144984	0.7600007	-0.189259	0.00831154	8.97E-115	0.020298	0.0766114	0.7910496	0.6057818
ENSG00000132849	PATJ	1	62418870	C	G	0.0497018	0.00489791	0.0277041	0.9299999	0.42925	0.0152074	2.77E-175	0.0114104	0.0645419	0.8596732	0.2909009
ENSG00000132879	FBXO44	1	11718908	T	G	0.202783	0.00705354	0.0176447	0.6899999	0.19307	0.01311	4.32E-49	0.0365335	0.0914237	0.6894461	0.9481855
ENSG00000132881	CPLANE2	1	16560820	C	A	0.298211	0.0166606	0.0156158	0.3400001	0.267769	0.00975343	6.26E-166	0.0622201	0.0583623	0.2863776	0.8566093
ENSG00000132906	CASP9	1	15835178	C	T	0.297217	0.00726026	0.0152811	0.6300007	0.334371	0.00838694	0	0.0217132	0.0457042	0.63473	0.02361865
ENSG00000132912	DCTN4	5	150113336	G	T	0.0685885	0.0201649	0.0273376	0.4899999	-0.137625	0.0159465	6.11E-18	-0.146521	0.199363	0.4623726	0.9460806
ENSG00000132932	ATP8A2	13	26273099	A	G	0.459245	-0.00546145	0.0143549	0.5500004	0.13504	0.00803463	2.16E-63	-0.0404433	0.106329	0.7036771	0.1997067
ENSG00000132950	ZMYM5	13	20417699	A	G	0.0377734	-0.007411	0.0358055	0.8499999	0.270324	0.0210078	6.83E-38	-0.0274153	0.132471	0.8360469	0.4909525
ENSG00000132952	USPL1	13	31212758	T	C	0.40159	0.0147937	0.0144713	0.3100002	0.263349	0.00796657	1.24E-239	0.0561752	0.0549773	0.3068803	0.2030799
ENSG00000132953	XPO4	13	21414328	C	T	0.516899	0.00529682	0.0141837	0.5999997	-0.138615	0.00833451	4.13E-62	-0.0382126	0.10235	0.7088879	0.5851852
ENSG00000132964	CDK8	13	26903825	G	A	0.0616302	0.0240421	0.0303384	0.4	0.281689	0.0217276	1.94E-38	0.0853498	0.107903	0.428951	0.2042358
ENSG00000132965	ALOX5AP	13	31324100	T	A	0.235586	0.00716191	0.0162865	0.64	0.593084	0.00859154	0	0.0120757	0.0274613	0.6601281	0.1419869
ENSG00000132970	WASF3	13	27197462	T	C	0.439364	0.0286151	0.0143268	0.04600023	-0.177199	0.00795556	6.66E-110	-0.161486	0.0811759	0.04666472	0.1653947
ENSG00000132972	RNF17	13	25396174	C	T	0.408549	-0.0141679	0.0143948	0.2700001	-0.0727372	0.00820221	7.45E-19	0.194782	0.199117	0.3279602	0.1191611
ENSG00000133026	MYH10	17	8455801	C	T	0.0208748	-0.0474293	0.0498498	0.3599996	0.287416	0.0285549	7.86E-24	-0.165019	0.174214	0.3435258	0.168472
ENSG00000133027	PEMT	17	17451949	T	C	0.384692	-0.0295947	0.0146264	0.04200007	0.183076	0.0083091	1.38E-107	-0.161653	0.0802287	0.04391542	0.2812608
ENSG00000133028	SCO1	17	10592673	C	G	0.471173	-0.00883557	0.0143694	0.5	-0.425438	0.00800367	0	0.0207682	0.0337778	0.5386559	0.562768
ENSG00000133030	MPRIP	17	17033426	T	C	0.167992	-0.011656	0.0178502	0.4199997	0.120649	0.0109886	4.80E-28	-0.0966111	0.148213	0.5145056	0.8007861
ENSG00000133048	CHI3L1	1	203151968	C	T	0.177932	0.00692279	0.017476	0.4100001	-0.792772	0.00961055	0	-0.00873238	0.0220444	0.6920116	0.1942934
ENSG00000133055	MYBPH	1	203140940	T	C	0.210736	0.00550438	0.016508	0.5099998	-0.528513	0.00886737	0	-0.0104148	0.0312353	0.7388083	0.2375762
ENSG00000133056	PIK3C2B	1	204427804	A	G	0.529821	0.0150185	0.0141848	0.2099999	0.128845	0.00795362	5.08E-59	0.116562	0.110326	0.2907307	0.3888256
ENSG00000133059	DSTYK	1	205146179	C	A	0.427435	0.0125149	0.0142635	0.4600002	-0.611572	0.00730264	0	-0.0204635	0.023324	0.3802916	0.04941179
ENSG00000133063	CHIT1	1	203212362	A	G	0.189861	-0.011042	0.0175517	0.3400001	-0.157122	0.0106714	4.55E-49	0.0702767	0.11181	0.5296507	0.1902767
ENSG00000133065	SLC41A1	1	205770548	T	C	0.39662	-0.00467557	0.0144259	0.7899998	0.21453	0.00803926	6.97E-157	-0.0217945	0.0672491	0.7458733	0.2983905
ENSG00000133067	LGR6	1	202225969	A	G	0.321074	0.0136105	0.0152745	0.35	0.282656	0.00847167	4.44E-244	0.0481522	0.0540585	0.3730672	0.3838245
ENSG00000133069	TMCC2	1	205219887	C	T	0.370775	0.00278194	0.0145677	0.98	-0.127547	0.00814973	3.30E-55	-0.0218112	0.114223	0.8485631	0.2473777
ENSG00000133101	CCNA1	13	37011493	G	A	0.287276	-0.00357899	0.0158016	0.6300007	0.410186	0.0130064	2.69E-218	-0.00872529	0.038524	0.8208207	0.5689358
ENSG00000133103	COG6	13	40297783	C	T	0.367793	-0.000416738	0.0146773	0.9	0.468991	0.0077968	0	-0.000888585	0.0312955	0.9773484	0.1956598
ENSG00000133104	SPART	13	36910046	T	C	0.245527	0.00449232	0.015899	0.81	0.317539	0.00876899	4.00E-287	0.0141473	0.0500709	0.7775256	0.5260179
ENSG00000133105	RXFP2	13	32345341	A	C	0.50994	0.0126193	0.014207	0.1900002	-0.206541	0.00797189	5.33E-148	-0.0610984	0.068826	0.3746894	0.9805654
ENSG00000133106	EPSTI1	13	43513465	A	C	0.269384	0.0016397	0.0164757	0.7800007	0.67651	0.0083061	0	0.00242376	0.024354	0.9207236	0.9919509
ENSG00000133111	RFXAP	13	37398301	T	C	0.158052	-0.0221228	0.0192539	0.17	-0.0714303	0.0108097	3.90E-11	0.309712	0.273593	0.2576269	0.5605499
ENSG00000133116	KL	13	33615244	C	T	0.400596	-0.00269391	0.0144618	0.9	0.193588	0.00810006	3.09E-126	-0.0139157	0.0747062	0.8522315	0.3349301
ENSG00000133119	RFC3	13	34466440	T	C	0.357853	0.00578182	0.0150549	0.89	-0.0822368	0.00821628	1.39E-23	-0.070307	0.183202	0.7011514	0.6566455
ENSG00000133193	VCF1	17	71218192	G	A	0.37674	0.00504819	0.0144774	0.6100002	-0.299037	0.00787564	0	-0.0168815	0.0484155	0.7273297	0.3699913
ENSG00000133195	SLC39A11	17	70865469	C	T	0.478131	0.0113379	0.0142216	0.4199997	0.290755	0.00780741	1.49E-303	0.0389947	0.0489239	0.4254229	0.4099883
ENSG00000133216	EPHB2	1	23139575	C	A	0.0944334	0.0100542	0.0267153	0.8499999	-0.424626	0.0138737	1.00E-205	-0.0236778	0.0629196	0.7066807	0.09790449
ENSG00000133226	SRRM1	1	24978982	A	G	0.428429	-0.0152089	0.0143736	0.28	0.082487	0.0119536	5.18E-12	-0.184379	0.176289	0.2956121	0.009750931
ENSG00000133243	BTBD2	19	2010163	A	G	0.148111	0.0135654	0.0210098	0.5700002	0.301534	0.0158673	1.60E-80	0.044988	0.0697167	0.5187342	0.1007073
ENSG00000133246	PRAM1	19	8561468	T	G	0.260437	-0.00230127	0.0156612	0.8	-0.282496	0.0132216	2.76E-101	0.0081462	0.0554399	0.8831815	0.5846264
ENSG00000133247	KMT5C	19	55855354	A	G	0.302187	0.0267843	0.015668	0.08100093	-0.116176	0.00940543	4.75E-35	-0.230549	0.13615	0.09038879	0.1799192
ENSG00000133256	PDE6B	4	641972	G	T	0.133201	0.0299415	0.0214909	0.1800002	0.289611	0.0145985	1.39E-87	0.103385	0.0743889	0.1645913	0.4849097
ENSG00000133265	HSPBP1	19	55782674	A	G	0.0417495	-0.00675526	0.0292241	0.9400001	-0.229261	0.0334638	7.33E-12	0.0294654	0.127544	0.8172975	0.7260983
ENSG00000133275	CSNK1G2	19	1961262	A	C	0.185885	0.00575343	0.018862	0.81	-0.209057	0.0129391	1.01E-58	-0.0275209	0.0902404	0.7603865	0.4238728
ENSG00000133302	SLF1	5	94014596	A	G	0.293241	0.00606298	0.015599	0.5099998	-0.10866	0.00887791	1.91E-34	-0.0557976	0.14363	0.6976596	0.9382987
ENSG00000133313	CNDP2	18	72175708	A	G	0.164016	-0.00758711	0.0178788	0.5700002	-0.669293	0.00922531	0	0.011336	0.0267134	0.671306	0.3469098
ENSG00000133317	LGALS12	11	63278901	T	G	0.356859	0.0159542	0.0144982	0.2399999	-0.298904	0.00809638	2.36E-298	-0.0533757	0.0485261	0.2713592	0.5672286
ENSG00000133318	RTN3	11	63488140	A	G	0.191849	-0.0136022	0.01788	0.3700002	0.220119	0.0098475	1.13E-110	-0.0617947	0.0812757	0.4470698	0.7613316
ENSG00000133321	PLAAT4	11	63309107	C	T	0.37674	0.0111584	0.014509	0.4299995	-0.0930963	0.00891556	1.59E-25	-0.119859	0.156271	0.4430875	0.8304334
ENSG00000133328	PLAAT2	11	63325548	G	A	0.0666004	-0.0332415	0.0327195	0.2	0.286361	0.0168746	1.37E-64	-0.116082	0.114464	0.3105169	0.2570348

ENSG00000133392	MYH11	16	15873941	T	C	0.28827	-0.00132108	0.0156721	0.8600001	0.565392	0.00850613	0	-0.00233657	0.027719	0.9328219	0.1515697
ENSG00000133393	CEP20	16	15971029	A	G	0.154076	-0.00838002	0.0196049	0.8700001	0.380297	0.012188	9.82E-214	-0.0220355	0.0515564	0.6690843	0.8922418
ENSG00000133398	MED10	5	6375350	G	A	0.496024	0.00294518	0.0141775	0.8	-0.298126	0.00776679	0	-0.00987898	0.0475561	0.8354373	0.6040792
ENSG00000133401	PDZD2	5	31875277	T	C	0.411531	-0.00666838	0.0144754	0.6899999	0.0527779	0.00886338	2.61E-09	-0.126348	0.27509	0.6460206	0.9431567
ENSG00000133422	MORC2	22	31343440	T	C	0.27833	-0.0037542	0.0158636	0.9299999	0.223027	0.00926209	4.09E-128	-0.0168329	0.0711319	0.8129333	0.02639444
ENSG00000133424	LARGE1	22	33993838	T	A	0.384692	0.00553048	0.0146973	0.99	-0.177693	0.00824462	5.01E-103	-0.0311238	0.0827244	0.7067423	0.8306748
ENSG00000133454	MYO18B	22	26282559	C	A	0.137177	0.0151652	0.0208319	0.4199997	0.206752	0.0112077	5.47E-76	0.0733496	0.100836	0.4669727	0.9858823
ENSG00000133460	SLC2A11	22	24213315	G	C	0.083499	-0.00945816	0.0255976	0.64	-0.442161	0.0178407	1.34E-135	0.0213908	0.0578984	0.7117901	0.9617194
ENSG00000133466	C1QTNF6	22	37585816	A	C	0.471173	0.0100959	0.0141894	0.6999999	0.412995	0.00761382	0	0.0244456	0.0343603	0.4768062	0.7628727
ENSG00000133488	SEC14L4	22	30893299	A	C	0.0178926	-0.00783699	0.0513959	0.7800007	-0.321281	0.0281534	3.65E-30	0.024393	0.159986	0.8788169	0.9163679
ENSG00000133561	GIMAP6	7	150325968	T	C	0.104374	0.00358872	0.0225827	0.6100002	-0.419242	0.0133063	6.97E-218	-0.00856002	0.0538663	0.8737378	0.677588
ENSG00000133574	GIMAP4	7	150267703	A	C	0.0497018	0.019494	0.0334869	0.7600007	-0.933993	0.0184141	0	-0.0208717	0.0358558	0.5604995	0.605584
ENSG00000133597	ADCK2	7	140384507	T	C	0.0715706	-0.0424455	0.0281229	0.05999983	-0.98612	0.0143572	0	0.0430429	0.0285256	0.1313188	0.2575774
ENSG00000133606	MKRN1	7	140166104	C	A	0.112326	0.0365037	0.021809	0.07199959	-0.199382	0.0225878	1.08E-18	-0.183084	0.111332	0.1000757	0.01696601
ENSG00000133612	AGAP3	7	150812220	A	G	0.284294	-0.00915285	0.0156642	0.3700002	-0.390392	0.00950329	0	0.0234453	0.0401283	0.5590472	0.3257518
ENSG00000133619	KRBA1	7	149421768	A	G	0.146123	0.00466886	0.0223715	0.84	0.228906	0.0141855	1.41E-58	0.0203964	0.0977406	0.8346986	0.522252
ENSG00000133624	NA	7	149283044	A	G	0.260437	0.0288465	0.01624	0.064	-0.287144	0.010036	4.83E-180	-0.10046	0.0566659	0.07625371	0.8046789
ENSG00000133627	ACTR3B	7	152504648	A	G	0.054672	-0.0137462	0.0371855	0.7600007	0.197139	0.0236975	8.87E-17	-0.0697284	0.188812	0.7119033	0.5996739
ENSG00000133639	BTG1	12	92537979	C	T	0.142147	0.0150177	0.018527	0.2599998	0.138095	0.0115044	3.40E-33	0.108749	0.134467	0.4186632	0.7154956
ENSG00000133641	RLIG1	12	88435780	C	T	0.294235	-0.00411121	0.0155407	0.8700001	-0.0485174	0.00874849	2.93E-08	0.0847369	0.320676	0.7915917	0.6765013
ENSG00000133657	ATP13A3	3	194171247	A	G	0.115308	0.025609	0.0250886	0.2399999	-0.19681	0.0152884	6.37E-38	-0.13012	0.127876	0.3088925	0.724077
ENSG00000133661	SFTPD	10	81719933	A	G	0.422465	0.00762006	0.0145556	0.6200004	0.318843	0.00855455	4.72E-304	0.0238991	0.0456559	0.6006526	0.2333086
ENSG00000133678	TMEM254	10	81845357	C	A	0.11829	0.00502036	0.0243135	0.9299999	0.59681	0.0146606	0	0.00841199	0.0407396	0.8364146	0.5736498
ENSG00000133687	TMTC1	12	29795732	G	A	0.380716	0.00433439	0.0145102	0.6499995	0.877267	0.00650296	0	0.00494079	0.0165403	0.7651594	0.3299234
ENSG00000133703	KRAS	12	25380796	A	G	0.412525	-0.0109589	0.0144582	0.4700002	-0.0689514	0.00797531	5.35E-18	0.158937	0.210491	0.450204	0.3887957
ENSG00000133704	IPO8	12	30815421	G	A	0.49006	-5.84E-05	0.0142016	0.9299999	0.769485	0.00667789	0	-7.59E-05	0.018456	0.996717	0.5699771
ENSG00000133706	LARS1	5	145527412	A	T	0.252485	0.0062031	0.0155824	0.5400003	-0.213372	0.0108885	1.67E-85	-0.0290718	0.0730444	0.690629	0.2967352
ENSG00000133731	IMPA1	8	82584562	C	G	0.314115	-0.019275	0.0157114	0.1900002	-0.509231	0.00821969	0	0.0378512	0.0308593	0.2199821	0.2312244
ENSG00000133739	LRRCC1	8	86038846	A	T	0.310139	-0.00471631	0.0151161	0.7700005	-0.176374	0.00852278	3.89E-95	0.0267404	0.0857146	0.7550631	0.05687014
ENSG00000133740	E2F5	8	86109423	A	C	0.0347913	0.0468848	0.0295215	0.05800027	0.195576	0.025754	3.10E-14	0.239727	0.154212	0.1200588	0.7118567
ENSG00000133773	CCDC59	12	82685022	A	G	0.0854871	-0.0269197	0.0239708	0.32	0.108878	0.0152146	8.29E-13	-0.247245	0.222856	0.2672393	0.5133171
ENSG00000133789	SWAP70	11	9730081	G	A	0.405567	-0.0332835	0.0143415	0.02699977	0.27406	0.00791284	7.57E-263	-0.121446	0.0524472	0.02058068	0.4888344
ENSG00000133794	BMAL1	11	13353506	G	A	0.426441	0.0217219	0.0143028	0.07299952	0.377583	0.0077744	0	0.0575288	0.0378984	0.1290208	0.8423684
ENSG00000133800	LYVE1	11	10605874	T	G	0.116302	-0.0156193	0.022542	0.4600002	-0.229977	0.0116371	6.27E-87	0.0679169	0.0980789	0.4886408	0.7565146
ENSG00000133805	AMPD3	11	10429493	C	T	0.110338	0.052358	0.0213721	0.007399971	0.249014	0.0119326	1.04E-96	0.210261	0.0864163	0.0149693	0.08592063
ENSG00000133808	NA	11	12339159	G	A	0.122266	0.0254329	0.0215082	0.2599998	0.565617	0.0115691	0	0.0449649	0.0380372	0.2371546	0.112069
ENSG00000133812	SBF2	11	10057984	C	G	0.427435	-0.0348777	0.0143252	0.02	0.243523	0.00799381	7.84E-204	-0.143222	0.0590125	0.01522548	0.7787436
ENSG00000133816	MICAL2	11	12200438	G	A	0.249503	-0.00313181	0.0165227	0.8499999	0.182389	0.0141483	5.05E-38	-0.0171711	0.0906003	0.849681	0.9535468
ENSG00000133818	RRAS2	11	14342762	C	T	0.244533	-0.0136027	0.0168738	0.3800004	-0.355561	0.0151725	1.90E-121	0.038257	0.0474848	0.4204351	0.57895
ENSG00000133835	HSD17B4	5	118880516	G	A	0.0497018	-0.0244884	0.0318818	0.64	0.549886	0.0217737	1.01E-140	-0.0445336	0.0580057	0.4426389	0.9940221
ENSG00000133872	SARAF	8	29930625	G	A	0.17495	-0.0192731	0.019086	0.35	0.211588	0.0114554	3.56E-76	-0.0910879	0.0903383	0.3133118	0.6147482
ENSG00000133874	RNF122	8	33414958	G	A	0.0188867	-0.027818	0.0446785	0.56	-0.446876	0.0228907	7.11E-85	0.0622499	0.10003	0.5337383	0.5128697
ENSG00000133884	DPF2	11	65110972	A	C	0.2833	0.00689925	0.0157737	0.5099998	-0.281893	0.00910337	1.56E-210	-0.0244747	0.0559619	0.6618606	0.1149659
ENSG00000133895	MEN1	11	64574874	T	C	0.0854871	-0.0290065	0.024424	0.17	0.212002	0.0125535	5.53E-64	-0.136822	0.115491	0.2361373	0.5663046
ENSG00000133937	GSC	14	95235557	G	A	0.257455	-0.0360923	0.0165876	0.01899984	-0.108465	0.00960653	1.46E-29	0.332754	0.155744	0.0326347	0.5562613
ENSG00000133943	DGLUCY	14	91609326	C	T	0.0477137	0.0527342	0.0342435	0.2099999	0.993084	0.0199448	0	0.0531015	0.0344985	0.1237455	0.3176419
ENSG00000133961	NUMB	14	73836081	C	T	0.219682	-0.00680724	0.0166383	0.8200001	-0.170886	0.00950876	3.26E-72	0.039835	0.0973903	0.6825215	0.5306705
ENSG00000133962	CATSPERB	14	92147045	A	G	0.370775	-0.0165554	0.0150118	0.2	0.239521	0.00898769	1.79E-156	-0.0691187	0.0627279	0.2705127	0.4231959
ENSG00000133983	COX16	14	70809123	C	T	0.306163	-0.0152476	0.0152279	0.28	-0.141715	0.00927346	1.01E-52	0.107593	0.107685	0.3177214	0.1619302
ENSG00000133985	TTC9	14	71125290	A	C	0.33002	0.00996521	0.0151838	0.35	0.305112	0.010535	1.99E-184	0.0326608	0.0497775	0.511736	0.8919902

ENSG00000133997	MED6	14	71057679	T	C	0.329026	0.00931511	0.0151709	0.3700002	0.183734	0.00845656	1.15E-104	0.0506989	0.0826029	0.5393695	0.890699
ENSG00000134001	EIF2S1	14	67839973	G	A	0.0168986	0.110353	0.0532539	0.0259998	-0.489432	0.0345835	1.81E-45	-0.225472	0.109968	0.04033104	NA
ENSG00000134013	LOXL2	8	23218771	A	G	0.239563	0.0170313	0.0165365	0.2	-0.405625	0.0138859	1.40E-187	-0.0419878	0.0407933	0.3033471	0.9261639
ENSG00000134014	ELP3	8	27997931	A	G	0.337972	-0.000390142	0.0152429	0.9599999	0.539155	0.00793547	0	-0.000723618	0.0282718	0.9795804	0.1282723
ENSG00000134028	ADAMDEC1	8	24252662	A	G	0.472167	-0.0163765	0.0142411	0.29	-0.210969	0.00860922	1.31E-132	0.0776252	0.0675776	0.250687	0.3672112
ENSG00000134030	CTIF	18	46227502	A	C	0.0805169	-0.00795101	0.0278852	0.81	-0.227938	0.0182742	1.05E-35	0.0348823	0.122369	0.7755988	0.5253154
ENSG00000134046	MBD2	18	51715118	G	C	0.122266	0.0251415	0.0206865	0.2	-0.227299	0.0118851	1.58E-81	-0.11061	0.0911938	0.2251638	0.3659896
ENSG00000134049	IER3IP1	18	44692079	G	A	0.394632	0.0187721	0.0144617	0.2200002	-0.0548918	0.0080866	1.14E-11	-0.341983	0.268232	0.2023255	0.189855
ENSG00000134056	MRPS36	5	68519771	C	T	0.210736	0.0231644	0.0176569	0.1800002	0.0613274	0.0097197	2.80E-10	0.377717	0.29407	0.1989857	0.5312888
ENSG00000134057	CCNB1	5	68468454	T	C	0.413519	0.00358736	0.014487	0.8	-0.128435	0.00801618	8.97E-58	-0.0279313	0.11281	0.8044466	0.8449096
ENSG00000134058	CDK7	5	68551959	A	G	0.338966	-0.00691696	0.0152578	0.7199992	0.164637	0.0154716	1.92E-26	-0.0420135	0.0927597	0.6506003	0.3125515
ENSG00000134061	CD180	5	66485365	C	G	0.202783	0.0258918	0.0172758	0.04300015	-0.197633	0.00988105	5.38E-89	-0.13101	0.0876587	0.1350339	0.07813693
ENSG00000134070	IRAK2	3	10245988	T	C	0.161034	0.0363879	0.0203667	0.05600025	-0.193506	0.011875	1.07E-59	-0.188046	0.105882	0.07573391	0.4741193
ENSG00000134072	CAMK1	3	9805351	C	G	0.104374	-0.000259498	0.0223172	0.8700001	0.161207	0.0125436	8.40E-38	-0.00160972	0.138438	0.9907226	0.8584702
ENSG00000134077	THUMPD3	3	9416500	A	G	0.149105	0.0299553	0.0180208	0.064	-0.455684	0.0102564	0	-0.065737	0.0395744	0.0966933	0.5225261
ENSG00000134086	VHL	3	10188298	A	G	0.124254	0.00620645	0.0213512	0.5999997	0.185831	0.0120905	2.60E-53	0.0333984	0.114917	0.7713328	0.6583888
ENSG00000134107	BHLHE40	3	5023904	C	T	0.341948	0.00909044	0.015078	0.3900004	0.0835158	0.00872264	1.02E-21	0.108847	0.180898	0.5473721	0.5198418
ENSG00000134108	ARL8B	3	5193250	G	A	0.293241	-0.00412938	0.0157733	0.7700005	-0.226886	0.00863226	2.96E-152	0.0182002	0.0695242	0.7934891	0.6603174
ENSG00000134109	EDEM1	3	5245486	T	C	0.0258449	0.0230212	0.0472764	0.58	-0.341779	0.0316544	3.55E-27	-0.0673569	0.138465	0.6266449	0.9340958
ENSG00000134121	CHL1	3	344684	G	A	0.413519	-0.0154354	0.0145101	0.2700001	-0.102294	0.00811446	1.95E-36	0.150892	0.142351	0.2891436	0.1871493
ENSG00000134146	DPH6	15	35673970	C	G	0.206759	0.00487768	0.0163584	0.5500004	0.161619	0.0112984	2.05E-46	0.0301801	0.101238	0.7656184	0.6715452
ENSG00000134152	KATNBL1	15	34467586	A	G	0.260437	-0.0214695	0.0166122	0.1	0.21208	0.0102972	2.98E-94	-0.101233	0.0784838	0.1971004	0.2542107
ENSG00000134153	EMC7	15	34385183	C	T	0.110338	0.0294946	0.0237562	0.14	0.133473	0.0142089	5.80E-21	0.220978	0.179533	0.2183792	0.7231906
ENSG00000134183	GNAT2	1	110150784	T	C	0.414513	0.00986695	0.0144786	0.4799997	0.0775804	0.00810679	1.07E-21	0.127184	0.1871	0.4966541	0.6151679
ENSG00000134184	GSTM1	1	110240871	T	C	0.230616	-0.000625167	0.0166374	0.9400001	0.58619	0.010018	0	-0.00106649	0.0283823	0.9700257	0.9110912
ENSG00000134186	PRPF38B	1	109239685	T	C	0.0447316	0.00996768	0.0344341	0.4799997	-0.247928	0.0231561	9.46E-27	-0.0402039	0.138938	0.7723017	0.5266502
ENSG00000134193	REG4	1	120345462	G	C	0.467197	0.0117923	0.0143728	0.3599996	0.116843	0.00798415	1.69E-48	0.100924	0.123202	0.4126877	0.1030092
ENSG00000134198	TSPAN2	1	115611376	C	G	0.100398	-0.00168485	0.0246768	0.99	-0.250374	0.014364	4.83E-68	0.00672934	0.0985606	0.9455658	0.9794735
ENSG00000134201	GSTM5	1	110257882	C	A	0.336978	0.00780679	0.0150278	0.5999997	-0.252425	0.00979774	2.27E-146	-0.0309272	0.0595458	0.6034928	0.3849215
ENSG00000134202	GSTM3	1	110280469	C	T	0.44831	-6.06E-05	0.0143718	0.9400001	-0.899464	0.00634462	0	6.74E-05	0.0159782	0.9966334	0.9861936
ENSG00000134215	VAV3	1	108310774	G	A	0.361829	-0.0236405	0.0146504	0.05899973	0.699081	0.00712042	0	-0.0338165	0.0209595	0.1066524	0.2297071
ENSG00000134222	PSRC1	1	109823993	G	A	0.212724	-0.0359439	0.0170673	0.06800017	0.489799	0.00934249	0	-0.0733851	0.0348737	0.03535119	0.4935023
ENSG00000134242	PTPN22	1	114385407	G	T	0.374751	-0.0291143	0.0148345	0.04700023	0.199167	0.00821485	7.50E-130	-0.14618	0.0747262	0.05044059	0.4979513
ENSG00000134243	SORT1	1	109896382	C	T	0.466203	-0.0209409	0.0143972	0.1499999	0.0828186	0.00809075	1.36E-24	-0.252853	0.175586	0.1498542	0.9476123
ENSG00000134247	PTGFRN	1	117492829	C	A	0.0775348	-0.0359188	0.026812	0.2399999	-0.316161	0.0156641	1.36E-90	0.113609	0.0849914	0.1813164	0.1286237
ENSG00000134248	LAMTOR5	1	110947217	G	T	0.510934	-0.00160572	0.0142355	0.7400005	0.240613	0.00875254	2.27E-166	-0.00667346	0.059164	0.9101923	0.5434359
ENSG00000134250	NOTCH2	1	120533208	C	T	0.0198807	0.046588	0.0561182	0.58	-0.462718	0.0262764	2.08E-69	-0.100683	0.121414	0.40696	0.9710262
ENSG00000134255	CEPT1	1	111704986	T	C	0.128231	-0.0216919	0.0201979	0.28	0.232235	0.0109094	1.48E-100	-0.0934048	0.0870823	0.2834489	0.5476232
ENSG00000134256	CD101	1	117561774	C	A	0.0775348	-0.0359188	0.026812	0.2399999	-0.862666	0.0147548	0	0.041637	0.0310885	0.1804723	0.3960479
ENSG00000134262	AP4B1	1	114442596	A	G	0.249503	0.00579328	0.0156971	0.7199992	-0.228533	0.00901424	8.46E-142	-0.0253499	0.0686937	0.712107	0.1658576
ENSG00000134265	NAPG	18	10539330	A	T	0.514911	-0.004504	0.0142095	0.6300007	-0.489184	0.0111328	0	0.00920716	0.0290481	0.7512718	0.4624909
ENSG00000134278	SPIRE1	18	12552322	A	G	0.146123	0.0125088	0.0189449	0.4700002	-0.413954	0.0102737	0	-0.0302178	0.0457718	0.5091357	0.7366328
ENSG00000134283	PPHLN1	12	42742883	G	A	0.428429	0.0279938	0.0142836	0.03799969	0.310515	0.00779459	0	0.0901527	0.0460553	0.05029005	0.111019
ENSG00000134285	FKBP11	12	49317779	A	G	0.49006	-0.00395996	0.014245	0.6899999	-0.294037	0.0078296	1.18958e-308	0.0134676	0.0484476	0.7810263	0.5425001
ENSG00000134287	ARF3	12	49324310	A	G	0.0795229	0.0239854	0.0254585	0.3900004	-0.590577	0.01305	0	-0.0406135	0.0431172	0.3462269	0.6977597
ENSG00000134291	TMEM106C	12	48360006	A	C	0.406561	-0.0350363	0.014577	0.008799946	0.31944	0.00792388	0	-0.10968	0.045714	0.01642769	0.006392181
ENSG00000134297	PLEKHA8P1	12	45588335	G	A	0.0526839	-0.0444342	0.0314091	0.09800089	0.23741	0.0325871	3.21E-13	-0.187162	0.13477	0.1649083	0.4253684
ENSG00000134308	YWHAQ	2	9747622	C	A	0.298211	0.00154395	0.0156231	0.9199999	0.276834	0.0140633	2.91E-86	0.00557717	0.0564356	0.9212784	0.362883
ENSG00000134313	KIDINS220	2	8921584	T	C	0.304175	-0.0133557	0.015649	0.32	-0.169128	0.017054	3.50E-23	0.0789679	0.0928695	0.3951522	0.7604243
ENSG00000134317	GRHL1	2	10113876	A	G	0.17992	0.0168045	0.0178531	0.33	-0.304002	0.0111515	1.22E-163	-0.0552776	0.0587619	0.3468563	0.7154219

ENSG00000134318	ROCK2	2	11404171	A	G	0.468191	-0.0307918	0.0141955	0.05600025	0.160205	0.00792122	5.92E-91	-0.192202	0.0891165	0.03102474	0.6028581
ENSG00000134321	RSAD2	2	7022153	G	A	0.0228628	-0.0339924	0.0344208	0.29	0.221393	0.0196128	1.50E-29	-0.153539	0.156068	0.3252156	0.9369016
ENSG00000134324	LPIN1	2	11892628	T	C	0.403579	0.00474526	0.0146332	0.5999997	-0.580197	0.00748682	0	-0.00817871	0.0252213	0.7457279	0.6765785
ENSG00000134326	CMPK2	2	6993733	T	C	0.0298211	-0.0390875	0.0322409	0.1900002	0.176341	0.0180286	1.36E-22	-0.221658	0.184231	0.228918	0.6974127
ENSG00000134330	IAH1	2	9625229	G	C	0.0347913	0.0825678	0.0408563	0.04799986	-0.578086	0.0527153	5.56E-28	-0.14283	0.0718652	0.04687092	0.03721558
ENSG00000134333	LDHA	11	18422953	G	T	0.418489	0.0147372	0.0142546	0.4100001	0.178248	0.00885406	3.89E-90	0.082678	0.0800759	0.3018402	0.8167084
ENSG00000134352	IL6ST	5	55260872	A	G	0.377734	-0.00218292	0.014644	0.9	-0.110749	0.00878666	2.00E-36	0.0197105	0.132236	0.8815099	0.8829895
ENSG00000134363	FST	5	52779601	G	T	0.0149105	0.0448762	0.065849	0.5199996	1.38663	0.0373525	1.20E-301	0.0323635	0.0474966	0.4956268	0.8130084
ENSG00000134369	NAV1	1	201694256	C	A	0.445328	-0.00758216	0.014377	0.6100002	0.271596	0.00784574	1.40E-262	-0.027917	0.0529414	0.5979714	0.2573947
ENSG00000134375	TIMM17A	1	201932204	T	C	0.467197	-0.0155244	0.014216	0.3400001	0.0692802	0.00797644	3.77E-18	-0.224081	0.206811	0.2785836	0.7167774
ENSG00000134419	RPS15A	16	18797161	A	G	0.0516899	0.00447677	0.0280335	0.95	-0.952683	0.0155848	0	-0.00469912	0.0294259	0.8731229	0.9811528
ENSG00000134440	NARS1	18	55278666	T	C	0.206759	-0.0229329	0.0176445	0.2099999	0.166189	0.00991416	4.57E-63	-0.137993	0.10649	0.195034	0.8036441
ENSG00000134444	RELCH	18	59914423	C	T	0.267396	0.0200334	0.0166617	0.1100001	-0.349979	0.00942774	1.23E-301	-0.0572418	0.0476327	0.2294673	0.3172065
ENSG00000134452	FBH1	10	5955545	C	G	0.147117	0.0203323	0.020241	0.3700002	0.432919	0.0110435	0	0.0469656	0.0467701	0.3152914	0.4895808
ENSG00000134453	RBM17	10	6145185	G	A	0.0168986	-0.0567209	0.0502661	0.2599998	-0.503436	0.0270857	4.11E-77	0.112668	0.10003	0.2600221	0.7849966
ENSG00000134460	IL2RA	10	6078470	T	C	0.0695825	0.0426603	0.0238444	0.03799969	0.233262	0.0148251	8.82E-56	0.182886	0.10288	0.07546027	0.8137158
ENSG00000134461	ANKRD16	10	5917779	A	G	0.00695825	0.0534049	0.0617793	0.32	-0.643341	0.0722401	5.31E-19	-0.0830118	0.0964801	0.3895672	0.1124983
ENSG00000134463	ECHDC3	10	11795217	A	G	0.286282	-0.0072783	0.016096	0.4700002	-0.511245	0.00838697	0	0.0142364	0.0314848	0.6511478	0.05565442
ENSG00000134470	IL15RA	10	6005594	A	G	0.441352	0.0126613	0.0142087	0.3800004	0.312007	0.00781069	0	0.0405801	0.045551	0.3729972	0.7213051
ENSG00000134480	CCNH	5	86698073	G	A	0.220676	-0.0165081	0.0175518	0.2999998	0.0905826	0.0104582	4.66E-18	-0.182244	0.194905	0.349768	0.5507943
ENSG00000134489	HRH4	18	22050257	T	C	0.0815109	0.0161322	0.0232596	0.5300002	-0.0758804	0.0130473	6.03E-09	-0.2126	0.308702	0.4910173	0.8964015
ENSG00000134490	TMEM241	18	20897516	A	G	0.454274	0.00212785	0.0142537	0.9299999	0.356982	0.00868999	0	0.00596066	0.0399286	0.8813303	0.6173277
ENSG00000134508	CABLES1	18	20777479	C	T	0.276342	0.00779835	0.0163035	0.6200004	-0.0561283	0.00916201	9.00E-10	-0.138938	0.291353	0.633453	0.877867
ENSG00000134516	DOCK2	5	169287318	G	T	0.133201	0.0253211	0.0217001	0.3599996	-0.221682	0.0108978	5.47E-92	-0.114223	0.0980493	0.244039	0.7314173
ENSG00000134531	EMP1	12	13359679	T	C	0.111332	0.0225796	0.0232511	0.2999998	-0.205046	0.0125698	8.03E-60	-0.110119	0.113595	0.332344	0.9365395
ENSG00000134539	KLRD1	12	10424253	T	C	0.241551	0.0176422	0.0166263	0.3100002	0.339568	0.00914602	1.02E-301	0.0519549	0.0489831	0.2888404	0.7969352
ENSG00000134545	KLRC1	12	10601073	C	T	0.348907	-0.0261918	0.015248	0.08799946	0.465763	0.00795804	0	-0.0562341	0.0327518	0.08598264	0.901348
ENSG00000134548	SPX	12	21684776	C	T	0.400596	0.00846015	0.0147796	0.83	0.380872	0.0154224	1.18E-134	0.0222126	0.038815	0.5671411	0.6119247
ENSG00000134571	MYBPC3	11	47363605	C	A	0.312127	-0.00210517	0.015256	0.9599999	-0.588859	0.00794898	0	0.003575	0.0259078	0.8902488	0.7205941
ENSG00000134574	DDB2	11	47248630	G	T	0.153082	0.00407466	0.0196156	0.8	-0.293081	0.0102167	5.66E-181	-0.0139029	0.0669308	0.8354476	0.2507657
ENSG00000134575	ACP2	11	47265655	C	T	0.190855	-0.00145161	0.0181022	0.9699999	-0.584059	0.00893599	0	0.00248538	0.0309938	0.9360864	0.3492674
ENSG00000134627	PIWIL4	11	94315796	A	G	0.4334	-0.0159421	0.0144302	0.1499999	0.129925	0.00807091	2.63E-58	-0.122702	0.111326	0.2703831	0.8130584
ENSG00000134644	PUM1	1	31471595	C	T	0.100398	0.00856554	0.0218693	0.6700003	0.222769	0.0127027	7.45E-69	0.0384503	0.0981947	0.6953745	0.5529605
ENSG00000134668	SPOCD1	1	32268837	G	T	0.327038	0.00852774	0.0153803	0.5500004	0.218801	0.00844094	3.82E-148	0.0389749	0.0703096	0.5793518	0.3728888
ENSG00000134684	YARS1	1	33262297	C	A	0.319085	0.0212762	0.015548	0.2300001	-0.088844	0.00848525	1.18E-25	-0.239478	0.176492	0.174819	0.03026982
ENSG00000134686	PHC2	1	33842938	T	G	0.194831	-0.00634629	0.017572	0.6999999	0.190315	0.0101205	6.90E-79	-0.0333463	0.0923484	0.7180302	0.7454712
ENSG00000134690	CDCA8	1	38166740	C	A	0.175944	0.00328137	0.0186318	0.89	-0.122561	0.00991694	4.37E-35	-0.0267733	0.152036	0.8602164	0.2037465
ENSG00000134697	GNL2	1	38046976	G	T	0.469185	0.0232815	0.0142546	0.07100027	0.204823	0.0117728	8.54E-68	0.113666	0.0699006	0.1039257	0.06564137
ENSG00000134698	AGO4	1	36298632	C	T	0.0228628	-0.0366812	0.0562501	0.4700002	-0.502756	0.0579278	3.99E-18	0.0729603	0.112199	0.5155139	0.07269858
ENSG00000134709	HOOK1	1	60311254	A	G	0.0487078	0.0429358	0.0361839	0.2700001	-0.592468	0.0211328	6.02E-173	-0.0724694	0.0611279	0.2358047	0.8712199
ENSG00000134716	CYP2J2	1	60375721	C	A	0.0755467	-0.0038923	0.0255311	0.9400001	0.274728	0.0146177	8.42E-79	-0.0141678	0.0929354	0.8788334	0.6250957
ENSG00000134744	TUT4	1	52946556	T	C	0.0497018	-0.0723621	0.0321532	0.02	0.357796	0.0171597	1.49E-96	-0.202244	0.0903865	0.02525072	0.04565834
ENSG00000134748	PRPF38A	1	52877114	T	C	0.0506958	-0.0735218	0.0318132	0.01499996	0.274663	0.0167731	2.88E-60	-0.26768	0.116974	0.02211601	0.03243401
ENSG00000134755	DSC2	18	28664159	G	A	0.387674	0.00357132	0.0143641	0.9	-0.161299	0.00800382	2.54E-90	-0.0221409	0.0890592	0.803663	0.0526253
ENSG00000134758	RNF138	18	29691671	A	G	0.055666	0.0126098	0.0347309	0.7800007	-0.622462	0.0179251	3.23E-264	-0.020258	0.0557991	0.7165663	0.3476931
ENSG00000134759	ELP2	18	33733658	G	A	0.375746	0.0161287	0.0147722	0.28	0.686833	0.0108411	0	0.0234827	0.0215109	0.27498	0.8139739
ENSG00000134765	DSC1	18	28726009	G	A	0.383698	0.00206051	0.0143215	0.9699999	-0.310979	0.00789017	0	-0.00662588	0.0460533	0.8855997	0.08257048
ENSG00000134775	FHOD3	18	34118849	A	G	0.265408	0.00164868	0.0159086	0.7499995	-0.0879227	0.0101382	4.23E-18	-0.0187515	0.180951	0.9174653	0.5852742
ENSG00000134779	TPGS2	18	34384572	C	G	0.277336	-0.00361876	0.0158176	1	-0.544309	0.00924234	0	0.00664836	0.0290602	0.8190408	0.3393772
ENSG00000134780	DAGLA	11	61481189	G	A	0.54672	-0.00144785	0.0142035	0.7600007	-0.120021	0.00797333	3.31E-51	0.0120633	0.118345	0.9188092	0.308676

ENSG00000134802	SLC43A3	11	57184740	C	T	0.0367793	-0.00495515	0.0347756	0.9699999	-0.219019	0.0197041	1.06E-28	0.0226243	0.158792	0.8867028	0.3671744
ENSG00000134809	TIMM10	11	57297106	T	C	0.441352	0.00139249	0.0143575	0.9599999	0.409585	0.00764995	0	0.00339976	0.0350539	0.9227369	0.269326
ENSG00000134812	CBLIF	11	59604857	G	A	0.0457256	0.0113716	0.0345632	0.7499995	0.491355	0.0299464	1.68E-60	0.0231433	0.0703567	0.742199	0.05051454
ENSG00000134815	DHX34	19	47869249	G	T	0.281312	-0.00796839	0.0160061	0.5	0.379533	0.00893556	0	-0.0209953	0.0421761	0.6186247	0.7539828
ENSG00000134824	FADS2	11	61597639	T	C	0.150099	-1.16E-05	0.0181821	0.7099994	0.999342	0.00972902	0	-1.16E-05	0.0181941	0.9994931	0.2295956
ENSG00000134825	TMEM258	11	61558354	A	G	0.313121	0.000736411	0.0152552	0.7800007	0.328118	0.00939484	3.07E-267	0.00224435	0.0464931	0.9614989	0.2305408
ENSG00000134827	TCN1	11	59627160	A	G	0.265408	0.01006	0.0162972	0.5500004	0.216587	0.0131141	2.83E-61	0.0464479	0.0752982	0.5373315	0.04325362
ENSG00000134851	TMEM165	4	56277231	G	A	0.423459	0.0144351	0.0144683	0.25	0.0882736	0.00806829	7.35E-28	0.163527	0.164583	0.3204261	0.1137154
ENSG00000134852	CLOCK	4	56353687	G	A	0.342942	0.0272123	0.0147455	0.03799969	0.223712	0.00833619	1.22E-158	0.12164	0.0660684	0.06560448	0.8517224
ENSG00000134864	GGACT	13	101212791	T	A	0.356859	-0.00774288	0.0150975	0.5400003	0.294985	0.0102069	1.18E-183	-0.0262484	0.0511886	0.6081068	0.7149663
ENSG00000134871	COL4A2	13	111061766	G	A	0.10338	0.017986	0.0249713	0.4899999	-0.0865754	0.0123845	2.74E-12	-0.20775	0.289961	0.4736988	0.0149779
ENSG00000134873	CLDN10	13	96158935	C	T	0.474155	0.0140658	0.014193	0.28	0.110982	0.00802585	1.73E-43	0.12674	0.128214	0.3229067	0.6362117
ENSG00000134882	UBAC2	13	99945858	A	T	0.284294	-0.0173424	0.0154741	0.2200002	0.146002	0.00858258	6.76E-65	-0.118782	0.106216	0.2634334	0.6128598
ENSG00000134884	ARGLU1	13	107207266	A	C	0.319085	-0.00603432	0.0154482	0.59	-0.0933512	0.00855821	1.06E-27	0.064641	0.165591	0.6962658	0.214996
ENSG00000134897	BIVM	13	103472642	A	G	0.17992	0.00113114	0.0188252	0.9199999	0.361012	0.0103745	2.59E-265	0.00313325	0.0521457	0.9520868	0.4548101
ENSG00000134899	ERCC5	13	103512769	T	C	0.415507	-0.0245561	0.014486	0.04300015	-0.210107	0.00797327	4.95E-153	0.116874	0.0690884	0.09071002	0.3969779
ENSG00000134900	TPP2	13	103290437	A	G	0.387674	0.00793068	0.0143934	0.5700002	-0.374969	0.00777623	0	-0.0211502	0.0383881	0.5816623	0.7080882
ENSG00000134905	CARS2	13	111329854	G	A	0.204771	-0.0135666	0.0188236	0.3400001	-0.425572	0.0145374	2.20E-188	0.0318785	0.0442447	0.4712144	0.1023301
ENSG00000134909	ARHGAP32	11	128992087	T	G	0.232604	-0.00785158	0.016901	0.7300002	0.242388	0.00981909	1.53E-134	-0.0323926	0.0697394	0.6423033	0.02765792
ENSG00000134910	STT3A	11	125478358	A	T	0.306163	-0.0282129	0.015623	0.14	-0.307675	0.012746	9.74E-129	0.091697	0.0509194	0.07173055	0.6954578
ENSG00000134917	ADAMTS8	11	130286854	G	T	0.494036	-0.0117646	0.0142645	0.4100001	-0.0642957	0.00833148	1.19E-14	0.182976	0.223121	0.4121723	0.8035754
ENSG00000134954	ETS1	11	128393046	A	C	0.191849	0.00490556	0.0172153	0.5700002	-0.0789766	0.00943523	5.74E-17	-0.0621141	0.218106	0.7758062	0.5094742
ENSG00000134955	SLC37A2	11	124946047	G	C	0.356859	0.00838953	0.0150622	0.5	-0.285061	0.0121483	9.29E-122	-0.0294307	0.0528534	0.5776398	0.07526662
ENSG00000134962	KLB	4	39430814	A	G	0.202783	0.0090091	0.017324	0.35	-0.0611158	0.0107341	1.24E-08	-0.14741	0.284642	0.6045419	0.8741013
ENSG00000134970	TMED7	5	114958947	A	G	0.44831	0.00919653	0.014258	0.3700002	0.0759197	0.00798684	1.99E-21	0.121135	0.188236	0.5198812	0.2461469
ENSG00000134982	APC	5	112112565	G	A	0.132207	-0.02741	0.0215608	0.2	-0.115754	0.0117304	5.74E-23	0.236796	0.187804	0.2073564	0.08231119
ENSG00000134986	NREP	5	111165739	T	C	0.260437	0.00220449	0.0167816	0.9400001	-0.271637	0.0210223	3.41E-38	-0.00811557	0.0617827	0.8954931	0.3440459
ENSG00000134987	WDR36	5	110446807	T	C	0.144135	0.0129752	0.0204902	0.5099998	0.121274	0.0106708	6.25E-30	0.106991	0.169221	0.5272179	0.7501207
ENSG00000134996	OSTF1	9	77732820	G	C	0.27336	0.010446	0.0158284	0.5199996	-0.217143	0.00872368	9.27E-137	-0.0481066	0.0729196	0.5094328	0.4143181
ENSG00000135002	RFK	9	79004933	T	C	0.185885	0.009876	0.0188108	0.7300002	0.121009	0.0105771	2.62E-30	0.0816141	0.155614	0.5999548	0.6779112
ENSG00000135018	UBQLN1	9	86298998	A	C	0.222664	0.0221448	0.0165784	0.1800002	0.104199	0.00923632	1.62E-29	0.212525	0.160215	0.1846754	0.7589739
ENSG00000135040	NAA35	9	88596637	A	G	0.204771	0.00740504	0.0183962	0.6700003	0.23066	0.0100598	2.40E-116	0.0321037	0.0797669	0.6873389	0.9226441
ENSG00000135045	C9orf40	9	77564649	C	A	0.12326	-0.0115878	0.0218515	0.58	0.228143	0.0123824	8.31E-76	-0.0507919	0.0958196	0.5960577	0.7696098
ENSG00000135046	ANXA1	9	75775991	A	G	0.260437	-0.0007248	0.0163706	0.98	-0.410361	0.00880091	0	0.00176625	0.0398932	0.9646856	0.6682251
ENSG00000135047	CTSL	9	90343371	G	C	0.457256	0.0131387	0.0143439	0.6600001	-0.547793	0.00813738	0	-0.0239848	0.0261873	0.3597228	0.05190693
ENSG00000135048	CEMIP2	9	74364944	T	C	0.129225	-0.0291094	0.0241208	0.16	0.219545	0.0133932	2.17E-60	-0.13259	0.110165	0.2287597	0.7735776
ENSG00000135049	AGTPBP1	9	88259199	A	G	0.115308	0.0111985	0.0230049	0.7600007	-0.297739	0.0133282	1.54E-110	-0.0376118	0.0772838	0.6264907	0.04995059
ENSG00000135052	GOLM1	9	88678074	C	T	0.253479	-0.0131318	0.0165622	0.5	0.432989	0.00898883	0	-0.0303282	0.038256	0.4279116	0.3219913
ENSG00000135069	PSAT1	9	80928534	C	T	0.173956	-0.00761064	0.0178324	0.4799997	0.517246	0.00987022	0	-0.0147138	0.0344768	0.6695446	0.2290274
ENSG00000135070	ISCA1	9	88888568	G	A	0.370775	-0.00619559	0.0149464	0.8200001	0.610399	0.0153834	0	-0.0101501	0.0244876	0.6785095	0.08957547
ENSG00000135074	ADAM19	5	156912662	A	G	0.0805169	0.00809746	0.028426	0.99	0.63609	0.0152407	0	0.0127301	0.0446897	0.7757557	0.01504527
ENSG00000135077	HAVCR2	5	156541361	A	G	0.228628	-0.00736416	0.016849	0.7600007	0.237046	0.00943796	3.31E-139	-0.0310664	0.0710899	0.66211	0.6466362
ENSG00000135083	CCNJL	5	159725531	C	T	0.122266	-0.0304093	0.0226136	0.07100027	0.166908	0.0121497	6.04E-43	-0.182192	0.136133	0.1807861	0.5471581
ENSG00000135090	TAOK3	12	118699178	C	T	0.474155	-0.00243324	0.0142695	0.5999997	-0.0927418	0.00878354	4.64E-26	0.0262367	0.153883	0.8646184	0.9080321
ENSG00000135093	USP30	12	109493362	T	C	0.101392	-0.0199462	0.0255471	0.4199997	-0.276913	0.0145516	9.66E-81	0.0720305	0.0923343	0.4353288	0.2624939
ENSG00000135094	SDS	12	113847178	C	T	0.0367793	-0.0458918	0.0379606	0.1900002	-0.233003	0.022985	3.78E-24	0.196958	0.164073	0.2299739	0.2160778
ENSG00000135100	HNF1A	12	121428622	T	C	0.363817	0.00584693	0.0146239	0.4799997	-0.0965021	0.00829883	2.96E-31	-0.0605886	0.151629	0.6894629	0.9787717
ENSG00000135108	FBXO21	12	117604741	A	C	0.501988	0.00764104	0.0144633	0.5099998	-0.0763662	0.00797709	1.04E-21	-0.100058	0.189682	0.5978445	0.9805625
ENSG00000135114	OASL	12	121467570	A	G	0.437376	0.0103586	0.0143243	0.5999997	0.361148	0.00776006	0	0.0286824	0.0396681	0.4696421	0.7764194
ENSG00000135116	HRK	12	117306597	C	T	0.0964215	-0.0197516	0.024126	0.4299995	0.776487	0.0125777	0	-0.0254371	0.0310735	0.4130071	0.2300481

ENSG00000135119	RNFT2	12	117233766	C	T	0.0964215	-0.0187123	0.0241573	0.4700002	0.240329	0.0134918	5.60E-71	-0.0778613	0.100613	0.4390071	0.2744361
ENSG00000135124	P2RX4	12	121659784	T	C	0.293241	0.00292391	0.0156163	0.6300007	0.492694	0.00832716	0	0.00593453	0.0316959	0.8514775	0.04961285
ENSG00000135127	BICDL1	12	120479985	C	G	0.116302	0.00585015	0.0227087	0.8700001	0.133982	0.0119237	2.70E-29	0.0436636	0.169535	0.7967547	0.7754753
ENSG00000135144	DTX1	12	113515173	A	G	0.262425	-0.0145025	0.015855	0.29	-0.0817618	0.00858032	1.59E-21	0.177375	0.194808	0.3625534	0.6451838
ENSG00000135148	TRAFD1	12	112577356	T	C	0.0129225	0.100561	0.0669528	0.2700001	-1.00991	0.0709318	5.34E-46	-0.0995739	0.0666634	0.1352592	0.2139107
ENSG00000135164	DMTF1	7	86803665	A	G	0.136183	-0.0116735	0.0190904	0.56	-0.0680297	0.0109888	5.99E-10	0.171594	0.281984	0.5428399	0.3217114
ENSG00000135185	TMEM243	7	86837690	A	G	0.145129	-0.00832773	0.0188706	0.6700003	-0.183913	0.0116975	1.06E-55	0.0452807	0.102646	0.6591164	0.6785081
ENSG00000135205	CCDC146	7	76855300	T	C	0.167992	0.0269609	0.018787	0.1299999	-0.959294	0.00983467	0	-0.0281049	0.0195863	0.1513078	0.9305196
ENSG00000135211	TMEM60	7	77425471	T	G	0.505964	-0.0192658	0.0141808	0.16	-0.465507	0.00820468	0	0.0413867	0.0304718	0.1744021	0.6748478
ENSG00000135213	NA	7	75080807	T	C	0.338966	-0.0215471	0.0151898	0.1199999	-0.490015	0.0176548	1.50E-169	0.0439723	0.0310391	0.1565779	0.07954179
ENSG00000135218	CD36	7	80153742	A	G	0.442346	0.0120849	0.0143454	0.35	-0.60168	0.00724288	0	-0.0200853	0.0238435	0.3995753	0.7974877
ENSG00000135226	UGT2B28	4	70153492	T	A	0.156064	0.00935301	0.0208567	0.8	-0.42945	0.0304782	4.35E-45	-0.021779	0.0485906	0.6539983	0.7950376
ENSG00000135241	PNPLA8	7	108160488	A	G	0.0198807	-0.126361	0.0632667	0.0179999	-0.655701	0.0320686	6.41E-93	0.192711	0.0969464	0.04683227	0.8339657
ENSG00000135245	HILPDA	7	128097187	G	A	0.371769	0.0176373	0.0148209	0.1800002	0.0873776	0.00975915	3.45E-19	0.201852	0.171111	0.2381376	0.4530641
ENSG00000135249	RINT1	7	105190328	A	G	0.0457256	-0.059065	0.0377377	0.1	0.268162	0.0222084	1.44E-33	-0.220259	0.141905	0.1206238	0.7461352
ENSG00000135250	SRPK2	7	104895453	C	T	0.475149	-0.015389	0.0142431	0.2200002	0.125746	0.00796342	3.62E-56	-0.122382	0.113534	0.2810639	0.1985013
ENSG00000135269	TES	7	115874692	A	G	0.299205	0.028334	0.016102	0.1100001	0.466984	0.00849026	0	0.0606745	0.0344985	0.07861873	0.04503779
ENSG00000135272	MDFIC	7	114610732	C	T	0.130219	-0.0102012	0.0228369	0.6499995	0.157291	0.0120365	5.03E-39	-0.0648555	0.145274	0.6552814	0.9787112
ENSG00000135297	MTO1	6	74195130	A	T	0.255467	0.00785429	0.0164767	0.5999997	-0.437793	0.00889931	0	-0.0179407	0.0376376	0.6335983	0.3138177
ENSG00000135299	ANKRD6	6	90243221	T	C	0.249503	0.00113971	0.0168608	0.98	0.138584	0.0092442	8.36E-51	0.00822399	0.121667	0.9461084	0.04130984
ENSG00000135314	KHDC1	6	73985562	T	C	0.0745527	0.0167437	0.0256967	0.6300007	0.278939	0.0148685	1.59E-78	0.0600263	0.0921784	0.5149194	0.879973
ENSG00000135315	CEP162	6	84885656	G	T	0.340954	0.0173653	0.0154526	0.3400001	0.0789471	0.00930936	2.24E-17	0.219961	0.197445	0.2652621	0.7902105
ENSG00000135317	SNX14	6	86259544	G	A	0.0526839	0.0277006	0.0306159	0.25	0.145867	0.0259948	2.01E-08	0.189903	0.2126	0.3717279	0.02309991
ENSG00000135318	NT5E	6	86182654	G	T	0.143141	-0.00921093	0.0202102	0.7300002	-0.768384	0.010278	0	0.0119874	0.0263027	0.6485712	0.1123796
ENSG00000135334	AKIRIN2	6	88398358	C	G	0.0894632	0.0380591	0.0263002	0.1	0.188039	0.0154595	4.87E-34	0.202399	0.140852	0.1507268	0.6397457
ENSG00000135336	ORC3	6	88338504	C	T	0.0129225	-0.124914	0.0588185	0.02999991	-0.576605	0.0521521	2.05E-28	0.216637	0.103873	0.03701553	0.1312037
ENSG00000135341	MAP3K7	6	91260028	G	A	0.303181	-0.014587	0.015941	0.35	-0.168732	0.00874894	7.04E-83	0.0864506	0.0945815	0.3606992	0.7313772
ENSG00000135362	PRR5L	11	36402296	A	G	0.028827	0.0206575	0.0422724	0.4799997	-0.626625	0.0238121	1.28E-152	-0.0329663	0.067472	0.6251309	0.687313
ENSG00000135363	LMO2	11	33896979	G	C	0.375746	-0.00681414	0.0147139	0.5700002	0.0566543	0.00826347	7.08E-12	-0.120276	0.260305	0.6440411	0.2742135
ENSG00000135365	PHF21A	11	46046928	G	A	0.0874751	-0.0303199	0.0271204	0.2300001	0.181749	0.0150283	1.14E-33	-0.166823	0.149855	0.2656107	0.4019524
ENSG00000135372	NAT10	11	34148183	A	G	0.0805169	0.0367551	0.0249551	0.2	-0.328489	0.0138169	6.15E-125	-0.111892	0.0761151	0.1415534	0.5228719
ENSG00000135378	PRRG4	11	32865579	C	T	0.393638	-0.0209817	0.0144262	0.2099999	0.288109	0.0087509	1.02E-237	-0.0728255	0.0501208	0.146224	0.290404
ENSG00000135387	CAPRIN1	11	34097966	G	C	0.0775348	0.0335799	0.0250521	0.2599998	-0.135466	0.0137278	5.73E-23	-0.247884	0.186631	0.1841102	0.3830501
ENSG00000135390	ATP5MC2	12	54048851	G	A	0.299205	0.0121022	0.0157245	0.4899999	0.129041	0.0087851	7.63E-49	0.0937858	0.122024	0.4421395	0.3398823
ENSG00000135392	DNAJC14	12	56219676	C	T	0.0208748	-0.0232036	0.0472596	0.4899999	0.310675	0.0304828	2.16E-24	-0.0746877	0.152295	0.6238417	0.4027578
ENSG00000135404	CD63	12	56121299	A	G	0.256461	-0.0234345	0.0160956	0.09400046	-0.151747	0.00883758	4.41E-66	0.154432	0.10645	0.1468493	0.4963344
ENSG00000135407	AVIL	12	58201823	T	C	0.318091	-0.0424367	0.0151441	0.008600031	0.409378	0.0134454	1.29E-203	-0.103661	0.0371492	0.005264281	0.7335927
ENSG00000135414	GDF11	12	56143987	A	G	0.327038	-0.0208443	0.0149914	0.1900002	-0.0674603	0.00861715	4.93E-15	0.308986	0.225703	0.1710015	0.6270025
ENSG00000135423	GLS2	12	56873467	A	G	0.32505	0.00833737	0.0151331	0.4400003	0.0652007	0.00853882	2.24E-14	0.127872	0.232704	0.5826572	0.8031343
ENSG00000135424	ITGA7	12	56094089	A	G	0.0397614	-0.0147791	0.045247	0.7300002	0.29911	0.028168	2.44E-26	-0.0494102	0.151344	0.7440633	0.06046839
ENSG00000135426	TESPA1	12	55360166	T	C	0.257455	-0.0198814	0.016162	0.1800002	0.515872	0.00990801	0	-0.0385394	0.0313382	0.218776	0.5294316
ENSG00000135437	RDH5	12	56116320	T	G	0.193837	-0.0190654	0.0166692	0.2599998	-0.770536	0.0192782	0	0.024743	0.0216421	0.2529215	0.06177988
ENSG00000135441	BLOC1S1	12	56111845	C	G	0.0427435	-0.0283512	0.0437392	0.4799997	0.437437	0.0413769	4.02E-26	-0.0648121	0.100178	0.5176497	0.1180912
ENSG00000135446	CDK4	12	58145653	G	A	0.0725646	-0.0598365	0.0267873	0.04700023	0.150461	0.0199726	4.94E-14	-0.397687	0.185696	0.0322256	0.2687456
ENSG00000135452	TSPAN31	12	58137895	G	A	0.0725646	-0.0598365	0.0267873	0.04700023	-0.556551	0.0177118	9.94E-217	0.107513	0.0482524	0.02587112	0.5021499
ENSG00000135457	TFCP2	12	51527186	G	T	0.140159	-0.0066894	0.0211602	0.6800001	-0.546652	0.0110445	0	0.012237	0.0387095	0.7519078	0.0775031
ENSG00000135469	COQ10A	12	56662696	G	A	0.0318091	-0.0986663	0.0499044	0.032	-0.33974	0.0323045	7.23E-26	0.290417	0.149463	0.05200791	0.3206183
ENSG00000135472	FAIM2	12	50279339	A	G	0.167992	0.0167582	0.0189848	0.3599996	0.187026	0.0111565	4.49E-63	0.0896035	0.101649	0.3780497	0.9937142
ENSG00000135473	PAN2	12	56718922	C	A	0.0656064	0.0728781	0.0285274	0.004700023	0.189018	0.0163692	7.63E-31	0.385561	0.154573	0.01261862	0.07274797
ENSG00000135480	KRT7	12	52636137	G	A	0.214712	-0.00939426	0.0179282	0.7199992	0.124051	0.0099433	1.01E-35	-0.0757292	0.144651	0.6006041	0.7731812

ENSG00000135482	ZC3H10	12	56514110	T	G	0.0139165	0.046917	0.0834763	0.5400003	-0.460314	0.0817609	1.80E-08	-0.101924	0.182248	0.5759844	0.3797136
ENSG00000135486	HNRNPA1	12	54677424	T	C	0.0854871	-0.0123788	0.0277159	0.6800001	0.169619	0.0193359	1.75E-18	-0.0729802	0.163613	0.6555583	0.9632786
ENSG00000135503	ACVR1B	12	52368156	C	T	0.209742	8.33E-05	0.0177332	0.99	0.184272	0.00989093	1.82E-77	0.000451961	0.0962338	0.9962528	0.4327306
ENSG00000135506	OS9	12	58101539	T	C	0.333996	-0.0380084	0.0150653	0.02199986	-0.0823396	0.00832436	4.54E-23	0.461606	0.188823	0.01449953	0.6468196
ENSG00000135519	KCNH3	12	49942515	T	A	0.0685885	0.0140303	0.0270438	0.4500005	-0.304468	0.0159962	8.95E-81	-0.0460814	0.0888562	0.6040352	0.7130734
ENSG00000135521	LTV1	6	144174715	G	A	0.192843	-0.0217217	0.0176668	0.28	0.0686851	0.00994543	4.98E-12	-0.31625	0.261259	0.226092	0.7468664
ENSG00000135525	MAP7	6	136767760	T	C	0.0397614	-0.04691	0.0406599	0.28	1.01701	0.0264125	0	-0.0461255	0.0399979	0.2488287	0.4053004
ENSG00000135535	CD164	6	109695739	A	G	0.316103	0.0100427	0.0153787	0.6499995	-0.198555	0.00925854	5.02E-102	-0.050579	0.077489	0.5139342	0.6966021
ENSG00000135537	AFG1L	6	108732048	T	G	0.0347913	-0.0840072	0.0393867	0.02	0.1383	0.0213927	1.01E-10	-0.607429	0.299892	0.04281675	0.81312
ENSG00000135540	NHSL1	6	138878444	T	C	0.2833	-0.0054292	0.0161194	0.59	-0.273582	0.0163951	1.63E-62	0.0198449	0.0589319	0.7363107	0.4861114
ENSG00000135541	AHI1	6	135711792	G	A	0.368787	0.0104192	0.0147101	0.5199996	-0.793237	0.00693741	0	-0.013135	0.0185448	0.4787659	0.5531504
ENSG00000135549	PKIB	6	122920290	T	C	0.230616	0.016439	0.0176665	0.4	-0.275904	0.0101293	2.30E-163	-0.0595823	0.0640686	0.3523837	0.1104124
ENSG00000135587	SMPD2	6	109763544	C	T	0.352883	-0.0126993	0.0150902	0.4100001	0.442788	0.00844525	0	-0.0286803	0.0340844	0.4000953	0.4174405
ENSG00000135596	MICAL1	6	109776218	A	T	0.279324	-0.0100822	0.0161085	0.5400003	-0.417359	0.00954616	0	0.0241571	0.0386002	0.5314271	0.8861356
ENSG00000135597	REPS1	6	139267014	C	T	0.0666004	-0.0566501	0.0266123	0.02999991	0.627858	0.0158272	0	-0.0902276	0.0424469	0.03353155	0.9024338
ENSG00000135604	STX11	6	144490585	C	T	0.0586481	-0.0625558	0.0347681	0.03599979	0.272993	0.0173415	7.77E-56	-0.229148	0.128188	0.07384186	0.09163019
ENSG00000135605	TEC	4	48204840	C	A	0.128231	-0.0271591	0.0201866	0.2200002	0.0977152	0.0111935	2.56E-18	-0.277942	0.209025	0.1836162	0.536626
ENSG00000135617	PRADC1	2	73457750	C	T	0.474155	-0.00396807	0.014181	0.6300007	-0.230121	0.00928745	1.56E-135	0.0172434	0.0616281	0.7796324	0.3427533
ENSG00000135622	SEMA4F	2	74895270	T	G	0.473161	-0.00946245	0.0142237	0.4799997	-0.0456261	0.00800543	1.20E-08	0.207391	0.313861	0.5087578	0.3964405
ENSG00000135624	CCT7	2	73470348	T	C	0.0745527	0.0568239	0.027205	0.03500016	-0.265577	0.0158342	3.89E-63	-0.213964	0.103228	0.03819841	0.3154617
ENSG00000135631	RAB11FIP5	2	73342179	A	G	0.21173	-0.00201504	0.017661	0.7400005	0.356183	0.00946211	0	-0.00565732	0.0495843	0.9091625	0.3238016
ENSG00000135632	SMYD5	2	73447857	T	C	0.0516899	0.0671299	0.030569	0.02100003	0.173962	0.0203775	1.38E-17	0.385887	0.181442	0.03343876	0.405974
ENSG00000135636	DYSF	2	71797375	T	C	0.129225	-0.0150441	0.0216817	0.6700003	0.314898	0.0122718	3.25E-145	-0.0477746	0.0688784	0.487928	0.05392083
ENSG00000135643	KCNMB4	12	70794064	G	A	0.0596421	-0.0107773	0.0316025	0.5	0.796472	0.0162182	0	-0.0135313	0.0396791	0.7330896	0.722887
ENSG00000135655	USP15	12	62732665	G	C	0.332008	0.0375386	0.0153237	0.01400006	0.267323	0.00831603	1.02E-226	0.140424	0.057489	0.01458083	0.2394435
ENSG00000135677	GNS	12	65130226	A	G	0.408549	-0.00109916	0.0143693	0.98	-0.172984	0.008074	7.85E-102	0.00635412	0.0830678	0.9390268	0.6336821
ENSG00000135678	CPM	12	69300663	G	A	0.444334	-0.0059355	0.0143474	0.59	0.132157	0.00801173	3.96E-61	-0.0449126	0.108598	0.6791901	0.1763233
ENSG00000135679	MDM2	12	69220585	A	G	0.208748	-0.00548331	0.0173456	0.83	-0.528707	0.00962585	0	0.0103712	0.0328082	0.7519146	0.334414
ENSG00000135686	KLHL36	16	84691711	T	C	0.347913	0.00360685	0.0152208	0.98	-0.440224	0.00827384	0	-0.00819322	0.0345755	0.8126831	0.604818
ENSG00000135698	MPHOSPH6	16	82192617	C	G	0.485089	-0.00402547	0.014545	0.7600007	0.601878	0.00778884	0	-0.00668819	0.0241662	0.781966	0.823528
ENSG00000135709	KIAA0513	16	85094605	C	A	0.130219	-0.0305318	0.0209969	0.1499999	0.632112	0.0108863	0	-0.0483013	0.0332275	0.1460422	0.7134317
ENSG00000135720	DYNC1LI2	16	66770248	A	C	0.430417	-0.0223174	0.0144945	0.04	-0.138897	0.00805137	1.09E-66	0.160676	0.104769	0.1251234	0.3443936
ENSG00000135723	FHOD1	16	67272425	A	G	0.0715706	0.037589	0.0260124	0.14	-0.546558	0.0146084	2.31E-306	-0.0687741	0.0476286	0.1487488	0.2274022
ENSG00000135736	CCDC102A	16	57558300	T	G	0.026839	0.00555822	0.0391916	0.7300002	-0.773502	0.0261919	1.12E-191	-0.00718578	0.0506683	0.8872221	0.385657
ENSG00000135747	ZNF670-ZNF695	1	247175481	C	T	0.16998	-0.00303762	0.0186217	1	0.142518	0.0103055	1.69E-43	-0.0213139	0.130671	0.8704308	0.02872861
ENSG00000135749	PCNX2	1	233275670	T	C	0.156064	-0.00844901	0.0180185	0.7099994	0.0859538	0.0102039	3.65E-17	-0.0982971	0.209955	0.6396539	0.5103908
ENSG00000135763	URB2	1	229778963	G	A	0.0536779	0.0315078	0.0335431	0.33	-0.161878	0.0189668	1.40E-17	-0.19464	0.208464	0.3504663	0.4011529
ENSG00000135766	EGLN1	1	231530143	A	C	0.131213	-0.00386363	0.0203591	0.7899998	-0.33739	0.0142223	2.11E-124	0.0114515	0.0603449	0.8494909	0.07903901
ENSG00000135775	COG2	1	230803981	G	C	0.407555	-0.00978617	0.0144299	0.4	0.128304	0.008004	7.89E-58	-0.0762732	0.112567	0.4980375	0.5093841
ENSG00000135778	NTPCR	1	233100449	C	T	0.198807	0.00844551	0.0168151	0.6899999	0.983473	0.00833729	0	0.00858744	0.0170978	0.6154896	0.2698354
ENSG00000135821	GLUL	1	182356090	G	C	0.182903	0.0284791	0.017083	0.03699985	-0.208363	0.00974198	1.72E-101	-0.13668	0.0822352	0.09650063	0.5775668
ENSG00000135823	STX6	1	180966954	C	T	0.12326	0.00520811	0.0221531	0.99	-0.369376	0.0122782	7.85E-199	-0.0140997	0.0599762	0.8141396	0.9148933
ENSG00000135828	RNASEL	1	182550580	G	A	0.306163	-0.0365861	0.0154388	0.01700004	0.248966	0.00876368	1.57E-177	-0.146952	0.0622272	0.0181988	0.189938
ENSG00000135837	CEP350	1	180003944	A	G	0.353877	-0.00184023	0.0149064	0.8499999	-0.163164	0.00883229	3.37E-76	0.0112784	0.0913603	0.9017511	0.1374093
ENSG00000135838	NPL	1	182778973	C	T	0.0606362	-0.00256377	0.0313225	0.8700001	-0.394579	0.017177	9.01E-117	0.00649748	0.0793825	0.9347658	0.05439947
ENSG00000135842	NIBAN1	1	184851770	G	A	0.492048	-0.0118968	0.0142125	0.4199997	-0.156559	0.00793032	9.44E-87	0.0759893	0.0908622	0.402978	0.7335862
ENSG00000135845	PIGC	1	172376279	C	T	0.280318	-0.00771841	0.0157877	0.8200001	0.244537	0.00962608	2.30E-142	-0.0315633	0.0645735	0.6249854	0.999519
ENSG00000135847	NA	1	180358302	G	T	0.0497018	0.0109431	0.0313561	0.5300002	-0.513305	0.0222089	3.46E-118	-0.0213189	0.0610937	0.7271235	0.01825213
ENSG00000135862	LAMC1	1	183053661	T	C	0.426441	0.0163971	0.0143596	0.17	-0.592862	0.00736977	0	-0.0276576	0.0242233	0.2535471	0.3365552
ENSG00000135870	RC3H1	1	173945893	G	A	0.0367793	-0.000532502	0.0410597	0.7300002	0.197815	0.0295132	2.05E-11	-0.00269191	0.207566	0.9896526	0.7971979

ENSG00000135898	GPR55	2	231798907	G	C	0.0397614	-0.00207731	0.0334908	0.7400005	0.308917	0.0185027	1.41E-62	-0.0067245	0.108414	0.9505422	0.3157316
ENSG00000135899	SP110	2	231061226	A	G	0.107356	-0.000302486	0.0213889	0.8800001	0.616726	0.0119541	0	-0.00049047	0.0346813	0.9887165	0.671713
ENSG00000135900	MRPL44	2	224827276	C	G	0.119284	0.0307973	0.0202367	0.07699987	-0.139023	0.0172212	6.87E-16	-0.221527	0.148128	0.1347809	0.3105666
ENSG00000135905	DOCK10	2	225768484	C	T	0.141153	0.0237741	0.0194918	0.16	0.46525	0.0118084	0	0.0510996	0.0419154	0.2228011	0.03802769
ENSG00000135912	TTLL4	2	219597853	A	C	0.466203	0.017693	0.01419	0.1499999	0.205387	0.00789367	3.00E-149	0.0861447	0.0691684	0.212972	0.4511535
ENSG00000135913	USP37	2	219374029	A	G	0.377734	0.00226273	0.0145433	0.6999999	0.149714	0.00817174	5.63E-75	0.0151136	0.0971438	0.876364	0.2950255
ENSG00000135916	ITM2C	2	231736658	T	C	0.125249	0.0144496	0.0209379	0.4700002	-0.355029	0.0110082	3.37E-228	-0.0406998	0.0589887	0.49022	0.5221695
ENSG00000135919	SERPINE2	2	224871932	C	A	0.442346	-0.000393493	0.0142559	0.8200001	0.666206	0.00714752	0	-0.000590648	0.0213986	0.9779795	0.9035872
ENSG00000135924	DNAJB2	2	220147805	T	G	0.167992	0.0157279	0.018515	0.32	0.148696	0.0109475	5.08E-42	0.105772	0.124759	0.3965429	0.2601642
ENSG00000135925	WNT10A	2	219754694	T	C	0.176938	-0.0249787	0.0180703	0.1800002	-0.0567733	0.0103292	3.88E-08	0.439973	0.3282	0.1800626	0.497835
ENSG00000135926	TMBIM1	2	219148112	G	A	0.369781	0.00154368	0.0144573	0.7099994	-0.200777	0.00809773	1.04E-135	-0.00768854	0.0720075	0.9149682	0.3944118
ENSG00000135929	CYP27A1	2	219663244	A	G	0.467197	0.0175027	0.0142148	0.1499999	0.854832	0.00635472	0	0.020475	0.0166295	0.2182295	0.2487974
ENSG00000135930	EIF4E2	2	233431558	A	G	0.259443	0.0308601	0.0158486	0.01499996	0.137213	0.00883718	2.29E-54	0.224906	0.116408	0.05335359	0.5715667
ENSG00000135931	ARMC9	2	232151404	C	T	0.322068	-0.0514561	0.0157761	2.00E-04	0.0598138	0.00968393	6.55E-10	-0.860272	0.298269	0.00392397	0.1892164
ENSG00000135932	CAB39	2	231631675	C	T	0.239563	0.00491581	0.0168707	0.9199999	-0.185078	0.00931005	6.14E-88	-0.0265607	0.0911642	0.7707835	0.3534093
ENSG00000135945	REV1	2	100061717	G	C	0.4334	-0.0173659	0.0142724	0.2599998	0.242668	0.00789061	1.08E-207	-0.0715624	0.0588606	0.2240627	0.3349828
ENSG00000135951	TSGA10	2	99692575	T	C	0.389662	0.00876821	0.0146687	0.6200004	-0.624893	0.00733919	0	-0.0140315	0.0234745	0.5500168	0.9299095
ENSG00000135953	MFSD9	2	103342823	G	A	0.233598	-0.0074585	0.017068	0.6499995	0.772762	0.00867277	0	-0.00965174	0.0220873	0.6621243	0.5589256
ENSG00000135956	TMEM127	2	96924022	T	G	0.309145	-0.0204545	0.0151772	0.1100001	0.252689	0.00824447	2.68E-206	-0.0809474	0.0601209	0.1781699	0.4560715
ENSG00000135960	EDAR	2	109558377	A	G	0.0457256	0.0296443	0.0306964	0.3800004	0.175374	0.0183265	1.08E-21	0.169035	0.175923	0.33663	0.8090917
ENSG00000135966	TGFBRAP1	2	105913681	A	C	0.132207	0.0260363	0.021869	0.2200002	-0.347413	0.0116105	1.01E-196	-0.0749434	0.062998	0.2341973	0.570506
ENSG00000135968	GCC2	2	109095444	T	C	0.420477	-0.0171778	0.0144095	0.2599998	-0.736116	0.00701244	0	0.0233357	0.0195763	0.2332458	0.03563202
ENSG00000135972	MRPS9	2	105685429	A	G	0.224652	0.0359512	0.0168217	0.02	-0.582784	0.00908692	0	-0.0616887	0.0288804	0.03267891	0.1052201
ENSG00000135974	C2orf49	2	105959742	T	C	0.11829	0.024405	0.0212978	0.2399999	0.135923	0.018875	5.97E-13	0.179551	0.158662	0.2577794	0.4180281
ENSG00000135976	ANKRD36	2	97854745	A	G	0.270378	0.00485517	0.0158822	0.6200004	-0.189248	0.00951523	5.07E-88	-0.025655	0.0839324	0.7598615	0.2688617
ENSG00000135999	EPC2	2	149473569	G	A	0.188867	-0.00259719	0.018469	0.9199999	-0.0977933	0.0103229	2.71E-21	0.026558	0.188878	0.888179	0.3683897
ENSG00000136003	ISCU	12	108959759	C	T	0.329026	-0.0148759	0.0150361	0.1900002	0.284786	0.00820126	3.35E-264	-0.0522355	0.0528194	0.3226904	0.7184531
ENSG00000136014	USP44	12	95927801	A	G	0.426441	0.0109443	0.0143931	0.4299995	0.204908	0.00873407	1.03E-121	0.0534107	0.0702785	0.4472629	0.8063089
ENSG00000136026	CKAP4	12	106664856	A	C	0.0168986	-0.0312558	0.0638731	0.5	-1.24259	0.0356532	3.97E-266	0.0251538	0.0514085	0.6246336	0.1088501
ENSG00000136040	PLXNC1	12	94621975	G	A	0.126243	0.0278611	0.0216895	0.2300001	0.257072	0.0122463	7.79E-98	0.108379	0.0845293	0.1997918	0.6413432
ENSG00000136044	APPL2	12	105598545	T	G	0.452286	0.0101879	0.0143841	0.5199996	-0.441254	0.00766777	0	-0.0230885	0.0326007	0.4788077	0.3388375
ENSG00000136045	PWP1	12	108093226	T	C	0.192843	-0.017601	0.0173061	0.5	0.495048	0.00920013	0	-0.0355541	0.0349647	0.3092207	0.7031785
ENSG00000136048	DRAM1	12	102338518	A	T	0.309145	0.00899251	0.0154265	0.7700005	0.426412	0.0080606	0	0.0210888	0.0361796	0.5599669	0.5353404
ENSG00000136051	WASHC4	12	105532007	T	C	0.0795229	-0.0121681	0.0266593	0.6999999	-0.35114	0.0139343	4.05E-140	0.0346531	0.0759346	0.6481347	0.6555388
ENSG00000136052	SLC41A2	12	105274426	G	T	0.141153	0.0528484	0.0213356	0.01099993	-0.210205	0.0172085	2.58E-34	-0.251413	0.103565	0.01519922	0.5918017
ENSG00000136059	VILL	3	38039114	C	A	0.141153	-0.0221379	0.0185275	0.3100002	0.183485	0.0102848	3.43E-71	-0.120653	0.101202	0.2331843	0.9002797
ENSG00000136068	FLNB	3	58076054	C	G	0.0725646	0.028833	0.0267192	0.2300001	-0.450332	0.0144184	3.79E-214	-0.0640261	0.0593677	0.2808249	0.8926815
ENSG00000136098	NEK3	13	52720385	G	C	0.33002	-0.0244409	0.014707	0.05999983	-0.232162	0.00802185	3.62E-184	0.105275	0.0634524	0.09709124	0.6503199
ENSG00000136099	PCDH8	13	53420442	T	C	0.145129	-0.00322467	0.0191312	0.8700001	0.373505	0.0108046	7.36E-262	-0.00863355	0.0512214	0.8661478	0.1314655
ENSG00000136100	VPS36	13	53005750	A	C	0.392644	-0.00937347	0.0143045	0.4500005	0.134133	0.00793581	4.33E-64	-0.069882	0.106724	0.5126034	0.2537391
ENSG00000136104	RNASEH2B	13	51514203	G	T	0.026839	-0.0173577	0.0440992	0.5300002	-1.16529	0.0234015	0	0.0148956	0.037845	0.6938812	0.7380271
ENSG00000136108	CKAP2	13	53040163	G	A	0.434394	-0.0076856	0.0141976	0.5500004	0.594101	0.00723458	0	-0.0129365	0.0238981	0.5882876	0.2901445
ENSG00000136111	TBC1D4	13	75957529	G	A	0.380716	0.00645709	0.0144326	0.8499999	0.40426	0.00779426	0	0.0159726	0.0357026	0.6546011	0.5861884
ENSG00000136114	THSD1	13	52965967	G	A	0.0417495	-0.0173702	0.0321382	0.58	0.165139	0.0188527	1.96E-18	-0.105186	0.194984	0.5895705	0.245807
ENSG00000136122	BORA	13	73316198	T	G	0.0218688	-0.0120491	0.0415146	0.95	-1.14089	0.0266311	0	0.0105611	0.0363887	0.7716395	0.7852673
ENSG00000136141	LRCH1	13	47227239	G	A	0.055666	-0.0052379	0.0276842	0.7700005	0.216521	0.0161018	3.21E-41	-0.0241912	0.127872	0.8499493	0.9567671
ENSG00000136143	SUCLA2	13	48561373	A	G	0.055666	0.0473878	0.0292254	0.14	-0.387701	0.016012	1.62E-129	-0.122228	0.0755501	0.105698	0.6556245
ENSG00000136144	RCBTB1	13	50132900	G	A	0.22167	-0.0113357	0.0182917	0.4199997	-0.543893	0.00892462	0	0.0208418	0.0336328	0.5354644	0.009626697
ENSG00000136146	MED4	13	48648363	A	G	0.055666	0.0473878	0.0292254	0.14	0.986216	0.0149724	0	0.0480501	0.0296428	0.1050244	0.5780236
ENSG00000136147	PHF11	13	50086434	G	A	0.482107	0.0245249	0.014225	0.08999948	-0.350238	0.00772748	0	-0.0700235	0.0406446	0.0849203	0.4190427

ENSG00000136152	COG3	13	46074912	T	C	0.417495	-0.0284287	0.0143441	0.02699977	-0.15349	0.00806588	9.70E-81	0.185215	0.0939582	0.04869566	0.0874105
ENSG00000136153	LMO7	13	76314287	A	G	0.126243	0.0140199	0.0233348	0.6600001	-0.117511	0.012519	6.20E-21	-0.119307	0.198982	0.5487808	0.3005123
ENSG00000136156	ITM2B	13	48822178	T	C	0.251491	0.0303719	0.0162384	0.05800027	0.237325	0.00902246	1.74E-152	0.127976	0.0685954	0.06208831	0.3465783
ENSG00000136158	SPRY2	13	80912598	C	A	0.342942	0.00574364	0.0151076	0.6899999	-0.13255	0.00847158	3.52E-55	-0.0433319	0.11401	0.7038935	0.3425466
ENSG00000136159	NUDT15	13	48616530	A	G	0.0407555	-0.0162755	0.0380211	0.7400005	0.143378	0.0199925	7.41E-13	-0.113515	0.265653	0.6691576	0.2071095
ENSG00000136161	RCBTB2	13	49085232	T	C	0.289264	0.0133597	0.0157204	0.4400003	-0.0720379	0.00874141	1.71E-16	-0.185454	0.219381	0.3979159	0.01398942
ENSG00000136167	LCP1	13	46743030	A	G	0.535785	-0.00202767	0.0141992	0.7800007	0.187508	0.00789175	8.66E-125	-0.0108138	0.0757272	0.8864488	0.1936234
ENSG00000136169	SETDB2	13	50043783	A	G	0.309145	0.0223876	0.015454	0.1299999	0.236997	0.0087278	2.25E-162	0.0944635	0.0653002	0.148008	0.9199961
ENSG00000136193	SCRN1	7	29994812	G	C	0.147117	0.0180921	0.0201971	0.3400001	-0.588848	0.0109831	0	-0.0307246	0.0343041	0.3704386	0.3033588
ENSG00000136197	C7orf25	7	42950114	C	T	0.129225	0.0287719	0.0210325	0.1499999	0.740856	0.0117527	0	0.038836	0.0283962	0.1714212	0.6355835
ENSG00000136205	TNS3	7	47468454	A	G	0.328032	-0.0218958	0.0152509	0.1900002	-0.134763	0.00844128	2.25E-57	0.162476	0.113625	0.1527357	0.1218372
ENSG00000136206	SPDYE1	7	44045104	A	G	0.411531	-0.00289457	0.0145179	0.81	0.175654	0.0141087	1.40E-35	-0.0164788	0.0826612	0.841986	0.1590217
ENSG00000136213	CHST12	7	2458732	T	A	0.152087	-0.0171404	0.0191568	0.29	-0.116434	0.0108355	6.22E-27	0.147211	0.165099	0.372577	0.801853
ENSG00000136235	GPNMB	7	23295156	A	G	0.379722	-0.00938633	0.0144386	0.4799997	-0.402791	0.00780015	0	0.0233032	0.0358492	0.51567	0.7447547
ENSG00000136237	RAPGEF5	7	22277309	A	G	0.055666	-0.0632998	0.0363923	0.05099998	-0.204611	0.0297305	5.89E-12	0.309366	0.183453	0.09172855	0.5922342
ENSG00000136238	RAC1	7	6428881	C	G	0.134195	0.0206316	0.0215132	0.2200002	0.30031	0.0128415	5.96E-121	0.068701	0.0716969	0.3379543	0.7294037
ENSG00000136240	KDELR2	7	6504728	C	T	0.246521	-0.00271973	0.0158465	0.8499999	-0.767693	0.00809189	0	0.00354273	0.0206418	0.8637289	0.522366
ENSG00000136243	NUP42	7	23231038	C	T	0.370775	-0.0158279	0.0145527	0.2399999	-0.422871	0.00781012	0	0.0374296	0.034421	0.276857	0.783395
ENSG00000136244	IL6	7	22768562	A	G	0.10338	-0.0305867	0.0213492	0.1100001	-0.117163	0.0123228	1.95E-21	0.261062	0.184275	0.1565728	0.8243235
ENSG00000136247	ZDHHC4	7	6623035	C	G	0.259443	0.0240528	0.0157316	0.1499999	-0.543562	0.00907205	0	-0.0442503	0.0289511	0.1264007	0.2589763
ENSG00000136250	AOAH	7	36658305	T	G	0.395626	-0.0274811	0.0145644	0.05699936	0.587656	0.0074777	0	-0.0467639	0.024791	0.05925126	0.2844075
ENSG00000136261	BZW2	7	16715952	G	C	0.473161	-0.0251865	0.0143188	0.07399971	0.113529	0.00800117	1.07E-45	-0.221852	0.127091	0.08087719	0.2690893
ENSG00000136270	TBRG4	7	45145672	A	G	0.274354	-0.0235393	0.0158784	0.1	0.280154	0.00870548	3.22E-227	-0.0840228	0.0567376	0.1386325	0.8218013
ENSG00000136271	DDX56	7	44609833	G	C	0.408549	0.00224975	0.0143817	0.7600007	0.279918	0.00810544	2.43E-261	0.00803717	0.0513788	0.8756942	0.7450119
ENSG00000136273	HUS1	7	47877253	T	C	0.0984095	0.00424869	0.0224928	0.5400003	0.289779	0.0131913	5.91E-107	0.0146618	0.0776233	0.8501836	0.5974425
ENSG00000136274	NACAD	7	45124275	C	T	0.434394	-0.0183195	0.0142491	0.14	-0.250983	0.0150949	4.44E-62	0.072991	0.0569426	0.1999009	0.1474138
ENSG00000136279	DBNL	7	44096647	G	C	0.0606362	0.0050418	0.0323325	0.98	-0.60536	0.0186799	2.18E-230	-0.0083286	0.053411	0.8760849	0.7238255
ENSG00000136280	CCM2	7	45077571	A	G	0.207753	0.0211576	0.0171796	0.2300001	0.602315	0.00906939	0	0.0351271	0.0285275	0.218195	0.04228685
ENSG00000136286	MYO1G	7	45010479	C	G	0.228628	0.0164078	0.0172847	0.29	0.435175	0.0103061	0	0.0377039	0.039729	0.3426066	0.1007279
ENSG00000136295	TTYH3	7	2688010	T	C	0.106362	-0.00592571	0.0226676	1	0.168688	0.0128389	1.97E-39	-0.0351283	0.134403	0.7938104	0.7748327
ENSG00000136315	NA	14	21387979	A	C	0.424453	-0.0119503	0.0144615	0.5099998	0.758059	0.0165943	0	-0.0157643	0.0190801	0.4086807	0.3876369
ENSG00000136319	TTC5	14	20749435	C	T	0.191849	0.0059175	0.0176254	0.6499995	-0.248543	0.0101071	1.58E-133	-0.0238087	0.0709214	0.7370928	0.6142235
ENSG00000136371	MTHFS	15	80157824	T	C	0.292247	0.00484668	0.0150329	0.6499995	-0.374367	0.00871075	0	-0.0129463	0.0401566	0.7471529	0.669253
ENSG00000136379	ABHD17C	15	81009993	G	A	0.503976	6.35E-05	0.0142123	0.7700005	-0.460387	0.00826773	0	-0.000137946	0.0308703	0.9964346	0.7626223
ENSG00000136381	IREB2	15	78761785	A	G	0.395626	-0.0250339	0.014576	0.09400046	0.484565	0.00775468	0	-0.0516626	0.0300919	0.08601105	0.0036439
ENSG00000136383	ALPK3	15	85388312	T	C	0.464215	0.0194291	0.014236	0.0649995	-0.0669023	0.00795112	3.96E-17	-0.29041	0.215569	0.1779223	0.5696395
ENSG00000136404	TM6SF1	15	83794882	G	A	0.324056	-0.00746242	0.0153106	0.6999999	0.357934	0.0124397	4.59E-182	-0.0208486	0.042781	0.6260229	0.8975297
ENSG00000136425	CIB2	15	78410417	C	A	0.252485	0.00455912	0.0161922	0.8200001	0.161663	0.00905931	3.16E-71	0.0282013	0.100172	0.7783057	0.1701887
ENSG00000136436	CALCOCO2	17	46926117	G	C	0.0467197	-0.0185695	0.0383764	0.5500004	0.454524	0.0185165	4.66E-133	-0.0408548	0.0844485	0.6285391	0.1732507
ENSG00000136444	RSAD1	17	48559748	T	C	0.372763	-0.00744093	0.0144975	0.5500004	0.188913	0.00803299	2.72E-122	-0.0393881	0.0767598	0.6078577	0.3243326
ENSG00000136448	NMT1	17	43157681	C	G	0.488072	-0.00546248	0.0144268	0.8200001	0.191525	0.00949951	2.13E-90	-0.0285209	0.0753391	0.7050091	0.9641787
ENSG00000136449	MYCBPAP	17	48597303	C	T	0.437376	0.01146	0.0143207	0.3800004	-0.293667	0.00791917	5.26E-301	-0.0390237	0.0487764	0.4236795	0.8611591
ENSG00000136451	VEZF1	17	56057265	T	C	0.0536779	-0.0163469	0.0296521	0.3900004	-0.180916	0.0160852	2.39E-29	0.0903562	0.164096	0.5818882	0.07818135
ENSG00000136478	TEX2	17	62282624	G	A	0.0198807	-0.0484668	0.058618	0.32	-0.394472	0.0297069	3.07E-40	0.122865	0.148886	0.4092431	0.6884238
ENSG00000136485	DCAF7	17	61649730	T	C	0.0994036	-0.0122578	0.0231507	0.3900004	0.0745345	0.0130997	1.27E-08	-0.164458	0.311946	0.5980541	0.310635
ENSG00000136490	LIMD2	17	61775897	A	G	0.264414	-0.0288103	0.0159457	0.0329997	-0.159231	0.00942227	4.55E-64	0.180934	0.100713	0.07240915	0.05321373
ENSG00000136492	BRIP1	17	59849754	T	C	0.289264	-0.0113489	0.0157141	0.32	0.0919085	0.00869177	3.93E-26	-0.12348	0.171374	0.4711971	0.857241
ENSG00000136514	RTP4	3	187087992	T	C	0.346918	-0.00104985	0.0148253	0.8499999	0.305076	0.00810944	1.016368e-309	-0.00344127	0.0485954	0.9435453	0.3445928
ENSG00000136521	NDUFB5	3	179333956	G	A	0.471173	-0.00825049	0.0142381	0.5999997	0.160278	0.00797784	8.95E-90	-0.0514761	0.0888707	0.5624375	0.003137511
ENSG00000136522	MRPL47	3	179314257	A	G	0.0795229	-0.0582405	0.027187	0.02300011	0.13064	0.0158314	1.56E-16	-0.445808	0.215004	0.03812677	0.371227

ENSG00000136527	TRA2B	3	185644809	A	G	0.0387674	0.0456429	0.0385338	0.25	-0.120054	0.0190677	3.05E-10	-0.380187	0.326601	0.2443958	0.8897786
ENSG00000136536	MARCHF7	2	160597179	G	C	0.0864811	-0.0158066	0.0242331	0.6300007	-0.270285	0.0134562	9.74E-90	0.0584813	0.0897049	0.5144467	0.6516023
ENSG00000136541	ERMN	2	158179681	T	C	0.0377734	0.0246619	0.0301683	0.3100002	-0.147241	0.0200497	2.08E-13	-0.167494	0.206156	0.4165282	0.1098345
ENSG00000136560	TANK	2	162043075	A	G	0.466203	0.0099832	0.0142016	0.2200002	-0.302358	0.00782379	0	-0.0330178	0.0469772	0.4821512	0.3379185
ENSG00000136573	BLK	8	11386811	A	G	0.266402	0.0238733	0.0160392	0.2200002	-0.443265	0.00851602	0	-0.0538579	0.036199	0.1367967	0.790119
ENSG00000136603	SKIL	3	170095044	T	C	0.0119284	0.0390583	0.0490928	0.4	-0.255303	0.0306376	7.88E-17	-0.152988	0.193167	0.4283608	0.9686999
ENSG00000136628	EPRS1	1	220180971	A	G	0.503976	0.0378933	0.0142009	0.004099964	-0.218361	0.00786522	1.22E-169	-0.173535	0.0653337	0.007904242	0.1965376
ENSG00000136630	HLX	1	221055411	G	A	0.316103	0.0194161	0.0147885	0.08600031	0.110357	0.00830204	2.55E-40	0.175938	0.134658	0.1913617	0.52813
ENSG00000136634	IL10	1	206943393	T	C	0.220676	0.00446121	0.0176937	0.9	-0.198846	0.00946127	4.60E-98	-0.0224356	0.0889885	0.8009503	0.6604849
ENSG00000136643	RPS6KC1	1	213336352	T	C	0.280318	-0.0175421	0.0153773	0.2399999	-0.101736	0.00863725	5.02E-32	0.172427	0.151856	0.2561801	0.5389497
ENSG00000136653	NA	1	206721747	G	A	0.055666	0.00945346	0.0362676	0.9699999	0.244743	0.0200635	3.17E-34	0.0386261	0.148221	0.7944017	0.6130956
ENSG00000136682	ZNG1B	2	114224517	C	G	0.161034	-0.0244851	0.0173922	0.1499999	-0.235057	0.0242988	3.90E-22	0.104167	0.074771	0.163576	0.2676725
ENSG00000136689	IL1RN	2	113878192	T	C	0.112326	0.0500871	0.0231634	0.04099964	0.173208	0.0120009	3.21E-47	0.289173	0.135224	0.0324785	0.126365
ENSG00000136699	SMPD4	2	130924652	T	C	0.342942	0.00333078	0.0147522	0.9400001	0.171135	0.00886887	5.78E-83	0.0194628	0.0862078	0.8213831	0.8613773
ENSG00000136709	WDR33	2	128513678	G	T	0.150099	0.0168248	0.0211716	0.6499995	0.1655	0.0113555	4.09E-48	0.10166	0.128115	0.4274816	0.4160554
ENSG00000136710	CCDC115	2	131097868	A	G	0.2167	-0.0124747	0.0174583	0.4500005	0.441554	0.0106886	0	-0.0282518	0.0395442	0.4749577	0.9712537
ENSG00000136715	SAP130	2	128742242	T	C	0.0646123	-0.000530432	0.0274064	0.7400005	-0.313114	0.0156372	3.43E-89	0.00169406	0.0875286	0.9845585	0.2327491
ENSG00000136717	BIN1	2	127835267	A	G	0.358847	0.0091866	0.015149	0.6300007	0.663793	0.00756544	0	0.0138395	0.0228224	0.544248	0.1481023
ENSG00000136718	IMP4	2	131102590	A	G	0.217694	-0.0132061	0.0174073	0.4299995	0.457648	0.00980616	0	-0.0288542	0.0380385	0.4481201	0.9875528
ENSG00000136720	HS6ST1	2	129035220	A	G	0.299205	0.0330109	0.0157821	0.0179999	0.361	0.0193021	4.71E-78	0.091443	0.0439903	0.03764424	0.6823287
ENSG00000136731	UGGT1	2	128901012	G	A	0.152087	0.0244761	0.0190112	0.17	0.094556	0.0110197	9.44E-18	0.258853	0.203308	0.2029453	0.6625617
ENSG00000136732	GYPC	2	127433877	G	T	0.392644	0.0266889	0.0144993	0.05800027	-0.288847	0.00796425	5.19E-288	-0.0923982	0.0502618	0.06601283	0.5376083
ENSG00000136738	STAM	10	17722018	G	A	0.423459	0.00665393	0.0142572	0.5999997	0.196198	0.0078887	1.54E-136	0.0339143	0.0726801	0.6407686	0.6987114
ENSG00000136754	ABI1	10	27092769	G	T	0.127237	-0.0263925	0.0222081	0.2099999	-0.225131	0.0124805	9.70E-73	0.117232	0.0988593	0.2356827	0.07883627
ENSG00000136758	YME1L1	10	27421789	C	T	0.0526839	-0.00462215	0.0331661	0.89	-0.269034	0.0180318	2.44E-50	0.0171805	0.123284	0.8891676	0.4904036
ENSG00000136770	DNAJC1	10	22169082	T	C	0.326044	0.0107892	0.0150869	0.32	-0.0873666	0.00854642	1.57E-24	-0.123493	0.173107	0.4756023	0.2858999
ENSG00000136783	NIPSNAP3A	9	107516186	C	T	0.462227	-0.00760044	0.0142883	0.3800004	-0.452544	0.00765282	0	0.0167949	0.0315746	0.5947863	0.5039562
ENSG00000136802	LRRC8A	9	131662354	T	G	0.0407555	-0.00016611	0.041459	0.84	0.305662	0.0211087	1.61E-47	-0.000543443	0.135637	0.9968032	0.04711458
ENSG00000136807	CDK9	9	130550512	T	C	0.0119284	0.0412011	0.0590781	0.6200004	-0.379402	0.0312204	5.57E-34	-0.108595	0.15597	0.4862692	0.4635315
ENSG00000136810	TXN	9	113012505	C	T	0.357853	0.0268569	0.0152097	0.04	-0.278456	0.00943568	2.08E-191	-0.0964492	0.0547192	0.07796425	0.04848575
ENSG00000136811	ODF2	9	131240352	C	T	0.246521	0.0226999	0.0176352	0.2999998	0.240185	0.00947131	7.11E-142	0.0945101	0.073518	0.1986045	0.09696196
ENSG00000136813	ECPAS	9	114184998	T	C	0.0318091	0.0449562	0.0344241	0.1199999	0.498072	0.0207052	7.33E-128	0.0902605	0.0692165	0.192223	0.447
ENSG00000136816	TOR1B	9	132569496	G	A	0.272366	-0.00639882	0.01668	0.7600007	-0.46234	0.00913741	0	0.0138401	0.0360784	0.7012666	0.8344231
ENSG00000136819	C9orf78	9	132593855	T	A	0.286282	7.84E-05	0.0162765	0.9599999	-0.764279	0.00822457	0	-0.000102524	0.0212965	0.9961589	0.5008694
ENSG00000136824	SMC2	9	106880119	C	A	0.414513	-0.0054628	0.014479	0.89	0.203425	0.00811409	1.04E-138	-0.0268541	0.0711841	0.7059889	0.9202054
ENSG00000136826	KLF4	9	110249948	G	A	0.0487078	-0.0189463	0.0303003	0.4899999	0.104037	0.0173992	2.24E-09	-0.182112	0.292834	0.5340121	0.1532797
ENSG00000136827	TOR1A	9	132580818	C	T	0.368787	-0.00293888	0.0149175	0.89	-0.314521	0.00835546	0	0.00934398	0.0474299	0.8438227	0.863457
ENSG00000136828	RALGPS1	9	129831249	A	T	0.265408	0.0325981	0.0157612	0.0179999	0.543193	0.00881438	0	0.060012	0.0290322	0.03872605	0.8077355
ENSG00000136830	NIBAN2	9	130304443	C	G	0.163022	-0.0224781	0.0181581	0.2200002	-0.195447	0.00997537	1.77E-85	0.115008	0.0930905	0.2166642	0.4464952
ENSG00000136840	ST6GALNAC4	9	130674741	G	C	0.127237	-0.0124095	0.0213705	0.4500005	-0.213647	0.0115103	6.60E-77	0.0580841	0.100076	0.5616451	0.481599
ENSG00000136842	TMOD1	9	100313742	C	G	0.464215	-0.0032752	0.0142338	0.8800001	-0.253945	0.0084026	1.21E-200	0.0128973	0.0560523	0.8180191	0.1313506
ENSG00000136848	DAB2IP	9	124438572	T	C	0.0139165	-0.0079208	0.0737786	0.8600001	0.239057	0.0379902	3.12E-10	-0.0331335	0.308668	0.9145165	0.5460342
ENSG00000136854	STXBP1	9	130416002	G	A	0.407555	0.0270909	0.0142446	0.025	-0.121754	0.00807101	2.02E-51	-0.222504	0.117921	0.05917372	0.3155533
ENSG00000136856	SLC2A8	9	130165062	G	A	0.363817	-0.00781481	0.014436	0.58	0.418457	0.00778404	0	-0.0186753	0.0344999	0.5882909	0.7995952
ENSG00000136861	CDK5RAP2	9	123246797	T	G	0.153082	0.0112931	0.0187514	0.5999997	-0.725595	0.00956198	0	-0.0155639	0.0258436	0.5470179	0.06721177
ENSG00000136866	ZFP37	9	115809849	T	G	0.0397614	-0.0155898	0.034398	0.6200004	0.616975	0.0195154	2.31E-219	-0.0252681	0.0557584	0.6504251	0.617315
ENSG00000136867	SLC31A2	9	115919819	G	C	0.420477	-0.00926864	0.0145199	0.35	0.238514	0.00800159	3.05E-195	-0.03886	0.0608905	0.5233473	0.4341514
ENSG00000136868	SLC31A1	9	116006241	C	T	0.528827	0.00915579	0.0143338	0.4100001	-0.365641	0.0115773	6.42E-219	-0.0250404	0.0392099	0.5230671	0.4617749
ENSG00000136869	TLR4	9	120472879	G	A	0.250497	0.00033355	0.0158355	0.9299999	-0.607764	0.00833668	0	-0.000548815	0.0260553	0.9831951	0.9587069
ENSG00000136870	ZNF189	9	104167048	A	G	0.0785288	-0.0240096	0.0256423	0.4600002	0.466142	0.0152038	1.97E-206	-0.051507	0.0550352	0.349329	0.3298124

ENSG00000136872	ALDOB	9	104190482	A	G	0.371769	-0.0074063	0.0144587	0.5199996	-0.133101	0.0083216	1.39E-57	0.0556443	0.108685	0.6086679	0.1006881
ENSG00000136874	STX17	9	102700766	A	C	0.446322	0.00835975	0.0142343	0.4400003	0.200172	0.0078703	1.07E-142	0.0417629	0.0711294	0.5571099	0.1964639
ENSG00000136875	PRPF4	9	116046404	C	G	0.193837	-0.0239634	0.0170684	0.28	0.163598	0.00977442	7.00E-63	-0.146477	0.104698	0.1617981	0.2921104
ENSG00000136877	FPGS	9	130566741	A	G	0.429423	-0.00243769	0.0144156	0.9199999	0.0681953	0.0084896	9.53E-16	-0.0357457	0.211434	0.8657468	0.1484645
ENSG00000136888	ATP6V1G1	9	117355339	T	C	0.028827	0.00279397	0.0419094	0.6300007	0.356708	0.0274652	1.44E-38	0.00783266	0.117491	0.9468476	0.2311552
ENSG00000136891	TEX10	9	103089790	A	G	0.222664	-0.0157971	0.0177192	0.5	-0.177943	0.00989086	2.31E-72	0.0887764	0.0997004	0.3732341	0.2565197
ENSG00000136897	MRPL50	9	104155405	T	C	0.230616	-0.0108569	0.0172194	0.4799997	-0.191359	0.00941253	6.95E-92	0.0567359	0.0900282	0.5285631	0.1784977
ENSG00000136908	DPM2	9	130699070	A	G	0.0884692	-0.0312912	0.0240024	0.16	0.299154	0.0136242	7.33E-107	-0.104599	0.0803756	0.1931291	0.2311628
ENSG00000136929	HEMGN	9	100698105	G	A	0.0298211	0.0701984	0.0609775	0.35	0.254022	0.0442933	9.75E-09	0.276347	0.244836	0.2590235	0.2427904
ENSG00000136930	PSMB7	9	127146734	G	A	0.0447316	-0.0903721	0.0448607	0.02300011	-0.344108	0.01885	1.89E-74	0.262627	0.13116	0.04524715	0.5162329
ENSG00000136932	TRMO	9	100675811	T	C	0.375746	-0.0134882	0.01454	0.2599998	0.428276	0.00768765	0	-0.0314942	0.0339548	0.3536498	0.1051098
ENSG00000136933	RABEPK	9	127979629	A	G	0.45328	-0.0055551	0.0142286	0.7800007	-0.436024	0.00762755	0	0.0127404	0.0326334	0.6962343	0.4131675
ENSG00000136935	GOLGA1	9	127675469	C	T	0.420477	-0.00370389	0.0143337	0.7899998	0.158944	0.00804599	7.35E-87	-0.0233032	0.0901887	0.7961119	0.6862669
ENSG00000136937	NCBP1	9	100415969	G	C	0.00497018	-0.0788412	0.0778236	0.3700002	-0.908146	0.124421	2.90E-13	0.0868156	0.0865166	0.3156407	0.1081512
ENSG00000136938	ANP32B	9	100761934	G	A	0.218688	0.0231676	0.0171126	0.14	-0.192016	0.0140838	2.52E-42	-0.120655	0.0895591	0.1779135	0.7468416
ENSG00000136950	ARPC5L	9	127632206	T	C	0.124254	0.0200705	0.0200954	0.2200002	-0.0903053	0.0118244	2.22E-14	-0.222252	0.224422	0.3220137	0.7462325
ENSG00000136960	ENPP2	8	120627509	A	G	0.228628	0.00381261	0.016079	0.9400001	0.231918	0.0089673	1.76E-147	0.0164395	0.0693335	0.8125735	0.3044796
ENSG00000136982	DSCC1	8	120857233	A	G	0.125249	0.00491758	0.0199632	0.7300002	0.348283	0.0108302	6.70E-227	0.0141195	0.0573205	0.8054306	0.651544
ENSG00000136986	DERL1	8	124040033	T	C	0.0616302	0.0447342	0.0260149	0.1100001	0.193276	0.0148809	1.43E-38	0.231453	0.135775	0.08825308	0.7441479
ENSG00000136997	MYC	8	128750677	A	G	0.385686	0.00785056	0.0149608	0.7400005	-0.0808447	0.00909205	6.01E-19	-0.0971067	0.185378	0.6003964	0.7835436
ENSG00000136999	CCN3	8	120432569	A	G	0.184891	-0.0195948	0.0170463	0.4500005	0.196368	0.00990618	1.89E-87	-0.099786	0.0869536	0.2511433	0.1811946
ENSG00000137038	DMAC1	9	7842435	G	A	0.313121	-0.00165781	0.0155404	0.9	0.259157	0.00943629	4.74E-166	-0.00639692	0.0599656	0.9150455	0.07906415
ENSG00000137040	RANBP6	9	6013330	C	G	0.315109	0.012887	0.0159341	0.56	-0.0760719	0.00949711	1.15E-15	-0.169406	0.210526	0.421006	0.7224238
ENSG00000137054	POLR1E	9	37494813	T	C	0.142147	0.00810548	0.0189541	0.4500005	-0.570067	0.0106336	0	-0.0142185	0.03325	0.6689251	0.7709188
ENSG00000137055	PLAA	9	26925771	C	T	0.213718	0.00507793	0.017207	0.5	0.0895473	0.00980502	6.68E-20	0.0567067	0.192256	0.7680287	0.1336462
ENSG00000137070	IL11RA	9	34656294	G	A	0.102386	-0.0297238	0.0241057	0.2999998	0.788934	0.0128099	0	-0.0376759	0.0305609	0.2176452	0.9390764
ENSG00000137073	UBAP2	9	33985319	C	T	0.0119284	-0.0269113	0.0567177	0.6700003	0.252331	0.0380807	3.44E-11	-0.106651	0.225351	0.6360233	0.802317
ENSG00000137074	APTX	9	32998885	T	A	0.417495	-0.00696605	0.0144481	0.5700002	-0.230939	0.00874375	1.00E-153	0.030164	0.0625728	0.6297614	0.177891
ENSG00000137075	RNF38	9	36411969	T	C	0.0675944	-0.0397242	0.0265568	0.1499999	-0.216999	0.0139743	2.23E-54	0.183062	0.122949	0.1365063	0.8612546
ENSG00000137094	DNAJB5	9	34994267	A	G	0.083499	0.0442685	0.0257594	0.05099998	0.482495	0.0153591	1.29E-216	0.0917492	0.0534677	0.08616718	0.3686839
ENSG00000137098	SPAG8	9	35810155	G	A	0.311133	-0.0156909	0.0153269	0.4	0.167243	0.00863968	1.76E-83	-0.0938207	0.0917723	0.3066293	0.39558
ENSG00000137100	DCTN3	9	34617031	A	G	0.0815109	0.0126926	0.0233387	0.3400001	-0.241987	0.0134994	7.42E-72	-0.0524515	0.0964903	0.5867213	0.4573867
ENSG00000137101	CD72	9	35628168	A	G	0.213718	-0.00839564	0.0172882	0.6300007	-0.158969	0.0145469	8.47E-28	0.0528132	0.10886	0.6275703	0.466962
ENSG00000137103	TMEM8B	9	35834646	T	C	0.0934394	0.0647927	0.0266239	0.02	0.254466	0.0151183	1.43E-63	0.254623	0.105715	0.01601457	0.1000058
ENSG00000137106	GRHPR	9	37429825	T	C	0.348907	0.0199036	0.0150585	0.1100001	0.854513	0.0070011	0	0.0232923	0.0176233	0.1862766	0.313171
ENSG00000137124	ALDH1B1	9	38395659	G	A	0.0646123	0.00187413	0.0271548	0.84	0.197934	0.0180078	4.19E-28	0.00946845	0.137194	0.9449775	0.8084376
ENSG00000137135	ARHGEF39	9	35667367	T	C	0.457256	0.00143699	0.0142657	0.7300002	0.139662	0.00885212	4.47E-56	0.0102891	0.102147	0.9197661	0.1534333
ENSG00000137145	DENND4C	9	19302286	C	T	0.465209	0.0165119	0.0143323	0.2399999	0.188199	0.0087249	3.41E-103	0.0877362	0.0762634	0.2499642	0.6603275
ENSG00000137154	RPS6	9	19377982	T	C	0.0954274	-0.0336573	0.0260792	0.1900002	-0.18511	0.0256948	5.84E-13	0.181823	0.143128	0.2039575	0.3921223
ENSG00000137161	CNPY3	6	42901981	A	G	0.474155	-0.0200382	0.014275	0.2	0.131467	0.00801279	1.70E-60	-0.15242	0.108979	0.1619278	0.58708
ENSG00000137166	FOXP4	6	41542143	A	G	0.227634	-0.00187847	0.0176863	0.9299999	0.109249	0.00980037	7.37E-29	-0.0171944	0.161898	0.9154192	0.06031551
ENSG00000137168	PPIL1	6	36832701	C	T	0.464215	0.00847977	0.0143085	0.6800001	0.145762	0.00793956	2.80E-75	0.0581753	0.0982143	0.5536296	0.7585814
ENSG00000137171	KLC4	6	43025676	A	G	0.027833	-0.0324145	0.0372642	0.4600002	0.262196	0.0252539	2.98E-25	-0.123627	0.142621	0.386041	0.5503779
ENSG00000137177	KIF13A	6	17873634	A	G	0.134195	-0.0295455	0.0203321	0.08600031	0.237357	0.0112496	8.09E-99	-0.124477	0.0858635	0.147139	0.7499119
ENSG00000137185	ZSCAN9	6	28196962	T	G	0.0646123	-0.0250018	0.0374655	0.3800004	1.13746	0.0192519	0	-0.0219804	0.0329399	0.5045894	0.04224266
ENSG00000137193	PIM1	6	37140590	T	C	0.0387674	0.00168055	0.0338294	0.6800001	0.403364	0.0235756	1.26E-65	0.00416633	0.0838685	0.9603798	0.190764
ENSG00000137198	GMPR	6	16267295	C	T	0.175944	-0.0345912	0.0196834	0.02999991	-0.0879521	0.0103773	2.34E-17	0.393296	0.228557	0.08529133	0.5883064
ENSG00000137200	CMTR1	6	37425799	T	C	0.054672	0.0739348	0.0384215	0.04300015	0.263951	0.0286101	2.81E-20	0.280108	0.148696	0.05959644	0.6800005
ENSG00000137207	YIPF3	6	43482146	C	G	0.026839	0.0193386	0.0423229	0.5300002	0.450416	0.0263063	1.02E-65	0.042935	0.0939974	0.6478383	0.5824174
ENSG00000137210	TMEM14B	6	10839324	T	C	0.0218688	-0.0666763	0.0462146	0.16	-0.542019	0.0285351	1.88E-80	0.123015	0.0855094	0.150261	0.2245298

ENSG00000137216	TMEM63B	6	44108953	T	A	0.0974155	-0.0142204	0.0221949	0.6999999	0.365391	0.0141016	4.95E-148	-0.0389183	0.0607615	0.5218411	0.4619102
ENSG00000137218	FRS3	6	41746097	T	C	0.318091	0.0253894	0.0155148	0.09400046	0.178294	0.00874752	2.41E-92	0.142402	0.0872983	0.1028452	0.3727049
ENSG00000137221	TJAP1	6	43459777	G	A	0.0298211	0.0160495	0.0433426	0.5999997	0.16787	0.0251212	2.35E-11	0.0956069	0.258588	0.7115862	0.7422499
ENSG00000137225	CAPN11	6	44139343	T	C	0.287276	-0.0417437	0.0163216	0.006900014	-0.241844	0.00887708	1.98E-163	0.172606	0.067785	0.01088473	0.9179418
ENSG00000137261	KIAA0319	6	24595357	T	C	0.33002	-0.01523	0.0147351	0.3100002	0.315332	0.00807952	0	-0.0482984	0.0467453	0.301499	0.2387022
ENSG00000137265	IRF4	6	401593	T	C	0.116302	0.0470769	0.0168804	3.90E-09	0.16724	0.0148136	1.48E-29	0.281493	0.103969	0.006779937	0.2949279
ENSG00000137266	SLC22A23	6	3363226	T	C	0.403579	0.0200038	0.0145462	0.1499999	-0.292622	0.00795377	2.63E-296	-0.0683604	0.0497445	0.16937	0.5207693
ENSG00000137267	TUBB2A	6	3155831	C	A	0.313121	-0.0148594	0.015244	0.2599998	0.70111	0.00956157	0	-0.0211941	0.0217446	0.3297169	0.5277265
ENSG00000137269	LRRC1	6	53724107	G	C	0.21173	-0.0138558	0.0167284	0.6300007	0.357205	0.00935422	0	-0.0387895	0.0468424	0.4076222	0.1510435
ENSG00000137270	GCM1	6	53002694	T	G	0.159046	-0.0131022	0.0187539	0.5500004	-0.330402	0.0109016	9.13E-202	0.0396554	0.056776	0.4848939	0.8785401
ENSG00000137274	BPHL	6	3136210	T	C	0.263419	-0.0110306	0.0161854	0.4400003	-0.145488	0.00892544	9.80E-60	0.0758178	0.111346	0.4959218	0.7946267
ENSG00000137275	RIPK1	6	3089823	C	A	0.0656064	-0.0108842	0.0377977	0.8499999	0.551543	0.0179227	5.93E-208	-0.0197341	0.0685338	0.7733875	0.4827241
ENSG00000137285	TUBB2B	6	3228229	G	A	0.315109	-0.0124489	0.0153253	0.2999998	-0.173142	0.0100414	1.27E-66	0.0719001	0.0886113	0.41713	0.6627748
ENSG00000137288	UQCC2	6	33672424	C	T	0.234592	-0.026492	0.0165363	0.06100002	-0.259284	0.0172529	4.78E-51	0.102174	0.0641382	0.1111545	0.5344676
ENSG00000137310	TCF19	6	31130627	T	C	0.112326	-0.0422786	0.028582	0.05600025	0.900721	0.0188554	0	-0.0469386	0.0317475	0.1392753	0.08590439
ENSG00000137312	FLOT1	6	30702998	G	A	0.0864811	0.0766462	0.0197344	2.60E-06	0.567285	0.0235411	2.65E-128	0.135111	0.0352364	0.000125869	0.2812636
ENSG00000137331	IER3	6	30711653	G	A	0.0864811	0.0766462	0.0197344	2.60E-06	0.562567	0.0186339	3.16E-200	0.136244	0.0353683	0.000117089	0.240508
ENSG00000137337	MDC1	6	30676625	C	T	0.0188867	-0.103639	0.0683469	0.08199927	0.748386	0.0555636	2.38E-41	-0.138483	0.0919027	0.1318497	0.1094713
ENSG00000137338	PGBD1	6	28259820	G	A	0.332008	0.00317756	0.0148305	0.6800001	0.142254	0.00839586	2.15E-64	0.0223372	0.104262	0.8303589	0.3238605
ENSG00000137364	TPMT	6	18141923	A	T	0.482107	0.00952626	0.0142854	0.58	0.404492	0.00766743	0	0.0235512	0.0353198	0.5049001	0.5182241
ENSG00000137393	RNF144B	6	18418942	T	C	0.398608	0.0021934	0.014425	0.8800001	-0.301333	0.0078644	0	-0.00727898	0.047871	0.8791441	0.8190446
ENSG00000137404	NRM	6	30657510	A	G	0.00497018	-0.00604718	0.0769828	0.7700005	0.820217	0.0367804	3.66E-110	-0.00737266	0.0938572	0.9373891	0.8492603
ENSG00000137409	MTCH1	6	36944995	A	G	0.000994036	-0.209816	0.208818	0.2599998	-1.0426	0.0530317	4.75E-86	0.201243	0.200547	0.3156343	NA
ENSG00000137411	VARS2	6	30885127	C	T	0.302187	0.0391615	0.0145986	2.00E-04	-0.257749	0.012729	3.63E-91	-0.151937	0.0571337	0.007829923	0.09886863
ENSG00000137414	FAM8A1	6	17606268	A	G	0.544732	-0.00625227	0.0142634	0.6700003	0.152014	0.00795068	1.73E-81	-0.0411296	0.0938543	0.6612207	0.07891972
ENSG00000137434	C6orf52	6	10683340	C	T	0.000994036	-0.0807593	0.0923205	0.28	0.596772	0.081963	3.31E-13	-0.135327	0.155812	0.385107	0.5775363
ENSG00000137441	FGFBP2	4	15966399	T	G	0.362823	-0.00710071	0.0146101	0.64	-0.357384	0.0079504	0	0.0198686	0.0408831	0.6269775	0.8274795
ENSG00000137449	CPEB2	4	15038037	C	G	0.33499	0.0210727	0.0148623	0.1199999	-0.286965	0.00824834	3.37E-265	-0.0734331	0.0518344	0.1565746	0.9934989
ENSG00000137460	FHDC1	4	153879176	T	C	0.410537	-0.00577133	0.0144634	0.91	0.176911	0.0080071	3.59E-108	-0.0326229	0.0817688	0.6899191	0.7726104
ENSG00000137462	TLR2	4	154624751	C	T	0.197813	-0.0188054	0.018419	0.2700001	0.158519	0.0164231	4.81E-22	-0.118632	0.116843	0.3099561	0.2602984
ENSG00000137474	MYO7A	11	76882796	A	G	0.0636183	0.014659	0.0278667	0.56	0.345742	0.0163272	1.60E-99	0.0423987	0.0806245	0.5989731	0.177301
ENSG00000137478	FCHSD2	11	72700548	T	A	0.45825	0.000234219	0.0143039	0.98	0.0587752	0.00797125	1.66E-13	0.00398499	0.243367	0.9869357	0.5200317
ENSG00000137486	ARRB1	11	75019049	T	C	0.131213	0.0233167	0.0215911	0.2999998	-0.347005	0.0127114	4.38E-164	-0.0671941	0.0622699	0.280553	0.9383013
ENSG00000137492	THAP12	11	76076507	C	T	0.0536779	-0.0789461	0.0332966	0.009400046	0.393264	0.0384437	1.46E-24	-0.200746	0.0869117	0.02090112	0.3044829
ENSG00000137494	ANKRD42	11	82938258	T	C	0.373757	-0.00465646	0.014529	0.58	0.156404	0.00812211	1.24E-82	-0.029772	0.0929068	0.7486274	0.8778378
ENSG00000137496	IL18BP	11	71713174	T	A	0.133201	-0.0370959	0.0219804	0.09200046	-0.145235	0.012459	2.11E-31	0.25542	0.152922	0.09486663	0.6600748
ENSG00000137497	NUMA1	11	71752824	A	G	0.486083	0.00790463	0.0142409	0.5500004	0.189046	0.00791568	4.67E-126	0.0418134	0.0753509	0.5789525	0.08706901
ENSG00000137500	CCDC90B	11	82983794	C	T	0.332008	0.00357196	0.0147135	0.9299999	0.0859737	0.00894944	7.50E-22	0.0415471	0.171194	0.8082455	0.5039669
ENSG00000137501	SYTL2	11	85463725	A	G	0.342942	0.0115525	0.0149773	0.4199997	-0.39339	0.00814244	0	-0.0293665	0.0380772	0.4405673	0.8227311
ENSG00000137502	RAB30	11	82733570	G	C	0.447316	-0.000594482	0.0142886	0.9699999	-0.0674196	0.0080336	4.77E-17	0.00881765	0.211938	0.9668137	0.5715147
ENSG00000137504	CREBZF	11	85382351	G	T	0.146123	0.00221615	0.0206259	0.89	-0.06607	0.0110833	2.50E-09	-0.0335425	0.312233	0.9144498	0.4433236
ENSG00000137507	LRRC32	11	76375179	A	G	0.22167	0.0132136	0.0177529	0.5300002	-0.108336	0.00968548	4.81E-29	-0.121969	0.164231	0.457685	0.5912554
ENSG00000137509	PRCP	11	82608085	T	G	0.27833	-0.0135008	0.0155511	0.4100001	-0.212462	0.00861267	2.32E-134	0.0635444	0.0732399	0.3856023	0.737602
ENSG00000137513	NARS2	11	78216463	A	G	0.234592	0.0200022	0.0178103	0.2099999	-0.457101	0.00925296	0	-0.0437588	0.0389737	0.2615313	0.5502853
ENSG00000137522	RNF121	11	71674195	A	G	0.0159046	-0.0129211	0.0601996	0.64	0.373261	0.0393885	2.63E-21	-0.0346168	0.161321	0.8300928	0.8469947
ENSG00000137547	MRPL15	8	55054115	C	G	0.182903	0.0170624	0.0202752	0.5400003	0.170506	0.0115978	6.30E-49	0.100069	0.119107	0.4008158	0.477695
ENSG00000137563	GGH	8	63939684	A	C	0.0924453	-0.00849026	0.023584	0.58	0.295061	0.0133018	5.13E-109	-0.0287746	0.0799398	0.7188819	0.7932794
ENSG00000137571	SLCO5A1	8	70665933	G	T	0.403579	-0.00709344	0.0144418	0.5400003	0.0530803	0.00824664	1.22E-10	-0.133636	0.272865	0.6243106	0.9971042
ENSG00000137574	TGS1	8	56711854	C	G	0.109344	0.0404901	0.0228864	0.09099971	-0.528189	0.0119086	0	-0.0766583	0.0433644	0.0770991	0.6501745
ENSG00000137575	SDCBP	8	59480451	G	A	0.0139165	0.0394284	0.0622897	0.4700002	0.287275	0.032426	8.04E-19	0.13725	0.217382	0.5277955	0.8223332

ENSG00000137601	NEK1	4	170424103	A	G	0.371769	-0.0170255	0.0145957	0.2300001	0.292853	0.00812584	2.00E-284	-0.0581367	0.0498658	0.2436698	0.8708373
ENSG00000137628	DDX60	4	169188701	G	T	0.0646123	0.0146937	0.030734	0.56	0.227176	0.0169398	5.23E-41	0.0646798	0.135373	0.6328003	0.2230975
ENSG00000137656	BUD13	11	116631295	C	T	0.421471	-0.0248109	0.0144035	0.07199959	0.145235	0.00796875	3.23E-74	-0.170833	0.0996156	0.08636025	0.5633758
ENSG00000137672	TRPC6	11	101532794	C	T	0.506958	0.00990117	0.0141931	0.4600002	0.209867	0.00786203	5.56E-157	0.0471782	0.067652	0.4855735	0.333511
ENSG00000137692	DCUN1D5	11	102947874	A	G	0.0646123	-0.00429334	0.0264455	0.7300002	0.158995	0.022564	1.84E-12	-0.027003	0.166373	0.8710667	0.5066804
ENSG00000137700	SLC37A4	11	118898220	G	A	0.270378	0.0104898	0.0159138	0.4	0.166537	0.00899384	1.51E-76	0.0629878	0.0956176	0.5100586	0.7597299
ENSG00000137709	POU2F3	11	120149001	T	C	0.0815109	-0.0344346	0.0277353	0.1499999	0.268466	0.0168062	1.93E-57	-0.128264	0.103622	0.2157861	0.8690622
ENSG00000137710	RDX	11	110106526	A	G	0.353877	-0.00292337	0.0150175	0.9199999	-0.139648	0.0127089	4.36E-28	0.0209338	0.107555	0.8456797	0.9388961
ENSG00000137713	PPP2R1B	11	111617391	C	G	0.326044	-0.0263656	0.0151316	0.07699987	0.278679	0.00837976	1.66E-242	-0.0946093	0.0543721	0.08185319	0.1989159
ENSG00000137714	FDX1	11	110318106	G	C	0.280318	0.00799077	0.015908	0.5700002	0.320498	0.00883877	6.79E-288	0.0249323	0.04964	0.6154824	0.3980909
ENSG00000137720	CFAP68	11	111753179	C	A	0.323062	0.0206215	0.0156424	0.2099999	-0.140385	0.00892559	9.66E-56	-0.146892	0.111816	0.1889467	0.5095388
ENSG00000137726	FXYD6	11	117727947	G	A	0.459245	0.0245715	0.0143174	0.08600031	0.333968	0.00772848	0	0.0735743	0.0429043	0.08637336	0.6107642
ENSG00000137731	FXYD2	11	117685929	A	G	0.290258	-0.0153856	0.0159471	0.3400001	-0.126423	0.00951137	2.58E-40	0.121699	0.126473	0.3359199	0.01439953
ENSG00000137747	TMPRSS13	11	117785766	A	G	0.494036	0.0142357	0.0142747	0.2599998	-0.0834492	0.00796089	1.04E-25	-0.170591	0.171831	0.3208147	0.3914702
ENSG00000137752	CASP1	11	104934164	G	A	0.0715706	0.0489139	0.0284057	0.1199999	0.476604	0.0147974	1.33E-227	0.10263	0.0596853	0.08551989	0.03857994
ENSG00000137757	CASP5	11	104879428	G	T	0.0715706	0.0498503	0.028333	0.1100001	0.539283	0.0146561	2.15E-296	0.0924381	0.0525983	0.07884368	0.1761499
ENSG00000137760	ALKBH8	11	107404962	T	C	0.446322	-0.00868995	0.0143007	0.7199992	-0.195632	0.00794033	4.97E-134	0.0444198	0.0731222	0.5435362	0.04392918
ENSG00000137764	MAP2K5	15	67967254	C	T	0.256461	-0.0317686	0.0160507	0.02100003	-0.5758	0.00852855	0	0.055173	0.0278875	0.04788183	0.4348282
ENSG00000137767	SQOR	15	45953419	C	A	0.196819	0.000413929	0.0175169	0.9299999	0.363821	0.0144729	1.91E-139	0.00113773	0.0481471	0.9811475	0.2938771
ENSG00000137776	SLTM	15	59198548	A	G	0.05666	-0.0412412	0.0283676	0.1299999	0.104775	0.0171235	9.43E-10	-0.393616	0.278285	0.1572336	0.919303
ENSG00000137801	THBS1	15	39882473	T	C	0.28827	-0.0303086	0.0161751	0.09499921	0.438253	0.00832921	0	-0.0691578	0.0369315	0.06112444	0.4397859
ENSG00000137802	MAPKBP1	15	42093342	T	C	0.45328	-0.00197923	0.0142833	0.7300002	-0.120451	0.00872286	2.26E-43	0.0164319	0.118588	0.8897958	0.2317025
ENSG00000137804	NUSAP1	15	41649070	T	C	0.000994036	0.087477	0.069151	0.2099999	1.27782	0.0833587	4.88E-53	0.0684579	0.0543002	0.2074066	0.4647264
ENSG00000137806	NDUFAF1	15	41687134	A	G	0.0387674	-0.0434498	0.0366736	0.2200002	0.649852	0.0286384	5.42E-114	-0.0668611	0.0565107	0.2367462	0.6007851
ENSG00000137807	KIF23	15	69723674	C	A	0.0506958	0.0139889	0.0402587	0.89	0.282871	0.0201157	6.48E-45	0.0494534	0.142365	0.728314	0.227992
ENSG00000137814	HAUS2	15	42851600	C	A	0.00596421	-0.0696954	0.0563974	0.1100001	-0.44728	0.0680397	4.90E-11	0.155821	0.128298	0.2245503	0.3879213
ENSG00000137815	RTF1	15	41738183	T	C	0.45825	0.0126559	0.0142313	0.3700002	-0.164035	0.00796946	3.90E-94	-0.0771535	0.0868384	0.3742872	0.6818924
ENSG00000137817	PARP6	15	72549431	T	G	0.0258449	0.00340979	0.0444802	0.9400001	0.260246	0.0247288	6.70E-26	0.0131022	0.17092	0.9388968	0.7152434
ENSG00000137818	RPLP1	15	69746689	C	T	0.0506958	0.0236077	0.0407259	0.6999999	0.266586	0.0236084	1.44E-29	0.0885556	0.152969	0.5626495	0.5450556
ENSG00000137819	PAQR5	15	69645702	T	C	0.140159	-0.0173205	0.0202774	0.28	0.0976454	0.010688	6.48E-20	-0.177382	0.208569	0.3950647	0.2706363
ENSG00000137822	TUBGCP4	15	43680356	C	T	0.195825	-0.00189707	0.0179752	0.7099994	-0.295239	0.0100378	3.79E-190	0.00642554	0.0608839	0.9159493	0.3161405
ENSG00000137824	RMDN3	15	41038065	G	T	0.507952	-0.00634718	0.0142009	0.7199992	0.283163	0.00877852	2.86E-228	-0.0224153	0.0501557	0.6549377	0.8846035
ENSG00000137825	ITPKA	15	41790669	G	A	0.274354	0.0114794	0.0160748	0.4299995	0.102004	0.00915043	7.37E-29	0.112539	0.157914	0.4760543	0.8381132
ENSG00000137831	UACA	15	71001412	C	G	0.106362	-0.0164402	0.0248158	0.3100002	0.272143	0.0127241	1.73E-101	-0.0604102	0.0912305	0.5078612	0.6395538
ENSG00000137834	SMAD6	15	67034452	T	C	0.250497	-0.0124983	0.0164491	0.2200002	-0.106486	0.00914518	2.46E-31	0.11737	0.1548	0.4483288	0.06229835
ENSG00000137841	PLCB2	15	40585256	G	A	0.517893	-0.0161615	0.0142109	0.2	-0.232042	0.00902326	7.74E-146	0.069649	0.0613026	0.2558936	0.5225814
ENSG00000137842	TMEM62	15	43446410	C	T	0.0178926	0.0558856	0.0546849	0.2999998	0.309406	0.0333361	1.67E-20	0.180622	0.17781	0.3097163	0.0648138
ENSG00000137843	PAK6	15	40539658	T	C	0.307157	-0.0108509	0.0152092	0.6300007	-0.0563103	0.00983309	1.02E-08	0.192698	0.272184	0.4789638	NA
ENSG00000137845	ADAM10	15	58964790	T	C	0.0864811	-0.00268967	0.0270393	0.7300002	0.655754	0.0148062	0	-0.00410164	0.041234	0.9207632	0.6238733
ENSG00000137857	DUOX1	15	45439952	T	C	0.132207	0.00099399	0.0221266	0.98	-0.171244	0.0117308	2.90E-48	-0.00580453	0.129212	0.964169	0.8577816
ENSG00000137871	ZNF280D	15	57066574	G	C	0.163022	-0.00865112	0.0201272	0.4	-0.0834393	0.0109916	3.17E-14	0.103682	0.241606	0.667825	0.4810676
ENSG00000137876	RSL24D1	15	55481134	A	G	0.191849	0.0123225	0.0175596	0.3700002	0.300956	0.0107752	1.14E-171	0.0409446	0.0583646	0.4829714	0.040459
ENSG00000137877	SPTBN5	15	42163310	A	C	0.432406	0.0064796	0.0143874	0.6800001	-0.28128	0.00801409	7.18E-270	-0.0230361	0.051154	0.6524731	0.1193976
ENSG00000137880	GCHFR	15	41058062	G	A	0.149105	-0.00958057	0.0194137	0.5300002	-0.318212	0.0103906	5.67E-206	0.0301075	0.0610167	0.6217079	0.7441963
ENSG00000137936	BCAR3	1	94170026	G	C	0.162028	0.0118355	0.0187564	0.33	-0.169672	0.0120722	7.20E-45	-0.0697551	0.110656	0.5284484	0.5726556
ENSG00000137942	FNBP1L	1	93966938	C	T	0.0914513	0.0259444	0.0250621	0.4400003	0.373501	0.0138062	3.50E-161	0.0694628	0.0671497	0.300927	0.3063145
ENSG00000137944	KYAT3	1	89430046	A	G	0.477137	-0.00381975	0.0141979	0.99	-0.424089	0.00827973	0	0.00900695	0.033479	0.7879046	0.09482785
ENSG00000137947	GTF2B	1	89338121	T	C	0.336978	-0.0195105	0.014609	0.29	0.129384	0.00836854	6.38E-54	-0.150795	0.113332	0.1833346	0.4394907
ENSG00000137955	RABGGTB	1	76256321	C	T	0.307157	0.0115421	0.0157598	0.5	-0.134593	0.00896892	6.65E-51	-0.0857555	0.117232	0.4644705	0.5272793
ENSG00000137959	IFI44L	1	79097045	C	G	0.335984	0.0130619	0.0151327	0.3400001	0.0612499	0.00914423	2.11E-11	0.213256	0.249108	0.3919543	0.6092896

ENSG00000137965	IFI44	1	79122622	G	A	0.38668	-0.00784124	0.0155342	0.6499995	0.0973175	0.00827629	6.38E-32	-0.0805738	0.159771	0.6140448	0.1220201
ENSG00000137968	SLC44A5	1	75872308	T	A	0.460239	-0.00504371	0.0141753	0.8	0.380743	0.00776234	0	-0.013247	0.0372316	0.7219901	0.7317714
ENSG00000137992	DBT	1	100687563	T	C	0.0188867	0.00474428	0.0510067	0.99	0.160788	0.026319	1.00E-09	0.0295064	0.317265	0.9259019	0.4619977
ENSG00000137996	RTCA	1	100745044	G	A	0.0526839	0.0094728	0.0321499	0.95	0.389067	0.0201312	3.21E-83	0.0243475	0.0826429	0.7682914	0.9222824
ENSG00000138002	IFT172	2	27689947	A	G	0.0387674	0.0196761	0.0322983	0.4299995	0.333849	0.0398644	5.54E-17	0.0589372	0.097001	0.5434569	0.6464821
ENSG00000138018	SELENOI	2	26575087	T	C	0.10338	-0.0174985	0.0234899	0.64	0.177713	0.0132794	7.64E-41	-0.0984648	0.132383	0.4570061	0.3193858
ENSG00000138028	CGREF1	2	27331876	A	G	0.37674	-0.0053803	0.0146791	0.5500004	0.0713669	0.00843621	2.68E-17	-0.0753893	0.205878	0.7142275	0.9827195
ENSG00000138029	HADHB	2	26489687	G	A	0.289264	0.0136458	0.0159345	0.2999998	0.174067	0.00910838	2.06E-81	0.0783941	0.0916344	0.392269	0.4054832
ENSG00000138030	KHK	2	27316627	C	T	0.383698	-0.00397434	0.0146443	0.6100002	0.462307	0.0084285	0	-0.00859675	0.0316769	0.7860924	0.9879286
ENSG00000138031	ADCY3	2	25092373	C	G	0.295229	0.0234264	0.0155923	0.17	0.120315	0.00941521	2.15E-37	0.194709	0.130488	0.1356582	0.1803994
ENSG00000138032	PPM1B	2	44433315	A	C	0.105368	0.0356144	0.0233733	0.1900002	-0.134126	0.0128918	2.38E-25	-0.265529	0.176123	0.1316471	0.9373512
ENSG00000138035	PNPT1	2	55891222	G	T	0.481113	0.0257728	0.0141928	0.05699936	0.153858	0.00790334	2.08E-84	0.167511	0.0926468	0.07059752	0.4539788
ENSG00000138036	DYNC2LI1	2	44019163	T	C	0.0248509	0.079883	0.0541009	0.1900002	-0.534407	0.0284917	1.71E-78	-0.14948	0.101549	0.1410204	0.974525
ENSG00000138041	NA	2	55810221	A	G	0.197813	-0.00416414	0.0176161	0.9299999	0.216514	0.0148056	1.98E-48	-0.0192326	0.0813729	0.8131597	0.05609797
ENSG00000138050	THUMPD2	2	39984803	A	G	0.462227	0.0106054	0.0143004	0.5500004	0.169173	0.00792073	3.27E-101	0.0626898	0.0845824	0.458591	0.9276131
ENSG00000138061	CYP1B1	2	38315580	T	C	0.130219	-0.0225501	0.0214174	0.2599998	0.423998	0.0109547	0	-0.0531845	0.0505317	0.2925713	0.517834
ENSG00000138069	RAB1A	2	65327537	C	A	0.187873	-0.0304555	0.0186479	0.07299952	-0.0581508	0.0100063	6.20E-09	0.523733	0.333104	0.1158861	0.6139035
ENSG00000138071	ACTR2	2	65476637	C	T	0.271372	-0.0156376	0.015772	0.4500005	-0.146189	0.00871296	3.51E-63	0.106968	0.108076	0.3222954	0.03090181
ENSG00000138073	PREB	2	27355583	C	G	0.166998	0.0092492	0.0199704	0.81	-0.183108	0.0119353	4.02E-53	-0.0505121	0.109113	0.6434113	0.6287603
ENSG00000138074	SLC5A6	2	27429140	C	T	0.145129	-0.0139235	0.0202989	0.5199996	-0.577621	0.0109361	0	0.0241049	0.0351452	0.4927976	0.2007464
ENSG00000138078	PREPL	2	44566210	C	A	0.054672	0.0145302	0.0306599	0.4899999	0.292418	0.0166873	9.50E-69	0.0496899	0.104888	0.6356837	0.2256708
ENSG00000138079	SLC3A1	2	44525616	T	G	0.17992	0.0224508	0.0191074	0.2	0.0727933	0.0106854	9.60E-12	0.308418	0.266364	0.2469117	0.5649659
ENSG00000138085	ATRAID	2	27437470	C	T	0.286282	-0.015503	0.0157311	0.2999998	0.359192	0.0096544	5.67E-303	-0.0431608	0.0438112	0.3245482	0.2613822
ENSG00000138092	CENPO	2	25030625	C	T	0.10338	0.0201109	0.0239483	0.4299995	-0.161457	0.0130071	2.22E-35	-0.124559	0.148665	0.4021161	0.2749968
ENSG00000138095	LRPPRC	2	44168395	C	T	0.411531	-0.0217637	0.014556	0.06800017	0.311379	0.00788523	0	-0.0698946	0.0467804	0.1351495	0.8605364
ENSG00000138101	DTNB	2	25748285	C	T	0.528827	-0.00749961	0.014257	0.4799997	0.237651	0.0130025	1.25E-74	-0.0315572	0.0600161	0.5990186	0.2586727
ENSG00000138107	ACTR1A	10	104250734	G	A	0.300199	-0.00373177	0.0148189	0.7300002	-0.15239	0.00832915	8.91E-75	0.0244883	0.0972524	0.8011947	0.9263877
ENSG00000138111	MFSD13A	10	104228975	T	C	0.126243	0.0342662	0.0233804	0.17	0.478495	0.0115295	0	0.0716125	0.0488929	0.1430077	0.382506
ENSG00000138115	CYP2C8	10	96812892	T	C	0.154076	0.0124639	0.0194614	0.4799997	-0.119751	0.0108388	2.23E-28	-0.104082	0.162788	0.522583	0.06139553
ENSG00000138119	MYOF	10	95154130	T	C	0.0576541	-0.0382201	0.0286721	0.1800002	-0.630633	0.018426	1.02E-256	0.060606	0.0455001	0.1828613	0.2291853
ENSG00000138131	LOXL4	10	100017727	T	C	0.450298	0.0027902	0.0143017	0.8600001	0.0556637	0.00808666	5.84E-12	0.050126	0.257033	0.8453793	0.1149836
ENSG00000138134	STAMBPL1	10	90687200	C	T	0.270378	0.00904928	0.0161165	0.6999999	0.209688	0.00881191	3.67E-125	0.0431559	0.0768807	0.5745689	0.49698
ENSG00000138138	ATAD1	10	89556184	A	G	0.292247	0.0276193	0.0155465	0.09699961	-0.110103	0.00858513	1.19E-37	-0.250851	0.142549	0.07844908	0.1360277
ENSG00000138160	KIF11	10	94384096	T	C	0.422465	0.00538296	0.0144763	0.7400005	0.0766605	0.00817694	6.90E-21	0.0702182	0.188985	0.7102248	0.8929916
ENSG00000138161	CUZD1	10	124615405	A	G	0.214712	-0.00620678	0.0178087	0.7600007	-0.095963	0.00982221	1.51E-22	0.0646789	0.185697	0.7276122	0.4418527
ENSG00000138166	DUSP5	10	112264449	C	T	0.300199	-0.00425706	0.0149521	0.8700001	-0.147551	0.00851272	2.65E-67	0.0288514	0.101349	0.7758933	0.436107
ENSG00000138172	CALHM2	10	105209601	A	G	0.0377734	0.0677442	0.0367671	0.04399973	-0.853314	0.0215664	0	-0.0793895	0.0431341	0.0656908	0.5754337
ENSG00000138175	ARL3	10	104455239	A	G	0.140159	0.00576373	0.0200086	0.7600007	0.262718	0.0165625	1.16E-56	0.0219389	0.0761727	0.7733351	0.1625749
ENSG00000138180	CEP55	10	95272619	A	G	0.33499	-0.0255108	0.0149639	0.07399971	-0.0489586	0.00832517	4.08E-09	0.521068	0.318228	0.1015455	0.6199077
ENSG00000138182	KIF20B	10	91498033	G	A	0.232604	0.0187564	0.0167345	0.3400001	0.230567	0.00902226	4.78E-144	0.0813489	0.0726494	0.2628221	0.5628482
ENSG00000138185	ENTPD1	10	97554279	T	G	0.0526839	0.00351567	0.033598	0.98	1.02457	0.0170069	0	0.00343138	0.0327925	0.9166622	0.9093554
ENSG00000138190	EXOC6	10	94705092	T	C	0.315109	-0.0151611	0.0151573	0.5999997	0.0828494	0.00869208	1.55E-21	-0.182996	0.183955	0.3198393	0.5990868
ENSG00000138231	DBR1	3	137886822	T	A	0.318091	0.0082435	0.015545	0.59	-0.121278	0.00904127	5.02E-41	-0.067972	0.128277	0.5961915	0.3346775
ENSG00000138246	DNAJC13	3	132197123	G	A	0.0516899	-0.0210692	0.0304433	0.3800004	0.51751	0.0190399	1.11E-162	-0.0407126	0.0588455	0.4890279	0.1024341
ENSG00000138286	FAM149B1	10	74966093	C	T	0.055666	0.0403855	0.0296829	0.2200002	0.414177	0.0178342	2.62E-119	0.0975078	0.07179	0.1743889	0.9710338
ENSG00000138293	NA	10	51577921	C	G	0.530815	0.00329794	0.0142379	0.9699999	0.368838	0.0077041	0	0.00894144	0.0386025	0.8168267	0.3082393
ENSG00000138297	NA	10	51607722	A	G	0.157058	-0.0308798	0.0204511	0.14	-0.0943559	0.0113365	8.56E-17	0.327269	0.220282	0.1373627	0.1544714
ENSG00000138303	ASCC1	10	73916585	C	T	0.358847	0.0142703	0.0145042	0.2700001	-0.332979	0.00794458	0	-0.0428564	0.0435708	0.3253109	0.6981824
ENSG00000138311	ZNF365	10	64282861	A	G	0.366799	-0.0216327	0.0148369	0.08799946	0.327802	0.00813567	0	-0.0659933	0.0452915	0.1450943	0.4250253
ENSG00000138326	RPS24	10	79805044	T	C	0.0447316	-0.040884	0.0445703	0.4299995	0.19203	0.0220198	2.76E-18	-0.212904	0.233381	0.361632	0.6249514

ENSG00000138346	DNA2	10	70202850	A	G	0.106362	-0.0354726	0.0256768	0.1	-0.328867	0.0138948	7.63E-124	0.107863	0.0782095	0.1678465	0.7244295
ENSG00000138363	ATIC	2	216195513	G	C	0.275348	-0.0156003	0.0155564	0.3400001	0.28567	0.0125789	3.55E-114	-0.0546094	0.0545088	0.3164181	0.8562334
ENSG00000138375	SMARCAL1	2	217312456	A	G	0.516899	-0.0183806	0.0141951	0.17	0.156658	0.00799837	2.03E-85	-0.11733	0.09081	0.1963451	0.3297667
ENSG00000138376	BARD1	2	215632399	T	G	0.261431	0.00268422	0.0155595	0.8200001	-0.587642	0.00824863	0	-0.00456778	0.0264779	0.8630344	0.7267675
ENSG00000138378	STAT4	2	191955312	C	T	0.412525	-0.0163272	0.0146047	0.1499999	-0.0553987	0.00892894	5.49E-10	0.294722	0.267874	0.2712346	0.22212
ENSG00000138380	CARF	2	203813998	A	G	0.0497018	-0.0276507	0.0302147	0.2	0.157697	0.017958	1.61E-18	-0.175341	0.192638	0.3627111	0.1243208
ENSG00000138381	ASNSD1	2	190530851	A	G	0.269384	-0.00267729	0.0162405	0.6600001	-0.823626	0.00755519	0	0.00325061	0.0197183	0.8690601	0.3270788
ENSG00000138382	METTL5	2	170674016	A	G	0.0318091	-0.0351358	0.0364904	0.5	-0.671006	0.0228528	1.67E-189	0.0523628	0.0544108	0.3358685	0.7480467
ENSG00000138385	SSB	2	170658508	A	G	0.318091	-0.0270558	0.015213	0.1	-0.109591	0.0140519	6.24E-15	0.24688	0.14238	0.08292637	0.144088
ENSG00000138386	NAB1	2	191534482	C	T	0.461233	0.0244982	0.0142763	0.04099964	-0.105753	0.0079944	6.02E-40	-0.231655	0.136128	0.08880291	0.1513672
ENSG00000138395	CDK15	2	202707728	T	C	0.0218688	0.0103139	0.0412336	0.95	0.274722	0.0238436	1.02E-30	0.0375431	0.150128	0.8025298	0.869987
ENSG00000138398	PPIG	2	170469383	G	A	0.252485	0.0215093	0.0160883	0.1900002	-0.191519	0.00888406	4.49E-103	-0.112309	0.084165	0.1820763	0.850464
ENSG00000138399	FASTKD1	2	170408322	C	G	0.130219	0.0126273	0.0203734	0.7099994	0.659317	0.0154364	0	0.0191521	0.030904	0.5354367	0.06629085
ENSG00000138411	HECW2	2	197261196	T	C	0.0785288	0.0366111	0.027518	0.2700001	-0.457551	0.0158402	1.82E-183	-0.0800154	0.0602057	0.1838371	0.4170156
ENSG00000138413	IDH1	2	209115874	G	T	0.100398	0.00887295	0.0221915	0.6800001	-0.314725	0.0187131	1.79E-63	-0.0281927	0.0705308	0.6893609	0.3020882
ENSG00000138430	OLA1	2	175025300	C	T	0.22664	0.0249805	0.0179116	0.089	0.0682612	0.00947809	5.93E-13	0.365954	0.267273	0.170931	0.2169852
ENSG00000138433	CIR1	2	175236596	C	A	0.322068	0.0130394	0.0153797	0.5	0.193704	0.00856187	2.52E-113	0.0673162	0.0794538	0.3968629	0.1512736
ENSG00000138434	ITPRID2	2	182776012	A	G	0.208748	0.00130821	0.0181082	0.8800001	0.111398	0.011236	3.60E-23	0.0117436	0.162559	0.9424092	0.2633888
ENSG00000138439	FAM117B	2	203567190	A	G	0.305169	-0.0288346	0.0155426	0.0259998	-0.86815	0.00794118	0	0.0332138	0.0179057	0.06360609	0.7951219
ENSG00000138448	ITGAV	2	187500210	G	A	0.335984	-0.00118861	0.015185	0.7099994	0.196577	0.00829959	5.12E-124	-0.00604652	0.0772473	0.9376094	0.2247004
ENSG00000138449	SLC40A1	2	190436894	T	C	0.317097	0.00632103	0.0152848	0.7400005	0.456797	0.00823916	0	0.0138377	0.0334617	0.6792121	0.8978847
ENSG00000138459	SLC35A5	3	112292490	C	T	0.0666004	-0.0177657	0.0273406	0.56	-0.639853	0.0141793	0	0.0277653	0.0427339	0.5158702	0.4829703
ENSG00000138463	SLC49A4	3	122556814	G	A	0.0705765	0.0204635	0.0271996	0.4299995	-0.974811	0.0151598	0	-0.0209923	0.0279044	0.4518742	0.663302
ENSG00000138468	SENP7	3	101137567	A	C	0.384692	-0.0185617	0.0146577	0.25	0.512879	0.00765645	0	-0.0361912	0.0285844	0.2054707	0.2642067
ENSG00000138495	COX17	3	119384830	T	C	0.191849	0.0205937	0.0169367	0.2099999	0.173458	0.0205983	3.73E-17	0.118724	0.098654	0.2288057	0.3403325
ENSG00000138496	PARP9	3	122265097	G	C	0.247515	0.0418812	0.0166391	0.004	0.085948	0.009517	1.70E-19	0.487285	0.200974	0.01532424	0.9326311
ENSG00000138592	USP8	15	50754928	A	G	0.182903	-0.00696646	0.0188582	0.58	-0.438022	0.0158998	4.56E-167	0.0159044	0.043057	0.7118448	0.8744679
ENSG00000138593	SECISBP2L	15	49309716	T	C	0.16004	-0.00256735	0.0188905	0.83	-0.112016	0.0105002	1.44E-26	0.0229194	0.168654	0.8919036	0.3999761
ENSG00000138594	TMOD3	15	52180658	G	A	0.0467197	0.000300158	0.0322132	0.99	-0.315851	0.0176924	2.77E-71	-0.000950314	0.101988	0.9925655	0.02818517
ENSG00000138600	SPPL2A	15	51028755	G	A	0.0228628	0.0126493	0.0433367	0.9199999	-0.68158	0.0259355	3.26E-152	-0.0185588	0.0635866	0.7703889	0.7328313
ENSG00000138604	GLCE	15	69508739	C	T	0.0198807	-0.0304025	0.0574308	0.64	0.948848	0.0294554	1.16E-227	-0.0320415	0.0605351	0.5965941	0.5255865
ENSG00000138606	SHF	15	45476392	T	C	0.114314	-0.00309944	0.024431	0.7499995	-0.124433	0.0133237	9.71E-21	0.0249085	0.196357	0.8990565	0.7945322
ENSG00000138613	APH1B	15	63584771	T	C	0.132207	-0.00693104	0.0207253	0.6200004	0.495891	0.0128297	0	-0.0139769	0.0417956	0.738069	0.1005087
ENSG00000138614	INTS14	15	65887359	T	C	0.0159046	-0.0394275	0.0520597	0.4400003	0.582225	0.0414826	9.47E-45	-0.0677187	0.0895452	0.4494984	0.2606857
ENSG00000138615	CILP	15	65496081	G	A	0.27833	0.0323048	0.0156177	0.0329997	0.113911	0.00867744	2.30E-39	0.283598	0.138797	0.04102639	0.1101839
ENSG00000138617	PARP16	15	65559877	C	T	0.44334	-0.0211957	0.0142574	0.14	-0.379857	0.00768549	0	0.0557992	0.0375506	0.1372861	0.1417798
ENSG00000138621	PPCDC	15	75362849	T	G	0.416501	0.0274561	0.014241	0.0259998	-0.0721396	0.00806029	3.55E-19	-0.380597	0.201937	0.05946652	0.7520741
ENSG00000138623	SEMA7A	15	74714219	G	C	0.455268	-0.00174404	0.0142039	0.83	0.0570113	0.00797495	8.75E-13	-0.0305911	0.249178	0.9022909	0.6154159
ENSG00000138629	UBL7	15	74745920	C	T	0.0318091	-0.0162896	0.0377741	0.5	0.271569	0.0233954	3.76E-31	-0.0599832	0.139192	0.666512	0.1620822
ENSG00000138639	ARHGAP24	4	86660045	A	C	0.295229	0.0187071	0.0155701	0.17	-0.432545	0.00835729	0	-0.0432489	0.0360062	0.2296921	0.8101431
ENSG00000138640	FAM13A	4	89839827	A	C	0.234592	-0.0422289	0.0173473	0.02199986	0.328345	0.00924755	3.94E-276	-0.128611	0.0529565	0.01515641	0.0140699
ENSG00000138641	HERC3	4	89535946	T	C	0.183897	-0.0045493	0.0180055	0.95	0.133931	0.0100493	1.60E-40	-0.0339674	0.134463	0.8005647	0.9993717
ENSG00000138642	HERC6	4	89332077	C	T	0.0705765	-0.0128435	0.0338014	0.5700002	0.577453	0.0192027	1.15E-198	-0.0222416	0.05854	0.7039906	0.03149904
ENSG00000138658	ZGRF1	4	113509321	T	C	0.436382	0.000749738	0.0142807	0.7800007	0.340265	0.00851989	0	0.0022034	0.0419694	0.9581303	0.9928804
ENSG00000138660	AP1AR	4	113172048	C	A	0.432406	-0.0240632	0.0144299	0.16	-0.0514464	0.00848474	1.33E-09	0.467733	0.290899	0.1078589	0.2503445
ENSG00000138669	PRKG2	4	82073027	C	A	0.186879	0.00842137	0.0184326	0.6700003	-0.288575	0.0101604	1.92E-177	-0.0291826	0.0638827	0.6478043	0.01886735
ENSG00000138670	RASGEF1B	4	82370314	A	G	0.260437	0.00374049	0.0164586	0.9400001	-0.05865	0.00929006	2.73E-10	-0.0637765	0.280806	0.8203308	0.3571373
ENSG00000138678	GPAT3	4	84492047	T	C	0.121272	-0.000550075	0.021035	0.7899998	-0.370803	0.0116773	2.79E-221	0.00148347	0.0567282	0.9791373	0.7342491
ENSG00000138685	FGF2	4	123783627	T	C	0.307157	0.0213582	0.0153568	0.2700001	-0.136001	0.00961382	1.97E-45	-0.157045	0.113461	0.1663204	0.2796429
ENSG00000138686	BBS7	4	122768623	T	C	0.290258	0.0135371	0.0163203	0.4400003	-0.10584	0.00971276	1.19E-27	-0.127902	0.154644	0.4081966	0.2399177

ENSG00000138688	BLTP1	4	123178700	G	A	0.239563	-0.00787037	0.0172113	0.33	-0.120168	0.00927253	2.07E-38	0.0654946	0.143316	0.6476748	0.03337296
ENSG00000138698	RAP1GDS1	4	99273773	A	G	0.109344	-0.0349478	0.0244658	0.1	-0.613966	0.0142057	0	0.0569214	0.0398706	0.1533911	0.9986521
ENSG00000138709	LARP1B	4	129063254	G	C	0.32505	-0.00685739	0.0148012	0.9	-0.159744	0.0088431	6.10E-73	0.0429275	0.0926865	0.6432588	0.7484546
ENSG00000138722	MMRN1	4	90838231	G	A	0.517893	0.0169443	0.0142115	0.1	0.13758	0.00791337	1.06E-67	0.123159	0.103539	0.2342425	0.05364572
ENSG00000138735	PDE5A	4	120482848	G	A	0.532803	-0.0113278	0.0142143	0.4	0.134478	0.00866046	2.25E-54	-0.0842354	0.105839	0.4261004	0.8551196
ENSG00000138738	PRDM5	4	121725049	T	G	0.317097	-0.0135788	0.01544	0.35	-0.341193	0.00834055	0	0.039798	0.0452634	0.3792642	0.3695968
ENSG00000138741	TRPC3	4	122836545	A	G	0.235586	-0.00740588	0.0167326	0.81	0.308303	0.00925793	3.67E-243	-0.0240214	0.054278	0.658082	0.09577991
ENSG00000138744	NAAA	4	76847006	A	T	0.436382	-0.0185253	0.0143833	0.25	0.782405	0.00681102	0	-0.0236774	0.0183846	0.1977836	0.1245627
ENSG00000138750	NUP54	4	77052740	C	T	0.309145	-0.00285374	0.0150075	0.7099994	0.217087	0.00830262	1.07E-150	-0.0131456	0.0691331	0.8491922	0.6749392
ENSG00000138755	CXCL9	4	76925534	A	T	0.261431	0.0394857	0.0157758	0.01499996	0.123503	0.00875755	3.67E-45	0.319716	0.129733	0.01372371	0.4067302
ENSG00000138756	BMP2K	4	79767511	A	C	0.0447316	0.00233692	0.0345803	0.9299999	-0.276633	0.0187797	4.11E-49	-0.00844774	0.125006	0.9461209	0.8073379
ENSG00000138757	G3BP2	4	76608837	C	T	0.375746	0.0107125	0.0146404	0.4100001	-0.456679	0.00784979	0	-0.0234574	0.0320609	0.464382	0.543028
ENSG00000138758	SEPTIN11	4	77916196	T	G	0.38668	-0.0119922	0.0143842	0.3700002	-0.246678	0.00790592	1.01E-213	0.0486148	0.0583324	0.4046141	0.1048714
ENSG00000138760	SCARB2	4	77107468	A	G	0.191849	-0.0180498	0.0187567	0.28	0.286527	0.00999486	9.70E-181	-0.062995	0.065499	0.3361649	0.5949727
ENSG00000138764	CCNG2	4	78216423	C	T	0.167992	0.00283491	0.0183251	0.8600001	-0.11174	0.0102268	8.64E-28	-0.0253706	0.164014	0.8770693	0.200053
ENSG00000138767	CNOT6L	4	78687655	G	A	0.0755467	-0.032501	0.0273115	0.2399999	-0.284773	0.0179751	1.58E-56	0.11413	0.0961764	0.2353584	0.2656225
ENSG00000138768	USO1	4	76692572	A	G	0.156064	0.0303577	0.0212219	0.08799946	-0.399783	0.0181926	5.00E-107	-0.0759355	0.0531959	0.153445	0.4670207
ENSG00000138772	ANXA3	4	79502135	A	G	0.0407555	-0.0261015	0.0353363	0.5	0.667401	0.0187911	2.71E-276	-0.0391092	0.0529576	0.4602107	0.215388
ENSG00000138777	PPA2	4	106342736	C	A	0.194831	-0.00212294	0.0190227	0.95	0.190426	0.0108851	1.59E-68	-0.0111484	0.0998977	0.9111421	0.8086693
ENSG00000138778	CENPE	4	104073264	C	G	0.225646	0.015703	0.0177492	0.4	0.0765059	0.00987402	9.32E-15	0.205252	0.233505	0.3793989	0.8837197
ENSG00000138780	GSTCD	4	106699410	C	A	0.235586	0.00867536	0.0169106	0.7700005	0.197473	0.0100178	1.69E-86	0.0439319	0.085664	0.6080636	0.18762
ENSG00000138785	INTS12	4	106710463	C	T	0.0636183	0.0250289	0.0287773	0.3400001	-0.260548	0.0181582	1.08E-46	-0.0960626	0.110652	0.3853116	0.07147487
ENSG00000138794	CASP6	4	110617262	A	G	0.362823	0.0330967	0.014741	0.025	0.0944257	0.0082631	3.05E-30	0.350505	0.159097	0.02758815	0.6427473
ENSG00000138796	HADH	4	108933600	T	G	0.241551	0.0109578	0.0168584	0.4100001	0.188868	0.00975146	1.43E-83	0.0580183	0.0893104	0.5159346	0.8614687
ENSG00000138798	EGF	4	110883731	A	G	0.392644	0.00563727	0.0144727	0.6200004	0.163421	0.00804135	8.12E-92	0.0344954	0.088577	0.6969514	0.2033448
ENSG00000138801	PAPSS1	4	108576520	C	A	0.317097	-0.0168134	0.0153126	0.3400001	0.939181	0.00657569	0	-0.0179022	0.0163047	0.2722139	0.8419071
ENSG00000138802	SEC24B	4	110408490	T	A	0.136183	0.045178	0.0187595	0.007699987	0.0740537	0.0110992	2.52E-11	0.610071	0.26932	0.02349884	0.875769
ENSG00000138814	PPP3CA	4	102107000	C	T	0.289264	0.00255662	0.0155916	0.6200004	-0.165523	0.00874643	7.15E-80	-0.0154457	0.0941997	0.8697563	0.03454969
ENSG00000138821	SLC39A8	4	103262306	G	A	0.16998	0.0155302	0.0181515	0.2599998	0.201807	0.0104279	1.94E-83	0.0769558	0.0900329	0.392688	0.9909472
ENSG00000138829	FBN2	5	127794239	A	G	0.0815109	-0.024596	0.0254995	0.3100002	1.26177	0.0127429	0	-0.0194933	0.0202103	0.3347842	0.3056487
ENSG00000138834	MAPK8IP3	16	1788251	G	A	0.188867	0.0219035	0.0180355	0.3700002	0.0985714	0.0100515	1.05E-22	0.22221	0.184367	0.2281034	0.8672529
ENSG00000138835	RGS3	9	116283514	G	A	0.147117	0.00926491	0.0197836	0.7700005	-0.273947	0.0106439	4.44E-146	-0.03382	0.0722287	0.6396164	0.4042861
ENSG00000138867	GUCD1	22	24944154	A	G	0.153082	0.0246224	0.0195588	0.25	0.175619	0.0114456	3.90E-53	0.140203	0.111745	0.2095967	0.1340739
ENSG00000138942	RNF185	22	31579583	G	C	0.291252	-0.0234473	0.0154055	0.1299999	0.568777	0.0133793	0	-0.0412241	0.0271027	0.1282513	0.8438271
ENSG00000138964	PARVG	22	44592124	T	C	0.484095	-0.0225782	0.0142071	0.14	-0.217838	0.00845756	2.72E-146	0.103647	0.0653426	0.1126933	0.1303061
ENSG00000139044	B4GALNT3	12	621102	A	G	0.294235	-0.00393411	0.0161838	0.8	0.535156	0.00908344	0	-0.00735134	0.0302416	0.8079378	0.8854737
ENSG00000139053	PDE6H	12	15130377	G	A	0.135189	-0.0220296	0.0205807	0.32	0.289109	0.0113058	3.14E-144	-0.0761984	0.0712491	0.2848605	0.2008397
ENSG00000139055	ERP27	12	15079492	T	G	0.39165	-0.00299391	0.0145331	0.7899998	-0.578863	0.00752282	0	0.00517206	0.0251064	0.8367866	0.4213392
ENSG00000139083	ETV6	12	11923673	G	A	0.277336	0.0138473	0.0154239	0.4600002	0.145972	0.0128869	9.62E-30	0.0948624	0.105994	0.3708003	0.7462146
ENSG00000139112	GABARAPL1	12	10370392	G	A	0.10338	-0.00389956	0.0256436	0.9	-0.237381	0.0148343	1.23E-57	0.0164274	0.108032	0.8791391	0.4552536
ENSG00000139116	KIF21A	12	39762111	C	G	0.00397614	0.0542174	0.133025	0.84	-0.837005	0.135574	6.67E-10	-0.0647755	0.159276	0.6842373	0.2338293
ENSG00000139117	CPNE8	12	39170928	G	T	0.0129225	-0.113622	0.0758645	0.06900014	0.764395	0.0373229	3.21E-93	-0.148643	0.0995129	0.135252	0.4461991
ENSG00000139131	YARS2	12	32894630	T	G	0.16501	-0.00301319	0.0203376	0.8600001	0.116852	0.0174037	1.89E-11	-0.0257863	0.174088	0.8822461	0.9751886
ENSG00000139132	FGD4	12	32675723	T	C	0.280318	0.018234	0.0161069	0.2700001	-0.102911	0.00884127	2.59E-31	-0.177183	0.157252	0.2598503	0.4849166
ENSG00000139133	ALG10	12	34178922	A	T	0.38668	0.00014763	0.0145021	0.7700005	-0.269908	0.0185321	4.73E-48	-0.000546963	0.0537297	0.9918778	0.01183688
ENSG00000139146	SINHCAF	12	31456755	T	C	0.254473	0.0200644	0.0166328	0.1299999	-0.0868065	0.014172	9.06E-10	-0.231139	0.195288	0.2365793	0.6521663
ENSG00000139154	AEBP2	12	19715357	G	C	0.188867	-0.00995407	0.019999	0.4400003	0.194856	0.0179654	2.08E-27	-0.0510841	0.102743	0.6190444	0.6001848
ENSG00000139160	ETFBKMT	12	31813071	G	A	0.500994	0.00550359	0.0142209	0.6100002	0.0534666	0.00805705	3.22E-11	0.102935	0.266429	0.699237	0.2075745
ENSG00000139163	ETNK1	12	22808327	A	G	0.0775348	-0.00738313	0.0261316	0.7199992	-0.273811	0.0144626	6.17E-80	0.0269643	0.0954471	0.7775563	0.7478189
ENSG00000139168	ZCRB1	12	42712900	C	T	0.0626243	-0.00614399	0.0265368	0.91	-0.377998	0.0152074	2.22E-136	0.016254	0.0702065	0.8169131	0.1558261

ENSG00000139173	TMEM117	12	44506657	A	G	0.0318091	-0.0201309	0.0355762	0.6600001	-0.162588	0.019564	9.52E-17	0.123815	0.219318	0.5723827	0.0321034
ENSG00000139174	PRICKLE1	12	42918148	T	C	0.0775348	-0.00866888	0.0268474	0.81	-0.315735	0.014913	1.74E-99	0.0274562	0.0850414	0.7468035	0.2145102
ENSG00000139178	C1RL	12	7252026	G	C	0.0924453	-0.0199365	0.0234919	0.5300002	-0.546297	0.0130438	0	0.0364939	0.0430109	0.3961707	0.1507967
ENSG00000139180	NDUFA9	12	4756282	A	G	0.456262	-0.00613304	0.014337	0.4199997	0.154529	0.0118033	3.66E-39	-0.0396885	0.092828	0.6689796	0.2311046
ENSG00000139182	CLSTN3	12	7296917	A	G	0.109344	-0.0176722	0.023477	0.5	-0.341331	0.0126143	2.99E-161	0.0517743	0.0688073	0.451778	0.0992769
ENSG00000139187	KLRG1	12	9132998	C	T	0.319085	0.00273659	0.01491	0.8	-0.488833	0.00808881	0	-0.00559821	0.0305013	0.8543745	0.05534179
ENSG00000139190	VAMP1	12	6575778	T	G	0.309145	0.015059	0.0159354	0.6300007	0.697593	0.00786872	0	0.0215871	0.0228447	0.3446848	0.1146261
ENSG00000139192	TAPBPL	12	6568269	T	C	0.304175	-0.000966727	0.0155457	0.95	0.847209	0.0073307	0	-0.00114107	0.0183493	0.9504146	0.03809367
ENSG00000139193	CD27	12	6557458	G	T	0.45328	-0.0144995	0.0145925	0.14	0.100483	0.00800263	3.68E-36	-0.144299	0.145678	0.321915	0.3922102
ENSG00000139194	RBP5	12	7278909	T	C	0.329026	-0.0204281	0.0151487	0.08400014	0.226089	0.00831043	5.59E-163	-0.0903541	0.0670854	0.178028	0.2062876
ENSG00000139197	PEX5	12	7356225	C	G	0.487078	0.0201703	0.0141648	0.1299999	-0.573933	0.010827	0	-0.035144	0.0246891	0.1546028	0.2831695
ENSG00000139200	PIANP	12	6806462	T	C	0.0874751	0.003921	0.0240717	0.4299995	0.147884	0.0181707	4.00E-16	0.026514	0.162807	0.870632	0.5187841
ENSG00000139211	AMIGO2	12	47471612	C	T	0.33499	-0.0151158	0.015206	0.3400001	-0.164052	0.00846495	1.13E-83	0.0921401	0.0928118	0.3208255	0.566651
ENSG00000139218	SCAF11	12	46349408	T	C	0.203777	-0.0121579	0.0173662	0.4899999	-0.0623913	0.0113699	4.08E-08	0.194865	0.280599	0.4873933	0.4184375
ENSG00000139233	LLPH	12	66520695	T	G	0.0864811	-0.00925181	0.0299408	0.6499995	-0.196047	0.0178517	4.66E-28	0.0471918	0.152783	0.7574119	0.7246493
ENSG00000139266	MARCHF9	12	58151535	C	A	0.312127	-0.041715	0.0151803	0.01	0.0697043	0.0084486	1.58E-16	-0.598457	0.229544	0.009129732	0.9326154
ENSG00000139278	GLIPR1	12	75886046	A	G	0.415507	0.00209868	0.0142972	0.8800001	-0.481602	0.0112645	0	-0.0043577	0.0296869	0.8832988	0.9658449
ENSG00000139289	PHLDA1	12	76423469	G	A	0.132207	-0.00911151	0.0214338	0.6899999	0.0694685	0.0113559	9.51E-10	-0.13116	0.309284	0.6715094	0.92709
ENSG00000139291	TMEM19	12	72088851	C	T	0.0606362	-0.0195018	0.0294383	0.5300002	0.325765	0.0171059	7.37E-81	-0.0598646	0.0904213	0.5079308	0.5484733
ENSG00000139318	DUSP6	12	89744028	G	C	0.243539	-0.0138366	0.0170057	0.5999997	0.24068	0.00953904	1.83E-140	-0.0574897	0.0706937	0.4160907	0.8080194
ENSG00000139323	POC1B	12	89866648	A	G	0.222664	0.027643	0.0166961	0.0659994	-0.300431	0.0092997	5.87E-229	-0.0920113	0.0556468	0.09823199	0.5466104
ENSG00000139324	TMTC3	12	88564868	T	C	0.0129225	0.0483018	0.0589215	0.2200002	0.464622	0.0427546	1.65E-27	0.103959	0.127176	0.4136754	0.3139134
ENSG00000139343	SNRPF	12	96275156	T	C	0.0894632	0.0387675	0.025372	0.05600025	0.232277	0.0224927	5.33E-25	0.166902	0.110421	0.130659	0.6389511
ENSG00000139344	AMDHD1	12	96349720	A	G	0.399602	-0.00279914	0.0143676	0.7700005	-0.659717	0.00709426	0	0.00424294	0.0217785	0.8455318	0.1845529
ENSG00000139350	NEDD1	12	97324065	A	G	0.454274	0.0230803	0.0142044	0.064	-0.423705	0.00761702	0	-0.0544726	0.0335386	0.104338	0.5056904
ENSG00000139351	SYCP3	12	102127838	G	C	0.394632	-0.00922086	0.0144041	0.7199992	-0.165051	0.00809579	2.17E-92	0.0558669	0.0873139	0.5222767	0.09226275
ENSG00000139354	GAS2L3	12	100994762	G	C	0.39165	-0.00463809	0.0143935	0.83	0.0449044	0.00817812	4.00E-08	-0.103288	0.321088	0.7476939	0.9054084
ENSG00000139370	SLC15A4	12	129293133	C	T	0.382704	-0.00958756	0.0148731	0.4100001	-0.1935	0.0083035	4.10E-120	0.0495482	0.0768931	0.5193305	0.9690021
ENSG00000139372	TDG	12	104371117	A	G	0.38668	0.0026065	0.0145984	0.7700005	0.155468	0.0122173	4.28E-37	0.0167655	0.093909	0.8583072	0.4968425
ENSG00000139405	RITA1	12	113626752	A	G	0.0934394	-0.0202045	0.027772	0.5500004	-0.627658	0.0158023	0	0.0321903	0.0442545	0.4669863	0.3095903
ENSG00000139410	SDSL	12	113868061	A	G	0.0357853	-0.0411845	0.0378169	0.25	0.759265	0.0213722	1.96E-276	-0.0542426	0.0498307	0.2763573	0.0665605
ENSG00000139428	MMAB	12	110002467	T	C	0.435388	0.00571418	0.0143066	0.7400005	-0.187099	0.00805115	1.85E-119	-0.0305409	0.0764766	0.6896356	0.3574539
ENSG00000139433	GLTP	12	110303520	A	C	0.43837	-0.000187501	0.014266	0.89	0.287147	0.0117586	1.04E-131	-0.000652979	0.0496818	0.9895135	0.4402781
ENSG00000139436	GIT2	12	110400900	G	T	0.0735586	0.0288423	0.0276585	0.35	0.461046	0.0244795	3.98E-79	0.0625584	0.0600826	0.29778	0.301217
ENSG00000139437	TCHP	12	110379857	G	A	0.0745527	0.0263375	0.02738	0.4	0.198399	0.0148405	9.20E-41	0.13275	0.138361	0.3373349	0.4673767
ENSG00000139496	NUP58	13	25899800	C	T	0.430417	-2.00E-05	0.0146931	0.9599999	-0.0931126	0.00830058	3.34E-29	0.000215016	0.157799	0.9989128	0.1680241
ENSG00000139505	MTMR6	13	25832227	T	C	0.16998	-0.0261935	0.0192038	0.14	0.333254	0.0106207	4.08E-216	-0.0785993	0.0576796	0.1729809	0.8853089
ENSG00000139508	SLC46A3	13	29283654	G	T	0.45825	0.00201423	0.0142714	0.7199992	0.313751	0.0087125	5.51E-284	0.00641983	0.0454867	0.8877622	0.3663923
ENSG00000139514	SLC7A1	13	30126686	A	G	0.115308	-0.0331995	0.0242582	0.16	0.615069	0.0130296	0	-0.0539768	0.0394563	0.1713077	0.9972123
ENSG00000139517	LNX2	13	28157295	T	C	0.347913	0.0126792	0.0145517	0.2999998	-0.159576	0.00810854	3.20E-86	-0.0794555	0.0912791	0.3840446	0.3389446
ENSG00000139531	SUOX	12	56395694	C	G	0.390656	-0.0142476	0.014368	0.35	-0.336612	0.00796648	0	0.0423265	0.0426959	0.321516	0.4212434
ENSG00000139546	TARBP2	12	53897460	T	C	0.151093	0.0261092	0.0194744	0.2300001	-0.0805373	0.0112545	8.31E-13	-0.324188	0.246013	0.1875822	0.7022198
ENSG00000139567	ACVRL1	12	52308918	A	G	0.130219	-0.0253545	0.0195658	0.25	-0.424789	0.0121851	2.85E-266	0.0596873	0.0460918	0.1953327	0.8936399
ENSG00000139572	GPR84	12	54757250	A	G	0.444334	-0.00105437	0.0142457	0.7499995	0.221891	0.00786453	3.93E-175	-0.00475175	0.0642016	0.9410001	0.1091141
ENSG00000139579	NABP2	12	56619718	T	C	0.0188867	-0.00262569	0.0539484	0.8600001	-0.571554	0.0576757	3.77E-23	0.00459395	0.0943901	0.9611824	0.9393626
ENSG00000139597	N4BP2L1	13	32988588	A	G	0.204771	-0.0143462	0.0183622	0.4799997	0.118334	0.0114427	4.58E-25	-0.121235	0.155615	0.4359387	0.2907625
ENSG00000139610	CELA1	12	51731345	C	T	0.406561	0.0134239	0.0143538	0.3800004	0.547763	0.0111862	0	0.0245067	0.0262092	0.349765	0.8465342
ENSG00000139613	SMARCC2	12	56570059	C	T	0.11332	0.00781751	0.021932	0.7300002	0.233572	0.0128803	1.72E-73	0.0334694	0.0939165	0.7215603	0.8595935
ENSG00000139618	BRCA2	13	32931708	A	G	0.215706	0.0110292	0.0160255	0.33	-0.124205	0.00927694	7.05E-41	-0.0887982	0.129195	0.4918807	0.1186645
ENSG00000139620	KANSL2	12	49061602	A	T	0.26839	-0.0189078	0.0164467	0.16	0.352246	0.00964137	3.11E-292	-0.0536779	0.0467141	0.2505262	0.4248308

ENSG00000139624	CERS5	12	50542431	A	G	0.400596	0.0152302	0.0146675	0.3800004	0.656034	0.00830904	0	0.0232156	0.0223598	0.2991425	0.1581801
ENSG00000139625	MAP3K12	12	53884060	C	T	0.536779	0.0116437	0.0142914	0.3700002	-0.130102	0.00803308	5.40E-59	-0.0894966	0.109986	0.4158135	0.4526517
ENSG00000139626	ITGB7	12	53593096	C	T	0.0805169	0.0191948	0.0267268	0.6600001	0.695844	0.0131344	0	0.0275849	0.0384127	0.4726836	0.3137831
ENSG00000139629	GALNT6	12	51765441	A	T	0.416501	-0.0108426	0.0144081	0.6499995	0.375611	0.00789416	0	-0.0288666	0.0383639	0.4517859	0.2125744
ENSG00000139631	CSAD	12	53563291	C	A	0.194831	-0.0162196	0.0185783	0.25	-0.191024	0.0100951	7.46E-80	0.0849087	0.0973598	0.3831471	0.1228291
ENSG00000139636	LMBR1L	12	49497801	T	C	0.33002	-0.0336481	0.0149834	0.04	-0.0716318	0.00833568	8.44E-18	0.469737	0.216197	0.02980087	0.9634692
ENSG00000139641	ESYT1	12	56525244	T	C	0.126243	0.01388	0.0214677	0.5199996	0.145926	0.0130587	5.43E-29	0.0951168	0.14736	0.5186206	0.4294596
ENSG00000139644	TMBIM6	12	50130112	A	G	0.0646123	0.00599386	0.0276622	0.6499995	0.176008	0.0161452	1.13E-27	0.0340545	0.157195	0.8284907	0.7055125
ENSG00000139651	ZNF740	12	53578846	A	G	0.136183	-0.0500209	0.0209157	0.007699987	0.144404	0.0118385	3.19E-34	-0.346395	0.147599	0.0189321	0.6536139
ENSG00000139668	WDFY2	13	52247407	A	G	0.16004	-0.0116228	0.018697	0.6300007	0.488733	0.0112642	0	-0.0237815	0.03826	0.5342207	0.2195672
ENSG00000139675	HNRNPA1L2	13	53204762	C	T	0.0397614	-0.0268974	0.032927	0.4400003	0.319224	0.0425746	6.48E-14	-0.0842586	0.103757	0.4167488	0.8737911
ENSG00000139679	LPAR6	13	48991273	G	A	0.128231	0.0132938	0.0209142	0.56	-0.132005	0.012201	2.79E-27	-0.100707	0.158708	0.5257278	0.3764637
ENSG00000139684	ESD	13	47358379	C	T	0.465209	-0.00259833	0.0142439	0.9	-0.281914	0.00854885	1.75E-238	0.00921676	0.0505265	0.8552576	0.03050908
ENSG00000139687	RB1	13	48967004	G	A	0.289264	0.0121014	0.0157285	0.5	-0.16208	0.00868075	8.49E-78	-0.0746631	0.0971239	0.4420472	0.05778563
ENSG00000139697	SBNO1	12	123811523	A	T	0.223658	-0.00290695	0.0177179	0.7499995	-0.11544	0.010754	7.00E-27	0.0251814	0.153499	0.8696923	0.6644179
ENSG00000139714	MORN3	12	122099780	T	C	0.394632	0.0131795	0.0143908	0.4799997	0.566985	0.0109468	0	0.0232449	0.0253853	0.3598321	0.4715043
ENSG00000139719	VPS33A	12	122732589	A	G	0.0586481	-0.0705027	0.0295941	0.009499921	0.167616	0.0164594	2.35E-24	-0.420619	0.181325	0.02035745	0.7999641
ENSG00000139722	VPS37B	12	123365436	C	T	0.135189	-0.00347478	0.0231245	0.58	0.372525	0.0113075	4.99E-238	-0.00932764	0.0620756	0.8805576	0.9478922
ENSG00000139725	RHOF	12	122223962	G	A	0.084493	-0.0122744	0.0258765	0.56	0.231365	0.0134266	1.53E-66	-0.0530521	0.111885	0.6353817	0.9777978
ENSG00000139726	DENR	12	123246466	T	C	0.497018	0.0395121	0.0143085	0.002100003	-0.183094	0.00953706	3.83E-82	-0.215802	0.0789525	0.006270095	0.284664
ENSG00000139737	SLAIN1	13	78305200	C	T	0.0924453	0.00485326	0.0285742	0.9599999	0.108877	0.0154248	1.68E-12	0.0445755	0.26252	0.8651686	0.8399939
ENSG00000139746	RBM26	13	79933287	T	C	0.408549	-0.000425525	0.0144052	0.81	-0.201613	0.0120034	2.59E-63	0.0021106	0.0714497	0.9764342	0.562824
ENSG00000139793	MBNL2	13	97960031	T	C	0.0944334	0.0100305	0.0250703	0.8800001	-0.195478	0.0129195	1.02E-51	-0.0513127	0.128296	0.6891896	0.8554789
ENSG00000139826	ABHD13	13	108878665	T	C	0.107356	-0.0166234	0.0236417	0.6999999	-0.111816	0.0136218	2.24E-16	0.148667	0.212208	0.4835693	0.4476283
ENSG00000139832	RAB20	13	111194748	A	G	0.466203	-0.0134485	0.0142065	0.32	0.335216	0.00840999	0	-0.040119	0.0423921	0.3439562	0.838211
ENSG00000139835	GRTP1	13	113998475	A	G	0.472167	-0.00876117	0.0142275	0.6200004	-0.148767	0.0086187	9.25E-67	0.0588917	0.0956967	0.5382908	0.2962881
ENSG00000139842	CUL4A	13	113890975	A	G	0.0387674	0.045439	0.038976	0.2399999	0.170898	0.0226367	4.37E-14	0.265884	0.230769	0.2492538	0.09567986
ENSG00000139880	CDH24	14	23521509	A	G	0.0954274	0.0163581	0.024497	0.3800004	0.0930991	0.0143155	7.85E-11	0.175706	0.264512	0.5065195	0.7914619
ENSG00000139890	REM2	14	23354634	G	C	0.151093	0.00323636	0.0193083	0.99	0.273662	0.0109456	5.81E-138	0.0118261	0.0705568	0.8668892	0.8601921
ENSG00000139899	CBLN3	14	24897949	G	A	0.239563	-0.00469394	0.0159951	0.7700005	-0.763549	0.00794243	0	0.00614753	0.0209485	0.7691708	0.3819015
ENSG00000139908	TSSK4	14	24676235	G	C	0.105368	-0.0244134	0.023323	0.2	0.0778069	0.0128449	1.38E-09	-0.313769	0.304197	0.3023229	0.9722977
ENSG00000139921	TMX1	14	51714819	G	A	0.111332	0.0111088	0.0246004	0.6499995	-0.325403	0.012419	2.52E-151	-0.0341386	0.0756111	0.6516272	0.01651312
ENSG00000139926	FRMD6	14	52076631	C	T	0.323062	-0.00742709	0.0147408	0.4700002	0.0953374	0.00839918	7.35E-30	-0.0779032	0.154769	0.6147181	0.3280501
ENSG00000139946	PELI2	14	56676388	A	C	0.326044	-0.00851017	0.0146726	0.6700003	-0.186461	0.00839227	2.29E-109	0.0456405	0.0787167	0.562045	0.02916392
ENSG00000139970	RTN1	14	60200189	G	C	0.0526839	-0.0424748	0.0348744	0.2099999	-0.825006	0.0192051	0	0.0514842	0.0422887	0.2234341	0.08034669
ENSG00000139974	SLC38A6	14	61499141	A	G	0.0109344	0.0107964	0.0602047	0.6899999	0.419992	0.0520397	7.00E-16	0.0257062	0.143383	0.8577148	0.3692852
ENSG00000139977	NAA30	14	57869948	T	A	0.203777	-0.00321207	0.0185376	0.9199999	0.190637	0.0119445	2.42E-57	-0.0168492	0.0972461	0.8624447	0.3205464
ENSG00000139990	DCAF5	14	69568732	T	G	0.00596421	0.0540738	0.0864655	0.3599996	-0.37228	0.0495488	5.76E-14	-0.14525	0.233062	0.5331367	0.8236076
ENSG00000139998	RAB15	14	65426013	C	T	0.0109344	-0.0763031	0.0653512	0.1900002	1.22956	0.0395423	2.82E-212	-0.0620571	0.0531874	0.2433061	0.6030581
ENSG00000140006	WDR89	14	64086168	A	G	0.0318091	-0.0958336	0.0427818	0.02199986	-0.239739	0.0227034	4.59E-26	0.399742	0.182423	0.02843062	0.4331363
ENSG00000140009	ESR2	14	64677890	A	G	0.39662	0.01807	0.0144043	0.17	0.181461	0.00818595	7.09E-109	0.0995805	0.0795065	0.2103941	0.9939783
ENSG00000140022	STON2	14	81814904	T	A	0.259443	0.038776	0.0155961	0.005800027	0.192687	0.013096	5.29E-49	0.201239	0.0820876	0.01422588	0.2665022
ENSG00000140025	EFCAB11	14	90341067	C	T	0.104374	0.048595	0.0225957	0.03500016	0.255456	0.0137569	5.70E-77	0.190229	0.0890438	0.03265089	0.5180104
ENSG00000140030	GPR65	14	88474943	T	C	0.321074	-0.0153333	0.0151379	0.28	-0.273163	0.00841579	4.12E-231	0.0561324	0.0554441	0.3113396	0.8452064
ENSG00000140043	PTGR2	14	74336038	A	G	0.0984095	-0.0549119	0.0250139	0.02	0.428878	0.0139441	9.86E-208	-0.128036	0.0584724	0.02854708	0.1818054
ENSG00000140044	JDP2	14	75917616	T	C	0.412525	-0.00387278	0.0144749	0.6999999	-0.068616	0.00814433	3.61E-17	0.0564414	0.211062	0.7891484	0.4102806
ENSG00000140090	SLC24A4	14	92875760	A	C	0.459245	-0.00279622	0.0143098	0.83	0.467183	0.00754296	0	-0.00598528	0.0306301	0.8450759	0.4999131
ENSG00000140092	FBLN5	14	92375043	A	G	0.450298	-0.000674002	0.0142442	0.95	-0.804291	0.00658145	0	0.000838007	0.0177102	0.9622601	0.5358269
ENSG00000140104	CLBA1	14	105464465	T	G	0.21173	0.0140184	0.0186658	0.4400003	0.218578	0.00991643	1.14E-107	0.0641345	0.0854461	0.4529024	0.6508246
ENSG00000140105	WARS1	14	100821633	G	C	0.192843	-0.00231503	0.0168846	0.99	0.795266	0.00946679	0	-0.00291101	0.0212314	0.8909448	0.8867011

ENSG00000140153	WDR20	14	102648512	G	A	0.177932	0.000692661	0.0186521	0.6999999	0.0591699	0.0105948	2.34E-08	0.0117063	0.315236	0.9703774	0.588783
ENSG00000140157	NIPA2	15	23019555	G	A	0.444334	-0.0150526	0.0143315	0.29	0.234956	0.00798917	4.18E-190	-0.0640657	0.0610355	0.2938806	0.7580613
ENSG00000140181	NA	15	23330254	G	A	0.2167	0.0178282	0.0247181	0.5	-0.860361	0.0681842	1.68E-36	-0.0207218	0.0287768	0.4714723	0.5350291
ENSG00000140199	SLC12A6	15	34577860	C	T	0.260437	0.0156476	0.0160532	0.3800004	-0.285376	0.00867018	1.36E-237	-0.0548316	0.0562776	0.3299042	0.3770892
ENSG00000140254	DUOXA1	15	45415852	T	C	0.204771	-0.0198754	0.0188127	0.2099999	0.0626296	0.0105412	2.83E-09	-0.317348	0.305092	0.2982602	0.3728166
ENSG00000140259	MFAP1	15	44106845	T	C	0.0904573	0.0284683	0.0244989	0.3100002	0.162585	0.0138696	9.79E-32	0.175098	0.151422	0.2475356	0.334421
ENSG00000140262	TCF12	15	57401150	C	T	0.193837	-0.0054862	0.0187056	0.5300002	-0.168517	0.0102401	7.52E-61	0.0325557	0.111019	0.7693347	0.4795244
ENSG00000140263	SORD	15	45342342	C	T	0.0874751	0.0213516	0.0239551	0.17	-0.240929	0.0155102	2.06E-54	-0.0886219	0.0995915	0.3735444	0.1717425
ENSG00000140264	SERF2	15	44082036	G	C	0.184891	-0.0093654	0.0185289	0.4100001	-0.265547	0.0101249	1.30E-151	0.0352684	0.0697894	0.6133099	0.6500431
ENSG00000140265	ZSCAN29	15	43656796	G	C	0.195825	-0.00276639	0.0179759	0.6700003	-0.5589	0.00960986	0	0.00494971	0.0321631	0.8776934	0.6551563
ENSG00000140280	LYSMD2	15	52029495	T	C	0.00497018	-0.123084	0.101515	0.2200002	-0.777206	0.0724533	7.60E-27	0.158367	0.131447	0.2282808	0.3020865
ENSG00000140284	SLC27A2	15	50501492	G	A	0.402584	0.0241193	0.014576	0.08700015	0.11921	0.0081399	1.45E-48	0.202325	0.123049	0.1001215	0.7512854
ENSG00000140299	BNIP2	15	59966539	C	T	0.382704	-0.015282	0.0146246	0.2700001	-0.176952	0.00857467	1.29E-94	0.0863624	0.0827531	0.2966637	0.2535254
ENSG00000140307	GTF2A2	15	59940000	A	G	0.355865	-0.0138707	0.0149754	0.29	0.343292	0.0120278	3.57E-179	-0.040405	0.0436459	0.3545785	0.2136689
ENSG00000140319	SRP14	15	40329664	G	A	0.333002	-0.0177729	0.0150478	0.2200002	0.511301	0.00915563	0	-0.0347601	0.029437	0.2376694	0.5847313
ENSG00000140320	BAHD1	15	40746180	C	T	0.416501	0.00360349	0.0144258	0.7600007	-0.089976	0.00803558	4.21E-29	-0.0400495	0.160369	0.8027941	0.9814763
ENSG00000140323	DISP2	15	40656846	G	C	0.151093	-0.00882209	0.0190149	0.6600001	0.252958	0.0116932	8.83E-104	-0.0348758	0.0751876	0.6427552	0.5765849
ENSG00000140326	CDAN1	15	43022540	T	G	0.220676	0.0128621	0.0173018	0.3900004	-0.115023	0.00961057	5.20E-33	-0.111822	0.15071	0.4581075	0.5852644
ENSG00000140332	TLE3	15	70365322	G	A	0.423459	0.00394241	0.0142762	0.5999997	-0.0838802	0.0119668	2.39E-12	-0.0470005	0.170329	0.7825953	0.8132142
ENSG00000140350	ANP32A	15	69092055	C	T	0.461233	0.00969309	0.0144058	0.7099994	0.073335	0.0118857	6.83E-10	0.132175	0.197603	0.5035628	0.05552218
ENSG00000140365	COMMD4	15	75631250	T	C	0.0417495	-0.0394336	0.0383785	0.28	-0.72092	0.0320868	8.57E-112	0.054699	0.0532911	0.304694	0.7651509
ENSG00000140367	UBE2Q2	15	76164520	T	G	0.0516899	0.0784641	0.0303778	0.007499978	-0.355205	0.0171798	5.72E-95	-0.220898	0.0861866	0.01037662	0.2858483
ENSG00000140368	PSTPIP1	15	77307686	A	T	0.287276	-0.0256894	0.0158233	0.1199999	0.396818	0.00869106	0	-0.0647385	0.0399006	0.1046981	0.6719185
ENSG00000140374	ETFA	15	76555754	C	G	0.136183	0.00943253	0.0220852	0.6800001	0.713253	0.0113755	0	0.0132247	0.0309648	0.6693157	0.6802649
ENSG00000140379	BCL2A1	15	80258509	C	A	0.409543	0.00317743	0.0144615	0.9299999	0.151182	0.0118792	4.21E-37	0.0210173	0.0956706	0.8261175	0.6323663
ENSG00000140382	HMG20A	15	77745351	G	T	0.224652	-0.00247313	0.0171744	0.7700005	0.233025	0.00984058	5.80E-124	-0.0106132	0.0737033	0.8855018	0.4251443
ENSG00000140386	SCAPER	15	76919155	C	T	0.331014	0.0110497	0.0151075	0.3900004	0.649151	0.00765251	0	0.0170218	0.0232736	0.4645485	0.655683
ENSG00000140391	TSPAN3	15	77356342	T	C	0.309145	0.027742	0.0152818	0.0659994	-0.510473	0.00789975	0	-0.0543457	0.0299484	0.06957831	0.2411309
ENSG00000140395	SKIC8	15	78581156	C	T	0.180915	-0.0189232	0.0180197	0.2399999	0.516432	0.0104808	0	-0.0366422	0.0349006	0.2937636	0.04833367
ENSG00000140396	NCOA2	8	71169018	G	A	0.17992	0.00627129	0.0195585	0.81	0.109111	0.010404	9.87E-26	0.0574761	0.179337	0.7485947	0.681792
ENSG00000140398	NEIL1	15	75643444	T	C	0.435388	-0.00821376	0.0143293	0.3700002	-0.053606	0.00870906	7.50E-10	0.153225	0.268465	0.5681727	0.6649983
ENSG00000140400	MAN2C1	15	75654552	C	T	0.281312	-0.0154631	0.0163303	0.4	0.91676	0.00802693	0	-0.0168671	0.0178137	0.3437082	0.3305685
ENSG00000140403	DNAJA4	15	78565483	G	A	0.465209	0.0245364	0.0143227	0.1800002	0.292336	0.00854035	8.66E-257	0.0839321	0.0490553	0.0870864	0.000311782
ENSG00000140406	TLNRD1	15	81294818	T	G	0.0656064	0.054546	0.0360843	0.2099999	-0.627334	0.020381	4.79E-208	-0.0869489	0.0575894	0.1310926	0.02091703
ENSG00000140416	TPM1	15	63349472	C	T	0.122266	0.0022234	0.0214056	0.7400005	-0.48387	0.0108591	0	-0.00459503	0.0442384	0.9172727	0.1661885
ENSG00000140443	IGF1R	15	99349979	T	C	0.183897	-0.0220328	0.016436	0.2099999	-0.198431	0.0102473	1.55E-83	0.111035	0.083028	0.1811181	0.5042206
ENSG00000140450	ARRDC4	15	98489926	T	C	0.251491	-0.00964452	0.0169999	0.6999999	0.75759	0.00866321	0	-0.0127305	0.0224399	0.5704994	0.8038853
ENSG00000140451	PIF1	15	65112849	A	G	0.0188867	-0.0343018	0.050459	0.3400001	0.336124	0.0281837	8.64E-33	-0.102051	0.150364	0.4973319	0.4420364
ENSG00000140455	USP3	15	63841816	C	T	0.393638	0.00662074	0.0149366	0.9199999	0.3962	0.00798864	0	0.0167106	0.0377011	0.6575933	0.5108188
ENSG00000140459	CYP11A1	15	74645090	T	C	0.162028	0.00997859	0.0204405	0.7600007	0.0826582	0.0113735	3.66E-13	0.120721	0.247847	0.6262018	0.2618619
ENSG00000140463	BBS4	15	73004672	A	C	0.0606362	-0.000719118	0.0265421	0.98	0.588602	0.0165474	3.98E-277	-0.00122174	0.0450935	0.9783852	0.4785151
ENSG00000140464	PML	15	74313583	T	G	0.406561	-0.0229975	0.0144436	0.08600031	0.206873	0.00806183	3.20E-145	-0.111167	0.0699529	0.1120217	0.002607296
ENSG00000140470	ADAMTS17	15	100697002	T	C	0.400596	-0.00638144	0.0145493	0.6300007	-0.191	0.0119248	9.72E-58	0.0334107	0.0762029	0.6610647	0.6236144
ENSG00000140471	LINS1	15	101121504	C	G	0.451292	0.0310379	0.0142048	0.0129999	-0.676746	0.00768941	0	-0.0458634	0.0209963	0.02893611	0.6563098
ENSG00000140474	ULK3	15	75132072	A	G	0.311133	-0.00500582	0.0158948	0.5400003	0.526887	0.00856626	0	-0.00950074	0.0301678	0.7528146	0.7470854
ENSG00000140479	PCSK6	15	101953111	A	C	0.302187	0.0145482	0.0156048	0.3900004	0.0603713	0.0087749	5.99E-12	0.240979	0.260843	0.3555661	0.6189194
ENSG00000140497	SCAMP2	15	75150888	T	G	0.507952	-0.0188649	0.0142512	0.07699987	-0.280284	0.00779815	6.68E-283	0.0673065	0.0508801	0.1858872	0.3541298
ENSG00000140511	HAPLN3	15	89429688	G	C	0.314115	-0.011215	0.015265	0.4700002	-0.11083	0.00853302	1.42E-38	0.101191	0.137953	0.4632446	0.3737615
ENSG00000140521	POLG	15	89868813	T	C	0.387674	-0.00156777	0.0146289	0.7099994	0.116021	0.008162	7.42E-46	-0.0135128	0.126092	0.914657	0.8221739
ENSG00000140526	ABHD2	15	89688140	T	C	0.126243	0.0236921	0.0226087	0.2999998	-0.287582	0.0116644	3.29E-134	-0.0823839	0.0786876	0.2951114	0.3326833

ENSG00000140543	DET1	15	89072348	T	C	0.440358	-0.00480581	0.0143736	0.8700001	0.22259	0.00800004	2.25E-170	-0.0215904	0.064579	0.7381338	0.1653105
ENSG00000140545	MFGE8	15	89449279	A	G	0.275348	-0.0217074	0.0157848	0.2099999	0.506632	0.00861368	0	-0.0428464	0.0311648	0.1691831	0.2776643
ENSG00000140548	ZNF710	15	90585031	C	T	0.289264	-0.00764267	0.0163924	0.6300007	0.106046	0.00971794	1.01E-27	-0.0720693	0.154719	0.6413532	0.9095371
ENSG00000140553	UNC45A	15	91484525	A	T	0.163022	0.0309841	0.0200342	0.1499999	0.166484	0.0104808	8.10E-57	0.186109	0.120906	0.1237355	0.7667821
ENSG00000140563	MCTP2	15	94899199	T	C	0.281312	0.0499967	0.0158231	0.00329997	0.230894	0.00889736	1.78E-148	0.216536	0.0690359	0.001709401	0.08157775
ENSG00000140564	FURIN	15	91419255	T	C	0.395626	-0.00727779	0.0146872	0.7099994	-0.178141	0.00828326	1.36E-102	0.040854	0.0824687	0.6203264	0.4707373
ENSG00000140575	IQGAP1	15	90988462	G	A	0.466203	0.0228269	0.0141825	0.09699961	-0.457766	0.00756657	0	-0.0498659	0.030993	0.1076289	0.2155886
ENSG00000140577	CRTC3	15	91130867	A	G	0.327038	-0.0114131	0.0151768	0.4799997	-0.174965	0.00935003	3.89E-78	0.0652307	0.0868118	0.4524101	0.2613319
ENSG00000140598	EFL1	15	82488837	C	T	0.159046	-0.0205772	0.0211398	0.2700001	-0.10785	0.0109623	7.71E-23	0.190795	0.196969	0.3327163	0.6460887
ENSG00000140612	SEC11A	15	85236361	T	G	0.269384	-0.0156249	0.0161095	0.2700001	0.183094	0.00913388	2.21E-89	-0.0853381	0.0880877	0.3326525	0.09811856
ENSG00000140632	GLYR1	16	4875273	A	G	0.084493	0.00606983	0.0260428	0.8700001	-0.200436	0.014693	2.27E-42	-0.0302832	0.12995	0.8157324	0.606229
ENSG00000140650	PMM2	16	8912934	A	G	0.0238569	0.00983204	0.0438814	0.7700005	1.03978	0.0363494	5.81E-180	0.00945589	0.0422039	0.8227164	0.538727
ENSG00000140675	SLC5A2	16	31498252	T	A	0.258449	-0.00364802	0.0157955	0.9	0.0565541	0.00925357	9.86E-10	-0.064505	0.279498	0.8174792	0.9893132
ENSG00000140678	ITGAX	16	31380386	C	T	0.318091	0.00450572	0.0153436	0.81	-0.721197	0.00810032	0	-0.00624756	0.0212753	0.769023	0.1435062
ENSG00000140682	TGFB1I1	16	31486093	T	C	0.257455	-0.00507989	0.0157905	0.9699999	0.0980801	0.00903182	1.80E-27	-0.0517933	0.161067	0.7477827	0.9905178
ENSG00000140688	RUSF1	16	31510711	A	G	0.255467	-0.0054752	0.0158725	0.9699999	0.0748422	0.00919414	3.95E-16	-0.0731566	0.21227	0.7303654	0.9940328
ENSG00000140691	ARMC5	16	31473944	T	C	0.257455	-0.00507989	0.0157905	0.9699999	0.150034	0.00908657	3.03E-61	-0.0338583	0.105266	0.7477221	0.9838163
ENSG00000140694	PARN	16	14628071	G	T	0.147117	-0.0123372	0.020503	0.5199996	0.161773	0.0168562	8.21E-22	-0.0762626	0.126988	0.5481415	0.4689908
ENSG00000140718	FTO	16	53946864	C	T	0.239563	0.00313368	0.0168862	0.8499999	-0.207933	0.00961932	1.26E-103	-0.0150706	0.0812128	0.8527823	0.3547854
ENSG00000140740	UQCRC2	16	21979481	A	G	0.194831	-0.0235693	0.0178794	0.17	-0.125828	0.0158793	2.30E-15	0.187314	0.144047	0.1934755	0.3878756
ENSG00000140743	CDR2	16	22402871	C	T	0.11332	-0.0254602	0.025288	0.33	-0.638973	0.0129363	0	0.0398455	0.0395842	0.3141268	0.779214
ENSG00000140749	IGSF6	16	21658295	A	G	0.335984	-0.0228852	0.0149984	0.2	0.218094	0.00926373	1.49E-122	-0.104933	0.0689146	0.1278468	0.4503289
ENSG00000140750	ARHGAP17	16	24978846	A	C	0.277336	-0.0184638	0.0154163	0.17	-0.104713	0.00855082	1.76E-34	0.176327	0.147926	0.2332639	0.02731763
ENSG00000140807	NKD1	16	50626444	C	T	0.263419	0.00368457	0.0152899	0.7800007	-0.0866731	0.00882852	9.48E-23	-0.0425111	0.176462	0.8096264	0.4872214
ENSG00000140829	DHX38	16	72137136	G	A	0.373757	-0.00913034	0.0146724	0.4700002	0.207886	0.00817971	1.73E-142	-0.04392	0.0706003	0.5338804	0.5919118
ENSG00000140830	TXNL4B	16	72103259	C	T	0.028827	-0.00394657	0.0405961	0.9199999	-0.256792	0.0222047	6.21E-31	0.0153687	0.158095	0.922558	0.1569332
ENSG00000140836	ZFHX3	16	72955190	G	T	0.321074	-5.52E-05	0.0149841	0.8	0.130767	0.00843559	3.37E-54	-0.000422179	0.114586	0.9970603	0.4142952
ENSG00000140848	CPNE2	16	57154163	A	G	0.0347913	0.00637263	0.0363672	0.8499999	0.525269	0.0197751	1.86E-155	0.0121321	0.0692369	0.8609018	0.7484701
ENSG00000140853	NLRC5	16	57070420	G	A	0.323062	0.0196269	0.0151429	0.2999998	0.417959	0.0118743	2.09E-271	0.0469589	0.0362551	0.1952393	0.04746565
ENSG00000140854	KATNB1	16	57780402	G	A	0.133201	-0.0293489	0.0199302	0.2099999	0.234453	0.0157165	2.53E-50	-0.125181	0.0854206	0.1427951	0.7647053
ENSG00000140876	NUDT7	16	77766284	C	T	0.0616302	-0.0235293	0.0305621	0.4500005	1.27177	0.0188142	0	-0.0185012	0.0240327	0.4413978	0.2488138
ENSG00000140931	CMTM3	16	66642786	T	C	0.467197	-0.0112557	0.014303	0.3400001	-0.217814	0.00787472	2.12E-168	0.0516758	0.0656927	0.4314994	0.8007906
ENSG00000140932	CMTM2	16	66617764	C	A	0.05666	-0.0049352	0.0380568	0.7400005	0.21131	0.0175181	1.67E-33	-0.0233552	0.18011	0.8968257	0.9325879
ENSG00000140939	NOL3	16	67206850	T	C	0.0318091	0.0245091	0.0405587	0.7700005	0.143528	0.0196851	3.07E-13	0.170762	0.283553	0.5470261	0.3326791
ENSG00000140941	MAP1LC3B	16	87427993	C	G	0.459245	-0.00201484	0.0143601	0.8499999	-0.380844	0.0114225	9.67E-244	0.00529045	0.0377063	0.8884175	0.1157376
ENSG00000140943	MBTPS1	16	84118939	G	A	0.294235	-0.015674	0.0154974	0.4400003	0.499102	0.00837452	0	-0.0314044	0.031055	0.3118969	0.464959
ENSG00000140945	CDH13	16	83245306	A	G	0.464215	-0.0107984	0.0143104	0.4	-0.138258	0.00797795	2.79E-67	0.0781033	0.103603	0.4509274	0.09118193
ENSG00000140948	ZCCHC14	16	87482751	C	T	0.124254	-0.0552121	0.0212302	0.006100002	-0.268929	0.0122072	1.47E-107	0.205303	0.0794916	0.009802866	0.2888801
ENSG00000140950	MEAK7	16	84549660	G	C	0.215706	0.0258993	0.0178635	0.1299999	0.355374	0.0183802	2.75E-83	0.0728791	0.050408	0.1482374	0.6495952
ENSG00000140961	OSGIN1	16	83990912	C	G	0.311133	0.00162347	0.0149507	0.9	-0.239422	0.00840626	1.99E-178	-0.00678079	0.0624454	0.9135297	0.04433258
ENSG00000140983	RHOT2	16	721130	A	G	0.128231	-0.0139106	0.0194846	0.6899999	0.133398	0.0128969	4.48E-25	-0.104279	0.146411	0.4763203	0.8627614
ENSG00000140986	RPL3L	16	2000791	G	A	0.191849	0.0252088	0.0178758	0.14	-0.232655	0.0127067	6.94E-75	-0.108353	0.0770615	0.1597078	0.1524409
ENSG00000140987	ZSCAN32	16	3441575	T	G	0.0705765	0.00415448	0.0302902	0.95	-0.282476	0.033569	3.94E-17	-0.0147074	0.107245	0.890922	0.5422015
ENSG00000140988	RPS2	16	2013457	G	A	0.191849	0.0252088	0.0178758	0.14	0.121732	0.0129772	6.57E-21	0.207085	0.148496	0.1631521	0.122724
ENSG00000140990	NDUFB10	16	2010742	C	T	0.32505	0.0453696	0.015579	0.005600025	-0.219178	0.0108595	1.38E-90	-0.206999	0.0718155	0.00394687	0.4101828
ENSG00000140993	TIGD7	16	3352280	A	G	0.347913	-0.0272308	0.0153027	0.08	0.251197	0.00866449	8.40E-185	-0.108404	0.0610337	0.07571079	0.3016047
ENSG00000140995	DEF8	16	90024400	T	C	0.138171	0.0183217	0.019329	0.4700002	0.190174	0.0114045	1.98E-62	0.0963418	0.101803	0.3439656	0.1253797
ENSG00000141002	TCF25	16	89958896	A	G	0.27336	-0.0149797	0.0155851	0.33	0.250203	0.00872128	5.26E-181	-0.0598702	0.0623247	0.3367452	0.4211752
ENSG00000141012	GALNS	16	88901760	T	G	0.290258	-0.0166066	0.016302	0.28	-0.378101	0.00989408	0	0.043921	0.0431307	0.3085244	0.1658575
ENSG00000141013	GAS8	16	90098710	A	C	0.397614	0.00489777	0.0143581	0.5300002	-0.16838	0.00832872	6.97E-91	-0.0290876	0.0852841	0.7330532	0.6740419

ENSG00000141026	MED9	17	17388420	G	C	0.298211	-0.00707796	0.0162763	0.4100001	-0.0514555	0.0087871	4.75E-09	0.137555	0.317189	0.664529	0.3520988
ENSG00000141027	NCOR1	17	16028108	G	C	0.483101	0.0103267	0.014236	0.4600002	-0.315371	0.00779104	0	-0.0327446	0.0451477	0.4682822	0.7650267
ENSG00000141028	NA	17	13928396	C	G	0.213718	-0.00432285	0.0175065	0.64	-0.972043	0.0201668	0	0.00444718	0.0180102	0.8049662	0.1297226
ENSG00000141030	COPS3	17	17167374	A	G	0.508946	-0.00785559	0.0141962	0.7700005	0.556864	0.00730384	0	-0.0141068	0.0254938	0.5800278	0.6700007
ENSG00000141034	GID4	17	17957162	T	C	0.375746	0.0235731	0.0153799	0.14	-0.192451	0.00968317	6.73E-88	-0.122489	0.0801532	0.1264669	0.7140563
ENSG00000141040	ZNF287	17	16463610	A	C	0.0437376	-0.0248852	0.0417812	0.5300002	0.244436	0.023665	5.21E-25	-0.101807	0.171213	0.5520976	0.07654468
ENSG00000141068	KSR1	17	25868565	G	A	0.359841	0.00592482	0.0150043	0.6700003	-0.361502	0.00883622	0	-0.0163894	0.0415073	0.6929489	0.8627758
ENSG00000141076	UTP4	16	69184067	T	C	0.0248509	0.00967118	0.041777	0.84	-0.888715	0.0233022	0	-0.0108822	0.0470092	0.8169333	0.8609124
ENSG00000141084	RANBP10	16	67798780	T	C	0.0387674	0.0174769	0.03356	0.64	0.153481	0.0210179	2.83E-13	0.11387	0.219214	0.6034494	0.7253824
ENSG00000141096	DPEP3	16	68012149	A	C	0.141153	-0.0224822	0.0213719	0.1800002	-0.432899	0.0111946	0	0.0519341	0.0493876	0.2930004	0.8324435
ENSG00000141098	GFOD2	16	67730879	G	T	0.114314	-0.0339731	0.0242804	0.1100001	0.147994	0.0126662	1.54E-31	-0.229558	0.165236	0.1647499	0.5128689
ENSG00000141101	NOB1	16	69782306	C	T	0.428429	0.0018123	0.0144168	0.7800007	-0.0743608	0.00901015	1.54E-16	-0.0243717	0.193899	0.899975	0.1571425
ENSG00000141127	PRPSAP2	17	18788989	T	G	0.442346	0.00221424	0.0142299	0.8700001	0.22351	0.00862016	3.15E-148	0.00990665	0.0636666	0.8763468	0.3321247
ENSG00000141140	NA	17	34875380	T	G	0.451292	0.028633	0.0142264	0.04700023	0.155964	0.00791946	2.44E-86	0.183587	0.0916908	0.04525918	0.8076149
ENSG00000141141	NA	17	35986640	C	T	0.246521	-0.00558707	0.0175973	0.6800001	-0.399442	0.00938581	0	0.0139872	0.0440559	0.7508741	0.992523
ENSG00000141161	UNC45B	17	33495600	C	A	0.110338	-0.0270491	0.02296	0.2200002	0.474303	0.0126704	1.10E-306	-0.0570292	0.0484318	0.2389905	0.9592066
ENSG00000141179	PCTP	17	53874265	G	T	0.054672	0.00539531	0.0305111	0.9299999	-0.426225	0.0184115	1.45E-118	-0.0126584	0.0715866	0.8596451	0.5068375
ENSG00000141219	C17orf80	17	71236731	C	G	0.445328	0.007387	0.0142471	0.59	-0.330943	0.0077444	0	-0.0223211	0.0430532	0.604142	0.2602508
ENSG00000141232	TOB1	17	48942461	A	C	0.27336	0.01806	0.0155761	0.2200002	-0.312109	0.00852451	1.77E-293	-0.0578645	0.049931	0.246502	0.6505372
ENSG00000141252	VPS53	17	518432	A	G	0.238569	-0.0180153	0.0165187	0.28	-0.504896	0.00902897	0	0.0356812	0.0327232	0.2755401	0.1641324
ENSG00000141258	SGSM2	17	2262572	T	G	0.0298211	0.00887621	0.0347397	0.7199992	-0.474298	0.0187213	1.33E-141	-0.0187144	0.0732482	0.7983422	0.6165237
ENSG00000141279	NPEPPS	17	45650474	G	C	0.475149	-0.00461378	0.0141927	0.9	-0.220976	0.0084588	1.96E-150	0.0208791	0.0642323	0.7451387	0.9216151
ENSG00000141293	SKAP1	17	46359219	C	T	0.241551	0.0256406	0.0166012	0.1100001	-0.354669	0.00919504	0	-0.0722944	0.046845	0.1227662	0.01072543
ENSG00000141294	LRRC46	17	45912036	T	C	0.487078	0.000335689	0.0142657	0.8700001	0.200497	0.00787225	4.37E-143	0.00167429	0.0711518	0.9812266	0.1994824
ENSG00000141295	SCRN2	17	45916878	T	C	0.158052	0.0366939	0.0193759	0.04300015	-0.681298	0.0150911	0	-0.0538588	0.0284647	0.0584741	0.5713946
ENSG00000141298	SSH2	17	28105125	A	T	0.518887	0.01961	0.0141879	0.0519996	-0.510241	0.0110446	0	-0.0384328	0.0278187	0.1671113	0.2347259
ENSG00000141316	SPACA3	17	31311144	C	T	0.151093	0.00766774	0.019698	0.64	0.217649	0.0114165	4.99E-81	0.0352299	0.0905226	0.6971402	0.2727061
ENSG00000141337	ARSG	17	66337097	C	T	0.45825	0.00916432	0.0142472	0.4199997	0.161044	0.00797912	1.38E-90	0.0569056	0.0885125	0.5202824	0.2401842
ENSG00000141349	G6PC3	17	42150906	T	A	0.479125	0.0036718	0.0142436	0.6899999	0.17944	0.00794518	6.11E-113	0.0204625	0.0793831	0.7965851	0.7859245
ENSG00000141376	BCAS3	17	59112506	T	C	0.10338	0.042024	0.0236123	0.0990011	0.104396	0.0127824	3.16E-16	0.402544	0.231488	0.08204515	0.3169617
ENSG00000141378	PTRH2	17	57768492	C	T	0.115308	-0.00727476	0.0253816	0.6100002	-0.162956	0.0136972	1.23E-32	0.0446425	0.155803	0.7744704	0.5659137
ENSG00000141380	SS18	18	23633879	A	G	0.265408	0.0197456	0.0157137	0.1900002	-0.0630743	0.00872904	4.98E-13	-0.313053	0.252869	0.2157144	0.9645498
ENSG00000141384	TAF4B	18	23888774	A	C	0.0984095	-0.00264291	0.0254558	0.9699999	0.181297	0.0154122	6.04E-32	-0.0145778	0.140415	0.9173126	0.9620541
ENSG00000141385	AFG3L2	18	12353128	A	G	0.0924453	0.000876765	0.0230841	0.91	-0.145764	0.0132342	3.26E-28	-0.00601497	0.158367	0.9697027	0.1862704
ENSG00000141391	PRELID3A	18	12420066	G	C	0.17992	0.0364019	0.0203274	0.09599973	0.156657	0.0116922	6.18E-41	0.232367	0.130911	0.07589857	0.0759279
ENSG00000141401	IMPA2	18	12005950	C	T	0.190855	0.023365	0.0173952	0.14	-0.452762	0.00957729	0	-0.0516054	0.0384357	0.179387	0.1818864
ENSG00000141404	GNAL	18	11787319	A	G	0.0596421	0.0656388	0.0313542	0.016	0.153728	0.0187373	2.32E-16	0.42698	0.210494	0.04251257	0.4161229
ENSG00000141424	SLC39A6	18	33698921	A	G	0.368787	0.0139204	0.0149253	0.3700002	0.0742012	0.00831759	4.62E-19	0.187603	0.202243	0.353607	0.7304889
ENSG00000141425	RPRD1A	18	33605944	A	C	0.0725646	0.025663	0.0289704	0.4799997	0.125126	0.0172521	4.08E-13	0.205097	0.23325	0.3792381	0.8080669
ENSG00000141428	C18orf21	18	33555643	T	C	0.0805169	0.00635654	0.0264815	1	-0.167232	0.0241288	4.18E-12	-0.0380103	0.158447	0.8104131	0.7112466
ENSG00000141429	GALNT1	18	33226439	G	A	0.439364	-0.00371957	0.0142291	0.6700003	-0.199445	0.00787127	1.21E-141	0.0186496	0.0713471	0.7937903	0.9546783
ENSG00000141447	OSBPL1A	18	21859926	C	T	0.389662	-0.0183156	0.0146968	0.2599998	0.270643	0.00813747	1.52E-242	-0.0676745	0.0543415	0.2130007	0.7517173
ENSG00000141452	RMC1	18	21097609	C	G	0.347913	0.0304624	0.0148974	0.06299992	-0.310603	0.00909683	1.64E-255	-0.0980751	0.0480488	0.04123531	0.6963061
ENSG00000141458	NPC1	18	21126584	T	G	0.207753	0.00268724	0.0180904	0.89	-0.330142	0.00981014	2.81E-248	-0.00813966	0.0547964	0.8819138	0.06112256
ENSG00000141469	SLC14A1	18	43318288	G	A	0.0785288	-0.0242137	0.0284673	0.3100002	0.420541	0.0139579	2.01E-199	-0.0575776	0.0677191	0.3951913	0.5142628
ENSG00000141480	ARRB2	17	4619289	A	G	0.218688	0.0196732	0.0165788	0.1900002	0.476801	0.00935828	0	0.0412608	0.0347803	0.2354935	0.8135079
ENSG00000141485	SLC13A5	17	6602459	T	A	0.463221	0.00608069	0.0142162	0.6499995	0.103892	0.00803298	2.92E-38	0.0585289	0.136911	0.6690182	0.233623
ENSG00000141497	ZMYND15	17	4646365	T	G	0.360835	-0.0163939	0.014867	0.4700002	0.257753	0.00843317	3.66E-205	-0.0636031	0.0577168	0.2704673	0.364213
ENSG00000141499	WRAP53	17	7598104	A	C	0.0984095	-0.0199345	0.0267723	0.4899999	0.0886295	0.0138143	1.40E-10	-0.224919	0.304097	0.4595246	0.819526
ENSG00000141503	MINK1	17	4769019	C	T	0.369781	-0.00244933	0.0152246	0.59	0.350203	0.00802568	0	-0.00699403	0.043474	0.872189	0.2533885

ENSG00000141504	SAT2	17	7530373	T	C	0.388668	-0.0142754	0.0146649	0.17	0.274896	0.00798005	4.84E-260	-0.0519302	0.0533684	0.3305277	0.952302
ENSG00000141505	ASGR1	17	7079816	G	A	0.233598	0.0046583	0.016657	0.7300002	-0.150086	0.0100432	1.70E-50	-0.0310375	0.111002	0.7797758	0.9856963
ENSG00000141506	PIK3R5	17	8825631	C	A	0.107356	0.022215	0.0232916	0.3900004	-0.155767	0.0124294	4.98E-36	-0.142616	0.14996	0.3415906	0.960344
ENSG00000141510	TP53	17	7577976	T	C	0.0248509	-0.0386632	0.0520578	0.4100001	-0.250815	0.0265931	4.04E-21	0.15415	0.208197	0.459054	0.3559889
ENSG00000141519	CCDC40	17	78042423	G	C	0.0765408	0.0034397	0.0261629	0.7700005	0.232015	0.0141585	2.37E-60	0.0148253	0.112767	0.8954049	0.1434103
ENSG00000141524	TMC6	17	76117513	T	C	0.0497018	0.00494037	0.0279369	0.7800007	0.446716	0.0157093	7.20E-178	0.0110593	0.0625396	0.8596365	0.1488128
ENSG00000141526	SLC16A3	17	80202639	G	A	0.313121	-0.000454044	0.014864	0.6899999	0.373446	0.00908846	0	-0.00121582	0.0398022	0.9756312	0.6507275
ENSG00000141527	CARD14	17	78163460	T	C	0.454274	-0.00151832	0.0142854	0.89	0.0996103	0.0092372	4.11E-27	-0.0152426	0.14342	0.9153605	0.274893
ENSG00000141540	TTYH2	17	72233904	C	A	0.154076	-0.0312152	0.019014	0.06100002	0.218942	0.0107079	6.41E-93	-0.142573	0.0871244	0.1017498	0.1498458
ENSG00000141542	RAB40B	17	80634726	G	A	0.39662	0.00431563	0.0143813	0.7899998	0.111129	0.00815605	2.83E-42	0.0388343	0.129442	0.7641669	0.2698105
ENSG00000141543	EIF4A3	17	78114997	T	C	0.345924	-0.00670297	0.0146938	0.6800001	-0.193093	0.00817852	3.06E-123	0.0347137	0.0761111	0.6483241	0.3838035
ENSG00000141551	CSNK1D	17	80214253	A	C	0.0258449	-0.029755	0.0365572	0.35	-0.821972	0.028537	1.93E-182	0.0361995	0.0444928	0.4158712	0.2204511
ENSG00000141556	TBCD	17	80805332	C	T	0.156064	-0.0113038	0.0197311	0.4600002	0.76413	0.0108638	0	-0.014793	0.0258225	0.5667303	0.4842206
ENSG00000141560	FN3KRP	17	80681381	T	C	0.409543	-0.00220623	0.0145705	0.6999999	-0.804631	0.0067941	0	0.00274192	0.0181083	0.8796464	0.3740902
ENSG00000141562	NARF	17	80431099	A	G	0.375746	0.0122916	0.015177	0.4799997	-0.105086	0.00896374	9.67E-32	-0.116968	0.144769	0.4191149	0.3487617
ENSG00000141564	RPTOR	17	78729395	C	T	0.180915	-0.0146632	0.0180101	0.3800004	-0.341037	0.0101999	4.20E-245	0.0429959	0.0528255	0.4156884	0.5450095
ENSG00000141568	FOXK2	17	80540063	A	G	0.343936	-0.0121109	0.0151623	0.4	-0.0679717	0.00859401	2.59E-15	0.178176	0.224202	0.4267831	0.8347454
ENSG00000141569	TRIM65	17	73884750	T	C	0.135189	0.00487414	0.020317	0.9599999	-0.246644	0.0110991	2.10E-109	-0.0197619	0.0823787	0.8104151	0.8197615
ENSG00000141570	CBX8	17	77770706	T	C	0.305169	0.00695132	0.0153163	0.59	0.0774952	0.0128066	1.44E-09	0.0897	0.198197	0.6508512	0.7621619
ENSG00000141574	SECTM1	17	80285425	G	C	0.0377734	-0.0444014	0.0352727	0.1800002	-0.344793	0.0227766	9.09E-52	0.128777	0.102654	0.2096698	0.5320164
ENSG00000141576	RNF157	17	74187494	A	G	0.43837	0.0320144	0.0142526	0.01400006	0.197265	0.00862714	1.02E-115	0.162292	0.0725989	0.02538754	0.9271296
ENSG00000141577	CEP131	17	79180096	G	A	0.454274	0.0137698	0.0142341	0.29	-0.214531	0.0112864	1.46E-80	-0.0641855	0.0664356	0.3339788	0.1505516
ENSG00000141580	WDR45B	17	80589433	A	C	0.293241	0.00735982	0.0152169	0.6899999	0.49063	0.0158571	3.39E-210	0.0150008	0.0310188	0.6286685	0.6676227
ENSG00000141582	CBX4	17	77810091	G	T	0.419483	0.0106763	0.0143189	0.4100001	-0.140193	0.00939343	2.28E-50	-0.0761542	0.102264	0.4564651	0.2326086
ENSG00000141622	ARK2C	18	43974937	A	C	0.203777	-0.0122229	0.0175687	0.5400003	-0.0652449	0.00986854	3.81E-11	0.187339	0.27076	0.4890002	0.5874338
ENSG00000141627	DYM	18	46778878	C	T	0.362823	-0.0104868	0.0150463	0.4700002	-0.226108	0.00896215	1.92E-140	0.0463796	0.0665702	0.4859887	0.8221701
ENSG00000141642	ELAC1	18	48504426	A	G	0.465209	0.0167432	0.0142925	0.17	0.0730082	0.00806257	1.36E-19	0.229333	0.197397	0.2453225	0.6729021
ENSG00000141644	MBD1	18	47800698	G	C	0.374751	0.00946413	0.0147983	0.59	0.0454206	0.00819246	2.95E-08	0.208366	0.327966	0.5252141	0.8407868
ENSG00000141646	SMAD4	18	48552912	A	G	0.410537	0.0194314	0.0145373	0.1299999	0.167484	0.00822259	3.16E-92	0.116019	0.0869846	0.1822729	0.6504805
ENSG00000141655	TNFRSF11A	18	60025518	C	T	0.277336	-0.00921028	0.01602	0.4799997	-0.0893267	0.00897268	2.39E-23	0.103108	0.179641	0.5659898	0.9045755
ENSG00000141664	ZCCHC2	18	60222591	A	G	0.150099	0.0298359	0.020008	0.14	0.300698	0.0155993	8.48E-83	0.099222	0.0667372	0.1370791	0.6279397
ENSG00000141665	FBXO15	18	71777844	T	C	0.0675944	0.0372202	0.0261689	0.17	-0.343921	0.0151065	9.87E-115	-0.108223	0.0762381	0.1557419	0.01364547
ENSG00000141682	PMAIP1	18	57569359	T	A	0.170974	-0.00612749	0.0196204	0.8499999	0.162516	0.0107957	3.26E-51	-0.0377039	0.120755	0.7548621	0.8214747
ENSG00000141696	P3H4	17	39963527	T	C	0.207753	0.0109985	0.0181269	0.4899999	0.106467	0.0109791	3.10E-22	0.103304	0.170591	0.544803	0.8644403
ENSG00000141698	NT5C3B	17	39986929	T	C	0.270378	0.0133866	0.0162503	0.3400001	0.975376	0.00820734	0	0.0137246	0.0166609	0.4100783	0.9226076
ENSG00000141699	RETREG3	17	40747086	C	T	0.0497018	0.0339489	0.0323578	0.3400001	-0.2595	0.019243	1.91E-41	-0.130824	0.12507	0.2955559	0.6331207
ENSG00000141720	NA	17	36939160	C	T	0.333002	-0.0275339	0.0151609	0.0329997	-0.0884452	0.00835935	3.67E-26	0.31131	0.173923	0.07346433	0.9345254
ENSG00000141736	ERBB2	17	37865423	G	A	0.328032	0.0209331	0.0151573	0.2599998	0.0686646	0.00842634	3.68E-16	0.30486	0.223892	0.1733114	0.4209293
ENSG00000141741	MIEN1	17	37885894	C	T	0.0526839	0.00955339	0.0314656	0.84	-0.187995	0.0191977	1.21E-22	-0.0508172	0.167455	0.7615333	0.9325023
ENSG00000141744	PNMT	17	37825481	G	A	0.327038	0.0149873	0.0150425	0.5099998	0.0598178	0.00856008	2.79E-12	0.250549	0.254015	0.3239588	0.5241133
ENSG00000141753	IGFBP4	17	38606842	A	T	0.145129	0.0220213	0.0197599	0.2200002	0.320122	0.0114174	5.58E-173	0.0687902	0.0617748	0.265466	0.7034406
ENSG00000141759	TXNL4A	18	77763408	A	T	0.368787	-0.00690703	0.014666	0.6899999	0.10173	0.00828687	1.22E-34	-0.067896	0.144273	0.6379199	0.8161921
ENSG00000141837	CACNA1A	19	13526030	T	C	0.377734	-0.0105532	0.0147928	0.3700002	-0.0952927	0.00868714	5.36E-28	0.110745	0.155563	0.4765285	0.7857577
ENSG00000141854	MISP3	19	14184611	G	C	0.377734	-0.0160667	0.0146195	0.2999998	0.145667	0.0113467	1.01E-37	-0.110298	0.10073	0.2735227	0.9706906
ENSG00000141858	SAMD1	19	14200250	C	A	0.389662	-0.0133208	0.014502	0.4899999	0.0797462	0.0125474	2.08E-10	-0.16704	0.183741	0.3632954	0.6006924
ENSG00000141873	SLC39A3	19	2736176	A	G	0.243539	-0.0244515	0.016771	0.2	0.44893	0.0100149	0	-0.0544662	0.0373775	0.1450632	0.7519661
ENSG00000141905	NFIC	19	3414388	A	G	0.11829	-0.0123261	0.0212708	0.6300007	0.121897	0.0141813	8.28E-18	-0.101119	0.174894	0.5631479	0.8540133
ENSG00000141933	TPGS1	19	513476	A	G	0.0178926	-0.0101568	0.0752525	0.8700001	1.17701	0.0479287	3.59E-133	-0.0086293	0.0639361	0.8926375	0.9755348
ENSG00000141934	PLPP2	19	286218	C	G	0.290258	-0.0100494	0.0153695	0.6100002	0.23697	0.00954825	5.70E-136	-0.0424078	0.0648808	0.5133524	0.9004554
ENSG00000141956	PRDM15	21	43258988	A	G	0.084493	0.0507877	0.0241968	0.0259998	0.452305	0.0146652	7.15E-209	0.112286	0.0536204	0.03625115	0.6892027

ENSG00000141959	PFKL	21	45733596	G	A	0.177932	0.0238324	0.0182253	0.1800002	-0.273838	0.0122436	8.48E-111	-0.0870309	0.0666686	0.191748	0.9800596
ENSG00000141968	VAV1	19	6815051	G	T	0.139165	-0.0132854	0.0216528	0.59	-0.0918307	0.0122412	6.29E-14	0.144673	0.236578	0.5408534	0.3543859
ENSG00000141971	MVB12A	19	17530532	A	G	0.415507	0.0135921	0.0142996	0.4400003	0.393263	0.0112754	1.60E-266	0.0345624	0.0363749	0.3420257	0.6756914
ENSG00000141977	CIB3	19	16278257	G	A	0.0755467	-0.0340595	0.0269122	0.1900002	-0.18277	0.0161426	1.02E-29	0.186352	0.148163	0.2084838	0.8234976
ENSG00000141994	DUS3L	19	5788039	C	T	0.199801	-0.012845	0.0185519	0.5500004	-0.709673	0.0150741	0	0.0180999	0.0261443	0.4887445	0.8939954
ENSG00000142002	DPP9	19	4699960	G	A	0.240557	0.00499948	0.0161435	0.6499995	0.280657	0.0108317	5.03E-148	0.0178135	0.0575244	0.7568136	0.8043573
ENSG00000142025	DMRTC2	19	42352603	T	G	0.374751	0.00859957	0.0146088	0.3400001	-0.17935	0.00817763	1.29E-106	-0.0479485	0.0814834	0.5562337	0.4857834
ENSG00000142039	CCDC97	19	41823441	A	C	0.314115	-0.0108703	0.0156036	0.33	0.0770492	0.00852012	1.52E-19	-0.141083	0.203115	0.4873089	0.9266302
ENSG00000142046	TMEM91	19	41873402	G	T	0.136183	0.0202075	0.0199625	0.2099999	-0.880771	0.0123702	0	-0.022943	0.0226671	0.3114564	0.1775824
ENSG00000142065	ZFP14	19	36848631	T	G	0.178926	0.0127828	0.0178887	0.4400003	0.509042	0.0137747	6.05E-299	0.0251115	0.0351484	0.4749549	0.7545852
ENSG00000142082	SIRT3	11	226194	T	C	0.213718	-0.00110037	0.0169596	0.8700001	0.206724	0.00965917	1.29E-101	-0.00532291	0.0820404	0.9482683	0.3478739
ENSG00000142089	IFITM3	11	323603	C	T	0.34493	0.00096282	0.0151057	0.7899998	0.557959	0.00931893	0	0.00172561	0.0270731	0.9491782	0.8701357
ENSG00000142102	PGGHG	11	292621	G	A	0.255467	-0.0155852	0.0162058	0.25	-0.8334	0.0109857	0	0.0187007	0.019447	0.3362366	0.7284113
ENSG00000142156	COL6A1	21	47413307	T	G	0.0745527	0.013339	0.0252403	0.7499995	0.40702	0.0144417	9.29E-175	0.0327724	0.0620234	0.5972305	0.8986438
ENSG00000142166	IFNAR1	21	34714451	T	A	0.196819	0.0245781	0.0176512	0.07900053	0.35604	0.00956205	1.87E-303	0.0690319	0.0496111	0.1640861	0.247141
ENSG00000142168	SOD1	21	33036589	T	C	0.0576541	0.0100419	0.0344451	0.99	0.216032	0.0259243	7.87E-17	0.0464834	0.159542	0.7707796	0.7957662
ENSG00000142173	COL6A2	21	47535387	A	C	0.129225	0.022773	0.0204189	0.1499999	-0.398665	0.014768	1.68E-160	-0.0571231	0.0512618	0.2651335	0.6112093
ENSG00000142178	SIK1	21	44840701	A	G	0.0854871	0.0223372	0.0219677	0.28	-0.145173	0.0137394	4.27E-26	-0.153866	0.15202	0.3114691	0.2727912
ENSG00000142185	TRPM2	21	45816505	C	T	0.181909	0.00678743	0.0173314	0.7199992	0.206096	0.00980626	4.60E-98	0.0329333	0.0841083	0.6953847	0.7249862
ENSG00000142186	SCYL1	11	65299361	T	C	0.0695825	-0.00836115	0.027489	0.8800001	-0.127325	0.0163492	6.82E-15	0.0656679	0.216061	0.7611799	0.2385143
ENSG00000142188	TMEM50B	21	34828555	C	G	0.446322	0.00639379	0.0142249	0.5199996	-0.156294	0.00798501	2.61E-85	-0.0409088	0.0910379	0.6531718	0.3669688
ENSG00000142192	APP	21	27398153	T	C	0.353877	0.00731638	0.0149413	0.56	0.217559	0.00833331	3.02E-150	0.0336293	0.0686889	0.6244249	0.6422104
ENSG00000142197	DOP1B	21	37597826	C	T	0.173956	-0.00399371	0.0194521	0.83	0.16247	0.011093	1.42E-48	-0.0245812	0.119739	0.8373457	0.9270551
ENSG00000142207	URB1	21	33724332	C	A	0.121272	0.0172995	0.0224771	0.5999997	0.493285	0.0186078	7.51E-155	0.03507	0.0455854	0.4416999	0.4697714
ENSG00000142208	AKT1	14	105248887	A	T	0.389662	-0.00445216	0.0146234	0.7800007	-0.286334	0.00941679	4.47E-203	0.0155488	0.0510736	0.7607933	0.5198495
ENSG00000142227	EMP3	19	48829288	C	T	0.0337972	0.0266039	0.0351209	0.32	-0.262413	0.0237345	2.05E-28	-0.101382	0.134152	0.4498152	0.8886648
ENSG00000142230	SAE1	19	47665208	A	G	0.266402	-0.0290208	0.0156529	0.06900014	-0.166968	0.00908464	1.93E-75	0.17381	0.0942235	0.06508719	0.01212406
ENSG00000142233	NTN5	19	49170501	T	C	0.440358	0.0256018	0.0141649	0.0032	0.229035	0.00799071	1.12E-180	0.111781	0.061969	0.07125819	0.8704996
ENSG00000142235	LMTK3	19	49002487	G	T	0.0367793	0.0383609	0.0425599	0.4199997	-0.338735	0.0297616	5.16E-30	-0.113248	0.126037	0.3689052	0.7067246
ENSG00000142252	GEMIN7	19	45588617	A	G	0.358847	0.0126228	0.0149019	0.2399999	-0.153988	0.0106761	3.68E-47	-0.0819728	0.0969401	0.3977743	0.08695166
ENSG00000142279	WTIP	19	34984566	A	G	0.12326	0.0063249	0.0227299	0.99	0.114805	0.0131539	2.60E-18	0.0550926	0.198088	0.7809186	0.6613925
ENSG00000142303	ADAMTS10	19	8660373	T	G	0.0586481	0.0571395	0.0263629	0.02	-0.368587	0.0219475	2.70E-63	-0.155023	0.0721175	0.03158745	0.3630652
ENSG00000142330	CAPN10	2	241541627	A	T	0.159046	0.0431428	0.0189082	0.01400006	-0.16897	0.0110878	1.94E-52	-0.255328	0.11315	0.02403654	0.4209133
ENSG00000142347	MYO1F	19	8614067	T	G	0.15507	0.0127817	0.0222482	0.4	0.201137	0.0126212	3.54E-57	0.0635472	0.110684	0.5658785	0.05706061
ENSG00000142396	ERVK3-1	19	58821869	G	A	0.198807	0.0198559	0.0186009	0.3900004	0.673484	0.0114419	0	0.0294824	0.0276235	0.2858389	0.8656287
ENSG00000142405	NLRP12	19	54312252	A	T	0.291252	0.00216033	0.0159307	0.8600001	0.326559	0.00915854	1.89E-278	0.00661544	0.0487839	0.892132	0.03189788
ENSG00000142408	CACNG8	19	54479881	C	T	0.474155	0.0248878	0.014322	0.06699926	-0.0888854	0.00871938	2.11E-24	-0.279999	0.163453	0.08670862	0.2533532
ENSG00000142409	ZNF787	19	56615690	T	C	0.0178926	0.0364489	0.0853277	0.7199992	0.438493	0.0373975	9.47E-32	0.0831232	0.194722	0.6694663	0.7713313
ENSG00000142444	TIMM29	19	11041810	C	T	0.439364	-0.00859309	0.0143251	0.6200004	-0.135633	0.00809545	5.28E-63	0.0633555	0.105684	0.5488539	0.9428805
ENSG00000142453	CARM1	19	11007821	A	G	0.151093	0.00800685	0.020324	0.4500005	-0.275156	0.0121327	7.25E-114	-0.0290993	0.0738747	0.6936542	0.2081878
ENSG00000142459	EVI5L	19	7912490	G	C	0.220676	0.0364558	0.017453	0.03899959	0.207342	0.0105292	2.53E-86	0.175825	0.0846474	0.03778812	0.5831861
ENSG00000142494	SLC47A1	17	19440522	A	G	0.299205	0.00904336	0.0156217	0.7099994	0.286237	0.00845612	3.67E-251	0.0315939	0.0545841	0.5627156	0.9717872
ENSG00000142507	PSMB6	17	4700614	T	C	0.0854871	0.0269837	0.0273253	0.4100001	0.151941	0.0135652	4.04E-29	0.177593	0.180539	0.3252713	0.9049283
ENSG00000142512	SIGLEC10	19	51917166	C	T	0.159046	0.0142837	0.01937	0.5300002	-0.307812	0.0106633	3.14E-183	-0.046404	0.0629486	0.4610171	0.1312865
ENSG00000142528	ZNF473	19	50542821	C	T	0.171968	0.00951125	0.0187389	0.58	0.130769	0.0102261	1.92E-37	0.0727329	0.14341	0.6120371	0.07426147
ENSG00000142534	RPS11	19	50001284	T	A	0.126243	-0.0038729	0.0249469	0.5999997	-0.161161	0.0152814	5.29E-26	0.0240313	0.154812	0.8766408	0.1825812
ENSG00000142541	RPL13A	19	49993188	A	G	0.0447316	0.0122776	0.0307092	0.8499999	0.57673	0.019978	2.99E-183	0.0212883	0.0532522	0.6893306	0.7283215
ENSG00000142544	CTU1	19	51606245	G	A	0.404573	-0.038077	0.0144414	0.01400006	-0.0724308	0.00815129	6.35E-19	0.525702	0.207974	0.01148046	0.09462023
ENSG00000142546	NOSIP	19	50076243	C	T	0.173956	-0.021632	0.0186659	0.08999948	0.212728	0.0102348	5.94E-96	-0.101688	0.0878815	0.2472284	0.3884079
ENSG00000142552	RCN3	19	50040547	T	C	0.083499	-0.0297727	0.026484	0.1499999	-0.613821	0.0154963	0	0.0485039	0.0431635	0.26113	0.7019914

ENSG00000142556	ZNF614	19	52524755	T	C	0.16998	-0.0200179	0.0191735	0.3100002	0.103066	0.0111662	2.70E-20	-0.194224	0.187217	0.299538	0.04042608
ENSG00000142583	SLC2A5	1	9121851	G	A	0.428429	-0.0198709	0.0143521	0.09800089	-0.217161	0.0079157	1.07E-165	0.0915031	0.0661738	0.1667356	0.2959596
ENSG00000142599	RERE	1	8645079	T	A	0.471173	-0.0192131	0.0143938	0.09499921	-0.443707	0.00766763	0	0.0433014	0.0324485	0.1820522	0.4585663
ENSG00000142606	MMEL1	1	2543279	T	G	0.310139	-0.00196226	0.0150083	1	-0.491624	0.014754	1.89E-243	0.00399138	0.0305282	0.8959778	0.03941987
ENSG00000142621	FHAD1	1	15650313	A	G	0.400596	-0.00863057	0.014518	0.5400003	0.2038	0.00996159	5.04E-93	-0.0423482	0.0712665	0.552363	0.8584159
ENSG00000142632	ARHGEF19	1	16531744	C	T	0.260437	0.0142274	0.0159826	0.3700002	0.650808	0.0084522	0	0.0218611	0.0245597	0.3734009	0.1023024
ENSG00000142634	EFHD2	1	15746615	C	A	0.0159046	0.0397049	0.0474619	0.3599996	-0.987173	0.0324413	2.24E-203	-0.0402208	0.0480968	0.4030156	0.9618858
ENSG00000142655	PEX14	1	10611580	A	G	0.505964	-0.0178058	0.0141985	0.2700001	-0.20476	0.00786389	1.84E-149	0.0869594	0.0694226	0.2103476	0.2875794
ENSG00000142657	PGD	1	10469425	T	C	0.485089	0.00022427	0.0142015	0.8600001	0.0925058	0.0118297	5.29E-15	0.00242439	0.15352	0.9874004	0.2203578
ENSG00000142669	SH3BGRL3	1	26606837	C	T	0.449304	-0.0271214	0.0142723	0.02999991	0.110536	0.00794549	5.37E-44	-0.245362	0.130318	0.05972765	0.05916036
ENSG00000142675	CNKSR1	1	26510135	T	C	0.319085	-0.00609612	0.0152218	0.8	0.132289	0.00856748	8.70E-54	-0.0460819	0.115104	0.6888976	0.2138296
ENSG00000142676	RPL11	1	24020592	A	G	0.176938	0.00953363	0.0197931	0.59	-0.157503	0.0108401	7.87E-48	-0.0605299	0.125737	0.6302323	0.4265752
ENSG00000142686	C1orf216	1	36182274	A	G	0.0954274	-0.0227285	0.0268878	0.3900004	-0.151928	0.0145461	1.55E-25	0.1496	0.177555	0.3994779	0.04742678
ENSG00000142687	KIAA0319L	1	35961321	G	T	0.00397614	-0.0691789	0.101402	0.32	-0.525409	0.036424	3.61E-47	0.131667	0.193212	0.4955789	0.7059632
ENSG00000142694	EVA1B	1	36788693	C	A	0.0775348	0.0121455	0.0248992	0.6100002	-0.60696	0.0238418	5.79E-143	-0.0200104	0.0410304	0.6257643	0.3134294
ENSG00000142733	MAP3K6	1	27687529	C	T	0.0139165	-0.0854363	0.0470983	0.07100027	0.939538	0.0406903	5.84E-118	-0.0909344	0.0502837	0.07054	0.2443683
ENSG00000142751	GPN2	1	27211280	T	C	0.366799	0.0162901	0.0145601	0.2599998	0.0780242	0.00878631	6.67E-19	0.208783	0.188085	0.2669808	0.5206321
ENSG00000142765	SYTL1	1	27674467	C	T	0.0258449	-0.00844127	0.0374888	0.8700001	-0.545783	0.0293732	4.58E-77	0.0154663	0.0686931	0.8218616	0.9403776
ENSG00000142784	WDTC1	1	27598058	A	G	0.0218688	0.0586283	0.0546453	0.3100002	-0.329105	0.037267	1.04E-18	-0.178145	0.167263	0.2868502	0.2021484
ENSG00000142794	NBPF3	1	21789059	A	G	0.45825	0.00929521	0.0142384	0.4299995	0.42145	0.00894324	0	0.0220553	0.0337876	0.5139083	0.1395148
ENSG00000142798	HSPG2	1	22206264	A	G	0.2167	6.87E-05	0.0169904	0.98	0.399338	0.0170613	3.71E-121	0.000172114	0.0425464	0.9967723	0.3802506
ENSG00000142856	ITGB3BP	1	63982916	C	T	0.318091	-0.00288789	0.0153534	0.84	0.46457	0.0121683	0	-0.00621626	0.033049	0.8508044	0.02898799
ENSG00000142867	BCL10	1	85737352	G	A	0.38171	0.00848061	0.0147436	0.6700003	0.248991	0.00813253	7.34E-206	0.0340599	0.0592238	0.5652205	0.5059186
ENSG00000142892	PIGK	1	77619895	C	G	0.245527	-0.0168165	0.0166744	0.3400001	0.246885	0.00935794	2.18E-153	-0.0681147	0.0675885	0.3135572	0.6555561
ENSG00000142920	AZIN2	1	33566418	C	T	0.463221	-0.0193546	0.0144	0.1800002	0.0891035	0.00877521	3.18E-24	-0.217215	0.163019	0.1827134	0.6923639
ENSG00000142937	RPS8	1	45242687	G	A	0.0954274	0.0232537	0.0199749	0.1199999	-0.557758	0.0149657	5.20E-304	-0.0416914	0.0358303	0.2445949	0.4012595
ENSG00000142949	PTPRF	1	44040100	C	A	0.256461	-0.00183749	0.0171405	0.8200001	-0.0757783	0.0095757	2.50E-15	0.0242482	0.226214	0.9146368	0.09896402
ENSG00000142959	BEST4	1	45251317	G	A	0.138171	0.00637253	0.0184676	0.3900004	0.112363	0.0105377	1.52E-26	0.0567137	0.164442	0.7301809	0.5603821
ENSG00000142961	MOB3C	1	47077975	G	A	0.295229	-0.00864017	0.0160653	0.7199992	0.36957	0.00959497	0	-0.023379	0.0434744	0.5907405	0.8723883
ENSG00000142973	CYP4B1	1	47254297	C	T	0.2167	-0.0171607	0.0178103	0.1900002	0.0881481	0.00993307	7.04E-19	-0.19468	0.203237	0.3381147	0.9778267
ENSG00000143013	LMO4	1	87803469	C	G	0.210736	-0.00930556	0.018144	0.5999997	0.152071	0.00998812	2.41E-52	-0.0611921	0.11938	0.6082439	0.4193089
ENSG00000143033	MTF2	1	93574715	A	G	0.178926	0.00746246	0.0177777	0.7300002	0.1669	0.00993806	2.70E-63	0.0447122	0.10655	0.6747527	0.6588958
ENSG00000143036	SLC44A3	1	95323350	C	T	0.408549	0.000981549	0.0146802	0.91	0.127364	0.00822648	4.58E-54	0.00770664	0.115263	0.946692	0.8260945
ENSG00000143067	ZNF697	1	120176220	T	A	0.117296	0.000697299	0.0216802	0.95	-0.267716	0.0135038	1.80E-87	-0.00260462	0.0809822	0.9743421	0.2531846
ENSG00000143093	STRIP1	1	110585731	A	G	0.469185	-0.0217447	0.0142925	0.1299999	-0.133522	0.0156111	1.20E-17	0.162855	0.108722	0.134161	0.7211307
ENSG00000143110	C1orf162	1	112018774	T	C	0.286282	0.0178305	0.0150926	0.16	-0.145514	0.00838523	1.85E-67	-0.122535	0.103959	0.2385262	0.1665097
ENSG00000143119	CD53	1	111429161	C	T	0.507952	-0.0124781	0.0142874	0.29	0.192429	0.0086085	1.12E-110	-0.0648451	0.0743042	0.3828277	0.669932
ENSG00000143126	CELSR2	1	109805506	A	G	0.465209	0.0139775	0.0143408	0.4100001	0.213213	0.00793013	3.16E-159	0.0655564	0.0673045	0.3300432	0.9342751
ENSG00000143127	ITGA10	1	145534379	A	C	0.0944334	-0.0358818	0.026378	0.14	0.231502	0.0214927	4.71E-27	-0.154996	0.114848	0.177153	0.5532726
ENSG00000143147	GPR161	1	168080409	G	A	0.32505	-0.014468	0.0154902	0.17	-0.0971096	0.00879632	2.46E-28	0.148986	0.160082	0.3520165	0.744557
ENSG00000143149	ALDH9A1	1	165649776	G	A	0.324056	-0.0167652	0.0147589	0.3800004	-0.409504	0.00809114	0	0.0409403	0.03605	0.2561021	0.6698835
ENSG00000143153	ATP1B1	1	169088447	A	G	0.543738	-0.00862493	0.0142202	0.4700002	-0.370492	0.00769563	0	0.0232796	0.038385	0.5441971	0.976678
ENSG00000143155	TIPRL	1	168159060	T	C	0.0437376	-0.0231195	0.0343589	0.4899999	-0.66517	0.0308435	3.75E-103	0.0347573	0.0516794	0.5012304	0.01509249
ENSG00000143156	NME7	1	169219487	C	T	0.384692	0.00909389	0.014538	0.8499999	-0.40255	0.00845871	0	-0.0225907	0.0361179	0.5316617	0.1701758
ENSG00000143157	POGK	1	166817131	T	C	0.417495	-0.0233729	0.0147059	0.1100001	-0.177111	0.0082671	8.05E-102	0.131968	0.0832603	0.1129664	0.1735833
ENSG00000143158	MPC2	1	167896122	T	C	0.340954	0.0134006	0.0149969	0.5500004	-0.0991542	0.00941492	6.18E-26	-0.135149	0.151792	0.3732731	0.305925
ENSG00000143162	CREG1	1	167510959	C	T	0.302187	0.00817162	0.0154509	0.56	-0.275567	0.00845211	3.68E-233	-0.0296539	0.0560769	0.5969393	0.5951318
ENSG00000143164	DCAF6	1	167975051	A	C	0.110338	-0.00533648	0.0250365	0.5500004	0.166415	0.0133944	1.93E-35	-0.0320674	0.150469	0.831236	0.7430098
ENSG00000143167	GPA33	1	167040970	G	C	0.083499	0.00841051	0.02818	0.7199992	-0.267264	0.0163366	3.70E-60	-0.0314689	0.105456	0.7653925	0.5338641
ENSG00000143178	TBX19	1	168266971	A	G	0.333002	0.014217	0.015137	0.4700002	0.235175	0.00820729	1.41E-180	0.0604529	0.0643994	0.3478757	0.2846387

ENSG00000143179	UCK2	1	165838811	A	T	0.455268	0.0165261	0.0142342	0.32	-0.0754355	0.00794911	2.31E-21	-0.219076	0.190101	0.2491481	0.1400721
ENSG00000143183	TMCO1	1	165746512	T	C	0.322068	0.00177963	0.0155569	0.7499995	-0.0892775	0.00928253	6.73E-22	-0.0199337	0.174266	0.9089312	0.4309356
ENSG00000143184	XCL1	1	168548513	T	C	0.17992	-0.0260729	0.0194712	0.2200002	0.536131	0.0106152	0	-0.0486316	0.0363308	0.1807077	0.5659084
ENSG00000143185	XCL2	1	168511619	T	C	0.17992	-0.0260729	0.0194712	0.2200002	-1.104	0.0201296	0	0.0236168	0.0176422	0.1806845	0.4008413
ENSG00000143190	POU2F1	1	167293324	T	A	0.467197	-0.00344448	0.0142553	0.6800001	-0.0523186	0.00800713	6.40E-11	0.0658367	0.272657	0.8091963	0.69869
ENSG00000143198	MGST3	1	165615565	T	C	0.452286	0.0160032	0.0142359	0.3400001	-0.80994	0.00710808	0	-0.0197585	0.0175773	0.2609752	0.2803603
ENSG00000143199	ADCY10	1	167831039	A	G	0.0447316	-0.0375096	0.0361361	0.2300001	0.13078	0.0218717	2.24E-09	-0.286815	0.280445	0.3064435	0.8950425
ENSG00000143207	COP1	1	176045298	T	C	0.131213	-0.0178333	0.0202214	0.3400001	0.203624	0.0110942	3.06E-75	-0.0875794	0.099422	0.3783795	0.8091122
ENSG00000143222	UFC1	1	161125606	T	C	0.00994036	0.0210102	0.0732174	0.9199999	0.698686	0.0659449	3.14E-26	0.030071	0.104831	0.7742263	0.7989519
ENSG00000143224	PPOX	1	161142001	G	C	0.399602	-0.0244076	0.0146328	0.1199999	-0.185975	0.00821103	1.41E-113	0.131241	0.0788947	0.09621215	0.1960123
ENSG00000143226	FCGR2A	1	161484511	G	A	0.220676	0.0028087	0.0178206	0.98	0.125819	0.00988645	4.22E-37	0.0223234	0.141648	0.8747739	0.3304918
ENSG00000143228	NUF2	1	163280960	T	C	0.319085	0.00685282	0.0154049	0.6899999	-0.0805331	0.00858379	6.47E-21	-0.0850932	0.191501	0.6567913	0.3318374
ENSG00000143248	RGS5	1	163134168	G	A	0.182903	0.00252635	0.0184797	0.8	-0.232336	0.0100566	4.34E-118	-0.0108737	0.0795402	0.8912623	0.03039509
ENSG00000143252	SDHC	1	161308515	C	T	0.0487078	0.00485475	0.0302596	0.9199999	-0.967967	0.0289967	2.50E-244	-0.00501541	0.0312614	0.8725386	0.2481337
ENSG00000143257	NR1I3	1	161203774	A	G	0.0506958	0.0298051	0.0337366	0.4	0.132864	0.0209789	2.40E-10	0.224329	0.256378	0.381578	0.07898335
ENSG00000143294	PRCC	1	156745504	G	A	0.340954	-0.0281659	0.0151527	0.08700015	0.055614	0.0084925	5.81E-11	-0.506453	0.283225	0.0737496	0.1081994
ENSG00000143297	FCRL5	1	157502738	T	C	0.238569	0.0273208	0.0173036	0.09499921	0.95377	0.00841914	0	0.0286451	0.0181441	0.1143922	0.0440444
ENSG00000143303	METTL25B	1	156702493	G	C	0.431412	0.0355834	0.0143041	0.009800089	-0.201808	0.00941593	6.64E-102	-0.176323	0.0713556	0.0134718	0.3725616
ENSG00000143314	MRPL24	1	156709238	A	G	0.280318	-0.0223956	0.0157343	0.2200002	-0.160419	0.00878316	1.59E-74	0.139607	0.0983798	0.1558818	0.0699763
ENSG00000143315	PIGM	1	159999622	C	T	0.0725646	-0.00326004	0.0313262	0.8200001	0.810953	0.0160804	0	-0.00402001	0.038629	0.9171159	0.5767078
ENSG00000143318	CASQ1	1	160165980	G	A	0.286282	0.00735092	0.0158172	0.64	0.503131	0.00826679	0	0.0146103	0.0314385	0.6421258	0.9789214
ENSG00000143319	ISG20L2	1	156695137	A	C	0.207753	-0.0234435	0.0175381	0.28	-0.151606	0.00984598	1.69E-53	0.154634	0.116117	0.1829556	0.02004355
ENSG00000143320	CRABP2	1	156672503	T	G	0.0328032	-0.00245737	0.0324719	0.81	-0.47855	0.0195304	1.38E-132	0.00513503	0.067855	0.9396765	0.8772985
ENSG00000143321	HDGF	1	156724308	T	G	0.0636183	0.00374958	0.0302235	0.89	-0.237016	0.0167299	1.46E-45	-0.0158199	0.127521	0.9012702	0.7513183
ENSG00000143322	ABL2	1	179133640	C	T	0.115308	0.0104379	0.0228105	0.6200004	0.184037	0.0126512	6.10E-48	0.0567163	0.124006	0.6474082	0.5181526
ENSG00000143324	XPR1	1	180730263	A	G	0.055666	0.00738364	0.0365205	0.95	-0.14469	0.0183906	3.62E-15	-0.0510307	0.252488	0.8398298	0.6387621
ENSG00000143333	RGS16	1	182570650	T	C	0.150099	0.00202618	0.0210908	0.9400001	0.121576	0.0114994	4.00E-26	0.0166659	0.173485	0.9234686	0.1608033
ENSG00000143337	TOR1AIP1	1	179869794	G	A	0.292247	-0.000908266	0.01556	0.8800001	0.113205	0.0131667	8.13E-18	-0.00802321	0.137453	0.9534535	0.3925416
ENSG00000143344	RGL1	1	183751437	C	G	0.365805	-0.014412	0.0146058	0.3700002	0.262054	0.00818885	1.05E-224	-0.0549963	0.0557623	0.3240043	0.26787
ENSG00000143353	LYPLAL1	1	219366696	C	A	0.494036	0.00993417	0.0142076	0.7600007	-0.330549	0.0115235	5.90E-181	-0.0300535	0.0429946	0.4845473	0.2775057
ENSG00000143363	PRUNE1	1	150994542	G	A	0.122266	-0.00246831	0.0218622	0.64	0.254134	0.0112276	1.97E-113	-0.00971264	0.0860274	0.9101085	0.9610464
ENSG00000143365	RORC	1	151791447	A	G	0.135189	-0.0149014	0.020437	0.35	0.124214	0.0116175	1.11E-26	-0.119965	0.164912	0.4669511	0.5369694
ENSG00000143367	TUFT1	1	151534420	A	G	0.2833	0.0268907	0.0165396	0.04	0.0985835	0.00908888	2.07E-27	0.272771	0.169647	0.1078626	0.3976909
ENSG00000143369	ECM1	1	150483401	T	C	0.0457256	0.020911	0.0289253	0.5199996	0.350827	0.0158649	2.35E-108	0.0596048	0.0824929	0.4699594	0.6034984
ENSG00000143374	TARS2	1	150469982	G	C	0.549702	-0.010728	0.0142255	0.4100001	0.0847873	0.00793369	1.17E-26	-0.126528	0.168196	0.4518899	0.3968962
ENSG00000143375	CGN	1	151497077	C	T	0.132207	-0.0287847	0.0213728	0.07799917	-0.069755	0.0120612	7.32E-09	0.412655	0.314596	0.1896232	0.8411915
ENSG00000143376	SNX27	1	151628054	T	C	0.33499	-0.0037709	0.0153226	0.7400005	0.20491	0.00838237	5.63E-132	-0.0184027	0.0747808	0.8056141	0.3214755
ENSG00000143379	SETDB1	1	150917976	A	G	0.367793	-0.0115694	0.0148593	0.5099998	-0.1242	0.00832696	2.62E-50	0.0931515	0.119803	0.4368415	0.7350164
ENSG00000143382	ADAMTSL4	1	150527648	A	G	0.055666	-0.0414955	0.037235	0.2200002	-0.882856	0.0259084	1.67E-254	0.0470014	0.0421982	0.2653537	0.9981062
ENSG00000143384	MCL1	1	150549549	T	G	0.241551	-0.0109744	0.0171386	0.4	-0.220546	0.00971457	4.22E-114	0.0497601	0.0777407	0.522122	0.7836956
ENSG00000143387	CTSK	1	150774741	G	A	0.487078	-0.00118771	0.0142553	0.95	0.59958	0.00729532	0	-0.0019809	0.0237755	0.9335995	0.6269464
ENSG00000143390	RFX5	1	151316474	A	G	0.205765	0.010474	0.0180571	0.6899999	-0.155084	0.0112118	1.63E-43	-0.0675377	0.116537	0.5622252	0.8390202
ENSG00000143398	PIP5K1A	1	151196218	T	C	0.0228628	0.0208818	0.0391217	0.64	0.424843	0.0383398	1.55E-28	0.0491518	0.0921919	0.5939318	0.3165318
ENSG00000143401	ANP32E	1	150199610	G	A	0.175944	0.00842286	0.0188373	0.5199996	0.217902	0.0109748	1.00E-87	0.0386544	0.0864705	0.6548577	0.05639913
ENSG00000143409	MINDY1	1	150974938	C	T	0.432406	-0.00840142	0.0147291	0.5199996	0.125169	0.00816444	4.75E-53	-0.0671206	0.117755	0.5686766	0.6713072
ENSG00000143412	ANXA9	1	150961301	T	C	0.0139165	0.0270668	0.0563769	0.56	0.438757	0.0396785	2.01E-28	0.0616897	0.128613	0.6314745	0.9491689
ENSG00000143416	SELENBP1	1	151340993	G	T	0.161034	-0.0297154	0.0182821	0.06299992	0.114244	0.0102586	8.34E-29	-0.260104	0.161722	0.1077603	0.8456366
ENSG00000143418	CERS2	1	150940269	G	T	0.372763	-0.00827967	0.0148152	0.6700003	0.218809	0.00964113	4.97E-114	-0.0378397	0.0677288	0.5763707	0.9003813
ENSG00000143420	ENSA	1	150587707	A	G	0.0914513	0.0172512	0.0259602	0.64	-0.128959	0.01345	8.98E-22	-0.133773	0.201789	0.5073724	0.8426456
ENSG00000143434	SEMA6C	1	151111632	T	C	0.194831	0.00351162	0.017023	0.7199992	0.128748	0.00964596	1.23E-40	0.027275	0.132235	0.8365861	0.755763

ENSG00000143436	MRPL9	1	151734079	T	C	0.462227	0.00989142	0.0142388	0.4	0.0728672	0.00798352	7.03E-20	0.135746	0.195973	0.4885121	0.7790633
ENSG00000143437	ARNT	1	150815712	A	T	0.0944334	0.0253421	0.0258113	0.4500005	0.318626	0.0130985	1.06E-130	0.0795355	0.081074	0.3265813	0.6001319
ENSG00000143442	POGZ	1	151403570	A	G	0.146123	-0.0217093	0.0188283	0.1100001	0.287393	0.0102856	8.41E-172	-0.0755387	0.0655698	0.2493071	0.5616465
ENSG00000143443	C1orf56	1	151022339	A	G	0.0228628	-0.0176781	0.0486384	0.6499995	-0.243597	0.0245765	3.70E-23	0.0725711	0.199802	0.7164438	0.6254602
ENSG00000143450	OAZ3	1	151739626	A	G	0.482107	0.0123926	0.0142053	0.2999998	0.100038	0.0118329	2.81E-17	0.12388	0.142754	0.3855122	0.8189503
ENSG00000143452	HORMAD1	1	150681950	G	A	0.408549	-0.0106842	0.0145525	0.5099998	0.250449	0.00798693	7.71E-216	-0.0426601	0.0581215	0.4629597	0.7344545
ENSG00000143457	GOLPH3L	1	150644165	G	T	0.372763	-0.00827967	0.0148152	0.6700003	0.101738	0.00868736	1.12E-31	-0.0813825	0.145787	0.5766888	0.7712565
ENSG00000143458	GABPB2	1	151070536	C	A	0.0795229	-0.0229376	0.0273596	0.56	0.413899	0.0175946	2.31E-122	-0.0554183	0.066144	0.4021189	0.8804075
ENSG00000143466	NA	1	206657007	G	A	0.246521	-0.00716023	0.016343	0.99	-0.473348	0.0136363	5.12E-264	0.0151268	0.0345291	0.661323	0.1806639
ENSG00000143476	DTL	1	212244830	A	C	0.0467197	-0.0706179	0.0364372	0.05499966	-0.138301	0.0217011	1.85E-10	0.510611	0.275377	0.06370671	0.3015036
ENSG00000143486	EIF2D	1	206765262	G	A	0.425447	0.0140241	0.0143184	0.28	0.31331	0.0153716	2.40E-92	0.0447612	0.0457532	0.3279175	0.5808953
ENSG00000143493	INTS7	1	212161312	T	C	0.0228628	0.0458312	0.0466429	0.2200002	0.32508	0.0313179	3.06E-25	0.140984	0.144123	0.3279633	0.3360026
ENSG00000143494	VASH2	1	213144620	T	G	0.256461	-0.0151648	0.0157136	0.25	0.0633958	0.00877766	5.11E-13	-0.239208	0.250068	0.3387829	0.3203475
ENSG00000143498	TAF1A	1	222747259	G	A	0.373757	0.00398466	0.0143683	0.7400005	-0.201072	0.00812775	4.07E-135	-0.0198171	0.0714629	0.7815455	0.6987666
ENSG00000143499	SMYD2	1	214482459	A	G	0.418489	-0.00946292	0.0142407	0.7899998	-0.288652	0.011756	3.96E-133	0.0327831	0.0493532	0.5065271	0.0290072
ENSG00000143502	SUSD4	1	223465852	C	T	0.0805169	-0.0200141	0.0277622	0.5300002	-0.856288	0.0270962	3.49E-219	0.0233731	0.03243	0.4710791	0.5088949
ENSG00000143507	DUSP10	1	221895142	T	C	0.324056	0.00885645	0.0151269	0.6200004	-0.0973091	0.00853499	4.12E-30	-0.0910136	0.155657	0.5587451	0.9605826
ENSG00000143514	TP53BP2	1	224000637	A	G	0.0904573	-0.0178011	0.0241331	0.4100001	-0.283198	0.0142348	4.51E-88	0.0628574	0.0852748	0.4610522	0.2576459
ENSG00000143515	ATP8B2	1	154310906	T	C	0.294235	0.0298282	0.0157797	0.04700023	0.205181	0.00878915	1.56E-120	0.145375	0.077158	0.05954836	0.1625386
ENSG00000143537	ADAM15	1	155029147	T	C	0.192843	-0.0229134	0.017158	0.17	-0.702347	0.00937135	0	0.032624	0.0244334	0.1818032	0.1163959
ENSG00000143545	RAB13	1	153956480	T	C	0.105368	-0.0590124	0.0220926	0.009099971	0.164524	0.0204601	8.90E-16	-0.358686	0.141497	0.01124668	0.9372868
ENSG00000143546	S100A8	1	153363086	A	G	0.105368	0.0218615	0.0222274	0.17	0.300492	0.0128472	5.43E-121	0.0727523	0.0740353	0.3257699	0.6784669
ENSG00000143549	TPM3	1	154147454	C	T	0.382704	0.00841904	0.0145794	0.7700005	0.154307	0.00815567	7.78E-80	0.0545604	0.0945272	0.5638094	0.005982075
ENSG00000143552	NUP210L	1	154046376	G	C	0.0337972	0.0199229	0.0362048	0.4299995	2.05218	0.0498231	0	0.00970817	0.0176437	0.5821587	0.3668817
ENSG00000143553	SNAPIN	1	153632718	A	G	0.39165	-0.0154429	0.0144349	0.2999998	0.176993	0.00874617	4.66E-91	-0.0872516	0.0816703	0.2853677	0.1708861
ENSG00000143554	SLC27A3	1	153749731	A	G	0.0725646	0.00576177	0.0296733	0.8600001	0.558567	0.0164373	4.15E-253	0.0103153	0.0531248	0.8460425	0.7968179
ENSG00000143569	UBAP2L	1	154218320	C	T	0.450298	0.0123777	0.0142536	0.6300007	0.246522	0.00785573	3.65E-216	0.0502093	0.0578409	0.3853621	0.02913418
ENSG00000143570	SLC39A1	1	153935881	C	G	0.284294	-0.0237473	0.0156708	0.06800017	0.2564	0.00857801	2.62E-196	-0.0926182	0.0611971	0.1301675	0.09751386
ENSG00000143575	HAX1	1	154246669	G	A	0.328032	0.0183051	0.0155789	0.1299999	0.253023	0.00871594	2.76E-185	0.0723457	0.0616215	0.2403823	0.365901
ENSG00000143578	CREB3L4	1	153943424	T	C	0.477137	0.013373	0.0142124	0.4	0.160064	0.0078962	2.32E-91	0.0835479	0.0888877	0.3472551	0.001339746
ENSG00000143595	AQP10	1	154295683	G	T	0.0228628	-0.0206773	0.0375956	0.6499995	0.687172	0.0216793	1.70E-220	-0.0300904	0.0547188	0.5823813	0.6012484
ENSG00000143603	KCNN3	1	154761329	G	A	0.5	-0.0104336	0.0147214	0.4299995	0.0575893	0.00901948	1.71E-10	-0.181172	0.257197	0.4811771	0.1212998
ENSG00000143612	C1orf43	1	154186143	A	G	0.384692	0.00538617	0.0145626	0.91	0.137958	0.00815143	2.97E-64	0.0390421	0.105583	0.7115502	0.02819956
ENSG00000143614	GATAD2B	1	153836326	T	C	0.187873	0.0426012	0.0185433	0.01099993	-0.0947254	0.0153511	6.80E-10	-0.449734	0.208886	0.03131788	0.01828774
ENSG00000143621	ILF2	1	153639018	A	T	0.442346	-0.00814254	0.0142438	0.8	0.0888604	0.00797269	7.52E-29	-0.0916329	0.160505	0.5680643	0.0364108
ENSG00000143622	RIT1	1	155874397	C	T	0.237575	0.0215374	0.0163098	0.1199999	-0.333059	0.0134353	1.15E-135	-0.0646654	0.0490391	0.1872863	0.2485481
ENSG00000143624	INTS3	1	153723549	C	T	0.426441	-0.0225535	0.0142981	0.14	0.0704455	0.0080751	2.69E-18	-0.320155	0.206258	0.1206125	0.7484227
ENSG00000143630	HCN3	1	155253506	G	T	0.263419	0.00217173	0.0163405	0.7400005	-0.127112	0.0151184	4.18E-17	-0.0170851	0.128568	0.8942818	0.6709427
ENSG00000143633	C1orf131	1	231368221	T	C	0.0974155	0.0643359	0.0212729	0.004499974	-0.189579	0.01211	3.08E-55	-0.339362	0.114286	0.002983733	0.2466045
ENSG00000143641	GALNT2	1	230305703	T	G	0.284294	-0.00955591	0.0158825	0.3599996	0.0724289	0.00882784	2.31E-16	-0.131935	0.219873	0.5484719	0.9513255
ENSG00000143643	TTC13	1	231078305	T	C	0.332008	-0.0267226	0.0153215	0.07100027	-0.217141	0.00852744	4.97E-143	0.123066	0.0707254	0.0818507	0.1021381
ENSG00000143653	SCCPDH	1	246909394	A	G	0.363817	-0.000709208	0.0147808	0.7800007	-0.307397	0.00811169	0	0.00230714	0.0480837	0.9617309	0.7819112
ENSG00000143669	LYST	1	235935640	G	A	0.188867	0.0155178	0.0186514	0.4500005	0.107403	0.0105566	2.59E-24	0.144482	0.174237	0.4069782	0.6633271
ENSG00000143674	MAP3K21	1	233492204	C	T	0.054672	0.0388496	0.0317815	0.17	0.570692	0.0194739	8.85E-189	0.0680746	0.0557379	0.2219593	0.4390274
ENSG00000143702	CEP170	1	243353190	G	T	0.354871	0.000774652	0.0151572	0.7300002	0.331368	0.0134128	9.36E-135	0.00233774	0.0457413	0.9592397	0.528478
ENSG00000143727	ACP1	2	271211	G	T	0.488072	-0.00369882	0.0142103	0.81	-0.116267	0.00794708	1.80E-48	0.0318131	0.12224	0.7946707	0.9282805
ENSG00000143740	SNAP47	1	227942583	T	G	0.107356	0.0201339	0.0230851	0.33	0.230739	0.0141433	7.80E-60	0.0872583	0.100191	0.3837998	0.8077595
ENSG00000143742	SRP9	1	225971849	A	G	0.215706	-0.0169316	0.0165523	0.3599996	-0.15348	0.0153471	1.51E-23	0.110318	0.10841	0.3088653	0.6331207
ENSG00000143748	NVL	1	224466562	T	C	0.0308151	0.00661711	0.0306446	0.9400001	-0.663543	0.0179311	9.48E-300	-0.0099724	0.0461841	0.8290446	0.9430119
ENSG00000143751	SDE2	1	226178717	C	G	0.105368	0.00347755	0.022554	0.8499999	-0.152733	0.0133882	3.81E-30	-0.0227688	0.147683	0.8774728	0.1218063

ENSG00000143753	DEGS1	1	224372300	G	A	0.0785288	−0.00567503	0.0272142	0.7499995	0.945859	0.0146204	0	−0.00599987	0.0287721	0.8348145	0.794146
ENSG00000143756	FBXO28	1	224325769	A	G	0.426441	0.0151908	0.0144416	0.2700001	0.51195	0.00763147	0	0.0296724	0.0282125	0.2929149	0.9719985
ENSG00000143761	ARF1	1	228278636	G	A	0.430417	0.00105625	0.0142148	0.7300002	0.058313	0.00803209	3.87E-13	0.0181135	0.24378	0.9407697	0.5350505
ENSG00000143771	CNIH4	1	224555856	C	G	0.249503	0.0227693	0.0172591	0.09699961	−0.462171	0.00937608	0	−0.0492659	0.0373569	0.1872389	0.2577901
ENSG00000143772	ITPKB	1	226873207	A	G	0.463221	0.00280025	0.014604	0.95	−0.120728	0.00816103	1.62E-49	−0.0231947	0.120976	0.8479542	0.8662597
ENSG00000143774	GUK1	1	228332174	A	G	0.374751	−0.0235693	0.0146327	0.09699961	0.0891012	0.0122351	3.28E-13	−0.264523	0.168195	0.1157842	0.2306773
ENSG00000143776	CDC42BPA	1	227341870	T	C	0.134195	−0.000485591	0.0201578	0.9400001	−0.157848	0.0120104	1.88E-39	0.00307632	0.127704	0.9807813	0.6690119
ENSG00000143797	MBOAT2	2	9068381	C	A	0.238569	0.0273913	0.0171717	0.1100001	−0.15617	0.00981299	5.02E-57	−0.175394	0.110506	0.1124698	0.1847881
ENSG00000143799	PARP1	1	226572086	C	T	0.16998	−0.0052943	0.0190344	0.7199992	0.447748	0.0103679	0	−0.0118243	0.0425123	0.7809064	0.2330341
ENSG00000143801	PSEN2	1	227070845	A	C	0.506958	−0.000666875	0.0142002	0.9599999	−0.141729	0.00792622	1.66E-71	0.00470529	0.100193	0.9625434	0.6783887
ENSG00000143811	PYCR2	1	226109778	C	T	0.0626243	0.0650622	0.0305902	0.0329997	0.640337	0.0189449	1.97E-250	0.101606	0.0478665	0.03377913	0.29535
ENSG00000143815	LBR	1	225602915	C	T	0.125249	−0.0251094	0.0212637	0.29	−0.0939804	0.0129048	3.27E-13	0.267177	0.229212	0.2437626	0.7550388
ENSG00000143819	EPHX1	1	226015527	T	C	0.111332	−0.0083817	0.0225451	0.7400005	−0.159197	0.0128003	1.65E-35	0.0526499	0.141681	0.7101841	0.4158416
ENSG00000143842	SOX13	1	204069553	A	G	0.146123	0.00721099	0.0211033	0.6600001	0.072648	0.0123287	3.80E-09	0.0992593	0.290975	0.7330083	0.9003929
ENSG00000143847	PPFIA4	1	203021747	T	C	0.441352	0.0030726	0.0142732	0.7199992	−0.656288	0.0077768	0	−0.00468179	0.0217485	0.8295569	0.4698622
ENSG00000143850	PLEKHA6	1	204268571	T	C	0.466203	−0.0350986	0.0141826	0.01	−0.148006	0.0117861	3.61E-36	0.237144	0.0976679	0.01517968	0.314309
ENSG00000143851	PTPN7	1	202123428	A	G	0.180915	0.0114696	0.0189179	0.4500005	−0.437891	0.0113115	0	−0.0261928	0.0432076	0.5443762	0.4567536
ENSG00000143862	ARL8A	1	202108200	A	G	0.0616302	0.0342856	0.0285349	0.2700001	0.230712	0.026304	1.77E-18	0.148608	0.124837	0.2338836	0.8061388
ENSG00000143869	GDF7	2	20869921	G	T	0.435388	−0.0010379	0.0142417	0.7099994	−0.124091	0.00939	7.17E-40	0.00836404	0.11477	0.9419045	NA
ENSG00000143870	PDIA6	2	10950810	T	C	0.473161	−0.0118938	0.0141951	0.28	−0.328031	0.0115463	1.52E-177	0.0362582	0.0432925	0.4023023	0.8625839
ENSG00000143878	RHOB	2	20648017	T	C	0.238569	−0.01942	0.0172316	0.2	−0.132398	0.00964961	7.64E-43	0.146678	0.130588	0.2613455	0.4914436
ENSG00000143882	ATP6V1C2	2	10893505	C	T	0.470179	0.00932956	0.0142263	0.3599996	0.0826842	0.00876301	3.89E-21	0.112834	0.172471	0.5129711	0.8938233
ENSG00000143889	HNRNPLL	2	38809924	A	G	0.116302	0.0121595	0.0223467	0.6499995	−0.305725	0.0136122	1.03E-111	−0.0397726	0.0731155	0.5864622	0.4388004
ENSG00000143891	GALM	2	38930715	A	G	0.412525	−0.0166685	0.0147821	0.32	−0.257699	0.00882924	2.84E-187	0.0646821	0.0574047	0.2598384	0.8811417
ENSG00000143919	CAMKMT	2	44794410	T	C	0.357853	−0.00188393	0.0150623	0.99	0.249006	0.00928871	2.65E-158	−0.0075658	0.0604903	0.9004646	0.2942026
ENSG00000143924	EML4	2	42478089	G	C	0.373757	0.0079298	0.0146176	0.5999997	−0.470086	0.00775775	0	−0.0168688	0.0310968	0.5875008	0.6901889
ENSG00000143933	CALM2	2	47338208	T	C	0.0924453	−0.00168264	0.0262512	0.9299999	−0.191574	0.0126528	8.71E-52	0.00878325	0.13703	0.9488929	0.9716992
ENSG00000143942	CHAC2	2	53998631	C	T	0.0318091	−0.017441	0.0467841	0.7800007	0.296257	0.0279911	3.54E-26	−0.0588711	0.158015	0.7094711	0.1698541
ENSG00000143951	WDPCP	2	63701747	T	C	0.296223	0.0118873	0.0154365	0.2999998	0.252869	0.00975477	3.70E-148	0.0470097	0.0610724	0.4414554	0.4293621
ENSG00000143952	VPS54	2	64183120	C	T	0.0198807	−0.0140496	0.0500479	0.95	−0.446939	0.0396399	1.74E-29	0.0314351	0.112014	0.7789895	0.4787321
ENSG00000143970	ASXL2	2	26030971	T	C	0.0904573	−0.0516225	0.0229533	0.0129999	0.131248	0.0131942	2.59E-23	−0.393321	0.179299	0.02826023	0.08265452
ENSG00000143971	ETAA1	2	67631064	A	G	0.0994036	0.00127699	0.023469	0.7600007	−0.158718	0.0135021	6.66E-32	−0.00804567	0.147868	0.9566076	0.9261713
ENSG00000143994	ABHD1	2	27350181	A	C	0.340954	−0.0146775	0.0148524	0.35	0.0876895	0.00845542	3.37E-25	−0.16738	0.170142	0.3252296	0.867915
ENSG00000143995	MEIS1	2	66730792	C	T	0.295229	−0.00289484	0.0157115	0.7700005	0.250324	0.0132943	4.34E-79	−0.0115644	0.0627677	0.8538244	NA
ENSG00000144021	CIAO1	2	96935478	T	A	0.332008	0.021191	0.0150847	0.05499966	0.0946092	0.00893365	3.31E-26	0.223985	0.160839	0.1637402	0.6411742
ENSG00000144026	ZNF514	2	95822116	A	G	0.420477	0.0015149	0.0142382	0.6300007	−0.554099	0.00745998	0	−0.00273399	0.0256962	0.9152676	0.2944444
ENSG00000144034	TPRKB	2	73960379	T	A	0.310139	−0.00275261	0.0149205	0.7199992	−0.11032	0.00895515	7.14E-35	0.0249511	0.135262	0.8536491	0.5809617
ENSG00000144040	SFXN5	2	73235956	C	G	0.0586481	−0.0304557	0.0294758	0.2200002	−0.321061	0.0169811	9.99E-80	0.0948595	0.0919444	0.3022104	0.4450252
ENSG00000144043	TEX261	2	71217571	A	G	0.228628	−0.00761125	0.0173809	0.5999997	0.171933	0.0101405	1.77E-64	−0.0442688	0.101125	0.6615582	0.681257
ENSG00000144048	DUSP11	2	73998297	T	C	0.166004	−0.0115897	0.0187333	0.5400003	−0.0777599	0.0108129	6.41E-13	0.149045	0.241802	0.5376361	0.7295697
ENSG00000144115	THNSL2	2	88477990	C	T	0.140159	0.00241199	0.020169	0.98	0.916423	0.00954825	0	0.00263196	0.0220084	0.9048088	0.2177325
ENSG00000144118	RALB	2	121024964	A	G	0.292247	0.0138017	0.0153523	0.3100002	−0.444212	0.0081092	0	−0.0310701	0.0345654	0.3687178	0.4247473
ENSG00000144120	TMEM177	2	120440413	G	A	0.181909	0.0180381	0.018396	0.2200002	−0.188036	0.0105574	5.83E-71	−0.0959291	0.0979806	0.3275493	0.7413677
ENSG00000144130	NT5DC4	2	113489759	G	A	0.5	−0.0115367	0.0142023	0.58	−0.142602	0.0109302	6.64E-39	0.0809015	0.0997869	0.4175138	0.8583851
ENSG00000144134	RABL2A	2	114392889	C	G	0.372763	0.0350858	0.0145296	0.006199976	0.368221	0.0189196	2.29E-84	0.0952845	0.0397614	0.01655675	0.6707314
ENSG00000144136	SLC20A1	2	113412419	G	A	0.5	−0.0115367	0.0142023	0.58	−0.500099	0.00753122	0	0.0230689	0.0284011	0.4166474	0.7825766
ENSG00000144152	FBLN7	2	112920876	T	C	0.0208748	−0.00828894	0.047158	0.83	−0.360585	0.0281936	1.87E-37	0.0229875	0.130794	0.860488	0.04647499
ENSG00000144161	ZC3H8	2	112992983	A	G	0.213718	0.000139913	0.0175527	0.7300002	0.192215	0.0097938	9.24E-86	0.000727898	0.091318	0.9936401	0.2737558
ENSG00000144182	LIPT1	2	99775519	T	C	0.367793	0.00855682	0.0147935	0.6200004	−0.505952	0.00771805	0	−0.0169123	0.0292401	0.5629974	0.9028968
ENSG00000144199	FAHD2B	2	97754969	A	C	0.105368	0.0121444	0.0241333	0.4700002	−0.551868	0.0324999	1.14E-64	−0.022006	0.0437494	0.6149637	0.6986864

ENSG00000144214	LYG1	2	99910953	T	C	0.445328	0.0130877	0.0143134	0.3599996	0.403536	0.0115548	3.26E-267	0.0324326	0.0354821	0.3606886	0.6168731
ENSG00000144218	AFF3	2	100461459	T	C	0.493042	-0.0112116	0.0141924	0.3700002	0.314384	0.00847193	2.02E-301	-0.0356621	0.0451537	0.4296486	0.4435338
ENSG00000144224	UBXN4	2	136520907	T	C	0.0298211	-0.00686471	0.0578	0.5199996	-0.170489	0.025024	9.56E-12	0.0402649	0.339077	0.9054746	0.6491776
ENSG00000144230	GPR17	2	128406826	G	A	0.526839	0.000865732	0.0143012	1	0.0812553	0.0097522	7.95E-17	0.0106545	0.176008	0.9517303	0.5656509
ENSG00000144231	POLR2D	2	128609785	C	T	0.33002	-0.0201839	0.0152465	0.2399999	0.24068	0.00833093	1.59E-183	-0.0838618	0.063414	0.1860182	0.509742
ENSG00000144233	AMMECR1L	2	128631350	A	G	0.0218688	-0.0701774	0.0488206	0.09299937	0.241873	0.0317069	2.38E-14	-0.290141	0.205396	0.157775	0.819547
ENSG00000144283	PKP4	2	159426433	C	T	0.492048	-0.00518318	0.0142243	0.64	0.355149	0.00841029	0	-0.0145944	0.0400532	0.7155776	0.6635226
ENSG00000144306	SCRN3	2	175277380	A	G	0.49503	-0.00334337	0.0142952	0.7700005	0.111946	0.00807681	1.10E-43	-0.0298659	0.127715	0.8151035	0.5628197
ENSG00000144320	LNPK	2	176828093	A	G	0.406561	0.0326867	0.0143269	0.01499996	-0.206419	0.0079632	3.80E-148	-0.158351	0.0696752	0.02304393	0.5683524
ENSG00000144354	CDCA7	2	174226636	C	G	0.054672	-0.00572239	0.0298224	0.64	0.315716	0.0152653	5.03E-95	-0.0181251	0.0944636	0.8478409	0.1297174
ENSG00000144357	UBR3	2	170812304	C	T	0.244533	-0.0173874	0.0164168	0.3900004	-0.18361	0.00910521	1.97E-90	0.0946974	0.0895344	0.2902084	0.2829656
ENSG00000144362	PHOSPHO2	2	170554596	C	T	0.28827	-0.0136947	0.0153972	0.3800004	-0.502765	0.00822875	0	0.0272387	0.0306283	0.3738238	0.08431274
ENSG00000144401	METTL21A	2	208468003	A	G	0.166998	0.00261578	0.0184673	0.9699999	-0.840692	0.0112615	0	-0.00311146	0.0219668	0.8873615	0.0136911
ENSG00000144407	PTH2R	2	209471832	G	A	0.441352	0.0138636	0.0144447	0.4500005	0.0724078	0.00814559	6.16E-19	0.191465	0.20065	0.3399698	0.5942286
ENSG00000144445	KANSL1L	2	210961127	T	C	0.311133	-0.0141679	0.0148631	0.3700002	-0.0620653	0.0103667	2.14E-09	0.228274	0.242492	0.3465156	0.9156811
ENSG00000144455	SUMF1	3	4125731	T	C	0.512922	-0.00496736	0.0142124	0.59	-0.508194	0.00741491	0	0.00977454	0.0279669	0.7267107	0.5035483
ENSG00000144468	RHBDD1	2	227782114	T	C	0.0248509	0.0297087	0.0637682	0.58	-0.326517	0.0359236	9.98E-20	-0.0909868	0.195555	0.6417345	0.4334953
ENSG00000144476	ACKR3	2	237483715	G	A	0.0755467	0.0173439	0.0283591	0.4400003	-0.312689	0.0177287	1.27E-69	-0.0554669	0.0907488	0.5410578	0.8945365
ENSG00000144485	HES6	2	239148105	G	A	0.33499	-0.0104928	0.0149961	0.4199997	0.273172	0.0082779	8.07E-239	-0.0384109	0.0549085	0.4842117	0.06786998
ENSG00000144488	ESPNL	2	239025363	T	C	0.426441	0.0349467	0.0142702	0.004600023	-0.143899	0.00804626	1.57E-71	-0.242855	0.100093	0.01525422	0.4510049
ENSG00000144504	ANKMY1	2	241463732	A	T	0.178926	0.0389223	0.0195853	0.064	0.608282	0.0104657	0	0.0639873	0.0322166	0.0470154	0.585718
ENSG00000144524	COPS7B	2	232660172	G	A	0.261431	-0.0100176	0.0169643	0.4199997	-0.0636385	0.00999128	1.90E-10	0.157414	0.267716	0.5565396	0.1305276
ENSG00000144535	DIS3L2	2	233017507	A	G	0.0636183	0.0153529	0.0295743	0.5999997	0.11977	0.0170686	2.27E-12	0.128186	0.2476	0.6046572	0.7646143
ENSG00000144550	CPNE9	3	9758541	C	T	0.250497	-0.011961	0.0167454	0.5700002	-0.106299	0.0157485	1.48E-11	0.112522	0.158411	0.477506	0.5353197
ENSG00000144554	FANCD2	3	10105856	T	C	0.201789	0.0232933	0.0168578	0.16	-0.378564	0.0094703	0	-0.0615307	0.0445575	0.1673013	0.7499615
ENSG00000144559	TAMM41	3	11860154	T	G	0.415507	-0.0199665	0.0143897	0.2099999	-0.62215	0.014094	0	0.0320928	0.0231404	0.1654814	0.4008517
ENSG00000144560	VGLL4	3	11679882	G	C	0.306163	-0.0107047	0.0157719	0.5099998	0.15077	0.0125984	5.27E-33	-0.0710004	0.104777	0.4980058	0.2412452
ENSG00000144566	RAB5A	3	20007619	C	T	0.431412	-0.0211971	0.0147194	0.08400014	-0.196083	0.00812995	1.60E-128	0.108103	0.0752008	0.1505706	0.4043441
ENSG00000144567	RETREG2	2	220045574	C	T	0.0477137	0.0108542	0.0369808	0.6200004	0.302654	0.0194148	8.67E-55	0.0358634	0.12221	0.7691728	0.5926034
ENSG00000144579	CTDSP1	2	219266821	C	T	0.383698	-0.000104481	0.0145166	0.9400001	0.0701271	0.00955098	2.10E-13	-0.00148988	0.207004	0.9942574	0.84548
ENSG00000144580	CNOT9	2	219447553	T	C	0.378728	0.00216358	0.0145104	0.7099994	-0.753686	0.0156516	0	-0.00287066	0.0192527	0.881471	0.2709637
ENSG00000144589	STK11IP	2	220471877	A	G	0.0387674	0.0123424	0.0450091	0.8700001	-0.189957	0.0296771	1.55E-10	-0.0649747	0.237161	0.7841086	0.05213374
ENSG00000144591	GMPPA	2	220367649	T	C	0.0586481	-0.00671914	0.026674	0.8600001	0.102886	0.0165193	4.72E-10	-0.0653068	0.25947	0.8012786	0.1824486
ENSG00000144597	EAF1	3	15476491	C	G	0.187873	0.0303504	0.0182267	0.05600025	-0.162073	0.0118719	1.97E-42	-0.187264	0.113294	0.09834892	0.8243313
ENSG00000144635	DYNC1LI1	3	32589914	A	C	0.382704	-0.0159346	0.0148548	0.33	0.783448	0.0120025	0	-0.0203391	0.0189634	0.2834751	0.1432443
ENSG00000144645	OSBPL10	3	31909227	A	G	0.028827	0.0511829	0.0452543	0.2099999	-0.454961	0.0256799	3.12E-70	-0.1125	0.0996711	0.2590207	0.7965719
ENSG00000144647	POMGNT2	3	43134146	C	T	0.464215	0.0031462	0.0142187	0.98	-0.120746	0.00882184	1.21E-42	-0.0260563	0.117772	0.8249032	0.1414225
ENSG00000144648	ACKR2	3	42897920	T	C	0.398608	0.00671895	0.014648	0.58	-0.0879719	0.00977705	2.30E-19	-0.0763761	0.166724	0.6468812	0.07836284
ENSG00000144655	CSRNP1	3	39189699	A	G	0.198807	-0.0114157	0.0176645	0.3100002	0.135103	0.00983682	6.32E-43	-0.0844964	0.130893	0.5185794	0.7757903
ENSG00000144659	SLC25A38	3	39431840	T	A	0.157058	-0.0251097	0.0193772	0.2399999	0.301923	0.0249239	8.93E-34	-0.0831658	0.0645454	0.1975767	0.1003426
ENSG00000144668	ITGA9	3	37679305	G	A	0.464215	0.0179038	0.0144374	0.3900004	0.195077	0.00796721	2.13E-132	0.091778	0.0741035	0.2155266	0.5200614
ENSG00000144671	SLC22A14	3	38341925	A	G	0.354871	0.0289642	0.0152151	0.08600031	-0.0636295	0.00925871	6.31E-12	-0.4552	0.248124	0.06656984	0.7016007
ENSG00000144674	GOLGA4	3	37346455	G	A	0.358847	-0.0189951	0.0145298	0.1499999	0.138753	0.00819039	2.24E-64	-0.136898	0.105028	0.1924229	0.02225446
ENSG00000144677	CTDSPL	3	37964705	A	T	0.157058	-0.0303532	0.0191065	0.08799946	0.0715183	0.010792	3.43E-11	-0.424412	0.274724	0.1223793	0.8354049
ENSG00000144681	STAC	3	36505667	T	C	0.247515	-0.00213236	0.0165402	0.9400001	0.0971588	0.00938007	3.85E-25	-0.0219472	0.170252	0.897429	0.3915133
ENSG00000144711	IQSEC1	3	13026668	T	C	0.198807	0.0152808	0.01713	0.33	-0.223654	0.0114182	1.97E-85	-0.0683233	0.0766708	0.3728619	0.6073241
ENSG00000144712	CAND2	3	12875693	T	C	0.0656064	0.0336867	0.0288876	0.16	0.749838	0.0178066	0	0.0449253	0.0385399	0.2437425	0.4348428
ENSG00000144713	RPL32	3	12879535	A	G	0.0914513	0.00650183	0.022984	0.6800001	0.153234	0.0129109	1.72E-32	0.0424306	0.150035	0.7773265	0.2378453
ENSG00000144724	PTPRG	3	61915265	A	G	0.218688	0.0242071	0.0177477	0.2	0.109448	0.0195856	2.29E-08	0.221174	0.166916	0.1851518	0.7629776
ENSG00000144736	SHQ1	3	72854746	T	C	0.232604	0.00419647	0.0165774	0.7700005	0.0971317	0.00961015	5.13E-24	0.0432039	0.170723	0.8002182	0.5006759

ENSG00000144741	SLC25A26	3	66278907	G	T	0.402584	-0.0057071	0.0150224	0.5099998	-0.139033	0.00903949	2.21E-53	0.0410486	0.108082	0.7041012	0.04026722
ENSG00000144744	UBA3	3	69116720	T	C	0.398608	0.00920184	0.0143724	0.4500005	0.1149	0.0119813	8.81E-22	0.0800854	0.125364	0.5229389	0.6099282
ENSG00000144746	ARL6IP5	3	69144656	A	G	0.37674	-0.0186784	0.0146962	0.33	0.168231	0.00815205	1.29E-94	-0.111029	0.087523	0.2045965	0.1468972
ENSG00000144747	TMF1	3	69085231	C	T	0.17495	0.0102041	0.018621	0.6300007	0.114903	0.0105613	1.44E-27	0.0888062	0.162264	0.5841761	0.931493
ENSG00000144749	LRIG1	3	66490454	C	A	0.28827	-0.0280962	0.015743	0.1199999	0.32023	0.00864897	4.50E-300	-0.0877377	0.0492187	0.07464993	0.308711
ENSG00000144791	LIMD1	3	45662358	A	C	0.241551	0.0308454	0.0159627	0.0129999	0.226663	0.00925066	1.39E-132	0.136085	0.0706434	0.0540593	0.3883088
ENSG00000144792	ZNF660	3	44630450	G	A	0.319085	0.0114482	0.0152785	0.5199996	-0.0944994	0.00950775	2.81E-23	-0.121146	0.162137	0.4549543	0.577677
ENSG00000144802	NFKBIZ	3	101563350	T	C	0.427435	0.00783601	0.0142128	0.3100002	-0.217608	0.00798368	1.39E-163	-0.0360097	0.0653271	0.5814815	0.3191204
ENSG00000144815	NXPE3	3	101522559	A	G	0.0685885	-0.0711269	0.0286277	0.008500021	-0.705606	0.0163098	0	0.100803	0.0406386	0.01312126	0.1454928
ENSG00000144820	ADGRG7	3	100371378	G	A	0.0149105	-0.00315437	0.0785009	0.9199999	1.99935	0.0372995	0	-0.0015777	0.0392632	0.9679476	0.06131928
ENSG00000144821	MYH15	3	108173692	C	T	0.309145	-0.0265971	0.0154274	0.05	0.0637744	0.00979939	7.62E-11	-0.41705	0.25025	0.09560727	0.9227509
ENSG00000144824	PHLDB2	3	111573354	C	T	0.429423	0.00553678	0.014429	0.7499995	-0.0765449	0.00872068	1.67E-18	-0.0723337	0.188684	0.7014531	0.2843977
ENSG00000144827	ABHD10	3	111705033	A	C	0.217694	-0.0183848	0.0175347	0.35	0.219249	0.0108913	3.98E-90	-0.0838536	0.0800847	0.2950711	0.5767355
ENSG00000144840	RABL3	3	120433684	G	C	0.338966	0.00701778	0.0150567	0.4100001	0.0930031	0.00924834	8.63E-24	0.0754575	0.162068	0.6415087	0.428446
ENSG00000144843	ADPRH	3	119303453	T	G	0.0318091	0.0489693	0.0349364	0.2	-0.791968	0.0213071	2.15E-302	-0.0618324	0.0441447	0.1613115	0.3842804
ENSG00000144847	IGSF11	3	118742159	C	T	0.0337972	0.0471985	0.036303	0.1499999	0.469409	0.0197304	4.12E-125	0.100549	0.0774531	0.1942221	0.3198188
ENSG00000144848	ATG3	3	112266124	A	G	0.0487078	-0.0209002	0.0292268	0.6300007	-0.183772	0.0190441	4.93E-22	0.113729	0.159475	0.4757539	0.7512065
ENSG00000144852	NR1I2	3	119518331	G	A	0.266402	-0.0124342	0.0157388	0.3599996	-0.0516881	0.00879438	4.17E-09	0.240562	0.307234	0.4336314	0.5993477
ENSG00000144867	SRPRB	3	133523746	T	C	0.171968	-0.0391659	0.0201385	0.05	-0.540882	0.010643	0	0.0724111	0.0372599	0.0519673	0.7646037
ENSG00000144893	MED12L	3	150979172	G	A	0.102386	-0.00243582	0.0269216	0.7400005	-0.124742	0.0141508	1.19E-18	0.0195268	0.215829	0.9279109	0.3796402
ENSG00000144895	EIF2A	3	150283247	C	T	0.143141	0.0314304	0.0200469	0.1100001	-0.527585	0.0109073	0	-0.0595741	0.0380174	0.1171099	0.5494149
ENSG00000144909	OSBPL11	3	125280818	A	G	0.297217	0.0183748	0.016019	0.2999998	0.154319	0.00879263	5.84E-69	0.11907	0.104026	0.2523662	0.3567728
ENSG00000144935	TRPC1	3	142484823	G	C	0.4334	-0.0163048	0.014404	0.3100002	-0.108836	0.00804333	1.02E-41	0.149811	0.132808	0.2593102	0.3381431
ENSG00000144959	NCEH1	3	172388523	C	T	0.0477137	0.0036647	0.0402127	0.6999999	-0.475786	0.0220482	2.80E-103	-0.00770241	0.0845192	0.9273876	0.7562659
ENSG00000145002	FAM86B2	8	12288414	A	G	0.0487078	-0.00875949	0.0342362	0.9199999	0.42202	0.0553381	2.42E-14	-0.0207561	0.0811703	0.7981742	0.8216733
ENSG00000145012	LPP	3	188239766	T	C	0.299205	0.0181571	0.0150775	0.25	0.28069	0.00843139	5.19E-243	0.0646873	0.0537509	0.2287967	0.03191749
ENSG00000145014	TMEM44	3	194331410	T	G	0.309145	0.0122961	0.0153565	0.3900004	-0.263439	0.00929105	7.45E-177	-0.0466753	0.0583156	0.4234845	0.9323774
ENSG00000145016	RUBCN	3	197437431	T	C	0.102386	0.0455688	0.024464	0.03599979	0.225469	0.0161114	1.69E-44	0.202107	0.10946	0.06483354	0.4990354
ENSG00000145020	AMT	3	49457198	T	C	0.23161	0.0289561	0.0162122	0.1199999	-0.499935	0.00863175	0	-0.0579198	0.0324441	0.07422566	0.01120695
ENSG00000145022	TCTA	3	49451773	T	A	0.23161	0.0289987	0.0162097	0.1199999	-0.0992181	0.00905358	6.02E-28	-0.292272	0.165537	0.07746268	0.02195998
ENSG00000145029	NICN1	3	49463569	G	A	0.232604	0.0294357	0.0162236	0.1100001	0.384393	0.00942833	0	0.0765771	0.0422476	0.06989642	0.02239208
ENSG00000145050	MANF	3	51424653	G	A	0.0198807	-0.129809	0.0553659	0.01099993	0.165339	0.023497	1.97E-12	-0.785108	0.352962	0.02612543	0.2154899
ENSG00000145088	EAF2	3	121579701	A	G	0.382704	-0.00101214	0.0147228	0.9199999	0.290693	0.00805901	6.58E-285	-0.00348182	0.0506474	0.9451916	0.9186368
ENSG00000145103	ILDR1	3	121723610	C	T	0.0785288	0.00995281	0.0274639	0.7499995	0.286935	0.0149484	4.07E-82	0.0346867	0.0957319	0.7171043	0.9071008
ENSG00000145107	TM4SF19	3	196054163	C	T	0.26839	6.00E-05	0.0159776	0.8499999	0.19973	0.00960648	5.20E-96	0.000300541	0.0799959	0.9970024	0.008874307
ENSG00000145113	MUC4	3	195506392	C	T	0.127237	0.0304242	0.0202909	0.1199999	0.252839	0.0129745	1.40E-84	0.12033	0.0804895	0.134919	0.01872224
ENSG00000145191	EIF2B5	3	184127686	A	G	0.553678	-0.0126428	0.0141883	0.33	-0.0880671	0.00835013	5.26E-26	0.143559	0.161682	0.3745898	0.3203514
ENSG00000145214	DGKQ	4	966679	A	G	0.44831	0.00165399	0.0142148	0.7899998	0.801478	0.00771382	0	0.00206368	0.0177357	0.9073698	0.9200046
ENSG00000145216	FIP1L1	4	54702624	T	C	0.378728	-0.0291331	0.0148942	0.03500016	-0.0647007	0.00914316	1.48E-12	0.450275	0.238834	0.05938849	0.9225233
ENSG00000145217	SLC26A1	4	980044	C	G	0.356859	-0.0227928	0.0150758	0.1199999	0.191119	0.00948208	2.39E-90	-0.11926	0.0791034	0.1316455	0.2614988
ENSG00000145220	LYAR	4	4280662	C	T	0.263419	-0.00737688	0.0153662	0.6499995	0.335894	0.00945977	3.75E-276	-0.0219619	0.0457514	0.6312075	0.2930304
ENSG00000145241	CENPC	4	68374422	T	G	0.0884692	0.00585523	0.022664	0.7600007	-0.932194	0.0239164	0	-0.00628113	0.0243131	0.7961417	0.8635481
ENSG00000145244	CORIN	4	47718069	A	G	0.327038	-0.0209426	0.0152776	0.17	0.431206	0.00812816	0	-0.0485675	0.0354417	0.1705786	0.2754188
ENSG00000145246	ATP10D	4	47541404	A	G	0.372763	0.0036149	0.0147024	0.7199992	-0.190088	0.00831887	1.45E-115	-0.019017	0.0773497	0.8057928	0.8524617
ENSG00000145247	OCIAD2	4	48897995	G	A	0.374751	-0.0273369	0.0148298	0.06100002	0.10631	0.00832222	2.28E-37	-0.257144	0.140941	0.06807988	0.9141477
ENSG00000145284	SCD5	4	83635351	T	C	0.210736	-0.00217956	0.0171566	0.7700005	0.802439	0.00890596	0	-0.00271617	0.0213806	0.8989095	0.4050001
ENSG00000145287	PLAC8	4	84034714	G	C	0.49006	-0.0135983	0.0144373	0.3599996	0.157545	0.00791697	4.10E-88	-0.086314	0.091742	0.3467903	0.3398637
ENSG00000145293	ENOPH1	4	83366979	T	C	0.104374	-0.0162024	0.0243483	0.3700002	0.162216	0.0127096	2.63E-37	-0.0998819	0.150302	0.5063447	0.1508111
ENSG00000145331	TRMT10A	4	100476527	G	A	0.0367793	0.0348135	0.0355136	0.2099999	-0.264796	0.025663	5.83E-25	-0.131473	0.134721	0.3291179	0.5913167
ENSG00000145332	KLHL8	4	88111507	A	C	0.114314	-0.00805355	0.0223146	0.7499995	0.229837	0.0125955	2.16E-74	-0.0350403	0.0971078	0.7182199	0.03007326

ENSG00000145335	SNCA	4	90702358	T	C	0.198807	0.0349697	0.0175306	0.03699985	0.272547	0.0100245	8.96E-163	0.128307	0.0644942	0.04665342	0.282669
ENSG00000145348	TBCK	4	107104063	T	G	0.178926	-0.00642026	0.0186357	0.8600001	0.361714	0.010247	6.02E-273	-0.0177496	0.0515231	0.7304721	0.600987
ENSG00000145349	CAMK2D	4	114527635	G	A	0.083499	-0.00137121	0.0239503	0.98	0.346027	0.0134813	2.72E-145	-0.00396273	0.0692154	0.9543443	0.3187128
ENSG00000145354	CISD2	4	103800267	G	T	0.492048	0.0101945	0.0141981	0.5999997	-0.193889	0.00840571	1.01E-117	-0.0525789	0.0732633	0.4729602	0.9456241
ENSG00000145358	DDIT4L	4	101109483	A	G	0.12326	-0.0146675	0.0219912	0.4	0.162775	0.0125142	1.11E-38	-0.0901093	0.13528	0.5053496	0.9105988
ENSG00000145365	TIFA	4	113201921	C	G	0.255467	0.0339741	0.016209	0.04499974	0.131222	0.0104251	2.48E-36	0.258905	0.125224	0.03868391	0.04052764
ENSG00000145375	AFG2A	4	124040019	C	T	0.142147	0.0141862	0.0198865	0.4700002	0.0721475	0.0113112	1.79E-10	0.196628	0.277355	0.4783627	0.7541905
ENSG00000145386	CCNA2	4	122741343	T	A	0.357853	-0.0048079	0.0145183	0.8200001	-0.0962961	0.00822537	1.17E-31	0.0499283	0.150828	0.7406226	0.1881795
ENSG00000145388	METTL14	4	119620164	G	A	0.428429	0.0185624	0.014352	0.1199999	-0.137832	0.00802814	4.56E-66	-0.134674	0.104422	0.1971506	0.5627634
ENSG00000145390	USP53	4	120175207	G	C	0.127237	-0.00716489	0.021488	0.6899999	0.903742	0.0108339	0	-0.00792802	0.0237769	0.7388066	0.2247931
ENSG00000145414	NAF1	4	164059649	A	G	0.203777	-0.027368	0.0181981	0.1299999	0.105006	0.0102291	1.01E-24	-0.260632	0.175155	0.1367483	0.8297799
ENSG00000145416	MARCHF1	4	164875326	T	G	0.460239	0.0067678	0.0142216	0.5300002	-0.374229	0.00772216	0	-0.0180846	0.0380042	0.634175	0.436787
ENSG00000145423	SFRP2	4	154706008	T	G	0.293241	0.026724	0.0156594	0.09099971	0.164477	0.00865732	1.75E-80	0.162479	0.0955905	0.08918075	0.4139707
ENSG00000145425	RPS3A	4	152023264	T	C	0.0248509	-0.0556493	0.0357162	0.1499999	-0.33864	0.0240095	3.57E-45	0.164332	0.106111	0.1214596	0.4788716
ENSG00000145428	RNF175	4	154656332	G	A	0.465209	0.00857421	0.0142647	0.59	-0.280253	0.00781804	2.05E-281	-0.0305945	0.0509065	0.5478437	0.3093061
ENSG00000145431	PDGFC	4	157787076	T	C	0.355865	0.00155209	0.0152089	0.95	-0.182585	0.00838329	3.62E-105	-0.00850066	0.0832987	0.9187167	0.4726743
ENSG00000145439	CBR4	4	169858173	C	T	0.128231	0.0119243	0.0218528	0.4	0.581447	0.0123433	0	0.020508	0.037586	0.5853217	0.4622483
ENSG00000145476	CYP4V2	4	187123642	T	C	0.17992	0.0393324	0.0188536	0.02399993	0.671856	0.0101752	0	0.0585429	0.028076	0.03705473	0.01121313
ENSG00000145491	ROPN1L	5	10456888	A	G	0.185885	-0.00232319	0.0180072	0.8800001	0.0862507	0.0108781	2.21E-15	-0.0269353	0.208805	0.8973597	0.9878448
ENSG00000145494	NDUFS6	5	1809116	A	G	0.12326	-0.0102753	0.0214109	0.5500004	-0.1029	0.01754	4.45E-09	0.0998568	0.208769	0.6324281	0.1738508
ENSG00000145495	MARCHF6	5	10394653	A	G	0.0884692	-0.00569959	0.0228849	0.8700001	-0.690931	0.0120544	0	0.00824915	0.0331222	0.8033204	0.9146586
ENSG00000145545	SRD5A1	5	6651565	T	C	0.121272	-0.0411033	0.0214488	0.08199927	0.432936	0.0163902	9.36E-154	-0.0949408	0.0496729	0.05596327	0.6218519
ENSG00000145555	MYO10	5	16800883	G	T	0.414513	-0.0111262	0.0144486	0.5	-0.211136	0.00810078	9.43E-150	0.052697	0.0684627	0.4414672	0.1621856
ENSG00000145569	OTULINL	5	14598500	G	A	0.164016	-0.0139788	0.019705	0.5300002	-0.235458	0.0106841	1.24E-107	0.0593687	0.0837315	0.4783019	0.09210661
ENSG00000145592	RPL37	5	40830400	A	G	0.285288	-0.00656308	0.0158579	0.5500004	-0.100713	0.0104206	4.25E-22	0.0651662	0.157601	0.6792482	0.2294187
ENSG00000145604	SKP2	5	36168256	C	T	0.122266	-0.0356999	0.0222373	0.089	0.441363	0.0121589	1.64E-288	-0.0808856	0.0504325	0.1087497	0.6968708
ENSG00000145632	PLK2	5	57752948	A	G	0.350895	-0.00916022	0.0150992	0.4500005	-0.0662334	0.00894634	1.33E-13	0.138302	0.228734	0.5454165	0.6185969
ENSG00000145649	GZMA	5	54402278	T	C	0.055666	0.0690512	0.0302669	0.01400006	0.254658	0.0170522	1.98E-50	0.271153	0.120232	0.02411792	0.1018611
ENSG00000145675	PIK3R1	5	67554598	G	A	0.208748	0.00703787	0.0176102	0.6499995	0.150184	0.00966795	2.04E-54	0.0468617	0.117296	0.6895136	0.8999068
ENSG00000145685	LHFPL2	5	77923441	C	A	0.280318	-0.015033	0.0155514	0.28	-0.184605	0.00854654	1.80E-103	0.0814333	0.0843257	0.3341949	0.454019
ENSG00000145687	SSBP2	5	80878228	C	T	0.284294	-0.00682807	0.0151711	0.7700005	-0.141987	0.00865553	1.78E-60	0.0480894	0.106889	0.6527814	0.2540212
ENSG00000145703	IQGAP2	5	75851515	A	G	0.511928	0.00342716	0.0142077	0.7400005	0.639945	0.00708879	0	0.00535539	0.0222015	0.8093864	0.2513694
ENSG00000145715	RASA1	5	86625726	C	A	0.245527	-0.00691981	0.0166557	0.4500005	0.102356	0.00917219	6.44E-29	-0.0676052	0.162836	0.6780143	0.005087153
ENSG00000145723	GIN1	5	102438779	A	G	0.271372	-0.0168469	0.0154755	0.2999998	-0.169104	0.00937291	9.15E-73	0.0996244	0.091681	0.2771954	0.6501826
ENSG00000145725	PPIP5K2	5	102497395	G	A	0.280318	-0.0102347	0.0152566	0.5300002	-0.801992	0.0109053	0	0.0127616	0.0190242	0.5023418	0.6320762
ENSG00000145730	PAM	5	102228247	C	T	0.331014	0.00265713	0.0147227	0.6499995	1.0317	0.00598452	0	0.0025755	0.0142704	0.8567771	0.05341715
ENSG00000145734	BDP1	5	70807545	A	G	0.299205	0.0257928	0.0154607	0.1199999	0.121267	0.0159498	2.89E-14	0.212695	0.130527	0.1032041	0.09911584
ENSG00000145736	GTF2H2	5	70347150	G	A	0.385686	-0.01351	0.0151406	0.25	0.594181	0.0211191	3.68E-174	-0.0227372	0.0254943	0.372471	0.1455807
ENSG00000145741	BTF3	5	72797846	G	A	0.287276	-0.021732	0.0160837	0.2099999	0.0860099	0.00930723	2.44E-20	-0.252669	0.188986	0.1812337	0.08193092
ENSG00000145743	FBXL17	5	107456267	A	G	0.143141	-0.0180148	0.0191546	0.4799997	-0.223095	0.0109488	2.72E-92	0.0807493	0.0859498	0.3474769	0.7509231
ENSG00000145779	TNFAIP8	5	118667340	C	T	0.218688	0.0230556	0.0169613	0.33	-0.163284	0.00923865	6.65E-70	-0.141199	0.104183	0.1753208	0.1100152
ENSG00000145780	FEM1C	5	114868599	A	G	0.437376	0.0199418	0.0143255	0.1499999	-0.383534	0.00769327	0	-0.0519948	0.0373659	0.1640718	0.1452322
ENSG00000145781	COMMD10	5	115584573	A	T	0.0994036	-0.0363757	0.0233636	0.06699926	0.493307	0.0122023	0	-0.0737385	0.0473963	0.1197591	0.8557039
ENSG00000145782	ATG12	5	115170724	C	T	0.454274	-0.0107556	0.01446	0.4400003	0.333593	0.00860287	0	-0.0322417	0.0433542	0.4570697	0.1156561
ENSG00000145817	YIPF5	5	143544000	T	G	0.172962	-0.00506674	0.0189062	0.4899999	-0.709679	0.0096582	0	0.00713948	0.0266407	0.7887056	0.371661
ENSG00000145819	ARHGAP26	5	142379262	A	T	0.0924453	-0.0412994	0.0261768	0.0990011	0.292855	0.0141796	9.14E-95	-0.141024	0.0896454	0.1156895	0.968997
ENSG00000145833	DDX46	5	134142646	G	T	0.028827	-0.0031046	0.0435629	0.9199999	0.201218	0.0259952	9.89E-15	-0.015429	0.216505	0.9431876	0.7958921
ENSG00000145850	TIMD4	5	156368279	G	T	0.474155	-0.0331917	0.0142538	0.009299937	-0.130662	0.00794852	1.01E-60	0.254026	0.110178	0.0211328	0.2034731
ENSG00000145860	RNF145	5	158610739	A	G	0.4334	-0.0269048	0.0142748	0.05899973	-0.141667	0.00798474	1.98E-70	0.189916	0.10133	0.0608987	0.3322
ENSG00000145868	FBXO38	5	147792948	T	G	0.395626	-0.000876574	0.0142945	0.9599999	-0.359332	0.00846696	0	0.00243945	0.0397808	0.9511024	0.8846052

ENSG00000145882	PCYOX1L	5	148743393	A	G	0.494036	-0.00358078	0.0141831	0.7700005	0.519437	0.00739237	0	-0.00689358	0.0273049	0.8006805	0.1485532
ENSG00000145901	TNIP1	5	150441322	A	G	0.227634	0.0197086	0.0163526	0.1800002	-0.254073	0.00932633	2.04E-163	-0.0775707	0.0644249	0.2285704	0.2499146
ENSG00000145907	G3BP1	5	151171476	G	A	0.397614	-0.000502656	0.0146398	0.99	-0.0685318	0.00820858	6.90E-17	0.00733464	0.213622	0.9726103	0.9559666
ENSG00000145908	ZNF300	5	150279249	G	A	0.149105	0.0142775	0.0218443	0.58	-0.133244	0.0115589	9.60E-31	-0.107153	0.164206	0.5140433	0.3528842
ENSG00000145916	RMND5B	5	177567781	C	A	0.028827	0.0947145	0.0451451	0.0519996	-0.448461	0.0252233	1.02E-70	-0.211199	0.101365	0.03720147	0.2307161
ENSG00000145919	BOD1	5	173039090	T	C	0.0705765	0.0143536	0.0298089	0.5700002	-0.189094	0.0172179	4.64E-28	-0.0759071	0.157792	0.630475	0.6830343
ENSG00000145920	CPLX2	5	175267168	A	G	0.21173	0.0105325	0.0208085	0.6499995	0.168862	0.0175641	6.98E-22	0.0623736	0.123399	0.6132335	0.901644
ENSG00000145936	KCNMB1	5	169810924	C	T	0.284294	0.00253581	0.0157658	0.95	0.296492	0.0084776	5.67E-268	0.0085527	0.053175	0.8722188	0.3806775
ENSG00000145945	FAM50B	6	3850585	A	C	0.115308	0.00802633	0.023016	0.7499995	-0.459415	0.0129621	3.72E-275	-0.0174708	0.0501009	0.727306	0.7758486
ENSG00000145949	MYLK4	6	2707531	T	C	0.149105	0.0154935	0.0189971	0.5199996	0.548764	0.0187203	6.90E-189	0.0282335	0.0346314	0.4149256	0.9756761
ENSG00000145979	TBC1D7	6	13301928	T	G	0.217694	-0.0133895	0.0166989	0.35	0.466548	0.00976888	0	-0.0286991	0.0357975	0.4227229	0.6999936
ENSG00000145982	FARS2	6	5516545	A	G	0.252485	-0.0301586	0.0164113	0.03400008	-0.323711	0.0133807	2.67E-129	0.0931651	0.0508434	0.06689275	0.9217397
ENSG00000145990	GFOD1	6	13422924	T	C	0.427435	-0.00749477	0.0142876	0.5700002	0.0725513	0.00868691	6.72E-17	-0.103303	0.197319	0.6006034	0.3488267
ENSG00000145996	CDKAL1	6	20883661	C	T	0.285288	-0.0113039	0.015958	0.3400001	-0.263125	0.00876383	4.77E-198	0.0429601	0.0606648	0.4788483	0.1731393
ENSG00000146007	ZMAT2	5	140082256	T	C	0.239563	-0.00965999	0.0170043	0.4700002	0.279929	0.00917197	1.41E-204	-0.0345087	0.0607555	0.57004	0.9578296
ENSG00000146021	KLHL3	5	137012484	G	A	0.37674	0.0234045	0.014517	0.064	0.224314	0.00804462	4.21E-171	0.104338	0.0648254	0.1075016	0.6965637
ENSG00000146054	TRIM7	5	180626608	C	T	0.257455	-0.0235788	0.0164605	0.1499999	-0.213223	0.00912732	1.07E-120	0.110583	0.0773434	0.1527853	0.5786389
ENSG00000146063	TRIM41	5	180656154	T	C	0.171968	0.000392351	0.0186945	0.98	-0.175623	0.0105332	2.05E-62	-0.00223405	0.106447	0.9832556	0.3515436
ENSG00000146066	HIGD2A	5	175816260	C	G	0.0904573	0.014983	0.0246252	0.6800001	-1.02116	0.0137507	0	-0.0146726	0.0241158	0.5429081	0.6590032
ENSG00000146067	FAM193B	5	176964165	A	G	0.368787	0.0101286	0.0151361	0.5099998	0.234759	0.00958073	1.37E-132	0.0431448	0.0644992	0.5035471	0.9765177
ENSG00000146070	PLA2G7	6	46687684	C	T	0.0705765	-0.0486412	0.0301211	0.05399953	-0.588766	0.0304877	4.29E-83	0.0826155	0.0513383	0.1075643	0.1389226
ENSG00000146072	TNFRSF21	6	47238454	A	G	0.107356	-0.00651726	0.0239092	0.9599999	-0.0898128	0.0130873	6.76E-12	0.0725649	0.266421	0.7853383	0.7701501
ENSG00000146083	RNF44	5	175959362	T	C	0.49006	-0.000507219	0.0145621	0.8	0.230329	0.00805093	5.19E-180	-0.00220215	0.0632231	0.9722142	0.6908356
ENSG00000146085	MMUT	6	49414488	G	A	0.333002	-0.0142195	0.0146567	0.3400001	0.652402	0.00810233	0	-0.0217956	0.0224674	0.3319965	0.01781305
ENSG00000146094	DOK3	5	176933591	A	G	0.0497018	0.0119432	0.0334984	0.8499999	0.288078	0.0182619	4.64E-56	0.0414583	0.116312	0.7215115	0.5261689
ENSG00000146109	ABT1	6	26598729	A	G	0.0318091	0.0177086	0.0463686	0.9199999	-0.607813	0.0306376	1.38E-87	-0.0291349	0.0763017	0.7025811	0.6761465
ENSG00000146122	DAAM2	6	39816395	G	T	0.100398	-0.00984257	0.0230827	0.5	0.186709	0.0126603	3.19E-49	-0.0527162	0.123681	0.6699428	0.6915701
ENSG00000146143	PRIM2	6	57346489	G	A	0.115308	0.0460236	0.0227846	0.04300015	0.111281	0.0196395	1.46E-08	0.413579	0.217369	0.05708514	0.6953869
ENSG00000146166	LGSN	6	64007869	C	T	0.442346	-0.00172322	0.0142212	0.8800001	-0.40094	0.00769388	0	0.00429795	0.0354698	0.9035546	0.7739703
ENSG00000146192	FGD2	6	36985134	C	G	0.498012	-0.0207548	0.0141728	0.1900002	0.463278	0.00754693	0	-0.0447999	0.0306011	0.1431955	0.5834391
ENSG00000146215	CRIP3	6	43271991	A	G	0.423459	-0.0142205	0.0145067	0.25	0.179379	0.008168	6.77E-107	-0.0792763	0.0809523	0.3274337	0.1352886
ENSG00000146221	TCTE1	6	44255969	C	T	0.447316	0.0169083	0.0142687	0.16	0.0645368	0.00828287	6.62E-15	0.261995	0.223636	0.2413894	0.6658356
ENSG00000146223	RPL7L1	6	42852509	A	G	0.26839	-0.00716983	0.0167431	0.5099998	-0.352165	0.0172544	1.36E-92	0.0203593	0.0475538	0.6685552	0.2883942
ENSG00000146232	NFKBIE	6	44229701	T	C	0.0367793	-0.0241064	0.0387166	0.4400003	-0.573007	0.0217995	2.82E-152	0.04207	0.0675863	0.5336377	0.612049
ENSG00000146242	TPBG	6	83076734	A	G	0.0159046	0.053927	0.0461427	0.2	1.04152	0.0348584	3.72E-196	0.051777	0.0443369	0.2428844	0.07396369
ENSG00000146243	IRAK1BP1	6	79592754	C	T	0.10835	-0.0731009	0.0224446	0.002900013	0.483664	0.0143849	7.71E-248	-0.15114	0.0466225	0.001187879	0.5575667
ENSG00000146247	PHIP	6	79719108	G	A	0.412525	0.0212687	0.0144997	0.1499999	-0.25323	0.00801813	6.48E-219	-0.0839896	0.0573207	0.1428506	0.06269764
ENSG00000146250	PRSS35	6	84228808	G	A	0.365805	-0.00602928	0.0149677	0.5300002	0.0632012	0.00832932	3.25E-14	-0.0953982	0.23716	0.6874979	0.614608
ENSG00000146263	MMS22L	6	97660565	A	G	0.247515	-0.025003	0.0161494	0.17	-0.153228	0.0107758	6.92E-46	0.163176	0.106018	0.1237714	0.7364874
ENSG00000146278	PNRC1	6	89792674	T	C	0.324056	-0.00273195	0.0152244	0.91	0.295883	0.00834597	2.68E-275	-0.00923321	0.0514548	0.8575897	0.07691153
ENSG00000146281	PM20D2	6	89865526	T	C	0.434394	-0.0101667	0.0143862	0.4600002	-0.0964147	0.00872011	2.04E-28	0.105448	0.149516	0.4806491	0.1898095
ENSG00000146282	RARS2	6	88261908	A	G	0.389662	0.0055069	0.0144936	0.5	-0.150381	0.00809844	5.70E-77	-0.0366195	0.0963991	0.7040387	0.5725221
ENSG00000146285	SCML4	6	108085414	A	G	0.150099	-0.00585346	0.0193212	0.91	-0.412386	0.0110048	2.50E-307	0.0141941	0.0468538	0.7619315	0.8857595
ENSG00000146350	TBC1D32	6	121528265	G	A	0.0298211	-0.00647363	0.0414531	0.7700005	-0.160952	0.0247618	8.03E-11	0.040221	0.257625	0.8759364	0.8930716
ENSG00000146373	RNF217	6	125348735	G	A	0.163022	-0.0152762	0.0194593	0.5	0.232114	0.0113307	2.91E-93	-0.0658134	0.0838967	0.4327717	0.814735
ENSG00000146376	ARHGAP18	6	129964323	G	A	0.28827	0.0109978	0.0165772	0.7099994	-0.104698	0.00907356	8.41E-31	-0.105043	0.158595	0.5077563	0.3579554
ENSG00000146386	ABRACL	6	139357129	A	G	0.294235	-0.0114108	0.0158261	0.29	-0.546373	0.00910626	0	0.0208846	0.0289678	0.4709345	0.4581283
ENSG00000146409	SLC18B1	6	133105105	G	T	0.0755467	0.00371678	0.0276292	0.91	-1.24138	0.0160666	0	-0.00299406	0.0222568	0.8929887	0.2560237
ENSG00000146414	SHPRH	6	146235470	G	T	0.40159	0.00133441	0.0145112	0.9599999	0.13271	0.00858456	6.54E-54	0.010055	0.109347	0.9267333	0.4834568
ENSG00000146416	AIG1	6	143521537	T	C	0.112326	0.0103788	0.0233635	0.58	0.337358	0.0122505	6.13E-167	0.030765	0.0692634	0.6569171	0.4145831

ENSG00000146425	DYNLT1	6	159061638	G	C	0.249503	0.0429381	0.0159382	0.01400006	-0.207927	0.00937866	6.66E-109	-0.206506	0.0772168	0.007487108	0.8055496
ENSG00000146426	TIAM2	6	155366344	T	C	0.124254	0.0293932	0.0219506	0.07900053	-0.699273	0.0124513	0	-0.0420339	0.0313995	0.1806746	0.2093028
ENSG00000146433	TMEM181	6	159006964	G	C	0.0745527	0.00120786	0.0237575	0.98	0.137722	0.0154083	3.96E-19	0.00877027	0.172506	0.9594527	0.8478038
ENSG00000146453	PNLDC1	6	160231518	T	C	0.0347913	-0.0975038	0.0458105	0.02199986	0.323332	0.0249882	2.70E-38	-0.301559	0.143587	0.03571188	0.2884108
ENSG00000146457	WTAP	6	160161984	G	A	0.027833	-0.0215182	0.0452054	0.4799997	0.838896	0.0256213	3.92E-235	-0.0256506	0.0538925	0.6341034	0.7248927
ENSG00000146463	ZMYM4	1	35811113	T	G	0.117296	-0.0459854	0.024491	0.05	-0.148718	0.0206776	6.38E-13	0.309213	0.170201	0.06925479	0.2588984
ENSG00000146476	ARMT1	6	151782329	T	G	0.0506958	-0.0387951	0.0315821	0.1499999	0.392792	0.0170159	6.73E-118	-0.0987675	0.0805179	0.2199529	0.6779269
ENSG00000146477	SLC22A3	6	160822657	C	T	0.0168986	-0.0670436	0.0529192	0.2200002	-0.321301	0.0379941	2.75E-17	0.208663	0.166541	0.2102338	0.9999789
ENSG00000146530	VWDE	7	12407039	C	T	0.0974155	0.0330016	0.0269218	0.29	0.762746	0.0158189	0	0.0432668	0.0353073	0.220411	0.3433251
ENSG00000146535	GNA12	7	2825852	C	G	0.111332	-0.00278589	0.0212962	0.6899999	-0.388704	0.0119066	9.07E-234	0.00716712	0.0547881	0.8959215	0.8135876
ENSG00000146540	C7orf50	7	1107259	A	C	0.430417	-0.0168287	0.0143315	0.25	0.177624	0.00801895	1.03E-108	-0.0947431	0.0807975	0.2409566	0.1707422
ENSG00000146555	SDK1	7	3824856	G	T	0.215706	0.00235287	0.0168611	0.95	0.135732	0.00923501	6.68E-49	0.0173346	0.124229	0.889025	0.2082748
ENSG00000146556	NA	2	114349159	G	C	0.372763	-0.0246328	0.0148208	0.0659994	-0.711242	0.0163411	0	0.0346335	0.0208531	0.09674718	0.4630003
ENSG00000146574	CCZ1B	7	6851420	A	G	0.451292	0.014071	0.0143176	0.3700002	-0.788919	0.0161061	0	-0.0178358	0.018152	0.3258148	0.5497696
ENSG00000146576	INTS15	7	6639002	G	A	0.0318091	-0.00187519	0.0320883	0.95	-0.489741	0.0213807	4.07E-116	0.00382894	0.0655212	0.9533996	0.08087328
ENSG00000146587	RBAK	7	5068101	T	C	0.353877	0.018073	0.0153846	0.3700002	0.261042	0.00960222	9.61E-163	0.0692341	0.0589903	0.2405349	0.2986692
ENSG00000146592	CREB5	7	28602225	T	G	0.301193	-0.010373	0.0157155	0.4700002	-0.527993	0.00828896	0	0.0196461	0.0297662	0.5092451	0.4467956
ENSG00000146666	LINC00525	7	47803722	G	A	0.477137	0.0169509	0.0143788	0.28	0.194235	0.00899736	2.33E-103	0.0872701	0.0741381	0.2391446	0.7083508
ENSG00000146670	CDCA5	11	64842704	T	C	0.363817	0.032424	0.0148581	0.032	-0.0453131	0.0081976	3.25E-08	-0.715555	0.352527	0.04237804	0.8512031
ENSG00000146674	IGFBP3	7	45956711	A	T	0.331014	-0.0126192	0.0152163	0.4	0.278741	0.00838064	1.46E-242	-0.0452722	0.0546064	0.4070685	0.8690275
ENSG00000146676	PURB	7	44920428	C	T	0.460239	0.00785223	0.0141863	0.6499995	0.0562318	0.00801274	2.25E-12	0.13964	0.253066	0.5810893	0.1763217
ENSG00000146700	SSC4D	7	76028831	C	T	0.421471	0.00391527	0.0143132	0.9699999	0.108721	0.00800973	5.74E-42	0.0360121	0.131678	0.7844788	0.2610462
ENSG00000146701	MDH2	7	75687097	A	G	0.054672	-0.00858027	0.0359722	0.6300007	-0.203246	0.0321246	2.50E-10	0.0422162	0.177114	0.8116052	0.8398749
ENSG00000146707	POMZP3	7	76247940	G	A	0.300199	-0.0142696	0.0150922	0.3599996	0.836707	0.00783027	0	-0.0170545	0.0180383	0.3444251	0.5118759
ENSG00000146729	NIPSNAP2	7	56043680	G	C	0.295229	0.0116613	0.0156016	0.58	0.121686	0.00850618	2.02E-46	0.095831	0.128387	0.4554111	0.6236168
ENSG00000146731	CCT6A	7	56125502	G	A	0.351889	-0.00595913	0.0153396	0.5700002	0.255742	0.00843216	4.70E-202	-0.0233013	0.0599856	0.6976843	0.1196469
ENSG00000146733	PSPH	7	56099020	C	T	0.234592	-0.0177457	0.0186827	0.33	0.198929	0.0110918	6.31E-72	-0.0892064	0.0940482	0.3428661	0.8710476
ENSG00000146757	ZNF92	7	64852375	C	A	0.0526839	-0.0243985	0.0303478	0.3400001	0.139015	0.016123	6.57E-18	-0.175509	0.219252	0.4234267	0.7836437
ENSG00000146776	ATXN7L1	7	105381282	T	C	0.17992	-0.0159802	0.0182648	0.32	0.0650976	0.0110018	3.28E-09	-0.245481	0.283626	0.3867604	0.6333039
ENSG00000146802	TMEM168	7	112418182	A	G	0.0238569	-0.0736835	0.0482417	0.14	0.177739	0.0292027	1.15E-09	-0.414559	0.279834	0.1384877	0.3317302
ENSG00000146826	TRAPPC14	7	99754190	A	G	0.162028	0.00578438	0.0183056	0.64	-0.327964	0.00999385	3.41E-236	-0.0176372	0.0558185	0.7520215	0.09593645
ENSG00000146828	SLC12A9	7	100444536	T	C	0.236581	-0.0116308	0.0168047	0.4100001	-0.174253	0.00925842	5.08E-79	0.0667467	0.0965038	0.4891582	0.8911782
ENSG00000146830	GIGYF1	7	100282100	A	G	0.111332	-0.0203738	0.0223842	0.4100001	0.320764	0.0124524	2.54E-146	-0.0635165	0.0698276	0.3630235	0.9333419
ENSG00000146833	TRIM4	7	99495902	A	C	0.466203	0.0202962	0.0144032	0.1100001	-0.690266	0.00715596	0	-0.0294034	0.0208684	0.1588367	0.08889334
ENSG00000146834	MEPCE	7	100029077	G	C	0.192843	0.0365566	0.0178665	0.04499974	-0.301183	0.0100063	4.95E-199	-0.121377	0.059458	0.04121245	0.4226328
ENSG00000146842	TMEM209	7	129826082	A	C	0.0755467	0.00360654	0.0284015	0.95	-0.683864	0.0154477	0	-0.00527377	0.0415311	0.8989534	0.4652663
ENSG00000146858	ZC3HAV1L	7	138715613	C	T	0.496024	0.0198211	0.0142127	0.06699926	-0.0819947	0.00803839	1.97E-24	-0.241736	0.174949	0.1670483	0.2081305
ENSG00000146859	TMEM140	7	134841737	C	A	0.517893	-0.0231976	0.014183	0.16	0.55392	0.00732824	0	-0.041879	0.0256108	0.1020052	0.1855347
ENSG00000146904	EPHA1	7	143096683	A	G	0.0795229	0.0279967	0.0237383	0.2300001	-0.764418	0.0160447	0	-0.0366248	0.0310636	0.2383869	0.2699957
ENSG00000146909	NOM1	7	156754146	T	C	0.241551	0.0226233	0.0162683	0.09200046	0.257823	0.00976071	9.36E-154	0.0877473	0.063186	0.1649198	0.5805424
ENSG00000146918	NCAPG2	7	158460761	T	C	0.0606362	-0.0291532	0.0268802	0.2700001	0.528685	0.0155466	1.79E-253	-0.0551429	0.0508694	0.2783607	0.6356263
ENSG00000146955	RAB19	7	140114946	T	C	0.193837	0.0165953	0.0178968	0.4	0.0589108	0.0104415	1.68E-08	0.281702	0.307871	0.3601906	0.2674607
ENSG00000146963	LUC7L2	7	139066651	C	G	0.276342	0.00397052	0.0168384	0.9199999	-0.0588055	0.0105299	2.34E-08	-0.0675195	0.286596	0.8137496	0.4471719
ENSG00000147316	MCPH1	8	6382628	C	T	0.219682	-0.0143705	0.0168614	0.4400003	0.155889	0.00937296	4.10E-62	-0.0921841	0.108305	0.3946831	0.7220507
ENSG00000147324	MFHAS1	8	8696009	C	A	0.414513	0.0231958	0.0143773	0.17	0.225044	0.00788625	4.15E-179	0.103072	0.0639885	0.1072254	0.08447825
ENSG00000147364	FBXO25	8	388826	T	G	0.296223	0.024981	0.0159584	0.1199999	0.124458	0.00910045	1.41E-42	0.200719	0.129061	0.1198918	0.8327029
ENSG00000147408	CSGALNACT1	8	19438606	A	G	0.450298	0.0217173	0.0142623	0.0990011	-0.589335	0.00728289	0	-0.0368505	0.024205	0.1278996	0.3166178
ENSG00000147416	ATP6V1B2	8	20069604	T	C	0.214712	-0.000803216	0.0173095	0.89	-0.0551833	0.00951371	6.62E-09	0.0145554	0.313683	0.9629901	0.9659047
ENSG00000147419	CCDC25	8	27610502	C	G	0.470179	0.00935888	0.0142334	0.5700002	0.309936	0.00776919	0	0.0301961	0.0459299	0.5108979	0.08425844
ENSG00000147421	HMBOX1	8	28835096	G	C	0.44831	0.0107404	0.0144523	0.59	0.0538267	0.00809667	2.97E-11	0.199537	0.270169	0.4601732	0.8556899

ENSG00000147437	GNRH1	8	25279473	T	C	0.364811	-0.000713688	0.0147506	0.6899999	-0.086812	0.0082601	7.79E-26	0.00822107	0.169916	0.9614109	0.146515
ENSG00000147439	BIN3	8	22502296	C	T	0.371769	-0.00102823	0.0147735	0.9400001	0.308284	0.00804159	0	-0.00333533	0.0479218	0.9445124	0.1399781
ENSG00000147443	DOK2	8	21768877	T	C	0.428429	-0.0237345	0.014369	0.09800089	-0.403772	0.00775712	0	0.0587819	0.0356048	0.09874796	0.8068215
ENSG00000147454	SLC25A37	8	23409647	A	G	0.28827	0.00905814	0.0159129	0.6300007	-0.49277	0.00829328	0	-0.0183821	0.0322942	0.5692161	0.6399832
ENSG00000147457	CHMP7	8	23110331	G	A	0.370775	-0.00558642	0.0146146	0.6700003	0.108371	0.00818016	4.63E-40	-0.0515491	0.134914	0.7023944	0.629304
ENSG00000147459	DOCK5	8	25158918	G	A	0.0904573	0.0242791	0.0242124	0.4100001	0.404791	0.0134441	3.67E-199	0.0599793	0.0598477	0.3162474	0.09835916
ENSG00000147471	PLPBP	8	37628697	T	C	0.130219	0.0117129	0.0201585	0.4799997	0.46864	0.012363	0	0.0249934	0.04302	0.5612593	0.6131973
ENSG00000147485	PXDNL	8	52477071	C	A	0.11332	0.0191452	0.0216457	0.3900004	0.0651894	0.0119011	4.31E-08	0.293686	0.336344	0.3825701	0.1227493
ENSG00000147526	TACC1	8	38648125	G	A	0.359841	-0.0112365	0.0147029	0.2599998	-0.354347	0.0080766	0	0.0317104	0.0414992	0.4447951	0.7386895
ENSG00000147533	GOLGA7	8	41358207	T	C	0.289264	-0.0330195	0.0158949	0.064	0.208963	0.00917931	1.03E-114	-0.158016	0.0763815	0.0385678	0.2950334
ENSG00000147535	PLPP5	8	38123704	C	T	0.237575	-0.00205338	0.0168612	0.95	0.0599025	0.00976916	8.69E-10	-0.0342787	0.281533	0.9030912	0.1923271
ENSG00000147548	NSD3	8	38186167	A	C	0.242545	-0.000430831	0.0167672	0.9599999	-0.187799	0.00966023	3.51E-84	0.00229411	0.0892829	0.9795007	0.118343
ENSG00000147570	DNAJC5B	8	66973273	G	A	0.16501	-0.0196273	0.0202513	0.28	-0.117376	0.0169589	4.48E-12	0.167218	0.174217	0.3371446	0.8800514
ENSG00000147576	ADHFE1	8	67363128	G	A	0.489066	-0.0130383	0.0142058	0.5199996	0.578693	0.0072722	0	-0.0225306	0.0245497	0.3587476	0.1466576
ENSG00000147592	LACTB2	8	71564481	C	A	0.0944334	0.0307155	0.0240157	0.1800002	0.601863	0.0141921	0	0.0510341	0.0399204	0.2011103	0.1386882
ENSG00000147601	TERF1	8	73940728	C	G	0.342942	-0.0067624	0.0152576	0.7700005	0.0753094	0.012844	4.53E-09	-0.0897949	0.203177	0.6585222	0.674423
ENSG00000147642	SYBU	8	110645113	A	T	0.206759	-0.0212455	0.0174074	0.2099999	0.0618884	0.00976219	2.30E-10	-0.343287	0.286436	0.2307306	0.7753646
ENSG00000147649	MTDH	8	98698702	A	G	0.027833	0.00685087	0.0418745	0.9599999	0.299301	0.0230967	2.10E-38	0.0228896	0.139919	0.8700525	0.1019428
ENSG00000147650	LRP12	8	105551355	C	G	0.365805	0.0150321	0.0148116	0.2999998	-0.174344	0.00822104	8.23E-100	-0.0862211	0.0850536	0.3107131	0.506592
ENSG00000147654	EBAG9	8	110565082	T	A	0.294235	-0.00312479	0.0153234	0.9299999	0.149451	0.00858815	7.97E-68	-0.0209085	0.102538	0.8384248	0.1212492
ENSG00000147677	EIF3H	8	117716766	A	C	0.205765	0.0114024	0.0175295	0.59	-0.114225	0.0140429	4.15E-16	-0.0998243	0.153955	0.5167268	0.254232
ENSG00000147679	UTP23	8	117820222	C	T	0.112326	-0.000750456	0.0242683	0.9699999	-0.599765	0.0128286	0	0.00125125	0.040463	0.9753307	0.3920618
ENSG00000147684	NDUFB9	8	125566047	C	T	0.236581	-0.00223522	0.0168443	0.89	0.204921	0.011721	1.92E-68	-0.0109077	0.0822012	0.8944345	0.4153127
ENSG00000147687	TATDN1	8	125526212	G	A	0.146123	-0.00782324	0.020433	0.6899999	-0.129026	0.0182379	1.50E-12	0.060633	0.158595	0.702229	0.7924399
ENSG00000147689	FAM83A	8	124206757	C	T	0.0815109	0.0369261	0.0245617	0.08700015	0.547397	0.0131904	0	0.0674576	0.0448994	0.1329897	0.52662
ENSG00000147789	ZNF7	8	146062871	T	C	0.305169	0.00411942	0.0152454	0.8800001	-0.101931	0.00880215	5.19E-31	-0.040414	0.149607	0.7870573	0.1998441
ENSG00000147799	ARHGAP39	8	145832878	T	C	0.457256	0.00259262	0.0142062	0.99	-0.121366	0.00865994	1.27E-44	-0.0213621	0.117063	0.8552031	0.03429202
ENSG00000147804	SLC39A4	8	145638702	C	A	0.0596421	-0.0189829	0.0267668	0.59	-0.414884	0.0197417	4.72E-98	0.0457547	0.0645531	0.4784534	0.968994
ENSG00000147813	NAPRT	8	144658887	C	T	0.199801	-0.0328482	0.0175562	0.032	-1.02281	0.00968511	0	0.0321158	0.0171674	0.06138144	0.9414252
ENSG00000147852	VLDLR	9	2638157	G	T	0.377734	0.0238514	0.0148753	0.1299999	0.257682	0.00840771	2.78E-206	0.0925614	0.0578063	0.1093253	0.4596419
ENSG00000147853	AK3	9	4726599	T	G	0.0497018	-0.0655348	0.0335475	0.02300011	-0.673553	0.0203005	2.15E-241	0.0972971	0.049893	0.05116233	0.312584
ENSG00000147854	UHRF2	9	6460102	A	T	0.0337972	0.0377027	0.0427337	0.3800004	0.150701	0.0241864	4.64E-10	0.250183	0.286395	0.3823596	0.871131
ENSG00000147862	NFIB	9	14240412	G	A	0.149105	0.0271362	0.0197419	0.1100001	-0.317321	0.0118416	3.48E-158	-0.0855166	0.0622961	0.169832	0.9231957
ENSG00000147872	PLIN2	9	19128830	G	A	0.082505	0.0033655	0.0316607	0.6899999	-0.970341	0.0180648	0	-0.00346837	0.0326285	0.9153453	0.7265195
ENSG00000147874	HAUS6	9	19078129	C	T	0.302187	0.013857	0.0149752	0.3800004	-0.152675	0.012554	4.99E-34	-0.0907616	0.0983691	0.3561829	0.9210789
ENSG00000147883	CDKN2B	9	22006132	G	T	0.0934394	0.0185303	0.023698	0.4100001	-0.654887	0.0143127	0	-0.0282954	0.0361917	0.4343198	0.6900967
ENSG00000147889	CDKN2A	9	21981525	G	C	0.141153	-0.0447605	0.0204779	0.02	0.122487	0.0129167	2.48E-21	-0.365431	0.171568	0.03317616	0.7202664
ENSG00000147894	C9orf72	9	27560204	T	A	0.281312	0.00866571	0.0162386	0.4199997	-0.564992	0.0084538	0	-0.0153378	0.0287422	0.5935963	0.5653863
ENSG00000147905	ZCCHC7	9	37239341	G	A	0.356859	0.011884	0.0147096	0.35	-0.247498	0.00828031	2.65E-196	-0.0480165	0.0594548	0.4193135	0.05924653
ENSG00000147912	FBXO10	9	37549880	C	T	0.28827	0.0132681	0.0154051	0.32	0.12593	0.00912216	2.38E-43	0.105361	0.122569	0.390005	0.8562319
ENSG00000147955	SIGMAR1	9	34636262	C	G	0.0318091	0.0382428	0.0381239	0.3100002	-0.80081	0.0201466	0	-0.0477551	0.0476218	0.3159576	0.7732576
ENSG00000148019	CEP78	9	80872792	T	C	0.0377734	0.0171551	0.0341061	0.6800001	-1.16849	0.0236522	0	-0.0146814	0.0291897	0.6149881	0.0226535
ENSG00000148057	IDNK	9	86248504	A	G	0.0149105	0.0670749	0.124182	0.4799997	-0.841515	0.0514663	4.29E-60	-0.0797073	0.14765	0.5893078	0.3000812
ENSG00000148090	AUH	9	94050146	C	T	0.10835	-0.0184762	0.0219655	0.35	-0.197417	0.0192047	8.71E-25	0.0935899	0.111637	0.4018373	0.5205937
ENSG00000148110	MFSD14B	9	97180078	G	A	0.0487078	-0.0145753	0.036084	0.5400003	1.36809	0.0148139	0	-0.0106538	0.0263758	0.6862693	0.8495422
ENSG00000148120	AOPEP	9	97669212	T	C	0.0576541	-0.00641984	0.0295094	0.7899998	0.544251	0.01804	6.01E-200	-0.0117957	0.0542216	0.8277823	0.3320898
ENSG00000148153	INIP	9	115464651	G	A	0.39165	0.024018	0.0143806	0.1499999	-0.26452	0.00877794	1.71E-199	-0.0907986	0.0544484	0.09539368	0.8255509
ENSG00000148154	UGCG	9	114678347	C	T	0.0884692	-0.0111545	0.0236623	0.6800001	0.186257	0.0131265	1.07E-45	-0.0598877	0.127111	0.6375379	0.7915551
ENSG00000148158	SNX30	9	115575192	C	T	0.237575	0.00458426	0.0159732	0.8800001	-0.323075	0.00956242	3.18E-250	-0.0141895	0.0494429	0.7741226	0.8430432
ENSG00000148175	STOM	9	124116943	C	A	0.0119284	-0.0766961	0.0854307	0.3599996	-0.914784	0.0337004	2.93E-162	0.0838406	0.0934399	0.3695762	0.983117

ENSG00000148180	GSN	9	124032599	G	A	0.455268	-0.00630606	0.0142553	0.7099994	0.610664	0.00717049	0	-0.0103266	0.0233442	0.6582287	0.2498122
ENSG00000148200	NR6A1	9	127406571	C	T	0.500994	0.00425048	0.0142486	0.83	0.0877769	0.00801865	6.90E-28	0.0484237	0.162388	0.7655521	0.1043748
ENSG00000148204	CRB2	9	126130526	T	C	0.250497	0.0224507	0.017528	0.32	-0.112586	0.00956404	5.45E-32	-0.199409	0.156604	0.2029001	0.6775105
ENSG00000148218	ALAD	9	116156105	A	C	0.0506958	-0.00902523	0.0307041	0.7199992	0.295275	0.01776	4.53E-62	-0.0305655	0.104001	0.7688372	0.674119
ENSG00000148219	ASTN2	9	119682426	C	G	0.0795229	0.00133545	0.0250149	0.98	-0.151626	0.0125276	1.01E-33	-0.00880753	0.164979	0.9574246	0.9439598
ENSG00000148225	WDR31	9	116089040	G	T	0.0785288	-0.0512626	0.028962	0.02900013	-0.110767	0.0148805	9.78E-14	0.462796	0.268757	0.0850724	0.02707495
ENSG00000148229	POLE3	9	116171233	G	A	0.0516899	-0.00530581	0.0303878	0.81	-0.279002	0.0258431	3.60E-27	0.0190171	0.10893	0.8614093	0.3588636
ENSG00000148248	SURF4	9	136235647	T	C	0.0715706	0.00174734	0.0292054	0.7800007	0.159449	0.0247032	1.09E-10	0.0109586	0.183173	0.9522936	0.9735507
ENSG00000148288	GBGT1	9	136033836	C	G	0.0984095	0.00430965	0.0241677	1	-0.17586	0.0144086	2.92E-34	-0.0245062	0.137441	0.8584845	0.5548138
ENSG00000148290	SURF1	9	136221081	C	T	0.488072	0.0137034	0.0141877	0.2300001	0.521593	0.0137142	0	0.0262722	0.0272095	0.3342677	0.4056878
ENSG00000148291	SURF2	9	136225736	C	T	0.0387674	-0.0244632	0.036596	0.5199996	0.622562	0.0502147	2.68E-35	-0.0392944	0.0588683	0.5044547	0.6418417
ENSG00000148296	SURF6	9	136200393	A	G	0.464215	-0.0145478	0.0142375	0.1900002	0.412283	0.011356	1.33E-288	-0.0352859	0.034547	0.3070696	0.1803562
ENSG00000148297	MED22	9	136210073	A	G	0.462227	-0.0150495	0.0142391	0.1800002	0.342619	0.00843326	0	-0.0439249	0.0415737	0.2907142	0.5855058
ENSG00000148308	GTF3C5	9	135919983	T	C	0.0129225	-0.0281536	0.062567	0.5700002	0.670116	0.0607286	2.60E-28	-0.042013	0.093445	0.6529978	NA
ENSG00000148331	ASB6	9	132401807	C	T	0.44334	0.00891443	0.0145222	0.56	-0.0870948	0.00880404	4.48E-23	-0.102353	0.167061	0.5400945	0.1405858
ENSG00000148334	PTGES2	9	130886856	A	C	0.110338	-0.0190929	0.0221812	0.2099999	-0.177504	0.012242	1.22E-47	0.107563	0.125182	0.3901989	0.2036406
ENSG00000148335	NTMT1	9	132384686	C	T	0.450298	0.00961748	0.0144665	0.5	0.121589	0.00983583	4.21E-35	0.0790986	0.119151	0.5067856	0.05139739
ENSG00000148337	CIZ1	9	130947502	T	C	0.374751	-0.0136136	0.0147976	0.3700002	-0.0669312	0.0122742	4.95E-08	0.203397	0.224211	0.3643185	0.5100078
ENSG00000148339	SLC25A25	9	130851002	A	G	0.156064	-0.00980393	0.0193011	0.3800004	0.163296	0.0107776	7.42E-52	-0.0600379	0.118264	0.6116908	0.2901207
ENSG00000148341	SH3GLB2	9	131779948	G	C	0.491054	-0.0201684	0.0142188	0.1199999	-0.396285	0.00770577	0	0.0508936	0.0358939	0.1562221	0.3184128
ENSG00000148343	MIGA2	9	131816630	T	A	0.0487078	0.0215542	0.033548	0.4100001	-0.153283	0.0181925	3.59E-17	-0.140617	0.219498	0.5217641	0.2313117
ENSG00000148344	PTGES	9	132507968	C	T	0.0596421	0.0354508	0.0307708	0.16	0.605311	0.0224967	1.84E-159	0.0585663	0.0508813	0.2497168	0.2418345
ENSG00000148346	LCN2	9	130913542	T	C	0.149105	-0.0202574	0.0209534	0.3800004	0.268499	0.0205646	5.84E-39	-0.0754468	0.0782526	0.3349738	0.8413258
ENSG00000148356	LRSAM1	9	130239772	A	C	0.151093	0.00885039	0.0192462	0.7099994	-0.154973	0.0109843	3.36E-45	-0.0571093	0.124257	0.6457982	0.4960676
ENSG00000148358	GPR107	9	132859076	A	G	0.185885	0.0175764	0.020188	0.32	-0.0753988	0.0108591	3.83E-12	-0.233113	0.269846	0.3876586	0.7827885
ENSG00000148362	PAXX	9	139887653	G	C	0.517893	-0.0270315	0.0142317	0.08400014	-0.132277	0.00911435	1.00E-47	0.204356	0.108508	0.0596562	0.3196599
ENSG00000148384	INPP5E	9	139328672	C	T	0.390656	0.0109657	0.0143039	0.3900004	-0.60431	0.00732483	0	-0.0181458	0.0236708	0.4433253	0.8529567
ENSG00000148396	SEC16A	9	139353345	G	A	0.410537	0.0139494	0.014239	0.32	-0.324849	0.00935158	2.16E-264	-0.0429412	0.0438501	0.3274455	0.9039828
ENSG00000148399	DPH7	9	140461371	G	A	0.187873	-0.0164089	0.0180754	0.2999998	-0.0986823	0.0105524	8.63E-21	0.16628	0.184029	0.3662311	0.04160638
ENSG00000148400	NOTCH1	9	139414605	C	T	0.0705765	0.0232188	0.0259298	0.3800004	-0.323446	0.0175673	1.06E-75	-0.0717858	0.0802622	0.3711125	0.4812647
ENSG00000148411	NACC2	9	138942757	G	T	0.355865	0.00463206	0.0152268	0.7400005	0.3205	0.00863245	1.02E-301	0.0144526	0.047511	0.7609799	0.2435049
ENSG00000148426	PROSER2	10	11889807	C	T	0.21173	0.0178974	0.0169436	0.2599998	0.277945	0.0103721	3.47E-158	0.0643918	0.0610075	0.2912091	0.5428182
ENSG00000148429	USP6NL	10	11578131	C	A	0.219682	-0.0112743	0.0167431	0.5400003	-0.179133	0.00930146	1.20E-82	0.0629381	0.0935245	0.5009736	0.3492194
ENSG00000148450	MSRB2	10	23397688	T	C	0.05666	-0.0249666	0.0290623	0.3800004	-0.957174	0.0154161	0	0.0260837	0.0303655	0.3903463	0.2333882
ENSG00000148459	PDSS1	10	27011157	G	A	0.308151	0.0181214	0.0154443	0.28	0.325315	0.00841312	0	0.0557041	0.0474967	0.2408763	0.5331194
ENSG00000148468	FAM171A1	10	15333351	A	C	0.367793	0.0123867	0.0147428	0.5	0.320778	0.00877076	7.52E-293	0.0386145	0.0459716	0.4009281	0.2632178
ENSG00000148481	MINDY3	10	15861344	G	A	0.281312	-0.00957381	0.0164192	0.3599996	-0.205325	0.0088052	2.87E-120	0.0466276	0.0799919	0.5599579	0.821509
ENSG00000148484	RSU1	10	16746068	G	C	0.481113	-0.0230144	0.0142566	0.05800027	0.689329	0.00694996	0	-0.0333867	0.0206846	0.1065092	0.6749071
ENSG00000148488	ST8SIA6	10	17428355	C	T	0.159046	-0.053142	0.0199066	0.006100002	0.120904	0.0122049	3.91E-23	-0.439538	0.170522	0.009948574	0.1359246
ENSG00000148498	PARD3	10	34751370	C	T	0.261431	0.00576895	0.0161319	0.7400005	-0.124393	0.0104044	6.05E-33	-0.0463767	0.129743	0.7207539	0.8072789
ENSG00000148516	ZEB1	10	31713083	A	G	0.254473	-7.17E-05	0.016758	0.98	-0.0699978	0.00941894	1.07E-13	0.00102487	0.239407	0.9965844	0.3613995
ENSG00000148572	NRBF2	10	64903916	C	T	0.28827	-0.00285933	0.0153365	0.8800001	-0.290022	0.0128597	1.26E-112	0.009859	0.0528822	0.8521051	0.6346392
ENSG00000148600	CDHR1	10	85966893	T	C	0.346918	-0.00221331	0.0149612	0.9	0.198711	0.00836691	1.11E-124	-0.0111384	0.0752928	0.882395	0.6451954
ENSG00000148634	HERC4	10	69758385	A	G	0.112326	-0.0335626	0.0240939	0.1499999	0.113892	0.0126797	2.65E-19	-0.294689	0.21408	0.1686556	0.2628526
ENSG00000148655	LRMDA	10	77755568	A	G	0.392644	-0.00119675	0.0145546	0.8700001	-0.591664	0.00849803	0	0.00202268	0.0245994	0.9344679	0.4140321
ENSG00000148660	CAMK2G	10	75603301	T	G	0.188867	0.0338834	0.0183073	0.07100027	0.0799544	0.0100703	2.03E-15	0.423784	0.235111	0.07146882	0.9568342
ENSG00000148671	ADIRF	10	88729310	G	A	0.362823	0.0041641	0.0144351	0.5400003	0.122726	0.00920648	1.54E-40	0.03393	0.117648	0.7730383	0.7564571
ENSG00000148672	GLUD1	10	88832433	A	G	0.154076	-0.0132005	0.0201898	0.3900004	-0.173146	0.0188206	3.59E-20	0.0762393	0.1169	0.5142881	0.4739729
ENSG00000148688	RPP30	10	92649892	C	T	0.0755467	-0.0130191	0.0244948	0.59	0.10371	0.0139827	1.20E-13	-0.125534	0.236792	0.5960113	0.6328255
ENSG00000148690	FRA10AC1	10	95444984	G	C	0.455268	0.00337511	0.0141976	0.8200001	0.238225	0.00788595	1.82E-200	0.0141677	0.0595993	0.8121009	0.6448674

ENSG00000148700	ADD3	10	111825724	C	T	0.153082	-0.0275661	0.0200541	0.16	0.369713	0.0106229	2.17E-265	-0.0745609	0.0542847	0.169592	0.575298
ENSG00000148719	DNAJB12	10	74103788	G	A	0.420477	-0.000379412	0.0146244	0.8800001	0.305178	0.00826003	8.32E-299	-0.00124325	0.0479208	0.9793022	0.2443468
ENSG00000148730	EIF4EBP2	10	72176254	T	C	0.414513	-0.00574835	0.0142716	0.5300002	-0.222462	0.00793947	9.32E-173	0.0258396	0.0641595	0.6871389	0.9540365
ENSG00000148737	TCF7L2	10	114818723	C	G	0.44334	0.0202375	0.0142581	0.17	0.192763	0.0079077	3.03E-131	0.104987	0.0740923	0.1564921	0.03816382
ENSG00000148773	MKI67	10	129909786	G	A	0.353877	0.000798205	0.0148017	0.81	0.0478117	0.00826278	7.19E-09	0.0166948	0.309597	0.9569955	0.8075744
ENSG00000148795	CYP17A1	10	104593789	A	T	0.405567	-0.0044168	0.01449	0.7199992	0.0775359	0.00812286	1.36E-21	-0.0569645	0.186976	0.7606237	0.1802457
ENSG00000148803	FUOM	10	135170093	A	G	0.0337972	-0.0207318	0.0393396	0.4899999	-0.389587	0.0276099	3.28E-45	0.0532148	0.101048	0.5984516	0.09641588
ENSG00000148814	LRRC27	10	134170312	A	G	0.119284	-0.00527677	0.0234017	0.8800001	0.333512	0.0118506	2.91E-174	-0.0158219	0.0701698	0.8216064	0.0741813
ENSG00000148824	MTG1	10	135221204	T	C	0.22167	0.038639	0.0176417	0.02	0.392123	0.0100174	0	0.0985379	0.0450606	0.028758	0.1910337
ENSG00000148832	PAOX	10	135198946	T	C	0.0238569	-0.0592603	0.0421526	0.1100001	0.41153	0.029936	5.31E-43	-0.144	0.102963	0.1619458	0.05853213
ENSG00000148834	GSTO1	10	106011165	A	C	0.336978	-0.0147812	0.0153117	0.2300001	0.200738	0.00858788	7.75E-121	-0.0736342	0.076342	0.3347797	0.5038222
ENSG00000148841	ITPRIP	10	106085028	T	C	0.475149	0.00210089	0.0142636	0.8499999	0.179681	0.00817884	5.70E-107	0.0116923	0.0793847	0.8829055	0.1756518
ENSG00000148842	CNNM2	10	104758209	G	T	0.0884692	0.000153717	0.0266099	0.98	-0.0833076	0.0137046	1.21E-09	-0.00184517	0.319417	0.9953909	0.109242
ENSG00000148843	PDCD11	10	105181227	A	G	0.124254	0.0126813	0.0205489	0.5500004	0.0706838	0.0125673	1.86E-08	0.179409	0.29246	0.5395813	0.6945558
ENSG00000148848	ADAM12	10	127888987	A	C	0.176938	-0.00149817	0.0186358	0.99	0.184656	0.0111067	4.55E-62	-0.0081133	0.100923	0.9359262	0.8569764
ENSG00000148908	RGS10	10	121280780	A	C	0.0407555	-0.00381855	0.0365877	0.91	-0.544054	0.0179207	1.91E-202	0.0070187	0.0672505	0.9168786	0.9078322
ENSG00000148925	BTBD10	11	13447196	T	A	0.153082	0.01432	0.0198733	0.4	-0.588769	0.0159098	8.97E-300	-0.0243219	0.0337604	0.4712614	0.9609852
ENSG00000148926	ADM	11	10327585	G	A	0.445328	0.0350238	0.0142928	0.02800013	-0.336872	0.00772457	0	-0.103968	0.042495	0.01442142	0.5150318
ENSG00000148935	GAS2	11	22740894	T	C	0.142147	0.0190175	0.0193084	0.33	0.197251	0.0111056	1.41E-70	0.0964127	0.0980379	0.3253992	0.08166773
ENSG00000148943	LIN7C	11	27522221	C	T	0.45328	0.0191579	0.014271	0.2099999	0.131764	0.00797024	2.16E-61	0.145396	0.108664	0.1808861	0.9409292
ENSG00000148985	PGAP2	11	3833277	C	T	0.260437	0.00751145	0.0162378	0.84	-0.106562	0.00900382	2.57E-32	-0.070489	0.152495	0.6439111	0.4751854
ENSG00000149016	TUT1	11	62351083	A	G	0.343936	-0.0121851	0.0144926	0.4100001	0.0698687	0.00828201	3.28E-17	-0.1744	0.208454	0.4027972	0.8837525
ENSG00000149054	ZNF215	11	6976749	G	T	0.318091	-0.00466154	0.0149932	0.7499995	0.503351	0.00787188	0	-0.009261	0.0297871	0.7558716	0.0315533
ENSG00000149084	HSD17B12	11	43728076	A	C	0.303181	0.00314826	0.0156071	0.9	-0.716516	0.0084998	0	-0.00439384	0.021782	0.8401363	0.5528826
ENSG00000149089	APIP	11	34906343	T	C	0.316103	0.0117087	0.0153743	0.5999997	0.798203	0.0154238	0	0.0146688	0.0192632	0.4463622	0.8718831
ENSG00000149091	DGKZ	11	46378279	G	A	0.32505	-0.00199326	0.0150391	0.7600007	0.0458841	0.0083923	4.57E-08	-0.0434412	0.327859	0.8945891	0.5982139
ENSG00000149100	EIF3M	11	32616576	G	T	0.0904573	-0.0177101	0.0240361	0.3900004	0.179208	0.013702	4.34E-39	-0.0988241	0.134337	0.4619461	0.2583035
ENSG00000149115	TNKS1BP1	11	57079769	G	A	0.411531	-0.00467883	0.0144916	0.89	-0.108672	0.0119957	1.31E-19	0.0430547	0.133437	0.7469527	0.3488877
ENSG00000149131	SERPING1	11	57373593	G	A	0.260437	0.0366322	0.0160481	0.007799917	0.302385	0.00888249	5.19E-254	0.121144	0.0531909	0.02275423	0.1654604
ENSG00000149136	SSRP1	11	57098405	G	T	0.0447316	0.0319605	0.0344083	0.3599996	-0.111164	0.0178091	4.32E-10	-0.287507	0.312935	0.3582297	0.3181994
ENSG00000149150	SLC43A1	11	57267633	T	C	0.439364	0.0018667	0.0143873	0.9299999	0.0847103	0.00805108	6.87E-26	0.0220363	0.169854	0.8967749	0.2015511
ENSG00000149177	PTPRJ	11	48095891	T	C	0.190855	0.00927815	0.0176518	0.5700002	0.244892	0.0108152	1.62E-113	0.0378867	0.0720993	0.5992502	0.9324762
ENSG00000149179	CSTPP1	11	47072088	A	G	0.131213	-0.00725029	0.0203203	0.7899998	0.132895	0.0120851	3.97E-28	-0.0545567	0.152986	0.7213818	0.7520687
ENSG00000149182	ARFGAP2	11	47192262	C	T	0.0198807	-0.0344207	0.0468326	0.3599996	0.360096	0.0295645	3.97E-34	-0.0955877	0.130293	0.463169	0.7170987
ENSG00000149196	HIKESHI	11	86035111	T	C	0.324056	-0.00486267	0.0155254	0.5999997	0.220922	0.00844079	5.38E-151	-0.0220108	0.0702806	0.7541401	0.3619226
ENSG00000149201	CCDC81	11	86109964	C	T	0.166998	0.0126114	0.0193383	0.56	0.120464	0.0113137	1.79E-26	0.10469	0.160833	0.5150939	0.2976673
ENSG00000149212	SESN3	11	94932204	A	G	0.408549	0.0240836	0.0142759	0.05499966	-0.334971	0.00781034	0	-0.0718977	0.0426513	0.09185218	0.2942036
ENSG00000149218	ENDOD1	11	94844391	A	G	0.181909	-0.0123558	0.0190918	0.3700002	-0.363622	0.0104664	1.83E-264	0.0339798	0.0525136	0.5175892	0.9406724
ENSG00000149231	CCDC82	11	96104510	T	C	0.163022	0.0031109	0.0201859	0.8800001	0.609226	0.0110436	0	0.00510632	0.0331338	0.8775215	0.2137476
ENSG00000149243	KLHL35	11	75137556	T	C	0.423459	-0.0066482	0.0144881	0.7800007	-0.36211	0.00779776	0	0.0183596	0.0400121	0.6463414	0.8418917
ENSG00000149256	TENM4	11	78757934	G	A	0.154076	-0.0163272	0.0190014	0.2300001	0.769858	0.0122033	0	-0.0212081	0.024684	0.3902394	0.8199382
ENSG00000149257	SERPINH1	11	75278464	A	T	0.470179	0.000217042	0.0144575	0.7899998	-0.25827	0.00788946	4.79E-235	-0.000840368	0.0559782	0.9880223	0.06034212
ENSG00000149260	CAPN5	11	76807590	C	G	0.428429	0.0260864	0.0142564	0.04	0.16341	0.00831715	6.09E-86	0.159637	0.0876205	0.06846735	0.3921245
ENSG00000149262	INTS4	11	77647745	C	T	0.282306	0.00270396	0.0157152	0.7099994	-0.467635	0.00829762	0	-0.0057822	0.0336059	0.8633909	0.4219409
ENSG00000149269	PAK1	11	77109216	T	C	0.145129	0.00682551	0.020367	0.7099994	-0.30046	0.0124252	3.47E-129	-0.0227169	0.0677925	0.7375544	0.9635338
ENSG00000149273	RPS3	11	75121927	C	T	0.0656064	-0.00140206	0.0297878	0.98	-0.14268	0.0173243	1.78E-16	0.00982664	0.208778	0.9624594	0.09455572
ENSG00000149289	ZC3H12C	11	110003326	A	C	0.304175	0.0320926	0.0154973	0.02699977	0.481855	0.0100608	0	0.0666021	0.0321918	0.03855378	0.6191104
ENSG00000149292	TTC12	11	113219758	G	T	0.485089	-0.00483531	0.014184	0.56	-0.43157	0.00760642	0	0.011204	0.0328666	0.7331843	0.9828537
ENSG00000149294	NCAM1	11	112990577	C	T	0.0337972	-0.026301	0.0441721	0.4700002	-0.727593	0.0239842	3.81E-202	0.036148	0.0607216	0.5516387	0.9895421
ENSG00000149305	HTR3B	11	113796343	G	T	0.23161	-0.016154	0.0163173	0.2999998	0.075089	0.00942737	1.65E-15	-0.215131	0.218978	0.3258867	0.6978648

ENSG00000149308	NPAT	11	108060655	G	T	0.378728	0.0294358	0.0142737	0.02	-0.14405	0.00805227	1.43E-71	-0.204344	0.0997445	0.0404941	0.07739155
ENSG00000149311	ATM	11	108166520	A	G	0.0149105	-0.0267856	0.0618478	0.6100002	-0.374491	0.0408503	4.85E-20	0.0715253	0.165336	0.6653007	0.1997332
ENSG00000149313	AASDHPPT	11	105957832	T	C	0.110338	-0.0134687	0.0240845	0.64	0.24235	0.0139768	2.37E-67	-0.0555755	0.0994308	0.5762052	0.4562394
ENSG00000149328	GLB1L2	11	134225001	A	G	0.260437	-0.0150737	0.0160228	0.33	0.129867	0.00878611	1.94E-49	-0.11607	0.123628	0.3477996	0.9216172
ENSG00000149346	SLX4IP	20	10516714	G	A	0.442346	0.0269241	0.0142855	0.08100093	0.167428	0.00878454	5.49E-81	0.16081	0.0857393	0.0607147	0.2492088
ENSG00000149357	LAMTOR1	11	71805687	G	A	0.0536779	-0.00274589	0.0336102	0.91	-0.427513	0.0197729	1.13E-103	0.00642294	0.0786185	0.9348872	0.1627893
ENSG00000149403	GRIK4	11	120621040	C	T	0.315109	-0.0138987	0.0149717	0.5400003	0.453914	0.0080647	0	-0.0306197	0.032988	0.3533011	0.1068568
ENSG00000149418	ST14	11	130054864	G	A	0.0994036	0.0162394	0.0219174	0.35	-0.564889	0.0127561	0	-0.0287479	0.0388049	0.4587949	0.2344013
ENSG00000149474	KAT14	20	18143765	A	G	0.44831	0.0033819	0.0143289	0.5999997	0.134783	0.00805389	7.26E-63	0.0250914	0.106321	0.8134357	0.5634847
ENSG00000149480	MTA2	11	62364999	T	C	0.269384	-0.00360952	0.0155106	0.7499995	0.078839	0.00893157	1.08E-18	-0.0457834	0.196806	0.816047	0.3585393
ENSG00000149483	TMEM138	11	61133227	C	A	0.153082	-0.00559495	0.0196288	0.5999997	-0.0724166	0.0128385	1.69E-08	0.0772605	0.2714	0.7758935	0.4151106
ENSG00000149485	FADS1	11	61581944	C	A	0.152087	-0.00455513	0.0181836	0.91	0.560653	0.0107382	0	-0.00812469	0.0324333	0.8021971	0.1139718
ENSG00000149488	TMC2	20	2569841	T	C	0.336978	0.030139	0.0150145	0.06299992	0.0781997	0.00861572	1.12E-19	0.385411	0.196641	0.04999974	0.4032973
ENSG00000149489	ROM1	11	62380893	G	A	0.32008	-0.00362313	0.0146802	0.83	0.106153	0.00841589	1.78E-36	-0.0341312	0.138319	0.8050966	0.4962436
ENSG00000149499	EML3	11	62374963	G	A	0.32505	-0.00610293	0.0146532	0.7099994	0.609762	0.00781971	0	-0.0100087	0.0240313	0.6770549	0.5034367
ENSG00000149503	INCENP	11	61906040	C	G	0.383698	-0.00530824	0.0146784	0.6100002	0.222014	0.00795708	2.57E-171	-0.0239095	0.0661204	0.7176466	0.243056
ENSG00000149516	MS4A3	11	59831330	G	A	0.449304	0.00764341	0.014539	0.3800004	-0.0752846	0.00892083	3.20E-17	-0.101527	0.193495	0.5997913	0.8979032
ENSG00000149527	PLCH2	1	2397194	T	C	0.219682	-0.00444665	0.0181529	0.84	0.703135	0.0110791	0	-0.00632404	0.0258173	0.8064924	0.554225
ENSG00000149531	NA	20	29622933	A	T	0.340954	0.0126601	0.014978	0.32	0.359959	0.0185204	3.84E-84	0.035171	0.0416496	0.398419	0.411427
ENSG00000149532	CPSF7	11	61183812	A	G	0.246521	-0.00478641	0.0160291	0.7099994	-0.0834215	0.0100743	1.23E-16	0.0573762	0.192271	0.7653875	0.4736363
ENSG00000149534	MS4A2	11	59859589	C	T	0.425447	0.00540272	0.0144038	0.6899999	0.210541	0.0118956	4.27E-70	0.0256612	0.0684287	0.7076562	0.7305529
ENSG00000149541	B3GAT3	11	62386207	A	C	0.275348	-0.00671597	0.0154169	0.6100002	0.0709684	0.0088188	8.46E-16	-0.0946332	0.217554	0.6635718	0.4226713
ENSG00000149547	EI24	11	125446843	T	C	0.365805	-0.0183676	0.0149678	0.2099999	-0.125368	0.00828417	9.75E-52	0.14651	0.119783	0.221282	0.3348784
ENSG00000149548	CCDC15	11	124867701	T	C	0.148111	-0.0200469	0.0198719	0.33	0.559294	0.0108423	0	-0.0358432	0.0355371	0.31316	0.1016494
ENSG00000149554	CHEK1	11	125520593	G	C	0.0725646	-0.0393695	0.0283565	0.17	0.148176	0.0165808	4.01E-19	-0.265695	0.193667	0.1700887	0.4510284
ENSG00000149557	FEZ1	11	125340929	T	C	0.191849	0.0332175	0.0181258	0.0649995	-0.22227	0.0103113	4.65E-103	-0.149447	0.0818427	0.067847	0.004105338
ENSG00000149564	ESAM	11	124627106	C	T	0.23161	-0.0222787	0.0168167	0.1499999	-0.0710766	0.00951953	8.24E-14	0.313446	0.240295	0.1920898	0.4507605
ENSG00000149571	KIRREL3	11	126583304	G	A	0.05666	-0.018138	0.029617	0.4400003	0.202563	0.0194372	1.98E-25	-0.0895427	0.146464	0.5409588	0.01364098
ENSG00000149573	MPZL2	11	118129684	T	C	0.480119	-0.0133692	0.0142395	0.2	-0.689049	0.00694809	0	0.0194024	0.0206664	0.3478133	0.882657
ENSG00000149575	SCN2B	11	118040027	A	G	0.225646	-0.00847348	0.0181802	0.3599996	0.142919	0.0108372	1.03E-39	-0.0592886	0.127286	0.6413643	0.467405
ENSG00000149577	SIDT2	11	117058804	T	C	0.201789	0.00866007	0.0189741	0.84	-1.05051	0.00775363	0	-0.00824368	0.0180619	0.6480932	0.1666956
ENSG00000149591	TAGLN	11	117072767	C	T	0.262425	0.0127955	0.016899	0.6200004	-0.834811	0.00752979	0	-0.0153274	0.0202434	0.4489556	0.6695294
ENSG00000149600	COMMD7	20	31311153	T	C	0.15507	0.00706612	0.0187366	0.81	0.309272	0.0118393	2.03E-150	0.0228476	0.0605892	0.7061068	0.6290448
ENSG00000149636	DSN1	20	35391212	T	G	0.0407555	-0.0139651	0.0365415	0.59	-0.520864	0.0230634	6.23E-113	0.0268114	0.0701656	0.7023751	0.6897659
ENSG00000149657	LSM14B	20	60703975	G	A	0.196819	0.0107	0.0177368	0.6899999	-0.191054	0.0143968	3.43E-40	-0.056005	0.0929323	0.5467461	0.6190523
ENSG00000149679	CABLES2	20	60973014	G	A	0.501988	-0.00220378	0.0141695	0.6999999	0.281096	0.0101276	1.50E-169	-0.00783994	0.0504087	0.8764056	0.07791272
ENSG00000149716	LTO1	11	69479014	G	A	0.346918	0.0130943	0.0146927	0.4199997	-0.114381	0.00910969	3.69E-36	-0.114479	0.128777	0.3740166	0.2831575
ENSG00000149743	TRPT1	11	63992498	A	G	0.0636183	-0.0426506	0.0291695	0.1299999	-0.413535	0.0157031	7.70E-153	0.103137	0.0706456	0.1443132	0.3326065
ENSG00000149761	NUDT22	11	63995785	T	G	0.0188867	0.0161879	0.0660023	0.95	-0.206806	0.0264425	5.24E-15	-0.0782756	0.319307	0.8063464	0.9957046
ENSG00000149781	FERMT3	11	63982752	T	C	0.385686	0.00333143	0.0143296	0.6600001	-0.130732	0.0080711	5.24E-59	-0.0254828	0.109621	0.8161792	0.03663975
ENSG00000149782	PLCB3	11	64027808	A	G	0.298211	-0.0027995	0.0153871	0.8	-0.144525	0.0128262	1.89E-29	0.0193703	0.10648	0.8556499	0.09525467
ENSG00000149792	MRPL49	11	64892249	C	T	0.0208748	0.00336591	0.0526657	0.95	-0.245943	0.0240705	1.65E-24	-0.0136857	0.214142	0.9490422	0.1474643
ENSG00000149798	CDC42EP2	11	65086094	G	T	0.145129	0.0120899	0.0195424	0.3100002	0.199847	0.0119146	3.83E-63	0.0604959	0.0978535	0.5364242	0.2900115
ENSG00000149806	FAU	11	64889022	G	C	0.510934	-0.0100039	0.0142327	0.7199992	-0.0906128	0.0118189	1.76E-14	0.110403	0.15773	0.483961	0.1631608
ENSG00000149809	TM7SF2	11	64881586	T	C	0.0347913	-0.00501962	0.0386648	0.8800001	-0.449673	0.0202151	1.28E-109	0.0111628	0.0859857	0.8967074	0.0547788
ENSG00000149922	TBX6	16	30100161	A	C	0.416501	-0.00849912	0.0144824	0.4	-0.202227	0.00793459	2.76E-143	0.0420275	0.0716334	0.5574032	0.05779673
ENSG00000149923	PPP4C	16	30091998	G	A	0.467197	0.0244951	0.0142204	0.05699936	-0.203013	0.00866394	2.02E-121	-0.120658	0.0702356	0.08581542	0.8471327
ENSG00000149929	HIRIP3	16	30005701	C	G	0.148111	0.00714895	0.0216968	0.9299999	-0.0969035	0.0141029	6.37E-12	-0.0737739	0.224158	0.7420687	0.7728824
ENSG00000150045	KLRF1	12	9988841	C	T	0.336978	-0.0281076	0.0153395	0.07399971	0.0549157	0.00841853	6.88E-11	-0.511831	0.290139	0.07771666	0.9918571
ENSG00000150048	CLEC1A	12	10243189	C	T	0.146123	-0.00344713	0.0196182	0.7899998	0.33664	0.011158	5.81E-200	-0.0102398	0.0582775	0.8605235	0.2736487

ENSG00000150054	MPP7	10	28481668	A	T	0.21173	0.00842981	0.0168173	0.8700001	-0.627168	0.0129952	0	-0.0134411	0.0268161	0.6162084	0.2599208
ENSG00000150076	NA	10	33002049	G	T	0.11829	-0.00371385	0.0232944	0.8499999	-0.265794	0.0141405	8.05E-79	0.0139727	0.087644	0.8733338	0.1922756
ENSG00000150093	ITGB1	10	33241983	A	G	0.459245	-0.00281174	0.0143291	0.9599999	0.0720175	0.00796266	1.50E-19	-0.0390425	0.199014	0.8444695	0.6831113
ENSG00000150316	CWC15	11	94701281	T	C	0.340954	-0.0146319	0.0152299	0.3800004	0.189913	0.00839454	2.55E-113	-0.0770452	0.0802663	0.3371206	0.3617064
ENSG00000150337	FCGR1A	1	149759150	G	A	0.0864811	-0.00870039	0.0258786	0.7899998	0.548756	0.0343961	2.67E-57	-0.0158548	0.0471692	0.7367762	0.3006845
ENSG00000150347	ARID5B	10	63758881	G	T	0.176938	0.00304015	0.0186683	0.6999999	0.440098	0.0122008	6.39E-285	0.0069079	0.042419	0.8706371	0.8706167
ENSG00000150401	DCUN1D2	13	114127700	G	A	0.159046	-0.0250123	0.0189726	0.1299999	0.102205	0.0102046	1.30E-23	-0.244726	0.187234	0.1911911	0.9287747
ENSG00000150403	TMCO3	13	114174926	T	G	0.0606362	-0.0149042	0.0267886	0.4600002	-0.670887	0.0142698	0	0.0222157	0.0399329	0.577989	0.3920339
ENSG00000150433	TMEM218	11	124974028	T	G	0.310139	-0.0117904	0.0153616	0.3900004	0.30928	0.00928819	4.10E-243	-0.0381221	0.0496821	0.442891	0.5071379
ENSG00000150455	TIRAP	11	126160850	T	C	0.110338	-0.000923098	0.0223301	0.9699999	0.151344	0.0122194	3.13E-35	-0.00609934	0.147546	0.967026	0.3664869
ENSG00000150456	EEF1AKMT1	13	21325483	G	T	0.486083	0.00958052	0.0142062	0.4100001	0.0854	0.00793179	4.94E-27	0.112184	0.166675	0.5009021	0.1495947
ENSG00000150457	LATS2	13	21591428	G	C	0.100398	-0.0143466	0.0234255	0.56	0.313714	0.0136148	1.76E-117	-0.0457315	0.0746979	0.5403929	0.7525129
ENSG00000150459	SAP18	13	21718937	G	A	0.33996	-0.00149607	0.0151188	0.9	-0.121157	0.00848912	3.27E-46	0.0123482	0.12479	0.9211765	0.8429771
ENSG00000150477	KIAA1328	18	34610602	C	T	0.299205	-0.00354806	0.0156878	0.5999997	0.223553	0.00852149	1.09E-151	-0.0158712	0.0701775	0.8210782	0.2506387
ENSG00000150527	MIA2	14	39795322	T	C	0.277336	-0.0121873	0.016066	0.35	0.0959548	0.00989775	3.18E-22	-0.127011	0.167945	0.44949	0.6584784
ENSG00000150540	HNMT	2	138747760	T	C	0.531809	-0.0115836	0.0142382	0.2200002	0.241179	0.00783198	3.14E-208	-0.0480291	0.0590565	0.4160609	0.487407
ENSG00000150556	LYPD6B	2	149983198	C	G	0.345924	-0.0194317	0.0157774	0.1	0.2776	0.0100155	4.36E-169	-0.0699989	0.0568911	0.2185466	0.9519639
ENSG00000150593	PDCD4	10	112645664	T	C	0.297217	0.0131663	0.0153696	0.3400001	0.200066	0.00849653	1.35E-122	0.0658097	0.0768734	0.3919539	0.8155726
ENSG00000150594	ADRA2A	10	112838724	A	T	0.305169	-0.00520853	0.0153572	0.7300002	-0.0548156	0.00869758	2.93E-10	0.0950191	0.280566	0.7348592	0.685911
ENSG00000150625	GPM6A	4	176738950	T	C	0.188867	0.000717058	0.0192517	0.9	-0.378224	0.00976013	0	-0.00189585	0.0509002	0.9702885	0.04297243
ENSG00000150627	WDR17	4	177045481	C	G	0.100398	-0.0242492	0.0238123	0.32	0.187002	0.0200637	1.16E-20	-0.129674	0.128095	0.3113836	0.6051848
ENSG00000150637	CD226	18	67563716	T	C	0.473161	-0.0163837	0.0142097	0.1800002	-0.347535	0.00773762	0	0.0471426	0.0409006	0.2490687	0.4182234
ENSG00000150656	CNDP1	18	72228061	G	A	0.419483	0.00650859	0.0145158	0.83	0.0483458	0.00838784	8.22E-09	0.134626	0.301156	0.654854	0.553104
ENSG00000150667	FSIP1	15	39983631	A	G	0.298211	-0.0180017	0.0156805	0.32	-0.120762	0.00884859	2.09E-42	0.149068	0.130305	0.2526275	0.08685631
ENSG00000150672	DLG2	11	84252510	T	C	0.502982	-0.0070595	0.0142559	0.6899999	0.108261	0.0155352	3.20E-12	-0.0652079	0.132012	0.6213394	0.6765021
ENSG00000150681	RGS18	1	192141266	G	C	0.487078	0.0168312	0.0142301	0.1900002	0.13328	0.00795875	6.02E-63	0.126284	0.107034	0.2380601	0.9709515
ENSG00000150687	PRSS23	11	86583026	A	G	0.0616302	0.0217397	0.029118	0.4500005	0.144646	0.0163327	8.28E-19	0.150296	0.20202	0.4568959	0.3914863
ENSG00000150712	MTMR12	5	32270107	G	C	0.00894632	-0.0274442	0.0595559	0.6700003	0.762463	0.0482859	3.61E-56	-0.0359942	0.0781432	0.6450727	0.1699069
ENSG00000150753	CCT5	5	10258278	T	C	0.146123	0.00993575	0.0202822	0.8	-0.485997	0.0111938	0	-0.020444	0.0417358	0.6242442	0.4098822
ENSG00000150756	ATPSCKMT	5	10238225	C	T	0.15507	0.00589275	0.0202986	0.9699999	0.131655	0.010805	3.75E-34	0.0447591	0.154224	0.7716472	0.9130484
ENSG00000150760	DOCK1	10	128922379	A	C	0.055666	-0.0765968	0.0347421	0.02100003	0.143584	0.0206068	3.22E-12	-0.533463	0.253787	0.03555245	0.6841273
ENSG00000150764	DIXDC1	11	111845588	C	G	0.326044	-0.00342139	0.0147767	0.9199999	0.242242	0.00851169	3.68E-178	-0.0141239	0.0610018	0.8169016	0.0840604
ENSG00000150768	DLAT	11	111915326	T	G	0.314115	-0.0116504	0.0148938	0.4799997	-0.0458447	0.00827642	3.04E-08	0.254127	0.328098	0.4386077	0.1393655
ENSG00000150776	NKAPD1	11	111950342	A	G	0.303181	0.000228163	0.0151477	0.8800001	0.0813734	0.00921807	1.07E-18	0.0028039	0.186151	0.9879823	0.3576541
ENSG00000150782	IL18	11	112024407	T	C	0.224652	-0.00294617	0.0162111	0.91	0.309576	0.013556	1.98E-115	-0.00951678	0.0523671	0.855793	0.3314296
ENSG00000150867	PIP4K2A	10	22913631	T	A	0.306163	0.00881156	0.0152143	0.35	-0.114273	0.00885534	4.25E-38	-0.0771098	0.133274	0.5628722	0.3678696
ENSG00000150907	FOXO1	13	41185269	T	C	0.359841	-0.00839452	0.014952	0.4	-0.0848553	0.0081612	2.55E-25	0.0989275	0.176463	0.5750603	0.9175313
ENSG00000150938	CRIM1	2	36680673	A	G	0.379722	0.0297663	0.014846	0.02699977	-0.159903	0.00917887	5.74E-68	-0.186152	0.0934565	0.04638689	0.2874155
ENSG00000150961	SEC24D	4	119701908	G	A	0.422465	0.0149664	0.0142844	0.2700001	0.0668877	0.00804538	9.27E-17	0.223754	0.215247	0.2985621	0.9756266
ENSG00000150967	ABCB9	12	123435847	A	G	0.308151	-0.00650202	0.0156786	0.5500004	0.290492	0.00858476	5.43E-251	-0.0223828	0.0539767	0.6783794	0.8659217
ENSG00000150977	RILPL2	12	123910600	G	A	0.321074	-0.0172051	0.0148081	0.3400001	0.409513	0.0121124	1.43E-250	-0.0420136	0.0361816	0.2455665	0.4077162
ENSG00000150990	DHX37	12	125452519	C	T	0.253479	-0.0133452	0.0158032	0.35	-0.0886577	0.00954027	1.50E-20	0.150525	0.178984	0.4003498	0.8289283
ENSG00000150991	UBC	12	125399032	C	T	0.000994036	0.245394	0.20569	0.3100002	-0.456885	0.0724248	2.82E-10	-0.537103	0.458181	0.2410969	0.8157661
ENSG00000150995	ITPR1	3	4712278	A	G	0.084493	-0.0992904	0.0277332	0.000309999	-0.419295	0.01421	2.34E-191	0.236803	0.0666275	0.000379226	0.0244989
ENSG00000151006	PRSS53	16	31097847	A	G	0.233598	0.0232233	0.0165927	0.1	0.205912	0.00958561	2.32E-102	0.112783	0.0807523	0.1625194	0.1033399
ENSG00000151012	SLC7A11	4	139124377	A	G	0.0447316	0.0535359	0.0368662	0.08799946	0.217156	0.0200732	2.82E-27	0.246532	0.171291	0.1500772	0.3701548
ENSG00000151014	NOCT	4	139951924	T	C	0.189861	0.0124025	0.0178522	0.4700002	0.137937	0.0151243	7.50E-20	0.0899145	0.129798	0.4884819	0.4300372
ENSG00000151023	ENKUR	10	25287996	A	G	0.286282	0.000350741	0.0156377	0.8600001	0.137559	0.00866078	8.31E-57	0.00254975	0.11368	0.9821056	0.9022111
ENSG00000151062	CACNA2D4	12	1964562	C	T	0.387674	-0.020025	0.014709	0.1	-0.0856693	0.00827931	4.30E-25	0.233748	0.173175	0.177087	0.5780211
ENSG00000151065	DCP1B	12	2084460	T	A	0.0347913	0.0016422	0.037619	0.9	-0.221951	0.0220684	8.52E-24	-0.00739893	0.169494	0.965181	0.6417389

ENSG00000151067	CACNA1C	12	2441030	C	T	0.127237	0.0271788	0.0211677	0.25	-0.0722915	0.0129937	2.64E-08	-0.375961	0.300507	0.2109014	0.6262262
ENSG00000151079	KCNA6	12	4939309	G	C	0.358847	0.00242765	0.0152631	0.7700005	-0.107771	0.00853833	1.60E-36	-0.022526	0.141637	0.8736366	0.4342192
ENSG00000151090	THRB	3	24347712	A	C	0.152087	0.0231988	0.019751	0.2700001	0.0739349	0.0112978	5.98E-11	0.313773	0.271409	0.2476444	0.4536426
ENSG00000151092	NGLY1	3	25795982	A	T	0.276342	0.00903311	0.0160229	0.5099998	-0.103296	0.00898087	1.29E-30	-0.0874487	0.155302	0.573376	0.3269776
ENSG00000151093	OXSM	3	25830216	A	G	0.196819	0.0207234	0.0194949	0.1900002	0.116793	0.0111682	1.35E-25	0.177438	0.167779	0.290253	0.5180498
ENSG00000151116	UEVLD	11	18580725	C	G	0.140159	0.01171	0.0218568	0.6300007	-0.226926	0.0116272	7.90E-85	-0.0516028	0.0963533	0.5922639	0.7160846
ENSG00000151117	TMEM86A	11	18720500	G	A	0.431412	-0.0125711	0.0142739	0.5	0.128994	0.00802032	3.34E-58	-0.0974549	0.110821	0.379191	0.6327436
ENSG00000151131	NOPCHAP1	12	105411801	C	A	0.10338	0.0127388	0.0248839	0.6800001	0.320059	0.0260516	1.08E-34	0.0398014	0.0778153	0.6090111	0.5141274
ENSG00000151135	TMEM263	12	107361026	T	C	0.462227	0.0226916	0.0141846	0.08300036	0.307253	0.00847404	7.41E-288	0.0738532	0.0462108	0.1100029	0.9221768
ENSG00000151136	ABTB3	12	107882804	A	G	0.484095	0.0104977	0.0142723	0.4	-0.566704	0.00727941	0	-0.0185241	0.0251859	0.462037	0.3988472
ENSG00000151148	UBE3B	12	109944857	G	A	0.0646123	-0.00778665	0.0275311	0.9	0.849634	0.0160148	0	-0.00916471	0.0324039	0.7773094	0.3424349
ENSG00000151150	ANK3	10	62140703	G	A	0.311133	0.0183648	0.0151693	0.1199999	-0.540122	0.00802422	0	-0.0340012	0.0280895	0.2261024	0.03125679
ENSG00000151151	IPMK	10	59989486	A	G	0.209742	0.0153169	0.0173901	0.33	-0.57443	0.0131052	0	-0.0266645	0.0302798	0.3785317	0.7889536
ENSG00000151164	RAD9B	12	110954675	C	A	0.203777	-0.0160062	0.0168398	0.3800004	0.190665	0.010455	2.63E-74	-0.0839491	0.0884411	0.3425139	0.4935923
ENSG00000151176	PLBD2	12	113811787	T	C	0.114314	-0.010484	0.024533	0.5700002	0.218322	0.0139506	3.34E-55	-0.0480208	0.112413	0.6692457	0.03680403
ENSG00000151208	DLG5	10	79618463	C	T	0.276342	0.0393463	0.0156767	0.003099988	-0.47414	0.00850864	0	-0.0829846	0.033097	0.01216517	0.3622232
ENSG00000151229	SLC2A13	12	40324357	G	C	0.457256	-0.0142022	0.0145152	0.2300001	-0.051137	0.00804698	2.09E-10	0.277729	0.287194	0.3335235	0.7563277
ENSG00000151233	GXYLT1	12	42507164	G	A	0.424453	0.0178348	0.0143088	0.1800002	0.218496	0.00797068	1.95E-165	0.0816255	0.0655555	0.213082	0.03547376
ENSG00000151239	TWF1	12	44193852	C	T	0.140159	-0.00260048	0.0192876	0.9	0.097453	0.0116143	4.83E-17	-0.0266845	0.197942	0.8927628	0.3589789
ENSG00000151240	DIP2C	10	527906	G	A	0.0616302	0.021456	0.0308494	0.6700003	0.410076	0.0152043	3.23E-160	0.052322	0.0752534	0.4868819	0.2368025
ENSG00000151247	EIF4E	4	99822311	G	A	0.426441	0.0165609	0.0145054	0.29	-0.27051	0.00947861	3.85E-179	-0.061221	0.0536653	0.2539559	0.8902861
ENSG00000151287	TEX30	13	103422250	A	C	0.475149	-0.00762668	0.0141803	0.7600007	0.0902312	0.00883926	1.83E-24	-0.0845238	0.157373	0.5912045	0.6054156
ENSG00000151292	CSNK1G3	5	122900266	T	C	0.0337972	0.0714322	0.0372986	0.08199927	0.316843	0.0215144	4.32E-49	0.22545	0.118711	0.05754435	0.1383526
ENSG00000151304	SRFBP1	5	121354460	G	C	0.0377734	0.0432867	0.0329616	0.1900002	-0.134724	0.0194307	4.10E-12	-0.321298	0.249009	0.1969443	0.3161846
ENSG00000151320	AKAP6	14	33049523	C	T	0.197813	0.00814612	0.0175258	0.6800001	-0.170928	0.0145439	6.85E-32	-0.0476581	0.102613	0.6423295	0.7832075
ENSG00000151327	FAM177A1	14	35548224	T	C	0.447316	-0.00561541	0.0142194	0.8499999	-0.21248	0.00787187	1.82E-160	0.026428	0.0669283	0.6929391	0.1209281
ENSG00000151332	MBIP	14	36778823	C	T	0.0596421	0.0445886	0.0308121	0.17	-0.120703	0.0164428	2.12E-13	-0.369407	0.260184	0.1556692	0.5854172
ENSG00000151348	EXT2	11	44192039	T	G	0.120278	0.0254848	0.0233739	0.3599996	0.73269	0.0124168	0	0.0347825	0.0319069	0.2756585	0.919123
ENSG00000151353	TMEM18	2	672387	G	A	0.159046	0.021753	0.01902	0.1800002	-0.253156	0.0109135	4.93E-119	-0.0859274	0.0752229	0.253328	0.8871989
ENSG00000151366	NDUFC2	11	77785307	T	C	0.166004	-0.009144	0.0195952	0.6700003	-0.350096	0.0110357	7.23E-221	0.0261185	0.0559769	0.6407901	0.2193147
ENSG00000151376	ME3	11	86267914	A	C	0.127237	-0.00284852	0.0219655	0.9199999	0.64985	0.0112136	0	-0.00438335	0.033801	0.8968186	0.3951504
ENSG00000151413	NUBPL	14	32144796	G	T	0.33499	-0.00323683	0.0148097	0.9199999	0.0623851	0.00835343	8.13E-14	-0.0518847	0.237493	0.8270645	0.8151784
ENSG00000151414	NEK7	1	198208821	T	G	0.16004	-0.00656238	0.0215821	0.7199992	0.123583	0.0123826	1.86E-23	-0.0531009	0.174717	0.7611851	0.3406578
ENSG00000151422	FER	5	108308032	G	A	0.0318091	0.00171649	0.0365266	0.8200001	0.137649	0.0184863	9.62E-14	0.0124701	0.265366	0.9625197	0.9971859
ENSG00000151445	VIPAS39	14	77908656	C	T	0.522863	0.0173022	0.0142568	0.2599998	0.113302	0.00905069	5.91E-36	0.152709	0.12642	0.2270675	0.160606
ENSG00000151458	ANKRD50	4	125609547	T	C	0.166004	0.00953009	0.0192255	0.64	-0.374546	0.0104314	2.49E-282	-0.0254444	0.051335	0.6201391	0.5979923
ENSG00000151461	UPF2	10	12023595	T	G	0.362823	0.0061556	0.0147273	0.5099998	0.0501759	0.00841991	2.53E-09	0.12268	0.294234	0.6767166	0.05551404
ENSG00000151465	CDC123	10	12265276	C	T	0.456262	-0.023044	0.0142752	0.08300036	0.344734	0.0115277	1.71E-196	-0.0668458	0.0414696	0.1069791	0.9335205
ENSG00000151466	SCLT1	4	129900420	G	C	0.316103	-0.0270699	0.015144	0.08199927	-0.355735	0.0152794	6.75E-120	0.0760957	0.0426963	0.07470767	0.3486742
ENSG00000151468	CCDC3	10	13040139	C	T	0.238569	0.0242164	0.0163921	0.1499999	0.368368	0.008769	0	0.0657397	0.0445267	0.1398342	0.2542939
ENSG00000151470	C4orf33	4	130024479	T	G	0.187873	0.00318299	0.018622	0.9699999	0.536015	0.0153785	3.61E-266	0.00593825	0.034742	0.8642833	0.1716114
ENSG00000151474	FRMD4A	10	14094923	G	C	0.354871	0.00897428	0.0149498	0.5300002	0.134694	0.00891178	1.31E-51	0.066627	0.111078	0.5486253	0.1595856
ENSG00000151490	PTPRO	12	15612832	A	G	0.0685885	-0.00681218	0.0261607	0.84	0.20525	0.0149737	9.17E-43	-0.0331896	0.127481	0.7945934	0.696006
ENSG00000151491	EPS8	12	15904177	G	A	0.233598	-0.0423453	0.0182409	0.01099993	0.111198	0.0100334	1.52E-28	-0.380809	0.167599	0.02307819	0.7571632
ENSG00000151498	ACAD8	11	134129569	T	C	0.255467	0.00990643	0.0156631	0.6700003	0.332335	0.00854128	0	0.0298085	0.0471366	0.5271348	0.254967
ENSG00000151500	THYN1	11	134120718	T	C	0.348907	-0.0176068	0.0149246	0.2999998	0.137745	0.00853521	1.37E-58	-0.127822	0.108639	0.2393643	0.7083549
ENSG00000151502	VPS26B	11	134106112	T	C	0.348907	-0.0176068	0.0149246	0.2999998	0.149915	0.0127115	4.21E-32	-0.117446	0.100051	0.2404517	0.6024486
ENSG00000151503	NCAPD3	11	134057681	T	G	0.0815109	0.00355001	0.0231961	0.8700001	0.385065	0.0128694	1.05E-196	0.00921926	0.0602403	0.8783656	0.7735422
ENSG00000151532	VTI1A	10	114392629	A	G	0.431412	0.0159592	0.0142386	0.3100002	-0.0584949	0.00808432	4.63E-13	-0.272831	0.246319	0.2680217	0.4933923
ENSG00000151552	QDPR	4	17487870	C	A	0.235586	0.0164621	0.0164357	0.2700001	-0.922868	0.00777375	0	-0.017838	0.01781	0.316551	0.8976855

ENSG00000151553	FHIP2A	10	116620547	A	G	0.511928	-0.0104725	0.0142146	0.3100002	0.235325	0.00785018	1.96E-197	-0.0445023	0.0604224	0.4614142	0.6042069
ENSG00000151575	TEX9	15	56637201	C	G	0.0536779	-0.00663859	0.0316337	0.91	0.126525	0.0179316	1.71E-12	-0.0524684	0.250129	0.8338508	0.4949757
ENSG00000151576	QTRT2	3	113765974	C	T	0.382704	-0.0403397	0.0148869	0.004499974	-0.0642416	0.0084747	3.44E-14	0.627937	0.246094	0.0107224	0.0446477
ENSG00000151611	MMAA	4	146560301	T	C	0.0974155	0.0228774	0.0232177	0.1800002	-0.253883	0.0142289	3.29E-71	-0.09011	0.0915897	0.3251922	0.9092349
ENSG00000151612	ZNF827	4	146769283	A	G	0.342942	-0.000811732	0.0148627	0.9400001	0.0892201	0.00851094	1.03E-25	-0.00909808	0.166587	0.9564455	0.04052677
ENSG00000151623	NR3C2	4	149182881	T	C	0.429423	-0.00970325	0.0145313	0.4600002	-0.0765647	0.00815629	6.16E-21	0.126733	0.190271	0.5053694	0.4213341
ENSG00000151632	AKR1C2	10	5045087	C	T	0.131213	0.00148887	0.0221735	0.83	0.899613	0.0108804	0	0.00165501	0.0246478	0.9464652	0.6523941
ENSG00000151640	DPYSL4	10	134009842	C	T	0.28827	0.0228573	0.0161824	0.1299999	0.620047	0.00955719	0	0.0368638	0.0261049	0.1579075	0.5907742
ENSG00000151650	VENTX	10	135053304	G	A	0.16998	0.029355	0.019213	0.1499999	-0.292113	0.0127311	1.66E-116	-0.100492	0.0659181	0.127385	0.335814
ENSG00000151651	ADAM8	10	135083139	C	T	0.455268	-0.00528969	0.0142647	0.7199992	-0.127968	0.00921325	7.32E-44	0.0413359	0.11151	0.7108672	0.3848156
ENSG00000151655	ITIH2	10	7768357	A	G	0.403579	0.0386485	0.0143775	0.004300015	-0.249003	0.00800024	1.12E-212	-0.155213	0.0579551	0.00740292	0.1460028
ENSG00000151657	KIN	10	7812589	C	G	0.43837	-0.0287565	0.0143338	0.03599979	-0.0771515	0.0080492	9.25E-22	0.372727	0.189814	0.04957067	0.3780222
ENSG00000151665	PIGF	2	46826167	T	A	0.364811	0.0216845	0.0147052	0.16	0.136771	0.00819489	1.55E-62	0.158546	0.107936	0.141862	0.5177684
ENSG00000151687	ANKAR	2	190582467	T	C	0.0437376	0.0219277	0.0325397	0.5500004	0.110981	0.0178037	4.56E-10	0.197581	0.294909	0.5028757	0.3562689
ENSG00000151689	INPP1	2	191222293	A	G	0.232604	-0.0316635	0.0164522	0.02699977	0.281168	0.00919734	3.01E-205	-0.112614	0.0586296	0.05476035	0.2299041
ENSG00000151690	MFSD6	2	191323506	G	A	0.44831	0.0186006	0.0143149	0.1100001	-0.587457	0.00741937	0	-0.0316629	0.0243709	0.193871	0.3812609
ENSG00000151692	RNF144A	2	7132970	A	G	0.213718	0.0305124	0.0179003	0.0990011	0.148756	0.0112573	7.27E-40	0.205117	0.12133	0.09091946	0.5603963
ENSG00000151693	ASAP2	2	9446353	C	G	0.109344	0.0390086	0.022775	0.08700015	0.131575	0.0144511	8.64E-20	0.296475	0.176132	0.09232545	0.2472009
ENSG00000151694	ADAM17	2	9662268	G	A	0.418489	0.0274873	0.0144534	0.08100093	-0.252718	0.00790864	4.65E-224	-0.108767	0.0572929	0.05763964	0.1613127
ENSG00000151702	FLI1	11	128619103	C	T	0.416501	-0.0224451	0.0142941	0.1299999	-0.0675524	0.00799323	2.88E-17	0.332262	0.215222	0.1226334	0.5912921
ENSG00000151715	TMEM45B	11	129707806	T	C	0.101392	0.0415693	0.0217984	0.07699987	-0.717864	0.0116877	0	-0.0579069	0.0303803	0.05664111	0.01046675
ENSG00000151718	WWC2	4	184131188	G	T	0.0318091	-0.038057	0.0359663	0.2099999	-0.251903	0.0207007	4.56E-34	0.151078	0.143317	0.2918135	0.07804443
ENSG00000151725	CENPU	4	185635529	C	T	0.310139	0.0171263	0.0153752	0.3800004	-0.572681	0.0144734	0	-0.0299055	0.0268584	0.2655152	0.9629639
ENSG00000151726	ACSL1	4	185712360	G	T	0.421471	-0.00538529	0.0143921	0.64	-0.0612253	0.00805533	2.95E-14	0.0879586	0.235352	0.7086044	0.1171817
ENSG00000151729	SLC25A4	4	186066414	G	A	0.481113	-0.0211189	0.0142438	0.07299952	-0.14067	0.0079553	5.71E-70	0.15013	0.101612	0.1395448	0.7067465
ENSG00000151743	AMN1	12	31853089	A	G	0.00994036	0.0149315	0.086363	0.9299999	0.703168	0.104281	1.55E-11	0.0212346	0.12286	0.8627807	NA
ENSG00000151746	BICD1	12	32398168	G	A	0.191849	-0.0100704	0.0185121	0.2999998	0.14501	0.00992307	2.31E-48	-0.0694463	0.127749	0.5867074	0.7356274
ENSG00000151748	SAV1	14	51116912	C	T	0.32008	-0.000303501	0.0149333	0.9	-0.195117	0.00855812	4.69E-115	0.00155549	0.0765353	0.9837851	0.7066279
ENSG00000151773	CCDC122	13	44425954	A	G	0.502982	-0.0231283	0.0142303	0.04099964	-0.234674	0.00783507	4.18E-197	0.0985552	0.0607279	0.1046112	0.3603854
ENSG00000151778	SERP2	13	44959825	A	G	0.0685885	-0.0016571	0.0290837	0.91	0.25457	0.017066	2.56E-50	-0.00650941	0.114247	0.9545639	0.8844791
ENSG00000151779	NBAS	2	15504243	A	G	0.311133	-0.00156305	0.0151582	0.9699999	-0.15179	0.0083639	1.33E-73	0.0102975	0.0998647	0.9178724	0.4897596
ENSG00000151789	ZNF385D	3	21937363	C	G	0.290258	0.0233124	0.0161377	0.14	-0.148207	0.00898277	3.73E-61	-0.157297	0.109303	0.1501259	0.4748248
ENSG00000151806	GUF1	4	44691693	G	C	0.417495	-0.00236351	0.0146457	0.8200001	0.280968	0.00950791	6.38E-192	-0.00841201	0.0521266	0.8717967	0.435915
ENSG00000151835	SACS	13	23955403	G	A	0.27833	0.0139494	0.0162096	0.58	-0.10582	0.00911931	3.93E-31	-0.131821	0.153601	0.390778	0.6084131
ENSG00000151849	CENPJ	13	25477094	T	C	0.027833	-0.030757	0.0474269	0.6200004	0.212846	0.0285799	9.52E-14	-0.144503	0.223666	0.5182347	0.9677723
ENSG00000151876	FBXO4	5	41933600	G	A	0.0526839	0.00349672	0.0314898	0.81	-0.15761	0.0184781	1.47E-17	-0.0221858	0.199812	0.9115898	0.9954954
ENSG00000151882	CCL28	5	43394620	G	A	0.400596	-0.00345933	0.0143477	0.4700002	-0.130994	0.00797837	1.41E-60	0.0264084	0.109542	0.8094924	0.9186509
ENSG00000151883	PARP8	5	50052044	T	G	0.182903	0.0358054	0.0180225	0.03400008	0.22422	0.0100324	1.22E-110	0.159689	0.0806956	0.04782699	0.1719594
ENSG00000151893	CACUL1	10	120474220	C	T	0.232604	-0.00410663	0.0171753	0.8700001	0.266708	0.0106801	1.22E-137	-0.0153975	0.0644004	0.8110358	0.9977856
ENSG00000151914	DST	6	56571105	C	A	0.351889	-0.0173066	0.0151191	0.2	0.108322	0.0086476	5.37E-36	-0.15977	0.140158	0.2543135	0.6528865
ENSG00000151923	TIAL1	10	121345370	C	T	0.196819	0.00594957	0.0169575	0.7199992	-0.0892926	0.0091984	2.80E-22	-0.0666301	0.190033	0.7258713	0.9852066
ENSG00000151929	BAG3	10	121424106	T	C	0.348907	0.00950137	0.0150217	0.4600002	-0.167484	0.00818152	3.91E-93	-0.05673	0.0897332	0.527251	0.670485
ENSG00000151948	GLT1D1	12	129403740	C	G	0.144135	0.00652034	0.0234485	0.7600007	-0.399754	0.0184102	1.52E-104	-0.0163109	0.0586621	0.7809756	0.06455513
ENSG00000151952	TMEM132D	12	129972240	T	C	0.150099	0.000888027	0.0192731	1	0.118203	0.0107478	3.92E-28	0.00751275	0.163053	0.96325	0.8660426
ENSG00000152022	NA	1	145489377	G	A	0.402584	0.0109314	0.0144711	0.5400003	-0.123061	0.0120554	1.83E-24	-0.0888289	0.117914	0.4512484	0.4099136
ENSG00000152056	AP1S3	2	224659573	A	G	0.475149	-0.0123441	0.0142415	0.4299995	0.183147	0.00788741	2.85E-119	-0.0673999	0.077814	0.3863989	0.9847088
ENSG00000152061	RABGAP1L	1	174546496	A	G	0.44831	0.0123389	0.0142632	0.33	0.212962	0.011789	6.08E-73	0.0579396	0.0670522	0.3875351	0.7812889
ENSG00000152078	TLCD4	1	95623028	C	T	0.0795229	0.0162545	0.0257506	0.6200004	0.187989	0.0140492	7.83E-41	0.0864651	0.137132	0.52835	0.9315753
ENSG00000152102	FAM168B	2	131828241	G	C	0.109344	0.0157311	0.0216601	0.3800004	0.11322	0.0124008	6.85E-20	0.138943	0.191915	0.4690756	0.5585372
ENSG00000152117	SMPD4BP	2	132264766	G	A	0.219682	0.0169059	0.0183212	0.4400003	-0.986886	0.0202493	0	-0.0171305	0.018568	0.3562233	0.2204905

ENSG00000152127	MGAT5	2	135044873	T	C	0.11829	-0.0529836	0.025533	0.02199986	0.181987	0.0149011	2.65E-34	-0.29114	0.142312	0.04077774	0.2402213
ENSG00000152128	TMEM163	2	135344950	G	A	0.428429	-0.000272635	0.0150405	0.6200004	-0.218696	0.00812982	2.17E-159	0.00124664	0.0687735	0.9855378	0.1275476
ENSG00000152133	GPATCH11	2	37318990	T	C	0.442346	-0.00728599	0.0143542	0.5999997	-0.0915578	0.00897256	1.90E-24	0.079578	0.156971	0.6121835	0.4851719
ENSG00000152147	GEMIN6	2	38994137	T	C	0.235586	-0.0168451	0.0163115	0.4299995	0.0862349	0.00918311	5.97E-21	-0.19534	0.190292	0.3046445	0.4918091
ENSG00000152193	OBI1	13	79210870	G	A	0.222664	-0.00434148	0.0171154	0.6700003	-0.344321	0.00920353	2.51E-306	0.0126088	0.0497089	0.7997633	0.8970108
ENSG00000152207	CYSLTR2	13	49282224	T	G	0.300199	0.0186054	0.0153827	0.3100002	0.0770687	0.00848195	1.03E-19	0.241413	0.201358	0.2305567	0.4973625
ENSG00000152213	ARL11	13	50205221	G	A	0.0904573	0.0258845	0.0271671	0.3900004	-0.184413	0.013364	2.58E-43	-0.140362	0.147668	0.3418454	0.5302657
ENSG00000152217	SETBP1	18	42454306	T	C	0.0914513	-0.00161008	0.0259758	0.7600007	0.121954	0.0144557	3.27E-17	-0.0132024	0.213002	0.950577	0.8935606
ENSG00000152219	ARL14EP	11	30352186	C	G	0.282306	-0.00723369	0.0160591	0.5199996	-0.249415	0.00966918	1.01E-146	0.0290027	0.064397	0.6524414	0.6137617
ENSG00000152223	EPG5	18	43487407	G	A	0.0944334	0.00315567	0.0245103	0.95	0.184751	0.0155727	1.82E-32	0.0170807	0.132675	0.8975625	0.4089208
ENSG00000152229	PSTPIP2	18	43607870	T	C	0.0944334	0.00320359	0.0244895	0.9599999	0.288946	0.0137695	9.07E-98	0.0110871	0.0847561	0.8959238	0.310546
ENSG00000152234	ATP5F1A	18	43674205	C	T	0.39165	-0.00671042	0.0143505	0.4799997	-0.272547	0.00789071	1.99E-261	0.0246211	0.0526581	0.640095	0.3187341
ENSG00000152240	HAUS1	18	43696298	A	C	0.0536779	-0.0107549	0.030063	0.9400001	0.394189	0.0194201	1.34E-91	-0.0272836	0.0762773	0.7205754	0.9729202
ENSG00000152242	ARK2N	18	43800234	A	G	0.257455	-0.00583626	0.0163063	0.6800001	-0.206801	0.00897097	1.40E-117	0.0282217	0.0788598	0.7204396	0.3392766
ENSG00000152256	PDK1	2	173454962	A	G	0.154076	0.0143694	0.0179201	0.4100001	-0.314131	0.0100777	2.65E-213	-0.0457433	0.0570654	0.4227877	0.7188192
ENSG00000152268	NA	11	14136780	T	C	0.410537	-0.0093875	0.0143575	0.4199997	0.280227	0.00788704	1.69E-276	-0.0334997	0.051244	0.5132866	0.7437702
ENSG00000152270	PDE3B	11	14778809	C	T	0.287276	-0.00906942	0.016026	0.5099998	0.135444	0.00898848	2.61E-51	-0.0669607	0.118405	0.5717198	0.2763762
ENSG00000152284	TCF7L1	2	85449022	C	T	0.44831	-0.00314666	0.014257	0.9299999	-0.12581	0.00797469	4.54E-56	0.0250111	0.113332	0.8253352	0.9954727
ENSG00000152291	TGOLN2	2	85550347	A	G	0.348907	-0.0116035	0.0150795	0.3100002	0.142038	0.0082851	6.99E-66	-0.0816929	0.106272	0.4420629	0.4606716
ENSG00000152315	KCNK13	14	90590155	C	G	0.296223	0.0348233	0.0158726	0.03799969	-0.269069	0.0087653	6.24E-207	-0.129422	0.0591414	0.02864459	0.7894104
ENSG00000152332	UHMK1	1	162483230	T	G	0.44334	-0.00726474	0.0142435	0.58	0.22965	0.00853787	2.33E-159	-0.031634	0.0620338	0.6100884	0.8214129
ENSG00000152348	ATG10	5	81420260	G	A	0.229622	-0.000124007	0.0163096	0.9	0.50595	0.00899509	0	-0.000245097	0.0322356	0.9939335	0.5067507
ENSG00000152359	POC5	5	74991631	T	C	0.186879	0.00464794	0.0173097	0.6700003	0.564987	0.00923427	0	0.00822664	0.0306376	0.7883038	0.8920134
ENSG00000152377	SPOCK1	5	136622527	C	T	0.234592	-0.00376108	0.0170827	0.98	-0.0590733	0.00978294	1.56E-09	0.063668	0.28937	0.8258534	0.8935951
ENSG00000152380	FAM151B	5	79811085	C	T	0.259443	0.00605133	0.0158533	0.64	-0.220278	0.00900131	2.95E-132	-0.0274713	0.0719782	0.7027125	0.9160754
ENSG00000152382	TADA1	1	166835655	G	A	0.372763	0.0150906	0.0145845	0.28	-0.122925	0.00826895	5.50E-50	-0.122763	0.118933	0.3019766	0.2492026
ENSG00000152404	CWF19L2	11	107262821	G	A	0.50497	0.0049421	0.0141734	0.8600001	-0.468243	0.00818064	0	-0.0105546	0.0302699	0.7273281	0.204546
ENSG00000152409	JMY	5	78577525	G	C	0.358847	-0.00546667	0.0146601	0.9	0.351034	0.00878391	0	-0.015573	0.0417644	0.7092391	0.822953
ENSG00000152433	ZNF547	19	57882889	T	G	0.234592	0.0154138	0.0181054	0.5300002	0.109049	0.00966901	1.68E-29	0.141347	0.166502	0.3959247	0.09030935
ENSG00000152439	ZNF773	19	58020527	A	G	0.475149	-0.00671928	0.0142492	0.5500004	0.212913	0.0118183	1.47E-72	-0.0315588	0.0669479	0.6373598	0.3145359
ENSG00000152443	ZNF776	19	58263845	T	C	0.405567	-0.0075321	0.0142703	0.81	0.130544	0.00799197	5.62E-60	-0.0576977	0.109371	0.5978178	0.2837667
ENSG00000152454	ZNF256	19	58455622	G	C	0.153082	0.0190262	0.0198504	0.29	-0.232502	0.0107594	1.47E-103	-0.0818324	0.0854613	0.3382958	0.2206677
ENSG00000152457	DCLRE1C	10	14967894	C	A	0.15507	-0.014074	0.0198944	0.4199997	0.156364	0.0107187	3.35E-48	-0.0900081	0.127381	0.4798121	0.3364773
ENSG00000152463	OLAH	10	15095038	A	G	0.0218688	0.0433447	0.0448828	0.25	0.270856	0.0246857	5.20E-28	0.160029	0.166348	0.3360437	0.9364573
ENSG00000152464	RPP38	10	15143654	G	C	0.234592	-0.0129232	0.0170292	0.35	0.174632	0.00951974	3.67E-75	-0.0740025	0.0975982	0.4483096	0.5381605
ENSG00000152465	NMT2	10	15177637	G	A	0.417495	-0.00588558	0.0143951	0.6200004	-0.562429	0.0111447	0	0.0104646	0.0255954	0.6826523	0.2076491
ENSG00000152475	ZNF837	19	58885706	A	G	0.414513	0.0135708	0.0143186	0.25	-0.120344	0.0112328	8.79E-27	-0.112767	0.119446	0.3451251	0.6038195
ENSG00000152484	USP12	13	27693163	C	T	0.0596421	-0.0137732	0.0306577	0.5400003	-0.358833	0.0289784	3.24E-35	0.0383833	0.0854933	0.653459	0.1223679
ENSG00000152487	NA	10	18944195	C	T	0.371769	0.0384357	0.0147512	0.01499996	0.161836	0.0169192	1.12E-21	0.237498	0.0944703	0.01193727	0.2650767
ENSG00000152492	CCDC50	3	191081662	A	G	0.243539	-0.033873	0.0169824	0.02100003	-0.264942	0.0100426	2.22E-153	0.12785	0.0642814	0.04671025	0.6259221
ENSG00000152495	CAMK4	5	110694967	T	C	0.0735586	-0.0259347	0.0283007	0.3599996	-0.471108	0.017268	6.92E-164	0.0550504	0.0601065	0.3597292	0.5192633
ENSG00000152503	TRIM36	5	114488351	A	G	0.43837	0.00940423	0.0143601	0.4400003	0.229786	0.00792495	7.54E-185	0.040926	0.0625092	0.5126486	0.1921749
ENSG00000152518	ZFP36L2	2	43451644	G	A	0.537773	-0.0176353	0.0142568	0.2099999	0.0718378	0.00806985	5.48E-19	-0.245488	0.200365	0.2204988	0.5662339
ENSG00000152520	PAN3	13	28791059	A	G	0.316103	-0.0205638	0.0154849	0.1900002	0.164549	0.00871568	1.68E-79	-0.124971	0.0943379	0.1852649	0.7734339
ENSG00000152527	PLEKHH2	2	43929769	G	A	0.387674	0.0148073	0.0147202	0.2099999	0.415431	0.00780978	0	0.0356432	0.0354399	0.314542	0.3434804
ENSG00000152556	PFKM	12	48519554	T	C	0.2167	-0.00252076	0.0171087	0.8499999	0.214003	0.0101185	2.78E-99	-0.0117791	0.0799481	0.8828681	0.3730754
ENSG00000152558	TMEM123	11	102304089	G	A	0.0864811	0.00345956	0.0242652	0.89	0.274517	0.0161058	3.83E-65	0.0126024	0.0883955	0.8866314	0.3644223
ENSG00000152580	IGSF10	3	151159834	G	A	0.398608	0.012947	0.0144234	0.4	-0.158215	0.00894175	4.66E-70	-0.0818315	0.0912803	0.3699934	0.09354695
ENSG00000152582	SPEF2	5	35716329	G	A	0.474155	0.00308822	0.0142628	0.98	0.148949	0.00792248	7.44E-79	0.0207334	0.0957626	0.8285915	0.8694998
ENSG00000152601	MBNL1	3	152072593	T	C	0.111332	0.0198359	0.0229229	0.4600002	-0.716823	0.0121469	0	-0.027672	0.0319819	0.3869081	0.9655851

ENSG00000152620	NADK2	5	36217537	G	A	0.452286	-0.0178614	0.0141827	0.3400001	-0.121844	0.00889138	9.66E-43	0.146593	0.116891	0.2098076	0.6746394
ENSG00000152642	GPD1L	3	32178693	G	T	0.261431	0.00293079	0.015941	0.6600001	-0.490324	0.00829495	0	-0.00597725	0.0325113	0.85413	0.8918521
ENSG00000152672	CLEC4F	2	71041753	A	G	0.317097	-0.0245343	0.0148937	0.14	0.899751	0.00672268	0	-0.0272679	0.0165544	0.0995233	0.7621162
ENSG00000152683	SLC30A6	2	32420190	C	A	0.104374	-0.00184086	0.0210494	0.6999999	-0.113648	0.0133693	1.89E-17	0.016198	0.185226	0.9303141	0.6350718
ENSG00000152684	PELO	5	52091827	G	T	0.0308151	-0.0201413	0.0357995	0.7099994	-1.28257	0.023748	0	0.0157039	0.0279138	0.5737185	0.3240473
ENSG00000152689	RASGRP3	2	33725604	C	A	0.304175	-0.0220522	0.0152124	0.1900002	-0.261916	0.00838426	3.15E-214	0.0841957	0.0581437	0.1475998	0.5862099
ENSG00000152700	SAR1B	5	133960897	G	A	0.189861	-0.015944	0.0187894	0.3800004	-0.208447	0.0102278	2.50E-92	0.0764893	0.0902179	0.3965332	0.8448548
ENSG00000152726	NA	10	47921717	A	G	0.197813	0.0260118	0.0202549	0.14	0.71534	0.0691109	4.16E-25	0.0363629	0.0285322	0.2025038	0.273797
ENSG00000152749	GPR180	13	95270528	T	G	0.357853	0.00504744	0.0146132	0.6600001	-0.647144	0.00807146	0	-0.00779957	0.0225813	0.729794	0.6550973
ENSG00000152760	DYNLT5	1	67231306	G	A	0.272366	-0.0124076	0.0158291	0.4199997	0.327293	0.00844504	0	-0.0379097	0.0483735	0.4332237	0.8803332
ENSG00000152763	DNAI4	1	67334569	C	T	0.142147	0.00482799	0.0209437	0.8600001	0.0870922	0.0120901	5.86E-13	0.0554354	0.240601	0.8177776	0.7038825
ENSG00000152766	ANKRD22	10	90596732	A	G	0.467197	-0.00669728	0.0143589	0.7499995	-0.123821	0.00798517	3.14E-54	0.0540883	0.116017	0.6410657	0.7966991
ENSG00000152767	FARP1	13	98948421	A	G	0.271372	-0.00282722	0.0155831	0.83	0.251163	0.00865258	2.96E-185	-0.0112565	0.062045	0.856034	0.386419
ENSG00000152778	IFIT5	10	91177550	T	C	0.276342	-0.00833742	0.0168015	0.5300002	-0.408548	0.00873255	0	0.0204074	0.0411272	0.6197513	0.4665868
ENSG00000152782	PANK1	10	91373980	T	C	0.258449	0.0154539	0.0161026	0.4899999	0.187303	0.00873167	4.47E-102	0.0825075	0.0860569	0.3376819	0.7830325
ENSG00000152784	PRDM8	4	81115258	T	C	0.370775	0.0104221	0.0150987	0.4	-0.201653	0.00836118	1.63E-128	-0.0516833	0.0749052	0.4902054	0.3241085
ENSG00000152795	HNRNPDL	4	83347505	G	A	0.0775348	-0.00116281	0.0271734	0.9699999	0.112145	0.0159116	1.82E-12	-0.0103688	0.242311	0.9658678	0.1268052
ENSG00000152804	HHEX	10	94451674	T	C	0.156064	-0.0188222	0.0192756	0.1900002	-0.27921	0.0103517	3.12E-160	0.0674124	0.0690815	0.3291444	0.739422
ENSG00000152818	UTRN	6	144890503	A	G	0.055666	0.035325	0.0325513	0.17	-0.254193	0.0177827	2.37E-46	-0.138969	0.128426	0.2792098	0.8899954
ENSG00000152904	GGPS1	1	235499256	G	A	0.114314	-0.00754296	0.0216958	0.6300007	0.394555	0.0125437	3.64E-217	-0.0191176	0.0549913	0.7281046	0.8048143
ENSG00000152926	ZNF117	7	64452711	G	A	0.0347913	0.0202848	0.0319526	0.56	-0.462735	0.0210267	2.47E-107	-0.0438368	0.0690804	0.5257047	0.9561117
ENSG00000152939	MARVELD2	5	68725548	T	G	0.05666	0.00819306	0.0320711	0.9299999	-0.126357	0.0192712	5.50E-11	-0.0648405	0.254006	0.7985132	0.7715256
ENSG00000152942	RAD17	5	68687874	A	G	0.0198807	0.0617527	0.0411478	0.07399971	0.450414	0.027814	5.58E-59	0.137102	0.0917471	0.1350842	0.5538781
ENSG00000152944	MED21	12	27197377	C	G	0.245527	0.0012006	0.0160263	0.9299999	-0.173905	0.00883565	3.06E-86	-0.00690377	0.0921562	0.9402833	0.8576019
ENSG00000152952	PLOD2	3	145834333	G	C	0.133201	-0.01459	0.0208697	0.35	0.121325	0.0112842	5.81E-27	-0.120255	0.172378	0.4854108	0.5325362
ENSG00000152953	STK32B	4	5277947	T	G	0.134195	-0.0196407	0.0198179	0.2	0.185532	0.0111397	2.78E-62	-0.105862	0.107006	0.3225122	0.3334855
ENSG00000152969	JAKMIP1	4	6115122	C	T	0.476143	0.000370256	0.0143375	0.9	0.265911	0.0085326	3.27E-213	0.00139241	0.0539185	0.9793975	0.5462639
ENSG00000152990	ADGRA3	4	22432185	T	C	0.284294	0.0105219	0.0157106	0.5999997	-0.123407	0.00872762	2.16E-45	-0.0852619	0.12745	0.5035066	0.4585647
ENSG00000153006	SREK1IP1	5	64040425	G	A	0.471173	0.0112871	0.0142181	0.6100002	-0.0933188	0.0086549	4.18E-27	-0.120952	0.152773	0.428529	0.9733112
ENSG00000153012	LGI2	4	25016485	T	A	0.133201	0.00830437	0.0199489	0.8800001	-0.169281	0.0113009	1.00E-50	-0.0490568	0.117891	0.6773211	0.5720628
ENSG00000153015	CWC27	5	64189673	G	A	0.338966	-0.0107592	0.0149724	0.81	-0.0808058	0.00850516	2.08E-21	0.133149	0.185818	0.4736484	0.8779788
ENSG00000153029	MR1	1	181017070	A	C	0.284294	-0.00895862	0.0156839	0.64	-0.249898	0.0087488	1.90E-179	0.0358491	0.0627737	0.5679425	0.8683694
ENSG00000153037	SRP19	5	112212830	C	A	0.430417	-0.00817125	0.0144081	0.56	0.118915	0.00869477	1.40E-42	-0.0687151	0.121267	0.5709575	0.5475662
ENSG00000153044	CENPH	5	68495779	T	C	0.442346	0.00210736	0.0143004	0.91	-0.0513663	0.00799599	1.33E-10	-0.0410262	0.278474	0.8828755	0.611814
ENSG00000153048	CARHSP1	16	8954832	G	A	0.450298	-0.00394797	0.0142609	0.7800007	-0.431623	0.00760917	0	0.0091468	0.0330405	0.7819064	0.4550463
ENSG00000153064	BANK1	4	102664206	C	A	0.420477	0.013282	0.0143138	0.3100002	-0.0754207	0.0080363	6.29E-21	-0.176106	0.190712	0.3557919	0.3379044
ENSG00000153066	TXNDC11	16	11804835	C	T	0.421471	-0.00180782	0.0143434	0.7600007	-0.185675	0.0118777	4.39E-55	0.00973646	0.0772524	0.8997047	0.9010281
ENSG00000153071	DAB2	5	39417091	T	A	0.435388	0.0138013	0.0144235	0.4	-0.388578	0.00849347	0	-0.0355174	0.0371268	0.3387425	0.4318633
ENSG00000153093	ACOXL	2	111682974	T	C	0.0198807	0.0176613	0.0523207	0.7300002	0.274027	0.0278754	8.33E-23	0.064451	0.191045	0.7358457	0.1495065
ENSG00000153094	BCL2L11	2	111901489	G	T	0.117296	0.0603408	0.0236107	0.008999948	0.164709	0.0127383	3.04E-38	0.366349	0.146121	0.01217082	0.6156939
ENSG00000153107	ANAPC1	2	112583057	C	T	0.337972	0.0230445	0.0149468	0.16	0.141321	0.0136463	3.93E-25	0.163065	0.106931	0.1272688	0.4993664
ENSG00000153113	CAST	5	95990412	T	G	0.151093	-0.0119946	0.0195544	0.4299995	0.932519	0.010288	0	-0.0128626	0.0209699	0.5396235	0.3788009
ENSG00000153130	SCOC	4	141241075	T	C	0.0377734	-0.0190606	0.0378903	0.4199997	0.474902	0.0208102	2.86E-115	-0.0401359	0.0798049	0.6150172	0.1819888
ENSG00000153140	CETN3	5	89696840	G	C	0.256461	-0.0354338	0.0159511	0.03799969	-0.115162	0.00918489	4.61E-36	0.307687	0.140667	0.02871791	0.1679974
ENSG00000153157	SYCP2L	6	10863790	C	T	0.184891	0.00201733	0.0185227	0.8800001	0.709025	0.00936182	0	0.00284522	0.0261242	0.913273	0.01416806
ENSG00000153162	BMP6	6	7804342	C	T	0.457256	0.010833	0.0142975	0.3700002	0.120538	0.00793933	4.63E-52	0.089872	0.118762	0.4492038	0.8896158
ENSG00000153179	RASSF3	12	65047820	T	C	0.260437	0.013877	0.0163647	0.4700002	0.122285	0.00908196	2.53E-41	0.113481	0.134089	0.3973815	0.1353896
ENSG00000153201	RANBP2	2	109369102	C	T	0.237575	-0.010621	0.0166868	0.56	0.135143	0.0110719	2.89E-34	-0.0785907	0.123643	0.5250192	0.5673003
ENSG00000153207	AHCTF1	1	247048840	A	G	0.128231	0.0225676	0.0223707	0.33	-0.470225	0.0126831	7.13E-301	-0.0479932	0.0475921	0.3132488	0.2805974
ENSG00000153208	MERTK	2	112721597	T	C	0.256461	-0.0122842	0.0165093	0.7499995	-0.547897	0.00859779	0	0.0224206	0.0301342	0.4568602	0.2973225

ENSG00000153214	TMEM87B	2	112844847	A	G	0.172962	-0.00264198	0.0188292	0.7800007	-0.351616	0.0118513	1.93E-193	0.00751383	0.0535511	0.888414	0.3746272
ENSG00000153234	NR4A2	2	157189902	A	G	0.302187	0.000361547	0.0157184	0.98	-0.0888712	0.00876303	3.61E-24	-0.00406821	0.176868	0.9816491	0.6305051
ENSG00000153250	RBMS1	2	161239483	C	A	0.185885	0.00179398	0.0177186	0.81	-0.0879321	0.0102356	8.64E-18	-0.0204019	0.201517	0.9193589	0.5075157
ENSG00000153253	SCN3A	2	166002304	T	C	0.107356	-0.0377199	0.0240784	0.14	0.0752185	0.0125632	2.13E-09	-0.501471	0.330889	0.1296389	0.05601169
ENSG00000153283	CD96	3	111322761	C	T	0.17992	0.00122268	0.0184268	0.9699999	0.427366	0.00986179	0	0.00286097	0.0431172	0.9470966	0.03212064
ENSG00000153291	SLC25A27	6	46633303	T	C	0.552684	-9.97E-05	0.0141857	0.9299999	0.124469	0.00813459	7.51E-53	-0.000801397	0.11397	0.9943896	0.7745453
ENSG00000153310	CYRIB	8	130940607	T	C	0.233598	0.0106582	0.0170732	0.59	0.0772634	0.00988099	5.31E-15	0.137946	0.221677	0.5337545	0.6667518
ENSG00000153317	ASAP1	8	131260129	G	C	0.197813	0.00437862	0.0173427	0.7800007	-0.45093	0.00966922	0	-0.0097102	0.0384604	0.8006758	0.4546452
ENSG00000153339	TRAPPC8	18	29471117	G	T	0.161034	-0.0339813	0.0191468	0.04799986	-0.189817	0.0117817	2.13E-58	0.179021	0.10148	0.07771418	0.02846398
ENSG00000153347	FAM81B	5	94756603	T	C	0.0447316	-0.129878	0.0450931	0.001700004	0.32571	0.0286027	4.83E-30	-0.398754	0.142805	0.005233692	0.8546621
ENSG00000153363	LINC00467	1	211581011	T	A	0.205765	0.0301307	0.0172632	0.0659994	0.340442	0.0126349	6.62E-160	0.0885048	0.0508145	0.08155721	0.7317171
ENSG00000153391	INO80C	18	33055074	G	C	0.406561	0.0146704	0.014707	0.35	0.255174	0.00801953	3.52E-222	0.0574917	0.0576635	0.318754	0.4938351
ENSG00000153395	LPCAT1	5	1490343	A	C	0.44334	-0.0211447	0.0142572	0.16	0.147517	0.00912162	7.92E-59	-0.143338	0.0970537	0.1397046	0.143066
ENSG00000153406	NMRAL1	16	4528722	T	C	0.277336	0.0271782	0.0161334	0.06900014	0.598072	0.00846417	0	0.045443	0.0269833	0.09215971	0.7734584
ENSG00000153443	UBALD1	16	4661956	G	C	0.181909	0.0187194	0.0184231	0.3599996	-0.0646694	0.0113766	1.31E-08	-0.289463	0.289397	0.3171995	0.5538631
ENSG00000153487	ING1	13	111369252	G	A	0.249503	-0.00141907	0.0175984	0.8800001	-0.297584	0.00954051	1.38E-213	0.00476864	0.0591378	0.9357314	0.4003963
ENSG00000153495	TEX29	13	111982563	T	C	0.148111	0.0187948	0.0193073	0.3800004	0.152244	0.0122127	1.14E-35	0.123452	0.127204	0.3317968	0.4224769
ENSG00000153531	ADPRHL1	13	114092049	A	C	0.148111	0.0300625	0.0213892	0.1499999	-0.108926	0.0119738	9.29E-20	-0.275991	0.198695	0.1648272	0.9749764
ENSG00000153551	CMTM7	3	32478861	A	T	0.296223	0.012768	0.0153844	0.2999998	-0.236833	0.00874105	1.15E-161	-0.0539113	0.0649892	0.4067975	0.3495929
ENSG00000153560	UBP1	3	33456345	G	A	0.026839	0.0752795	0.0417271	0.07399971	0.173896	0.0238305	2.94E-13	0.4329	0.247179	0.07988376	0.4384302
ENSG00000153561	RMND5A	2	86976230	A	C	0.348907	-0.00563373	0.0151105	0.5199996	0.194031	0.0100119	1.14E-83	-0.0290352	0.0778911	0.7093223	0.5629642
ENSG00000153563	CD8A	2	87023624	T	C	0.217694	-0.00302221	0.0163403	0.56	-0.196244	0.00906069	5.02E-104	0.0154003	0.0832684	0.8532701	0.774006
ENSG00000153574	RPIA	2	89020807	T	C	0.028827	0.021422	0.0393483	0.7300002	-0.549429	0.0201254	4.21E-164	-0.0389895	0.0716309	0.586227	0.736759
ENSG00000153575	NA	15	22853643	G	A	0.0934394	0.0120021	0.0228633	0.59	-0.23373	0.0133154	5.60E-69	-0.0513502	0.0978628	0.5997807	0.1221257
ENSG00000153714	LURAP1L	9	12798575	T	C	0.116302	0.0103235	0.0223714	0.8499999	-0.275566	0.0234193	5.80E-32	-0.0374629	0.0812458	0.6447231	0.9555261
ENSG00000153721	CNKSR3	6	154779113	G	A	0.149105	-0.0184087	0.0199516	0.2399999	-0.262305	0.0109629	1.62E-126	0.0701805	0.0761191	0.3565377	0.02519651
ENSG00000153767	GTF2E1	3	120481700	C	T	0.303181	-0.00261646	0.0153352	0.9	0.099046	0.00864789	2.27E-30	-0.0264166	0.154846	0.8645391	0.3181684
ENSG00000153774	CFDP1	16	75397489	G	A	0.245527	0.0118864	0.0173409	0.4899999	0.417678	0.0097656	0	0.0284583	0.0415228	0.4931126	0.1010635
ENSG00000153786	ZDHHC7	16	85026464	A	G	0.236581	-0.00662293	0.0161969	0.8200001	0.438664	0.00893713	0	-0.015098	0.0369246	0.6826227	0.2006718
ENSG00000153790	SPMIP4	7	25197145	C	T	0.0218688	-0.0756716	0.0463157	0.1199999	-0.262072	0.0271634	5.01E-22	0.288743	0.179245	0.1072043	0.1547328
ENSG00000153814	JAZF1	7	28045277	C	G	0.353877	0.0151756	0.0146258	0.1900002	0.463915	0.00779987	0	0.0327121	0.0315317	0.299534	0.6370937
ENSG00000153815	CMIP	16	81612071	G	C	0.324056	-0.00417233	0.0154509	0.7199992	0.146725	0.0087425	3.25E-63	-0.0284363	0.105318	0.7871579	0.6244583
ENSG00000153823	PID1	2	229925621	C	T	0.16004	0.00277446	0.0195717	0.8600001	-0.559214	0.00989364	0	-0.00496135	0.0349987	0.8872709	0.7893483
ENSG00000153827	TRIP12	2	230709942	T	C	0.162028	0.0100277	0.0191554	0.3400001	0.260213	0.0114272	8.81E-115	0.0385365	0.0736338	0.6007281	0.1122382
ENSG00000153832	FBXO36	2	230832421	C	T	0.512922	0.0166236	0.0142565	0.16	0.0977562	0.0118821	1.92E-16	0.170052	0.147295	0.2482957	0.9484869
ENSG00000153879	CEBPG	19	33868913	C	T	0.220676	0.00657236	0.0178641	0.8200001	-0.0857537	0.0101964	4.09E-17	-0.0766423	0.208518	0.7132034	0.9174057
ENSG00000153885	KCTD15	19	34296752	A	G	0.0506958	0.0176764	0.0303887	0.58	-0.381226	0.0166307	2.74E-116	-0.0463672	0.0797386	0.5609106	0.8807769
ENSG00000153896	ZNF599	19	35256556	C	T	0.0109344	0.0549646	0.0577851	0.2999998	0.265316	0.0350428	3.70E-14	0.207167	0.219509	0.3452867	0.8993812
ENSG00000153898	MCOLN2	1	85427032	C	A	0.295229	-0.0107556	0.0154213	0.6300007	-0.345816	0.00833208	0	0.0311021	0.0446002	0.4855822	0.4962364
ENSG00000153904	DDAH1	1	85914107	T	C	0.0954274	-0.0410946	0.0265967	0.05600025	-0.115576	0.014789	5.50E-15	0.355563	0.234577	0.1295799	0.4115132
ENSG00000153922	CHD1	5	98226574	A	G	0.0974155	-0.0439855	0.0253947	0.04900044	0.0941179	0.0133066	1.52E-12	-0.467345	0.27779	0.09249833	0.6846396
ENSG00000153933	DGKE	17	54928748	A	G	0.109344	-0.0271373	0.0229004	0.1800002	-0.196364	0.0115919	2.29E-64	0.138199	0.116907	0.237156	0.4725894
ENSG00000153936	HS2ST1	1	87491332	C	T	0.206759	0.0207211	0.0172609	0.2	-0.199543	0.00971561	9.77E-94	-0.103843	0.0866497	0.2307538	0.6322887
ENSG00000153944	MSI2	17	55547629	T	G	0.347913	0.0144886	0.0150165	0.32	-0.0710473	0.00836086	1.94E-17	-0.203929	0.212717	0.3377173	0.9183467
ENSG00000153975	ZUP1	6	116973369	T	G	0.290258	-0.00407695	0.0159571	0.5500004	-0.164886	0.0131006	2.51E-36	0.0247258	0.0967962	0.7983819	0.3266037
ENSG00000153976	HS3ST3A1	17	13452125	C	T	0.373757	-0.011427	0.0149099	0.4100001	-0.0714099	0.00996621	7.77E-13	0.16002	0.209984	0.4460262	0.6788915
ENSG00000154001	PPP2R5E	14	63924083	T	C	0.0815109	-0.0402744	0.0271885	0.17	0.199261	0.0230461	5.32E-18	-0.202119	0.138435	0.1442817	0.5009915
ENSG00000154016	GRAP	17	18937468	G	T	0.187873	0.0103885	0.0181892	0.6899999	-0.410309	0.00999175	0	-0.0253187	0.0443347	0.5679452	0.9736971
ENSG00000154025	SLC5A10	17	18889783	G	C	0.185885	0.0113212	0.0182137	0.6300007	-0.0874528	0.00974469	2.85E-19	-0.129455	0.208768	0.5351982	0.8678014
ENSG00000154027	AK5	1	77886693	C	T	0.127237	-0.000659194	0.0191271	0.5999997	0.513102	0.0106992	0	-0.00128472	0.0372774	0.9725073	0.8630553

ENSG00000154035	NA	17	21149452	T	C	0.275348	0.00220632	0.0152802	0.9599999	-0.295418	0.00947465	2.00E-213	-0.00746847	0.0517246	0.8851931	0.9970185
ENSG00000154040	CABYR	18	21730254	A	G	0.158052	-0.0118348	0.0197452	0.5400003	0.105606	0.0109832	6.90E-22	-0.112066	0.187334	0.5496965	0.4019289
ENSG00000154059	IMPACT	18	22020039	A	G	0.0626243	0.018372	0.0263328	0.6100002	0.101952	0.0145463	2.40E-12	0.180203	0.259563	0.4875231	0.2777336
ENSG00000154099	DNAAF1	16	84195619	C	T	0.343936	0.00922175	0.0147359	0.5999997	0.396717	0.0165368	3.54E-127	0.0232451	0.0371572	0.5315847	0.1983772
ENSG00000154102	C16orf74	16	85754212	G	A	0.124254	0.0122528	0.0221042	0.5999997	-0.413353	0.0145366	7.39E-178	-0.0296425	0.0534856	0.5794319	0.6838742
ENSG00000154122	ANKH	5	14788398	T	C	0.537773	0.0264616	0.0142524	0.06199976	0.296699	0.00780273	0	0.0891867	0.0480938	0.06367731	0.1093525
ENSG00000154124	OTULIN	5	14682296	A	G	0.0705765	-0.0183856	0.0277198	0.4600002	0.363457	0.016498	1.47E-107	-0.0505854	0.0763017	0.5073526	0.3589467
ENSG00000154127	UBASH3B	11	122605782	T	G	0.146123	0.0203864	0.0200761	0.28	0.173669	0.011783	3.62E-49	0.117386	0.115874	0.311034	0.830444
ENSG00000154134	ROBO3	11	124743324	C	A	0.447316	0.0152865	0.0143684	0.2	0.511839	0.0113252	0	0.0298658	0.0280799	0.2875088	0.8029525
ENSG00000154144	TBRG1	11	124499009	C	T	0.0178926	0.0127817	0.0481517	0.4600002	0.2275	0.0376905	1.58E-09	0.0561833	0.211861	0.790863	0.6909167
ENSG00000154146	NRGN	11	124613849	A	G	0.0735586	-0.068353	0.0268836	0.007299952	0.168388	0.0148548	8.74E-30	-0.405927	0.16362	0.01310465	0.2814874
ENSG00000154153	RETREG1	5	16545157	G	T	0.245527	-0.00193677	0.016498	0.9	0.178057	0.00898117	1.79E-87	-0.0108772	0.0926573	0.9065493	0.5296001
ENSG00000154165	GPR15	3	98251351	T	C	0.423459	0.00772261	0.014344	0.7400005	-0.0477285	0.00807343	3.38E-09	-0.161803	0.301777	0.591843	0.4150958
ENSG00000154174	TOMM70	3	100101258	A	T	0.248509	-0.00296061	0.0163753	0.6499995	-0.209044	0.00926622	1.08E-112	0.0141626	0.0783367	0.8565312	0.3185019
ENSG00000154175	ABI3BP	3	100590179	C	G	0.336978	-0.0097087	0.0147453	0.3100002	-0.0899644	0.00851517	4.32E-26	0.107917	0.164219	0.5110833	0.4932422
ENSG00000154188	ANGPT1	8	108386002	C	T	0.409543	-0.0132728	0.0143237	0.3400001	-0.369264	0.00784327	0	0.0359439	0.0387973	0.3542108	0.6784678
ENSG00000154217	PITPNC1	17	65533473	G	A	0.382704	0.0109728	0.0145537	0.2399999	-0.234374	0.00813884	2.34E-182	-0.0468174	0.0621173	0.4510328	0.7545594
ENSG00000154222	CC2D1B	1	52821630	G	A	0.0516899	-0.0762006	0.0320451	0.01499996	0.36945	0.0171765	1.28E-102	-0.206254	0.0872659	0.01810238	0.02585198
ENSG00000154227	CERS3	15	101012900	A	G	0.321074	-0.0143578	0.0156543	0.2	0.214133	0.00943797	5.83E-114	-0.067051	0.0731654	0.359441	0.06674328
ENSG00000154229	PRKCA	17	64552807	A	C	0.346918	0.01468	0.0150112	0.2300001	-0.199096	0.00828937	1.80E-127	-0.0737334	0.0754594	0.3285064	0.9657431
ENSG00000154237	LRRK1	15	101534868	T	C	0.297217	-0.00595484	0.0167265	0.8800001	-0.367524	0.0101849	3.90E-285	0.0162026	0.0455135	0.7218443	0.9717112
ENSG00000154240	CEP112	17	63909929	T	C	0.0387674	-0.00169293	0.0344453	0.7600007	-0.224655	0.0207914	3.25E-27	0.00753567	0.153327	0.9608015	0.9954166
ENSG00000154262	ABCA6	17	67106436	A	G	0.297217	-0.00781849	0.0155546	0.64	-0.0586925	0.00858379	8.05E-12	0.133211	0.265734	0.6161632	0.9640588
ENSG00000154263	ABCA10	17	67192171	C	G	0.269384	-0.0114667	0.0157396	0.5099998	0.180876	0.0129683	3.25E-44	-0.0633953	0.0871373	0.4668991	0.8675439
ENSG00000154265	ABCA5	17	67281918	C	T	0.141153	0.0117859	0.020053	0.4400003	0.279033	0.0116423	6.12E-127	0.0422384	0.0718877	0.5568271	0.05880123
ENSG00000154269	ENPP3	6	132009067	G	A	0.084493	-0.0195698	0.0254956	0.2700001	0.2814	0.0127198	1.90E-108	-0.0695444	0.0906572	0.4430137	0.2614097
ENSG00000154274	C4orf19	4	37540340	A	G	0.356859	0.0048471	0.0150237	0.7199992	-0.0664332	0.0083056	1.26E-15	-0.072962	0.226331	0.7471738	0.66592
ENSG00000154277	UCHL1	4	41264451	G	C	0.166998	0.00416212	0.0186999	0.6600001	0.0971314	0.011143	2.86E-18	0.0428504	0.192584	0.8239232	0.8965509
ENSG00000154305	MIA3	1	222816391	A	G	0.163022	0.0126482	0.0189635	0.5700002	-0.0805187	0.0117966	8.76E-12	-0.157084	0.236638	0.5068092	0.7095149
ENSG00000154309	DISP1	1	223083871	G	A	0.523857	-0.00123347	0.0142058	0.8499999	0.232522	0.00940129	4.72E-135	-0.00530475	0.0610948	0.9308082	0.0259951
ENSG00000154310	TNIK	3	170978662	A	G	0.28827	-0.00690149	0.0157585	0.8	0.131435	0.0087656	7.99E-51	-0.0525088	0.119947	0.6615553	0.6969667
ENSG00000154316	TDH	8	11211553	A	G	0.488072	-0.00523967	0.0142146	0.91	0.1037	0.0127861	5.05E-16	-0.050527	0.137215	0.7127005	0.0483748
ENSG00000154319	FAM167A	8	11305598	A	G	0.266402	0.0238733	0.0160392	0.2200002	0.746637	0.0085986	0	0.0319744	0.0214851	0.1366939	0.6693204
ENSG00000154328	NEIL2	8	11636001	G	A	0.393638	0.0212082	0.0144137	0.2700001	0.412658	0.0113824	8.53E-288	0.0513941	0.0349577	0.1415126	0.8095066
ENSG00000154330	PGM5	9	71058896	T	A	0.274354	-0.0100414	0.0172717	0.5999997	0.957467	0.00747736	0	-0.0104875	0.0180391	0.5609892	0.2533651
ENSG00000154358	OBSCN	1	228481204	C	T	0.329026	-0.00182419	0.0147995	0.99	0.175666	0.00832029	6.04E-99	-0.0103844	0.0842493	0.9019027	0.1336508
ENSG00000154359	LONRF1	8	12596492	G	T	0.135189	0.000113168	0.0212435	0.9400001	-0.220313	0.0118492	3.66E-77	-0.000513669	0.0964241	0.9957495	0.1606439
ENSG00000154370	TRIM11	1	228587957	A	G	0.0775348	-0.0355841	0.0228062	0.08600031	0.108873	0.0139702	6.53E-15	-0.326841	0.213633	0.1260365	0.3541395
ENSG00000154429	CCSAP	1	229467899	G	T	0.33002	0.0253399	0.0149847	0.06100002	0.382781	0.00809195	0	0.0661995	0.039172	0.09103331	0.2382243
ENSG00000154447	SH3RF1	4	170103831	C	A	0.474155	-0.00334044	0.0142856	0.7199992	-0.252315	0.00782249	2.98E-228	0.0132391	0.0566195	0.8151197	0.4835573
ENSG00000154451	GBP5	1	89731588	G	A	0.424453	-0.0131066	0.0143681	0.33	0.327245	0.0115767	8.65E-176	-0.0400513	0.043929	0.3619126	0.2115397
ENSG00000154473	BUB3	10	124919339	C	T	0.120278	-0.024973	0.0216443	0.16	0.222365	0.0124361	1.67E-71	-0.112306	0.0975391	0.2495692	0.5620045
ENSG00000154511	DIPK1A	1	93367390	A	G	0.0397614	0.0030589	0.0361345	0.84	0.839786	0.0214894	0	0.00364248	0.0430283	0.9325373	0.8207732
ENSG00000154518	ATP5MC3	2	176045160	G	A	0.206759	-0.0206182	0.0181751	0.1900002	0.123621	0.0101737	5.67E-34	-0.166786	0.147662	0.2586834	0.3701558
ENSG00000154548	SRSF12	6	89816739	C	T	0.436382	-0.0101504	0.0143804	0.4600002	-0.0572968	0.00830196	5.14E-12	0.177155	0.25229	0.4825627	0.4173821
ENSG00000154556	SORBS2	4	186692202	C	G	0.485089	0.0279057	0.0142382	0.03400008	0.118984	0.00797299	2.32E-50	0.234533	0.120692	0.05198827	0.07844288
ENSG00000154589	LY96	8	74922454	G	A	0.150099	-0.0132761	0.0215745	0.3400001	0.563695	0.0109989	0	-0.0235519	0.0382761	0.5383458	0.3053914
ENSG00000154640	BTG3	21	18975618	C	T	0.517893	0.00333543	0.0142198	0.8	-0.117222	0.00792933	1.87E-49	-0.0284538	0.121321	0.8145713	0.5707894
ENSG00000154642	C21orf91	21	19176493	C	T	0.243539	-0.0158938	0.0157833	0.32	-0.137521	0.00896243	3.87E-53	0.115573	0.115017	0.3149741	0.554973
ENSG00000154645	CHODL	21	19456635	C	T	0.361829	0.0153423	0.0146624	0.33	-0.107435	0.00841042	2.29E-37	-0.142806	0.136934	0.2970051	0.912959

ENSG00000154655	L3MBTL4	18	6184970	C	A	0.427435	-0.012567	0.0147144	0.3800004	-0.16671	0.00805182	3.15E-95	0.0753825	0.0883386	0.3934732	0.9553894
ENSG00000154710	RABGEF1	7	66241085	G	T	0.0506958	0.0135028	0.0340793	0.7499995	-0.544924	0.0228026	3.26E-126	-0.0247792	0.0625481	0.6919851	0.4660663
ENSG00000154719	MRPL39	21	26968898	T	G	0.264414	0.0217044	0.0163545	0.32	-0.199368	0.00875903	1.11E-114	-0.108866	0.0821711	0.185214	0.959135
ENSG00000154721	JAM2	21	27050729	G	T	0.121272	0.00194989	0.0213512	0.9699999	0.163369	0.0117016	2.69E-44	0.0119355	0.130696	0.9272362	0.1454003
ENSG00000154734	ADAMTS1	21	28213167	C	T	0.265408	-0.0115806	0.0169434	0.4500005	-0.283741	0.00959673	4.04E-192	0.040814	0.0597303	0.4944142	0.4196036
ENSG00000154736	ADAMTS5	21	28314531	T	C	0.0477137	0.0302452	0.0312735	0.33	0.207698	0.0188112	2.42E-28	0.145621	0.151149	0.3353316	0.5463709
ENSG00000154743	TSEN2	3	12553526	T	G	0.0576541	-0.00904821	0.028815	0.6899999	0.656354	0.0156726	0	-0.0137856	0.0439028	0.7535198	0.8456708
ENSG00000154760	SLFN13	17	33768985	T	C	0.317097	-0.0115546	0.0150623	0.4600002	0.278779	0.0091479	5.64E-204	-0.0414471	0.0540466	0.4431543	0.4460552
ENSG00000154764	WNT7A	3	13889686	G	T	0.345924	0.00857174	0.0153408	0.6100002	-0.128833	0.0086457	3.23E-50	-0.0665337	0.119159	0.5765972	0.1601745
ENSG00000154767	XPC	3	14203465	A	G	0.0248509	-0.050584	0.0500903	0.2	-0.48964	0.0276661	4.33E-70	0.103309	0.102467	0.3133507	0.4485482
ENSG00000154781	CCDC174	3	14703718	T	G	0.349901	0.0248628	0.0149569	0.03799969	0.212006	0.00933698	3.91E-114	0.117274	0.0707384	0.09734583	0.925638
ENSG00000154803	FLCN	17	17128014	C	A	0.150099	0.0409501	0.0206428	0.05399953	0.575056	0.011843	0	0.0712106	0.035927	0.04746873	0.2651522
ENSG00000154813	DPH3	3	16302982	C	T	0.463221	0.00586292	0.0142639	0.7300002	0.204656	0.00791216	1.61E-147	0.0286477	0.0697058	0.6810871	0.3381576
ENSG00000154814	OXNAD1	3	16342764	C	G	0.162028	-0.0130377	0.0203805	0.3900004	0.227525	0.0121591	3.92E-78	-0.0573022	0.089627	0.5226012	0.9778015
ENSG00000154822	PLCL2	3	16988122	G	A	0.227634	0.00967048	0.0172226	0.3900004	0.358582	0.00913631	0	0.0269687	0.0480346	0.5744963	0.5288897
ENSG00000154832	CXXC1	18	47811693	T	C	0.147117	-0.0216205	0.0212741	0.2099999	-0.162371	0.0118857	1.74E-42	0.133155	0.131384	0.3108299	0.196285
ENSG00000154839	SKA1	18	47910954	G	A	0.295229	-0.0109457	0.0153567	0.3900004	-0.0478711	0.0085533	2.18E-08	0.228649	0.323384	0.4795334	0.801963
ENSG00000154845	PPP4R1	18	9581013	G	A	0.475149	-0.0142832	0.0142645	0.29	-0.307802	0.00780984	0	0.0464038	0.046358	0.3168326	0.3713173
ENSG00000154856	APCDD1	18	10471661	C	G	0.157058	0.032678	0.0209413	0.1800002	-0.268532	0.0120654	9.78E-110	-0.121691	0.0781757	0.119557	0.9450464
ENSG00000154874	NA	17	18480415	T	C	0.327038	0.0229363	0.0154572	0.07199959	0.110901	0.0116253	1.43E-21	0.206819	0.141055	0.1425864	0.008906639
ENSG00000154889	MPPE1	18	11895921	A	C	0.11829	0.0151867	0.0209217	0.4	-0.670383	0.0115939	0	-0.0226538	0.031211	0.4679457	0.3219873
ENSG00000154914	USP43	17	9590508	T	C	0.199801	-0.0142259	0.0169888	0.4299995	-0.105029	0.0105179	1.76E-23	0.135447	0.16232	0.4040327	0.2559877
ENSG00000154917	RAB6B	3	133578881	A	G	0.352883	-0.0256246	0.0153142	0.06699926	-0.284731	0.00838148	5.87E-253	0.0899957	0.0538499	0.09467596	0.7891224
ENSG00000154928	EPHB1	3	134647976	G	A	0.332008	0.00427515	0.0148064	0.8	-0.277966	0.00817486	2.05E-253	-0.0153801	0.0532689	0.7727909	0.3790921
ENSG00000154930	ACSS1	20	25013242	G	C	0.230616	-0.00119615	0.0165342	0.89	-0.290013	0.00936435	1.37E-210	0.00412447	0.0570121	0.9423283	0.3809381
ENSG00000154945	ANKRD40	17	48777918	A	G	0.322068	-0.0117129	0.0157328	0.4400003	-0.120062	0.0087033	2.73E-43	0.0975571	0.13123	0.4572351	0.2638364
ENSG00000154957	ZNF18	17	11890794	C	T	0.055666	-0.0404758	0.0271464	0.14	-0.14673	0.0159452	3.51E-20	0.275851	0.187421	0.1410684	0.9577793
ENSG00000154978	VOPP1	7	55572215	A	G	0.0298211	-0.040418	0.043291	0.2700001	-0.755033	0.0261412	1.96E-183	0.0535314	0.0573665	0.3507437	0.6813852
ENSG00000155011	DKK2	4	108023961	T	C	0.455268	0.00563042	0.0142409	0.6300007	-0.0827974	0.00886596	9.74E-21	-0.0680023	0.172151	0.6928312	0.1799224
ENSG00000155016	CYP2U1	4	108863569	T	C	0.0149105	-0.0271175	0.0506255	0.4600002	0.643364	0.0318867	1.57E-90	-0.0421495	0.0787164	0.5923316	0.9169988
ENSG00000155026	RSPH10B	7	5987747	G	C	0.191849	-0.00997477	0.018757	0.6499995	0.202774	0.0204422	3.43E-23	-0.0491915	0.0926347	0.5954003	0.5048031
ENSG00000155034	FBXL18	7	5512197	T	C	0.199801	0.000880848	0.0167756	0.7199992	0.0864396	0.0106178	3.92E-16	0.0101903	0.194077	0.958125	0.3564059
ENSG00000155066	PROM2	2	95948628	T	C	0.235586	-0.00708281	0.0162493	0.6999999	-0.0774191	0.00974022	1.89E-15	0.0914866	0.210203	0.6633954	0.2120774
ENSG00000155085	AK9	6	109913239	C	T	0.219682	0.000237286	0.0169382	0.8700001	0.354693	0.00985732	1.56E-283	0.00066899	0.0477545	0.9888229	0.7171406
ENSG00000155090	KLF10	8	103664568	A	G	0.347913	0.0167573	0.0147101	0.1800002	0.118994	0.00870044	1.40E-42	0.140825	0.124048	0.2562754	0.617467
ENSG00000155093	PTPRN2	7	157856115	T	C	0.0437376	0.0486607	0.0362967	0.16	1.15436	0.0191444	0	0.0421537	0.0314508	0.180146	0.7000375
ENSG00000155096	AZIN1	8	103872338	A	G	0.263419	0.0144636	0.0162315	0.3800004	-0.291662	0.00879066	2.18E-241	-0.0495902	0.0556717	0.3730574	0.02403746
ENSG00000155097	ATP6V1C1	8	104059285	A	G	0.101392	0.0270684	0.0230851	0.28	-0.404657	0.0123332	4.20E-236	-0.0668922	0.057085	0.2412772	0.3148226
ENSG00000155099	PIP4P2	8	92029658	C	T	0.38171	-0.0159861	0.0145725	0.1800002	0.0979718	0.00809912	1.10E-33	-0.16317	0.149352	0.274604	0.1203151
ENSG00000155100	OTUD6B	8	92090873	T	C	0.0188867	-0.04865	0.0401595	0.1499999	0.266315	0.0223516	9.91E-33	-0.182679	0.151575	0.2281238	0.1491325
ENSG00000155111	CDK19	6	111034171	G	T	0.258449	-0.0376541	0.0164701	0.02100003	0.162807	0.00887203	3.26E-75	-0.231281	0.101945	0.02328835	0.0755059
ENSG00000155130	NA	6	114181594	T	A	0.32008	-0.00739418	0.0151386	0.6300007	0.196769	0.012554	2.29E-55	-0.0375779	0.0769731	0.6254116	0.111967
ENSG00000155158	TTC39B	9	15239100	T	C	0.45328	0.00312766	0.0143798	0.6899999	-0.675863	0.00773035	0	-0.00462766	0.0212763	0.8278163	0.5207633
ENSG00000155189	AGPAT5	8	6591531	T	C	0.319085	-0.0148664	0.0150388	0.3800004	0.17256	0.00836848	1.81E-94	-0.0861522	0.0872514	0.3234454	0.102772
ENSG00000155229	MMS19	10	99238316	T	C	0.454274	0.00646259	0.0143366	0.5999997	0.145277	0.00877127	1.29E-61	0.0444847	0.0987213	0.6522715	0.1693883
ENSG00000155252	PI4K2A	10	99390161	C	G	0.357853	-0.0291257	0.0144679	0.07900053	0.0976068	0.00813553	3.66E-33	-0.298398	0.150298	0.04710324	0.1154227
ENSG00000155254	MARVELD1	10	99475418	C	T	0.232604	-0.00939179	0.0155923	0.5400003	0.110164	0.0101392	1.69E-27	-0.0852528	0.141754	0.5475658	0.6110463
ENSG00000155256	ZFYVE27	10	99508771	G	A	0.16998	1.91E-05	0.0173155	0.9	-0.156871	0.00971661	1.24E-58	-0.000121766	0.110381	0.9991198	0.9738138
ENSG00000155265	GOLGA7B	10	99618889	A	G	0.350895	-0.0140324	0.0147231	0.56	-0.176387	0.00900594	2.05E-85	0.0795547	0.0835693	0.3411168	0.7830634
ENSG00000155275	TRMT44	4	8466562	T	C	0.0387674	0.0611617	0.0377447	0.07699987	-0.200176	0.025471	3.87E-15	-0.305539	0.192523	0.1125076	0.3384023

ENSG00000155287	SLC25A28	10	101375324	T	C	0.471173	0.00160393	0.0142423	0.81	0.129616	0.00793167	4.99E-60	0.0123745	0.109883	0.9103361	0.5391662
ENSG00000155307	SAMSN1	21	15906636	T	G	0.489066	-0.0183891	0.014232	0.2999998	-0.106432	0.00794841	6.88E-41	0.172777	0.13434	0.1984006	0.8536823
ENSG00000155324	GRAMD2B	5	125764005	A	G	0.189861	0.0158109	0.0179186	0.4	-0.186606	0.010115	5.37E-76	-0.0847287	0.0961334	0.3781197	0.38493
ENSG00000155363	MOV10	1	113229565	C	G	0.184891	0.0118368	0.0191724	0.6100002	0.273768	0.0107385	2.29E-143	0.0432366	0.070052	0.5370985	0.5581554
ENSG00000155366	RHOC	1	113246892	C	A	0.0397614	-0.0430383	0.0339039	0.1900002	0.515289	0.0208705	1.38E-134	-0.0835226	0.0658828	0.2048887	0.7679529
ENSG00000155368	DBI	2	120127311	G	A	0.199801	-0.00990736	0.0174742	0.5300002	-0.362701	0.0143801	2.28E-140	0.0273155	0.0481902	0.5708314	0.3400927
ENSG00000155380	SLC16A1	1	113477052	A	G	0.43837	0.0128655	0.0142823	0.2099999	0.123125	0.00802809	4.34E-53	0.104491	0.116198	0.3685192	0.8649875
ENSG00000155393	HEATR3	16	50120075	G	A	0.290258	-0.0355005	0.0156209	0.01400006	-0.67809	0.00804247	0	0.0523537	0.023045	0.0230984	0.5385316
ENSG00000155428	TRIM74	7	72435006	A	G	0.0467197	-0.0232853	0.0344749	0.5300002	-0.436144	0.0533653	3.01E-16	0.053389	0.0793142	0.5008628	0.36033
ENSG00000155438	NIFK	2	122489510	A	G	0.0417495	-0.0347783	0.0308255	0.2	0.559002	0.0187026	2.73E-196	-0.0622149	0.055183	0.2595613	0.4431315
ENSG00000155463	OXA1L	14	23238369	G	A	0.521869	0.00139979	0.0142808	0.9400001	-0.12377	0.0118023	9.91E-26	-0.0113096	0.115387	0.9219206	0.08490814
ENSG00000155465	SLC7A7	14	23270730	A	G	0.137177	0.0162732	0.0200788	0.3800004	-0.363026	0.0109102	9.18E-243	-0.0448265	0.0553259	0.41781	0.3643736
ENSG00000155506	LARP1	5	154144814	C	T	0.501988	-0.0233336	0.0142085	0.1199999	0.286829	0.00855271	1.41E-246	-0.0813503	0.0495959	0.1009505	0.6366265
ENSG00000155508	CNOT8	5	154246733	C	T	0.0218688	0.0438927	0.0578149	0.4600002	-0.326961	0.0290549	2.23E-29	-0.134245	0.177227	0.4487673	0.3004266
ENSG00000155530	LRGUK	7	133880697	A	G	0.263419	-0.00780837	0.0164937	0.4500005	-0.521305	0.0181588	3.02E-181	0.0149785	0.0316436	0.6359628	0.01964896
ENSG00000155542	SETD9	5	56213223	T	C	0.267396	0.017785	0.0164557	0.1800002	-0.718787	0.0161314	0	-0.0247431	0.0229004	0.2799362	0.7976309
ENSG00000155561	NUP205	7	135288086	T	C	0.202783	0.0342222	0.0174962	0.04399973	-0.143653	0.010743	8.84E-41	-0.238228	0.123091	0.0529433	0.9079294
ENSG00000155592	ZKSCAN2	16	25258287	T	G	0.307157	-0.0214047	0.0152283	0.17	0.115904	0.00922139	3.13E-36	-0.184677	0.132207	0.1624498	0.1298528
ENSG00000155621	C9orf85	9	74563698	C	G	0.145129	-0.0426013	0.0209475	0.02300011	-0.121305	0.0144301	4.23E-17	0.351191	0.177666	0.04807618	0.7227773
ENSG00000155629	PIK3AP1	10	98416670	G	T	0.0675944	-0.0489171	0.0288757	0.06299992	0.237761	0.0161242	3.28E-49	-0.205741	0.122247	0.09237753	0.6174112
ENSG00000155636	RBM45	2	178989341	G	A	0.355865	-0.00599241	0.0146216	0.8200001	-0.150596	0.00812706	1.18E-76	0.0397913	0.0971152	0.6820022	0.1528316
ENSG00000155657	TTN	2	179543122	G	A	0.138171	0.00566095	0.0209834	0.8700001	-0.0746427	0.0113401	4.64E-11	-0.0758407	0.281354	0.7875019	0.3622274
ENSG00000155660	PDIA4	7	148712943	A	G	0.215706	-0.0288635	0.0180914	0.07799917	0.237272	0.0110002	3.45E-103	-0.121647	0.0764559	0.1115915	0.8162667
ENSG00000155666	KDM8	16	27223948	G	A	0.351889	0.0161375	0.0147972	0.2700001	-0.0975035	0.00916731	2.03E-26	-0.165507	0.152556	0.2779705	0.7765035
ENSG00000155729	KCTD18	2	201369091	A	G	0.0795229	-0.000602835	0.0274626	0.8700001	-0.210614	0.0139746	2.51E-51	0.00286227	0.130393	0.982487	0.4435573
ENSG00000155744	HYCC2	2	201889805	G	A	0.0178926	-0.0481942	0.0561206	0.4700002	0.350852	0.0355437	5.56E-23	-0.137363	0.160559	0.3922579	0.719886
ENSG00000155749	FLACC1	2	202187557	G	A	0.494036	-0.0127546	0.0142007	0.3700002	-0.157265	0.00791604	7.92E-88	0.0811027	0.0903903	0.3695853	0.832313
ENSG00000155755	TMEM237	2	202496600	A	G	0.39165	-0.000501467	0.014519	0.91	0.174891	0.00888897	3.53E-86	-0.00286732	0.0830178	0.9724476	0.9469201
ENSG00000155760	FZD7	2	202901235	T	G	0.292247	0.031932	0.0152448	0.03599979	0.0742146	0.0087377	2.00E-17	0.430266	0.211569	0.04198315	0.6959386
ENSG00000155761	SPAG17	1	118612165	A	G	0.432406	-0.0249685	0.0142154	0.1100001	0.208335	0.0126125	2.71E-61	-0.119848	0.068618	0.08070784	0.5004558
ENSG00000155792	DEPTOR	8	120974554	G	A	0.45825	0.00146946	0.0142411	0.99	-0.201867	0.00858956	3.95E-122	-0.00727936	0.0705478	0.9178174	0.7913541
ENSG00000155827	RNF20	9	104310877	C	T	0.158052	0.00613747	0.019043	0.6200004	-0.222805	0.01121	6.62E-88	-0.0275464	0.0854806	0.7472611	0.9450187
ENSG00000155849	ELMO1	7	37191406	C	T	0.196819	-0.0135091	0.0177518	0.29	0.538525	0.00944692	0	-0.0250854	0.0329667	0.4466982	0.8137808
ENSG00000155850	SLC26A2	5	149356659	G	A	0.444334	0.0217115	0.014396	0.07900053	-0.0549919	0.00813514	1.38E-11	-0.394813	0.26822	0.1410285	0.1988759
ENSG00000155858	LSM11	5	157179210	G	T	0.350895	0.0270949	0.0150603	0.14	-0.0635312	0.00838806	3.62E-14	-0.426482	0.24365	0.08005093	0.9821574
ENSG00000155868	MED7	5	156575226	G	T	0.0119284	0.0480893	0.0922594	0.7099994	-0.347923	0.0637642	4.86E-08	-0.138218	0.266379	0.6038459	0.7457917
ENSG00000155876	RRAGA	9	19050195	T	C	0.399602	-0.00885803	0.014759	0.7300002	-0.0736051	0.00829615	7.17E-19	0.120345	0.200974	0.5492998	0.2718723
ENSG00000155893	PXYLP1	3	140980658	G	A	0.427435	0.00477812	0.0143894	0.64	0.584253	0.00809132	0	0.00817816	0.024629	0.7398479	0.7304878
ENSG00000155903	RASA2	3	141270036	C	T	0.360835	0.00858279	0.0145976	0.4400003	0.123942	0.0124263	1.98E-23	0.0692484	0.117982	0.5572439	0.4755573
ENSG00000155906	RMND1	6	151749624	A	G	0.358847	0.0082332	0.0148658	0.4600002	-0.28382	0.0089034	5.44E-223	-0.0290085	0.0523855	0.579749	0.4193431
ENSG00000155926	SLA	8	134082135	G	T	0.0218688	0.0947075	0.0441187	0.01499996	0.846073	0.0242374	5.68E-267	0.111938	0.0522437	0.03214486	0.957093
ENSG00000155957	TMBIM4	12	66540780	C	T	0.329026	0.0360876	0.0147195	0.01	-0.307117	0.00809872	0	-0.117504	0.048028	0.01442193	0.09273986
ENSG00000155970	MICU3	8	16932450	A	G	0.313121	-0.00713129	0.0152743	0.7600007	0.117469	0.00943115	1.31E-35	-0.0607079	0.13012	0.640819	0.9150414
ENSG00000155974	GRIP1	12	66969588	T	A	0.203777	0.0169113	0.0176865	0.28	-0.116767	0.0108915	8.11E-27	-0.144829	0.152069	0.3408994	0.3089374
ENSG00000155975	VPS37A	8	17132008	T	G	0.500994	0.0112496	0.0142718	0.5700002	-0.116628	0.00795173	1.05E-48	-0.096457	0.122547	0.4312211	0.9784933
ENSG00000156011	PSD3	8	18663525	C	G	0.417495	-0.00871325	0.0143949	0.4400003	-0.0589796	0.00801179	1.82E-13	0.147733	0.24489	0.5463322	0.2370122
ENSG00000156017	CARNMT1	9	77619637	T	G	0.255467	-0.0125261	0.0156975	0.5500004	0.109645	0.00908697	1.59E-33	-0.114242	0.143479	0.4258992	0.9584393
ENSG00000156030	MIDEAS	14	74219406	C	G	0.450298	-0.00256183	0.0143929	0.7300002	-0.0530553	0.00935721	1.43E-08	0.048286	0.271414	0.8587976	0.2720163
ENSG00000156042	CFAP70	10	75066067	C	G	0.0536779	0.0341327	0.0296068	0.32	-0.504193	0.0251245	1.41E-89	-0.0676977	0.058818	0.2497449	0.9813607
ENSG00000156050	FAM161B	14	74407660	C	T	0.135189	-0.0158188	0.0197229	0.5400003	-0.221804	0.012795	2.55E-67	0.071319	0.0890157	0.4230186	0.958261

ENSG00000156052	GNAQ	9	80488688	T	A	0.305169	-0.00675848	0.0159122	0.5300002	-0.146364	0.00894009	3.05E-60	0.0461759	0.108753	0.6711335	0.427413
ENSG00000156110	ADK	10	76190010	C	T	0.16501	0.0133963	0.0203317	0.4100001	1.09738	0.0105148	0	0.0122075	0.0185278	0.5099765	0.9450633
ENSG00000156113	KCNMA1	10	79017854	C	T	0.488072	0.0149199	0.0141916	0.25	0.506407	0.00751141	0	0.0294623	0.0280275	0.2931707	0.7911761
ENSG00000156127	BATF	14	76001063	G	T	0.10338	-0.0414168	0.0233276	0.06900014	0.170751	0.0126495	1.59E-41	-0.242557	0.137795	0.07836014	0.9357081
ENSG00000156136	DCK	4	71877443	A	C	0.026839	0.0309289	0.0532735	0.7499995	-0.622249	0.031012	1.50E-89	-0.049705	0.0856503	0.5616953	0.4561194
ENSG00000156162	DPY19L4	8	95768997	C	T	0.452286	-0.0268189	0.014429	0.03400008	-0.126457	0.00796226	8.44E-57	0.21208	0.114881	0.0648804	0.05996008
ENSG00000156170	NDUFAF6	8	96018339	G	C	0.131213	0.0109802	0.0204487	0.6700003	0.239509	0.0122748	8.65E-85	0.0458446	0.0854098	0.5914334	0.2690732
ENSG00000156171	DRAM2	1	111671396	G	A	0.437376	-0.012852	0.0142146	0.4	0.454732	0.008275	0	-0.0282628	0.0312635	0.3659856	0.4930799
ENSG00000156172	CFAP418	8	96269288	C	T	0.0228628	0.0623521	0.0565932	0.29	0.33839	0.0272979	2.74E-35	0.184261	0.167902	0.2724519	0.6709717
ENSG00000156194	PPEF2	4	76802372	G	C	0.425447	-0.0175029	0.0144412	0.28	0.0760397	0.00835289	8.75E-20	-0.230181	0.191592	0.2295921	0.1718935
ENSG00000156206	CFAP161	15	81370445	C	T	0.252485	0.0145543	0.0166591	0.5	0.298739	0.00882408	3.06E-251	0.0487191	0.0557833	0.3824648	0.5645823
ENSG00000156232	WHAMM	15	83490995	T	C	0.463221	0.00676463	0.0142047	0.6800001	-0.246604	0.00860334	1.08E-180	-0.0274312	0.0576093	0.6339611	0.2099724
ENSG00000156239	N6AMT1	21	30251103	T	G	0.121272	-0.044785	0.0225161	0.01700004	0.141191	0.0111684	1.24E-36	-0.317195	0.161435	0.04943151	0.3538876
ENSG00000156253	RWDD2B	21	30384202	T	C	0.287276	-0.0139799	0.01624	0.3400001	0.402843	0.00881393	0	-0.0347031	0.0403207	0.3894151	0.1117523
ENSG00000156256	USP16	21	30411879	C	T	0.290258	0.0128885	0.0164026	0.4400003	0.0590623	0.00897521	4.69E-11	0.218219	0.27969	0.435263	0.9650858
ENSG00000156261	CCT8	21	30437122	A	G	0.132207	0.0291462	0.0220521	0.1199999	0.992612	0.0114144	0	0.0293631	0.0222188	0.1863198	0.2609609
ENSG00000156265	MAP3K7CL	21	30499001	G	A	0.338966	-0.00457047	0.0146445	0.81	0.271694	0.00895293	2.76E-202	-0.0168221	0.0539036	0.7549811	0.5347757
ENSG00000156273	BACH1	21	30784731	A	G	0.506958	0.00123545	0.0142374	0.8499999	0.0729063	0.00795104	4.76E-20	0.0169457	0.195292	0.9308535	0.2573064
ENSG00000156299	TIAM1	21	32711512	A	G	0.240557	-0.00611006	0.0165143	0.83	-0.387817	0.00950532	0	0.015755	0.0425845	0.7114042	0.9962079
ENSG00000156304	SCAF4	21	33073872	C	T	0.082505	0.0214051	0.0286585	0.5300002	-0.143591	0.0158829	1.56E-19	-0.14907	0.200264	0.4566549	0.5618644
ENSG00000156345	CDK20	9	90585525	A	C	0.0506958	-0.0343908	0.032919	0.4400003	0.129326	0.0195422	3.65E-11	-0.265923	0.257695	0.3021047	0.2484016
ENSG00000156374	PCGF6	10	105086722	G	A	0.516899	-0.0236943	0.0142046	0.05099998	0.197054	0.0130653	2.12E-51	-0.120243	0.0725245	0.09732494	0.5073825
ENSG00000156381	ANKRD9	14	102974657	T	C	0.257455	0.0132366	0.0167612	0.4400003	-0.354984	0.00920624	0	-0.0372878	0.0472266	0.4297899	0.408646
ENSG00000156384	SFR1	10	105883979	G	A	0.163022	0.0503113	0.0190523	0.007399971	0.179785	0.0132244	4.29E-42	0.279841	0.107953	0.009535174	0.1110319
ENSG00000156395	SORCS3	10	106712926	T	C	0.0516899	-0.0489255	0.0298578	0.09599973	0.233838	0.0167242	2.01E-44	-0.209228	0.12856	0.1036355	0.3903366
ENSG00000156398	SFXN2	10	104486623	T	C	0.167992	0.0567102	0.019614	0.0025	-0.352404	0.0101773	1.01E-262	-0.160924	0.0558514	0.003960571	0.7367299
ENSG00000156411	ATP5MJ	14	104386615	G	A	0.0337972	-0.0121076	0.0466541	0.6200004	-0.688435	0.0241926	4.06E-178	0.0175871	0.0677712	0.7952436	0.8828717
ENSG00000156414	TDRD9	14	104456901	T	G	0.285288	-0.00944353	0.0157927	0.3900004	0.56052	0.00812934	0	-0.0168478	0.0281761	0.5498763	0.4464656
ENSG00000156453	PCDH1	5	141245874	C	T	0.168986	0.0260703	0.0192917	0.16	-0.115042	0.0109138	5.59E-26	-0.226615	0.169064	0.1801142	0.384134
ENSG00000156467	UQCRB	8	97243005	A	G	0.362823	0.0106813	0.014597	0.58	0.130167	0.00810637	5.07E-58	0.0820583	0.112257	0.4647867	0.1380182
ENSG00000156469	MTERF3	8	97262732	T	C	0.0606362	-0.0605189	0.0314431	0.04499974	-0.14626	0.0218285	2.08E-11	0.413777	0.223675	0.06432761	0.3151956
ENSG00000156471	PTDSS1	8	97311583	G	A	0.456262	0.00412022	0.0143072	0.59	-0.0631165	0.00806003	4.85E-15	-0.0652796	0.226833	0.7735092	0.2520878
ENSG00000156475	PPP2R2B	5	146216141	G	A	0.336978	-0.000385668	0.0148203	0.84	-0.1892	0.00814303	2.04E-119	0.00203841	0.0783315	0.979239	0.5028803
ENSG00000156482	RPL30	8	99047888	A	G	0.440358	-0.018146	0.0143893	0.1299999	-0.0627045	0.00870823	5.99E-13	0.289389	0.232971	0.2141739	0.7983267
ENSG00000156502	SUPV3L1	10	70954421	C	T	0.342942	0.00758897	0.0152642	0.4899999	0.342297	0.00827446	0	0.0221707	0.0445967	0.6190914	0.8914798
ENSG00000156508	EEF1A1	6	74229496	C	T	0.306163	0.0220915	0.0153507	0.16	0.533512	0.00969572	0	0.0414077	0.0287828	0.1502559	0.2491483
ENSG00000156510	HKDC1	10	71003687	A	T	0.341948	0.00957297	0.015244	0.4	0.434472	0.00815831	0	0.0220336	0.0350887	0.5300438	0.9023805
ENSG00000156515	HK1	10	71095689	A	G	0.253479	0.00493646	0.0161163	0.7800007	-0.177888	0.00928287	7.54E-82	-0.0277504	0.0906098	0.7594043	0.2020124
ENSG00000156521	TYSND1	10	71902084	G	T	0.412525	-0.0149672	0.0142774	0.35	-0.294231	0.00787229	9.77E-306	0.0508689	0.0485436	0.2946838	0.07903424
ENSG00000156535	CD109	6	74471774	A	G	0.431412	-0.000928272	0.0143104	0.7400005	-0.124259	0.00797572	1.00E-54	0.00747049	0.115167	0.9482804	0.4102421
ENSG00000156564	LRFN2	6	40457264	A	G	0.33996	0.017995	0.0152377	0.1900002	0.188164	0.00924519	4.40E-92	0.0956349	0.0811173	0.2384103	0.5216577
ENSG00000156574	NODAL	10	72199889	G	C	0.266402	0.00257969	0.0160226	0.6700003	-0.0604701	0.00941521	1.34E-10	-0.0426606	0.265051	0.8721307	0.7643707
ENSG00000156587	UBE2L6	11	57327443	G	A	0.235586	0.0182584	0.0165583	0.1499999	0.164065	0.00956414	5.85E-66	0.111288	0.101133	0.2711568	0.4372028
ENSG00000156599	ZDHHC5	11	57451939	A	G	0.312127	0.0143747	0.0151117	0.1800002	0.251855	0.00847352	3.92E-194	0.0570753	0.0600323	0.3417348	0.2675368
ENSG00000156603	MED19	11	57475439	A	G	0.246521	-0.0156222	0.0171127	0.2999998	0.524899	0.00871804	0	-0.0297623	0.0326056	0.3613498	0.2075419
ENSG00000156639	ZFAND3	6	37954837	C	T	0.083499	0.00925521	0.0247063	0.6999999	0.370603	0.0140157	4.51E-154	0.0249734	0.0666719	0.707979	0.4517979
ENSG00000156650	KAT6B	10	76688860	C	T	0.241551	0.00584498	0.0165751	0.5400003	-0.070729	0.0104914	1.57E-11	-0.082639	0.234667	0.7247222	0.8974092
ENSG00000156671	SAMD8	10	76900612	T	C	0.054672	0.00189883	0.0320087	0.81	0.137155	0.0175975	6.49E-15	0.0138444	0.233383	0.9526968	0.9671783
ENSG00000156675	RAB11FIP1	8	37736560	T	G	0.261431	0.0504837	0.0162523	0.002	-0.0584033	0.0105884	3.47E-08	-0.864399	0.31937	0.006798159	0.2283017
ENSG00000156689	GLYATL2	11	58636615	G	A	0.28827	0.0169866	0.0152618	0.1299999	0.223705	0.00899426	1.50E-136	0.075933	0.0682911	0.2661805	0.1088171

ENSG00000156711	MAPK13	6	36101714	A	G	0.387674	-0.0304671	0.0145823	0.02800013	-0.132399	0.00821883	2.20E-58	0.230116	0.111062	0.03826856	0.001208858
ENSG00000156735	BAG4	8	38052435	A	G	0.258449	0.00150495	0.0166645	0.9199999	-0.299819	0.0107521	4.08E-171	-0.00501953	0.0555822	0.9280423	0.1812366
ENSG00000156738	MS4A1	11	60230729	A	G	0.00695825	-0.0156348	0.0526037	0.8200001	-0.279342	0.0467377	2.28E-09	0.0559701	0.188546	0.7665796	0.5186357
ENSG00000156787	TBC1D31	8	124109300	T	C	0.45825	0.00925549	0.0142551	0.4100001	-0.154382	0.00871458	3.19E-70	-0.0599518	0.0923984	0.5164418	0.2211421
ENSG00000156795	NTAQ1	8	124454217	C	T	0.311133	-0.00721533	0.014916	0.7300002	-0.515938	0.0078376	0	0.0139849	0.0289112	0.6285861	0.2456781
ENSG00000156804	FBXO32	8	124531787	A	G	0.435388	0.000354729	0.0142773	0.9400001	0.153964	0.00794447	1.14E-83	0.00230398	0.0927316	0.9801781	0.178512
ENSG00000156831	NSMCE2	8	126241641	C	T	0.055666	0.0429996	0.0397611	0.4	-0.163476	0.0204513	1.31E-15	-0.263034	0.245439	0.2838614	0.5164069
ENSG00000156853	ZNF689	16	30624606	A	G	0.127237	0.0196163	0.0211736	0.1100001	-0.230707	0.0130047	2.05E-70	-0.0850269	0.0919021	0.354867	0.2943304
ENSG00000156858	PRR14	16	30664899	A	G	0.27833	0.0142044	0.0162167	0.4299995	-0.114802	0.00877567	4.18E-39	-0.12373	0.141575	0.3821428	0.4918783
ENSG00000156869	FRRS1	1	100203223	T	C	0.243539	-0.0146696	0.016462	0.29	0.0830436	0.00993867	6.51E-17	-0.176649	0.199357	0.3755669	0.7990878
ENSG00000156873	PHKG2	16	30766040	C	T	0.218688	0.0236201	0.0169538	0.06199976	-0.092723	0.0101027	4.39E-20	-0.254738	0.184938	0.168381	0.7948749
ENSG00000156875	MFSD14A	1	100526293	G	T	0.0139165	0.00855533	0.066454	0.95	-0.328131	0.0342702	1.02E-21	-0.0260729	0.202541	0.8975721	0.7720886
ENSG00000156876	SASS6	1	100573815	C	T	0.027833	-0.0108846	0.0343422	0.6600001	0.48515	0.0203629	1.83E-125	-0.0224355	0.070793	0.7513064	0.8867625
ENSG00000156886	ITGAD	16	31421229	T	C	0.12326	0.0111931	0.0197445	0.4600002	0.419373	0.0113243	3.37E-300	0.0266901	0.0470865	0.5708292	0.7742344
ENSG00000156928	MALSU1	7	23344853	G	C	0.388668	-0.00926407	0.0144498	0.4500005	-0.13033	0.0157445	1.25E-16	0.0710815	0.111203	0.5226885	0.9210721
ENSG00000156931	VPS8	3	184650166	A	G	0.0924453	-0.00479238	0.0245016	0.5099998	-0.405417	0.0129456	2.74E-215	0.0118209	0.0604368	0.8449305	0.4189219
ENSG00000156958	GALK2	15	49553959	A	G	0.358847	0.00769291	0.0147198	0.7099994	-0.198185	0.00814163	7.01E-131	-0.0388168	0.0742901	0.6013203	0.6127087
ENSG00000156966	B3GNT7	2	232263064	C	T	0.279324	-0.0225614	0.0168904	0.17	-0.0805366	0.00913652	1.20E-18	0.280139	0.212118	0.1866095	0.1402447
ENSG00000156973	PDE6D	2	232624058	C	T	0.349901	-0.00797263	0.0151733	0.4600002	-0.174057	0.00849217	2.33E-93	0.0458047	0.0872029	0.5993986	0.2419772
ENSG00000156976	EIF4A2	3	186504341	T	C	0.176938	-0.0166199	0.018914	0.35	-0.125374	0.0103073	4.86E-34	0.132562	0.151253	0.3807991	0.8430518
ENSG00000156983	BRPF1	3	9781557	T	C	0.0258449	0.0486286	0.0545511	0.3700002	0.406537	0.0340181	6.45E-33	0.119617	0.134558	0.3740233	0.6912511
ENSG00000156990	RPUSD3	3	9882909	T	C	0.055666	-0.0088048	0.0280074	0.83	-0.521931	0.0164634	1.44E-220	0.0168697	0.0536638	0.7532487	0.336669
ENSG00000157014	TATDN2	3	10308593	G	T	0.265408	0.0277014	0.0163439	0.08999948	0.338252	0.00900287	6.255446e-309	0.0818957	0.0483678	0.09042014	0.1554099
ENSG00000157017	GHRL	3	10330995	G	T	0.261431	-0.00589453	0.0165868	0.59	-0.221448	0.0130074	5.38E-65	0.0266181	0.0749179	0.7223668	0.1354165
ENSG00000157020	SEC13	3	10348838	C	T	0.358847	-0.0158175	0.014663	0.4199997	0.335025	0.00891957	9.423221e-309	-0.0472129	0.0437849	0.2809039	0.540063
ENSG00000157036	EXOG	3	38560635	A	G	0.0357853	-0.0162129	0.0336004	0.6499995	-0.156743	0.0206176	2.91E-14	0.103436	0.214798	0.6301242	0.9250303
ENSG00000157045	NTAN1	16	15140815	C	G	0.408549	-0.015186	0.0144566	0.28	0.255967	0.0118586	2.48E-103	-0.0593279	0.0565451	0.2940802	0.6525869
ENSG00000157077	ZFYVE9	1	52710202	A	G	0.0815109	-0.0660149	0.0231479	0.003699985	0.0734891	0.0133219	3.46E-08	-0.898295	0.354587	0.01129767	0.2962533
ENSG00000157087	ATP2B2	3	10557711	T	A	0.513917	0.00237643	0.0142052	0.9199999	0.0622889	0.00827496	5.18E-14	0.0381517	0.22811	0.867172	0.5057562
ENSG00000157106	SMG1	16	18876975	A	G	0.128231	-0.00480063	0.0195164	0.58	0.181089	0.0109966	6.25E-61	-0.0265097	0.107784	0.8057195	0.9853319
ENSG00000157107	FCHO2	5	72319078	A	G	0.44334	0.0114532	0.0142448	0.3900004	0.560025	0.00733063	0	0.0204512	0.0254374	0.4214073	0.1539101
ENSG00000157110	RBPMS	8	30335861	C	G	0.195825	0.0212517	0.0171264	0.16	-0.161295	0.00977816	3.96E-61	-0.131757	0.106481	0.2159469	0.4481636
ENSG00000157111	TMEM171	5	72421881	G	A	0.168986	0.0328924	0.0200793	0.05399953	-0.358973	0.0109577	2.20E-235	-0.0916291	0.0560053	0.1018227	0.4865095
ENSG00000157152	SYN2	3	12139388	A	G	0.240557	0.00430564	0.0163583	0.8800001	-0.195456	0.00907092	5.58E-103	-0.0220286	0.0836991	0.7924052	0.3269455
ENSG00000157168	NRG1	8	32059725	G	T	0.479125	0.0350576	0.0142869	0.01700004	-0.710169	0.00689393	0	-0.0493652	0.0201233	0.01416182	0.1706403
ENSG00000157181	ODR4	1	186367700	G	A	0.33002	0.00626677	0.0149955	0.6999999	0.0811697	0.00844779	7.37E-22	0.0772058	0.184917	0.6763015	0.5877395
ENSG00000157184	CPT2	1	53670985	G	T	0.207753	-0.00367176	0.0170132	0.89	-0.262005	0.00994788	7.08E-153	0.0140141	0.0649368	0.829135	0.9520505
ENSG00000157191	NECAP2	1	16776870	G	T	0.305169	-0.00535107	0.0159371	0.6100002	0.487364	0.00838554	0	-0.0109796	0.0327011	0.7370547	0.8203171
ENSG00000157193	LRP8	1	53752479	G	C	0.291252	-0.0122673	0.0160146	0.4299995	-0.102719	0.00948727	2.56E-27	0.119426	0.156297	0.4448093	0.02424269
ENSG00000157212	PAXIP1	7	154765095	T	C	0.187873	-0.0181905	0.0176974	0.29	0.0643259	0.0101438	2.28E-10	-0.282787	0.278712	0.3102865	0.9715927
ENSG00000157216	SSBP3	1	54785671	A	G	0.161034	0.00569978	0.0184173	0.8200001	-0.0815173	0.0105494	1.10E-14	-0.0699211	0.226112	0.7571452	0.1564618
ENSG00000157224	CLDN12	7	90077875	T	C	0.21173	0.00824443	0.0166994	0.4799997	0.0839469	0.00978402	9.49E-18	0.09821	0.199257	0.6220967	0.715134
ENSG00000157227	MMP14	14	23312001	C	G	0.336978	-0.0369242	0.0151736	0.00659994	0.0850819	0.00925265	3.74E-20	-0.433984	0.18448	0.01864926	0.7903294
ENSG00000157240	FZD1	7	90895953	G	T	0.194831	-0.00338778	0.0169566	0.8	-0.413467	0.00927083	0	0.00819359	0.0410112	0.8416454	0.4717028
ENSG00000157259	GATAD1	7	92082458	T	G	0.147117	0.0135807	0.021985	0.4600002	-0.228188	0.0122269	9.94E-78	-0.0595153	0.0963986	0.5369791	0.05508153
ENSG00000157303	SUSD3	9	95834204	A	C	0.434394	0.0027441	0.0142573	0.84	0.306416	0.00787962	0	0.00895549	0.0465299	0.8473761	0.000399928
ENSG00000157306	NA	14	24005478	C	A	0.0904573	0.00494238	0.0249313	0.7899998	0.241695	0.0330482	2.60E-13	0.0204488	0.10319	0.8429143	0.102564
ENSG00000157315	TMED6	16	69381431	C	T	0.357853	-0.0216926	0.015019	0.16	-0.405236	0.00889407	0	0.0535308	0.037081	0.1488463	0.9467259
ENSG00000157322	CLEC18A	16	69991475	T	A	0.397614	0.00750283	0.0146011	0.6200004	-0.968364	0.0140562	0	-0.00774794	0.0150785	0.6073642	0.9151852
ENSG00000157326	DHRS4	14	24430641	G	A	0.471173	-0.00848061	0.014184	0.5400003	-0.14865	0.0131279	1.01E-29	0.0570509	0.0955518	0.550462	0.4082013

ENSG00000157335	CLEC18C	16	70214244	T	G	0.311133	0.00583766	0.0152731	0.5400003	-0.556339	0.0207753	5.71E-158	-0.010493	0.0274557	0.7023282	0.3565603
ENSG00000157343	ARMC12	6	35710832	A	G	0.177932	-0.0129235	0.0182405	0.5400003	-0.108748	0.0112882	5.76E-22	0.118839	0.168184	0.4798166	0.5861448
ENSG00000157349	DDX19B	16	70346376	C	T	0.10835	-0.0349567	0.0227452	0.1299999	0.181119	0.018029	9.57E-24	-0.193004	0.127043	0.1287103	0.4326621
ENSG00000157350	ST3GAL2	16	70443239	A	G	0.0815109	0.0174461	0.0286664	0.4799997	0.189369	0.0207564	7.28E-20	0.0921277	0.151715	0.543691	0.8818402
ENSG00000157368	IL34	16	70654191	A	C	0.0805169	0.0167456	0.027601	0.4799997	0.128079	0.0160215	1.30E-15	0.130744	0.216119	0.5452036	0.480027
ENSG00000157379	DHRS1	14	24764421	A	G	0.0626243	-0.0162506	0.029828	0.4299995	-0.801308	0.0147872	0	0.0202801	0.037226	0.585903	0.1764889
ENSG00000157388	CACNA1D	3	53687586	G	A	0.296223	0.0129976	0.0160498	0.56	-0.0850823	0.00873355	2.00E-22	-0.152765	0.189289	0.4196401	0.2459427
ENSG00000157423	HYDIN	16	71052953	G	A	0.0168986	0.0718494	0.0544985	0.16	0.384287	0.0245896	4.69E-55	0.186968	0.142321	0.1889446	0.6990989
ENSG00000157426	AASDH	4	57229061	G	A	0.501988	0.0288129	0.0141873	0.02699977	-0.504948	0.00742355	0	-0.0570612	0.0281091	0.04235764	0.8335423
ENSG00000157429	ZNF19	16	71548722	G	A	0.149105	-0.0312792	0.0209458	0.09299937	0.0842791	0.0118167	9.88E-13	-0.371138	0.253918	0.1438387	0.6220823
ENSG00000157445	CACNA2D3	3	54632579	C	T	0.485089	0.00706966	0.014522	0.84	0.168543	0.00789755	4.71E-101	0.0419457	0.0861843	0.6264727	0.733033
ENSG00000157450	RNF111	15	59273496	G	A	0.0636183	-0.030844	0.0294954	0.29	-0.118395	0.0158054	6.85E-14	0.260517	0.251542	0.3003518	0.7640942
ENSG00000157470	FAM81A	15	59740320	G	A	0.535785	-0.0168987	0.014211	0.2099999	0.128786	0.00796215	7.59E-59	-0.131215	0.110643	0.2356509	0.8902102
ENSG00000157483	MYO1E	15	59546106	A	G	0.539761	-0.0167101	0.014195	0.2200002	0.175985	0.0079591	2.47E-108	-0.0949517	0.0807744	0.2397878	0.9011708
ENSG00000157500	APPL1	3	57284630	A	G	0.388668	0.0156582	0.0145163	0.4	0.0660666	0.00816282	5.79E-16	0.237006	0.221665	0.2849753	0.4416351
ENSG00000157538	VPS26C	21	38617991	C	T	0.350895	0.0177037	0.0152857	0.29	0.183411	0.00838993	6.14E-106	0.0965249	0.0834583	0.2474501	0.5768839
ENSG00000157540	DYRK1A	21	38813458	T	C	0.10338	0.036636	0.0221049	0.04399973	0.133589	0.0126457	4.38E-26	0.274245	0.167494	0.1015591	0.2375056
ENSG00000157551	KCNJ15	21	39601438	T	C	0.507952	-0.0151845	0.0143561	0.14	0.498421	0.00749227	0	-0.0304652	0.0288068	0.2902515	0.09992813
ENSG00000157557	ETS2	21	40187055	T	C	0.365805	-0.000943145	0.0148192	0.99	0.269816	0.00818145	1.62E-238	-0.00349551	0.0549234	0.9492542	0.6588125
ENSG00000157570	TSPAN18	11	44850993	A	G	0.23161	0.0147699	0.0175908	0.3400001	0.0860654	0.00955857	2.18E-19	0.171613	0.205276	0.4031489	0.8199914
ENSG00000157578	LCA5L	21	40797750	T	G	0.308151	0.00839606	0.0151524	0.4100001	0.32655	0.00855557	0	0.0257114	0.0464064	0.5795452	0.7029893
ENSG00000157593	SLC35B2	6	44223562	A	C	0.10338	0.00577949	0.025787	0.7600007	0.266991	0.0134254	5.27E-88	0.0216468	0.0965899	0.8226718	0.8894286
ENSG00000157601	MX1	21	42811686	T	C	0.389662	-0.00803509	0.0144805	0.5500004	-0.467407	0.00770207	0	0.0171908	0.0309818	0.5789855	0.8195482
ENSG00000157617	C2CD2	21	43339610	T	C	0.350895	-0.00477066	0.0149823	0.6499995	0.187049	0.00839845	6.94E-110	-0.0255049	0.0801066	0.7501912	0.6597347
ENSG00000157637	SLC38A10	17	79244073	C	T	0.129225	0.00866352	0.0217985	0.5099998	0.506882	0.0139946	2.96E-287	0.0170918	0.0430077	0.6910632	0.7545728
ENSG00000157653	C9orf43	9	116182149	G	A	0.0954274	0.0114052	0.0242428	0.7499995	-0.18256	0.0145501	4.13E-36	-0.0624738	0.132887	0.6382644	0.3705261
ENSG00000157657	ZNF618	9	116728716	C	T	0.267396	0.0306741	0.0162749	0.09400046	-0.0629986	0.0100294	3.36E-10	-0.486901	0.269716	0.07103776	0.5680592
ENSG00000157693	TMEM268	9	117391094	G	A	0.228628	0.00821283	0.0166749	0.6800001	-0.301297	0.00916818	7.33E-237	-0.0272582	0.0553499	0.6223863	0.3155759
ENSG00000157734	SNX22	15	64446797	T	C	0.0616302	-0.020332	0.0298739	0.4	0.18524	0.0177722	1.95E-25	-0.10976	0.161615	0.4970442	0.1900789
ENSG00000157741	UBN2	7	138954041	C	T	0.203777	0.00983302	0.0175094	0.4899999	0.0772165	0.0116846	3.89E-11	0.127343	0.227574	0.5757739	0.8900885
ENSG00000157764	BRAF	7	140524753	C	T	0.204771	-0.0256422	0.0172671	0.1199999	0.0883164	0.0141878	4.82E-10	-0.290345	0.201001	0.1485998	0.5660734
ENSG00000157778	PSMG3	7	1608803	G	A	0.317097	-0.0252079	0.0149732	0.09200046	-0.198238	0.00985506	5.41E-90	0.12716	0.0757953	0.09341172	0.03470935
ENSG00000157782	CABP1	12	121091741	C	G	0.426441	-0.00123399	0.0143638	0.8499999	0.160322	0.00814544	3.05E-86	-0.00769696	0.0895944	0.9315388	0.7728408
ENSG00000157796	WDR19	4	39235727	C	A	0.423459	-0.0280953	0.0143891	0.0519996	-0.348829	0.00821964	0	0.0805417	0.0412933	0.05111927	0.3369146
ENSG00000157800	SLC37A3	7	140048863	A	G	0.446322	0.02448	0.014331	0.07100027	0.291258	0.00781207	3.08E-304	0.0840492	0.0492554	0.08793456	0.1249568
ENSG00000157823	AP3S2	15	90405702	C	G	0.259443	0.00587089	0.0159851	0.64	0.516207	0.00928468	0	0.0113731	0.0309671	0.7134216	0.6949816
ENSG00000157827	FMNL2	2	153349049	A	G	0.45825	0.00850094	0.0143826	0.7099994	0.124546	0.00865753	6.35E-47	0.0682557	0.115578	0.5548159	0.3680882
ENSG00000157833	GAREM2	2	26404246	T	C	0.0298211	0.0643606	0.0405511	0.1100001	-0.285488	0.0281613	3.76E-24	-0.225441	0.143772	0.1168702	0.2065546
ENSG00000157837	SPPL3	12	121271243	G	C	0.455268	0.0123969	0.0142245	0.3700002	0.205482	0.007888	1.35E-149	0.0603307	0.0692636	0.3837377	0.9889954
ENSG00000157856	DRC1	2	26652181	A	G	0.0715706	-0.00109276	0.0277763	0.8499999	-0.160343	0.0190893	4.48E-17	0.00681515	0.173233	0.9686185	0.4921898
ENSG00000157870	PRXL2B	1	2520419	C	T	0.384692	0.0412728	0.0146859	0.00659994	0.400085	0.00998317	0	0.10316	0.0367971	0.005055369	0.0420713
ENSG00000157873	TNFRSF14	1	2491949	C	T	0.480119	-0.0132399	0.014217	0.4100001	0.369594	0.00847905	0	-0.0358228	0.0384753	0.3518226	0.04591361
ENSG00000157881	PANK4	1	2449005	T	C	0.26839	-0.0334974	0.0162628	0.03899959	0.187066	0.00922005	1.61E-91	-0.179068	0.0873832	0.04044032	0.1513304
ENSG00000157895	C12orf43	12	121447265	C	G	0.39662	-0.00354843	0.0146558	0.6100002	0.334412	0.00874771	0	-0.010611	0.0438265	0.8086927	0.9123376
ENSG00000157911	PEX10	1	2340736	T	C	0.300199	0.0127914	0.0153075	0.5	0.132862	0.00979605	6.65E-42	0.0962759	0.115432	0.4042539	0.6352642
ENSG00000157916	RER1	1	2329228	C	T	0.311133	0.0163705	0.0153202	0.4	-0.0889541	0.00848022	9.64E-26	-0.184033	0.173117	0.287757	0.4268442
ENSG00000157933	SKI	1	2200846	C	T	0.38171	-0.00768112	0.0145846	0.5999997	0.0793676	0.00878932	1.72E-19	-0.096779	0.184072	0.5990507	0.3923001
ENSG00000157954	WIPI2	7	5251638	T	C	0.253479	0.0553748	0.0167752	0.000909997	0.461129	0.00889982	0	0.120085	0.0364523	0.000986625	0.9827507
ENSG00000157978	LDLRAP1	1	25882724	A	G	0.473161	-0.0152404	0.0142364	0.29	0.607863	0.00809112	0	-0.0250721	0.0234228	0.2844326	0.221479
ENSG00000157985	AGAP1	2	236718965	C	G	0.34493	0.0397877	0.0148079	0.002300011	0.260473	0.00828804	8.49E-217	0.152752	0.0570574	0.007424976	0.6564183

ENSG00000157992	KRTCAP3	2	27667290	G	A	0.374751	0.00975769	0.0145177	0.56	0.384532	0.0116548	1.01E-238	0.0253755	0.037762	0.5015937	0.0119698
ENSG00000157999	ANKRD61	7	6073512	A	G	0.473161	0.00392148	0.0142837	0.81	0.185842	0.0199942	1.47E-20	0.0211011	0.0768928	0.7837599	0.3019409
ENSG00000158006	PAFAH2	1	26305453	G	A	0.199801	-0.0468247	0.0174454	0.004600023	-0.323328	0.00989795	4.73E-234	0.144821	0.0541376	0.007471667	0.00193997
ENSG00000158019	BABAM2	2	28337288	G	A	0.270378	0.000787456	0.0161586	0.9400001	-0.0627292	0.00904713	4.10E-12	-0.0125533	0.257599	0.9611331	0.4035695
ENSG00000158023	CFAP251	12	122398800	T	C	0.314115	-0.013308	0.0155751	0.5	0.167378	0.0128149	5.49E-39	-0.0795089	0.0932526	0.393871	0.5405698
ENSG00000158042	MRPL17	11	6703322	T	C	0.167992	-0.0245044	0.0200456	0.2	-0.153093	0.0103068	6.59E-50	0.160063	0.13138	0.2231047	0.8118194
ENSG00000158050	DUSP2	2	96810042	A	G	0.0337972	-0.0946012	0.0382777	0.01199997	-0.307117	0.0312314	8.07E-23	0.30803	0.128512	0.0165343	0.2801648
ENSG00000158062	UBXN11	1	26626336	A	G	0.0139165	-0.0588087	0.0497408	0.28	0.457839	0.0321896	6.58E-46	-0.128449	0.109017	0.2387012	0.3663858
ENSG00000158079	PTPDC1	9	96832607	C	T	0.389662	-0.0084127	0.0148437	0.4799997	0.0569137	0.00820532	4.03E-12	-0.147815	0.26168	0.5721624	0.4633224
ENSG00000158089	GALNT14	2	31255700	G	A	0.374751	-0.022696	0.014693	0.1100001	-0.198839	0.00813302	5.23E-132	0.114142	0.0740412	0.1231691	0.2233276
ENSG00000158092	NCK1	3	136624857	T	G	0.237575	0.00528768	0.0173956	0.91	-0.12486	0.00982449	5.27E-37	-0.042349	0.139361	0.7612196	0.8781515
ENSG00000158104	HPD	12	122289467	C	T	0.372763	0.0273686	0.0148551	0.0519996	0.522403	0.0116979	0	0.0523899	0.0284603	0.06565017	0.9460245
ENSG00000158106	RHPN1	8	144458723	C	T	0.236581	0.0155196	0.0159422	0.4100001	-0.140667	0.00947161	6.81E-50	-0.110329	0.113576	0.3313456	0.899646
ENSG00000158109	TPRG1L	1	3544128	G	A	0.196819	0.00645247	0.0173701	0.5199996	0.142498	0.0107191	2.51E-40	0.0452812	0.121945	0.7103955	0.1572566
ENSG00000158113	LRRC43	12	122670151	C	A	0.449304	0.0215929	0.0142356	0.1100001	0.133856	0.00803169	2.32E-62	0.161314	0.10679	0.1308953	0.5484211
ENSG00000158122	PRXL2C	9	99409722	G	A	0.130219	0.0318765	0.0224021	0.1299999	-0.184444	0.0132301	3.56E-44	-0.172825	0.122088	0.1569013	0.1849486
ENSG00000158156	XKR8	1	28290290	A	G	0.335984	-0.0147765	0.0151201	0.33	-0.167196	0.00875114	2.27E-81	0.0883784	0.0905517	0.3290648	0.2212074
ENSG00000158158	CNNM4	2	97452133	C	T	0.385686	-0.0213982	0.0145324	0.08100093	0.201623	0.00800766	6.84E-140	-0.10613	0.0722002	0.141579	0.5295744
ENSG00000158161	EYA3	1	28356031	G	T	0.0576541	-0.0633044	0.0328642	0.03599979	0.812386	0.0195617	0	-0.077924	0.0404974	0.05433287	0.1829452
ENSG00000158163	DZIP1L	3	137807746	A	G	0.0487078	-0.0740457	0.0421582	0.04099964	-0.162992	0.0205949	2.49E-15	0.45429	0.264945	0.08640764	0.283061
ENSG00000158169	FANCC	9	97970663	C	T	0.0397614	-0.0127258	0.0389918	0.8600001	-0.273841	0.025838	3.03E-26	0.0464715	0.142456	0.7442605	0.8308478
ENSG00000158186	MRAS	3	138095457	C	A	0.0208748	-0.00244813	0.0471748	0.8700001	-0.473856	0.0348383	3.92E-42	0.0051664	0.0995559	0.9586128	0.7241841
ENSG00000158195	WASF2	1	27773699	C	T	0.135189	-0.0101714	0.0251287	0.4400003	0.129835	0.01308	3.20E-23	-0.0783412	0.193705	0.685892	0.7829945
ENSG00000158201	ABHD3	18	19257812	A	G	0.204771	0.0312004	0.0184353	0.16	-0.15812	0.0104526	1.07E-51	-0.197321	0.117318	0.09258172	0.6913327
ENSG00000158234	FAIM	3	138339833	C	A	0.471173	-0.0208648	0.0142591	0.1199999	-0.226213	0.0082977	1.19E-163	0.0922353	0.0631248	0.1439719	0.8928875
ENSG00000158270	COLEC12	18	410041	G	C	0.263419	0.00483396	0.0164323	0.7700005	-0.222745	0.00954374	1.77E-120	-0.0217017	0.0737776	0.7686426	0.433542
ENSG00000158286	RNF207	1	6273447	T	C	0.271372	0.00185669	0.016042	0.8200001	-0.298698	0.00979378	2.71E-204	-0.00621595	0.0537069	0.9078598	0.7744193
ENSG00000158292	GPR153	1	6314220	A	C	0.0487078	-0.00555785	0.0333096	0.91	-0.200761	0.0228137	1.37E-18	0.027684	0.165947	0.8675083	0.4551558
ENSG00000158321	AUTS2	7	69660979	C	T	0.154076	0.0331968	0.0187161	0.07100027	0.391475	0.0104039	60608399999999e-3	0.0847992	0.0478622	0.07643847	0.5729116
ENSG00000158373	H2BC5	6	26164963	C	T	0.33499	-0.00366706	0.0146162	0.8200001	-0.711809	0.00729048	0	0.00515175	0.020534	0.8018997	0.4077922
ENSG00000158406	H4C8	6	26283522	C	T	0.421471	-0.00454073	0.0141849	0.9699999	0.424509	0.00773218	0	-0.0106964	0.0334154	0.748889	0.8803021
ENSG00000158411	MITD1	2	99787705	T	C	0.352883	0.01077	0.0149273	0.5199996	0.256287	0.0121387	6.02E-99	0.0420233	0.0582786	0.4708628	0.9549335
ENSG00000158428	CATIP	2	219227200	C	G	0.106362	-0.0375971	0.0223323	0.1100001	-0.377239	0.0144582	4.54E-150	0.0996639	0.0593225	0.09295021	0.4714167
ENSG00000158435	CNOT11	2	101878021	G	A	0.436382	-0.00665868	0.014462	0.58	-0.14435	0.00886529	1.31E-59	0.0461287	0.100227	0.6453425	0.9198514
ENSG00000158445	KCNB1	20	48042065	T	C	0.267396	-0.00871032	0.0158489	0.4700002	-0.242155	0.0136576	2.44E-70	0.03597	0.0654808	0.582785	0.7448055
ENSG00000158457	TSPAN33	7	128796691	C	T	0.45825	0.0186783	0.0143338	0.1499999	0.323616	0.0082582	0	0.0577175	0.0443171	0.1927883	0.3105057
ENSG00000158467	AHCYL2	7	128967458	G	C	0.305169	0.00917677	0.0156097	0.4899999	0.116475	0.00876954	2.95E-40	0.0787873	0.134149	0.5569929	0.103833
ENSG00000158470	B4GALT5	20	48289948	A	G	0.445328	-0.0211841	0.0143212	0.0990011	0.10756	0.00798195	2.18E-41	-0.196952	0.133946	0.1414588	0.9522157
ENSG00000158473	CD1D	1	158152211	C	T	0.460239	0.00652006	0.0142156	0.5700002	-0.0840186	0.00796717	5.32E-26	-0.0776026	0.169356	0.6467929	0.5327854
ENSG00000158477	CD1A	1	158225993	C	T	0.083499	-0.00694237	0.0240714	0.9199999	-0.680459	0.0132699	0	0.0102025	0.0353758	0.7730384	0.1781509
ENSG00000158480	SPATA2	20	48526004	T	C	0.0447316	-0.05692	0.0326628	0.07299952	-0.151625	0.0202968	8.00E-14	0.3754	0.221202	0.08967936	0.1293285
ENSG00000158481	CD1C	1	158261498	C	T	0.460239	0.00652006	0.0142156	0.5700002	-0.114812	0.00795513	3.24E-47	-0.0567892	0.123879	0.646648	0.2368684
ENSG00000158483	FAM86C1P	11	71505419	G	C	0.431412	0.00178444	0.014427	0.81	0.898268	0.0151363	0	0.00198653	0.0160609	0.9015629	0.3263914
ENSG00000158485	CD1B	1	158299531	C	T	0.460239	0.00652006	0.0142156	0.5700002	-0.192532	0.00790763	6.15E-131	-0.0338649	0.0738482	0.6465402	0.3313492
ENSG00000158488	CD1E	1	158325298	G	A	0.460239	0.00638027	0.0142119	0.5700002	-0.256833	0.00787646	3.19E-233	-0.0248421	0.0553404	0.6535067	0.3287981
ENSG00000158517	NCF1	7	74195984	C	G	0.193837	0.0328821	0.0175549	0.06299992	0.429353	0.0114479	7.64E-308	0.0765853	0.0409379	0.06137706	0.5764076
ENSG00000158525	CPA5	7	129996600	T	G	0.204771	-0.0013593	0.0174397	0.98	0.869877	0.0126315	0	-0.00156263	0.0200485	0.9378735	0.4285408
ENSG00000158528	PPP1R9A	7	94731120	T	C	0.462227	0.0040304	0.0142183	0.8600001	0.242227	0.00860348	2.11E-174	0.016639	0.0587013	0.7768309	0.5618146
ENSG00000158545	ZC3H18	16	88667581	T	G	0.463221	-0.0128743	0.0142947	0.4100001	-0.0861039	0.0086554	2.57E-23	0.149521	0.166696	0.369737	0.930761
ENSG00000158552	ZFAND2B	2	220067166	A	G	0.250497	-0.010525	0.0164963	0.5700002	0.0589593	0.00995766	3.20E-09	-0.178513	0.281411	0.5258526	0.1415548

ENSG00000158555	GDPD5	11	75191316	A	C	0.223658	-0.0200372	0.0180618	0.1299999	0.668107	0.00882594	0	-0.029991	0.0270372	0.2673224	0.4817307
ENSG00000158560	DYNC1I1	7	95570750	A	G	0.254473	0.0213246	0.0155899	0.1100001	-0.0906294	0.00914783	3.87E-23	-0.235295	0.17365	0.1754196	0.5254385
ENSG00000158604	TMED4	7	44619689	G	A	0.4334	-0.00429027	0.0142561	0.6899999	-0.317495	0.00785125	0	0.0135129	0.044903	0.7634645	0.7961176
ENSG00000158615	PPP1R15B	1	204376717	A	G	0.114314	-0.0271558	0.0224815	0.2300001	-0.0828385	0.0126032	4.94E-11	0.327816	0.275934	0.2348246	0.1350534
ENSG00000158623	COPG2	7	130249843	C	T	0.161034	-0.0486398	0.019957	0.008999948	-0.540133	0.0112721	0	0.0900515	0.0369961	0.01492955	0.0291573
ENSG00000158669	GPAT4	8	41458613	G	A	0.465209	-0.0195227	0.0141996	0.2599998	0.148765	0.0117784	1.44E-36	-0.131231	0.0960134	0.1716871	0.1936947
ENSG00000158683	PKD1L1	7	47901169	C	T	0.482107	-0.00841783	0.0142006	0.5300002	0.185822	0.00789119	1.32E-122	-0.0453006	0.0764448	0.5534541	0.9522439
ENSG00000158691	ZSCAN12	6	28357121	A	G	0.0477137	-0.00971192	0.0333309	0.5099998	-0.274351	0.0170263	2.05E-58	0.0353996	0.12151	0.770798	0.06784813
ENSG00000158710	TAGLN2	1	159891709	A	G	0.429423	0.0106896	0.0143672	0.5	-0.248279	0.00800751	4.50E-211	-0.0430548	0.0578838	0.456989	0.5742247
ENSG00000158711	ELK4	1	205589080	C	T	0.324056	0.00950217	0.0148742	0.5300002	0.0891944	0.00817847	1.08E-27	0.106533	0.167048	0.5236413	0.6690119
ENSG00000158714	SLAMF8	1	159801789	A	G	0.0506958	0.00275555	0.0319421	0.91	0.639746	0.0319783	4.92E-89	0.00430726	0.0499298	0.9312548	0.252568
ENSG00000158715	SLC45A3	1	205638283	T	C	0.437376	-0.0155255	0.0142559	0.2599998	-0.115482	0.00802039	5.28E-47	0.13444	0.123799	0.2774985	0.7792421
ENSG00000158716	DUSP23	1	159751527	C	G	0.170974	0.0112447	0.0184358	0.5400003	0.292022	0.0107263	3.31E-163	0.0385064	0.0631475	0.5420041	0.1167625
ENSG00000158717	RNF166	16	88767866	T	G	0.319085	0.00164378	0.0156251	0.9699999	-0.40187	0.00957356	0	-0.00409032	0.0388811	0.9162164	0.4529663
ENSG00000158747	NBL1	1	19975996	T	G	0.426441	-0.014958	0.0143347	0.14	-0.459959	0.00834944	0	0.0325203	0.0311708	0.2968118	0.6149573
ENSG00000158748	HTR6	1	19998917	A	G	0.341948	0.000675955	0.0149228	0.91	-0.285745	0.00847432	3.05E-249	-0.00236559	0.0522243	0.9638708	0.2238955
ENSG00000158764	ITLN2	1	160919702	T	C	0.224652	0.0327206	0.0172121	0.03699985	0.177059	0.00978539	3.54E-73	0.184801	0.0977463	0.05867545	0.3816699
ENSG00000158769	F11R	1	160986892	T	C	0.228628	0.0327601	0.017186	0.03400008	0.180909	0.00956605	9.15E-80	0.181086	0.0954792	0.05788096	0.2761093
ENSG00000158773	USF1	1	161012404	C	T	0.370775	0.036158	0.0145999	0.008600031	-0.397068	0.00781031	0	-0.0910624	0.0368128	0.01337373	0.9886643
ENSG00000158792	SPATA2L	16	89765432	G	C	0.456262	0.027376	0.0144858	0.04399973	-0.325051	0.00798266	0	-0.0842205	0.0446126	0.0590503	0.8559419
ENSG00000158793	NIT1	1	161091555	C	G	0.0735586	-0.00895559	0.026224	0.8700001	-0.157683	0.0147843	1.47E-26	0.0567948	0.166393	0.7328562	0.5809716
ENSG00000158805	ZNF276	16	89797059	T	C	0.388668	-0.0108032	0.0144166	0.6700003	0.239399	0.00797603	6.29E-198	-0.0451264	0.0602388	0.4537819	0.4971977
ENSG00000158806	NPM2	8	21888022	T	G	0.0854871	-0.0547045	0.0276321	0.03799969	-0.359645	0.0240382	1.31E-50	0.152107	0.0775014	0.0496884	0.5652965
ENSG00000158815	FGF17	8	21903114	C	A	0.0854871	-0.0546057	0.0274278	0.03799969	-0.0969246	0.0160355	1.50E-09	0.563383	0.297936	0.05863035	0.3489911
ENSG00000158825	CDA	1	20930421	T	C	0.107356	-0.0416346	0.0213567	0.03500016	1.11551	0.0103896	0	-0.0373232	0.0191483	0.05127569	0.7574275
ENSG00000158828	PINK1	1	20968976	T	C	0.296223	-0.0419634	0.015254	0.004	0.234956	0.00845206	4.50E-170	-0.178601	0.0652398	0.006188846	0.06736569
ENSG00000158850	B4GALT3	1	161144193	A	C	0.239563	-0.0245589	0.0168359	0.1299999	-0.166295	0.0104409	4.10E-57	0.147683	0.101665	0.1463231	0.7660014
ENSG00000158856	DMTN	8	21923272	C	G	0.0497018	0.0170477	0.0330233	0.6899999	0.156107	0.0229731	1.08E-11	0.109205	0.212152	0.6067283	0.256504
ENSG00000158859	ADAMTS4	1	161164192	T	C	0.00596421	-0.214179	0.0855011	0.009099971	-0.877423	0.143085	8.67E-10	0.2441	0.105263	0.020397	0.9291702
ENSG00000158863	FHIP2B	8	21954539	A	G	0.44831	-0.0012223	0.0142681	0.98	0.0796719	0.00916149	3.43E-18	-0.0153417	0.179094	0.9317347	0.2152082
ENSG00000158864	NDUFS2	1	161175539	T	C	0.319085	-0.0312496	0.0152938	0.04499974	-0.225171	0.00863498	6.72E-150	0.138782	0.068129	0.04164588	0.3906686
ENSG00000158865	SLC5A11	16	24890055	T	C	0.16004	-0.0125612	0.017911	0.5300002	0.529272	0.00995427	0	-0.023733	0.0338438	0.4831464	0.3887397
ENSG00000158869	FCER1G	1	161187756	T	G	0.269384	-0.0111854	0.0160018	0.5999997	0.817431	0.0116551	0	-0.0136836	0.0195767	0.4845682	0.03142134
ENSG00000158874	APOA2	1	161192751	C	T	0.378728	-0.0236378	0.014698	0.16	0.14286	0.00859731	5.27E-62	-0.165462	0.103365	0.1094317	0.1199856
ENSG00000158882	TOMM40L	1	161198100	G	A	0.38668	0.032481	0.0146792	0.05600025	0.288814	0.00871427	7.24E-241	0.112464	0.050939	0.02725768	0.07889388
ENSG00000158887	MPZ	1	161277143	A	G	0.0109344	0.0296629	0.045576	0.5	-0.422962	0.0213718	3.59E-87	-0.0701314	0.107813	0.5153738	0.9388678
ENSG00000158941	CCAR2	8	22470586	T	A	0.337972	-0.00589504	0.0150814	0.6100002	-0.318418	0.0101145	1.57E-217	0.0185135	0.0473672	0.6959071	0.2877097
ENSG00000158966	CACHD1	1	65047584	A	G	0.0954274	-0.0107524	0.0224489	0.7099994	-0.219014	0.0126249	2.05E-67	0.0490945	0.102539	0.6320882	0.6732762
ENSG00000158985	CDC42SE2	5	130657663	A	G	0.202783	-0.0233858	0.0182044	0.1800002	-0.478482	0.010414	0	0.048875	0.0380611	0.1990994	0.156187
ENSG00000158987	RAPGEF6	5	130865271	C	T	0.224652	0.00431786	0.0179873	0.9	0.20206	0.0106968	1.38E-79	0.0213692	0.0890268	0.8103061	0.5957322
ENSG00000159023	EPB41	1	29330078	C	T	0.0646123	0.00357466	0.0262379	0.8200001	0.170952	0.0152176	2.78E-29	0.0209104	0.153493	0.8916392	0.07459752
ENSG00000159055	MIS18A	21	33645955	G	C	0.26839	-0.00527929	0.0158946	0.5999997	0.205875	0.0104167	6.08E-87	-0.0256432	0.0772159	0.7398162	0.2931065
ENSG00000159063	ALG8	11	77831344	T	C	0.172962	-0.0165236	0.0193856	0.4199997	0.207046	0.0110774	5.88E-78	-0.0798066	0.0937269	0.3945029	0.6947782
ENSG00000159069	FBXW5	9	139837017	G	C	0.252485	-0.0271743	0.0161597	0.1100001	0.0789258	0.00922633	1.18E-17	-0.344302	0.208664	0.09893673	0.3892889
ENSG00000159079	CFAP298	21	33974782	C	T	0.0397614	-0.0234435	0.032447	0.4299995	-0.19646	0.0200613	1.21E-22	0.119329	0.165607	0.4711813	0.2312577
ENSG00000159082	SYNJ1	21	34050714	T	C	0.0248509	0.0864665	0.0506287	0.1	0.397557	0.0306952	2.30E-38	0.217494	0.128452	0.0904177	0.2583662
ENSG00000159086	PAXBP1	21	34125189	G	A	0.0685885	-0.00175075	0.0281757	0.7300002	0.343942	0.0166046	2.61E-95	-0.00509025	0.0819204	0.9504541	0.1153631
ENSG00000159110	IFNAR2	21	34620087	A	C	0.463221	0.046364	0.0142967	0.001199997	0.348474	0.00817946	0	0.133049	0.0411453	0.001222271	0.9913028
ENSG00000159111	MRPL10	17	45904769	G	A	0.390656	-0.0134634	0.0143831	0.4299995	-0.23186	0.00802866	2.19E-183	0.058067	0.0620662	0.3494969	0.3370894
ENSG00000159128	IFNGR2	21	34813428	T	C	0.141153	0.0238898	0.0205526	0.16	0.644771	0.0107396	0	0.0370516	0.0318818	0.2451722	0.5256929

ENSG00000159131	GART	21	34896017	G	C	0.269384	0.0147245	0.0167407	0.4	-0.135145	0.00936255	3.13E-47	-0.108954	0.124102	0.3799792	0.128838
ENSG00000159140	SON	21	34932368	A	G	0.285288	-0.0169025	0.0154891	0.2099999	-0.0737517	0.00876077	3.82E-17	0.229181	0.211774	0.279165	0.01289934
ENSG00000159164	SV2A	1	149882152	G	C	0.0377734	-0.038577	0.0361398	0.3700002	0.435955	0.0413103	4.91E-26	-0.0884884	0.0833209	0.288227	0.1539855
ENSG00000159166	LAD1	1	201355554	A	G	0.0178926	0.00812897	0.042663	0.7400005	0.282485	0.0374987	4.95E-14	0.0287767	0.151076	0.8489344	NA
ENSG00000159176	CSRP1	1	201465621	C	T	0.307157	-0.0183558	0.0157202	0.2200002	0.173913	0.00847894	1.71E-93	-0.105546	0.0905374	0.2437077	0.8037184
ENSG00000159189	C1QC	1	22972363	G	C	0.303181	-0.0148264	0.0154352	0.1900002	0.16211	0.00851989	1.02E-80	-0.0914591	0.0953358	0.3373895	0.4359629
ENSG00000159199	ATP5MC1	17	46971680	G	A	0.435388	-0.0257342	0.0142376	0.1299999	0.293034	0.00818224	6.83E-281	-0.0878199	0.0486487	0.07104589	0.7959049
ENSG00000159200	RCAN1	21	35936440	T	C	0.496024	-0.00490108	0.0142279	0.64	-0.0653515	0.00796523	2.31E-16	0.0749957	0.217905	0.7307211	0.4814833
ENSG00000159202	UBE2Z	17	46996074	C	A	0.171968	-0.0245179	0.0189195	0.1499999	0.408187	0.00988494	0	-0.0600654	0.0463729	0.1952276	0.6918416
ENSG00000159210	SNF8	17	47014578	T	C	0.237575	-0.0230368	0.0163087	0.29	-0.0936275	0.0107672	3.45E-18	0.246047	0.17647	0.1632359	0.1213401
ENSG00000159212	CLIC6	21	36066106	A	G	0.205765	-0.00493306	0.0167972	0.8200001	0.189887	0.00951404	1.26E-88	-0.0259789	0.0884685	0.7690241	0.594098
ENSG00000159214	CCDC24	1	44459615	T	C	0.26839	0.00855892	0.0168573	0.6499995	0.636597	0.00888508	0	0.0134448	0.026481	0.6116532	0.2775618
ENSG00000159216	RUNX1	21	36758572	C	T	0.167992	0.0227219	0.0189295	0.16	-0.0697708	0.0110747	2.98E-10	-0.325665	0.27619	0.2383457	0.9177823
ENSG00000159228	CBR1	21	37443851	T	A	0.027833	0.0445476	0.0504805	0.4400003	-1.15363	0.0289057	0	-0.0386151	0.0437686	0.3776385	0.3544579
ENSG00000159231	CBR3	21	37513037	C	A	0.429423	0.0226616	0.0143354	0.08100093	-0.61043	0.00726032	0	-0.037124	0.0234883	0.1139845	0.2484998
ENSG00000159239	NA	2	74645011	G	T	0.0168986	0.0176209	0.046404	0.5300002	-0.186147	0.0329453	1.60E-08	-0.094661	0.249849	0.7047819	0.3014354
ENSG00000159247	NA	9	141058193	G	C	0.459245	0.00865154	0.0142791	0.3900004	-0.555804	0.0179541	2.05E-210	-0.0155658	0.0256958	0.544666	0.4132426
ENSG00000159256	MORC3	21	37725466	A	C	0.127237	-0.0100439	0.0214024	0.59	0.554527	0.0114991	0	-0.0181126	0.0385976	0.6388793	0.9476888
ENSG00000159259	CHAF1B	21	37773400	G	A	0.0318091	0.0415182	0.0454466	0.28	0.290129	0.045658	2.09E-10	0.143102	0.158253	0.3658561	0.4804563
ENSG00000159263	SIM2	21	38096825	G	T	0.454274	-0.00258055	0.0143215	0.98	0.0643661	0.00811719	2.20E-15	-0.0400918	0.222558	0.8570422	0.5654089
ENSG00000159267	HLCS	21	38243014	G	T	0.39165	0.000222607	0.014608	0.6899999	0.400685	0.00785854	0	0.000555566	0.0364576	0.9878417	0.4444091
ENSG00000159314	ARHGAP27	17	43491531	T	C	0.299205	0.0100625	0.0161005	0.83	0.482248	0.00841637	0	0.0208658	0.0333883	0.5320082	0.5684454
ENSG00000159322	ADPGK	15	73060948	T	C	0.0427435	-0.0192048	0.0375421	0.4799997	0.728446	0.0215137	2.61E-251	-0.0263641	0.0515431	0.6090043	0.4259628
ENSG00000159339	PADI4	1	17662594	A	C	0.214712	0.0168303	0.0173622	0.5	-0.454613	0.00943874	0	-0.0370212	0.0381989	0.3324613	0.8689172
ENSG00000159346	ADIPOR1	1	202918825	C	T	0.354871	0.00686984	0.0146918	0.6800001	0.135675	0.00822841	4.43E-61	0.0506345	0.10833	0.6402073	0.6901926
ENSG00000159348	CYB5R1	1	202933702	C	T	0.286282	0.01416	0.0154246	0.3599996	0.317608	0.00877769	1.11E-286	0.0445833	0.0485806	0.3587661	0.8519305
ENSG00000159352	PSMD4	1	151233567	T	C	0.229622	0.00467245	0.0169844	0.5199996	-0.197922	0.0101843	3.98E-84	-0.0236075	0.0858222	0.7832587	0.7892388
ENSG00000159363	ATP13A2	1	17325438	G	A	0.452286	-0.00884936	0.014312	0.4199997	-0.162975	0.00796335	4.36E-93	0.054299	0.0878574	0.5365519	0.6606667
ENSG00000159374	M1AP	2	74830237	T	C	0.0168986	0.0808306	0.0472633	0.06100002	-0.246015	0.0336739	2.76E-13	-0.32856	0.197309	0.09587238	0.1578011
ENSG00000159377	PSMB4	1	151373215	G	A	0.16501	-0.0164689	0.0187165	0.16	-0.103322	0.0103635	2.07E-23	0.159393	0.181851	0.3807548	0.3424095
ENSG00000159388	BTG2	1	203276674	T	G	0.416501	0.0131692	0.0142338	0.2	-0.407947	0.00777993	0	-0.0322817	0.0348968	0.3549335	0.235639
ENSG00000159399	HK2	2	75090797	A	G	0.271372	-0.0038273	0.0159031	0.64	0.475493	0.00922459	0	-0.00804913	0.0334459	0.8098178	0.1336208
ENSG00000159403	C1R	12	7216358	A	G	0.243539	0.00156122	0.0161195	0.6800001	0.115138	0.00894391	6.35E-38	0.0135595	0.140005	0.9228454	0.2017585
ENSG00000159423	ALDH4A1	1	19213600	G	A	0.0457256	-0.0788118	0.0372679	0.03400008	-0.356721	0.0235146	5.57E-52	0.220934	0.105484	0.03621688	0.6107433
ENSG00000159433	STARD9	15	42940518	C	T	0.235586	-0.00164379	0.0164182	0.9299999	-0.107639	0.0103479	2.43E-25	0.0152713	0.152537	0.9202527	0.9925299
ENSG00000159445	THEM4	1	151864172	A	G	0.395626	-0.00781005	0.0145103	0.5999997	-0.599229	0.00733179	0	0.0130335	0.0242155	0.5904182	0.9673769
ENSG00000159459	UBR1	15	43316703	C	T	0.27833	-0.00818588	0.0154036	0.5500004	-0.096768	0.00873483	1.60E-28	0.0845929	0.159364	0.5955463	0.788028
ENSG00000159461	AMFR	16	56427407	A	G	0.472167	0.00267714	0.0142592	0.8700001	-0.548017	0.00795708	0	-0.00488514	0.0260197	0.8510745	0.2180559
ENSG00000159479	MED8	1	43852533	G	A	0.371769	0.000699439	0.014552	0.9699999	-0.246002	0.00938352	1.73E-151	-0.00284322	0.0591541	0.9616647	0.6702284
ENSG00000159496	RGL4	22	24035843	A	G	0.167992	0.0198964	0.0183361	0.2700001	0.166605	0.0109058	1.09E-52	0.119423	0.110335	0.2790892	0.8891157
ENSG00000159556	ISL2	15	76631941	C	T	0.408549	0.00863476	0.014503	0.5199996	0.242062	0.00799136	1.53E-201	0.0356717	0.0599259	0.551668	0.6351344
ENSG00000159579	RSPRY1	16	57247218	A	G	0.493042	-0.000223546	0.0142786	0.8	0.147457	0.00792918	3.41E-77	-0.001516	0.0968321	0.9875088	0.05542814
ENSG00000159588	CCDC17	1	46087722	T	C	0.286282	0.0198555	0.015426	0.28	-0.146872	0.00909096	1.03E-58	-0.135189	0.105363	0.1994643	0.5610569
ENSG00000159592	GPBP1L1	1	46123380	A	T	0.0854871	0.00754273	0.0265431	0.6200004	-0.149341	0.0248042	1.74E-09	-0.0505067	0.177933	0.7765229	0.5984083
ENSG00000159593	NAE1	16	66871968	A	T	0.437376	-0.0195896	0.014423	0.06100002	-0.143386	0.00801971	1.71E-71	0.136621	0.100878	0.1756352	0.03684541
ENSG00000159596	TMEM69	1	46156500	G	A	0.242545	-0.0135779	0.0166362	0.4100001	-0.0979075	0.0108574	1.92E-19	0.138681	0.170612	0.4163078	0.2747442
ENSG00000159618	ADGRG5	16	57600963	G	A	0.286282	0.0174948	0.0154621	0.4299995	-0.208972	0.0084305	1.22E-135	-0.0837184	0.0740683	0.2583555	0.6999325
ENSG00000159625	DRC7	16	57747211	T	C	0.23161	0.0272378	0.0174861	0.1900002	0.213749	0.0102381	8.52E-97	0.127429	0.0820341	0.1203358	0.8417999
ENSG00000159640	ACE	17	61576813	A	G	0.366799	0.0033665	0.0147591	0.91	0.0750549	0.00887908	2.84E-17	0.0448538	0.196716	0.8196357	0.7365826
ENSG00000159648	SPMIP8	16	58016179	A	C	0.142147	-0.00964876	0.0191002	0.8800001	-0.212944	0.0111223	1.05E-81	0.0453113	0.0897272	0.6135661	0.3316733

ENSG00000159658	EFCAB14	1	47154595	C	T	0.23161	0.00808558	0.0166274	0.7199992	-0.23949	0.0100717	5.59E-125	-0.0337617	0.0694429	0.6268404	0.8735763
ENSG00000159674	SPON2	4	1181735	A	G	0.131213	0.000278954	0.0218939	0.9299999	0.24586	0.0121264	2.15E-91	0.0011346	0.0890502	0.9898343	0.4834528
ENSG00000159685	CHCHD6	3	126551156	T	G	0.145129	0.0315766	0.0199282	0.06699926	-0.089969	0.011193	9.13E-16	-0.350972	0.225763	0.1200412	0.02001327
ENSG00000159692	CTBP1	4	1224488	G	C	0.44831	-0.0100465	0.0144444	0.4299995	0.396056	0.00919291	0	-0.0253664	0.0364754	0.4867806	0.05446349
ENSG00000159713	TPPP3	16	67425575	A	G	0.0357853	0.0277434	0.0353697	0.4299995	0.358393	0.022583	1.02E-56	0.0774107	0.0988103	0.4333765	0.8599849
ENSG00000159714	ZDHHC1	16	67439529	C	G	0.0357853	0.0300694	0.0353037	0.3900004	0.546295	0.0230673	5.45E-124	0.0550424	0.0646657	0.3946667	0.8965199
ENSG00000159720	ATP6V0D1	16	67493528	G	A	0.0387674	0.0257757	0.0344042	0.5	-1.09702	0.0197316	0	-0.023496	0.0313643	0.4537764	0.8881934
ENSG00000159723	AGRP	16	67517095	A	G	0.0367793	0.0375567	0.0348301	0.2999998	0.323379	0.0215012	4.02E-51	0.116138	0.107983	0.282141	0.9823337
ENSG00000159733	ZFYVE28	4	2345849	A	G	0.300199	0.00775677	0.0152519	0.81	0.195653	0.00824823	2.21E-124	0.0396456	0.0779718	0.6111305	0.8175266
ENSG00000159753	CARMIL2	16	67685147	G	A	0.164016	-0.0263246	0.020002	0.16	-0.203677	0.0262328	8.22E-15	0.129247	0.0996054	0.1944288	0.9386508
ENSG00000159761	C16orf86	16	67701690	T	G	0.0347913	0.0124418	0.0352305	0.7400005	-0.645523	0.0249617	1.85E-147	-0.019274	0.0545817	0.7239975	0.5664086
ENSG00000159784	FAM131B	7	143055178	T	C	0.182903	-0.0125174	0.0191151	0.2999998	0.180507	0.0102666	3.38E-69	-0.069346	0.10597	0.5128607	0.3237657
ENSG00000159788	RGS12	4	3368197	T	G	0.0506958	0.0145587	0.0314181	0.7099994	-0.525042	0.0174304	2.48E-199	-0.0277286	0.0598463	0.6431272	0.09826929
ENSG00000159792	PSKH1	16	67945378	C	T	0.00497018	-0.0942059	0.142583	0.5099998	0.531048	0.093732	1.47E-08	-0.177396	0.270313	0.5116557	0.7934388
ENSG00000159840	ZYX	7	143083188	C	G	0.303181	0.0138643	0.0158902	0.5500004	-0.608155	0.00818008	0	-0.0227973	0.0261303	0.382965	0.7756291
ENSG00000159842	ABR	17	1019536	G	A	0.497018	0.0259786	0.0142154	0.06100002	0.254776	0.0101206	7.72E-140	0.101967	0.0559426	0.06834877	0.8180513
ENSG00000159860	NA	7	143424571	C	A	0.345924	-0.00602686	0.0148333	0.7499995	-0.439467	0.0204401	1.55E-102	0.013714	0.0337589	0.684571	0.09338512
ENSG00000159871	LYPD5	19	44315719	A	G	0.467197	-0.00444362	0.0142143	0.7800007	-0.120232	0.00792628	5.69E-52	0.0369587	0.118249	0.7546227	0.670402
ENSG00000159873	CCDC117	22	29176972	A	G	0.294235	0.0104861	0.0155045	0.7499995	0.155933	0.00840217	6.95E-77	0.0672477	0.0994968	0.4991184	0.1807994
ENSG00000159882	ZNF230	19	44512589	T	C	0.116302	0.0134271	0.0209391	0.4299995	0.112236	0.0114033	7.39E-23	0.119633	0.186958	0.522245	0.4094977
ENSG00000159884	CCDC107	9	35659904	G	A	0.0328032	0.022093	0.0449283	0.6700003	-0.358398	0.0377523	2.24E-21	-0.0616438	0.125527	0.6233694	0.4477289
ENSG00000159904	NA	7	5172523	C	T	0.352883	0.0173822	0.0154064	0.4	0.538127	0.013396	0	0.0323013	0.028641	0.2594041	0.2346411
ENSG00000159917	ZNF235	19	44771040	A	G	0.455268	-0.00958313	0.0142323	0.58	0.129142	0.00871447	1.10E-49	-0.0742064	0.110321	0.5011746	0.5671491
ENSG00000159921	GNE	9	36245745	C	T	0.515905	0.0139643	0.0142032	0.33	0.160961	0.00792327	9.47E-92	0.0867557	0.0883431	0.3260849	0.1489854
ENSG00000159958	TNFRSF13C	22	42321933	C	T	0.0218688	0.0858596	0.042386	0.064	-0.423797	0.0236592	9.40E-72	-0.202596	0.100652	0.04413253	0.1320942
ENSG00000160007	ARHGAP35	19	47465133	T	C	0.354871	-0.0116535	0.0149256	0.2999998	0.0714169	0.00930489	1.65E-14	-0.163176	0.210071	0.4372981	0.0700919
ENSG00000160013	PTGIR	19	47126050	C	T	0.202783	-0.0143071	0.0164588	0.4299995	-0.34882	0.00974694	1.72E-280	0.0410158	0.0471982	0.384841	0.6876023
ENSG00000160014	CALM3	19	47109190	C	T	0.474155	0.0230438	0.0142954	0.09699961	0.161816	0.00796639	9.99E-92	0.142407	0.088621	0.1080717	0.4075179
ENSG00000160049	DFFA	1	10524581	A	G	0.506958	-0.0201903	0.0142026	0.2	-0.205329	0.00790492	9.54E-149	0.0983316	0.0692736	0.1557628	0.2934539
ENSG00000160072	ATAD3B	1	1420185	A	T	0.0119284	0.0308965	0.0583887	0.5	-1.03094	0.0787241	3.49E-39	-0.0299693	0.0566826	0.5969993	0.8732366
ENSG00000160075	SSU72	1	1493651	C	T	0.324056	0.0231585	0.0154358	0.1100001	0.544062	0.00844429	0	0.0425659	0.0283791	0.1336392	0.03868158
ENSG00000160087	UBE2J2	1	1199277	C	T	0.0347913	-0.0557085	0.043025	0.2200002	0.167563	0.0189029	7.69E-19	-0.332464	0.259494	0.200124	0.4035645
ENSG00000160094	ZNF362	1	33744233	G	A	0.290258	-0.0159873	0.0155946	0.25	-0.345952	0.0085075	0	0.0462125	0.0450917	0.3054311	0.6055214
ENSG00000160097	FNDC5	1	33332976	A	G	0.40159	0.0275713	0.0145504	0.06199976	-0.0640476	0.00821144	6.20E-15	-0.430481	0.233789	0.06557405	0.6957896
ENSG00000160111	CPAMD8	19	17070691	T	A	0.202783	0.0126494	0.0177509	0.4400003	0.312689	0.00975829	2.71E-225	0.0404536	0.0567826	0.4761986	0.3079041
ENSG00000160113	NR2F6	19	17349720	G	A	0.346918	-0.00690834	0.0149548	0.58	0.453796	0.0090375	0	-0.0152234	0.0329563	0.6441326	0.7878241
ENSG00000160117	ANKLE1	19	17395454	T	C	0.109344	-0.0251396	0.024336	0.32	-0.324509	0.014952	1.91E-104	0.0774696	0.0750781	0.302141	0.9138172
ENSG00000160145	KALRN	3	124119453	G	A	0.209742	-0.0118338	0.0176109	0.2999998	-0.19195	0.0108608	6.70E-70	0.0616505	0.0918137	0.5019188	0.9489033
ENSG00000160172	NA	11	67565963	C	T	0.399602	0.00197962	0.0146446	0.9	0.125714	0.0103844	9.82E-34	0.0157471	0.116499	0.8924782	0.7821681
ENSG00000160179	ABCG1	21	43668576	G	T	0.144135	0.0100192	0.0211911	0.6600001	0.121472	0.0113642	1.15E-26	0.0824813	0.174623	0.6366838	0.7141152
ENSG00000160180	TFF3	21	43733769	A	G	0.0864811	-0.0318814	0.0250748	0.16	0.335405	0.0133959	2.36E-138	-0.0950533	0.074856	0.2041503	0.9902797
ENSG00000160183	TMPRSS3	21	43804477	T	C	0.528827	0.00270672	0.0142104	0.89	-0.187266	0.00790252	3.87E-124	-0.0144539	0.075886	0.8489421	0.8497595
ENSG00000160185	UBASH3A	21	43845899	C	A	0.276342	0.0132104	0.0155704	0.2099999	0.537456	0.00836013	0	0.0245795	0.0289731	0.3962395	0.6808429
ENSG00000160190	SLC37A1	21	43958839	G	C	0.198807	-0.0472344	0.0170215	0.004200007	-0.179318	0.00983859	3.21E-74	0.263411	0.0960174	0.006081226	0.4673298
ENSG00000160191	PDE9A	21	44134682	C	A	0.306163	0.0270672	0.0151192	0.0179999	0.653733	0.00861202	0	0.0414041	0.0231339	0.07349318	0.2875807
ENSG00000160193	WDR4	21	44281441	C	A	0.0596421	0.0183888	0.025642	0.59	-0.47786	0.0135418	9.07E-273	-0.0384815	0.0536711	0.4733816	0.382817
ENSG00000160194	NDUFV3	21	44314768	T	C	0.0755467	0.0974567	0.0299791	0.000929994	-0.854349	0.0165453	0	-0.114071	0.0351595	0.00117699	0.412493
ENSG00000160199	PKNOX1	21	44424396	A	G	0.454274	-0.00117137	0.0142515	0.91	0.240805	0.00785136	1.41E-206	-0.00486439	0.0591829	0.9344937	0.878398
ENSG00000160200	CBS	21	44485177	T	C	0.454274	-0.0021535	0.0142542	0.9599999	0.745282	0.00678394	0	-0.00288951	0.0191259	0.8799141	0.4725949
ENSG00000160201	U2AF1	21	44520381	G	A	0.26839	-0.00347996	0.0156109	0.7400005	-0.414487	0.0126682	8.49E-235	0.00839583	0.0376641	0.8236029	0.4907471

ENSG00000160207	HSF2BP	21	45014223	A	G	0.430417	0.0116253	0.0142327	0.4100001	0.0714741	0.00830708	7.70E-18	0.16265	0.200026	0.4161342	0.2796942
ENSG00000160208	RRP1B	21	45097693	C	T	0.38171	0.0147361	0.014364	0.2599998	0.173753	0.00810587	6.25E-102	0.0848109	0.0827639	0.3054896	0.2156971
ENSG00000160209	PDXK	21	45160581	A	G	0.151093	0.0143046	0.0201832	0.4100001	0.73866	0.0101268	0	0.0193656	0.0273254	0.4785079	0.4195302
ENSG00000160213	CSTB	21	45194359	A	G	0.314115	-0.00481475	0.0153712	0.5	-0.769836	0.00767757	0	0.00625426	0.019967	0.7541057	0.1412792
ENSG00000160214	RRP1	21	45217284	A	G	0.360835	0.00755853	0.0149088	0.7800007	-0.129604	0.0097745	3.98E-40	-0.0583202	0.115118	0.6124255	0.4845961
ENSG00000160216	AGPAT3	21	45345742	A	G	0.105368	-0.00896552	0.0230161	0.7099994	0.175376	0.0134192	4.95E-39	-0.0511217	0.131297	0.6970101	0.004367347
ENSG00000160218	TRAPPC10	21	45479316	T	G	0.187873	0.0305338	0.0176502	0.08300036	-0.138065	0.00986193	1.56E-44	-0.221155	0.128812	0.08600062	0.5396727
ENSG00000160221	GATD3	21	45559546	T	C	0.11332	0.031759	0.0242257	0.2099999	-0.179161	0.0137105	5.06E-39	-0.177265	0.135897	0.192092	0.2122874
ENSG00000160223	ICOSLG	21	45651861	A	C	0.437376	0.010611	0.0144753	0.4700002	-0.233747	0.00906135	9.83E-147	-0.0453953	0.0619523	0.4637134	0.1187042
ENSG00000160224	AIRE	21	45712126	G	A	0.214712	-0.0125789	0.0174747	0.56	-0.0677214	0.011648	6.10E-09	0.185745	0.260008	0.474992	0.7663968
ENSG00000160226	CFAP410	21	45754056	A	G	0.238569	-0.0382131	0.0173302	0.01899984	0.672392	0.0101604	0	-0.0568315	0.0257882	0.02753999	0.2441784
ENSG00000160229	ZNF66	19	20975516	G	A	0.242545	0.0176616	0.0171349	0.32	-0.401796	0.0180216	4.11E-110	-0.0439567	0.0426914	0.3031797	0.2184482
ENSG00000160233	LRRC3	21	45877054	G	A	0.0447316	-0.0204255	0.0369563	0.59	0.538092	0.027314	2.15E-86	-0.0379591	0.0687073	0.5806223	0.52583
ENSG00000160255	ITGB2	21	46328886	T	C	0.177932	-0.0208611	0.0184067	0.3700002	-0.74679	0.0110222	0	0.0279344	0.0246512	0.2571369	0.4240863
ENSG00000160256	SLX9	21	46378414	A	G	0.161034	0.00881944	0.0195446	0.5099998	-0.261273	0.0147502	3.32E-70	-0.0337556	0.0748295	0.6519171	0.4619862
ENSG00000160271	RALGDS	9	136006204	T	C	0.403579	-0.0199149	0.0143358	0.1800002	0.342613	0.0090173	0	-0.0581265	0.0418705	0.1650625	0.314197
ENSG00000160282	FTCD	21	47565828	G	A	0.243539	-0.005555	0.0165771	0.7400005	-0.189325	0.00938398	1.61E-90	0.029341	0.0875708	0.7375839	0.0185809
ENSG00000160284	SPATC1L	21	47592726	G	A	0.243539	-0.005555	0.0165771	0.7400005	-0.645035	0.00968543	0	0.00861193	0.0256999	0.7375522	0.2210946
ENSG00000160285	LSS	21	47628549	C	A	0.146123	0.0171078	0.021167	0.4700002	-0.705079	0.0103011	0	-0.0242637	0.0300229	0.4189907	0.01487312
ENSG00000160293	VAV2	9	136742371	G	A	0.425447	-0.00905208	0.0142718	0.4500005	0.277747	0.00961416	1.62E-183	-0.0325911	0.0513965	0.5260087	0.02548278
ENSG00000160294	MCM3AP	21	47680629	A	G	0.361829	0.0321116	0.0145254	0.02	0.412442	0.00784391	0	0.0778572	0.0352491	0.0271907	0.03380848
ENSG00000160298	C21orf58	21	47731942	T	C	0.429423	-0.0261708	0.0143328	0.05	-0.156929	0.00808131	5.35E-84	0.166768	0.0917357	0.06907654	0.001349255
ENSG00000160299	PCNT	21	47804859	C	T	0.345924	-0.0213549	0.0151267	0.16	0.421991	0.00821718	0	-0.0506051	0.0358596	0.1581849	0.01396
ENSG00000160305	DIP2A	21	47934369	C	T	0.345924	-0.0213549	0.0151267	0.16	0.93902	0.00674832	0	-0.0227417	0.0161099	0.1580492	0.002211552
ENSG00000160307	S100B	21	48021998	C	T	0.345924	-0.0213549	0.0151267	0.16	0.887827	0.0103283	0	-0.024053	0.0170402	0.1580839	0.01009677
ENSG00000160310	PRMT2	21	48070057	C	T	0.345924	-0.0213549	0.0151267	0.16	0.670176	0.00767695	0	-0.0318646	0.0225742	0.1580822	0.01485597
ENSG00000160318	CLDND2	19	51871304	T	G	0.289264	-0.0237675	0.0160443	0.17	0.172623	0.00959646	2.41E-72	-0.137684	0.0932588	0.1398448	0.3540663
ENSG00000160321	ZNF208	19	22154755	G	C	0.335984	0.00552256	0.0152276	0.8800001	-0.264651	0.0124894	1.18E-99	-0.0208673	0.0575469	0.7168927	0.1603233
ENSG00000160323	ADAMTS13	9	136301993	T	C	0.126243	-0.00953726	0.0213809	0.8200001	-0.270898	0.0158578	1.99E-65	0.035206	0.0789528	0.6556605	0.3195842
ENSG00000160325	CACFD1	9	136330529	A	C	0.333996	0.0156996	0.014793	0.16	0.181396	0.00983228	5.31E-76	0.0865489	0.0816858	0.2893566	0.6560256
ENSG00000160326	SLC2A6	9	136340238	C	T	0.100398	0.00688634	0.0237237	0.5999997	0.189328	0.0262475	5.47E-13	0.0363725	0.125406	0.7717873	0.1418566
ENSG00000160336	ZNF761	19	53948371	C	T	0.310139	0.0110876	0.0158286	0.5300002	-0.214869	0.00873771	1.58E-133	-0.0516017	0.0736962	0.4838056	0.2693891
ENSG00000160345	PIERCE1	9	138390303	G	A	0.510934	0.00798107	0.0142148	0.6200004	-0.054836	0.009688	1.51E-08	-0.145544	0.260496	0.5763529	0.3855285
ENSG00000160352	ZNF714	19	21286519	A	G	0.17992	0.0409822	0.0189364	0.01700004	0.154896	0.0121505	3.19E-37	0.264578	0.124001	0.03286946	0.5101572
ENSG00000160360	GPSM1	9	139237994	C	T	0.455268	-0.0246318	0.014249	0.07499978	0.345262	0.0116534	6.59E-193	-0.0713423	0.0413403	0.08439533	0.3384965
ENSG00000160392	C19orf47	19	40839938	A	G	0.0854871	0.0119954	0.0280606	0.7800007	-0.192321	0.0148033	1.36E-38	-0.0623717	0.145984	0.6691967	0.3399439
ENSG00000160404	TOR2A	9	130495703	G	A	0.141153	-0.00249696	0.0190293	0.9	-0.0963704	0.0112611	1.15E-17	0.02591	0.197483	0.8956162	0.8366132
ENSG00000160408	ST6GALNAC6	9	130657643	G	A	0.172962	-0.00291185	0.0190014	0.8800001	-0.744637	0.0123218	0	0.00391043	0.0255178	0.8782063	0.01076942
ENSG00000160410	SHKBP1	19	41090031	G	C	0.486083	0.0189366	0.014241	0.1499999	0.516119	0.00745529	0	0.0366904	0.0275976	0.1836901	0.6584964
ENSG00000160439	RDH13	19	55566567	A	G	0.499006	-0.0134775	0.0142448	0.5500004	0.660492	0.0104924	0	-0.0204052	0.0215694	0.3441344	0.5641655
ENSG00000160445	ZER1	9	131513379	G	A	0.0417495	-0.0206471	0.037018	0.64	-0.220279	0.0208345	3.98E-26	0.0937317	0.168284	0.5775384	0.5312134
ENSG00000160446	ZDHHC12	9	131484777	G	A	0.420477	0.00995169	0.0143544	0.5999997	0.0510425	0.00805569	2.36E-10	0.194969	0.282903	0.4907151	0.9375723
ENSG00000160447	PKN3	9	131473999	A	G	0.159046	-0.0215515	0.0201405	0.3400001	0.52998	0.0125014	0	-0.0406647	0.0380145	0.2847467	0.6210436
ENSG00000160469	BRSK1	19	55808670	G	A	0.459245	0.0114239	0.0142512	0.4400003	-0.142889	0.00867252	5.46E-61	-0.0799498	0.0998545	0.423327	0.5651088
ENSG00000160471	COX6B2	19	55863428	G	A	0.463221	0.00226251	0.0145197	0.8700001	-0.0946239	0.00966319	1.22E-22	-0.0239106	0.153466	0.8761877	0.3576555
ENSG00000160505	NLRP4	19	56370582	T	C	0.39662	0.013401	0.0145876	0.32	0.0928034	0.00833768	8.90E-29	0.144402	0.157723	0.3599062	0.3885309
ENSG00000160539	PLPP7	9	134174865	A	G	0.389662	0.00389808	0.0145417	0.8700001	-0.127355	0.00980456	1.41E-38	-0.0306079	0.114206	0.7886954	0.153058
ENSG00000160563	MED27	9	134845394	G	A	0.318091	0.0266841	0.0152143	0.1	0.112716	0.00931721	1.09E-33	0.236738	0.13639	0.08261045	0.3597741
ENSG00000160570	DEDD2	19	42713521	C	T	0.0924453	0.0207889	0.0235901	0.3599996	0.0930136	0.0143182	8.24E-11	0.223504	0.255943	0.3825232	0.5697587
ENSG00000160584	SIK3	11	116841635	C	A	0.0636183	0.0214331	0.0308641	0.4799997	-0.240096	0.0168735	6.04E-46	-0.0892689	0.128702	0.4879274	0.3367186

ENSG00000160588	MPZL3	11	118110237	G	A	0.50497	0.0131416	0.0142138	0.2099999	0.257928	0.00782819	4.43E-238	0.0509507	0.0551293	0.3553809	0.9356693
ENSG00000160593	JAML	11	118080132	A	G	0.200795	-0.0160886	0.0180547	0.2300001	0.44193	0.00984455	0	-0.0364053	0.0408623	0.372968	0.5164255
ENSG00000160602	NEK8	17	27061694	C	A	0.0725646	0.00136988	0.0251328	0.7800007	0.0784567	0.0136656	9.40E-09	0.0174603	0.320354	0.9565343	0.3165257
ENSG00000160606	TLCD1	17	27053159	G	A	0.0725646	0.000303915	0.0251342	0.7499995	-0.131318	0.0132953	5.24E-23	-0.00231435	0.1914	0.9903525	0.762232
ENSG00000160613	PCSK7	11	117089147	G	A	0.389662	0.0155148	0.0146788	0.4600002	0.317899	0.00790311	0	0.0488042	0.0461904	0.2906996	0.1759102
ENSG00000160633	SAFB	19	5645767	T	C	0.346918	0.0162297	0.0148819	0.2200002	0.0567636	0.0083811	1.26E-11	0.285918	0.265551	0.2816154	0.5687674
ENSG00000160654	CD3G	11	118220467	A	T	0.300199	0.0104062	0.0159913	0.6600001	-0.116397	0.0131919	1.11E-18	-0.0894023	0.137758	0.5163521	0.8092214
ENSG00000160678	S100A1	1	153602457	C	G	0.475149	0.00431452	0.0142626	0.9	0.16317	0.00875037	1.33E-77	0.0264419	0.087421	0.7622968	0.04849093
ENSG00000160679	CHTOP	1	153612653	T	C	0.0258449	-0.157618	0.0485596	0.000389996	0.619792	0.0278261	6.63E-110	-0.254308	0.0791757	0.001318411	0.09105057
ENSG00000160683	CXCR5	11	118761491	T	C	0.223658	0.0162276	0.0170538	0.2	0.0657083	0.0114855	1.06E-08	0.246964	0.263103	0.3479063	0.1832465
ENSG00000160685	ZBTB7B	1	154983062	C	T	0.246521	0.0186142	0.0160791	0.1499999	0.110362	0.00950839	3.81E-31	0.168665	0.146417	0.2493422	0.03388729
ENSG00000160691	SHC1	1	154940822	C	T	0.027833	0.0412366	0.0409297	0.4799997	0.802952	0.0213381	6.975057e-310	0.0513562	0.0509923	0.3138688	0.6058688
ENSG00000160695	VPS11	11	118945545	T	C	0.154076	0.0325624	0.0198179	0.07299952	-0.381325	0.0109861	5.62E-264	-0.0853927	0.0520293	0.1007471	0.07128166
ENSG00000160703	NLRX1	11	119046001	A	G	0.084493	-0.0438511	0.0269668	0.0990011	0.162339	0.0149503	1.82E-27	-0.27012	0.167966	0.1077954	0.2689429
ENSG00000160710	ADAR	1	154577506	C	A	0.471173	-0.0374695	0.0142001	0.005099998	-0.17313	0.00791117	3.67E-106	0.216425	0.0826142	0.008800602	0.5445066
ENSG00000160712	IL6R	1	154409797	C	G	0.266402	0.0304977	0.0172176	0.1	0.111805	0.0091812	4.09E-34	0.272776	0.155617	0.07962544	0.715779
ENSG00000160714	UBE2Q1	1	154526278	A	C	0.0367793	0.118106	0.0436903	0.002800013	0.50892	0.0289107	2.33E-69	0.232072	0.0868554	0.007541579	0.0901959
ENSG00000160716	CHRNB2	1	154546379	T	C	0.327038	0.0382718	0.0154937	0.008099906	0.156969	0.00896046	1.04E-68	0.243817	0.0996816	0.01444687	0.4841908
ENSG00000160741	CRTC2	1	153925623	A	G	0.28827	-0.0302389	0.0154954	0.02999991	-0.0565771	0.00859312	4.58E-11	0.534473	0.285658	0.06134228	0.1127848
ENSG00000160746	ANO10	3	43564718	G	A	0.456262	0.0137026	0.0143964	0.3700002	-0.166403	0.0088101	1.44E-79	-0.0823457	0.0866248	0.3418069	0.7931143
ENSG00000160752	FDPS	1	155284498	A	C	0.271372	-0.00266653	0.0161934	0.98	-0.0930443	0.0096413	4.89E-22	0.0286587	0.174065	0.8692244	0.5986354
ENSG00000160766	NA	1	155190415	A	G	0.477137	0.00432001	0.0141729	0.6600001	0.645464	0.0105382	0	0.00669288	0.021958	0.7605154	0.02238014
ENSG00000160767	ENTREP3	1	155221135	C	A	0.0168986	0.126364	0.0441137	0.002399993	-0.243147	0.029804	3.40E-16	-0.519702	0.192287	0.006876975	0.4733398
ENSG00000160781	PAQR6	1	156215543	C	T	0.536779	-0.00424359	0.0141903	0.7499995	0.797391	0.00656166	0	-0.00532184	0.017796	0.7649036	0.2425939
ENSG00000160783	PMF1	1	156197829	T	C	0.343936	0.0138583	0.0150449	0.33	-0.119641	0.00920726	1.32E-38	-0.115832	0.126066	0.3581878	0.1239229
ENSG00000160785	SLC25A44	1	156173233	C	G	0.0168986	0.027146	0.0468191	0.4799997	-0.29257	0.0351587	8.69E-17	-0.0927847	0.160415	0.5629915	0.7230103
ENSG00000160789	LMNA	1	156081122	G	C	0.0656064	-0.00772564	0.034675	0.7499995	0.67149	0.0187115	4.98E-282	-0.0115052	0.0516399	0.8236934	0.7077385
ENSG00000160791	CCR5	3	46414665	G	A	0.463221	0.00395393	0.0142767	0.9599999	-0.221061	0.00869429	1.30E-142	-0.0178861	0.0645864	0.7818312	0.5734524
ENSG00000160796	NBEAL2	3	47036183	T	C	0.327038	-0.00245809	0.0148728	0.95	0.147254	0.00834724	1.19E-69	-0.0166929	0.101006	0.8687339	0.1254673
ENSG00000160799	CCDC12	3	46993358	G	A	0.414513	0.023923	0.0143348	0.14	-0.1147	0.00809026	1.26E-45	-0.208569	0.125839	0.09743208	0.922497
ENSG00000160803	UBQLN4	1	156014338	T	C	0.397614	7.58E-05	0.0146881	0.9599999	0.401451	0.0172237	3.68E-120	0.00018876	0.0365876	0.9958836	0.6341137
ENSG00000160813	PPP1R35	7	100033546	G	A	0.195825	0.0372948	0.0178804	0.04099964	-0.0933557	0.0112298	9.32E-17	-0.399492	0.197466	0.04306428	0.7397603
ENSG00000160818	GPATCH4	1	156567783	G	A	0.245527	0.0281144	0.0168664	0.14	0.108452	0.00910122	9.75E-33	0.259234	0.157034	0.09877635	0.4664241
ENSG00000160828	NA	7	74209518	G	C	0.2833	0.0135488	0.0158183	0.5199996	-0.856682	0.0243952	3.74E-270	-0.0158154	0.0184701	0.3918472	0.2034652
ENSG00000160856	FCRL3	1	157658459	G	A	0.475149	0.00396561	0.0142538	0.6499995	0.725004	0.00703571	0	0.00546978	0.0196604	0.7808487	0.08172613
ENSG00000160867	FGFR4	5	176519516	C	T	0.260437	-0.00697728	0.0165069	0.56	0.138694	0.00931606	3.97E-50	-0.0503071	0.119065	0.6726467	0.7849335
ENSG00000160877	NACC1	19	13240436	C	T	0.245527	-0.0023732	0.0162583	0.9199999	-0.129	0.0139393	2.15E-20	0.018397	0.126049	0.8839606	0.3600777
ENSG00000160883	HK3	5	176317101	C	A	0.280318	-0.00795199	0.0161244	0.3800004	0.0879188	0.008856	3.16E-23	-0.0904469	0.183627	0.6223252	0.8777988
ENSG00000160908	ZNF394	7	99091044	T	C	0.00795229	-0.0306489	0.0583472	0.6300007	-0.348597	0.0357962	2.07E-22	0.0879206	0.16762	0.5999153	0.7966852
ENSG00000160917	CPSF4	7	99045769	G	A	0.0308151	0.00406236	0.0363936	0.99	-0.308671	0.022807	9.85E-42	-0.0131608	0.117908	0.9111254	0.6508729
ENSG00000160932	LY6E	8	144102324	C	G	0.0775348	-0.030712	0.0293349	0.29	0.380942	0.0153753	1.62E-135	-0.0806212	0.0770749	0.2955561	0.8870069
ENSG00000160948	VPS28	8	145651465	G	A	0.474155	0.00923461	0.0141893	0.6899999	-0.539007	0.00858908	0	-0.0171326	0.0263263	0.5151885	0.3495898
ENSG00000160949	TONSL	8	145661996	T	C	0.471173	0.00947909	0.0141959	0.6700003	-0.42648	0.0140744	1.08E-201	-0.0222264	0.0332943	0.5044067	0.5696127
ENSG00000160953	PWWP3A	19	1332160	A	G	0.291252	-0.00900467	0.0163111	0.4299995	0.217208	0.010052	1.50E-103	-0.0414564	0.0751187	0.5810321	0.72038
ENSG00000160959	LRRC14	8	145746966	C	T	0.489066	-0.00681866	0.0141809	0.4799997	-0.518822	0.00792932	0	0.0131426	0.0273336	0.6306437	0.1336953
ENSG00000160961	ZNF333	19	14822585	A	G	0.302187	0.0096813	0.0156305	0.3599996	-0.545574	0.0087298	0	-0.0177452	0.028651	0.5356827	0.2565629
ENSG00000160963	COL26A1	7	101104202	T	C	0.534791	0.0199163	0.0142	0.25	-0.519598	0.00925517	0	-0.0383302	0.0273373	0.1608798	0.2549772
ENSG00000160972	PPP1R16A	8	145715428	C	T	0.494036	0.00557472	0.0141755	0.8200001	-0.450902	0.00754799	0	-0.0123635	0.0314387	0.6941305	0.3281683
ENSG00000160991	ORAI2	7	102085410	A	G	0.0705765	-0.00235727	0.0265567	0.7800007	-0.721167	0.014996	0	0.00326869	0.0368247	0.9292699	0.1310733
ENSG00000160993	ALKBH4	7	102101004	T	C	0.0735586	-0.00466839	0.026488	0.83	-0.22784	0.0163859	5.94E-44	0.0204898	0.116266	0.8601122	0.641351

ENSG00000160999	SH2B2	7	101945291	A	G	0.299205	-0.0221721	0.0154463	0.1800002	-0.0942859	0.00927153	2.72E-24	0.235158	0.165448	0.1552175	0.8960748
ENSG00000161010	MRNIP	5	179275304	A	G	0.0377734	-0.00449889	0.0413838	0.9	-0.340419	0.0248884	1.38E-42	0.0132157	0.121571	0.9134341	0.4985063
ENSG00000161011	SQSTM1	5	179249233	G	A	0.452286	0.01462	0.0142278	0.3100002	-0.218413	0.00787689	3.18E-169	-0.0669375	0.0651865	0.304486	0.5403519
ENSG00000161013	MGAT4B	5	179229274	C	T	0.0149105	0.0531383	0.0477113	0.1199999	-0.746893	0.0318098	6.53E-122	-0.0711458	0.0639515	0.265925	0.3583592
ENSG00000161016	RPL8	8	146016561	G	A	0.462227	0.0220972	0.01422	0.1900002	0.596565	0.0073508	0	0.0370407	0.0238408	0.1202638	0.1084624
ENSG00000161021	MAML1	5	179191681	C	T	0.498012	0.0228977	0.0142127	0.07799917	-0.0600855	0.00849506	1.52E-12	-0.381085	0.2426	0.1162201	0.04007383
ENSG00000161031	PGLYRP2	19	15594611	A	G	0.485089	-0.011786	0.0142611	0.4100001	-0.153568	0.00935945	1.68E-60	0.0767478	0.0929828	0.4091459	0.8513068
ENSG00000161036	LRWD1	7	102109495	T	C	0.264414	0.026658	0.0159739	0.1199999	0.463525	0.00821137	0	0.0575114	0.0344768	0.09529221	0.2698631
ENSG00000161040	FBXL13	7	102584297	A	G	0.0258449	0.00547823	0.0382416	0.8200001	0.938407	0.0221735	0	0.0058378	0.0407519	0.8860909	0.9531618
ENSG00000161048	NAPEPLD	7	102765115	G	A	0.0516899	-0.00395603	0.038414	0.9699999	-0.299259	0.0208805	1.38E-46	0.0132194	0.128367	0.9179777	0.7806824
ENSG00000161055	SCGB3A1	5	180017821	C	T	0.0934394	-0.00340485	0.0225428	0.9699999	1.1783	0.0112795	0	-0.00288962	0.0191316	0.8799448	0.32599
ENSG00000161057	PSMC2	7	102997271	A	G	0.0218688	-0.00644846	0.0487894	0.8700001	-0.151758	0.023841	1.95E-10	0.0424919	0.321565	0.8948729	0.3734741
ENSG00000161091	MFSD12	19	3556273	A	C	0.294235	-0.0313582	0.0163081	0.03599979	-0.316468	0.0143575	1.14E-107	0.0990882	0.0517274	0.0554179	0.04632352
ENSG00000161132	PRODHLP	22	20294011	A	G	0.459245	0.00663846	0.0142543	0.6600001	0.131834	0.0127374	4.18E-25	0.0503545	0.108232	0.6417556	0.3003934
ENSG00000161179	YDJC	22	21983365	C	T	0.248509	-0.0117025	0.0167224	0.7499995	-0.115794	0.0106145	1.04E-27	0.101063	0.144712	0.4849431	0.04121466
ENSG00000161180	CCDC116	22	21989311	T	C	0.193837	-0.0191242	0.0180032	0.14	-0.0619832	0.00993226	4.36E-10	0.308538	0.294631	0.2950055	0.6332305
ENSG00000161202	DVL3	3	183882287	A	G	0.497018	-0.0052407	0.0141949	0.5500004	-0.0729269	0.00796128	5.18E-20	0.0718624	0.194804	0.7122049	0.9178835
ENSG00000161203	AP2M1	3	183897178	T	C	0.137177	-0.017788	0.01913	0.3800004	-0.330474	0.0109128	1.90E-201	0.0538257	0.0579138	0.3526764	0.1191626
ENSG00000161204	ABCF3	3	183907805	C	A	0.0238569	-0.130766	0.0460102	0.004300015	0.396008	0.025351	5.24E-55	-0.33021	0.118092	0.005170664	0.2720189
ENSG00000161217	PCYT1A	3	195977960	G	T	0.361829	-0.00510217	0.0149107	0.5	-0.147534	0.00906588	1.52E-59	0.0345829	0.101088	0.732271	0.1111305
ENSG00000161243	FBXO27	19	39502389	C	T	0.377734	0.00237708	0.014745	0.9400001	0.0990356	0.00829167	6.98E-33	0.0240023	0.148899	0.8719375	0.3717443
ENSG00000161249	DMKN	19	35996341	A	G	0.0864811	0.0193822	0.0242384	0.4500005	-0.190321	0.0137192	9.28E-44	-0.10184	0.127567	0.4246829	0.2802919
ENSG00000161265	U2AF1L4	19	36234887	C	G	0.156064	-0.0576867	0.0201169	0.000589997	0.78308	0.0102249	0	-0.0736664	0.0257075	0.004162644	0.3349299
ENSG00000161267	BDH1	3	197268424	T	C	0.394632	-0.00913393	0.0149632	0.4500005	0.156293	0.00902407	3.35E-67	-0.0584409	0.0957974	0.5418304	0.425901
ENSG00000161277	THAP8	19	36535775	C	T	0.329026	0.013532	0.0153069	0.3800004	-0.163327	0.00840392	3.93E-84	-0.0828522	0.0938163	0.3771647	0.3853453
ENSG00000161326	NA	17	35861770	A	C	0.33499	0.0020982	0.0148004	0.89	0.637345	0.00758648	0	0.0032921	0.023222	0.8872647	0.9104361
ENSG00000161328	LRRC56	11	546221	T	G	0.112326	-0.000417451	0.0221809	0.95	-0.603549	0.0152886	0	0.00069166	0.0367508	0.9849845	0.9405546
ENSG00000161381	PLXDC1	17	37265101	G	A	0.279324	0.00638321	0.0158058	0.7600007	0.323512	0.00878926	1.40E-296	0.019731	0.0488599	0.6863391	0.7750099
ENSG00000161395	PGAP3	17	37840212	T	C	0.318091	0.0261016	0.0152852	0.14	0.354957	0.00822543	0	0.0735345	0.0430958	0.08795161	0.6873318
ENSG00000161405	IKZF3	17	37970819	T	C	0.489066	0.00139056	0.0142034	0.95	-0.359302	0.0077104	0	-0.00387017	0.0395306	0.9220092	0.3703577
ENSG00000161513	FDXR	17	72863887	G	A	0.275348	-0.0054105	0.0160787	0.7300002	-0.150377	0.010291	2.34E-48	0.0359796	0.106951	0.7365603	0.02124212
ENSG00000161533	ACOX1	17	73956551	G	A	0.311133	-0.00110727	0.0151022	0.9299999	-0.760978	0.00743965	0	0.00145506	0.0198458	0.9415527	0.8200768
ENSG00000161542	PRPSAP1	17	74343084	C	A	0.300199	-0.00333655	0.0154518	0.7899998	-0.159863	0.00849362	5.02E-79	0.0208713	0.0966626	0.8290512	0.2237136
ENSG00000161544	CYGB	17	74535347	T	C	0.222664	-0.00597512	0.0172994	0.8700001	-0.0785227	0.00990323	2.21E-15	0.0760942	0.22052	0.7300437	0.5763644
ENSG00000161547	SRSF2	17	74731826	C	T	0.502982	0.00732463	0.0142245	0.59	0.0604693	0.00796445	3.14E-14	0.12113	0.235775	0.6074265	0.7921916
ENSG00000161551	ZNF577	19	52376629	C	T	0.138171	-0.0170462	0.0223267	0.4100001	-0.20986	0.0115592	1.17E-73	0.0812266	0.106483	0.4455736	0.1102359
ENSG00000161558	TMEM143	19	48851553	A	G	0.254473	0.0357975	0.0167215	0.06199976	-0.140135	0.00910798	2.03E-53	-0.25545	0.120473	0.03397402	0.4675491
ENSG00000161570	NA	17	34203146	A	G	0.153082	0.0124274	0.0186192	0.6200004	-0.165482	0.0101268	5.04E-60	-0.0750984	0.112609	0.5048394	0.9619286
ENSG00000161618	ALDH16A1	19	49965365	T	C	0.247515	0.00222656	0.0168252	0.95	0.622423	0.0139319	0	0.00357725	0.0270319	0.8947199	0.04896223
ENSG00000161638	ITGA5	12	54801144	C	T	0.10338	-0.0224506	0.0251996	0.4100001	-0.182543	0.0168354	2.16E-27	0.122988	0.138513	0.3745845	0.4267502
ENSG00000161640	SIGLEC11	19	50458335	T	C	0.243539	-0.0196814	0.0164572	0.2700001	0.521969	0.0100885	0	-0.0377061	0.0315375	0.2318547	0.3242915
ENSG00000161642	ZNF385A	12	54773999	T	C	0.0188867	0.00870319	0.0511167	0.95	-0.427174	0.0356793	4.95E-33	-0.0203739	0.119675	0.8648183	0.7815057
ENSG00000161643	SIGLEC16	19	50475964	C	T	0.171968	0.00668427	0.0187784	0.7600007	0.294697	0.0111261	1.37E-154	0.0226819	0.0637269	0.7218982	0.134947
ENSG00000161647	MPP3	17	41894352	A	G	0.303181	-0.00550494	0.015675	0.6300007	0.0634523	0.00900621	1.85E-12	-0.0867571	0.247343	0.7257708	0.9233817
ENSG00000161653	NAGS	17	42084172	G	A	0.327038	0.00948949	0.0154552	0.3800004	-0.0664897	0.00856936	8.56E-15	-0.142721	0.233172	0.5404807	0.8242643
ENSG00000161654	LSM12	17	42128495	A	G	0.0318091	-0.00811485	0.0495427	0.8200001	0.245676	0.0315576	6.97E-15	-0.0330307	0.201704	0.869921	0.1146655
ENSG00000161664	ASB16	17	42252133	A	T	0.27336	-0.00759433	0.0161732	0.58	-0.0913638	0.00905992	6.48E-24	0.0831218	0.177212	0.6390308	0.6061329
ENSG00000161671	EMC10	19	50983132	A	G	0.337972	0.0376547	0.0148446	0.009099971	-0.116963	0.00933973	5.57E-36	-0.321936	0.129494	0.01291494	0.2229431
ENSG00000161677	JOSD2	19	51011932	C	T	0.455268	0.00286243	0.0142932	0.7600007	0.140005	0.00794637	1.77E-69	0.0204451	0.102097	0.8412833	0.05791879
ENSG00000161681	SHANK1	19	51193895	G	A	0.291252	0.0293064	0.0151525	0.02	-0.0547238	0.00902229	1.32E-09	-0.535533	0.290627	0.0653755	0.6125592

ENSG00000161692	DBF4B	17	42807804	T	C	0.146123	0.0013751	0.0199631	0.8800001	-0.462676	0.0105801	0	-0.00297206	0.043147	0.9450835	0.3073793
ENSG00000161791	FMNL3	12	50066836	C	T	0.102386	0.0007841	0.0237816	0.8200001	-0.345235	0.0133469	1.60E-147	-0.00227121	0.0688853	0.9736978	0.906094
ENSG00000161800	RACGAP1	12	50398812	G	T	0.0656064	0.00123501	0.0275024	0.95	0.219643	0.0162984	2.15E-41	0.0056228	0.125215	0.9641828	0.2096768
ENSG00000161860	SYCE2	19	13019845	A	G	0.484095	0.0125413	0.0143908	0.4500005	0.0980465	0.00998783	9.55E-23	0.127912	0.147352	0.385358	0.2250843
ENSG00000161888	SPC24	19	11254340	A	G	0.225646	-0.00610172	0.0174662	0.6499995	-0.102352	0.00943907	2.14E-27	0.0596153	0.170737	0.7269667	0.7163046
ENSG00000161904	LEMD2	6	33747946	C	T	0.167992	0.00627656	0.0183788	0.7499995	-0.240943	0.011219	2.59E-102	-0.0260499	0.0762882	0.7327515	0.6225332
ENSG00000161905	ALOX15	17	4539893	T	C	0.244533	0.0172283	0.0163798	0.25	-0.463015	0.00909998	0	-0.0372089	0.0353839	0.2929937	0.8592503
ENSG00000161911	TREML1	6	41119577	C	T	0.00397614	0.285375	0.161038	0.08999948	-1.26463	0.0751638	1.60E-63	-0.225659	0.128044	0.07801035	0.5999284
ENSG00000161912	NA	6	41088666	C	G	0.132207	-0.0156477	0.0231447	0.5700002	0.892333	0.0134789	0	-0.0175357	0.0259386	0.4990114	0.4754793
ENSG00000161914	ZNF653	19	11605490	A	T	0.0387674	-0.0262808	0.0424364	0.4299995	0.156921	0.0237027	3.58E-11	-0.167477	0.271612	0.5374942	0.7798004
ENSG00000161920	MED11	17	4635814	T	C	0.423459	0.0119594	0.0142837	0.3800004	0.116718	0.00797224	1.55E-48	0.102464	0.122577	0.4032054	0.7227624
ENSG00000161921	CXCL16	17	4640019	G	A	0.439364	0.00268638	0.0143331	0.95	-0.43896	0.00760475	0	-0.00611988	0.0326526	0.8513283	0.5243948
ENSG00000161929	SCIMP	17	5125205	A	G	0.395626	-0.00613337	0.0148302	0.8	0.26451	0.0089266	5.84E-193	-0.0231876	0.0560721	0.6792164	0.0965691
ENSG00000161944	ASGR2	17	7011830	T	C	0.300199	0.00780227	0.0152048	0.4500005	0.641008	0.0078536	0	0.0121719	0.0237206	0.6078569	0.967537
ENSG00000161955	TNFSF13	17	7463267	A	G	0.100398	-0.03556	0.0213408	0.1299999	-0.524471	0.0144999	1.76E-286	0.0678017	0.0407333	0.09600726	0.7789733
ENSG00000161956	SENP3	17	7470239	A	G	0.0795229	-0.0283064	0.0224046	0.33	0.142212	0.0138505	9.86E-25	-0.199044	0.158732	0.209856	0.8916861
ENSG00000161958	FGF11	17	7345438	A	G	0.0785288	0.0175804	0.0274074	0.4100001	-0.133644	0.0152051	1.50E-18	-0.131547	0.205624	0.5223379	0.8951504
ENSG00000161960	EIF4A1	17	7479173	T	C	0.0775348	-0.00747095	0.028815	0.84	-0.0991243	0.0174306	1.29E-08	0.0753695	0.290998	0.7956322	0.3637246
ENSG00000161973	CCDC42	17	8640894	T	C	0.50994	-0.00655707	0.0142273	0.7600007	-0.0549346	0.00805021	8.85E-12	0.119361	0.259576	0.6456369	0.8720038
ENSG00000161980	POLR3K	16	100017	C	A	0.198807	0.0357317	0.0181304	0.04399973	-0.570585	0.0120595	0	-0.0626229	0.0318027	0.04894052	0.1212347
ENSG00000161981	SNRNP25	16	105339	C	G	0.218688	0.0102534	0.0175704	0.6700003	-0.633435	0.0111476	0	-0.016187	0.0277398	0.5595364	0.01279492
ENSG00000161996	WDR90	16	708572	A	G	0.115308	-0.00484132	0.0214652	0.7700005	-0.501072	0.0158003	1.04E-220	0.00966193	0.0428397	0.8215615	0.7635003
ENSG00000161999	JMJD8	16	733100	C	T	0.456262	0.0011862	0.0144306	0.8499999	0.169907	0.0120786	6.08E-45	0.00698145	0.0849335	0.9344885	0.676263
ENSG00000162004	CCDC78	16	774768	A	G	0.469185	0.00396318	0.0142509	0.7800007	-0.117868	0.00926777	4.70E-37	-0.033624	0.120935	0.7809864	0.6535693
ENSG00000162063	CCNF	16	2494125	C	G	0.372763	-0.0158748	0.0152263	0.17	0.143837	0.00942486	1.38E-52	-0.110367	0.106105	0.2982626	0.4029824
ENSG00000162066	AMDHD2	16	2575890	T	C	0.156064	0.00779583	0.0203287	0.6300007	-0.222253	0.0164272	1.05E-41	-0.0350764	0.0915032	0.7014715	0.3746107
ENSG00000162069	BICDL2	16	3082305	A	G	0.321074	0.015859	0.0148671	0.4100001	0.464668	0.00968876	0	0.0341297	0.032003	0.2862185	0.2109807
ENSG00000162073	PAQR4	16	3021368	C	T	0.0308151	0.0114517	0.0499011	0.7600007	0.936814	0.0337591	1.74E-169	0.0122241	0.0532686	0.8184958	0.131684
ENSG00000162076	FLYWCH2	16	2941285	G	C	0.409543	-0.02076	0.0143094	0.14	0.385263	0.00765986	0	-0.0538852	0.0371573	0.1470052	0.8652197
ENSG00000162078	ZG16B	16	2884568	C	T	0.317097	-0.0165434	0.0153827	0.25	-0.384793	0.00869659	0	0.042993	0.0399884	0.2823132	0.5021031
ENSG00000162086	ZNF75A	16	3362129	G	C	0.280318	-0.0196556	0.0162106	0.17	-0.235586	0.00908959	4.15E-148	0.0834329	0.068885	0.2258224	0.2260343
ENSG00000162104	ADCY9	16	4084787	C	T	0.394632	-0.00403161	0.0148752	0.6200004	0.481577	0.00775506	0	-0.00837169	0.0308888	0.7863707	0.528933
ENSG00000162129	CLPB	11	72074580	G	A	0.0487078	-0.0403134	0.035187	0.2099999	-0.156617	0.0185856	3.55E-17	0.2574	0.226735	0.2562723	0.4530696
ENSG00000162148	SAX04	11	61253497	A	G	0.00894632	0.00897284	0.0719142	0.9	1.1502	0.102604	3.64E-29	0.00780114	0.0625273	0.9007106	0.6991085
ENSG00000162174	ASRGL1	11	62132901	A	G	0.116302	0.0138692	0.0220767	0.6100002	0.70009	0.0129975	0	0.0198106	0.0315362	0.5298826	0.2527849
ENSG00000162191	UBXN1	11	62445268	A	T	0.306163	-0.0122906	0.015645	0.3100002	0.0600361	0.00961994	4.35E-10	-0.20472	0.26265	0.4357196	0.8555146
ENSG00000162194	LBHD1	11	62435007	G	A	0.270378	-0.0131923	0.0152819	0.35	0.233426	0.0145308	4.55E-58	-0.056516	0.0655624	0.3886769	0.5982578
ENSG00000162222	TTC9C	11	62501653	T	C	0.193837	0.0290332	0.016791	0.07599937	0.288743	0.010573	3.28E-164	0.10055	0.0582684	0.08441264	0.5310354
ENSG00000162227	TAF6L	11	62546794	A	G	0.0467197	-0.0624794	0.0325661	0.03400008	-0.134421	0.0165686	4.94E-16	0.464803	0.248951	0.06189501	0.2659263
ENSG00000162231	NXF1	11	62566684	T	C	0.37674	-0.00284635	0.0147418	0.7899998	0.202612	0.00809146	2.23E-138	-0.0140483	0.072761	0.8469006	0.3412435
ENSG00000162236	STX5	11	62586964	T	C	0.0437376	-0.0837909	0.0335279	0.005999983	-0.190652	0.0172676	2.42E-28	0.439496	0.180308	0.01479012	0.1887197
ENSG00000162241	SLC25A45	11	65146917	T	C	0.084493	-0.00525156	0.0234649	0.9599999	0.226119	0.0138735	1.01E-59	-0.0232248	0.103782	0.8229255	0.08045655
ENSG00000162244	RPL29	3	52028787	T	C	0.00894632	0.00569833	0.0911104	0.9400001	-0.831631	0.112972	1.82E-13	-0.006852	0.10956	0.9501321	0.9341678
ENSG00000162267	ITIH3	3	52835904	A	T	0.340954	0.00673201	0.0150183	0.8499999	0.115426	0.00863163	8.77E-41	0.0583233	0.130185	0.6541516	0.8198444
ENSG00000162290	NA	3	53349542	T	G	0.236581	0.00322962	0.0164944	0.7099994	0.0938418	0.0093996	1.80E-23	0.0344156	0.175802	0.8447954	0.04723989
ENSG00000162302	RPS6KA4	11	64133153	A	G	0.441352	0.00754781	0.0144158	0.6899999	-0.47074	0.00756015	0	-0.0160339	0.0306248	0.6005843	0.08471788
ENSG00000162341	TPCN2	11	68837218	T	C	0.280318	0.00792798	0.0157208	0.35	-0.437016	0.00983107	0	-0.0181412	0.0359754	0.6140742	0.6884997
ENSG00000162366	PDZK1IP1	1	47652990	A	G	0.181909	-0.0113718	0.0178549	0.7899998	-0.854848	0.00969334	0	0.0133027	0.0208872	0.5242003	0.0529649
ENSG00000162367	TAL1	1	47689927	T	G	0.389662	0.00530572	0.0144196	0.4500005	-0.154916	0.00807833	5.78E-82	-0.0342489	0.093097	0.7129596	0.3696857
ENSG00000162368	CMPK1	1	47821990	G	A	0.2833	0.00779108	0.0151759	0.4799997	-0.215518	0.00878568	6.97E-133	-0.0361505	0.0704313	0.6077606	0.4802324

ENSG00000162373	BEND5	1	49217918	G	A	0.0775348	-0.0492933	0.0298177	0.09599973	-0.223037	0.0145301	3.54E-53	0.221009	0.134462	0.1002483	0.1475875
ENSG00000162377	COA7	1	53158273	C	G	0.365805	0.0132733	0.0147813	0.28	0.213651	0.00916161	2.77E-120	0.0621262	0.0692358	0.3695513	0.002823827
ENSG00000162378	ZYG11B	1	53242570	T	C	0.494036	0.0203258	0.0142035	0.25	0.450831	0.00754489	0	0.0450851	0.0315142	0.1525359	0.1166183
ENSG00000162383	SLC1A7	1	53580572	T	C	0.0397614	-0.0060586	0.0327803	0.91	-0.570105	0.0198074	3.55E-182	0.0106272	0.0574999	0.8533697	0.007764164
ENSG00000162384	CZIB	1	53683030	C	T	0.315109	0.000241216	0.0155632	0.84	0.192684	0.00926624	4.88E-96	0.00125187	0.0807706	0.987634	0.9331831
ENSG00000162390	ACOT11	1	55056397	C	A	0.460239	-0.0112284	0.0142191	0.4	0.133351	0.00804785	1.15E-61	-0.0842016	0.10675	0.4302435	0.8779358
ENSG00000162396	PARS2	1	55226379	C	G	0.427435	0.00749738	0.0142774	0.5400003	0.283906	0.00787278	9.04E-285	0.026408	0.0502945	0.5995369	0.5834158
ENSG00000162398	CIMAP2	1	55289830	A	G	0.239563	0.00174822	0.0175822	0.8800001	0.192784	0.00988373	9.92E-85	0.00906828	0.0912027	0.9207969	0.2625332
ENSG00000162402	USP24	1	55606535	C	T	0.0308151	0.0523142	0.0354378	0.08600031	-0.355626	0.0193842	3.54E-75	-0.147105	0.0999712	0.141164	0.4780814
ENSG00000162407	PLPP3	1	57035696	G	C	0.50497	0.00916222	0.0142555	0.4	0.103427	0.0080041	3.39E-38	0.088586	0.138001	0.5209244	0.7062824
ENSG00000162408	NOL9	1	6598001	G	A	0.139165	0.04113	0.0211067	0.05999983	-0.351834	0.012014	1.59E-188	-0.116902	0.0601232	0.05185078	0.9314938
ENSG00000162413	KLHL21	1	6662725	C	T	0.00795229	-0.0593993	0.0817437	0.3599996	-0.658972	0.0643528	1.31E-24	0.0901393	0.124359	0.4685562	0.9242331
ENSG00000162415	ZSWIM5	1	45626976	G	A	0.0924453	0.00436591	0.0213714	0.4799997	-0.329546	0.0133249	4.89E-135	-0.0132482	0.0648532	0.8381343	0.2072664
ENSG00000162419	GMEB1	1	29020554	A	G	0.0894632	-0.0239874	0.025605	0.4	0.0868592	0.0146361	2.95E-09	-0.276164	0.298438	0.3547755	0.9163313
ENSG00000162426	SLC45A1	1	8391056	G	A	0.489066	-0.00723337	0.0142924	0.3800004	-0.0492013	0.00874939	1.87E-08	0.147016	0.291662	0.614218	0.6513066
ENSG00000162430	SELENON	1	26135690	A	G	0.491054	-0.00962904	0.0142338	0.3400001	-0.110346	0.00848216	1.08E-38	0.0872622	0.129167	0.4993084	0.007407049
ENSG00000162433	AK4	1	65655530	A	C	0.05666	-0.0413886	0.0286018	0.1199999	-0.331362	0.015684	4.45E-99	0.124904	0.086518	0.1488289	0.2472119
ENSG00000162434	JAK1	1	65365549	G	A	0.222664	0.0042453	0.0178209	0.95	-0.0724204	0.0102283	1.44E-12	-0.0586203	0.246215	0.8118147	0.4404834
ENSG00000162437	RAVER2	1	65254846	A	C	0.00994036	0.0321113	0.0643098	0.8600001	1.94957	0.0547633	1.40E-277	0.0164709	0.0329898	0.6175871	0.9679876
ENSG00000162438	CTRC	1	15770336	T	C	0.371769	0.0215066	0.014623	0.1100001	-0.324037	0.0138002	6.44E-122	-0.0663708	0.045216	0.1421419	0.2391627
ENSG00000162441	LZIC	1	9992819	G	A	0.0238569	0.0196708	0.0435888	0.59	0.194685	0.0294512	3.83E-11	0.101039	0.224416	0.6525424	0.9004311
ENSG00000162444	RBP7	1	10066671	G	A	0.172962	-0.0240016	0.0195677	0.2700001	-0.313358	0.0102334	6.39E-206	0.0765949	0.0624953	0.2203455	0.556191
ENSG00000162461	SLC25A34	1	16065395	A	G	0.210736	-7.86E-05	0.0170104	0.9	-0.49595	0.00920702	0	0.000158478	0.0342986	0.9963133	0.2159268
ENSG00000162482	AKR7A3	1	19612398	A	G	0.27833	0.0171306	0.016359	0.33	-0.132776	0.00919285	2.76E-47	-0.129019	0.123531	0.2962887	0.9078019
ENSG00000162490	DRAXIN	1	11768850	C	T	0.180915	0.00436427	0.0181129	0.7700005	-0.331715	0.0131302	8.03E-141	-0.0131567	0.0546062	0.8096038	0.6625402
ENSG00000162496	DHRS3	1	12652838	G	A	0.38668	0.00565722	0.0147335	0.7700005	-0.152828	0.00814967	1.84E-78	-0.037017	0.0964263	0.7010604	0.3060242
ENSG00000162511	LAPTM5	1	31217991	T	C	0.272366	0.00693747	0.0161921	0.6100002	0.317408	0.00919137	2.54E-261	0.0218566	0.0510174	0.6683491	0.7491809
ENSG00000162512	SDC3	1	31361961	A	G	0.263419	0.023861	0.016424	0.08700015	-0.127197	0.008999	2.32E-45	-0.187591	0.129803	0.148402	0.3699181
ENSG00000162517	PEF1	1	32102980	T	A	0.347913	0.0205257	0.0146815	0.14	-0.156852	0.00816334	2.82E-82	-0.13086	0.0938484	0.1632031	0.4037445
ENSG00000162520	SYNC	1	33157352	T	C	0.326044	-0.00449191	0.0156442	1	0.057181	0.00999423	1.06E-08	-0.078556	0.273935	0.7742896	0.1929595
ENSG00000162521	RBBP4	1	33131500	T	C	0.0357853	0.0716489	0.0385188	0.0659994	-0.111265	0.0199829	2.58E-08	-0.643948	0.364997	0.07768864	0.136902
ENSG00000162522	NHSL3	1	33224028	C	T	0.0397614	0.0647168	0.0410018	0.1199999	-0.482786	0.021091	5.76E-116	-0.134049	0.0851292	0.1153372	0.5507336
ENSG00000162542	TMCO4	1	20067572	C	T	0.342942	0.000913696	0.0149195	0.9	-0.139058	0.00860082	8.48E-59	-0.00657061	0.107291	0.951167	0.254983
ENSG00000162543	UBXN10	1	20517559	G	C	0.152087	0.00189471	0.0191498	0.8800001	0.127447	0.0123273	4.71E-25	0.0148667	0.150264	0.9211883	0.1454214
ENSG00000162545	CAMK2N1	1	20810798	A	C	0.119284	0.0285343	0.0206932	0.1299999	-0.287459	0.0122639	1.70E-121	-0.099264	0.0721111	0.168654	0.4363481
ENSG00000162551	ALPL	1	21870381	T	C	0.250497	-0.0436661	0.0166093	0.006900014	0.13329	0.00958326	5.62E-44	-0.327603	0.126817	0.00978683	0.2210816
ENSG00000162572	SCNN1D	1	1221612	C	T	0.191849	0.00310378	0.0181556	0.6700003	-0.133483	0.0117193	4.69E-30	-0.0232522	0.136029	0.8642747	0.01020353
ENSG00000162576	MXRA8	1	1292613	T	C	0.0168986	-0.025998	0.0550601	0.6499995	0.276154	0.0500823	3.51E-08	-0.0941432	0.200112	0.6380312	0.7979061
ENSG00000162585	FAAP20	1	2130031	G	T	0.218688	0.0220756	0.0169466	0.2	-0.381859	0.0102182	1.18E-305	-0.0578109	0.0444062	0.1929622	0.4311315
ENSG00000162591	MEGF6	1	3467271	A	G	0.460239	0.0196852	0.0142905	0.09499921	-0.418872	0.0174839	7.70E-127	-0.0469958	0.034173	0.1690598	0.1413782
ENSG00000162594	IL23R	1	67678872	A	G	0.285288	-0.00547595	0.0153058	0.91	-0.170805	0.00864801	7.90E-87	0.0320596	0.0896244	0.7205597	0.5500102
ENSG00000162598	C1orf87	1	60496191	C	T	0.10338	-0.0122739	0.0248681	0.5300002	0.935949	0.0125075	0	-0.0131139	0.0265705	0.6216245	0.626613
ENSG00000162599	NFIA	1	61629698	G	A	0.27336	0.0139416	0.016727	0.3900004	-0.0906614	0.00910895	2.45E-23	-0.153777	0.185145	0.4062157	0.2948304
ENSG00000162600	OMA1	1	58946765	A	G	0.181909	0.0462049	0.0184196	0.02	0.347109	0.0104055	5.52E-244	0.133114	0.0532156	0.01237029	0.8470444
ENSG00000162601	MYSM1	1	59143087	A	G	0.377734	-0.0336742	0.0149573	0.03699985	-0.0901052	0.00832226	2.56E-27	0.373721	0.169549	0.02750983	0.7618117
ENSG00000162604	TM2D1	1	62168907	C	T	0.241551	-0.0358004	0.016956	0.03599979	0.430926	0.00922386	0	-0.0830778	0.039388	0.03492579	0.3049735
ENSG00000162607	USP1	1	62909721	T	C	0.425447	0.00406867	0.0145882	0.8200001	0.129563	0.00807223	5.68E-58	0.0314031	0.112613	0.7803528	0.5195375
ENSG00000162613	FUBP1	1	78427267	G	A	0.0397614	-0.0125856	0.0438648	0.7499995	0.155202	0.0253002	8.55E-10	-0.0810919	0.28294	0.774415	0.9648892
ENSG00000162614	NEXN	1	78381889	A	G	0.208748	0.0213572	0.0173781	0.1900002	-0.172293	0.00992526	1.69E-67	-0.123959	0.101116	0.2202349	0.7468294
ENSG00000162616	DNAJB4	1	78464253	A	G	0.208748	0.0213572	0.0173781	0.1900002	0.226779	0.00989697	3.37E-116	0.0941762	0.0767402	0.2197443	0.8725417

ENSG00000162620	LRRIQ3	1	74577785	A	C	0.464215	0.013948	0.0141908	0.32	-0.275317	0.00859017	2.18E-225	-0.0506617	0.0515678	0.3258888	0.05617475
ENSG00000162623	TYW3	1	75215599	C	G	0.314115	-0.0236047	0.0152934	0.1199999	0.205455	0.00920937	2.99E-110	-0.11489	0.0746145	0.123615	0.3570252
ENSG00000162627	SNX7	1	99176646	A	G	0.0248509	0.0776917	0.0469748	0.05800027	0.946024	0.024318	0	0.0821244	0.0496998	0.0984512	0.03809202
ENSG00000162630	B3GALT2	1	193151979	C	T	0.375746	-0.00782585	0.0143994	0.5199996	0.0648713	0.00804117	7.18E-16	-0.120637	0.222472	0.5876426	0.9261303
ENSG00000162631	NTNG1	1	107854354	T	C	0.0437376	0.00839625	0.0332728	0.7700005	0.51579	0.0198032	1.50E-149	0.0162784	0.0645115	0.800783	0.2993627
ENSG00000162636	EEIG2	1	109145116	A	G	0.0357853	0.0124858	0.0370879	0.8499999	0.327319	0.0216704	1.51E-51	0.0381457	0.113336	0.7364406	0.1914495
ENSG00000162639	HENMT1	1	109197530	C	T	0.308151	-0.0054505	0.015471	0.8200001	0.163732	0.00963537	9.28E-65	-0.0332892	0.0945102	0.7246668	0.2685118
ENSG00000162642	C1orf52	1	85720497	G	A	0.214712	-0.0041159	0.0170026	0.8200001	0.0903	0.00984647	4.70E-20	-0.0455803	0.188356	0.8087876	0.07491485
ENSG00000162643	DNAI3	1	85531825	A	G	0.327038	-0.0125321	0.0149832	0.32	-0.328709	0.00897103	6.32E-294	0.0381252	0.0455938	0.4030467	0.8585556
ENSG00000162645	GBP2	1	89593977	A	G	0.0347913	-0.0466699	0.0393356	0.2200002	-0.162238	0.0234197	4.29E-12	0.287664	0.245987	0.2422317	0.4398539
ENSG00000162650	ATXN7L2	1	110030763	C	T	0.173956	-0.022185	0.0184967	0.25	0.151891	0.0152559	2.37E-23	-0.146059	0.122657	0.2337346	0.7468815
ENSG00000162654	GBP4	1	89655723	G	T	0.304175	0.00688746	0.0155528	0.7099994	-0.229181	0.008851	7.94E-148	-0.0300525	0.0678725	0.6579256	0.8293335
ENSG00000162676	GFI1	1	92946376	A	T	0.176938	-0.0140373	0.0195136	0.5	-0.113488	0.0111082	1.67E-24	0.12369	0.17237	0.4730143	0.2357883
ENSG00000162688	AGL	1	100352609	G	T	0.32505	-0.00472703	0.0155059	0.8	-0.122616	0.00867044	2.10E-45	0.0385516	0.126489	0.7605316	0.8892074
ENSG00000162692	VCAM1	1	101194953	T	C	0.202783	0.0244819	0.0173682	0.16	-0.289136	0.00937474	7.17E-209	-0.0846727	0.0601321	0.1590979	0.2452654
ENSG00000162694	EXTL2	1	101349748	T	C	0.107356	-0.0193008	0.0246183	0.35	-0.578517	0.013386	0	0.0333625	0.0425611	0.4331145	0.6022069
ENSG00000162695	SLC30A7	1	101404470	T	C	0.138171	0.0295937	0.0217733	0.16	-0.197234	0.0129285	1.51E-52	-0.150044	0.110831	0.1757965	0.7737269
ENSG00000162702	ZNF281	1	200377505	A	G	0.264414	0.0112881	0.0160159	0.4500005	0.353944	0.0130909	5.36E-161	0.0318923	0.0452652	0.48108	0.707372
ENSG00000162704	ARPC5	1	183598646	G	A	0.400596	-0.0139664	0.0144261	0.3900004	0.549764	0.00756287	0	-0.0254043	0.0262428	0.3330203	0.5294828
ENSG00000162711	NLRP3	1	247595934	T	A	0.403579	0.0257667	0.0145091	0.07100027	0.270898	0.00799922	2.15E-251	0.095116	0.0536329	0.0761521	0.3856263
ENSG00000162714	ZNF496	1	247477931	C	T	0.335984	0.0104657	0.0145171	0.25	0.207149	0.00885458	4.85E-121	0.0505225	0.0701136	0.4711679	0.6843255
ENSG00000162722	TRIM58	1	248031004	C	T	0.257455	-0.0169236	0.0162265	0.28	-0.373895	0.00900795	0	0.045263	0.0434123	0.297119	0.9042783
ENSG00000162723	SLAMF9	1	159922663	C	A	0.0785288	0.00892963	0.0303637	0.9	0.130008	0.0167359	7.96E-15	0.0686853	0.23372	0.7688508	0.8964896
ENSG00000162729	IGSF8	1	160064931	C	T	0.190855	0.00978627	0.0172371	0.6600001	-0.188504	0.00930625	3.17E-91	-0.0519155	0.0914776	0.5703598	0.8134141
ENSG00000162734	PEA15	1	160180146	G	A	0.286282	0.00735092	0.0158172	0.64	0.334696	0.00853193	0	0.021963	0.0472618	0.6421395	0.9926677
ENSG00000162735	PEX19	1	160251370	G	A	0.0308151	-0.00355976	0.0382817	1	-0.482271	0.0235213	2.00E-93	0.00738125	0.0793789	0.9259134	0.4997192
ENSG00000162736	NCSTN	1	160320902	C	T	0.486083	-0.0105301	0.0142276	0.4299995	0.5225	0.00738453	0	-0.0201533	0.0272314	0.4592531	0.8938639
ENSG00000162739	SLAMF6	1	160473936	A	C	0.192843	-0.0216836	0.017631	0.17	-0.200111	0.0100615	5.09E-88	0.108358	0.0882743	0.2196303	0.01418024
ENSG00000162745	OLFML2B	1	161973313	A	G	0.332008	0.0183209	0.0149907	0.1100001	0.0857141	0.00848402	5.36E-24	0.213744	0.176167	0.2250129	0.5788061
ENSG00000162746	FCRLB	1	161694643	C	T	0.22664	0.0362741	0.0166265	0.01700004	0.292789	0.00902557	7.49E-231	0.123891	0.0569148	0.02949649	0.5670255
ENSG00000162747	FCGR3B	1	161597369	T	G	0.0149105	-0.00955325	0.0535536	0.64	0.788871	0.0310888	4.79E-142	-0.01211	0.067888	0.8584228	0.4406604
ENSG00000162755	KLHDC9	1	161069143	T	C	0.0477137	0.0104359	0.0313059	0.5099998	0.253206	0.0182884	1.36E-43	0.0412151	0.123674	0.7389411	0.4710997
ENSG00000162757	C1orf74	1	209956782	A	G	0.0129225	0.0388936	0.0664316	0.6899999	0.23212	0.0313922	1.42E-13	0.167558	0.28709	0.5594614	0.6314732
ENSG00000162769	FLVCR1	1	213052151	C	T	0.190855	0.0332996	0.0178362	0.04099964	0.490195	0.00963653	0	0.0679314	0.0364104	0.06208181	0.2573293
ENSG00000162772	ATF3	1	212766397	C	A	0.11332	0.018335	0.0219769	0.2999998	0.152203	0.0126526	2.49E-33	0.120464	0.144738	0.4052476	0.6196749
ENSG00000162775	RBM15	1	110885213	G	A	0.0805169	-0.0082593	0.0271679	0.5199996	-0.438473	0.0235126	1.30E-77	0.0188365	0.0619685	0.7611514	0.9011629
ENSG00000162777	DENND2D	1	111738476	G	A	0.0924453	-0.0212874	0.0228038	0.33	-0.307908	0.0126574	1.04E-130	0.0691357	0.074115	0.3509151	0.6044224
ENSG00000162783	IER5	1	181058807	G	C	0.394632	0.0068243	0.0146281	0.6800001	0.292293	0.00805065	1.26E-288	0.0233474	0.0500501	0.640871	0.4845651
ENSG00000162804	SNED1	2	241986619	T	C	0.0208748	-0.0735335	0.0462617	0.2200002	0.272519	0.032335	3.52E-17	-0.269829	0.172749	0.1182939	0.8104291
ENSG00000162813	BPNT1	1	220247314	G	C	0.149105	0.0534539	0.018385	0.001499996	0.0955869	0.0102293	9.24E-21	0.559218	0.201433	0.005499952	0.06620968
ENSG00000162817	C1orf115	1	220867843	G	A	0.2167	-0.0191599	0.0179129	0.2099999	0.470546	0.00908512	0	-0.0407185	0.0380765	0.2848955	0.2426666
ENSG00000162819	BROX	1	222897216	A	G	0.0228628	-0.0304792	0.0411667	0.5700002	0.8192	0.0260092	9.67E-218	-0.037206	0.0502662	0.4591906	0.5975288
ENSG00000162836	ACP6	1	147130894	G	A	0.284294	0.0270426	0.0155532	0.09099971	-0.552953	0.012304	0	-0.0489057	0.0281485	0.08231371	0.2985317
ENSG00000162849	KIF26B	1	245595510	C	A	0.0755467	-0.068985	0.0291918	0.01700004	0.174457	0.0165774	6.71E-26	-0.395426	0.171496	0.02112486	0.3839828
ENSG00000162851	TFB2M	1	246716744	G	A	0.501988	0.0131525	0.0142269	0.32	0.0810177	0.00798047	3.25E-24	0.162341	0.176329	0.3572219	0.2805985
ENSG00000162852	CNST	1	246780816	G	T	0.476143	0.00718251	0.0142435	0.7700005	0.111026	0.0079207	1.22E-44	0.0646921	0.128373	0.6143037	0.6345304
ENSG00000162869	PPP1R21	2	48705131	T	C	0.0506958	0.0702515	0.0312304	0.01499996	-0.407845	0.0187651	9.75E-105	-0.172251	0.0769833	0.02525324	0.1371398
ENSG00000162873	KLHDC8A	1	205315693	T	C	0.154076	-0.019461	0.0196948	0.2999998	0.0753545	0.0110193	8.01E-12	-0.258259	0.264076	0.3280882	0.5243688
ENSG00000162877	PM20D1	1	205808205	A	G	0.39662	-0.00292304	0.014417	0.89	-0.34657	0.00789323	0	0.00843419	0.0415995	0.8393325	0.3133808
ENSG00000162878	PKDCC	2	42280414	A	C	0.390656	0.0021738	0.0145265	0.9199999	-0.115146	0.00871964	8.17E-40	-0.0188786	0.126165	0.8810533	0.5434776

ENSG00000162881	OXER1	2	42990521	T	C	0.240557	-0.00879804	0.0173934	0.5700002	-0.222275	0.00944076	1.44E-122	0.0395818	0.0782697	0.6130605	0.3864437
ENSG00000162882	HAAO	2	43006981	G	A	0.241551	0.0339509	0.0162029	0.02100003	-0.16011	0.00993176	1.82E-58	-0.212048	0.10205	0.03772031	0.04162151
ENSG00000162885	B3GALNT2	1	235640509	A	C	0.511928	0.0111818	0.0141943	0.2999998	0.605472	0.0071805	0	0.0184679	0.0234444	0.4308532	0.6910531
ENSG00000162888	IKBKE-AS1	1	206667755	T	C	0.232604	-0.0465766	0.0170507	0.005800027	-0.377969	0.0236989	2.90E-57	0.123229	0.0457683	0.007092993	0.9650949
ENSG00000162889	MAPKAPK2	1	206882958	A	G	0.316103	-0.00452596	0.0154791	0.5999997	0.133297	0.012624	4.62E-26	-0.0339539	0.116169	0.7700728	0.2949045
ENSG00000162892	IL24	1	207074136	C	T	0.475149	-0.011997	0.0142064	0.3400001	0.145037	0.00872685	5.03E-62	-0.082717	0.0980767	0.3990101	0.913969
ENSG00000162894	FCMR	1	207087161	G	A	0.11332	0.0172754	0.0218311	0.32	-0.284902	0.0133364	2.97E-101	-0.0606363	0.0766793	0.4290734	0.9815227
ENSG00000162909	CAPN2	1	223926507	T	C	0.0129225	-0.12364	0.0740028	0.08300036	-0.977535	0.0644387	5.59E-52	0.126481	0.0761612	0.09677251	0.9285267
ENSG00000162910	MRPL55	1	228295696	C	T	0.299205	0.000750917	0.0151568	0.98	-0.176663	0.00856556	1.64E-94	-0.00425057	0.0857953	0.9604865	0.3253145
ENSG00000162913	OBSCN-AS1	1	228396286	C	G	0.362823	-0.00760449	0.0145256	0.7300002	-0.34836	0.0190323	7.75E-75	0.0218294	0.0417142	0.600759	0.08231204
ENSG00000162924	REL	2	61129650	C	T	0.173956	-0.0167116	0.0200358	0.3800004	0.171806	0.0107164	7.64E-58	-0.0972703	0.116777	0.404867	0.2248171
ENSG00000162927	PUS10	2	61206375	C	G	0.201789	0.00596388	0.0184982	0.6499995	0.428838	0.0146832	1.63E-187	0.0139071	0.0431382	0.7471621	0.5061645
ENSG00000162928	PEX13	2	61260377	C	T	0.415507	-0.00806083	0.0142931	0.6600001	-0.0774933	0.00797289	2.49E-22	0.10402	0.184753	0.5734216	0.2755014
ENSG00000162929	SANBR	2	61342483	C	T	0.409543	0.000401574	0.014434	0.9699999	0.605747	0.00871354	0	0.00066294	0.0238284	0.9778046	0.1288056
ENSG00000162931	TRIM17	1	228600101	G	A	0.0765408	-0.0429092	0.0252622	0.04900044	0.0757929	0.0134892	1.92E-08	-0.566137	0.348202	0.1039737	0.9710908
ENSG00000162944	RFTN2	2	198488146	G	A	0.217694	0.0187572	0.0177836	0.33	-0.146866	0.0103805	1.92E-45	-0.127717	0.121424	0.2928784	0.1959994
ENSG00000162946	DISC1	1	231969789	A	C	0.408549	0.0115879	0.0144878	0.4100001	-0.385053	0.00778286	0	-0.0300943	0.0376304	0.4238649	0.8474019
ENSG00000162949	CAPN13	2	30994522	A	G	0.362823	-0.0245314	0.0146715	0.08600031	-0.0761365	0.00819712	1.57E-20	0.322203	0.195797	0.09984757	0.159278
ENSG00000162961	DPY30	2	32178879	C	A	0.175944	0.0294771	0.0194961	0.1499999	0.0708896	0.0117484	1.60E-09	0.415817	0.283523	0.1424827	0.9277875
ENSG00000162972	MAIP1	2	200846651	A	G	0.178926	-0.0317195	0.0179193	0.16	0.0816737	0.0118364	5.19E-12	-0.388369	0.226506	0.08641661	0.669327
ENSG00000162976	SLC66A3	2	11307162	G	A	0.11332	0.0505374	0.0225156	0.0129999	-0.480882	0.0135147	2.64E-277	-0.105093	0.0469145	0.02508456	0.3586237
ENSG00000162980	ARL5A	2	152665252	G	A	0.365805	-0.0205558	0.0148553	0.1900002	0.142775	0.00905677	5.47E-56	-0.143974	0.104447	0.168069	0.7467678
ENSG00000162994	CLHC1	2	55430813	G	T	0.159046	-0.00559178	0.0192869	0.7700005	0.984235	0.0107756	0	-0.00568134	0.0195959	0.7718737	0.8766412
ENSG00000162997	PRORSD1P	2	55510556	G	A	0.0377734	-0.0112812	0.0478819	0.7800007	0.558282	0.029711	9.05E-79	-0.020207	0.0857732	0.8137538	0.7258854
ENSG00000162999	DUSP19	2	183954010	T	C	0.0536779	-0.0425252	0.0345672	0.17	0.401184	0.0289121	8.85E-44	-0.105999	0.086501	0.2204202	0.3964141
ENSG00000163001	CFAP36	2	55759877	C	T	0.373757	0.0411807	0.0145171	0.003799969	-0.169826	0.00807979	4.43E-98	-0.242488	0.0862572	0.004935466	0.5922753
ENSG00000163002	NUP35	2	184004324	G	A	0.199801	0.0288303	0.0174825	0.05099998	0.205884	0.00987491	1.55E-96	0.140032	0.0851795	0.1001844	0.4779072
ENSG00000163006	CCDC138	2	109448123	T	G	0.0526839	-0.0011798	0.0294469	0.98	-0.192453	0.0166716	7.93E-31	0.00613032	0.153009	0.9680412	0.0173064
ENSG00000163009	NA	2	10316680	T	C	0.428429	-0.0108454	0.0143932	0.3900004	0.0470254	0.00808831	6.10E-09	-0.230628	0.308632	0.4549073	0.6465597
ENSG00000163013	FBXO41	2	73496684	G	A	0.141153	0.0193895	0.0203074	0.28	-0.210828	0.0128588	2.06E-60	-0.0919684	0.0964853	0.3404964	0.08083096
ENSG00000163016	NA	2	73892374	T	C	0.208748	-0.00470713	0.0171007	0.83	0.39874	0.0171114	4.17E-120	-0.011805	0.0428899	0.7831316	0.528771
ENSG00000163017	ACTG2	2	74133216	G	A	0.133201	0.0264897	0.0214254	0.2700001	-0.133957	0.0119743	4.72E-29	-0.197748	0.160917	0.2191143	0.5757032
ENSG00000163026	WDCP	2	24262327	G	A	0.141153	0.00322888	0.0202561	0.84	0.137628	0.0111391	4.55E-35	0.0234609	0.147192	0.8733619	0.8901503
ENSG00000163029	SMC6	2	17913294	A	G	0.175944	-0.00342032	0.0186133	0.98	-0.567561	0.00964541	0	0.00602635	0.0327954	0.854205	0.777844
ENSG00000163040	CCDC74A	2	132288243	C	T	0.323062	0.00208263	0.0156703	0.9400001	0.525394	0.0221985	7.70E-124	0.00396394	0.0298263	0.8942717	0.04791261
ENSG00000163041	H3-3A	1	226254627	T	C	0.285288	-0.00908778	0.016286	0.5500004	0.65011	0.0163481	0	-0.0139788	0.0250536	0.5768738	0.9608031
ENSG00000163050	COQ8A	1	227130241	T	C	0.506958	-0.000772172	0.0142002	0.95	-0.69708	0.00758397	0	0.00110772	0.020371	0.9566344	0.4232402
ENSG00000163069	SGCB	4	52895760	T	G	0.254473	0.0112428	0.0155721	0.2599998	-0.192242	0.00874295	3.74E-107	-0.0584826	0.0810463	0.4705437	0.6667998
ENSG00000163071	SPATA18	4	52940477	C	A	0.427435	0.0133816	0.0143041	0.2599998	-0.188437	0.00805343	4.44E-121	-0.0710136	0.0759697	0.3499114	0.8828375
ENSG00000163082	SGPP2	2	223357451	C	T	0.432406	0.000991552	0.0144403	0.81	0.0526336	0.00806225	6.65E-11	0.0188388	0.27437	0.9452588	0.9350244
ENSG00000163083	INHBB	2	121106551	G	A	0.292247	0.0143963	0.0153573	0.29	-0.25588	0.00836275	1.32E-205	-0.0562619	0.0600457	0.3487667	0.361727
ENSG00000163093	BBS5	2	170359060	A	C	0.084493	-0.0673886	0.025325	0.003599979	0.0998746	0.0132071	3.96E-14	-0.674732	0.268808	0.01207003	0.7712935
ENSG00000163104	SMARCAD1	4	95170602	C	T	0.389662	-0.0132915	0.0145157	0.1900002	-0.227723	0.00849413	2.50E-158	0.0583669	0.0637799	0.3601233	0.647634
ENSG00000163106	HPGDS	4	95241856	C	T	0.0238569	0.0325098	0.0390161	0.35	0.146576	0.023498	4.44E-10	0.221795	0.268548	0.4088581	0.09390545
ENSG00000163110	PDLIM5	4	95481207	C	T	0.445328	0.0217764	0.0143406	0.1100001	0.0886117	0.00800641	1.80E-28	0.245751	0.163353	0.1324733	0.01749481
ENSG00000163113	NA	1	149947428	T	C	0.100398	-0.00032518	0.0244917	0.9299999	0.234877	0.0211872	1.47E-28	-0.00138447	0.104275	0.9894067	0.4690565
ENSG00000163125	RPRD2	1	150392304	T	C	0.380716	0.0120629	0.0145122	0.35	-0.148454	0.0120316	5.61E-35	-0.0812571	0.0979774	0.4069093	0.6837108
ENSG00000163126	ANKRD23	2	97506967	T	C	0.0854871	0.0170567	0.0275277	0.5400003	-0.133848	0.01562	1.04E-17	-0.127434	0.206201	0.5365714	0.7550076
ENSG00000163131	CTSS	1	150720492	C	G	0.431412	-0.0108881	0.0144539	0.5199996	0.334136	0.00858616	0	-0.0325859	0.0432657	0.4513554	0.677433
ENSG00000163138	PACRGL	4	20726217	C	T	0.492048	-0.0242173	0.0142169	0.16	0.121024	0.00871603	7.79E-44	-0.200104	0.118353	0.09088628	0.8622889

ENSG00000163141	BNIPL	1	151014561	A	G	0.228628	-0.00645709	0.016832	0.56	0.337094	0.00893126	0	-0.0191552	0.0499353	0.7012749	0.9924215
ENSG00000163154	TNFAIP8L2	1	151130665	A	G	0.0308151	-0.0601716	0.0474311	0.1800002	-0.401473	0.0249703	3.64E-58	0.149877	0.11851	0.2059861	0.9344396
ENSG00000163155	LYSMD1	1	151135324	T	A	0.193837	0.000799631	0.0175735	0.99	-0.157205	0.0103981	1.22E-51	-0.00508656	0.111788	0.9637073	0.8713914
ENSG00000163156	SCNM1	1	151135956	A	G	0.0765408	0.019621	0.0256889	0.25	0.541359	0.0282031	4.07E-82	0.036244	0.0474902	0.4453507	0.9835159
ENSG00000163161	ERCC3	2	128033309	A	G	0.249503	0.0139361	0.0156147	0.4100001	-0.376903	0.00866654	0	-0.0369753	0.0414377	0.3722263	0.4240995
ENSG00000163162	RNF149	2	101906422	G	A	0.247515	-0.00515297	0.0168131	0.84	0.12923	0.00926909	3.52E-44	-0.0398745	0.130134	0.7592914	0.3367051
ENSG00000163166	IWS1	2	128239122	A	T	0.167992	0.0214271	0.0181324	0.3400001	0.216484	0.010281	1.98E-98	0.0989776	0.0838903	0.2380615	0.04109964
ENSG00000163170	BOLA3	2	74368823	G	T	0.388668	0.00333563	0.0146405	0.83	0.0505795	0.00884475	1.07E-08	0.0659483	0.289685	0.8199143	0.6959767
ENSG00000163171	CDC42EP3	2	37917321	C	A	0.22664	-0.00688862	0.0172464	0.7899998	0.211939	0.00938513	6.45E-113	-0.0325029	0.0813872	0.6896271	0.1871931
ENSG00000163191	S100A11	1	152012682	T	C	0.0328032	-0.022988	0.0355968	0.6700003	0.377724	0.0196033	9.89E-83	-0.0608592	0.0942931	0.5186512	0.9311167
ENSG00000163214	DHX57	2	39063973	C	T	0.298211	-0.000793343	0.0153511	0.9599999	-0.254496	0.00847997	6.97E-198	0.00311731	0.0603198	0.9587838	0.2328192
ENSG00000163219	ARHGAP25	2	68980349	A	G	0.385686	0.00242679	0.0146535	0.7700005	0.1877	0.00812223	3.72E-118	0.0129291	0.0780708	0.8684659	0.2438087
ENSG00000163220	S100A9	1	153331916	T	C	0.104374	0.0216119	0.0222496	0.17	0.388978	0.0128542	3.77E-201	0.0555607	0.0572296	0.3316286	0.5977941
ENSG00000163221	S100A12	1	153347154	G	A	0.104374	0.0232486	0.022232	0.1499999	0.947158	0.0117999	0	0.0245457	0.0234743	0.2957281	0.5305125
ENSG00000163235	TGFA	2	70727868	T	C	0.109344	0.0193755	0.0218792	0.3400001	0.265023	0.0125394	3.78E-99	0.0731087	0.0826282	0.3762695	0.6522077
ENSG00000163249	CCNYL1	2	208601413	A	C	0.082505	0.0205028	0.0273048	0.3900004	-0.162596	0.0142999	5.87E-30	-0.126097	0.168296	0.453704	0.797984
ENSG00000163251	FZD5	2	208630798	T	C	0.471173	-0.00268974	0.0143627	1	-0.0632122	0.00859235	1.88E-13	0.042551	0.227288	0.8514945	0.332496
ENSG00000163257	DCAF16	4	17807329	A	C	0.290258	-0.00690121	0.0157367	0.6999999	0.166512	0.00875597	1.23E-80	-0.0414456	0.0945328	0.6610776	0.3037227
ENSG00000163281	GNPDA2	4	44716248	C	G	0.244533	-0.0245157	0.0169148	0.1499999	0.169072	0.0094606	1.98E-71	-0.145001	0.100373	0.1485644	0.9235352
ENSG00000163291	PAQR3	4	79834436	T	A	0.0437376	0.00856088	0.0345487	0.7899998	0.17589	0.0184929	1.88E-21	0.0486718	0.196489	0.8043606	0.3940952
ENSG00000163293	NIPAL1	4	47979173	G	A	0.327038	-0.0207659	0.0152937	0.1800002	0.231885	0.00845827	1.81E-165	-0.0895525	0.0660346	0.175053	0.2342279
ENSG00000163297	ANTXR2	4	80934455	G	A	0.407555	0.00809089	0.0144885	0.4799997	-0.234441	0.00798267	1.38E-189	-0.0345115	0.0618115	0.5766161	0.6018384
ENSG00000163312	HELQ	4	84352752	A	G	0.16004	-0.018656	0.0195819	0.2599998	-0.108198	0.0160683	1.66E-11	0.172425	0.182785	0.3455166	0.8298887
ENSG00000163319	MRPS18C	4	84383986	C	T	0.513917	-0.00102386	0.0141957	0.81	0.141556	0.0079482	5.93E-71	-0.00723291	0.100284	0.9425032	0.7633433
ENSG00000163320	CGGBP1	3	88150064	C	A	0.122266	-0.0301431	0.0212456	0.1199999	0.565119	0.0116037	0	-0.0533393	0.0376108	0.156135	0.07917243
ENSG00000163322	ABRAXAS1	4	84413296	G	A	0.513917	-0.00188975	0.0141975	0.7600007	0.254529	0.0116843	3.30E-105	-0.00742449	0.0557805	0.8941128	0.8044154
ENSG00000163328	GPR155	2	175324394	A	C	0.319085	-0.00163524	0.0148088	0.9199999	-0.182635	0.00840839	1.31E-104	0.00895358	0.0810851	0.9120746	0.5395113
ENSG00000163344	PMVK	1	154903338	A	G	0.437376	-0.00302621	0.014349	0.9599999	-0.145386	0.00794798	9.55E-75	0.020815	0.0987025	0.8329759	0.48968
ENSG00000163346	PBXIP1	1	154922575	G	C	0.437376	-0.00227753	0.0143461	0.9199999	-0.166814	0.00795263	1.09E-97	0.0136531	0.0860029	0.8738647	0.2083393
ENSG00000163349	HIPK1	1	114496120	A	G	0.431412	0.0136285	0.0143493	0.3100002	0.0765271	0.00815785	6.55E-21	0.178087	0.188465	0.3446911	0.1546239
ENSG00000163359	COL6A3	2	238277832	G	C	0.0785288	-0.0293299	0.0257519	0.29	-0.358537	0.0146843	1.15E-131	0.0818044	0.071903	0.2552441	0.0147625
ENSG00000163362	INAVA	1	200872519	T	C	0.506958	-0.0271746	0.0143018	0.04300015	-0.107848	0.0142792	4.26E-14	0.251972	0.136743	0.06537769	0.9648779
ENSG00000163374	YY1AP1	1	155644014	C	T	0.297217	0.00140738	0.0157718	0.9199999	-0.158963	0.00872042	3.05E-74	-0.00885352	0.0992182	0.9288968	0.8271376
ENSG00000163376	KBTBD8	3	67055180	G	T	0.467197	-0.000131871	0.0142342	0.9299999	-0.0800263	0.00794763	7.56E-24	0.00164785	0.177869	0.9926082	0.1588853
ENSG00000163378	EOGT	3	69043738	A	C	0.255467	-0.0208512	0.0165743	0.17	-0.313618	0.00976347	2.20E-226	0.0664859	0.0528891	0.2087245	0.7358088
ENSG00000163382	NAXE	1	156562822	C	T	0.295229	0.0297002	0.0159052	0.1100001	-0.257715	0.00858881	8.22E-198	-0.115244	0.0618356	0.0623609	0.5928524
ENSG00000163386	NA	1	145330037	T	C	0.379722	0.0105054	0.0148998	0.4899999	-0.102789	0.0181503	1.49E-08	-0.102204	0.146074	0.4841346	0.8466616
ENSG00000163389	POGLUT1	3	119200670	A	G	0.0725646	0.0119816	0.0288274	0.6700003	1.01357	0.0171485	0	0.0118212	0.0284421	0.6776866	0.2149779
ENSG00000163393	SLC22A15	1	116565897	A	G	0.432406	0.00291597	0.0143627	0.95	0.498282	0.00750779	0	0.00585205	0.0288246	0.8391172	0.4821016
ENSG00000163406	SLC15A2	3	121637942	A	G	0.382704	-0.00113215	0.0147224	0.91	0.244741	0.00813613	8.64E-199	-0.0046259	0.0601551	0.9387034	0.9348474
ENSG00000163412	EIF4E3	3	71766182	C	T	0.347913	-0.0234929	0.014969	0.17	-0.442803	0.00808371	0	0.053055	0.033819	0.1166962	0.406697
ENSG00000163421	PROK2	3	71827582	T	C	0.224652	0.000584129	0.0167677	0.95	-0.458923	0.00853365	0	-0.00127283	0.0365371	0.9722101	0.5206435
ENSG00000163428	LRRC58	3	120055771	A	C	0.133201	0.0173531	0.0228195	0.4100001	-0.68338	0.0158947	0	-0.025393	0.0333973	0.4470564	0.1321875
ENSG00000163430	FSTL1	3	120140620	T	C	0.353877	0.00122827	0.014679	0.9699999	-0.285822	0.00919339	3.30E-212	-0.00429733	0.0513574	0.9333148	0.2761645
ENSG00000163431	LMOD1	1	201890647	T	C	0.289264	0.00793824	0.0162517	0.56	-0.148005	0.00900143	9.51E-61	-0.0536349	0.109853	0.6253794	0.6549101
ENSG00000163435	ELF3	1	201981694	G	A	0.390656	0.0111404	0.0145229	0.64	-0.167429	0.0080429	3.04E-96	-0.066538	0.0867995	0.4433362	0.1677993
ENSG00000163444	TMEM183A	1	202985245	C	T	0.512922	0.00109892	0.0141796	0.7700005	-0.254627	0.009572	6.57E-156	-0.00431581	0.055688	0.938226	0.3283024
ENSG00000163449	TMEM169	2	216957047	C	T	0.305169	-0.00591117	0.0160266	0.5999997	0.22067	0.00876244	6.06E-140	-0.0267874	0.0726349	0.7122802	0.6029563
ENSG00000163453	IGFBP7	4	57936745	T	C	0.379722	-0.0107008	0.0145991	0.6499995	0.206134	0.00814722	3.10E-141	-0.0519118	0.0708529	0.4637602	0.6889064
ENSG00000163462	TRIM46	1	155151660	T	C	0.477137	0.00633841	0.0141814	0.5500004	0.0724945	0.00793643	6.58E-20	0.087433	0.195854	0.6552951	0.1270947

ENSG00000163463	KRTCAP2	1	155150815	A	G	0.49503	-0.0289386	0.0141866	0.02900013	-0.0651913	0.00796123	2.64E-16	0.443903	0.224265	0.04777506	0.2003578
ENSG00000163464	CXCR1	2	219029643	T	C	0.499006	-0.00834269	0.0142075	0.6600001	-0.317695	0.00774319	0	0.02626	0.0447251	0.5571076	0.9384062
ENSG00000163466	ARPC2	2	219100448	A	C	0.478131	-0.00282268	0.0142145	0.9599999	-0.100878	0.00794928	6.70E-37	0.0279811	0.140925	0.8426123	0.8598605
ENSG00000163468	CCT3	1	156308211	T	A	0.269384	0.000431374	0.0156258	0.9299999	-0.328711	0.0086039	0	-0.00131232	0.0475366	0.977976	0.3019333
ENSG00000163472	TMEM79	1	156257851	C	T	0.536779	-0.00424359	0.0141903	0.7499995	0.471347	0.00751618	0	-0.00900311	0.0301062	0.7649055	0.4875557
ENSG00000163481	RNF25	2	219532860	C	T	0.027833	0.0559257	0.0384071	0.1800002	-0.125725	0.0211197	2.63E-09	-0.444824	0.31449	0.1572356	0.976242
ENSG00000163482	STK36	2	219552094	T	G	0.377734	0.00299248	0.0145099	0.6700003	-0.168898	0.00855715	1.03E-86	-0.0177177	0.0859141	0.8366145	0.2884449
ENSG00000163485	ADORA1	1	203098157	T	C	0.335984	-0.0169651	0.0150822	0.4199997	0.505763	0.00803558	0	-0.0335435	0.0298254	0.2607319	0.08054917
ENSG00000163486	NA	1	206576990	T	C	0.240557	0.00805043	0.015798	0.3700002	-0.26487	0.00985671	4.67E-159	-0.0303939	0.0596552	0.6104062	0.5978129
ENSG00000163491	NEK10	3	27281263	C	T	0.271372	0.0143257	0.0165428	0.6899999	-0.0649816	0.0102969	2.78E-10	-0.220458	0.256962	0.3909266	0.8128142
ENSG00000163492	CCDC141	2	179804648	G	A	0.338966	0.00919909	0.0151258	0.5300002	-0.126124	0.00840378	6.51E-51	-0.0729369	0.120027	0.5434041	0.07518879
ENSG00000163507	CIP2A	3	108288603	A	G	0.133201	-0.00139728	0.020515	0.7700005	0.319215	0.0111639	8.07E-180	-0.00437724	0.0642672	0.9456981	0.1873936
ENSG00000163508	EOMES	3	27760823	G	A	0.177932	-0.0230248	0.0187257	0.33	0.19838	0.0102728	4.32E-83	-0.116064	0.0945841	0.2197858	0.1914057
ENSG00000163510	CWC22	2	180840721	T	C	0.282306	0.0221473	0.0169058	0.28	0.150046	0.0135162	1.24E-28	0.147604	0.113453	0.1932537	0.02436947
ENSG00000163512	AZI2	3	28373556	G	A	0.476143	-0.0285169	0.0142031	0.06100002	0.245153	0.00786785	3.84E-213	-0.116323	0.0580557	0.04510878	0.6173541
ENSG00000163513	TGFBR2	3	30691814	A	G	0.124254	0.00646608	0.0209888	0.8499999	0.393963	0.0116574	2.33E-250	0.0164129	0.0532782	0.7580366	0.3050129
ENSG00000163516	ANKZF1	2	220097935	T	C	0.0437376	0.018347	0.0322158	0.4700002	0.304916	0.0219668	8.28E-44	0.0601706	0.105743	0.5693396	0.3262516
ENSG00000163517	HDAC11	3	13534570	G	C	0.427435	-0.0160582	0.0152217	0.25	-0.0856853	0.00856117	1.40E-23	0.187409	0.178631	0.2941126	0.02901854
ENSG00000163519	TRAT1	3	108557698	G	A	0.0904573	-0.00543684	0.0221067	0.8	0.421021	0.0115903	6.43E-289	-0.0129135	0.0525086	0.8057359	0.715164
ENSG00000163520	FBLN2	3	13626873	A	T	0.292247	-0.0193293	0.0150976	0.35	0.0514771	0.00850664	1.44E-09	-0.375493	0.29978	0.2103647	0.4099702
ENSG00000163521	GLB1L	2	220105764	T	C	0.178926	0.0101772	0.0183534	0.6200004	-0.743786	0.00991184	0	-0.013683	0.0246763	0.5792381	0.8795276
ENSG00000163527	STT3B	3	31626621	A	G	0.0765408	0.00897009	0.0238961	0.7899998	-0.274014	0.0133439	1.05E-93	-0.0327359	0.0872221	0.7074254	0.259162
ENSG00000163528	CHCHD4	3	14159975	C	T	0.248509	0.0042199	0.0155451	0.6300007	-0.0799847	0.0130527	8.91E-10	-0.0527589	0.194542	0.7862405	0.7662298
ENSG00000163534	FCRL1	1	157777044	A	T	0.402584	0.00321866	0.0144983	0.7700005	0.153208	0.00799414	7.26E-82	0.0210085	0.0946381	0.8243235	0.3409343
ENSG00000163535	SGO2	2	201411618	T	C	0.201789	0.00533448	0.0171879	0.8200001	-0.0958339	0.00956522	1.26E-23	-0.0556638	0.179437	0.7563984	0.6298248
ENSG00000163536	SERPINI1	3	167498193	T	C	0.223658	0.0266557	0.0167796	0.1199999	-0.141609	0.00945144	9.51E-51	-0.188234	0.119157	0.1141703	0.1774342
ENSG00000163539	CLASP2	3	33648792	C	A	0.274354	-0.0113454	0.0162193	0.5700002	-0.0904021	0.00903479	1.43E-23	0.125499	0.179851	0.4853042	0.3614197
ENSG00000163541	SUCLG1	2	84668908	C	T	0.172962	-0.0201733	0.0174584	0.25	0.133359	0.00978262	2.58E-42	-0.151271	0.131383	0.2495768	0.9681401
ENSG00000163545	NUAK2	1	205281035	G	A	0.421471	-0.0160023	0.0146126	0.33	0.287756	0.00804032	1.63E-280	-0.0556107	0.050805	0.2736961	0.6155684
ENSG00000163554	SPTA1	1	158618383	T	C	0.238569	0.0223179	0.0167762	0.16	0.0962165	0.00967642	2.69E-23	0.231955	0.175912	0.1873088	0.895682
ENSG00000163558	PRKCI	3	169981961	C	A	0.482107	0.0160514	0.0142868	0.3100002	-0.093906	0.0118258	2.01E-15	-0.170931	0.153655	0.2659519	0.9432213
ENSG00000163563	MNDA	1	158810201	G	A	0.417495	0.0123177	0.0143732	0.3800004	-0.341109	0.00784915	0	-0.0361107	0.0421448	0.391542	0.06535137
ENSG00000163564	PYHIN1	1	158923715	T	C	0.152087	0.00126225	0.0206474	0.9400001	-0.334161	0.0107668	1.76E-211	-0.00377737	0.0617889	0.9512529	0.05468504
ENSG00000163565	IFI16	1	158997351	C	A	0.162028	-0.0375545	0.0188038	0.01899984	0.23814	0.00999898	2.26E-125	-0.157699	0.0792384	0.04656992	0.3231897
ENSG00000163568	AIM2	1	159074580	T	C	0.0407555	-0.0184276	0.0320649	0.59	-0.390991	0.016475	1.67E-124	0.0471305	0.0820333	0.5656102	0.02557865
ENSG00000163576	EFHB	3	19954740	T	G	0.349901	0.0147693	0.0145411	0.3400001	0.562249	0.00845339	0	0.0262682	0.0258654	0.3098318	0.6717677
ENSG00000163577	EIF5A2	3	170616343	T	C	0.387674	0.0290924	0.0144829	0.0329997	-0.0703095	0.00822641	1.27E-17	-0.413776	0.211601	0.05052891	0.9691086
ENSG00000163584	RPL22L1	3	170585468	G	A	0.232604	-0.00213757	0.016303	0.9599999	0.9086	0.00877172	0	-0.0023526	0.017943	0.8956843	0.04955088
ENSG00000163590	PPM1L	3	160635042	T	C	0.0417495	-0.00165483	0.0319066	0.99	-0.659011	0.0180088	3.58E-293	0.00251108	0.0484159	0.9586364	0.7981795
ENSG00000163596	ICA1L	2	203688699	A	G	0.450298	-0.013788	0.0143339	0.2599998	-0.256216	0.00784349	4.75E-234	0.0538141	0.0559689	0.3363015	0.1252282
ENSG00000163597	SNHG16	17	74557639	G	A	0.385686	-0.00336011	0.014647	0.9400001	0.571424	0.0118836	0	-0.00588024	0.0256328	0.8185554	0.9477123
ENSG00000163599	CTLA4	2	204735596	A	G	0.450298	-0.0172946	0.0143471	0.1	0.142302	0.00802436	2.30E-70	-0.121534	0.101054	0.2291052	0.2013812
ENSG00000163600	ICOS	2	204813885	A	G	0.188867	-0.0126524	0.0181228	0.3400001	0.124287	0.00977392	4.81E-37	-0.1018	0.146034	0.4857412	0.4928178
ENSG00000163602	RYBP	3	72459906	C	G	0.427435	0.00645858	0.0143097	0.6999999	0.451649	0.00756624	0	0.0143	0.0316841	0.6517518	0.6099576
ENSG00000163606	CD200R1	3	112667012	A	C	0.299205	0.0142264	0.0151348	0.17	0.395721	0.00843985	0	0.0359506	0.0382538	0.347325	0.4087317
ENSG00000163607	GTPBP8	3	112721836	C	G	0.277336	-0.00912153	0.0162669	0.2999998	-0.269431	0.0087651	1.71E-207	0.0338548	0.060385	0.5750372	0.7869226
ENSG00000163608	NEPRO	3	112729997	A	G	0.0417495	0.0297402	0.041985	0.58	0.606982	0.0208294	1.10E-186	0.0489968	0.0691905	0.478856	0.7883922
ENSG00000163617	CCDC191	3	113729222	T	G	0.304175	-0.012828	0.0158453	0.3700002	0.174922	0.00970754	1.38E-72	-0.0733355	0.0906763	0.4186522	0.05633726
ENSG00000163624	CDS1	4	85538311	C	A	0.397614	0.0138488	0.0144512	0.33	0.0597826	0.00810063	1.58E-13	0.231653	0.243759	0.3419415	0.7267444
ENSG00000163625	WDFY3	4	85739124	T	C	0.350895	-0.00443632	0.0147335	0.8	-0.100443	0.00838578	4.64E-33	0.0441674	0.146731	0.7634075	0.2408097

ENSG00000163626	COX18	4	73928634	G	A	0.120278	0.000502143	0.0210291	0.84	0.0663954	0.0118534	2.13E-08	0.00756292	0.316728	0.9809497	0.5121621
ENSG00000163629	PTPN13	4	87625896	C	T	0.214712	-0.00287582	0.0162234	0.9400001	-0.103578	0.00933404	1.30E-28	0.0277648	0.15665	0.859319	0.6150688
ENSG00000163631	ALB	4	74274980	G	A	0.149105	-0.00632224	0.0198496	0.7899998	-0.110243	0.0185395	2.74E-09	0.057348	0.180311	0.7504461	0.2874406
ENSG00000163633	C4orf36	4	87827356	G	A	0.260437	0.0012915	0.016264	0.99	-0.0748769	0.00999798	6.93E-14	-0.0172483	0.217222	0.9367113	0.5101593
ENSG00000163634	THOC7	3	63834562	T	C	0.175944	0.00350875	0.0189753	0.8600001	0.144551	0.0116679	3.01E-35	0.0242734	0.131285	0.8533147	0.8727425
ENSG00000163635	ATXN7	3	63919685	C	T	0.39662	-0.00368707	0.0147809	0.8600001	-0.0513812	0.00911628	1.74E-08	0.0717591	0.287953	0.8032027	0.8683662
ENSG00000163636	PSMD6	3	64002941	C	A	0.175944	-0.00273514	0.0193115	0.8800001	-0.0784601	0.0108851	5.68E-13	0.0348603	0.246179	0.8873917	0.9958975
ENSG00000163637	PRICKLE2	3	64255347	G	C	0.390656	-0.0137747	0.0145984	0.2999998	-0.070701	0.00812637	3.31E-18	0.19483	0.207692	0.3482058	0.4526245
ENSG00000163644	PPM1K	4	89194618	C	A	0.523857	0.00531239	0.0142238	0.9599999	0.161005	0.0117683	1.32E-42	0.0329953	0.088377	0.7088903	0.58635
ENSG00000163655	GMPS	3	155623391	T	C	0.0218688	0.0239486	0.0649261	0.7199992	0.368391	0.065613	1.97E-08	0.0650087	0.176622	0.7128241	0.9488409
ENSG00000163659	TIPARP	3	156407791	G	A	0.0298211	0.00823073	0.0414153	0.89	-0.248146	0.0198285	6.21E-36	-0.0331689	0.16692	0.8424885	0.355467
ENSG00000163660	CCNL1	3	156871423	T	A	0.256461	0.00250933	0.0164163	0.95	0.372386	0.00931725	0	0.00673853	0.0440845	0.8785127	0.8726392
ENSG00000163661	PTX3	3	157157997	G	A	0.27833	0.00647676	0.0160057	0.6300007	-0.0545617	0.00926721	3.92E-09	-0.118705	0.294043	0.6864326	0.8922823
ENSG00000163681	SLMAP	3	57828036	C	T	0.40159	0.0100014	0.0144885	0.6200004	0.107413	0.0080261	7.60E-41	0.0931114	0.135065	0.4905824	0.1648301
ENSG00000163682	RPL9	4	39458156	T	C	0.0357853	0.0217032	0.0322438	0.4	0.500909	0.0195966	4.14E-144	0.0433276	0.0643928	0.5010341	0.5505904
ENSG00000163683	SMIM14	4	39596625	A	G	0.498012	0.0203464	0.0142714	0.1199999	0.141168	0.00880649	7.89E-58	0.144129	0.101494	0.1555869	0.140793
ENSG00000163684	RPP14	3	58298895	G	A	0.383698	-0.0180265	0.0145989	0.2200002	0.111616	0.00838273	1.89E-40	-0.161505	0.131357	0.2188809	0.2202457
ENSG00000163686	ABHD6	3	58252326	C	T	0.251491	-0.010792	0.0168842	0.5	0.174668	0.00909832	3.86E-82	-0.0617859	0.0967183	0.5229378	0.4677154
ENSG00000163687	DNASE1L3	3	58189204	C	T	0.350895	0.0182915	0.014705	0.2	-0.359096	0.0120181	3.62E-196	-0.0509376	0.0409855	0.2139341	0.288416
ENSG00000163694	RBM47	4	40529082	A	G	0.367793	0.00362493	0.0145993	0.59	0.207278	0.00892532	2.63E-119	0.0174882	0.0704374	0.8039173	0.7103166
ENSG00000163697	APBB2	4	41015387	A	G	0.166998	0.00416754	0.0187048	0.6600001	0.375773	0.011916	2.87E-218	0.0110906	0.0497781	0.8236909	0.6085242
ENSG00000163701	IL17RE	3	9951191	T	C	0.0218688	-0.0421659	0.0463222	0.3100002	0.351947	0.0337994	2.17E-25	-0.119808	0.132119	0.3645042	0.5882936
ENSG00000163702	IL17RC	3	9967036	C	T	0.451292	0.0115239	0.014235	0.32	-0.187011	0.00792541	4.20E-123	-0.0616215	0.0761632	0.4184744	0.4568145
ENSG00000163703	CRELD1	3	9981301	T	C	0.431412	-0.0125134	0.0144804	0.2999998	-0.336496	0.0085348	0	0.0371874	0.0430433	0.3876133	0.3938498
ENSG00000163704	PRRT3	3	9990652	A	G	0.0258449	0.0793688	0.0490243	0.1	0.495172	0.0306396	9.47E-59	0.160285	0.0995	0.1072003	0.9530906
ENSG00000163710	PCOLCE2	3	142571404	G	T	0.387674	-0.000386415	0.0148411	0.8600001	0.066613	0.00837583	1.82E-15	-0.0058009	0.222797	0.9792281	0.4179685
ENSG00000163714	U2SURP	3	142749788	T	C	0.382704	-0.0119371	0.0146673	0.4199997	-0.0633063	0.0100457	2.94E-10	0.188561	0.233612	0.4195774	0.8647531
ENSG00000163728	TTC14	3	180327767	A	G	0.162028	-0.0195655	0.0203076	0.3400001	0.187114	0.0112653	5.92E-62	-0.104565	0.108713	0.3361298	0.5300534
ENSG00000163735	CXCL5	4	74862927	G	C	0.114314	-0.0178685	0.0220657	0.4600002	0.823623	0.0117386	0	-0.021695	0.0267928	0.418094	0.8622989
ENSG00000163737	PF4	4	74847317	A	C	0.0516899	-0.0153211	0.0346524	0.6700003	-0.1526	0.0216184	1.68E-12	0.1004	0.227525	0.6590161	0.8277553
ENSG00000163738	MTHFD2L	4	75074353	T	C	0.0357853	-0.0319796	0.0353985	0.28	0.267044	0.0195866	2.51E-42	-0.119754	0.132848	0.3673544	0.04171858
ENSG00000163739	CXCL1	4	74736034	G	A	0.181909	-0.0242011	0.0179974	0.14	-0.22799	0.00976095	1.16E-120	0.10615	0.07907	0.1794414	0.7009724
ENSG00000163743	RCHY1	4	76422110	C	T	0.218688	-0.0202646	0.0174261	0.17	0.488322	0.00919603	0	-0.0414984	0.0356942	0.2449881	0.4054022
ENSG00000163751	CPA3	3	148599013	C	T	0.389662	0.0100338	0.0145344	0.4799997	0.115863	0.00812536	3.92E-46	0.0866007	0.125592	0.490483	0.6898809
ENSG00000163754	GYG1	3	148727273	T	G	0.336978	-0.0130677	0.015275	0.5099998	-0.280361	0.0126852	3.06E-108	0.0466103	0.0545242	0.392631	0.02495264
ENSG00000163755	HPS3	3	148869445	T	C	0.0248509	0.0544453	0.0395737	0.08799946	0.533352	0.0223059	2.36E-126	0.102081	0.0743209	0.1695897	0.2612521
ENSG00000163781	TOPBP1	3	133348878	A	G	0.183897	-0.0143483	0.0174913	0.4	0.170541	0.0101679	3.89E-63	-0.0841342	0.102686	0.4125974	0.8749818
ENSG00000163785	RYK	3	133881856	A	G	0.310139	-0.002988	0.0151291	0.8200001	0.117166	0.00888212	9.86E-40	-0.0255023	0.12914	0.8434535	0.7524293
ENSG00000163788	SNRK	3	43397130	G	A	0.238569	-0.0195363	0.0161239	0.2300001	-0.13675	0.0087039	1.26E-55	0.142861	0.118258	0.2270283	0.9059795
ENSG00000163795	ZNF513	2	27601877	A	G	0.124254	-0.0459416	0.0225138	0.04900044	-0.11766	0.0117306	1.12E-23	0.390459	0.195265	0.04553975	0.01718375
ENSG00000163798	SLC4A1AP	2	27902089	A	G	0.464215	0.0199587	0.014337	0.1100001	-0.173994	0.0118391	6.78E-49	-0.114709	0.0827682	0.1657751	0.7207348
ENSG00000163803	PLB1	2	28773333	C	A	0.233598	0.00651754	0.0175087	0.7400005	0.47295	0.0100853	0	0.0137806	0.0370214	0.7097186	0.7252208
ENSG00000163807	KIAA1143	3	44791153	G	T	0.524851	-0.00816929	0.0141774	0.6999999	-0.284308	0.00897994	5.47E-220	0.028734	0.0498747	0.5645315	0.8334898
ENSG00000163811	WDR43	2	29144298	A	G	0.454274	-0.00782646	0.0142081	0.4700002	-0.0944854	0.00875172	3.59E-27	0.0828325	0.150569	0.5822307	0.2861429
ENSG00000163812	ZDHHC3	3	44987213	C	T	0.394632	0.00672958	0.0143674	0.5500004	0.0999302	0.00805603	2.47E-35	0.0673428	0.143877	0.6397426	0.583269
ENSG00000163814	CDCP1	3	45155842	C	T	0.410537	-0.0191375	0.0144784	0.1199999	0.142891	0.00805327	1.95E-70	-0.133931	0.101606	0.1874554	0.1680709
ENSG00000163815	CLEC3B	3	45060301	T	C	0.382704	0.00587201	0.014555	0.5700002	0.411434	0.00893168	0	0.0142721	0.0353776	0.6866389	0.4193544
ENSG00000163817	SLC6A20	3	45817484	G	A	0.436382	0.0112019	0.0142258	0.2999998	-0.0567964	0.00825529	5.99E-12	-0.197229	0.252105	0.4340218	0.4652928
ENSG00000163818	LZTFL1	3	45911171	A	G	0.0755467	0.0174913	0.0259903	0.6100002	0.297852	0.0153494	7.03E-84	0.0587249	0.0873117	0.5012088	0.9565662
ENSG00000163820	FYCO1	3	45998356	T	C	0.0208748	-0.00226926	0.0514202	0.8499999	-0.454822	0.0285309	3.27E-57	0.00498933	0.113056	0.9647996	0.9641804

ENSG00000163823	CCR1	3	46246543	C	T	0.255467	-0.00320215	0.0157681	0.9	0.581428	0.00829065	0	-0.00550739	0.0271197	0.8390749	0.5318313
ENSG00000163827	LRRC2	3	46589251	G	A	0.335984	-0.00431222	0.0154972	0.5999997	-0.0765062	0.00857117	4.42E-19	0.0563643	0.20266	0.780918	0.8128004
ENSG00000163832	ELP6	3	47546190	T	C	0.156064	0.00977322	0.0192446	0.5300002	0.146883	0.0135519	2.26E-27	0.0665373	0.131163	0.6119544	0.2799023
ENSG00000163833	FBXO40	3	121330552	C	T	0.247515	-0.00542579	0.0166385	0.7700005	0.125102	0.00936284	1.01E-40	-0.0433708	0.133039	0.7444233	0.4211477
ENSG00000163840	DTX3L	3	122288567	C	T	0.209742	0.00956901	0.0165245	0.5700002	-0.207315	0.00941162	1.57E-107	-0.0461568	0.0797346	0.56267	0.6873401
ENSG00000163848	ZNF148	3	125019301	A	G	0.246521	-0.00705666	0.0164547	0.6700003	0.152645	0.0098316	2.31E-54	-0.0462291	0.107838	0.6681487	0.9061818
ENSG00000163864	NMNAT3	3	139337940	A	G	0.447316	0.00689478	0.0143305	0.5500004	-0.391063	0.00769574	0	-0.0176309	0.0366467	0.6304421	0.4617759
ENSG00000163866	SMIM12	1	35251877	A	G	0.34493	-0.00616655	0.0146981	0.7800007	0.375706	0.00892667	0	-0.0164132	0.0391233	0.6748313	0.8889698
ENSG00000163867	ZMYM6	1	35472351	T	C	0.0308151	-0.0962047	0.0413208	0.005399953	-0.276355	0.0257185	6.23E-27	0.34812	0.15299	0.02287962	0.3534193
ENSG00000163870	TPRA1	3	127304503	A	G	0.082505	0.00587243	0.0229643	0.7899998	-0.443132	0.0122463	1.05E-286	-0.0132521	0.051824	0.798172	0.4508028
ENSG00000163872	YEATS2	3	183473009	A	G	0.172962	0.0238273	0.0188019	0.1900002	0.202928	0.0106353	3.66E-81	0.117417	0.092857	0.2060522	0.8513918
ENSG00000163873	GRIK3	1	37380429	G	A	0.331014	-0.0144773	0.015056	0.3900004	-0.0746837	0.00847177	1.19E-18	0.193848	0.202792	0.3391258	0.9902931
ENSG00000163874	ZC3H12A	1	37945066	C	G	0.132207	0.0375449	0.0215196	0.05499966	0.151577	0.0117829	7.16E-38	0.247695	0.143271	0.0838348	0.02564693
ENSG00000163875	MEAF6	1	37969275	A	G	0.112326	-0.00135843	0.0196151	0.99	-0.591104	0.0107838	0	0.00229812	0.0331839	0.9447872	0.1984667
ENSG00000163877	SNIP1	1	38011022	C	G	0.054672	-0.00342656	0.0256717	0.9299999	-0.465021	0.0148796	2.08E-214	0.00736861	0.055206	0.8938178	0.245344
ENSG00000163882	POLR2H	3	184082945	T	C	0.2833	0.0174407	0.0157815	0.2399999	-0.0899679	0.00935937	7.07E-22	-0.193855	0.176568	0.2722465	0.2226047
ENSG00000163885	CFAP100	3	126134590	A	G	0.479125	0.0150145	0.0143318	0.3900004	-0.0631659	0.00877157	5.97E-13	-0.2377	0.22928	0.2998655	0.8232332
ENSG00000163898	LIPH	3	185247225	C	T	0.026839	0.0865205	0.0388036	0.02399993	-0.268733	0.0260002	4.85E-25	-0.321957	0.147716	0.02928983	0.3724853
ENSG00000163900	TMEM41A	3	185205564	A	G	0.488072	0.00635146	0.0142121	0.4799997	-0.0532869	0.00798689	2.53E-11	-0.119194	0.267307	0.6556652	0.4271595
ENSG00000163902	RPN1	3	128369367	A	G	0.251491	-0.0397469	0.0160165	0.01499996	0.245979	0.00971067	1.46E-141	-0.161587	0.065425	0.01351894	0.01230942
ENSG00000163904	SENP2	3	185325811	G	A	0.323062	0.0241952	0.0150014	0.17	0.0696644	0.00836789	8.42E-17	0.347311	0.219342	0.1133254	0.1725992
ENSG00000163913	IFT122	3	129199083	A	G	0.156064	0.00338758	0.0197399	0.9599999	-0.108535	0.0110529	9.27E-23	-0.0312118	0.181903	0.8637641	0.7603495
ENSG00000163918	RFC4	3	186516258	T	C	0.276342	-0.0160579	0.0165887	0.2999998	-0.440708	0.00941685	0	0.0364366	0.0376491	0.3331466	0.7731417
ENSG00000163923	RPL39L	3	186868716	G	A	0.246521	-0.00147384	0.0171744	0.95	0.188451	0.00929843	2.52E-91	-0.00782081	0.0911353	0.9316132	0.4040928
ENSG00000163930	BAP1	3	52439697	G	A	0.12326	0.0093029	0.0229432	0.6800001	-0.0943361	0.0143311	4.62E-11	-0.0986144	0.243668	0.685692	0.6327302
ENSG00000163931	TKT	3	53274395	T	C	0.17495	0.0152098	0.018295	0.32	-0.817231	0.0136859	0	-0.0186114	0.0223887	0.4058137	0.2312234
ENSG00000163932	PRKCD	3	53208379	A	G	0.286282	0.022869	0.0161753	0.16	0.203815	0.00885706	3.57E-117	0.112205	0.0795122	0.158197	0.3137631
ENSG00000163933	RFT1	3	53143488	C	T	0.403579	-0.0119707	0.0142752	0.5099998	0.298212	0.00860861	6.08E-263	-0.0401416	0.0478834	0.4018507	0.4492453
ENSG00000163935	SFMBT1	3	53009177	A	C	0.0924453	0.0266503	0.0232031	0.2099999	0.183027	0.013258	2.38E-43	0.145609	0.127212	0.2523701	0.6059874
ENSG00000163938	GNL3	3	52721840	A	G	0.521869	0.00307335	0.0141923	0.99	-0.137836	0.00790832	4.95E-68	-0.0222972	0.102973	0.8285717	0.7090366
ENSG00000163939	PBRM1	3	52649650	C	T	0.210736	-0.0113863	0.0178841	0.28	0.07527	0.0105349	9.01E-13	-0.151273	0.238541	0.5259771	0.3587794
ENSG00000163945	UVSSA	4	1361445	T	C	0.0437376	-0.00237486	0.0331114	0.8700001	1.13853	0.0214664	0	-0.0020859	0.0290826	0.9428221	0.3184875
ENSG00000163946	TASOR	3	56685713	T	C	0.110338	0.0201488	0.0240402	0.33	-0.194022	0.0151183	1.06E-37	-0.103848	0.124168	0.4029593	0.4207642
ENSG00000163947	ARHGEF3	3	56937401	A	G	0.239563	0.0023467	0.0161891	0.5400003	-0.264475	0.00907824	1.38E-186	-0.00887306	0.0612131	0.8847474	0.2828609
ENSG00000163950	SLBP	4	1704404	G	A	0.441352	-0.0105068	0.0142091	0.56	-0.224108	0.00853107	4.27E-152	0.0468828	0.0634281	0.4598166	0.3037999
ENSG00000163956	LRPAP1	4	3521194	A	G	0.328032	0.020188	0.0152567	0.1199999	0.262409	0.00975588	2.35E-159	0.0769334	0.0582113	0.1862935	0.320736
ENSG00000163958	ZDHHC19	3	195931314	A	G	0.39165	0.00100484	0.0144421	0.6899999	-0.208441	0.00877389	9.30E-125	-0.00482073	0.0692864	0.9445305	0.3349871
ENSG00000163959	SLC51A	3	195954203	T	C	0.204771	0.0269736	0.0172295	0.1	-0.183932	0.0107052	3.65E-66	-0.14665	0.0940612	0.1189754	0.281916
ENSG00000163960	UBXN7	3	196116939	T	A	0.363817	0.0207122	0.0147864	0.2599998	0.0882516	0.00911032	3.42E-22	0.234695	0.169291	0.1656428	0.4161834
ENSG00000163961	RNF168	3	196213146	G	T	0.281312	0.00282666	0.0156177	0.95	0.161153	0.00970756	6.88E-62	0.0175402	0.0969178	0.8563832	0.8709031
ENSG00000163964	PIGX	3	196414762	T	C	0.333996	-0.0190902	0.0152862	0.2599998	0.223524	0.00914656	6.75E-132	-0.0854057	0.0684766	0.2123152	0.9842623
ENSG00000163975	MELTF	3	196736089	G	A	0.163022	0.0214774	0.0187829	0.4199997	-0.136088	0.0113628	4.71E-33	-0.157821	0.138648	0.2550041	0.09982681
ENSG00000163993	S100P	4	6696846	T	C	0.248509	0.0304835	0.0168459	0.06699926	-1.1049	0.00710721	0	-0.0275894	0.0152476	0.07038461	0.3431173
ENSG00000163995	ABLIM2	4	8063799	G	A	0.180915	-0.00783811	0.0191781	0.6300007	-0.118148	0.0122233	4.21E-22	0.0663415	0.162468	0.6830269	0.9050762
ENSG00000164002	EXO5	1	40978320	T	C	0.191849	0.0149061	0.0188162	0.4700002	0.223068	0.0110086	2.73E-91	0.0668231	0.0844163	0.4285996	0.321622
ENSG00000164008	C1orf50	1	43248454	A	C	0.446322	0.0200753	0.0143644	0.1800002	-0.060472	0.00802506	4.87E-14	-0.331977	0.241589	0.1693985	0.02055602
ENSG00000164010	ERMAP	1	43296727	G	A	0.16501	-0.00275647	0.018304	0.9599999	-0.627912	0.0100466	0	0.0043899	0.0291507	0.8802965	0.1286983
ENSG00000164011	ZNF691	1	43315214	A	G	0.144135	0.00736285	0.0203476	0.6499995	-0.0763064	0.0115402	3.79E-11	-0.0964906	0.267056	0.7178656	0.4961632
ENSG00000164022	AIMP1	4	107253542	T	C	0.217694	-0.0149684	0.0175488	0.4600002	-0.117619	0.00962036	2.26E-34	0.127262	0.149564	0.3948303	0.9427697
ENSG00000164023	SGMS2	4	108790961	C	T	0.0149105	-0.018493	0.0432919	0.9299999	-0.223808	0.0356451	3.41E-10	0.0826287	0.19388	0.6699732	0.4816056

ENSG00000164024	METAP1	4	99950367	A	G	0.157058	-0.0491222	0.0194068	0.002399993	0.0988368	0.010449	3.11E-21	-0.497003	0.203261	0.01447907	0.1031592
ENSG00000164031	DNAJB14	4	100842644	G	C	0.11829	-0.0122397	0.0250151	0.35	0.0990457	0.0144901	8.18E-12	-0.123576	0.253207	0.6255189	0.8650932
ENSG00000164032	H2AZ1	4	100870394	G	T	0.258449	-0.000431373	0.0172216	0.7099994	-0.354898	0.0155174	9.03E-116	0.00121548	0.0485255	0.9800164	0.9137772
ENSG00000164038	SLC9B2	4	103974005	T	C	0.407555	0.0104687	0.0146034	0.58	-0.0761826	0.00906547	4.33E-17	-0.137416	0.192386	0.4750583	0.7567081
ENSG00000164039	BDH2	4	104010816	T	C	0.407555	0.0104687	0.0146034	0.58	0.52467	0.0112551	0	0.0199529	0.0278368	0.4735083	0.790079
ENSG00000164045	CDC25A	3	48214264	G	A	0.291252	0.0123726	0.0151842	0.25	0.0911894	0.00892176	1.60E-24	0.13568	0.167041	0.4166443	0.7143958
ENSG00000164048	ZNF589	3	48311666	G	A	0.327038	0.00781364	0.0147739	0.4899999	0.810487	0.00755452	0	0.00964068	0.0182287	0.5968922	0.651381
ENSG00000164050	PLXNB1	3	48458427	T	C	0.284294	0.010101	0.0152721	0.32	-0.0731387	0.00919359	1.79E-15	-0.138107	0.20953	0.5098135	0.08798125
ENSG00000164053	ATRIP	3	48497614	C	T	0.173956	0.0125887	0.0176183	0.33	-0.335139	0.0118796	4.24E-175	-0.0375626	0.052587	0.4750448	0.1545529
ENSG00000164054	SHISA5	3	48525728	T	A	0.494036	-0.0136898	0.0142285	0.3400001	-0.158849	0.00799716	8.47E-88	0.0861811	0.0896774	0.3365458	0.3433569
ENSG00000164056	SPRY1	4	124321430	C	G	0.267396	0.00121699	0.0165217	0.9599999	-0.0787832	0.00900914	2.23E-18	-0.0154473	0.209718	0.9412829	0.9659907
ENSG00000164061	BSN	3	49650450	A	C	0.170974	-0.0230996	0.0186429	0.1800002	-0.138905	0.0176783	3.92E-15	0.166297	0.135872	0.220978	0.5585489
ENSG00000164062	APEH	3	49716415	G	A	0.324056	-0.0115258	0.0155824	0.4500005	0.305245	0.0094074	5.91E-231	-0.0377592	0.0510621	0.4596182	0.05188045
ENSG00000164068	RNF123	3	49742947	T	C	0.16004	0.0634121	0.018722	0.00129999	0.104964	0.0107281	1.32E-22	0.604134	0.188752	0.001371059	0.4486947
ENSG00000164073	MFSD8	4	128863055	G	A	0.028827	0.0089727	0.0463939	0.9	0.180162	0.0273023	4.15E-11	0.0498034	0.257622	0.8467087	0.6747058
ENSG00000164074	ABHD18	4	128923663	A	G	0.0228628	0.0398508	0.0597889	0.5	-0.399649	0.0311783	1.30E-37	-0.0997145	0.149806	0.5056503	0.9444281
ENSG00000164077	MON1A	3	49956954	A	T	0.0745527	-0.0170667	0.0255263	0.6300007	-0.313121	0.0156923	1.39E-88	0.0545051	0.0815678	0.5039946	0.3028229
ENSG00000164080	RAD54L2	3	51636385	G	T	0.373757	-0.00913617	0.0146146	0.7899998	0.0569285	0.00903083	2.90E-10	-0.160485	0.257978	0.5338837	0.7140576
ENSG00000164081	TEX264	3	51717524	A	G	0.109344	0.0176164	0.0219931	0.3100002	0.317052	0.0222633	5.10E-46	0.0555632	0.0694772	0.4238657	0.4487355
ENSG00000164082	GRM2	3	51746857	T	C	0.119284	-0.00134807	0.0206138	0.7700005	0.34856	0.0117448	1.48E-193	-0.00386754	0.05914	0.9478585	0.07048774
ENSG00000164087	POC1A	3	52148987	G	A	0.0944334	-0.00843097	0.0272908	0.9199999	0.325535	0.0152842	1.16E-100	-0.0258988	0.0838425	0.7573988	0.7668924
ENSG00000164088	PPM1M	3	52282227	G	C	0.517893	0.00392414	0.0141807	0.9199999	-0.374597	0.00767561	0	-0.0104756	0.0378565	0.7819955	0.2888838
ENSG00000164091	WDR82	3	52305236	A	T	0.473161	3.67E-05	0.0142251	0.8600001	0.112235	0.00916896	1.88E-34	0.000327333	0.126743	0.9979393	0.1902651
ENSG00000164096	C4orf3	4	120221903	T	C	0.422465	0.0149664	0.0142844	0.2700001	0.303114	0.00860644	1.01E-271	0.0493754	0.0471463	0.29497	0.9178771
ENSG00000164104	HMGB2	4	174254561	T	A	0.472167	-0.00548881	0.0142864	0.6100002	-0.0976087	0.00795896	1.41E-34	0.0562328	0.146436	0.7009711	0.859321
ENSG00000164105	SAP30	4	174294901	G	T	0.474155	-0.00492984	0.0142723	0.6300007	-0.114154	0.00793207	5.86E-47	0.043186	0.125063	0.7298586	0.6819978
ENSG00000164106	SCRG1	4	174318415	T	A	0.437376	-0.0152182	0.0143723	0.2	-0.0848201	0.00825355	8.97E-25	0.179417	0.170342	0.2922126	0.3810342
ENSG00000164109	MAD2L1	4	120984403	A	C	0.00795229	-0.0329913	0.0577767	0.6200004	1.2395	0.0894867	1.25E-43	-0.0266167	0.0466526	0.5683187	0.162092
ENSG00000164111	ANXA5	4	122603689	A	C	0.180915	0.000412547	0.0176377	0.8	0.81509	0.00908192	0	0.000506137	0.021639	0.9813391	0.1784135
ENSG00000164112	SMIM43	4	122683335	T	C	0.17495	0.0221344	0.0197651	0.32	-0.0827649	0.0113647	3.27E-13	-0.267437	0.241617	0.2683533	0.08662924
ENSG00000164114	MAP9	4	156280966	A	G	0.43837	0.0175415	0.0142435	0.17	-0.0771616	0.00809552	1.55E-21	-0.227334	0.186128	0.2219381	0.9516238
ENSG00000164116	GUCY1A1	4	156620682	C	T	0.0129225	-0.0279935	0.0535112	0.64	-0.499624	0.0394	7.56E-37	0.0560292	0.107194	0.6011908	0.2996257
ENSG00000164117	FBXO8	4	175181612	G	A	0.141153	0.013476	0.0194921	0.4700002	0.062548	0.0108851	9.13E-09	0.215451	0.313882	0.4924567	0.3975871
ENSG00000164118	CEP44	4	175229679	C	T	0.472167	0.00617019	0.0142275	0.6800001	0.126774	0.00961855	1.14E-39	0.0486706	0.112288	0.6646908	0.2096792
ENSG00000164120	HPGD	4	175427816	C	G	0.331014	-0.0126854	0.0153284	0.3800004	-0.387053	0.00831181	0	0.0327744	0.0396091	0.4079853	0.7710159
ENSG00000164124	TMEM144	4	159149659	A	G	0.148111	-0.0188881	0.0198856	0.29	-0.740938	0.0102016	0	0.0254922	0.0268407	0.3422358	0.458569
ENSG00000164125	GASK1B	4	159070048	A	C	0.156064	-0.008974	0.019583	0.6300007	0.8493	0.00983383	0	-0.0105664	0.0230581	0.6467742	0.4987359
ENSG00000164134	NAA15	4	140281898	C	T	0.195825	-0.0158092	0.0191582	0.29	0.11335	0.0101198	4.04E-29	-0.139473	0.169477	0.4105296	0.8937699
ENSG00000164136	IL15	4	142606446	A	C	0.38171	0.00283316	0.0144814	0.8600001	-0.175383	0.00812453	2.39E-103	-0.0161542	0.0825737	0.8448972	0.8197379
ENSG00000164142	FHIP1A	4	152457576	C	T	0.468191	-0.0143098	0.0142668	0.2099999	0.203931	0.00974273	2.76E-97	-0.0701699	0.0700393	0.3164091	0.08038299
ENSG00000164144	ARFIP1	4	153770352	T	C	0.410537	-0.00577133	0.0144634	0.91	0.713995	0.00700075	0	-0.00808315	0.0202572	0.6898734	0.05331971
ENSG00000164151	ICE1	5	5455562	C	G	0.331014	-0.0111018	0.014996	0.5099998	-0.0507508	0.00925333	4.14E-08	0.218751	0.298163	0.4631536	0.9159393
ENSG00000164164	OTUD4	4	146066651	A	G	0.520875	0.0170076	0.014199	0.14	-0.0438663	0.00797627	3.81E-08	-0.387714	0.331276	0.2418539	0.2249882
ENSG00000164167	LSM6	4	147108994	C	T	0.306163	0.00495002	0.0158202	0.5099998	-0.190566	0.00908939	1.35E-97	-0.0259753	0.083026	0.7543889	0.2467414
ENSG00000164171	ITGA2	5	52337882	G	A	0.44334	-0.020164	0.014436	0.14	-0.292475	0.00782543	9.99E-306	0.0689427	0.0493926	0.1627713	0.703864
ENSG00000164172	MOCS2	5	52398701	A	G	0.267396	-0.0189053	0.0159445	0.2	0.166387	0.0088971	4.84E-78	-0.113623	0.0960205	0.2366831	0.6217147
ENSG00000164175	SLC45A2	5	33964778	A	C	0.138171	0.0446344	0.0202659	0.007799917	0.159909	0.0125487	3.41E-37	0.279125	0.128613	0.029987	0.8304241
ENSG00000164181	ELOVL7	5	60093917	C	T	0.187873	-0.00354658	0.0174023	0.95	-0.265613	0.0103871	3.17E-144	0.0133524	0.0655195	0.8385152	0.7668556
ENSG00000164182	NDUFAF2	5	60344904	T	C	0.382704	0.00938573	0.0148387	0.5	0.138593	0.00881704	1.12E-55	0.0677214	0.107153	0.5273833	0.06624482
ENSG00000164187	LMBRD2	5	36125288	A	G	0.525845	0.0176546	0.0142039	0.16	-0.234676	0.0086254	5.28E-163	-0.0752295	0.0605886	0.2143674	0.4762949

ENSG00000164209	SLC25A46	5	110087347	A	C	0.0914513	0.00633645	0.0249981	0.84	-0.340887	0.0138617	1.54E-133	-0.0185881	0.0733365	0.7999098	0.004726386
ENSG00000164211	STARD4	5	110840009	C	T	0.146123	0.0152134	0.0203427	0.4299995	-0.254477	0.0110207	5.73E-118	-0.0597831	0.0799812	0.4547838	0.1352128
ENSG00000164219	PGGT1B	5	114572548	C	T	0.264414	-0.00508758	0.0165445	0.8200001	-0.281062	0.0102833	1.77E-164	0.0181013	0.0588681	0.7584709	0.5636627
ENSG00000164221	CCDC112	5	114617706	C	T	0.11332	-0.0172305	0.021494	0.5500004	0.190383	0.0172111	1.93E-28	-0.0905046	0.113195	0.4239742	0.8453264
ENSG00000164236	ANKRD33B	5	10607375	G	A	0.237575	0.00795124	0.0165933	0.9199999	0.146574	0.0200397	2.59E-13	0.0542472	0.11345	0.6325372	0.9658672
ENSG00000164237	CMBL	5	10292062	C	T	0.214712	-0.00625041	0.0172267	0.7800007	0.697346	0.00850796	0	-0.00896314	0.0247035	0.7167325	0.4492879
ENSG00000164241	C5orf63	5	126393717	T	C	0.49503	0.0241239	0.0142496	0.07499978	-0.0877185	0.00895965	1.24E-22	-0.275015	0.164858	0.09527684	0.2507378
ENSG00000164244	PRRC1	5	126872041	C	A	0.293241	0.00553915	0.0154239	0.6499995	0.184884	0.00867196	7.44E-101	0.0299601	0.0834364	0.7195377	0.4550312
ENSG00000164251	F2RL1	5	76122949	G	T	0.486083	-0.0134802	0.0141992	0.35	0.782635	0.00994051	0	-0.0172241	0.0181441	0.3424709	0.6974155
ENSG00000164252	AGGF1	5	76343067	G	A	0.367793	0.00221831	0.0145862	0.7499995	0.0785567	0.00816225	6.31E-22	0.0282383	0.1857	0.8791366	0.5022282
ENSG00000164253	WDR41	5	76819115	T	C	0.151093	-0.0171936	0.0190644	0.4299995	-0.769924	0.0108859	0	0.0223316	0.0247634	0.3671652	0.5784324
ENSG00000164258	NDUFS4	5	52917815	A	T	0.162028	0.0213506	0.0187557	0.1299999	0.21331	0.0160436	2.46E-40	0.100092	0.0882487	0.2567094	0.2482175
ENSG00000164265	SCGB3A2	5	147255999	G	A	0.469185	0.0278125	0.0142454	0.07499978	-0.593142	0.00737042	0	-0.0468902	0.0240239	0.05096073	0.6237576
ENSG00000164283	ESM1	5	54296095	C	T	0.455268	0.00849292	0.0143179	0.59	-0.176733	0.00798294	1.34E-108	-0.0480552	0.0810435	0.5532111	0.8460441
ENSG00000164284	GRPEL2	5	148729569	G	T	0.402584	-0.0187826	0.0141807	0.32	-0.111575	0.00795871	1.19E-44	0.168341	0.127662	0.1872874	0.9348224
ENSG00000164291	ARSK	5	94915773	T	C	0.119284	-0.0150395	0.0232341	0.28	0.0712491	0.0127861	2.51E-08	-0.211083	0.328289	0.5202375	0.8485945
ENSG00000164292	RHOBTB3	5	95104656	A	C	0.0357853	-0.0166335	0.0352803	0.7199992	-0.233645	0.0241948	4.60E-22	0.0711914	0.15118	0.6377077	0.9776577
ENSG00000164296	TIGD6	5	149376705	T	C	0.16998	0.0492877	0.0197171	0.006800017	-0.146765	0.0124209	3.23E-32	-0.335826	0.137318	0.01446053	0.1423456
ENSG00000164300	SERINC5	5	79479474	G	A	0.101392	0.011809	0.0260872	0.6499995	-0.322908	0.0135077	2.69E-126	-0.0365708	0.0808028	0.6508419	0.6784851
ENSG00000164305	CASP3	4	185559756	A	C	0.115308	0.0125207	0.0219764	0.6499995	-0.489507	0.0118499	0	-0.0255782	0.0448992	0.5688949	0.9864456
ENSG00000164306	PRIMPOL	4	185593442	A	G	0.161034	0.0187033	0.0196432	0.5	0.260376	0.0191273	3.36E-42	0.0718319	0.075626	0.3421983	0.4177229
ENSG00000164307	ERAP1	5	96120162	A	C	0.230616	0.00905665	0.01694	0.4199997	0.277865	0.0103283	2.01E-159	0.0325937	0.0609768	0.592978	0.7167381
ENSG00000164308	ERAP2	5	96233531	C	T	0.426441	-0.00389284	0.014263	0.83	1.0119	0.00618304	0	-0.00384707	0.0140953	0.7849052	0.1213756
ENSG00000164309	CMYA5	5	79040881	C	A	0.0964215	0.00106716	0.024374	0.9400001	0.0924111	0.0137141	1.60E-11	0.011548	0.263762	0.9650783	0.9089535
ENSG00000164323	CFAP97	4	186105738	A	G	0.355865	-0.0316895	0.0153341	0.01899984	0.401221	0.00893794	0	-0.0789826	0.038259	0.03897823	0.6526977
ENSG00000164327	RICTOR	5	39006265	A	T	0.166998	-0.0254512	0.0188841	0.17	0.102107	0.0105402	3.41E-22	-0.24926	0.186725	0.1819087	0.6886291
ENSG00000164329	TENT2	5	78945207	C	A	0.186879	0.00114016	0.0192685	0.8	-0.0966137	0.0105476	5.20E-20	-0.0118012	0.199443	0.9528159	0.9645213
ENSG00000164330	EBF1	5	158324848	G	A	0.0377734	0.0181373	0.0364029	0.5999997	-0.122949	0.0215905	1.24E-08	-0.147519	0.297212	0.6196534	0.686675
ENSG00000164331	ANKRA2	5	72854835	A	G	0.218688	-0.0218034	0.0169049	0.1800002	-0.356011	0.00930381	0	0.0612436	0.0475111	0.1973856	0.0475923
ENSG00000164332	UBLCP1	5	158701566	G	A	0.124254	0.0184255	0.0229739	0.4400003	0.334682	0.0197013	1.01E-64	0.0550537	0.0687203	0.4230584	0.8090367
ENSG00000164338	UTP15	5	72869531	C	T	0.321074	0.00865457	0.0149698	0.5700002	0.159905	0.0091676	3.93E-68	0.0541232	0.0936683	0.563387	0.007542247
ENSG00000164342	TLR3	4	186998280	C	G	0.370775	-0.00897116	0.0147496	0.4100001	-0.142497	0.00836424	4.41E-65	0.0629571	0.103574	0.5432913	0.4678617
ENSG00000164344	KLKB1	4	187154879	T	C	0.17992	0.0393324	0.0188536	0.02399993	0.662081	0.010161	0	0.0594072	0.0284908	0.03705727	0.01687006
ENSG00000164346	NSA2	5	74067777	T	C	0.136183	0.0108845	0.0214204	0.5700002	0.986903	0.0210926	0	0.0110289	0.0217059	0.6113782	0.3542053
ENSG00000164347	GFM2	5	74040112	T	G	0.250497	0.0236027	0.016714	0.1800002	-0.215057	0.00914049	2.11E-122	-0.109751	0.0778587	0.1586535	0.3993728
ENSG00000164366	CCDC127	5	211601	G	A	0.508946	-0.019831	0.014215	0.1100001	-0.433435	0.00856003	0	0.0457531	0.0328086	0.1631527	0.9880332
ENSG00000164379	FOXQ1	6	1313833	C	T	0.204771	0.00420589	0.0168297	0.5	-0.0784924	0.00974722	8.09E-16	-0.0535834	0.214515	0.8027509	0.7938313
ENSG00000164398	ACSL6	5	131245309	C	T	0.233598	0.00191962	0.0179384	0.9599999	0.175383	0.00990296	3.50E-70	0.0109453	0.102283	0.9147812	0.6257324
ENSG00000164402	SEPTIN8	5	132114721	T	C	0.142147	-0.0114908	0.0224672	0.6100002	-0.170017	0.0131139	1.94E-38	0.0675863	0.13225	0.6093158	0.7500027
ENSG00000164403	SHROOM1	5	132162211	T	C	0.146123	-0.0258906	0.0195189	0.1	-0.482437	0.0119847	0	0.0536663	0.0404809	0.1849332	0.08259971
ENSG00000164404	GDF9	5	132199724	G	A	0.40159	0.0119583	0.0143932	0.4299995	-0.0826935	0.00806766	1.18E-24	-0.14461	0.174626	0.407606	0.2331407
ENSG00000164405	UQCRQ	5	132202987	T	C	0.00298211	-0.0145674	0.192635	0.7199992	-0.841902	0.0918866	5.08E-20	0.017303	0.228817	0.9397221	0.4123304
ENSG00000164406	LEAP2	5	132209376	G	A	0.321074	0.00363089	0.0146797	0.7700005	0.10294	0.00829066	2.13E-35	0.0352719	0.142633	0.8046824	0.3243009
ENSG00000164414	SLC35A1	6	88201197	T	C	0.459245	-0.00618762	0.0142043	0.4899999	-0.182308	0.00829995	6.23E-107	0.0339404	0.0779289	0.6631775	0.7997543
ENSG00000164430	CGAS	6	74142618	A	G	0.280318	0.0153395	0.0164327	0.32	0.238942	0.0102795	1.62E-119	0.0641976	0.0688282	0.3509634	0.1788108
ENSG00000164440	TXLNB	6	139587237	G	A	0.230616	-0.0180606	0.017233	0.32	0.100637	0.0100442	1.25E-23	-0.179463	0.172173	0.2972552	0.1272367
ENSG00000164442	CITED2	6	139694575	C	T	0.432406	0.0456505	0.0143798	0.002699977	-0.129313	0.00807801	1.12E-57	-0.353024	0.113367	0.001845803	0.04741213
ENSG00000164458	TBXT	6	166576666	C	T	0.333002	0.00729214	0.0150375	0.4600002	0.069142	0.00878373	3.50E-15	0.105466	0.217899	0.6283768	0.4689539
ENSG00000164463	CREBRF	5	172524823	T	G	0.507952	-0.00895368	0.0142507	0.7199992	-0.348887	0.00939922	1.40E-301	0.0256636	0.0408521	0.529868	0.9922383
ENSG00000164465	DCBLD1	6	117833000	C	A	0.292247	-0.00139192	0.0156999	0.8800001	-0.413829	0.00842462	0	0.00336351	0.0379382	0.9293539	0.2483011

ENSG00000164466	SFXN1	5	174930405	A	G	0.136183	-0.0241142	0.0208352	0.1499999	-0.367787	0.011029	8.00E-244	0.0655656	0.0566842	0.2474025	0.5294679
ENSG00000164483	SAMD3	6	130576015	A	C	0.502982	0.00434315	0.0144953	0.7800007	0.473153	0.00753223	0	0.00917916	0.0306359	0.764466	0.6214786
ENSG00000164484	TMEM200A	6	130725543	T	G	0.0745527	-0.0237044	0.0282249	0.3700002	0.468319	0.0155824	1.92E-198	-0.0506159	0.0602921	0.4011819	0.1037708
ENSG00000164494	PDSS2	6	107627264	A	G	0.346918	-0.00600094	0.0148566	0.6899999	0.236125	0.00836278	2.16E-175	-0.0254143	0.0629249	0.6862989	0.4236182
ENSG00000164506	STXBP5	6	147616213	A	T	0.510934	0.00548247	0.0142138	0.83	-0.19134	0.00788687	5.11E-130	-0.028653	0.0742949	0.6997442	0.531775
ENSG00000164509	IL31RA	5	55182942	C	T	0.00894632	0.201481	0.076415	0.004900044	0.840174	0.0485807	5.18E-67	0.239809	0.0920023	0.009145956	0.5087883
ENSG00000164512	ANKRD55	5	55462346	A	G	0.377734	-0.00218292	0.014644	0.9	-0.598063	0.00810025	0	0.00364998	0.0244858	0.8815019	0.9973704
ENSG00000164530	PI16	6	36927411	A	G	0.145129	0.00442884	0.0196785	0.83	0.853352	0.00958059	0	0.00518994	0.0230603	0.8219333	0.2458194
ENSG00000164535	DAGLB	7	6486289	T	C	0.238569	-0.000318502	0.0159646	0.59	0.625471	0.00872303	0	-0.000509219	0.0255241	0.9840828	0.3369099
ENSG00000164542	MATCAP2	7	36396782	T	G	0.0129225	-0.0121179	0.055158	0.9	0.362239	0.0444956	3.92E-16	-0.0334528	0.152325	0.8261715	0.2808273
ENSG00000164543	STK17A	7	43644371	G	A	0.466203	-0.0210708	0.0142586	0.08199927	-0.0833902	0.00876315	1.80E-21	0.252677	0.173036	0.1442191	0.6477921
ENSG00000164548	TRA2A	7	23558029	C	G	0.117296	0.000573045	0.0199881	0.83	-0.121742	0.0210117	6.87E-09	-0.00470704	0.164186	0.9771286	0.3823285
ENSG00000164556	FAM183BP	7	38725791	T	C	0.497018	0.0150426	0.0141913	0.4	-0.259375	0.0097625	1.57E-155	-0.0579956	0.054757	0.2895338	0.1060982
ENSG00000164574	GALNT10	5	153685417	A	C	0.0487078	0.0230889	0.0309469	0.3599996	-0.409133	0.0174552	1.71E-121	-0.0564337	0.0756785	0.4558465	0.7273925
ENSG00000164576	SAP30L	5	153830704	C	A	0.482107	0.0126321	0.0142319	0.3100002	-0.137002	0.00793871	9.84E-67	-0.0922041	0.104019	0.3753918	0.5420201
ENSG00000164587	RPS14	5	149826036	C	T	0.256461	-0.0304075	0.0160428	0.0659994	-0.226874	0.00872345	4.08E-149	0.134028	0.0708999	0.0587065	0.7594841
ENSG00000164597	COG5	7	107023574	G	T	0.266402	-0.022863	0.0168472	0.1900002	0.933362	0.00793951	0	-0.0244953	0.0180512	0.1747845	0.7728148
ENSG00000164603	BMT2	7	112519586	C	T	0.0497018	-0.0744368	0.034335	0.02800013	-0.203417	0.0192138	3.42E-26	0.365932	0.172294	0.03367986	0.9134247
ENSG00000164604	GPR85	7	112724150	A	G	0.413519	0.0103587	0.0144472	0.4799997	-0.08732	0.00844798	4.83E-25	-0.118629	0.165849	0.4744329	0.21151
ENSG00000164609	SLU7	5	159838683	A	G	0.104374	0.00345278	0.024361	0.7700005	-0.204772	0.0144273	1.01E-45	-0.0168616	0.118972	0.8872958	0.5190341
ENSG00000164610	RP9	7	33141711	C	T	0.00994036	0.0420494	0.0593004	0.4799997	0.388396	0.0350665	1.64E-28	0.108264	0.152993	0.4791672	0.4132
ENSG00000164611	PTTG1	5	159852288	T	C	0.377734	0.00713487	0.0145244	0.5300002	0.13676	0.0120834	1.07E-29	0.0521709	0.106304	0.6235882	0.9486374
ENSG00000164615	CAMLG	5	134081019	A	G	0.133201	0.0288587	0.0238061	0.1499999	0.74662	0.0131486	0	0.0386525	0.0318924	0.2255263	0.5654626
ENSG00000164620	RELL2	5	141018580	A	G	0.184891	-0.0448746	0.0172498	0.002	-0.212179	0.01355	2.88E-55	0.211494	0.0824125	0.01027949	0.642341
ENSG00000164626	KCNK5	6	39176987	C	T	0.525845	0.0193536	0.0142509	0.1299999	-0.048273	0.00796136	1.33E-09	-0.40092	0.302529	0.1850956	0.184461
ENSG00000164631	ZNF12	7	6737309	T	C	0.157058	0.0325113	0.0193653	0.1100001	-0.0880473	0.0108291	4.27E-16	-0.369248	0.224582	0.1001434	0.07225599
ENSG00000164638	SLC29A4	7	5328848	A	G	0.408549	-0.0181129	0.0144996	0.1900002	0.122376	0.00812947	3.28E-51	-0.14801	0.118891	0.2131609	0.01690102
ENSG00000164649	CDCA7L	7	21963110	A	G	0.256461	0.001473	0.0158442	0.91	0.278567	0.00851859	1.51E-234	0.00528777	0.0568777	0.9259295	0.0623604
ENSG00000164654	MIOS	7	7627531	C	T	0.129225	-0.0104324	0.02165	0.6700003	0.238789	0.0170284	1.13E-44	-0.0436889	0.0907195	0.630103	0.554627
ENSG00000164659	ELAPOR2	7	86597618	T	C	0.0715706	0.0331839	0.0250714	0.29	0.411605	0.0136893	1.28E-198	0.0806207	0.0609703	0.1860698	0.9623271
ENSG00000164663	USP49	6	41810366	T	C	0.235586	-0.0563485	0.0167861	0.000519996	0.212466	0.0139498	2.21E-52	-0.265212	0.0809021	0.001044792	0.9100685
ENSG00000164669	NA	7	64648101	T	G	0.43837	0.00421849	0.0142576	0.9	-0.10325	0.00967018	1.30E-26	-0.0408569	0.138141	0.767411	0.6406499
ENSG00000164674	SYTL3	6	159128477	A	G	0.0159046	-0.0121665	0.0620165	0.8200001	-0.825778	0.0503468	1.86E-60	0.0147334	0.0751061	0.844479	0.1763601
ENSG00000164683	HEY1	8	80678171	T	C	0.148111	0.014602	0.0198103	0.35	0.410094	0.0109842	4.46E-305	0.0356065	0.0483161	0.4611542	0.1867321
ENSG00000164684	ZNF704	8	81663851	C	T	0.0695825	0.0306941	0.0263199	0.2	-0.125818	0.0178658	1.89E-12	-0.243956	0.212039	0.2499275	0.8748193
ENSG00000164687	FABP5	8	82194805	A	C	0.0854871	-0.00196081	0.0273764	0.9400001	-0.454697	0.0324297	1.16E-44	0.00431234	0.0602088	0.9429018	0.9239603
ENSG00000164691	TAGAP	6	159460842	T	C	0.055666	-0.0389202	0.0274818	0.1499999	-0.416054	0.0154171	2.12E-160	0.093546	0.0661443	0.1572823	0.4497314
ENSG00000164707	SLC13A4	7	135389468	C	A	0.148111	0.0282711	0.0189086	0.09299937	0.278132	0.0107705	4.82E-147	0.101646	0.0680981	0.1355301	0.9152942
ENSG00000164713	BRI3	7	97909426	C	T	0.481113	-0.0347859	0.0141719	0.008500021	-0.274421	0.0116241	3.19E-123	0.126761	0.0519212	0.01463011	0.06533507
ENSG00000164715	LMTK2	7	97787571	A	G	0.0506958	0.014546	0.0328634	0.6800001	-0.496678	0.0168321	2.29E-191	-0.0292866	0.0661739	0.6580766	0.9455084
ENSG00000164729	SLC35G3	17	33520475	G	A	0.111332	-0.0242096	0.0229584	0.2700001	-0.229949	0.0279281	1.82E-16	0.105283	0.100657	0.2955817	0.992993
ENSG00000164733	CTSB	8	11713495	A	G	0.277336	0.012452	0.0157281	0.4700002	-0.370598	0.00846085	0	-0.0335998	0.0424467	0.4286087	0.7498572
ENSG00000164751	PEX2	8	77902887	T	C	0.0188867	0.0187158	0.0537946	0.6499995	-0.536906	0.0257697	2.09E-96	-0.0348586	0.100208	0.7279425	0.3724007
ENSG00000164754	RAD21	8	117872639	A	T	0.40159	-0.00220848	0.0143962	0.9299999	-0.412593	0.00781637	0	0.00535269	0.0348922	0.8780777	0.05234378
ENSG00000164758	MED30	8	118542726	T	A	0.466203	0.00390385	0.014386	0.8700001	-0.178647	0.00795103	8.46E-112	-0.0218523	0.0805334	0.7861259	0.6836928
ENSG00000164776	PHKG1	7	56154564	T	C	0.157058	-0.0187419	0.0177438	0.4400003	-0.358984	0.0190058	1.43E-79	0.0522081	0.049505	0.291607	0.961382
ENSG00000164808	SPIDR	8	48411017	T	C	0.420477	-0.0169085	0.014666	0.1499999	-0.0870501	0.0088852	1.16E-22	0.194239	0.16964	0.2522075	0.8039446
ENSG00000164815	ORC5	7	103807641	G	T	0.470179	-0.00615835	0.0144206	0.4600002	-0.106296	0.00869282	2.20E-34	0.057936	0.135748	0.6695308	0.5088885
ENSG00000164818	DNAAF5	7	797764	C	T	0.463221	-0.00536068	0.0142297	0.6100002	0.253023	0.00875663	1.38E-183	-0.0211866	0.0562436	0.706402	0.1416532
ENSG00000164821	DEFA4	8	6794602	T	A	0.131213	0.0154646	0.0212408	0.35	0.310515	0.0215695	5.48E-47	0.0498031	0.0684926	0.4671452	0.6091662

ENSG00000164823	OSGIN2	8	90927101	T	G	0.306163	0.0389487	0.0151621	0.0129999	0.287287	0.00869498	2.13E-239	0.135574	0.0529362	0.01043441	0.2757859
ENSG00000164828	SUN1	7	895800	G	A	0.429423	-0.0219896	0.0144309	0.07399971	0.138671	0.00804143	1.23E-66	-0.158574	0.104471	0.1290468	0.2299685
ENSG00000164830	OXR1	8	107523697	C	T	0.0397614	0.0146185	0.0346803	0.7099994	-0.245401	0.0206257	1.22E-32	-0.0595699	0.14141	0.6735671	0.4280375
ENSG00000164845	NA	12	8390326	C	A	0.171968	0.0131971	0.0210273	0.5	0.457321	0.0197571	1.55E-118	0.0288574	0.0459962	0.5304057	0.8338356
ENSG00000164849	GPR146	7	1091554	G	C	0.12326	-0.00676878	0.0200192	0.4799997	0.710293	0.0112436	0	-0.00952956	0.0281848	0.7352806	0.3510147
ENSG00000164850	GPER1	7	1127647	A	C	0.219682	-0.0306481	0.0164432	0.03599979	0.272077	0.00976723	9.09E-171	-0.112645	0.060571	0.06292537	0.3246064
ENSG00000164855	TMEM184A	7	1593126	G	A	0.389662	-0.0361311	0.0142992	0.01400006	-0.268036	0.0142978	2.06E-78	0.134799	0.0538305	0.0122747	0.2659073
ENSG00000164867	NOS3	7	150699879	A	G	0.16004	-0.013002	0.0202343	0.5199996	-0.294259	0.0110968	6.09E-155	0.0441856	0.0687839	0.5206241	0.8415159
ENSG00000164877	MICALL2	7	1483619	A	G	0.464215	-0.0182153	0.0144526	0.16	0.134053	0.00923065	8.71E-48	-0.135881	0.108218	0.2092509	0.01155135
ENSG00000164880	INTS1	7	1527701	G	A	0.466203	-0.0202921	0.0144496	0.1199999	-0.403902	0.00885745	0	0.0502401	0.0357919	0.1604169	0.002995062
ENSG00000164889	SLC4A2	7	150763955	G	T	0.263419	-0.0222867	0.0162474	0.1	0.249331	0.0165096	1.57E-51	-0.0893858	0.0654321	0.1719122	0.9506868
ENSG00000164896	FASTK	7	150775832	T	C	0.173956	-0.00230756	0.0181682	0.7400005	0.107889	0.0112904	1.23E-21	-0.0213884	0.168413	0.8989406	0.3998883
ENSG00000164897	TMUB1	7	150779400	A	C	0.027833	0.0285871	0.0491931	0.5999997	0.504104	0.0355669	1.34E-45	0.0567087	0.0976671	0.5614883	0.4923716
ENSG00000164898	FMC1	7	139027634	G	A	0.275348	0.00338608	0.0168754	0.9400001	-0.115223	0.0100039	1.07E-30	-0.0293873	0.146481	0.8409946	0.5565179
ENSG00000164902	PHAX	5	125949452	G	C	0.0765408	0.0239681	0.0269036	0.4600002	0.142299	0.0145016	9.94E-23	0.168435	0.189842	0.3749502	0.5466386
ENSG00000164904	ALDH7A1	5	125904321	G	T	0.0974155	0.013098	0.0238368	0.7300002	0.476745	0.0185201	3.96E-146	0.0274738	0.0500104	0.5827575	0.4060935
ENSG00000164916	FOXK1	7	4747231	A	G	0.400596	0.00580289	0.0144331	0.59	0.328959	0.00918506	6.55E-281	0.0176402	0.0438778	0.6876627	0.48399
ENSG00000164919	COX6C	8	100895859	T	C	0.180915	0.0330306	0.0183905	0.07699987	0.43965	0.0102278	0	0.0751294	0.0418664	0.07273294	0.8326346
ENSG00000164924	YWHAZ	8	101947184	T	C	0.427435	0.0181092	0.0145295	0.2599998	0.228465	0.00834334	4.38E-165	0.0792647	0.0636621	0.2131003	0.3162073
ENSG00000164929	BAALC	8	104197735	T	C	0.175944	-0.0362642	0.0196972	0.04900044	0.221208	0.010708	8.24E-95	-0.163937	0.0893968	0.06668134	0.585326
ENSG00000164930	FZD6	8	104327877	G	A	0.26839	-0.0158948	0.0162731	0.28	-0.464725	0.00874995	0	0.0342026	0.0350226	0.3287732	0.03123091
ENSG00000164933	SLC25A32	8	104419140	C	A	0.168986	0.022034	0.0204541	0.33	0.136289	0.0108358	2.80E-36	0.161671	0.150628	0.2831312	0.06965651
ENSG00000164934	DCAF13	8	104441311	C	T	0.167992	0.0227843	0.0204595	0.32	-0.288029	0.0199615	3.39E-47	-0.0791042	0.071244	0.266858	0.1428594
ENSG00000164938	TP53INP1	8	95949919	G	C	0.56163	-0.00841186	0.0142241	0.8200001	-0.253174	0.00783716	6.09E-229	0.0332256	0.0561925	0.5543317	0.08827752
ENSG00000164941	INTS8	8	95859756	C	T	0.538767	0.0156271	0.0141959	0.14	0.199548	0.00786557	5.43E-142	0.0783125	0.0712072	0.2714267	0.02469423
ENSG00000164944	VIRMA	8	95532839	A	G	0.338966	-0.0423233	0.0157329	0.002	-0.137647	0.00848823	3.87E-59	0.307478	0.115861	0.007958021	0.02875927
ENSG00000164949	GEM	8	95268029	T	G	0.446322	-0.00405881	0.0142104	0.8700001	-0.0721476	0.00821333	1.57E-18	0.0562571	0.197067	0.7752828	0.8755233
ENSG00000164951	PDP1	8	94904164	C	T	0.405567	0.00808311	0.0145074	0.84	0.163423	0.0080705	3.60E-91	0.0494613	0.0888058	0.5775543	0.3402288
ENSG00000164961	WASHC5	8	126070292	A	T	0.0536779	0.0440447	0.0402812	0.3900004	0.273715	0.0201409	4.59E-42	0.160915	0.147641	0.2757534	0.7959706
ENSG00000164967	RPP25L	9	34611292	G	A	0.287276	-0.0118274	0.0152646	0.6800001	0.296833	0.00937306	4.19E-220	-0.0398453	0.0514403	0.4385798	0.6478192
ENSG00000164975	SNAPC3	9	15444326	A	G	0.0854871	0.0414998	0.0259562	0.09400046	-0.332836	0.0129484	1.03E-145	-0.124685	0.0781356	0.1105433	0.9623778
ENSG00000164978	NUDT2	9	34336606	G	A	0.141153	0.0400587	0.0197532	0.02199986	0.96589	0.00976429	0	0.0414734	0.0204551	0.04260783	0.05977835
ENSG00000164983	TMEM65	8	125354582	T	C	0.323062	0.00378743	0.0146843	0.5500004	0.0926248	0.00827126	4.15E-29	0.04089	0.158577	0.7965185	0.193946
ENSG00000164985	PSIP1	9	15487540	C	G	0.121272	-0.0244031	0.0234803	0.2700001	-0.0886747	0.0121682	3.16E-13	0.275198	0.267471	0.3035313	0.427005
ENSG00000164989	CCDC171	9	15807278	T	G	0.0516899	-0.0147718	0.0344607	0.4899999	0.397574	0.0180854	4.17E-107	-0.0371548	0.0866939	0.6682326	0.6734712
ENSG00000165006	UBAP1	9	34215762	T	C	0.457256	-0.010764	0.0142275	0.6700003	0.138842	0.00798953	1.21E-67	-0.0775269	0.10257	0.4497409	0.2917028
ENSG00000165025	SYK	9	93612450	C	A	0.482107	0.010142	0.014227	0.4500005	-0.451402	0.0075918	0	-0.0224678	0.0315196	0.475958	0.472963
ENSG00000165028	NIPSNAP3B	9	107533088	G	A	0.135189	0.00799221	0.0207843	0.4199997	0.316822	0.0121608	1.25E-149	0.0252262	0.0656096	0.7006159	0.9138913
ENSG00000165029	ABCA1	9	107616900	T	C	0.234592	-0.0181109	0.0176187	0.1900002	-0.141457	0.00940563	4.04E-51	0.128031	0.124842	0.3051068	0.3747589
ENSG00000165030	NFIL3	9	94178735	C	G	0.338966	0.00974478	0.0149727	0.29	0.110107	0.00857414	9.56E-38	0.0885025	0.136157	0.5156908	0.8048246
ENSG00000165046	LETM2	8	38255385	C	G	0.0198807	-0.032011	0.0455127	0.5199996	0.247524	0.0427414	6.99E-09	-0.129325	0.185223	0.4850449	0.9400225
ENSG00000165055	METTL2B	7	128121275	T	C	0.290258	0.0298987	0.0156098	0.03500016	0.255336	0.00974484	2.51E-151	0.117095	0.0612974	0.05609679	0.6476142
ENSG00000165060	FXN	9	71682634	A	G	0.202783	-0.00134479	0.0186507	0.8499999	-0.0578226	0.00991062	5.40E-09	0.0232572	0.322575	0.9425235	0.9318843
ENSG00000165061	ZMAT4	8	40571730	G	A	0.0695825	-0.0312015	0.0317161	0.17	0.630384	0.0158281	0	-0.049496	0.0503277	0.3253738	0.859724
ENSG00000165066	NKX6-3	8	41505776	T	C	0.444334	0.0290427	0.0142483	0.07199959	-0.155969	0.00828452	4.57E-79	-0.186208	0.0918871	0.04271514	0.8874885
ENSG00000165071	TMEM71	8	133735105	C	T	0.126243	-0.0022344	0.0210428	0.99	0.491651	0.0116472	0	-0.00454469	0.0428004	0.915437	0.5031594
ENSG00000165092	ALDH1A1	9	75605468	A	G	0.260437	-0.0007248	0.0163706	0.98	-0.377355	0.00882336	0	0.00192074	0.0433825	0.9646856	0.6767901
ENSG00000165097	KDM1B	6	18189822	G	A	0.0954274	0.000371408	0.0237407	0.6999999	-0.362003	0.014369	4.73E-140	-0.00102598	0.0655815	0.9875181	0.5370124
ENSG00000165102	HGSNAT	8	43026777	T	G	0.0576541	-0.00623349	0.0293112	0.8	0.152544	0.0160124	1.63E-21	-0.0408636	0.192197	0.831629	0.6296329
ENSG00000165113	GKAP1	9	86399383	T	A	0.425447	-0.00205539	0.0144973	0.8499999	-0.20811	0.01184	3.71E-69	0.00987647	0.0696641	0.8872595	0.8522563

ENSG00000165115	KIF27	9	86493977	T	C	0.0149105	-0.00127875	0.0548905	0.8700001	1.08056	0.038236	1.06E-175	-0.00118341	0.050798	0.9814139	0.5508322
ENSG00000165118	QNG1	9	86562563	G	A	0.280318	-0.00908461	0.0157882	0.4799997	0.241268	0.00886897	5.89E-163	-0.0376536	0.0654531	0.5651044	0.791853
ENSG00000165121	NA	9	88447653	A	G	0.265408	-0.0111864	0.016447	0.4500005	-0.3219	0.0216832	7.43E-50	0.0347511	0.0511471	0.4968619	0.6277901
ENSG00000165125	TRPV6	7	142576231	C	G	0.0487078	0.0272164	0.042658	0.5700002	0.635076	0.0329547	9.37E-83	0.0428553	0.0672067	0.523692	0.2246302
ENSG00000165131	LLCFC1	7	142637197	A	G	0.197813	0.0531952	0.0186274	0.007699987	-0.617023	0.0141326	0	-0.0862126	0.0302537	0.004376589	0.3472285
ENSG00000165138	ANKS6	9	101526429	C	G	0.117296	-0.00592911	0.0223645	0.8800001	0.17332	0.0117861	5.95E-49	-0.034209	0.129057	0.7909559	0.6115944
ENSG00000165140	FBP1	9	97383973	G	C	0.112326	0.0204477	0.0251058	0.6100002	0.327008	0.0142322	7.99E-117	0.0625296	0.0768225	0.4156736	0.8784044
ENSG00000165152	PGAP4	9	104265636	C	A	0.16004	0.0112476	0.0201879	0.5999997	-0.101238	0.0130728	9.62E-15	-0.1111	0.199925	0.5784096	0.6255786
ENSG00000165171	METTL27	7	73252892	T	C	0.274354	0.0156896	0.0165511	0.4600002	0.948411	0.0132969	0	0.016543	0.0174529	0.343198	0.05748027
ENSG00000165178	NCF1C	7	74580146	A	G	0.0914513	0.0557668	0.0235986	0.02300011	0.970035	0.0314304	3.75E-209	0.0574895	0.0243988	0.01846083	0.1258811
ENSG00000165181	SHOC1	9	114502870	G	A	0.241551	0.00377605	0.0163768	0.9400001	0.167461	0.00906919	3.96E-76	0.0225489	0.0978025	0.8176604	0.6163591
ENSG00000165185	KIAA1958	9	115340402	G	C	0.109344	0.00754632	0.0215143	0.56	0.0861158	0.0145172	2.99E-09	0.0876299	0.250266	0.7262286	0.3035646
ENSG00000165209	STRBP	9	125951317	A	G	0.0387674	0.0246633	0.0462507	0.5999997	-0.232482	0.0244553	1.97E-21	-0.106087	0.199256	0.5944383	0.7606849
ENSG00000165219	GAPVD1	9	128076779	C	A	0.45328	-0.00489171	0.0142536	0.8200001	-0.0679406	0.00806359	3.59E-17	0.0719998	0.209969	0.7316685	0.6717188
ENSG00000165233	CARD19	9	95867032	G	A	0.229622	-0.0163034	0.0169919	0.33	-0.502096	0.0133741	1.867347e-308	0.0324707	0.033853	0.3374744	0.4367505
ENSG00000165238	WNK2	9	96015026	G	A	0.349901	-0.0299072	0.014896	0.0530005	-0.14145	0.00817515	4.51E-67	0.211433	0.106016	0.04611363	0.8170863
ENSG00000165244	ZNF367	9	99164417	T	C	0.230616	-0.00339329	0.0167332	0.7300002	0.0692559	0.00928165	8.55E-14	-0.0489964	0.241703	0.8393593	0.4473311
ENSG00000165264	NDUFB6	9	32563078	T	C	0.483101	-0.0109998	0.0142144	0.4600002	0.081881	0.00796415	8.57E-25	-0.134339	0.174089	0.4403121	0.3168778
ENSG00000165271	NOL6	9	33467683	T	C	0.240557	-0.0219126	0.0162344	0.2	0.279402	0.00924523	1.25E-200	-0.0784268	0.058162	0.1775236	0.2646808
ENSG00000165272	AQP3	9	33444380	A	G	0.249503	0.015083	0.0162793	0.32	0.194156	0.00920173	7.95E-99	0.0776848	0.0839271	0.3546424	0.3535336
ENSG00000165275	TRMT10B	9	37766386	C	G	0.422465	-0.0339012	0.0143337	0.01499996	0.353856	0.00985109	1.46E-282	-0.095805	0.0405948	0.0182735	0.827148
ENSG00000165280	VCP	9	35064653	G	T	0.317097	0.00833304	0.0157008	0.4799997	-0.317564	0.00847854	4.98E-307	-0.0262405	0.0494464	0.595636	0.7500974
ENSG00000165282	PIGO	9	35092638	C	T	0.459245	0.013294	0.0142241	0.2300001	-0.192829	0.00793789	2.37E-130	-0.068942	0.07382	0.3503451	0.8074997
ENSG00000165283	STOML2	9	35101521	A	G	0.219682	0.00974856	0.0175589	0.4899999	0.0812394	0.0100611	6.77E-16	0.119998	0.216648	0.5796581	0.8628748
ENSG00000165300	SLITRK5	13	88328369	T	C	0.194831	0.0521348	0.0176759	0.001499996	0.121362	0.00987855	1.09E-34	0.429581	0.149785	0.004130896	0.8191734
ENSG00000165309	ARMC3	10	23271735	G	A	0.246521	-0.0218664	0.0164337	0.2599998	-0.230894	0.010116	2.61E-115	0.094703	0.0712949	0.1840699	0.8337088
ENSG00000165312	OTUD1	10	23729753	A	G	0.0178926	-0.0354467	0.0585467	0.4500005	-0.531653	0.0392312	7.73E-42	0.0666726	0.110232	0.5452854	0.9891325
ENSG00000165322	ARHGAP12	10	32156053	A	G	0.478131	-0.00751956	0.0142269	0.6800001	0.308741	0.00777607	0	-0.0243556	0.0460844	0.5971535	0.5353434
ENSG00000165338	HECTD2	10	93222341	A	C	0.0159046	-0.0633935	0.0393394	0.09599973	0.374599	0.0247009	5.99E-52	-0.16923	0.105609	0.1090606	0.9180511
ENSG00000165355	FBXO33	14	39884288	A	C	0.470179	0.0042292	0.0142827	0.95	0.086716	0.00795793	1.19E-27	0.0487707	0.164768	0.7672322	0.828534
ENSG00000165389	SPTSSA	14	34916778	C	T	0.279324	-0.0183919	0.0156844	0.2	0.121997	0.0104809	2.58E-31	-0.150756	0.129214	0.2433241	0.3869348
ENSG00000165392	WRN	8	30961301	C	T	0.201789	-0.022979	0.0184224	0.2099999	0.289519	0.00963602	2.50E-198	-0.0793696	0.0636859	0.2126665	0.01613689
ENSG00000165406	MARCHF8	10	46020194	T	C	0.0626243	0.0108193	0.0350738	0.6499995	0.27152	0.018478	7.02E-49	0.0398472	0.129204	0.7577746	0.4820266
ENSG00000165409	TSHR	14	81516989	G	T	0.505964	0.00119841	0.0142644	0.99	0.105698	0.00795666	2.86E-40	0.0113381	0.134957	0.9330466	0.06115682
ENSG00000165410	CFL2	14	35181811	T	C	0.149105	-0.00809257	0.0197577	0.5700002	0.536079	0.0111762	0	-0.0150959	0.0368573	0.6821172	0.3161372
ENSG00000165416	SUGT1	13	53244638	T	C	0.447316	0.020102	0.0143857	0.1499999	-0.136357	0.00813439	4.55E-63	-0.147422	0.105866	0.163762	0.9223299
ENSG00000165417	GTF2A1	14	81664758	T	C	0.232604	-0.00296575	0.0168507	0.91	-0.128632	0.00971568	5.18E-40	0.0230561	0.131011	0.8603049	0.01389012
ENSG00000165424	ZCCHC24	10	81173732	T	C	0.392644	-0.0167347	0.0170679	0.14	-0.199601	0.0108967	5.99E-75	0.0838406	0.0856323	0.3275422	0.1312507
ENSG00000165434	PGM2L1	11	74075440	G	T	0.477137	0.00441173	0.0142719	0.95	0.22262	0.00787294	6.71E-176	0.0198173	0.0641126	0.7572443	0.09897211
ENSG00000165457	FOLR2	11	71930319	G	T	0.105368	-0.0171952	0.0245578	0.28	0.11197	0.0129087	4.17E-18	-0.153569	0.220038	0.4852255	0.8031256
ENSG00000165458	INPPL1	11	71942447	T	C	0.0725646	-0.0384686	0.030839	0.14	-0.416591	0.0162136	1.36E-145	0.0923414	0.0741142	0.2127888	0.5817641
ENSG00000165475	CRYL1	13	21038901	C	T	0.239563	0.00981167	0.0165889	0.5999997	-0.250222	0.00917021	6.15E-164	-0.0392118	0.0663123	0.5543053	0.9165171
ENSG00000165476	REEP3	10	65333003	C	G	0.254473	0.0036488	0.0158189	0.5	-0.165353	0.0100853	2.06E-60	-0.0220668	0.0956772	0.8175961	0.6190893
ENSG00000165487	MICU2	13	22122594	C	T	0.197813	-0.0156365	0.0184939	0.3700002	-0.331733	0.0107584	8.93E-209	0.0471357	0.0557702	0.3980118	0.6752949
ENSG00000165490	DDIAS	11	82640168	T	C	0.476143	-0.0054518	0.0142139	0.6899999	-0.131922	0.00864029	1.25E-52	0.041326	0.107779	0.7013986	0.6635031
ENSG00000165494	PCF11	11	82883261	T	C	0.0755467	0.018556	0.0284326	0.6100002	0.103835	0.0165026	3.13E-10	0.178707	0.275295	0.5162424	0.9964201
ENSG00000165501	LRR1	14	50073402	G	C	0.258449	-0.0169968	0.0163964	0.3700002	-0.463378	0.0088504	0	0.0366802	0.0353915	0.3000087	0.574296
ENSG00000165502	RPL36AL	14	50086320	T	C	0.243539	-0.0196825	0.0167783	0.29	-0.730208	0.0180248	0	0.0269546	0.0229871	0.2409558	0.5721747
ENSG00000165506	DNAAF2	14	50096920	A	G	0.300199	-0.00532684	0.0154008	0.9199999	-0.0889728	0.00996046	4.16E-19	0.0598704	0.173225	0.729627	0.4569976
ENSG00000165507	DEPP1	10	45470343	G	A	0.170974	0.00437057	0.0187423	0.95	-0.119249	0.0101724	9.74E-32	-0.0366508	0.1572	0.8156475	0.09984874

ENSG00000165511	ZNF22-AS1	10	45494741	A	G	0.182903	-0.025128	0.0181182	0.1900002	0.115211	0.0103904	1.43E-28	-0.218105	0.158487	0.1687691	0.6105851
ENSG00000165512	ZNF22	10	45498348	G	T	0.055666	0.004894	0.0306649	0.6899999	0.157558	0.0167351	4.74E-21	0.0310616	0.194654	0.8732173	0.5487538
ENSG00000165516	KLHDC2	14	50242117	G	A	0.00894632	-0.00181773	0.053157	0.9599999	-0.490687	0.0392092	6.21E-36	0.00370446	0.108332	0.9727214	0.1324675
ENSG00000165521	EML5	14	89168935	C	T	0.0109344	-0.0531789	0.073794	0.3400001	0.382132	0.049567	1.26E-14	-0.139164	0.193953	0.4730582	0.6466349
ENSG00000165525	NEMF	14	50284959	C	T	0.0198807	0.0141105	0.0493278	0.84	-0.422761	0.077259	4.45E-08	-0.033377	0.116839	0.7751342	0.8184076
ENSG00000165526	RPUSD4	11	126076790	T	C	0.197813	-0.03175	0.0175162	0.06100002	-0.517786	0.00942122	0	0.0613187	0.0338474	0.07004451	0.04249139
ENSG00000165527	ARF6	14	50360650	C	T	0.140159	-0.0292358	0.0222395	0.2200002	-0.122078	0.0192887	2.47E-10	0.239484	0.186062	0.1980535	0.2812257
ENSG00000165548	TMEM63C	14	77654374	C	T	0.370775	0.00841405	0.014568	0.5500004	0.406598	0.00880397	0	0.0206938	0.0358318	0.5635845	0.6617235
ENSG00000165555	NOXRED1	14	77875112	C	T	0.497018	0.0174123	0.0142318	0.2999998	-0.0797325	0.00890528	3.45E-19	-0.218384	0.180153	0.2254309	0.1825803
ENSG00000165568	AKR1E2	10	4859537	A	G	0.202783	-0.0154425	0.017974	0.4899999	-0.224011	0.00986607	3.98E-114	0.0689363	0.0802945	0.3905926	0.8560314
ENSG00000165609	NUDT5	10	12222733	T	C	0.22664	-0.0383796	0.0174697	0.0129999	0.419092	0.0176076	3.20E-125	-0.0915779	0.0418618	0.02869693	0.7864421
ENSG00000165626	BEND7	10	13525729	T	C	0.479125	0.0320913	0.0143946	0.0259998	0.194797	0.0086662	6.84E-112	0.164742	0.0742579	0.02651985	0.3335213
ENSG00000165629	ATP5F1C	10	7839935	T	C	0.371769	0.0155184	0.0150671	0.4	0.156422	0.0141856	2.84E-28	0.0992088	0.0967429	0.3051325	0.1490362
ENSG00000165630	PRPF18	10	13650897	G	A	0.106362	-0.0074365	0.0208394	0.6800001	-0.142518	0.0122909	4.35E-31	0.0521795	0.146292	0.721332	0.09921013
ENSG00000165633	VSTM4	10	50275022	T	C	0.00894632	0.0197534	0.118339	0.99	0.406654	0.051716	3.74E-15	0.0485755	0.291072	0.8674609	0.6241486
ENSG00000165637	VDAC2	10	76980559	G	C	0.44831	-0.0231299	0.0142792	0.17	0.0980515	0.00850374	9.27E-31	-0.235896	0.14706	0.1086964	0.5932347
ENSG00000165644	COMTD1	10	76994757	A	C	0.0506958	0.00523049	0.0329656	0.8800001	0.630461	0.0173128	2.39E-290	0.0082963	0.0522886	0.8739339	0.8485569
ENSG00000165646	SLC18A2	10	119019772	C	G	0.499006	-0.00938326	0.0142412	0.1800002	-0.189144	0.00791886	4.36E-126	0.0496092	0.0753217	0.5101329	0.5601727
ENSG00000165650	PDZD8	10	119087489	G	A	0.277336	0.0166189	0.0148034	0.1100001	0.571085	0.00787698	0	0.0291006	0.0259246	0.2616473	0.3038823
ENSG00000165655	ZNF503	10	77159626	T	C	0.383698	0.000407853	0.0145554	0.83	-0.396412	0.00869622	0	-0.00102886	0.0367179	0.9776456	0.8902422
ENSG00000165659	NA	13	72226714	T	C	0.0377734	0.00580997	0.0355046	0.7600007	0.286615	0.0212834	2.46E-41	0.020271	0.123885	0.8700241	0.3850878
ENSG00000165660	ABRAXAS2	10	126507796	T	C	0.2833	-0.00277341	0.0161105	0.83	-0.0526331	0.0095561	3.63E-08	0.0526933	0.30624	0.8633863	0.2279648
ENSG00000165661	QSOX2	9	139117933	G	A	0.0159046	0.0235265	0.0426606	0.5199996	0.252557	0.0290924	3.92E-18	0.0931534	0.169255	0.5820647	0.4484557
ENSG00000165669	FAM204A	10	120083620	A	G	0.027833	0.0631893	0.0548299	0.16	0.335717	0.0360033	1.11E-20	0.188222	0.164565	0.252724	0.9635388
ENSG00000165672	PRDX3	10	120932780	C	T	0.441352	0.00161659	0.0142343	0.8800001	0.225896	0.0117336	1.36E-82	0.00715636	0.0630138	0.9095802	0.1225678
ENSG00000165678	GHITM	10	85906098	A	G	0.0646123	-0.00272019	0.0306231	0.9699999	0.533594	0.0166135	2.46E-226	-0.00509786	0.0573905	0.9292188	0.2876156
ENSG00000165682	CLEC1B	12	10152132	T	A	0.409543	0.00688503	0.0143558	0.6100002	0.447561	0.00766786	0	0.0153834	0.0320767	0.6315239	0.389144
ENSG00000165684	SNAPC4	9	139281639	G	C	0.0258449	0.0116025	0.0425772	0.99	-0.888979	0.0280514	2.06E-220	-0.0130515	0.0478963	0.7852415	0.8622728
ENSG00000165685	TMEM52B	12	10333770	C	T	0.445328	0.0132868	0.0142622	0.2399999	-0.0602063	0.00894038	1.65E-11	-0.220688	0.239145	0.3561006	0.4614876
ENSG00000165688	PMPCA	9	139311661	C	T	0.4334	0.00588236	0.0142002	0.6300007	0.050069	0.00798422	3.59E-10	0.117485	0.284231	0.6793546	0.5727839
ENSG00000165689	ENTR1	9	139300719	C	T	0.27336	0.0217937	0.0157426	0.1800002	-0.458304	0.00833543	0	-0.047553	0.0343606	0.1663772	0.5976096
ENSG00000165695	AK8	9	135677564	G	A	0.276342	0.00043865	0.016098	0.89	-0.136905	0.00954284	1.12E-46	-0.00320404	0.117585	0.9782614	0.4867196
ENSG00000165699	TSC1	9	135793377	A	C	0.144135	0.0278921	0.0201046	0.2200002	-0.178054	0.0110985	6.40E-58	-0.15665	0.113334	0.1669132	0.730081
ENSG00000165714	BORCS5	12	12564926	A	C	0.469185	-0.0028419	0.0143074	0.6300007	0.122901	0.00792439	3.01E-54	-0.0231235	0.116424	0.8425638	0.5562868
ENSG00000165716	DIPK1B	9	139612762	T	C	0.426441	0.0277902	0.0146632	0.05600025	-0.390208	0.0102441	0	-0.071219	0.0376244	0.05837214	0.1887592
ENSG00000165724	ZMYND19	9	140480736	T	C	0.0218688	0.0159839	0.0416092	0.56	-0.232755	0.0278622	6.61E-17	-0.0686725	0.178957	0.7011727	0.1624215
ENSG00000165730	STOX1	10	70621243	C	T	0.133201	-0.00539004	0.0210402	0.5700002	0.817941	0.0100553	0	-0.00658976	0.0257235	0.7978142	0.2316317
ENSG00000165731	RET	10	43599137	C	T	0.236581	0.000156849	0.0169069	0.91	-0.137063	0.00917325	1.77E-50	-0.00114436	0.123351	0.992598	0.362788
ENSG00000165732	DDX21	10	70730356	G	A	0.0944334	-0.00721123	0.0248427	0.9400001	-0.130236	0.0147066	8.32E-19	0.0553705	0.190854	0.7717246	0.4040322
ENSG00000165733	BMS1	10	43302552	G	A	0.0367793	0.0186357	0.0393729	0.5500004	-0.270601	0.0227354	1.15E-32	-0.0688678	0.145617	0.6362567	0.9966536
ENSG00000165752	STK32C	10	134083173	G	A	0.189861	0.0259566	0.0167744	0.07900053	0.486089	0.0105494	0	0.0533989	0.0345284	0.1219786	0.9359997
ENSG00000165782	PIP4P1	14	20927824	T	C	0.0238569	0.0712513	0.0327588	0.0179999	-0.168778	0.0242748	3.58E-12	-0.42216	0.20337	0.03790998	NA
ENSG00000165792	METTL17	14	21461559	A	C	0.310139	0.0135288	0.0152249	0.3800004	-0.327201	0.00928267	3.60E-272	-0.041347	0.0465455	0.3743715	0.4439698
ENSG00000165795	NDRG2	14	21511976	A	G	0.0427435	-0.038539	0.0381346	0.3700002	-0.588471	0.0267796	5.04E-107	0.0654901	0.0648713	0.3127169	0.2913517
ENSG00000165801	ARHGEF40	14	21548414	T	C	0.0477137	-0.000762868	0.0331905	0.98	0.41352	0.0213986	3.34E-83	-0.00184481	0.0802634	0.9816626	0.8840058
ENSG00000165802	NSMF	9	140347904	G	C	0.263419	-2.15E-07	0.0166013	0.83	0.475848	0.0142001	3.44E-246	-4.53E-07	0.0348878	0.9999896	0.3961108
ENSG00000165804	ZNF219	14	21565543	T	C	0.0397614	-0.015559	0.0379949	0.5099998	-0.157603	0.0187806	4.79E-17	0.0987226	0.241366	0.6825282	0.5744459
ENSG00000165806	CASP7	10	115464802	C	T	0.254473	-0.0262004	0.0161692	0.07199959	-0.592229	0.00830595	0	0.0442403	0.0273093	0.1052385	0.5139904
ENSG00000165810	BTNL9	5	180477874	A	G	0.295229	-0.0133991	0.0156592	0.35	-0.311732	0.00892932	5.05E-267	0.0429827	0.0502479	0.3923228	0.9797036
ENSG00000165813	CCDC186	10	115907300	A	G	0.0725646	-0.0192723	0.024755	0.3400001	-0.142274	0.0143691	4.10E-23	0.135459	0.174532	0.4376745	0.9854831

ENSG00000165819	METTL3	14	21972897	C	T	0.587475	0.00972478	0.0163258	0.6300007	0.129106	0.015079	1.11E-17	0.0753238	0.126758	0.5523562	0.6469594
ENSG00000165832	TRUB1	10	116717691	T	C	0.479125	-0.000530852	0.0141878	0.8800001	0.0861597	0.00793937	1.95E-27	-0.00616126	0.16467	0.9701534	0.7568788
ENSG00000165861	ZFYVE1	14	73465039	A	G	0.180915	0.0139164	0.0186122	0.3900004	0.116722	0.0108714	6.85E-27	0.119227	0.159844	0.4557299	0.9596924
ENSG00000165863	SPMIP5	10	118426491	A	T	0.0606362	-0.0514444	0.0334368	0.1100001	-0.131413	0.0181936	5.09E-13	0.391472	0.26015	0.1323763	0.8015568
ENSG00000165879	FRAT1	10	99080347	A	G	0.33499	-0.0109606	0.0151612	0.58	0.32725	0.00830232	0	-0.033493	0.0463369	0.469793	0.4739303
ENSG00000165886	UBTD1	10	99294795	A	G	0.0447316	0.0539867	0.0339209	0.05899973	0.178721	0.0208562	1.04E-17	0.302072	0.193044	0.1176329	0.2361777
ENSG00000165895	ARHGAP42	11	100710526	T	C	0.0367793	-0.126272	0.0400863	0.002300011	0.217881	0.0315934	5.33E-12	-0.579546	0.202266	0.004166642	0.3254134
ENSG00000165898	ISCA2	14	74962116	T	C	0.349901	-0.00672977	0.0145766	0.5300002	0.545942	0.00754056	0	-0.0123269	0.0267004	0.6443153	0.1422907
ENSG00000165905	LARGE2	11	45946909	A	G	0.0308151	0.0123344	0.0404014	0.6600001	-0.330797	0.0289837	3.59E-30	-0.0372869	0.122177	0.7602236	0.3535267
ENSG00000165914	TTC7B	14	91144877	T	C	0.326044	0.0157567	0.0151872	0.1900002	0.0836564	0.00937537	4.54E-19	0.18835	0.182766	0.3027491	0.5183634
ENSG00000165915	SLC39A13	11	47433365	T	C	0.293241	0.00593848	0.0156955	0.81	-0.0593324	0.00878946	1.47E-11	-0.100088	0.26495	0.7056069	0.6584836
ENSG00000165916	PSMC3	11	47444156	A	G	0.291252	0.00842263	0.0157167	0.6999999	-0.126035	0.00876325	6.69E-47	-0.0668278	0.124788	0.5922823	0.9096743
ENSG00000165917	RAPSN	11	47465019	C	T	0.422465	-0.00429794	0.0143971	0.9599999	-0.0804248	0.00847773	2.39E-21	0.0534405	0.179102	0.7654128	0.9238934
ENSG00000165923	AGBL2	11	47709042	C	T	0.142147	-0.00384374	0.020441	0.7899998	0.100455	0.0110833	1.26E-19	-0.0382634	0.203528	0.8508765	0.4934508
ENSG00000165929	TC2N	14	92289987	C	T	0.16998	-0.0347701	0.0190223	0.05399953	-0.62033	0.00996956	0	0.056051	0.030678	0.06768898	0.1318009
ENSG00000165934	CPSF2	14	92609518	C	T	0.333996	0.00930686	0.0151448	0.56	-0.519731	0.0116985	0	-0.0179071	0.0291425	0.5389072	0.2114468
ENSG00000165943	MOAP1	14	93649907	A	G	0.377734	-0.0180751	0.015282	0.17	0.110164	0.00859772	1.38E-37	-0.164074	0.13931	0.2388909	0.6396393
ENSG00000165948	IFI27L1	14	94558910	A	G	0.481113	0.00418999	0.0141956	0.8	-0.400186	0.00842762	0	-0.0104701	0.0354731	0.7678751	0.04538225
ENSG00000165949	IFI27	14	94577107	A	G	0.354871	0.000738239	0.0146711	0.9599999	0.227556	0.00807671	1.20E-174	0.0032442	0.0644725	0.959868	0.6109605
ENSG00000165959	CLMN	14	95720489	C	T	0.185885	0.000365401	0.0184899	0.7499995	0.482045	0.0149879	5.96E-227	0.000758022	0.0383572	0.9842331	0.6756346
ENSG00000165983	PTER	10	16517339	T	C	0.451292	-0.0152033	0.014348	0.2599998	-0.544351	0.0073602	0	0.0279292	0.0263607	0.2893711	0.7781099
ENSG00000165985	C1QL3	10	16559873	T	C	0.451292	-0.0152033	0.014348	0.2599998	-0.378342	0.00849865	0	0.040184	0.0379341	0.2894581	0.6046455
ENSG00000165995	CACNB2	10	18630202	C	T	0.428429	0.0439636	0.0142757	0.004399973	0.138691	0.00805959	2.30E-66	0.316989	0.104567	0.002433832	0.7325857
ENSG00000165996	HACD1	10	17645667	C	T	0.434394	0.00877022	0.0142349	0.4100001	0.132915	0.00793866	6.39E-63	0.0659834	0.10717	0.538099	0.7646763
ENSG00000165997	ARL5B	10	18959451	A	G	0.0904573	0.075996	0.0258431	0.00329997	0.0950625	0.015041	2.61E-10	0.799432	0.29984	0.00767143	0.3616587
ENSG00000166002	SMCO4	11	93244156	T	G	0.168986	0.00562646	0.0187487	0.6800001	0.7267	0.0108392	0	0.00774248	0.0258	0.7641042	0.5792796
ENSG00000166004	CEP295	11	93429163	A	G	0.00994036	-0.036199	0.0779803	0.6100002	0.310466	0.0459024	1.35E-11	-0.116596	0.251763	0.6432801	0.4940472
ENSG00000166012	TAF1D	11	93490335	T	C	0.00497018	0.234491	0.131693	0.09299937	1.34235	0.0553837	9.02E-130	0.174687	0.098371	0.07576547	0.1883769
ENSG00000166016	ABTB2	11	34276045	G	A	0.351889	0.00348466	0.0147829	0.8700001	0.245267	0.0081722	6.76E-198	0.0142076	0.0602745	0.8136538	0.04312207
ENSG00000166024	R3HCC1L	10	99949520	C	T	0.220676	0.0396776	0.0176062	0.01700004	-0.541373	0.0116748	0	-0.0732907	0.0325598	0.02438817	0.05060298
ENSG00000166025	AMOTL1	11	94524757	T	C	0.0745527	-0.0338362	0.0270949	0.1299999	-0.281282	0.0202407	6.62E-44	0.120293	0.0967148	0.2135764	0.5368509
ENSG00000166033	HTRA1	10	124247732	A	C	0.318091	0.00200538	0.015431	0.8499999	-0.243973	0.00892579	1.70E-164	-0.00821968	0.0632495	0.8966009	0.5287016
ENSG00000166035	LIPC	15	58781959	T	C	0.0864811	-0.00268967	0.0270393	0.7300002	0.565209	0.0149254	0	-0.00475872	0.0478397	0.9207633	0.5474085
ENSG00000166037	CEP57	11	95544493	G	A	0.373757	0.00293251	0.0145875	0.83	0.280483	0.00818635	2.86E-257	0.0104552	0.0520093	0.8406786	0.4125143
ENSG00000166046	TCP11L2	12	106718250	C	T	0.151093	-0.0178191	0.0196645	0.3100002	0.433172	0.011029	0	-0.0411363	0.0454086	0.364981	0.7269791
ENSG00000166068	SPRED1	15	38596988	T	C	0.0715706	-0.0164118	0.0283352	0.56	-0.285428	0.0142476	2.82E-89	0.0574989	0.0993141	0.5626162	0.6755942
ENSG00000166086	JAM3	11	133980358	A	G	0.245527	-0.010741	0.0172005	0.3700002	0.145593	0.00943264	9.52E-54	-0.0737743	0.118238	0.5326616	0.6985747
ENSG00000166091	CMTM5	14	23847499	T	G	0.0805169	-0.0834348	0.0333449	0.01	-0.181059	0.0178102	2.81E-24	0.460816	0.189663	0.0151129	0.4856562
ENSG00000166105	GLB1L3	11	134166798	C	G	0.412525	-0.00900854	0.0145466	0.6899999	0.0838805	0.00825206	2.85E-24	-0.107397	0.173742	0.5364816	0.8683246
ENSG00000166123	GPT2	16	46941749	G	A	0.0258449	-0.0716294	0.0453308	0.09400046	-0.490804	0.030501	2.93E-58	0.145943	0.0928045	0.115815	0.9437092
ENSG00000166126	AMN	14	103394463	A	G	0.2167	0.0179103	0.0176525	0.4400003	-0.0571808	0.01015	1.76E-08	-0.313222	0.31368	0.3180179	0.3355361
ENSG00000166128	RAB8B	15	63520824	A	G	0.0308151	0.0195821	0.0457383	0.3900004	-0.146307	0.02475	3.39E-09	-0.133842	0.313437	0.6693684	0.2748458
ENSG00000166130	IKBIP	12	99023037	C	G	0.50497	0.0146385	0.0142353	0.1900002	-0.265768	0.00781893	3.10E-253	-0.05508	0.0535874	0.3040187	0.5648665
ENSG00000166133	RPUSD2	15	40864079	C	T	0.213718	-0.000376307	0.0173108	0.91	-0.0697815	0.00926221	4.92E-14	0.00539265	0.248073	0.9826568	0.9675159
ENSG00000166135	HIF1AN	10	102299296	A	G	0.191849	0.0145161	0.0171191	0.3400001	-0.335145	0.00941818	2.40E-277	-0.0433129	0.0510942	0.3966012	0.04782542
ENSG00000166136	NDUFB8	10	102278480	C	A	0.192843	0.0145017	0.0170955	0.35	-0.0797083	0.00959139	9.54E-17	-0.181935	0.21559	0.3987303	0.02897022
ENSG00000166140	ZFYVE19	15	41103025	G	T	0.384692	-0.00689683	0.0148296	0.6300007	-0.52319	0.00761617	0	0.0131823	0.0283452	0.6418876	0.4741719
ENSG00000166145	SPINT1	15	41143310	A	C	0.242545	-0.0175651	0.0167571	0.25	-0.128307	0.00927826	1.71E-43	0.136899	0.130976	0.2959213	0.2794543
ENSG00000166147	FBN1	15	48819274	G	A	0.256461	-0.00268588	0.0165023	0.98	-0.214784	0.00923652	1.30E-119	0.012505	0.0768339	0.8707122	0.3444923
ENSG00000166164	BRD7	16	50375121	C	T	0.157058	0.00813825	0.0192756	0.6300007	0.767604	0.0112319	0	0.0106022	0.0251119	0.6728815	0.2068941

ENSG00000166165	CKB	14	103987722	A	G	0.481113	0.0212386	0.0142113	0.09299937	-0.212854	0.00791377	2.41E-159	-0.0997803	0.0668686	0.1356506	0.5727693
ENSG00000166166	TRMT61A	14	103999465	C	G	0.270378	-0.0226594	0.0157636	0.1800002	0.222045	0.00888521	7.77E-138	-0.102049	0.0711102	0.1512647	0.6053845
ENSG00000166167	BTRC	10	103215449	T	C	0.152087	0.0111514	0.0213936	0.4600002	0.184721	0.0122215	1.30E-51	0.0603689	0.115885	0.6024088	0.05469128
ENSG00000166169	POLL	10	103343333	C	T	0.298211	-0.00645056	0.0155531	0.6600001	0.104099	0.00873208	9.16E-33	-0.0619659	0.149498	0.6785127	0.2760037
ENSG00000166170	BAG5	14	104026024	T	C	0.261431	-0.00995663	0.0157779	0.5199996	0.186894	0.00891204	1.21E-97	-0.0532741	0.0844596	0.5281951	0.136272
ENSG00000166171	DPCD	10	103349871	T	C	0.0765408	-0.0444218	0.0264041	0.09200046	0.169342	0.0150701	2.68E-29	-0.26232	0.157659	0.09614479	0.7784158
ENSG00000166181	API5	11	43349796	T	C	0.361829	0.000431998	0.0145442	0.8499999	-0.175966	0.0120541	2.89E-48	-0.002455	0.0826535	0.9763044	0.2585838
ENSG00000166188	ZNF319	16	58031464	A	C	0.227634	-0.018376	0.0165405	0.3100002	-0.402634	0.00997875	0	0.0456394	0.0410963	0.2667625	0.6572361
ENSG00000166189	HPS6	10	103826469	A	G	0.0626243	0.0138165	0.0320865	0.5300002	0.604263	0.0285577	2.26E-99	0.022865	0.0531112	0.6668229	0.03459275
ENSG00000166199	ALKBH3	11	43922088	G	A	0.4334	0.0101332	0.0143516	0.4400003	-0.191683	0.00796874	7.51E-128	-0.0528643	0.0749037	0.4803351	0.5791046
ENSG00000166224	SGPL1	10	72608323	T	A	0.0516899	-0.0769226	0.0292262	0.01499996	0.714676	0.0167799	0	-0.107633	0.0409723	0.008615072	0.5891457
ENSG00000166225	FRS2	12	69918845	A	G	0.466203	1.81E-05	0.0142073	0.98	-0.0681828	0.00799536	1.49E-17	-0.000264757	0.208371	0.9989862	0.5303295
ENSG00000166226	CCT2	12	69987232	T	C	0.32505	0.0106436	0.0149502	0.4700002	0.130764	0.00833976	2.09E-55	0.0813955	0.114447	0.4769577	0.542669
ENSG00000166228	PCBD1	10	72645289	T	G	0.319085	-0.00587374	0.0147872	0.59	-0.155244	0.00827388	1.51E-78	0.0378354	0.0952724	0.6912724	0.5391906
ENSG00000166260	COX11	17	53037704	C	A	0.271372	-0.00935566	0.0157098	0.7600007	-0.310731	0.00884191	1.49E-270	0.0301085	0.0505648	0.5515463	0.7683866
ENSG00000166261	ZNF202	11	123603634	T	C	0.424453	-0.00183264	0.0142824	0.9699999	0.0953317	0.00799561	8.98E-33	-0.0192238	0.149827	0.8979059	0.7592936
ENSG00000166262	FAM227B	15	49766143	T	C	0.0666004	0.000888171	0.0315959	0.8200001	0.259366	0.0307161	3.07E-17	0.00342439	0.12182	0.9775743	0.7707914
ENSG00000166263	STXBP4	17	53143867	T	C	0.271372	-0.00756632	0.0157222	0.6800001	0.277615	0.00866616	3.62E-225	-0.0272547	0.0566394	0.6303769	0.9189498
ENSG00000166265	CYYR1	21	27892065	T	C	0.296223	-0.00224439	0.0157495	0.8600001	0.282248	0.00854566	3.20E-239	-0.00795184	0.0558008	0.8866818	0.3645843
ENSG00000166266	CUL5	11	107928981	T	G	0.0606362	-0.0101146	0.0332762	0.7600007	-0.703188	0.017996	0	0.0143839	0.0473234	0.7611661	0.4809058
ENSG00000166272	WBP1L	10	104539874	C	T	0.394632	0.0240562	0.0147415	0.1299999	0.145605	0.00894049	1.24E-59	0.165216	0.10175	0.1044317	0.001581185
ENSG00000166275	BORCS7	10	104623485	T	C	0.251491	-0.0198669	0.0165781	0.1499999	-0.67685	0.00852642	0	0.029352	0.0244958	0.2308214	0.5925408
ENSG00000166278	C2	6	31889505	A	G	0.0675944	-0.052454	0.026633	0.09499921	-0.255966	0.0269983	2.52E-21	0.204926	0.10627	0.05381253	0.2707934
ENSG00000166295	ANAPC16	10	73985702	C	T	0.0298211	0.0146644	0.0368851	0.5	-0.410367	0.0269567	2.48E-52	-0.0357348	0.0899138	0.6910473	0.2723702
ENSG00000166311	SMPD1	11	6413941	G	C	0.10835	0.00120512	0.0225706	0.8700001	-0.133591	0.0134677	3.43E-23	-0.00902094	0.168955	0.9574191	0.698746
ENSG00000166321	NUDT13	10	74880901	G	T	0.0596421	0.0402215	0.0287446	0.2	-0.560594	0.0151403	4.29E-300	-0.071748	0.0513119	0.162031	0.9648516
ENSG00000166323	C11orf65	11	108258752	C	T	0.171968	-0.0169499	0.0196735	0.4299995	0.0750553	0.0113284	3.46E-11	-0.225832	0.264327	0.3929019	0.6520341
ENSG00000166326	TRIM44	11	35757064	T	A	0.132207	0.015928	0.0199512	0.3700002	0.253118	0.0114118	5.31E-109	0.0629272	0.0788728	0.4249684	0.7723021
ENSG00000166333	ILK	11	6628531	A	G	0.168986	0.0207751	0.0193597	0.2599998	-0.104681	0.0104945	1.97E-23	-0.198461	0.186007	0.2859926	0.5582589
ENSG00000166337	TAF10	11	6630712	A	T	0.489066	0.00577618	0.0141865	0.5400003	0.075584	0.00794383	1.82E-21	0.0764207	0.187864	0.6841637	0.4278102
ENSG00000166340	TPP1	11	6637346	G	A	0.426441	0.0096406	0.0144001	0.6200004	-0.350578	0.00778371	0	-0.0274992	0.0410799	0.5032356	0.2273834
ENSG00000166341	DCHS1	11	6659820	C	T	0.16004	0.0177916	0.0197009	0.4700002	0.379841	0.0108993	4.24E-266	0.0468396	0.0518836	0.3666413	0.475236
ENSG00000166347	CYB5A	18	71939890	C	T	0.261431	0.00676631	0.0157572	0.5099998	0.2074	0.00883156	5.94E-122	0.0326244	0.0759875	0.6676766	0.7887135
ENSG00000166352	IFTAP	11	36655437	G	A	0.12326	-0.0144321	0.023295	0.5400003	0.14941	0.0123606	1.23E-33	-0.0965939	0.156118	0.536098	0.3974539
ENSG00000166359	WDR88	19	33644848	A	C	0.337972	0.000568323	0.0153431	0.7600007	0.103894	0.0084753	1.51E-34	0.00547024	0.147682	0.9704525	0.8488773
ENSG00000166377	ATP9B	18	76983784	G	C	0.354871	0.0271282	0.0146314	0.0519996	-0.425361	0.00784093	0	-0.0637769	0.0344177	0.06387859	0.162942
ENSG00000166387	PPFIBP2	11	7606443	G	A	0.372763	-0.0183356	0.014604	0.2099999	-0.707576	0.0071286	0	0.0259133	0.0206411	0.2093268	0.2970008
ENSG00000166394	CYB5R2	11	7692392	A	G	0.149105	-0.00165236	0.0200264	0.8700001	0.663207	0.0106057	0	-0.00249147	0.0301963	0.9342419	0.943409
ENSG00000166398	GARRE1	19	34795974	A	G	0.0487078	0.0193431	0.0375165	0.89	0.633555	0.0164492	0	0.0305311	0.0592212	0.6061737	0.415895
ENSG00000166401	SERPINB8	18	61654718	C	T	0.286282	-0.000228548	0.0161621	1	-0.500265	0.0085711	0	0.000456854	0.0323071	0.9887175	0.9318272
ENSG00000166402	TUB	11	8084225	G	A	0.512922	-0.000646641	0.0142984	0.99	-0.0813737	0.00793627	1.14E-24	0.00794656	0.175714	0.9639285	0.5003532
ENSG00000166405	RIC3	11	8159099	C	T	0.0934394	-0.00532511	0.0246137	0.6999999	-0.313765	0.0134559	2.91E-120	0.0169717	0.0784498	0.8287242	0.3425126
ENSG00000166411	IDH3A	15	78444065	G	A	0.300199	0.000356884	0.0159462	0.9400001	-0.099732	0.0149425	2.48E-11	-0.00357843	0.159891	0.9821445	0.2596123
ENSG00000166428	PLD4	14	105395363	T	C	0.465209	-0.0176561	0.0142213	0.33	0.201802	0.00912357	2.08E-108	-0.087492	0.0705823	0.215133	0.1476265
ENSG00000166435	XRRA1	11	74589514	A	G	0.2833	-0.0289334	0.0157203	0.05800027	0.837179	0.00755867	0	-0.0345606	0.0187803	0.06573039	0.02776707
ENSG00000166436	TRIM66	11	8663498	T	C	0.378728	-0.0162789	0.0143747	0.3800004	0.881853	0.00789702	0	-0.0184599	0.0163014	0.2574618	0.7686218
ENSG00000166439	RNF169	11	74506685	G	A	0.476143	0.0352431	0.0141841	0.009800089	-0.205886	0.00865153	3.54E-125	-0.171178	0.0692676	0.0134639	0.4652653
ENSG00000166441	RPL27A	11	8720132	G	T	0.177932	0.0203888	0.0193632	0.25	-0.22077	0.0126501	3.32E-68	-0.0923533	0.0878672	0.2932333	0.4693143
ENSG00000166444	DENND2B	11	8823698	T	C	0.379722	-0.0164423	0.0143701	0.3700002	0.0863274	0.00810266	1.67E-26	-0.190464	0.167418	0.2552623	0.7840846
ENSG00000166451	CENPN	16	81053411	G	A	0.220676	0.0285961	0.0170967	0.05899973	0.322721	0.017486	4.67E-76	0.0886094	0.0531939	0.09575672	0.9717229

ENSG00000166452	AKIP1	11	8937158	G	A	0.417495	-0.0101564	0.0142568	0.6499995	0.586314	0.00816477	0	-0.0173225	0.0243172	0.4762451	0.7086597
ENSG00000166454	ATMIN	16	81075207	C	A	0.0735586	0.0415909	0.0270122	0.1299999	0.191705	0.0155357	5.54E-35	0.216953	0.141998	0.1265473	0.9491755
ENSG00000166471	TMEM41B	11	9319264	C	T	0.212724	-0.0098285	0.0179413	0.64	-0.847166	0.0166584	0	0.0116016	0.0211793	0.5838413	0.9656561
ENSG00000166478	ZNF143	11	9515968	A	G	0.0715706	0.0210455	0.0262089	0.5	0.861875	0.0145808	0	0.0244183	0.030412	0.422023	0.8730901
ENSG00000166479	TMX3	18	66361730	A	G	0.11829	0.00168862	0.0253278	0.8600001	0.423887	0.0145513	1.48E-186	0.00398365	0.0597514	0.9468441	0.4847463
ENSG00000166483	WEE1	11	9605116	G	T	0.239563	-0.00954882	0.0167736	0.8200001	0.22691	0.00944247	1.33E-127	-0.0420819	0.0739425	0.5692767	0.5163334
ENSG00000166484	MAPK7	17	19283945	G	T	0.220676	0.0072865	0.0173296	0.7700005	-0.133848	0.00928534	4.16E-47	-0.0544386	0.129527	0.6742759	0.8513334
ENSG00000166501	PRKCB	16	24039627	C	A	0.411531	0.00692428	0.0143703	0.58	-0.547296	0.00745708	0	-0.0126518	0.0262575	0.6299223	0.8155902
ENSG00000166503	HDGFL3	15	83830545	A	G	0.0616302	-0.00279946	0.028161	0.98	0.819941	0.014718	0	-0.00341422	0.0343452	0.9208136	0.9784385
ENSG00000166507	NDST2	10	75566629	T	C	0.243539	-0.00765437	0.0157183	0.6899999	-0.297042	0.00854317	7.01E-265	0.0257686	0.0529212	0.6263121	0.4760743
ENSG00000166508	MCM7	7	99694957	C	T	0.0675944	-0.0962284	0.0346525	0.003099988	0.572698	0.0188251	2.81E-203	-0.168026	0.060759	0.005684315	0.9351354
ENSG00000166523	CLEC4E	12	8689730	G	A	0.176938	0.00696059	0.0185576	0.7800007	0.634049	0.0102893	0	0.010978	0.0292689	0.7076059	0.190321
ENSG00000166526	ZNF3	7	99670913	C	G	0.326044	0.0266732	0.0159167	0.08500021	-0.0968669	0.0088226	4.80E-28	-0.275359	0.166218	0.09759749	0.0215666
ENSG00000166527	CLEC4D	12	8668516	A	G	0.223658	-0.00352807	0.0181668	0.6800001	0.696723	0.00948448	0	-0.00506381	0.0260747	0.8460165	0.394209
ENSG00000166529	ZSCAN21	7	99655025	C	T	0.328032	0.0241013	0.0157293	0.1100001	-0.228707	0.00958217	6.59E-126	-0.105381	0.0689164	0.1262378	0.0119002
ENSG00000166532	RIMKLB	12	8884943	T	C	0.319085	0.0125419	0.0156611	0.3900004	0.116051	0.0092253	2.73E-36	0.108073	0.135224	0.4241666	0.0447424
ENSG00000166546	BEAN1	16	66494316	G	C	0.0497018	-0.0151371	0.0462705	0.7499995	0.375268	0.0245929	1.43E-52	-0.0403368	0.123328	0.7436161	0.5764607
ENSG00000166548	TK2	16	66564176	G	C	0.135189	0.012291	0.0225731	0.6999999	0.712605	0.0115019	0	0.017248	0.0316781	0.5861135	0.6136213
ENSG00000166557	TMED3	15	79653869	C	T	0.00397614	0.0573437	0.0917546	0.4500005	-1.00883	0.0568898	2.33E-70	-0.056842	0.0910083	0.5322465	0.2151288
ENSG00000166562	SEC11C	18	56816388	A	G	0.437376	-0.00918837	0.0143653	0.6800001	-0.148566	0.0119007	9.15E-36	0.0618472	0.0968201	0.522963	0.6528102
ENSG00000166575	TMEM135	11	86891843	A	G	0.487078	-0.0101222	0.0142345	0.4199997	-0.0730903	0.00793476	3.22E-20	0.138489	0.195332	0.4783283	0.4776222
ENSG00000166578	IQCD	12	113646072	G	A	0.0894632	-0.021611	0.0278636	0.5400003	0.188668	0.0175796	7.19E-27	-0.114545	0.148071	0.4391779	0.4060742
ENSG00000166579	NDEL1	17	8355089	A	G	0.422465	0.0208695	0.0145322	0.1499999	-0.136857	0.00810727	6.23E-64	-0.152491	0.106569	0.1524537	0.9823934
ENSG00000166582	CENPV	17	16251409	C	T	0.464215	-0.00880033	0.0142066	0.3900004	0.405514	0.00837474	0	-0.0217017	0.0350364	0.5356509	0.5538629
ENSG00000166592	RRAD	16	66957564	A	C	0.00994036	-0.166675	0.0781411	0.02100003	0.756922	0.0920091	1.93E-16	-0.220201	0.106649	0.03894884	0.8499079
ENSG00000166598	HSP90B1	12	104335654	A	G	0.15507	0.00742429	0.0205558	0.4899999	-0.133024	0.0170894	7.03E-15	-0.0558116	0.154693	0.718257	0.9342049
ENSG00000166619	BLCAP	20	36143574	C	G	0.276342	-0.00234104	0.0169496	0.7199992	0.0645649	0.00972304	3.13E-11	-0.0362587	0.262577	0.890171	0.4648313
ENSG00000166664	CHRFAM7A	15	30669747	A	G	0.11332	-0.00515008	0.0220347	0.8	0.634118	0.029161	7.64E-105	-0.00812165	0.0347506	0.8152083	0.3213311
ENSG00000166667	NA	7	101991540	G	A	0.202783	0.00949191	0.0175914	0.5	-0.506748	0.0213641	2.26E-124	-0.018731	0.0347232	0.5895852	0.1644892
ENSG00000166669	ATF7IP2	16	10498893	T	G	0.122266	0.0254559	0.0194258	0.2300001	0.397811	0.0107826	5.83E-298	0.06399	0.0488626	0.1903341	0.9019464
ENSG00000166685	COG1	17	71196887	A	G	0.358847	-0.00426194	0.0146805	0.7099994	0.332343	0.00789434	0	-0.0128239	0.0441738	0.7715821	0.6373607
ENSG00000166689	PLEKHA7	11	16917916	G	A	0.402584	0.0149964	0.0147715	0.25	0.193425	0.00815899	3.05E-124	0.0775308	0.076438	0.3104417	0.7166681
ENSG00000166704	ZNF606	19	58501569	A	G	0.388668	-0.00494909	0.0144148	0.8700001	0.100773	0.00809668	1.47E-35	-0.0491113	0.143097	0.7314455	0.5208014
ENSG00000166710	B2M	15	45007375	G	C	0.0357853	0.0160233	0.0347372	0.4400003	-0.206168	0.0188992	1.05E-27	-0.0777197	0.16864	0.6448989	0.7368275
ENSG00000166716	ZNF592	15	85320762	C	T	0.284294	-0.00753979	0.0158841	0.5500004	0.269835	0.013328	3.87E-91	-0.0279422	0.0588821	0.6351114	0.4060705
ENSG00000166736	HTR3A	11	113853319	A	G	0.11829	0.00350005	0.0223717	0.91	0.138488	0.0123575	3.78E-29	0.0252733	0.161558	0.8756904	0.2352738
ENSG00000166743	ACSM1	16	20672385	C	T	0.17992	-0.000352387	0.0181113	0.89	0.242135	0.0100478	2.60E-128	-0.00145533	0.0747984	0.9844767	0.959109
ENSG00000166747	AP1G1	16	71803008	T	C	0.296223	-0.0162786	0.0156348	0.3100002	-0.0988908	0.00893	1.68E-28	0.164612	0.158799	0.2999196	0.644414
ENSG00000166750	SLFN5	17	33585364	C	A	0.400596	-0.00115816	0.0145432	0.83	0.577808	0.00751532	0	-0.0020044	0.0251696	0.9365269	0.8311016
ENSG00000166762	CATSPER2	15	43940508	C	A	0.0954274	0.0304517	0.0243137	0.2700001	-0.518541	0.0248363	8.42E-97	-0.0587257	0.0469729	0.2112255	0.4408578
ENSG00000166763	NA	15	44001034	A	G	0.282306	0.00388779	0.015905	0.8800001	0.638301	0.0193944	1.50E-237	0.00609085	0.0249184	0.8068967	0.5925401
ENSG00000166770	ZNF667-AS1	19	57000327	C	T	0.241551	0.018154	0.0161165	0.1499999	-0.294326	0.0171205	3.08E-66	-0.0616799	0.0548747	0.2610075	0.6218521
ENSG00000166780	BMERB1	16	15623518	A	G	0.239563	0.0285894	0.0170763	0.1100001	-0.176954	0.00965031	4.22E-75	-0.161564	0.0969028	0.09545876	0.002040172
ENSG00000166783	MARF1	16	15712633	G	A	0.462227	-0.0117803	0.0142137	0.16	-0.0790818	0.00794936	2.57E-23	0.148963	0.180357	0.4088395	0.4658381
ENSG00000166788	SAAL1	11	18109560	T	C	0.354871	0.0179075	0.0148873	0.1499999	-0.404005	0.0119903	7.03E-249	-0.0443249	0.0368728	0.2293228	0.9877185
ENSG00000166793	YPEL4	11	57414988	C	G	0.399602	-0.00396125	0.0143892	0.6600001	-0.0915614	0.00817264	3.92E-29	0.0432633	0.157201	0.7831547	0.3135926
ENSG00000166794	PPIB	15	64451707	C	G	0.200795	-0.00464332	0.0172456	0.58	0.0517438	0.00949115	4.99E-08	-0.0897367	0.333694	0.7879921	0.9457875
ENSG00000166796	LDHC	11	18453729	A	G	0.115308	-0.00752022	0.0223801	0.3900004	0.844938	0.0111231	0	-0.00890032	0.0264875	0.7368563	0.7910588
ENSG00000166797	CIAO2A	15	64375487	T	C	0.185885	0.0142769	0.0176545	0.4799997	-0.164654	0.010155	4.02E-59	-0.0867087	0.107355	0.4192752	0.60692
ENSG00000166800	LDHAL6A	11	18489259	G	T	0.126243	0.0195011	0.021233	0.3800004	0.0850932	0.012524	1.09E-11	0.229173	0.251796	0.3627403	0.7723344

ENSG00000166801	FAM111A	11	58916366	T	C	0.0795229	-0.00165034	0.0253358	0.8600001	0.295937	0.0149184	1.43E-87	-0.00557666	0.0856126	0.9480639	0.9066932
ENSG00000166803	PCLAF	15	64668539	C	T	0.0646123	-0.0245282	0.0295851	0.32	0.245971	0.0164287	1.12E-50	-0.0997199	0.120463	0.4077812	0.2201417
ENSG00000166816	LDHD	16	75148213	G	A	0.217694	-0.0153204	0.0168725	0.3800004	-0.110912	0.00969392	2.60E-30	0.138131	0.152603	0.3653785	0.1399263
ENSG00000166819	PLIN1	15	90215127	A	G	0.2167	-0.0113742	0.0174425	0.4799997	0.13102	0.0101942	8.34E-38	-0.0868127	0.1333	0.5148792	0.9505849
ENSG00000166821	PEX11A	15	90227504	A	G	0.2167	-0.0120126	0.0173854	0.4600002	0.303649	0.0099355	3.92E-205	-0.0395608	0.0572696	0.4897022	0.9785928
ENSG00000166822	TMEM170A	16	75488173	C	T	0.0864811	-0.0140445	0.0301203	0.6700003	0.425895	0.0167451	1.06E-142	-0.0329764	0.0707342	0.641071	0.3736443
ENSG00000166823	MESP1	15	90293216	A	C	0.392644	-0.00992498	0.014843	0.56	-0.259824	0.00817256	8.27E-222	0.0381988	0.0571397	0.5038042	0.972875
ENSG00000166825	ANPEP	15	90343376	C	A	0.446322	0.00116767	0.0143004	0.8200001	-0.743474	0.00684753	0	-0.00157056	0.0192346	0.9349227	0.3635527
ENSG00000166831	RBPMS2	15	65049938	A	T	0.0387674	0.00470433	0.0329079	0.7899998	1.37883	0.0149044	0	0.00341182	0.0238665	0.8863263	0.2006331
ENSG00000166833	NAV2	11	19757707	C	G	0.374751	0.020725	0.0145824	0.1800002	-0.156842	0.00809282	1.13E-83	-0.132139	0.0932248	0.1563579	0.9664819
ENSG00000166839	ANKDD1A	15	65227571	T	C	0.4334	0.0313635	0.0146076	0.032	0.907194	0.00653253	0	0.034572	0.0161039	0.03180835	0.6509486
ENSG00000166845	C18orf54	18	51897937	A	G	0.0656064	-0.00478833	0.0301754	0.9	-0.390684	0.0176905	4.46E-108	0.0122563	0.0772393	0.8739217	0.04617502
ENSG00000166847	DCTN5	16	23666954	A	G	0.213718	0.0347916	0.0175199	0.03699985	1.01661	0.00759671	0	0.0342233	0.0172356	0.04707613	0.0640753
ENSG00000166851	PLK1	16	23695332	G	A	0.21173	0.0347215	0.0175067	0.03500016	0.197516	0.00956227	8.66E-95	0.175791	0.0890418	0.04835372	0.06285151
ENSG00000166855	CLPX	15	65459118	A	G	0.44334	-0.020174	0.014259	0.16	-0.0661535	0.00798512	1.19E-16	0.304957	0.218665	0.1631261	0.3916164
ENSG00000166860	ZBTB39	12	57396424	A	G	0.120278	-0.0208828	0.0246618	0.3100002	-0.0908036	0.0130735	3.77E-12	0.229978	0.273606	0.4006037	0.4274497
ENSG00000166863	TAC3	12	57413225	T	C	0.0417495	-0.00485852	0.0359679	0.8600001	0.178251	0.0226886	3.95E-15	-0.0272567	0.201813	0.8925649	0.3677465
ENSG00000166881	NEMP1	12	57465636	A	G	0.0159046	0.0142096	0.0484184	0.6700003	1.22255	0.0364299	6.63E-247	0.0116229	0.0396058	0.7691675	0.6495811
ENSG00000166886	NAB2	12	57485968	C	T	0.0675944	-0.0141081	0.031818	0.6100002	-0.100369	0.0171383	4.73E-09	0.140563	0.317918	0.6583918	0.2048251
ENSG00000166887	VPS39	15	42475706	C	T	0.133201	-0.00819614	0.0206626	0.6700003	0.0723673	0.0124287	5.79E-09	-0.113257	0.286186	0.6922908	0.9978766
ENSG00000166888	STAT6	12	57507556	C	T	0.388668	-0.0272186	0.0149695	0.0329997	0.508228	0.00761049	0	-0.0535559	0.0294652	0.06912577	0.9953807
ENSG00000166889	PATL1	11	59420321	T	C	0.0457256	0.010372	0.0344736	0.7800007	-0.239813	0.0201311	1.02E-32	-0.0432503	0.143798	0.7635884	0.3148579
ENSG00000166896	ATP23	12	58343188	T	A	0.313121	-0.0245946	0.0151028	0.09699961	-0.705652	0.0111188	0	0.0348537	0.0214097	0.1035368	0.1543634
ENSG00000166897	ELFN2	22	37793752	A	G	0.0785288	-0.0162006	0.0281604	0.4	0.0987354	0.0174075	1.41E-08	-0.164081	0.286674	0.5670777	0.7339527
ENSG00000166900	STX3	11	59527141	A	G	0.0457256	0.0104607	0.0345027	0.7700005	-0.414493	0.018472	1.64E-111	-0.0252373	0.0832483	0.76177	0.0857583
ENSG00000166902	MRPL16	11	59575976	T	G	0.084493	-0.0181646	0.0267421	0.4199997	-0.109791	0.0157819	3.48E-12	0.165447	0.244731	0.4990176	0.2073088
ENSG00000166908	PIP4K2C	12	57991077	G	A	0.426441	0.00809159	0.014506	0.7499995	0.121873	0.00804229	7.13E-52	0.0663937	0.119106	0.5772321	0.04247075
ENSG00000166912	MTMR10	15	31257477	T	G	0.187873	-0.0208693	0.0194749	0.2300001	0.39933	0.011418	5.63E-268	-0.0522608	0.0487918	0.2841257	0.05628773
ENSG00000166913	YWHAB	20	43525745	A	G	0.249503	0.00550792	0.0171522	0.91	0.125833	0.0092527	4.03E-42	0.0437717	0.136347	0.748187	0.3266817
ENSG00000166922	SCG5	15	32961588	G	T	0.253479	-0.000940969	0.0170929	0.8600001	-0.115733	0.0100122	6.63E-31	0.00813052	0.147694	0.9560989	0.7706442
ENSG00000166925	TSC22D4	7	100068942	G	T	0.197813	-0.0194439	0.0178473	0.2300001	-0.143474	0.00992199	2.16E-47	0.135522	0.124747	0.2773109	0.3702758
ENSG00000166927	MS4A7	11	60154689	T	C	0.349901	0.00214534	0.0146945	0.99	0.232837	0.00820249	2.99E-177	0.00921389	0.0631114	0.883926	0.2434185
ENSG00000166928	MS4A14	11	60165582	T	C	0.270378	0.00539624	0.0157977	0.8200001	-0.696657	0.00884347	0	-0.00774591	0.0226767	0.7326665	0.2287439
ENSG00000166938	DIS3L	15	66605895	G	A	0.0467197	0.00998735	0.030599	0.89	0.836702	0.015562	0	0.0119366	0.0365716	0.7441303	0.3896853
ENSG00000166946	CCNDBP1	15	43482356	C	T	0.0685885	-0.0336581	0.0280458	0.1900002	-0.118379	0.0181882	7.59E-11	0.284324	0.240908	0.2379141	0.2752134
ENSG00000166949	SMAD3	15	67421817	A	C	0.214712	-0.0264271	0.0169981	0.07299952	0.169724	0.00996477	4.72E-65	-0.155706	0.100568	0.1215567	0.3518662
ENSG00000166963	MAP1A	15	43813487	G	A	0.296223	0.0134418	0.0155423	0.6200004	0.154458	0.0086534	2.92E-71	0.0870256	0.100743	0.3876762	0.07549763
ENSG00000166965	RCCD1	15	91502224	T	G	0.140159	-0.0343174	0.0211661	0.07799917	0.796828	0.011244	0	-0.0430675	0.0265699	0.1050361	0.6359401
ENSG00000166971	AKTIP	16	53531807	C	T	0.460239	0.00403956	0.0142129	0.83	0.464417	0.0112497	0	0.00869814	0.0306045	0.7762486	0.2757833
ENSG00000166974	MAPRE2	18	32640163	C	T	0.139165	-0.00976277	0.0210754	0.6300007	0.356081	0.0113302	8.49E-217	-0.0274173	0.0591936	0.6432352	0.5994902
ENSG00000166979	EVA1C	21	33836010	C	T	0.116302	0.0136379	0.0222164	0.5	-0.560808	0.0136718	0	-0.0243183	0.0396194	0.5393491	0.09167304
ENSG00000166986	MARS1	12	57890290	G	A	0.358847	-0.00338823	0.0147705	0.81	0.153776	0.0122396	3.34E-36	-0.0220335	0.0960679	0.8185942	0.4611285
ENSG00000166987	MBD6	12	57919212	T	C	0.17495	0.0135006	0.0207313	0.5	0.110478	0.0118407	1.05E-20	0.122202	0.188107	0.5159262	0.5635893
ENSG00000166997	CNPY4	7	99720185	C	T	0.27833	0.049263	0.0159242	0.002100003	-0.302547	0.00877516	1.73E-260	-0.162827	0.0528452	0.002061578	0.2998042
ENSG00000167004	PDIA3	15	44052033	C	A	0.282306	0.00326988	0.0159049	0.8499999	-0.117301	0.00967638	8.04E-34	-0.027876	0.13561	0.8371347	0.6756542
ENSG00000167005	NUDT21	16	56474578	A	G	0.373757	-0.00200687	0.0144271	0.8700001	0.103071	0.00813157	8.09E-37	-0.0194708	0.139981	0.8893744	0.02938801
ENSG00000167034	NKX3-1	8	23538323	A	G	0.265408	0.0100966	0.0154664	0.3400001	0.870924	0.00764508	0	0.011593	0.0177589	0.5138865	0.9598647
ENSG00000167037	SGSM1	22	25262890	T	C	0.104374	0.0217463	0.0223082	0.25	0.358937	0.0142045	6.97E-141	0.0605852	0.0621969	0.3300131	0.4760747
ENSG00000167065	DUSP18	22	31055957	T	C	0.297217	0.0159156	0.0156424	0.2300001	-0.714442	0.00793473	0	-0.022277	0.021896	0.308963	0.2523382
ENSG00000167074	TEF	22	41779333	C	G	0.233598	0.0502735	0.0170248	0.00329997	0.536022	0.00916291	0	0.09379	0.0318018	0.003185958	0.4442417

ENSG00000167077	MEI1	22	42145481	G	A	0.194831	0.0356179	0.0184166	0.06699926	0.551673	0.00986126	0	0.0645635	0.0334031	0.05325395	0.005528761
ENSG00000167081	PBX3	9	128619640	A	G	0.363817	-0.0057098	0.0147089	0.7400005	-0.0850254	0.00841886	5.56E-24	0.0671541	0.173122	0.6980898	0.6975778
ENSG00000167083	GNGT2	17	47284044	T	C	0.335984	-0.0124193	0.0153152	0.4500005	0.390487	0.00821294	0	-0.0318047	0.0392265	0.4174832	0.7407309
ENSG00000167085	PHB1	17	47486830	A	G	0.212724	0.0138337	0.0184854	0.5199996	-0.126823	0.0102472	3.51E-35	-0.109079	0.146024	0.4550673	0.8418501
ENSG00000167094	TTC16	9	130486112	A	G	0.494036	-0.0239335	0.0143097	0.04700023	0.274367	0.00781681	6.84E-270	-0.0872317	0.0522145	0.09479257	0.580868
ENSG00000167100	SAMD14	17	48197325	C	T	0.306163	-0.0102304	0.015298	0.4500005	-0.107728	0.00958365	2.57E-29	0.0949652	0.142257	0.5044141	0.3036997
ENSG00000167103	PIP5KL1	9	130688117	A	G	0.127237	-0.012594	0.0213705	0.4400003	0.168215	0.0115723	7.17E-48	-0.0748686	0.127147	0.5559731	0.6234999
ENSG00000167105	TMEM92	17	48353805	A	G	0.298211	-0.00287259	0.0156998	0.84	-0.255661	0.00869262	3.94E-190	0.0112359	0.0614098	0.8548245	0.8370187
ENSG00000167106	EEIG1	9	130722825	G	T	0.128231	-0.00976729	0.0213154	0.5199996	0.257997	0.0111299	7.15E-119	-0.0378582	0.082635	0.6468537	0.4285737
ENSG00000167107	ACSF2	17	48527862	C	G	0.444334	-0.0184268	0.0142181	0.1800002	0.171839	0.00790596	9.50E-105	-0.107233	0.0828876	0.1957648	0.03561703
ENSG00000167113	COQ4	9	131090583	T	C	0.0357853	0.0916646	0.0452378	0.0519996	0.156909	0.0279535	1.99E-08	0.584191	0.306516	0.05666251	0.9416899
ENSG00000167114	SLC27A4	9	131113213	C	A	0.0417495	0.0832507	0.0403052	0.05399953	0.177433	0.0224122	2.44E-15	0.469195	0.234761	0.04565109	0.6733206
ENSG00000167118	URM1	9	131143306	C	T	0.0447316	0.0567321	0.0338277	0.16	-0.478436	0.017097	2.57E-172	-0.118578	0.0708316	0.09411363	0.8664122
ENSG00000167136	ENDOG	9	131582854	T	A	0.296223	-0.00044928	0.0156711	0.91	-0.527527	0.00826346	0	0.000851672	0.0297067	0.9771283	0.5752616
ENSG00000167173	C15orf39	15	75496247	G	A	0.082505	-0.0046621	0.0254705	0.84	-0.123255	0.0141471	2.98E-18	0.0378248	0.206694	0.8547989	0.4618536
ENSG00000167182	SP2	17	45989919	T	C	0.209742	-0.0186984	0.0178311	0.16	0.0817296	0.0100358	3.83E-16	-0.228784	0.219973	0.2983153	0.468755
ENSG00000167183	PRR15L	17	46032288	G	A	0.0666004	0.00733437	0.0279584	0.95	0.0879455	0.0153039	9.11E-09	0.0833968	0.318237	0.7932762	0.5847173
ENSG00000167186	COQ7	16	19085169	C	T	0.377734	0.00781397	0.0145337	0.7400005	0.228832	0.00798393	1.15E-180	0.0341471	0.0635236	0.5908875	0.3246573
ENSG00000167191	GPRC5B	16	19883052	A	G	0.0467197	0.034959	0.0386706	0.4299995	0.215269	0.0249477	6.20E-18	0.162397	0.180622	0.3685998	0.1988559
ENSG00000167193	CRK	17	1345219	C	G	0.0914513	-0.017252	0.0235197	0.4	-0.224868	0.0138955	6.67E-59	0.0767204	0.104701	0.4637046	0.0561419
ENSG00000167196	FBXO22	15	76211904	T	C	0.0149105	-0.0240626	0.0454846	0.5999997	-0.29792	0.0262295	6.75E-30	0.0807686	0.152839	0.5971839	0.09908784
ENSG00000167202	TBC1D2B	15	78323222	A	G	0.215706	-0.00225567	0.01725	0.8700001	-0.262609	0.0105303	2.85E-137	0.00858946	0.0656879	0.8959638	0.22253
ENSG00000167207	NOD2	16	50747251	C	T	0.260437	0.0408494	0.0157532	0.001499996	0.839151	0.00762101	0	0.0486794	0.018778	0.0095318	0.6518639
ENSG00000167208	SNX20	16	50707737	C	T	0.00596421	-0.122859	0.104805	0.1800002	-0.287417	0.0443011	8.71E-11	0.42746	0.37055	0.2486712	0.911408
ENSG00000167210	LOXHD1	18	44146965	T	C	0.451292	0.0063303	0.0142492	0.9199999	-0.497836	0.00824302	0	-0.0127156	0.028623	0.6568658	0.09905705
ENSG00000167216	KATNAL2	18	44562556	G	T	0.0775348	0.0512836	0.0266322	0.04099964	0.160566	0.0144454	1.06E-28	0.319393	0.168335	0.05778009	0.05410017
ENSG00000167220	HDHD2	18	44655332	G	A	0.0616302	0.0573847	0.0310171	0.05999983	0.576152	0.0161625	2.59E-278	0.0995999	0.0539073	0.06465848	0.2002043
ENSG00000167232	ZNF91	19	23533077	T	C	0.248509	0.0224261	0.0161472	0.1800002	0.441699	0.0103072	0	0.0507724	0.0365762	0.165099	0.577557
ENSG00000167236	NA	17	34342550	T	C	0.10338	0.038815	0.0236127	0.07399971	-0.278312	0.0129726	4.21E-102	-0.139466	0.0850913	0.1012102	0.8712781
ENSG00000167244	IGF2	11	2160587	T	C	0.322068	0.00620249	0.0158547	0.81	-0.0999	0.0097717	1.56E-24	-0.062087	0.158822	0.6958548	0.6140109
ENSG00000167257	RNF214	11	117130251	T	C	0.429423	-0.0156199	0.0142473	0.3400001	0.0966915	0.00799975	1.24E-33	-0.161544	0.147953	0.2748952	0.5840042
ENSG00000167258	CDK12	17	37669462	G	A	0.0854871	0.0402626	0.0241144	0.17	-0.477674	0.0155698	1.07E-206	-0.0842888	0.0505576	0.09547811	0.4285553
ENSG00000167261	DPEP2	16	68027893	G	A	0.50497	-0.0259996	0.014257	0.02199986	0.240164	0.00872761	1.08E-166	-0.108258	0.0594939	0.06881293	0.5735811
ENSG00000167264	DUS2	16	68067436	C	A	0.183897	-0.00966755	0.0185698	0.4	0.784482	0.0105154	0	-0.0123235	0.023672	0.6026501	0.4834036
ENSG00000167272	POP5	12	121017884	A	G	0.312127	-0.0177328	0.0158131	0.1299999	0.282464	0.00855206	3.15E-239	-0.0627791	0.0560151	0.2623928	0.8307585
ENSG00000167280	ENGASE	17	77077851	G	A	0.0576541	-0.0644529	0.0306543	0.01400006	-0.769241	0.0175391	0	0.0837877	0.0398958	0.03571469	0.7202722
ENSG00000167283	ATP5MG	11	118287040	C	T	0.0785288	-0.0216988	0.025312	0.4199997	0.268398	0.015332	1.29E-68	-0.0808455	0.0944206	0.391872	0.7331067
ENSG00000167286	CD3D	11	118211564	G	C	0.228628	-0.0036962	0.0161816	1	0.140015	0.00916378	1.05E-52	-0.0263986	0.115583	0.8193396	0.4775907
ENSG00000167291	TBC1D16	17	77957894	T	C	0.305169	0.00786962	0.0153529	0.5500004	0.313763	0.0096717	7.16E-231	0.0250814	0.0489375	0.6082885	0.3942331
ENSG00000167302	TEPSIN	17	79207484	T	C	0.460239	-0.000213986	0.0142632	0.81	0.201015	0.013025	9.82E-54	-0.00106453	0.070956	0.9880301	0.5023033
ENSG00000167306	MYO5B	18	47535323	C	T	0.276342	0.00533947	0.0159321	0.8200001	0.188905	0.00869762	1.35E-104	0.0282653	0.0843492	0.7375502	0.5264833
ENSG00000167315	ACAA2	18	47325099	G	C	0.219682	0.0442101	0.0179351	0.01899984	0.261817	0.0159252	9.83E-61	0.168859	0.0692681	0.01477877	0.4916596
ENSG00000167323	STIM1	11	3995098	G	T	0.33996	0.00819204	0.0155601	0.6600001	0.154667	0.00842983	3.45E-75	0.0529656	0.100645	0.5987071	0.6928184
ENSG00000167325	RRM1	11	4138021	A	C	0.2833	0.00383698	0.0160688	0.7199992	0.419691	0.00887886	0	0.00914239	0.0382877	0.8112751	0.3276681
ENSG00000167333	TRIM68	11	4624695	T	C	0.0606362	0.0161137	0.027763	0.5099998	-0.63228	0.0176497	4.71E-281	-0.0254851	0.0439151	0.5616949	0.3408299
ENSG00000167363	FN3K	17	80701262	T	C	0.348907	0.00314245	0.0152924	0.9400001	-0.324607	0.00851852	0	-0.00968079	0.0471112	0.8371909	0.5483819
ENSG00000167377	ZNF23	16	71489249	C	G	0.326044	-0.0107655	0.0151015	0.4100001	0.270616	0.00847856	1.52E-223	-0.0397814	0.0558181	0.4760324	0.6383789
ENSG00000167378	IRGQ	19	44094404	A	G	0.359841	0.00992923	0.0148042	0.5199996	-0.0587482	0.00908907	1.02E-10	-0.169013	0.253347	0.5046945	0.832439
ENSG00000167380	ZNF226	19	44675880	G	A	0.00596421	-0.040324	0.0676412	0.6999999	-0.891586	0.0833507	1.05E-26	0.0452273	0.0759839	0.5516952	NA
ENSG00000167383	NA	19	44937225	G	A	0.111332	-0.00756371	0.0249788	0.64	0.0903149	0.0145961	6.11E-10	-0.0837482	0.276906	0.7623142	0.97244

ENSG00000167394	ZNF668	16	31078902	C	T	0.400596	-0.0234743	0.0146013	0.05699936	0.34672	0.00796932	0	-0.067704	0.0421414	0.1081445	0.1580217
ENSG00000167395	ZNF646	16	31090630	T	G	0.374751	0.0147173	0.0148623	0.28	-0.109002	0.0149317	2.88E-13	-0.135019	0.137598	0.3264661	0.6506685
ENSG00000167397	VKORC1	16	31104732	G	T	0.399602	-0.0248037	0.0145979	0.04700023	0.118745	0.00980622	9.44E-34	-0.208881	0.124139	0.09244403	0.4099369
ENSG00000167414	GNG8	19	47137637	G	A	0.39165	-0.0198704	0.0144362	0.1800002	-0.312041	0.00799196	0	0.0636789	0.0462926	0.1689535	0.923423
ENSG00000167434	CA4	17	58237778	G	T	0.0367793	-0.0098278	0.0388233	0.58	-0.461674	0.0207239	6.12E-110	0.0212873	0.0840978	0.8001713	0.05391005
ENSG00000167447	SMG8	17	57289684	C	G	0.341948	0.00236327	0.015199	0.8700001	0.124391	0.00971271	1.50E-37	0.0189988	0.122197	0.8764452	0.6466564
ENSG00000167460	TPM4	19	16195822	T	C	0.0188867	-0.0522592	0.0527864	0.33	-0.854014	0.0411402	1.03E-95	0.0611924	0.06188	0.3227176	0.2437516
ENSG00000167461	RAB8A	19	16233741	G	T	0.151093	0.0245248	0.0204572	0.35	-0.51743	0.0113405	0	-0.0473973	0.0395498	0.2307531	0.6996036
ENSG00000167468	GPX4	19	1105361	G	A	0.447316	0.00140914	0.0144741	0.9	0.55144	0.00860825	0	0.00255538	0.0262479	0.9224438	0.3188459
ENSG00000167470	MIDN	19	1253847	T	C	0.347913	-0.026366	0.0162109	0.1800002	-0.131955	0.0135852	2.65E-22	0.19981	0.124562	0.1086911	0.5704723
ENSG00000167476	JSRP1	19	2261005	G	A	0.316103	0.0271178	0.0149078	0.1	0.122703	0.0123275	2.43E-23	0.221004	0.123507	0.07355066	0.9582351
ENSG00000167483	NIBAN3	19	17649379	A	G	0.262425	0.0106738	0.0165337	0.4899999	-0.128143	0.00976357	2.38E-39	-0.083296	0.129181	0.5190575	0.1261696
ENSG00000167487	KLHL26	19	18764542	G	A	0.375746	0.0102026	0.0146192	0.5099998	0.0846219	0.00939512	2.12E-19	0.120567	0.173277	0.486551	0.2306159
ENSG00000167491	GATAD2A	19	19558189	A	G	0.327038	-0.0151743	0.0148857	0.2700001	0.484772	0.00793692	0	-0.0313019	0.0307109	0.3080863	0.1496487
ENSG00000167508	MVD	16	88723956	G	A	0.188867	0.0308624	0.0182045	0.16	-0.225725	0.0120969	1.05E-77	-0.136725	0.0809811	0.09134154	0.263157
ENSG00000167513	CDT1	16	88872643	T	C	0.338966	0.0313237	0.0153031	0.03099988	-0.0587052	0.00985814	2.60E-09	-0.533576	0.275646	0.05290113	0.2919701
ENSG00000167515	TRAPPC2L	16	88925861	C	T	0.34493	-0.0180209	0.0148847	0.2999998	-0.273692	0.00955754	2.38E-180	0.0658438	0.0544335	0.2264251	0.6182525
ENSG00000167522	ANKRD11	16	89445503	T	A	0.455268	-0.00396633	0.0141939	1	-0.0474686	0.0080086	3.08E-09	0.0835569	0.299349	0.7801458	0.5286923
ENSG00000167523	SPATA33	16	89730945	G	T	0.485089	0.0231928	0.0143237	0.07799917	0.125989	0.008715	2.28E-47	0.184085	0.114401	0.1075882	0.5707733
ENSG00000167525	PROCA1	17	27034543	T	C	0.0725646	0.00021545	0.0251405	0.7499995	-0.432342	0.0134634	2.96E-226	-0.000498332	0.0581496	0.9931623	0.6889647
ENSG00000167526	RPL13	16	89629007	T	C	0.166998	-0.000445454	0.0177946	0.84	0.720179	0.0117891	0	-0.000618533	0.0247086	0.9800286	0.01877055
ENSG00000167528	ZNF641	12	48739494	A	G	0.210736	0.0247059	0.0169992	0.1299999	-0.903993	0.00776565	0	-0.0273297	0.018806	0.1461562	0.5105619
ENSG00000167535	CACNB3	12	49215150	T	C	0.177932	0.034004	0.0182388	0.02	0.485158	0.0100849	0	0.0700886	0.0376218	0.06246529	0.02236872
ENSG00000167536	DHRS13	17	27227444	A	G	0.166004	-0.0166922	0.0187169	0.4600002	-0.23088	0.0106217	9.25E-105	0.0722983	0.081136	0.3728887	0.2115216
ENSG00000167543	TP53I13	17	27896622	T	C	0.310139	0.00142904	0.0154622	0.83	-0.161058	0.00898307	7.00E-72	-0.00887285	0.0960055	0.9263641	0.08463895
ENSG00000167549	CORO6	17	27945849	T	G	0.525845	0.0192856	0.0141857	0.05899973	0.130631	0.00791154	3.04E-61	0.147635	0.108961	0.1754415	0.329882
ENSG00000167550	RHEBL1	12	49461138	A	G	0.305169	-0.0213953	0.0154871	0.16	0.0532107	0.00840558	2.45E-10	-0.402087	0.297903	0.1771041	0.0759122
ENSG00000167552	TUBA1A	12	49580843	C	T	0.027833	0.0483983	0.0421834	0.1299999	0.499298	0.0466843	1.07E-26	0.0969327	0.0849702	0.2539593	0.4032157
ENSG00000167553	TUBA1C	12	49624816	C	T	0.343936	-0.0296653	0.0149006	0.06800017	-0.33618	0.010052	3.14E-245	0.0882422	0.0444017	0.04688236	0.9481462
ENSG00000167554	ZNF610	19	52855264	T	C	0.0377734	-0.0313471	0.0353189	0.4299995	0.166267	0.0212216	4.70E-15	-0.188535	0.213782	0.3778287	0.7215217
ENSG00000167555	ZNF528	19	52911383	C	T	0.238569	0.00304389	0.0173365	0.89	-0.245838	0.00936334	6.20E-152	-0.0123817	0.0705215	0.8606294	0.9539324
ENSG00000167562	ZNF701	19	53074751	G	A	0.234592	0.02348	0.0166402	0.07100027	-0.276098	0.010216	7.30E-161	-0.0850422	0.0603512	0.1587993	0.6988582
ENSG00000167565	SERTAD3	19	40948681	C	T	0.333002	0.0178192	0.0150373	0.2099999	-0.501603	0.00813605	0	-0.0355245	0.029984	0.236104	0.576027
ENSG00000167566	NCKAP5L	12	50203731	C	T	0.32505	0.0105801	0.0151293	0.5500004	-0.0569866	0.00823625	4.55E-12	-0.185659	0.266841	0.4865746	0.5404416
ENSG00000167578	RAB4B	19	41293484	C	T	0.333996	0.00981857	0.0149586	0.3900004	0.22262	0.00834958	1.29E-156	0.0441047	0.0672139	0.5117057	0.7283527
ENSG00000167595	PROSER3	19	36255487	G	A	0.115308	-0.0269786	0.0249929	0.1900002	0.0991456	0.013316	9.65E-14	-0.272111	0.254718	0.2853933	0.01488644
ENSG00000167600	CYP2S1	19	41706199	G	A	0.305169	0.0263999	0.015542	0.09200046	-0.502011	0.00898024	0	-0.0525882	0.0309737	0.08953928	0.02433416
ENSG00000167601	AXL	19	41746389	A	G	0.43837	0.0371049	0.014352	0.0064	0.132452	0.0079709	5.25E-62	0.280137	0.109659	0.0106306	0.2984002
ENSG00000167608	TMC4	19	54670395	C	T	0.447316	-0.0137054	0.0143436	0.4	-0.535639	0.00853893	0	0.025587	0.0267816	0.3393777	0.02482722
ENSG00000167613	LAIR1	19	54873713	A	C	0.159046	-0.00157477	0.0218481	0.99	-0.398247	0.019291	1.10E-94	0.00395426	0.054861	0.9425401	0.1481545
ENSG00000167618	LAIR2	19	55015498	T	C	0.138171	-0.0016175	0.0216927	0.9199999	-0.598289	0.019205	4.66E-213	0.00270355	0.036258	0.9405616	0.4021593
ENSG00000167632	TRAPPC9	8	141105632	C	T	0.519881	0.00391529	0.0142094	0.83	-0.29111	0.00778927	1.06E-305	-0.0134495	0.0488124	0.7829054	0.8264708
ENSG00000167633	KIR3DL1	19	55307208	A	G	0.210736	0.0343147	0.018978	0.08400014	0.402273	0.0162804	8.56E-135	0.0853021	0.0473031	0.07133975	0.6234582
ENSG00000167634	NLRP7	19	55456278	C	T	0.483101	-0.0170398	0.0141809	0.3800004	0.0611339	0.00798552	1.92E-14	-0.278729	0.234804	0.2352004	0.2954977
ENSG00000167635	ZNF146	19	36717590	A	G	0.382704	-0.000333394	0.0147765	0.9400001	-0.0847588	0.00836876	4.15E-24	0.00393344	0.174336	0.9819993	0.9449245
ENSG00000167637	ZNF283	19	44342375	A	G	0.22664	0.00182154	0.0175199	0.7700005	-0.087047	0.0108792	1.23E-15	-0.0209259	0.201286	0.9172003	0.3854303
ENSG00000167641	PPP1R14A	19	38744554	G	A	0.33996	-0.0167043	0.0156552	0.2300001	-0.258614	0.00850917	6.92E-203	0.0645916	0.0605723	0.2862628	0.2662232
ENSG00000167642	SPINT2	19	38758964	A	C	0.209742	0.0249796	0.0162672	0.17	-0.282847	0.00919724	1.10E-207	-0.0883148	0.057584	0.1251108	0.2238624
ENSG00000167645	YIF1B	19	38801833	A	G	0.182903	-0.0257561	0.0199962	0.17	0.517943	0.0108046	0	-0.0497277	0.0386209	0.1978905	0.3643173
ENSG00000167657	DAPK3	19	3964786	C	T	0.44334	-0.00196112	0.0145477	0.7800007	0.108804	0.00934165	2.37E-31	-0.0180243	0.133714	0.8927723	0.5063927

ENSG00000167658	EEF2	19	3980760	T	C	0.0238569	0.0354267	0.0432861	0.3599996	-0.481429	0.0334414	5.47E-47	-0.0735865	0.0900569	0.4138645	0.2503367
ENSG00000167664	TMIGD2	19	4297328	G	T	0.402584	-0.00296893	0.0146849	0.81	-0.141899	0.00942587	3.24E-51	0.0209228	0.103498	0.8397936	0.4061162
ENSG00000167670	CHAF1A	19	4423837	G	A	0.265408	0.0105869	0.0158344	0.4299995	-0.300714	0.00908153	1.95E-240	-0.0352059	0.0526667	0.5038368	0.7027255
ENSG00000167671	UBXN6	19	4451407	T	C	0.429423	-0.0101397	0.0143039	0.4500005	0.179073	0.00794546	1.77E-112	-0.0566232	0.0799169	0.4786185	0.314911
ENSG00000167674	HDGFL2	19	4487253	C	T	0.526839	0.0213925	0.0141781	0.16	0.0858588	0.00853365	8.20E-24	0.249159	0.166979	0.1356585	0.7161686
ENSG00000167676	PLIN4	19	4509960	C	T	0.0347913	0.0349599	0.0325007	0.1900002	0.364195	0.0335614	1.96E-27	0.0959923	0.0896772	0.2844303	0.4939211
ENSG00000167680	SEMA6B	19	4551210	G	A	0.0506958	0.0337884	0.0326724	0.3599996	0.162419	0.0235712	5.56E-12	0.208032	0.203414	0.3064481	0.5478637
ENSG00000167685	ZNF444	19	56658115	G	A	0.00994036	0.0332555	0.0665132	0.5199996	-0.59359	0.108768	4.83E-08	-0.0560244	0.112522	0.6185557	0.4021388
ENSG00000167693	NXN	17	792781	T	C	0.378728	-0.0252695	0.0149286	0.04799986	-0.0989244	0.00984537	9.39E-24	0.255443	0.153036	0.09508356	0.8981598
ENSG00000167695	TLCD3A	17	640929	C	T	0.49006	0.00855491	0.0142109	0.7800007	-0.0830671	0.00793185	1.15E-25	-0.102988	0.17136	0.5478372	0.5866395
ENSG00000167699	GLOD4	17	672959	A	T	0.201789	0.0395232	0.0176217	0.0179999	-0.211214	0.00966877	8.67E-106	-0.187124	0.083869	0.02567173	0.3319449
ENSG00000167700	MFSD3	8	145735526	T	G	0.027833	0.0548266	0.0441156	0.1	-0.517624	0.0411714	2.99E-36	-0.10592	0.0856426	0.2161739	0.1417767
ENSG00000167702	KIFC2	8	145695505	T	C	0.0715706	-0.0194133	0.0256435	0.5199996	-0.572153	0.0262537	2.69E-105	0.0339303	0.0448464	0.449296	0.9390792
ENSG00000167703	SLC43A2	17	1504926	A	G	0.182903	0.0154998	0.0176979	0.2999998	-0.112212	0.011615	4.42E-22	-0.138129	0.158365	0.3830872	0.08584771
ENSG00000167711	SERPINF2	17	1652346	A	G	0.225646	-0.021936	0.0167525	0.2300001	0.512071	0.0121174	0	-0.0428378	0.0327309	0.190606	0.2746623
ENSG00000167716	WDR81	17	1630855	A	G	0.33996	-0.00996899	0.0149377	0.6300007	0.365909	0.00807828	0	-0.0272444	0.040828	0.5045813	0.9613022
ENSG00000167720	SRR	17	2217615	T	C	0.34493	-0.00997449	0.0149114	0.7099994	-0.574418	0.00868981	0	0.0173645	0.0259605	0.5035702	0.3866571
ENSG00000167723	TRPV3	17	3437542	T	A	0.129225	-0.00693281	0.0218417	0.64	-0.073433	0.0115971	2.42E-10	0.09441	0.297811	0.7512333	0.5962247
ENSG00000167740	CYB5D2	17	4068533	C	A	0.22664	0.00827524	0.017472	0.8499999	0.359725	0.00958013	1.457286e-308	0.0230043	0.0485743	0.6357916	0.7010759
ENSG00000167747	C19orf48P	19	51304573	G	A	0.301193	0.0148517	0.0157549	0.32	0.416323	0.00896791	0	0.0356735	0.0378507	0.3459482	0.8355999
ENSG00000167748	KLK1	19	51324723	C	A	0.0974155	-0.00499333	0.0236178	0.83	0.248746	0.0124877	2.77E-88	-0.020074	0.094953	0.832567	0.762884
ENSG00000167765	NA	19	52096334	G	C	0.550696	-0.0264178	0.0146049	0.06699926	-0.341671	0.0456109	6.84E-14	0.0773193	0.043974	0.07869692	0.3439783
ENSG00000167766	ZNF83	19	53145531	A	G	0.311133	0.008875	0.0159477	0.4600002	-0.725468	0.00778614	0	-0.0122335	0.021983	0.5778714	0.4867945
ENSG00000167767	KRT80	12	52574282	G	T	0.171968	-0.0111747	0.0186816	0.3700002	-0.136377	0.0113864	4.68E-33	0.0819396	0.137155	0.550226	0.0927264
ENSG00000167768	KRT1	12	53071355	C	A	0.382704	0.0185001	0.0144277	0.2700001	0.771931	0.00742929	0	0.023966	0.0186918	0.1997848	0.9379614
ENSG00000167769	ACER1	19	6319896	C	T	0.393638	-0.00638642	0.0144752	0.5999997	-0.105802	0.0104635	4.91E-24	0.0603617	0.136944	0.6593737	0.621635
ENSG00000167770	OTUB1	11	63761304	C	A	0.0427435	-0.0240769	0.0386547	0.4799997	0.202298	0.0206477	1.15E-22	-0.119017	0.191464	0.5341942	0.250977
ENSG00000167775	CD320	19	8370125	C	A	0.0427435	0.0565885	0.0384865	0.1199999	-0.694422	0.0222137	1.58E-214	-0.0814901	0.0554836	0.1419079	0.8959403
ENSG00000167778	SPRYD3	12	53465796	C	T	0.175944	-0.00271658	0.0195957	0.6600001	0.189605	0.0102355	1.32E-76	-0.0143276	0.103353	0.8897445	0.6408514
ENSG00000167779	IGFBP6	12	53493674	G	A	0.0815109	0.0638409	0.0250801	0.01899984	0.170842	0.0141723	1.83E-33	0.373685	0.15004	0.01275422	0.972003
ENSG00000167780	SOAT2	12	53507812	G	A	0.197813	-0.00864371	0.0189086	0.4400003	-0.199884	0.00977342	5.79E-93	0.0432436	0.0946215	0.6476588	0.2755627
ENSG00000167785	ZNF558	19	8931692	G	C	0.297217	-0.000953568	0.0153949	1	-0.374602	0.0188504	7.06E-88	0.00254555	0.0410968	0.9506104	0.1652526
ENSG00000167797	CDK2AP2	11	67275044	G	A	0.0457256	-0.0166945	0.0451714	0.59	-0.144403	0.0207851	3.72E-12	0.115611	0.313258	0.7120835	0.2170055
ENSG00000167799	NUDT8	11	67396405	G	A	0.192843	0.00344869	0.0178196	0.8600001	0.0634996	0.0106715	2.67E-09	0.0543104	0.280774	0.8466212	0.1510568
ENSG00000167815	PRDX2	19	12910164	C	G	0.439364	-0.0046507	0.0144729	0.5700002	0.235476	0.00955092	3.27E-134	-0.0197502	0.0614675	0.7479746	0.5866576
ENSG00000167840	ZNF232	17	5017623	T	C	0.112326	0.0114461	0.0233661	0.6700003	-0.262027	0.0125285	3.96E-97	-0.043683	0.089199	0.6243286	0.6366738
ENSG00000167842	MIS12	17	5391869	G	A	0.163022	0.0314687	0.0214488	0.1299999	-0.387473	0.0106598	2.73E-289	-0.0812152	0.0554006	0.142659	0.3787508
ENSG00000167850	CD300C	17	72539764	G	C	0.082505	0.0406427	0.0242482	0.06699926	-0.969558	0.0114973	0	-0.0419188	0.0250145	0.09378108	0.6314935
ENSG00000167851	CD300A	17	72471744	A	G	0.0487078	0.0264289	0.032529	0.3800004	-0.841242	0.0208897	0	-0.0314165	0.0386757	0.4166157	0.4552734
ENSG00000167861	HID1	17	72958049	G	T	0.480119	-0.0128581	0.0143645	0.4400003	0.0823025	0.00895409	3.87E-20	-0.15623	0.175359	0.3729744	0.8636696
ENSG00000167862	MRPL58	17	73013060	A	G	0.0129225	-0.0599148	0.0788775	0.4100001	-0.476264	0.0386202	6.09E-35	0.125802	0.165931	0.4483575	0.9202502
ENSG00000167863	ATP5PD	17	73039019	C	G	0.142147	-0.0254784	0.019775	0.1800002	0.366879	0.0229317	1.30E-57	-0.0694463	0.0540751	0.1990521	0.8070272
ENSG00000167874	TMEM88	17	7758900	T	C	0.171968	0.00669047	0.0179436	0.5500004	-0.158786	0.0100011	9.17E-57	-0.0421352	0.113036	0.7093283	0.9981597
ENSG00000167880	EVPL	17	74012058	T	C	0.287276	0.00564235	0.0160866	0.6800001	0.177781	0.00930636	2.37E-81	0.0317376	0.0905006	0.7258214	0.1787189
ENSG00000167881	SRP68	17	74051959	T	C	0.380716	-0.0110162	0.0146616	0.3400001	-0.155818	0.00812408	5.46E-82	0.070699	0.0941664	0.4527801	0.4138055
ENSG00000167889	MGAT5B	17	74905506	T	C	0.0685885	-0.0236828	0.0293554	0.4100001	0.145251	0.0201732	6.01E-13	-0.163047	0.203365	0.4227009	0.1786383
ENSG00000167895	TMC8	17	76132950	G	C	0.314115	0.00357546	0.0155583	0.8499999	0.412662	0.0145775	2.74E-176	0.00866437	0.0377035	0.8182449	0.2377257
ENSG00000167900	TK1	17	76176737	G	C	0.0457256	-0.0159787	0.0307767	0.4299995	0.109747	0.0183579	2.26E-09	-0.145596	0.281489	0.6049922	0.8062268
ENSG00000167904	TMEM68	8	56647474	T	C	0.162028	0.0192501	0.0199295	0.4299995	-0.151418	0.0106378	5.64E-46	-0.127132	0.131922	0.3351996	0.2952327
ENSG00000167912	TOX-DT	8	60032752	G	T	0.167992	-0.0151999	0.0189636	0.4299995	0.343457	0.0127496	7.74E-160	-0.0442556	0.0552384	0.4230299	0.3050028

ENSG00000167914	GSDMA	17	38126622	C	A	0.0924453	0.0228972	0.0233174	0.3400001	0.763119	0.0128869	0	0.0300047	0.0305596	0.3261766	0.9534159
ENSG00000167920	KRT10-AS1	17	38983940	T	C	0.178926	-0.0368915	0.019745	0.07699987	0.415487	0.0105266	0	-0.0887909	0.0475757	0.06199839	0.8750062
ENSG00000167930	FAM234A	16	302243	A	C	0.17992	0.0194377	0.0183796	0.35	-0.321882	0.0102846	5.06E-215	-0.0603876	0.057133	0.2905271	0.1376695
ENSG00000167962	ZNF598	16	2053739	T	C	0.141153	-0.00559826	0.0213186	0.81	-0.31323	0.0145775	2.05E-102	0.0178727	0.0680656	0.7928742	0.8720845
ENSG00000167965	MLST8	16	2256833	G	C	0.489066	0.015617	0.0141845	0.33	0.349928	0.0102896	1.71E-253	0.0446292	0.0405567	0.2711518	0.7178035
ENSG00000167967	E4F1	16	2279655	T	C	0.0119284	-0.0966027	0.0691752	0.17	0.831394	0.108858	2.22E-14	-0.116194	0.0845834	0.1695293	0.888636
ENSG00000167968	DNASE1L2	16	2287264	T	C	0.0576541	-0.0198736	0.0322638	0.4500005	0.154329	0.0210115	2.06E-13	-0.128774	0.209792	0.539336	0.3208533
ENSG00000167969	ECI1	16	2295848	T	C	0.161034	-0.0268776	0.0192923	0.1199999	-0.568887	0.0267246	1.50E-100	0.047246	0.0339849	0.1644671	0.6787466
ENSG00000167972	ABCA3	16	2358314	A	G	0.127237	-0.0444093	0.0214228	0.02800013	-0.197374	0.015118	5.90E-39	0.225	0.109899	0.04062427	0.394733
ENSG00000167977	KCTD5	16	2745753	C	T	0.144135	0.032077	0.0209011	0.1100001	0.132299	0.0140132	3.69E-21	0.242459	0.160058	0.129818	0.9571373
ENSG00000167978	SRRM2	16	2812434	C	A	0.0149105	-0.0595582	0.0632382	0.32	0.247118	0.0263066	5.79E-21	-0.241011	0.257185	0.3487023	0.3825732
ENSG00000167981	ZNF597	16	3489823	G	C	0.445328	0.00486771	0.014345	0.7199992	-0.144947	0.00794179	2.02E-74	-0.0335826	0.0989841	0.7344042	0.4699244
ENSG00000167984	NLRC3	16	3608219	A	C	0.138171	0.0278474	0.0202832	0.1499999	-0.51675	0.0131698	0	-0.0538895	0.0392755	0.1700355	0.05398664
ENSG00000167985	SDHAF2	11	61206257	T	G	0.00596421	0.0649568	0.0718018	0.3599996	-0.892027	0.0748746	1.01E-32	-0.0728193	0.0807246	0.3670191	0.8665719
ENSG00000167987	VPS37C	11	60913408	G	A	0.447316	0.0169001	0.0141991	0.09299937	0.0801575	0.00800183	1.28E-23	0.210836	0.178386	0.2372414	0.389824
ENSG00000167992	VWCE	11	61044329	T	C	0.0178926	0.0241173	0.0390991	0.32	-0.393395	0.0291542	1.71E-41	-0.0613055	0.0994926	0.5377747	0.9707316
ENSG00000167994	RAB3IL1	11	61676257	T	C	0.0666004	0.0228023	0.0311578	0.3800004	0.553158	0.0172374	5.98E-226	0.041222	0.0563417	0.4643868	0.6733499
ENSG00000167995	BEST1	11	61725140	T	C	0.34493	0.00504301	0.0149399	0.84	-0.358468	0.00839252	0	-0.0140682	0.0416784	0.7357079	0.2650881
ENSG00000167996	FTH1	11	61731161	G	A	0.054672	-0.0112726	0.0315251	0.7800007	-0.54206	0.0177994	1.06E-203	0.0207958	0.0581619	0.7206795	0.08297726
ENSG00000168000	BSCL2	11	62467532	T	C	0.0159046	0.0833835	0.0465692	0.1199999	0.883529	0.0261437	2.33E-250	0.0943755	0.0527821	0.0737726	0.3670163
ENSG00000168002	POLR2G	11	62531599	A	G	0.0228628	0.0606391	0.0603217	0.33	-0.22943	0.0399858	9.59E-09	-0.264303	0.266924	0.3220862	0.3464736
ENSG00000168003	SLC3A2	11	62639935	G	A	0.243539	0.00120802	0.0166163	0.8499999	0.0593846	0.00961881	6.67E-10	0.0203423	0.279827	0.9420481	0.5551431
ENSG00000168004	PLAAT5	11	63243771	C	T	0.440358	0.013588	0.0144762	0.4899999	-0.108312	0.0121702	5.59E-19	-0.125452	0.134394	0.350579	0.8733312
ENSG00000168005	SPINDOC	11	63588025	A	G	0.0894632	0.00481769	0.0278723	0.8800001	0.285001	0.0179728	1.25E-56	0.0169041	0.097803	0.8627785	0.9064027
ENSG00000168010	ATG16L2	11	72540036	T	C	0.44831	0.00566547	0.0142271	0.6999999	-0.0951395	0.00867728	5.68E-28	-0.0595491	0.149638	0.6906638	0.863078
ENSG00000168014	C2CD3	11	73803009	C	T	0.469185	-0.0107963	0.0142438	0.3700002	-0.0592098	0.00798642	1.23E-13	0.18234	0.241819	0.4508286	0.4871633
ENSG00000168016	TRANK1	3	36927429	T	G	0.236581	0.0229696	0.0169024	0.1499999	-0.354977	0.010388	6.35E-256	-0.0647073	0.0476532	0.1745013	0.1836445
ENSG00000168026	TTC21A	3	39164773	C	T	0.125249	-0.0247107	0.0216638	0.2300001	0.298782	0.0135952	4.78E-107	-0.0827047	0.0726045	0.2546566	0.3557548
ENSG00000168028	RPSA	3	39451106	C	T	0.307157	0.00966316	0.0152003	0.4799997	0.128969	0.0101982	1.17E-36	0.0749263	0.118009	0.5254806	0.3634309
ENSG00000168036	CTNNB1	3	41268957	T	C	0.436382	-0.000763693	0.0142093	0.8800001	-0.265521	0.00786378	6.42E-250	0.0028762	0.0535148	0.9571376	0.7633905
ENSG00000168038	ULK4	3	41646006	C	T	0.191849	0.000894282	0.0192655	0.84	0.806728	0.0105665	0	0.00110853	0.023881	0.9629764	0.4099311
ENSG00000168040	FADD	11	70051382	T	C	0.364811	0.0144497	0.0145058	0.2999998	0.278461	0.00798869	3.35E-266	0.0518913	0.052114	0.3193833	0.9684705
ENSG00000168056	LTBP3	11	65316338	A	G	0.184891	0.022939	0.0178611	0.16	0.436735	0.0106054	0	0.0525238	0.0409168	0.1992556	0.6115856
ENSG00000168060	NAALADL1	11	64819155	C	A	0.37674	0.029392	0.014816	0.04900044	-0.224974	0.00843976	1.50E-156	-0.130646	0.0660385	0.04789145	0.8113975
ENSG00000168061	SAC3D1	11	64810336	G	T	0.082505	0.0458919	0.0255215	0.04	-0.428117	0.0147519	3.56E-185	-0.107195	0.0597277	0.07269761	0.6221259
ENSG00000168062	BATF2	11	64759966	T	C	0.150099	0.0233243	0.0193208	0.16	-0.0731613	0.0116096	2.94E-10	-0.318806	0.268887	0.2357595	0.2256629
ENSG00000168067	MAP4K2	11	64563501	T	G	0.0795229	-0.0119697	0.0254363	0.56	-0.104002	0.0132784	4.79E-15	0.115091	0.245017	0.638549	0.4204555
ENSG00000168070	MAJIN	11	64722273	A	G	0.247515	-0.00490122	0.0166105	0.6499995	-0.0651887	0.0111244	4.63E-09	0.0751851	0.255129	0.7682275	0.6267942
ENSG00000168071	CCDC88B	11	64116350	T	C	0.367793	-0.029135	0.0146041	0.02999991	-0.433019	0.00781231	0	0.0672834	0.033748	0.04618521	0.149943
ENSG00000168079	SCARA5	8	27788821	A	G	0.217694	-0.0106313	0.0172287	0.4	0.209164	0.00925375	4.03E-113	-0.0508275	0.0823999	0.5373407	0.8720693
ENSG00000168090	COPS6	7	99688200	C	T	0.0666004	-0.0967733	0.0346338	0.002900013	-0.838186	0.0183732	0	0.115456	0.0413974	0.005287756	0.9703494
ENSG00000168092	PAFAH1B2	11	117031296	G	A	0.130219	0.00687907	0.0199438	0.7899998	0.462319	0.0157097	2.34E-190	0.0148795	0.0431415	0.7301703	0.03884057
ENSG00000168096	ANKS3	16	4765446	G	A	0.420477	-0.0272962	0.0144273	0.04600023	0.0813611	0.00803503	4.24E-24	-0.335495	0.180393	0.06291434	0.1082092
ENSG00000168101	NUDT16L1	16	4744777	G	T	0.0208748	-0.00790617	0.0457959	0.8800001	-0.231561	0.0253233	6.01E-20	0.0341429	0.197805	0.8629592	0.2868725
ENSG00000168116	KIAA1586	6	56915685	A	C	0.241551	0.00324193	0.0171117	0.9299999	-0.201865	0.00941484	5.52E-102	-0.0160599	0.0847711	0.8497405	0.3521543
ENSG00000168118	RAB4A	1	229424231	C	T	0.33002	0.0250582	0.0149739	0.06299992	0.185059	0.00894377	4.14E-95	0.135406	0.0811783	0.09531333	0.3328563
ENSG00000168152	THAP9	4	83831479	G	A	0.243539	0.000424645	0.016273	0.7400005	0.0578875	0.00960596	1.68E-09	0.0073357	0.281117	0.9791817	0.4573534
ENSG00000168159	RNF187	1	228679114	A	G	0.0775348	-0.0355841	0.0228062	0.08600031	0.406528	0.0138742	1.01E-188	-0.0875318	0.0561794	0.1192156	0.5316452
ENSG00000168209	DDIT4	10	74034736	G	T	0.360835	-0.00513824	0.0153425	0.6200004	-0.0769881	0.00868372	7.60E-19	0.0667407	0.199426	0.7378788	0.6972282
ENSG00000168214	RBPJ	4	26300809	C	T	0.356859	-0.0148004	0.0149737	0.29	0.267441	0.00829869	7.41E-228	-0.0553408	0.0560151	0.3231714	0.4230341

ENSG00000168216	LMBRD1	6	70446348	G	T	0.432406	-0.00413268	0.0146527	0.6600001	0.185944	0.0203093	5.41E-20	-0.0222254	0.0788392	0.7780137	0.9603287
ENSG00000168229	PTGDR	14	52738936	A	C	0.293241	0.0310352	0.0159169	0.1	-0.515943	0.0081246	0	-0.0601524	0.0308646	0.05130636	0.141351
ENSG00000168234	TTC39C	18	21644155	A	G	0.239563	-0.00574122	0.0179899	0.6499995	0.373256	0.00923872	0	-0.0153814	0.0481987	0.7496311	0.3725513
ENSG00000168237	GLYCTK	3	52325188	G	A	0.518887	0.00472801	0.0141827	0.8700001	-0.385943	0.00764408	0	-0.0122505	0.036749	0.7388646	0.2883618
ENSG00000168246	UBTD2	5	171673859	C	T	0.0974155	-0.00679032	0.0223307	0.8	-0.107629	0.0127218	2.67E-17	0.06309	0.207612	0.7612164	0.5429025
ENSG00000168255	POLR2J3-UPK3BL2	7	102205628	G	C	0.147117	0.0194428	0.0201472	0.4600002	-0.427517	0.0244771	2.60E-68	-0.0454784	0.0471979	0.3352627	0.9953732
ENSG00000168256	NKIRAS2	17	40170529	A	G	0.0139165	0.0397496	0.115737	0.8499999	-0.815194	0.0441739	4.82E-76	-0.0487609	0.141999	0.7313064	0.5415091
ENSG00000168260	NA	14	50554865	A	G	0.450298	-0.00748074	0.0142378	0.6300007	-0.10352	0.00869056	1.03E-32	0.0722636	0.13767	0.5996502	0.278479
ENSG00000168264	IRF2BP2	1	234742643	T	G	0.166004	0.0151911	0.0177065	0.1299999	0.130954	0.0105387	1.89E-35	0.116003	0.135533	0.3920522	0.7656925
ENSG00000168268	NT5DC2	3	52563728	A	G	0.462227	-0.00209044	0.014288	0.7499995	-0.372058	0.00771619	0	0.00561859	0.0384028	0.8836793	0.1904728
ENSG00000168269	FOXI1	5	169534814	A	G	0.22664	0.0270145	0.0176394	0.1900002	0.0755071	0.00957921	3.21E-15	0.357774	0.237981	0.1327428	0.155161
ENSG00000168273	UQCC5	3	52590641	G	C	0.0576541	0.0256363	0.0326296	0.33	0.378272	0.025369	2.80E-50	0.0677722	0.0863794	0.4326953	0.5010033
ENSG00000168274	NA	6	26217438	T	C	0.157058	-0.00884102	0.0214863	0.5500004	-0.421109	0.0109625	0	0.0209946	0.051026	0.6807435	0.6754145
ENSG00000168275	COA6	1	234514498	G	C	0.50497	-0.00609141	0.0142531	0.5999997	-0.389541	0.00854317	0	0.0156374	0.0365911	0.6691203	0.05443224
ENSG00000168280	KIF5C	2	149758046	C	T	0.0228628	0.0441143	0.0541885	0.4400003	0.562169	0.033375	1.16E-63	0.0784716	0.0965044	0.4161375	0.8924122
ENSG00000168282	MGAT2	14	50088843	T	C	0.242545	-0.0186184	0.0168082	0.3100002	-0.165214	0.00985116	3.98E-63	0.112693	0.101958	0.2690343	0.6567716
ENSG00000168288	MMADHC	2	150435239	C	T	0.173956	-0.0184847	0.0168132	0.4299995	-0.0981101	0.00961148	1.83E-24	0.188408	0.172362	0.2743525	0.591111
ENSG00000168291	PDHB	3	58416470	A	G	0.345924	-0.0196949	0.0151241	0.2300001	0.288415	0.00813326	1.95E-275	-0.0682867	0.052474	0.1931413	0.4046634
ENSG00000168297	PXK	3	58365177	T	C	0.277336	0.0290701	0.0154991	0.05899973	0.310482	0.00928283	2.86E-245	0.093629	0.0499979	0.06111575	0.3338038
ENSG00000168298	H1-4	6	26156951	C	T	0.0109344	-0.0820341	0.0641803	0.1900002	0.309006	0.0385342	1.07E-15	-0.265477	0.210321	0.2068596	0.811373
ENSG00000168300	PCMTD1	8	52770937	T	C	0.196819	0.017412	0.0193489	0.4600002	-0.301638	0.0112118	1.99E-159	-0.0577248	0.064182	0.3684433	0.02320019
ENSG00000168301	KCTD6	3	58482964	T	C	0.251491	-0.00441044	0.0158609	0.84	0.0662344	0.00925657	8.34E-13	-0.0665883	0.239647	0.7811197	0.3463109
ENSG00000168306	ACOX2	3	58506954	A	G	0.054672	-0.0295095	0.0288805	0.16	-0.19252	0.0260211	1.38E-13	0.15328	0.151437	0.3114556	0.90782
ENSG00000168310	IRF2	4	185352300	G	A	0.251491	0.0236421	0.0177956	0.2099999	-0.231003	0.00971423	5.40E-125	-0.102346	0.0771565	0.1846848	0.03806941
ENSG00000168329	CX3CR1	3	39314105	A	G	0.0765408	-0.0299323	0.0257114	0.25	0.317728	0.0154402	4.32E-94	-0.0942072	0.081052	0.2451112	0.787052
ENSG00000168350	DEGS2	14	100619628	A	G	0.0119284	-0.0218999	0.0515283	0.7499995	0.401431	0.0446342	2.39E-19	-0.0545546	0.128505	0.6711768	0.9730508
ENSG00000168374	ARF4	3	57570518	C	T	0.0109344	-0.167578	0.0715263	0.01199997	1.03588	0.0509411	6.33E-92	-0.161774	0.0695059	0.01993925	0.6423383
ENSG00000168385	SEPTIN2	2	242273978	A	G	0.102386	-0.0268938	0.0233747	0.2200002	1.19892	0.0115716	0	-0.0224317	0.0194977	0.2499459	0.3139919
ENSG00000168386	FILIP1L	3	99691171	G	T	0.05666	-0.0423082	0.0269582	0.09699961	-0.101687	0.0153194	3.18E-11	0.416062	0.272418	0.1266884	0.4654521
ENSG00000168389	MFSD2A	1	40428220	A	G	0.511928	0.0053596	0.0149617	0.6200004	-0.0454973	0.0083179	4.51E-08	-0.1178	0.329553	0.7207508	0.8781681
ENSG00000168393	DTYMK	2	242620781	C	T	0.171968	-0.0292458	0.0194735	0.1199999	-0.167826	0.0145712	1.08E-30	0.174263	0.117016	0.1364298	0.8918087
ENSG00000168394	TAP1	6	32817370	C	T	0.398608	0.0181964	0.0144288	0.2599998	0.117048	0.0122774	1.52E-21	0.155461	0.124346	0.2112171	0.555353
ENSG00000168395	ING5	2	242655171	C	G	0.370775	0.0225421	0.0144001	0.05699936	-0.426912	0.00936954	0	-0.0528027	0.0337508	0.1177026	0.1387043
ENSG00000168397	ATG4B	2	242594950	A	T	0.234592	-0.0397035	0.0169295	0.02	-0.423165	0.0173278	1.02E-131	0.0938251	0.0401909	0.01957029	0.4308891
ENSG00000168404	MLKL	16	74720305	A	G	0.269384	0.0229219	0.0161653	0.17	0.315243	0.00887516	2.44E-276	0.0727118	0.0513197	0.1565295	0.248411
ENSG00000168405	CMAHP	6	25149996	C	T	0.233598	0.0212283	0.0169686	0.2099999	0.751922	0.00983148	0	0.0282321	0.02257	0.210983	0.05945492
ENSG00000168411	RFWD3	16	74678035	C	T	0.187873	0.0317249	0.0180494	0.08999948	0.542692	0.00956705	0	0.0584584	0.033275	0.07894723	0.1622317
ENSG00000168421	RHOH	4	40219477	A	G	0.300199	0.0150818	0.0153465	0.2700001	-0.0873943	0.00885041	5.36E-23	-0.172572	0.176468	0.3281136	0.8058834
ENSG00000168427	KLHL30	2	239054475	T	C	0.255467	-0.0211273	0.0164714	0.1299999	-0.253946	0.0104178	3.06E-131	0.0831961	0.0649516	0.200231	0.7247889
ENSG00000168434	COG7	16	23432157	C	T	0.193837	-0.0125423	0.0180307	0.4100001	0.138421	0.00972629	5.83E-46	-0.09061	0.130416	0.4871941	0.001522561
ENSG00000168439	STIP1	11	63962379	A	T	0.38668	0.00358566	0.0143332	0.6499995	0.0457131	0.00811639	1.78E-08	0.0784384	0.313856	0.8026506	0.06952529
ENSG00000168461	RAB31	18	9785357	G	A	0.358847	0.0152214	0.0149512	0.4500005	0.320376	0.00821219	0	0.0475111	0.0466836	0.3088084	0.5342293
ENSG00000168476	REEP4	8	21997498	C	T	0.188867	0.0251938	0.018522	0.2200002	0.508892	0.0108808	0	0.0495072	0.0364121	0.1739456	0.3265847
ENSG00000168477	TNXB	6	32046021	A	G	0.412525	-0.0435341	0.0147274	0.000409996	0.434818	0.0181727	1.60E-126	-0.10012	0.0341277	0.003349581	0.6146769
ENSG00000168487	BMP1	8	22046044	G	A	0.312127	-0.0141997	0.0156035	0.56	0.204668	0.00893301	3.58E-116	-0.0693793	0.0762983	0.3631833	0.105215
ENSG00000168488	ATXN2L	16	28841457	A	G	0.318091	0.00419348	0.0156564	0.7199992	0.0787621	0.0101257	7.34E-15	0.0532423	0.198899	0.7889412	0.821513
ENSG00000168495	POLR3D	8	22106051	A	G	0.50497	-0.0118948	0.0142906	0.2200002	-0.0851785	0.00793274	6.78E-27	0.139645	0.168276	0.4066169	0.172852
ENSG00000168497	CAVIN2	2	192705504	G	A	0.298211	-0.0201139	0.0155375	0.1199999	-0.0872218	0.00863616	5.55E-24	0.230606	0.179595	0.19913	0.3391566
ENSG00000168517	HEXIM2	17	43242737	C	G	0.526839	0.0042002	0.0142567	0.84	0.161309	0.00849109	1.79E-80	0.0260383	0.0883921	0.7683171	0.4443255
ENSG00000168522	FNTA	8	42915134	G	A	0.110338	-0.055991	0.0218845	0.01499996	-0.0953213	0.0130021	2.28E-13	0.587392	0.243166	0.01570914	0.3500764

ENSG00000168528	SERINC2	1	31894968	C	T	0.432406	0.022578	0.0143932	0.08799946	-0.333785	0.00782616	0	-0.0676424	0.0431503	0.1169751	0.8332109
ENSG00000168538	TRAPPC11	4	184607582	G	A	0.390656	-0.0181603	0.0143008	0.2099999	-0.12957	0.00895119	1.74E-47	0.140158	0.110795	0.2058637	0.2950104
ENSG00000168546	GFRA2	8	21608892	A	G	0.427435	-0.0240283	0.0143522	0.09299937	-0.449767	0.00767655	0	0.0534238	0.0319233	0.09422768	0.8777409
ENSG00000168556	ING2	4	184429198	G	A	0.220676	-0.0150854	0.0172928	0.4199997	0.175794	0.0105971	8.40E-62	-0.0858131	0.0985058	0.3836735	0.1576437
ENSG00000168564	CDKN2AIP	4	184367547	A	G	0.200795	0.0203028	0.0174396	0.2700001	-0.149472	0.00991207	2.20E-51	-0.13583	0.117022	0.2457541	0.2988336
ENSG00000168566	SNRNP48	6	7601316	A	G	0.0964215	-0.02213	0.0231155	0.4400003	-0.584629	0.0135444	0	0.0378531	0.0395485	0.3385012	0.8597736
ENSG00000168569	TMEM223	11	62549297	G	A	0.355865	-0.00682803	0.0150018	0.6600001	-0.305865	0.00892444	2.00E-257	0.0223237	0.0490515	0.6490321	0.7193131
ENSG00000168575	SLC20A2	8	42335531	G	A	0.416501	-0.00697346	0.0144115	0.5700002	0.295256	0.00862225	5.45E-257	-0.0236184	0.0488151	0.6285042	0.582103
ENSG00000168591	TMUB2	17	42266718	T	C	0.271372	-0.00597265	0.016145	0.6499995	-0.115617	0.0088233	3.14E-39	0.0516589	0.139698	0.7115384	0.8452424
ENSG00000168594	ADAM29	4	175825075	T	G	0.348907	-0.0016078	0.0150633	0.89	-0.0946089	0.00866223	9.05E-28	0.0169942	0.159224	0.9150023	0.06345675
ENSG00000168610	STAT3	17	40502964	G	A	0.379722	0.0140439	0.0149253	0.4600002	-0.139939	0.00823965	1.09E-64	-0.100357	0.106819	0.3474715	0.7860448
ENSG00000168612	ZSWIM1	20	44511885	T	A	0.441352	-0.00568337	0.0144192	0.6499995	-0.0804479	0.00813591	4.69E-23	0.0706466	0.179379	0.6936991	0.06270572
ENSG00000168614	NA	1	144821078	T	C	0.0427435	0.0512715	0.0414944	0.2599998	-0.471124	0.0509224	2.21E-20	-0.108828	0.0888574	0.2206694	NA
ENSG00000168615	ADAM9	8	38908525	C	T	0.445328	-0.000187603	0.0144964	0.84	-0.156799	0.00797847	5.47E-86	0.00119645	0.092452	0.9896746	0.4832465
ENSG00000168646	AXIN2	17	63541223	A	G	0.295229	0.0145229	0.015852	0.3400001	0.408426	0.00937965	0	0.0355582	0.038821	0.3596916	0.4259779
ENSG00000168653	NDUFS5	1	39496149	A	G	0.44334	0.00763898	0.0143997	0.6600001	-0.533192	0.0074418	0	-0.0143269	0.0270073	0.5957783	0.4439485
ENSG00000168661	ZNF30	19	35426940	A	G	0.338966	0.0138427	0.0155768	0.33	-0.323214	0.00872208	1.39E-300	-0.0428283	0.0482073	0.3743155	0.1798692
ENSG00000168672	LRATD2	8	127567662	C	T	0.0646123	-0.0337795	0.0249398	0.1900002	-0.74646	0.0130404	0	0.045253	0.0334201	0.1757164	0.2603975
ENSG00000168675	LDLRAD4	18	13435125	T	C	0.455268	-0.000681912	0.0142892	0.7800007	0.207263	0.00882603	6.06E-122	-0.00329007	0.0689424	0.9619377	0.5922653
ENSG00000168679	SLC16A4	1	110919587	G	T	0.0745527	-0.0141179	0.0283271	0.3400001	-0.249902	0.0167297	1.88E-50	0.0564937	0.113416	0.6184056	0.841792
ENSG00000168685	IL7R	5	35866251	C	T	0.298211	0.000561695	0.0152105	0.9299999	0.291463	0.00836317	4.14E-266	0.00192716	0.0521868	0.9705423	0.959581
ENSG00000168710	AHCYL1	1	110546832	A	G	0.16501	0.00644087	0.0184577	0.84	-0.126236	0.0100747	5.12E-36	-0.0510226	0.146273	0.7272261	0.2429351
ENSG00000168724	DNAJC21	5	34944383	T	C	0.465209	0.0103062	0.0143038	0.6600001	-0.163565	0.00794269	3.16E-94	-0.0630096	0.0875035	0.471475	0.7468173
ENSG00000168734	PKIG	20	43206657	T	A	0.151093	-0.00370247	0.0201261	0.8700001	-0.212838	0.0114594	5.30E-77	0.0173957	0.0945652	0.8540491	0.2118697
ENSG00000168754	FAM178B	2	97612897	G	A	0.299205	0.00302795	0.0153675	0.59	-0.0541231	0.00855944	2.56E-10	-0.0559456	0.284074	0.8438742	0.7051938
ENSG00000168763	CNNM3	2	97490815	C	G	0.0974155	0.0278288	0.027067	0.28	-0.125408	0.0156854	1.29E-15	-0.221906	0.217609	0.307848	0.5315794
ENSG00000168765	GSTM4	1	110203410	G	T	0.408549	0.00759526	0.0145759	0.58	-0.690845	0.0078282	0	-0.0109942	0.021099	0.602315	0.6072424
ENSG00000168769	TET2	4	106134002	G	C	0.163022	0.0297476	0.02051	0.1800002	0.0796658	0.0107563	1.30E-13	0.373405	0.262341	0.154632	0.06331377
ENSG00000168778	TCTN2	12	124174304	C	T	0.358847	0.00062953	0.0144971	0.7800007	0.17609	0.00823457	1.87E-101	0.00357505	0.0823281	0.9653632	0.3992608
ENSG00000168785	TSPAN5	4	99485649	T	C	0.055666	-0.0222921	0.0306393	0.29	0.1385	0.0139336	2.79E-23	-0.160953	0.221814	0.4680691	0.5372421
ENSG00000168792	ABHD15	17	27890860	A	G	0.315109	0.00280489	0.0154445	0.7600007	0.381235	0.00836879	0	0.00735738	0.0405121	0.8558892	0.07389776
ENSG00000168795	ZBTB5	9	37451753	C	T	0.345924	-0.00694465	0.0151939	0.4899999	0.101772	0.00845941	2.45E-33	-0.0682375	0.149402	0.6478588	0.9909743
ENSG00000168803	ADAL	15	43634484	G	A	0.126243	-0.00208355	0.0213227	0.7800007	-0.473369	0.0116716	0	0.00440153	0.0450447	0.9221587	0.07398817
ENSG00000168806	LCMT2	15	43621388	A	C	0.0765408	-0.00385596	0.0241301	0.8200001	-0.687376	0.0164981	0	0.00560968	0.0351049	0.8730405	0.2873706
ENSG00000168807	SNTB2	16	69281993	T	A	0.196819	0.0227064	0.0169984	0.1100001	0.221079	0.0100218	7.69E-108	0.102707	0.0770291	0.1824157	0.03993112
ENSG00000168811	IL12A	3	159710171	G	A	0.375746	-0.0215701	0.0143743	0.1900002	-0.0598332	0.00803476	9.56E-14	0.360504	0.245068	0.1412823	0.3971521
ENSG00000168813	ZNF507	19	32857535	A	G	0.124254	-0.0108266	0.0247508	0.4700002	0.0773214	0.0128385	1.72E-09	-0.140021	0.320946	0.6626372	0.4932558
ENSG00000168818	STX18	4	4480771	A	G	0.296223	0.0128828	0.0159178	0.4299995	0.0621892	0.00875386	1.21E-12	0.207155	0.257613	0.421321	0.3499906
ENSG00000168824	NSG1	4	4385326	G	A	0.207753	0.00102204	0.0168071	0.84	-0.962161	0.0088754	0	-0.00106223	0.0174681	0.9515105	0.1865504
ENSG00000168826	ZBTB49	4	4307718	A	G	0.431412	0.0111261	0.014221	0.5099998	0.228711	0.00788178	3.93E-185	0.048647	0.0622015	0.4341635	0.592711
ENSG00000168827	GFM1	3	158386215	G	T	0.39662	0.00857477	0.0152074	0.32	-0.251125	0.00870754	6.76E-183	-0.0341454	0.0605686	0.5729261	0.1391447
ENSG00000168852	NA	13	41446158	T	C	0.291252	0.00935888	0.0151183	0.4500005	-0.303217	0.0205243	2.17E-49	-0.0308653	0.0499034	0.5362455	0.5874053
ENSG00000168872	DDX19A	16	70394009	G	A	0.0258449	0.0251981	0.0449986	0.6100002	0.237534	0.0349222	1.03E-11	0.106082	0.190081	0.5767849	0.5971729
ENSG00000168874	ATOH8	2	85996828	C	A	0.228628	-0.00546933	0.0171919	0.9400001	0.773792	0.0085777	0	-0.00706822	0.0222179	0.7503847	0.07409642
ENSG00000168878	SFTPB	2	85890150	T	C	0.406561	0.0318079	0.0146436	0.06699926	-0.121336	0.00831521	3.16E-48	-0.262148	0.122017	0.03167696	0.5896156
ENSG00000168883	USP39	2	85853191	T	A	0.366799	-0.0197153	0.0147892	0.1900002	-0.411281	0.00817886	0	0.0479364	0.0359715	0.1826567	0.1956514
ENSG00000168884	TNIP2	4	2750739	T	C	0.0357853	-0.00586581	0.0364507	0.9699999	-0.442458	0.0310632	4.90E-46	0.0132573	0.0823875	0.872161	0.4411825
ENSG00000168887	C2orf68	2	85836483	G	A	0.405567	0.0321234	0.0146463	0.064	-0.0970332	0.00902786	6.04E-27	-0.331056	0.154052	0.03163509	0.04160392
ENSG00000168890	TMEM150A	2	85827995	T	C	0.241551	-0.0432464	0.0168832	0.007799917	-0.127278	0.00962669	6.60E-40	0.339778	0.135114	0.01191187	0.000973254
ENSG00000168894	RNF181	2	85823792	A	C	0.236581	-0.00340675	0.016945	0.95	0.111393	0.015749	1.52E-12	-0.0305833	0.152181	0.8407249	0.09567002

ENSG00000168899	VAMP5	2	85816033	G	A	0.11332	0.00153513	0.0214787	0.7600007	-0.655234	0.0117862	0	-0.00234287	0.0327802	0.943022	0.06085352
ENSG00000168903	BTNL3	5	180424786	C	T	0.293241	-0.0142114	0.0156811	0.32	0.906747	0.00787369	0	-0.0156729	0.0172943	0.3648048	0.7775763
ENSG00000168904	LRRC28	15	99861250	C	T	0.269384	-0.00837359	0.0156131	0.6800001	0.3499	0.0085412	0	-0.0239314	0.0446255	0.5917704	0.2507816
ENSG00000168906	MAT2A	2	85769345	G	A	0.157058	0.00992718	0.018896	0.4299995	0.322371	0.0195481	4.24E-61	0.0307942	0.0586454	0.5995189	0.1711759
ENSG00000168913	ENHO	9	34522038	T	C	0.352883	0.00929395	0.0152193	0.5300002	-0.0525511	0.00950738	3.25E-08	-0.176856	0.291372	0.5438671	0.9191472
ENSG00000168916	ZNF608	5	124028554	A	G	0.164016	0.0224511	0.0184951	0.33	0.121022	0.0112736	6.97E-27	0.185512	0.153798	0.2277376	0.6473596
ENSG00000168917	SLC35G2	3	136556111	T	C	0.343936	0.0124907	0.0152866	0.5300002	0.067181	0.00960277	2.63E-12	0.185926	0.22909	0.4170301	0.9381538
ENSG00000168918	INPP5D	2	234020613	G	T	0.32505	0.0119101	0.0154303	0.4	0.187382	0.00851119	2.02E-107	0.0635604	0.0823972	0.4404755	0.5108876
ENSG00000168936	TMEM129	4	1720382	C	T	0.440358	-0.010014	0.0141999	0.58	-0.151317	0.00861373	4.41E-69	0.0661788	0.0939174	0.4810289	0.3857644
ENSG00000168944	CEP120	5	122719932	A	G	0.152087	-0.036122	0.0195758	0.08500021	0.135202	0.0108053	6.37E-36	-0.26717	0.146355	0.06792647	0.3483973
ENSG00000168958	MFF	2	228206208	A	G	0.408549	0.0126957	0.0147126	0.64	0.229175	0.00800564	3.13E-180	0.0553974	0.0642273	0.3884005	0.257055
ENSG00000168961	LGALS9	17	25966705	G	A	0.251491	-0.00121368	0.016129	0.9	-0.74282	0.00897244	0	0.00163388	0.0217132	0.9400172	0.862299
ENSG00000168993	CPLX1	4	799365	T	C	0.134195	-0.00942143	0.0212609	0.6899999	-0.0763032	0.0123885	7.31E-10	0.123474	0.279357	0.6584953	0.1519923
ENSG00000168994	PXDC1	6	3737554	T	C	0.4334	0.0153754	0.0143157	0.4100001	0.106083	0.00889807	9.09E-33	0.144938	0.135495	0.2847581	0.051586
ENSG00000168995	SIGLEC7	19	51651169	T	G	0.285288	-0.0661361	0.0151688	1.30E-05	0.216386	0.0104669	6.01E-95	-0.305639	0.0716426	1.99E-05	0.02605669
ENSG00000169018	FEM1B	15	68579172	G	T	0.354871	0.00529118	0.0151511	0.7099994	-0.0976514	0.00874182	5.68E-29	-0.0541844	0.155231	0.7270466	0.6726455
ENSG00000169019	COMMD8	4	47459310	G	A	0.327038	-0.0207659	0.0152937	0.1800002	0.11605	0.00841604	2.96E-43	-0.178939	0.132422	0.1766082	0.3421481
ENSG00000169026	SLC49A3	4	679424	T	C	0.318091	0.0265267	0.0152913	0.08799946	-0.315196	0.0116544	4.36E-161	-0.0841595	0.0486134	0.08341611	0.7972377
ENSG00000169031	COL4A3	2	228104394	A	T	0.242545	-0.000488854	0.0159683	0.9599999	-0.135848	0.00900291	1.90E-51	0.00359852	0.117545	0.9755774	0.1546002
ENSG00000169032	MAP2K1	15	66731902	G	A	0.240557	-0.000405392	0.0166335	0.83	-0.203235	0.0136824	6.57E-50	0.00199469	0.0818437	0.9805559	0.7939805
ENSG00000169045	HNRNPH1	5	179051482	C	T	0.347913	-0.0138896	0.0148838	0.3400001	-0.379979	0.00812382	0	0.0365536	0.0391778	0.3508113	0.3946913
ENSG00000169047	IRS1	2	227632116	T	A	0.275348	-0.0142687	0.015868	0.3700002	0.242744	0.00922592	1.43E-152	-0.0587808	0.0654074	0.3688191	0.4695617
ENSG00000169062	UPF3A	13	115059171	C	T	0.173956	0.00981638	0.0199566	0.7099994	0.556512	0.0118559	0	0.0176391	0.0358621	0.6228187	0.1709787
ENSG00000169071	ROR2	9	94518908	T	C	0.0387674	-0.0440852	0.0379869	0.2099999	0.471965	0.0186058	5.89E-142	-0.0934077	0.0805708	0.246324	0.9171707
ENSG00000169085	VXN	8	67401498	C	T	0.501988	-0.0133728	0.0141996	0.4700002	-0.348795	0.00778185	0	0.03834	0.0407194	0.3464153	0.1861162
ENSG00000169087	HSPBAP1	3	122485758	G	C	0.208748	0.0145704	0.0170523	0.2999998	-0.768821	0.00957358	0	-0.0189516	0.022181	0.3928805	0.4497419
ENSG00000169105	CHST14	15	40764256	C	T	0.413519	0.00400646	0.014406	0.7300002	-0.0838215	0.00798954	9.46E-26	-0.0477975	0.171926	0.7810025	0.9616983
ENSG00000169116	PARM1	4	75916815	T	C	0.0894632	-0.0164319	0.0260122	0.58	-0.388473	0.0151058	7.58E-146	0.0422987	0.0669804	0.5277076	0.3591788
ENSG00000169118	CSNK1G1	15	64553079	T	C	0.114314	0.00116946	0.0217152	0.9699999	0.295647	0.0113903	1.56E-148	0.0039556	0.07345	0.9570512	0.3323273
ENSG00000169122	FAM110B	8	59011953	C	T	0.456262	0.0153636	0.0143962	0.1900002	0.0992304	0.00800168	2.57E-35	0.154828	0.145615	0.2876602	0.4602905
ENSG00000169129	AFAP1L2	10	116109549	A	G	0.468191	0.00980389	0.0142144	0.5300002	-0.142165	0.00794476	1.31E-71	-0.0689613	0.100059	0.4906952	0.802095
ENSG00000169131	ZNF354A	5	178148148	A	G	0.252485	-0.00068291	0.0170975	0.98	-0.439455	0.00964087	0	0.00155399	0.0389061	0.9681393	0.1710844
ENSG00000169136	ATF5	19	50434575	T	C	0.0367793	-0.00574299	0.0372682	0.6200004	0.21948	0.0206663	2.40E-26	-0.0261663	0.16982	0.8775445	0.2437594
ENSG00000169155	ZBTB43	9	129583887	A	G	0.218688	0.0137406	0.017066	0.5400003	-0.137127	0.00951332	4.21E-47	-0.100203	0.124648	0.4214596	0.5797161
ENSG00000169169	CPT1C	19	50205571	T	C	0.345924	-0.0308257	0.0150291	0.1100001	-0.117922	0.00867648	4.53E-42	0.261408	0.128893	0.04254977	0.1451975
ENSG00000169180	XPO6	16	28166270	T	C	0.286282	-0.00729005	0.0156231	0.4500005	0.251132	0.00843508	8.90E-195	-0.0290287	0.0622182	0.6408128	0.4616994
ENSG00000169184	MN1	22	28170875	T	C	0.026839	-0.00128538	0.0321017	0.81	0.389934	0.0241311	9.81E-59	-0.0032964	0.0823262	0.9680606	0.09949211
ENSG00000169189	NSMCE1	16	27258213	C	T	0.293241	0.0235027	0.0155026	0.0659994	0.110826	0.0128641	6.98E-18	0.212068	0.142031	0.1354092	0.3599475
ENSG00000169193	CCDC126	7	23660662	A	G	0.227634	-0.00377693	0.0162027	0.7899998	0.383703	0.0091029	0	-0.00984338	0.0422279	0.8156827	0.5032351
ENSG00000169203	NPIPB12	16	29506165	C	G	0.431412	0.0239673	0.0142163	0.03099988	0.398576	0.0184562	1.97E-103	0.0601323	0.0357762	0.09280392	0.7081843
ENSG00000169217	CD2BP2	16	30364384	T	C	0.00497018	0.0348593	0.091275	0.98	-0.885488	0.0596819	8.47E-50	-0.0393673	0.103113	0.7026182	0.05035024
ENSG00000169220	RGS14	5	176792220	A	G	0.27833	-0.0182233	0.0160911	0.2	0.151364	0.00899767	1.67E-63	-0.120394	0.106548	0.2584971	0.3767842
ENSG00000169221	TBC1D10B	16	30375004	C	T	0.343936	0.0149234	0.0149384	0.2399999	-0.0745025	0.00838992	6.69E-19	-0.200307	0.201774	0.3208398	0.3517322
ENSG00000169223	LMAN2	5	176768708	A	G	0.343936	-0.00717575	0.0153272	0.4100001	0.119829	0.008653	1.30E-43	-0.0598835	0.127982	0.6398535	0.5163653
ENSG00000169224	GCSAML	1	247705676	C	A	0.333996	-0.00597339	0.0147486	0.6300007	-0.12885	0.00906106	6.87E-46	0.0463592	0.11451	0.6855879	0.7323242
ENSG00000169228	RAB24	5	176729472	T	A	0.163022	-0.026187	0.0189574	0.1900002	-0.168959	0.00995108	1.17E-64	0.15499	0.112572	0.1685706	0.6532685
ENSG00000169230	PRELID1	5	176732367	G	C	0.175944	-0.0187287	0.0191203	0.3100002	-0.871989	0.0195089	0	0.0214781	0.0219325	0.3274397	0.6656672
ENSG00000169231	THBS3	1	155172110	C	G	0.39662	0.0223357	0.0145376	0.04799986	-0.525256	0.00751019	0	-0.0425235	0.0276839	0.124529	0.2121034
ENSG00000169241	SLC50A1	1	155109574	A	G	0.00497018	-0.0735437	0.0792938	0.32	-0.749682	0.132462	1.52E-08	0.0980999	0.107181	0.3600474	0.712734
ENSG00000169242	EFNA1	1	155103634	A	G	0.17992	0.0379325	0.0181772	0.005699936	-0.0842302	0.0114902	2.29E-13	-0.450343	0.224378	0.0447415	0.5614871

ENSG00000169245	CXCL10	4	76943461	C	A	0.491054	-0.0203852	0.0142616	0.2700001	-0.185104	0.00795831	1.15E-119	0.110128	0.0771918	0.1536706	0.3230965
ENSG00000169246	NPIPB3	16	21436723	A	G	0.0129225	-0.0209278	0.0551268	0.5400003	-0.756989	0.0668246	9.54E-30	0.0276461	0.0728646	0.7043781	0.8037652
ENSG00000169247	SH3TC2	5	148372964	T	C	0.140159	0.0145315	0.0194147	0.2700001	0.122087	0.0109291	5.67E-29	0.119026	0.15938	0.4551814	0.6319052
ENSG00000169251	NMD3	3	160896902	T	C	0.279324	0.0105743	0.0157604	0.56	-0.293273	0.0126815	2.54E-118	-0.0360562	0.0537624	0.5024379	0.3819377
ENSG00000169252	ADRB2	5	148207176	A	G	0.414513	0.016658	0.0143359	0.1199999	0.126274	0.00801464	6.31E-56	0.13192	0.113839	0.2465252	0.8632383
ENSG00000169255	B3GALNT1	3	160812421	C	T	0.269384	0.00130191	0.0158825	0.9299999	-0.257136	0.00865502	5.79E-194	-0.00506311	0.0617671	0.9346697	0.02423947
ENSG00000169258	GPRIN1	5	176029968	T	C	0.409543	0.00337891	0.014384	0.8800001	0.182642	0.00869409	5.59E-98	0.0185002	0.0787601	0.8142919	0.1044114
ENSG00000169282	KCNAB1	3	156006017	T	C	0.336978	-0.0222096	0.0153064	0.09099971	-0.233113	0.00859184	4.14E-162	0.095274	0.0657547	0.147357	0.2383706
ENSG00000169288	MRPL1	4	78828809	G	A	0.0119284	0.0355881	0.0612824	0.6600001	1.42062	0.0763554	2.91E-77	0.0250511	0.0431587	0.5616177	0.7822328
ENSG00000169291	SHE	1	154458418	C	T	0.280318	0.029925	0.0169601	0.1100001	0.119078	0.00997958	8.04E-33	0.251305	0.143977	0.08090562	0.9140588
ENSG00000169299	PGM2	4	37846406	G	T	0.409543	-0.00964494	0.0145065	0.4	0.289658	0.00794249	3.39E-291	-0.0332977	0.0500898	0.5062042	0.6286657
ENSG00000169313	P2RY12	3	151078884	G	C	0.363817	0.0040855	0.0148497	0.8	0.195835	0.00834653	9.74E-122	0.020862	0.075833	0.783236	0.2725584
ENSG00000169314	C22orf15	22	24106628	C	G	0.0705765	-0.0138016	0.0271683	0.59	-0.165632	0.0153371	3.46E-27	0.083327	0.16421	0.6118442	0.6344655
ENSG00000169330	MINAR1	15	79744745	A	G	0.395626	-0.00909794	0.0146019	0.4299995	-0.238583	0.0088036	9.69E-162	0.0381332	0.0612188	0.5333497	0.8998488
ENSG00000169359	SLC33A1	3	155558261	T	C	0.289264	-0.0100003	0.0159023	0.5099998	-0.264018	0.0129595	2.93E-92	0.0378773	0.0602605	0.5296376	0.4461078
ENSG00000169371	SNUPN	15	75904617	A	G	0.281312	-0.0153226	0.016339	0.4100001	0.410705	0.0103111	0	-0.037308	0.0397938	0.3484844	0.3931488
ENSG00000169375	SIN3A	15	75704951	A	G	0.244533	0.0174948	0.0160801	0.2	0.0504427	0.00904639	2.46E-08	0.346825	0.324791	0.2855921	0.4146781
ENSG00000169379	ARL13B	3	93736747	G	A	0.430417	-0.00802876	0.0144623	0.5500004	-0.0547922	0.00802815	8.79E-12	0.146531	0.26482	0.5800419	0.06608244
ENSG00000169385	RNASE2	14	21424103	C	T	0.271372	0.00127384	0.016278	0.9299999	0.503073	0.00873807	0	0.00253212	0.0323572	0.937625	0.346205
ENSG00000169397	RNASE3	14	21360032	C	T	0.271372	0.00127384	0.016278	0.9299999	0.484868	0.00963447	0	0.00262719	0.0335721	0.937625	0.3136728
ENSG00000169398	PTK2	8	141840157	A	G	0.459245	0.00266455	0.014487	0.7800007	-0.164576	0.00800196	5.42E-94	-0.0161904	0.0880296	0.8540765	0.3761788
ENSG00000169403	PTAFR	1	28497062	A	G	0.05666	-0.0628159	0.0327358	0.03699985	-0.117561	0.0199067	3.51E-09	0.534326	0.292788	0.06800773	0.8214497
ENSG00000169410	PTPN9	15	75815546	T	A	0.282306	-0.0106395	0.0163046	0.5999997	0.0576434	0.0092088	3.86E-10	-0.184574	0.284386	0.5163198	0.3896339
ENSG00000169413	RNASE6	14	21249918	T	A	0.494036	-0.00449582	0.0142629	0.81	0.73648	0.00710916	0	-0.00610447	0.0193664	0.7526027	0.145942
ENSG00000169429	CXCL8	4	74607828	T	C	0.0715706	-0.007299	0.0288875	0.6600001	-0.481359	0.0168779	6.60E-179	0.0151633	0.0600147	0.800531	0.8689007
ENSG00000169432	SCN9A	2	167142099	A	C	0.44831	-0.0129508	0.0142125	0.56	-0.338559	0.00774926	0	0.0382527	0.0419885	0.3622807	0.5790774
ENSG00000169435	RASSF6	4	74461807	G	T	0.0248509	-0.0388318	0.0497687	0.33	0.574898	0.0271345	1.26E-99	-0.0675455	0.0866282	0.4355575	0.8434155
ENSG00000169439	SDC2	8	97564789	T	C	0.440358	-0.00656732	0.0142724	0.6800001	0.093045	0.00798258	2.14E-31	-0.0705822	0.153512	0.6456719	0.437383
ENSG00000169442	CD52	1	26645731	G	A	0.206759	0.0355966	0.0182956	0.0329997	-0.244961	0.0103125	1.00E-124	-0.145315	0.0749379	0.0524839	0.08750146
ENSG00000169490	TM2D2	8	38850335	C	A	0.368787	0.00169285	0.0145468	0.6999999	0.2192	0.00822708	2.12E-156	0.00772286	0.0663638	0.9073581	0.6886373
ENSG00000169495	HTRA4	8	38838932	G	A	0.440358	0.000329661	0.0145287	0.8600001	0.0516975	0.00905481	1.13E-08	0.00637673	0.281035	0.9818974	0.5031088
ENSG00000169499	PLEKHA2	8	38795090	T	C	0.306163	0.0146624	0.0157281	0.5	0.173952	0.00958053	1.13E-73	0.08429	0.0905356	0.3518453	0.8131395
ENSG00000169504	CLIC4	1	25121331	G	C	0.27336	0.00149013	0.0161966	0.9599999	0.287751	0.00874406	1.68E-237	0.00517853	0.056287	0.9266962	0.000351267
ENSG00000169507	SLC38A11	2	165782365	A	G	0.158052	0.0210148	0.0191347	0.2700001	0.17492	0.0107428	1.31E-59	0.120139	0.10964	0.2731811	0.3976722
ENSG00000169508	GPR183	13	99953221	T	C	0.154076	-0.0240488	0.0197267	0.2700001	-0.0877639	0.0113654	1.15E-14	0.274017	0.227554	0.2285182	0.5823274
ENSG00000169519	METTL15	11	28242424	G	A	0.140159	0.0192246	0.0193923	0.32	0.136351	0.0214058	1.89E-10	0.140994	0.143936	0.3273034	0.4876617
ENSG00000169564	PCBP1	2	70315458	C	A	0.0129225	0.0469221	0.0507411	0.33	0.243571	0.0349717	3.29E-12	0.192643	0.21015	0.3593046	0.6566941
ENSG00000169567	HINT1	5	130501074	G	A	0.0357853	0.0108503	0.0418447	0.7800007	-0.694672	0.0333016	1.24E-96	-0.0156193	0.0602413	0.7954198	0.5516058
ENSG00000169570	DTWD2	5	118248628	T	C	0.480119	-0.00822047	0.0141818	0.56	-0.14008	0.00794192	1.26E-69	0.0586843	0.101296	0.5623621	0.6622575
ENSG00000169575	VPREB1	22	22599507	G	A	0.33996	0.0159163	0.0149428	0.3400001	-0.134636	0.0085003	1.67E-56	-0.118217	0.111237	0.2878962	0.2326983
ENSG00000169583	CLIC3	9	139890171	G	C	0.517893	-0.0270315	0.0142317	0.08400014	-0.259227	0.00899939	1.86E-182	0.104277	0.0550197	0.05805655	0.1932626
ENSG00000169592	INO80E	16	30011864	C	G	0.444334	0.0265965	0.0142272	0.02100003	-0.305033	0.00782635	0	-0.0871923	0.0466952	0.06186453	0.9648073
ENSG00000169598	DFFB	1	3787919	C	T	0.459245	0.0113293	0.0142423	0.5300002	0.078366	0.00800372	1.23E-22	0.144569	0.18234	0.4278617	0.2516208
ENSG00000169599	NFU1	2	69643821	A	G	0.38668	-0.00568687	0.0144365	0.83	0.267776	0.0119309	1.47E-111	-0.0212374	0.0539209	0.6936826	0.8373588
ENSG00000169609	C15orf40	15	83668793	C	A	0.483101	0.000532357	0.0141909	0.89	0.176186	0.00789818	3.15E-110	0.00302156	0.080545	0.9700752	0.8657912
ENSG00000169612	RAMAC	15	83657384	G	C	0.0119284	0.0619583	0.0503705	0.2099999	-0.691696	0.0588267	6.41E-32	-0.0895744	0.0732191	0.2211879	0.4866096
ENSG00000169621	APLF	2	68776348	T	C	0.0715706	0.000204065	0.0258836	0.8499999	0.126925	0.0157487	7.67E-16	0.00160776	0.203928	0.9937096	0.6773799
ENSG00000169629	RGPD8	2	113159867	T	C	0.16004	0.0134066	0.0197142	0.8200001	-0.323435	0.0126345	1.55E-144	-0.0414507	0.0609742	0.496626	0.6910201
ENSG00000169635	HIC2	22	21788722	C	G	0.382704	-0.0049923	0.0144837	0.8800001	0.0569696	0.00818506	3.40E-12	-0.0876309	0.254547	0.7306492	0.0112915
ENSG00000169641	LUZP1	1	23457408	A	G	0.146123	-0.0123387	0.0190081	0.56	0.2483	0.0104593	1.41E-124	-0.0496928	0.0765817	0.5164121	0.9347851

ENSG00000169660	HEXD	17	80388357	G	T	0.333002	0.00490388	0.0155194	0.7700005	0.407944	0.00844429	0	0.012021	0.0380438	0.7520199	0.3351765
ENSG00000169682	SPNS1	16	28990705	G	C	0.262425	0.00343693	0.0155084	0.7099994	0.608614	0.00809963	0	0.00564714	0.0254816	0.8246126	0.692937
ENSG00000169683	LRRC45	17	79985102	T	C	0.32008	-0.00477546	0.0153623	0.9400001	-0.28927	0.0100697	1.77E-181	0.0165086	0.0531102	0.7559241	0.6264853
ENSG00000169689	CENPX	17	79979280	C	A	0.44334	-0.0025571	0.014304	0.99	-0.263222	0.00923271	8.88E-179	0.00971463	0.0543431	0.8581225	0.8319951
ENSG00000169692	AGPAT2	9	139574735	T	C	0.129225	0.015414	0.0221731	0.3100002	-0.208457	0.018037	6.79E-31	-0.0739434	0.10656	0.4877371	0.6377548
ENSG00000169696	ASPSCR1	17	79954982	C	G	0.379722	0.0086287	0.0148749	0.4400003	-0.260652	0.00971397	1.34E-158	-0.0331043	0.0570814	0.561949	0.3684028
ENSG00000169704	GP9	3	128780429	T	C	0.111332	0.0123503	0.0221543	0.6700003	-0.0679482	0.0124488	4.81E-08	-0.18176	0.327743	0.5791803	0.281362
ENSG00000169710	FASN	17	80046211	T	C	0.480119	8.53E-06	0.0144539	0.7099994	0.389883	0.0127681	8.78E-205	2.19E-05	0.0370724	0.999529	0.7254586
ENSG00000169714	CNBP	3	128895546	A	G	0.0208748	-0.0226991	0.0467688	0.7099994	-0.265699	0.0380048	2.73E-12	0.0854316	0.176445	0.6282569	0.8848114
ENSG00000169715	MT1E	16	56660205	T	C	0.393638	0.0185655	0.0145529	0.2200002	-0.263254	0.00877274	7.67E-198	-0.0705231	0.0553308	0.2024601	0.4086659
ENSG00000169727	GPS1	17	80011956	A	G	0.0994036	0.0262021	0.0260455	0.2200002	-0.120484	0.0184113	5.99E-11	-0.217475	0.218714	0.3200615	0.9251777
ENSG00000169733	RFNG	17	80007742	T	C	0.483101	-0.00185881	0.0144195	0.6100002	0.0603222	0.00911294	3.61E-11	-0.0308147	0.239087	0.8974486	0.9240365
ENSG00000169738	DCXR	17	79994310	G	C	0.0984095	-0.0164111	0.0218088	0.3700002	0.950059	0.0128296	0	-0.0172738	0.0229564	0.4517744	0.7306802
ENSG00000169740	ZNF32	10	44141805	G	A	0.323062	-0.00568733	0.0149328	0.7300002	-0.205717	0.00838572	6.77E-133	0.0276464	0.0725979	0.70334	0.9829417
ENSG00000169744	LDB2	4	16701798	T	C	0.305169	0.00595484	0.0156532	0.5199996	-0.113367	0.00904075	4.53E-36	-0.0525269	0.138139	0.7037612	0.3802659
ENSG00000169758	TMEM266	15	76436820	T	C	0.267396	0.00726021	0.0160099	0.5099998	-0.055987	0.00913507	8.85E-10	-0.129677	0.286739	0.6510916	0.8452543
ENSG00000169762	TAPT1	4	16195580	T	C	0.379722	0.0154767	0.0148188	0.2300001	0.2571	0.00823998	1.02E-213	0.0601973	0.0576706	0.2965725	0.6408852
ENSG00000169764	UGP2	2	64093385	C	A	0.0626243	-0.0206773	0.0342697	0.4700002	-0.291698	0.01855	1.02E-55	0.070886	0.11757	0.5465579	0.7381517
ENSG00000169813	HNRNPF	10	43892839	T	C	0.307157	0.0173452	0.0149577	0.1800002	-0.193669	0.00844537	2.23E-116	-0.089561	0.077332	0.2468081	0.4242396
ENSG00000169814	BTD	3	15665088	A	G	0.260437	0.0220704	0.0159759	0.09800089	0.0890598	0.0091454	2.07E-22	0.247816	0.18118	0.171379	0.4277892
ENSG00000169826	CSGALNACT2	10	43657345	A	G	0.168986	-0.00392308	0.0189662	0.95	0.242796	0.0172922	8.77E-45	-0.0161579	0.0781243	0.8361478	0.1855264
ENSG00000169855	ROBO1	3	79231677	T	C	0.493042	-0.0101305	0.0142114	0.5099998	-0.0906694	0.0079355	3.11E-30	0.11173	0.157043	0.4767993	0.8568093
ENSG00000169857	AVEN	15	34244902	G	T	0.207753	0.0189637	0.017485	0.28	0.131409	0.0108882	1.54E-33	0.14431	0.133594	0.2800457	0.3993551
ENSG00000169860	P2RY1	3	152554288	G	T	0.134195	0.00787144	0.0208231	0.84	0.0815366	0.0115934	2.02E-12	0.0965388	0.255752	0.7058244	0.8940112
ENSG00000169871	TRIM56	7	100731868	T	C	0.0685885	0.0257854	0.0255096	0.33	-0.205082	0.0162117	1.12E-36	-0.125732	0.124784	0.3136465	0.07632484
ENSG00000169877	AHSP	16	31539654	T	C	0.154076	0.00386836	0.0192827	0.98	-0.205771	0.0103717	1.36E-87	-0.0187994	0.0937143	0.8410092	0.704367
ENSG00000169884	WNT10B	12	49362334	A	G	0.429423	-0.00460131	0.0142924	0.9	-0.115246	0.00794546	1.13E-47	0.0399261	0.124047	0.7475574	0.9834611
ENSG00000169885	CALML6	1	1847500	G	A	0.295229	0.00907249	0.0153499	0.4700002	0.102437	0.0085444	4.07E-33	0.0885664	0.150029	0.5549704	0.9419855
ENSG00000169896	ITGAM	16	31307762	G	A	0.324056	0.0074792	0.0153411	0.6600001	-0.166212	0.00884059	7.41E-79	-0.044998	0.0923295	0.626001	0.1419713
ENSG00000169902	TPST1	7	65777858	A	G	0.372763	-0.00618604	0.0146947	0.6800001	-0.420099	0.00779704	0	0.0147252	0.0349802	0.6737858	0.3264731
ENSG00000169905	TOR1AIP2	1	179828018	G	A	0.0139165	0.0359877	0.0694148	0.64	-0.364307	0.0562758	9.57E-11	-0.098784	0.191149	0.6053031	0.5329683
ENSG00000169908	TM4SF1	3	149091230	C	T	0.332008	-0.00803817	0.0153691	0.6200004	-0.13221	0.00928403	5.13E-46	0.0607984	0.116326	0.6012136	0.5608636
ENSG00000169914	OTUD3	1	20224222	A	G	0.05666	-0.0176605	0.0278234	0.7199992	0.311531	0.0179845	3.20E-67	-0.0566893	0.0893716	0.5258787	0.8233933
ENSG00000169918	OTUD7A	15	31969160	T	C	0.308151	-0.0235692	0.0151731	0.1499999	0.0666402	0.00854033	6.05E-15	-0.353678	0.232155	0.1276435	0.2063892
ENSG00000169919	GUSB	7	65436486	A	C	0.106362	-0.0243281	0.0236945	0.28	0.434706	0.0131545	1.76E-239	-0.0559645	0.0545332	0.304776	0.8995925
ENSG00000169925	BRD3	9	136914223	T	C	0.423459	0.0090077	0.0145748	0.6999999	-0.337207	0.00896183	78008800000001e-3	-0.0267127	0.043228	0.5366089	0.2267652
ENSG00000169926	KLF13	15	31673463	G	C	0.335984	0.0193872	0.0148273	0.2300001	-0.111141	0.00837915	3.74E-40	-0.174438	0.134056	0.1931808	0.4613101
ENSG00000169957	ZNF768	16	30536733	A	G	0.38171	0.0148012	0.014457	0.1299999	-0.0678393	0.00833242	3.90E-16	-0.21818	0.214785	0.3097206	0.3095178
ENSG00000169962	TAS1R3	1	1268690	G	C	0.0596421	0.023986	0.0326203	0.4400003	0.43189	0.0224111	9.37E-83	0.0555373	0.0755842	0.4624769	0.8031594
ENSG00000169964	TMEM42	3	44905261	A	G	0.2167	-0.00978599	0.0177351	0.5999997	0.142388	0.0104191	1.62E-42	-0.0687277	0.124656	0.5814024	0.6370071
ENSG00000169967	MAP3K2	2	128101173	G	A	0.233598	-0.00677167	0.0169325	0.83	-0.533461	0.0091028	0	0.0126938	0.0317416	0.6892212	0.5310898
ENSG00000169972	PUSL1	1	1245502	A	G	0.101392	0.0276415	0.0217056	0.1199999	-0.25264	0.0165345	1.05E-52	-0.109411	0.0862131	0.204415	0.07220896
ENSG00000169976	SF3B5	6	144416386	A	G	0.374751	-0.0017072	0.0149309	0.7499995	-0.0927853	0.00824436	2.20E-29	0.0183995	0.160927	0.9089729	0.9052696
ENSG00000169981	ZNF35	3	44696251	G	A	0.224652	-0.00941132	0.0174189	0.5500004	0.280773	0.00971007	7.60E-184	-0.0335194	0.06205	0.5890596	0.4833297
ENSG00000169991	IFFO2	1	19256977	A	G	0.449304	-0.00610945	0.0144407	0.4299995	-0.297444	0.00969725	1.30E-206	0.0205399	0.048554	0.6722724	0.09159677
ENSG00000169992	NLGN2	17	7315686	G	T	0.101392	-0.0108012	0.024762	0.5500004	-0.120571	0.013121	3.96E-20	0.0895835	0.205603	0.6630471	0.6025342
ENSG00000169994	MYO7B	2	128344341	C	G	0.45328	-0.00784049	0.0144532	0.56	-0.211353	0.00803321	1.48E-152	0.0370967	0.0683987	0.5875712	0.5585649
ENSG00000170004	CHD3	17	7802101	T	C	0.171968	0.00669047	0.0179436	0.5500004	-0.111014	0.0100555	2.44E-28	-0.0602667	0.161725	0.70941	0.5787994
ENSG00000170006	TMEM154	4	153570550	T	A	0.0218688	0.0729019	0.0625237	0.3900004	0.593437	0.0326365	7.01E-74	0.122847	0.105575	0.2445868	0.5882295
ENSG00000170011	MYRIP	3	40076108	T	C	0.362823	0.0124541	0.0146781	0.5	0.0750894	0.00856989	1.92E-18	0.165857	0.196389	0.3983726	0.3656123

ENSG00000170017	ALCAM	3	105190748	C	T	0.34493	-0.00603082	0.0153857	0.6800001	-0.487399	0.00800501	0	0.0123735	0.0315676	0.6950822	0.9124598
ENSG00000170027	YWHAG	7	75972232	C	A	0.441352	0.000829617	0.0142832	0.8	0.253752	0.00788565	3.47E-227	0.0032694	0.0562881	0.9536823	0.1339267
ENSG00000170035	UBE2E3	2	181886643	C	T	0.286282	-0.00882438	0.0157111	0.7700005	0.14541	0.00937512	2.96E-54	-0.0606862	0.108118	0.5745948	0.5803716
ENSG00000170037	CNTROB	17	7844327	C	G	0.459245	0.0165397	0.0143401	0.2999998	0.321857	0.0116113	4.08E-169	0.0513883	0.0445928	0.2491605	0.450827
ENSG00000170043	TRAPPC1	17	7834552	G	A	0.0218688	-0.0400049	0.0501696	0.2700001	-0.270573	0.0258728	1.35E-25	0.147852	0.185958	0.4265639	0.8217342
ENSG00000170049	KCNAB3	17	7829149	A	C	0.449304	-0.00680659	0.0142511	0.6899999	-0.169501	0.00794526	5.54E-101	0.0401567	0.0840979	0.6330069	0.8988761
ENSG00000170074	FAM153A	5	177172690	T	G	0.0994036	0.0365842	0.0247576	0.17	1.02313	0.0331655	5.76E-209	0.0357573	0.0242258	0.139943	0.8486892
ENSG00000170085	SIMC1	5	175719179	A	G	0.188867	0.0419901	0.0180794	0.032	-0.211228	0.0111082	1.27E-80	-0.198791	0.0862281	0.02114386	0.2397405
ENSG00000170088	TMEM192	4	166063465	C	A	0.0785288	-0.0334573	0.0262231	0.16	-0.0878253	0.0143651	9.73E-10	0.380953	0.305015	0.211678	0.1207797
ENSG00000170089	NA	5	177307070	A	G	0.392644	-0.00116938	0.0159135	0.7300002	0.581953	0.0180777	2.30E-227	-0.00200941	0.0273451	0.9414215	0.8399372
ENSG00000170091	NSG2	5	173571555	A	C	0.287276	0.0248812	0.0154599	0.1	0.0641827	0.00889031	5.22E-13	0.387662	0.246786	0.1162192	0.06472565
ENSG00000170092	SPDYE5	7	75128514	T	C	0.444334	-0.0122587	0.0143768	0.4	-0.577354	0.0184536	7.06E-215	0.0212325	0.0249104	0.3940166	0.00868484
ENSG00000170100	ZNF778	16	89289740	T	C	0.0188867	0.0470344	0.0600812	0.4400003	0.329996	0.0378349	2.73E-18	0.14253	0.182798	0.4355599	0.3268612
ENSG00000170113	NIPA1	15	23071641	A	G	0.367793	-0.00685543	0.0146778	0.6300007	0.243087	0.00870573	1.42E-171	-0.0282016	0.0603894	0.6405029	0.708451
ENSG00000170128	GPR25	1	200842694	A	G	0.292247	0.0304832	0.0154659	0.032	0.142556	0.00887162	4.22E-58	0.213833	0.109303	0.05042602	0.9681867
ENSG00000170142	UBE2E1	3	23890100	A	C	0.262425	-0.0306952	0.0159013	0.04	-0.0722035	0.00883418	3.00E-16	0.425121	0.226288	0.0602894	0.3892938
ENSG00000170145	SIK2	11	111537346	A	G	0.341948	0.0208886	0.0155356	0.25	0.225513	0.00863088	1.73E-150	0.0926271	0.0689812	0.179341	0.7846957
ENSG00000170153	RNF150	4	141960378	C	G	0.083499	-0.00971831	0.0271698	0.5199996	1.19775	0.0148741	0	-0.00811382	0.0226843	0.7205795	0.2374836
ENSG00000170160	CCDC144A	17	16650309	G	A	0.0725646	0.0131658	0.0270508	0.6999999	-0.240955	0.0383236	3.23E-10	-0.05464	0.112601	0.6274956	0.42076
ENSG00000170175	CHRNB1	17	7354703	C	T	0.34493	-0.0185308	0.0147789	0.2300001	0.293731	0.00805353	3.04E-291	-0.0630876	0.0503441	0.210159	0.9169959
ENSG00000170180	GYPA	4	145046180	C	T	0.55169	-0.00113274	0.0143664	0.9199999	0.149892	0.00937764	1.65E-57	-0.00755703	0.095846	0.9371555	0.8051077
ENSG00000170185	USP38	4	144125526	G	A	0.398608	-0.0010775	0.0144751	0.8499999	-0.199359	0.0080105	1.02E-136	0.00540483	0.0726086	0.940662	0.6596891
ENSG00000170190	SLC16A5	17	73093039	T	C	0.33996	-0.00808306	0.0151931	0.56	0.217033	0.00840361	4.50E-147	-0.0372434	0.0700184	0.5947891	0.1254059
ENSG00000170191	NANP	20	25599191	C	T	0.407555	-0.0048155	0.0144002	0.5400003	-0.337254	0.00787277	0	0.0142785	0.0426996	0.7380816	0.8399227
ENSG00000170209	ANKK1	11	113264826	T	C	0.134195	-0.0203273	0.0207277	0.29	0.367095	0.0115661	4.53E-221	-0.0553734	0.056491	0.32698	0.1785607
ENSG00000170222	ADPRM	17	10607730	G	C	0.483101	-0.00701242	0.0143638	0.56	-0.171366	0.00967744	3.65E-70	0.0409207	0.0838512	0.6255389	0.9348942
ENSG00000170231	FABP6	5	159640058	G	C	0.0765408	-0.00206456	0.0261728	0.7800007	0.0881045	0.0153846	1.02E-08	-0.0234331	0.297094	0.9371325	0.2832211
ENSG00000170234	PWWP2A	5	159517619	T	C	0.33996	-0.00944264	0.0148227	0.6800001	0.123236	0.00919023	5.33E-41	-0.0766227	0.120415	0.5245669	0.880254
ENSG00000170242	USP47	11	11921920	G	A	0.403579	-0.0252617	0.0143344	0.09299937	0.206851	0.00801929	1.03E-146	-0.122125	0.0694597	0.07871107	0.000402549
ENSG00000170248	PDCD6IP	3	33875519	T	G	0.233598	0.00219281	0.0161892	0.8700001	-0.484701	0.00845931	0	-0.00452405	0.0334005	0.8922572	0.6730149
ENSG00000170260	ZNF212	7	148944721	A	G	0.402584	0.00155176	0.0144234	0.8800001	0.287059	0.00804044	3.69E-279	0.00540572	0.0502457	0.9143243	0.2323412
ENSG00000170264	FAM161A	2	62066633	C	G	0.0367793	-0.0212035	0.039578	0.6499995	-0.230714	0.0336789	7.36E-12	0.0919039	0.17207	0.5932663	0.3379169
ENSG00000170265	ZNF282	7	148907958	C	T	0.421471	0.0157324	0.0146526	0.33	-0.288651	0.00813719	1.28E-275	-0.0545033	0.0507857	0.2831805	0.7865072
ENSG00000170266	GLB1	3	33088411	C	A	0.260437	-0.0203696	0.0168061	0.3400001	0.199849	0.00942647	9.39E-100	-0.101925	0.0842314	0.2262558	0.03772585
ENSG00000170270	GON7	14	93671339	C	T	0.161034	0.00128365	0.018717	0.9299999	-0.207362	0.0111905	1.18E-76	-0.00619038	0.090263	0.9453227	0.9374837
ENSG00000170271	FAXDC2	5	154218431	A	T	0.476143	-0.0102367	0.0142271	0.58	-0.476683	0.0081901	0	0.0214749	0.0298483	0.4718537	0.01343844
ENSG00000170275	CRTAP	3	33170312	T	C	0.17992	0.00743282	0.0185154	0.6300007	0.15479	0.0103327	9.83E-51	0.0480188	0.119659	0.6882023	0.363817
ENSG00000170291	ELP5	17	7158997	T	G	0.364811	-0.00954025	0.014554	0.7700005	-0.266549	0.00907238	9.80E-190	0.0357917	0.0546151	0.5122463	0.9708948
ENSG00000170293	CMTM8	3	32345994	A	C	0.128231	0.0085854	0.020578	0.6100002	0.826814	0.0207807	0	0.0103837	0.0248897	0.6765397	0.3066724
ENSG00000170298	LGALS9B	17	20361780	A	G	0.399602	0.01134	0.0145684	0.59	0.785069	0.0152777	0	0.0144446	0.018559	0.4363876	0.7525154
ENSG00000170310	STX8	17	9316848	C	G	0.262425	0.035501	0.0165335	0.01099993	0.453952	0.00909676	0	0.0782043	0.0364549	0.03193436	0.561691
ENSG00000170322	NFRKB	11	129749508	G	A	0.229622	0.0442112	0.016858	0.01700004	-0.269773	0.00932822	6.67E-184	-0.163883	0.062746	0.009005434	0.1770033
ENSG00000170340	B3GNT2	2	62437557	T	C	0.349901	-0.00111322	0.0147783	0.89	-0.412055	0.00803836	0	0.00270163	0.0358649	0.9399538	0.1361636
ENSG00000170345	FOS	14	75747205	T	C	0.152087	-0.0040711	0.0188082	0.8600001	0.0657276	0.0106576	6.95E-10	-0.061939	0.28633	0.8287381	0.4135449
ENSG00000170348	TMED10	14	75620753	G	A	0.218688	0.00557239	0.0164712	0.7199992	-0.132183	0.0140128	3.99E-21	-0.0421568	0.12469	0.7352925	0.1486798
ENSG00000170356	OR2A20P	7	143948594	C	T	0.274354	-0.0116842	0.018533	0.5700002	0.890585	0.0258045	5.17E-261	-0.0131197	0.0208134	0.5284665	0.6777583
ENSG00000170364	SETMAR	3	4352119	G	T	0.026839	-0.0247141	0.0371908	0.7300002	0.360885	0.0187846	2.95E-82	-0.0684819	0.103116	0.5066105	0.1281409
ENSG00000170365	SMAD1	4	146440788	A	G	0.496024	-0.0191707	0.01423	0.1	0.0899555	0.00792909	7.85E-30	-0.213113	0.159301	0.1809604	0.3242253
ENSG00000170385	SLC30A1	1	211748497	C	T	0.189861	-0.00178625	0.0178049	0.7899998	-0.0730594	0.00997316	2.38E-13	0.0244493	0.243727	0.920095	0.4674363
ENSG00000170390	DCLK2	4	151089017	G	A	0.224652	-0.00714536	0.0166817	0.7300002	0.486097	0.00936822	0	-0.0146994	0.0343188	0.6684177	0.6067741

ENSG00000170396	ZNF804A	2	185633656	G	A	0.139165	0.000149561	0.0198728	0.95	0.0763896	0.0110896	5.64E-12	0.00195787	0.260151	0.9939952	0.4641168
ENSG00000170412	GPRC5C	17	72434391	G	A	0.0536779	0.0199577	0.0267022	0.6300007	0.970288	0.0137914	0	0.0205688	0.0275214	0.4548368	0.1258329
ENSG00000170425	ADORA2B	17	15863645	C	T	0.429423	-0.0178921	0.0143664	0.2399999	0.404959	0.00842151	0	-0.0441825	0.0354881	0.2131334	0.6532357
ENSG00000170430	MGMT	10	131415859	C	T	0.380716	0.00552209	0.0147036	0.59	-0.714779	0.00730954	0	-0.0077256	0.020571	0.7072458	0.1947838
ENSG00000170439	TMT1B	12	56076862	A	G	0.256461	-0.0234345	0.0160956	0.09400046	-0.188569	0.00884537	7.66E-101	0.124275	0.0855553	0.1463423	0.5595137
ENSG00000170442	KRT86	12	52673015	A	G	0.171968	0.0115123	0.0187906	0.5199996	-0.257062	0.0114076	1.92E-112	-0.0447842	0.0731246	0.5402491	0.5111269
ENSG00000170445	HARS1	5	140062183	A	G	0.00397614	-0.297734	0.166032	0.05899973	1.46364	0.132928	3.39E-28	-0.20342	0.114932	0.0767415	0.5108472
ENSG00000170448	NFXL1	4	47882955	G	A	0.327038	-0.0207659	0.0152937	0.1800002	1.03082	0.006134	0	-0.020145	0.0148369	0.1745389	0.2916064
ENSG00000170456	DENND5B	12	31639594	T	C	0.382704	-0.0218127	0.0145051	0.1199999	-0.270555	0.00805007	1.24E-247	0.0806221	0.0536661	0.1330217	0.9410864
ENSG00000170458	CD14	5	140012299	G	A	0.234592	-0.00768098	0.0171065	0.64	-0.338727	0.00911088	1.53E-302	0.022676	0.050506	0.6534485	0.9301038
ENSG00000170464	DNAJC18	5	138762229	C	T	0.294235	-0.0101555	0.0161007	0.3700002	0.298133	0.00899264	5.13E-241	-0.0340637	0.0540149	0.5282793	0.6367792
ENSG00000170469	SPATA24	5	138736014	T	C	0.378728	-0.0154867	0.0151106	0.1800002	0.186553	0.00881706	2.32E-99	-0.083015	0.0810939	0.3059819	0.8929344
ENSG00000170473	PYM1	12	56310799	T	C	0.201789	0.00625162	0.017786	0.84	0.110188	0.0117187	5.31E-21	0.0567358	0.161527	0.725404	0.4731077
ENSG00000170484	KRT74	12	52963587	A	G	0.247515	0.0196281	0.0162931	0.2300001	-0.235304	0.0100876	2.42E-120	-0.0834159	0.069335	0.2289436	0.159759
ENSG00000170485	NPAS2	2	101524952	T	C	0.0576541	0.0247614	0.0306833	0.5300002	0.279648	0.0159079	3.56E-69	0.088545	0.109837	0.4201562	0.7912457
ENSG00000170486	KRT72	12	52987347	G	C	0.247515	0.0183236	0.016286	0.2599998	-0.765528	0.00778995	0	-0.0239359	0.0212756	0.2605721	0.2734731
ENSG00000170500	LONRF2	2	100914474	A	G	0.514911	0.00386966	0.0142117	0.8600001	-0.112453	0.00813672	1.92E-43	-0.0344114	0.126404	0.785442	0.03164968
ENSG00000170502	NUDT9	4	88362170	G	A	0.373757	-0.0041015	0.014925	0.5700002	0.17528	0.0081616	2.60E-102	-0.0233997	0.0851564	0.7834815	0.7742679
ENSG00000170509	HSD17B13	4	88234499	T	C	0.251491	-0.0436492	0.0158319	0.002900013	0.505337	0.00858699	0	-0.0863764	0.0313638	0.005886809	0.3612028
ENSG00000170522	ELOVL6	4	111043678	A	G	0.0318091	-0.0193023	0.0364881	0.4700002	-0.397628	0.0212606	4.71E-78	0.0485437	0.0918012	0.5969504	0.5549559
ENSG00000170525	PFKFB3	10	6232188	G	A	0.538767	-0.0154244	0.0143828	0.3900004	-0.112569	0.007957	1.94E-45	0.137021	0.128135	0.2849112	0.04545995
ENSG00000170540	ARL6IP1	16	18807995	G	A	0.39165	-0.000133005	0.0145295	0.9	-0.272004	0.00842174	7.51E-229	0.000488982	0.0534166	0.9926962	0.9737833
ENSG00000170542	SERPINB9	6	2895507	C	T	0.247515	-0.0231104	0.0170381	0.1100001	-0.515302	0.0101079	0	0.0448483	0.033076	0.175126	0.2760713
ENSG00000170545	SMAGP	12	51651667	T	C	0.102386	0.0152814	0.0215536	0.5700002	-0.275655	0.0121062	9.14E-115	-0.0554367	0.0782285	0.4785406	0.06356438
ENSG00000170558	CDH2	18	25644170	A	G	0.218688	0.0132557	0.0166559	0.2700001	-0.510905	0.00915658	0	-0.0259455	0.0326041	0.4261627	0.6584116
ENSG00000170571	EMB	5	49715554	C	T	0.332008	0.0324913	0.0148308	0.0129999	-0.334379	0.00976065	3.33E-257	-0.097169	0.0444438	0.02879127	0.3635198
ENSG00000170581	STAT2	12	56744660	C	T	0.0656064	0.0727182	0.0285226	0.004799986	-0.387329	0.0162511	1.48E-125	-0.187743	0.0740594	0.01124371	0.0473191
ENSG00000170584	NUDCD2	5	162883820	T	A	0.0377734	0.0510468	0.0323281	0.07799917	-0.151546	0.0199298	2.87E-14	-0.33684	0.217873	0.1220947	0.9889394
ENSG00000170604	IRF2BP1	19	46388121	C	T	0.17992	0.00298663	0.0180998	0.8499999	-0.0597555	0.0102662	5.86E-09	-0.0499808	0.303019	0.8689891	0.1573106
ENSG00000170606	HSPA4	5	132414897	A	G	0.0685885	0.0160984	0.0245849	0.5199996	-0.523833	0.0148771	1.38E-271	-0.0307319	0.0469408	0.512664	0.1661666
ENSG00000170615	SLC26A5	7	103039900	T	C	0.0606362	-0.00438154	0.0343032	0.84	0.96953	0.0197511	0	-0.00451924	0.0353814	0.8983632	0.8098937
ENSG00000170619	COMMD5	8	146072774	C	G	0.353877	0.0111782	0.0146609	0.5700002	0.250605	0.00866742	8.08E-184	0.0446048	0.0585223	0.4459497	0.1672798
ENSG00000170624	SGCD	5	155746076	C	A	0.365805	0.0119675	0.0146694	0.5199996	0.296883	0.0118964	1.86E-137	0.0403105	0.0494378	0.4148558	0.5377308
ENSG00000170627	GTSF1	12	54858560	C	G	0.133201	0.00597931	0.0192595	0.4799997	0.278252	0.0156933	2.43E-70	0.0214888	0.0692267	0.7562471	0.7374106
ENSG00000170629	NA	7	102868218	C	T	0.0248509	0.009448	0.0387146	0.7499995	-0.315328	0.024509	7.01E-38	-0.0299624	0.122798	0.8072321	0.9631345
ENSG00000170631	ZNF16	8	146166009	T	G	0.255467	-0.0254607	0.0167555	0.1199999	0.116728	0.0096509	1.12E-33	-0.218121	0.144672	0.1316337	0.3297324
ENSG00000170632	ARMC10	7	102727766	A	C	0.181909	1.37E-05	0.0186601	0.9699999	0.0857069	0.0106005	6.21E-16	0.000159545	0.21772	0.9994153	0.9961738
ENSG00000170633	RNF34	12	121853116	T	G	0.0795229	-0.00811998	0.0230515	0.5700002	0.459574	0.0124829	1.01E-296	-0.0176685	0.0501607	0.72466	0.9311389
ENSG00000170634	ACYP2	2	54437488	T	A	0.475149	0.00613318	0.0142725	0.5300002	-0.40421	0.00776556	0	-0.0151733	0.0353108	0.6674099	0.3400086
ENSG00000170638	TRABD	22	50631185	A	G	0.0586481	0.0586791	0.0276538	0.009599973	-0.839403	0.0205556	0	-0.0699058	0.0329891	0.03408546	0.51487
ENSG00000170647	NA	11	100863737	C	G	0.313121	-0.0164006	0.0154033	0.2200002	0.052996	0.0085854	6.71E-10	-0.309469	0.294943	0.2940625	0.7195692
ENSG00000170667	RASA4	7	102153551	C	T	0.270378	0.0304967	0.0159374	0.07100027	0.488776	0.0197082	8.83E-136	0.062394	0.0327037	0.05640932	0.7318815
ENSG00000170677	SOCS6	18	67976786	A	G	0.17495	0.0200438	0.0191164	0.29	-0.134331	0.0157116	1.23E-17	-0.149212	0.143374	0.2980071	0.3195762
ENSG00000170727	NA	8	145500568	A	G	0.403579	0.00256556	0.0144652	0.9599999	-0.13382	0.0178232	5.99E-14	-0.0191717	0.108125	0.8592638	0.6312865
ENSG00000170734	POLH	6	43565294	G	A	0.026839	0.021411	0.0422464	0.5	0.224283	0.0264609	2.33E-17	0.0954642	0.188698	0.6129207	0.6569952
ENSG00000170759	KIF5B	10	32321648	A	G	0.114314	-0.0211548	0.0217117	0.2200002	0.365534	0.0120413	2.05E-202	-0.0578737	0.0594278	0.3301317	0.09264368
ENSG00000170776	AKAP13	15	86108194	C	T	0.424453	0.00529314	0.014462	0.6700003	0.239387	0.00789062	3.60E-202	0.0221112	0.060417	0.7143825	0.1632796
ENSG00000170779	CDCA4	14	105481697	A	G	0.319085	0.0215311	0.0157545	0.16	-0.22977	0.00843142	1.59E-163	-0.093707	0.0686524	0.1722693	0.359515
ENSG00000170791	CHCHD7	8	57127801	T	C	0.228628	0.015834	0.0167775	0.2200002	-0.184496	0.00999792	4.90E-76	-0.0858232	0.091056	0.3459203	0.4819906
ENSG00000170801	HTRA3	4	8290165	G	A	0.487078	-0.00789437	0.0144431	0.6200004	0.152174	0.00915377	4.66E-62	-0.0518772	0.0949629	0.5848673	0.08208662

ENSG00000170802	FOXN2	2	48574104	A	G	0.12326	-0.0104236	0.0215994	0.56	0.768003	0.0183131	0	-0.0135724	0.028126	0.6294114	0.1455355
ENSG00000170819	BFSP2	3	133156452	G	A	0.425447	0.000855421	0.0147444	0.91	0.175428	0.0121382	2.41E-47	0.00487619	0.0840488	0.9537357	0.7840821
ENSG00000170835	CEL	9	135942306	G	A	0.382704	0.019352	0.014678	0.2200002	0.141629	0.0085895	4.43E-61	0.136639	0.103968	0.1887651	0.7335991
ENSG00000170837	GPR27	3	71804424	A	G	0.176938	0.0027793	0.0189493	0.89	0.246817	0.0100175	4.88E-134	0.0112606	0.076776	0.8833943	0.2245526
ENSG00000170846	MRFAP1L2	4	6676476	G	A	0.0367793	0.002993	0.0327094	0.9599999	0.884467	0.0199414	0	0.00338396	0.0369821	0.9270933	0.5425144
ENSG00000170852	KBTBD2	7	32920763	C	T	0.432406	0.0118913	0.014437	0.2399999	0.418107	0.00778945	0	0.0284408	0.0345335	0.4101831	0.3592666
ENSG00000170854	RIOX2	3	97677193	T	C	0.284294	-0.00433159	0.0156415	0.7899998	0.144071	0.0086584	3.61E-62	-0.0300658	0.108583	0.7818638	0.140928
ENSG00000170855	TRIAP1	12	120882989	T	C	0.0606362	-0.0441106	0.0306494	0.1199999	0.108042	0.0163269	3.66E-11	-0.408274	0.290313	0.159628	0.80419
ENSG00000170866	NA	19	54804903	G	A	0.17992	0.028833	0.0170216	0.0129999	-1.04998	0.00991008	0	-0.0274605	0.0162134	0.09032418	0.1387019
ENSG00000170871	KIAA0232	4	6834499	C	T	0.0984095	-0.00903031	0.0264816	0.6200004	-0.389833	0.013632	7.36E-180	0.0231646	0.0679355	0.7331194	0.8024175
ENSG00000170873	MTSS1	8	125651880	C	T	0.333996	-0.0319065	0.0150274	0.05699936	0.378313	0.00834485	0	-0.0843389	0.0397657	0.03393049	0.7439962
ENSG00000170876	TMEM43	3	14175809	C	T	0.135189	-0.0406916	0.0225314	0.04600023	0.173585	0.012139	2.19E-46	-0.234419	0.130832	0.0731711	0.7895264
ENSG00000170881	RNF139	8	125493567	A	G	0.223658	-0.00568472	0.0174944	0.9	0.14435	0.0150302	7.69E-22	-0.0393815	0.121264	0.7453633	0.4092802
ENSG00000170889	RPS9	19	54728736	C	T	0.421471	0.00258065	0.0144324	0.6499995	-0.754277	0.0158701	0	-0.00342136	0.0191342	0.8580883	0.70743
ENSG00000170891	CYTL1	4	5018756	T	G	0.134195	-0.0196407	0.0198179	0.2	0.560871	0.0107311	0	-0.0350182	0.0353405	0.321744	0.3794639
ENSG00000170892	TSEN34	19	54695687	T	A	0.276342	-0.00907217	0.0158423	0.2200002	-0.305162	0.00832472	3.49E-294	0.029729	0.0519207	0.5669257	0.443113
ENSG00000170899	GSTA4	6	52851463	T	C	0.500994	0.00434565	0.0142334	0.7400005	-0.184979	0.00794892	8.72E-120	-0.0234926	0.0769525	0.7601474	0.6878434
ENSG00000170903	MSANTD4	11	105879740	C	T	0.365805	-0.011486	0.0147851	0.4500005	-0.15453	0.00907967	5.90E-65	0.0743284	0.0957772	0.4377164	0.4084367
ENSG00000170906	NDUFA3	19	54609300	G	A	0.0994036	-0.0138251	0.0239374	0.7199992	0.764205	0.0242	7.22E-219	-0.0180908	0.0313285	0.5636316	0.4145908
ENSG00000170909	OSCAR	19	54601966	T	C	0.483101	-0.00312406	0.0142209	0.9299999	0.593474	0.00738243	0	-0.00526402	0.0239622	0.8261203	0.8528686
ENSG00000170915	PAQR8	6	52249397	T	C	0.275348	0.00964303	0.0155307	0.35	-0.505371	0.00838938	0	-0.0190811	0.0307329	0.5346863	0.8890766
ENSG00000170917	NUDT6	4	123828926	G	C	0.132207	0.0195676	0.0207338	0.33	0.281864	0.0118737	1.44E-124	0.0694221	0.0736177	0.3456764	0.8563671
ENSG00000170919	NA	13	45940676	A	G	0.132207	-0.0392978	0.0205278	0.02800013	0.390805	0.0155432	1.68E-139	-0.100556	0.052679	0.0562821	0.1247489
ENSG00000170921	TANC2	17	61295988	T	C	0.45328	-0.00735353	0.0142232	0.6700003	0.418577	0.00929789	0	-0.0175679	0.0339821	0.6051733	0.274501
ENSG00000170949	ZNF160	19	53588274	G	A	0.0964215	0.0461833	0.0212379	0.03099988	0.227756	0.012927	1.77E-69	0.202776	0.0939562	0.03091279	0.2419831
ENSG00000170954	ZNF415	19	53623731	A	G	0.132207	-0.0116533	0.0216371	0.6100002	0.579041	0.012065	0	-0.0201252	0.0373695	0.5902004	0.7330478
ENSG00000170955	CAVIN3	11	6341026	C	T	0.233598	0.0253231	0.0166293	0.07000032	0.65292	0.00995589	0	0.0387844	0.025476	0.1279114	0.7893488
ENSG00000170956	CEACAM3	19	42307980	G	A	0.535785	-0.00282876	0.0142027	0.5500004	-0.094847	0.00861009	3.21E-28	0.0298245	0.149768	0.8421548	0.6240307
ENSG00000170962	PDGFD	11	103906510	A	G	0.112326	-0.00335814	0.0228677	0.8200001	-0.280507	0.0125832	4.40E-110	0.0119717	0.0815245	0.8832523	0.3632866
ENSG00000170989	S1PR1	1	101704759	C	T	0.392644	0.0161212	0.0144665	0.2300001	-0.108493	0.00893431	6.22E-34	-0.148592	0.133901	0.2671203	0.8571365
ENSG00000171033	PKIA	8	79472938	T	G	0.199801	0.00821996	0.0186019	0.7099994	-0.758262	0.00945184	0	-0.0108405	0.0245326	0.658575	0.8375025
ENSG00000171044	XKR6	8	10906215	A	T	0.38171	-0.0204537	0.0144768	0.28	0.168228	0.00810876	1.32E-95	-0.121584	0.0862542	0.1586591	0.2858603
ENSG00000171045	TSNARE1	8	143389021	T	C	0.350895	0.00946376	0.0148111	0.3800004	0.193708	0.00835754	7.66E-119	0.0488559	0.0764901	0.5230042	0.1026238
ENSG00000171049	FPR2	19	52264529	G	T	0.40159	-0.0153498	0.0145582	0.4899999	-0.706067	0.00713873	0	0.0217399	0.0206199	0.2917387	0.0689744
ENSG00000171051	FPR1	19	52277894	T	C	0.335984	-0.00167646	0.0150788	0.7800007	-0.4844	0.00812113	0	0.0034609	0.0311289	0.9114738	0.2670066
ENSG00000171055	FEZ2	2	36825900	G	T	0.406561	0.0199706	0.0146518	0.1199999	0.652938	0.00743375	0	0.0305857	0.0224425	0.17293	0.4280584
ENSG00000171056	SOX7	8	10639317	A	G	0.387674	0.0303773	0.0146445	0.09299937	0.0514536	0.0084714	1.25E-09	0.590383	0.300756	0.04964685	0.09843538
ENSG00000171067	C11orf24	11	68034136	C	T	0.240557	0.00334237	0.0164122	0.8	0.127829	0.00926282	2.54E-43	0.0261473	0.128406	0.8386431	0.207491
ENSG00000171084	NA	3	125642165	T	G	0.192843	-0.0108425	0.0186774	0.6200004	0.208813	0.0146502	4.28E-46	-0.0519245	0.0895199	0.561892	0.3725552
ENSG00000171097	KYAT1	9	131619997	A	C	0.295229	-0.00131553	0.0157206	0.9400001	0.187652	0.00879449	5.11E-101	-0.00701049	0.0837761	0.9333098	0.6908643
ENSG00000171101	NA	19	51673682	A	G	0.192843	-0.0306422	0.0189966	0.06800017	0.136759	0.0132493	5.60E-25	-0.22406	0.140591	0.1110046	0.07575825
ENSG00000171103	TRMT61B	2	29082927	A	C	0.411531	-0.000425073	0.0143513	0.8200001	0.256948	0.0118332	1.51E-104	-0.00165431	0.055853	0.9763708	0.4968424
ENSG00000171105	INSR	19	7203155	G	A	0.258449	0.0286355	0.0177444	0.1900002	0.0867737	0.009791	7.82E-19	0.330002	0.207853	0.1123608	0.8713029
ENSG00000171109	MFN1	3	179089099	C	T	0.0387674	-0.0559681	0.0353344	0.1299999	0.931277	0.0218156	0	-0.0600982	0.037968	0.1134521	0.2139145
ENSG00000171115	GIMAP8	7	150162099	C	T	0.421471	0.00846453	0.0144577	0.5700002	-0.309768	0.00781865	0	-0.0273254	0.0466778	0.5582756	0.935772
ENSG00000171121	KCNMB3	3	178971160	T	C	0.194831	-0.0324753	0.0183162	0.05899973	0.113471	0.0114236	2.99E-23	-0.2862	0.163969	0.08090689	0.5170913
ENSG00000171130	ATP6V0E2	7	149573922	A	C	0.432406	0.0205688	0.0145023	0.2700001	0.457089	0.00767482	0	0.0449995	0.0317365	0.1562168	0.5666934
ENSG00000171132	PRKCE	2	46146806	A	G	0.262425	-0.0175666	0.0154765	0.1900002	0.120534	0.00890009	8.72E-42	-0.14574	0.12885	0.2580196	0.3099153
ENSG00000171135	JAGN1	3	9934135	G	A	0.446322	0.00956001	0.0142342	0.4	0.245337	0.00862597	6.17E-178	0.0389668	0.0580351	0.5019432	0.5346988
ENSG00000171148	TADA3	3	9828119	T	C	0.11829	0.00905338	0.0192991	0.7099994	0.441586	0.0101526	0	0.020502	0.0437066	0.6390113	0.7108435

ENSG00000171150	SOCS5	2	46958179	C	A	0.359841	-0.0106843	0.0149518	0.3599996	0.108533	0.00830931	5.46E-39	-0.098443	0.137969	0.4755266	0.9801329
ENSG00000171159	BBLN	9	130924373	A	C	0.32505	-0.0280165	0.0152635	0.07799917	-0.121372	0.00856423	1.37E-45	0.230831	0.126808	0.06871053	0.7149795
ENSG00000171160	MORN4	10	99383827	A	G	0.213718	-0.02926	0.0168789	0.14	-0.269747	0.00960891	2.12E-173	0.108472	0.0626922	0.08358927	0.5803064
ENSG00000171161	ZNF672	1	249138062	G	C	0.145129	-0.0183261	0.019638	0.35	0.0935574	0.0107872	4.21E-18	-0.195881	0.211115	0.3534904	0.8635822
ENSG00000171163	ZNF692	1	249148774	G	A	0.208748	-0.0128361	0.0177754	0.6100002	0.0729918	0.0112949	1.03E-10	-0.175857	0.245042	0.4729666	0.9749506
ENSG00000171174	RBKS	2	28059098	T	C	0.0596421	0.00857733	0.0368363	0.99	-0.212677	0.0193748	4.93E-28	-0.0403304	0.173242	0.8159186	0.7442672
ENSG00000171189	GRIK1	21	31110802	C	T	0.352883	-0.00853324	0.0146095	0.7300002	-0.142806	0.00843566	2.76E-64	0.0597543	0.102364	0.5593945	0.3683364
ENSG00000171204	TMEM126B	11	85343604	G	A	0.534791	0.00302111	0.0142519	0.7300002	0.0621051	0.00798102	7.16E-15	0.0486451	0.229565	0.8321844	0.7224888
ENSG00000171206	TRIM8	10	104411164	T	C	0.390656	0.0113405	0.0143297	0.5400003	-0.235676	0.00798626	2.13E-191	-0.048119	0.0608244	0.4288776	0.4766309
ENSG00000171208	NETO2	16	47144761	G	T	0.0467197	-0.0969099	0.0403724	0.0179999	0.131425	0.0172859	2.89E-14	-0.73738	0.322137	0.02207778	0.03497287
ENSG00000171217	CLDN20	6	155591414	T	C	0.267396	0.0281552	0.016464	0.1100001	0.0728356	0.0091941	2.34E-15	0.386558	0.23125	0.09460267	0.2922147
ENSG00000171219	CDC42BPG	11	64601450	C	A	0.125249	-0.0362649	0.0240257	0.09099971	0.142898	0.0171078	6.67E-17	-0.253781	0.170855	0.1374476	0.4977377
ENSG00000171222	SCAND1	20	34544466	T	C	0.105368	0.0461253	0.0236913	0.05600025	-0.449079	0.0126117	1.05E-277	-0.102711	0.0528341	0.05189229	0.2506518
ENSG00000171223	JUNB	19	12903217	A	G	0.37674	-0.00155378	0.0149766	0.6800001	0.0571895	0.00881629	8.77E-11	-0.027169	0.26191	0.9173805	0.5156468
ENSG00000171224	FAM241B	10	71391679	T	C	0.209742	0.0289746	0.0168662	0.14	-0.241089	0.00948421	1.52E-142	-0.120182	0.0701181	0.08652881	0.59002
ENSG00000171236	LRG1	19	4538262	A	G	0.124254	0.00338328	0.0234196	0.7800007	-0.0791328	0.0140936	1.97E-08	-0.0427544	0.296051	0.8851721	0.9369389
ENSG00000171246	NPTX1	17	78446295	C	T	0.180915	-0.0144914	0.0180089	0.3800004	-0.105647	0.0103842	2.60E-24	0.137168	0.170995	0.4224519	0.669177
ENSG00000171262	FAM98B	15	38763119	C	T	0.257455	0.0039446	0.0163096	0.6999999	0.122948	0.00932836	1.14E-39	0.0320836	0.132677	0.8089221	0.9721363
ENSG00000171282	NA	17	79403448	C	T	0.214712	-0.0146619	0.0178766	0.4899999	0.206463	0.0129084	1.40E-57	-0.0710147	0.0866989	0.4127313	0.223963
ENSG00000171291	ZNF439	19	11977053	T	C	0.115308	-0.00856824	0.0222453	0.4100001	-0.329246	0.0130481	1.73E-140	0.0260238	0.0675722	0.7001441	0.2496364
ENSG00000171295	ZNF440	19	11935557	A	G	0.441352	0.0110973	0.0142663	0.58	-0.0679301	0.00803469	2.80E-17	-0.163364	0.210901	0.4385781	0.5742991
ENSG00000171298	GAA	17	78084516	C	T	0.246521	-0.000825478	0.0161449	0.8600001	0.698954	0.00829167	0	-0.00118102	0.0230987	0.9592225	0.4850844
ENSG00000171302	CANT1	17	76996874	C	T	0.106362	-0.0144417	0.0222207	0.5400003	-0.313111	0.0120276	2.11E-149	0.0461232	0.0709896	0.515874	0.6298071
ENSG00000171307	ZDHHC16	10	99211527	C	T	0.136183	0.0145809	0.0194733	0.3400001	-0.0862203	0.0114163	4.27E-14	-0.169112	0.226962	0.4562048	0.9970486
ENSG00000171310	CHST11	12	105002432	C	T	0.470179	0.00985166	0.0142513	0.4400003	-0.123698	0.00793577	8.87E-55	-0.0796429	0.115324	0.4898153	0.4992166
ENSG00000171311	EXOSC1	10	99200836	C	T	0.289264	-0.0126904	0.0157552	0.4799997	0.169375	0.00882181	3.73E-82	-0.0749248	0.0931014	0.4209548	0.9833263
ENSG00000171314	PGAM1	10	99189557	T	C	0.05666	0.0160716	0.0281866	0.5300002	-0.99563	0.0339455	4.27E-189	-0.0161421	0.0283157	0.5686247	0.7936865
ENSG00000171316	CHD7	8	61685401	G	A	0.446322	-0.0125796	0.0142457	0.33	-0.220769	0.00873463	5.99E-141	0.0569807	0.0645669	0.3775027	0.3752231
ENSG00000171368	TPPP	5	677196	T	G	0.190855	-0.00665799	0.0177582	0.9400001	-0.167194	0.0104653	1.88E-57	0.0398219	0.106242	0.7077931	0.6005401
ENSG00000171403	KRT9	17	39725203	T	C	0.0149105	-0.043597	0.0663044	0.56	0.387906	0.0519451	8.17E-14	-0.112391	0.171591	0.5124717	0.4286753
ENSG00000171408	PDE7B	6	136344773	C	A	0.352883	-0.00777533	0.0149491	0.7899998	0.0748634	0.00845208	8.19E-19	-0.10386	0.200029	0.6036033	0.670277
ENSG00000171421	MRPL36	5	1799990	C	T	0.356859	0.0181661	0.0146367	0.1800002	0.588166	0.0168424	3.47E-267	0.030886	0.024901	0.2148458	0.2937483
ENSG00000171425	ZNF581	19	56151906	G	A	0.109344	-0.00826848	0.0201629	0.56	-0.149364	0.0125112	7.46E-33	0.0553581	0.135072	0.6819217	0.5199982
ENSG00000171428	NAT1	8	18054592	C	T	0.475149	-0.00243595	0.0143292	0.89	0.104111	0.00797467	5.94E-39	-0.0233976	0.137646	0.8650222	0.6789207
ENSG00000171443	ZNF524	19	56112946	C	T	0.156064	-0.0131845	0.0183918	0.4299995	0.109317	0.0109928	2.67E-23	-0.120608	0.168679	0.474601	0.9773556
ENSG00000171444	MCC	5	112591161	G	A	0.496024	0.0222231	0.014205	0.08999948	-0.278529	0.00779485	1.24E-279	-0.0797872	0.0510488	0.1180623	0.6224411
ENSG00000171451	DSEL	18	65179018	T	G	0.50994	0.00318673	0.0142842	0.7800007	0.154616	0.0117794	2.34E-39	0.0206106	0.0923983	0.8234868	0.9374126
ENSG00000171453	POLR1C	6	43487381	T	C	0.0337972	-0.020919	0.0410647	0.4700002	-0.194995	0.0237088	1.96E-16	0.10728	0.210997	0.6111439	0.8642341
ENSG00000171456	ASXL1	20	30986638	G	T	0.349901	0.0299307	0.0146543	0.03799969	0.17568	0.00804446	9.98E-106	0.170371	0.0837789	0.04199423	0.802314
ENSG00000171462	DLK2	6	43421230	A	G	0.286282	-0.0153393	0.0154558	0.2700001	-0.250886	0.00861869	2.72E-186	0.0611406	0.0616407	0.3212531	0.5402712
ENSG00000171466	ZNF562	19	9772553	T	C	0.054672	0.0269191	0.031445	0.5500004	0.160306	0.0177008	1.35E-19	0.167924	0.197031	0.3940642	0.8411362
ENSG00000171467	ZNF318	6	43318347	A	C	0.0894632	-0.0245536	0.0232596	0.2700001	0.509128	0.0131428	0	-0.0482268	0.0457021	0.291315	0.1741393
ENSG00000171469	ZNF561	19	9723715	A	G	0.154076	0.0133935	0.0204277	0.5	-0.713663	0.0183632	0	-0.0187673	0.0286278	0.5121069	0.310149
ENSG00000171476	HOPX	4	57531110	C	A	0.21173	-0.0368422	0.0173915	0.02199986	-0.204022	0.0101379	4.48E-90	0.180579	0.0857141	0.03513813	0.4867956
ENSG00000171488	LRRC8C	1	90248720	G	A	0.313121	0.0165248	0.0149531	0.25	-0.45242	0.00788922	0	-0.0365253	0.0330575	0.2692014	0.04950284
ENSG00000171490	RSL1D1	16	11937249	G	A	0.0308151	0.0570108	0.0577399	0.3599996	-0.169678	0.0276944	8.97E-10	-0.335994	0.344682	0.3296617	0.8333737
ENSG00000171492	LRRC8D	1	90344371	C	T	0.399602	0.0159512	0.0143612	0.2399999	0.325488	0.0078467	0	0.0490071	0.0441379	0.2668624	0.1309533
ENSG00000171497	PPID	4	159637417	C	G	0.293241	-0.00864599	0.01552	0.5999997	0.0727307	0.00886852	2.38E-16	-0.118877	0.213882	0.5783434	0.2373772
ENSG00000171502	COL24A1	1	86408771	G	A	0.0198807	-0.00520387	0.0503402	0.7199992	-0.270258	0.0291176	1.67E-20	0.0192552	0.186279	0.9176712	0.7851975
ENSG00000171503	ETFDH	4	159612026	T	C	0.245527	-0.00722339	0.0159519	0.6600001	0.36874	0.00976302	0	-0.0195894	0.0432636	0.6506995	0.1134427

ENSG00000171517	LPAR3	1	85318090	G	A	0.276342	0.0260832	0.016152	0.1199999	0.302948	0.00878929	2.44E-260	0.0860981	0.0533746	0.1067251	0.858239
ENSG00000171522	PTGER4	5	40686718	G	A	0.225646	0.0128787	0.0177282	0.3599996	-0.270226	0.00961927	1.22E-173	-0.0476589	0.0656269	0.46771	0.7545294
ENSG00000171530	TBCA	5	77075797	T	G	0.342942	0.00244305	0.0154379	0.99	0.333701	0.0158516	2.21E-98	0.00732108	0.046264	0.8742632	0.7743934
ENSG00000171552	BCL2L1	20	30282023	T	C	0.232604	0.0285477	0.0173832	0.089	-0.0738765	0.0101814	3.99E-13	-0.386425	0.241252	0.109212	0.7366421
ENSG00000171574	ZNF584	19	58921282	T	C	0.248509	0.0140005	0.017172	0.4500005	-0.278482	0.00956567	2.49E-186	-0.0502744	0.0616872	0.4150779	0.2350566
ENSG00000171595	DNAI2	17	72290704	A	G	0.143141	-0.0270684	0.0194166	0.1199999	0.290516	0.010713	6.04E-162	-0.0931736	0.0669232	0.1638476	0.8634737
ENSG00000171596	NMUR1	2	232391538	C	T	0.347913	-0.00515022	0.0148405	0.5199996	-0.172821	0.00815246	9.84E-100	0.0298009	0.0858837	0.7285976	0.02245312
ENSG00000171603	CLSTN1	1	9836834	T	C	0.0129225	-0.0166403	0.0504068	0.89	-1.03036	0.0431097	3.00E-126	0.01615	0.0489263	0.741333	0.5636438
ENSG00000171604	CXXC5	5	139045175	T	C	0.294235	0.00386059	0.0154345	0.8200001	-0.111461	0.00865156	5.58E-38	-0.0346361	0.1385	0.8025253	0.4118616
ENSG00000171606	ZNF274	19	58709662	G	A	0.0228628	0.0264562	0.0504912	0.6300007	-0.19974	0.0319635	4.13E-10	-0.132453	0.253671	0.60157	0.5400346
ENSG00000171608	PIK3CD	1	9750481	A	G	0.170974	-0.0130758	0.0189581	0.5400003	0.0751479	0.0102337	2.09E-13	-0.174001	0.253388	0.4922727	0.8997085
ENSG00000171611	PTCRA	6	42888650	G	A	0.26839	-0.0061762	0.0167511	0.5500004	-0.0852871	0.00950231	2.82E-19	0.0724166	0.196574	0.7125799	0.2503577
ENSG00000171617	ENC1	5	73930241	C	G	0.11332	0.0190445	0.0211557	0.3599996	1.14306	0.010185	0	0.0166609	0.0185085	0.3680257	0.3942968
ENSG00000171621	SPSB1	1	9391265	A	G	0.354871	0.00812888	0.0150925	0.7800007	0.303789	0.0094048	6.66E-229	0.0267583	0.0496878	0.5902118	0.5973545
ENSG00000171631	P2RY6	11	72992606	A	G	0.166004	0.0330899	0.0194944	0.05600025	0.0646146	0.0112221	8.52E-09	0.512112	0.31454	0.1034972	0.2006071
ENSG00000171634	BPTF	17	65901067	G	C	0.281312	-0.0086486	0.016251	0.7099994	0.0764449	0.0096908	3.06E-15	-0.113135	0.213068	0.5954314	0.5357727
ENSG00000171643	S100Z	5	76181650	A	T	0.266402	-0.00272759	0.0161565	0.7899998	0.256487	0.00890594	2.18E-182	-0.0106344	0.0629927	0.8659381	0.8485608
ENSG00000171649	ZIK1	19	58097484	A	C	0.301193	-0.0259094	0.015863	0.05399953	-0.216087	0.00960857	5.32E-112	0.119903	0.0736036	0.1033062	0.04239503
ENSG00000171658	NA	3	185688195	T	C	0.447316	0.0185018	0.0142912	0.29	0.197086	0.0113459	1.38E-67	0.0938769	0.0727137	0.196687	0.1497865
ENSG00000171680	PLEKHG5	1	6553136	A	G	0.136183	0.0426713	0.0211262	0.05	-0.335215	0.0118957	1.04E-174	-0.127295	0.0631846	0.04394031	0.7723595
ENSG00000171681	ATF7IP	12	14585153	G	A	0.526839	-0.025872	0.0142131	0.03599979	0.0939714	0.00795114	3.13E-32	-0.275318	0.153033	0.07200622	0.2663429
ENSG00000171703	TCEA2	20	62692444	T	G	0.232604	-0.00366353	0.0165486	0.8800001	-0.174837	0.0107678	2.76E-59	0.020954	0.0946605	0.8248125	0.6941354
ENSG00000171714	ANO5	11	22259812	T	G	0.203777	0.0101117	0.0181142	0.5400003	0.286699	0.00995512	2.20E-182	0.0352694	0.0631939	0.5767661	0.4762555
ENSG00000171720	HDAC3	5	141008440	T	G	0.417495	0.0105359	0.0145502	0.3400001	-0.123642	0.0121846	3.40E-24	-0.0852128	0.117979	0.4701283	0.4914109
ENSG00000171723	GPHN	14	67311322	G	A	0.198807	0.00423266	0.0168866	0.8	0.091173	0.00972803	7.10E-21	0.0464245	0.185281	0.8021525	0.8029037
ENSG00000171729	TMEM51	1	15513002	C	A	0.176938	-0.00887178	0.0197953	0.6100002	0.90226	0.00976439	0	-0.00983285	0.02194	0.6540299	0.4936348
ENSG00000171735	CAMTA1	1	7337575	T	C	0.198807	0.0315264	0.0178779	0.05699936	0.304831	0.00983523	6.53E-211	0.103423	0.0587434	0.07830931	0.1593957
ENSG00000171747	LGALS4	19	39298157	G	C	0.279324	-0.00236693	0.0153759	0.7099994	0.18869	0.00847164	6.72E-110	-0.012544	0.0814894	0.8776618	0.2439415
ENSG00000171757	LRRC34	3	169520995	T	C	0.131213	-0.0169779	0.0224752	0.3400001	-0.0909377	0.0129287	2.01E-12	0.186698	0.248571	0.4526004	0.427045
ENSG00000171763	AFG2B	15	45704073	C	A	0.274354	0.016218	0.0162362	0.4299995	0.868275	0.00742179	0	0.0186784	0.0187001	0.3178709	0.9182492
ENSG00000171766	GATM	15	45673869	G	A	0.275348	0.0169248	0.0162397	0.4100001	-0.576781	0.00822595	0	-0.0293436	0.0281589	0.2973785	0.9882006
ENSG00000171777	RASGRP4	19	38908320	A	G	0.0586481	-0.014738	0.0295553	0.5	-0.466701	0.0169991	6.14E-166	0.0315791	0.0633385	0.618078	0.9383131
ENSG00000171786	NHLH1	1	160339747	G	C	0.355865	-0.000322007	0.0146061	0.9599999	0.0480621	0.00826093	5.96E-09	-0.00669981	0.303903	0.9824113	0.1894535
ENSG00000171790	SLFNL1	1	41485089	C	T	0.054672	0.055942	0.0325004	0.1800002	-0.160718	0.0186297	6.30E-18	-0.348075	0.206206	0.09141116	0.8478068
ENSG00000171791	BCL2	18	60888970	C	T	0.196819	-0.0148428	0.01737	0.2700001	0.0636455	0.00970176	5.37E-11	-0.233211	0.275224	0.3968003	0.00027417
ENSG00000171792	RHNO1	12	2992025	G	A	0.348907	0.00218979	0.0149215	0.91	-0.542054	0.00860321	0	-0.0040398	0.0275278	0.8833265	0.377369
ENSG00000171793	CTPS1	1	41461621	T	G	0.314115	-0.011541	0.015336	0.5300002	-0.356184	0.0091878	0	0.0324018	0.0430645	0.4518098	0.4489709
ENSG00000171794	UTF1	10	135044420	A	C	0.135189	0.00371806	0.0220175	0.84	0.23415	0.0141146	8.34E-62	0.015879	0.0940366	0.865907	0.2273934
ENSG00000171798	KNDC1	10	135006933	A	T	0.16004	0.0191243	0.0199193	0.28	0.371808	0.0129168	3.32E-182	0.051436	0.053604	0.337279	0.4883259
ENSG00000171806	METTL18	1	169762888	C	A	0.0964215	-0.0104715	0.0243018	0.7199992	-0.818372	0.0143612	0	0.0127955	0.0296962	0.6665544	0.9976721
ENSG00000171811	CFAP46	10	134689111	A	G	0.455268	-0.0011438	0.0143197	0.9599999	0.12345	0.0107971	2.84E-30	-0.00926531	0.115999	0.9363374	0.6426407
ENSG00000171812	COL8A2	1	36575829	T	G	0.0208748	0.0229614	0.0466998	0.7800007	0.213406	0.0290372	1.99E-13	0.107595	0.21932	0.6237205	0.05931916
ENSG00000171813	PWWP2B	10	134221019	T	A	0.187873	0.0238704	0.0167159	0.1100001	-0.118524	0.00984942	2.37E-33	-0.201397	0.142023	0.1561744	0.9163999
ENSG00000171817	ZNF540	19	38073653	T	C	0.218688	-0.0118583	0.0176775	0.4700002	-0.0822817	0.00973724	2.91E-17	0.144118	0.215517	0.5036808	0.5862864
ENSG00000171823	FBXL14	12	1689245	C	T	0.276342	-0.00990867	0.015674	0.5500004	0.053018	0.0089709	3.42E-09	-0.186893	0.297322	0.5296198	0.08683288
ENSG00000171824	EXOSC10	1	11143306	C	T	0.258449	0.0196192	0.0162215	0.35	0.119983	0.00906205	5.14E-40	0.163516	0.135761	0.2284188	0.4279796
ENSG00000171840	NINJ2	12	723203	A	G	0.364811	0.00425245	0.014731	0.91	-0.401227	0.00800806	0	-0.0105986	0.0367155	0.7728349	0.5510143
ENSG00000171843	MLLT3	9	20482102	C	T	0.228628	-0.0301018	0.0165785	0.05899973	-0.127709	0.00929669	6.09E-43	0.235707	0.130944	0.07185163	0.4852237
ENSG00000171847	FAM90A1	12	8377035	T	C	0.308151	0.0152676	0.0182741	0.3900004	0.295585	0.0179851	1.07E-60	0.0516522	0.0619034	0.4040556	0.5766269
ENSG00000171853	TRAPPC12	2	3436155	T	G	0.410537	0.00171314	0.0147092	0.99	0.550741	0.00848441	0	0.00311061	0.0267081	0.9072824	0.8274711

ENSG00000171860	C3AR1	12	8214982	A	G	0.309145	-0.00673578	0.0153976	0.8	-0.919768	0.00670079	0	0.00732334	0.0167408	0.6617819	0.7992873
ENSG00000171861	MRM3	17	690631	A	G	0.109344	-0.00227718	0.0216936	0.7600007	0.225058	0.0123676	5.41E-74	-0.0101182	0.0963928	0.9164009	0.6468474
ENSG00000171862	PTEN	10	89677278	G	C	0.247515	0.0138258	0.0162657	0.35	0.114616	0.0173913	4.39E-11	0.120628	0.143091	0.3992203	0.6278931
ENSG00000171863	RPS7	2	3625652	T	C	0.0487078	-0.0117665	0.0340127	0.5400003	0.203778	0.0241152	2.91E-17	-0.0577416	0.16705	0.7296022	0.4105468
ENSG00000171865	RNASEH1	2	3599294	A	G	0.204771	-0.0229859	0.0186643	0.33	-0.096191	0.0103607	1.63E-20	0.238961	0.195733	0.222143	0.7743849
ENSG00000171867	PRNP	20	4674559	G	A	0.479125	0.0120452	0.0142598	0.3700002	0.169038	0.00791059	2.62E-101	0.0712572	0.0844243	0.3986487	0.6781564
ENSG00000171914	TLN2	15	62909777	A	G	0.248509	-0.00489814	0.016355	0.9699999	0.0708611	0.00889902	1.68E-15	-0.0691231	0.230967	0.7647285	0.05046688
ENSG00000171916	LGALS9C	17	18389155	G	T	0.446322	0.0183253	0.0142148	0.14	-0.828576	0.0145537	0	-0.0221166	0.0171601	0.1974537	0.1152175
ENSG00000171928	TVP23B	17	18697167	G	A	0.497018	-0.0169695	0.0141922	0.17	-0.487561	0.0178997	2.28E-163	0.0348049	0.0291366	0.2322662	0.2907366
ENSG00000171940	ZNF217	20	52205025	G	A	0.0397614	0.0609845	0.0410774	0.1199999	0.654848	0.0225443	1.67E-185	0.0931278	0.0628101	0.1381571	0.9289613
ENSG00000171943	SRGAP2C	1	121118536	G	A	0.38668	0.00575175	0.0159877	0.84	0.630698	0.0175888	1.38E-281	0.00911966	0.0253505	0.7190395	0.3621754
ENSG00000171953	ATPAF2	17	17911623	T	C	0.0427435	0.0234058	0.0382933	0.59	-0.791058	0.0217364	5.43E-290	-0.029588	0.0484145	0.5411081	0.03242629
ENSG00000171954	CYP4F22	19	15641215	G	A	0.0248509	-0.0151011	0.0478325	0.7099994	1.12628	0.0299616	3.049837e-309	-0.013408	0.0424711	0.7522327	0.8279329
ENSG00000171960	PPIH	1	43133262	C	T	0.464215	0.00678379	0.014237	0.7099994	0.117225	0.00794282	2.71E-49	0.0578699	0.121514	0.633902	0.2161815
ENSG00000171962	DRC3	17	17898165	T	C	0.0337972	-0.0370293	0.038864	0.4899999	0.416444	0.0237477	7.58E-69	-0.0889178	0.093461	0.3414072	0.601574
ENSG00000171984	SHLD1	20	5787798	G	T	0.486083	-0.019926	0.0142791	0.16	-0.286481	0.00781623	4.24E-294	0.0695543	0.0498792	0.16318	0.3477981
ENSG00000171988	JMJD1C	10	65076351	G	A	0.485089	-0.0215031	0.0142045	0.1900002	0.0522472	0.00805166	8.64E-11	-0.411564	0.279171	0.1404177	0.4739112
ENSG00000172005	MAL	2	95705579	G	A	0.235586	-0.00767124	0.0162511	0.6700003	0.271032	0.00930881	2.27E-186	-0.0283038	0.0599679	0.6369394	0.1376873
ENSG00000172006	ZNF554	19	2827821	A	G	0.294235	-0.0181613	0.0156649	0.2200002	0.210722	0.0089746	6.56E-122	-0.0861862	0.0744298	0.2468837	0.4106464
ENSG00000172007	RAB33B	4	140386074	C	T	0.255467	0.00197088	0.0160907	0.95	-0.289656	0.00896225	3.71E-229	-0.0068042	0.0555514	0.9025151	0.9041235
ENSG00000172009	THOP1	19	2799528	C	T	0.236581	-0.0220544	0.01739	0.1299999	0.220017	0.00934255	1.26E-122	-0.10024	0.0791539	0.2053742	0.8041741
ENSG00000172031	EPHX4	1	92512316	C	T	0.0377734	-0.000257331	0.0337429	0.8600001	0.599917	0.0186053	4.20E-228	-0.000428944	0.0562459	0.9939152	0.9947182
ENSG00000172037	LAMB2	3	49164549	T	G	0.0248509	0.00579566	0.0446811	0.9599999	0.577644	0.031313	5.47E-76	0.0100333	0.0773525	0.8967971	0.8713789
ENSG00000172057	ORMDL3	17	38080574	C	G	0.485089	0.00381947	0.0141934	0.83	0.732676	0.00680812	0	0.00521304	0.0193721	0.787852	0.7090942
ENSG00000172058	SERF1A	5	70205424	C	T	0.267396	0.00931847	0.0161598	0.6800001	0.281913	0.019467	1.58E-47	0.0330544	0.0573673	0.5644878	0.07724734
ENSG00000172059	KLF11	2	10188969	A	G	0.185885	0.000344635	0.0186932	0.89	-0.358321	0.010716	3.83E-245	-0.000961805	0.0521689	0.9852907	0.3786236
ENSG00000172070	NA	20	641851	T	C	0.459245	0.000339552	0.0142177	0.95	-0.692866	0.00881265	0	-0.000490069	0.0205201	0.9809465	0.04385541
ENSG00000172071	EIF2AK3	2	88891676	C	G	0.298211	0.0103475	0.0157881	0.7099994	0.090337	0.00881199	1.16E-24	0.114543	0.175126	0.513071	0.3991651
ENSG00000172081	MOB3A	19	2083854	A	G	0.147117	0.0139595	0.0213258	0.4199997	-0.11182	0.0191901	5.64E-09	-0.124839	0.191914	0.515375	0.1359041
ENSG00000172086	KRCC1	2	88340986	G	A	0.220676	-0.0100264	0.0169055	0.7099994	0.154183	0.00969434	5.91E-57	-0.0650294	0.109722	0.5533999	0.6901273
ENSG00000172113	NME6	3	48338964	G	T	0.284294	0.00956066	0.0152742	0.33	-0.253414	0.00890603	4.32E-178	-0.0377274	0.0602883	0.5314567	0.2832761
ENSG00000172115	CYCS	7	25162345	T	C	0.0506958	-0.0356178	0.0307807	0.2700001	0.257016	0.0258635	2.86E-23	-0.138582	0.120571	0.2503989	0.3607071
ENSG00000172116	CD8B	2	87065754	G	T	0.199801	0.0118494	0.0174469	0.4100001	-0.0666025	0.0102617	8.56E-11	-0.177912	0.263386	0.4993701	0.6022926
ENSG00000172123	SLFN12	17	33749190	A	G	0.0884692	0.0220611	0.0250572	0.4199997	0.714025	0.0138365	0	0.0308968	0.035098	0.378696	0.7801983
ENSG00000172159	FRMD3	9	86005683	G	A	0.0447316	-0.0700459	0.0331777	0.01899984	-0.525255	0.0189808	1.48E-168	0.133356	0.0633485	0.03528114	0.1330732
ENSG00000172164	SNTB1	8	121686749	T	C	0.082505	-0.0501752	0.0285085	0.1299999	0.225822	0.0152333	1.02E-49	-0.222189	0.12713	0.08051014	0.9768071
ENSG00000172171	TEFM	17	29229096	C	T	0.107356	-0.00213668	0.0229476	0.99	-0.725339	0.0160175	0	0.00294577	0.0316371	0.9258153	0.1040515
ENSG00000172172	MRPL13	8	121425321	G	A	0.313121	-0.00211658	0.0152139	0.9	0.0932271	0.00844418	2.44E-28	-0.0227035	0.163205	0.8893629	0.4478299
ENSG00000172175	MALT1	18	56377994	G	C	0.243539	0.0307581	0.0167537	0.06299992	-0.218368	0.0092481	2.89E-123	-0.140854	0.0769539	0.06719394	0.3761935
ENSG00000172183	ISG20	15	89189549	G	A	0.418489	0.00808907	0.0144426	0.7600007	0.417974	0.00779451	0	0.019353	0.0345557	0.5754429	0.1562577
ENSG00000172197	MBOAT1	6	20156802	A	G	0.241551	0.0205959	0.0165705	0.14	-0.235731	0.00932592	5.72E-141	-0.0873702	0.070379	0.2144485	0.5332368
ENSG00000172215	CXCR6	3	45986135	C	T	0.0944334	-0.00954197	0.0266944	0.7899998	-0.318932	0.0133651	7.42E-126	0.0299185	0.0837088	0.7207834	0.8246233
ENSG00000172216	CEBPB	20	48808294	T	C	0.246521	-0.0274539	0.0168348	0.07799917	-0.146691	0.00918009	1.78E-57	0.187155	0.11536	0.1047272	0.761451
ENSG00000172236	TPSAB1	16	1291626	G	A	0.370775	0.0109987	0.0152282	0.4700002	-0.356549	0.0101107	2.04E-272	-0.0308476	0.0427189	0.4702285	0.09358371
ENSG00000172239	PAIP1	5	43542114	A	T	0.501988	0.00388305	0.0141943	0.5099998	-0.145871	0.00936381	1.02E-54	-0.0266197	0.0973221	0.7844521	0.9259713
ENSG00000172243	CLEC7A	12	10276116	C	T	0.146123	-0.00344713	0.0196182	0.7899998	0.508788	0.0108473	0	-0.00677519	0.038559	0.8605221	0.08117907
ENSG00000172244	C5orf34	5	43501025	T	C	0.502982	0.00378188	0.0141947	0.5199996	-0.169603	0.00804093	9.34E-99	-0.0222985	0.0837006	0.7899252	0.7957365
ENSG00000172247	C1QTNF4	11	47613713	T	C	0.356859	-0.0099434	0.0148483	0.64	-0.458121	0.00884103	0	0.0217047	0.032414	0.5031065	0.776227
ENSG00000172250	SERHL	22	42902575	C	G	0.144135	-0.0170506	0.0207706	0.29	-0.117	0.0118618	5.99E-23	0.145731	0.17814	0.4133158	0.9073559
ENSG00000172260	NEGR1	1	72308521	A	G	0.33499	-0.0141131	0.0146912	0.6200004	-0.404076	0.011814	2.17E-256	0.0349268	0.0363718	0.3369187	0.4361583

ENSG00000172262	ZNF131	5	43128700	T	C	0.336978	0.00462426	0.0153104	0.8800001	-0.0827566	0.008443	1.11E-22	-0.0558778	0.185093	0.7627354	0.05401001
ENSG00000172264	MACROD2	20	15004928	A	G	0.102386	-0.0300798	0.0244719	0.16	-0.135243	0.0130864	4.91E-25	0.222413	0.182223	0.2222549	0.3878253
ENSG00000172269	DPAGT1	11	118973127	G	A	0.463221	-0.0249258	0.0143558	0.05499966	0.177279	0.00795509	5.17E-110	-0.140602	0.0812239	0.08344434	0.3029108
ENSG00000172270	BSG	19	577395	G	T	0.109344	-0.0199296	0.0252963	0.3700002	-0.528608	0.0170594	8.25E-211	0.0377021	0.0478701	0.4309354	0.6949436
ENSG00000172273	HINFP	11	118999524	G	A	0.468191	-0.0216795	0.01433	0.0990011	-0.345258	0.00778871	0	0.0627922	0.0415294	0.1305352	0.1442409
ENSG00000172292	CERS6	2	169472008	G	A	0.280318	0.00218102	0.0164424	0.7400005	0.174123	0.0102384	7.31E-65	0.0125257	0.0944327	0.8944765	0.4685038
ENSG00000172296	SPTLC3	20	13068519	C	T	0.498012	0.0385767	0.0142592	0.009200046	0.0805318	0.00796068	4.68E-24	0.479024	0.183285	0.008960848	0.7373728
ENSG00000172301	COPRS	17	30182619	G	A	0.162028	0.0106763	0.0199637	0.59	-0.227553	0.0123452	7.19E-76	-0.0469178	0.0877689	0.5929537	0.04265639
ENSG00000172315	TP53RK	20	45315711	T	C	0.0228628	-0.0197557	0.0436757	0.4899999	-0.435821	0.0227346	6.59E-82	0.0453298	0.100243	0.6511241	0.4499686
ENSG00000172322	CLEC12A	12	10126104	T	A	0.224652	0.0256367	0.0160046	0.06699926	1.22241	0.00608912	0	0.0209722	0.0130931	0.1092038	0.8733521
ENSG00000172331	BPGM	7	134348062	A	C	0.0168986	-0.02295	0.0515652	0.6499995	-0.212241	0.029665	8.39E-13	0.108132	0.243425	0.6568915	0.1276768
ENSG00000172336	POP7	7	100304397	T	C	0.0636183	0.0215602	0.0359332	0.6499995	0.189956	0.0199996	2.14E-21	0.113501	0.189543	0.5492968	0.2344713
ENSG00000172339	ALG14	1	95493399	C	A	0.0407555	0.0378478	0.0367378	0.2399999	-0.138811	0.0216311	1.39E-10	-0.272657	0.268049	0.3090633	0.9928087
ENSG00000172340	SUCLG2	3	67557961	C	T	0.33002	-0.00174432	0.015103	0.9599999	0.375651	0.00820608	0	-0.00464346	0.040205	0.9080531	0.09267806
ENSG00000172345	STARD5	15	81608959	G	T	0.0854871	-0.0241405	0.0237518	0.3400001	0.307704	0.0145411	2.19E-99	-0.0784537	0.0772794	0.310013	0.4146154
ENSG00000172346	CSDC2	22	41965256	G	T	0.196819	0.0360204	0.0183756	0.06299992	0.12564	0.0109731	2.36E-30	0.286695	0.148384	0.05334482	0.008986524
ENSG00000172348	RCAN2	6	46324092	A	C	0.0735586	-0.0154179	0.0245663	0.4199997	-0.167659	0.0161699	3.45E-25	0.0919598	0.146793	0.5310152	0.8733481
ENSG00000172349	IL16	15	81528510	C	T	0.410537	0.00672683	0.0146369	0.7800007	0.355299	0.00796334	0	0.0189328	0.0411981	0.645835	0.4166731
ENSG00000172354	GNB2	7	100273975	G	C	0.409543	0.00729447	0.0144804	0.4500005	0.0464782	0.00811817	1.03E-08	0.156944	0.312756	0.6158018	0.2327458
ENSG00000172366	MCRIP2	16	695143	T	G	0.371769	0.0102849	0.0148808	0.4899999	-0.0627211	0.00935383	2.01E-11	-0.163978	0.23851	0.4917615	0.8951765
ENSG00000172367	NHERF4	11	119058549	T	C	0.464215	-0.0236763	0.0143297	0.07699987	0.195899	0.00794179	2.43E-134	-0.12086	0.0733125	0.0992379	0.1423599
ENSG00000172375	C2CD2L	11	118981080	A	G	0.529821	-0.0291362	0.0142087	0.0259998	0.361169	0.00768156	0	-0.080672	0.0393783	0.04049721	0.382108
ENSG00000172379	ARNT2	15	80793485	A	G	0.284294	0.00615928	0.0160998	0.6499995	-0.0579433	0.00922605	3.38E-10	-0.106298	0.278369	0.7025645	0.6122016
ENSG00000172403	SYNPO2	4	119896199	G	A	0.497018	-0.000627008	0.014216	0.9599999	0.159351	0.00885561	2.16E-72	-0.00393475	0.0892119	0.9648202	0.2388193
ENSG00000172409	CLP1	11	57422902	G	C	0.314115	0.0162799	0.0151102	0.14	0.107987	0.00847157	3.24E-37	0.150758	0.140425	0.2830095	0.3761457
ENSG00000172456	FGGY	1	59997828	T	C	0.152087	-0.00666405	0.0211307	0.6200004	0.255851	0.0113783	5.72E-112	-0.0260466	0.0825981	0.7525021	0.1100965
ENSG00000172458	IL17D	13	21286751	A	G	0.129225	-0.018308	0.0216446	0.3400001	0.113483	0.0132154	8.91E-18	-0.161328	0.191653	0.3999154	0.3533055
ENSG00000172460	PRSS30P	16	2891157	T	C	0.459245	-0.00500885	0.0142887	0.6999999	-0.475131	0.00857934	0	0.010542	0.0300738	0.7259341	0.541973
ENSG00000172466	ZNF24	18	32918811	T	G	0.500994	-0.022307	0.0142605	0.1100001	-0.194966	0.00871179	6.22E-111	0.114415	0.0733218	0.118655	0.8948399
ENSG00000172469	MANEA	6	96041376	C	T	0.427435	-0.023505	0.0143923	0.06299992	0.610484	0.0072445	0	-0.0385022	0.0235796	0.1024988	0.9374905
ENSG00000172493	AFF1	4	87959180	A	G	0.236581	-0.00793395	0.0169148	0.6600001	-0.0820375	0.00939999	2.61E-18	0.0967113	0.206481	0.6395145	0.8022906
ENSG00000172500	FIBP	11	65653611	A	G	0.189861	0.031563	0.0177301	0.0530005	0.325287	0.0102248	4.20E-222	0.0970312	0.0545912	0.0755004	0.1407699
ENSG00000172508	CARNS1	11	67187758	T	C	0.37674	-0.00936023	0.0145196	0.6499995	0.114697	0.00905209	8.58E-37	-0.0816081	0.126754	0.5196864	0.9604829
ENSG00000172530	BANP	16	88046887	C	A	0.0149105	-0.0652239	0.0565242	0.1800002	0.435353	0.0355141	1.51E-34	-0.149819	0.130409	0.250625	0.9453829
ENSG00000172531	PPP1CA	11	67177154	G	C	0.0188867	-0.0361325	0.0830722	0.5500004	-0.188771	0.033113	1.19E-08	0.19141	0.441348	0.6645118	0.3038442
ENSG00000172538	FAM170B	10	50340626	A	G	0.254473	0.0138858	0.0157206	0.4100001	0.128446	0.0110766	4.31E-31	0.108106	0.122745	0.3784609	0.5858715
ENSG00000172543	CTSW	11	65649246	C	T	0.188867	0.0342279	0.0177547	0.03799969	-0.991472	0.00856759	0	-0.0345223	0.0179099	0.05391057	0.1501888
ENSG00000172548	NIPAL4	5	156894376	T	C	0.347913	-0.0237189	0.0150977	0.05499966	-0.145129	0.00912598	6.06E-57	0.163434	0.104536	0.1179546	0.6139593
ENSG00000172554	SNTG2	2	1158969	T	C	0.429423	-0.00273777	0.0144716	0.8200001	-0.106876	0.00892105	4.51E-33	0.0256163	0.135422	0.8499685	0.9244364
ENSG00000172575	RASGRP1	15	38819040	A	G	0.258449	0.00271403	0.0163906	0.7499995	-0.119268	0.0099488	4.10E-33	-0.0227557	0.13744	0.8684963	0.9802095
ENSG00000172578	KLHL6	3	183239398	G	A	0.471173	0.0155785	0.0143131	0.2300001	-0.152347	0.0079451	5.99E-82	-0.102256	0.0941017	0.2771874	0.9756135
ENSG00000172590	MRPL52	14	23301667	G	T	0.487078	-0.0182706	0.0142291	0.17	0.459568	0.00831296	0	-0.039756	0.0309702	0.1992524	0.346333
ENSG00000172594	SMPDL3A	6	123120590	G	A	0.230616	0.0166739	0.0176673	0.4	-0.415706	0.00913786	0	-0.0401098	0.0425086	0.34539	0.1243963
ENSG00000172602	RND1	12	49255304	C	T	0.0119284	0.0356645	0.0715161	0.6600001	0.470181	0.0436744	5.00E-27	0.0758528	0.152267	0.6183729	0.9734912
ENSG00000172613	RAD9A	11	67162528	G	C	0.0357853	0.0256703	0.040335	0.6899999	0.323819	0.0200995	2.14E-58	0.0792737	0.124658	0.5248211	0.3856751
ENSG00000172638	EFEMP2	11	65637487	T	G	0.143141	-0.0295797	0.0209494	0.1199999	0.505819	0.0111857	0	-0.0584788	0.041437	0.1581646	0.0142838
ENSG00000172640	OR10AD1	12	48596625	C	G	0.542744	-0.0215705	0.0142722	0.09499921	0.237338	0.0145225	4.90E-60	-0.090885	0.060391	0.1323387	0.3441741
ENSG00000172653	NA	17	34188925	A	G	0.0467197	0.0144504	0.0317071	0.56	0.311297	0.0178365	3.28E-68	0.04642	0.10189	0.648684	0.7440469
ENSG00000172660	NA	17	34164039	G	C	0.400596	-0.00701657	0.0148206	0.33	-0.0822611	0.0122778	2.08E-11	0.0852964	0.180615	0.636744	0.8590737
ENSG00000172661	WASHC2C	10	46255528	C	G	0.33499	0.0159422	0.015478	0.28	-0.401418	0.0198826	1.21E-90	-0.0397147	0.0386084	0.3036427	0.7440714

ENSG00000172663	TMEM134	11	67234283	T	C	0.0198807	0.0684886	0.0668069	0.33	-0.333774	0.0280407	1.14E-32	-0.205195	0.200897	0.307069	0.1965435
ENSG00000172667	ZMAT3	3	178762539	G	T	0.38171	-0.0168464	0.0142851	0.3900004	-0.347817	0.00789624	0	0.0484347	0.0410855	0.2384474	0.5934595
ENSG00000172671	ZFAND4	10	46139635	C	G	0.302187	0.00642698	0.015474	0.7700005	-0.186479	0.00954003	4.37E-85	-0.0344649	0.0829986	0.6779612	0.3482418
ENSG00000172673	THEMIS	6	128134496	T	C	0.137177	0.00158602	0.0199793	0.9299999	-0.161424	0.0122343	9.45E-40	-0.00982517	0.123771	0.936729	0.74733
ENSG00000172687	ZNF738	19	21551918	C	T	0.333996	0.00558642	0.0152434	0.8700001	-0.427181	0.0179301	1.85E-125	-0.0130774	0.0356879	0.7140384	0.153693
ENSG00000172716	SLFN11	17	33689022	T	C	0.0636183	0.0384419	0.029203	0.1299999	0.397348	0.0168718	1.23E-122	0.0967462	0.0736095	0.1887396	0.9934901
ENSG00000172725	CORO1B	11	67208405	A	G	0.398608	-0.0132187	0.014362	0.4600002	-0.207646	0.00869234	4.04E-126	0.0636598	0.0692171	0.3577234	0.9187182
ENSG00000172728	FUT10	8	33279641	G	A	0.374751	-0.0140632	0.014603	0.2300001	0.703134	0.00706259	0	-0.0200007	0.0207694	0.3355526	0.655925
ENSG00000172731	LRRC20	10	72100555	T	G	0.407555	0.00153279	0.0145678	0.99	0.128416	0.0080473	2.52E-57	0.0119362	0.113445	0.9162049	0.6471048
ENSG00000172732	MUS81	11	65629860	G	A	0.332008	0.00293701	0.0150526	0.84	-0.18055	0.00843937	1.53E-101	-0.016267	0.0833742	0.8453081	0.01985537
ENSG00000172738	TMEM217	6	37202943	A	G	0.114314	-0.0184833	0.0214024	0.4600002	-0.137752	0.0159045	4.67E-18	0.134178	0.15614	0.3901488	0.3585497
ENSG00000172748	ZNF596	8	189739	T	C	0.280318	-0.00451774	0.0152514	0.9299999	-0.10036	0.00858581	1.45E-31	0.0450156	0.152016	0.7671362	0.8823998
ENSG00000172757	CFL1	11	65609995	G	A	0.332008	0.00293701	0.0150526	0.84	-0.305318	0.0123308	2.38E-135	-0.00961952	0.049303	0.8453065	0.00992351
ENSG00000172765	TMCC1	3	129489527	C	T	0.082505	0.0274133	0.0262336	0.35	-0.198427	0.0148858	1.55E-40	-0.138153	0.132613	0.297517	0.738379
ENSG00000172766	NAA16	13	41918253	G	A	0.279324	-0.0099599	0.0161293	0.33	-0.199491	0.00916671	5.25E-105	0.0499265	0.0808847	0.5370665	0.4008498
ENSG00000172771	EFCAB12	3	129133829	A	G	0.416501	-0.0100567	0.0143259	0.58	-0.164445	0.00888909	2.08E-76	0.0611555	0.0871796	0.4829976	0.7383042
ENSG00000172775	PSME3IP1	16	57203203	C	T	0.0506958	0.0492112	0.0307811	0.1199999	-0.319513	0.0175908	1.00E-73	-0.154019	0.09671	0.1112522	0.1976138
ENSG00000172780	RAB43	3	128824028	G	A	0.0159046	0.0316713	0.0563527	0.5400003	0.187687	0.0292357	1.36E-10	0.168745	0.301396	0.5755628	0.7232677
ENSG00000172785	ZNG1A	9	155010	A	T	0.413519	0.00671481	0.0145277	0.7099994	0.584722	0.0166018	9.74E-272	0.0114838	0.0248476	0.6439612	0.5568179
ENSG00000172794	RAB37	17	72705095	A	G	0.124254	-0.0427384	0.0217357	0.05099998	0.1492	0.0130178	2.07E-30	-0.286451	0.14781	0.05262764	0.914009
ENSG00000172795	DCP2	5	112334533	A	G	0.494036	0.0220911	0.0142081	0.09099971	0.245672	0.00783011	4.40E-216	0.0899211	0.0579046	0.1204426	0.7106645
ENSG00000172803	SNX32	11	65612739	G	A	0.190855	0.0216382	0.017967	0.2300001	0.369187	0.010198	5.68E-287	0.0586104	0.0486933	0.2287194	0.04547425
ENSG00000172819	RARG	12	53615559	G	C	0.140159	-0.0173762	0.0218452	0.28	0.118889	0.0129616	4.63E-20	-0.146155	0.184434	0.4280983	0.5478173
ENSG00000172824	CES4A	16	67033076	C	T	0.354871	-0.0350412	0.015227	0.00519996	-0.0964276	0.00978107	6.29E-23	0.363394	0.162156	0.02502541	0.9619147
ENSG00000172830	SSH3	11	67075498	C	T	0.054672	-0.00998134	0.0313682	0.4600002	-0.547173	0.0167297	1.25E-234	0.0182416	0.0573304	0.7503452	0.7927799
ENSG00000172831	CES2	16	66973673	A	G	0.17992	0.0334538	0.0198239	0.1299999	-0.0928005	0.010756	6.25E-18	-0.360492	0.217666	0.09768818	0.9874978
ENSG00000172845	SP3	2	174800808	G	A	0.254473	-0.00856386	0.0161641	0.6300007	-0.0647368	0.0092265	2.28E-12	0.132287	0.2504	0.5972888	0.3924184
ENSG00000172867	KRT2	12	53042145	C	A	0.321074	0.0336031	0.0151922	0.02	-0.133009	0.00835682	4.89E-57	-0.252638	0.115317	0.02846544	0.4280543
ENSG00000172869	DMXL1	5	118479150	A	T	0.389662	-0.000395753	0.0145727	0.84	-0.0694527	0.00826071	4.18E-17	0.00569816	0.209823	0.9783345	0.2434417
ENSG00000172878	METAP1D	2	172905824	T	C	0.365805	0.000975709	0.0146823	0.8600001	0.117023	0.00908195	5.45E-38	0.00833777	0.125467	0.9470165	0.9799617
ENSG00000172890	NADSYN1	11	71201691	T	A	0.299205	0.00124594	0.0172111	0.5	-0.142018	0.00854174	4.49E-62	-0.00877309	0.12119	0.9422908	0.8656321
ENSG00000172893	DHCR7	11	71151576	T	A	0.258449	-0.0019241	0.0183192	0.4799997	-0.0517344	0.00913616	1.49E-08	0.0371919	0.354162	0.9163648	0.5682059
ENSG00000172915	NBEA	13	35881791	G	A	0.00795229	0.19304	0.0907058	0.05600025	0.862596	0.0420426	1.51E-93	0.22379	0.105719	0.0342733	0.3075809
ENSG00000172922	RNASEH2C	11	65485392	A	G	0.355865	0.00600178	0.0146932	0.5	-0.313866	0.00909681	7.26E-261	-0.0191221	0.0468168	0.6829472	0.4125704
ENSG00000172932	ANKRD13D	11	67062987	A	T	0.0347913	-0.0401725	0.0311709	0.3400001	0.42414	0.0216365	1.46E-85	-0.0947152	0.0736507	0.198441	0.8442629
ENSG00000172936	MYD88	3	38182241	T	A	0.0805169	0.0030321	0.0273962	0.9299999	0.157333	0.0157113	1.32E-23	0.0192718	0.174139	0.9118787	0.9208609
ENSG00000172939	OXSR1	3	38251779	T	C	0.0626243	0.0519273	0.0292848	0.08500021	0.193254	0.0171094	1.39E-29	0.2687	0.153392	0.07982044	0.866705
ENSG00000172954	LCLAT1	2	30768591	T	C	0.224652	-0.0109538	0.0165658	0.7199992	1.03732	0.00749033	0	-0.0105597	0.0159699	0.5084698	0.9496159
ENSG00000172965	MIR4435-2HG	2	112109015	A	G	0.0675944	0.00638731	0.0294076	0.8200001	0.703197	0.0470384	1.57E-50	0.00908325	0.0418243	0.8280709	0.007957727
ENSG00000172967	XKR3	22	17283445	A	G	0.39165	0.00597456	0.0152841	0.7099994	0.837673	0.0122881	0	0.00713233	0.0182462	0.6958756	0.3103008
ENSG00000172977	KAT5	11	65483271	T	G	0.468191	-0.0271384	0.0142406	0.02100003	-0.0560047	0.00806264	3.75E-12	0.484573	0.263671	0.06609245	0.3682407
ENSG00000172985	SH3RF3	2	110004005	C	T	0.252485	-0.0198647	0.0161449	0.16	-0.230817	0.00942194	1.56E-132	0.0860626	0.070035	0.2191273	0.3552088
ENSG00000172992	DCAKD	17	43119590	T	C	0.414513	0.000172771	0.0147074	0.8700001	-0.497183	0.00836046	0	-0.0003475	0.0295815	0.9906273	0.9112773
ENSG00000172995	ARPP21	3	35758212	A	G	0.236581	-0.0375201	0.0161268	0.01700004	-0.062718	0.00930837	1.61E-11	0.598235	0.27203	0.02786699	0.9416595
ENSG00000173011	TADA2B	4	7050997	C	G	0.203777	-0.00372923	0.0182536	0.8800001	-0.365429	0.0185035	8.15E-87	0.0102051	0.0499538	0.8381268	0.4440209
ENSG00000173039	RELA	11	65425816	A	G	0.11332	0.0304409	0.0213791	0.1299999	0.0804386	0.0136138	3.45E-09	0.378437	0.27339	0.1662859	0.6906783
ENSG00000173040	EVC2	4	5627887	T	C	0.0805169	0.00522215	0.0267861	0.9400001	0.204466	0.014834	3.20E-43	0.0255405	0.131018	0.8454415	0.8278627
ENSG00000173041	ZNF680	7	64001873	C	G	0.0765408	0.00237486	0.0264392	0.81	-0.218594	0.0141315	5.65E-54	-0.0108642	0.120953	0.9284287	0.125304
ENSG00000173064	HECTD4	12	112708944	T	C	0.00198807	0.094501	0.15007	0.5099998	0.664401	0.0425408	5.49E-55	0.142235	0.226056	0.5292171	0.9981444
ENSG00000173065	FAM222B	17	27132623	A	T	0.187873	-0.0134377	0.0178792	0.4600002	-0.141404	0.0109463	3.56E-38	0.0950305	0.126654	0.4530656	0.1186057

ENSG00000173068	BNC2	9	16643622	G	A	0.435388	0.0182064	0.014614	0.4100001	-0.248642	0.00857827	1.01E-184	-0.0732234	0.0588295	0.2132531	0.2238986
ENSG00000173083	HPSE	4	84234960	T	C	0.0506958	-0.0315014	0.0334304	0.28	-0.919043	0.0212409	0	0.0342763	0.0363838	0.3461544	0.1202241
ENSG00000173085	COQ2	4	84194378	G	T	0.277336	-0.00725658	0.0154336	0.56	0.246839	0.00858854	1.19E-181	-0.029398	0.0625333	0.6382709	0.04482617
ENSG00000173110	HSPA6	1	161495358	G	A	0.364811	0.0154743	0.0149948	0.3100002	-0.187963	0.0130825	8.26E-47	-0.0823261	0.0799806	0.3033265	0.4551447
ENSG00000173113	TRMT112	11	64084744	C	G	0.0626243	-0.0441019	0.0303291	0.1199999	-0.899889	0.0155586	0	0.0490081	0.0337138	0.1460427	0.3055026
ENSG00000173114	LRRN3	7	110748286	A	G	0.11332	0.0212731	0.0211973	0.4500005	-0.112016	0.0115291	2.58E-22	-0.189911	0.190241	0.318151	0.2104916
ENSG00000173137	ADCK5	8	145607623	T	C	0.366799	0.00622463	0.0148033	0.8499999	-0.193771	0.0179285	3.15E-27	-0.0321236	0.0764535	0.6743602	0.9699167
ENSG00000173141	MRPL57	13	21752003	T	C	0.00894632	-0.111562	0.0798182	0.14	-0.486547	0.0706964	5.89E-12	0.229293	0.167399	0.1707684	0.9440508
ENSG00000173145	NOC3L	10	96098860	C	T	0.180915	0.00651013	0.0194357	0.7400005	0.435631	0.0103772	0	0.0149441	0.0446164	0.7376653	0.210803
ENSG00000173153	ESRRA	11	64078629	A	T	0.00596421	-0.052626	0.169256	0.5300002	0.41756	0.0423111	5.68E-23	-0.126032	0.405547	0.7559745	0.438883
ENSG00000173156	RHOD	11	66831886	A	G	0.0745527	-0.0026572	0.0253424	0.7400005	-0.150478	0.0145442	4.35E-25	0.0176584	0.168422	0.9164976	0.9556335
ENSG00000173163	COMMD1	2	62245120	G	A	0.295229	-0.0130504	0.015649	0.4100001	0.101489	0.00966741	8.82E-26	-0.12859	0.154681	0.4057896	0.9403053
ENSG00000173166	RAPH1	2	204329600	A	G	0.173956	0.0131202	0.0190717	0.5400003	-0.0816439	0.010313	2.44E-15	-0.1607	0.234476	0.4931181	0.1199776
ENSG00000173171	MTX1	1	155181052	A	G	0.455268	0.0126096	0.014201	0.2599998	-0.2981	0.0172663	8.66E-67	-0.0422999	0.0477014	0.3752052	0.08940252
ENSG00000173193	PARP14	3	122424576	C	T	0.10338	-0.0351582	0.0261258	0.1499999	-0.668859	0.0130237	0	0.0525644	0.0390737	0.1785395	0.1324134
ENSG00000173200	PARP15	3	122327171	G	A	0.430417	-0.00310556	0.0142237	0.99	0.344001	0.00773378	0	-0.00902776	0.0413483	0.8271686	0.6940831
ENSG00000173208	ABCD2	12	39978694	A	G	0.164016	0.0117535	0.0198475	0.6100002	-0.217787	0.0111642	9.46E-85	-0.0539678	0.0911746	0.5539056	0.8325939
ENSG00000173209	AHSA2P	2	61409619	A	G	0.410537	0.00700236	0.0144097	0.64	-0.531626	0.0111907	0	-0.0131716	0.0271064	0.6270223	0.2386515
ENSG00000173210	ABLIM3	5	148580575	A	G	0.028827	0.0188476	0.0363152	0.4500005	-0.380045	0.0232335	3.84E-60	-0.0495931	0.0956032	0.6039426	0.5719277
ENSG00000173212	MAB21L3	1	116666118	G	A	0.026839	-0.0454401	0.0443397	0.3800004	0.36903	0.0296391	1.38E-35	-0.123134	0.120558	0.3070822	0.6865355
ENSG00000173214	MFSD4B	6	111586460	C	A	0.312127	0.0118249	0.0159	0.56	0.189116	0.0085972	3.05E-107	0.0625273	0.0841235	0.4573123	0.1031504
ENSG00000173218	VANGL1	1	116212709	T	C	0.0944334	-0.018617	0.0234386	0.33	-0.213504	0.0130019	1.35E-60	0.0871972	0.109909	0.4275678	0.408411
ENSG00000173221	GLRX	5	95122866	G	A	0.256461	-0.0174414	0.0156284	0.3599996	0.46096	0.0153973	6.36E-197	-0.0378371	0.0339276	0.264751	0.9179893
ENSG00000173226	IQCB1	3	121521268	C	T	0.383698	0.000225765	0.0147215	1	-0.540573	0.00766877	0	-0.00041764	0.0272331	0.9877643	0.9643212
ENSG00000173230	GOLGB1	3	121425324	C	T	0.126243	-0.00388949	0.0208535	0.81	-0.804254	0.0116501	0	0.00483615	0.0259291	0.8520415	0.6348552
ENSG00000173239	LIPM	10	90571478	G	A	0.129225	0.000572528	0.0217818	0.7700005	-0.102828	0.0136164	4.29E-14	-0.00556781	0.211828	0.9790304	0.1265542
ENSG00000173258	ZNF483	9	114313781	G	A	0.146123	0.0187647	0.0195588	0.4299995	0.0982424	0.0103907	3.24E-21	0.191004	0.200109	0.3398317	0.4559146
ENSG00000173261	PLAC8L1	5	145473940	A	G	0.324056	0.00751975	0.0147611	0.4500005	-0.0556688	0.00943091	3.57E-09	-0.13508	0.266145	0.6117735	0.5807335
ENSG00000173262	SLC2A14	12	8004426	A	G	0.112326	-0.00790258	0.0221304	0.91	0.659325	0.020108	8.42E-236	-0.0119859	0.0335672	0.7210391	0.4739496
ENSG00000173264	GPR137	11	64047253	T	C	0.323062	0.0219215	0.0155216	0.1100001	0.121795	0.00868224	1.05E-44	0.179988	0.128085	0.1599564	0.2224637
ENSG00000173269	MMRN2	10	88712267	A	G	0.111332	-0.0382467	0.0231957	0.1	0.113755	0.014683	9.38E-15	-0.336219	0.208476	0.106799	0.8496786
ENSG00000173272	MZT2A	2	132236394	T	C	0.219682	0.0180202	0.0183453	0.4100001	0.362706	0.0110976	2.69E-234	0.0496827	0.0506019	0.3261809	0.1468011
ENSG00000173273	TNKS	8	9526640	G	A	0.0347913	-0.00801235	0.0383223	0.9199999	-0.171323	0.021417	1.25E-15	0.0467674	0.22376	0.8344431	0.8690455
ENSG00000173276	ZBTB21	21	43418718	A	G	0.27833	-0.0161601	0.0162249	0.2999998	0.0735754	0.0101263	3.71E-13	-0.21964	0.222583	0.3237516	0.5280549
ENSG00000173281	PPP1R3B	8	9001424	T	C	0.132207	-0.0314165	0.0219242	0.28	-0.155679	0.0120846	5.65E-38	0.201803	0.141698	0.1543954	0.03216627
ENSG00000173295	FAM86B3P	8	8094252	A	C	0.519881	-0.0248178	0.0142582	0.2	0.157239	0.0100031	1.12E-55	-0.157835	0.0912329	0.08362594	0.8564719
ENSG00000173320	STOX2	4	184859631	G	A	0.406561	-0.0316686	0.014443	0.04	-0.0961398	0.00897614	9.08E-27	0.329401	0.153345	0.03170506	0.5058755
ENSG00000173327	MAP3K11	11	65374039	A	G	0.440358	-0.0243709	0.014339	0.04399973	0.447689	0.00758745	0	-0.0544372	0.0320422	0.0893345	0.7853003
ENSG00000173334	TRIB1	8	126446605	C	T	0.345924	0.00264335	0.0149394	0.8700001	0.0640707	0.00843591	3.08E-14	0.0412568	0.233234	0.8595947	0.618031
ENSG00000173338	KCNK7	11	65361896	C	T	0.244533	0.024864	0.016521	0.09800089	-0.202832	0.00925135	1.51E-106	-0.122584	0.0816432	0.1332365	0.8894945
ENSG00000173369	C1QB	1	22983643	A	C	0.385686	-0.0170496	0.0146785	0.1900002	0.424357	0.00790186	0	-0.0401775	0.0345981	0.2455346	0.5821834
ENSG00000173372	C1QA	1	22964550	A	G	0.0924453	-0.0115794	0.0258145	0.6899999	-0.407017	0.0139016	1.95E-188	0.0284495	0.0634311	0.6537857	0.08852451
ENSG00000173391	OLR1	12	10317819	A	T	0.358847	0.00407755	0.0147801	0.6100002	-0.0577276	0.0082402	2.46E-12	-0.0706344	0.25623	0.7828035	0.2796999
ENSG00000173401	GLIPR1L1	12	75746379	A	T	0.37674	0.00601092	0.0145357	0.6499995	0.0880741	0.00864913	2.36E-24	0.0682485	0.165176	0.6794692	0.6658199
ENSG00000173402	DAG1	3	49539597	C	T	0.432406	-0.0180603	0.0142839	0.2999998	-0.0851519	0.00800225	1.92E-26	0.212095	0.168926	0.2092794	0.2781694
ENSG00000173406	DAB1	1	58236428	C	G	0.119284	0.00834982	0.0226081	0.7300002	-0.698371	0.0152567	0	-0.0119561	0.0323737	0.7118917	0.004774577
ENSG00000173409	ARV1	1	231125534	C	T	0.105368	-0.0820849	0.0235334	5.00E-04	-0.497172	0.0132743	5.21E-307	0.165104	0.0475393	0.000514696	0.274986
ENSG00000173418	NAA20	20	20006029	T	C	0.100398	-0.00329455	0.0231547	0.59	-0.130034	0.0118824	7.15E-28	0.0253361	0.178081	0.886865	0.1214771
ENSG00000173436	MICOS10	1	19939895	G	A	0.45825	-0.0232352	0.0143006	0.06800017	0.0739895	0.00886802	7.22E-17	-0.314034	0.19691	0.1107541	0.3081139
ENSG00000173442	EHBP1L1	11	65351815	T	A	0.185885	0.0278839	0.0177538	0.08300036	-0.159955	0.0131354	4.10E-34	-0.174324	0.111912	0.1193079	0.8630323

ENSG00000173451	THAP2	12	72065604	T	C	0.0606362	0.0454425	0.0272989	0.1100001	-0.0846397	0.0144535	4.74E-09	-0.536893	0.335308	0.1093342	0.9044954
ENSG00000173456	RNF26	11	119206630	T	G	0.416501	0.0312889	0.0146391	0.03899959	0.0968522	0.0086768	6.24E-29	0.323058	0.153895	0.03579805	0.351466
ENSG00000173457	PPP1R14B	11	64013184	G	C	0.177932	0.0421895	0.0184201	0.01099993	0.162765	0.0177618	5.01E-20	0.259204	0.116651	0.02627987	0.8441617
ENSG00000173473	SMARCC1	3	47725179	T	C	0.0208748	0.00718382	0.0590464	0.8700001	-0.265478	0.0312122	1.81E-17	-0.0270599	0.222438	0.903175	0.0562822
ENSG00000173480	ZNF417	19	58419821	G	C	0.370775	0.00654961	0.0145739	0.5700002	0.224892	0.0120676	1.64E-77	0.0291234	0.064823	0.6532324	0.2955869
ENSG00000173482	PTPRM	18	7986819	G	A	0.127237	-0.00876476	0.0217719	0.6700003	-0.582561	0.0120552	0	0.0150452	0.037374	0.6872733	0.8972069
ENSG00000173486	FKBP2	11	64010039	T	C	0.173956	0.0442492	0.0187484	0.0129999	0.081207	0.0121183	2.07E-11	0.544894	0.244773	0.02600583	0.2416163
ENSG00000173511	VEGFB	11	64004134	G	C	0.302187	-0.00346818	0.0153681	0.7499995	-0.110929	0.00850421	6.88E-39	0.0312649	0.138561	0.8214813	0.1046903
ENSG00000173517	PEAK1	15	77556478	T	C	0.410537	0.00387616	0.0142812	0.7700005	0.171298	0.00885746	2.50E-83	0.0226282	0.0833787	0.7860909	0.431503
ENSG00000173530	TNFRSF10D	8	23007322	T	C	0.413519	0.0238137	0.0144922	0.09499921	0.104181	0.00818785	4.36E-37	0.228581	0.140262	0.1031704	0.04832945
ENSG00000173531	MST1	3	49724157	T	C	0.0705765	0.00591629	0.0304985	0.84	0.462882	0.0374161	3.74E-35	0.0127814	0.0658963	0.8462053	0.4466988
ENSG00000173535	TNFRSF10C	8	22958409	G	A	0.230616	-0.0200603	0.0173322	0.1800002	-0.317577	0.00985035	4.83E-228	0.0631667	0.0546115	0.2474131	0.2747839
ENSG00000173542	MOB1B	4	71828104	G	A	0.0437376	-0.0358986	0.0361973	0.1900002	0.150064	0.0186926	9.91E-16	-0.239221	0.243045	0.3249848	0.1990743
ENSG00000173545	ZNF622	5	16458764	A	G	0.357853	0.0031063	0.0146958	0.91	-0.102592	0.0081207	1.38E-36	-0.0302782	0.143265	0.8326191	0.8316129
ENSG00000173548	SNX33	15	75947444	G	C	0.282306	0.0187687	0.0156051	0.3100002	0.105758	0.0087259	8.28E-34	0.177468	0.14828	0.231365	0.2976319
ENSG00000173559	NABP1	2	192548022	T	C	0.355865	-0.00822429	0.0148185	0.6499995	-0.0861114	0.00924418	1.22E-20	0.0955076	0.17239	0.5795658	0.8882578
ENSG00000173566	NA	8	21965657	G	C	0.298211	-0.00463263	0.0154924	0.64	0.667931	0.00948449	0	-0.00693579	0.0231948	0.7649223	0.6313594
ENSG00000173567	ADGRF3	2	26550363	T	C	0.208748	0.00966279	0.0176782	0.56	0.115419	0.00961138	3.20E-33	0.0837191	0.153324	0.5850465	0.3541023
ENSG00000173575	CHD2	15	93498587	T	C	0.241551	0.0190425	0.0164891	0.17	0.17961	0.00930199	4.53E-83	0.106021	0.0919689	0.248995	0.4774729
ENSG00000173578	XCR1	3	46063875	T	C	0.214712	0.00289231	0.0174446	0.9599999	0.166608	0.0128762	2.70E-38	0.01736	0.104713	0.8683253	0.9465218
ENSG00000173581	CCDC106	19	56158477	T	G	0.028827	0.0184206	0.0441985	0.6200004	-0.279012	0.0270748	6.68E-25	-0.0660209	0.15854	0.6770958	0.6491736
ENSG00000173585	CCR9	3	45936331	A	G	0.392644	0.0118033	0.0146037	0.25	-0.123488	0.0082234	5.72E-51	-0.0955829	0.118432	0.4196249	0.3627777
ENSG00000173588	CEP83	12	94776994	A	G	0.292247	-0.0216591	0.0153672	0.1900002	-0.150244	0.00938196	1.02E-57	0.144159	0.102677	0.160316	0.0440323
ENSG00000173597	SULT1B1	4	70623122	C	T	0.44831	-0.0222337	0.0142028	0.14	-0.103293	0.00798657	2.92E-38	0.215248	0.138503	0.1201601	0.06830214
ENSG00000173598	NUDT4	12	93784341	G	T	0.197813	0.0236091	0.0189668	0.2	-0.109494	0.0108515	6.10E-24	-0.21562	0.174535	0.2166838	0.4514237
ENSG00000173611	SCAI	9	127810082	G	A	0.437376	0.000400837	0.0142837	0.8700001	-0.0518098	0.00808619	1.48E-10	-0.0077367	0.275698	0.9776125	0.5181446
ENSG00000173614	NMNAT1	1	10024522	A	G	0.027833	0.0186488	0.0486878	0.8600001	0.392658	0.0578033	1.10E-11	0.0474938	0.124192	0.7021493	0.6479935
ENSG00000173621	LRFN4	11	66626032	T	C	0.335984	-0.0112935	0.0150719	0.33	-0.327524	0.00811782	0	0.0344814	0.0460256	0.4537492	0.1778255
ENSG00000173626	TRAPPC3L	6	116841462	T	A	0.304175	-0.0158807	0.015493	0.2099999	0.053544	0.00953045	1.93E-08	-0.296592	0.294127	0.3132727	0.9932513
ENSG00000173638	SLC19A1	21	46938905	A	C	0.468191	0.0199591	0.014501	0.1299999	0.12222	0.010544	4.56E-31	0.163305	0.11948	0.1716903	0.9008362
ENSG00000173653	RCE1	11	66612161	A	G	0.116302	-0.00907902	0.022359	0.84	0.312461	0.0148003	6.20E-99	-0.0290564	0.0715709	0.6847562	0.3229966
ENSG00000173660	UQCRH	1	46775875	G	A	0.117296	-0.0221154	0.0240684	0.35	-0.329576	0.0143448	8.25E-117	0.0671027	0.0730869	0.3585548	0.5014354
ENSG00000173678	SPDYE2B	7	102296309	A	G	0.312127	-0.00200738	0.0153567	0.84	0.607565	0.022137	7.84E-166	-0.00330398	0.0252761	0.8960004	0.5563483
ENSG00000173692	PSMD1	2	231979559	A	G	0.367793	-0.0463524	0.0153729	0.000369999	0.193444	0.00820946	9.11E-123	-0.239616	0.0801173	0.002782331	0.06175256
ENSG00000173706	HEG1	3	124729678	A	G	0.37674	0.0108436	0.0146179	0.3599996	0.215442	0.00819058	1.74E-152	0.050332	0.0678778	0.4583855	0.5426211
ENSG00000173715	TOP6BL	11	66561454	A	G	0.116302	-0.00907902	0.022359	0.84	-0.498884	0.0146197	3.24E-255	0.0181987	0.0448212	0.6847221	0.170279
ENSG00000173726	TOMM20	1	235282451	T	C	0.136183	-0.00755956	0.0194711	0.56	0.151552	0.0110191	4.85E-43	-0.0498809	0.128529	0.697949	0.1710388
ENSG00000173727	NA	11	65228378	T	C	0.313121	0.000783569	0.0150455	0.7800007	0.313867	0.0203261	8.59E-54	0.0024965	0.0479362	0.9584652	0.3282494
ENSG00000173744	AGFG1	2	228379126	G	A	0.357853	0.00245864	0.0151034	0.91	-0.135791	0.00836841	3.27E-59	-0.0181061	0.111231	0.8706924	0.8976128
ENSG00000173757	STAT5B	17	40389955	C	T	0.0815109	0.0271237	0.0250834	0.2099999	0.147622	0.0143415	7.55E-25	0.183737	0.170851	0.2821855	0.4060275
ENSG00000173762	CD7	17	80274111	T	C	0.167992	0.014863	0.0179235	0.3100002	-0.154546	0.0124985	4.03E-35	-0.096172	0.116236	0.4080172	0.8821389
ENSG00000173786	CNP	17	40122976	G	A	0.281312	0.015038	0.0161375	0.2599998	-0.312575	0.0132507	4.95E-123	-0.04811	0.0516678	0.3517809	0.6624389
ENSG00000173801	JUP	17	39859437	A	C	0.0894632	0.0230278	0.0259017	0.3900004	1.47416	0.0121215	0	0.0156209	0.0175709	0.3739912	0.8077697
ENSG00000173805	HAP1	17	39882445	A	G	0.0258449	-0.0237671	0.0513489	0.6200004	0.325559	0.0487233	2.36E-11	-0.073004	0.158103	0.6442616	0.6212952
ENSG00000173809	TDRD12	19	33265571	C	T	0.475149	0.00266118	0.0142037	0.8200001	0.105063	0.00900872	1.98E-31	0.0253293	0.135209	0.8513987	0.8543098
ENSG00000173811	CCDC13-AS1	3	42781163	A	T	0.183897	0.00591569	0.0182467	0.7600007	0.541447	0.027849	3.39E-84	0.0109257	0.0337046	0.7458162	0.04117467
ENSG00000173818	ENDOV	17	78400425	G	A	0.0367793	-0.0138948	0.0350521	0.81	-0.327747	0.0238258	4.69E-43	0.0423949	0.106993	0.6919282	0.4026438
ENSG00000173821	RNF213	17	78303625	G	A	0.314115	-0.00320753	0.0150817	0.6499995	0.0566092	0.00928782	1.09E-09	-0.0566609	0.26658	0.8316798	0.4707865
ENSG00000173825	TIGD3	11	65123661	G	T	0.145129	0.0120899	0.0195424	0.3100002	0.15234	0.0119368	2.67E-37	0.0793614	0.128432	0.5366254	0.2688844
ENSG00000173838	MARCHF10	17	60832190	A	C	0.125249	-0.00826774	0.0216532	0.6999999	0.2085	0.0125314	3.69E-62	-0.0396534	0.10388	0.7026655	0.9456552

ENSG00000173846	PLK3	1	45268779	A	G	0.16998	-0.0143205	0.0191154	0.5099998	0.0666362	0.0107717	6.16E-10	-0.214906	0.288958	0.4570419	0.9161108
ENSG00000173848	NET1	10	5477470	C	T	0.10338	0.0392876	0.0242478	0.1	0.28154	0.0158845	2.73E-70	0.139546	0.0864849	0.1066302	0.9329285
ENSG00000173852	DPY19L1	7	35023185	T	A	0.459245	0.00131314	0.0142671	0.9199999	-0.540101	0.00810394	0	-0.00243129	0.0264156	0.9266665	0.5927716
ENSG00000173868	PHOSPHO1	17	47304426	T	C	0.243539	-0.00572924	0.016587	0.7099994	0.183835	0.00920399	9.38E-89	-0.0311651	0.0902412	0.7298284	0.6416959
ENSG00000173875	ZNF791	19	12732233	A	G	0.326044	-0.0113206	0.0150002	0.4899999	-0.117415	0.00833983	5.13E-45	0.0964154	0.127937	0.4510807	0.4574892
ENSG00000173889	PHC3	3	169852028	T	G	0.316103	0.0168248	0.0158528	0.2999998	0.0855216	0.00921927	1.75E-20	0.196732	0.186575	0.2916836	0.7908723
ENSG00000173890	GPR160	3	169779454	G	T	0.104374	-0.00270923	0.0218296	0.7499995	0.841643	0.0170805	0	-0.00321898	0.025937	0.90123	0.3297859
ENSG00000173905	GOLIM4	3	167770114	T	A	0.0954274	0.0103913	0.0225366	0.8600001	0.478793	0.0123134	0	0.0217031	0.0470729	0.6447605	0.9521334
ENSG00000173915	ATP5MK	10	105152510	A	G	0.337972	-0.0161875	0.0154385	0.2	0.697186	0.00796504	0	-0.0232183	0.0221456	0.294436	0.2046817
ENSG00000173917	HOXB2	17	46620848	T	A	0.361829	0.0104873	0.0146676	0.6999999	-0.798461	0.0069179	0	-0.0131344	0.0183702	0.474619	0.0990948
ENSG00000173926	MARCHF3	5	126284953	T	C	0.468191	-0.0230491	0.0142325	0.1199999	-0.204452	0.00794219	3.90E-146	0.112736	0.0697504	0.106035	0.1236169
ENSG00000173928	SWSAP1	19	11486494	A	G	0.185885	-0.00592226	0.0195122	0.64	0.211511	0.012329	5.71E-66	-0.0279997	0.0922658	0.761533	0.05832662
ENSG00000173930	SLCO4C1	5	101600971	T	C	0.361829	-0.00876336	0.0145894	0.5099998	0.320415	0.0118986	1.01E-159	-0.02735	0.0455441	0.5481618	0.3302135
ENSG00000173950	XXYLT1	3	194890452	G	A	0.2167	-0.0174475	0.0167488	0.4500005	-0.19502	0.0103802	9.54E-79	0.0894653	0.0860145	0.2982847	0.5168318
ENSG00000173960	UBXN2A	2	24188967	C	T	0.429423	0.0134408	0.0142701	0.25	-0.304825	0.00777734	0	-0.0440935	0.0468276	0.3463905	0.312132
ENSG00000173988	LRRC63	13	46818792	A	T	0.311133	0.00818174	0.0154652	0.6200004	0.234607	0.022666	4.16E-25	0.0348743	0.0660057	0.5972549	0.9266585
ENSG00000173992	CCS	11	66366891	A	G	0.487078	0.00521641	0.0142053	0.7800007	-0.545267	0.00733214	0	-0.00956671	0.0260523	0.7134614	0.5336272
ENSG00000174004	NRROS	3	196377716	C	T	0.456262	0.00664195	0.0142613	0.91	-0.305294	0.00850587	4.04E-282	-0.0217559	0.0467172	0.6414351	0.5171916
ENSG00000174007	CEP19	3	196436156	C	G	0.319085	0.0110487	0.0148022	0.3599996	-0.479689	0.00897918	0	-0.023033	0.0308609	0.455456	0.6929929
ENSG00000174013	FBXO45	3	196305706	C	A	0.370775	0.00177757	0.0160359	0.9699999	0.0767725	0.0103297	1.07E-13	0.0231537	0.208899	0.9117455	0.6193143
ENSG00000174021	GNG5	1	84968128	T	G	0.0586481	9.89E-05	0.0310709	0.89	0.419049	0.0169902	2.59E-134	0.000235961	0.0741462	0.9974608	0.7618644
ENSG00000174032	SLC25A30	13	45980020	A	G	0.0506958	0.048093	0.0289663	0.08300036	0.296038	0.0210698	7.66E-45	0.162455	0.0985272	0.0991811	0.9117571
ENSG00000174038	SPATA31G1	9	35043540	T	C	0.0656064	-0.0336211	0.0332012	0.4100001	-0.11313	0.01654	7.93E-12	0.297189	0.296676	0.316475	0.6329358
ENSG00000174059	CD34	1	208071170	T	C	0.201789	0.00131241	0.0172726	0.9400001	-0.109143	0.00935625	1.92E-31	-0.0120247	0.158261	0.9394345	0.5334935
ENSG00000174080	CTSF	11	66333623	C	T	0.244533	0.00570337	0.0165574	0.7899998	0.645463	0.00874416	0	0.00883609	0.0256523	0.7305028	0.1446623
ENSG00000174083	NA	17	8738517	C	T	0.00596421	0.107833	0.0793297	0.09599973	-0.459803	0.0712697	1.11E-10	-0.23452	0.176318	0.1834854	0.1759994
ENSG00000174093	NA	17	36379787	A	C	0.157058	0.0121902	0.0206193	0.8200001	-0.272552	0.0280784	2.82E-22	-0.0447262	0.075793	0.555117	0.4120961
ENSG00000174099	MSRB3	12	65777223	A	G	0.471173	0.00212966	0.0142525	0.9	-0.0517959	0.00795758	7.57E-11	-0.0411164	0.275239	0.8812504	0.5571954
ENSG00000174100	NA	17	36466045	T	C	0.152087	0.0050102	0.0213807	0.9299999	0.305477	0.0135445	1.24E-112	0.0164013	0.0699951	0.8147364	0.2297644
ENSG00000174106	LEMD3	12	65602729	T	C	0.302187	0.00724129	0.0151984	0.5	-0.446456	0.00826238	0	-0.0162195	0.0340437	0.6337664	0.2873629
ENSG00000174123	TLR10	4	38779235	A	C	0.249503	-0.0527118	0.0183027	2.90E-05	-0.154731	0.00984269	1.10E-55	0.340667	0.120256	0.00461344	0.05050214
ENSG00000174125	TLR1	4	38825368	T	G	0.210736	-0.0516947	0.0195246	1.00E-04	-0.612527	0.0252547	6.00E-130	0.0843957	0.0320648	0.008487542	0.003667388
ENSG00000174130	TLR6	4	38841887	A	G	0.27833	0.0167039	0.0155562	0.2300001	0.135641	0.00845726	6.89E-58	0.123148	0.114943	0.283999	0.03271635
ENSG00000174132	FAM174A	5	99896727	T	C	0.515905	0.0176133	0.0142631	0.1199999	-0.558889	0.00783967	0	-0.0315149	0.0255243	0.216942	0.2140657
ENSG00000174136	RGMB	5	98119350	T	C	0.469185	0.0122455	0.0142421	0.33	-0.405659	0.00849933	0	-0.0301867	0.0351143	0.3899707	0.9799573
ENSG00000174137	FAM53A	4	1652835	T	C	0.212724	-0.0180969	0.0180724	0.35	0.433105	0.0104789	0	-0.0417841	0.0417397	0.3167969	0.6525565
ENSG00000174151	CYB561D1	1	110041114	C	G	0.254473	-0.000918697	0.0164541	0.95	-0.143621	0.0101953	4.57E-45	0.00639669	0.114567	0.9554745	0.6500779
ENSG00000174165	ZDHHC24	11	66300908	A	G	0.00397614	-0.0558409	0.067788	0.35	-0.752235	0.0477931	8.12E-56	0.0742333	0.0902388	0.4107173	0.1731394
ENSG00000174171	LOC105370792	15	42187288	T	C	0.055666	0.0667644	0.029839	0.01400006	0.883653	0.0356441	1.12E-135	0.075555	0.033905	0.02585185	0.8326971
ENSG00000174173	TRMT10C	3	101282998	T	G	0.436382	-0.0143048	0.01433	0.2599998	-0.0552841	0.00886845	4.55E-10	0.258751	0.262509	0.3242884	0.03687068
ENSG00000174175	SELP	1	169578759	T	C	0.235586	-0.00734244	0.0161327	0.6999999	-0.126009	0.00934492	1.94E-41	0.0582693	0.128101	0.6492037	0.3927985
ENSG00000174177	CTU2	16	88777332	T	C	0.427435	0.00142381	0.0142481	0.9199999	-0.249187	0.010317	6.94E-129	-0.00571381	0.0571788	0.9204007	0.2671376
ENSG00000174194	NA	10	51298001	T	C	0.437376	0.0281599	0.0143835	0.08600031	0.801599	0.0146694	0	0.0351297	0.017955	0.05040198	0.009407345
ENSG00000174196	NA	10	51189985	G	C	0.0775348	-0.00468783	0.0271829	0.7499995	-0.623603	0.0458242	3.56E-42	0.00751733	0.0435936	0.8630906	0.2336616
ENSG00000174197	MGA	15	41987781	A	G	0.380716	0.019186	0.0149313	0.17	0.145967	0.00913961	2.04E-57	0.131441	0.102623	0.2002594	0.4688581
ENSG00000174227	PIGG	4	513487	T	C	0.0347913	-0.0325281	0.0380012	0.2999998	-0.303713	0.0225263	1.98E-41	0.107101	0.125374	0.3929633	0.423724
ENSG00000174231	PRPF8	17	1571049	A	C	0.0208748	-0.0373938	0.0550121	0.4199997	-0.264733	0.0329409	9.24E-16	0.141251	0.208544	0.4982034	0.7402929
ENSG00000174233	ADCY6	12	49171397	C	G	0.0357853	-0.0537494	0.0305855	0.08300036	-0.136324	0.0191876	1.20E-12	0.394276	0.23112	0.0880195	0.7072268
ENSG00000174238	PITPNA	17	1443561	G	A	0.119284	-0.0121665	0.0212361	0.5099998	0.579721	0.0115628	0	-0.0209868	0.036634	0.5667269	0.422984
ENSG00000174243	DDX23	12	49235232	C	T	0.331014	-0.013125	0.0150065	0.4100001	0.172215	0.00847464	8.35E-92	-0.0762129	0.0872189	0.3822206	0.1308354

ENSG00000174255	ZNF80	3	113954954	A	G	0.333996	-0.0262591	0.0153557	0.1199999	-0.244798	0.0085969	2.38E-178	0.107268	0.0628409	0.08782573	0.04705261
ENSG00000174282	ZBTB4	17	7375133	G	T	0.0417495	0.0190443	0.0373837	0.4899999	-0.38042	0.0230485	3.37E-61	-0.0500612	0.0983163	0.6106223	0.7753925
ENSG00000174292	TNK1	17	7288473	T	C	0.175944	0.00775463	0.0204782	0.91	0.14853	0.0130086	3.41E-30	0.0522091	0.137948	0.705082	0.5959011
ENSG00000174306	ZHX3	20	39876700	G	A	0.0894632	0.0772452	0.0253739	0.00179999	0.153586	0.0147866	2.85E-25	0.502946	0.17216	0.003484782	0.4228799
ENSG00000174307	PHLDA3	1	201436492	A	G	0.0497018	-0.0287886	0.0440421	0.32	0.325703	0.0242407	3.71E-41	-0.088389	0.135381	0.513828	0.6295665
ENSG00000174326	SLC16A11	17	6946095	T	C	0.214712	0.00194314	0.0169015	0.8700001	-0.112583	0.0105303	1.12E-26	-0.0172596	0.150133	0.9084754	0.9417821
ENSG00000174348	PODN	1	53539514	A	G	0.22664	-0.00288097	0.0176968	0.6600001	0.228977	0.00970297	3.98E-123	-0.0125819	0.0772883	0.870682	0.938349
ENSG00000174353	NA	7	72458336	T	C	0.0695825	-0.00879455	0.0276844	0.59	0.769433	0.0433425	1.65E-70	-0.0114299	0.035986	0.7507726	0.06118285
ENSG00000174358	SLC6A19	5	1213471	A	G	0.129225	0.00993384	0.0222443	0.8200001	-0.122918	0.0150697	3.45E-16	-0.0808169	0.18124	0.6556613	0.8803641
ENSG00000174365	SNHG11	20	37077392	T	C	0.159046	0.00868347	0.0197683	0.7400005	0.0774127	0.0122869	2.97E-10	0.112171	0.255982	0.6612423	0.06507435
ENSG00000174370	KCNJ5-AS1	11	128772695	A	T	0.290258	-0.00880648	0.0159283	0.4500005	-0.119916	0.00908926	9.60E-40	0.0734384	0.132945	0.5806759	0.000532147
ENSG00000174373	RALGAPA1	14	36143034	G	A	0.147117	-0.0403986	0.0218018	0.02900013	0.590947	0.016885	2.39E-268	-0.0683625	0.0369447	0.06425562	0.5686574
ENSG00000174374	NA	7	74465645	T	C	0.166998	-0.0214084	0.0202193	0.2300001	-0.12096	0.0158146	2.03E-14	0.176988	0.168751	0.2942663	0.1698971
ENSG00000174428	GTF2IRD2B	7	74536993	C	T	0.243539	0.00127998	0.0162154	0.95	0.267113	0.0226325	3.80E-32	0.0047919	0.0607074	0.9370849	0.07528664
ENSG00000174437	ATP2A2	12	110753729	T	C	0.0119284	-0.0463257	0.0665407	0.4700002	0.455059	0.0444836	1.46E-24	-0.101802	0.146563	0.4873096	0.9465199
ENSG00000174442	ZWILCH	15	66819706	C	A	0.175944	0.0275248	0.0221324	0.16	0.145576	0.012674	1.55E-30	0.189075	0.152922	0.2163037	0.4041948
ENSG00000174444	RPL4	15	66803612	A	G	0.431412	0.0274867	0.0143285	0.02199986	0.0502015	0.00886045	1.46E-08	0.547527	0.301336	0.06921686	0.4619309
ENSG00000174446	SNAPC5	15	66786397	C	T	0.0715706	-0.01613	0.0261845	0.4700002	-0.318195	0.0156584	8.39E-92	0.0506922	0.0823286	0.5380724	0.6481522
ENSG00000174456	C12orf76	12	110488681	C	T	0.0576541	-0.0112937	0.030352	0.6300007	-0.240098	0.0165535	1.14E-47	0.0470378	0.126457	0.7099164	0.4765843
ENSG00000174469	CNTNAP2	7	146965771	T	C	0.414513	-0.00704165	0.0142612	0.6999999	0.262003	0.00788719	5.76E-242	-0.0268762	0.0544374	0.6215122	0.7171161
ENSG00000174482	LINGO2	9	28309179	T	C	0.161034	-0.00433179	0.0185603	0.81	0.304644	0.0105228	2.74E-184	-0.0142192	0.0609266	0.8154644	0.9543809
ENSG00000174483	BBS1	11	66289587	A	G	0.388668	-0.00627013	0.0144954	0.6999999	0.121882	0.00807295	1.68E-51	-0.0514442	0.118978	0.6654634	0.6903941
ENSG00000174485	DENND4A	15	66017507	G	T	0.394632	-0.00534613	0.0143722	0.6300007	-0.0792878	0.00869421	7.54E-20	0.0674269	0.181417	0.71014	0.2199085
ENSG00000174500	GCSAM	3	111845920	G	A	0.418489	-0.00628532	0.0145155	0.5999997	-0.186728	0.00896264	2.13E-96	0.0336604	0.077753	0.6650773	0.8598867
ENSG00000174501	ANKRD36C	2	96586064	G	A	0.367793	-0.0153598	0.0148382	0.1800002	0.117954	0.00956999	6.61E-35	-0.130219	0.126239	0.3022966	0.3137001
ENSG00000174514	MFSD4A	1	205555029	G	A	0.2167	0.0209934	0.0171404	0.29	0.136559	0.0102218	1.04E-40	0.153732	0.126043	0.2225879	0.42236
ENSG00000174516	PELI3	11	66239512	C	A	0.242545	-0.0126134	0.0165801	0.4	-0.257901	0.00912072	6.74E-176	0.0489079	0.0643119	0.4469676	0.6699305
ENSG00000174527	MYO1H	12	109859926	T	A	0.0646123	-0.0068813	0.0275234	0.9299999	0.131267	0.0173556	3.93E-14	-0.0524221	0.209789	0.8026803	0.2516311
ENSG00000174529	TMEM81	1	205052951	T	G	0.425447	0.012179	0.0142818	0.5	-0.125925	0.0081415	5.79E-54	-0.0967166	0.113588	0.3945081	0.3134197
ENSG00000174564	IL20RB	3	136697499	A	G	0.238569	0.00643359	0.0173919	0.8600001	-0.0645984	0.0100355	1.22E-10	-0.0995936	0.269675	0.7118971	0.9979259
ENSG00000174574	AKIRIN1	1	39464313	C	T	0.141153	0.00839172	0.0221217	0.8499999	-0.200409	0.0108284	1.79E-76	-0.0418731	0.110406	0.7044921	0.4912796
ENSG00000174579	MSL2	3	135891923	T	G	0.426441	0.0124483	0.0144903	0.4100001	-0.0896308	0.00888877	6.53E-24	-0.138884	0.162252	0.3920103	0.8702456
ENSG00000174586	ZNF497	19	58869968	T	G	0.400596	-0.0161373	0.014669	0.2999998	0.0780433	0.00881846	8.75E-19	-0.206774	0.189406	0.2749678	0.2998523
ENSG00000174600	CMKLR1	12	108707469	C	T	0.385686	-0.00855724	0.0145804	0.5300002	0.497866	0.00775512	0	-0.0171878	0.029287	0.5572872	0.4915886
ENSG00000174606	ANGEL2	1	213177346	G	C	0.353877	-0.0127345	0.0146855	0.29	0.35659	0.00800935	0	-0.0357119	0.041191	0.3859511	0.5656621
ENSG00000174607	UGT8	4	115559495	G	A	0.163022	-0.00718484	0.0211428	0.8800001	0.236264	0.0111768	3.50E-99	-0.0304102	0.0894995	0.7340218	0.05326348
ENSG00000174611	KY	3	134346229	A	G	0.329026	0.00217256	0.0147984	0.89	-0.134267	0.00837152	6.87E-58	-0.0161808	0.11022	0.8832865	0.5741884
ENSG00000174628	IQCK	16	19798342	A	G	0.00894632	-0.0741646	0.200041	0.5300002	0.907319	0.0658174	3.12E-43	-0.0817404	0.220555	0.7109258	0.8867424
ENSG00000174652	ZNF266	19	9534763	G	C	0.439364	-0.00458807	0.0143514	0.7300002	-0.927703	0.00607315	0	0.00494562	0.0154698	0.7492001	0.656395
ENSG00000174669	SLC29A2	11	66134838	G	C	0.33499	0.00305031	0.0154641	1	0.0584538	0.00931564	3.50E-10	0.0521833	0.264683	0.8437073	0.2087523
ENSG00000174672	BRSK2	11	1447524	C	T	0.483101	-0.0260456	0.0142779	0.03899959	-0.0619759	0.00931744	2.90E-11	0.420254	0.238885	0.07853822	0.1407553
ENSG00000174680	NA	21	31128407	G	C	0.11829	0.0265353	0.0231744	0.2099999	0.187443	0.0149395	4.14E-36	0.141565	0.124148	0.2541664	0.106296
ENSG00000174684	B4GAT1	11	66114003	T	C	0.215706	-0.0116635	0.017757	0.4299995	-0.150716	0.0106403	1.52E-45	0.0773873	0.117944	0.5117376	0.7100709
ENSG00000174697	LEP	7	127889509	G	C	0.440358	0.00520555	0.0143226	0.7499995	0.0754821	0.00809077	1.06E-20	0.0689641	0.189892	0.7164745	0.438723
ENSG00000174705	SH3PXD2B	5	171816856	A	G	0.380716	-0.00476633	0.0148169	0.7300002	-0.261945	0.00838173	2.10E-214	0.0181959	0.0565679	0.7477065	0.5229861
ENSG00000174718	RESF1	12	32129171	G	A	0.413519	0.043926	0.0145321	0.001700004	0.305212	0.00881208	7.36E-263	0.14392	0.0477942	0.002601812	0.01688197
ENSG00000174720	LARP7	4	113568434	C	T	0.117296	0.0112917	0.024208	0.4799997	0.181652	0.0136935	3.66E-40	0.0621612	0.133348	0.6411032	0.8604275
ENSG00000174721	FGFBP3	10	93667793	T	C	0.0864811	0.0254692	0.0281263	0.3700002	0.119374	0.016426	3.66E-13	0.213355	0.237436	0.3688758	0.8816014
ENSG00000174738	NR1D2	3	24004430	A	G	0.0666004	-0.0301915	0.02762	0.25	0.227447	0.0152161	1.61E-50	-0.132741	0.121759	0.2756287	0.7885162
ENSG00000174744	BRMS1	11	66108700	G	A	0.0914513	0.0178642	0.0255807	0.5199996	-0.114151	0.0151785	5.45E-14	-0.156496	0.225059	0.4868326	0.1044073

ENSG00000174748	RPL15	3	23961609	C	T	0.44831	0.0207538	0.0142413	0.08100093	0.0510408	0.00806714	2.50E-10	0.406612	0.286323	0.1555746	0.05779072
ENSG00000174749	FAM241A	4	113088395	T	G	0.298211	0.0267844	0.0154472	0.08400014	-0.373188	0.00834574	0	-0.0717718	0.0414236	0.08316141	0.798824
ENSG00000174775	HRAS	11	534764	T	G	0.202783	-0.00143081	0.017085	0.9	-0.293897	0.0115326	2.97E-143	0.00486841	0.0581329	0.9332583	0.4954114
ENSG00000174776	WDR49	3	167284121	C	T	0.0735586	-0.0016486	0.0271657	0.9299999	0.128041	0.0175264	2.76E-13	-0.0128756	0.212172	0.9516103	0.1020642
ENSG00000174780	SRP72	4	57351460	G	A	0.212724	-0.00900272	0.0177783	0.5999997	-0.330999	0.00988692	9.91E-246	0.0271986	0.0537172	0.6126249	0.4441805
ENSG00000174788	PCP2	19	7697565	G	A	0.385686	0.0126789	0.0147031	0.3100002	-0.428343	0.0117231	2.72E-292	-0.0295999	0.0343351	0.3886389	0.9555855
ENSG00000174791	RIN1	11	66101012	A	G	0.0894632	0.0181419	0.0257326	0.4899999	-0.781904	0.0145058	0	-0.0232022	0.032913	0.4808377	0.00764986
ENSG00000174792	ODAPH	4	76486176	A	G	0.451292	-0.00943406	0.0141971	0.4500005	0.0743533	0.00828616	2.88E-19	-0.126881	0.191464	0.5075282	0.5854458
ENSG00000174796	THAP6	4	76457419	T	C	0.389662	0.0244146	0.0145148	0.0649995	0.399385	0.009412	0	0.0611305	0.0363714	0.09281562	0.7874646
ENSG00000174799	CEP135	4	56857283	C	T	0.437376	-0.000616949	0.0143355	0.98	-0.139215	0.00877234	1.03E-56	0.00443162	0.102974	0.9656726	0.01328242
ENSG00000174807	CD248	11	66083236	T	C	0.481113	-0.0106409	0.0142269	0.3700002	0.0435666	0.00798303	4.83E-08	-0.244245	0.329608	0.4586841	0.7716646
ENSG00000174837	ADGRE1	19	6914023	C	T	0.271372	-0.0234984	0.0155809	0.1299999	0.56149	0.00805435	0	-0.0418501	0.0277557	0.1316054	0.7286862
ENSG00000174839	DENND6A	3	57645000	G	A	0.171968	0.0185019	0.0180514	0.3900004	0.235508	0.0113716	2.80E-95	0.0785618	0.0767427	0.3059753	0.3593521
ENSG00000174840	PDE12	3	57544843	A	G	0.472167	0.00622687	0.0142961	0.6800001	0.120762	0.00878503	5.35E-43	0.051563	0.118441	0.6633115	0.4638652
ENSG00000174851	YIF1A	11	66054346	G	A	0.173956	0.0117218	0.019025	0.6200004	-0.192082	0.0107529	2.28E-71	-0.0610251	0.0991053	0.5380529	0.4008639
ENSG00000174871	CNIH2	11	66049216	A	G	0.172962	0.0189088	0.0192052	0.3900004	0.113974	0.0109818	3.11E-25	0.165905	0.169262	0.3270042	0.8506539
ENSG00000174885	NLRP6	11	281862	G	C	0.244533	0.00456698	0.0165771	0.5999997	-0.221209	0.00920241	1.11E-127	-0.0206455	0.0749435	0.7829465	0.5876611
ENSG00000174886	NDUFA11	19	5897652	G	A	0.55666	-0.0249186	0.0142036	0.0530005	-0.124302	0.00899698	2.04E-43	0.200468	0.115184	0.08178693	0.4053824
ENSG00000174891	RSRC1	3	158043581	C	A	0.233598	-0.00940437	0.0159836	0.6800001	-0.325062	0.0131269	2.25E-135	0.028931	0.0491849	0.5563912	0.7785982
ENSG00000174915	PTDSS2	11	469830	G	A	0.311133	-0.0181742	0.0152826	0.1900002	-0.171078	0.00994076	2.24E-66	0.106233	0.0895439	0.2354724	0.4121105
ENSG00000174917	MICOS13	19	5679669	A	G	0.0238569	-0.0107363	0.0551515	0.81	0.268032	0.0272418	7.65E-23	-0.040056	0.205805	0.8456817	0.7183679
ENSG00000174928	C3orf33	3	155502270	C	T	0.364811	-0.0259571	0.0146205	0.05600025	0.0671177	0.00824218	3.85E-16	-0.38674	0.222951	0.08280412	0.9542222
ENSG00000174943	KCTD13	16	29927344	A	G	0.482107	-0.0130911	0.0142755	0.2	-0.0911239	0.00794205	1.79E-30	0.143663	0.15716	0.3606551	0.2768375
ENSG00000174944	P2RY14	3	150963080	T	G	0.187873	-0.00801682	0.0190935	0.6700003	-0.412409	0.0105802	0	0.019439	0.0463002	0.674597	0.19767
ENSG00000174945	AMZ1	7	2761957	A	C	0.184891	0.0199785	0.0193497	0.25	0.0952457	0.0120794	3.15E-15	0.209757	0.20489	0.30595	0.5722137
ENSG00000174946	GPR171	3	150918303	T	C	0.491054	-0.0197172	0.0142322	0.1100001	-0.0681979	0.00802706	1.96E-17	0.289117	0.211446	0.1715206	0.9785106
ENSG00000174950	CD164L2	1	27707768	A	G	0.120278	-0.0118509	0.0256301	0.4299995	0.342402	0.0155483	1.78E-107	-0.034611	0.0748702	0.6438804	0.722876
ENSG00000174953	DHX36	3	154016310	A	T	0.23161	-0.00276001	0.0162579	0.9199999	0.344854	0.00916272	41967299999999e-3	-0.00800342	0.0471448	0.8651969	0.7326351
ENSG00000174989	FBXW8	12	117408857	C	T	0.0964215	-0.0187123	0.0241573	0.4700002	0.483286	0.0131613	3.48E-295	-0.0387189	0.0499966	0.4386765	0.4289512
ENSG00000174996	KLC2	11	66030048	T	C	0.171968	0.0231119	0.0192213	0.29	-0.0937321	0.0104637	3.31E-19	-0.246574	0.206905	0.23337	0.9138574
ENSG00000175003	SLC22A1	6	160561285	G	C	0.253479	0.0166327	0.0158408	0.28	-0.229087	0.00866311	4.28E-154	-0.0726042	0.0692019	0.2941024	0.2286143
ENSG00000175029	CTBP2	10	126763080	A	G	0.274354	0.0363634	0.0156966	0.01	0.0610578	0.00904802	1.50E-11	0.595557	0.271805	0.02844299	0.1937143
ENSG00000175040	CHST2	3	142839986	C	T	0.432406	0.0111486	0.0143671	0.5400003	-0.110765	0.00807712	8.44E-43	-0.100651	0.129916	0.4384922	0.5238749
ENSG00000175048	ZDHHC14	6	157950671	T	G	0.404573	0.0154981	0.0144935	0.25	-0.231044	0.00809229	2.71E-179	-0.0670784	0.0627743	0.2852659	0.8757686
ENSG00000175061	SNHG29	17	16362064	T	C	0.2833	-0.0125034	0.0156218	0.2399999	0.215461	0.00991564	1.08E-104	-0.058031	0.0725533	0.423804	0.1572701
ENSG00000175063	UBE2C	20	44443405	G	T	0.395626	0.0222134	0.0147027	0.1499999	-0.0761299	0.00822617	2.15E-20	-0.291783	0.195683	0.1359355	0.2451081
ENSG00000175066	GK5	3	141913431	C	T	0.140159	-0.012799	0.0200589	0.32	-0.197963	0.016778	3.95E-32	0.0646533	0.101474	0.5240337	0.03767281
ENSG00000175084	DES	2	220287280	A	C	0.343936	0.00892119	0.0152542	0.6999999	-0.131903	0.00847695	1.36E-54	-0.0676344	0.115729	0.5589362	0.485104
ENSG00000175104	TRAF6	11	36520199	A	T	0.143141	0.00115519	0.0198883	0.7700005	-0.0940786	0.0113083	8.84E-17	-0.012279	0.211406	0.9536829	0.8770954
ENSG00000175105	ZNF654	3	88191034	G	C	0.157058	0.00803356	0.0195924	0.3800004	-0.320069	0.0122053	1.42E-151	-0.0250994	0.0612205	0.6818174	0.05425939
ENSG00000175106	TVP23C	17	15404057	T	C	0.327038	0.00959651	0.0152638	0.59	-0.153464	0.0094744	5.23E-59	-0.0625325	0.0995364	0.5298485	0.3616977
ENSG00000175110	MRPS22	3	138900356	G	T	0.368787	0.00302772	0.0144659	0.7400005	0.0504731	0.00804507	3.52E-10	0.0599868	0.286765	0.8343045	0.7084002
ENSG00000175115	PACS1	11	65925026	C	T	0.26839	0.0259499	0.015997	0.1100001	-0.236316	0.00893843	4.99E-154	-0.10981	0.0678206	0.1054197	0.5088452
ENSG00000175130	MARCKSL1	1	32800706	A	G	0.0328032	-0.0165801	0.0368575	0.5099998	0.101349	0.0178898	1.47E-08	-0.163594	0.364813	0.6538416	0.6490546
ENSG00000175137	SH3BP5L	1	249112740	C	T	0.11829	0.00879539	0.0212811	0.6300007	-0.237597	0.0115603	7.26E-94	-0.0370181	0.0895862	0.6794511	0.1579997
ENSG00000175155	YPEL2	17	57444070	A	G	0.442346	-0.0252023	0.0143082	0.04499974	-0.181516	0.00805734	2.21E-112	0.138844	0.0790668	0.0790834	0.5242742
ENSG00000175161	CADM2	3	85565855	C	T	0.347913	-0.00148152	0.0149444	0.9299999	0.0585443	0.00862925	1.17E-11	-0.025306	0.255294	0.9210391	0.9598437
ENSG00000175164	ABO	9	136140835	T	C	0.282306	-0.0467594	0.0162659	8.50E-05	-0.657473	0.015682	0	0.0711199	0.0247981	0.004131343	0.7569702
ENSG00000175166	PSMD2	3	184021669	T	G	0.471173	0.0275755	0.0142158	0.05800027	-0.273975	0.0116421	1.87E-122	-0.10065	0.0520632	0.05320886	0.2261031
ENSG00000175170	NA	20	25796481	G	A	0.321074	0.0231336	0.0157707	0.06699926	0.368902	0.0217793	2.35E-64	0.0627093	0.0429104	0.1439051	0.1492997

ENSG00000175182	FAM131A	3	184058888	T	C	0.248509	0.0007237	0.0170577	0.9599999	-0.081291	0.00970848	5.61E-17	-0.00890258	0.209838	0.9661591	0.06723805
ENSG00000175193	PARL	3	183574947	T	C	0.451292	0.0194257	0.0142545	0.08700015	-0.162919	0.00800995	5.74E-92	-0.119235	0.0876905	0.173916	0.2554251
ENSG00000175197	DDIT3	12	57912335	A	G	0.17495	0.0123526	0.0207292	0.5300002	0.19095	0.0117857	4.90E-59	0.0646901	0.108632	0.5515088	0.6319947
ENSG00000175198	PCCA	13	100961977	C	G	0.394632	-0.00745459	0.0144174	0.5500004	0.195224	0.00790119	8.73E-135	-0.0381848	0.0738666	0.6051967	0.308251
ENSG00000175203	DCTN2	12	57932499	G	C	0.385686	-0.015406	0.0147618	0.4700002	-0.0471611	0.00826808	1.17E-08	0.326668	0.318204	0.30461	0.2063966
ENSG00000175206	NPPA	1	11907084	C	T	0.12326	0.010038	0.0199032	0.5999997	0.344588	0.0136701	3.32E-140	0.0291305	0.057771	0.6140927	0.4565331
ENSG00000175220	ARHGAP1	11	46710397	A	G	0.135189	-0.0137958	0.0218279	0.4199997	0.168598	0.0126086	8.86E-41	-0.0818267	0.129612	0.5278306	0.2912055
ENSG00000175221	MED16	19	880589	A	G	0.256461	0.00925442	0.0161581	0.6100002	0.616187	0.00992284	0	0.0150188	0.0262238	0.566836	0.4753741
ENSG00000175224	ATG13	11	46667597	T	C	0.0795229	0.0343855	0.0259862	0.2200002	-0.170934	0.0158766	4.96E-27	-0.201162	0.153168	0.1890685	0.1760423
ENSG00000175262	C1orf127	1	11024311	G	A	0.271372	-0.0106018	0.016981	0.32	-0.0659956	0.0102331	1.12E-10	0.160644	0.258508	0.5343181	0.5604325
ENSG00000175265	GOLGA8A	15	34775986	A	G	0.0119284	0.0274944	0.0609053	0.6700003	1.28648	0.0759359	2.22E-64	0.0213719	0.0473596	0.651796	0.5103083
ENSG00000175274	TP53I11	11	44940147	C	T	0.306163	-0.0193702	0.0160685	0.32	0.183221	0.00909527	3.00E-90	-0.105721	0.0878572	0.228851	0.399831
ENSG00000175279	CENPS	1	10501184	A	G	0.0685885	-0.0157107	0.0273556	0.6499995	0.323343	0.0161002	1.04E-89	-0.0485884	0.0846371	0.5659142	0.2556939
ENSG00000175287	PHYHD1	9	131693747	A	G	0.409543	-0.00948711	0.0145195	0.33	-0.11775	0.00818051	5.64E-47	0.0805698	0.123435	0.5139287	0.3299141
ENSG00000175294	CATSPER1	11	65789105	C	T	0.446322	0.0263432	0.0142234	0.05	-0.0968094	0.00799774	9.99E-34	-0.272114	0.148632	0.067131	0.5668553
ENSG00000175305	CCNE2	8	95900452	A	G	0.358847	0.000580312	0.0144926	0.7800007	-0.0754457	0.00804668	6.85E-21	-0.00769179	0.192095	0.96806	0.3741033
ENSG00000175309	PHYKPL	5	177647645	C	T	0.337972	-0.00467935	0.0152535	0.8200001	0.137768	0.010579	9.08E-39	-0.0339655	0.11075	0.7590815	0.1234997
ENSG00000175322	ZNF519	18	14094972	C	G	0.229622	0.00850676	0.0164491	0.4500005	0.206548	0.0101323	2.26E-92	0.0411853	0.0796636	0.605163	0.2319782
ENSG00000175334	BANF1	11	65770585	G	T	0.440358	0.0199741	0.0142479	0.1299999	-0.337215	0.00782397	0	-0.0592325	0.042274	0.1611672	0.1759432
ENSG00000175348	TMEM9B	11	8977699	T	G	0.0586481	-0.0156362	0.0271277	0.5300002	-0.681446	0.0141973	0	0.0229456	0.0398119	0.5643781	0.9862166
ENSG00000175352	NRIP3	11	9013859	A	G	0.373757	-0.000708607	0.0147472	0.8700001	-0.129024	0.00814708	1.73E-56	0.00549207	0.114299	0.9616764	0.8122228
ENSG00000175354	PTPN2	18	12857559	C	T	0.306163	-0.00503043	0.0157691	0.6899999	0.108752	0.00867435	4.67E-36	-0.0462559	0.145047	0.7498006	0.247142
ENSG00000175376	EIF1AD	11	65766831	G	C	0.0238569	0.055302	0.0460068	0.2399999	0.229897	0.0277091	1.07E-16	0.240552	0.202209	0.234196	0.2807476
ENSG00000175387	SMAD2	18	45407718	T	G	0.457256	0.00372482	0.0142802	0.99	0.121431	0.00956346	6.11E-37	0.0306743	0.117624	0.7942598	0.09315425
ENSG00000175390	EIF3F	11	8007603	C	T	0.276342	0.00475868	0.0160031	0.8800001	0.21093	0.00957779	1.74E-107	0.0225605	0.0758762	0.7662125	0.4476282
ENSG00000175395	ZNF25	10	38252030	G	A	0.101392	0.0219913	0.0253472	0.4	0.431004	0.014765	2.53E-187	0.0510234	0.0588356	0.3858216	0.6275737
ENSG00000175414	ARL10	5	175810668	T	C	0.0576541	0.0594585	0.0321792	0.1199999	-0.2444	0.0171398	3.93E-46	-0.243283	0.132767	0.06689028	0.7029404
ENSG00000175416	CLTB	5	175831513	A	G	0.442346	-0.00780012	0.0144575	0.7199992	0.412803	0.0080903	0	-0.0188955	0.0350248	0.5895484	0.2099796
ENSG00000175445	LPL	8	19791998	G	C	0.0984095	0.00500162	0.0258033	0.9400001	0.863847	0.0145349	0	0.00578994	0.0298704	0.8463048	0.1975748
ENSG00000175455	CCDC14	3	123648358	T	C	0.0149105	-0.0663142	0.109737	0.35	-0.325857	0.0374221	3.10E-18	0.203507	0.337574	0.5466077	0.9423256
ENSG00000175463	TBC1D10C	11	67174473	C	T	0.0755467	-0.0119084	0.0294131	0.3900004	-0.186243	0.0162488	2.05E-30	0.06394	0.158027	0.6857606	0.5141569
ENSG00000175467	SART1	11	65738229	T	C	0.429423	0.0190655	0.0143311	0.1800002	-0.104111	0.0080375	2.25E-38	-0.183126	0.138376	0.1857034	0.1494062
ENSG00000175470	PPP2R2D	10	133760643	A	G	0.525845	0.00597311	0.0142415	0.81	-0.197744	0.0117266	8.45E-64	-0.0302063	0.0720423	0.675007	0.9313494
ENSG00000175471	MCTP1	5	94329862	T	A	0.491054	0.00112795	0.0142171	0.9400001	-0.488092	0.00796568	0	-0.00231094	0.0291279	0.9367642	0.5354656
ENSG00000175482	POLD4	11	67121345	G	A	0.0705765	-0.00408321	0.0313943	0.5700002	-0.162866	0.0164249	3.55E-23	0.025071	0.192778	0.8965261	0.4989288
ENSG00000175489	LRRC25	19	18505190	T	A	0.326044	0.00415172	0.0150825	0.7400005	0.522918	0.00995122	0	0.00793953	0.0288434	0.7831137	0.05571899
ENSG00000175505	CLCF1	11	67136643	G	T	0.11332	-0.0140456	0.0240936	0.7700005	-0.136184	0.0133603	2.13E-24	0.103137	0.177209	0.5605608	0.3337532
ENSG00000175513	TSGA10IP	11	65720175	T	C	0.440358	0.0184977	0.01423	0.1499999	-0.146672	0.0155054	3.10E-21	-0.126116	0.0979309	0.1978135	0.3237176
ENSG00000175518	UBQLNL	11	5536779	C	T	0.295229	0.00748055	0.0154854	0.4700002	0.480509	0.00841234	0	0.015568	0.0322283	0.6290575	0.5144073
ENSG00000175536	LIPT2	11	74203767	A	G	0.397614	-0.000878872	0.0146502	0.9699999	0.0727222	0.00905	9.31E-16	-0.0120853	0.20146	0.9521646	0.1824719
ENSG00000175538	KCNE3	11	74172330	G	A	0.455268	0.0144512	0.0144311	0.2700001	0.184723	0.00811392	9.92E-115	0.0782317	0.0781985	0.3171046	0.03601889
ENSG00000175548	ALG10B	12	38714082	T	A	0.0258449	0.0757844	0.0432063	0.07900053	-0.20762	0.022243	1.02E-20	-0.365015	0.211745	0.08473648	0.9199188
ENSG00000175550	DRAP1	11	65687880	T	G	0.0258449	0.0454005	0.0456029	0.33	0.234175	0.0253081	2.18E-20	0.193874	0.195862	0.322248	0.2856399
ENSG00000175564	UCP3	11	73715903	G	A	0.26839	-0.0266358	0.015733	0.08600031	-0.165831	0.00873376	2.17E-80	0.16062	0.0952499	0.09173816	0.368141
ENSG00000175567	UCP2	11	73690032	G	T	0.134195	-0.0125661	0.0205016	0.5500004	0.57867	0.0107505	0	-0.0217155	0.0354311	0.5399471	0.5513207
ENSG00000175573	C11orf68	11	65685433	G	A	0.0238569	0.0544383	0.047157	0.2200002	0.564653	0.0311471	1.90E-73	0.0964103	0.0836842	0.2492915	0.06925876
ENSG00000175575	PAAF1	11	73613267	A	G	0.0815109	-0.0195542	0.0285187	0.4500005	-1.10085	0.0130403	0	0.0177628	0.0259069	0.4929403	0.8757764
ENSG00000175581	MRPL48	11	73537269	T	G	0.186879	0.0198505	0.0185575	0.4	0.511277	0.0146281	1.22E-267	0.0388253	0.0363133	0.2849909	0.6576805
ENSG00000175582	RAB6A	11	73429560	G	A	0.307157	-0.00316568	0.015897	0.6499995	-0.262825	0.00862188	4.31E-204	0.0120448	0.0604864	0.8421589	0.9776991
ENSG00000175591	P2RY2	11	72938370	A	C	0.225646	-0.0294977	0.0159017	0.0530005	0.301206	0.00905865	1.99E-242	-0.0979318	0.0528754	0.06400817	0.04772267

ENSG00000175595	ERCC4	16	14030108	T	C	0.338966	0.00408302	0.014985	0.98	0.0511224	0.00849377	1.76E-09	0.0798676	0.29342	0.7854721	0.3363297
ENSG00000175600	SUGCT	7	40537468	C	A	0.0218688	0.04622	0.0473729	0.17	0.451349	0.0362558	1.42E-35	0.102404	0.10528	0.3307121	0.5370306
ENSG00000175602	CCDC85B	11	65658490	A	G	0.183897	0.0332048	0.0179847	0.07599937	-0.0725078	0.0106254	8.85E-12	-0.457948	0.256956	0.07471616	0.3551719
ENSG00000175606	TMEM70	8	74889845	G	A	0.168986	-0.0190362	0.0202897	0.2	-0.204784	0.010767	1.17E-80	0.0929577	0.0991993	0.3487172	0.1568704
ENSG00000175611	ERCC6L2-AS1	9	98579886	C	A	0.133201	0.019338	0.020001	0.1800002	0.88777	0.0186608	0	0.0217827	0.0225341	0.333718	0.0498433
ENSG00000175634	RPS6KB2	11	67199401	A	G	0.398608	-0.0132187	0.014362	0.4600002	0.325587	0.00859413	0	-0.0405996	0.0441241	0.3575087	0.9645114
ENSG00000175643	RMI2	16	11394547	A	G	0.308151	-0.00134061	0.0150511	0.9699999	0.573061	0.00889625	0	-0.00233939	0.0262644	0.9290259	0.1360251
ENSG00000175662	TOM1L2	17	17811282	T	C	0.0337972	-0.0370293	0.038864	0.4899999	0.19769	0.0263995	6.97E-14	-0.18731	0.198176	0.3445712	0.6978735
ENSG00000175691	ZNF77	19	2939092	A	G	0.0487078	-0.00361014	0.0317263	0.8700001	0.829434	0.0243296	9.86E-255	-0.00435253	0.0382507	0.9094047	0.8769081
ENSG00000175701	MTLN	2	110986051	G	C	0.0397614	-0.0239448	0.0363768	0.6499995	0.808437	0.0283898	2.30E-178	-0.0296186	0.0450085	0.5104945	0.7126321
ENSG00000175711	B3GNTL1	17	80954858	T	C	0.296223	0.0172744	0.0160516	0.4400003	0.448502	0.00864299	0	0.0385158	0.035797	0.2819505	0.2739618
ENSG00000175727	MLXIP	12	122572947	G	T	0.441352	0.0132684	0.0142974	0.3800004	-0.172809	0.0117997	1.45E-48	-0.0767808	0.0829013	0.354357	0.5750203
ENSG00000175730	NA	20	31277769	A	G	0.485089	-0.0313269	0.0142472	0.02399993	-0.152668	0.0189778	8.65E-16	0.205196	0.0967443	0.03392098	0.8540446
ENSG00000175746	LINC02915	15	39544965	G	A	0.0755467	-0.00812631	0.0243709	0.7600007	-0.293247	0.0136681	4.12E-102	0.0277115	0.0831172	0.7388302	0.3333105
ENSG00000175756	AURKAIP1	1	1309992	T	C	0.00894632	-0.139297	0.0834782	0.08100093	1.0529	0.184061	1.06E-08	-0.132298	0.0825881	0.1091776	0.9038702
ENSG00000175764	TTLL11	9	124720046	C	G	0.372763	-0.0230545	0.0149961	0.1199999	0.0857309	0.00839117	1.67E-24	-0.268917	0.17689	0.1284478	0.4560575
ENSG00000175768	TOMM5	9	37587641	T	A	0.158052	-0.0276744	0.0198642	0.14	0.424079	0.0115317	4.65E-296	-0.0652577	0.0468744	0.1638676	0.7886091
ENSG00000175782	SLC35E3	12	69163815	T	C	0.234592	-0.0132527	0.0167642	0.5	-0.433437	0.009411	0	0.0305759	0.0386831	0.4292829	0.2747741
ENSG00000175787	ZNF169	9	97042664	T	C	0.394632	-0.00998791	0.0145034	0.6100002	0.15377	0.0081445	1.66E-79	-0.0649536	0.0943816	0.4913254	0.336632
ENSG00000175792	RUVBL1	3	127828189	A	G	0.244533	0.00262657	0.0165529	0.9	0.209869	0.0100058	1.12E-97	0.0125153	0.0788749	0.8739267	0.5243379
ENSG00000175793	SFN	1	27190290	T	C	0.498012	-0.0239839	0.0143053	0.07499978	-0.0936848	0.00800848	1.30E-31	0.256006	0.154256	0.09699163	0.8988048
ENSG00000175800	OR52B3P	11	4399984	C	G	0.363817	0.00540664	0.0147685	0.6200004	-0.629842	0.0207117	4.05E-203	-0.00858411	0.0234496	0.7143157	0.7992295
ENSG00000175806	MSRA	8	10099089	C	G	0.451292	-0.0226423	0.0143647	0.1900002	0.319316	0.00790221	0	-0.0709087	0.04502	0.1152453	0.8660006
ENSG00000175826	CTDNEP1	17	7151360	A	G	0.372763	-0.00849362	0.0146233	0.7600007	0.366538	0.0120591	6.38E-203	-0.0231726	0.039903	0.5614279	0.9558483
ENSG00000175854	SWI5	9	131044463	A	C	0.141153	0.0151613	0.021583	0.6200004	0.0808978	0.0133437	1.34E-09	0.187413	0.268578	0.4853045	0.1750433
ENSG00000175857	GAPT	5	57790089	A	G	0.362823	0.000886297	0.0146696	0.91	-0.676722	0.00722724	0	-0.00130969	0.0216774	0.9518233	0.4315375
ENSG00000175866	BAIAP2	17	79050090	C	A	0.455268	0.0106182	0.01435	0.3900004	0.286319	0.00886805	1.08E-228	0.0370852	0.0501321	0.4594517	0.4840455
ENSG00000175873	NA	7	2482441	A	T	0.181909	-0.00879501	0.0201159	0.5999997	0.919692	0.0227839	0	-0.009563	0.0218737	0.6619727	0.6767548
ENSG00000175893	ZDHHC21	9	14654499	C	G	0.420477	0.0188082	0.0143798	0.16	-0.193058	0.0080475	3.55E-127	-0.0974226	0.074595	0.1915457	0.8483789
ENSG00000175894	TSPEAR	21	46024635	C	T	0.333996	-0.00534208	0.015121	0.7199992	0.134992	0.010397	1.51E-38	-0.0395733	0.112055	0.7239699	0.6178412
ENSG00000175895	PLEKHF2	8	96157472	A	G	0.170974	-0.0181272	0.0200006	0.2999998	0.303199	0.0107231	6.94E-176	-0.0597865	0.0659992	0.365006	0.2571613
ENSG00000175898	NA	19	10332990	T	C	0.184891	-0.00388909	0.0189652	0.7700005	-0.294978	0.0198049	3.59E-50	0.0131844	0.0642998	0.8375369	0.4538526
ENSG00000175899	A2M	12	9244542	G	C	0.264414	0.0036919	0.0154212	0.6899999	-0.205963	0.00964941	4.38E-101	-0.0179251	0.0748784	0.8108039	0.04509813
ENSG00000175906	ARL4D	17	41477416	T	C	0.304175	-0.028297	0.015112	0.04499974	0.127911	0.00855065	1.36E-50	-0.221224	0.119067	0.06317096	0.3212389
ENSG00000175911	NA	17	78978033	C	T	0.405567	0.000938335	0.0142997	0.8499999	-0.117761	0.0142086	1.15E-16	-0.00796814	0.121434	0.9476826	0.5012753
ENSG00000175920	DOK7	4	3484116	A	G	0.449304	0.000621895	0.0142406	0.9199999	-0.320425	0.0101508	1.06E-218	-0.00194085	0.044443	0.965167	0.4109991
ENSG00000175928	LRRN1	3	3865254	A	G	0.206759	0.00141796	0.0171844	0.84	-0.270938	0.00942662	1.15E-181	-0.00523352	0.0634258	0.934238	0.8085759
ENSG00000175931	UBE2O	17	74417410	G	A	0.359841	-0.0107971	0.0147588	0.3800004	-0.190848	0.00830921	9.69E-117	0.0565745	0.0773722	0.4646573	0.6504641
ENSG00000175938	ORAI3	16	30964084	A	G	0.0984095	-0.00900304	0.0223527	0.7300002	0.0754542	0.0132223	1.15E-08	-0.119318	0.296979	0.6878515	0.1585253
ENSG00000175970	UNC119B	12	121154840	C	T	0.422465	-0.0158533	0.0145097	0.2300001	-0.270802	0.00875965	7.58E-210	0.058542	0.0536139	0.274869	0.9558044
ENSG00000175984	DENND2C	1	115169256	A	C	0.0367793	-0.00205267	0.0348849	1	0.439943	0.0224957	3.61E-85	-0.00466576	0.0792944	0.9530788	0.1809017
ENSG00000175985	PLEKHD1	14	69973312	C	T	0.256461	-0.00615975	0.0170424	0.6100002	0.31612	0.010768	1.93E-189	-0.0194855	0.0539152	0.7177934	0.31966
ENSG00000176014	TUBB6	18	12325993	G	A	0.134195	0.0540901	0.0234841	0.02699977	0.930158	0.0114482	0	0.0581515	0.0252576	0.02131601	0.2001034
ENSG00000176022	B3GALT6	1	1169025	G	A	0.100398	0.0662177	0.022051	0.000619998	-0.8069	0.0142347	0	-0.0820643	0.0273664	0.002711081	0.4709611
ENSG00000176024	ZNF613	19	52441206	G	T	0.468191	-0.00494839	0.0142717	0.7499995	0.358677	0.00780299	0	-0.0137962	0.039791	0.7288035	0.00690931
ENSG00000176049	JAKMIP2	5	147065164	T	G	0.477137	-0.0072067	0.0142351	0.5999997	0.534073	0.0073923	0	-0.0134939	0.0266545	0.612681	0.7182817
ENSG00000176054	RPL23P2	21	30370144	G	C	0.341948	-0.00518872	0.0146491	0.7800007	0.15053	0.0220946	9.56E-12	-0.0344696	0.097448	0.7235466	0.198097
ENSG00000176055	MBLAC2	5	89762302	G	A	0.33002	0.0323946	0.0155541	0.05600025	0.121031	0.00853992	1.36E-45	0.267655	0.129894	0.03934371	0.5122881
ENSG00000176058	TPRN	9	140092357	G	A	0.242545	0.00945793	0.0163769	0.7800007	0.103168	0.0106664	3.96E-22	0.0916752	0.159023	0.5642842	0.2110768
ENSG00000176083	ZNF683	1	26694569	C	T	0.187873	0.0320857	0.0180529	0.04099964	-0.320259	0.00954421	7.53E-247	-0.100187	0.0564488	0.07592634	0.02414953

ENSG00000176087	SLC35A4	5	139946364	C	T	0.281312	0.00263002	0.0154698	0.9699999	-0.2533	0.00847527	2.91E-196	-0.010383	0.0610741	0.8650045	0.2603462
ENSG00000176092	CRYBG2	1	26664485	T	G	0.287276	0.00771273	0.0154512	0.6200004	0.106094	0.00880182	1.86E-33	0.0726974	0.145762	0.6179629	0.01009982
ENSG00000176101	SSNA1	9	140083960	A	C	0.251491	0.00553943	0.016293	0.9400001	0.171131	0.011765	6.21E-48	0.0323695	0.0952336	0.7339352	0.395961
ENSG00000176102	CSTF3	11	33141325	T	A	0.2833	0.0336176	0.0157657	0.04200007	0.166406	0.00878807	5.83E-80	0.202022	0.0953414	0.03409619	0.000694107
ENSG00000176105	YES1	18	767067	T	C	0.321074	0.00112593	0.0149704	0.89	-0.149498	0.0125686	1.26E-32	-0.0075314	0.10014	0.9400485	0.4048815
ENSG00000176108	CHMP6	17	78974357	T	C	0.206759	-0.00721798	0.0187518	0.6700003	0.370425	0.0115803	1.63E-224	-0.0194857	0.050626	0.7003159	0.4838311
ENSG00000176124	DLEU1	13	50977253	A	G	0.134195	0.0603804	0.0218325	0.004700023	-0.391932	0.01204	1.92E-232	-0.154058	0.0559054	0.005856799	0.009037686
ENSG00000176125	UFSP1	7	100486842	G	A	0.462227	-0.00359999	0.0141928	0.6700003	-0.359273	0.00769173	0	0.0100202	0.0395048	0.7997696	0.9219533
ENSG00000176142	TMEM39A	3	119168012	A	G	0.365805	0.0226519	0.0151471	0.1800002	-0.0958927	0.0083793	2.52E-30	-0.236221	0.159302	0.1381132	0.994469
ENSG00000176148	TCP11L1	11	33094226	A	T	0.341948	-0.00199385	0.015152	0.91	0.463149	0.0080057	0	-0.00430499	0.0327153	0.895309	0.09922106
ENSG00000176153	GPX2	14	65407700	T	C	0.186879	0.015518	0.0177742	0.5199996	-0.129421	0.0100388	4.99E-38	-0.119904	0.137651	0.383717	0.2739655
ENSG00000176155	CCDC57	17	80115021	T	C	0.480119	8.53E-06	0.0144539	0.7099994	-0.340056	0.00829772	0	-2.51E-05	0.0425044	0.999529	0.7642189
ENSG00000176170	SPHK1	17	74378303	T	C	0.267396	-0.0132748	0.0159695	0.32	0.368766	0.00856184	0	-0.0359979	0.0433133	0.4059144	0.8520723
ENSG00000176171	BNIP3	10	133788506	C	T	0.2167	-0.00408056	0.0163616	0.7199992	-0.130363	0.0170986	2.46E-14	0.0313014	0.125575	0.8031556	0.9448478
ENSG00000176182	MYPOP	19	46399570	T	C	0.0904573	0.0350417	0.0218891	0.06800017	0.176555	0.0135983	1.52E-38	0.198475	0.124918	0.112096	0.9297715
ENSG00000176208	ATAD5	17	29190937	T	C	0.107356	-0.00233959	0.0229467	1	0.204084	0.0127082	4.93E-58	-0.0114638	0.11244	0.918792	0.03235555
ENSG00000176209	SMIM19	8	42402224	T	C	0.390656	-0.000965487	0.0145615	0.8700001	0.567655	0.0082348	0	-0.00170084	0.0256521	0.9471358	0.8026239
ENSG00000176222	ZNF404	19	44391026	G	A	0.513917	0.0138327	0.0142057	0.33	-0.185659	0.00861351	4.81E-103	-0.0745059	0.076593	0.3306772	0.6878941
ENSG00000176225	RTTN	18	67772105	G	T	0.0924453	-0.0115049	0.0266235	0.5500004	0.149523	0.0142189	7.31E-26	-0.0769441	0.178207	0.6659094	0.1366727
ENSG00000176261	ZBTB8OS	1	33091138	A	T	0.0298211	0.0796038	0.0401924	0.04300015	-0.285676	0.031809	2.68E-19	-0.27865	0.144073	0.05310128	0.1036048
ENSG00000176268	NA	13	41437886	C	T	0.28827	0.00929987	0.0152435	0.4199997	-0.535813	0.0196986	6.41E-163	-0.0173566	0.0284564	0.5419054	0.6107747
ENSG00000176273	SLC35G1	10	95684774	C	T	0.0149105	-0.115148	0.121448	0.2300001	-0.3242	0.0383342	2.74E-17	0.355176	0.376955	0.3460783	0.05002362
ENSG00000176293	ZNF135	19	58584142	A	G	0.299205	-0.0146234	0.0159103	0.3400001	0.21391	0.0132679	1.78E-58	-0.0683625	0.0744994	0.358815	0.05423362
ENSG00000176340	COX8A	11	63743047	G	A	0.439364	0.0147129	0.0142849	0.1900002	-0.164324	0.0149006	2.80E-28	-0.0895359	0.0873096	0.3051279	0.3749198
ENSG00000176358	TAC4	17	47920525	T	G	0.0516899	0.0146927	0.0283436	0.8200001	0.260483	0.0168032	3.36E-54	0.0564056	0.108873	0.6043965	0.03060962
ENSG00000176371	ZSCAN2	15	85157622	G	A	0.28827	0.00180358	0.015577	0.7600007	0.106973	0.00866054	4.76E-35	0.0168601	0.145622	0.9078271	0.04976035
ENSG00000176386	CDC26	9	116027992	G	A	0.336978	-0.020311	0.0148164	0.2399999	-0.497669	0.0129729	0	0.0408122	0.0297906	0.1706958	0.2490107
ENSG00000176390	CRLF3	17	29124100	G	T	0.109344	0.00826578	0.0220237	0.6200004	-0.453363	0.0131228	1.55E-261	-0.0182322	0.0485814	0.7074442	0.0641303
ENSG00000176393	RNPEP	1	201963387	G	A	0.390656	0.0111404	0.0145229	0.64	-0.885306	0.0063903	0	-0.0125837	0.0164046	0.4430338	0.2505588
ENSG00000176396	EID2	19	40029880	G	A	0.446322	0.0100213	0.0141992	0.3900004	-0.123985	0.00794825	7.39E-55	-0.0808269	0.114641	0.4807829	0.6321684
ENSG00000176401	EID2B	19	40022562	T	C	0.259443	-0.00703359	0.0160445	0.6200004	-0.142392	0.00908296	2.18E-55	0.0493961	0.112723	0.6612349	0.05579778
ENSG00000176410	DNAJC30	7	73097192	C	T	0.0328032	0.0184433	0.0361302	0.5	0.506016	0.0337254	6.91E-51	0.0364481	0.0714426	0.6099306	0.7130194
ENSG00000176422	SPRYD4	12	56863532	A	G	0.233598	0.000564263	0.0177407	0.9400001	0.674742	0.00915662	0	0.000836265	0.0262926	0.9746267	0.7053977
ENSG00000176428	VPS37D	7	73084298	A	G	0.404573	-0.00679456	0.0146635	0.4799997	-0.0753572	0.0124241	1.32E-09	0.0901647	0.195154	0.6440675	0.2376248
ENSG00000176438	SYNE3	14	95913002	T	C	0.259443	-0.0233668	0.0155263	0.14	0.132079	0.00998407	5.97E-40	-0.176915	0.118311	0.1348266	0.1271489
ENSG00000176444	CLK2	1	155240470	A	G	0.00795229	-0.160581	0.100338	0.1100001	-0.816147	0.114492	1.02E-12	0.196755	0.126001	0.1183987	0.8684621
ENSG00000176454	LPCAT4	15	34655292	A	G	0.327038	0.0139499	0.0152275	0.4299995	0.0945517	0.00849429	8.84E-29	0.147537	0.161594	0.3612361	0.5940668
ENSG00000176463	SLCO3A1	15	92556295	T	G	0.354871	0.00120878	0.0151098	0.9400001	0.483343	0.00802911	0	0.00250087	0.031261	0.9362375	0.8679689
ENSG00000176472	ZNF575	19	44034965	A	C	0.172962	0.00718657	0.0181502	0.64	0.336086	0.011073	2.38E-202	0.0213831	0.0540092	0.6921668	0.7772043
ENSG00000176473	WDR25	14	100919697	T	C	0.475149	0.00302441	0.0141938	0.7800007	0.552157	0.00749189	0	0.00547745	0.0257062	0.8312655	0.2022615
ENSG00000176476	SGF29	16	28584173	T	C	0.526839	0.0043215	0.0142513	0.7499995	0.56027	0.00734761	0	0.00771325	0.0254367	0.7617122	0.9868088
ENSG00000176485	PLAAT3	11	63362511	C	G	0.463221	-0.00224216	0.0143296	0.8200001	0.762778	0.00681146	0	-0.00293947	0.0187861	0.8756623	0.7488614
ENSG00000176490	DIRAS1	19	2717990	C	G	0.187873	-1.94E-05	0.0176297	0.7700005	0.305985	0.0110084	4.92E-170	-6.34E-05	0.0576162	0.9991221	0.736167
ENSG00000176531	PHLDB3	19	43994096	T	C	0.083499	-0.0102498	0.0249977	0.7400005	0.761923	0.0149682	0	-0.0134525	0.0328098	0.6817939	0.7483446
ENSG00000176533	GNG7	19	2606962	G	A	0.215706	0.0026021	0.0171175	0.6899999	-0.297761	0.0101228	3.56E-190	-0.0087389	0.0574882	0.8791775	0.9539334
ENSG00000176542	USF3	3	113391362	G	C	0.322068	0.00221704	0.0149717	0.84	-0.548252	0.018539	3.34E-192	-0.00404384	0.0273084	0.8822795	0.7094087
ENSG00000176593	ZNF606-AS1	19	58518014	C	T	0.296223	-0.0107652	0.0154332	0.5199996	0.37151	0.0112937	2.56E-237	-0.0289769	0.0415512	0.485566	0.2527777
ENSG00000176595	KBTBD11	8	1938573	G	A	0.201789	-0.0138074	0.0182448	0.6200004	0.360602	0.0103706	6.54E-265	-0.0382899	0.0506074	0.4492858	0.7313807
ENSG00000176597	B3GNT5	3	182993662	G	T	0.0616302	-0.0313955	0.0287328	0.2	0.432154	0.0162452	6.45E-156	-0.0726488	0.0665434	0.2749428	0.9467451
ENSG00000176619	LMNB2	19	2442315	T	A	0.248509	-0.00408846	0.0164902	0.83	0.148129	0.00997003	6.23E-50	-0.0276007	0.111339	0.8042132	0.09221613

ENSG00000176623	RMDN1	8	87503536	C	T	0.482107	0.00137641	0.0141891	0.8499999	-0.260408	0.00871786	4.74E-196	-0.00528559	0.0544883	0.922723	0.224568
ENSG00000176624	MEX3C	18	48722797	C	T	0.437376	-0.038979	0.014257	0.004	-0.146766	0.00794654	3.65E-76	0.265586	0.0981994	0.006839635	0.2433349
ENSG00000176641	RNF152	18	59518388	C	T	0.306163	-0.00357246	0.0150812	0.81	-0.0576808	0.00873792	4.08E-11	0.061935	0.261628	0.8128668	0.972438
ENSG00000176658	MYO1D	17	31011867	T	C	0.197813	-0.000533397	0.0181715	0.95	0.326302	0.0100709	2.72E-230	-0.00163468	0.0556893	0.9765827	0.2563459
ENSG00000176659	LINC02910	20	58639494	T	C	0.429423	-0.002991	0.0144823	0.6100002	0.293015	0.00785505	1.48E-304	-0.0102077	0.0494258	0.836381	0.7728525
ENSG00000176681	LRRC37A	17	44392629	G	T	0.227634	0.00628136	0.0171064	0.5099998	0.825571	0.0194205	0	0.0076085	0.0207215	0.7134849	0.8263802
ENSG00000176700	NA	15	85180188	G	C	0.469185	0.0179301	0.0142074	0.08799946	-0.271369	0.00867055	5.00E-215	-0.0660727	0.052397	0.2073086	0.1802189
ENSG00000176715	ACSF3	16	89188518	A	G	0.360835	0.0123986	0.0148701	0.3900004	-0.166143	0.0104348	4.46E-57	-0.0746263	0.0896247	0.4050404	0.5090071
ENSG00000176720	BOK	2	242505841	C	T	0.327038	0.0227024	0.0157643	0.1900002	-0.290232	0.0085498	1.39E-252	-0.0782214	0.054365	0.1502014	0.04013955
ENSG00000176731	RBIS	8	86129480	A	G	0.272366	-0.0286598	0.0160827	0.05800027	-0.302487	0.00912217	4.12E-241	0.0947471	0.0532449	0.07516456	0.07154683
ENSG00000176749	CDK5R1	17	30815955	G	C	0.482107	-0.0195257	0.0142095	0.28	0.63184	0.00716572	0	-0.0309029	0.0224918	0.1694531	0.6441869
ENSG00000176761	NA	19	44974651	A	G	0.178926	0.00372001	0.0175199	0.8	-0.0765498	0.013994	4.50E-08	-0.048596	0.229042	0.8319738	0.7840923
ENSG00000176783	RUFY1	5	179007293	G	A	0.365805	-0.00391522	0.0145772	0.7499995	-0.666184	0.0071895	0	0.00587708	0.0218817	0.7882498	0.6486592
ENSG00000176788	BASP1	5	17247306	T	C	0.505964	7.51E-05	0.0142649	0.9599999	0.259188	0.00783921	1.01E-239	0.000289746	0.0550369	0.9957995	0.9938014
ENSG00000176824	NA	2	145220162	C	T	0.0775348	-0.000341967	0.0260877	0.81	0.228827	0.0353411	9.49E-11	-0.00149444	0.114007	0.9895414	0.9858455
ENSG00000176834	VSIG10	12	118537614	A	G	0.456262	0.0111656	0.0143712	0.4799997	-0.396627	0.00846189	0	-0.0281514	0.0362386	0.4372555	0.4063476
ENSG00000176845	METRNL	17	81045215	A	G	0.353877	0.00740238	0.0144337	0.7400005	0.170108	0.010722	1.10E-56	0.0435157	0.0848945	0.6082408	0.00351636
ENSG00000176853	FAM91A1	8	124804194	T	G	0.191849	-0.0282705	0.0190169	0.1199999	0.119668	0.0102749	2.39E-31	-0.236242	0.160204	0.1403108	0.3467134
ENSG00000176871	WSB2	12	118485473	A	G	0.456262	0.0111656	0.0143712	0.4799997	-0.350171	0.00860064	0	-0.0318861	0.0410479	0.437276	0.3574509
ENSG00000176890	TYMS	18	665591	G	C	0.450298	0.00199045	0.0142371	0.91	-0.132001	0.00797397	1.50E-61	-0.015079	0.10786	0.8888162	0.1168253
ENSG00000176903	PNMA1	14	74179811	A	G	0.358847	-0.0297182	0.0154482	0.0530005	0.159021	0.00856933	7.16E-77	-0.186882	0.0976663	0.0556869	0.08688804
ENSG00000176909	MAMSTR	19	49219488	C	T	0.138171	-0.0381339	0.0217702	0.02999991	-0.0688408	0.0115369	2.42E-09	0.553943	0.329584	0.0928148	0.1203396
ENSG00000176915	ANKLE2	12	133320364	A	G	0.249503	0.00521035	0.0160961	0.7600007	0.318713	0.0108552	1.75E-189	0.0163481	0.0505065	0.7461781	0.115321
ENSG00000176919	C8G	9	139840562	G	C	0.517893	-0.0270315	0.0142317	0.08400014	-0.187783	0.0117407	1.40E-57	0.143951	0.0763207	0.05927698	0.4340138
ENSG00000176927	EFCAB5	17	28345844	A	G	0.513917	0.0251516	0.0141851	0.02699977	-0.143943	0.0079086	5.09E-74	-0.174733	0.0990133	0.07760653	0.7198795
ENSG00000176928	GCNT4	5	74325006	A	G	0.0536779	-0.0387317	0.034349	0.2200002	-0.319415	0.0210518	5.35E-52	0.121258	0.107834	0.2608043	0.2542467
ENSG00000176945	MUC20	3	195457873	A	G	0.153082	-0.0151284	0.0191513	0.4700002	0.707135	0.0103227	0	-0.0213939	0.0270848	0.4295928	0.4585159
ENSG00000176946	THAP4	2	242550342	A	C	0.311133	0.0226913	0.0157815	0.1900002	0.111127	0.0098047	8.90E-30	0.204193	0.143152	0.1537502	0.1411944
ENSG00000176953	NFATC2IP	16	28970273	A	G	0.534791	-0.0161672	0.014201	0.1900002	-0.0868409	0.00887272	1.28E-22	0.18617	0.164632	0.2581264	0.4349979
ENSG00000176973	FAM89B	11	65340744	T	C	0.0477137	-0.0733354	0.0311908	0.016	-0.471261	0.0173327	8.74E-163	0.155615	0.0664328	0.01915787	0.5517373
ENSG00000176974	SHMT1	17	18249021	T	C	0.383698	-0.00659222	0.0147344	0.5500004	-0.48831	0.00882297	0	0.0135001	0.0301753	0.6545944	0.6821101
ENSG00000176978	DPP7	9	140007311	A	G	0.357853	0.00535313	0.0147694	0.64	-0.260249	0.0100482	6.66E-148	-0.0205693	0.0567567	0.7170443	0.08459027
ENSG00000176994	SMCR8	17	18222570	T	C	0.308151	0.00791288	0.0152419	0.4500005	0.102451	0.00860789	1.16E-32	0.0772357	0.148914	0.6039979	0.002197044
ENSG00000176998	NA	6	29759833	T	C	0.206759	0.0235928	0.0163574	0.05899973	-0.524574	0.0219529	3.42E-126	-0.0449752	0.031239	0.149949	0.9636602
ENSG00000177000	MTHFR	1	11856378	T	C	0.280318	0.00190528	0.0160441	0.98	0.730039	0.00778287	0	0.00260983	0.0219771	0.9054714	0.6730996
ENSG00000177025	C19orf18	19	58477853	A	G	0.0815109	0.0161762	0.0246143	0.4100001	0.164745	0.0151965	2.20E-27	0.0981891	0.149682	0.5118351	0.7746809
ENSG00000177030	DEAF1	11	675474	A	G	0.298211	-0.0190033	0.0151418	0.29	0.280075	0.00856364	1.32E-234	-0.0678506	0.0541031	0.2098058	0.7607331
ENSG00000177034	MTX3	5	79281333	T	C	0.115308	0.00727672	0.0213203	0.6200004	-0.229985	0.0210856	1.06E-27	-0.03164	0.0927484	0.7329999	0.7575164
ENSG00000177042	TMEM80	11	700322	T	C	0.492048	0.0194307	0.0142409	0.2	-0.595756	0.00739326	0	-0.0326152	0.0239073	0.172494	0.416289
ENSG00000177045	SIX5	19	46270092	T	C	0.467197	0.0195771	0.0142119	0.1800002	0.144507	0.00862809	5.82E-63	0.135475	0.0986798	0.16979	0.6275229
ENSG00000177051	FBXO46	19	46224024	C	T	0.170974	-0.00729802	0.0194668	0.4899999	0.0835966	0.0104687	1.40E-15	-0.0873004	0.233122	0.7080448	0.9249263
ENSG00000177054	ZDHHC13	11	19168307	G	A	0.154076	5.11E-05	0.0204758	0.9299999	0.351118	0.011309	1.23E-211	0.000145443	0.058316	0.99801	0.170052
ENSG00000177058	SLC38A9	5	54995347	A	G	0.0159046	0.00836143	0.0526801	0.9699999	-0.474283	0.0283764	1.04E-62	-0.0176296	0.111078	0.8738945	0.8759216
ENSG00000177076	ACER2	9	19430471	G	A	0.00894632	-0.0368169	0.0600672	0.5500004	0.427058	0.0574327	1.04E-13	-0.0862106	0.141131	0.5412944	0.1616473
ENSG00000177082	WDR73	15	85191786	C	G	0.182903	0.00661785	0.0186093	0.7499995	0.184927	0.0114146	4.96E-59	0.0357862	0.100654	0.7221889	0.8380614
ENSG00000177084	POLE	12	133306867	G	T	0.129225	-0.0116044	0.0207125	0.7099994	0.103878	0.0124158	5.93E-17	-0.111712	0.199839	0.5761557	0.9000315
ENSG00000177096	PHETA2	22	42472850	T	A	0.33499	0.0252465	0.015149	0.08199927	-0.276047	0.00918278	1.55E-198	-0.0914572	0.0549626	0.09611431	0.09108879
ENSG00000177098	SCN4B	11	118013847	G	T	0.403579	0.00863383	0.0144233	0.6600001	-0.0550726	0.0081276	1.24E-11	-0.156772	0.262916	0.5509877	0.189799
ENSG00000177103	DSCAML1	11	117493364	T	C	0.0725646	0.0216372	0.0248747	0.1900002	0.161437	0.0152527	3.53E-26	0.134028	0.154602	0.3859831	0.135885
ENSG00000177105	RHOG	11	3855210	G	A	0.101392	0.0484701	0.024197	0.0649995	0.199084	0.0138688	9.94E-47	0.243465	0.122719	0.04726456	0.7840638

ENSG00000177106	EPS8L2	11	711082	C	T	0.550696	-0.0173358	0.0142212	0.2099999	-0.198694	0.00913806	7.94E-105	0.0872486	0.0716857	0.2235662	0.02538881
ENSG00000177112	IRAG1-AS1	11	10592149	T	G	0.116302	-0.0156193	0.022542	0.4600002	-0.472282	0.0143343	4.56E-238	0.033072	0.0477405	0.4884699	0.9892663
ENSG00000177119	ANO6	12	45721978	T	G	0.0178926	0.0105495	0.0490658	0.9599999	-0.176924	0.0307673	8.90E-09	-0.0596272	0.27752	0.8298788	0.9767638
ENSG00000177125	ZBTB34	9	129635550	T	A	0.456262	0.0244435	0.0142338	0.04799986	0.101875	0.0080198	5.69E-37	0.239935	0.140989	0.08879188	0.2939372
ENSG00000177150	FAM210A	18	13695004	A	G	0.15507	0.00468419	0.019626	0.7899998	0.168319	0.0125154	3.12E-41	0.0278292	0.116618	0.8113885	0.2368261
ENSG00000177156	TALDO1	11	756176	A	G	0.0775348	0.0141074	0.0239188	0.5199996	0.205989	0.0163902	3.17E-36	0.0684861	0.116245	0.555757	0.7158926
ENSG00000177169	ULK1	12	132393454	G	C	0.114314	0.0134418	0.024846	0.6499995	0.139711	0.0131958	3.40E-26	0.0962112	0.17807	0.5889904	0.9762715
ENSG00000177173	NA	1	117076136	T	A	0.121272	-0.0406519	0.0234114	0.1100001	-0.255788	0.0309371	1.36E-16	0.158928	0.0935233	0.08925461	0.7211029
ENSG00000177191	B3GNT8	19	41932949	T	C	0.430417	-0.00130363	0.0142725	0.89	-0.0879512	0.00804368	7.91E-28	0.0148222	0.162283	0.9272261	0.6280251
ENSG00000177192	PUS1	12	132421075	G	A	0.00397614	0.0323154	0.100849	0.7700005	0.544774	0.0604808	2.11E-19	0.0593189	0.185238	0.7487933	0.9976966
ENSG00000177200	CHD9	16	53226003	G	C	0.343936	0.0062106	0.0149488	0.9199999	-0.140932	0.00830083	1.19E-64	-0.0440682	0.106103	0.6778976	0.5032961
ENSG00000177236	NA	11	780436	G	A	0.511928	-0.0222553	0.0142261	0.08199927	-0.904498	0.0148039	0	0.0246051	0.0157333	0.1178442	0.02629576
ENSG00000177239	MAN1B1	9	139992507	A	G	0.357853	0.00535313	0.0147694	0.64	-0.443417	0.00973464	0	-0.0120724	0.0333092	0.7170263	0.09299123
ENSG00000177272	KCNA3	1	111215982	T	C	0.559642	0.0117339	0.0142609	0.6999999	0.106853	0.00792387	1.92E-41	0.109813	0.133711	0.4114897	0.02764945
ENSG00000177283	FZD8	10	35928769	C	A	0.222664	0.00500414	0.0171513	0.5999997	0.0719165	0.0112559	1.67E-10	0.0695827	0.238738	0.770699	0.8382025
ENSG00000177294	FBXO39	17	6689981	C	A	0.412525	0.00891765	0.0145443	0.56	0.053767	0.00831739	1.02E-10	0.165857	0.27172	0.5415984	0.1282217
ENSG00000177301	KCNA2	1	111155149	G	A	0.255467	-0.0255657	0.0163008	0.1499999	-0.0532477	0.00910248	4.92E-09	0.480128	0.316943	0.1298053	0.557278
ENSG00000177302	TOP3A	17	18196531	A	G	0.415507	0.0060902	0.0143206	0.4500005	-0.193085	0.00797293	1.45E-129	-0.0315415	0.0741787	0.6706837	0.06851002
ENSG00000177303	CASKIN2	17	73504003	G	A	0.142147	-0.0138521	0.0209472	0.32	0.129211	0.0122675	6.10E-26	-0.107205	0.162435	0.5092622	0.473862
ENSG00000177311	ZBTB38	3	141105844	G	A	0.427435	0.00315427	0.0142628	0.89	0.240486	0.00865482	6.33E-170	0.0131162	0.05931	0.8249783	0.2802132
ENSG00000177335	LY6S-AS1	8	144130992	C	T	0.362823	-0.00405593	0.0147784	0.84	-0.421553	0.00838609	0	0.00962139	0.0350575	0.7837421	0.2027064
ENSG00000177337	NA	18	3596040	T	C	0.15507	-0.0110161	0.021296	0.5099998	-0.858951	0.014463	0	0.0128251	0.024794	0.604971	0.1570702
ENSG00000177352	CCDC71	3	49201861	C	T	0.323062	0.00371478	0.015058	0.8700001	0.129336	0.00915331	2.48E-45	0.0287219	0.116443	0.805171	0.03544129
ENSG00000177359	OVOS2	12	31311837	A	T	0.474155	0.00140513	0.0145406	0.9199999	0.238001	0.00977633	6.61E-131	0.00590389	0.0610952	0.9230169	0.5436398
ENSG00000177369	FLJ40194	17	47330812	C	G	0.235586	0.00724842	0.0170301	0.6499995	0.0698013	0.011627	1.93E-09	0.103844	0.244592	0.6711586	0.7822368
ENSG00000177370	TIMM22	17	902882	C	T	0.248509	0.012634	0.0170048	0.4500005	0.428188	0.0102488	0	0.0295057	0.0397197	0.457573	0.6986235
ENSG00000177374	HIC1	17	1960214	C	T	0.403579	0.00404045	0.0145043	0.99	0.0643402	0.00814763	2.86E-15	0.0627982	0.225571	0.7807084	0.4928221
ENSG00000177380	PPFIA3	19	49638464	G	T	0.240557	-0.00119823	0.0162622	0.9599999	0.0525376	0.00942547	2.49E-08	-0.0228071	0.309562	0.9412686	0.808572
ENSG00000177383	MAGEF1	3	184428996	T	C	0.236581	0.00251245	0.0164457	0.8499999	0.347253	0.0088641	0	0.00723522	0.0473598	0.8785786	0.259688
ENSG00000177398	UMODL1	21	43523086	A	G	0.116302	-0.00325287	0.0253177	0.7800007	0.162428	0.0150391	3.43E-27	-0.0200265	0.155881	0.8977747	0.7526039
ENSG00000177406	NINJ2-AS1	12	756464	A	G	0.130219	-0.0162033	0.0191453	0.4799997	0.940981	0.0127248	0	-0.0172196	0.0203474	0.3973979	0.411943
ENSG00000177409	SAMD9L	7	92768525	A	G	0.0427435	0.0612749	0.0310987	0.05	-1.24047	0.0172094	0	-0.0493965	0.0250795	0.04888415	0.7401805
ENSG00000177410	ZFAS1	20	47900256	C	G	0.163022	0.00988238	0.019221	0.59	0.358045	0.0131663	7.65E-163	0.0276009	0.0536928	0.6072149	0.8190636
ENSG00000177426	TGIF1	18	3435791	A	G	0.263419	0.016186	0.0165263	0.4199997	-0.400914	0.00925623	0	-0.0403727	0.0412321	0.3275018	0.7842382
ENSG00000177427	MIEF2	17	18166857	A	G	0.308151	0.00763125	0.0152454	0.4600002	-0.0755261	0.00991599	2.60E-14	-0.101041	0.202291	0.6174389	0.01178448
ENSG00000177432	NAP1L5	4	89618226	C	G	0.0576541	-0.0500871	0.0400635	0.2700001	-0.307052	0.0390125	3.53E-15	0.163123	0.132114	0.2169373	0.9071109
ENSG00000177453	NIM1K	5	43236562	T	G	0.124254	0.0134581	0.021725	0.5199996	0.16769	0.0140895	1.16E-32	0.0802556	0.12973	0.536155	0.2166261
ENSG00000177463	NR2C2	3	15042099	G	A	0.0656064	-0.0183181	0.0300259	0.4199997	0.191291	0.0165102	4.84E-31	-0.0957604	0.157182	0.5423702	0.6411252
ENSG00000177465	ACOT4	14	74060805	A	G	0.392644	-0.00643453	0.0147322	0.64	0.561176	0.0113419	0	-0.0114662	0.0262534	0.6622931	0.2885304
ENSG00000177469	CAVIN1	17	40565002	A	G	0.0487078	0.00150431	0.0268781	0.5700002	-0.173181	0.0175857	7.01E-23	-0.00868637	0.155205	0.9553681	0.6664413
ENSG00000177479	ARIH2	3	48990034	T	G	0.33499	-0.00542225	0.0149458	0.4299995	-0.163253	0.00833692	2.20E-85	0.0332137	0.0915654	0.7168051	0.02308174
ENSG00000177483	RBM44	2	238729241	A	T	0.178926	-0.00504311	0.0197991	0.7899998	0.254209	0.0105759	1.15E-127	-0.0198384	0.0778895	0.798955	0.6175835
ENSG00000177494	ZBED2	3	111313018	G	A	0.141153	0.0240796	0.0200635	0.2599998	0.124505	0.0125747	4.11E-23	0.193403	0.162326	0.2334776	0.2827614
ENSG00000177508	IRX3	16	54318945	A	G	0.131213	0.0379609	0.0211616	0.09699961	0.81349	0.0116062	0	0.0466643	0.0260219	0.07292968	0.2472769
ENSG00000177519	RPRM	2	154334587	A	G	0.237575	-0.021643	0.0175149	0.14	0.359597	0.00959154	1.29E-307	-0.0601869	0.0487335	0.216823	0.1577311
ENSG00000177542	SLC25A22	11	794395	G	C	0.109344	0.00741995	0.0219158	0.6899999	-0.121535	0.0139145	2.45E-18	-0.0610522	0.180461	0.735128	0.274768
ENSG00000177556	ATOX1	5	151136985	T	C	0.422465	0.0142027	0.0143809	0.2399999	-0.292945	0.00788697	5.71E-302	-0.0484825	0.0491081	0.3235154	0.5519963
ENSG00000177570	SAMD12	8	119417966	C	T	0.0795229	0.01704	0.024433	0.32	0.682277	0.0132748	0	0.0249752	0.0358143	0.4855818	0.09911041
ENSG00000177575	CD163	12	7639949	T	A	0.0477137	0.0340873	0.0315803	0.3400001	0.181224	0.0156114	3.74E-31	0.188095	0.175013	0.2824872	0.5204087
ENSG00000177576	C18orf32	18	47010825	T	G	0.50994	-0.0224422	0.0141894	0.09499921	-0.0907454	0.00797089	4.99E-30	0.247309	0.157867	0.1172148	0.5296815

ENSG00000177590	GIMAP3P	7	150444398	A	G	0.432406	-0.0102114	0.0143926	0.3800004	0.397916	0.0184624	4.98E-103	-0.0256622	0.0361895	0.478259	0.4806605
ENSG00000177595	PIDD1	11	804466	T	G	0.360835	-0.0211809	0.0152158	0.1100001	-0.302936	0.011405	1.88E-155	0.0699186	0.0502966	0.1644908	0.08632276
ENSG00000177599	ZNF491	19	11914459	G	A	0.156064	3.91E-05	0.0198003	0.6600001	-0.108346	0.0105282	7.74E-25	-0.000361214	0.182751	0.998423	0.345274
ENSG00000177600	RPLP2	11	811263	G	C	0.357853	-0.0207783	0.0152282	0.1199999	0.150799	0.0127525	2.90E-32	-0.137788	0.101654	0.1752684	0.167925
ENSG00000177606	JUN	1	59248125	C	A	0.421471	0.00849851	0.0145537	0.4899999	-0.228224	0.00804141	3.46E-177	-0.0372376	0.0637828	0.5593428	0.4988851
ENSG00000177613	CSTF2T	10	53457301	G	A	0.15507	0.0164659	0.0206402	0.4899999	-0.213846	0.0115581	2.00E-76	-0.0769989	0.0966087	0.4254405	0.8932496
ENSG00000177614	PGBD5	1	230509433	T	C	0.16998	0.00614842	0.0188504	0.6600001	0.150795	0.0104287	2.18E-47	0.0407734	0.125039	0.7443591	0.3106956
ENSG00000177628	GBA1	1	155209446	A	T	0.33996	-0.0142385	0.0155442	0.3800004	0.2597	0.012817	2.77E-91	-0.0548268	0.0599157	0.3601569	0.3161928
ENSG00000177646	ACAD9	3	128616674	A	G	0.0178926	0.0144901	0.0751239	0.7099994	1.11661	0.0284534	0	0.0129768	0.0672792	0.8470527	0.2724027
ENSG00000177663	IL17RA	22	17581213	C	G	0.185885	0.00597293	0.0170414	0.7400005	0.246514	0.00950303	2.32E-148	0.0242296	0.0691358	0.725991	0.5534322
ENSG00000177666	PNPLA2	11	822237	A	G	0.307157	-0.0412301	0.0160291	0.004499974	0.234252	0.0102957	1.36E-114	-0.176008	0.0688627	0.0105907	0.3211716
ENSG00000177669	MBOAT4	8	29995771	A	G	0.120278	-0.0308354	0.0223199	0.17	0.24272	0.0151507	9.21E-58	-0.127041	0.0922986	0.1686941	0.0122779
ENSG00000177674	AGTRAP	1	11805500	T	A	0.206759	-0.0243554	0.0188813	0.1499999	0.27878	0.0102972	2.03E-161	-0.0873642	0.0678051	0.1975858	0.824063
ENSG00000177675	CD163L1	12	7565887	C	T	0.40159	-0.007799	0.0143892	0.6200004	0.148209	0.00809727	7.75E-75	-0.0526215	0.0971296	0.5879795	0.7661407
ENSG00000177683	THAP5	7	108202590	A	G	0.489066	-0.0120332	0.014249	0.25	-0.121079	0.00794806	2.11E-52	0.099383	0.117864	0.3991168	0.3572025
ENSG00000177685	CRACR2B	11	829067	T	C	0.184891	-0.0107068	0.0171838	0.6200004	0.374152	0.0111029	6.08E-249	-0.0286162	0.0459352	0.5333051	0.001909408
ENSG00000177697	CD151	11	836337	G	A	0.285288	-0.00987958	0.0163854	0.4500005	-0.932493	0.00895618	0	0.0105948	0.0175719	0.5465486	0.1719555
ENSG00000177700	POLR2L	11	839950	A	C	0.126243	-0.0177396	0.0201247	0.3900004	-0.185518	0.0132405	1.33E-44	0.0956222	0.108693	0.3789976	0.4294641
ENSG00000177706	FAM20C	7	246840	G	A	0.478131	-0.00677834	0.0143472	0.6700003	-0.259775	0.00857558	1.44E-201	0.0260931	0.0552361	0.6366464	0.1612131
ENSG00000177707	NECTIN3	3	110891664	C	T	0.417495	-0.00102466	0.0146502	0.89	-0.08227	0.00820796	1.21E-23	0.0124548	0.178079	0.9442414	0.310625
ENSG00000177710	SLC35G5	8	11189057	T	G	0.530815	-0.00902416	0.0141987	0.6700003	0.141597	0.00963491	6.82E-49	-0.0637315	0.100369	0.5254487	0.4884781
ENSG00000177721	ANXA2R	5	43041303	C	T	0.191849	0.0146426	0.0185741	0.4299995	0.340547	0.0118257	2.33E-182	0.0429973	0.0545624	0.4306739	0.8391729
ENSG00000177728	TMEM94	17	73466705	C	G	0.295229	-0.00832428	0.0161948	0.5099998	0.148886	0.00934026	3.33E-57	-0.0559105	0.10883	0.6074321	0.6755598
ENSG00000177731	FLII	17	18155190	G	A	0.348907	0.00115361	0.0149101	0.98	0.0850766	0.00836742	2.77E-24	0.0135597	0.17526	0.9383302	0.2705832
ENSG00000177732	SOX12	20	308536	G	T	0.0298211	0.0134355	0.040938	0.7600007	0.225034	0.0237458	2.62E-21	0.0597042	0.182028	0.7429156	0.0866147
ENSG00000177733	HNRNPA0	5	137088557	C	G	0.183897	0.00542337	0.0189278	0.5	-0.0991325	0.0120705	2.16E-16	-0.0547083	0.19105	0.7746061	0.730071
ENSG00000177738	ANXA2R-OT1	5	43041018	T	C	0.281312	-0.0382133	0.0152405	0.006800017	-0.402018	0.010515	0	0.0950537	0.0379914	0.01235018	0.6334959
ENSG00000177807	KCNJ10	1	160023647	A	G	0.422465	-0.00327811	0.0143397	0.8800001	0.146713	0.00800251	4.48E-75	-0.0223436	0.0977472	0.8191907	0.7604457
ENSG00000177830	CHID1	11	891207	C	T	0.367793	-0.0108725	0.0145423	0.2700001	0.0596565	0.00932599	1.59E-10	-0.182252	0.245427	0.457729	0.7600158
ENSG00000177853	ZNF518A	10	97927258	T	C	0.00695825	0.0115332	0.0710751	0.99	-0.339676	0.0522788	8.17E-11	-0.0339535	0.209309	0.8711348	0.8652429
ENSG00000177868	SVBP	1	43277838	T	C	0.296223	-0.0241032	0.0158102	0.09200046	0.97636	0.00677149	0	-0.0246868	0.0161939	0.1273964	0.06252319
ENSG00000177873	ZNF619	3	40525165	A	G	0.317097	0.0132194	0.0152514	0.5400003	0.103761	0.00895141	4.55E-31	0.127402	0.147396	0.3873939	0.3459422
ENSG00000177875	CCDC184	12	48578537	T	C	0.422465	0.0059176	0.0143842	0.5400003	-0.0604426	0.0104161	6.52E-09	-0.0979045	0.238579	0.6815376	0.4112899
ENSG00000177879	AP3S1	5	115213478	C	T	0.0258449	-0.0527976	0.0486836	0.3599996	0.428797	0.047828	3.09E-19	-0.12313	0.114363	0.2816339	0.6120438
ENSG00000177885	GRB2	17	73357973	T	C	0.261431	-0.0181919	0.016996	0.1299999	-0.185515	0.0092058	2.58E-90	0.0980615	0.0917442	0.2851341	0.473143
ENSG00000177888	ZBTB41	1	197148622	T	C	0.0884692	0.0122552	0.0237728	0.7800007	-0.123122	0.0208335	3.43E-09	-0.0995371	0.193817	0.6075573	0.4831856
ENSG00000177889	UBE2N	12	93817743	C	T	0.347913	0.000929456	0.0152422	0.6999999	-0.0682647	0.00845844	7.00E-16	-0.0136155	0.223287	0.9513772	0.8822961
ENSG00000177917	ARL6IP6	2	153596087	C	T	0.33996	-0.0326046	0.0151661	0.0179999	-0.113038	0.00925674	2.70E-34	0.28844	0.136232	0.03423668	0.01387694
ENSG00000177932	ZNF354C	5	178498977	T	C	0.304175	0.00108409	0.0152997	0.7800007	0.0877849	0.00865541	3.59E-24	0.0123494	0.17429	0.943513	0.4690527
ENSG00000177943	MAMDC4	9	139750322	G	A	0.435388	0.0299979	0.0143156	0.05499966	0.0784582	0.00928609	2.94E-17	0.382343	0.187989	0.04196665	0.809913
ENSG00000177946	CENPBD1P	16	90037574	C	A	0.183897	0.0150443	0.017428	0.58	0.315295	0.0108827	1.48E-184	0.047715	0.0552997	0.3882238	0.1009923
ENSG00000177947	CIMAP1A	11	198499	A	G	0.082505	0.00995915	0.0271845	0.6100002	-0.145233	0.0192811	4.98E-14	-0.0685737	0.1874	0.7144237	0.6640123
ENSG00000177951	BET1L	11	187606	C	T	0.43837	-0.00759163	0.0143608	0.6899999	0.385595	0.00865334	0	-0.0196881	0.0372458	0.5970841	0.4200882
ENSG00000177963	RIC8A	11	211312	G	C	0.272366	-0.00203096	0.016469	0.81	0.251787	0.00902337	2.39E-171	-0.00806618	0.0654091	0.9018545	0.7599726
ENSG00000177971	IMP3	15	75936236	T	C	0.270378	-0.00390243	0.0164052	0.9599999	-0.153942	0.00930716	1.88E-61	0.02535	0.106578	0.8119951	0.1967228
ENSG00000177981	ASB8	12	48558283	T	C	0.224652	0.0169416	0.0167863	0.32	-0.195334	0.00906304	4.98E-103	-0.0867315	0.0860306	0.313384	0.7398626
ENSG00000177989	CIMAP1B	22	50969574	T	G	0.134195	0.0260238	0.0191045	0.1499999	-0.405302	0.012154	8.02E-244	-0.0642084	0.0471758	0.173499	0.7852174
ENSG00000177990	DPY19L2	12	64007706	C	A	0.196819	0.00287385	0.017816	0.8499999	-0.400924	0.0140457	3.31E-179	-0.00716807	0.0444381	0.8718534	0.7081434
ENSG00000178026	LRRC75B	22	24985381	G	T	0.366799	-0.00507383	0.0149375	0.7099994	-0.19404	0.0102517	6.76E-80	0.0261483	0.0769938	0.7341458	0.8284495
ENSG00000178028	DMAP1	1	44682740	C	G	0.407555	-0.024063	0.0146116	0.1199999	-0.114709	0.00819692	1.69E-44	0.209774	0.128258	0.1019325	0.9712651

ENSG00000178035	IMPDH2	3	49064299	T	C	0.114314	-0.0131154	0.0228892	0.3900004	-0.0982367	0.0129785	3.76E-14	0.133508	0.233667	0.5677554	0.3020851
ENSG00000178038	ALS2CL	3	46722840	G	A	0.0666004	-0.0240515	0.0307698	0.3700002	0.204612	0.015769	1.68E-38	-0.117547	0.150654	0.4352468	0.6492499
ENSG00000178053	MLF1	3	158306996	G	T	0.158052	0.00190835	0.0192054	0.6999999	0.26809	0.0155778	2.24E-66	0.00711832	0.0716391	0.9208496	0.01407562
ENSG00000178057	NDUFAF3	3	49059410	A	C	0.219682	0.0020546	0.0167758	0.9	-0.144347	0.0106752	1.16E-41	-0.0142337	0.116223	0.9025276	0.007877076
ENSG00000178075	GRAMD1C	3	113606525	A	G	0.235586	0.0200249	0.0161916	0.08400014	-0.39831	0.00958776	0	-0.0502747	0.0406688	0.2163848	0.4033731
ENSG00000178078	STAP2	19	4333411	T	C	0.300199	0.0107373	0.0151794	0.3800004	0.0941452	0.00955828	6.88E-23	0.11405	0.161649	0.4804725	0.7478672
ENSG00000178093	TSSK6	19	19625113	G	T	0.16998	-0.0443359	0.0190543	0.02100003	0.196694	0.011429	2.23E-66	-0.225406	0.0977543	0.02111961	0.2029782
ENSG00000178096	BOLA1	1	149865895	A	G	0.028827	-0.0657199	0.042229	0.16	0.376555	0.044426	2.33E-17	-0.174529	0.11402	0.1258469	0.05444099
ENSG00000178104	PDE4DIP	1	144956171	T	C	0.377734	0.00039855	0.0147069	0.9599999	0.0971226	0.0103724	7.71E-21	0.00410358	0.151427	0.9783804	0.4524131
ENSG00000178105	DDX10	11	108673704	A	G	0.162028	-0.0185982	0.021635	0.3800004	0.226524	0.0112137	9.67E-91	-0.0821025	0.095595	0.3904191	0.6800378
ENSG00000178127	NDUFV2	18	9118485	G	A	0.172962	0.030581	0.0184888	0.1100001	0.21595	0.022116	1.60E-22	0.141612	0.0868359	0.1029332	0.2054433
ENSG00000178149	DALRD3	3	49056323	A	C	0.220676	0.00140836	0.0167636	0.8600001	-0.167412	0.010654	1.22E-55	-0.00841254	0.100135	0.9330471	0.01050944
ENSG00000178162	NA	2	131181632	A	G	0.0974155	0.0116099	0.022948	0.7199992	0.265784	0.0365068	3.33E-13	0.0436817	0.086549	0.6137661	0.1215407
ENSG00000178163	ZNF518B	4	10450266	C	G	0.34493	-0.00894967	0.0150919	0.6700003	-0.237712	0.00909124	1.06E-150	0.0376492	0.0635044	0.5532755	0.2841278
ENSG00000178184	PARD6G	18	77960272	T	C	0.428429	0.0101968	0.0146116	0.81	0.0974295	0.0080699	1.46E-33	0.104658	0.150221	0.4859946	0.727417
ENSG00000178188	SH2B1	16	28871723	G	A	0.329026	0.000174953	0.0144807	0.9299999	-0.0590017	0.00819105	5.88E-13	-0.00296522	0.245429	0.9903604	0.8260074
ENSG00000178199	ZC3H12D	6	149787495	G	A	0.177932	0.006697	0.0195828	0.99	0.227861	0.0127928	5.74E-71	0.0293908	0.0859578	0.7324105	0.1682907
ENSG00000178201	VN1R1	19	57967198	G	A	0.247515	0.0219368	0.0162429	0.2099999	-0.148728	0.00918538	5.76E-59	-0.147496	0.109591	0.1783433	0.7004846
ENSG00000178202	POGLUT3	11	108355995	T	C	0.149105	-0.0026289	0.0206142	0.9299999	-0.732957	0.0101318	0	0.00358671	0.0281248	0.8985222	0.6021433
ENSG00000178209	PLEC	8	145020111	T	G	0.375746	0.0039983	0.0148103	0.8600001	-0.303602	0.0095223	4.55E-223	-0.0131695	0.0487837	0.7871927	0.005744751
ENSG00000178222	RNF212	4	1078694	A	G	0.300199	0.0229542	0.0153461	0.1299999	-0.161895	0.00853599	3.25E-80	-0.141784	0.0950846	0.1359254	0.3391332
ENSG00000178226	PRSS36	16	31155830	A	G	0.289264	0.019371	0.0156837	0.14	0.13901	0.00947438	9.71E-49	0.13935	0.113223	0.218416	0.3940401
ENSG00000178229	ZNF543	19	57837010	T	C	0.526839	-0.0163783	0.0143288	0.2399999	-0.247193	0.00836501	6.41E-192	0.0662572	0.0580094	0.2533794	0.1113616
ENSG00000178234	GALNT11	7	151771092	T	C	0.327038	0.0188707	0.0155729	0.16	0.119231	0.00867661	5.72E-43	0.158271	0.131119	0.2274009	0.06918248
ENSG00000178252	WDR6	3	49048940	G	A	0.335984	-0.00498936	0.0149473	0.4500005	0.643398	0.00750447	0	-0.00775471	0.023232	0.7385349	0.05880055
ENSG00000178295	GEN1	2	17950878	T	C	0.523857	0.0137843	0.0141993	0.16	-0.146406	0.00793159	4.45E-76	-0.0941512	0.0971198	0.332329	0.2410678
ENSG00000178297	TMPRSS9	19	2408003	G	A	0.0397614	-0.039152	0.0342793	0.3599996	0.90896	0.0202447	0	-0.0430734	0.0377249	0.2535465	0.4639377
ENSG00000178301	AQP11	11	77310918	A	G	0.214712	0.00170282	0.0165621	0.7600007	0.101969	0.00960067	2.38E-26	0.0166994	0.162431	0.9181142	0.7924306
ENSG00000178338	ZNF354B	5	178301038	G	A	0.477137	-0.00849744	0.0142222	0.4799997	0.0658621	0.00866539	2.95E-14	-0.129019	0.216605	0.551416	0.3302053
ENSG00000178342	KCNG2	18	77641926	G	A	0.250497	0.00692209	0.0159575	0.4700002	0.10888	0.00885963	1.03E-34	0.0635757	0.146652	0.6646422	0.6169511
ENSG00000178381	ZFAND2A	7	1196051	T	A	0.423459	0.0243471	0.014456	0.05	0.713693	0.00700303	0	0.0341142	0.020258	0.09218338	0.7798872
ENSG00000178386	ZNF223	19	44563834	A	G	0.10835	0.0111775	0.0213773	0.4899999	0.202579	0.0115688	1.19E-68	0.0551759	0.105573	0.6012284	0.2588863
ENSG00000178397	FAM220A	7	6378826	A	G	0.261431	-0.0167534	0.0160185	0.6100002	0.148987	0.0100386	7.91E-50	-0.112449	0.107783	0.2968136	0.05408285
ENSG00000178404	CEP295NL	17	76883145	A	G	0.439364	0.000590096	0.0143569	0.9299999	0.089074	0.00950345	7.06E-21	0.00662479	0.161181	0.967215	0.3385113
ENSG00000178409	BEND3	6	107411429	T	C	0.410537	-0.0224881	0.0147487	0.05699936	-0.0847419	0.00905671	8.22E-21	0.265372	0.176338	0.1323495	0.9537282
ENSG00000178425	NT5DC1	6	116494433	G	A	0.451292	0.00605026	0.0142168	0.3900004	-0.168535	0.00797164	3.28E-99	-0.0358992	0.0843724	0.6704832	0.9935097
ENSG00000178430	NA	16	3726235	C	T	0.209742	-0.00370858	0.0177718	0.56	0.165815	0.024842	2.48E-11	-0.0223657	0.107231	0.8347793	0.4255568
ENSG00000178440	TIMM23B-AGAP6	10	51737168	G	A	0.328032	0.0131709	0.0152655	0.3100002	-0.124108	0.0169667	2.58E-13	-0.106124	0.123854	0.3915285	0.1186409
ENSG00000178445	GLDC	9	6589057	A	G	0.250497	0.00615504	0.0165051	0.7700005	0.277654	0.00915773	6.42E-202	0.022168	0.0594493	0.7092309	0.7528985
ENSG00000178449	COX14	12	50532482	T	C	0.348907	-0.00797613	0.0147349	0.84	0.293954	0.00911593	3.98E-228	-0.0271339	0.0501336	0.5883474	0.5035561
ENSG00000178464	RPL10P16	19	12754411	G	A	0.0974155	0.01003	0.0229085	0.56	-0.305388	0.0373171	2.75E-16	-0.0328435	0.0751217	0.6619638	0.4938959
ENSG00000178467	P4HTM	3	49035953	A	G	0.33499	-0.00554992	0.0149477	0.4299995	-0.32448	0.0094747	4.83E-257	0.017104	0.0460694	0.7104384	0.01800061
ENSG00000178498	DTX3	12	58000996	T	C	0.292247	0.0117925	0.01603	0.4500005	0.0730169	0.00894202	3.20E-16	0.161504	0.220427	0.4637509	0.5538813
ENSG00000178502	KLHL11	17	40015740	G	A	0.260437	0.0186092	0.0164645	0.1900002	0.131257	0.00981783	9.15E-41	0.141777	0.125885	0.2600621	0.7103156
ENSG00000178538	CA8	8	61146938	C	T	0.378728	-0.00891634	0.0147138	0.4600002	-0.188082	0.00807531	5.47E-120	0.0474067	0.0782573	0.5446609	0.1299625
ENSG00000178562	CD28	2	204586877	T	A	0.232604	0.0100665	0.0172285	0.7400005	-0.124786	0.0142645	2.17E-18	-0.08067	0.138372	0.559897	0.8092901
ENSG00000178567	EPM2AIP1	3	37031076	T	C	0.00994036	-0.00558274	0.0601618	0.9599999	0.577202	0.0355617	3.04E-59	-0.00967206	0.104232	0.9260673	0.1477892
ENSG00000178573	MAF	16	79627175	A	G	0.333996	-0.0157407	0.0156843	0.33	0.109863	0.00850073	3.30E-38	-0.143275	0.143192	0.3170281	0.6312841
ENSG00000178585	CTNNBIP1	1	9939364	G	A	0.172962	0.0152239	0.0183481	0.29	0.285803	0.0104494	1.05E-164	0.0532671	0.064228	0.4069094	0.3093192
ENSG00000178607	ERN1	17	62163928	T	G	0.140159	0.0386279	0.019327	0.04900044	-0.412751	0.0108056	0	-0.0935864	0.0468889	0.04594271	0.09170862

ENSG00000178623	GPR35	2	241557762	G	A	0.452286	-0.0108931	0.0142287	0.4199997	0.29265	0.00851378	6.23E-259	-0.0372223	0.0486322	0.4440438	0.555979
ENSG00000178636	NA	4	120123863	G	T	0.127237	-0.00612404	0.0215414	0.7300002	0.548011	0.0332329	4.33E-61	-0.011175	0.0393142	0.7762193	0.4690965
ENSG00000178642	NA	1	2318366	G	A	0.252485	-0.00772582	0.0164406	0.7300002	-0.403273	0.025232	1.69E-57	0.0191578	0.0407855	0.6385549	0.09739178
ENSG00000178665	ZNF713	7	55982543	T	C	0.05666	-0.0259235	0.0316831	0.4400003	0.400619	0.0177729	1.65E-112	-0.0647086	0.0791375	0.4135435	0.948202
ENSG00000178685	PARP10	8	145069130	C	T	0.387674	0.0171542	0.0145875	0.2300001	-0.227372	0.00944399	4.48E-128	-0.0754456	0.0642336	0.2401741	0.1045772
ENSG00000178691	SUZ12	17	30296050	A	G	0.131213	0.03751	0.0215372	0.08400014	-0.083697	0.0123739	1.34E-11	-0.448164	0.265717	0.09167566	0.3049652
ENSG00000178694	NSUN3	3	93814574	G	A	0.0387674	-0.0540351	0.0436789	0.16	0.153957	0.0230613	2.46E-11	-0.350974	0.288537	0.2238362	0.921428
ENSG00000178695	KCTD12	13	77457426	T	G	0.0586481	-0.00417647	0.030545	0.9	-0.432741	0.0156923	2.12E-167	0.00965119	0.0705858	0.8912442	0.519971
ENSG00000178700	DHFR2	3	93774456	C	T	0.23161	-0.0313968	0.0162001	0.04700023	-0.139108	0.00940921	1.85E-49	0.225702	0.117454	0.05465343	0.6730413
ENSG00000178715	RPS16P1	1	16154945	C	A	0.45328	-0.0063571	0.0142265	0.6999999	-0.25254	0.0187997	3.86E-41	0.0251726	0.0563648	0.6551623	0.3753659
ENSG00000178718	RPP25	15	75248281	T	C	0.0248509	-0.00735568	0.0596864	0.7800007	-0.219845	0.0285098	1.25E-14	0.0334585	0.271528	0.9019305	0.1526839
ENSG00000178719	GRINA	8	145065904	A	C	0.369781	0.020185	0.0147027	0.16	-0.491646	0.0116386	0	-0.0410559	0.0299208	0.1700159	0.2698854
ENSG00000178726	THBD	20	23028324	A	G	0.110338	-0.00803132	0.024096	0.7700005	-0.593083	0.0129149	0	0.0135416	0.0406294	0.7389106	0.1533894
ENSG00000178732	GP5	3	194117772	C	T	0.0417495	0.0255116	0.0384293	0.4899999	0.585377	0.0341041	4.91E-66	0.0435815	0.0656979	0.5070987	0.5274323
ENSG00000178741	COX5A	15	75221320	C	T	0.333996	1.43E-05	0.01564	0.7300002	-0.11858	0.0127259	1.19E-20	-0.000120364	0.131894	0.9992719	0.1560807
ENSG00000178752	ERFE	2	239072582	G	A	0.196819	0.00479322	0.0184383	0.9400001	-0.636633	0.0230492	6.30E-168	-0.00752902	0.0289635	0.7949035	0.111465
ENSG00000178761	FAM219B	15	75195895	A	G	0.335984	-0.00300055	0.0156059	0.5999997	-0.219836	0.00954059	1.76E-117	0.013649	0.0709912	0.8475358	0.6724811
ENSG00000178764	ZHX2	8	123890191	C	T	0.338966	-0.000382799	0.0146007	0.6999999	0.253649	0.00818795	1.05E-210	-0.00150917	0.0575625	0.9790836	0.6644814
ENSG00000178773	CPNE7	16	89652915	G	C	0.180915	0.0274114	0.0185569	0.0649995	-0.166214	0.0109672	6.96E-52	-0.164917	0.112174	0.141511	0.5679003
ENSG00000178789	CD300LB	17	72522463	T	C	0.0168986	-0.00101078	0.0401335	0.98	0.790706	0.0267174	1.72E-192	-0.00127833	0.0507566	0.9799071	0.5659598
ENSG00000178802	MPI	15	75187072	A	G	0.489066	-0.0199852	0.0143178	0.07299952	-0.279715	0.00783569	4.31E-279	0.0714483	0.0512261	0.1630873	0.2353129
ENSG00000178803	ADORA2A-AS1	22	24858110	T	A	0.389662	-0.00853403	0.0145223	0.4799997	-0.0600896	0.00967876	5.35E-10	0.142022	0.242758	0.5585241	0.2044754
ENSG00000178809	TRIM73	7	75032308	T	A	0.444334	-0.0126044	0.014379	0.3900004	0.539866	0.0178325	2.51E-201	-0.0233473	0.0266456	0.3809122	0.004629873
ENSG00000178852	EFCAB13	17	45459667	G	A	0.082505	-0.0424185	0.0283027	0.08500021	-0.312383	0.016926	4.68E-76	0.13579	0.0909009	0.135221	0.2037541
ENSG00000178860	MSC	8	72755243	T	C	0.291252	0.0116992	0.0158478	0.5400003	0.361227	0.0087583	0	0.0323874	0.0438792	0.4604512	0.6710065
ENSG00000178878	APOLD1	12	12911632	G	A	0.419483	-0.0150174	0.0144531	0.2300001	0.283972	0.00789644	3.29E-283	-0.0528834	0.0509175	0.2989861	0.2460281
ENSG00000178904	DPY19L3	19	32936728	T	C	0.141153	-0.00654668	0.0195401	0.6600001	0.279898	0.0107221	3.22E-150	-0.0233895	0.0698173	0.7376171	0.9181361
ENSG00000178913	TAF7	5	140699193	G	T	0.0477137	0.0182944	0.0346738	0.6600001	-0.427379	0.0196769	1.33E-104	-0.042806	0.0811551	0.5978749	0.9905374
ENSG00000178917	ZNF852	3	44546295	A	G	0.425447	-0.00200381	0.0145326	0.8499999	0.0584978	0.0101902	9.43E-09	-0.0342545	0.248502	0.8903637	0.5373057
ENSG00000178921	PFAS	17	8162372	A	G	0.0705765	-0.026801	0.0299173	0.6200004	0.344523	0.0188165	6.94E-75	-0.0777915	0.0869407	0.3709123	0.5744884
ENSG00000178922	HYI	1	43918242	A	G	0.0159046	0.0912964	0.056789	0.1199999	0.806242	0.0539076	1.42E-50	0.113237	0.0708424	0.1099463	0.6764786
ENSG00000178927	CYBC1	17	80404585	C	A	0.127237	0.00888194	0.0212445	0.6700003	-0.453022	0.011387	0	-0.019606	0.0468977	0.6759041	0.5908228
ENSG00000178935	ZNF552	19	58320745	A	G	0.321074	0.0277245	0.015358	0.09400046	-0.155611	0.00847082	2.28E-75	-0.178166	0.0991704	0.0724052	0.788086
ENSG00000178950	GAK	4	884612	T	C	0.0427435	-0.00599409	0.0344087	0.9400001	0.16145	0.0238366	1.26E-11	-0.0371266	0.213193	0.8617514	0.9616986
ENSG00000178951	ZBTB7A	19	4055652	G	A	0.282306	-0.00835192	0.0158088	0.35	0.128241	0.0107552	8.92E-33	-0.0651267	0.123395	0.5976448	0.4029202
ENSG00000178952	TUFM	16	28855730	G	A	0.341948	-0.00334924	0.0144617	0.8600001	0.846998	0.00659152	0	-0.00395425	0.0170741	0.8168538	0.9595438
ENSG00000178966	RMI1	9	86607305	T	C	0.27833	-0.00734318	0.016237	0.56	0.211594	0.00889177	3.62E-125	-0.0347041	0.0767505	0.6511479	0.9324982
ENSG00000178971	CTC1	17	8140776	A	G	0.27833	0.000361417	0.0158175	0.9299999	-0.748573	0.00879877	0	-0.000482808	0.0211302	0.9817706	0.5652052
ENSG00000178974	FBXO34	14	55783328	T	C	0.440358	0.00789409	0.0143517	0.3800004	0.18344	0.00796858	2.91E-117	0.0430337	0.0782589	0.5823957	0.41764
ENSG00000178977	LINC00324	17	8125660	G	A	0.228628	0.00271837	0.0169753	0.9	-0.69862	0.011379	0	-0.00389106	0.0242984	0.8727738	0.856111
ENSG00000178980	SELENOW	19	48284886	C	T	0.397614	-0.0120949	0.0148522	0.28	-0.308049	0.00809478	0	0.0392629	0.0482247	0.4155508	0.08376027
ENSG00000178982	EIF3K	19	39118665	G	A	0.0934394	0.000531322	0.0252573	0.8200001	0.346298	0.0209656	2.75E-61	0.00153429	0.0729353	0.9832167	0.4444679
ENSG00000178988	MRFAP1L1	4	6710517	C	T	0.0407555	-0.00575777	0.0382287	0.8700001	0.147561	0.0222801	3.52E-11	-0.0390195	0.259137	0.8803111	NA
ENSG00000178996	SNX18	5	53828002	G	A	0.357853	-0.0202843	0.0149403	0.1100001	0.178254	0.00908197	9.06E-86	-0.113795	0.0840152	0.1755917	0.1452777
ENSG00000178999	AURKB	17	8110987	C	T	0.299205	0.00186746	0.0155417	0.9699999	-0.238917	0.0128623	5.12E-77	-0.00781636	0.065052	0.90436	0.7396436
ENSG00000179010	MRFAP1	4	6643145	G	T	0.247515	0.030828	0.0168644	0.06100002	-0.14613	0.00939297	1.42E-54	-0.210963	0.116201	0.06944664	0.4663249
ENSG00000179021	C3orf38	3	88203005	G	T	0.157058	0.00813508	0.0195819	0.3800004	-0.169992	0.0111737	2.87E-52	-0.0478556	0.115236	0.6779341	0.1419599
ENSG00000179023	KLHDC7A	1	18809951	T	C	0.511928	0.0006289	0.0141864	0.9199999	-0.104901	0.00798542	2.03E-39	-0.00599516	0.135236	0.9646406	0.1633067
ENSG00000179029	TMEM107	17	8078136	A	G	0.342942	-0.00227732	0.0149542	0.64	0.656495	0.00818655	0	-0.00346891	0.0227789	0.8789613	0.6062571
ENSG00000179051	RCC2	1	17749738	C	T	0.410537	-0.0139907	0.0145565	0.3700002	0.217205	0.00836493	1.19E-148	-0.0644123	0.0670631	0.3368172	0.422645

ENSG00000179057	IGSF22	11	18736814	C	T	0.397614	-0.00801473	0.0145139	0.7099994	0.176941	0.00906944	9.10E-85	-0.045296	0.0820595	0.5809555	0.8640731
ENSG00000179082	NA	9	132085239	T	C	0.0437376	-0.0898799	0.0342836	0.004399973	-0.141291	0.021711	7.63E-11	0.636133	0.261594	0.01502595	0.0368569
ENSG00000179085	DPM3	1	155112719	G	A	0.407555	-0.0284706	0.0144259	0.03599979	-0.0875622	0.00810416	3.27E-27	0.325147	0.167476	0.05220337	0.41892
ENSG00000179088	C12orf42	12	103760559	C	T	0.0387674	-0.028715	0.0388003	0.5400003	0.155815	0.0238198	6.09E-11	-0.184289	0.250604	0.462108	0.798492
ENSG00000179091	CYC1	8	145151179	C	T	0.0884692	0.0163475	0.0253272	0.4799997	-0.132209	0.0174027	3.03E-14	-0.123649	0.19226	0.5201363	0.4568564
ENSG00000179104	TMTC2	12	83304654	T	A	0.333002	0.0210654	0.0150539	0.14	-0.104656	0.00823685	5.49E-37	-0.201283	0.144712	0.1642492	0.9444879
ENSG00000179115	FARSA	19	13039072	G	A	0.313121	-0.0198846	0.0146889	0.2	0.167758	0.00828639	3.93E-91	-0.118531	0.0877556	0.1767916	0.5836816
ENSG00000179119	SPTY2D1	11	18642143	C	T	0.511928	-0.00465481	0.0142722	0.6999999	0.0536587	0.00795651	1.54E-11	-0.0867484	0.266292	0.7446025	0.373688
ENSG00000179134	SAMD4B	19	39854693	T	C	0.0437376	-0.0365939	0.0335974	0.2999998	-0.343261	0.0202921	3.43E-64	0.106607	0.0980798	0.2770644	0.6247535
ENSG00000179144	GIMAP7	7	150215039	T	C	0.424453	0.00561686	0.0144008	0.4700002	-0.179659	0.00794623	3.50E-113	-0.0312639	0.080168	0.6965513	0.512957
ENSG00000179152	TCAIM	3	44415277	G	A	0.135189	-0.0248279	0.0176688	0.3800004	-0.0981171	0.011798	9.07E-17	0.253043	0.182631	0.1658861	0.9650416
ENSG00000179163	FUCA1	1	24183175	G	A	0.412525	0.00948222	0.0143392	0.3599996	-0.485505	0.00763647	0	-0.0195306	0.0295362	0.508456	0.06195827
ENSG00000179195	ZNF664	12	124478189	T	G	0.368787	-0.0210534	0.0149896	0.1199999	-0.176586	0.00846084	9.83E-97	0.119224	0.0850774	0.1611052	0.8498606
ENSG00000179241	LDLRAD3	11	36109608	A	G	0.370775	-0.00741702	0.0145718	0.58	0.0851966	0.00819766	2.67E-25	-0.0870577	0.171242	0.6111807	0.01951382
ENSG00000179242	CDH4	20	60171577	G	A	0.450298	-0.0162988	0.0144088	0.1800002	-0.107036	0.00903609	2.27E-32	0.152274	0.135229	0.2601456	0.7794652
ENSG00000179253	LOC100128310	20	60293683	T	C	0.409543	-0.00985376	0.0145879	0.4	-0.297356	0.0142061	2.76E-97	0.0331379	0.0490842	0.4995973	0.1746224
ENSG00000179262	RAD23A	19	13060562	C	G	0.0497018	-0.0091955	0.0372988	0.7300002	0.522668	0.0204711	8.70E-144	-0.0175934	0.0713656	0.8052762	0.7052725
ENSG00000179294	NA	17	36829574	T	G	0.356859	-0.00693109	0.0145763	0.58	0.102954	0.0100884	1.88E-24	-0.0673221	0.141734	0.634795	0.9858801
ENSG00000179295	PTPN11	12	112901936	C	G	0.082505	-0.000417624	0.026019	0.9199999	0.250372	0.0235047	1.71E-26	-0.00166801	0.103921	0.9871939	0.6747558
ENSG00000179296	NA	10	49228820	C	T	0.210736	0.019148	0.0184975	0.3400001	0.33496	0.0227326	3.85E-49	0.057165	0.0553591	0.3017809	0.09872041
ENSG00000179299	NSUN7	4	40781958	G	C	0.369781	0.00139979	0.0145959	0.6999999	0.364519	0.00795271	0	0.0038401	0.0400416	0.9235978	0.9142761
ENSG00000179314	WSCD1	17	5851650	T	C	0.248509	0.0145984	0.0160363	0.28	-0.0631438	0.00911745	4.34E-12	-0.231193	0.256149	0.3667537	0.1364602
ENSG00000179344	HLA-DQB1	6	32631702	T	C	0.428429	0.0124069	0.0145593	0.3400001	0.663622	0.008737	0	0.0186957	0.0219405	0.3941533	0.1273514
ENSG00000179348	GATA2	3	128205149	G	A	0.101392	0.0247019	0.0229996	0.2999998	-0.192735	0.0123057	2.74E-55	-0.128165	0.119613	0.2839456	0.9754823
ENSG00000179361	ARID3B	15	74861995	G	A	0.107356	0.018797	0.023506	0.3800004	-0.276424	0.0210209	1.70E-39	-0.0680005	0.085193	0.4247581	0.7521018
ENSG00000179364	PACS2	14	105815692	T	C	0.319085	0.025867	0.0153734	0.0519996	0.0709172	0.00874916	5.25E-16	0.364749	0.221401	0.09946333	0.4935352
ENSG00000179388	EGR3	8	22547993	C	G	0.225646	-0.00612996	0.017548	0.6200004	0.0698251	0.0101905	7.28E-12	-0.0877902	0.25164	0.7271853	0.4409958
ENSG00000179397	CATSPERE	1	244711079	A	G	0.34493	0.0124686	0.0151474	0.5400003	0.133148	0.012345	4.03E-27	0.0936443	0.114094	0.4117805	0.7360929
ENSG00000179406	LINC00174	7	65853678	T	C	0.0506958	0.0136236	0.0340813	0.7499995	0.82881	0.0231606	1.86E-280	0.0164375	0.0411233	0.6893677	0.7548698
ENSG00000179409	GEMIN4	17	652446	T	G	0.11332	-0.0213503	0.0237357	0.2700001	-0.336048	0.0127223	9.41E-154	0.0635334	0.0706727	0.3686631	0.9637318
ENSG00000179428	IL6-AS1	7	22766126	A	G	0.410537	0.00322479	0.0143674	0.81	0.0901109	0.010409	4.84E-18	0.0357869	0.159495	0.8224644	0.783166
ENSG00000179454	KLHL28	14	45454598	T	C	0.0129225	-0.083625	0.056334	0.1199999	0.373942	0.050748	1.72E-13	-0.223631	0.153676	0.14561	0.1271335
ENSG00000179455	MKRN3	15	23841759	A	T	0.413519	-0.0111172	0.0147919	0.3599996	-0.0720508	0.00898591	1.07E-15	0.154297	0.206198	0.4542832	0.8366379
ENSG00000179456	ZBTB18	1	244217681	A	G	0.22664	-0.000815531	0.0173925	0.9599999	-0.0712286	0.0117214	1.23E-09	0.0114495	0.244186	0.9626022	0.8208075
ENSG00000179467	NA	20	55097634	A	G	0.341948	-0.00458367	0.0150649	0.6200004	0.144617	0.0201562	7.24E-13	-0.0316953	0.104265	0.7611368	0.7187224
ENSG00000179526	SHARPIN	8	145158281	C	A	0.0884692	0.0107185	0.0253942	0.6200004	0.41593	0.0160058	7.09E-149	0.0257699	0.061062	0.6730039	0.5844043
ENSG00000179528	LBX2	2	74728418	T	C	0.158052	-0.0109269	0.0207379	0.5999997	0.260872	0.0120348	3.43E-104	-0.0418861	0.0795182	0.5983677	0.4578703
ENSG00000179532	DNHD1	11	6566739	C	T	0.369781	-0.00689934	0.0151085	0.4400003	0.240779	0.00904518	4.03E-156	-0.0286542	0.0627576	0.6479689	0.4850235
ENSG00000179562	GCC1	7	127227168	G	A	0.214712	-0.0282146	0.0175227	0.08	-0.22526	0.00940537	9.20E-127	0.125253	0.0779642	0.1081536	0.9799997
ENSG00000179583	CIITA	16	10998567	A	G	0.417495	0.0116173	0.014551	0.4400003	0.136995	0.0082936	2.71E-61	0.0848007	0.106339	0.4251874	0.8509762
ENSG00000179588	ZFPM1	16	88560648	T	A	0.190855	-0.0172789	0.0172051	0.3900004	0.3573	0.01226	1.01E-186	-0.0483596	0.0481817	0.3155263	0.2375263
ENSG00000179593	ALOX15B	17	7947393	A	G	0.0188867	-0.0459114	0.0506557	0.2700001	-0.408403	0.0256554	4.69E-57	0.112417	0.124234	0.3655307	0.4066358
ENSG00000179598	PLD6	17	17106969	A	C	0.05666	0.0141474	0.0283892	0.4199997	0.996392	0.0167378	0	0.0141986	0.028493	0.6182586	0.7256281
ENSG00000179604	CDC42EP4	17	71294038	T	C	0.394632	0.00390028	0.0146729	0.7899998	-0.108181	0.00811039	1.38E-40	-0.0360532	0.135659	0.7904222	0.6817185
ENSG00000179627	ZBTB42	14	105268991	A	G	0.274354	-0.00720144	0.01658	0.4500005	0.0936879	0.0135254	4.30E-12	-0.0768662	0.177318	0.6646562	0.4906843
ENSG00000179630	LACC1	13	44460744	G	C	0.190855	-0.0249294	0.0203433	0.1499999	-0.0886675	0.0115842	1.95E-14	0.281156	0.232355	0.226269	0.5451028
ENSG00000179632	MAF1	8	145160958	G	A	0.0884692	0.0108808	0.0253979	0.6100002	-0.79953	0.0156625	0	-0.013609	0.0317671	0.6683612	0.3403932
ENSG00000179639	FCER1A	1	159268759	G	C	0.271372	0.0294536	0.016112	0.02300011	0.246857	0.00912136	2.64E-161	0.119315	0.0654174	0.06816756	0.09529508
ENSG00000179698	WDR97	8	145167923	T	C	0.0884692	0.0170241	0.0253413	0.4600002	-0.883665	0.0181913	0	-0.0192653	0.0286802	0.5017566	0.5201011
ENSG00000179715	PCED1B	12	47551914	T	C	0.483101	-0.0299732	0.0142531	0.02900013	0.328241	0.00861835	0	-0.0913146	0.0434888	0.03575289	0.3893811

ENSG00000179743	SPEN-AS1	1	16167601	T	C	0.415507	-0.0101151	0.0143612	0.5	-0.236425	0.00941323	3.31E-139	0.0427836	0.0607671	0.4813963	0.4234609
ENSG00000179750	APOBEC3B	22	39383580	A	G	0.261431	0.000905739	0.0163283	0.9699999	0.328308	0.00955987	1.84E-258	0.00275881	0.0497348	0.9557637	0.1657788
ENSG00000179818	PCBP1-AS1	2	70252686	C	G	0.0725646	0.0465044	0.0308367	0.14	-0.785367	0.0252036	3.61E-213	-0.0592136	0.03931	0.1319843	0.9177478
ENSG00000179820	MYADM	19	54374584	T	C	0.0149105	-0.0748777	0.0621009	0.1199999	-0.176107	0.029075	1.39E-09	0.425183	0.35955	0.2369916	0.3811333
ENSG00000179832	MROH1	8	145259881	G	A	0.0149105	0.0272608	0.042761	0.56	0.912551	0.0860021	2.65E-26	0.0298732	0.0469433	0.5245366	0.7230001
ENSG00000179833	SERTAD2	2	64918102	G	A	0.460239	-0.00225315	0.014291	0.9	-0.144264	0.00808049	2.72E-71	0.0156182	0.0990652	0.8747279	0.4569505
ENSG00000179837	NA	3	51432030	A	G	0.213718	-0.00637118	0.0172714	0.8499999	0.202601	0.00961779	1.66E-98	-0.031447	0.0852615	0.7122547	0.2005646
ENSG00000179846	NKPD1	19	45658208	T	G	0.127237	-0.00684052	0.0219826	0.5300002	-0.134693	0.0126844	2.44E-26	0.050786	0.163275	0.7557662	0.08375168
ENSG00000179855	GIPC3	19	3589545	A	G	0.117296	0.0107006	0.0226509	0.4299995	-0.521326	0.0189006	1.80E-167	-0.0205257	0.043455	0.6366808	0.1981876
ENSG00000179859	RNF227	17	7817956	G	A	0.00497018	-0.0502129	0.112503	0.5300002	1.04796	0.0727546	4.88E-47	-0.047915	0.107406	0.6555172	0.6018551
ENSG00000179862	CITED4	1	41327373	G	A	0.114314	-0.0399823	0.0233346	0.0659994	0.70002	0.0108404	0	-0.0571159	0.0333459	0.08674358	0.6991389
ENSG00000179869	ABCA13	7	48449073	T	A	0.171968	-0.0311694	0.0184664	0.06199976	0.0807779	0.010051	9.22E-16	-0.385865	0.233594	0.09856304	0.4880613
ENSG00000179886	TIGD5	8	144681279	C	G	0.38171	-0.0223818	0.0146419	0.07100027	-0.286476	0.00921349	2.99E-212	0.078128	0.0511721	0.1268184	0.7174944
ENSG00000179889	PDXDC1	16	15150822	G	A	0.33499	0.0126711	0.0150352	0.4100001	-0.0859372	0.0101533	2.58E-17	-0.147446	0.175821	0.4016851	0.6741599
ENSG00000179902	CFAP276	1	109652526	G	A	0.0884692	0.00309087	0.0255931	0.98	0.20871	0.0168897	4.45E-35	0.0148094	0.122631	0.903878	0.4810251
ENSG00000179909	ZNF154	19	58214657	T	C	0.0248509	-0.00924673	0.0488467	0.9299999	0.614152	0.02678	2.17E-116	-0.0150561	0.0795379	0.849862	0.03497149
ENSG00000179912	R3HDM2	12	57734090	G	A	0.467197	-0.0149418	0.0145145	0.5500004	-0.0591067	0.00803451	1.89E-13	0.252794	0.247957	0.3079627	0.9664119
ENSG00000179913	B3GNT3	19	17914764	A	G	0.299205	-0.00172186	0.0158176	0.64	-0.266932	0.00955579	1.03E-171	0.00645057	0.0592576	0.9133164	0.3149178
ENSG00000179914	ITLN1	1	160850644	A	G	0.195825	-0.00326597	0.0179053	0.9699999	-0.529641	0.0138908	0	0.00616639	0.0338069	0.8552686	0.3635911
ENSG00000179915	NRXN1	2	50702658	C	T	0.422465	0.00272965	0.0143267	0.7099994	0.172277	0.0118958	1.57E-47	0.0158446	0.0831682	0.8489076	0.7622865
ENSG00000179918	SEPHS2	16	30456227	C	T	0.383698	-0.00124155	0.0150822	0.6499995	0.14251	0.00820797	1.59E-67	-0.00871201	0.105834	0.9343939	0.2214888
ENSG00000179921	GPBAR1	2	219126400	T	A	0.368787	0.00530087	0.0145409	0.5199996	0.241479	0.00807677	2.09E-196	0.0219517	0.0602204	0.7154683	0.5475504
ENSG00000179922	ZNF784	19	56134037	C	T	0.166004	0.000455631	0.0178642	0.9599999	0.0728634	0.0103476	1.90E-12	0.00625322	0.245176	0.9796521	0.4085214
ENSG00000179933	C14orf119	14	23566819	G	C	0.0497018	0.00266551	0.0306965	0.98	-0.169429	0.0169226	1.35E-23	-0.0157323	0.181183	0.9308058	0.244949
ENSG00000179934	CCR8	3	39373184	G	A	0.0129225	0.0104471	0.0659762	0.9199999	0.640222	0.0705479	1.14E-19	0.0163179	0.103068	0.8742028	0.1612241
ENSG00000179941	BBS10	12	76740238	C	T	0.234592	0.0188988	0.0158988	0.2599998	-0.241312	0.00897578	3.31E-159	-0.0783169	0.0659492	0.2350173	0.8864526
ENSG00000179943	FIZ1	19	56108041	C	T	0.384692	-0.0195707	0.0144294	0.14	-0.059614	0.00815133	2.60E-13	0.328291	0.246175	0.1823455	0.9712712
ENSG00000179950	PUF60	8	144905271	G	A	0.0318091	0.0996283	0.037487	0.005899973	0.164102	0.0284969	8.48E-09	0.607112	0.251592	0.01581817	0.6954202
ENSG00000179954	SSC5D	19	56015118	T	G	0.184891	-0.00421989	0.0185582	0.8600001	0.237024	0.0136951	4.15E-67	-0.0178036	0.0783034	0.8201382	0.3450344
ENSG00000179958	DCTPP1	16	30438168	T	C	0.521869	-0.017936	0.0141972	0.07399971	0.0558687	0.00799723	2.83E-12	-0.321039	0.258239	0.2138003	0.9782609
ENSG00000179965	ZNF771	16	30429769	T	C	0.54672	-0.0182295	0.014189	0.08799946	0.0582834	0.0079779	2.76E-13	-0.312774	0.247184	0.2057476	0.9844458
ENSG00000179967	NA	4	140036306	C	T	0.203777	0.0135661	0.0168962	0.3100002	0.161896	0.0226923	9.72E-13	0.0837954	0.105024	0.424945	0.2305278
ENSG00000179978	NA	5	69407231	A	G	0.10835	-0.0170346	0.0235311	0.56	-0.429448	0.0449957	1.37E-21	0.0396663	0.0549512	0.4703897	0.8366515
ENSG00000179979	NA	4	1387560	A	G	0.139165	0.00266226	0.0204873	0.7600007	0.846046	0.0114774	0	0.00314671	0.0242154	0.8966086	0.3836957
ENSG00000179981	TSHZ1	18	72962307	C	T	0.468191	-0.0032914	0.0143807	0.7600007	-0.182725	0.00806446	1.16E-113	0.0180129	0.0787055	0.8189742	0.4624732
ENSG00000179988	PSTK	10	124735463	G	A	0.270378	-0.00205581	0.0153539	0.9400001	-0.256344	0.00925306	6.30E-169	0.00801973	0.0598964	0.8934869	0.7129186
ENSG00000180011	PTGR3	18	72914183	G	A	0.449304	0.00173682	0.0144673	0.95	-0.595596	0.00734557	0	-0.00291611	0.0242905	0.9044425	0.317274
ENSG00000180066	NA	10	134260371	T	C	0.0198807	-0.105093	0.0694074	0.1299999	0.625748	0.0513919	4.17E-34	-0.167948	0.111773	0.1329491	0.1116187
ENSG00000180071	ANKRD18A	9	38580610	C	G	0.134195	0.0299957	0.0212121	0.2200002	0.154387	0.0135773	5.83E-30	0.194289	0.138454	0.1605348	0.4333428
ENSG00000180089	TMEM86B	19	55739827	T	C	0.370775	-0.013468	0.0144454	0.4299995	-0.296222	0.0137287	2.97E-103	0.045466	0.048811	0.3516109	0.816961
ENSG00000180096	SEPTIN1	16	30398383	C	T	0.341948	0.0111959	0.0148525	0.4	-0.0669463	0.00915768	2.66E-13	-0.167237	0.223033	0.4533571	0.4072736
ENSG00000180104	EXOC3	5	457662	T	C	0.399602	0.0148854	0.0144303	0.09599973	-0.646742	0.00807583	0	-0.023016	0.0223142	0.3023288	0.2575165
ENSG00000180113	TDRD6	6	46663834	A	G	0.0238569	-0.0759023	0.0518127	0.08100093	-0.429903	0.0252415	4.79E-65	0.176557	0.120967	0.1444146	0.9416686
ENSG00000180138	CSNK1A1L	13	37678600	T	C	0.468191	0.00327956	0.0142188	0.8600001	0.0901285	0.00804632	4.02E-29	0.0363876	0.157795	0.817625	0.4068187
ENSG00000180139	ACTA2-AS1	10	90696398	C	T	0.531809	-0.0426015	0.0141599	0.001700004	0.193341	0.018895	1.42E-24	-0.220343	0.076338	0.003896524	0.04002966
ENSG00000180155	LYNX1	8	143852696	A	C	0.40159	-0.00691221	0.0144959	0.95	0.264685	0.00903801	1.57E-188	-0.0261148	0.0547738	0.6335218	0.9269677
ENSG00000180185	FAHD1	16	1883588	T	G	0.180915	-0.0279435	0.019703	0.14	0.418634	0.0102292	0	-0.0667492	0.0470932	0.1563704	0.44441
ENSG00000180190	TDRP	8	467792	C	G	0.0924453	-0.0312839	0.0240765	0.14	-0.114501	0.0143881	1.75E-15	0.27322	0.213058	0.1997118	0.1809789
ENSG00000180198	RCC1	1	28849133	C	T	0.0218688	-0.00740961	0.0509704	0.99	0.926735	0.0344393	1.71E-159	-0.0079954	0.0550008	0.8844198	0.9177065
ENSG00000180228	PRKRA	2	179306190	A	G	0.0248509	-0.0178277	0.0437637	0.7199992	0.503889	0.0278213	2.58E-73	-0.0353802	0.0868739	0.6838172	0.9175187

ENSG00000180233	ZNRF2	7	30388020	A	G	0.0139165	-0.0830554	0.0575464	0.1100001	-0.441161	0.0365878	1.77E-33	0.188265	0.131374	0.1518443	0.1429284
ENSG00000180257	ZNF816	19	53448276	C	T	0.406561	0.00263752	0.0146732	0.8700001	0.292017	0.00919265	1.89E-221	0.00903209	0.0502486	0.8573504	0.4335246
ENSG00000180263	FGD6	12	95540891	C	T	0.148111	-0.00224476	0.0183503	0.9599999	-0.476099	0.015424	3.27E-209	0.0047149	0.0385433	0.9026398	0.7869723
ENSG00000180279	LINC01869	19	51321535	T	A	0.27336	0.00803188	0.0161269	0.64	0.254128	0.0103125	4.42E-134	0.0316057	0.0634728	0.6185258	0.8384146
ENSG00000180316	PNPLA1	6	36243676	T	C	0.364811	0.0180881	0.0147924	0.1800002	0.111846	0.00828098	1.43E-41	0.161723	0.132798	0.2232939	0.3238377
ENSG00000180340	FZD2	17	42635916	T	C	0.184891	-0.000806427	0.0175423	0.95	0.378555	0.00993806	0	-0.00213028	0.0463402	0.9633338	0.1373927
ENSG00000180346	TIGD2	4	90035009	C	T	0.0904573	0.0151876	0.0231094	0.4	-0.29961	0.0137361	1.79E-105	-0.0506912	0.0771666	0.5112412	0.9482775
ENSG00000180353	HCLS1	3	121365010	C	A	0.0924453	-0.00847838	0.0234609	0.7499995	-0.853602	0.0120913	0	0.00993247	0.0274849	0.7178164	0.8953914
ENSG00000180354	MTURN	7	30188402	T	C	0.0437376	0.0527263	0.0453847	0.25	-0.458476	0.0276201	7.04E-62	-0.115003	0.0992324	0.2464852	0.3622115
ENSG00000180357	ZNF609	15	64884877	G	C	0.0129225	-0.0459419	0.0529878	0.35	-0.218774	0.027667	2.63E-15	0.209997	0.243655	0.3887632	0.8584842
ENSG00000180370	PAK2	3	196513123	A	G	0.138171	0.0139101	0.0215859	0.5400003	0.182524	0.0131547	8.95E-44	0.0762096	0.118391	0.5197616	0.8350509
ENSG00000180376	CCDC66	3	56623517	C	T	0.110338	0.0205091	0.0240482	0.32	0.663317	0.0225432	2.69E-190	0.030919	0.0362697	0.3939505	0.4851265
ENSG00000180385	NA	3	10038625	G	A	0.143141	0.0315852	0.0189599	0.06800017	0.162527	0.0144899	3.38E-29	0.194338	0.117937	0.09938963	0.8349298
ENSG00000180398	MCFD2	2	47149001	C	T	0.159046	-0.0189743	0.0194417	0.17	-0.451657	0.0108635	0	0.0420104	0.0430571	0.3292181	0.3394278
ENSG00000180409	OR10AA1P	1	158778657	G	A	0.0139165	0.0021609	0.0704263	0.98	1.48145	0.109045	4.88E-42	0.00145864	0.047539	0.9755223	0.7493402
ENSG00000180422	LINC00304	16	89228103	A	G	0.0576541	-0.0212896	0.0302626	0.4500005	0.308357	0.0304857	4.75E-24	-0.0690421	0.0983787	0.4828039	0.9670804
ENSG00000180423	HARBI1	11	46631935	A	C	0.107356	-0.0234796	0.022574	0.2300001	0.1007	0.012786	3.39E-15	-0.233164	0.226117	0.3024642	0.5293759
ENSG00000180425	C11orf71	11	114266699	G	A	0.419483	0.0106778	0.0143329	0.2999998	-0.071007	0.00880455	7.34E-16	-0.150377	0.202711	0.4581922	0.3073752
ENSG00000180447	GAS1	9	89560691	A	G	0.178926	0.00339141	0.0188715	0.9	0.317603	0.0102196	4.79E-212	0.0106781	0.0594194	0.8573821	0.9071033
ENSG00000180448	ARHGAP45	19	1076274	T	G	0.162028	0.0346014	0.0196773	0.05	0.149693	0.0132479	1.32E-29	0.231149	0.133033	0.08229397	0.01895461
ENSG00000180479	ZNF571	19	38065678	T	C	0.167992	-0.00504078	0.0187836	0.5999997	-0.179336	0.0104866	1.45E-65	0.028108	0.104753	0.7884471	0.6249599
ENSG00000180481	GLIPR1L2	12	75805659	T	C	0.37674	0.00590436	0.0144465	0.6600001	-0.26346	0.00814463	1.49E-229	-0.0224108	0.0548381	0.6827799	0.5713824
ENSG00000180509	KCNE1	21	35851780	T	C	0.296223	0.0201233	0.0155773	0.1299999	-0.295709	0.00850251	5.05E-265	-0.068051	0.0527141	0.1967229	0.1462549
ENSG00000180530	NRIP1	21	16385438	C	T	0.464215	0.0227672	0.0142472	0.1199999	-0.147677	0.00791436	1.06E-77	-0.154169	0.0968284	0.1113434	0.470906
ENSG00000180537	RNF182	6	13952605	C	T	0.337972	0.0477332	0.0147939	0.003099988	0.769274	0.00766124	0	0.0620497	0.0192409	0.001260248	0.1803285
ENSG00000180539	LINC02908	9	139926575	A	G	0.272366	0.0231388	0.0158197	0.08799946	0.0706173	0.0117939	2.13E-09	0.327665	0.230607	0.1553524	0.08940594
ENSG00000180543	TSPYL5	8	98287946	T	C	0.0506958	-0.0137043	0.0328516	0.5099998	-0.487277	0.0174591	2.04E-171	0.0281243	0.0674263	0.6765971	0.2001111
ENSG00000180549	FUT7	9	139926044	C	T	0.375746	0.018024	0.0146776	0.14	0.509364	0.00925252	0	0.0353853	0.0288227	0.219564	0.4609078
ENSG00000180573	H2AC6	6	26131858	T	C	0.212724	0.00180393	0.0176613	0.7899998	0.102975	0.0108187	1.76E-21	0.0175181	0.17152	0.91865	0.01266714
ENSG00000180596	H2BC4	6	26119627	T	C	0.250497	-0.0275794	0.0156324	0.07100027	0.0887677	0.0100318	8.86E-19	-0.310692	0.179571	0.08359614	0.0162529
ENSG00000180611	MB21D2	3	192575277	A	G	0.0795229	0.0102773	0.0301369	0.7199992	-0.392072	0.0189471	4.01E-95	-0.0262128	0.0768761	0.733123	0.1604357
ENSG00000180626	ZNF594	17	5089004	G	C	0.101392	0.0299224	0.0267537	0.1800002	-0.124053	0.0145629	1.62E-17	-0.241207	0.217515	0.2674637	0.7183623
ENSG00000180644	PRF1	10	72359817	G	A	0.0149105	-0.0860992	0.0636838	0.1100001	-0.464636	0.0389626	8.75E-33	0.185304	0.13794	0.1791509	0.3766546
ENSG00000180667	YOD1	1	207221759	G	A	0.0109344	-0.0618347	0.0650477	0.2700001	0.369344	0.0538952	7.23E-12	-0.167418	0.177803	0.3464027	0.4313897
ENSG00000180694	TMEM64	8	91719041	G	A	0.0357853	-0.0183089	0.0376568	0.56	0.377756	0.0212008	5.12E-71	-0.0484676	0.0997227	0.6269505	0.08789654
ENSG00000180712	LINC02363	4	185268519	C	T	0.122266	-0.0364634	0.0226066	0.04799986	0.293952	0.0145363	6.27E-91	-0.124045	0.07715	0.1078686	0.546073
ENSG00000180730	SHISA2	13	26621966	A	G	0.459245	-0.00546145	0.0143549	0.5500004	0.14236	0.0080306	2.59E-70	-0.0383637	0.100858	0.7036698	0.26769
ENSG00000180739	S1PR5	19	10626115	C	T	0.351889	0.00481238	0.0149474	0.81	0.176582	0.00894593	1.00E-86	0.027253	0.0846598	0.7475197	0.4132579
ENSG00000180747	SMG1P3	16	21494884	A	G	0.0109344	0.0782804	0.210419	0.9	-0.572207	0.0790341	4.49E-13	-0.136804	0.368218	0.7102418	0.4027113
ENSG00000180758	GPR157	1	9174796	G	C	0.417495	0.00973551	0.0145061	0.4700002	0.0576834	0.00805274	7.88E-13	0.168775	0.252579	0.5040023	0.1981664
ENSG00000180767	CHST13	3	126252630	C	T	0.38668	0.0125277	0.0146958	0.3900004	-0.832239	0.00666679	0	-0.015053	0.0176586	0.3939654	0.8136022
ENSG00000180771	NA	11	94802222	T	G	0.215706	-0.00822625	0.0170101	0.7099994	0.151151	0.0104574	2.37E-47	-0.0544239	0.1126	0.6288549	0.8639732
ENSG00000180773	SLC36A4	11	92904235	G	A	0.05666	0.0137508	0.0293984	0.5199996	0.32499	0.0186224	3.35E-68	0.0423115	0.0904919	0.6400908	0.3906405
ENSG00000180776	ZDHHC20	13	21991886	A	G	0.445328	0.0047852	0.0143438	0.7600007	-0.225579	0.00797614	5.80E-176	-0.021213	0.0635911	0.7386932	0.6409837
ENSG00000180787	ZFP3	17	4990606	G	A	0.474155	-0.0096681	0.0141905	0.4700002	-0.060269	0.00794037	3.19E-14	0.160416	0.236399	0.4974042	0.6046087
ENSG00000180817	PPA1	10	71978127	G	T	0.365805	0.00598686	0.0151782	0.5500004	0.675295	0.0113135	0	0.00886554	0.0224769	0.693264	0.1290522
ENSG00000180822	PSMG4	6	3267622	G	A	0.156064	0.0143871	0.0187834	0.6899999	-0.414099	0.0141646	7.01E-188	-0.0347431	0.0453752	0.4438638	0.9311406
ENSG00000180834	MAP6D1	3	183538523	G	A	0.470179	-0.0117125	0.0142432	0.2700001	-0.486322	0.00748853	0	0.0240838	0.0292899	0.410931	0.553064
ENSG00000180846	CSNK1G2-AS1	19	1953557	T	C	0.384692	-0.0161337	0.0146161	0.2099999	0.0696944	0.0114118	1.01E-09	-0.231492	0.213115	0.2773768	0.8212709
ENSG00000180855	ZNF443	19	12546223	A	G	0.210736	0.00925158	0.0176444	0.4700002	-0.0759887	0.0114145	2.79E-11	-0.121749	0.232917	0.6011716	0.8538828

ENSG00000180871	CXCR2	2	218995994	G	A	0.494036	0.00952305	0.0141778	0.6100002	0.264182	0.00855782	2.99E-209	0.0360473	0.0536794	0.5018839	0.8712833
ENSG00000180875	GREM2	1	240714161	T	G	0.516899	0.000361634	0.0142092	0.9	-0.130515	0.0079226	5.66E-61	-0.00277082	0.10887	0.9796955	0.6705768
ENSG00000180878	C11orf42	11	6229579	C	G	0.265408	0.0159852	0.0158097	0.3800004	0.088217	0.0139126	2.29E-10	0.181203	0.181478	0.3180437	0.9890393
ENSG00000180881	CAPS2	12	75727233	A	G	0.380716	0.00410896	0.0144488	0.7499995	0.137764	0.00824803	1.25E-62	0.0298262	0.104896	0.7761502	0.7003214
ENSG00000180884	ZNF792	19	35451105	A	G	0.512922	0.00136472	0.014236	0.9699999	0.223541	0.00862508	4.22E-148	0.006105	0.0636844	0.9236291	0.6181635
ENSG00000180891	CUEDC1	17	55985644	G	A	0.371769	0.00907478	0.0146	0.5	-0.164889	0.00815572	6.85E-91	-0.0550357	0.0885862	0.5344241	0.2149361
ENSG00000180900	SCRIB	8	144885319	G	T	0.00695825	0.0119953	0.0828899	0.8600001	-0.839695	0.0660397	4.88E-37	-0.0142853	0.0987207	0.8849444	0.2508546
ENSG00000180901	KCTD2	17	73045327	T	C	0.356859	-0.000945896	0.0152743	0.84	-0.39808	0.00833015	0	0.00237614	0.0383699	0.9506208	0.3156001
ENSG00000180902	D2HGDH	2	242691112	C	A	0.343936	-0.0342126	0.0149796	0.03500016	-0.194614	0.00889437	3.98E-106	0.175797	0.077389	0.02311042	0.007574824
ENSG00000180914	OXTR	3	8801704	G	A	0.264414	6.74E-05	0.0153909	0.98	0.0787972	0.00860983	5.59E-20	0.00085586	0.195323	0.9965039	0.4654724
ENSG00000180917	CMTR2	16	71319455	T	C	0.203777	0.0145015	0.0180604	0.3700002	0.528751	0.0106758	0	0.0274259	0.0341612	0.4220678	0.1364424
ENSG00000180921	FAM83H	8	144811037	A	G	0.259443	-0.0139183	0.0172177	0.4199997	0.149852	0.010274	3.47E-48	-0.0928804	0.115074	0.4195899	0.4681186
ENSG00000180953	ST20	15	80203613	G	C	0.366799	0.00265274	0.0147031	0.8200001	-0.654585	0.0134622	0	-0.00405255	0.0224619	0.8568235	0.228313
ENSG00000180957	PITPNB	22	28281889	T	C	0.05666	-0.0341048	0.0313809	0.2300001	0.389781	0.02555	1.51E-52	-0.0874974	0.0807132	0.2783409	0.3625192
ENSG00000180979	LRRC57	15	42837860	A	T	0.083499	-4.47E-05	0.0245875	0.9699999	0.449148	0.013607	6.13E-239	-9.95E-05	0.0547425	0.9985492	0.882176
ENSG00000180992	MRPL14	6	44088194	G	C	0.50497	-0.00713115	0.0141898	0.7199992	0.30704	0.00779735	0	-0.0232255	0.0462186	0.6153062	0.1451226
ENSG00000180999	C1orf105	1	172413899	C	T	0.397614	-0.00245246	0.0143051	0.9400001	0.175646	0.00808303	1.06E-104	-0.0139625	0.0814454	0.8638823	0.9752947
ENSG00000181004	BBS12	4	123659977	A	G	0.213718	0.0266891	0.0167125	0.14	0.211327	0.00945956	1.51E-110	0.126293	0.0792856	0.1111849	0.574121
ENSG00000181007	ZFP82	19	36891790	A	G	0.198807	0.0127398	0.0170051	0.3900004	-0.566569	0.00917848	0	-0.0224859	0.0300164	0.4537848	0.4074474
ENSG00000181016	LSMEM1	7	112126012	A	G	0.250497	0.0235705	0.0168607	0.1299999	0.371416	0.00988613	6.795831e-309	0.0634612	0.0454272	0.1624171	0.9088296
ENSG00000181019	NQO1	16	69750876	A	G	0.106362	0.00351563	0.0216328	0.7300002	0.363781	0.0120637	9.22E-200	0.00966413	0.0594674	0.8709031	0.1635412
ENSG00000181026	AEN	15	89170020	A	G	0.0298211	-0.0494839	0.0483023	0.2599998	0.788593	0.0222887	3.36E-274	-0.0627496	0.0612769	0.3058195	0.3766396
ENSG00000181027	FKRP	19	47264774	G	T	0.427435	-0.00232247	0.014421	0.8	0.372902	0.00785925	0	-0.00622809	0.0386725	0.8720565	0.8581484
ENSG00000181031	RPH3AL	17	149169	G	A	0.319085	-0.000784175	0.0152234	0.8600001	0.394095	0.0131215	3.51E-198	-0.00198981	0.0386288	0.9589183	0.8190702
ENSG00000181035	SLC25A42	19	19199252	T	C	0.0785288	0.0127097	0.0251542	0.4799997	-0.125328	0.0145356	6.57E-18	-0.101411	0.201051	0.613976	0.6180923
ENSG00000181036	FCRL6	1	159778171	C	T	0.201789	0.000519404	0.0179993	0.95	-0.578757	0.00977566	0	-0.000897447	0.0310999	0.9769787	0.1850708
ENSG00000181038	METTL23	17	74726465	G	T	0.084493	-0.0138442	0.0283326	0.4600002	-0.108365	0.0174697	5.54E-10	0.127756	0.262266	0.6261716	0.3561231
ENSG00000181039	NA	1	145473077	T	C	0.148111	-0.0262038	0.0214894	0.16	-0.22763	0.0221124	7.48E-25	0.115116	0.0950651	0.2259273	0.6400941
ENSG00000181045	SLC26A11	17	78210398	A	G	0.312127	0.0188494	0.0150207	0.1800002	0.105562	0.00902811	1.39E-31	0.178562	0.14311	0.2121301	0.5415753
ENSG00000181061	HIGD1A	3	42822346	T	C	0.214712	-0.00478735	0.0170708	0.7800007	-0.113239	0.0103247	5.46E-28	0.0422764	0.150799	0.7792096	0.05719102
ENSG00000181074	OR52N4	11	5776441	T	C	0.0437376	0.00833239	0.0369884	0.8200001	0.419033	0.0215532	3.42E-84	0.0198848	0.0882768	0.8217807	0.4392746
ENSG00000181090	EHMT1	9	140638956	T	C	0.129225	-0.00229624	0.0212067	0.81	0.308161	0.0110049	1.53E-172	-0.00745142	0.0688174	0.913775	0.1391807
ENSG00000181097	NA	8	144779453	T	C	0.167992	0.00063847	0.0192714	0.9299999	-0.42277	0.0130337	8.32E-231	-0.00151021	0.0455837	0.9735706	0.3489789
ENSG00000181104	F2R	5	76021737	A	G	0.210736	-0.0212781	0.0167048	0.1199999	-0.387338	0.00902879	0	0.0549342	0.0431462	0.2029434	0.9673474
ENSG00000181126	NA	6	29762159	A	G	0.0318091	-0.00606373	0.0357027	0.6999999	0.658908	0.0384355	7.07E-66	-0.0092027	0.0541873	0.8651429	0.9967155
ENSG00000181135	ZNF707	8	144781345	C	T	0.2167	-0.00291695	0.0173188	0.6100002	0.274623	0.00923766	3.28E-194	-0.0106217	0.063065	0.8662497	0.2353242
ENSG00000181143	MUC16	19	9025769	C	G	0.0705765	0.00169883	0.0281644	0.83	-0.18655	0.0196059	1.82E-21	-0.00910655	0.150978	0.9519031	0.3494218
ENSG00000181192	DHTKD1	10	12138097	G	A	0.457256	0.00564851	0.0143499	0.8600001	0.228025	0.00789538	2.08E-183	0.0247715	0.0629371	0.6938833	0.2004812
ENSG00000181215	C4orf50	4	5975195	C	T	0.207753	-0.0106215	0.0180872	0.5300002	0.103955	0.0111282	9.49E-21	-0.102174	0.174334	0.5578207	0.5882017
ENSG00000181218	H2AC25	1	228645312	A	T	0.204771	0.0152569	0.0182473	0.5099998	-0.275072	0.0109313	1.00E-139	-0.0554651	0.0663731	0.4033479	0.2435716
ENSG00000181220	ZNF746	7	149182396	T	C	0.260437	0.029483	0.0162527	0.05800027	-0.254597	0.00908124	6.00E-173	-0.115803	0.0639705	0.0702568	0.8553686
ENSG00000181222	POLR2A	17	7402809	A	G	0.347913	-0.0189743	0.0147712	0.2200002	-0.0734803	0.00831564	9.89E-19	0.258223	0.203135	0.2036626	0.9242826
ENSG00000181234	TMEM132C	12	128972204	A	G	0.154076	0.0153766	0.0185661	0.4100001	-0.0969072	0.0118584	3.03E-16	-0.158673	0.192568	0.4099466	0.647027
ENSG00000181240	SLC25A41	19	6429919	T	C	0.488072	-0.00374847	0.0144335	0.5500004	-0.234694	0.00871385	8.90E-160	0.0159717	0.061502	0.7950997	0.1015315
ENSG00000181264	TLCD5	11	120200114	A	G	0.389662	0.0209471	0.0145439	0.14	0.0472957	0.00836441	1.56E-08	0.442896	0.317329	0.1628043	0.7442293
ENSG00000181274	FRAT2	10	99093356	G	A	0.33499	-0.0109549	0.0151581	0.59	0.549585	0.00791762	0	-0.019933	0.0275825	0.4698826	0.8809091
ENSG00000181291	TMEM132E	17	32937052	G	A	0.502982	-0.00193878	0.0142602	0.99	-0.0447121	0.00809093	3.27E-08	0.0433614	0.31903	0.8918875	0.8643735
ENSG00000181315	ZNF322	6	26648249	C	G	0.380716	0.00612404	0.0143261	0.4100001	-0.420286	0.0186454	1.65E-112	-0.0145711	0.0340927	0.6690898	0.492422
ENSG00000181350	LRRC75A	17	16370179	T	C	0.124254	-0.0151682	0.0204235	0.25	-0.268045	0.0133786	2.71E-89	0.0565883	0.0762467	0.457982	0.6692987
ENSG00000181381	DDX60L	4	169368411	G	A	0.324056	-0.0195848	0.0152329	0.2	0.467256	0.00995309	0	-0.0419145	0.032613	0.1987193	0.1597086

ENSG00000181396	OGFOD3	17	80361806	G	T	0.333002	0.00490388	0.0155194	0.7700005	-0.242972	0.0106667	7.47E-115	-0.0201829	0.0638793	0.7520378	0.2813883
ENSG00000181404	WASHC1	9	22125	T	C	0.435388	-0.0146226	0.0156876	0.32	-0.806497	0.0180488	0	0.018131	0.0194558	0.3513834	0.6163741
ENSG00000181409	AATK	17	79115486	T	G	0.390656	0.00697705	0.0144496	0.64	-0.530118	0.00991192	0	-0.0131613	0.0272584	0.6292132	0.7755964
ENSG00000181444	ZNF467	7	149465919	T	C	0.156064	-0.0024519	0.0206076	0.7800007	-0.684657	0.0112618	0	0.00358121	0.0300992	0.9052911	0.3176836
ENSG00000181450	ZNF678	1	227799415	G	A	0.397614	0.00946189	0.0146326	0.3900004	-0.0492114	0.00822245	2.16E-09	-0.19227	0.299072	0.5202958	0.7197575
ENSG00000181458	TMEM45A	3	100253875	A	C	0.166004	-0.0321948	0.0190922	0.04	0.3672	0.0101239	4.72E-288	-0.0876765	0.0520502	0.09209265	0.4723742
ENSG00000181467	RAP2B	3	152883147	G	A	0.137177	0.0498107	0.0198763	0.01099993	-0.0721039	0.011375	2.32E-10	-0.690818	0.296423	0.01977899	0.005980837
ENSG00000181472	ZBTB2	6	151698967	A	T	0.0387674	-0.0240371	0.0356721	0.4	-0.181724	0.0216416	4.58E-17	0.132272	0.196929	0.5017903	0.8561607
ENSG00000181481	RNF135	17	29311366	G	C	0.0119284	-0.0686112	0.0910065	0.33	-0.656912	0.0419345	2.62E-55	0.104445	0.138697	0.4514236	0.0804419
ENSG00000181513	ACBD4	17	43215757	G	T	0.517893	0.00925871	0.0141962	0.5	-0.126841	0.00857045	1.47E-49	-0.0729947	0.11203	0.5146825	0.5067634
ENSG00000181523	SGSH	17	78187618	A	G	0.055666	-0.0166503	0.0358535	0.59	0.666577	0.0298734	2.74E-110	-0.0249788	0.0537992	0.6424345	0.85382
ENSG00000181555	SETD2	3	47131688	T	A	0.394632	0.0266496	0.0144316	0.08400014	-0.103556	0.00815223	5.71E-37	-0.257344	0.140825	0.06763869	0.5459633
ENSG00000181585	TMIE	3	46747599	G	T	0.390656	-0.00747943	0.0148884	0.4400003	0.124728	0.00826814	2.02E-51	-0.0599659	0.119433	0.6156057	0.9204599
ENSG00000181610	MRPS23	17	55922129	G	T	0.275348	-0.0228528	0.0157561	0.2200002	0.0566705	0.00881976	1.32E-10	-0.403257	0.285025	0.1571236	0.8127324
ENSG00000181619	GPR135	14	59913900	C	G	0.469185	0.00880746	0.0142625	0.5999997	0.135019	0.00812675	5.50E-62	0.0652311	0.105706	0.5371691	0.6468845
ENSG00000181631	P2RY13	3	151045718	A	G	0.172962	0.0141839	0.0181878	0.4500005	0.457363	0.010018	0	0.0310124	0.0397725	0.4355415	0.3796517
ENSG00000181649	PHLDA2	11	2950094	G	A	0.430417	-0.00164732	0.0142955	0.89	-0.172348	0.0112207	3.05E-53	0.00955811	0.082948	0.9082626	0.01296453
ENSG00000181652	ATG9B	7	150715441	G	C	0.269384	0.00470606	0.0166775	0.7899998	-0.0611223	0.0089045	6.69E-12	-0.0769942	0.273085	0.7779879	0.6020131
ENSG00000181666	ZNF875	19	37832003	A	G	0.407555	0.0204488	0.0143358	0.0990011	-0.265135	0.00801004	2.94E-240	-0.0771261	0.0541201	0.1541305	0.3218928
ENSG00000181722	ZBTB20	3	114461529	G	C	0.0228628	0.0591918	0.0434337	0.2399999	-0.386709	0.046982	1.86E-16	-0.153065	0.113845	0.1787855	0.3634709
ENSG00000181744	DIPK2A	3	143729100	A	G	0.485089	0.00942404	0.0143294	0.7099994	0.172355	0.00796029	5.85E-104	0.0546782	0.0831774	0.510944	0.228047
ENSG00000181751	MACIR	5	102604382	A	T	0.00795229	0.0863611	0.0577142	0.14	0.503426	0.0426943	4.32E-32	0.171547	0.115562	0.1376889	0.4043896
ENSG00000181754	AMIGO1	1	110049578	T	C	0.542744	0.0138292	0.0141933	0.3100002	-0.234559	0.00784449	1.90E-196	-0.0589583	0.0605427	0.3301409	0.7501741
ENSG00000181773	GPR3	1	27720733	T	G	0.132207	-0.0123702	0.0252414	0.3800004	-0.0827778	0.0132904	4.71E-10	0.149439	0.305872	0.6251492	0.5599721
ENSG00000181778	TMEM252	9	71153639	T	A	0.274354	-0.0100414	0.0172717	0.5999997	0.283826	0.0101616	1.11E-171	-0.0353787	0.0608663	0.56107	0.2302058
ENSG00000181788	SIAH2	3	150470089	G	A	0.0228628	0.100784	0.0467044	0.04499974	-0.460799	0.0254703	3.71E-73	-0.218716	0.102074	0.03213528	0.7022316
ENSG00000181789	COPG1	3	128982531	G	A	0.183897	-0.0125623	0.0171608	0.6300007	0.139327	0.0109348	3.47E-37	-0.0901642	0.123372	0.4648833	0.5479648
ENSG00000181790	ADGRB1	8	143578580	T	G	0.128231	0.0206195	0.0212428	0.2999998	-0.204067	0.0132955	3.62E-53	-0.101043	0.104305	0.3326834	0.1065467
ENSG00000181804	SLC9A9	3	143275718	T	C	0.485089	0.0101941	0.014331	0.6700003	0.121782	0.00796438	8.81E-53	0.083708	0.117805	0.4773547	0.2250333
ENSG00000181817	LSM10	1	36860166	A	G	0.359841	-0.0285181	0.0148908	0.0329997	-0.100346	0.00831267	1.50E-33	0.284199	0.150251	0.05855869	0.7846423
ENSG00000181826	RELL1	4	37640210	G	A	0.026839	-0.00830176	0.0387799	0.98	0.200631	0.0266989	5.71E-14	-0.0413782	0.193368	0.8305572	0.4529785
ENSG00000181827	RFX7	15	56457480	C	T	0.473161	0.0232404	0.0142738	0.1199999	0.230221	0.00866594	1.67E-155	0.100948	0.0621167	0.1041335	0.2373504
ENSG00000181830	SLC35C1	11	45830094	G	A	0.253479	0.0108672	0.0175186	0.58	0.318418	0.00956295	4.27E-243	0.0341288	0.0550272	0.5351156	0.7746339
ENSG00000181847	TIGIT	3	114012447	G	A	0.26839	-0.0208791	0.0170923	0.2700001	0.327437	0.00940763	2.01E-265	-0.0637652	0.0522324	0.2221624	0.3711295
ENSG00000181852	RNF41	12	56607001	C	T	0.114314	-0.00360666	0.0217923	0.8600001	-0.114737	0.0128089	3.32E-19	0.0314342	0.189965	0.8685714	0.6726143
ENSG00000181856	SLC2A4	17	7188281	C	A	0.432406	-0.00719417	0.0143523	0.7700005	0.0476748	0.00808376	3.69E-09	-0.150901	0.302132	0.617459	0.9876454
ENSG00000181873	IBA57	1	228361737	T	C	0.338966	-0.00240638	0.014712	0.95	0.159592	0.0091526	4.34E-68	-0.0150783	0.0921891	0.8700786	0.1786569
ENSG00000181885	CLDN7	17	7165262	A	G	0.172962	0.0223763	0.0190704	0.29	-0.154479	0.0106389	9.02E-48	-0.14485	0.123852	0.2421861	0.5666134
ENSG00000181894	ZNF329	19	58652048	A	G	0.170974	-0.0156394	0.0173009	0.64	0.295716	0.0097232	3.65E-203	-0.0528866	0.058531	0.3662258	0.1963711
ENSG00000181896	ZNF101	19	19786961	A	G	0.201789	-0.0113143	0.0176492	0.5999997	-0.172702	0.0101678	1.06E-64	0.0655133	0.102267	0.5217762	0.009665946
ENSG00000181904	C5orf24	5	134188398	T	C	0.148111	0.0369861	0.0224664	0.06199976	0.0680968	0.012202	2.39E-08	0.54314	0.343974	0.1143325	0.428638
ENSG00000181908	LINC02724	11	64217836	T	C	0.43837	0.0101381	0.0143607	0.56	-0.120376	0.0094665	4.82E-37	-0.0842203	0.119482	0.4808874	0.1269258
ENSG00000181915	ADO	10	64566377	T	C	0.388668	-0.00756464	0.0146969	0.3800004	0.216732	0.00802858	1.69E-160	-0.0349032	0.0678237	0.6068211	0.9367056
ENSG00000181924	COA4	11	73585872	T	C	0.0308151	0.0561518	0.0399059	0.1900002	-0.225554	0.0296439	2.77E-14	-0.24895	0.179924	0.1664675	0.5941757
ENSG00000181938	GINS3	16	58384516	T	C	0.0417495	-0.02716	0.036819	0.28	-0.2172	0.0199669	1.47E-27	0.125046	0.169906	0.4617475	0.8431843
ENSG00000181963	OR52K2	11	4471058	A	G	0.309145	-0.00546182	0.015382	0.7400005	-0.310912	0.00876326	1.04E-275	0.0175671	0.0494763	0.722544	0.3034671
ENSG00000181982	CCDC149	4	24894782	C	T	0.121272	-0.0210646	0.0201077	0.35	0.304126	0.0114429	1.23E-155	-0.0692628	0.0661678	0.2952029	0.1159731
ENSG00000181991	MRPS11	15	89016453	C	T	0.0149105	-0.0613145	0.065443	0.3599996	-0.482548	0.0383723	2.88E-36	0.127064	0.135996	0.3501363	0.03039791
ENSG00000182004	SNRPE	1	203835204	T	C	0.109344	-0.0199734	0.0216137	0.35	0.305253	0.0259295	5.42E-32	-0.0654323	0.0710237	0.3569072	0.09704305
ENSG00000182010	RTKN2	10	63985630	C	G	0.247515	-0.0112947	0.0164621	0.35	0.471244	0.00865374	0	-0.0239679	0.0349361	0.4926823	0.6380322

ENSG00000182013	PNMA8A	19	46972284	T	C	0.247515	0.00407016	0.0163504	0.84	0.511284	0.00974525	0	0.00796067	0.0319795	0.8034145	0.3247604
ENSG00000182022	CHST15	10	125810195	T	A	0.248509	-0.0233598	0.0160894	0.1199999	0.199107	0.0100317	1.15E-87	-0.117323	0.0810236	0.1476153	0.5821132
ENSG00000182048	NA	11	3644960	A	G	0.115308	0.00284958	0.0222286	0.98	0.357816	0.0139999	4.43E-144	0.00796381	0.0621238	0.8979965	0.1095249
ENSG00000182054	IDH2	15	90636006	A	G	0.00994036	0.0464241	0.0952817	0.7199992	-1.11199	0.0761891	3.01E-48	-0.0417485	0.0857331	0.6262876	0.9346703
ENSG00000182057	NA	22	42668480	T	C	0.457256	-0.0101652	0.0142528	0.3700002	-0.0808879	0.00949434	1.60E-17	0.12567	0.176821	0.477257	0.01761901
ENSG00000182087	TMEM259	19	1013642	A	G	0.172962	-0.0321285	0.0179385	0.08	0.237927	0.0148721	1.32E-57	-0.135035	0.0758659	0.0750896	0.1325927
ENSG00000182093	GET1	21	40776312	T	G	0.308151	0.00839606	0.0151524	0.4100001	0.750005	0.00759661	0	0.0111947	0.0202034	0.5795112	0.8259056
ENSG00000182107	TMEM30B	14	61746323	A	G	0.183897	-0.00420686	0.0193738	0.7700005	0.10494	0.0119536	1.65E-18	-0.0400881	0.184674	0.8281497	0.4669104
ENSG00000182108	DEXI	16	11029532	A	G	0.395626	0.0175716	0.0144066	0.2700001	0.336727	0.0086195	0	0.0521835	0.0428051	0.2228074	0.3401362
ENSG00000182109	NA	1	39999905	A	G	0.0397614	0.0068256	0.0349658	0.84	-0.580377	0.0228274	1.35E-142	-0.0117606	0.0602485	0.8452347	0.1103618
ENSG00000182117	NOP10	15	34634647	A	G	0.168986	0.00771478	0.0193173	0.6600001	0.828256	0.00999604	0	0.00931448	0.0233231	0.6896229	0.2904614
ENSG00000182118	FAM89A	1	231165348	G	T	0.316103	0.0251789	0.0148937	0.1100001	0.404121	0.00806523	0	0.0623054	0.0368756	0.09110212	0.4340684
ENSG00000182134	TDRKH	1	151753237	T	C	0.486083	0.0125203	0.0142004	0.3100002	0.424067	0.00757971	0	0.0295244	0.0334904	0.3780056	0.7119117
ENSG00000182141	ZNF708	19	21493094	C	A	0.333996	0.00532548	0.0152271	0.89	-0.258684	0.0091063	1.65E-177	-0.0205868	0.0588683	0.726556	0.1492997
ENSG00000182149	IST1	16	71941024	A	G	0.282306	-0.0276511	0.0159068	0.07900053	-0.222533	0.0174541	3.13E-37	0.124256	0.0721419	0.08499996	0.6971857
ENSG00000182150	ERCC6L2	9	98707412	T	C	0.104374	0.013548	0.0252359	0.7400005	0.200347	0.0143017	1.38E-44	0.0676226	0.126053	0.5916404	0.278096
ENSG00000182154	MRPL41	9	140446329	G	C	0.317097	0.025925	0.0149424	0.0649995	0.0957133	0.00866137	2.18E-28	0.270861	0.158029	0.08652893	0.1023913
ENSG00000182158	CREB3L2	7	137623269	T	C	0.373757	-0.0271573	0.0146281	0.08500021	0.0650047	0.00814493	1.45E-15	-0.417774	0.231039	0.07056948	0.290673
ENSG00000182165	TP53TG1	7	86964686	C	T	0.258449	0.0179883	0.0157974	0.1299999	-0.293049	0.0096371	4.25E-203	-0.0613833	0.0539448	0.2551665	0.4764739
ENSG00000182173	TSEN54	17	73516480	T	C	0.106362	-0.00189467	0.0245807	0.81	0.417116	0.0138476	2.50E-199	-0.00454231	0.0589303	0.9385604	0.8253025
ENSG00000182175	RGMA	15	93609534	A	G	0.337972	-0.00373	0.0152118	0.8700001	-0.16748	0.00884116	5.02E-80	0.0222713	0.0908352	0.8063141	0.8002561
ENSG00000182179	UBA7	3	49847009	T	C	0.474155	0.0238187	0.0141836	0.07299952	-0.582424	0.00732751	0	-0.0408958	0.0243581	0.09316412	0.1142711
ENSG00000182180	MRPS16	10	75009480	A	G	0.0646123	0.0446034	0.0286908	0.1499999	0.357878	0.0153979	1.71E-119	0.124633	0.0803483	0.1208639	0.9141531
ENSG00000182183	SHISAL2A	1	53117185	C	T	0.124254	-0.0213015	0.0191905	0.35	0.45453	0.0108554	0	-0.0468649	0.0422353	0.267166	0.1205306
ENSG00000182185	RAD51B	14	68741715	T	C	0.0208748	0.0433585	0.0434118	0.14	0.175893	0.0286153	7.91E-10	0.246505	0.250045	0.3242102	0.06435317
ENSG00000182196	ARL6IP4	12	123465894	A	G	0.308151	-0.00650202	0.0156786	0.5500004	-0.201654	0.00866629	9.18E-120	0.0322434	0.0777623	0.6784049	0.9435532
ENSG00000182197	EXT1	8	118965410	C	T	0.209742	-0.00105081	0.0183835	0.95	0.320572	0.00993722	2.57E-228	-0.00327792	0.0573461	0.9544174	0.554591
ENSG00000182199	SHMT2	12	57625914	C	T	0.340954	0.0108179	0.0151406	0.6499995	0.0823941	0.00840876	1.14E-22	0.131295	0.184246	0.4760907	0.3055305
ENSG00000182208	MOB2	11	1506582	C	T	0.121272	-0.0343607	0.0224483	0.09800089	-0.144321	0.0134965	1.10E-26	0.238086	0.15713	0.129718	0.02687248
ENSG00000182218	HHIPL1	14	100129176	A	G	0.236581	0.00513512	0.0161093	0.4700002	0.0680039	0.00985364	5.15E-12	0.0755121	0.23714	0.7501604	0.8437337
ENSG00000182224	CYB5D1	17	7763332	T	C	0.171968	0.00669047	0.0179436	0.5500004	-0.456979	0.00970596	0	-0.0146406	0.0392669	0.7092605	0.9958582
ENSG00000182230	NA	5	175523476	G	A	0.480119	-0.0100299	0.0144626	0.56	-0.585325	0.0122837	0	0.0171356	0.0247113	0.4880381	0.1624886
ENSG00000182240	BACE2	21	42594126	A	C	0.32505	0.00657874	0.0156742	0.7700005	-0.215804	0.00855725	2.49E-140	-0.0304848	0.0726418	0.6747341	0.3775939
ENSG00000182247	UBE2E2	3	23438897	C	T	0.413519	-0.00846714	0.0143376	0.5999997	-0.199048	0.00794648	1.81E-138	0.0425381	0.0720508	0.5549286	0.9874467
ENSG00000182253	SYNM	15	99657109	G	T	0.337972	-0.0255185	0.0151889	0.05099998	-0.681452	0.00746492	0	0.0374472	0.0222928	0.09299796	0.6874356
ENSG00000182257	NA	22	46447691	G	A	0.425447	0.00693486	0.0141411	0.4799997	-0.145709	0.00954658	1.35E-52	-0.0475938	0.0971001	0.6240266	0.766735
ENSG00000182272	B4GALNT4	11	375956	T	C	0.295229	-0.0034509	0.0158856	0.9599999	0.185995	0.0111485	1.73E-62	-0.0185538	0.0854161	0.82804	0.547715
ENSG00000182307	C8orf33	8	146279590	G	A	0.207753	-0.0059534	0.0180911	0.81	0.394372	0.0109274	3.22E-285	-0.0150959	0.0458751	0.7421064	0.7391085
ENSG00000182308	DCAF4L1	4	41986094	G	A	0.0954274	0.050563	0.0232168	0.02399993	-0.136043	0.0151136	2.23E-19	-0.371669	0.175582	0.03427793	0.6618547
ENSG00000182310	SPACA6	19	52206116	A	G	0.181909	0.0178232	0.0173546	0.2099999	0.165827	0.0108493	9.69E-53	0.107481	0.104891	0.305509	0.4226437
ENSG00000182318	ZSCAN22	19	58846041	G	C	0.332008	0.00146139	0.0151525	0.9199999	-0.0591868	0.00927954	1.79E-10	-0.0246912	0.256041	0.9231755	0.6942087
ENSG00000182319	NA	8	8209633	G	A	0.315109	0.0130095	0.0149991	0.4799997	0.150958	0.00912031	1.55E-61	0.0861799	0.0994961	0.3864004	0.6310785
ENSG00000182324	KCNJ14	19	48964066	A	G	0.00894632	0.0379204	0.0549343	0.8200001	-0.228115	0.0408209	2.29E-08	-0.166234	0.242649	0.4932938	0.8044186
ENSG00000182325	FBXL6	8	145581063	A	G	0.0606362	-0.0202798	0.0261021	0.5199996	-0.153191	0.0263125	5.81E-09	0.132382	0.171899	0.4412321	0.7087353
ENSG00000182326	C1S	12	7137343	T	C	0.0258449	-0.0192393	0.0559197	0.6700003	-0.161102	0.0290933	3.07E-08	0.119423	0.347778	0.731305	0.9942276
ENSG00000182359	KBTBD3	11	105935158	T	C	0.189861	0.00650225	0.0183095	0.7600007	-0.183812	0.010669	1.62E-66	-0.0353744	0.0996309	0.7225488	0.655445
ENSG00000182362	YBEY	21	47711958	C	G	0.214712	0.0484855	0.0180002	0.01400006	0.775379	0.00919112	0	0.0625313	0.0232265	0.00709745	0.04541669
ENSG00000182372	CLN8	8	1719341	A	C	0.311133	0.00821875	0.0157748	0.7700005	-0.268362	0.00894618	1.06E-197	-0.0306256	0.0587906	0.602418	0.5009197
ENSG00000182389	CACNB4	2	152822441	A	G	0.166998	-0.0350061	0.0192277	0.06199976	0.117486	0.00980599	4.47E-33	-0.297961	0.165539	0.07186934	0.7527228
ENSG00000182393	IFNL1	19	39788139	G	A	0.112326	0.00243846	0.0230942	1	-0.397192	0.0154179	2.38E-146	-0.00613925	0.0581442	0.9159103	0.383204

ENSG00000182397	NA	15	100338746	T	C	0.291252	-0.0217533	0.0159024	0.1299999	0.572765	0.020102	1.43E-178	-0.0379795	0.0277962	0.1718286	0.7922326
ENSG00000182400	TRAPPC6B	14	39628375	C	T	0.0656064	0.0212236	0.0281848	0.3900004	-0.265936	0.0172598	1.45E-53	-0.0798071	0.10611	0.45198	0.4551691
ENSG00000182405	PGBD4	15	34395432	G	A	0.111332	0.0276726	0.0236986	0.17	0.192059	0.0126016	1.90E-52	0.144084	0.123754	0.2443117	0.6136411
ENSG00000182446	NPLOC4	17	79569704	G	A	0.158052	0.00519637	0.0190983	0.8600001	0.28795	0.013914	3.84E-95	0.0180461	0.0663307	0.7855744	0.8079693
ENSG00000182463	TSHZ2	20	51848214	T	C	0.345924	-0.0194503	0.0148887	0.29	-0.182647	0.00938569	2.39E-84	0.106491	0.0816995	0.1924224	0.1848485
ENSG00000182472	CAPN12	19	39240685	C	T	0.291252	0.0207267	0.0165372	0.3100002	-0.618384	0.00866675	0	-0.0335175	0.0267467	0.2101531	0.3683708
ENSG00000182473	EXOC7	17	74097377	A	T	0.379722	-0.0114673	0.0146712	0.32	0.179798	0.00874863	7.45E-94	-0.0637787	0.0816571	0.4347701	0.6513938
ENSG00000182481	KPNA2	17	66037296	C	T	0.244533	-0.0013513	0.0169659	1	-0.078756	0.0108146	3.28E-13	0.017158	0.215436	0.936521	0.3978487
ENSG00000182487	NA	7	72642295	G	A	0.0109344	-0.0317989	0.0750339	0.6100002	-0.62934	0.0978513	1.26E-10	0.0505274	0.119485	0.6723852	NA
ENSG00000182500	NA	12	122072519	A	C	0.163022	0.00994088	0.018966	0.5400003	0.522817	0.0101016	0	0.0190141	0.0362784	0.6001984	0.1628077
ENSG00000182502	NA	22	22470805	A	G	0.475149	0.00850215	0.0142967	0.4100001	0.363363	0.0187044	4.60E-84	0.0233985	0.0393639	0.5522348	0.4513379
ENSG00000182504	CEP97	3	101466087	G	T	0.371769	-0.0136928	0.014849	0.2	-0.125974	0.00816071	9.29E-54	0.108696	0.118084	0.357314	0.3808586
ENSG00000182511	FES	15	91432965	T	C	0.323062	-0.0346538	0.015225	0.007299952	-0.386948	0.00825084	0	0.0895567	0.0393927	0.02299994	0.02224656
ENSG00000182512	GLRX5	14	96005450	C	T	0.423459	-0.0133332	0.0143721	0.4100001	0.265604	0.00790966	3.32E-247	-0.0501995	0.0541317	0.3537395	0.3078301
ENSG00000182534	MXRA7	17	74687865	G	A	0.0894632	0.00929045	0.0269444	0.8700001	1.09806	0.013624	0	0.00846075	0.0245383	0.7302465	0.556404
ENSG00000182541	LIMK2	22	31642145	A	G	0.27336	-0.0309957	0.0156532	0.05399953	-0.0619015	0.00898764	5.68E-12	0.500726	0.263116	0.05703263	0.4505165
ENSG00000182544	MFSD5	12	53646612	C	T	0.445328	0.0078437	0.0142845	0.6800001	-0.256672	0.00787468	4.89E-233	-0.0305592	0.0556606	0.5829864	0.389781
ENSG00000182551	ADI1	2	3512598	G	C	0.384692	0.0156167	0.015033	0.35	0.351409	0.00819437	0	0.0444402	0.0427918	0.2990265	0.2926522
ENSG00000182552	RWDD4	4	184570583	T	C	0.479125	0.0232895	0.0142338	0.14	-0.172193	0.0175285	8.91E-23	-0.135252	0.0838007	0.1065325	0.4970682
ENSG00000182557	SPNS3	17	4364243	A	G	0.331014	0.0172263	0.0150983	0.2	0.0601144	0.00837248	6.97E-13	0.286559	0.254311	0.2598248	0.1899293
ENSG00000182566	CLEC4G	19	7796317	T	G	0.162028	0.0369778	0.0196676	0.0659994	-0.546176	0.0103117	0	-0.0677031	0.0360323	0.0602507	0.1296893
ENSG00000182568	SATB1	3	18436972	T	C	0.427435	0.00918003	0.0144513	0.7700005	0.159439	0.00800807	3.35E-88	0.057577	0.0906845	0.5254832	0.2644313
ENSG00000182578	CSF1R	5	149462894	A	G	0.295229	-0.00564254	0.015491	0.6600001	0.27127	0.00861928	2.07E-217	-0.0208004	0.0571092	0.7156923	0.326209
ENSG00000182580	EPHB3	3	184289884	A	G	0.442346	-0.0166329	0.0142586	0.2700001	-0.227676	0.00788373	2.18E-183	0.0730552	0.0626779	0.2437897	0.2703887
ENSG00000182584	NA	20	32255317	T	C	0.481113	0.00761405	0.0142214	0.3800004	0.101686	0.00888479	2.49E-30	0.0748777	0.140008	0.5927819	0.1280076
ENSG00000182586	LINC00334	21	46666450	G	A	0.167992	0.00905516	0.0201548	0.6499995	0.175324	0.0168312	2.08E-25	0.0516482	0.115064	0.6535303	0.4636484
ENSG00000182600	SNORC	2	233732699	C	T	0.202783	-0.0152886	0.0191622	0.2599998	0.0994757	0.0102502	2.88E-22	-0.153692	0.193282	0.426515	0.7993792
ENSG00000182606	TRAK1	3	42161337	T	C	0.366799	0.0030095	0.0150132	0.9599999	-0.0777788	0.00837321	1.56E-20	-0.0386931	0.193069	0.8411597	0.1281021
ENSG00000182612	TSPAN10	17	79609988	C	T	0.328032	0.0135579	0.0145863	0.29	0.1286	0.00810741	1.16E-56	0.105427	0.113619	0.3534575	0.9326656
ENSG00000182621	PLCB1	20	8530913	A	G	0.293241	-0.00196442	0.0158631	0.91	-0.590391	0.00814135	0	0.00332732	0.0268688	0.9014454	0.8819968
ENSG00000182628	SKA2	17	57209971	C	T	0.342942	0.00191847	0.0152134	0.9	-0.156623	0.00954725	1.76E-60	-0.012249	0.097137	0.8996526	0.6975544
ENSG00000182636	NDN	15	23931507	G	A	0.470179	0.00709092	0.0142272	0.4199997	0.377058	0.00776683	0	0.0188059	0.0377341	0.6182165	0.8288339
ENSG00000182670	TTC3	21	38510469	A	G	0.152087	-0.00931371	0.0190878	0.64	-0.52016	0.00996263	0	0.0179055	0.0366976	0.6256071	0.159017
ENSG00000182685	BRICD5	16	2260602	C	T	0.477137	-0.00340186	0.0142113	0.9599999	0.169927	0.0123617	5.37E-43	-0.0200196	0.0836447	0.8108413	0.4237095
ENSG00000182718	ANXA2	15	60667207	T	C	0.271372	0.0127182	0.0163758	0.5400003	0.28109	0.00918197	8.17E-206	0.0452461	0.058277	0.4375151	0.4641625
ENSG00000182732	RGS6	14	72714905	T	C	0.265408	0.000622445	0.0165452	0.84	0.165597	0.00912592	1.39E-73	0.00375879	0.0999125	0.96999	0.4030091
ENSG00000182742	HOXB4	17	46655174	C	T	0.515905	-0.00637165	0.0142075	0.8800001	0.322474	0.00776221	0	-0.0197586	0.0440604	0.6538323	0.3015694
ENSG00000182747	SLC35D3	6	137245089	A	G	0.186879	0.0351771	0.018161	0.05699936	-0.402578	0.01111	1.67E-287	-0.0873795	0.0451761	0.05308892	0.1087269
ENSG00000182749	PAQR7	1	26192722	T	C	0.467197	-0.00382383	0.0142623	0.6200004	-0.0834828	0.00797962	1.29E-25	0.0458038	0.170897	0.7886843	0.005775093
ENSG00000182768	NGRN	15	90812677	C	G	0.178926	0.0017671	0.0191608	0.8700001	-0.0960693	0.011411	3.80E-17	-0.018394	0.19946	0.9265239	0.6515247
ENSG00000182782	HCAR2	12	123186865	A	G	0.436382	0.0407059	0.0144312	0.003099988	-0.11936	0.0104781	4.62E-30	-0.341035	0.124556	0.00618145	0.01835197
ENSG00000182809	CRIP2	14	105942899	C	T	0.2833	-0.0105687	0.0159886	0.4799997	-0.372748	0.0116698	7.18E-224	0.0283535	0.0429031	0.5086936	0.7547811
ENSG00000182810	DDX28	16	68056474	G	A	0.150099	-0.0152342	0.0209764	0.29	0.0950532	0.0110805	9.62E-18	-0.16027	0.22147	0.4692711	0.3414438
ENSG00000182827	ACBD3	1	226353405	C	T	0.185885	-0.0267253	0.0191964	0.16	-0.283123	0.0109597	3.76E-147	0.0943947	0.0679007	0.1644723	0.01109996
ENSG00000182831	HAPSTR1	16	9200501	A	G	0.388668	-0.0135968	0.0144339	0.32	0.325348	0.00792714	0	-0.0417915	0.0443761	0.3463173	0.2840004
ENSG00000182841	NA	22	42964636	G	T	0.389662	-0.021676	0.0150655	0.14	0.471958	0.0187211	3.11E-140	-0.0459278	0.0319732	0.1508753	0.9177224
ENSG00000182853	VMO1	17	4689154	A	G	0.175944	-0.0022988	0.0184747	0.8200001	-0.370703	0.0105148	2.83E-272	0.00620118	0.0498372	0.9009757	0.9799489
ENSG00000182858	ALG12	22	50304486	C	T	0.2167	-0.0260653	0.0177275	0.08700015	0.183331	0.00971268	1.82E-79	-0.142176	0.0969898	0.1426781	0.3765471
ENSG00000182870	GALNT9	12	132793429	A	G	0.518887	0.000639178	0.0142124	0.8600001	0.165683	0.00911638	8.26E-74	0.00385784	0.085781	0.9641287	0.8414218
ENSG00000182871	COL18A1	21	46879343	T	C	0.234592	-0.00352183	0.0166409	0.6300007	-0.807671	0.00943452	0	0.00436048	0.0206036	0.8323908	0.8746728

ENSG00000182873	PRKCZ-AS1	1	2114530	T	C	0.331014	-0.00416831	0.0152094	0.7099994	-0.403102	0.02066	8.82E-85	0.0103406	0.0377346	0.7840582	0.3570421
ENSG00000182885	ADGRG3	16	57713037	G	C	0.232604	0.0289139	0.0174524	0.16	0.344249	0.00996243	1.21E-261	0.0839913	0.0507553	0.09795934	0.5780646
ENSG00000182901	RGS7	1	241226042	T	C	0.192843	-0.0111133	0.0171382	0.4600002	-0.109844	0.00969739	9.62E-30	0.101173	0.156278	0.5173783	0.4629205
ENSG00000182903	ZNF721	4	456274	T	C	0.232604	0.0195668	0.0168353	0.28	-0.400828	0.0110322	4.97E-289	-0.0488159	0.0420228	0.245376	0.470115
ENSG00000182919	C11orf54	11	93486336	A	G	0.364811	-0.00246484	0.0146216	0.8	0.371189	0.00799191	0	-0.00664039	0.0393915	0.8661316	0.01435369
ENSG00000182923	CEP63	3	134249222	C	G	0.308151	-0.00508044	0.0151811	0.7300002	-0.730211	0.00782521	0	0.00695749	0.0207901	0.7378865	0.8131668
ENSG00000182934	SRPRA	11	126135926	T	C	0.341948	0.018847	0.0146996	0.1299999	0.081571	0.00820073	2.60E-23	0.23105	0.181697	0.2035071	0.15785
ENSG00000182952	HMGN4	6	26542557	G	T	0.442346	0.0122713	0.0142092	0.2700001	0.477721	0.0112011	0	0.0256872	0.0297498	0.3878959	0.8526934
ENSG00000182957	SPATA13	13	24724504	C	A	0.340954	0.00890987	0.0150762	0.4700002	-0.239636	0.00830152	3.15E-183	-0.0371809	0.0629261	0.5546107	0.6932385
ENSG00000182979	MTA1	14	105911612	T	G	0.00298211	0.0588893	0.10492	0.5300002	0.84686	0.125409	1.45E-11	0.0695384	0.12432	0.5759234	NA
ENSG00000182985	CADM1	11	115207806	G	C	0.2167	0.0158732	0.0178242	0.4	-0.178316	0.00969476	1.49E-75	-0.0890174	0.100076	0.3737344	0.04407079
ENSG00000182986	ZNF320	19	53383994	C	A	0.486083	-0.00370182	0.0142052	0.8800001	0.270839	0.00786656	9.22E-260	-0.013668	0.0524503	0.7944097	0.2701557
ENSG00000182993	C12orf60	12	15008013	C	T	0.410537	-0.0134662	0.0144105	0.4400003	0.185408	0.00802028	3.09E-118	-0.0726301	0.0777866	0.3504541	0.6859885
ENSG00000183011	NAA38	17	7774279	A	C	0.0934394	-0.000258793	0.0227142	0.89	-0.226541	0.0136154	3.66E-62	0.00114237	0.100265	0.9909095	0.8319918
ENSG00000183018	SPNS2	17	4422231	A	G	0.0258449	0.0215902	0.0400158	0.4600002	-0.740956	0.0395333	2.22E-78	-0.0291383	0.054028	0.5896671	0.4209951
ENSG00000183019	MCEMP1	19	7743113	C	T	0.260437	0.0400205	0.0165697	0.02199986	-0.0842244	0.010864	9.00E-15	-0.475165	0.206059	0.02111261	0.03466556
ENSG00000183020	AP2A2	11	968566	C	T	0.0874751	0.0343497	0.0254384	0.2700001	-0.312785	0.016957	5.64E-76	-0.109819	0.0815463	0.1780751	0.7969545
ENSG00000183023	SLC8A1	2	40588739	A	G	0.33002	-0.0139787	0.0152255	0.2700001	-0.239651	0.00840844	1.13E-178	0.0583293	0.0635648	0.3588099	0.3208163
ENSG00000183044	ABAT	16	8823427	G	A	0.468191	0.0148219	0.0141899	0.29	-0.34023	0.0080144	0	-0.0435644	0.0417195	0.2963824	0.5084272
ENSG00000183049	CAMK1D	10	12634513	T	C	0.22664	-0.0383796	0.0174697	0.0129999	0.639972	0.00908412	0	-0.0599708	0.0273109	0.02810222	0.8914561
ENSG00000183060	LYSMD4	15	100264836	T	C	0.11829	0.0138144	0.0224926	0.32	0.689263	0.0128257	0	0.0200423	0.032635	0.539126	0.9305601
ENSG00000183066	WBP2NL	22	42424594	A	G	0.276342	0.011673	0.0155059	0.3900004	-0.10359	0.00904794	2.38E-30	-0.112685	0.150009	0.4525395	0.01173018
ENSG00000183077	AFMID	17	76193590	T	C	0.0119284	-0.00849954	0.0643504	0.7499995	0.462098	0.0465627	3.27E-23	-0.0183934	0.139269	0.8949286	0.8290169
ENSG00000183087	GAS6	13	114545285	G	T	0.354871	0.0117739	0.0145576	0.56	0.171163	0.00902036	2.73E-80	0.0687876	0.0851283	0.4190637	0.1081777
ENSG00000183091	NEB	2	152466425	C	T	0.0248509	-0.0686616	0.0471426	0.1100001	0.816834	0.0220951	3.61E-299	-0.0840582	0.0577586	0.145576	0.6713127
ENSG00000183092	BEGAIN	14	101028618	C	T	0.411531	0.0369146	0.0146671	0.016	-0.587766	0.0074934	0	-0.0628049	0.0249668	0.01188517	0.611977
ENSG00000183103	NA	19	42344954	C	T	0.374751	0.00838282	0.0146048	0.35	-0.216798	0.0127196	3.85E-65	-0.0386665	0.0674042	0.5662034	0.5561236
ENSG00000183111	ARHGEF37	5	148973020	G	C	0.263419	0.0486906	0.0160906	0.002300011	-0.0544943	0.00919021	3.04E-09	-0.893499	0.331498	0.007031738	0.09555585
ENSG00000183117	CSMD1	8	3822743	C	T	0.153082	-0.0379704	0.0201196	0.032	0.21444	0.0107759	4.07E-88	-0.177068	0.094245	0.0602712	0.7360838
ENSG00000183134	PTGDR2	11	60620928	C	A	0.44831	0.0227763	0.014457	0.1499999	0.113773	0.00893556	3.90E-37	0.200191	0.128038	0.1179285	0.9486652
ENSG00000183137	CEP57L1	6	109450724	A	G	0.137177	0.00518737	0.0214614	0.98	-0.0963279	0.0127854	4.91E-14	-0.0538512	0.22291	0.8091035	0.489994
ENSG00000183150	GPR19	12	12831483	T	C	0.102386	0.0184363	0.0244267	0.3900004	0.656453	0.0131738	0	0.0280847	0.0372144	0.4504449	0.8720119
ENSG00000183155	RABIF	1	202853174	C	T	0.359841	0.00277532	0.0147458	0.5	0.0659605	0.00842968	5.08E-15	0.0420755	0.22362	0.850754	0.5343742
ENSG00000183160	TMEM119	12	108987859	C	T	0.027833	-0.0337513	0.0559248	0.4299995	1.16402	0.0270263	0	-0.0289954	0.0480492	0.5462074	0.9755155
ENSG00000183161	FANCF	11	22645733	C	T	0.234592	-0.00762491	0.0171881	0.59	0.182199	0.0100389	1.30E-73	-0.0418494	0.0943652	0.6574163	0.168604
ENSG00000183172	SMDT1	22	42497900	A	G	0.33499	0.0260351	0.0151507	0.07299952	-0.800966	0.00779859	0	-0.0325046	0.0189182	0.08576587	0.1188397
ENSG00000183207	RUVBL2	19	49507978	C	A	0.0954274	-0.0286629	0.0273514	0.3100002	-0.375257	0.0218097	2.39E-66	0.076382	0.0730221	0.2955556	0.2064558
ENSG00000183208	GDPGP1	15	90781177	T	C	0.264414	0.0144556	0.0161517	0.25	0.24405	0.00999304	9.99E-132	0.0592321	0.0662263	0.3711138	0.807475
ENSG00000183246	RIMBP3C	22	21902698	T	C	0.054672	-0.0115866	0.0322556	0.7700005	-0.581584	0.0519234	4.04E-29	0.0199225	0.0554902	0.7195745	0.410095
ENSG00000183248	PRR36	19	7936465	G	C	0.10338	-0.00502027	0.0230396	0.89	0.205771	0.0175357	8.49E-32	-0.0243973	0.111986	0.8275383	0.337764
ENSG00000183250	LINC01547	21	46356278	G	A	0.218688	0.00742518	0.0175882	0.5300002	0.199089	0.0136609	4.14E-48	0.0372958	0.0883805	0.6730314	0.7993683
ENSG00000183255	PTTG1IP	21	46281626	C	G	0.454274	-0.00525552	0.0143168	0.6700003	0.531731	0.00747628	0	-0.00988379	0.0269252	0.7135573	0.4567257
ENSG00000183260	ABHD16B	20	62493453	A	G	0.0248509	-0.0616613	0.0476073	0.29	-0.281439	0.0241558	2.27E-31	0.219093	0.170199	0.1979977	0.1117368
ENSG00000183281	PLGLB1	2	87239328	T	C	0.318091	-0.0242457	0.0160994	0.1100001	0.658556	0.0210807	3.08E-214	-0.0368165	0.0244749	0.1325162	0.5861164
ENSG00000183283	DAZAP2	12	51636288	G	A	0.141153	-0.018165	0.0219614	0.4299995	-0.210896	0.0115894	5.41E-74	0.0861325	0.104241	0.4086453	0.852634
ENSG00000183291	SELENOF	1	87354119	C	T	0.209742	0.016963	0.0171329	0.28	0.112808	0.00998606	1.36E-29	0.15037	0.152458	0.3239847	0.1397303
ENSG00000183307	TMEM121B	22	17599723	T	C	0.247515	-0.00666223	0.0162878	0.6600001	-0.675919	0.00843248	0	0.00985655	0.0240976	0.6825201	0.3246234
ENSG00000183309	ZNF623	8	144727041	T	C	0.352883	-0.0231758	0.0149209	0.06699926	0.169024	0.00828925	2.02E-92	-0.137115	0.0885323	0.1214401	0.2425639
ENSG00000183317	EPHA10	1	38205178	C	A	0.201789	-0.00903427	0.0174375	0.6200004	-0.0582527	0.0102047	1.14E-08	0.155088	0.300573	0.6058729	0.8343655
ENSG00000183323	CCDC125	5	68602319	C	T	0.467197	-0.0107688	0.0142521	0.5	-0.371068	0.0078281	0	0.0290211	0.0384132	0.4499503	0.6406826

ENSG00000183347	GBP6	1	89841668	T	C	0.314115	-0.00396663	0.0147613	0.8700001	0.109794	0.00836051	2.15E-39	-0.0361281	0.134474	0.7881898	0.199601
ENSG00000183354	KIAA2026	9	5944748	G	A	0.32505	0.0200439	0.0158968	0.29	-0.157038	0.00939761	1.10E-62	-0.127637	0.101517	0.2086442	0.7575701
ENSG00000183386	FHL3	1	38466860	A	G	0.255467	-0.0136382	0.0170004	0.5300002	0.922476	0.00889603	0	-0.0147843	0.0184296	0.4224349	0.4541814
ENSG00000183397	TEKTIP1	19	3541590	C	T	0.149105	0.00769124	0.0187331	0.5199996	-0.265239	0.0176819	7.28E-51	-0.0289974	0.0706537	0.6815009	0.1184085
ENSG00000183401	CCDC159	19	11460490	A	G	0.185885	-0.00592226	0.0195122	0.64	0.617112	0.0235011	5.66E-152	-0.00959674	0.0316207	0.7615123	0.01934118
ENSG00000183421	RIPK4	21	43173397	A	C	0.303181	-0.0315326	0.0153649	0.02800013	0.145006	0.00841256	1.41E-66	-0.217458	0.106709	0.04156437	0.1372027
ENSG00000183423	LRIT3	4	110781414	G	T	0.145129	-0.00789661	0.0208097	0.7300002	-0.137783	0.0116785	4.00E-32	0.0573121	0.151111	0.7044862	0.7804878
ENSG00000183426	NPIPA1	16	15031287	T	C	0.397614	0.00141674	0.0144269	0.7899998	-0.637381	0.016308	0	-0.00222275	0.0226347	0.9217727	0.2613174
ENSG00000183431	SF3A3	1	38439620	A	G	0.425447	0.00662247	0.0143828	0.6200004	0.362455	0.00781586	0	0.0182711	0.0396835	0.645214	0.7267823
ENSG00000183439	TRIM61	4	165887209	T	C	0.0457256	-0.0202544	0.0312869	0.4700002	-0.642233	0.0481009	1.16E-40	0.0315374	0.048773	0.51788	0.797771
ENSG00000183444	OR7E38P	7	97595868	A	G	0.28827	0.0374054	0.0154683	0.008700015	0.323677	0.0209424	6.92E-54	0.115564	0.0483707	0.01688828	0.2493047
ENSG00000183458	PKD1P3	16	15017486	C	G	0.151093	-0.0401083	0.0195859	0.02800013	0.243799	0.0265212	3.83E-20	-0.164514	0.0823055	0.04562791	0.8427131
ENSG00000183473	NA	22	37604320	T	C	0.420477	-0.0162447	0.0142927	0.28	-0.157795	0.00820603	2.11E-82	0.102948	0.0907355	0.2565459	0.6438308
ENSG00000183474	GTF2H2C	5	68873292	G	T	0.323062	-0.000447766	0.0152386	0.9400001	0.247958	0.0200759	4.81E-35	-0.00180582	0.0614566	0.9765587	0.7210572
ENSG00000183475	ASB7	15	101167324	G	A	0.193837	-0.0321129	0.0177697	0.05399953	-0.0824365	0.00969864	1.90E-17	0.389547	0.220374	0.07711772	0.6253823
ENSG00000183484	GPR132	14	105523755	A	C	0.361829	-0.00416409	0.0154197	0.7099994	-0.072387	0.00950061	2.55E-14	0.0575254	0.213151	0.7872522	0.9332371
ENSG00000183486	MX2	21	42757593	A	G	0.0606362	-0.0270494	0.0270648	0.2	-0.555031	0.015399	1.76E-284	0.048735	0.0487815	0.3177719	0.6832293
ENSG00000183495	EP400	12	132499755	A	C	0.0626243	-0.0132905	0.0314666	0.4799997	-0.335964	0.0174596	1.63E-82	0.0395593	0.0936832	0.6728303	0.9771334
ENSG00000183496	MEX3B	15	82336300	C	G	0.2167	0.00753513	0.0169891	0.6800001	0.105048	0.00950075	2.03E-28	0.0717304	0.161857	0.6576421	0.9758666
ENSG00000183506	PI4KAP2	22	21849555	T	C	0.229622	0.0103306	0.0168588	0.3400001	-0.47953	0.0237013	5.10E-91	-0.0215432	0.035173	0.5402129	0.3456502
ENSG00000183508	TENT5C	1	118159775	C	T	0.166998	0.0279815	0.0189074	0.1900002	-0.189934	0.0104244	3.57E-74	-0.147322	0.0998751	0.140195	0.1898888
ENSG00000183513	COA5	2	99220375	C	T	0.271372	0.00167031	0.0163073	1	0.191846	0.010327	4.91E-77	0.00870649	0.0850031	0.9184189	0.5275096
ENSG00000183520	UTP11	1	38482713	C	A	0.467197	0.0126385	0.0142641	0.3700002	0.239635	0.00808365	4.00E-193	0.0527406	0.0595509	0.3758115	0.3010153
ENSG00000183527	PSMG1	21	40551236	G	A	0.200795	0.00833327	0.0177303	0.6200004	-0.208653	0.00963882	6.46E-104	-0.0399383	0.0849949	0.638433	0.8321466
ENSG00000183542	KLRC4	12	10561169	C	T	0.222664	0.0077895	0.0167842	0.6999999	0.349209	0.0209634	2.65E-62	0.0223062	0.0480822	0.6427071	0.346281
ENSG00000183562	NA	11	3011828	T	C	0.101392	-0.082806	0.0238851	0.000309999	0.821525	0.0318043	4.02E-147	-0.100795	0.0293348	0.000590288	0.8879869
ENSG00000183569	SERHL2	22	42960005	T	C	0.32505	-0.00827659	0.0151715	0.6100002	-0.257271	0.0153571	5.42E-63	0.0321707	0.0590022	0.5855841	0.5854836
ENSG00000183570	PCBP3	21	47212988	C	T	0.403579	-0.0105696	0.0145431	0.4299995	0.214094	0.00810988	1.40E-153	-0.0493691	0.0679544	0.4675308	0.2925197
ENSG00000183576	SETD3	14	99905649	A	G	0.429423	-0.0130717	0.0144691	0.1800002	0.307536	0.00780631	0	-0.0425046	0.0470608	0.3664281	0.4603611
ENSG00000183579	ZNRF3	22	29366527	A	G	0.157058	0.0142117	0.0198232	0.6300007	-0.0858632	0.0118207	3.76E-13	-0.165516	0.231991	0.4755636	0.1316284
ENSG00000183597	TANGO2	22	20028993	G	A	0.119284	0.000801566	0.0216337	0.6300007	-0.31106	0.0149689	6.51E-96	-0.00257689	0.0695485	0.9704438	0.2080294
ENSG00000183604	NA	16	30290462	C	G	0.431412	0.0239673	0.0142163	0.03099988	-0.639158	0.0171799	5.92E-303	-0.0374982	0.0222651	0.09214795	0.7918871
ENSG00000183605	SFXN4	10	120912729	T	C	0.562624	0.0260505	0.0142364	0.1100001	-0.719836	0.00684783	0	-0.0361895	0.0197803	0.06731359	0.3465026
ENSG00000183615	FAM167B	1	32713645	T	C	0.0328032	-0.0169304	0.0368551	0.5	0.340888	0.017764	4.50E-82	-0.0496656	0.108146	0.6460576	0.6203038
ENSG00000183617	MRPL54	19	3765617	G	A	0.372763	0.0110773	0.0147245	0.5300002	-0.724173	0.0106709	0	-0.0152965	0.0203341	0.4518961	0.001464531
ENSG00000183621	ZNF438	10	31227214	A	C	0.423459	-0.012247	0.0146112	0.4100001	0.163094	0.00810992	5.98E-90	-0.0750918	0.0896655	0.402331	0.6117718
ENSG00000183624	HMCES	3	129011350	C	A	0.0924453	0.0541046	0.0235797	0.03799969	-0.294677	0.0144098	6.03E-93	-0.183606	0.0805209	0.02259389	0.3706901
ENSG00000183625	CCR3	3	46256646	A	T	0.336978	0.0150689	0.0148683	0.2200002	0.599022	0.0077491	0	0.0251558	0.0248231	0.3108669	0.9917653
ENSG00000183628	DGCR6	22	18897646	C	T	0.371769	0.0266436	0.0146773	0.09800089	-0.394816	0.0114386	4.60E-261	-0.0674836	0.0372264	0.06986458	0.8021135
ENSG00000183647	ZNF530	19	58117671	T	C	0.0218688	0.0500633	0.0467035	0.4299995	-0.159523	0.0265651	1.91E-09	-0.313831	0.297397	0.2913074	0.4456351
ENSG00000183648	NDUFB1	14	92585363	T	G	0.33499	0.00845335	0.015143	0.5999997	-0.185559	0.00885653	1.81E-97	-0.0455562	0.0816366	0.5768193	0.3219836
ENSG00000183655	KLHL25	15	86320407	A	G	0.367793	0.00234852	0.0151372	0.8499999	-0.056919	0.00829965	6.98E-12	-0.0412607	0.266011	0.8767352	0.3436489
ENSG00000183657	NA	3	171518647	C	T	0.135189	0.0167573	0.0218285	0.4199997	1.01638	0.0315261	4.99E-228	0.0164873	0.0214829	0.4428067	0.04352145
ENSG00000183662	TAFA1	3	68324067	T	G	0.384692	-0.0081307	0.0144124	0.58	0.449461	0.0077027	0	-0.0180899	0.0320675	0.5726726	0.6416708
ENSG00000183665	TRMT12	8	125468719	C	T	0.49503	0.00542025	0.0142048	0.5300002	0.737825	0.00680497	0	0.00734626	0.0192524	0.7027756	0.4502439
ENSG00000183666	NA	5	21443414	T	C	0.140159	-0.042562	0.0210448	0.04099964	0.356615	0.0279404	2.62E-37	-0.11935	0.059749	0.04576815	0.05573196
ENSG00000183682	BMP8A	1	39974462	A	G	0.0397614	0.0068256	0.0349658	0.84	-0.854657	0.0433392	1.45E-86	-0.00798637	0.0409141	0.8452377	0.1695734
ENSG00000183688	RFLNB	17	292749	C	G	0.161034	0.0110156	0.0189519	0.4799997	0.220517	0.0114842	3.57E-82	0.0499536	0.0859826	0.5612581	0.4953968
ENSG00000183691	NOG	17	54672005	G	A	0.0705765	-0.022765	0.0322661	0.3100002	-0.269276	0.0172839	1.00E-54	0.0845415	0.119948	0.4809242	0.1778261
ENSG00000183696	UPP1	7	48138277	G	A	0.422465	0.00615099	0.0143154	0.6700003	-0.337871	0.00777051	0	-0.0182051	0.0423715	0.6674463	0.6775125

ENSG00000183718	TRIM52	5	180684768	T	C	0.225646	-0.012664	0.0169342	0.5300002	0.108101	0.00991645	1.14E-27	-0.11715	0.15702	0.4556177	0.6859989
ENSG00000183722	LHFPL6	13	40047347	T	C	0.484095	-0.0251607	0.0145352	0.05099998	-0.151763	0.00802597	9.62E-80	0.165789	0.0961761	0.08474207	0.01374835
ENSG00000183723	CMTM4	16	66689631	T	C	0.0109344	-0.039957	0.0531094	0.35	0.229207	0.0252264	1.03E-19	-0.174327	0.232502	0.4533832	0.3940872
ENSG00000183726	TMEM50A	1	25676630	A	G	0.388668	0.00647447	0.0144501	0.6200004	-0.493538	0.00920448	0	-0.0131185	0.0292796	0.6541233	0.1159787
ENSG00000183734	ASCL2	11	2290953	A	T	0.430417	-0.0231676	0.0143994	0.1100001	-0.360069	0.00781321	0	0.0643421	0.040015	0.1078465	0.64746
ENSG00000183735	TBK1	12	64870774	G	A	0.122266	0.0042217	0.0242559	0.7199992	-0.385838	0.0122865	1.81E-216	-0.0109416	0.0628665	0.8618295	0.09571814
ENSG00000183741	CBX6	22	39262887	A	C	0.171968	0.0222886	0.0182749	0.33	-0.406295	0.00967645	0	-0.0548582	0.0449984	0.2228006	0.003279613
ENSG00000183751	TBL3	16	2027486	A	G	0.0775348	0.0264996	0.0268108	0.4299995	-0.159346	0.0219817	4.20E-13	-0.166303	0.169812	0.3274163	0.232068
ENSG00000183762	KREMEN1	22	29516693	A	C	0.482107	-0.0179184	0.0142453	0.2200002	-0.321826	0.00773676	0	0.0556773	0.0442842	0.2086552	0.5317423
ENSG00000183765	CHEK2	22	29111070	C	A	0.112326	-0.00854783	0.0221421	0.6200004	0.098188	0.0116279	3.06E-17	-0.0870558	0.225743	0.6997622	0.9085572
ENSG00000183773	AIFM3	22	21327522	T	C	0.0705765	0.0636279	0.030238	0.0530005	-0.480697	0.0193307	1.69E-136	-0.132366	0.0631294	0.03601634	0.6792434
ENSG00000183780	SLC35F3	1	234250470	G	A	0.323062	-0.00314192	0.0152233	0.7300002	0.533333	0.00792074	0	-0.00589111	0.0285439	0.8364879	0.2606235
ENSG00000183784	DOCK8-AS1	9	214500	G	A	0.432406	0.0122509	0.0143167	0.3599996	0.167663	0.00796013	1.75E-98	0.0730687	0.0854604	0.3925502	0.9178912
ENSG00000183793	NPIPA5	16	15466210	T	C	0.397614	0.00141674	0.0144269	0.7899998	0.833207	0.0160633	0	0.00170035	0.0173149	0.9217726	0.3014699
ENSG00000183801	OLFML1	11	7519613	A	G	0.532803	0.0148326	0.0142306	0.2399999	-0.0738534	0.00833936	8.29E-19	-0.200839	0.194017	0.300595	0.6258488
ENSG00000183813	CCR4	3	32995453	T	C	0.209742	0.0116418	0.0170886	0.35	0.22408	0.00940413	1.72E-125	0.0519538	0.0762924	0.4958823	0.2515587
ENSG00000183814	LIN9	1	226458210	G	A	0.32008	0.020298	0.0152015	0.2	0.104015	0.0086081	1.29E-33	0.195145	0.147037	0.1844477	0.07946942
ENSG00000183822	NCF4-AS1	22	37254949	C	A	0.290258	-0.0323468	0.0159409	0.02999991	0.167838	0.0225092	8.89E-14	-0.192727	0.0984322	0.0502344	0.6106387
ENSG00000183826	BTBD9	6	38372075	T	C	0.388668	0.0130321	0.0143492	0.3400001	-0.0600718	0.00808024	1.05E-13	-0.216942	0.240643	0.3673178	0.8389069
ENSG00000183828	NUDT14	14	105643467	G	A	0.383698	-0.00418499	0.0145518	0.89	-0.187485	0.00913607	1.39E-93	0.0223217	0.0776234	0.7736802	0.646127
ENSG00000183844	FAM3B	21	42702748	T	G	0.137177	-0.00425201	0.0197619	0.6499995	1.20997	0.00895779	0	-0.00351413	0.0163325	0.8296412	0.854941
ENSG00000183864	TOB2	22	41836261	T	A	0.220676	0.0395499	0.0176547	0.02999991	-0.168201	0.0146519	1.67E-30	-0.235134	0.106942	0.02789797	0.1229312
ENSG00000183873	SCN5A	3	38640356	A	G	0.0238569	0.0616721	0.0460958	0.1900002	0.290777	0.0292921	3.18E-23	0.212094	0.15996	0.1848652	0.2158769
ENSG00000183889	NPIPA9	16	16427874	T	C	0.237575	-0.0117147	0.016871	0.6100002	-0.50332	0.0281383	1.48E-71	0.0232748	0.0335447	0.4877785	0.4844918
ENSG00000183891	TTC32	2	20099075	A	C	0.109344	0.0372906	0.0249852	0.2	1.10996	0.0136109	0	0.0335963	0.0225137	0.1356316	0.9056552
ENSG00000183914	DNAH2	17	7678867	T	C	0.313121	-0.0153898	0.0154722	0.29	-0.0861363	0.00885428	2.29E-22	0.178668	0.180561	0.322411	0.8757297
ENSG00000183935	HTR7P1	12	13155288	T	A	0.45328	-0.00194128	0.0143757	1	-0.124002	0.00905864	1.18E-42	0.0156552	0.115937	0.8925862	0.8613933
ENSG00000183955	KMT5A	12	123881112	C	T	0.33002	-0.00107478	0.0155582	0.95	0.650889	0.0171878	0	-0.00165125	0.023903	0.944925	0.9793425
ENSG00000183960	KCNH8	3	19383542	C	T	0.269384	0.0154592	0.016433	0.33	0.0733841	0.00905999	5.50E-16	0.210662	0.225437	0.3500668	0.8452714
ENSG00000183977	PP2D1	3	20037637	A	G	0.266402	-0.0239753	0.0170773	0.07100027	-0.0616296	0.0110388	2.36E-08	0.389022	0.285722	0.1733432	0.8333005
ENSG00000184005	ST6GALNAC3	1	76820345	G	C	0.175944	-0.00811527	0.0175065	0.6700003	0.374424	0.00975804	0	-0.021674	0.0467592	0.642989	0.8373344
ENSG00000184007	PTP4A2	1	32391239	A	G	0.409543	0.010092	0.0145966	0.4899999	-0.0476865	0.00816215	5.15E-09	-0.211632	0.308231	0.492333	0.6663625
ENSG00000184009	ACTG1	17	79483935	T	C	0.204771	-0.00934093	0.0171751	0.4199997	-0.141196	0.0106178	2.38E-40	0.0661558	0.121742	0.5868473	0.3407425
ENSG00000184014	DENND5A	11	9223654	C	T	0.316103	-0.0180409	0.0160738	0.2999998	0.167381	0.00894648	4.17E-78	-0.107783	0.0962036	0.2625582	0.7996216
ENSG00000184047	DIABLO	12	122702145	T	C	0.459245	-0.0119341	0.0143354	0.33	0.142465	0.0087237	5.96E-60	-0.0837686	0.100755	0.4057409	0.1712176
ENSG00000184056	VPS33B	15	91553739	T	G	0.214712	-0.0089561	0.0177478	0.4100001	-0.377924	0.00921482	0	0.0236982	0.0469649	0.6138445	0.568513
ENSG00000184060	ADAP2	17	29259851	G	A	0.0168986	0.0135159	0.0523911	0.6300007	-0.339584	0.0439615	1.12E-14	-0.0398013	0.154366	0.7965326	0.5615963
ENSG00000184068	NA	22	42228944	G	C	0.420477	-0.0172828	0.0143109	0.17	-0.610931	0.0181911	2.86E-247	0.0282893	0.0234399	0.2274757	0.01894792
ENSG00000184076	UQCR10	22	30164880	G	A	0.0467197	-0.00823679	0.0334119	0.6999999	0.368201	0.0179659	2.42E-93	-0.0223704	0.0907503	0.8052915	0.7567716
ENSG00000184106	NA	6	41183337	C	G	0.228628	0.0217689	0.0174149	0.1800002	1.1342	0.00819953	0	0.0191932	0.015355	0.2113124	0.5282828
ENSG00000184110	EIF3C	16	28723465	A	G	0.301193	-0.00453415	0.0148501	0.83	0.375083	0.0194094	3.32E-83	-0.0120884	0.0395965	0.7601454	0.9994302
ENSG00000184113	CLDN5	22	19512807	C	T	0.210736	-0.0116597	0.0175122	0.6200004	0.345562	0.00986157	5.29E-269	-0.0337412	0.0506866	0.5056129	0.8281952
ENSG00000184117	NIPSNAP1	22	29964061	A	G	0.517893	-0.000774507	0.0141926	0.95	-0.295703	0.00782064	0	0.00261921	0.0479962	0.9564801	0.595732
ENSG00000184164	CRELD2	22	50316501	G	A	0.198807	-0.0177025	0.0181481	0.2599998	0.718227	0.00929002	0	-0.0246475	0.0252699	0.3293773	0.2594101
ENSG00000184178	SCFD2	4	53985695	A	G	0.0795229	0.018688	0.0277055	0.6899999	-0.261765	0.0140307	1.12E-77	-0.0713924	0.10591	0.5002587	0.5017055
ENSG00000184182	UBE2F	2	238913352	C	A	0.415507	-0.0285013	0.0145979	0.032	0.497676	0.0114093	0	-0.0572688	0.0293615	0.05111991	0.1402499
ENSG00000184185	KCNJ12	17	21301344	C	G	0.219682	0.00728897	0.0191073	0.9	-0.285453	0.0286197	1.98E-23	-0.0255347	0.0669857	0.7030571	0.9608564
ENSG00000184203	PPP1R2	3	195255715	T	C	0.400596	-0.00741035	0.0146306	0.4700002	0.141707	0.0178046	1.73E-15	-0.0522935	0.103454	0.6132258	0.8553989
ENSG00000184207	PGP	16	2263403	C	T	0.477137	-0.00340186	0.0142113	0.9599999	0.215023	0.0104186	1.24E-94	-0.0158209	0.0660965	0.8108257	0.4726241
ENSG00000184209	SNRNP35	12	123949944	T	C	0.159046	-0.0231156	0.0180478	0.35	-0.111679	0.0121644	4.28E-20	0.206983	0.16317	0.2046145	0.3543108

ENSG00000184220	CMSS1	3	99717062	A	G	0.431412	-0.00947779	0.0144902	0.5199996	-0.413349	0.0087051	0	0.0229293	0.0350589	0.5130985	0.397954
ENSG00000184221	OLIG1	21	34443588	A	G	0.241551	0.0303019	0.0165403	0.03699985	0.650534	0.00883882	0	0.0465801	0.0254336	0.06703535	0.45682
ENSG00000184226	PCDH9	13	67340717	A	C	0.390656	-0.00403452	0.0146338	0.8600001	0.0493413	0.00863968	1.12E-08	-0.0817677	0.296929	0.7830258	0.346786
ENSG00000184227	ACOT1	14	74007158	T	C	0.256461	-0.00718009	0.0159904	0.6300007	-0.256831	0.00959903	1.05E-157	0.0279565	0.0622693	0.6534592	0.6653885
ENSG00000184232	OAF	11	120091258	A	G	0.0944334	-0.0353424	0.025684	0.1499999	0.455022	0.0151481	3.11E-198	-0.0776718	0.0565048	0.1692537	0.8342682
ENSG00000184271	POU6F1	12	51596098	T	G	0.303181	-0.0197183	0.0156573	0.28	-0.323305	0.00921399	1.01E-269	0.0609897	0.04846	0.2081902	0.2548848
ENSG00000184277	TM2D3	15	102177220	T	C	0.34493	0.0160556	0.014665	0.1499999	-0.14605	0.00827692	1.10E-69	-0.109933	0.100604	0.2745154	0.990806
ENSG00000184281	TSSC4	11	2423412	T	C	0.0218688	0.0593279	0.0497142	0.2700001	0.24428	0.0360156	1.18E-11	0.242869	0.20664	0.2398645	0.5266058
ENSG00000184292	TACSTD2	1	59042132	G	C	0.17495	0.0479238	0.0187088	0.01700004	1.14265	0.00863977	0	0.0419409	0.0163762	0.0104346	0.6796658
ENSG00000184293	CLECL1P	12	9877175	A	T	0.496024	-0.0139882	0.014181	0.32	-0.610197	0.0076607	0	0.0229241	0.0232418	0.3239718	0.6082708
ENSG00000184304	PRKD1	14	30353395	A	G	0.329026	-0.00675829	0.0148918	0.4299995	0.325795	0.00828535	0	-0.020744	0.0457122	0.6499756	0.2394616
ENSG00000184307	ZDHHC23	3	113675498	A	C	0.0457256	-0.05727	0.0365544	0.1299999	0.152481	0.0221746	6.14E-12	-0.375587	0.245874	0.1266219	0.1490725
ENSG00000184313	MROH7	1	55157720	T	A	0.452286	-0.0101937	0.0142301	0.4600002	-0.179475	0.0102122	3.86E-69	0.0567973	0.0793531	0.4741436	0.9005776
ENSG00000184319	NA	22	51217556	A	C	0.0775348	0.0390736	0.0272592	0.08600031	-0.317675	0.0265308	4.87E-33	-0.122999	0.0864212	0.1546644	0.7759253
ENSG00000184349	EFNA5	5	106859593	T	C	0.0805169	0.0369686	0.0253776	0.09599973	0.114729	0.0148775	1.24E-14	0.322224	0.225107	0.1523083	0.3631271
ENSG00000184350	MRGPRE	11	3251249	A	G	0.472167	-0.020776	0.0142886	0.14	-0.17053	0.00878422	5.97E-84	0.121832	0.0840242	0.1470683	0.2336504
ENSG00000184361	SPATA32	17	43335619	G	C	0.532803	0.00183884	0.0142231	0.7400005	0.224717	0.00885202	3.60E-142	0.0081829	0.0632941	0.8971331	0.7393341
ENSG00000184363	PKP3	11	398761	G	A	0.440358	-0.00532353	0.0142644	0.81	0.141256	0.0100972	1.80E-44	-0.0376872	0.101019	0.7090955	0.6305922
ENSG00000184371	CSF1	1	110463239	T	A	0.0487078	0.0636782	0.0320808	0.05099998	-0.198466	0.0160603	4.43E-35	-0.320851	0.163716	0.05001788	0.7571687
ENSG00000184381	PLA2G6	22	38554599	G	A	0.459245	-0.00614698	0.0142613	0.6999999	0.169044	0.00795181	2.74E-100	-0.0363631	0.0843816	0.6665144	0.2845844
ENSG00000184384	MAML2	11	95893072	C	T	0.110338	-0.0184293	0.0235002	0.4500005	-0.0991147	0.0128573	1.27E-14	0.185939	0.238325	0.4352778	0.7400788
ENSG00000184385	UMODL1-AS1	21	43525444	C	T	0.274354	0.0219631	0.0161133	0.25	0.56002	0.00870137	0	0.0392184	0.0287792	0.1729657	0.887619
ENSG00000184389	A3GALT2	1	33775429	C	G	0.234592	-0.00486235	0.0166332	0.6899999	0.238721	0.0109269	8.30E-106	-0.0203683	0.0696826	0.7700559	0.440616
ENSG00000184402	SS18L1	20	60738181	G	T	0.127237	0.00488024	0.0205018	0.9299999	-0.135766	0.0117357	5.94E-31	-0.035946	0.15104	0.8118895	0.9944155
ENSG00000184428	TOP1MT	8	144414351	A	G	0.138171	0.00673932	0.0191167	0.8600001	0.153091	0.0122632	9.15E-36	0.0440216	0.124921	0.7245419	0.3326772
ENSG00000184432	COPB2	3	139091508	T	A	0.366799	0.00289858	0.0144661	0.7400005	0.12603	0.00801832	1.14E-55	0.0229991	0.114792	0.8412035	0.7219959
ENSG00000184441	NA	21	45753425	A	G	0.238569	-0.0382131	0.0173302	0.01899984	0.540249	0.0217509	3.49E-136	-0.0707324	0.0322043	0.02806599	0.3708317
ENSG00000184445	KNTC1	12	123061368	T	G	0.101392	-0.017384	0.0235588	0.3700002	0.259105	0.0127309	4.42E-92	-0.0670926	0.0909836	0.4608707	0.02414793
ENSG00000184451	CCR10	17	40833421	T	C	0.0367793	0.0172231	0.0366336	0.83	0.132984	0.0196454	1.29E-11	0.129513	0.276138	0.6390589	0.4871805
ENSG00000184465	WDR27	6	169979733	C	G	0.0815109	-0.016317	0.0277502	0.3900004	-0.173272	0.014856	1.96E-31	0.09417	0.160358	0.5570358	0.5196724
ENSG00000184470	TXNRD2	22	19896190	G	T	0.172962	-0.0203156	0.0190506	0.2200002	-0.248178	0.0112995	6.42E-107	0.0818589	0.0768522	0.2868093	0.9622487
ENSG00000184489	PTP4A3	8	142421856	T	C	0.0874751	0.00140835	0.0234447	0.9599999	-0.297952	0.0151821	9.42E-86	-0.00472677	0.0786865	0.9520992	0.7171485
ENSG00000184497	TMEM255B	13	114488571	C	T	0.312127	-0.00635011	0.015051	0.5199996	-0.261875	0.0117849	2.14E-109	0.0242486	0.0574843	0.673149	0.9623446
ENSG00000184500	PROS1	3	93642395	T	C	0.196819	0.020339	0.018422	0.2700001	-0.25484	0.0152615	1.35E-62	-0.0798107	0.0724462	0.2706117	0.000436033
ENSG00000184508	HDDC3	15	91474973	T	C	0.257455	-0.0342917	0.0161823	0.0259998	-0.175192	0.00920792	1.03E-80	0.195738	0.0929402	0.03519876	0.7710832
ENSG00000184517	ZFP1	16	75194262	C	G	0.298211	0.00341877	0.0154021	0.8499999	-0.42445	0.00845952	0	-0.0080546	0.0362876	0.8243407	0.2430322
ENSG00000184524	CEND1	11	788613	T	C	0.440358	0.0204692	0.014232	0.1199999	0.0644302	0.00936949	6.13E-12	0.317696	0.22567	0.1591933	0.13767
ENSG00000184545	DUSP8	11	1584212	T	C	0.05666	-0.0489204	0.0322321	0.14	0.290368	0.0266456	1.19E-27	-0.168477	0.112076	0.1327761	0.2676878
ENSG00000184551	NA	17	80208323	C	T	0.0258449	-0.0252663	0.0363019	0.4100001	-0.413986	0.0488294	2.29E-17	0.0610318	0.0879837	0.4878883	0.3199009
ENSG00000184574	LPAR5	12	6736807	T	G	0.134195	0.0171882	0.0193426	0.33	-0.347763	0.0109024	2.86E-223	-0.049425	0.0556416	0.3743932	0.4941786
ENSG00000184575	XPOT	12	64821518	T	C	0.461233	0.00385021	0.0143168	0.95	-0.103724	0.00837697	3.27E-35	-0.0371199	0.138061	0.7880327	0.6516218
ENSG00000184584	STING1	5	138858819	T	C	0.153082	-0.018126	0.0211612	0.25	-0.324106	0.0116663	7.28E-170	0.0559261	0.065322	0.3919088	0.7435992
ENSG00000184588	PDE4B	1	66549228	G	A	0.265408	0.0214726	0.0164758	0.16	0.123513	0.00929875	2.91E-40	0.173848	0.134033	0.1946131	0.05786087
ENSG00000184602	SNN	16	11767642	G	T	0.0208748	0.101628	0.056176	0.09400046	-0.222752	0.0261735	1.73E-17	-0.456238	0.257826	0.07680016	0.3505684
ENSG00000184613	NELL2	12	45108844	C	T	0.146123	0.0136515	0.0201657	0.4500005	-0.583162	0.0104677	0	-0.0234094	0.0345825	0.498459	0.8612079
ENSG00000184616	SPDYE12	7	74325784	G	A	0.188867	-0.00349597	0.0181148	0.6700003	0.572165	0.0273248	2.34E-97	-0.00611008	0.0316615	0.8469734	0.0201735
ENSG00000184619	KRBA2	17	8275992	T	C	0.284294	-0.00661725	0.0151231	0.4799997	-0.128417	0.00834482	1.95E-53	0.0515295	0.117813	0.6618341	0.3990929
ENSG00000184635	ZNF93	19	20029053	G	A	0.32008	-0.0108203	0.015382	0.3599996	0.112198	0.00856079	3.04E-39	-0.0964396	0.137295	0.4824123	0.7948627
ENSG00000184640	SEPTIN9	17	75386664	T	C	0.435388	-0.0211775	0.0145517	0.17	0.134893	0.00803812	3.33E-63	-0.156995	0.108281	0.1470902	0.1647902
ENSG00000184661	CDCA2	8	25340974	A	G	0.352883	-0.00881841	0.014866	0.35	0.072033	0.00835747	6.75E-18	-0.122422	0.206866	0.553989	0.3218223

ENSG00000184669	OR7E14P	11	17055064	C	A	0.393638	-0.0116216	0.0145324	0.5500004	0.522155	0.0105691	0	-0.022257	0.0278352	0.4239438	0.210133
ENSG00000184674	NA	22	24380406	G	T	0.436382	-0.00301945	0.01424	0.84	-0.790113	0.00726992	0	0.00382154	0.0180228	0.8320761	0.5205447
ENSG00000184677	ZBTB40	1	22817997	T	C	0.371769	-0.00320001	0.0145873	0.84	0.24437	0.00805858	5.50E-202	-0.0130949	0.0596951	0.8263666	0.258088
ENSG00000184682	NA	11	1911229	T	C	0.385686	-0.00528232	0.0147034	0.7499995	0.282129	0.0203272	8.44E-44	-0.0187231	0.0521333	0.7194918	0.2014719
ENSG00000184702	SEPTIN5	22	19707141	C	G	0.0685885	-0.0295045	0.0268274	0.25	0.114585	0.0152135	5.00E-14	-0.25749	0.236609	0.2764851	0.5405983
ENSG00000184708	EIF4ENIF1	22	31863721	C	T	0.499006	-0.0257914	0.0141789	0.1100001	-0.078411	0.00795131	6.12E-23	0.328926	0.183878	0.07364355	0.223449
ENSG00000184719	RNLS	10	90188954	G	A	0.476143	-0.0161039	0.0143404	0.2399999	-0.14536	0.00796247	1.87E-74	0.110787	0.098841	0.2623493	0.4585399
ENSG00000184730	APOBR	16	28508130	T	C	0.134195	0.0062326	0.0209832	0.7899998	-0.280735	0.0144304	2.67E-84	-0.022201	0.0747525	0.7664715	0.8831809
ENSG00000184731	FAM110C	2	42842	A	G	0.0218688	-0.049215	0.0475388	0.2599998	0.29775	0.0352017	2.71E-17	-0.16529	0.160852	0.3041421	0.9229
ENSG00000184743	ATL3	11	63415476	A	T	0.124254	-0.00847521	0.0203517	0.6899999	0.282233	0.0143581	5.06E-86	-0.0300291	0.0721257	0.6771583	0.240777
ENSG00000184752	NDUFA12	12	95344188	T	C	0.354871	0.0034229	0.0150216	0.8499999	0.686493	0.00752602	0	0.00498607	0.0218817	0.8197516	0.8386229
ENSG00000184787	UBE2G2	21	46205444	G	C	0.316103	0.0138589	0.0151068	0.2700001	-0.246093	0.00831899	2.54E-192	-0.0563157	0.061416	0.3591668	0.6043679
ENSG00000184792	OSBP2	22	31196790	G	C	0.254473	0.00241046	0.0162172	0.9400001	-0.103022	0.0090396	4.34E-30	-0.0233974	0.157428	0.8818508	0.1037405
ENSG00000184811	TRARG1	17	1193619	T	C	0.415507	0.00572439	0.0146126	0.6899999	0.116946	0.00893635	3.93E-39	0.0489488	0.125007	0.6953774	0.8413054
ENSG00000184828	ZBTB7C	18	45745083	A	G	0.180915	0.0222554	0.0175935	0.2099999	0.157922	0.0120091	1.70E-39	0.140926	0.11192	0.2079703	0.6089268
ENSG00000184838	PRR16	5	119911500	A	G	0.131213	-0.0521212	0.0207953	0.0129999	-0.186735	0.0119313	3.28E-55	0.279119	0.112782	0.01332889	0.1956289
ENSG00000184840	TMED9	5	177021142	T	C	0.444334	-0.00477439	0.01428	0.4100001	-0.464561	0.0082744	0	0.0102772	0.0307392	0.7381267	0.7846792
ENSG00000184857	TMEM186	16	8882873	T	C	0.177932	0.025742	0.0194945	0.14	-0.0707686	0.0109366	9.75E-11	-0.363749	0.281145	0.1957309	0.5541287
ENSG00000184860	SDR42E1	16	82038157	A	G	0.151093	0.0326404	0.020818	0.1199999	0.147863	0.0136212	1.88E-27	0.220747	0.142253	0.1207124	0.3069341
ENSG00000184886	NA	17	34893003	T	C	0.466203	-0.0237311	0.0142021	0.1	-0.217138	0.0117165	1.13E-76	0.109291	0.0656713	0.09607121	0.7376346
ENSG00000184887	BTBD6	14	105716128	A	G	0.296223	0.0316258	0.0156601	0.02399993	-0.221037	0.00966866	1.13E-115	-0.143079	0.0711242	0.04425361	0.9570275
ENSG00000184897	H1-10	3	129034367	A	G	0.289264	0.0090312	0.0151899	0.5300002	-0.126618	0.00932756	5.67E-42	-0.0713264	0.120081	0.5525231	0.05767689
ENSG00000184898	RBM43	2	152111423	A	G	0.437376	0.0254676	0.0142006	0.0519996	-0.695147	0.00765807	0	-0.0366363	0.0204322	0.07296217	0.7514084
ENSG00000184900	SUMO3	21	46232113	A	G	0.0964215	-0.0120051	0.0259099	0.59	0.26112	0.0144817	1.11E-72	-0.0459753	0.0992586	0.6432301	0.9106025
ENSG00000184903	IMMP2L	7	110752841	T	C	0.508946	0.0211248	0.0142028	0.089	0.562452	0.00843439	0	0.0375584	0.0252578	0.1370151	0.5303056
ENSG00000184916	JAG2	14	105621239	G	A	0.38668	-0.0016612	0.0150502	0.7300002	-0.0470699	0.00861581	4.68E-08	0.0352922	0.319807	0.912128	0.7248607
ENSG00000184922	FMNL1	17	43311749	G	C	0.32008	-0.00197605	0.0154752	0.7300002	0.0692203	0.00867205	1.44E-15	-0.0285473	0.223593	0.8984061	0.714374
ENSG00000184923	NUTM2A	10	88990058	G	A	0.191849	-0.0166775	0.0187201	0.2200002	-0.786638	0.025908	1.70E-202	0.021201	0.0238078	0.3731953	0.4240475
ENSG00000184925	LCN12	9	139846976	T	G	0.437376	0.0279437	0.0143336	0.07799917	0.0889901	0.00946039	5.12E-21	0.314009	0.164492	0.05626756	0.5103596
ENSG00000184939	ZFP90	16	68586984	G	A	0.208748	-0.00229801	0.0167105	0.8700001	-0.844498	0.00824115	0	0.00272115	0.0197875	0.8906207	0.9134831
ENSG00000184956	MUC6	11	1024763	T	C	0.121272	-0.00995552	0.0214193	0.5500004	0.104344	0.0157815	3.80E-11	-0.0954105	0.205782	0.6429001	0.969738
ENSG00000184967	NOC4L	12	132633003	T	C	0.106362	0.00282161	0.0223857	0.81	-0.279797	0.016007	2.05E-68	-0.0100845	0.0800091	0.8996987	0.856467
ENSG00000184979	USP18	22	18646415	T	C	0.028827	-0.00672804	0.0437702	0.8700001	0.56326	0.0327021	1.75E-66	-0.0119448	0.0777117	0.877841	0.8461214
ENSG00000184983	NDUFA6	22	42484244	A	G	0.0298211	-0.01606	0.0373714	0.5199996	1.11146	0.0226811	0	-0.0144495	0.0336251	0.6673965	0.03381283
ENSG00000184985	SORCS2	4	7469409	C	A	0.284294	0.0110649	0.0153972	0.5400003	0.12984	0.00914675	9.81E-46	0.0852196	0.118738	0.4729352	0.4054018
ENSG00000184986	TMEM121	14	105994739	C	A	0.254473	-0.0201949	0.0165913	0.17	-0.341219	0.00977822	8.61E-267	0.0591846	0.0486532	0.2238104	0.2506311
ENSG00000184988	TMEM106A	17	41367957	A	C	0.366799	-0.0185896	0.015026	0.1499999	-0.191311	0.0106198	1.50E-72	0.0971694	0.0787271	0.2171079	0.3213986
ENSG00000184990	SIVA1	14	105227134	C	T	0.106362	-0.00949643	0.0218567	0.6499995	-0.276484	0.0159391	2.11E-67	0.0343472	0.0790772	0.6640345	0.574558
ENSG00000184992	BRI3BP	12	125497011	G	A	0.34493	-0.00113033	0.0152056	0.99	0.285548	0.0084755	7.83E-249	-0.00395846	0.0532507	0.9407428	0.516614
ENSG00000185000	DGAT1	8	145545263	G	A	0.501988	0.00787508	0.0143995	0.5	0.357934	0.0145614	2.02E-133	0.0220015	0.0402394	0.5845407	0.9154481
ENSG00000185009	AP3M1	10	75896172	C	T	0.153082	0.0150789	0.020423	0.35	0.431614	0.0110367	0	0.0349361	0.0473262	0.4603943	0.8590558
ENSG00000185015	CA13	8	86164559	A	G	0.335984	0.00208118	0.0147292	0.8600001	-0.209062	0.00827314	6.83E-141	-0.00995483	0.0704547	0.8876377	0.06512799
ENSG00000185019	UBOX5	20	3114530	G	T	0.429423	-0.00601921	0.0142012	0.9400001	0.236506	0.00783726	4.73E-200	-0.0254506	0.0600519	0.6717041	0.60293
ENSG00000185022	MAFF	22	38605203	C	G	0.425447	-0.0032381	0.0143985	0.8200001	0.13232	0.0080161	3.28E-61	-0.0244717	0.108826	0.8220799	0.1586004
ENSG00000185024	BRF1	14	105728774	C	T	0.322068	0.0271437	0.0153155	0.04200007	0.103679	0.00872324	1.41E-32	0.261805	0.149353	0.07961555	0.3035729
ENSG00000185031	NA	1	65451030	G	A	0.237575	0.00943332	0.016688	0.56	-0.0752355	0.0109625	6.74E-12	-0.125384	0.222561	0.5731844	0.6334743
ENSG00000185033	SEMA4B	15	90738373	A	G	0.32505	-0.00722912	0.0155336	0.7400005	0.254997	0.00849485	5.75E-198	-0.0283499	0.0609242	0.6416951	0.1809413
ENSG00000185041	NA	5	17498635	T	C	0.414513	0.00580209	0.0145945	0.8600001	-0.0716783	0.00992004	4.99E-13	-0.0809463	0.203919	0.6914023	0.8739041
ENSG00000185043	CIB1	15	90775243	T	C	0.519881	0.000588152	0.0141916	0.7800007	0.720253	0.00687594	0	0.00081659	0.0197036	0.9669422	0.7610339
ENSG00000185049	NELFA	4	2014035	T	C	0.131213	0.0268748	0.0198037	0.1499999	-0.0872423	0.0145661	2.11E-09	-0.308048	0.23275	0.1856651	0.579404

ENSG00000185052	SLC24A3	20	19448435	T	G	0.355865	0.00177106	0.0148102	0.8600001	-0.36737	0.00811382	0	-0.00482092	0.0403143	0.9048132	0.8194068
ENSG00000185065	NA	22	19436522	C	T	0.182903	0.0109612	0.0181892	0.4899999	-0.347402	0.0257931	2.39E-41	-0.0315519	0.0524102	0.5471614	0.7054088
ENSG00000185068	NA	6	158602202	G	C	0.430417	-0.0102397	0.0142948	0.3900004	0.385628	0.00828792	0	-0.0265533	0.0370733	0.4738441	0.5722304
ENSG00000185085	INTS5	11	62417547	G	A	0.270378	-0.0131923	0.0152819	0.35	0.513677	0.00873503	0	-0.0256821	0.0297532	0.388044	0.4104354
ENSG00000185087	NA	15	99019001	A	C	0.22167	-0.0266452	0.0179338	0.1900002	-0.174175	0.00970347	4.82E-72	0.152979	0.103316	0.1386893	0.4548003
ENSG00000185088	RPS27L	15	63434145	C	T	0.0795229	0.0237445	0.0247387	0.29	-0.158067	0.0128562	9.64E-35	-0.150217	0.156983	0.3386174	0.1670376
ENSG00000185090	MANEAL	1	38263141	G	A	0.44334	-0.00819227	0.014342	0.4299995	0.0554317	0.008119	8.65E-12	-0.14779	0.259637	0.5692071	0.717416
ENSG00000185100	ADSS1	14	105202092	A	G	0.139165	0.0418721	0.0205026	0.03699985	0.136108	0.0132975	1.37E-24	0.30764	0.153604	0.04519827	0.2460419
ENSG00000185101	ANO9	11	429972	C	A	0.367793	-0.00653595	0.0146288	0.4899999	0.319624	0.0106706	3.95E-197	-0.0204489	0.0457738	0.6550651	0.41357
ENSG00000185104	FAF1	1	51165542	C	A	0.0914513	0.0128019	0.0229586	0.64	-0.142199	0.011713	6.47E-34	-0.0900283	0.161625	0.5775132	0.08803944
ENSG00000185112	FAM43A	3	194408192	T	G	0.404573	0.00597689	0.0144985	0.7300002	-0.499986	0.00830941	0	-0.0119541	0.0289985	0.6801692	0.427203
ENSG00000185115	NSMCE3	15	29561193	T	C	0.202783	-6.62E-05	0.0184167	0.99	0.0620439	0.0105116	3.58E-09	-0.0010667	0.296834	0.9971327	0.5428849
ENSG00000185122	HSF1	8	145526832	G	A	0.501988	0.00787508	0.0143995	0.5	-0.0891607	0.0119743	9.62E-14	-0.0883245	0.161936	0.5854576	0.8778182
ENSG00000185127	C6orf120	6	170104317	A	G	0.137177	-0.0228809	0.0209667	0.2	0.312306	0.0111252	2.16E-173	-0.0732644	0.0671859	0.2755046	0.5959722
ENSG00000185128	NA	17	36289443	T	C	0.208748	0.0243717	0.0180968	0.2	-0.390267	0.0230845	4.06E-64	-0.0624488	0.0465172	0.1794378	0.8402634
ENSG00000185156	MFSD6L	17	8701551	G	C	0.470179	0.00828335	0.0143451	0.5099998	-0.134371	0.0088031	1.33E-52	-0.0616456	0.106834	0.563925	0.2794506
ENSG00000185158	LRRC37B	17	30357707	G	A	0.154076	0.0252735	0.0203618	0.28	0.275041	0.012616	2.27E-105	0.09189	0.0741518	0.2152661	0.05262653
ENSG00000185163	DDX51	12	132625009	G	A	0.0675944	-0.00650221	0.0304699	0.6300007	0.184449	0.0152917	1.68E-33	-0.0352521	0.16522	0.8310427	0.901283
ENSG00000185168	NA	17	79280309	C	T	0.363817	-0.00530292	0.0149804	0.9400001	-0.270551	0.0166239	1.49E-59	0.0196005	0.0553831	0.7234087	0.5701015
ENSG00000185187	SIGIRR	11	411585	T	C	0.217694	0.00943751	0.0179242	0.4500005	-0.344847	0.0108579	2.32E-221	-0.0273672	0.0519844	0.5985749	0.2515689
ENSG00000185189	NRBP2	8	144919982	G	A	0.0318091	0.0996283	0.037487	0.005899973	0.581086	0.0282179	3.18E-94	0.171452	0.0650471	0.008393537	0.1161214
ENSG00000185198	PRSS57	19	690490	C	T	0.397614	-0.00525962	0.0145193	0.7400005	-0.40103	0.0105603	0	0.0131153	0.0362067	0.7171773	0.5442164
ENSG00000185201	IFITM2	11	311451	G	C	0.324056	-0.0237068	0.0155301	0.1299999	-0.245634	0.0108357	9.06E-114	0.0965129	0.0633679	0.1277444	0.8537074
ENSG00000185215	TNFAIP2	14	103596777	G	A	0.248509	0.031651	0.0170924	0.07399971	0.186655	0.0102349	2.62E-74	0.169569	0.0920429	0.0654333	0.8515937
ENSG00000185219	ZNF445	3	44500212	T	G	0.230616	-0.0021125	0.0170951	0.7899998	-0.0632336	0.00966312	6.00E-11	0.0334079	0.270397	0.9016705	0.3602235
ENSG00000185236	RAB11B	19	8462091	T	C	0.328032	-0.0134431	0.0149323	0.2099999	0.0633116	0.00839721	4.71E-14	-0.212332	0.23753	0.3713648	0.4227726
ENSG00000185238	PRMT3	11	20469958	T	C	0.176938	0.00614082	0.019529	0.81	0.122935	0.0105794	3.25E-31	0.0499519	0.158915	0.7532695	0.8220511
ENSG00000185245	GP1BA	17	4836958	C	T	0.207753	-0.0085888	0.018185	0.4400003	0.0971946	0.0100672	4.70E-22	-0.0883671	0.187323	0.6371144	0.7757532
ENSG00000185246	PRPF39	14	45569393	A	T	0.0626243	-0.0340925	0.0325907	0.16	0.136317	0.0179544	3.14E-14	-0.250097	0.241339	0.3000665	0.7162168
ENSG00000185250	PPIL6	6	109736896	A	G	0.300199	0.000329602	0.0158084	0.9699999	0.0600151	0.00998489	1.85E-09	0.00549198	0.263408	0.9833656	0.7156023
ENSG00000185252	ZNF74	22	20755575	C	T	0.426441	-0.0263382	0.0144491	0.07699987	0.216061	0.00795286	1.56E-162	-0.121902	0.0670255	0.06895154	0.5395189
ENSG00000185261	KIAA0825	5	93721490	G	A	0.473161	0.00131102	0.014226	0.83	0.139645	0.00881221	1.48E-56	0.00938821	0.101874	0.9265748	0.6499312
ENSG00000185262	UBALD2	17	74264329	T	C	0.297217	-0.0265433	0.0152583	0.09099971	0.148023	0.00945859	3.34E-55	-0.179319	0.103715	0.08381831	0.2835221
ENSG00000185271	KLHL33	14	20900385	T	C	0.525845	-0.00505071	0.014245	0.6899999	-0.0588921	0.0106353	3.07E-08	0.085762	0.242378	0.7234621	0.9678963
ENSG00000185272	RBM11	21	15594572	A	G	0.434394	-0.0106606	0.0142675	0.6100002	0.291392	0.00783493	9.40E-303	-0.0365851	0.0489731	0.455037	0.4335775
ENSG00000185298	CCDC137	17	79637164	C	T	0.0586481	-0.00880233	0.0330739	0.7300002	-0.387909	0.0182578	3.57E-100	0.0226917	0.0852687	0.7901465	0.7381341
ENSG00000185305	ARL15	5	53393093	T	C	0.15507	-0.00113278	0.0195332	0.9599999	0.210736	0.0109858	5.17E-82	-0.00537534	0.0926906	0.9537548	0.6711941
ENSG00000185324	CDK10	16	89754958	T	C	0.0506958	-0.0101644	0.0266755	0.6700003	-0.944769	0.0170061	0	0.0107586	0.0282356	0.7031811	0.08370404
ENSG00000185338	SOCS1	16	11349149	T	C	0.296223	-0.0295084	0.0158261	0.03599979	0.0788231	0.00874102	1.92E-19	-0.374362	0.205027	0.0678627	0.5043521
ENSG00000185339	TCN2	22	31013045	A	G	0.44334	0.00085551	0.0143813	0.95	0.383414	0.00774185	0	0.0022313	0.0375086	0.9525637	0.1734929
ENSG00000185340	GAS2L1	22	29705673	G	C	0.22664	0.0165644	0.0172773	0.2300001	0.144509	0.0101077	2.28E-46	0.114625	0.119827	0.3387746	0.6910701
ENSG00000185344	ATP6V0A2	12	124221207	A	T	0.374751	0.0107864	0.0143922	0.3400001	0.10887	0.00818009	2.05E-40	0.0990755	0.132405	0.4542937	0.3037389
ENSG00000185347	TEDC1	14	105961052	G	A	0.253479	-0.0211038	0.0165192	0.16	-0.182268	0.00982144	7.00E-77	0.115784	0.0908458	0.2024809	0.2731783
ENSG00000185359	HGS	17	79660262	A	G	0.027833	-0.074744	0.0468219	0.1100001	0.39365	0.0309858	5.60E-37	-0.189874	0.119878	0.1132183	0.8774978
ENSG00000185361	TNFAIP8L1	19	4647555	G	T	0.488072	-0.0051279	0.0142501	0.99	0.0851572	0.00922813	2.76E-20	-0.0602169	0.167466	0.7191636	0.8219882
ENSG00000185379	RAD51D	17	33437676	C	A	0.110338	-0.0270491	0.02296	0.2200002	0.285671	0.0142878	6.21E-89	-0.0946862	0.0805116	0.2395721	0.8037871
ENSG00000185404	SP140L	2	231230173	T	C	0.322068	-0.0187364	0.0155915	0.1199999	-0.66935	0.00766625	0	0.0279919	0.0232957	0.2295215	0.2285642
ENSG00000185418	TARS3	15	102229304	T	A	0.483101	-0.000559281	0.0142249	0.7800007	-0.207025	0.00789598	1.61E-151	0.00270152	0.0687112	0.9686377	0.6416443
ENSG00000185420	SMYD3	1	246291628	T	A	0.137177	0.0293781	0.0221094	0.2599998	0.265071	0.0118606	1.24E-110	0.110831	0.0835567	0.1847012	0.8105427
ENSG00000185432	TMT1A	12	51321777	C	A	0.328032	-0.022227	0.0148372	0.28	-0.284907	0.00840537	7.66E-252	0.0780148	0.0521281	0.1344979	0.06969167

ENSG00000185436	IFNLR1	1	24497548	T	C	0.45328	0.00649685	0.0145699	0.5400003	-0.186671	0.00908701	8.96E-94	-0.0348038	0.0780697	0.6557388	0.8824036
ENSG00000185442	FAM174B	15	93256893	T	C	0.399602	-0.00759508	0.0145538	0.6200004	-0.194429	0.00881701	9.22E-108	0.0390635	0.0748749	0.6018679	0.9276051
ENSG00000185475	TMEM179B	11	62556382	A	G	0.364811	-0.00830764	0.014967	0.59	0.113924	0.00823213	1.48E-43	-0.0729223	0.131482	0.5791561	0.9061314
ENSG00000185477	GPRIN3	4	90197295	A	C	0.513917	-0.0249564	0.0142027	0.1199999	0.12386	0.0079323	5.79E-55	-0.201489	0.115391	0.08078709	0.01631975
ENSG00000185480	PARPBP	12	102552627	C	A	0.215706	0.0144806	0.0171778	0.1900002	0.082313	0.0097352	2.79E-17	0.175921	0.209723	0.4015677	0.9825388
ENSG00000185482	STAC3	12	57641106	G	A	0.256461	-0.0193381	0.0157807	0.4899999	-0.101268	0.00876265	6.82E-31	0.190959	0.156704	0.2229976	0.9544195
ENSG00000185483	ROR1	1	64443437	G	A	0.210736	0.000179713	0.0171683	0.8700001	0.0728106	0.00997788	2.94E-13	0.00246823	0.235794	0.9916481	0.6562082
ENSG00000185485	NA	3	195701904	A	G	0.45825	0.0215894	0.0144433	0.1900002	0.521239	0.0164703	8.31E-220	0.0414194	0.0277405	0.1354098	0.2643319
ENSG00000185499	MUC1	1	155160503	T	G	0.128231	-0.0161879	0.0228571	0.4600002	-0.243883	0.0137644	3.02E-70	0.0663758	0.0937965	0.4791584	0.6703618
ENSG00000185504	FAAP100	17	79513949	A	G	0.254473	0.0241883	0.0167724	0.2200002	-0.159334	0.0112696	2.20E-45	-0.151809	0.105812	0.1513713	0.7997707
ENSG00000185507	IRF7	11	614276	A	C	0.171968	0.000769082	0.0188761	1	-0.230781	0.010408	6.20E-109	-0.00333251	0.0817922	0.9675003	0.8538107
ENSG00000185513	L3MBTL1	20	42157955	T	C	0.428429	0.0110069	0.0145744	0.3400001	-0.0564617	0.00896304	2.99E-10	-0.194944	0.259977	0.4533439	0.4158987
ENSG00000185522	LMNTD2	11	557817	A	C	0.0725646	-0.0310947	0.0318305	0.25	0.7628	0.0389493	2.10E-85	-0.0407639	0.0417804	0.3292276	0.4220262
ENSG00000185527	PDE6G	17	79623815	G	A	0.0447316	0.0304674	0.0353455	0.3599996	0.24834	0.0215704	1.13E-30	0.122684	0.142725	0.3900194	0.2608158
ENSG00000185532	PRKG1	10	53404527	A	G	0.0815109	-0.0261379	0.029751	0.35	0.267234	0.0237994	2.95E-29	-0.0978091	0.11167	0.3810961	0.7913973
ENSG00000185559	DLK1	14	101196790	C	T	0.364811	0.00256484	0.0150438	1	-0.144174	0.00896056	3.01E-58	-0.0177899	0.104351	0.8646311	0.6552582
ENSG00000185585	OLFML2A	9	127558300	G	A	0.32505	-0.0105947	0.0156472	0.58	0.0726967	0.00999707	3.55E-13	-0.145738	0.21617	0.5001953	0.4860213
ENSG00000185591	SP1	12	53792095	A	G	0.150099	0.00349774	0.0196543	0.83	0.404069	0.0160705	1.66E-139	0.00865629	0.0486422	0.8587555	0.06079669
ENSG00000185596	NA	15	102509062	G	A	0.240557	-0.00088021	0.0178048	0.6800001	0.557858	0.0194452	5.26E-181	-0.00157784	0.0319164	0.9605714	0.5957568
ENSG00000185608	MRPL40	22	19421511	G	C	0.125249	0.0230541	0.022264	0.3599996	-0.232891	0.0127153	6.19E-75	-0.098991	0.0957511	0.3012124	0.5353268
ENSG00000185614	INKA1	3	49841575	T	C	0.466203	0.0225386	0.0142442	0.08700015	-0.137057	0.00886776	6.92E-54	-0.164447	0.104473	0.1154706	0.06512294
ENSG00000185619	PCGF3	4	731982	A	G	0.358847	-0.00497664	0.0147514	0.6999999	0.381864	0.00848263	0	-0.0130325	0.038631	0.7358472	0.3647739
ENSG00000185621	LMLN	3	197728831	G	A	0.188867	-0.0150141	0.0188725	0.4	0.176985	0.0114059	2.66E-54	-0.0848326	0.106773	0.4268981	0.465603
ENSG00000185627	PSMD13	11	244764	G	A	0.269384	-0.0103348	0.0165022	0.4500005	0.393898	0.00905046	0	-0.0262373	0.041899	0.5311817	0.3493175
ENSG00000185630	PBX1	1	164696677	A	C	0.223658	0.00389415	0.0162709	0.6499995	0.147489	0.00916938	3.25E-58	0.0264029	0.110331	0.8108686	0.2059014
ENSG00000185633	NDUFA4L2	12	57631592	G	T	0.417495	-0.00494746	0.0142574	0.98	0.0464708	0.00843451	3.60E-08	-0.106464	0.307412	0.7290994	0.4481917
ENSG00000185640	KRT79	12	53221636	C	A	0.245527	0.0173537	0.0156643	0.14	0.152792	0.00891667	8.05E-66	0.113577	0.102734	0.2689247	0.385278
ENSG00000185650	ZFP36L1	14	69258783	T	A	0.0765408	-0.00274158	0.0267522	0.98	-0.307915	0.0148333	1.03E-95	0.00890368	0.0868828	0.9183762	0.1523857
ENSG00000185651	UBE2L3	22	21941029	C	A	0.193837	-0.0189789	0.017976	0.1299999	0.731873	0.00892525	0	-0.025932	0.0245637	0.2911037	0.754476
ENSG00000185658	BRWD1	21	40624793	G	A	0.084493	0.0149116	0.0228148	0.5300002	0.347631	0.0136818	2.05E-142	0.0428949	0.0656511	0.5135132	0.6781087
ENSG00000185664	PMEL	12	56357495	A	C	0.201789	0.00550354	0.0177995	0.8800001	0.113803	0.0114045	1.89E-23	0.0483601	0.156481	0.757285	0.4600271
ENSG00000185666	SYN3	22	33181448	T	C	0.363817	0.00125825	0.0148564	0.7700005	0.0620488	0.00850123	2.90E-13	0.0202784	0.239447	0.9325091	0.9915522
ENSG00000185669	SNAI3	16	88748495	T	C	0.309145	0.00295483	0.0148623	0.8600001	0.464003	0.0103619	0	0.00636812	0.0320309	0.84241	0.2129208
ENSG00000185670	ZBTB3	11	62518725	G	A	0.0427435	-0.0877781	0.0343185	0.004799986	0.112898	0.0181654	5.13E-10	-0.777501	0.328714	0.01801656	0.3364363
ENSG00000185684	NA	12	132590928	G	T	0.11332	0.00489059	0.0212249	0.81	-0.3671	0.0118213	1.00E-211	-0.0133222	0.0578194	0.8177722	0.6728074
ENSG00000185689	TEX56P	6	4105312	G	T	0.415507	-0.012155	0.014441	0.4199997	-0.0699978	0.00835137	5.22E-17	0.173648	0.207344	0.4023181	0.6318424
ENSG00000185697	MYBL1	8	67500446	C	T	0.501988	-0.0133728	0.0141996	0.4700002	0.12433	0.00979302	6.24E-37	-0.107559	0.114523	0.3476319	0.272711
ENSG00000185710	SMG1P4	16	21910568	T	G	0.00497018	0.0539894	0.0906383	0.7099994	1.38433	0.106345	9.74E-39	0.0390004	0.065543	0.55182	0.9225647
ENSG00000185721	DRG1	22	31860117	G	A	0.0795229	0.00373711	0.0290306	0.7899998	-0.217242	0.0153795	2.65E-45	-0.0172025	0.133638	0.8975755	0.9174199
ENSG00000185722	ANKFY1	17	4117237	G	T	0.38171	-0.000405578	0.0145987	0.9	0.100057	0.00820889	3.57E-34	-0.00405349	0.145905	0.9778362	0.7227608
ENSG00000185728	YTHDF3	8	64103229	A	G	0.188867	-0.0132437	0.0185268	0.4199997	-0.262068	0.0109803	6.71E-126	0.0505354	0.0707264	0.4749059	0.9791168
ENSG00000185730	ZNF696	8	144376038	T	C	0.483101	-0.00651288	0.014209	0.6999999	0.484174	0.00815502	0	-0.0134515	0.0293478	0.6467015	0.9339966
ENSG00000185736	ADARB2	10	1503871	C	T	0.356859	0.0277515	0.0149062	0.04600023	0.604643	0.00758075	0	0.0458974	0.0246596	0.06271098	0.4908322
ENSG00000185745	IFIT1	10	91158024	A	C	0.207753	-0.0106849	0.018201	0.4299995	-0.174196	0.00966895	1.46E-72	0.0613385	0.104541	0.5573792	0.4907936
ENSG00000185760	KCNQ5	6	73620047	A	C	0.380716	0.0120195	0.0146167	0.4100001	0.170083	0.00819307	1.01E-95	0.0706683	0.0860059	0.4112656	0.6363782
ENSG00000185761	ADAMTSL5	19	1509310	A	G	0.136183	0.0198184	0.0233156	0.4400003	0.209496	0.0181134	6.14E-31	0.0946005	0.111594	0.3965941	0.03902677
ENSG00000185774	KCNIP4	4	21340330	C	T	0.464215	0.0188108	0.0143108	0.2999998	0.0495451	0.00811816	1.04E-09	0.37967	0.295467	0.1987985	0.9109665
ENSG00000185798	WDR53	3	196288300	C	A	0.451292	0.00934303	0.0142335	0.3700002	0.123936	0.00876826	2.32E-45	0.075386	0.114969	0.5120142	0.4671234
ENSG00000185800	DMWD	19	46291132	T	A	0.468191	0.0187562	0.014217	0.2	0.243306	0.00784476	3.37E-211	0.0770889	0.0584854	0.1874743	0.6744287
ENSG00000185808	PIGP	21	38440308	A	G	0.351889	0.015187	0.0150229	0.3800004	0.691895	0.00741183	0	0.0219499	0.021714	0.3120815	0.4325469

ENSG00000185811	IKZF1	7	50408259	T	C	0.0417495	-0.0147943	0.034909	0.58	0.987119	0.018029	0	-0.0149873	0.0353656	0.6717237	0.3914954
ENSG00000185818	NAT8L	4	2066027	C	T	0.286282	-0.00040416	0.0158077	0.8	0.148945	0.0115395	4.09E-38	-0.00271348	0.106131	0.9796025	0.533684
ENSG00000185829	ARL17A	17	44625578	C	A	0.230616	0.00837895	0.0162367	0.4299995	0.371456	0.0193356	2.99E-82	0.022557	0.0437267	0.6059494	0.9689969
ENSG00000185838	GNB1L	22	19806604	T	C	0.116302	0.00324878	0.0226295	0.99	0.792939	0.0124115	0	0.00409714	0.0285388	0.8858451	0.7224629
ENSG00000185862	EVI2B	17	29635957	A	G	0.2833	-0.000683674	0.0154399	0.95	-0.117582	0.00853514	3.54E-43	0.00581445	0.131313	0.9646816	0.3636466
ENSG00000185864	NPIPB4	16	21869019	T	C	0.027833	-0.0136015	0.0382639	0.5199996	-0.688312	0.0505399	3.08E-42	0.0197606	0.0556098	0.7223315	0.4322714
ENSG00000185869	ZNF829	19	37393109	C	G	0.137177	-0.0599819	0.0212389	0.003899959	0.0737057	0.0134588	4.34E-08	-0.813803	0.324219	0.01207161	0.5878576
ENSG00000185875	THNSL1	10	25310590	C	A	0.34493	-0.00760193	0.014879	0.5400003	-0.135061	0.00828688	1.02E-59	0.056285	0.110219	0.609585	0.7805716
ENSG00000185880	TRIM69	15	45040606	T	C	0.107356	-0.0152229	0.0225535	0.59	-0.47553	0.0128078	9.82E-302	0.0320125	0.0474359	0.4997667	0.2491416
ENSG00000185885	IFITM1	11	314389	G	C	0.324056	-0.0237068	0.0155301	0.1299999	-0.353669	0.0106767	1.28E-240	0.0670309	0.0439579	0.127287	0.9325082
ENSG00000185897	FFAR3	19	35850374	T	A	0.237575	0.0302086	0.0161029	0.0329997	0.347786	0.0153173	3.96E-114	0.0868597	0.0464589	0.06153909	0.686965
ENSG00000185899	TAS2R60	7	143141024	A	G	0.362823	0.00931651	0.0148779	0.6499995	0.167972	0.00936057	5.29E-72	0.0554648	0.0886278	0.531435	0.6512906
ENSG00000185905	C16orf54	16	29755555	G	A	0.166998	0.0384091	0.0185371	0.01899984	0.40891	0.0103567	0	0.0939304	0.0453953	0.03853061	0.1181043
ENSG00000185909	KLHDC8B	3	49211480	G	A	0.11332	-0.0175044	0.0228462	0.28	0.34586	0.0127809	2.87E-161	-0.0506113	0.0660827	0.4437493	0.5336694
ENSG00000185917	SETD4	21	37429263	C	T	0.338966	-0.0151938	0.0149411	0.28	0.397255	0.00811265	0	-0.0382469	0.0376189	0.3092991	0.154333
ENSG00000185920	PTCH1	9	98242300	T	G	0.100398	-0.0108618	0.0237858	0.59	0.488162	0.0138269	4.90E-273	-0.0222504	0.0487293	0.6479497	0.915885
ENSG00000185924	RTN4RL1	17	1883738	A	G	0.171968	0.00232693	0.0206882	0.89	0.127414	0.0174674	3.00E-13	0.0182627	0.162389	0.9104564	0.4619589
ENSG00000185928	NA	16	29829522	A	G	0.0815109	-0.0120384	0.0233688	0.7700005	0.154578	0.0179101	6.09E-18	-0.0778789	0.151447	0.6070889	0.4195493
ENSG00000185933	CALHM1	10	105215894	T	C	0.482107	0.0198519	0.0142655	0.14	-0.064592	0.00800139	6.88E-16	-0.307343	0.224113	0.1702582	0.0412187
ENSG00000185946	RNPC3	1	104083087	T	C	0.298211	-0.0135066	0.0160465	0.35	-0.0794193	0.00922086	7.12E-18	0.170067	0.20301	0.4021849	0.4311645
ENSG00000185950	IRS2	13	110422549	T	C	0.173956	-0.0205997	0.0193999	0.1499999	0.1199	0.010469	2.28E-30	-0.171808	0.162495	0.2903697	0.04189842
ENSG00000185955	SPACDR	7	100058066	G	A	0.195825	0.0371888	0.0178625	0.04099964	0.105502	0.0101954	4.27E-25	0.352495	0.172703	0.04124601	0.4157217
ENSG00000185963	BICD2	9	95500369	C	T	0.265408	0.0131202	0.0157172	0.33	0.432128	0.0085756	0	0.0303619	0.0363766	0.4039134	0.5025738
ENSG00000185986	NA	5	1581686	G	A	0.368787	0.0105603	0.0146888	0.5500004	0.107602	0.009141	5.48E-32	0.0981419	0.136764	0.4730049	0.6120603
ENSG00000185989	RASA3	13	114822640	A	G	0.309145	0.0191578	0.0155947	0.17	0.21888	0.00893636	1.75E-132	0.0875265	0.0713373	0.2198455	0.4544725
ENSG00000186001	LRCH3	3	197566702	A	G	0.116302	0.0115191	0.0227122	0.64	-0.140699	0.0137273	1.19E-24	-0.0818705	0.161622	0.6124657	0.1416598
ENSG00000186010	NDUFA13	19	19635415	G	A	0.16998	-0.0439179	0.0190131	0.02199986	0.192874	0.0128241	4.02E-51	-0.227702	0.0997334	0.0224241	0.2681155
ENSG00000186017	ZNF566	19	36958412	T	C	0.215706	0.0386572	0.016677	0.01199997	-0.157489	0.00963035	4.11E-60	-0.24546	0.106952	0.02173002	0.06117917
ENSG00000186020	ZNF529	19	37060927	T	C	0.34493	0.0110709	0.0148532	0.59	-0.204702	0.00820852	2.90E-137	-0.0540829	0.0725923	0.4562585	0.02787694
ENSG00000186026	ZNF284	19	44585031	A	G	0.107356	0.0107727	0.0213922	0.5	-0.159975	0.0128376	1.21E-35	-0.0673399	0.133831	0.6148447	0.4098662
ENSG00000186047	DLEU7	13	51351609	T	C	0.313121	0.00422572	0.0149263	0.8700001	0.0853526	0.00835578	1.70E-24	0.049509	0.174945	0.7771791	0.7657162
ENSG00000186049	KRT73	12	53006848	G	T	0.247515	0.0183861	0.0162863	0.2599998	-0.74206	0.00858184	0	-0.0247771	0.0219493	0.2589678	0.5171078
ENSG00000186056	MATN1-AS1	1	31195307	G	A	0.365805	0.0160518	0.0147631	0.1800002	-0.0863215	0.0112877	2.05E-14	-0.185954	0.172745	0.2817188	0.9151606
ENSG00000186063	AIDA	1	222863953	G	A	0.0228628	-0.0326747	0.0415874	0.5500004	-0.171573	0.0248801	5.35E-12	0.190442	0.243957	0.4350159	0.5848256
ENSG00000186073	CDIN1	15	36987130	C	T	0.392644	0.0177319	0.0145992	0.1299999	-0.151367	0.00905294	9.34E-63	-0.117145	0.0967031	0.2257466	0.3685637
ENSG00000186074	CD300LF	17	72699784	A	C	0.200795	0.00963049	0.0183218	0.6899999	-0.386161	0.00983373	0	-0.0249391	0.0474503	0.5991779	0.2193089
ENSG00000186081	KRT5	12	52911415	G	T	0.370775	0.0252693	0.0152569	0.1199999	-0.201061	0.00930751	1.72E-103	-0.12568	0.0761047	0.09865547	0.3862262
ENSG00000186088	GSAP	7	76992892	C	T	0.446322	-0.00411248	0.0142544	0.8499999	0.52444	0.0090643	0	-0.00784167	0.0271806	0.7729622	0.707153
ENSG00000186104	CYP2R1	11	14906675	A	G	0.380716	0.014342	0.0144474	0.2	0.0678071	0.00821885	1.58E-16	0.211512	0.214603	0.3243317	0.3873221
ENSG00000186105	LRRC70	5	61875918	T	C	0.310139	0.0101693	0.0156378	0.5700002	-0.183242	0.0129544	2.00E-45	-0.0554966	0.0854298	0.5159401	0.3967627
ENSG00000186106	ANKRD46	8	101546996	T	C	0.424453	-0.00494215	0.0143023	0.81	0.0776763	0.00804102	4.46E-22	-0.063625	0.184245	0.7298475	0.9092579
ENSG00000186111	PIP5K1C	19	3665329	G	C	0.159046	-0.0383351	0.0183307	0.02399993	0.237823	0.0118452	1.16E-89	-0.161192	0.0774941	0.03752072	0.8654183
ENSG00000186115	CYP4F2	19	15998882	A	G	0.203777	-0.0110283	0.0185199	0.5500004	0.111238	0.0113534	1.15E-22	-0.0991412	0.166796	0.5522533	0.6730569
ENSG00000186130	ZBTB6	9	125672972	A	G	0.12326	-0.0242317	0.0228731	0.2700001	-0.164686	0.0125618	2.89E-39	0.147139	0.139342	0.2909887	0.3720151
ENSG00000186132	C2orf76	2	120092102	A	T	0.354871	0.00692074	0.0149314	0.64	-0.215575	0.0153327	6.71E-45	-0.0321037	0.0693008	0.6431846	0.6064235
ENSG00000186141	POLR3C	1	145601815	C	T	0.437376	0.0111452	0.0145208	0.4600002	-0.0674671	0.0120284	2.04E-08	-0.165195	0.217234	0.4469876	0.7577489
ENSG00000186152	NA	19	55210533	T	C	0.481113	0.00979811	0.0144108	0.32	0.809672	0.0179801	0	0.0121013	0.0178003	0.4966077	0.7410429
ENSG00000186153	WWOX	16	78689937	A	G	0.389662	0.00756203	0.0144256	0.5199996	-0.186825	0.00800441	1.73E-120	-0.0404766	0.0772342	0.6002251	0.3698422
ENSG00000186162	CIDECP1	3	10061990	A	G	0.393638	-0.0024567	0.0145803	0.9299999	0.0686624	0.00856054	1.05E-15	-0.0357794	0.212394	0.8662235	0.1501099
ENSG00000186166	CENATAC	11	118877676	C	T	0.134195	-0.00801953	0.0206571	0.7800007	0.46326	0.0216908	3.33E-101	-0.0173111	0.0445981	0.6978996	0.2763418

ENSG00000186174	BCL9L	11	118780450	A	C	0.44831	-0.0237733	0.0142562	0.07799917	-0.0468751	0.00799835	4.61E-09	0.507163	0.316204	0.1087337	0.5049783
ENSG00000186184	POLR1D	13	28218225	C	T	0.431412	0.0102177	0.0142428	0.4	-0.399357	0.00765952	0	-0.0255854	0.0356677	0.4731729	0.7897728
ENSG00000186187	ZNRF1	16	75088910	G	T	0.683897	-0.0386537	0.015325	0.008400014	-0.173142	0.0084273	8.46E-94	0.223248	0.0891755	0.01229823	0.584783
ENSG00000186188	FFAR4	10	95338125	C	A	0.357853	-0.00185564	0.0148625	0.91	0.240341	0.00920112	2.12E-150	-0.00772088	0.06184	0.9006404	0.283318
ENSG00000186193	SAPCD2	9	139960810	C	G	0.364811	0.011483	0.0146666	0.4100001	-0.815268	0.0118433	0	-0.0140849	0.0179911	0.4336948	0.1010939
ENSG00000186197	EDARADD	1	236579888	T	C	0.177932	0.000417255	0.0200303	0.89	-0.174983	0.0107609	1.87E-59	-0.00238455	0.11447	0.9833803	0.1723524
ENSG00000186204	CYP4F12	19	15795775	A	G	0.329026	0.00404102	0.0157756	0.9400001	0.326899	0.00843825	0	0.0123617	0.0482593	0.7978344	0.1965041
ENSG00000186205	MTARC1	1	220973918	G	C	0.0447316	-0.0445525	0.0322399	0.1299999	0.753638	0.019751	0	-0.0591166	0.0428071	0.1672789	0.1788644
ENSG00000186222	BLOC1S4	4	6718614	A	G	0.357853	-0.0213063	0.0150339	0.2399999	-0.0884554	0.010215	4.74E-18	0.240871	0.172221	0.16193	0.9192222
ENSG00000186231	KLHL32	6	97480617	A	G	0.219682	-0.0153987	0.0164314	0.3599996	-0.0672544	0.00926267	3.85E-13	0.228962	0.246344	0.3526606	0.6148716
ENSG00000186235	LINC02610	2	239136755	G	A	0.246521	-0.0339434	0.0172519	0.03899959	0.452742	0.0128377	1.90E-272	-0.0749729	0.0381646	0.04947656	0.1797215
ENSG00000186260	MRTFB	16	14262904	C	T	0.101392	0.0164237	0.028239	0.7199992	0.167865	0.015433	1.48E-27	0.0978389	0.168465	0.5613977	0.7991444
ENSG00000186265	BTLA	3	112200611	T	C	0.0904573	-0.00569931	0.0256802	0.6800001	-0.143802	0.0158651	1.26E-19	0.0396331	0.178634	0.824417	0.9937777
ENSG00000186272	ZNF17	19	57927919	G	A	0.292247	0.00620464	0.0163129	0.89	-0.0870463	0.00879628	4.34E-23	-0.0712798	0.187543	0.7038926	0.2089612
ENSG00000186275	NA	1	146420645	A	G	0.5	-0.00515619	0.0142381	0.7099994	0.240591	0.0188267	2.14E-37	-0.0214314	0.0592035	0.7173558	0.9224463
ENSG00000186281	GPAT2	2	96696446	C	A	0.0377734	0.00634219	0.0337755	0.8499999	-0.453676	0.0331329	1.12E-42	-0.0139796	0.0744555	0.851067	0.474258
ENSG00000186283	TOR3A	1	179058835	T	C	0.2833	0.011352	0.0156714	0.4500005	-0.218185	0.0127306	7.65E-66	-0.0520292	0.0718903	0.4692311	0.7495923
ENSG00000186298	PPP1CC	12	111169114	A	G	0.106362	0.0287883	0.0246039	0.1	0.230275	0.0150053	3.76E-53	0.125017	0.107156	0.2433379	0.6378142
ENSG00000186300	ZNF555	19	2850957	A	G	0.109344	-0.0105581	0.0227999	0.5	-0.162404	0.0130449	1.41E-35	0.0650112	0.140487	0.6435389	0.1976698
ENSG00000186301	NA	1	16974491	G	A	0.44831	0.00293984	0.0144033	0.9299999	-0.420004	0.0170181	1.76E-134	-0.00699955	0.0342944	0.8382739	0.7345621
ENSG00000186318	BACE1	11	117171688	C	T	0.464215	-0.000714023	0.0142461	0.99	0.208225	0.00791533	1.61E-152	-0.00342909	0.0684169	0.9600264	0.04725432
ENSG00000186340	THBS2	6	169635007	C	T	0.105368	0.00255272	0.0212538	0.98	-0.207366	0.0129247	6.28E-58	-0.0123102	0.102497	0.9044014	0.9539472
ENSG00000186350	RXRA	9	137270687	A	G	0.294235	-0.000962751	0.0156736	0.9400001	0.0620553	0.0096034	1.03E-10	-0.0155144	0.252586	0.9510229	0.3493967
ENSG00000186352	ANKRD37	4	186319478	A	G	0.301193	-0.00607557	0.0158245	0.4400003	-0.0663159	0.00873037	3.05E-14	0.0916155	0.238927	0.7013899	0.7301864
ENSG00000186354	NA	9	91608416	A	G	0.519881	0.00287848	0.0142147	0.98	0.0659612	0.00795154	1.08E-16	0.043639	0.215565	0.8395728	0.9057013
ENSG00000186364	NUDT17	1	145588288	C	T	0.363817	0.00911053	0.0147716	0.5300002	0.220781	0.0121268	4.63E-74	0.041265	0.0669444	0.5376264	0.2548288
ENSG00000186395	KRT10	17	38976608	C	T	0.183897	-0.0279145	0.0191918	0.14	0.382522	0.0149786	7.50E-144	-0.072975	0.0502531	0.1464604	0.7121978
ENSG00000186399	GOLGA8R	15	30701203	T	C	0.116302	-0.00692773	0.0217693	0.7099994	0.253688	0.0156276	2.93E-59	-0.027308	0.0858277	0.7503539	0.5881619
ENSG00000186407	CD300E	17	72612961	C	A	0.173956	-0.00120137	0.0193735	0.8600001	-0.390638	0.0164413	8.74E-125	0.00307541	0.0495947	0.9505542	0.8356719
ENSG00000186409	CCDC30	1	43024668	C	T	0.351889	0.017534	0.0147119	0.2399999	-0.092016	0.00839654	6.03E-28	-0.190554	0.160827	0.2360815	0.8820496
ENSG00000186431	FCAR	19	55393693	A	G	0.262425	0.00423461	0.016523	0.8499999	0.411377	0.0130833	5.27E-217	0.0102938	0.0401665	0.7977367	0.4502941
ENSG00000186432	KPNA4	3	160248079	C	T	0.475149	0.0136498	0.0142453	0.3100002	-0.155362	0.0120139	2.98E-38	-0.0878583	0.0919427	0.3392859	0.7730111
ENSG00000186446	ZNF501	3	44774831	G	A	0.224652	-0.00941132	0.0174189	0.5500004	0.263622	0.00972542	8.25E-162	-0.0357	0.0660884	0.5890686	0.5652996
ENSG00000186448	ZNF197	3	44658171	G	A	0.224652	-0.00941132	0.0174189	0.5500004	0.33709	0.00968583	2.24E-265	-0.0279193	0.0516806	0.5890395	0.7557819
ENSG00000186468	RPS23	5	81571786	G	A	0.299205	0.00226965	0.0154846	0.9299999	-0.859965	0.00731747	0	-0.00263923	0.0180061	0.8834678	0.6435761
ENSG00000186469	GNG2	14	52369486	G	A	0.102386	-0.0164484	0.0233303	0.3800004	0.218386	0.0127167	4.22E-66	-0.075318	0.106921	0.4811652	0.7247302
ENSG00000186470	BTN3A2	6	26371966	C	A	0.112326	-0.0183525	0.0209564	0.4600002	-1.34457	0.010194	0	0.0136494	0.0155863	0.3811782	0.4301653
ENSG00000186480	INSIG1	7	155095715	A	T	0.492048	0.00482804	0.0142536	0.8499999	-0.431533	0.00759725	0	-0.0111881	0.0330308	0.734822	0.2945359
ENSG00000186487	MYT1L	2	2063958	G	A	0.430417	-0.00141146	0.0143672	0.8800001	0.167984	0.00817562	8.19E-94	-0.00840232	0.0855279	0.9217412	0.7996457
ENSG00000186496	ZNF396	18	32951981	C	T	0.475149	0.0250265	0.0141685	0.1100001	0.290515	0.00782531	1.11E-301	0.0861454	0.0488255	0.07767273	0.5018117
ENSG00000186501	TMEM222	1	27655771	A	G	0.0516899	-0.0250221	0.039535	0.5400003	0.372248	0.0239613	2.00E-54	-0.0672189	0.106294	0.527135	0.06673388
ENSG00000186517	ARHGAP30	1	161028248	A	G	0.0208748	0.0469692	0.0412728	0.1800002	0.299754	0.0266718	2.64E-29	0.156693	0.138393	0.2575384	0.6073346
ENSG00000186522	SEPTIN10	2	110336171	A	G	0.259443	0.015103	0.0165246	0.3900004	-0.172677	0.00919941	1.32E-78	-0.0874641	0.0958102	0.3613009	0.4405327
ENSG00000186523	FAM86B1	8	12045623	A	G	0.0487078	-0.00875949	0.0342362	0.9199999	0.834446	0.0417958	1.11E-88	-0.0104974	0.041032	0.7980793	0.07505304
ENSG00000186529	CYP4F3	19	15762670	C	T	0.119284	-0.00720931	0.0225908	0.84	-0.447106	0.0122913	1.03E-289	0.0161244	0.0505287	0.7496403	0.7261622
ENSG00000186532	SMYD4	17	1708353	A	G	0.33996	-0.0074998	0.0151159	0.5700002	-0.21492	0.00824069	6.08E-150	0.0348957	0.0703453	0.6198498	0.9489161
ENSG00000186567	CEACAM19	19	45176588	G	A	0.434394	-0.0189227	0.0144786	0.1900002	-0.107475	0.00808433	2.50E-40	0.176067	0.135366	0.1933706	0.3248364
ENSG00000186577	SMIM29	6	34215702	G	C	0.161034	-0.0391147	0.0196258	0.04399973	0.0947928	0.0112696	4.05E-17	-0.412634	0.212771	0.05246131	0.3012206
ENSG00000186583	SPATC1	8	145094257	T	C	0.411531	0.0341133	0.0143959	0.016	-0.174305	0.00930143	2.35E-78	-0.19571	0.0832479	0.01872642	0.242494
ENSG00000186591	UBE2H	7	129531680	C	A	0.116302	-0.00490714	0.0226943	0.7400005	0.147371	0.0139095	3.14E-26	-0.0332979	0.154027	0.8288452	0.1979578

ENSG00000186594	MIR22HG	17	1617636	T	C	0.317097	-0.00225045	0.0158133	0.6999999	0.460335	0.011337	0	-0.00488872	0.0343519	0.8868329	0.4417379
ENSG00000186615	KTN1-AS1	14	56006412	A	G	0.0576541	-0.0305275	0.0244484	0.17	-0.223057	0.0167604	2.06E-40	0.136859	0.110087	0.2137976	0.1826867
ENSG00000186625	KATNA1	6	149943058	T	G	0.338966	-0.0292553	0.0151216	0.03699985	0.195294	0.0182682	1.13E-26	-0.149801	0.0786876	0.05694328	0.1300482
ENSG00000186635	ARAP1	11	72450379	A	G	0.4334	0.00408457	0.0143209	0.9699999	0.065807	0.00877684	6.49E-14	0.0620689	0.217777	0.7756355	0.7302629
ENSG00000186638	KIF24	9	34290788	G	A	0.0526839	-0.0101667	0.0299729	0.6899999	-0.266572	0.017212	4.21E-54	0.0381387	0.112465	0.734523	0.03533682
ENSG00000186642	PDE2A	11	72336410	A	G	0.143141	-0.0100919	0.0204174	0.7700005	0.136224	0.0114003	6.56E-33	-0.074083	0.150009	0.6214071	0.07306809
ENSG00000186648	CARMIL3	14	24530071	G	A	0.471173	-0.00848061	0.014184	0.5400003	0.133387	0.00842411	1.81E-56	-0.0635788	0.106413	0.550191	0.2558663
ENSG00000186654	PRR5	22	45099077	T	C	0.345924	0.00579398	0.0151225	0.8800001	0.111843	0.00900939	2.19E-35	0.0518048	0.135277	0.7017538	0.7370408
ENSG00000186660	ZFP91	11	58367549	C	T	0.240557	0.00701755	0.0167539	0.6600001	0.234565	0.00942991	1.40E-136	0.0299173	0.0714355	0.6753613	0.603598
ENSG00000186666	BCDIN3D	12	50234242	T	G	0.0536779	-0.069488	0.0381752	0.06699926	0.101715	0.0169979	2.18E-09	-0.683161	0.392293	0.0816039	0.4165625
ENSG00000186687	LYRM7	5	130523811	T	C	0.0924453	0.00880951	0.0263943	0.8600001	-0.229004	0.0142717	6.10E-58	-0.0384687	0.115282	0.7386109	0.2221424
ENSG00000186704	NA	7	76640513	A	G	0.192843	0.00690671	0.0194943	0.9199999	0.292271	0.0245038	8.50E-33	0.0236312	0.0667287	0.7232357	0.09188086
ENSG00000186715	MST1L	1	17089068	G	A	0.452286	-0.00884936	0.014312	0.4199997	0.55869	0.0176662	1.68E-219	-0.0158395	0.025622	0.5364439	0.7403676
ENSG00000186716	BCR	22	23591057	G	T	0.377734	-0.0207381	0.0148925	0.1800002	0.523591	0.00963913	0	-0.0396075	0.0284524	0.1639035	0.05891791
ENSG00000186765	FSCN2	17	79499789	A	G	0.361829	0.00129647	0.0147469	0.7499995	-0.0623825	0.00936843	2.76E-11	-0.0207826	0.236415	0.9299505	0.1221382
ENSG00000186777	ZNF732	4	281787	A	G	0.0437376	-0.0868072	0.0365133	0.02	-0.337766	0.0574804	4.20E-09	0.257004	0.116615	0.02753332	0.928595
ENSG00000186792	HYAL3	3	50333580	A	G	0.463221	0.00500559	0.0142016	0.9299999	-0.67389	0.00819014	0	-0.0074279	0.0210742	0.7244908	0.1757226
ENSG00000186806	VSIG10L	19	51840084	A	G	0.139165	0.0311793	0.0205965	0.1299999	0.207024	0.013389	6.24E-54	0.150607	0.0999641	0.1319101	0.6021769
ENSG00000186812	ZNF397	18	32834045	C	T	0.0258449	0.0625628	0.0532987	0.3100002	-0.452888	0.0273417	1.27E-61	-0.138142	0.117981	0.2416477	0.8101598
ENSG00000186814	ZSCAN30	18	32850614	C	T	0.475149	0.0250265	0.0141685	0.1100001	-0.105052	0.00973968	4.01E-27	-0.238229	0.136668	0.08131115	0.395902
ENSG00000186815	TPCN1	12	113697622	C	T	0.10338	0.0207682	0.0229335	0.3400001	0.175166	0.0144081	5.24E-34	0.118563	0.131287	0.3664826	0.8189368
ENSG00000186818	LILRB4	19	55168575	A	G	0.0984095	0.00946254	0.0220618	0.6600001	0.502648	0.0131275	0	0.0188254	0.0438939	0.6680079	0.8725804
ENSG00000186827	TNFRSF4	1	1148112	G	A	0.100398	0.0662177	0.022051	0.000619998	-0.358504	0.0150517	2.16E-125	-0.184706	0.0619953	0.002888622	0.5106432
ENSG00000186831	NA	17	18332668	G	T	0.446322	0.0183253	0.0142148	0.14	-0.574764	0.0176614	2.59E-232	-0.0318832	0.024751	0.1976902	0.157877
ENSG00000186834	HEXIM1	17	43227076	C	A	0.524851	0.00398272	0.014258	0.8499999	0.0708005	0.0085345	1.08E-16	0.0562527	0.201497	0.7801112	0.9298923
ENSG00000186854	TRABD2A	2	85091453	G	A	0.054672	-0.00772046	0.0369712	0.84	-0.814294	0.0263601	1.57E-209	0.00948117	0.0454038	0.8345897	0.5868173
ENSG00000186862	PDZD7	10	102779228	C	A	0.230616	-0.00318988	0.0166357	0.8700001	0.10942	0.0111416	9.16E-23	-0.0291527	0.152065	0.8479674	0.8251181
ENSG00000186866	POFUT2	21	46695828	A	G	0.0526839	-0.00195695	0.0324797	0.99	0.399183	0.0171874	2.52E-119	-0.00490238	0.0813656	0.9519555	0.7275757
ENSG00000186868	MAPT	17	44038724	G	T	0.227634	0.00628136	0.0171064	0.5099998	0.0647023	0.00999449	9.56E-11	0.0970809	0.264811	0.7139142	0.2513147
ENSG00000186889	TMEM17	2	62733192	G	C	0.286282	0.00734822	0.0160922	0.6899999	0.0613307	0.00931144	4.50E-11	0.119813	0.263014	0.6487215	0.695223
ENSG00000186891	TNFRSF18	1	1140479	C	T	0.131213	0.0411752	0.0198454	0.0129999	-0.373647	0.013369	6.82E-172	-0.110198	0.0532588	0.03853627	0.2886446
ENSG00000186908	ZDHHC17	12	77202422	A	G	0.121272	-0.01122	0.0221459	0.6600001	0.378243	0.0116159	1.39E-232	-0.0296635	0.0585565	0.6124504	0.9498595
ENSG00000186918	ZNF395	8	28231660	T	C	0.0666004	-0.00959614	0.0289618	0.7199992	0.177332	0.0192363	3.01E-20	-0.0541139	0.163425	0.7405508	0.4795727
ENSG00000186951	PPARA	22	46593038	G	A	0.276342	-0.0127967	0.0154068	0.33	-0.0785087	0.00929031	2.90E-17	0.162997	0.197189	0.4084611	0.3740637
ENSG00000186994	KANK3	19	8397807	C	T	0.0854871	-0.0610358	0.0236494	0.01099993	0.252608	0.0188753	7.60E-41	-0.241623	0.0953461	0.01127146	0.588099
ENSG00000186998	EMID1	22	29628713	A	G	0.0377734	-0.00113629	0.0482669	0.95	-0.217549	0.0217297	1.36E-23	0.00522314	0.221867	0.9812181	0.5508328
ENSG00000187010	RHD	1	25627910	A	G	0.388668	0.00647447	0.0144501	0.6200004	-0.794959	0.00741569	0	-0.0081444	0.0181773	0.6541148	0.09331695
ENSG00000187017	ESPN	1	6503139	A	G	0.0367793	-0.0161549	0.0361054	0.6200004	0.79107	0.0223728	7.40E-274	-0.0204216	0.0456449	0.6545853	0.6904083
ENSG00000187037	GPR141	7	37798394	C	T	0.33499	-0.0123813	0.0150789	0.4299995	0.245469	0.00836444	2.63E-189	-0.0504394	0.061453	0.4117717	0.8985602
ENSG00000187045	TMPRSS6	22	37483539	T	C	0.291252	-0.0114981	0.0157986	0.56	-0.0679834	0.0101298	1.93E-11	0.169131	0.233752	0.4693419	0.2004407
ENSG00000187049	TMEM216	11	61162747	G	A	0.132207	0.0138644	0.0200606	0.5500004	0.246501	0.0117058	1.93E-98	0.0562448	0.0814253	0.4897202	0.423862
ENSG00000187091	PLCD1	3	38060120	C	G	0.133201	-0.0245047	0.0190688	0.28	0.307943	0.0107818	2.03E-179	-0.0795754	0.0619857	0.1992228	0.8820066
ENSG00000187097	ENTPD5	14	74455407	A	G	0.0775348	0.050501	0.0313769	0.17	-0.311238	0.0171753	2.17E-73	-0.162259	0.10121	0.1088938	0.08371506
ENSG00000187098	MITF	3	69903037	G	A	0.237575	-0.0058213	0.0169483	0.6499995	0.086183	0.00978152	1.24E-18	-0.0675458	0.196804	0.731438	0.7332881
ENSG00000187105	HEATR4	14	73985420	C	T	0.379722	0.00964447	0.0149577	0.5099998	-0.0780179	0.00836276	1.07E-20	-0.123619	0.192179	0.5200629	0.6218155
ENSG00000187109	NAP1L1	12	76458741	A	G	0.414513	-0.0141659	0.0145371	0.2700001	-0.248367	0.0184591	2.88E-41	0.0570361	0.058684	0.3310905	0.8159414
ENSG00000187116	LILRA5	19	54821381	C	A	0.0964215	0.00163678	0.021581	0.4700002	-0.569656	0.0172547	5.01E-239	-0.00287328	0.0378844	0.9395437	0.009593002
ENSG00000187118	CMC1	3	28324854	G	A	0.177932	-0.0230248	0.0187257	0.33	0.298859	0.0112067	1.11E-156	-0.0770423	0.0627239	0.2193426	0.06572987
ENSG00000187134	AKR1C1	10	4980135	G	C	0.132207	0.00714466	0.022149	0.6300007	0.21182	0.0177656	8.98E-33	0.0337299	0.104604	0.7471083	0.8542245
ENSG00000187145	NA	1	150273851	A	T	0.407555	-0.019701	0.0145568	0.1199999	-0.419642	0.0115933	6.81E-287	0.0469471	0.0347128	0.1762336	0.2539722

ENSG00000187147	RNF220	1	44994131	T	C	0.137177	0.00316986	0.0194824	0.83	-0.133782	0.0114297	1.20E-31	-0.0236941	0.145642	0.8707641	0.8895943
ENSG00000187164	SHTN1	10	118764919	C	T	0.269384	0.0129082	0.0164673	0.4400003	1.01872	0.00711794	0	0.0126711	0.016165	0.4331241	0.5539051
ENSG00000187166	H1-7	12	48723412	C	T	0.447316	0.00939351	0.0142856	0.4700002	0.144442	0.008322	1.76E-67	0.0650332	0.0989732	0.5111297	0.4659234
ENSG00000187189	TSPYL4	6	116573206	C	T	0.233598	-0.00931492	0.0173269	0.5099998	-0.137726	0.00939291	1.12E-48	0.0676336	0.125891	0.5911034	0.6340222
ENSG00000187193	MT1X	16	56717222	C	A	0.417495	0.00360792	0.0143425	0.9400001	0.372336	0.00776492	0	0.00968996	0.0385209	0.8013881	0.4585256
ENSG00000187210	GCNT1	9	79078542	G	A	0.250497	-0.00647848	0.0167621	0.6800001	-0.18131	0.00901564	5.96E-90	0.0357315	0.0924671	0.6991824	0.848947
ENSG00000187231	SESTD1	2	180047968	A	C	0.269384	-0.0278943	0.016265	0.05699936	0.544558	0.00862383	0	-0.0512237	0.0298793	0.08646317	0.5860959
ENSG00000187239	FNBP1	9	132727469	T	C	0.0934394	-0.0101354	0.0226584	0.6700003	0.582984	0.0143749	0	-0.0173854	0.0388686	0.6546686	0.8364691
ENSG00000187240	DYNC2H1	11	103165375	C	T	0.360835	0.00262531	0.0146162	0.7099994	-0.0629298	0.00903022	3.20E-12	-0.0417181	0.232339	0.8575005	0.1744645
ENSG00000187244	BCAM	19	45318500	T	G	0.178926	0.00449091	0.018061	0.83	0.0880474	0.0105317	6.26E-17	0.0510056	0.205219	0.8037147	0.6372109
ENSG00000187257	RSBN1L	7	77367384	C	T	0.528827	-0.0137925	0.014189	0.33	0.156106	0.00791342	1.27E-86	-0.0883532	0.0910034	0.3316091	0.4303575
ENSG00000187260	WDR86	7	151090404	G	A	0.480119	0.0329917	0.0143197	0.02900013	-0.440389	0.00908327	0	-0.0749149	0.0325527	0.02137236	0.5515419
ENSG00000187266	EPOR	19	11491627	A	G	0.185885	-0.00592226	0.0195122	0.64	0.33272	0.0108233	1.60E-207	-0.0177995	0.0586473	0.7615081	0.0803897
ENSG00000187391	MAGI2	7	78364641	C	G	0.298211	-0.000346344	0.0156033	0.98	-0.0614084	0.00866421	1.36E-12	0.00564001	0.254092	0.982291	0.298812
ENSG00000187446	CHP1	15	41548540	A	G	0.0984095	-0.0021708	0.0228413	0.9400001	-0.324513	0.0154449	5.20E-98	0.00668941	0.0703872	0.9242851	0.9992306
ENSG00000187456	NA	17	34251423	C	T	0.158052	0.0156842	0.0184409	0.4700002	-0.0862922	0.0104019	1.08E-16	-0.181757	0.214823	0.39751	0.9302291
ENSG00000187474	FPR3	19	52313929	T	C	0.374751	-0.0170472	0.0148813	0.2999998	0.205958	0.00823405	4.41E-138	-0.0827701	0.0723297	0.2524803	0.2477296
ENSG00000187486	KCNJ11	11	17409142	T	C	0.349901	0.000559518	0.0148144	0.9599999	0.157188	0.012183	4.37E-38	0.00355955	0.0942467	0.9698724	0.9260524
ENSG00000187498	COL4A1	13	110880407	A	G	0.161034	0.0193425	0.0195328	0.3100002	-0.217442	0.0108596	3.47E-89	-0.0889547	0.0899397	0.3226394	0.3267503
ENSG00000187513	GJA4	1	35259973	A	G	0.351889	-0.00397338	0.0147503	0.99	-0.0934856	0.00855892	8.99E-28	0.0425026	0.157829	0.7877032	0.8684817
ENSG00000187514	PTMA	2	232574928	C	T	0.026839	-0.0187316	0.0590553	0.7600007	-0.246912	0.0434858	1.36E-08	0.0758633	0.239548	0.751476	0.2805882
ENSG00000187522	HSPA14	10	14896951	A	G	0.0924453	0.000772596	0.0244153	0.8700001	-0.192341	0.0140047	6.35E-43	-0.0040168	0.126938	0.9747561	0.05395016
ENSG00000187535	IFT140	16	1611269	G	T	0.195825	0.042888	0.0183099	0.0179999	0.161519	0.0100401	3.13E-58	0.265529	0.114556	0.02045481	0.8687558
ENSG00000187546	AGMO	7	15420791	G	A	0.411531	0.0178616	0.0144155	0.2200002	0.134828	0.00903019	2.08E-50	0.132477	0.107285	0.216901	0.01485875
ENSG00000187554	TLR5	1	223299686	A	G	0.44334	-0.0255519	0.0143323	0.06900014	-0.212036	0.00802927	1.11E-153	0.120507	0.0677475	0.07527743	0.7851737
ENSG00000187555	USP7	16	9022161	G	A	0.0636183	0.000313628	0.0286517	0.9299999	-0.133312	0.0175726	3.29E-14	-0.00235258	0.214922	0.9912664	0.04586226
ENSG00000187556	NANOS3	19	13982224	G	C	0.163022	-0.0281434	0.0185761	0.1	-0.0796763	0.014258	2.29E-08	0.353222	0.241561	0.1436737	0.9329466
ENSG00000187566	NHLRC1	6	18121784	A	G	0.488072	-0.00683628	0.0141968	0.6100002	0.0784725	0.00794755	5.41E-23	-0.0871168	0.181129	0.6305413	0.7178123
ENSG00000187569	DPPA3	12	7867102	G	A	0.428429	0.0188412	0.0144222	0.2200002	0.380414	0.0149314	3.51E-143	0.0495282	0.0379617	0.1919989	0.9767246
ENSG00000187583	PLEKHN1	1	906561	T	G	0.0447316	0.0191771	0.031652	0.5700002	-0.361831	0.0246616	9.77E-49	-0.0530002	0.087552	0.5449416	0.8432766
ENSG00000187595	ZNF385C	17	40197488	A	G	0.158052	0.00696553	0.0198533	0.8499999	0.376645	0.0219403	4.71E-66	0.0184936	0.0527219	0.7257559	0.9065904
ENSG00000187605	TET3	2	74279769	C	T	0.293241	0.0122587	0.0151206	0.4799997	0.0872645	0.0126624	5.52E-12	0.140477	0.174468	0.4207181	0.7822557
ENSG00000187607	ZNF286A	17	15621882	G	C	0.370775	0.0306814	0.014717	0.02399993	-0.702496	0.016155	0	-0.0436748	0.0209736	0.03730893	0.03828957
ENSG00000187608	ISG15	1	949361	C	T	0.0417495	-0.0221592	0.0380359	0.3700002	-0.62249	0.0304295	5.23E-93	0.0355977	0.0611276	0.5603307	0.2647846
ENSG00000187609	EXD3	9	140259531	A	G	0.363817	0.0134846	0.0144552	0.2099999	-0.134742	0.0107431	4.39E-36	-0.100077	0.107577	0.3522235	0.2474958
ENSG00000187621	LOC124903372	14	96131502	A	C	0.145129	-0.00827454	0.0209889	0.7099994	0.19284	0.0131731	1.58E-48	-0.0429088	0.10888	0.6935137	0.4153945
ENSG00000187624	LIAT1	17	266814	A	G	0.239563	0.00391798	0.0165798	0.91	1.00566	0.00726706	0	0.00389594	0.0164866	0.813192	0.1859538
ENSG00000187626	ZKSCAN4	6	28219706	G	C	0.114314	-0.0144457	0.01932	0.6300007	0.288767	0.0115758	2.37E-137	-0.0500254	0.0669351	0.4548394	0.67828
ENSG00000187627	RGPD1	2	87188090	T	G	0.204771	0.0121873	0.0183375	0.56	0.330016	0.0485804	1.10E-11	0.0369294	0.0558307	0.508322	0.6848427
ENSG00000187630	DHRS4L2	14	24457382	C	G	0.209742	0.0125296	0.0173564	0.4500005	-0.534497	0.0230645	8.33E-119	-0.0234418	0.0324881	0.4705707	0.2894389
ENSG00000187642	PERM1	1	914038	T	G	0.0447316	0.0191771	0.031652	0.5700002	-0.302645	0.0329693	4.33E-20	-0.063365	0.104812	0.5454728	0.8209224
ENSG00000187650	VMAC	19	5907866	C	T	0.577535	-0.0243013	0.0142672	0.06699926	-0.129179	0.0101473	4.01E-37	0.188122	0.11143	0.09136246	0.6268263
ENSG00000187667	NA	15	23198232	T	C	0.127237	-0.0427804	0.0239883	0.04300015	-0.356468	0.0214164	3.31E-62	0.120012	0.0676795	0.0761895	0.4208013
ENSG00000187676	B3GLCT	13	31840243	A	G	0.256461	-0.0287039	0.0165681	0.08	-0.507904	0.00929516	0	0.0565144	0.0326369	0.08334307	0.2286723
ENSG00000187688	TRPV2	17	16329586	A	G	0.384692	0.0310108	0.0145485	0.01499996	-0.169092	0.00810462	1.15E-96	-0.183397	0.0864871	0.03396321	0.9156218
ENSG00000187699	C2orf88	2	190906272	G	A	0.00397614	0.147355	0.145873	0.35	1.58289	0.108558	3.71E-48	0.0930925	0.0923771	0.3135774	0.4571247
ENSG00000187726	DNAJB13	11	73671387	A	G	0.131213	-0.0112359	0.0208465	0.5999997	0.212056	0.0113215	2.80E-78	-0.0529856	0.0983474	0.5900528	0.6254426
ENSG00000187730	GABRD	1	1956486	G	T	0.12326	0.00343461	0.0200586	0.9199999	0.190657	0.0156938	5.84E-34	0.0180146	0.105218	0.8640572	0.4105259
ENSG00000187735	TCEA1	8	54907100	T	C	0.195825	0.0170358	0.0198774	0.5400003	0.180512	0.0239629	4.96E-14	0.0943748	0.110827	0.3944638	0.8718108
ENSG00000187741	FANCA	16	89843511	T	A	0.183897	-0.00687981	0.0187985	0.5199996	-0.39851	0.0112175	1.98E-276	0.0172638	0.0471745	0.7143971	0.4343108

ENSG00000187742	SECISBP2	9	91953989	C	T	0.0914513	-0.0263278	0.0261326	0.1499999	0.421618	0.013327	1.16E-219	-0.0624446	0.0620131	0.3139544	0.02587272
ENSG00000187764	SEMA4D	9	92044373	C	T	0.417495	0.00865532	0.0146048	0.5500004	0.311155	0.00797729	0	0.0278167	0.0469428	0.553471	0.6272177
ENSG00000187775	DNAH17	17	76496627	G	C	0.274354	-0.0198733	0.015988	0.1900002	-0.0763613	0.0104728	3.07E-13	0.260254	0.212394	0.2204488	0.4703578
ENSG00000187778	MCRS1	12	49956131	G	A	0.0328032	0.0225335	0.037509	0.5999997	-0.115838	0.0191779	1.54E-09	-0.194525	0.325402	0.5499736	0.8082692
ENSG00000187790	FANCM	14	45637618	G	A	0.129225	-0.0101835	0.021924	0.6800001	0.158404	0.0124106	2.62E-37	-0.0642882	0.138497	0.6425161	0.268067
ENSG00000187792	ZNF70	22	24088524	T	C	0.250497	0.0152122	0.016222	0.2999998	-0.0614083	0.00954138	1.23E-10	-0.247722	0.266955	0.353432	0.5871424
ENSG00000187796	CARD9	9	139262244	A	G	0.393638	0.00619401	0.0143346	0.58	0.590152	0.00799173	0	0.0104956	0.0242901	0.6656726	0.8465271
ENSG00000187800	PEAR1	1	156874858	A	G	0.111332	0.027274	0.022592	0.1900002	0.248262	0.0135818	1.22E-74	0.10986	0.0911987	0.2283512	0.8719406
ENSG00000187801	ZFP69B	1	40922582	G	C	0.0616302	0.0211879	0.0294735	0.4100001	0.145223	0.0190821	2.73E-14	0.145899	0.203857	0.4741804	0.6965845
ENSG00000187815	ZFP69	1	40952451	T	C	0.305169	0.0161051	0.0157143	0.2399999	-0.187283	0.00954503	1.02E-85	-0.0859933	0.084021	0.3060839	0.9228987
ENSG00000187824	TMEM220	17	10617982	G	A	0.470179	-0.00504208	0.0144134	0.6700003	-0.238582	0.00795202	9.05E-198	0.0211335	0.0604168	0.7264924	0.4438756
ENSG00000187837	H1-2	6	26056333	G	C	0.355865	-0.0279809	0.0151578	0.02399993	-0.160136	0.00827986	2.46E-83	0.174732	0.0950861	0.06611809	0.06836522
ENSG00000187838	PLSCR3	17	7300231	A	G	0.177932	0.00978622	0.020474	0.83	-0.473892	0.0123838	0	-0.0206507	0.0432073	0.632689	0.373687
ENSG00000187860	CCDC157	22	30763635	C	T	0.253479	0.0282077	0.0165558	0.1299999	-0.137893	0.0104164	5.29E-40	-0.204563	0.121053	0.09105533	0.7710357
ENSG00000187862	TTC24	1	156553040	A	G	0.298211	0.0365184	0.0161022	0.02999991	0.229253	0.00932962	2.48E-133	0.159293	0.0705362	0.02392599	0.9699416
ENSG00000187866	PABIR1	9	71396786	A	G	0.350895	0.00304465	0.0165332	0.7300002	-0.0470307	0.00839534	2.12E-08	-0.0647375	0.35173	0.8539709	0.9138483
ENSG00000187870	NA	17	20652230	A	G	0.302187	0.0173733	0.0153653	0.35	-0.248916	0.0238653	1.81E-25	-0.069796	0.0620906	0.2609714	0.1282037
ENSG00000187902	SHISA7	19	55947168	C	G	0.537773	0.000796602	0.0141837	0.7600007	0.0931326	0.0104697	5.82E-19	0.00855342	0.152299	0.9552127	0.5051139
ENSG00000187912	CLEC17A	19	14707932	T	G	0.340954	0.00773417	0.0149509	0.56	-0.101785	0.00840875	9.99E-34	-0.0759857	0.147022	0.6052728	0.9189831
ENSG00000187922	LCN10	9	139635213	T	G	0.142147	-0.0306789	0.0204778	0.09699961	-0.0992211	0.0125743	3.00E-15	0.309197	0.210072	0.1410585	0.04419598
ENSG00000187950	OVCH1	12	29608013	A	G	0.161034	0.0108486	0.0203884	0.6100002	0.110577	0.0130926	3.02E-17	0.0981088	0.184747	0.5953884	0.9879608
ENSG00000187953	PMS2CL	7	6771626	T	C	0.238569	0.0238183	0.0170524	0.2	-0.149168	0.00933524	1.79E-57	-0.159675	0.114753	0.1640844	0.203682
ENSG00000187954	ZFTRAF1	8	145683012	C	T	0.479125	0.00200725	0.0142187	0.9699999	0.100192	0.00792571	1.25E-36	0.020034	0.141923	0.8877428	0.5380155
ENSG00000187961	KLHL17	1	898531	G	A	0.101392	-0.000878732	0.0247198	0.8800001	-0.25237	0.0193135	5.08E-39	0.00348192	0.0979511	0.9716431	0.5521674
ENSG00000187980	PLA2G2C	1	20495831	T	C	0.153082	0.00371054	0.0190963	0.95	0.0952633	0.0115997	2.16E-16	0.0389504	0.200514	0.8459783	0.2008704
ENSG00000187984	NA	9	95611322	T	C	0.385686	0.0226639	0.0143811	0.09299937	-0.16368	0.00900778	8.76E-74	-0.138464	0.0881907	0.116402	0.1367349
ENSG00000187990	NA	6	26216650	T	C	0.0516899	-0.0179936	0.0341427	0.4299995	0.672315	0.0172952	0	-0.0267636	0.0507884	0.5982191	0.4632813
ENSG00000187994	RINL	19	39363694	C	T	0.506958	-0.008842	0.0142478	0.6999999	-0.183291	0.00790525	6.30E-119	0.0482402	0.077761	0.5350176	0.2503339
ENSG00000187997	C17orf99	17	76152346	C	G	0.162028	0.0245117	0.0206063	0.32	-0.174334	0.0124885	2.75E-44	-0.140602	0.118628	0.2359268	0.3527167
ENSG00000188000	OR7D2	19	9297886	A	G	0.0656064	-0.0293983	0.0331246	0.2099999	1.18224	0.0173752	0	-0.0248667	0.028021	0.3748469	0.5123755
ENSG00000188002	NA	5	1616577	C	G	0.235586	0.00229523	0.0170506	0.95	-0.765291	0.0164764	0	-0.00299916	0.02228	0.8929184	0.144097
ENSG00000188004	SNHG28	1	159814700	T	C	0.241551	-0.00345346	0.0167532	0.7700005	-0.133875	0.0116233	1.07E-30	0.0257962	0.125161	0.8367094	0.6424136
ENSG00000188010	MORN2	2	39129658	A	G	0.117296	-0.0457709	0.0219594	0.05999983	0.0808203	0.0142636	1.46E-08	-0.566329	0.289507	0.05044317	0.1183315
ENSG00000188011	RTP5	2	242813863	A	G	0.104374	0.0278911	0.0240278	0.1499999	-0.288431	0.0205044	6.08E-45	-0.0966995	0.0835884	0.2473326	0.7769445
ENSG00000188015	S100A3	1	153520826	C	T	0.00994036	0.0264619	0.0971467	0.8499999	1.14967	0.103332	9.37E-29	0.023017	0.0845251	0.785384	0.4554916
ENSG00000188026	RILPL1	12	123987095	T	G	0.0357853	0.0273452	0.0376855	0.58	0.5421	0.0222459	3.68E-131	0.0504431	0.0695484	0.4682714	0.5484252
ENSG00000188033	ZNF490	19	12719843	G	A	0.0974155	0.01003	0.0229085	0.56	0.292987	0.02472	2.10E-32	0.0342337	0.0782429	0.6617268	0.7692718
ENSG00000188037	CLCN1	7	143031197	T	C	0.405567	-0.0181981	0.01444	0.2599998	0.321126	0.00790958	0	-0.0566697	0.0449884	0.2077958	0.06100575
ENSG00000188039	NWD1	19	16879780	G	A	0.358847	0.0204523	0.0153058	0.28	-0.0817613	0.0133641	9.48E-10	-0.250146	0.191614	0.1917329	0.2599722
ENSG00000188042	ARL4C	2	235403691	A	G	0.45825	-0.0241177	0.0143011	0.1199999	0.187675	0.00787711	1.82E-125	-0.128508	0.0763919	0.09252737	0.4109627
ENSG00000188051	TMEM221	19	17552847	T	C	0.404573	-0.0133393	0.0144437	0.5300002	0.103001	0.0121346	2.10E-17	-0.129507	0.141057	0.3585564	0.664609
ENSG00000188056	TREML4	6	41201341	G	A	0.228628	0.0217695	0.0174147	0.1800002	1.08904	0.00861835	0	0.0199896	0.0159917	0.2112983	0.7301187
ENSG00000188076	SCGB1C1	11	193826	A	G	0.210736	-0.00475724	0.0168747	0.95	0.549053	0.01805	3.13E-203	-0.00866445	0.0307355	0.7780176	0.1916794
ENSG00000188092	GPR89B	1	147433129	T	G	0.207753	0.012831	0.017234	0.3800004	0.18261	0.0249979	2.77E-13	0.0702645	0.0948649	0.4588877	NA
ENSG00000188152	NUTM2G	9	99696563	A	G	0.150099	0.027641	0.0216878	0.1900002	0.420939	0.0250503	2.29E-63	0.065665	0.0516704	0.2037837	0.4206112
ENSG00000188157	AGRN	1	973499	T	G	0.0427435	-0.04054	0.0385984	0.17	-0.236801	0.0385267	7.93E-10	0.171199	0.165362	0.3005307	0.1117904
ENSG00000188167	TMPPE	3	33135103	G	A	0.0198807	-0.115	0.131498	0.3700002	-0.478081	0.0665851	6.97E-13	0.240545	0.277087	0.3853275	0.4746
ENSG00000188171	ZNF626	19	20823634	A	G	0.224652	-0.00920455	0.0167998	0.6899999	0.548723	0.0109518	0	-0.0167745	0.030618	0.5837855	0.3757336
ENSG00000188177	ZC3H6	2	113065405	G	A	0.389662	-0.00615443	0.0147628	0.7199992	-0.116143	0.00816382	6.27E-46	0.0529899	0.127163	0.6768914	0.6327571
ENSG00000188185	NA	7	39802961	T	C	0.371769	-0.00310913	0.0146994	0.8600001	0.672922	0.0177054	0	-0.00462034	0.0218445	0.8324887	0.1513004

ENSG00000188186	LAMTOR4	7	99750048	G	A	0.368787	-0.0129866	0.0144514	0.4299995	0.247856	0.00887311	1.05E-171	-0.0523957	0.0583358	0.3690923	0.07119759
ENSG00000188191	PRKAR1B	7	678060	C	T	0.318091	0.0079559	0.0156455	0.4	0.356486	0.0200253	6.84E-71	0.0223176	0.043906	0.6112408	0.7267368
ENSG00000188199	NUTM2B	10	81468710	C	T	0.40159	0.0010891	0.0147781	0.6800001	-0.627165	0.0179439	1.23E-267	-0.00173654	0.0235634	0.9412517	0.8935101
ENSG00000188211	NCR3LG1	11	17386080	A	G	0.272366	0.00815768	0.0161444	0.81	0.132683	0.0106831	2.04E-35	0.0614827	0.121778	0.6136452	0.9494074
ENSG00000188215	DCUN1D3	16	20890551	G	T	0.419483	0.0066275	0.0143977	0.6999999	0.099099	0.0134019	1.42E-13	0.0668776	0.145567	0.6459274	0.8326077
ENSG00000188227	ZNF793	19	38018752	A	C	0.153082	-0.0219885	0.0193806	0.25	0.275145	0.0105673	1.87E-149	-0.079916	0.0705046	0.2570099	0.2604668
ENSG00000188234	AGAP4	10	46335182	A	C	0.447316	0.0121133	0.0143479	0.5500004	-0.651973	0.0157285	0	-0.0185794	0.0220114	0.3986242	0.7028424
ENSG00000188242	SLC9A3-OT1	5	469726	A	C	0.133201	0.00431868	0.0219443	0.8499999	-0.141506	0.019578	4.91E-13	-0.0305194	0.155134	0.8440396	0.02261724
ENSG00000188243	COMMD6	13	76111462	G	A	0.122266	0.00428023	0.0194745	0.7400005	-0.168227	0.0113452	9.66E-50	-0.0254432	0.115776	0.8260563	0.7871622
ENSG00000188266	HYKK	15	78814810	C	A	0.364811	0.0576951	0.0150835	0.00015	-0.0546898	0.00944328	6.98E-09	-1.05495	0.330526	0.001414233	0.8630541
ENSG00000188277	C15orf62	15	41063401	T	C	0.105368	0.0162576	0.0216759	0.3900004	-0.106387	0.0168319	2.61E-10	-0.152815	0.205174	0.4563893	0.4605415
ENSG00000188282	RUFY4	2	218927493	T	G	0.138171	-0.0159507	0.0207362	0.3100002	-0.173465	0.0111061	5.42E-55	0.0919536	0.119686	0.4423156	0.6091881
ENSG00000188283	ZNF383	19	37721828	A	G	0.314115	-0.0042047	0.015503	0.6300007	-0.0659098	0.0084669	7.00E-15	0.0637948	0.235358	0.7863495	0.116516
ENSG00000188290	HES4	1	934947	A	G	0.208748	0.0105076	0.0181208	0.81	0.470331	0.0131125	9.37E-282	0.0223409	0.0385328	0.5620574	0.3129673
ENSG00000188295	ZNF669	1	247265469	C	T	0.336978	0.00431071	0.0150451	0.6499995	0.240515	0.00834595	1.27E-182	0.0179229	0.0625569	0.7744913	0.200368
ENSG00000188305	PEAK3	19	2278406	G	A	0.354871	0.0282367	0.014619	0.08999948	0.414331	0.00926299	0	0.0681501	0.0353163	0.05364295	0.6321663
ENSG00000188312	CENPP	9	95235290	A	T	0.172962	0.0184972	0.0195503	0.3800004	0.887598	0.0159125	0	0.0208396	0.0220292	0.3441497	0.03314907
ENSG00000188313	PLSCR1	3	146247809	G	C	0.257455	-0.0210008	0.0162952	0.1900002	-0.479268	0.0128229	9.63E-306	0.0438185	0.0340204	0.1977436	0.6950241
ENSG00000188315	C3orf62	3	49310688	G	C	0.232604	0.0290518	0.0162075	0.1100001	-0.116967	0.00984106	1.41E-32	-0.248376	0.140132	0.07632023	0.02539932
ENSG00000188316	ENO4	10	118640160	C	T	0.270378	0.0135448	0.0164295	0.4199997	0.195581	0.0111253	3.52E-69	0.0692543	0.084096	0.4102155	0.5341479
ENSG00000188321	ZNF559	19	9448143	T	C	0.0864811	0.0225724	0.0248932	0.5	-1.1031	0.0149895	0	-0.0204627	0.0225683	0.364565	0.5469763
ENSG00000188322	SBK1	16	28319505	C	T	0.44831	0.00748395	0.0144001	0.7499995	0.361194	0.00909788	0	0.02072	0.0398715	0.6032932	0.6426428
ENSG00000188342	GTF2F2	13	45776443	C	T	0.0606362	-0.00280901	0.0292836	0.98	0.868459	0.0164014	0	-0.00323448	0.0337191	0.9235808	0.07236588
ENSG00000188352	FOCAD	9	20827131	T	C	0.239563	0.00868627	0.0167805	0.5099998	-0.127558	0.0104769	4.21E-34	-0.0680964	0.13167	0.605035	0.3600025
ENSG00000188372	ZP3	7	76049111	A	C	0.405567	0.00230413	0.014464	0.7700005	-0.269946	0.00858581	5.59E-217	-0.00853553	0.0535819	0.8734333	0.7842283
ENSG00000188375	H3-5	12	31944649	G	A	0.116302	0.00192809	0.0220091	0.91	0.334359	0.0129809	2.64E-146	0.00576653	0.0658252	0.9301917	0.8992786
ENSG00000188385	JAKMIP3	10	133958244	C	G	0.101392	0.0555472	0.0258828	0.03400008	0.236154	0.0166733	1.54E-45	0.235216	0.110852	0.03384742	0.4068905
ENSG00000188389	PDCD1	2	242796546	G	A	0.533797	-0.000447398	0.0141807	0.7499995	-0.19755	0.0104989	5.56E-79	0.00226473	0.0717829	0.9748311	0.7169045
ENSG00000188393	CLEC2A	12	10068126	C	G	0.0308151	-0.00651302	0.0359905	0.9699999	0.465622	0.0192552	3.47E-129	-0.0139878	0.0772977	0.856399	0.4684235
ENSG00000188404	SELL	1	169670323	C	T	0.279324	0.00477709	0.0153426	0.7700005	-0.631734	0.00792095	0	-0.00756187	0.0242867	0.7555279	0.8509128
ENSG00000188428	BLOC1S5	6	8039223	A	T	0.340954	0.009975	0.0149885	0.5199996	-0.203429	0.00919821	2.21E-108	-0.0490342	0.0737125	0.5059167	0.06688856
ENSG00000188452	CERKL	2	182473397	G	A	0.462227	0.0191313	0.0143421	0.1800002	0.179913	0.00802983	3.47E-111	0.106336	0.0798579	0.1830021	0.5696142
ENSG00000188467	SLC24A5	15	48424019	T	A	0.170974	-0.0458766	0.0196589	0.01899984	0.359962	0.011226	1.35E-225	-0.127448	0.0547583	0.01994003	0.02819917
ENSG00000188486	H2AX	11	118965370	A	T	0.370775	-0.0179084	0.0149951	0.2099999	-0.0844132	0.00844909	1.67E-23	0.212152	0.178904	0.2356851	0.5019796
ENSG00000188487	INSC	11	15201362	G	T	0.441352	0.0105942	0.014412	0.5	-0.166515	0.00869595	9.94E-82	-0.063623	0.0866143	0.4626107	0.4216713
ENSG00000188493	ACTMAP	19	41252109	C	A	0.274354	0.00865247	0.0158432	0.7099994	0.376671	0.00866212	0	0.0229709	0.0420645	0.5850052	0.8386675
ENSG00000188511	MIR3667HG	22	49929683	A	G	0.276342	0.00415818	0.0158942	1	0.371596	0.00872923	0	0.01119	0.0427736	0.7936212	0.07149427
ENSG00000188522	FAM83G	17	18890109	G	A	0.154076	-0.0154435	0.0198396	0.4199997	0.514847	0.0155069	1.04E-241	-0.0299963	0.0385455	0.4364487	0.5226595
ENSG00000188529	SRSF10	1	24299355	C	T	0.482107	-0.0108848	0.0143477	0.56	-0.169601	0.0146815	7.21E-31	0.064179	0.0847792	0.4490417	0.1613246
ENSG00000188542	DUSP28	2	241501451	T	C	0.147117	-0.031981	0.0199889	0.14	-0.306853	0.0152578	5.89E-90	0.104223	0.0653475	0.1107348	0.325323
ENSG00000188549	CCDC9B	15	40628410	G	A	0.464215	-0.00345987	0.014257	0.9400001	0.194742	0.0117757	1.97E-61	-0.0177664	0.0732176	0.8082746	0.5554923
ENSG00000188554	NBR1	17	41343103	G	A	0.361829	-0.02106	0.0150602	0.1199999	-0.162604	0.00830376	2.20E-85	0.129517	0.0928546	0.1630654	0.5616858
ENSG00000188559	RALGAPA2	20	20531731	G	A	0.0347913	-0.0146577	0.0362912	0.8499999	0.189275	0.0228598	1.23E-16	-0.0774414	0.191966	0.6866454	0.7344984
ENSG00000188566	NDOR1	9	140105804	A	G	0.0467197	0.0491399	0.0309757	0.04900044	-0.461509	0.0345933	1.34E-40	-0.106477	0.0675911	0.1151865	0.5029143
ENSG00000188573	FBLL1	5	167956880	A	C	0.17992	-0.0389797	0.0180803	0.0329997	0.292412	0.0123414	4.20E-124	-0.133304	0.062087	0.03178921	0.6804774
ENSG00000188582	PAQR9	3	142675092	T	C	0.0864811	-0.043968	0.0265995	0.0649995	-0.15066	0.0176823	1.59E-17	0.291835	0.179845	0.1046516	0.2076636
ENSG00000188599	NPIPP1	16	15211818	T	C	0.152087	-0.0382136	0.0195703	0.03500016	0.837819	0.0241113	1.49E-264	-0.0456108	0.0233955	0.05122899	0.989284
ENSG00000188603	CLN3	16	28492439	A	G	0.525845	0.00328858	0.0142453	0.8200001	0.200622	0.00863085	1.61E-119	0.0163919	0.0710091	0.8174373	0.9980882
ENSG00000188610	FAM72B	1	120846476	G	A	0.38668	0.00575175	0.0159877	0.84	0.3526	0.0189515	2.91E-77	0.0163124	0.0453507	0.7190762	0.3228041
ENSG00000188612	SUMO2	17	73171243	A	G	0.328032	0.0172104	0.0157709	0.25	-0.189181	0.0144419	3.32E-39	-0.0909734	0.0836531	0.2768121	0.8061215

ENSG00000188613	NANOS1	10	120791541	A	G	0.40159	0.00278192	0.0143595	0.6899999	-0.326235	0.0085569	0	-0.00852735	0.0440164	0.8463863	0.7125263
ENSG00000188636	RTL6	22	44891315	G	A	0.444334	0.0187296	0.0143153	0.16	0.54393	0.00741272	0	0.0344338	0.0263224	0.1908209	0.1191249
ENSG00000188641	DPYD	1	97964952	T	G	0.196819	-0.00338512	0.0177001	0.7899998	-0.332581	0.0104906	1.43E-220	0.0101783	0.0532213	0.8483335	0.959548
ENSG00000188647	PTAR1	9	72349656	T	C	0.0646123	-0.0171138	0.0313499	0.6600001	0.585572	0.0204216	8.00E-181	-0.0292258	0.0535469	0.5852051	0.6854353
ENSG00000188649	CC2D2B	10	97763113	T	C	0.0526839	0.00380739	0.033615	0.9699999	0.449425	0.0181799	6.36E-135	0.0084717	0.0747964	0.9098217	0.9653918
ENSG00000188659	SAXO2	15	82566211	A	C	0.0328032	-0.0227229	0.0359658	0.56	1.21385	0.0340765	6.56E-278	-0.0187197	0.0296342	0.5275878	0.9712799
ENSG00000188672	RHCE	1	25722711	C	T	0.44334	-0.0105628	0.0142635	0.4299995	-0.0911986	0.0086874	8.84E-26	0.115822	0.156789	0.460082	0.2043213
ENSG00000188676	IDO2	8	39833021	T	A	0.392644	6.00E-05	0.0146116	0.7800007	-0.0966167	0.0093112	3.18E-25	-0.000620881	0.151233	0.9967243	0.4633357
ENSG00000188677	PARVB	22	44480098	C	T	0.409543	-0.0083239	0.014498	0.5700002	0.216631	0.00864582	1.49E-138	-0.0384243	0.0669423	0.5659742	0.5491558
ENSG00000188687	SLC4A5	2	74506955	C	A	0.028827	-0.0193142	0.0406427	0.5	0.357217	0.026373	8.50E-42	-0.0540685	0.113846	0.6348389	0.2254757
ENSG00000188690	UROS	10	127494481	G	T	0.441352	-0.00368559	0.0142484	0.8800001	-0.541755	0.00735532	0	0.00680306	0.0263006	0.7958935	0.4608254
ENSG00000188707	NA	7	150028373	C	T	0.244533	-0.000427921	0.0163773	0.9299999	-0.477422	0.0095384	0	0.000896316	0.0343036	0.9791545	0.1070754
ENSG00000188725	SMIM15	5	60455918	A	G	0.457256	0.0106982	0.0143014	0.4299995	-0.0494859	0.00892699	2.97E-08	-0.216187	0.291619	0.4584914	0.8337771
ENSG00000188732	FAM221A	7	23731308	G	A	0.0198807	0.00550103	0.046717	0.95	0.367513	0.0288078	2.84E-37	0.0149683	0.127122	0.906268	0.1085745
ENSG00000188735	TMEM120B	12	122185782	A	C	0.251491	0.0140174	0.0161126	0.4	-0.165105	0.0171065	4.84E-22	-0.0848998	0.0979855	0.3862421	0.4260327
ENSG00000188739	RBM34	1	235309635	C	A	0.125249	-0.00112647	0.0208306	0.9	-0.0918804	0.0135557	1.22E-11	0.0122602	0.226722	0.9568747	0.9483344
ENSG00000188747	NOXA1	9	140323330	C	G	0.436382	0.000494532	0.0144226	0.9599999	0.291653	0.0102276	7.34E-179	0.00169562	0.0494512	0.972647	0.2322487
ENSG00000188760	TMEM198	2	220411851	A	C	0.196819	0.00695985	0.0175812	0.5999997	-0.0703451	0.0111341	2.65E-10	-0.0989386	0.250418	0.6927735	0.317518
ENSG00000188761	BCL2L15	1	114425479	T	G	0.270378	0.00294606	0.0153017	0.9299999	-0.107142	0.00927769	7.52E-31	-0.0274967	0.142836	0.8473468	0.1960519
ENSG00000188779	SKOR1	15	68119470	A	T	0.256461	-0.0320867	0.016069	0.02	-0.199105	0.0168069	2.24E-32	0.161155	0.0818445	0.04894937	0.0984109
ENSG00000188785	ZNF548	19	57907002	G	A	0.129225	0.0396812	0.0207149	0.05800027	0.653629	0.0111241	0	0.060709	0.031709	0.05554735	0.7853266
ENSG00000188786	MTF1	1	38302571	T	C	0.275348	0.00137871	0.0159644	0.8800001	-0.110014	0.00949655	4.93E-31	-0.0125322	0.145117	0.9311809	0.09857353
ENSG00000188811	NHLRC3	13	39618344	G	A	0.147117	-0.0130344	0.0197953	0.59	-0.155475	0.0130037	6.03E-33	0.0838361	0.127514	0.5108832	0.2099148
ENSG00000188818	ZDHHC11	5	780786	G	A	0.0785288	-0.036899	0.0349589	0.2200002	0.194031	0.03126	5.40E-10	-0.190171	0.182758	0.2980803	0.6027766
ENSG00000188820	CALHM6	6	116783733	G	T	0.0914513	-0.00521469	0.0251764	0.83	-1.03711	0.0131624	0	0.00502809	0.0242756	0.8359119	0.8802061
ENSG00000188822	CNR2	1	24241282	C	A	0.423459	0.00953279	0.0143229	0.3700002	0.100418	0.00923762	1.59E-27	0.0949306	0.142899	0.5064862	0.1137878
ENSG00000188825	LINC00910	17	41456890	T	G	0.0854871	-0.0540253	0.0257823	0.0179999	-0.112946	0.0186638	1.43E-09	0.478328	0.241568	0.04769266	0.4547799
ENSG00000188827	SLX4	16	3646390	A	G	0.0208748	0.0247927	0.0391579	0.3800004	-0.269299	0.0320152	4.04E-17	-0.0920637	0.145818	0.5278046	0.864706
ENSG00000188846	RPL14	3	40501322	C	T	0.317097	0.0128278	0.0152518	0.56	0.321557	0.00949474	2.05E-251	0.0398927	0.0474456	0.4004551	0.2982849
ENSG00000188848	BEND4	4	42133925	T	C	0.236581	0.0330113	0.0166931	0.02900013	-0.0801973	0.0103507	9.34E-15	-0.411626	0.214823	0.05535047	0.1994494
ENSG00000188859	FAM78B	1	166081440	G	A	0.0119284	-0.0160493	0.0524489	0.9299999	0.571266	0.0882728	9.70E-11	-0.0280943	0.0919143	0.7598656	NA
ENSG00000188878	FBF1	17	73921438	G	C	0.359841	-0.0118838	0.0149442	0.35	-0.112018	0.015684	9.18E-13	0.106088	0.134233	0.4293363	0.1337025
ENSG00000188895	MSL1	17	38285795	G	A	0.028827	0.0136035	0.039328	0.7199992	-0.55965	0.0307723	6.57E-74	-0.0243072	0.0702852	0.7294659	0.4440311
ENSG00000188897	LOC400499	16	11535573	C	T	0.353877	0.0228735	0.0147836	0.1499999	-0.1226	0.0096873	1.04E-36	-0.186569	0.121481	0.1245907	0.9470893
ENSG00000188906	LRRK2	12	40676816	T	C	0.450298	0.00551328	0.014213	0.7199992	-0.333585	0.00849744	0	-0.0165274	0.0426089	0.6981011	0.3450822
ENSG00000188921	HACD4	9	21013470	A	G	0.287276	-0.0204094	0.0154326	0.07100027	0.328285	0.0155569	7.59E-99	-0.0621698	0.047102	0.1868695	0.0904553
ENSG00000188931	CFAP126	1	161336092	T	C	0.0487078	0.00443695	0.0302634	0.91	-0.131282	0.0156762	5.54E-17	-0.0337971	0.230558	0.8834567	0.3964051
ENSG00000188933	NA	17	16704828	G	A	0.0725646	0.0131658	0.0270508	0.6999999	-0.293973	0.0382568	1.54E-14	-0.0447858	0.0922024	0.6271564	0.4744053
ENSG00000188938	FAM120AOS	9	96212325	A	T	0.149105	0.0229638	0.0195668	0.29	-0.17825	0.0106874	1.88E-62	-0.128829	0.110043	0.2417131	0.195723
ENSG00000188958	UTS2B	3	191016641	A	T	0.345924	0.00701308	0.0148421	0.6999999	0.151954	0.00920176	2.93E-61	0.0461526	0.0977149	0.6366989	0.09675362
ENSG00000188959	C9orf152	9	112961398	G	T	0.484095	0.0150465	0.0141999	0.2599998	-0.216526	0.00898103	2.00E-128	-0.0694907	0.065644	0.2897828	0.08542471
ENSG00000188971	NA	2	69690039	T	C	0.415507	0.0154144	0.0144205	0.4	-0.136419	0.0224536	1.24E-09	-0.112993	0.107331	0.292454	0.7937002
ENSG00000188976	NOC2L	1	887136	A	G	0.212724	-0.00138302	0.0178184	0.7400005	-0.247736	0.0175934	4.96E-45	0.00558264	0.0719261	0.9381332	0.9118139
ENSG00000188986	NELFB	9	140158811	G	A	0.271372	-0.0197589	0.0167	0.1299999	-0.3569	0.0172384	3.20E-95	0.0553625	0.0468681	0.2375072	0.5381203
ENSG00000188994	ZNF292	6	87918232	G	A	0.370775	-0.00620823	0.0145353	0.7499995	-0.100901	0.00892689	1.27E-29	0.0615282	0.144158	0.6695182	0.5384076
ENSG00000188997	KCTD21	11	77891081	C	T	0.16998	-0.0107808	0.0194086	0.59	-0.269674	0.0127337	1.52E-99	0.0399772	0.0719953	0.5787072	0.4676327
ENSG00000189007	ADAT2	6	143759968	T	A	0.311133	0.00615499	0.0156731	0.7400005	0.325488	0.0127789	4.15E-143	0.01891	0.0481583	0.6945678	0.849153
ENSG00000189013	KIR2DL4	19	55280987	G	C	0.0387674	-0.0397578	0.0393447	0.2999998	0.346384	0.0538232	1.23E-10	-0.11478	0.114979	0.3181491	0.6317307
ENSG00000189014	NA	10	47400219	A	G	0.206759	0.0190753	0.0188747	0.3400001	-0.167046	0.0233122	7.74E-13	-0.114192	0.11411	0.3169604	0.2095347
ENSG00000189042	ZNF567	19	37198558	A	G	0.304175	0.0343948	0.0152931	0.01899984	0.0795792	0.00849352	7.29E-21	0.432208	0.197634	0.02874851	0.2462119

ENSG00000189043	NDUFA4	7	10975730	T	C	0.460239	-0.0171712	0.0143351	0.2300001	0.05405	0.0079977	1.40E-11	-0.317691	0.269353	0.2382151	0.7677498
ENSG00000189045	ANKDD1B	5	74937477	A	G	0.256461	0.0262711	0.0164982	0.17	-0.0740506	0.0108451	8.61E-12	-0.354772	0.228775	0.1209617	0.158093
ENSG00000189046	ALKBH2	12	109528714	T	C	0.0397614	0.0383227	0.0341062	0.2	-0.42232	0.0197436	1.65E-101	-0.0907434	0.0808706	0.2618278	0.9970626
ENSG00000189050	RNFT1	17	58035861	C	T	0.154076	0.0407316	0.017839	0.02900013	-0.456268	0.0179199	5.25E-143	-0.0892711	0.0392545	0.02295615	0.1244456
ENSG00000189057	FAM111B	11	58884770	G	A	0.425447	0.00185491	0.014419	0.8600001	-0.0729813	0.00806905	1.50E-19	-0.0254162	0.197591	0.8976501	0.5145977
ENSG00000189058	APOD	3	195303324	T	C	0.302187	-0.0168595	0.0156894	0.25	0.154355	0.0109755	6.36E-45	-0.109226	0.101941	0.2839645	0.5742285
ENSG00000189060	H1-0	22	38202278	T	C	0.457256	-0.0230535	0.0142727	0.05600025	0.681384	0.00708354	0	-0.0338333	0.0209496	0.1063131	0.6888087
ENSG00000189067	LITAF	16	11686045	A	G	0.0675944	-0.0112507	0.0272353	0.5999997	-0.203152	0.014474	9.44E-45	0.0553808	0.134122	0.6796698	0.09245202
ENSG00000189068	VSTM1	19	54555643	T	C	0.483101	-0.00312406	0.0142209	0.9299999	0.756502	0.0102553	0	-0.00412961	0.0187983	0.8261204	0.7884617
ENSG00000189077	TMEM120A	7	75620066	A	G	0.171968	-0.00687911	0.0192434	0.8499999	0.176448	0.0102749	4.24E-66	-0.0389866	0.109084	0.7207913	0.7027477
ENSG00000189079	ARID2	12	46212635	A	C	0.498012	0.0217572	0.0142481	0.1	0.0579817	0.00870126	2.67E-11	0.375242	0.252104	0.1366342	0.6802842
ENSG00000189091	SF3B3	16	70583255	G	A	0.414513	-0.00262061	0.0143468	0.99	0.183195	0.00797494	9.01E-117	-0.014305	0.0783166	0.855068	0.7108195
ENSG00000189114	BLOC1S3	19	45683531	T	C	0.128231	-0.00578065	0.0219339	0.5700002	-0.165513	0.0115192	8.17E-47	0.0349257	0.132543	0.7921619	0.05901286
ENSG00000189127	ANKRD34B	5	79859440	C	A	0.257455	0.00555253	0.0158505	0.6600001	-0.650829	0.00832422	0	-0.00853147	0.0243546	0.7261115	0.7441666
ENSG00000189129	PLAC9	10	81898296	A	C	0.0526839	0.0534362	0.0288697	0.025	0.685279	0.0321769	1.21E-100	0.0779773	0.0422872	0.06518416	0.2861176
ENSG00000189134	NKAPL	6	28227917	G	C	0.104374	-0.0634517	0.0238968	0.002800013	0.0968159	0.0147473	5.20E-11	-0.655385	0.266251	0.01383464	0.2185808
ENSG00000189136	NA	15	85092229	T	C	0.122266	0.00515353	0.0228295	0.7300002	0.171307	0.0206857	1.22E-16	0.0300837	0.133316	0.8214689	0.7676751
ENSG00000189143	CLDN4	7	73230443	T	A	0.274354	0.0157213	0.0165485	0.4600002	0.137615	0.014317	7.12E-22	0.114241	0.120838	0.344451	0.100626
ENSG00000189144	ZNF573	19	38267337	C	G	0.388668	-0.0104765	0.0146683	0.3400001	0.281953	0.00861052	3.55E-235	-0.0371569	0.0520362	0.4751917	0.5322284
ENSG00000189159	JPT1	17	73147859	G	A	0.374751	0.00837448	0.0149767	0.5400003	-0.10809	0.00845977	2.20E-37	-0.0774766	0.13869	0.5764125	0.2848229
ENSG00000189171	S100A13	1	153599068	T	G	0.519881	-0.00216337	0.0142104	0.99	-0.346778	0.0084652	0	0.00623848	0.0409786	0.8789997	0.03519294
ENSG00000189180	ZNF33A	10	38326797	T	C	0.116302	0.0283678	0.0236084	0.2599998	-0.113199	0.0139528	4.94E-16	-0.250601	0.210831	0.2345847	0.1940417
ENSG00000189182	KRT77	12	53090328	C	A	0.0487078	-0.00199798	0.0355901	0.95	0.765629	0.0198199	0	-0.00260959	0.0464849	0.9552314	0.2110458
ENSG00000189190	ZNF600	19	53278746	G	C	0.491054	0.00391502	0.0145847	0.7300002	0.560087	0.0128119	0	0.00699002	0.0260406	0.7883697	0.4359826
ENSG00000189223	PAX8-AS1	2	113996843	C	A	0.383698	-0.00910596	0.0148497	0.3100002	-0.730686	0.00959323	0	0.0124622	0.0203236	0.5397517	0.4894485
ENSG00000189227	C15orf61	15	67816517	A	G	0.139165	0.0298568	0.0218571	0.2999998	0.219876	0.0121582	4.21E-73	0.135789	0.0996895	0.1731601	0.08229987
ENSG00000189233	NUGGC	8	27910434	G	A	0.0119284	0.0502912	0.058157	0.28	0.259938	0.036359	8.73E-13	0.193474	0.225365	0.3906211	0.4720541
ENSG00000189238	LINC00943	12	127220807	A	G	0.0427435	0.000460154	0.0324045	0.8600001	-0.116462	0.0208908	2.48E-08	-0.00395111	0.278242	0.9886702	0.6999997
ENSG00000189241	TSPYL1	6	116599403	C	T	0.233598	-0.00931492	0.0173269	0.5099998	0.205636	0.00943406	2.47E-105	-0.0452981	0.0842856	0.5909669	0.6132488
[truncated: 1,330,543 more chars]
